# *meta*‐Selective C−H Borylation of Benzylamine‐, Phenethylamine‐, and Phenylpropylamine‐Derived Amides Enabled by a Single Anionic Ligand

**DOI:** 10.1002/anie.201708967

**Published:** 2017-09-19

**Authors:** Holly J. Davis, Georgi R. Genov, Robert J. Phipps

**Affiliations:** ^1^ Department of Chemistry University of Cambridge Lensfield Road Cambridge CB2 1EW UK

**Keywords:** C−H activation, homogeneous catalysis, hydrogen bonding, noncovalent interactions, regioselectivity

## Abstract

Selective functionalization at the meta position of arenes remains a significant challenge. In this work, we demonstrate that a single anionic bipyridine ligand bearing a remote sulfonate group enables selective iridium‐catalyzed borylation of a range of common amine‐containing aromatic molecules at the arene meta position. We propose that this selectivity is the result of a key hydrogen bonding interaction between the substrate and catalyst. The scope of this meta‐selective borylation is demonstrated on amides derived from benzylamines, phenethylamines and phenylpropylamines; amine‐containing building blocks of great utility in many applications.

The employment of non‐covalent interactions to direct transition metal catalysis is a strategy of great potential but remains relatively underexplored, particularly for addressing issues of regioselectivity and site‐selectivity.^1^, ^2^ A commonly encountered regioselectivity choice arises during the functionalization of aromatic rings. In electrophilic aromatic substitution, regioselectivity is determined largely by the electronic predisposition of the substrate, while proximity to a directing group is the determining factor in directed *ortho*‐metalation chemistry and many catalytic arene C−H functionalizations.^3^ The application of C−H functionalization strategies to distal positions is more challenging^4^, ^5^ and we believe that the design of catalytic systems which incorporate attractive non‐covalent interactions constitutes a powerful strategy to address these challenges.1c

Iridium‐catalyzed C−H borylation has evolved into a uniquely powerful arene functionalization method that operates under mild conditions, is highly tolerant of functionality and delivers boronate esters that can be readily transformed into a variety of common groups.^6^ Perhaps most interesting is the regioselectivity of this process, which is largely controlled by steric considerations.6a, 7 Whilst highly effective for borylation of 1,3‐disubstituted arenes, isomeric mixtures are typically obtained when borylation is applied to monosubstituted and unsymmetrical 1,2‐disubstituted arenes, limiting broader application. There have been a number of developments in *ortho*‐selective borylation,^8^, ^9^ but much fewer achieve *meta*‐10 or *para*‐selectivity.^11^ The productive harnessing of non‐covalent interactions has played a crucial role in several recent advances.9i, 11b This includes “outer sphere” hydrogen bond‐directed approaches such as those developed by Singleton, Maleczka, Smith and co‐workers for *ortho*‐borylation of aniline derivatives9d,9e (Figure 1 a) and by Kuninobu, Kanai and co‐workers for *meta*‐selective borylation of arenes bearing carbonyl and phosphoryl groups (Figure 1 b).10a We have recently disclosed a bipyridine ligand bearing a distal anionic sulfonate group to direct iridium‐catalyzed borylation to the arene *meta* position in two classes of aromatic quaternary ammonium salts, by virtue of a putative ion‐pairing interaction between the active catalyst and cationic substrate (Figure 1 c).10c Herein, we report that this same anionic ligand scaffold is highly proficient as a hydrogen bond acceptor to direct borylation to the *meta* position in a range of aromatic substrates bearing amide hydrogen bond donors. Effective on benzylamine, phenethylamine and phenylpropylamine‐derived amides, our method delivers versatile, multifunctional arenes with broad potential applicability in pharmaceuticals and materials chemistry.

**Figure 1:**
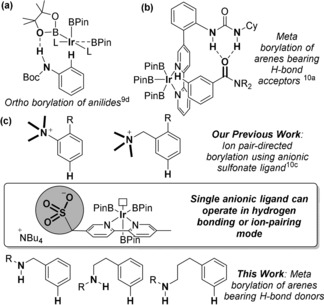
Selected examples of the use of hydrogen bonds to control regioselectivity in iridium‐catalyzed C−H borylation.

At the outset of our studies, we envisioned that an appropriately designed bipyridine ligand bearing a remote hydrogen bond accepting group may enable the *meta* selective borylation of arenes bearing hydrogen bond donors. If successful, this would represent an electronically inverted strategy to that of Kuninobu and Kanai and may provide a complementary scope of compatible substrates. We already possessed four bipyridine ligands that bear a sulfonate group from our previous study on ion‐pair directed borylation (**1 a**, **1 b**, **1 i** and **1 j**). In addition to the sulfonate group, we targeted three neutral hydrogen bond acceptor groups: amide, phosphonate and sulfoxide, for comparison. This resulted in a library of 16 bifunctional bipyridine ligands with the hydrogen bond acceptor extending from the 5‐ (**1 a**–**h**) or 4‐ (**1 i**–**p**) position, combined with either one or two methylene units between the bipyridine and the hydrogen bond acceptor (Figure 2 a). These were tested in the borylation of **2 a** and the outcome was that only ligand **1 a**, which was optimal in our ion pair‐directed approach, resulted in good *meta* selectivity (8:1 *m*:*p*).^12^ Of the others, none gave greater than a 2.6:1 *m*:*p* ratio. We investigated alternative *N*‐protecting groups and although an *N*‐mesyl protected substrate gave similarly high selectivity, we elected to proceed with trifluoroacetyl for its ease of deprotection (Figure 2 b).^13^ Additionally, borylation of **2 a** at room temperature increased selectivity to 13:1. A variety of solvents were compatible with the borylation and THF was found to give the best balance between selectivity and conversion.^13^ In order to evaluate the generality of ligand **1 a** for the borylation of substrates bearing different chain lengths between the amide and arene, we synthesized and tested the two‐ and three‐ carbon analogues of **2 a** (Figure 2 c). Excellent *meta*‐selectivity was achieved in both cases using ligand **1 a** despite the much increased flexibility in the chain when compared with benzylamine‐derived substrate **2 a**.^14^ In both cases, the use of the standard borylation ligand 4,4‐di‐*tert*‐butylbipyridine (**dtbpy**) resulted in poor regiocontrol.6c

**Figure 2:**
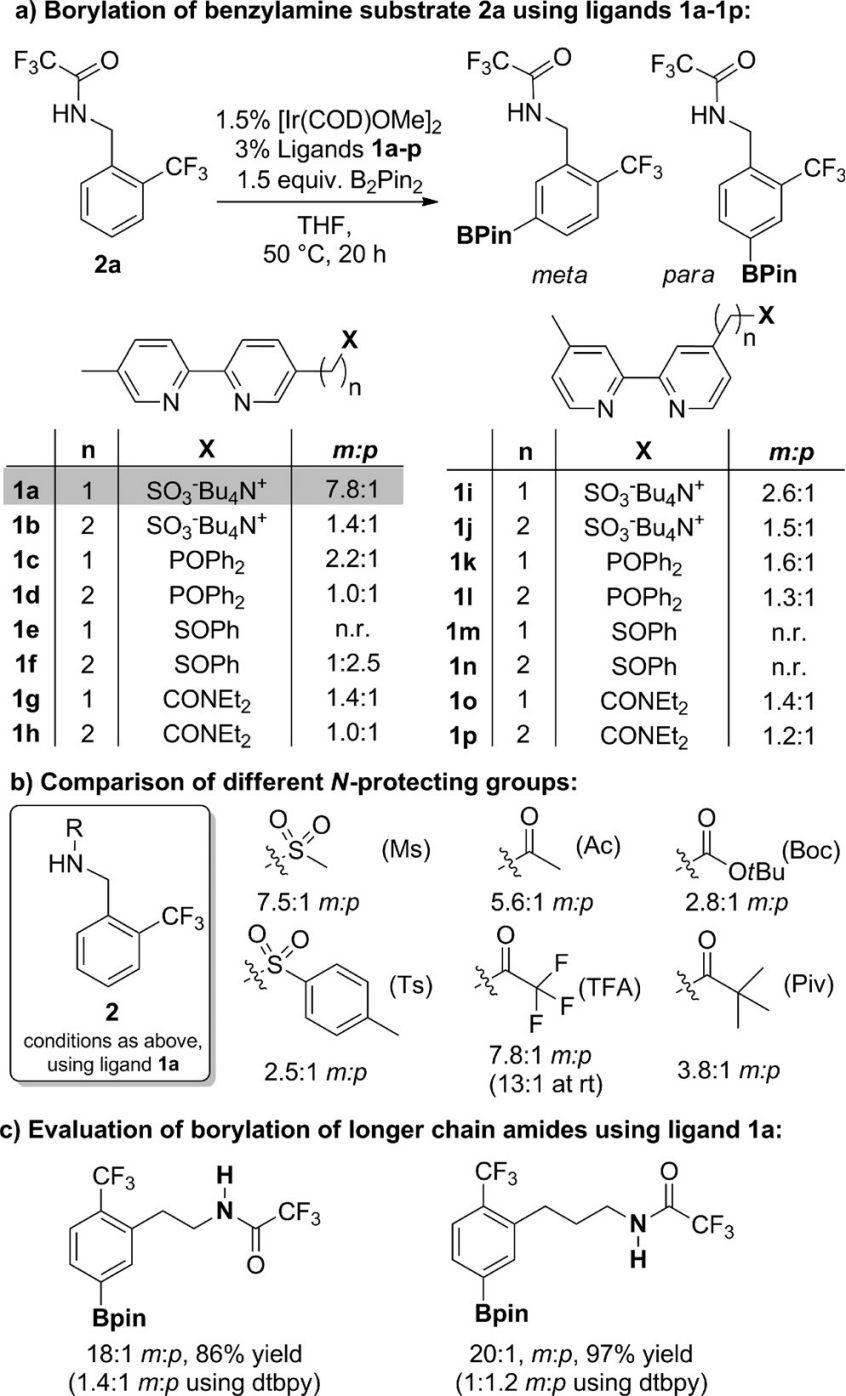
Initial evaluation and optimization studies.

In evaluation of the scope of the transformation we found that a range of trifluoroacetylated benzylamine (Table 1 a), phenethylamine (Table 1 b) and phenylpropylamine (Table 1 c) derived amides were borylated with good to excellent regioselectivity for the *meta* position using ligand **1 a**. In all cases, poor selectivity was obtained in the control reaction with **dtbpy**. Electron withdrawing groups such as trifluoromethyl (**3 a**, **5 a**, **7 a**) and esters (**3 g**, **5 g**, **7 g**) are tolerated, as are halides including bromides (**5 f**, **7 c**), chlorides (**3 d**, **5 h**, **7 d**) and even iodide (**5 d**). Electron donating alkyl groups do not affect the selectivity (**3 b**, **5 i**, **7 i**), although substrates bearing a 2‐methoxy substituent underwent non‐selective borylation using **1 a** (**3 c**, **5 j**, **7 j**). Several studies have shown that alkoxy groups exhibit a small but significant electronic *meta*‐directing effect in borylation, which could go some way to accounting for this.^15^ A substrate bearing an unprotected hydroxyl group likely undergoes in situ *O*‐borylation (**3 f**).9d,9e Unsubstituted arenes lead to *meta*‐diborylated products (**3 h, 5 l, 7 b**) and, in the case of **3 i**, a methyl substituent in the benzylic position is tolerated. Fluorine is sufficiently small to allow borylation at the adjacent position and this could be controlled on monofluorinated (**3 e**, **3 j, 5 e**, **7 e**, **7 f**) and difluorinated (**7 h**) arenes. Our ligand‐directed approach was also effective on pyridine substrates from all three classes (Table 1 d). In these cases, borylation was favored at the 4‐position whilst **dtbpy** gave mixtures of borylation at the 4‐ and 5‐ positions.

**Table 1:** Scope of the hydrogen bond‐directed *meta*‐selective borylation.^[a,b]^

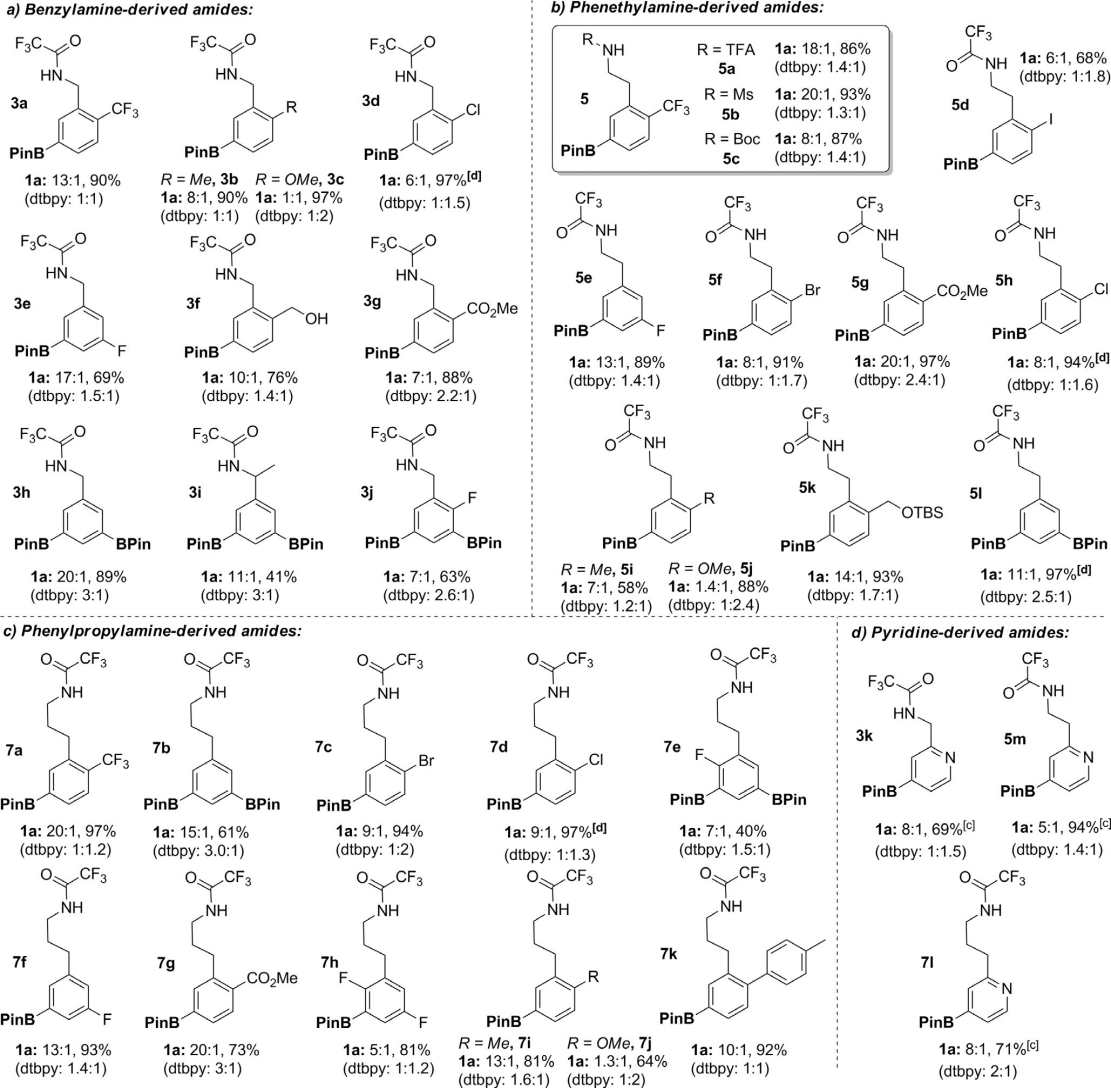

[a] Typically: Substrate (0.25 mmol), B_2_Pin_2_ (0.375–1.0 mmol), [Ir(COD)OMe]_2_ (1.5 mol %), **1 a** (3 mol %), THF (0.2 m), RT to 70 °C (see the Supporting Information for details). [b] Isomeric ratios are *meta*:*para* taken from analysis of crude ^1^H‐NMR spectra; yields shown are isolated unless otherwise indicated and include *meta* and *para* isomers which are generally inseparable. [c] NMR yield quoted due to decomposition on silica gel. [d] Product isolated as mixture of mono‐ and diborylated compounds.

We anticipated that there would come a point where further increasing the flexibility of the substrate would have a negative impact on the borylation regioselectivity as the entropic price for achieving an organized transition state becomes prohibitively high. This point is reached with substrates bearing chains longer than three carbons; despite screening all four sulfonate ligands poor regioselectivity was observed.^13^

With regard to the fact that a single ligand is compatible with three different classes of amine, we speculate that the sulfonate group, with its negative charge distributed across all three oxygen atoms, represents a relatively diffuse area of high electron density at a fixed distance from the iridium metal centre. Thus, it seems reasonable that as long as a given substrate is able to achieve a low‐energy conformation that will allow a productive hydrogen bond to form in the borylation transition state then this may plausibly lead to *meta*‐selective borylation. To probe this hypothesis, we evaluated several substrates which possess restricted conformational freedom to assess the impact on regioselectivity (Figure 3 a). For aminoindane‐derived substrate **11**, in which rotation about all but the C−N bond is impossible, very poor regioselectivity was obtained. Further support for the importance of a productive hydrogen bonding interaction between catalyst and substrate was obtained in the borylation of the *cis* and *trans* isomers of **12**. For the *cis* isomer, in which the amino group occupies an axial position, poor regioselectivity was obtained whilst the *trans* isomer gave far better regiocontrol. This can be rationalized by deducing that the lowest energy conformation of *cis*‐**12** is likely to involve the N−H (rather than the trifluoroacetyl group) projecting across the cyclohexane ring, away from the arene, in order to avoid unfavourable 1,3‐diaxial interactions that would arise in the alternative conformation. This likely precludes productive hydrogen‐bonding interactions with the catalyst in the transition state. Conversely, *trans*‐**12** is likely to have far more conformational freedom and a favourable substrate–ligand interaction may be easily achievable. We also investigated the borylation of anilides and found that acetanilide gave a good 12:1 *m*:*p* ratio (Figure 3 b). However, introducing an *ortho*‐substituent resulted in disappointing regioselectivity. This can be rationalized by considering that the acetyl group will occupy a position *anti* to the *ortho* substituent, placing the substituent *syn* to the N−H bond. Therefore, any hydrogen bonding interaction in the transition state would have to overcome significant catalyst–substrate steric interactions (Figure 3 b, inset).

**Figure 3:**
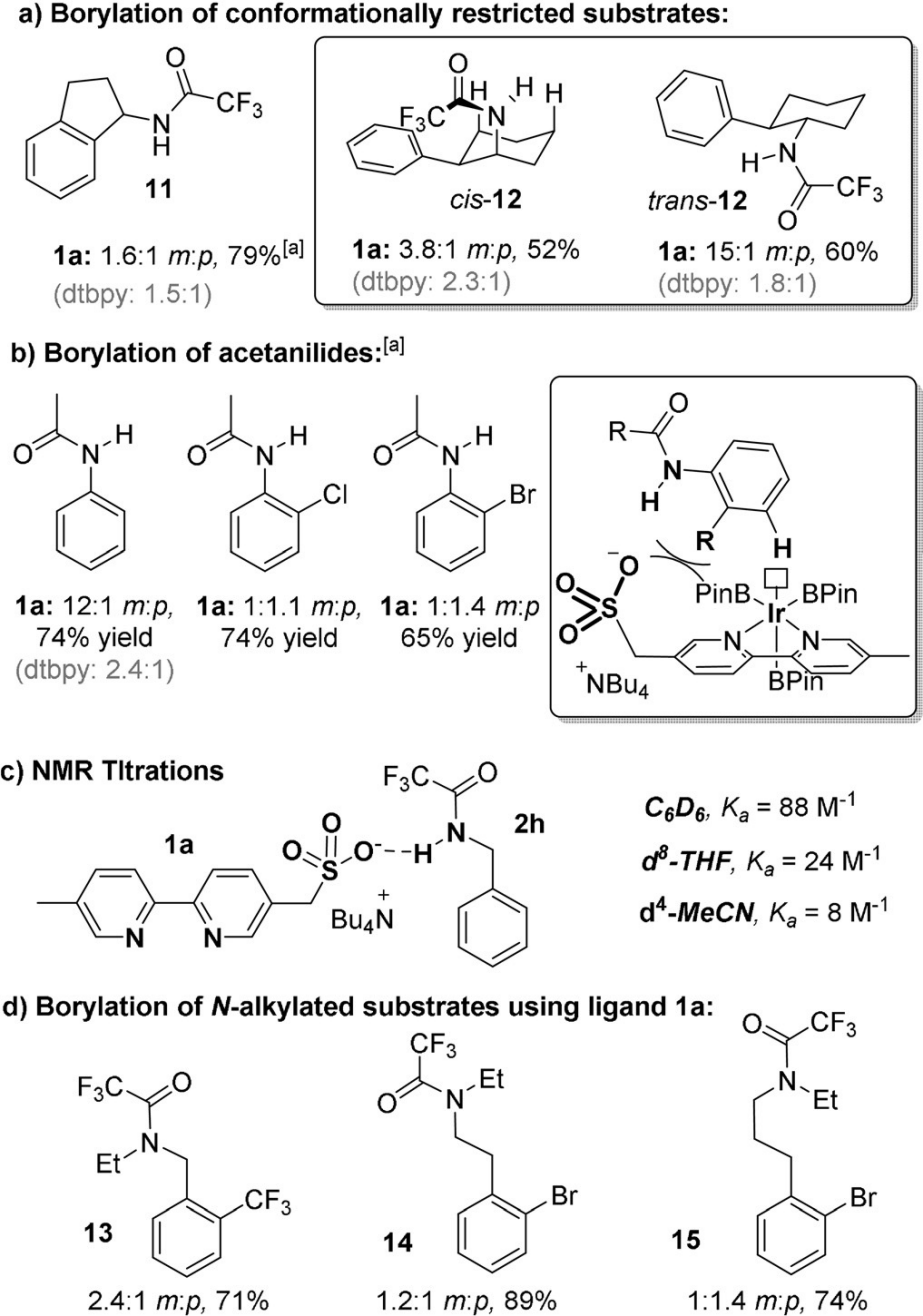
Borylation of conformationally restricted substrates and mechanistic experiments. [a] Total yield determined by ^1^H‐NMR spectroscopy using internal standard (1,2‐dimethoxyethane).

To provide quantitative evidence for the proposed ligand‐substrate hydrogen bonding interaction, we investigated the extent of interaction between amide substrate **2 h** and ligand **1 a** in various solvents (Figure 3 c). NMR titrations showed that even in polar MeCN, significant association is seen.^16^ Background titrations of 5,5′‐dimethylbipyridine against **2 h** showed negligible interaction, demonstrating that the sulfonate group is responsible for the observed shifts of the N‐H proton. Finally, *N*‐alkylated substrates resulted in poor regioselectivity upon borylation with ligand **1 a**, emphasizing the importance of the N−H in order to achieve high levels of regiocontrol and providing support for the crucial role of hydrogen bonding (Figure 3 d).

In conclusion, we have developed a method for the *meta*‐selective borylation of benzylamine, phenethylamine and phenylpropylamine derivatives, using a single anionic bipyridine ligand. This ligand, previously successful in “ion‐pairing mode” has been found to be highly effectively in “hydrogen‐bond accepting mode” and underlines the general nature of this strategy and ligand scaffold for regioselective borylation. Our method provides valuable and practical access to pharmaceutically relevant scaffolds bearing complex substitution patterns and more broadly highlights the potential of harnessing non‐covalent interactions to exert regiocontrol in C−H activation chemistry.

## Conflict of interest

The authors declare no conflict of interest.

## Supporting information

As a service to our authors and readers, this journal provides supporting information supplied by the authors. Such materials are peer reviewed and may be re‐organized for online delivery, but are not copy‐edited or typeset. Technical support issues arising from supporting information (other than missing files) should be addressed to the authors.

Supplementary
