# Supplementary material for: meta‐Selective C−H Borylation of Benzylamine‐, Phenethylamine‐, and Phenylpropylamine‐Derived Amides Enabled by a Single Anionic Ligand
Source: Angew Chem Int Ed Engl. 2017 Sep 19;56(43):13351–5. doi: 10.1002/anie.201708967 (PMC5656929; doi:10.1002/anie.201708967)

Angewandte Chemie
Eine Zeitschrift der Gesellschaft Deutscher Chemiker

Supporting Information

Meta-Selective C–H Borylation of Benzylamine, Phenethylamine and Phenylpropylamine-Derived Amides Enabled by a Single Anionic Ligand

*Holly J. Davis, Georgi R. Genov, and Robert J. Phipps**

anie_201708967_sm_miscellaneous_information.pdf

Contents

1. General Information S2
2. Evaluation of H-bond accepting ligands on 2a: S3
3. Comparison of N-protecting groups S4
4. Optimisation of Solvent and other Reaction Parameters on Substrate 2a S5
5. Examination of substrates bearing longer tether lengths S7
6. Synthesis of Substrates S8
7. Borylations of Substrates S31
8. Borylation of Longer Chain Substrates S72
9. Borylation of conformationally restricted substrates (Figure 3a) S77
10. Borylation of Acetanilides (Figure 3b) S82
11. NMR Titrations (Figure 3c) S86
12. Synthesis & borylation of N-Ethyl Substrates (Figure 3d) S90
13. Synthesis of Bipyridine Ligands S95
14. References S103
15. NMRs of Ligands, Substrates and Borylated Products S104

General Information

All reagents, unless otherwise stated, were used as supplied from commercial sources without further purification. $[Ir(COD)OMe]_2$ was purchased from Sigma-Aldrich, used as received and stored in a desiccator. CH_2Cl_2, THF and Et_2O were purified by distillation on site under inert atmosphere *via* the following processes: THF and Et_2O were pre-dried over sodium wire then distilled from calcium hydride and lithium aluminium hydride. CH_2Cl_2, *n*-hexane and toluene were distilled from calcium hydride. NEt_3 and $^iPr_2NEt$ were purified by distillation from calcium hydride and stored over 4 Å molecular sieves. Ligand **1a** was prepared according the procedure detailed in our previous publication.[1]

Reactions were carried out in 4 mL 15x45mm crimp top vials, which were purged with Argon. Vials were heated in deep-welled heating blocks (IKA DB 5.2).

NMR spectra: ^1H NMR spectra were recorded on a 600 MHz Bruker Avance DRX-600 spectrometer, 500 MHz Bruker DCH Cryoprobe or 400 MHz QNP Cryoprobe. Chemical shifts are reported in parts per million (ppm) and the spectra are calibrated to the resonance resulting from incomplete deuteration of the solvent ($CDCl_3$: 7.26 ppm, CD_3OD: 3.31 ppm, $(CD_3)_2SO$: 2.50 ppm). ^{13}C NMR spectra were recorded the same spectrometer with complete proton decoupling. Chemical shifts are reported in ppm with the solvent resonance as the internal standard ($^{13}CDCl_3$: 77.16 ppm, t; DMSO-d_6: 39.51 ppm, s). Data are reported as follows: chemical shift δ/ppm, integration (^1H only), multiplicity (s = singlet, d = doublet, t = triplet, q = quartet, br = broad, m = multiplet or combinations thereof; ^{13}C signals are singlets unless otherwise stated), coupling constants *J* in Hz, assignment. ^1H-COSY, DEPT-135, HMQC, HMBC and NOESY were used where appropriate to facilitate structural determination of regioisomers. The carbon attached to boron was generally not observed by ^{13}C spectroscopy due to quadrupolar relaxation. ^{19}F NMR spectra were recorded on a 400 MHz Bruker Avance III HD Spectrometer.

High Resolution Mass Spectrometry (HRMS): Some were recorded on a Waters Micromass LCT Premier spectrometer using a positive electrospray ionization (ESI+). Other were measured at the EPSRC Mass Spectrometry Service at the University of Swansea. Measured values are reported to 4 decimal places are within ±5 ppm of the calculated value. The calculated values are based on the most abundant isotope.

Chromatography: Analytical thin layer chromatography was performed using precoated Merck glass backed silica gel plates (Silicagel 60 F254). Visualisation was by ultraviolet fluorescence (λ = 254 nm) and/or staining with cerium ammonium molybdate (CAM) or potassium permanganate ($KMnO_4$). Flash column chromatography was performed using silica gel 60 (0.040-0.063 µm) from Fluorochem.

Evaluation of H-bond accepting ligands on 2a:

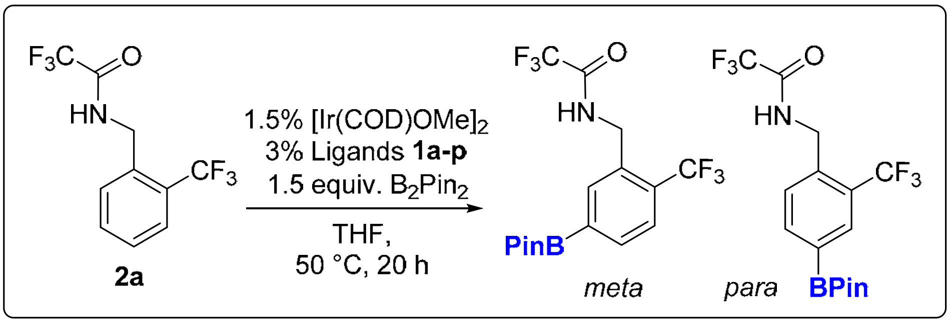

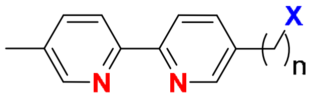

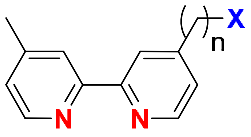

n = 1, **X** = $SO_3^-Bu_4N^+$ **1a**
n = 2, **X** = $SO_3^-Bu_4N^+$ **1b**
n = 1, **X** = $POPh_2$ **1c**
n = 2, **X** = $POPh_2$ **1d**
n = 1, **X** = SOPh **1e**
n = 2, **X** = SOPh **1f**
n = 1, **X** = $CONEt_2$ **1g**
n = 2, **X** = $CONEt_2$ **1h**

n = 1, **X** = $SO_3^-Bu_4N^+$ **1i**
n = 2, **X** = $SO_3^-Bu_4N^+$ **1j**
n = 1, **X** = $POPh_2$ **1k**
n = 2, **X** = $POPh_2$ **1l**
n = 1, **X** = SOPh **1m**
n = 2, **X** = SOPh **1n**
n = 1, **X** = $CONEt_2$ **1o**
n = 2, **X** = $CONEt_2$ **1p**

Ligand	*Meta*	*para*	SM	NMR yield[a]	*m/p*
**1a**	86	11	0	97	7.8
**1b**	48	40	0	88	1.4
**1c**	65	29	0	94	2.2
**1d**	50	48	0	98	1.0
**1e**	0	0	91	0	N/A
**1f**	10	24	62	34	0.4
**1g**	52	38	0	90	1.4
**1h**	47	45	0	92	1.0
**1i**	63	24	0	87	2.6
**1j**	43	28	5	71[b]	1.5
**1k**	53	33	0	86	1.6
**1l**	48	36	0	84	1.3
**1m**	0	0	97	0	N/A
**1n**	0	0	95	0	N/A
**1o**	33	24	0	57[c]	1.4
**1p**	47	39	0	86	1.2

a) Dimethoxyethane used as internal standard. b) 10% NMR yield of mixture of unidentified borylated compounds, c) 28% NMR yield of mixture of unidentified borylated compounds

Comparison of *N*-protecting groups:

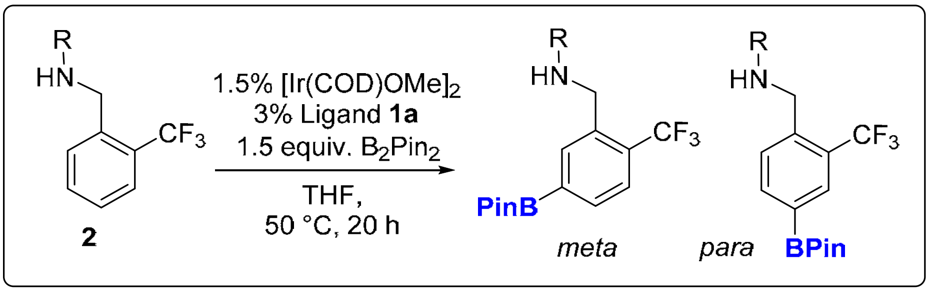

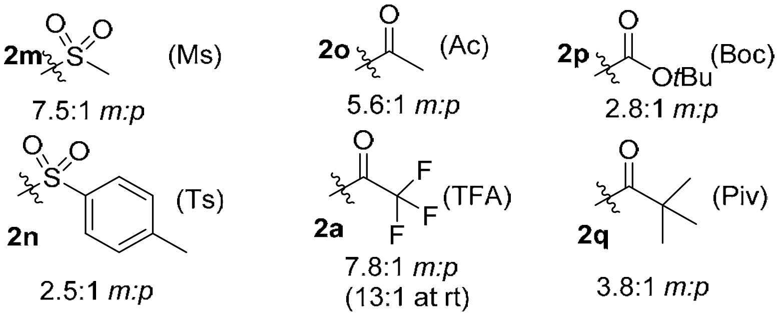

Protecting Group	*Meta*	*para*	SM	NMR yield [a] / %	*m/p*
**Ms**	83	11	6	94	7.5
**Ts**	63	25	12	88	2.5
**Ac**	77	14	0	91	5.6
**TFA**	86	11	0	97	7.8
**Boc**	73	27	0	100	2.8
**Piv**	79	21	0	3.8	3.8

a) Dimethoxyethane used as internal standard.

See later for full details of isolation and characterisation.

Optimisation of Solvent and other Reaction Parameters on Substrate 2a

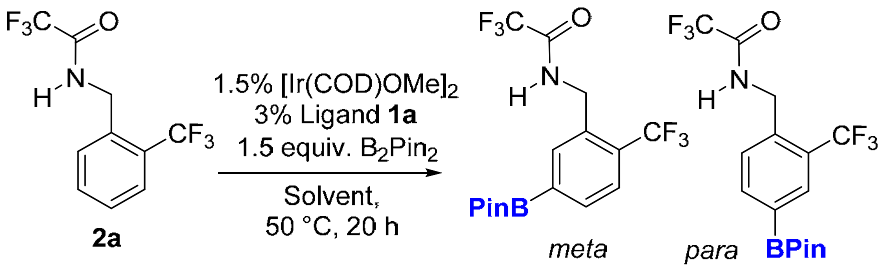

Solvent	*meta*	*para*	SM	NMR yield [a] / %	*m/p*
**Hexane**	72	14	2	86	5.1
**Cyclohexane**	61	9	15	80	6.8
***p*-xylene**	72	12	2	84	6.0
**Et_2O**	75	15	0	90	5.0
**MTBE**	75	16	0	91	4.7
**2-MeTHF**	82	12	0	94	6.8
**EtOAc**	77	13	3	90	5.9
**THF**	86	11	0	97	7.8
**CH_2Cl_2**	29	3	54	32	9.7
**1,2-DCE**	12	2	75	14	6.0
**CH_3CN**	57	7	18	64	8.1
***n*-Octane**	69	13	1	82	5.3
**THF:CH_2Cl_2 1:1**	55	8	24	63	6.9
**Hexane:THF 1:1**	74	12	0	86	6.2
**THF:CH_2Cl_2***	24	3	63	27	8.0

*Substrate dissolved in CH_2Cl_2 added to the catalyst complex preformed in THF to overall dilution 0.1 M.
[a]Dimethoxyethane used as internal standard.

All further optimisations using 1.5 equiv. B_2pin_2, 50 °C, 0.2 M in THF, 3% cat. loading apart from change indicated:

Dilution (M)	*meta*	*para*	SM	NMR yield / %	*m/p*
**1.0**	66	15	0	81	4.4
**0.4**	76	12	0	88	6.3
**0.2**	86	11	0	97	7.8
**0.13**	80	11	0	91	7.3

B_2pin_2 equiv.	*meta*	*para*	SM	NMR yield / %	*m/p*
**1.0**	64	12	9	76	5.3
**1.5**	86	11	0	97	7.8
**2.0**	82	10	0	92	8.2
**3.0**	82	10	0	92	8.2

Cat. loading	*meta*	*para*	SM	NMR yield / %	*m/p*
**1.5**	84	10	0	94	8.4
**3**	86	11	0	97	7.8
**5**	80	10	0	90	8
**6**	63	13	3	79*	4.8

*4% NMR yield of mixture of unidentified borylated compounds

Using 2 equiv. B_2pin_2

**Temperature / °C**	***meta***	***para***	**SM**	**NMR yield / %**	***m/p***
**rt (22)**	78	6	0	84	13.0
**30**	85	8	0	93	10.6
**40**	81	8.5	0	89.5	9.5
**50**	82	10	0	92	8.2

Examination of substrates bearing longer tether lengths

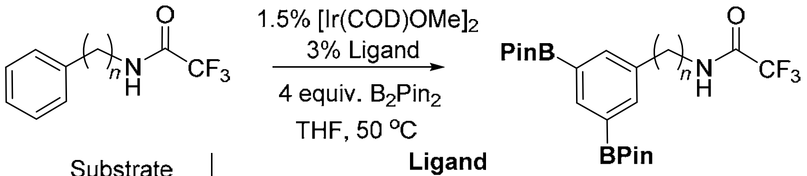

Substrate Chain Length	Ligand 1a	1b	1i	1j	
n = 3 (**7b**)	15 : 1	5.6 : 1	3.4 : 1	2.4 : 1	*m:p* ratio
n = 4 (**8**)	7.5 : 1	5.0 : 1	2.8 : 1	2.7 : 1	
n = 5 (**9**)	5.7 : 1	6.7 : 1	3.5 : 1	3.2 : 1	
n = 6 (**10**)	4.3 : 1	5.7 : 1	3.2 : 1	3.6 : 1	

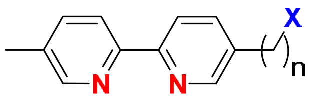

n = 1, **X** = $SO_3^-Bu_4N^+$ **1a**
n = 2, **X** = $SO_3^-Bu_4N^+$ **1b**

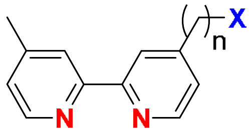

n = 1, **X** = $SO_3^-Bu_4N^+$ **1i**
n = 2, **X** = $SO_3^-Bu_4N^+$ **1j**

Synthesis of Substrates

General procedure C:

A flask was charged with amine (1.1 equiv.), CH_2Cl_2 (ca. 0.4 M) and triethylamine (1.2 equiv.) and cooled to 0 °C. Trifluoroacetic anhydride (1 equiv.) was added dropwise over 5 mins with rapid stirring. The reaction was warmed to rt and stirred for specified time before being diluted with CH_2Cl_2 (ca. 0.2 M) and quenched with 1M HCl. The organic layer was separated and washed sequentially with 1M HCl and saturated $NaHCO_3$ before being dried ($MgSO_4$) and solvents removed *in vacuo*. The crude product was washed with Pet. Ether (40-60) or purified by silica gel chromatography as specified.

General procedure D:

A flask was charged with acetonitrile (10 equiv.) in THF (ca. 10 M) and cooled to -78 °C. *n*-BuLi (1.5 equiv.) was added dropwise over 15 mins and the solution stirred for 1 hour before the addition of the specified electrophile (1 equiv.) over 15 mins still at -78 °C. The reaction was stirred for a further hour before the injection of water (2 mL) and the mixture warmed to rt. EtOAc was added and the aqueous phase separated and extracted twice with EtOAc. Solvents were removed *in vacuo* and the crude compounds purified by silica gel chromatography.

General procedure E:

A flask was charged with $AlCl_3$ (1 equiv.) in THF (ca. 0.35 M) and cooled to 0 °C. $LiAlH_4$ (2.4M in THF, 1.3 equiv.) was added over 5 mins and stirred for a further 10 mins before the addition of the specified nitrile (1 equiv.) in THF (ca. 1 M) dropwise over 5 mins. The mixture was warmed to rt and stirred overnight before cooling again to 0 °C, diluting with Et_2O (to ca. 0.1 M) and quenching, slowly, with water. The mixture was basified with 10% NaOH to ca. pH 10 and the organic layer separated. The aqueous phase was extracted twice more with EtOAc and the combined organic phases were dried ($MgSO_4$) and solvents removed *in vacuo*. The amine was used crude in subsequent steps.

2,2,2-Trifluoro-*N*-(2-(trifluoromethyl)benzyl)acetamide (2a)

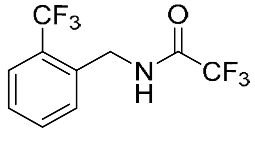

Following general procedure C with 2-trifluoromethylbenzylamine (964 mg, 5.5 mmol), stirred for 1 hour and purified by washing with Pet. Ether (40-60) **2a** was isolated as white crystals (1.21 g, 4.5 mmol, 89%).

^{1}H NMR (600 MHz, $CDCl_3$) δ 7.70 (d, J = 7.7 Hz, 1H), 7.58 (t, J = 7.5 Hz, 1H), 7.53 (d, J = 7.7 Hz, 1H), 7.46 (d, J = 7.5 Hz, 1H), 6.77 (br s, 1H); **^{13}C NMR** (151 MHz, $CDCl_3$) δ 157.2 (q, $^2J_{C\text{-}F}$ = 37.2 Hz), 134.1 (q, $^4J_{C\text{-}F}$ = 1 Hz), 132.7, 140.0, 128.5, 128.4 (q, $^2J_{C\text{-}F}$ = 31 Hz), 126.3 (q, $^3J_{C\text{-}F}$ = 6 Hz), 124.3 (q, $^1J_{C\text{-}F}$ = 275 Hz), 115.8 (q, $^1J_{C\text{-}F}$ = 289 Hz), 40.6 (q, $^4J_{C\text{-}F}$ = 2 Hz); **HRMS** m/z: $[M + NH_4]^+$ calc'd for $[C_{10}H_{11}F_6N_2O]^+$ expect 289.0770; found 289.0773.

2,2,2-Trifluoro-*N*-(2-methylbenzyl)acetamide (2b)

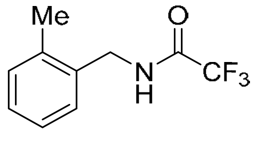

Following general procedure C with 2-methylbenzylamine (400 mg, 3.3 mmol), stirred for 2 hours and purified by washing with Pet. Ether (40-60) **2b** was isolated as white crystals (411 mg, 1.9 mmol, 63%).

^{1}H NMR (600 MHz, $CDCl_3$) δ 7.29-7.26 (m, 1H), 7.24-7.21 (m, 3H), 6.88 (br s, 1H), 4.50 (d, *J* = 5.7 Hz, 2H), 2.34 (s, 3H); **^{13}C NMR** (151 MHz, $CDCl_3$) δ 157.1 (q, $^{2}J_{C\text{-}F}$ = 38 Hz), 136.5, 133.6, 130.8, 128.7, 128.5, 126.4, 115.9 (q, $^{1}J_{C\text{-}F}$ = 287 Hz), 42.0, 18.9. **HRMS** m/z: $[M + NH_4]^+$ calc'd for $[C_{10}H_{14}F_3N_2O]^+$ expect 235.1053; found 235.1054.

2,2,2-Trifluoro-*N*-(2-methoxybenzyl)acetamide (2c)

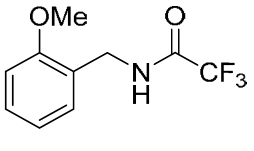

Following general procedure A with 2-methoxybenzylamine (288 mg, 2.1 mmol), stirred for 4 hours and purified by washing with Pet. Ether (40-60) **2c** was isolated as white crystals (281 mg, 1.2 mmol, 63%).

^{1}H NMR (600 MHz, $CDCl_3$) δ 7.34 (dt, *J* = 1.4, 7.7 Hz, 1H), 7.27 (dd, *J* = 1.5, 7.5 Hz, 1H), 6.96 (br s, 1H), 6.96 (dt, *J* = 0.7, 7.4 Hz, 1H), 6.93 (d, *J* = 8.3 Hz, 1H), 4.53 (d, *J* = 6.0 Hz, 2H), 3.89 (s, 3H); **^{13}C NMR** (151 MHz, $CDCl_3$) δ 157.6, 156.8 (q, $^{2}J_{C\text{-}F}$ = 37.0 Hz), 130.1, 129.8, 123.9, 120.9, 116.0 (q, $^{1}J_{C\text{-}F}$ = 286.6 Hz), 110.5, 55.4, 40.1; **HRMS** m/z: $[M + H]^+$ calc'd for $[C_{10}H_{11}F_3NO_2]^+$ expect 234.0736; found 234.0736.

***N*-(2-chlorobenzyl)-2,2,2-trifluoroacetamide (2d)**

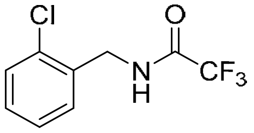

Following general procedure C with 2-chlorobenzylamine (850 mg, 6.0 mmol), stirred for 2 hours and purified by washing with Pet. Ether (40-60) **2d** was isolated as white crystals (941 mg, 4.0 mmol, 71%).

^{1}H NMR (600 MHz, $CDCl_3$) δ 7.61 (br s, 1H), 7.37 (dd, *J* = 1.7, 7.5 Hz, 1H), 7.29-7.01 (m, 3H), 4.54 (d, 6.1 Hz, 2H); **^{13}C NMR** (151 MHz, $CDCl_3$) δ 157.6 (q, $^{2}J_{C\text{-}F}$ = 37 Hz), 133.5, 133.4, 129.7, 129.7, 129.5, 127.2, 115.9 (q, $^{1}J_{C\text{-}F}$ = 287 Hz), 41.6; **HRMS** m/z: $[M + NH_4]^+$ calc'd for $[C_9H_{11}ClF_3N_2O]^+$ expect 255.0507; found 255.0509.

2,2,2-Trifluoro-*N*-(3-fluorobenzyl)acetamide (2e)

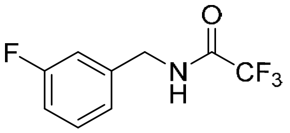

Following general procedure C with 3-fluorobenzylamine (275 mg, 2.2 mmol), stirred for 2 hours and purified by washing with Pet. Ether (40-60) **2e** was isolated as white crystals (302 mg, 1.4 mmol, 68%).

^{1}H NMR (600 MHz, $CDCl_3$) δ 7.73 (br s, 1H), 7.30 (dt, *J* = 7.9, 6.1 Hz, 1H), 7.03 (d, *J* = 7.9 Hz, 1H), 7.00-6.95 (m, 2H), 4.44 (d, *J* = 6.3 Hz); **^{13}C NMR** (151 MHz, $CDCl_3$) δ 162.9 (d, $^{1}J_{C\text{-}F}$ = 245.1 Hz), 157.7 (q, $^{2}J_{C\text{-}F}$ = 37.6 Hz), 138.5 (d, $^{3}J_{C\text{-}F}$ = 7 Hz), 130.4 (d, $^{3}J_{C\text{-}F}$ = 8 Hz), 123.3 (d, $^{4}J_{C\text{-}F}$ = 2.9 Hz), 115.8 (q, $^{1}J_{C\text{-}F}$ = 287.0

Hz), 114.9 (d, $^{2}J_{C-F}$ = 21.1 Hz), 114.6 (d, $^{2}J_{C-F}$ = 22.2 Hz), 43.1; **HRMS** m/z: $[M + H]^+$ calc'd for $[C_9H_8F_4NO]^+$ expect 222.0542; found 222.0542.

2,2,2-trifluoro-N-(2-(hydroxymethyl)benzyl)acetamide (2f)

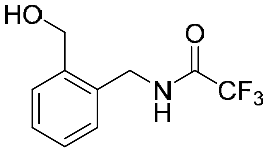

To a stirred solution of 2-cyanobenzaldehyde (2.6g, 20 mmol) in THF (40 ml) at 0°C was added 10.4 ml of a 2.4M THF solution of $LiAlH_4$ (25 mmol). The reaction was stirred at rt for 3h then quenched by addition of water at 0 °C. The solvent was removed and the residue taken up in CH_2Cl_2, dried (Na_2SO_4) and evaporated to give the crude amino alcohol. This was taken up in methanol (10 ml) and ethyl trifluoroacetate (2.4 m, 20.2 mmol) was added at rt and the reaction stirred for 3h. After this time the solvent was removed *in vacuo* to give the title compound as a yellowish solid (4.4g, 18.8 mmol, 94% over two steps).

^{1}H NMR (600 MHz, $CDCl_3$) δ 7.97 (br, 1H), 7.42 – 7.29 (m, 4H), 4.75 (s, 2H), 4.58 (d, *J* = 5.9 Hz, 2H), 2.83 (br, 1H); **^{13}C NMR** (151 MHz, $CDCl_3$) δ 156.8 (q, *J* = 36.9 Hz), 138.4, 135.5, 130.6, 129.9, 129.2, 128.8, 115.9 (q, *J* = 287.6 Hz), 64.1, 41.8. **HRMS**: m/z: $[M + Na]^+$ calc'd for $[C_{10}H_{10}F_3NO_2Na]^+$ expect 256.0556; found 256.0551.

Methyl 2-((2,2,2-trifluoroacetamido)methyl)benzoate (2g)

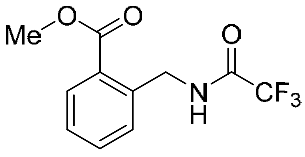

A flask was charged with methyl 2-(aminomethyl)benzoate hydrochloride salt (424 mg, 2.1 mmol) and CH_2Cl_2 (5 mL) and cooled to -78 °C. Trifluoroacetic anhydride (0.28 mL, 2.0 mmol) was added dropwise over 2 mins followed by the dropwise addition of triethylamine (0.66 mL, 4.8 mmol) over 5 mins. The reaction mixture was warmed to rt and stirred for 18 h before being diluted with CH_2Cl_2 (5 mL) and quenched with 1M HCl (5 mL). The organic layer was separated, dried ($MgSO_4$), and concentrated *in vacuo*. The crude product was purified by silica gel chromatography with Pet. Ether (40-60):EtOAc (19:1) to yield 2g as white crystals (302 mg, 1.2 mmol, 58%).

^{1}H NMR (600 MHz, $CDCl_3$) δ 8.01 (d, *J* = 7.8 Hz, 1H), 7.88 (br s, 1H), 7.54-7.50 (m, 2H), 7.42-7.39 (m, 1H), 4.67 (d, *J* = 6.7 Hz, 2H), 3.93 (s, 3H); **^{13}C NMR** (151 MHz, $CDCl_3$) δ 168.0, 156.8 (q, $^{2}J_{C-F}$ = 37 Hz), 137.8, 133.3, 131.9, 131.2, 128.8, 128.5, 115.8 (q, $^{1}J_{C-F}$ = 288 Hz), 52.5, 42.9; **HRMS** m/z: $[M + H]^+$ calc'd for $[C_{11}H_{11}F_3NO_3]^+$ expect 262.0691; found 262.0687.

***N*-benzyl-2,2,2-trifluoroacetamide (2h)**

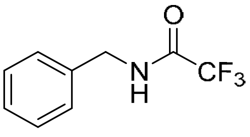

Following general procedure C with benzylamine (268 mg, 2.5 mmol), stirred for 4 hours and purified by washing with Pet. Ether (40-60) **2h** was isolated as white crystals (436 mg, 2.1 mmol, 89%).

^{1}H NMR (600 MHz, $CDCl_3$) δ 7.39-7.33 (m, 4H), 7.28 (dd, J = 1.5, 8.3 Hz, 2H), 4.47 (d, J = 6.1 Hz, 2H); **^{13}C NMR** (151 MHz, $CDCl_3$) δ 157.4 (q, $^{2}J_{C\text{-}F}$ = 37 Hz), 136.0, 128.9, 128.1, 127.9, 115.9 (q, $^{1}J_{C\text{-}F}$ = 285 Hz), 43.8.

Data is in accordance with those previously reported.[2]

(S)-2,2,2-Trifluoro-*N*-(1-phenylethyl)acetamide (2i)

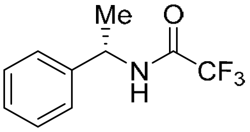

Following general procedure C with (S)-1-phenylethan-1-amine (267 mg, 2.2 mmol), stirred for 2 hours and purified by washing with Pet. Ether (40-60) **2i** was isolated as white crystals (374 mg, 1.7 mmol, 86%).

^{1}H NMR (600 MHz, $CDCl_3$) δ 7.40-7.37 (m, 2H), 7.34-7.32 (m, 3H), 6.99 (br s, 1H), 5.14 (quint, J = 7.3 Hz, 1H), 1.58 (d, J = 7.1 Hz, 3H); **^{13}C NMR** (151 MHz, $CDCl_3$) δ 156.4 (q, $^{2}J_{C\text{-}F}$ = 37 Hz), 141.1, 128.9, 128.1, 126.2, 115.8 (q, $^{1}J_{C\text{-}F}$ = 287 Hz), 49.8, 21.0.

Data is in accordance with those previously reported.[3]

2,2,2-Trifluoro-*N*-(2-fluorobenzyl)acetamide (2j)

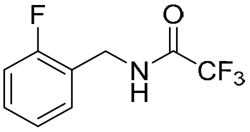

Following general procedure C with 2-fluorobenzylamine (413 mg, 3.3 mmol), stirred for 18 hours and purified by washing with Pet. Ether (40-60) **2j** was isolated as white crystals (485 mg, 2.2 mmol, 73%).

^{1}H NMR (600 MHz, $CDCl_3$) δ 7.33-7.30 (m, 2H), 7.15-7.06 (m, 3H), 4.55 (d, J = 6.1 Hz, 2H); **^{13}C NMR** (151 MHz, $CDCl_3$) δ 160.9 (d, $^{1}J_{C\text{-}F}$ = 246.3 Hz), 157.3 (q, $^{2}J_{C\text{-}F}$ = 37.3 Hz), 130.3 (d, $^{3}J_{C\text{-}F}$ = 3.8 Hz), 130.2 (d, $^{2}J_{C\text{-}F}$ = 8.1 Hz), 124.5 (d, $^{3}J_{C\text{-}F}$ = 3.6 Hz), 123.0 (d, $^{2}J_{C\text{-}F}$ = 14.7 Hz), 115.8 (q, $^{1}J_{C\text{-}F}$ = 289 Hz), 115.6 (d, $^{2}J_{C\text{-}F}$ = 21 Hz), 37.9 (d, $^{3}J_{C\text{-}F}$ = 4 Hz). **HRMS** m/z: $[M + NH_4]^+$ calc'd for $[C_9H_{11}F_4N_2O]^+$ expect 239.0802; found 239.0804.

2,2,2-Trifluoro-*N*-(pyridin-2-ylmethyl)acetamide (2k)

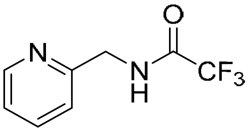

A flask was charged with 2-picolylamine (324 mg, 3.0 mmol), CH_2Cl_2 (5 mL) and triethylamine (0.42 mL, 3.3 mmol) before cooling to 0 °C. Trifluoroacetic anhydride (0.42 mL, 3.0 mmol) was added dropwise over 5 mins with rapid stirring. The reaction was warmed to rt and stirred for 2 hours before being diluted with CH_2Cl_2 (5 mL) and quenched with H_2O (5 mL). The organic layer was separated and washed with saturated $NaHCO_3$ (5 mL) before being dried ($MgSO_4$) and solvents removed *in vacuo.* The crude product was purified by silica gel chromatography with Pet. Ether (40-60):EtOAc (6:4) to yield **2k** as a white solid (498 mg, 2.4 mmol, 81%).

^{1}**H NMR** (600 MHz, $CDCl_3$) δ 8.61 (br s, 1H), 8.49 (d, *J* = 4.9 Hz, 1H), 7.68 (dt, *J* = 1.7, 7.6 Hz, 1H), 7.27 (d, *J* = 7.9 Hz, 1H), 7.22 (dd, *J* = 5.3, 7.5 Hz, 1H), 4.57 (d, *J* = 5.2 Hz, 2H); ^{13}**C NMR** (151 MHz, $CDCl_3$) δ 157.3 (q, $^2J_{C-F}$ = 37 Hz), 154.1, 148.9, 137.2, 122.9, 122.2, 115.9 (q, $^1J_{C-F}$ = 287 Hz), 44.1; **HRMS** m/z: $[M + H]^+$ calc'd for $[C_8H_8F_3N_2O]^+$ expect 205.0583; found 205.0574.

N-(2-(trifluoromethyl)benzyl)methanesulfonamide (2m)

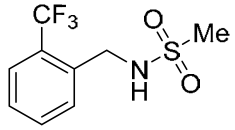

A flask was charged with 2-trifluoromethyl benzylamine (525 mg, 3.0 mmol), THF (10 mL) and triethylamine (0.46 mL, 3.3 mmol) before cooling to 0 °C. Mesyl chloride (0.26 mL, 3.3 mmol) was added dropwise over 5 mins with rapid stirring. The reaction was warmed to rt and stirred for 18 h before being quenched with 1M HCl (5 mL). The organic layer was separated and washed with a further portion of 1M HCl (5 mL) and H_2O (5 mL) before being dried ($MgSO_4$) and solvents removed *in vacuo.* The crude product was washed with Pet. Ether (40-60) to yield **2m** as a white solid (706 mg, 2.8 mmol, 93%).

^{1}**H NMR** (600 MHz, $CDCl_3$) δ 7.64 (d, *J* = 7.7 Hz, 1H), 7.62 (d, *J* = 7.7 Hz, 1H), 7.52 (t, *J* = 7.7 Hz, 1H), 7.38 (t, *J* = 7.7 Hz, 1H), 5.39 (br s, 1H), 4.44 (d, *J* = 6.6 Hz, 2H), 2.86 (s, 3H); ^{13}**C NMR** (151 MHz, $CDCl_3$) δ 135.4, 132.4, 130.3, 128.0, 127.9 (q, $^2J_{C-F}$ = 30 Hz), 126.1 (q, $^3J_{C-F}$ = 6 Hz), 124.3 (q, $^1J_{C-F}$ = 273 Hz), 43.4 (q, $^4J_{C-F}$ = 2 Hz), 40.6; **HRMS** m/z: $[M + H]^+$ calc'd for $[C_9H_8B_2NO_2F_2S]^+$ expect 254.0430; found 254.0420.

4-Methyl-*N*-(2-(trifluoromethyl)benzyl)benzenesulfonamide (2n)

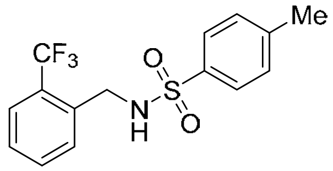

A solution of 2-trifluoromethyl benzylamine (438 mg, 2.5 mmol) and triethylamine (0.35 mL, 2.5 mmol) in CH_2Cl_2 (5 mL) was prepared. This was added dropwise over 5 mins to a suspension of tosyl chloride (572 mg, 3.0 mmol) in CH_2Cl_2 (5 mL) at 0 °C with rapid stirring. The reaction was warmed to rt and stirred for 18 h before being quenched with 1M HCl (5 mL). The organic layer was separated and washed with a further portion of 1M HCl (5 mL) and H_2O (5 mL) before being dried ($MgSO_4$) and solvents removed *in vacuo.* The crude product was washed with Pet. Ether (40-60) to yield **2n** as a white solid (491 mg, 1.5 mmol, 60%)

^{1}**H NMR** (600 MHz, $CDCl_3$) δ 7.74 (d, *J* = 8.2 Hz, 2H), 7.58-7.56 (m, 2H), 7.45 (t, *J* = 7.6 Hz, 1H), 7.33 (t, *J* = 7.6 Hz, 1H), 7.27 (d, *J* = 7.3 Hz, 2H), 5.40 (br s, 1H), 4.29 (d, *J* = 6.5 Hz, 2H), 2.41 (s, 3H); ^{13}**C NMR** (151 MHz, $CDCl_3$) δ 143.6, 136.8, 135.1, 132.2, 130.4, 129.7, 127.8 (q, $^2J_{C-F}$ = 30 Hz), 127.8, 127.1, 125.8 (q, $^3J_{C-F}$ = 6 Hz), 124.2 (q, $^1J_{C-F}$ = 273 Hz), 43.5, 21.5; **HRMS** m/z: $[M + H]^+$ calc'd for $[C_{15}H_{15}F_3NO_2S]^+$ expect 330.0776; found 333.0778.

N-(2-(trifluoromethyl)benzyl)acetamide (2o)

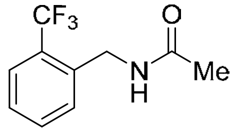

A flask was charged with 2-trifluoromethyl benzylamine (438 mg, 2.5 mmol), CH_2Cl_2 (5 mL) and triethylamine (0.35 mL, 2.5 mmol) before cooling to 0 °C. Acetyl chloride (0.18 mL, 2.5 mmol) was added dropwise over 5 mins with rapid stirring. The reaction was warmed to rt and stirred for 2 h before being diluted with CH_2Cl_2 (5 mL) and quenched with 1M HCl (2 mL). The organic layer was separated and washed with a further portion of 1M HCl (2 mL) and saturated $NaHCO_3$ (2 mL) before being dried ($MgSO_4$) and solvents removed *in vacuo.* The crude product was washed with Pet. Ether (40-60) to yield **2o** as a white solid (488 mg, 2.2 mmol, 90%).

^{1}H NMR (600 MHz, $CDCl_3$) δ 7.60 (d, J = 7.8 Hz, 1H), 7.48-7.45 (m, 2H), 7.33 (t, J = 7.3 Hz, 1H), 6.50 (br s, 1H), 4.53 (d, J= 5.8 Hz, 2H), 1.95 (s, 3H); **^{13}C NMR** (151 MHz, $CDCl_3$) δ 170.3, 136.8 (q, $^3J_{C\text{-}F}$ = 1 Hz), 132.2, 130.3, 128.0 (q, $^2J_{C\text{-}F}$ = 30 Hz), 127.4, 125.8 (q, $^3J_{C\text{-}F}$ = 6 Hz), 124.4 (q, $^1J_{C\text{-}F}$ = 274 Hz), 40.0 (q, $^4J_{C\text{-}F}$ = 2 Hz), 22.9; **HRMS** m/z: $[M + H]^+$ calc'd for $[C_{10}H_{11}F_3NO]^+$ expect 218.0787; found 218.0789.

***tert*-Butyl (2-(trifluoromethyl)benzyl)carbamate (2p)**

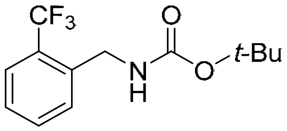

A flask was charged with 2-trifluoromethyl benzylamine (350 mg, 2.0 mmol) and EtOAc (10 mL). Boc anhydride (524 mg, 2.4 mmol) was added at rt and the reaction stirred overnight open to air. Volatiles were removed *in vacuo* and the crude product purified by silica gel chromatography with Pet. Ether (40-60):EtOAc (19:1) to yield **2p** as a white solid (290 mg, 1.1 mmol, 53%).

^{1}H NMR (600 MHz, $CDCl_3$) δ 7.63 (d, J = 7.8 Hz, 1H), 7.58 (d, J = 7.6 Hz, 1H), 7.52 (t, J = 7.8 Hz, 1H), 7.36 (t, J = 7.6 Hz, 1H), 4.99 (br s, 1H), 4.50 (d, J = 6.0 Hz, 2H), 1.46 (s, 9H); **^{13}C NMR** (151 MHz, $CDCl_3$) δ 155.8, 137.5, 132.2, 129.9, 127.9 (q, $^2J_{C\text{-}F}$ = 32 Hz), 127.3, 125.9 (q, $^3J_{C\text{-}F}$ = 6 Hz), 124.4 (q, $^1J_{C\text{-}F}$ = 275 Hz), 79.7, 41.1, 28.3.

Data is in accordance with those previously reported.[4]

***N*-(2-(trifluoromethyl)benzyl)pivalamide (2q)**

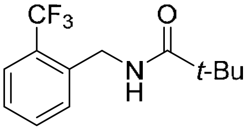

A flask was charged with 2-trifluoromethyl benzylamine (385 mg, 2.2 mmol), CH_2Cl_2 (5 mL) and triethylamine (0.32 mL, 2.3 mmol) before cooling to 0 °C. Pivaloyl chloride (0.31 mL, 2.6 mmol) was added dropwise over 5 mins with rapid stirring. The reaction was warmed to rt and stirred for 18 h before being diluted with CH_2Cl_2 (5 mL) and quenched with 1M HCl (2 mL). The organic layer was separated and washed with a further portion of 1M HCl (2 mL) and saturated $NaHCO_3$ (2 mL) before being dried ($MgSO_4$) and solvents removed *in vacuo.* The crude product was washed with Pet. Ether (40-60) to yield **2q** as a white solid (375 mg, 1.4 mmol, 64%).

^{1}H NMR (600 MHz, $CDCl_3$) δ 7.66 (d, *J* = 7.7 Hz, 1H), 7.54-7.53 (m, 2H), 7.40-7.38 (m, 1H), 6.00 (br s, 1H), 4.62 (d, *J* = 6.0, 2H), 1.21 (s, 9H); **^{13}C NMR** (151 MHz, $CDCl_3$) δ 178.4, 137.1, 132.2, 129.8, 127.9 (q, $^2J_{C\text{-}F}$ = 31 Hz), 127.3, 125.9 (q, $^3J_{C\text{-}F}$ = 6 Hz), 124.5 (q, $^1J_{C\text{-}F}$ = 274 Hz), 40.0, 38.6, 27.4; **HRMS** m/z: [M + H]$^+$ calc'd for $[C_{13}H_{17}NOF_3]^+$ expect 260.1262; found 260.1271.

(E)-1-(2-nitrovinyl)-2-(trifluoromethyl)benzene

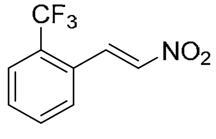

2-(trifluoromethyl)benzaldehyde (3.79 ml; 28.7 mmol; 1eq) and ammonium acetate (885 mg; 11.48 mmol; 0.4eq) were dissolved in acetic acid (20 ml). Then nitromethane (4.66 ml; 86.1 mmol; 3eq) was added and the mixture was refluxed for 16 hours. The solvent was then evaporated under reduced pressure and the crude was retaken into CH_2Cl_2 (50 ml). The organics were then washed with sat. $NaHCO_3$ (2x50 ml), dried with $MgSO_4$ and concentrated. The crude was then purified by recrystalisation from methanol to give the final product as yellow crystals (2.83 g; 45%).

^{1}H NMR (600 MHz, $CDCl_3$) δ 8.39 (dd, *J* = 13.5, 1.7 Hz, 1H), 7.81 (d, *J* = 7.2 Hz, 1H), 7.72 – 7.59 (m, 3H), 7.51 (d, *J* = 13.5 Hz, 1H); **^{13}C NMR** (151 MHz, $CDCl_3$) δ 129.9 (q, *J* = 30.9 Hz), 126.8 (q, *J* = 5.4 Hz), 123.6 (q, *J* = 274.0 Hz); m.p. 58-60 °C (lit. 58-59 °C).

The data corresponds to that reported in the literature.[5]

2,2,2-trifluoro-N-(2-(trifluoromethyl)phenethyl)acetamide (4a)

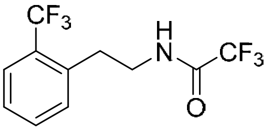

(E)-1-(2-nitrovinyl)-2-trifluoromethyl)benzene (750 mg; 3.45 mmol; 1eq) was dissolved in dry THF (5 ml) under argon atmosphere and cooled to 0 °C. A solution of $LiAlH_4$ (2.4M in THF; 4.31 ml; 10.35 mmol; 3eq) was added dropwise at this temperature and the reaction mixture was then stirred at room temperature for 16 hours. The reaction was carefully quenched with water at 0 °C and then acidified with conc. HCl. The mixture was washed with diethyl ether (2x30 ml) and the aqueous layer was brought to basic pH with 10% NaOH solution. It was extracted with CH_2Cl_2 (2x40 ml) and the organic layer was dried with $MgSO_4$ and concentrated to give a light orrange oil (622 mg). The crude was dissolved in dry CH_2Cl_2 (10 ml) and mixture was put under argon atmosphere. Then trifluoroacetic anhydride (0.46 ml; 3.29 mmol; 1eq) and triethylamine (0.92 ml; 6.58 mmol; 2eq) were added dropwise, and the reaction was left stirring at room temperature for 16 hours. The mixture was washed with sat. $NaHCO_3$ (50 ml), the organic layer was dried with $MgSO_4$ and concentrated to give a brown oil. The crude was then purified by column chromatography (SiO_2, 60% CH_2Cl_2 in Petroleum Ether 40-60 °C) to give the final product as an off-white solid (140 mg; 15% over two steps).

^{1}H NMR (600 MHz, $CDCl_3$) δ 7.67 (d, *J* = 7.8 Hz, 1H), 7.52 (t, *J* = 7.6 Hz, 1H), 7.37 (m, 2H), 6.73 (br, 1H), 3.63 (dd, *J* = 6.8, 6.5 Hz, 2H), 3.09 (t, *J* = 7.3 Hz, 2H); **^{13}C NMR** (151 MHz, $CDCl_3$) δ 157.4 (q, *J* = 37.1 Hz), 136.2 (d, *J* = 1.6 Hz), 132.1, 131.5, 128.9 (q, *J* = 29.8 Hz), 127.1, 126.3 (q, *J* = 5.7 Hz), 124.4 (q, *J* = 273.5 Hz), 115.8 (q, *J* = 287.7 Hz), 40.8, 31.8. **HRMS**: m/z: [M - H]$^{•+}$ calc'd for $[C_{11}H_8NOF_6]^{•+}$ expect 284.0510; found 284.0498.

N-(2-(trifluoromethyl)phenethyl)methanesulfonamide (4b)

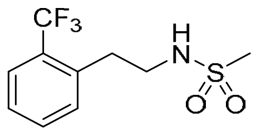

(E)-1-(2-nitrovinyl)-2-(trifluoromethyl)benzene (600 mg; 2.76 mmol; 1eq) was dissolved in dry THF (5 ml) under argon atmosphere and cooled to 0 °C. A solution of $LiAlH_4$ (2.4M in THF; 3.45 ml; 8.28 mmol; 3eq) was added dropwise at this temperature and the reaction mixture was then stirred at room temperature for 4 hours. The reaction was carefully quenched with water at 0 °C and then acidified with conc. HCl. The mixture was washed with diethyl ether (2x30 ml) and the aqueous layer was brought to basic pH with 10% NaOH solution. It was extracted with CH_2Cl_2 (2x40 ml) and the organic layer was dried with $MgSO_4$ and concentrated to give a light yellow oil (522 mg). The crude was dissolved in dry CH_2Cl_2 (10 ml) and mixture was put under argon atmosphere. Then methanesulfonyl chloride (0.12 ml; 1.59 mmol; 1.1eq) and triethylamine (0.40 ml; 2.89 mmol; 2eq) were added dropwise, and the reaction was left stirring at room temperature for 16 hours. The mixture was washed with sat. $NaHCO_3$ (50 ml), the organic layer was dried with $MgSO_4$ and concentrated to give a yellow oil. The crude was then purified by column chromatography (SiO_2, 30% to 40% EtOAc in Petroleum Ether 40-60 °C) to give the final product as an off-white solid (291 mg; 39% over two steps).

^{1}H NMR (600 MHz, $CDCl_3$) δ 7.65 (d, *J* = 7.9 Hz, 1H), 7.51 (t, *J* = 7.5 Hz, 1H), 7.41 (d, *J* = 7.7 Hz, 1H), 7.35 (t, *J* = 7.7 Hz, 1H), 4.77 (br t, *J* = 5.8 Hz, 1H), 3.39 (dt, *J* = 7.4, 6.7 Hz, 2H), 3.08 (t, *J* = 7.4 Hz, 2H), 2.88 (s, 3H); **^{13}C NMR** (151 MHz, $CDCl_3$) δ 136.4 (d, *J* = 1.5 Hz), 132.1, 131.8 (s), 128.8 (q, *J* = 29.8 Hz), 127.0 (s), 126.3 (q, *J* = 5.7 Hz), 124.5 (q, *J* = 273.7 Hz), 44.1, 40.3, 33.5. **HRMS:** m/z: $[M + H]^+$ calc'd for $[C_{10}H_{13}NO_2F_3S]^+$ expect 268.0619; found 268.0618.

tert-butyl (2-(trifluoromethyl)phenethyl)carbamate (4c)

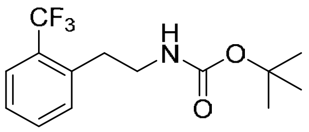

(E)-1-(2-nitrovinyl)-2-(trifluoromethyl)benzene (750 mg; 3.45 mmol; 1eq) was dissolved in dry THF (5 ml) under argon atmosphere and cooled to 0 °C. A solution of $LiAlH_4$ (2.4M in THF; 4.31 ml; 10.35 mmol; 3eq) was added dropwise at this temperature and the reaction mixture was then stirred at room temperature for 16 hours. The reaction was carefully quenched with water at 0 °C and then acidified with conc. HCl. The mixture was washed with diethyl ether (2x30 ml) and the aqueous layer was brought to basic pH with 10% NaOH solution. It was extracted with CH_2Cl_2 (2x40 ml) and the organic layer was dried with $MgSO_4$ and concentrated to give a light orange oil (619 mg). Then di-tert-butyl dicarbonate (718 mg; 3.29 mmol; 1eq) and triethylamine (0.92 ml; 6.58 mmol; 2eq) were added dropwise, and the reaction was left stirring at room temperature for 16 hours. The mixture was washed with sat. $NaHCO_3$ (50 ml), the organic layer was dried with $MgSO_4$ and concentrated to give a brown oil. The crude was then purified by column chromatography (SiO_2, 85% CH_2Cl_2 in Petroleum Ether 40-60 °C) to give the final product as an off-white solid (241 mg; 25% over two steps).

^{1}H NMR (600 MHz, $CDCl_3$) δ 7.64 (d, *J* = 7.9 Hz, 1H), 7.49 (t, *J* = 7.5 Hz, 1H), 7.38 (d, *J* = 7.2 Hz, 1H), 7.32 (t, *J* = 7.6 Hz, 1H), 4.71 (br, 1H), 3.62 – 3.29 (m, 2H), 3.00 (t, *J* = 6.9 Hz, 2H), 1.45 (s, 9H); **^{13}C NMR** (151 MHz, $CDCl_3$) δ 155.8, 137.6, 131.8, 131.6, 128.8 (q, *J* = 29.7 Hz), 126.5, 126.0 (dq *J* = 5.6 Hz), 124.5 (q, *J* = 273.6 Hz), 79.3, 41.6, 33.1, 28.4. **HRMS**: m/z: $[M + H]^+$ calc'd for $[C_{14}H_{19}F_3NO_2]^+$ expect 290.1362; found 290.1375.

2,2,2-trifluoro-N-(2-iodophenethyl)acetamide (4d)

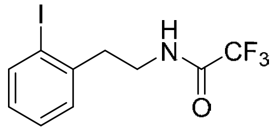

2-iodoacetonitrile (729 mg; 3 mmol; 1eq) was dissolved in dry THF (10 ml) under argon atmosphere and cooled to 0 °C. A solution of BH_3 (1M in THF; 6 ml; 6 mmol; 2eq) was added dropwise at this temperature and the reaction mixture was then stirred at room temperature for 16 hours. The reaction was quenched with a 3M $HCl_{(aq)}$ solution and 10% $NaOH_{(aq)}$ was added until basic pH was reached. The mixture was extracted with EtOAc (2x50 ml) and the organic layer was dried with $MgSO_4$ and concentrated to give a colourless oil. The crude was dissolved in dry CH_2Cl_2 (20 ml) and mixture was put under argon atmosphere. Then trifluoroacetic anhydride (0.5 ml; 3.6 mmol; 1.2eq) and triethylamine (0.84 ml; 6 mmol; 2eq) were added dropwise, and the reaction was left stirring at room temperature for 3 hours. The mixture was washed with sat. $NaHCO_3$ (50 ml), the organic layer was dried with $MgSO_4$ and concentrated to give an orange oil. The crude was then purified by column chromatography (SiO_2, 10% EtOAc in Petroleum Ether 40-60 °C) to give the final product as an off-white solid (445 mg; 43% over two steps).

^{1}H NMR (600 MHz, $CDCl_3$) δ 7.85 (d, *J* = 7.9 Hz, 1H), 7.32 (t, *J* = 7.5 Hz, 1H), 7.21 (dd, *J* = 7.6, 1.6 Hz, 1H), 6.96 (t, *J* = 7.6 Hz, 1H), 6.66 (br, 1H), 3.62 (dt, *J* = 6.8, 6.8 Hz, 2H), 3.04 (t, *J* = 7.2 Hz, 2H); **^{13}C NMR** (151 MHz, $CDCl_3$) δ 157.4 (q, *J* = 36.9 Hz), 140.4, 139.9, 130.0, 128.9, 128.7, 115.8 (q, *J* = 287.8 Hz), 100.4, 39.8, 39.4. **HRMS**: m/z: $[M + H]^+$ calc'd for $[C_{10}H_{10}NOF_3I]^+$ expect 343.9759; found 343.9759.

(E)-1-fluoro-3-(2-nitrovinyl)benzene

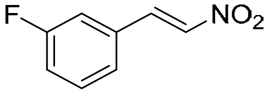

3-fluorobenzaldehyde (1.71 ml; 16.1 mmol; 1eq) and ammonium acetate (497 mg; 6.44 mmol; 0.4eq) were dissolved in acetic acid (20 ml). Then nitromethane (2.62 ml; 48.3 mmol; 3eq) was added and the mixture was refluxed for 16 hours. The solvent was then evaporated under reduced pressure and the crude was retaken into CH_2Cl_2 (50 ml). The organics were then washed with sat. $NaHCO_3$ (2x50 ml), dried with $MgSO_4$ and concentrated. The crude was then crushed out from methanol to give the final product as a green solid (654 mg; 24%).

^{1}H NMR (600 MHz, $CDCl_3$) δ 7.97 (d, *J* = 13.7 Hz, 1H), 7.57 (d, *J* = 13.7 Hz, 1H), 7.45 (td, *J* = 8.0, 5.7 Hz, 1H), 7.35 (d, *J* = 7.7 Hz, 1H), 7.28 – 7.24 (m, 1H), 7.21 (ddd, *J* = 8.3, 5.1, 1.8 Hz, 1H); **^{13}C NMR** (151 MHz, $CDCl_3$) δ 163.0 (d, *J* = 248.6 Hz), 138.1, 137.6 (d, *J* = 2.8 Hz), 132.1 (d, *J* = 7.9 Hz), 131.1 (d, *J* = 8.3 Hz), 125.1 (d, *J* = 3.0 Hz), 119.1 (d, *J* = 21.3 Hz), 115.4 (d, *J* = 22.4 Hz).

The data corresponds to that reported in the literature.[6]

2,2,2-trifluoro-N-(3-fluorophenethyl)acetamide (4e)

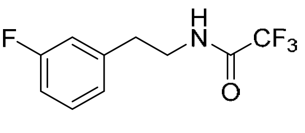

(E)-1-fluoro-3-(2-nitrovinyl)benzene (501 mg; 3 mmol; 1eq) was dissolved in dry THF (10 ml) under argon atmosphere and cooled to 0 °C. A solution of $LiAlH_4$ (2.4M in THF; 3.75 ml; 9 mmol; 3eq) was added dropwise at this temperature and the reaction mixture was then stirred at room temperature

for 16 hours. The reaction was carefully quenched with water at 0 °C and then acidified with conc. HCl. The mixture was washed with diethyl ether (2x30 ml) and the aqueous layer was brought to basic pH with 10% NaOH solution. It was extracted with CH_2Cl_2 (2x40 ml) and the organic layer was dried with $MgSO_4$ and concentrated to give an orange oil (260 mg). The crude was dissolved in dry CH_2Cl_2 (10 ml) and mixture was put under argon atmosphere. Then trifluoroacetic anhydride (0.31 ml; 2.24 mmol) and triethylamine (0.52 ml; 3.74 mmol) were added dropwise, and the reaction was left stirring at room temperature for 3 hours. The mixture was washed with sat. $NaHCO_3$ (50 ml), the organic layer was dried with $MgSO_4$ and concentrated to give an orange oil. The crude was then purified by column chromatography (SiO_2, 60% CH_2Cl_2 in Petroleum Ether 40-60 °C) to give the final product as an off-white solid (189 mg; 28% over two steps).

^{1}H NMR (600 MHz, $CDCl_3$) δ 7.35 – 7.22 (m, 1H), 6.96 (m, 2H), 6.91 (ddd, *J* = 9.7, 1.8, 1.8 Hz, 1H), 6.54 (br, 1H), 3.62 (dt, *J* = 6.5, 6.4 Hz, 2H), 2.90 (t, *J* = 7.1 Hz, 2H); **^{13}C NMR** (151 MHz, $CDCl_3$) δ 163.0 (d, *J* = 246.6 Hz), 157.3 (q, *J* = 37.1 Hz), 140.1 (d, *J* = 7.2 Hz), 130.4 (d, *J* = 8.4 Hz), 124.3 (d, *J* = 2.9 Hz), 115.7 (q, *J* = 287.7 Hz), 115.5 (d, *J* = 21.2 Hz), 113.9 (d, *J* = 21.0 Hz), 40.8, 34.7. **HRMS**: m/z: $[M + H]^+$ calc'd for $[C_{10}H_{10}NOF_4]^+$ expect 236.0699; found 236.0704.

N-(2-bromophenethyl)-2,2,2-trifluoroacetamide (4f)

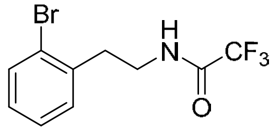

2-bromoacetonitrile (0.26 ml; 2 mmol; 1eq) was dissolved in dry THF (6 ml) under argon atmosphere and cooled to 0 °C. A solution of BH_3 (1M in THF; 4 ml; 4 mmol; 2eq) was added dropwise at this temperature and the reaction mixture was then stirred at room temperature for 16 hours. The reaction was quenched with a 3M $HCl_{(aq)}$ solution and 10% $NaOH_{(aq)}$ was added until basic pH was reached. The mixture was extracted with EtOAc (2x40 ml) and the organic layer was dried with $MgSO_4$ and concentrated to give a colourless oil. The crude was dissolved in dry CH_2Cl_2 (20 ml) and mixture was put under argon atmosphere. Then trifluoroacetic anhydride (0.33 ml; 2.4 mmol; 1.2eq) and triethylamine (0.56 ml; 4 mmol; 2eq) were added dropwise, and the reaction was left stirring at room temperature for 3 hours. The mixture was washed with sat. $NaHCO_3$ (50 ml), the organic layer was dried with $MgSO_4$ and concentrated to give an orange oil. The crude was then purified by column chromatography (SiO_2, 15% EtOAc in Petroleum Ether 40-60 °C) to give the final product as a white solid (338 mg; 57% over two steps).

^{1}H NMR (600 MHz, $CDCl_3$) δ 7.58 (dd, *J* = 8.0, 1.1 Hz, 1H), 7.29 (td, *J* = 7.5, 1.2 Hz, 1H), 7.22 (dd, *J* = 7.6, 1.7 Hz, 1H), 7.14 (td, *J* = 7.8, 1.7 Hz, 1H), 6.48 (br, 1H), 3.66 (q, *J* = 6.7 Hz, 2H), 3.06 (t, *J* = 7.0 Hz, 2H); **^{13}C NMR** (151 MHz, $CDCl_3$) δ 157.3 (q, *J* = 36.9 Hz), 137.1, 133.2, 131.0, 128.8, 127.9, 124.5, 115.7 (q, *J* = 287.9 Hz), 39.6, 35.1. **HRMS**: m/z: $[M + H]^+$ calc'd for $[C_{10}H_{10}NOF_3Br]^+$ expect 295.9898; found 295.9903.

methyl 2-(2-(2,2,2-trifluoroacetamido)ethyl)benzoate (4g)

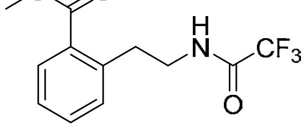

Methyl 2-(cyanomethyl)benzoate (500 mg; 2.85 mmol; 1eq) was dissolved in methanol (30 ml). Then 10% Pd/C (240 mg) was added followed by solution of HCl (4M in dioxane; 1.4 ml; 5.7 mmol; 2eq). The reaction mixture was purged with hydrogen and left stirring under hydrogen atmosphere for 16 hours at room temperature. The reaction mixture was then filtered through a pad of celite and the filtrate

concentrated to give a white solid. The solid was dissolved in trifluoroacetic anhydride (20 ml) under argon atmosphere and triethylamine (0.87 ml; 6.27 mmol; 2.2eq) was added dropwise. Reaction was stirred at room temperature for 16 hours. Solvent was evaporated, the crude was taken into EtOAc (30 ml) and washed with sat. $NaHCO_3$ (30 ml) and brine (30 ml). The organic layer was dried with $MgSO_4$ and concentrated to give a yellow oil. It was then purified by column chromatography (SiO_2, 20% EtOAc in Petroleum Ether 40-60 ^oC) to give the final product as a light yellow oil (747 mg; 95% over two steps).

^{1}H NMR (600 MHz, $CDCl_3$) δ 7.90 (dd, *J* = 7.9, 1.3 Hz, 1H), 7.51 (td, *J* = 7.6, 1.4 Hz, 1H), 7.34 (td, *J* = 7.7, 1.2 Hz, 1H), 7.31 (d, *J* = 7.7 Hz, 1H), 3.94 (s, 3H), 3.68 (dd, *J* = 6.6, 5.7 Hz, 2H), 3.34 – 3.11 (t, *J* = 6.7 Hz, 2H); **^{13}C NMR** (151 MHz, $CDCl_3$) δ 168.9, 157.4 (q, *J* = 36.7 Hz), 140.0, 132.7, 131.5, 130.7, 129.7, 127.1, 115.8 (q, *J* = 287.7 Hz), 52.5, 42.0, 32.1. **HRMS**: m/z: $[M + Na]^+$ calc'd for $[C_{12}H_{12}O_3NF_3Na]^+$ expect 298.0661; found 298.0650.

(E)-1-chloro-2-(2-nitrovinyl)benzene

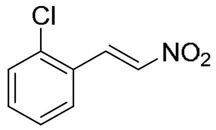

2-chlorobenzaldehyde (2 ml; 17.8 mmol; 1eq) and ammonium acetate (549 mg; 7.12 mmol; 0.4eq) were dissolved in acetic acid (20 ml). Then nitromethane (2.89 ml; 53.4 mmol; 3eq) was added and the mixture was refluxed for 16 hours. The solvent was then evaporated under reduced pressure and the crude was retaken into CH_2Cl_2 (50 ml). The organics were then washed with sat. $NaHCO_3$ (2x50 ml), dried with $MgSO_4$ and concentrated. The crude was then purified by recrystalisation from methanol to give the final product as yellow crystals (1.51 g; 46%).

^{1}H NMR (600 MHz, $CDCl_3$) δ 8.42 (d, *J* = 13.7 Hz, 1H), 7.65 – 7.58 (m, 2H), 7.52 (dd, *J* = 8.1, 1.2 Hz, 1H), 7.45 (td, *J* = 7.7, 1.6 Hz, 1H), 7.36 (ddd, *J* = 8.1, 7.4, 0.8 Hz, 1H); **^{13}C NMR** (151 MHz, $CDCl_3$) δ 138.8, 136.0, 135.1, 132.8, 130.8, 128.6, 128.5, 127.5; m.p. 47-49 ^oC (lit. 48 ^oC).

The data corresponds to that reported in the literature.[7]

N-(2-chlorophenethyl)-2,2,2-trifluoroacetamide (4h)

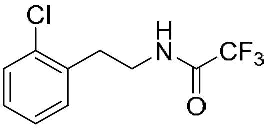

(E)-1-chloro-2-(2-nitrovinyl)benzene (918 mg; 5 mmol; 1eq) was added to a stirring solution of $LiALH_4$ (1M in THF; 20 ml; 20 mmol; 4 eq) at 0 ^oC. The mixture was warmed to room temperature and stirred overnight before cooling again to 0 °C, diluting with Et_2O (to ca. 0.1 M) and quenching slowly with water. The mixture was acidified and washed with CH_2Cl_2 (2 x 15 mL) then the aqueous basified with 10% NaOH to ca. pH 10 and extracted with CH_2Cl_2 (3 x 30 mL) and the combined organic phases were dried ($MgSO_4$) and solvents removed *in vacuo*. The crude amine was dissolved in CH_2Cl_2 (5 mL) and triethylamine (0.35 mL, 2.5 mmol, 1.2eq) and cooled to 0 °C. Trifluoroacetic anhydride (0.32 mL, 2.3 mmol, 1.1eq) was added dropwise over 5 mins with rapid stirring. The reaction was warmed to room temperature and stirred for 2 hours before being diluted with CH_2Cl_2 (to 10 mL) and quenched with 1M HCl. The organic layer was separated and washed sequentially with 1M HCl and saturated $NaHCO_3$ before being dried ($MgSO_4$) and solvents removed *in vacuo*. The crude product was washed with Petroleum Ether 40-60 ^oC to yield a white solid (461 mg, 1.8 mmol, 36% over 2 steps).

^{1}H NMR (600 MHz, $CDCl_3$) δ 7.44 – 7.36 (m, 1H), 7.25 – 7.18 (m, 3H), 6.59 (br, 1H), 3.65 (dt, *J* = 6.8, 6.7 Hz, 1H), 3.05 (t, *J* = 7.0 Hz, 1H); **^{13}C NMR** (151 MHz, $CDCl_3$) δ 157.3 (q, *J* = 36.8 Hz), 135.4, 134.1, 131.0, 129.8, 128.6, 127.2, 115.8 (q, *J* = 287.8 Hz), 39.6, 32.7. **HRMS**: m/z: $[M + H]^+$ calc'd for $[C_{10}H_{10}NOF_3Cl]^+$ expect 252.0403; found 252.0399.

2,2,2-trifluoro-N-(2-methylphenethyl)acetamide (4i)

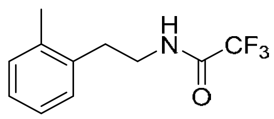

O-Tolylacetonitrile (262 mg; 2 mmol; 1eq) was dissolved in dry THF (5 ml) under argon atmosphere and cooled to 0 °C. A solution of BH_3 (1M in THF; 4 ml; 4 mmol; 2eq) was added dropwise at this temperature and the reaction mixture was then stirred at room temperature for 5 hours. The reaction was quenched with a 3M $HCl_{(aq)}$ solution and 10% $NaOH_{(aq)}$ was added until basic pH was reached. The mixture was extracted with EtOAc (2x40 ml) and the organic layer was dried with $MgSO_4$ and concentrated to give a colourless oil. The crude was dissolved in dry CH_2Cl_2 (20 ml) and mixture was put under argon atmosphere. Then trifluoroacetic anhydride (0.33 ml; 2.4 mmol; 1.2eq) and triethylamine (0.56 ml; 4 mmol; 2eq) were added dropwise, and the reaction was left stirring at room temperature for 3 hours. The mixture was washed with sat. $NaHCO_3$ (50 ml), the organic layer was dried with $MgSO_4$ and concentrated to give an orange oil. The crude was then purified by column chromatography (SiO_2, 60% to 100% CH_2Cl_2 in Petroleum Ether 40-60 °C) to give the final product as a colourless oil (211 mg; 46% over two steps).

^{1}H NMR (600 MHz, $CDCl_3$) δ 7.19 (m, 3H), 7.14 – 7.10 (m, 1H), 6.65 (br, 1H), 3.59 (dt, *J* = 6.8, 6.5 Hz, 2H), 2.92 (t, *J* = 7.4 Hz, 2H), 2.36 (s, 3H); **^{13}C NMR** (151 MHz, $CDCl_3$) δ 157.3 (q, *J* = 36.9 Hz), 136.3, 135.7, 130.7, 129.3, 127.1, 126.3, 115.8 (q, *J* = 287.8 Hz), 39.9, 32.3, 19.2. **HRMS**: m/z: $[M + H]^+$ calc'd for $[C_{11}H_{13}NOF_3]^+$ expect 232.0949; found 232.0954.

2,2,2-trifluoro-N-(2-methoxyphenethyl)acetamide (4j)

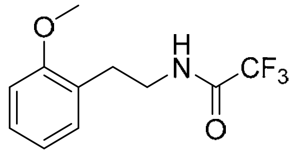

(E)-1-methoxy-2-(2-nitrovinyl)benzene (538 mg; 3 mmol; 1eq) was dissolved in dry THF (10 ml) under argon atmosphere and cooled to 0 °C. A solution of $LiAlH_4$ (2.4M in THF; 3.75 ml; 9 mmol; 3eq) was added dropwise at this temperature and the reaction mixture was then stirred at room temperature for 16 hours. The reaction was carefully quenched with water at 0 °C and then acidified with conc. HCl. The mixture was washed with diethyl ether (2x30 ml) and the aqueous layer was brought to basic pH with 10% NaOH solution. It was extracted with DCM (2x40 ml) and the organic layer was dried with $MgSO_4$ and concentrated to give an orange oil (317 mg). The crude was dissolved in dry DCM (10 ml) and mixture was put under argon atmosphere. Then trifluoroacetic anhydride (0.35 ml; 2.52 mmol; 1.2eq) and triethylamine (0.59 ml; 4.2 mmol; 2eq) were added dropwise, and the reaction was left stirring at room temperature for 3 hours. The mixture was washed with sat. $NaHCO_3$ (50 ml), the organic layer was dried with $MgSO_4$ and concentrated to give an orange oil. The crude was then purified by column chromatography (SiO_2, 15% EtOAc in Petroleum Ether 40-60 °C) to give the final product as a yellow oil (330 mg; 44% over two steps).

^{1}H NMR (600 MHz, $CDCl_3$) δ 7.28 (td, *J* = 8.1, 1.7 Hz, 1H), 7.16 (dd, *J* = 7.4, 1.7 Hz, 1H), 7.10 (br, 1H), 6.96 (td, *J* = 7.4, 1.0 Hz, 1H), 6.93 (d, *J* = 8.2 Hz, 1H), 3.88 (s, 3H), 3.58 (dt, *J* = 6.7, 5.7 Hz, 2H), 2.97 –

2.90 (t, *J* = 6.5 Hz, 2H); **^{13}C NMR** (151 MHz, $CDCl_3$) δ 157.1, 157.1 (q, *J* = 36.6 Hz), 130.8, 128.5, 126.6, 121.2, 115.9 (q, *J* = 287.9 Hz), 110.5, 55.2, 41.1, 29.3. **HRMS**: m/z: $[M + H]^+$ calc'd for $[C_{11}H_{13}NO_2F_3]^+$ expect 248.0898; found 248.0908.

N-(2-(((tert-butyldimethylsilyl)oxy)methyl)phenethyl)-2,2,2-trifluoroacetamide (4k)

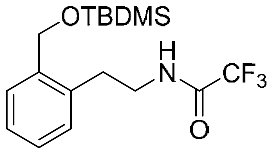

Methyl 2-(cyanomethyl)benzoate (1.5 g; 8.56 mmol; 1eq) was dissolved in dry THF (15 ml) under argon atmosphere and cooled to 0 °C. A solution of $LiAlH_4$ (2.4M in THF; 14.3 ml; 34.2 mmol; 4eq) was added dropwise at this temperature and the reaction mixture was then stirred at room temperature for 16 hours. The reaction was carefully quenched with water at 0 °C and then acidified with conc. HCl. The mixture was washed with diethyl ether (2x40 ml) and the aqueous layer was brought to basic pH with 10% NaOH solution. It was extracted with CH_2Cl_2 (2x50 ml) and the organic layer was dried with $MgSO_4$ and concentrated to give a yellow oil (668 mg). The crude was dissolved in dry methanol (40 ml) and mixture was put under argon atmosphere. Then trifluoroacetic anhydride (0.86 ml; 6.19 mmol; 1.4eq) and triethylamine (1.85 ml; 13.3 mmol; 3eq) were added dropwise, and the reaction was left stirring at room temperature for 16 hours. The solvent was removed under reduced pressure, residue was taken into EtOAc (40 ml) and washed with sat. $NaHCO_3$ (40 ml), water (40 ml) and brine (40 ml), the organic layer was dried with $MgSO_4$ and concentrated to give an orange oil. The crude was then purified by column chromatography (SiO_2, 30% EtOAc in Petroleum Ether 40-60 °C) to give a product not sufficiently pure for characterisation (567 mg), which was further dissolved in dry DMF (20 ml) under argon atmosphere. Imidazole (187 mg; 2.75 mmol; 1.2eq) and tert-butyldimethylsilyl chloride (346 mg; 2.29 mmol; 1eq) were added to the solution, and reaction mixture was left stirring at room temperature for 16 hours. CH_2Cl_2 (50 ml) was added and the mixture was washed with water (4x50 ml) to give an orange oil. The crude was purified by column chromatography (SiO_2, CH_2Cl_2) to give the final product as a colourless oil (747 mg; 11% over three steps).

^{1}H NMR (600 MHz, $CDCl_3$) δ 7.39 (dd, *J* = 7.0, 1.9 Hz, 1H), 7.34 – 7.23 (m, 2H), 7.17 (dd, *J* = 7.0, 1.8 Hz, 1H), 6.73 (br, 1H), 4.76 (s, 2H), 3.65 (dt, *J* = 6.7, 6.5 Hz, 2H), 2.98 (t, *J* = 7.1 Hz, 2H), 0.97 (s, 9H), 0.15 (s, 6H); **^{13}C NMR** (151 MHz, $CDCl_3$) δ 157.2 (q, *J* = 36.9 Hz), 138.7, 135.7, 129.5, 128.7, 128.1, 127.1, 115.8 (q, *J* = 287.7 Hz), 63.9, 40.4, 30.9, 26.0, 18.5, -5.3. **HRMS**: m/z: $[M + H]^+$ calc'd for $[C_{17}H_{27}O_2NF_3Si]^+$ expect 362.1758; found 362.1745.

2,2,2-trifluoro-N-phenethylacetamide (4l)

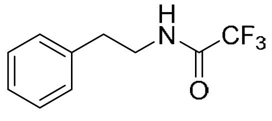

Phenethylamine (1 ml; 7.94 mmol; 1eq) was dissolved in dry CH_2Cl_2 (12 ml) and mixture was put under argon atmosphere. Then trifluoroacetic anhydride (1.32 ml; 9.53 mmol; 1.2eq) and triethylamine (2.22 ml; 15.9 mmol; 2eq) were added dropwise, and the reaction was left stirring at room temperature for 16 hours. The mixture was diluted with CH_2Cl_2 (40 ml), washed with sat. $NaHCO_3$ (50 ml), the organic layer was dried with $MgSO_4$ and concentrated to give an orange oil. The crude was then purified by column chromatography (SiO_2, 20% EtOAc in Petroleum Ether 40-60 °C) to give the final product as off-white solid (1.64 g; 95%).

^{1}H NMR (600 MHz, $CDCl_3$) δ 7.39 – 7.33 (m, 2H), 7.31 – 7.26 (m, 1H), 7.23 – 7.20 (m, 2H), 6.61 (br, 1H), 3.63 (dt, *J* = 13.4, 6.8 Hz, 1H), 2.91 (t, *J* = 7.1 Hz, 1H); **^{13}C NMR** (151 MHz, $CDCl_3$) δ 157.3 (q, *J* = 36.9 Hz),

137.6, 128.9, 128.7, 127.0, 115.8 (q, *J* = 287.7 Hz), 41.1, 34.9. **HRMS**: m/z: $[M + H]^+$ calc'd for $[C_{10}H_{11}NOF_3]^+$ expect 218.0793; found 218.0794.

2,2,2-trifluoro-N-(2-(pyridin-2-yl)ethyl)acetamide (4m)

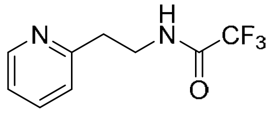

2-(2-aminoethyl)pyridine (0.48 ml; 4 mmol; 1eq) was dissolved in dry CH_2Cl_2 (30 ml) and mixture was put under argon atmosphere. Then trifluoroacetic anhydride (0.67 ml; 4.8 mmol; 1.2eq) and triethylamine (1.12 ml; 8 mmol; 2eq) were added dropwise, and the reaction was left stirring at room temperature for 16 hours. The mixture was diluted with CH_2Cl_2 (50 ml), washed with sat. $NaHCO_3$ (50 ml), the organic layer was dried with $MgSO_4$ and concentrated to give an red oil. The crude was then purified by column chromatography (SiO_2, 70% EtOAc in Petroleum Ether 40-60 ^oC) to give the final product as yellow solid (794 mg; 91%).

^{1}H NMR (600 MHz, $CDCl_3$) δ 8.49 (dd, *J* = 4.7, 1.3 Hz, 1H), 8.49 (br, 1H), 7.63 (td, *J* = 7.7, 1.8 Hz, 1H), 7.17 (m, 2H), 3.75 (dd, *J* = 6.1, 5.8 Hz, 1H), 3.03 (t, *J* = 6.1 Hz, 1H); **^{13}C NMR** (151 MHz, $CDCl_3$) δ 158.9, 157.0 (q, *J* = 36.6 Hz), 149.0 (s), 136.9, 123.5, 121.9, 116.0 (q, *J* = 287.7 Hz), 38.7, 35.2. **HRMS**: m/z: $[M + H]^+$ calc'd for $[C_9H_{10}N_2OF_3]^+$ expect 219.0745; found 219.0750.

3-(2-(Trifluoromethyl)phenyl)propan-1-amine

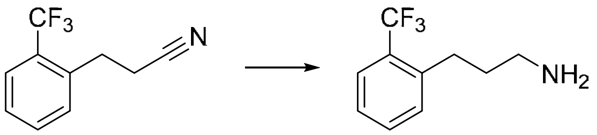

Following general procedure D with 1-(bromomethyl)-2-(trifluoromethyl)benzene (3.59 g, 15.0 mmol) and purified by silica gel chromatography Pet. Ether (40-60):EtOAc (19:1) the nitrile was isolated, with a close running impurity, as a colourless oil and used directly in the next step. Separately, a flask was charged with $AlCl_3$ (636 mg, 4.77) in THF (15 mL) and cooled to 0 °C. $LiAlH_4$ (2.4M in THF, 2.6 mL, 6.2 mmol) was added over 5 mins and stirred for a further 10 mins before the addition of the crude nitrile in THF (5 mL) dropwise over 5 mins. The mixture was warmed to rt and stirred overnight before cooling again to 0 °C, diluting with Et_2O (20 mL) and quenching, slowly, with water (2 mL). The mixture was acidified with 3 M HCl to pH 1 and the organic layer separated and further extracted with 3 M HCl (2 x 10 mL). The aqueous phases were combined and basified with 10% NaOH to ca. pH 10 and extracted with CH_2Cl_2 (3 x 30 mL), dried ($MgSO_4$) and solvents removed *in vacuo* to yield the amine as a colourless oil (496 mg, 2.44 mmol, 16% over 2 steps).

^{1}H NMR (600 MHz, $CDCl_3$) δ 7.62 (d, *J* = 7.8 Hz, 1H), 7.47 (t, *J* = 7.6 Hz, 1H), 7.35 (d, *J* = 7.8 Hz, 1H), 7.29 (t, *J* = 7.6 Hz, 1H), 2.84-2.78 (m, 4H), 1.78 (tt, *J* = 7.8, 7.3 Hz, 2H); **^{13}C NMR** (151 MHz, $CDCl_3$) δ 140.7, 131.7, 130.9, 128.3 (q, $^2J_{C-F}$ = 29 Hz), 125.9, 124.6 (q, $^1J_{C-F}$ = 274 Hz), 41.5, 34.7, 29.9; **HRMS** m/z: $[M + H]^+$ calc'd for $[C_{10}H_{13}F_3N]^+$ expect 204.1000; found 204.1003.

2,2,2-Trifluoro-*N*-(3-(2-(trifluoromethyl)phenyl)propyl)acetamide (6a)

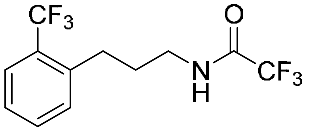

A flask was charged with 3-(2-(Trifluoromethyl)phenyl)propan-1-amine (406 mg, 2.0 mmol) and CH_2Cl_2 10 mL and cooled to 0 °C. Triethylamine (0.33 mL, 2.4 mmol) was added followed by the dropwise

addition of trifluoroacetic anhydride (0.28 mL, 2.0 mmol). The reaction mixture was warmed to rt and stirred for 4 hours before the addition of 1M HCl (5 mL), the organics separated and washed with sat. $NaHCO_3$ (5 mL). The organic phase was dried ($MgSO_4$) and solvents removed *in vacuo*. The crude amide was purified by silica gel chromatography with Pet. Ether (40-60):EtOAc (19:1) to yield **6a** as white crystals (391 mg, 1.3 mmol, 65%).

^{1}H NMR (600 MHz, $CDCl_3$) δ 7.62 (d, *J* = 8.0 Hz, 1H), 7.48 (t, *J* = 7.5 Hz, 1H), 7.33-7.30 (m, 2H), 6.83 (br, s), 3.44 (q, *J* = 6.6 Hz, 2H), 2.83 (t, *J* = 8.2 Hz, 2H), 1.93 (m, 2H); **^{13}C NMR** (151 MHz, $CDCl_3$) δ 157.5 (q, $^2J_{C-F}$ = 36.8 Hz), 132.0, 130.9, 128.3 (q, $^2J_{C-F}$ = 30.4 Hz), 126.4, 126.1 (q, $^3J_{C-F}$ = 6 Hz), 124.6 (q, $^1J_{C-F}$ = 274 Hz), 115.9 (q, $^1J_{C-F}$ = 287 Hz), 39.6, 30.6, 29.6; **HRMS** m/z: $[M + NH_4]^+$ calc'd for $[C_{12}H_{15}F_6N_2O]^+$ expect 317.1083; found 317.1083.

2,2,2-Trifluoro-*N*-(3-phenylpropyl)acetamide (6b)

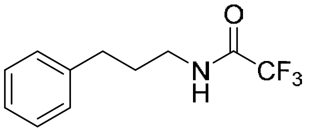

Following general procedure C with 3-phenylpropan-1-amine (406 mg, 3.0 mmol), stirred for 1 hour and purified by washing with Pet. Ether (40-60) 6a was isolated as a white solid (430 mg, 1.9 mmol, 68%).

^{1}H NMR (600 MHz, $CDCl_3$) δ 7.31 (t, *J* = 7.6 Hz, 2H), 7.23 (t, *J* = 7.6 Hz, 1H), 7.19 (d, *J* = 7.6 Hz, 2H), 6.68 (br s, 1H), 3.39 (q, *J* = 6.9 Hz, 2H), 2.69 (t, *J* = 7.7 Hz, 2H), 1.94 (tt, *J* = 6.9, 7.7 Hz, 2H); **^{13}C NMR** (151 MHz, $CDCl_3$) δ 157.3 (q, $^2J_{C-F}$ = 37 Hz), 140.7, 128.6, 128.3, 126.3, 115.9 (q, $^1J_{C-F}$ = 287 Hz), 39.6, 33.0, 30.3.

Data is in accordance with those previously reported.[8]

***N*-(3-(2-bromophenyl)propyl)-2,2,2-trifluoroacetamide (6c)**

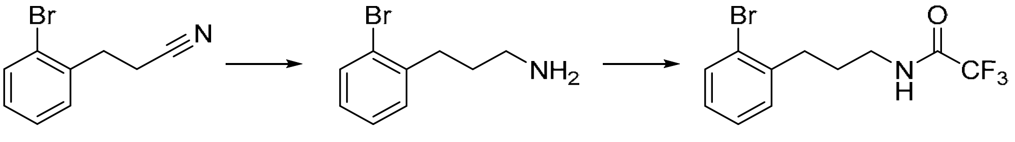

Following general procedure D with 1-(bromomethyl)-2-bromobenzene (5.00 g, 20.0 mmol) and purified by silica gel chromatography Pet. Ether (40-60):EtOAc (19:1) to yield precursor 3-(2-bromophenyl)propanenitrile as a pale yellow oil with ~20% impurity by NMR and used crude in the subsequent reaction. To a suspension of $AlCl_3$ (1.54 g, 11.6 mmol) in THF (50 mL) cooled to 0 °C was added dropwise $LiAlH_4$ (2.4 M in THF, 6.28 ml, 15.1 mmol) over 5 mins and the mixture stirred for 15 mins at this temperature before the dropwise addition of the crude nitrile in THF (5 ml) over 5 mins. The mixture was warmed to rt and stirred for 14 h before quenching with water (10 mL) and the addition of 10% NaOH (30 mL). The aqueous was extracted into Et_2O (3 x 50 mL), dried ($MgSO_4$) and concentrated *in vacuo*. The residue was redissolved in CH_2Cl_2 (20 mL), cooled to 0 °C and triethylamine (2.1 mL, 15 mmol) added followed by trifluoroacetic anhydride (1.61 mL, 11.6 mmol) dropwise over 5 mins with rapid stirring. The reaction was warmed to rt and stirred for a further 2 h before quenching with 1M HCl (5 mL) and the organic layer separated and washed with a further portion of 1M HCl (10 mL) and sat. $NaHCO_3$ (10 mL). The organic layer was dried ($MgSO_4$), concentrated *in vacuo* and purified by silica gel chromatography with Pet. Ether (40-60):EtOAc (19:1) to yield **6c** as a white solid (1.89 g, 6.1 mmol, 30% yield over 3 steps).

^{1}H NMR (600 MHz, $CDCl_3$) δ 7.53 (br s, 1H), 7.52 (d, *J* = 8.3 Hz, 1H), 7.24-7.19 (m, 2H), 7.06 (dt, *J*= 1.8, 7.7 Hz, 1H), 3.41 (q, *J* = 6.7 Hz, 2H), 2.79 (t, *J* = 7.4 Hz, 2H), 1.93 (tt, *J* = 6.7, 7.4 Hz, 2H);); **^{13}C NMR** (151

MHz, $CDCl_3$) δ 157.8 (q, $^2J_{C\text{-}F}$ = 36 Hz), 140.2, 132.9, 130.3, 128.0, 127.7, 124.3, 116.0 (q, $^1J_{C\text{-}F}$ = 286 Hz), 39.6, 33.2, 28.9.

Data is in accordance with those previously reported.[9]

3-(2-Chlorophenyl)propanenitrile

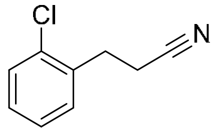

Following general procedure D with 1-(bromomethyl)-2-chlorobenzene (1.23 g, 6.0 mmol), purified by silica gel chromatography with Pet. Ether (40-60):EtOAc (19:1) the nitrile was isolated as a colourless oil (563 mg, 3.41 mmol, 57%).

^{1}H NMR (600 MHz, $CDCl_3$) δ 7.39 (dd, *J* = 1.6, 7.1 Hz, 1H), 7.31 (dd, *J* = 2.1, 7.0 Hz, 1H), 7.28-7.23 (m, 2H), 3.10 (t, *J* = 7.4 Hz, 2H), 2.69 (t, *J* = 7.4 Hz, 2H); **^{13}C NMR** (151 MHz, $CDCl_3$) δ 135.5, 133.7, 130.8, 129.8, 128.9, 127.3, 118.8, 29.7, 17.5.

The data was in accordance with those previously reported.[10]

***N*-(3-(2-chlorophenyl)propyl)-2,2,2-trifluoroacetamide (6d)**

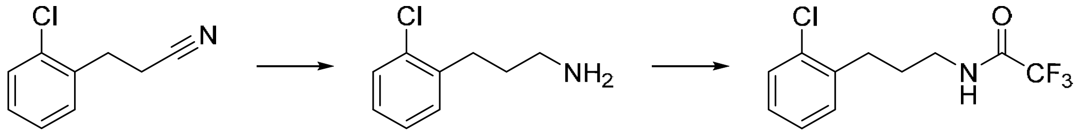

Following general procedure E using 3-(2-Chlorophenyl)propanenitrile (578 mg, 3.5 mmol) yielded the crude amine which was redissolved in CH_2Cl_2 (10 mL) and cooled to 0 °C. Triethylamine (0.33 mL, 2.4 mmol) was added followed by trifluoroacetic anhydride (0.28 mL, 2.0 mmol) dropwise over 5 mins. The reaction was warmed to rt and left to stir for 2 hours. At this time, the reaction was quenched with 1 M HCl (3 mL), the organics separated and the aqueous phase extracted once more with CH_2Cl_2 (5 mL). The combined organic layers were washed once with sat. $NaHCO_3$ (5 mL), dried ($MgSO_4$) and solvents removed *in vacuo*. The crude product was purified by silica gel chromatography with Pet. Ether (40-60):EtOAc (8:2) to yield 6d as a white solid (464 mg, 1.75 mmol, 50% over 2 steps).

^{1}H NMR (600 MHz, $CDCl_3$) δ 7.35 (d, *J* = 7.6 Hz, 1H), 7.21-7.15 (m, 3H), 6.84 (br s, 1H), 3.40 (q, *J* = 6.8 Hz, 2H), 2.79 (t, *J* = 8.1 Hz, 2H), 1.93 (quint, *J* = 7.4 Hz, 2H); **^{13}C NMR** (151 MHz, $CDCl_3$) δ 157.4 (q, $^2J_{C\text{-}F}$ = 37 Hz), 138.3, 133.8, 130.4, 129.6, 127.8, 127.0, 115.9 (q, $^1J_{C\text{-}F}$ = 288 Hz), 39.5, 30.6, 28.8; **HRMS** m/z: $[M + H]^+$ calc'd for $[C_{11}H_{12}ClF_3NO]^+$ expect 266.0559; found 266.0558.

***N*-(3-(2-fluorophenyl)propyl)-2,2,2-trifluoroacetamide (6e)**

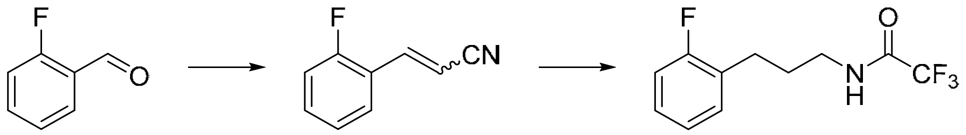

To a solution of 2-fluorobenzaldehyde (2.10 ml, 20 mmol) in CH_2Cl_2 (20 ml) was added tetrabutylammonium iodide (74 mg, 0.2 mmol) and 2M NaOH solution (20 ml). With rapid stirring, diethyl cyanomethylphosphonate (3.5 ml, 22 mmol) was added and a mild exotherm was observed. After stirring for 12 h at room temperature, the phases were separated and the organic phase washed

with water and brine, dried (Na_2SO_4) and evaporated *in vacuo*. The crude residue was purified on silica gel to give the title compound as a mixture of E and Z isomers (2.06 g, 14.0 mmol, 70% yield).

E isomer: **^{1}H NMR** (600 MHz, $CDCl_3$) δ 7.47 (d, *J* = 17.2 Hz, 1H), 7.38 - 7.45 (m, 2H), 7.19 (dt, *J* = 7.6, 1.0 Hz, 1H), 7.09 - 7.14 (m, 1H), 6.02 (dd, *J* = 16.8, 1.0 Hz, 1H); **^{13}C NMR** (151 MHz, $CDCl_3$) δ 161.0 (d, *J* = 254.8 Hz), 143.6 (d, *J* = 2.4 Hz), 132.7 (d, *J* = 8.9 Hz), 128.7 (d, *J* = 2.6 Hz), 124.7 (d, *J* = 3.7 Hz), 121.6 (d, *J* = 11.5 Hz), 118.0, 116.1 (d, *J* = 21.7 Hz), 99.2 (d, *J* = 9.0 Hz).
Z isomer: **^{1}H NMR** (600 MHz, $CDCl_3$) δ 8.19 (dt, *J* = 7.8, 1.6 Hz, 1H), 7.38 - 7.45 (m, 2H), 7.23 (t, *J* = 7.6 Hz, 1H), 7.09 - 7.14 (m, 1H), 5.55 (d, *J* = 12.2 Hz, 1H); **^{13}C NMR** (151 MHz, $CDCl_3$) δ 160.5 (d, *J* = 253.4 Hz), 140.4 (d, *J* = 7.0 Hz), 132.7 (d, *J* = 8.8 Hz), 128.3 (d, *J* = 1.6 Hz), 124.6 (d, *J* = 3.8 Hz), 121.7 (d, *J* = 11.6 Hz), 116.9, 115.8 (d, *J* = 21.7 Hz), 97.1 (d, *J* = 2.2 Hz).

This matched literature data.[11]

This compound was taken up in methanol (50 ml), *p*TsOH (4.0g, 21.0 mmol) and Pd/C (200 mg) were added and the reaction stirred under a balloon of hydrogen for 20h. After this time, it was filtered through Celite and the solvent evaporated. The crude residue was taken up in CH_2Cl_2 (50 ml) and triethylamine (7.7 ml, 56 mmol) and trifluoroacetic anhydride (2.9 ml, 21.0 mmol) were added at 0 ^{o}C. After stirring at rt for 3h, water was added and the organics were washed with satd bicarb solution, dried (Na_2SO_4) and evaporated. The crude residue was purified on silica gel to give the title compound as a colourless oil (1.95 g, 7.83 mmol, 56% yield over two steps).

^{1}H NMR (600 MHz, $CDCl_3$) δ 7.22-7.17 (m, 2H), 7.08 (t, *J* = 7.1 Hz, 1H), 7.02 (dd, *J* = 8.3, 9.6 Hz, 1H), 6.62 (br s, 1H), 3.39 (q, *J* = 6.7 Hz, 2H), 2.72 (t, *J* = 7.5 Hz, 2H), 1.92 (quint, *J* = 7.3 Hz, 2H); **^{13}C NMR** (151 MHz, $CDCl_3$) δ 161.1 (d, $^1J_{C-F}$ = 244.2 Hz), 157.3 (q, $^2J_{C-F}$ = 36.5 Hz), 130.6 (d, $^3J_{C-F}$ = 4.8 Hz), 128.1 (d, $^3J_{C-F}$ = 8.1 Hz), 127.4 (d, $^2J_{C-F}$ = 14 Hz), 124.3 (d, $^4J_{C-F}$ = 3.5 Hz), 115.8 (q, $^1J_{C-F}$ = 288 Hz), 115.3 (d, $^2J_{C-F}$ = 23 Hz), 39.3, 29.1, 26.1 (d, $^3J_{C-F}$ = 3 Hz); **^{19}F NMR** (376 MHz, $CDCl_3$) δ -76.99 ($COCF_3$), -119.97 (Ar-F); **HRMS** m/z: $[M + H]^+$ calc'd for $[C_{11}H_{12}F_4NO]^+$ expect 250.0855; found 250.0861.

***N*-(3-(3-fluorophenyl)propyl)-2,2,2-trifluoroacetamide (6f)**

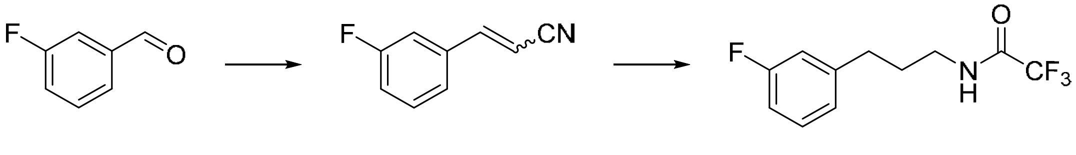

To a solution of 3-fluorobenzaldehyde (2.12 ml, 20 mmol) in CH_2Cl_2 (20 ml) was added tetrabutylammonium iodide (74 mg, 0.2 mmol) and 2M NaOH solution (20 ml). With rapid stirring, Diethyl cyanomethylphosphonate (3.5 ml, 22 mmol) was added and a mild exotherm was observed. After stirring for 12h at room temperature, the phases were separated and the organic phase washed with water and brine, dried (Na_2SO_4) and evaporated in vacuo. The crude residue was purified on silica gel to give the title compound as a mixture of E and Z isomers (1.62g, 11.0 mmol, 55% yield).

E isomer: **^{1}H NMR** (600 MHz, $CDCl_3$) δ 7.36-7.40 (m, 1H), 7.35 (d, *J* = 16.6 Hz, 1H), 7.23 (d, *J* = 7.7 Hz, 1H), 7.08-7.16 (m, 2H), 5.89 (d, *J* = 16.7 Hz, 1H); **^{13}C NMR** (151 MHz, $CDCl_3$) δ 163.0 (d, *J* = 247.7 Hz), 149.2, 135.6 (d, *J* = 8 Hz), 130.8 (d, *J* = 8. Hz), 123.5 (d, *J* = 3 Hz), 118.1 (d, *J* = 21 Hz), 117.7, 113.7 (d, *J* = 22 Hz), 98.0. *Z* isomer: **^{1}H NMR** (600 MHz, $CDCl_3$) δ 7.57 (d, *J* = 7.9 Hz, 1H), 7.51 (dt, *J* = 10.0, 2.0 Hz, 1H), 7.36-7.40 (m, 1H), 7.08-7.16 (m, 2H), 5.52 (d, *J* = 12.1 Hz, 1H); **^{13}C NMR** (151 MHz, $CDCl_3$) δ 162.7 (d, *J* = 247 Hz), 147.3 (d, *J* = 3 Hz), 135.5 (d, *J* = 8 Hz), 130.6 (d, *J* = 8 Hz), 124.8 (d, *J* = 3 Hz), 117.9 (d, *J* = 21 Hz), 116.9, 115.5 (d, *J* = 23 Hz), 96.63.

This matched literature data.[11]

This compound was taken up in methanol (40 ml), *p*TsOH (3.1g, 16.5 mmol) and Pd/C (200 mg) were added and the reaction stirred under a balloon of hydrogen for 20h. After this time, it was filtered through Celite and the solvent evaporated. The crude residue was taken up in CH_2Cl_2 (50 ml) and triethylamine (6.0 ml, 44 mmol) and trifluoroacetic anhydride (2.3 ml, 16.5 mmol) were added at 0 °C. After stirring at rt for 3h, water was added and the organics were washed with satd bicarb solultion, dried (Na_2SO_4) and evaporated. The crude residue was purified on silica gel to give the title compound as an oil that solidified on standing (1.64g, 6.59 mmol, 60% yield over two steps).

^{1}H NMR (600 MHz, $CDCl_3$) δ 7.25 (dt, *J* = 6.5, 7.8 Hz, 1H), 7.04 (br s, 1H), 6.95 (d, *J* = 7.8 Hz, 1H), 6.91-6.87 (m, 2H), 3.38 (dt, *J* = 6.6, 6.9 Hz, 2H), 2.67 (t, *J* = 7.9 Hz, 2H), 1.92 (quint, 7.4 Hz, 2H); **^{13}C NMR** (151 MHz, $CDCl_3$) δ 163.0 (d, $^1J_{C-F}$ = 246 Hz), 157.5 (q, $^2J_{C-F}$ = 37 Hz), 143.3 (d, $^3J_{C-F}$ = 7 Hz), 130.0 (d $^3J_{C-F}$ = 8 Hz), 124.0 (d, $^4J_{C-F}$ = 3 Hz), 115.8 (q, $^1J_{C-F}$ = 288 Hz), 115.1 (d, $^2J_{C-F}$ = 21 Hz), 113.1 (d, $^2J_{C-F}$ = 21 Hz), 39.5, 32.6 (d, $^4J_{C-F}$ = 12 Hz), 30.0; **HRMS** m/z: $[M + H]^+$ calc'd for $[C_{11}H_{12}F_4NO]^+$ expect 250.0855; found 250.0867.

Methyl 2-(3-(2,2,2-trifluoroacetamido)propyl)benzoate (6g)

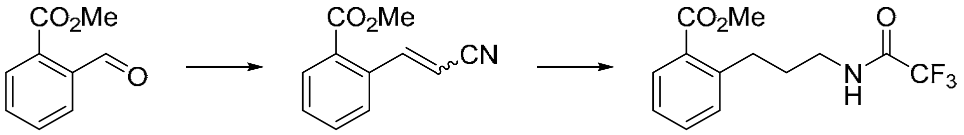

To a solution of NaH (60%) (144 mg, 3.6 mmol) in THF (5 ml) was added diethyl cyanomethylphosphonate (580 mg, 3.3 mmol) at 0 °C. After 15 mins, methyl 2-formylbenzoate (500 mg, 3.05 mmol) in THF (2 ml) was added dropwise. The ice bath was removed and after stirring for 2h at room temperature, the reaction was diluted with diethyl ether and the organic phase washed with water and brine, dried (Na_2SO_4) and evaporated *in vacuo*. The crude residue was purified on silica gel eluting with 25% Et_2O/Petrol to give the title compound as a mixture of E and Z isomers (440 mg, 2.35 mmol, 77% yield).

^{1}H NMR (400 MHz, $CDCl_3$) δ 8.28 (d, *J* = 16.6 Hz, 1.2H), 8.04 (dd, *J* = 1.0, 7.8 Hz, 1H), 7.98 (dd, *J* = 0.8, 7.9 Hz, 1.2H), 7.95 (d, *J* = 11.8 Hz, 1H), 7.80 (d, *J* = 7.8 Hz, 1H), 7.61 (dt, *J* = 1.0, 7.6 Hz, 1H), 7.44 – 7.58 (m, 4.8H) 5.77 (d, *J* = 16.6 Hz, 1.2H), 5.57 (d, *J* = 11.8 Hz, 1H), 3.91 (s, 3.6H), 3.89 (s, 3H); **^{13}C NMR** (100 MHz, $CDCl_3$) δ 150.0, 149.9, 135.5, 135.3, 132.8, 132.7, 131.1, 131.0, 130.3, 129.9, 129.4, 129.1, 128.8, 127.3, 117.9, 116.8, 99.0, 97.8, 52.5, 52.4; **HRMS** m/z: $[M + H]^+$ calc'd for $[C_{11}H_{10}NO_2]^+$ expect 188.0712; found 188.0710.

This material was taken up in methanol (20 ml), *p*TsOH (760 mg, 4.0 mmol) and Pd/C (100 mg) were added and the reaction stirred under a balloon of hydrogen for 20h. After this time, it was filtered through Celite and the solvent evaporated. The crude residue was taken up in CH_2Cl_2 (20 ml) and triethylamine (1.4 ml, 10 mmol) and trifluoroacetic anhydride (0.56 ml, 4.0 mmol) were added at 0 °C. After stirring at rt for 3h, water was added and the organics were washed with satd. bicarb solution, dried (Na_2SO_4) and evaporated. The crude residue was purified twice on silica gel to remove close running impurities (25% Et_2O/Petrol) to give the title compound as a colourless oil (132 mg, 0.46 mmol, 20% yield over two steps).

^{1}H NMR (600 MHz, $CDCl_3$) δ 7.92 (dd, *J* = 1.1, 7.8 Hz, 1H), 7.66 (br s, 1H), 7.46 (dt, *J* = 1.3, 7.5 Hz, 1H), 7.25-7.31 (m, 2H), 3.90 (s, 3H), 3.37 (q, *J* = 5.9 Hz, 2H), 3.00 (t, *J* = 7.5 Hz, 2H), 1.90-1.95 (m, 2H); **^{13}C NMR** (151 MHz, $CDCl_3$) δ 168.2, 157.5 (q, *J* = 36 Hz) 143.2, 132.5, 131.1, 130.9, 128.9, 126.4, 116.0 (q,

J = 287 Hz), 52.2, 39.1, 30.9, 30.4; **HRMS** m/z: $[M + H]^+$ calc'd for $[C_{13}H_{15}NO_3F_3]^+$ expect 290.1004; found 290.1000.

N-(3-(2,5-difluorophenyl)propyl)-2,2,2-trifluoroacetamide (6h)

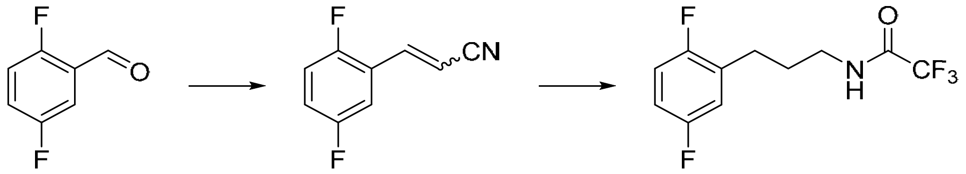

To a solution of 2,5-difluorobenzaldehyde (2.17 ml, 20 mmol) in CH_2Cl_2 (20 ml) was added tetrabutylammonium iodide (74 mg, 0.2 mmol) and 2M NaOH solution (20 ml). With rapid stirring, Diethyl cyanomethylphosphonate (3.5 ml, 22 mmol) was added and a mild exotherm was observed. After stirring for 12h at room temperature, the phases were separated and the organic phase washed with water and brine, dried (Na_2SO_4) and evaporated *in vacuo*. The crude residue was eluted on silica gel to give the cyanoacrylate as a mixture of E and Z isomers together with unreacted aldehyde. A portion of 1.14 g, (~7 mmol without taking into account aldehyde impurity) of this mixture was taken up in methanol (25 ml), *p*TsOH (2.6g, 14.0 mmol) and Pd/C (250 mg) were added and the reaction stirred under a balloon of hydrogen for 20h. After this time, it was filtered through Celite and the solvent evaporated. The crude residue was taken up in CH_2Cl_2 (20 ml) and triethylamine (0.9 ml, 6.3 mmol) and trifluoroacetic anhydride (0.67 ml, 4.7 mmol) were added at 0 °C. After stirring at rt for 3h, water was added and the organics were washed with satd bicarb solution, dried (Na_2SO_4) and evaporated. The crude residue was purified on silica gel to give the title compound as an oil that solidified on standing (670 mg, 2.5 mmol, 36% yield over two steps).

^{1}H NMR (600 MHz, $CDCl_3$) δ 6.98-6.94 (m, 2H), 6.89-6.84 (m, 2H), 3.38 (dt, J = 6.9, 6.6 Hz, 2H), 2.67 (t, J = 7.7 Hz, 2H), 1.91 (quint, J = 7.4 Hz, 2H); **^{13}C NMR** (151 MHz, $CDCl_3$) δ 158.6 (dd, $^1J_{C-F}$ = 242.2 Hz, $^4J_{C-F}$ 2.2 Hz), 157.5 (q, $^2J_{C-F}$ = 36.8 Hz), 156.9 (dd, $^1J_{C-F}$ = 240.1 Hz, $^4J_{C-F}$ = 2.5 Hz), 129.9 (dd, $^2J_{C-F}$ = 18.6 Hz, $^3J_{C-F}$ = 7.6 Hz), 116.7 (dd, $^2J_{C-F}$ = 23.8 Hz, $^3J_{C-F}$ = 5.2 Hz), 116.2 (dd, $^2J_{C-F}$ = 25.5 Hz, $^3J_{C-F}$ = 8.9 Hz), 115.8 (q, $^1J_{C-F}$ = 287.8 Hz), 114.2 (dd, $^2J_{C-F}$ = 24.0 Hz, $^3J_{C-F}$ = 8.6 Hz), 39.3, 28.8, 26.1; **HRMS** m/z: $[M + H]^+$ calc'd for $[C_{11}H_{11}F_5NO]^+$ expect 268.0761; found 268.0766.

2,2,2-Trifluoro-N-(3-(o-tolyl)propyl)acetamide (6i)

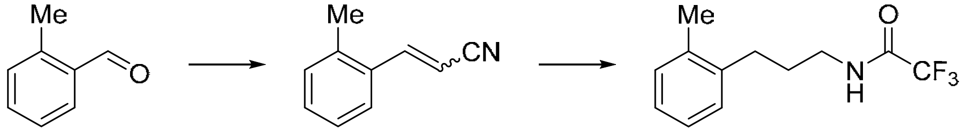

To a solution of NaH (60%) (560 mg, 14.0 mmol) in THF (20 ml) was added diethyl cyanomethylphosphonate (1.94 ml, 12.0 mmol) at 0 °C. After 15 mins, 2-methylbenzaldehyde (1.15 ml, 10 mmol) was added dropwise. The ice bath was removed and after stirring for 2 h at room temperature, the reaction was diluted with diethyl ether and the organic phase washed with water and brine, dried (Na_2SO_4) and evaporated *in vacuo*. The crude residue was purified on silica gel to give the title compound as a mixture of E and Z isomers (1.20 g, 8.39 mmol, 84% yield).

E Isomer: **^{1}H NMR** (400 MHz, $CDCl_3$) δ 7.69 (d, J = 16.6 Hz, 1H), 7.47 (d, J = 7.8 Hz, 1H), 7.20 – 7.38 (m, 4H), 5.80 (d, J = 16.6 Hz, 1H), 2.40 (s, 3H); **^{13}C NMR** (100 MHz, $CDCl_3$) δ 148.4, 137.3, 132.6, 131.1, 131.0, 126.6, 125.6, 118.4, 97.2, 19.6.

Z Isomer: **^{1}H NMR** (400 MHz, $CDCl_3$) δ 7.96 (d, *J* = 7.3 Hz, 1H), 7.42 (d, *J* = 11.9 Hz, 1H), 7.20 – 7.38 (m, 4H), 5.54 (d, *J* = 12.0 Hz, 1H), 2.36 (s, 3H); **^{13}C NMR** (100 MHz, $CDCl_3$) δ 147.6, 137.2, 132.8, 130.6 (two resonances overlapping), 127.7, 126.4, 117.2, 97.0, 19.7.

This matched literature data. [11-12]

This material was taken up in methanol (40 ml), *p*TsOH (2.28 g, 12.0 mmol) and Pd/C (250 mg) were added and the reaction stirred under a balloon of hydrogen for 20h. After this time, it was filtered through Celite and the solvent evaporated. The crude residue was taken up in CH_2Cl_2 (40 ml) and triethylamine (4.1 ml, 30 mmol) and trifluoroacetic anhydride (1.7 ml, 12.0 mmol) were added at 0 °C. After stirring at rt for 3h, water was added and the organics were washed with satd. bicarb solution, dried (Na_2SO_4) and evaporated. The crude residue was purified twice on silica gel to remove close running impurities (20% Et_2O/Petrol) to give the title compound as a colourless oil that solidified on standing (692 mg, 2.82 mmol, 34% yield over two steps).

^{1}H NMR (600 MHz, $CDCl_3$) δ 7.11-7.29 (m, 4H), 6.57 (br s, 1H), 3.44 (q, *J* = 6.8 Hz, 2H), 2.69 (t, *J* = 7.7 Hz, 2H), 2.32 (s, 3H), 1.87-1.93 (m, 2H); **^{13}C NMR** (150 MHz, $CDCl_3$) δ 157.3 (q, J = 36 Hz), 138.8, 135.8, 130.5, 128.7, 126.5, 126.2, 115.8 (q, J = 288 Hz), 39.8, 30.4, 29.1, 19.2; **HRMS** m/z: $[M + NH_4]^+$ calc'd for $[C_{12}H_{15}F_3NO]^+$ expect 246.1106; found 246.1115.

2,2,2-Trifluoro-*N*-(3-(o-tolyl)propyl)acetamide (6j)

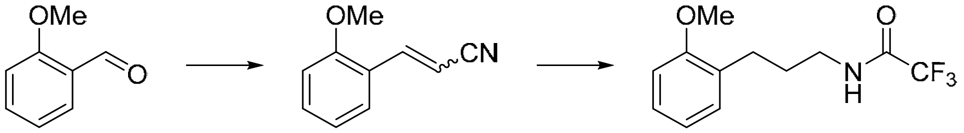

To a solution of 2-methoxybenzaldehyde (2.72 g, 20 mmol) in CH_2Cl_2 (20 ml) was added tetrabutylammonium iodide (74 mg, 0.2 mmol) and 2M NaOH solution (20 ml). With rapid stirring, Diethyl cyanomethylphosphonate (3.5 ml, 22 mmol) was added and a mild exotherm was observed. After stirring for 12 h at room temperature, the phases were separated and the organic phase washed with water and brine, dried (Na_2SO_4) and evaporated *in vacuo*. The crude residue was eluted on silica gel to give the cyanoacrylate as a mixture of E and Z isomers together with unreacted aldehyde. This mixture was taken up in methanol (50 ml), *p*TsOH (4.8g, 25.1 mmol) and Pd/C (300 mg) were added and the reaction stirred under a balloon of hydrogen for 20 h. After this time, it was filtered through Celite and the solvent evaporated. The crude residue was taken up in CH_2Cl_2 (40 ml) and triethylamine (9.2 ml, 67 mmol) and trifluoroacetic anhydride (3.5 ml, 25.1 mmol) were added at 0 °C. After stirring at rt for 3 h, water was added and the organics were washed with satd bicarb solution, dried (Na_2SO_4) and evaporated. The crude residue was purified on silica gel to give the title compound as an oil that solidified on standing (1.75 g, 6.7 mmol, 34% yield over three steps).

^{1}H NMR (600 MHz, $CDCl_3$) δ 7.23 (dt, *J* = 1.7, 8.0 Hz, 1H), 7.14 (dd, *J* = 1.6, 7.4 Hz, 1H), 6.94 (dt, *J* = 0.8, 7.4 Hz, 1H), 6.90 (d, *J* = 8.0 Hz, 1H), 6.80 (br s, 1H), 3.86 (s, 3H), 3.31 (q, *J* = 6.4 Hz, 2H), 2.72 (t, *J* = 7.1 Hz, 2H), 1.88 (quint, *J* = 6.8 Hz, 2H), ; **^{13}C NMR** (151 MHz, $CDCl_3$) δ 157.6, 156.8 (q, $^2J_{C-F}$ = 37 Hz), 130.2, 128.8, 127.7, 121.1, 115.9 (q, $^1J_{C-F}$ = 287 Hz), 110.6, 55.4, 38.6, 29.1, 26.4; **HRMS** m/z: $[M + H]^+$ calc'd for $[C_{12}H_{15}F_3NO_2]^+$ expect 262.1049; found 262.1048.

2,2,2-Trifluoro-*N*-(3-(4'-methyl-[1,1'-biphenyl]-2-yl)propyl)acetamide (6k)

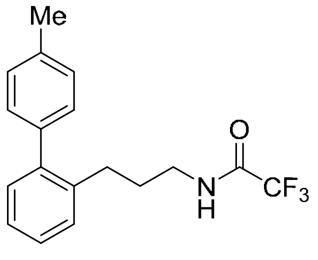

To a microwave tube was added *N*-(3-(2-bromophenyl)propyl)-2,2,2-trifluoroacetamide (775 mg, 2.5 mmol), *p*-tolylboronic acid (680 mg, 5.0 mmol), Xhos (23.8 mg, 5.0 x 10^{-2} mmol), $Pd(OAc)_2$ (5.6 mg, 2.5 x 10^{-2} mmol) and KF (436 mg, 7.5 mmol). The tube was backfilled with argon, THF (7 mL) added and the reaction heated to 60 °C for 2 hours. The mixture was cooled and filtered through celite and the solid washed with CH_2Cl_2 (2 x 10 mL). The combined filtrates were concentrated *in vacuo* and the crude product purified by silica gel chromatography:EtOAc (19:1) to yield **6k** as a white solid (649 mg, 2.0 mmol, 81%).

^{1}H NMR (600 MHz, $CDCl_3$) δ 7.38-7.27 (m, 8H), 6.51 (br s, 1H), 3.23 (q, *J* = 6.5 Hz, 2H), 2.77 (t, *J* = 7.3 Hz, 2H), 2.49 (s, 3H), 1.79 (tt, *J* = 6.7, 7.4 Hz, 2H); **^{13}C NMR** (151 MHz, $CDCl_3$) δ 157.3 (q, $^2J_{C-F}$ = 37 Hz), 142.0, 138.6, 138.4, 136.9, 130.3, 129.4, 129.1, 127.6, 126.3, 115.9 (q, $^1J_{C-F}$ = 288 Hz), 39.3, 30.3, 29.9, 21.1; **HRMS** m/z: $[M + H]^+$ calc'd for $[C_{18}H_{19}F_3NO]^+$ expect 322.1419; found 322.1424.

2,2,2-Trifluoro-*N*-(3-(pyridin-2-yl)propyl)acetamide (6l)

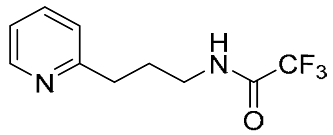

To a solution of 2-[3-(pyridin-2-yl)propyl]isoindoline-1,3-dione[8] (2.50 g, 9.3 mmol) in ethanol (50 ml) was added hydrazine monohydrate (1.0 ml, 20 mmol). After stirring at room temperature for 5h, the reaction was filtered and the solid washed with further ethanol. The solvent was evaporated and taken up in CH_2Cl_2 (50 ml). At 0 °C, triethylamine (5.2 ml, 37.5 mmol) and trifluoroacetic anhydride (3.8 ml, 27 mmol) were added. After stirring for 4 h at rt, satd. bicarb was added and the organic layer separated, dried (Na_2SO_4) and evaporated. Purification on silica (eluting 1:2 CH_2Cl_2:Pet. Ether (40-60)) gave the title compound as a colourless oil (650 mg, 2.80 mmol, 30%).

^{1}H NMR (600 MHz, $CDCl_3$) δ 9.54 (br s, 1H), 8.49 (d, *J* = 4.8 Hz, 1H), 7.64 (dt, *J* = 1.6, 7.8 Hz, 1H), 7.20-7.16 (m, 2H), 3.44 (dt, *J* = 6.2, 5.6 Hz, 2H), 2.98 (br t, *J* = 6.3 Hz, 2H), 2.07-2.03 (m, 2H); **^{13}C NMR** (151 MHz, $CDCl_3$) δ 160.6, 157.4 (q, $^2J_{C-F}$ = 37 Hz), 148.5, 137.1, 123.4, 121.6, 116.2 (q, $^1J_{C-F}$ = 288 Hz), 40.4, 35.7, 26.5; **HRMS** m/z: $[M + NH_4]^+$ calc'd for $[C_{10}H_{12}F_3N_2O]^+$ expect 233.0902; found 233.0902.

2,2,2-Trifluoro-*N*-(4-phenylbutyl)acetamide (10a)

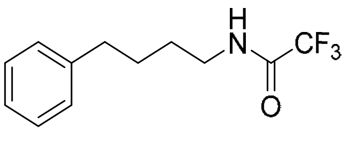

Following general procedure C with 4-phenylbutyl-1-amine (328 mg, 2.2 mmol), stirred for 3 hours and purified by washing with Pet. Ether (40-60) the title compound was isolated as a white solid (439 mg, 1.8 mmol, 81%).

^{1}**H NMR** (600 MHz, $CDCl_3$) δ 7.31 (t, *J* = 7.4 Hz, 2H), 7.24-7.19 (m, 3H), 6.83 (br s, 1H), 3.37 (q, *J* = 6.7 Hz, 2H), 2.67 (t, *J*= 7.6 Hz, 2H), 1.72-1.67 (m, 2H), 1.66-1.61 (m, 2H); ^{13}**C NMR** (151 MHz, $CDCl_3$) δ 157.4 (q, $^{2}J_{C\text{-}F}$ = 37 Hz), 141.7, 128.4, 128.4, 126.0, 115.9 (q, $^{1}J_{C\text{-}F}$ = 286 Hz), 39.8, 35.3, 28.4, 28.3; **HRMS** m/z: $[M + NH_4]^+$ calc'd for $[C_{12}H_{18}F_3N_2O]^+$ expect 263.1366; found 263.1364.

5-Phenylpentanenitrile

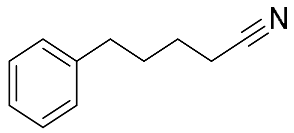

Following general procedure D with (3-bromopropyl)benzene (1.39 g, 7.0 mmol), purified by silica gel chromatography with Pet. Ether (40-60):EtOAc (17:3) the title compound was isolated as a colourless oil (225 mg, 1.42 mmol, 20%).

^{1}**H NMR** (600 MHz, $CDCl_3$) δ 7.32 (t, *J* = 7.4 Hz, 2H), 7.24-7.20 (m, 3H), 2.68 (t, *J* = 7.4 Hz, 2H), 2.35 (t, *J* = 7.1 Hz, 2H), 1.84-1.78 (m, 2H), 1.72-1.67 (m, 2H); ^{13}**C NMR** (151 MHz, $CDCl_3$) δ 141.3, 128.5, 128.4, 126.1, 119.7, 35.0, 30.3, 24.9, 17.0.

Data is in accordance with those previously reported.[13]

2,2,2-Trifluoro-*N*-(5-phenylpentyl)acetamide (11a)

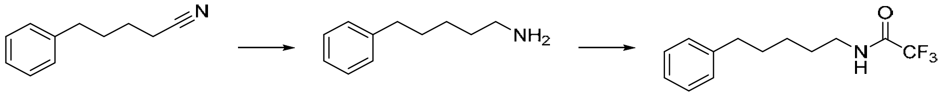

Following general procedure E using 5-phenylpentanenitrile (225 mg, 1.42 mmol) yielded the crude amine which was redissolved in CH_2Cl_2 (5 mL) and cooled to 0 °C. Triethylamine (0.12 mL, 0.83 mmol) was added followed by trifluoroacetic anhydride (0.11 mL, 0.83 mmol) dropwise over 1 min. The reaction was warmed to rt and left to stir for 18 hours. At this time, the reaction was quenched with 1 M HCl (1 mL), the organics separated and the aqueous phase extracted once more with CH_2Cl_2 (5 mL). The combined organic layers were washed once with sat. $NaHCO_3$ (2 mL), dried ($MgSO_4$) and solvents removed *in vacuo*. The crude product was purified by silica gel chromatography with Pet. Ether (40-60):EtOAc (19:1) to yield the title compound as a white solid (189 mg, 0.73 mmol, 51% over 2 steps).

^{1}**H NMR** (600 MHz, $CDCl_3$) δ 7.30 (t, *J* = 7.7 Hz, 2H), 7.22-7.18 (m, 3H), 6.48 (br s), 3.36 (q, *J*= 6.6 Hz, 2H), 2.64 (t, *J* = 7.7 Hz, 2H), 1.67 (tt, *J* = 7.5, 7.7 Hz, 2H), 1.62 (tt, *J* = 7.5 Hz, 2H), 1.42-1.37 (m, 2H); ^{13}**C NMR** (151 MHz, $CDCl_3$) δ 157.2 (q, $^{2}J_{C\text{-}F}$ = 36 Hz), 142.1, 128.4, 128.3, 125.8, 115.9 (q, $^{1}J_{C\text{-}F}$ = 289 Hz), 39.9, 35.6, 30.8, 28.8, 26.2; **HRMS** m/z: $[M + H]^+$ calc'd for $[C_{13}H_{17}F_3NO]^+$ expect 260.1262; found 260.1258.

2-(6-phenylhexyl)isoindoline-1,3-dione

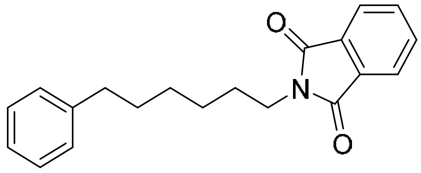

A mixture of (6-chlorohexyl)benzene (610 mg, 3.1 mmol) and K-phthalimide (0.78 g, 2.81 mmol) in DMF (2.5 mL) was refluxed for 2 hours. After cooling the mixture was poured into H_2O and the

separated solid was collected by filtration to yield the title compound as a white solid (770 mg, 2.5 mmol, 81%).

^{1}H NMR (600 MHz, $CDCl_3$) δ 7.82-7.80 (m, 2H), 7.66-7.65 (m, 2H), 7.25 (t, *J* = 7.5 Hz, 2H), 7.16-7.14 (m, 3H), 3.67 (t, *J* = 7.3 Hz, 2H), 2.59 (t, *J* = 8.0 Hz, 2H), 1.68 (quint, *J* = 7.4 Hz, 2H), 1.69, (quint, *J* = 7.9 Hz, 2H), 1.39-1.38 (m, 4H); **^{13}C NMR** (151 MHz, $CDCl_3$) δ 168.3, 142.6, 133.8, 132.2, 128.4, 128.2, 125.6, 123.1, 38.0, 35.8, 31.3, 28.8, 28.5, 26.7.

Data is in accordance with those previously reported.[13]

2,2,2-Trifluoro-*N*-(6-phenylhexyl)acetamide (12a)

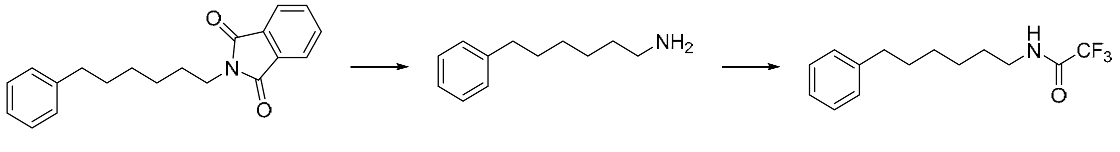

2-(6-Phenylhexyl)isoindoline-1,3-dione (770 mg, 2.5 mmol) was dissolved in EtOH (20 mL), hydrazine monohydrate (1.25 ml, 25 mmol) added and the reaction stirred overnight at rt. The resulting solid was filtered off and volatiles were removed *in vacuo*. The crude amine was further dried under high vacuum for 1 hour before being dissolved in CH_2Cl_2 (10 mL) and cooled to 0 °C. Triethylamine (0.84 ml, 6.0 mmol) was added followed by the dropwise addition of trifluoroacetic anhydride (0.53 mL, 3.8 mmol). The reaction mixture was warmed to rt and stirred for 4 hours before the addition of 1M HCl (3 mL), the organics separated and washed with a further portion of 1M HCl (3 mL) and sat. $NaHCO_3$ (5 mL). The organic phase was dried ($MgSO_4$) and solvents removed *in vacuo*. The crude amide was purified by silica gel chromatography with Pet. Ether (40-60):EtOAc (9:1) to yield the title compound as a white solid (400 mg, 1.46 mmol, 59% over 2 steps).

^{1}H NMR (600 MHz, $CDCl_3$) δ 7.31 (t, *J* 7.5 Hz, 2H), 7.22-7.20 (m, 3H), 6.71 (s, 1H), 3.35 (q, *J* = 6.9 Hz, 2H), 2.64 (t, *J* = 7.8 Hz, 2H), 1.68-1.64 (m, 2H), 1.62-1.57 (m, 2H), 1.40-1.38 (m, 4H); **^{13}C NMR** (151 MHz, $CDCl_3$) δ 157.3 (q, $^2J_{C\text{-}F}$ = 37 Hz), 142.5, 128.4, 128.3, 125.7, 115.9 (q, $^1J_{C\text{-}F}$ = 290 Hz), 40.0, 35.8, 31.2, 28.8, 28.7, 26.5; **HRMS** m/z: $[M + H]^+$ calc'd for $[C_{14}H_{19}F_3NO]^+$ expect 274.1419; found 274.1425.

Borylation of substrates

General procedure F:

To a vial was weighed substrate, ligand, B_2pin_2, and $[Ir(COD)OMe]_2$ in succession. The vial was sealed and backfilled with argon before the addition of solvent and stirred for the specified time at the specified temperature. Solvents were removed and regioselectivity analysed before and after the purification as described. For consistency the *meta* and *para* isomers are described with respect to the directing amide substituent.

2,2,2-Trifluoro-*N*-(5-(4,4,5,5-tetramethyl-1,3,2-dioxaborolan-2-yl)-2-(trifluoromethyl)benzyl)acetamide (3a)

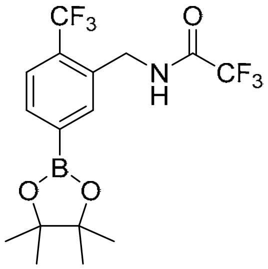

meta

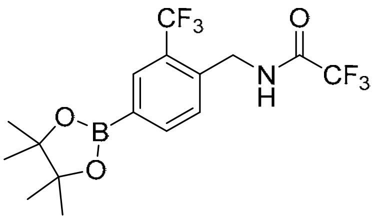

para

1a in THF:

Following general procedure F using 2,2,2-trifluoro-*N*-(2-(trifluoromethyl)benzyl)acetamide (67.8 mg, 0.25 mmol), B_2pin_2 (127 mg, 0.50 mmol), $[Ir(COD)OMe]_2$ (2.5 mg, 0.00375 mmol) and **1a** (3.8 mg, 0.0075 mmol) in THF (1.25 mL). The reaction was stirred at rt for 20 hours before cooling and the solvents removed. Analysis of crude ^{1}H NMR using internal standard 1,2-dimethoxyethane showed 13.1:1 *meta*:*para* borylation in 99% yield. The crude product was purified by silica gel chromatography (Pet. Ether (40-60):EtOAc (9:1-7:3) to give the title compound in two fractions (as a 10:1:3.6 mixture of *meta*:*para*:B_2pin_2 ratio, as determined by ^{1}H NMR) as a colourless oil (83.0 mg, 0.17 mmol, 69%*) and (a >20:1 mixture of *meta*:*para* ratio, as determined by ^{1}H NMR and used for characterisation of the *meta* product) as an off white solid (20.5 mg, 0.05 mmol, 21%) giving a combined yield of 89.4 mg, 0.23 mmol, 90% as quoted.

Meta ^{1}H NMR (500 MHz, $CDCl_3$) δ 7.92 (s, 1H), 7.88 (d, *J* = 7.8 Hz, 1H), 7.70 (d, *J* = 7.8 Hz, 1H), 6.54 (br s, 1H), 4.71 (d, *J* = 5.7 Hz, 2H), 1.36 (s, 12H); ^{13}**C NMR** (126 MHz, $CDCl_3$) δ 156.9 (q, $^2J_{C\text{-}F}$ = 37.0 Hz), 137.6, 135.0, 132.8, 130.9 (q, $^2J_{C\text{-}F}$ = 36.8 Hz), 125.7 (q, $^3J_{C\text{-}F}$ = 5.3 Hz), 124.3 (q, $^1J_{C\text{-}F}$ = 273.8 Hz), 115.7 (q, $^1J_{C\text{-}F}$ = 287.3 Hz), 84.6, 40.9, 24.9; **HRMS** m/z: [M + H]$^+$ calc'd for $[C_{16}H_{19}BF_6NO_3]^+$ expect 398.1362; found 398.1367.

*Yield calculated from a weighted average mass of B_2pin_2, and borylated products.

Dtbpy in THF:

Following general procedure F using 2,2,2-trifluoro-*N*-(2-(trifluoromethyl)benzyl)acetamide (67.8 mg, 0.25 mmol), B_2pin_2 (127 mg, 0.50 mmol), $[Ir(COD)OMe]_2$ (2.5 mg, 0.00375 mmol) and dtbpy (2.0 mg, 0.0075 mmol) in THF (1.25 mL). The reaction was stirred at rt for 20 hours before cooling and the solvents removed. Analysis of crude ^{1}H NMR using internal standard 1,2-dimethoxyethane showed 1:1 *meta*:*para* borylation in 99% yield. The crude product was purified by silica gel chromatography (Pet. Ether (40-60):EtOAc (9:1-7:1) to give the title compound (as a 1:1.1:0.7 mixture of *meta*:*para*:B_2pin_2 ratio, as determined by ^{1}H NMR) as a colourless oil (50.1 mg, 0.13 mmol, 50%). By subtracting the resonances for the *meta* isomer, the NMR data of the *para* isomer can be assigned as follows:

Para ^{1}H NMR (600 MHz, $CDCl_3$) δ 8.10 (s, 1H), 7.97 (d, *J* = 7.7 Hz, 1H), 7.51 (d, *J* = 7.7 Hz, 1H), 6.86 (br s, 1H), 4.71 (d, *J* = 7 Hz, 2H), 1.34 (s, 12H); **^{13}C NMR** (151 MHz, $CDCl_3$) δ 157.2 (q, $^2J_{C-F}$ = 37 Hz), 138.9, 137.8, 132.4 (q, $^3J_{C-F}$ = 6 Hz), 130.1, 127.7 (q, $^2J_{C-F}$ = 31 Hz), 124.4 (q, $^1J_{C-F}$ = 274 Hz), 115.7 (q, $^1J_{C-F}$ = 285 Hz), 84.4, 40.6, 24.8.

2,2,2-Trifluoro-*N*-(2-methyl-5-(4,4,5,5-tetramethyl-1,3,2-dioxaborolan-2-yl)benzyl)acetamide (3b)

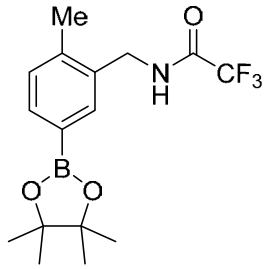

meta

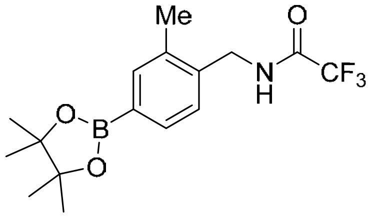

para

1a in THF:

Following general procedure F using 2,2,2-trifluoro-*N*-(2-methylbenzyl)acetamide (54.3 mg, 0.25 mmol), B_2pin_2 (127 mg, 0.50 mmol), $[Ir(COD)OMe]_2$ (2.5 mg, 0.00375 mmol) and **1a** (3.8 mg, 0.0075 mmol) in THF (1.25 mL). The reaction was stirred at 50 °C for 20 hours before cooling and the solvents removed. Analysis of crude ^{1}H NMR using internal standard 1,2-dimethoxyethane showed 7.8:1 *meta*:*para* borylation in 93% yield. The crude product was purified by silica gel chromatography (Pet. Ether (40-60):EtOAc (17:3)* to give the title compound (as a 7.7:1 mixture of *meta*:*para* ratio, as determined by ^{1}H NMR) as a white solid (77 mg, 0.20 mmol, 90%).

Meta ^{1}H NMR (600 MHz, $CDCl_3$) δ 7.70 (dd, *J* = 1.1, 7.6 Hz, 1H), 7.67 (s, 1H), 7.24 (d, *J* = 7.6 Hz, 1H), 6.45 (s, 1H), 4.53 (d, *J* = 5.4 Hz, 2H), 2.35 (s, 3H), 1.35 (s, 12H); **^{13}C NMR** (151 MHz, $CDCl_3$) δ 156.7 (q, $^2J_{C-F}$ = 37.2 Hz), 140.3, 135.8, 135.3, 132.9, 130.5, 115.8 (q, $^1J_{C-F}$ = 288.3 Hz), 83.9, 42.2, 24.8, 19.2; **HRMS** m/z: $[M + Na]^+$ calc'd for $[C_{16}H_{21}BF_3NO_3Na]^+$ expect 366.1464; found 366.1463.

*Caution, column must be run quickly to avoid decomposition

Dtbpy in THF:

Following general procedure F using 2,2,2-trifluoro-*N*-(2-methylbenzyl)acetamide (54.3 mg, 0.25 mmol), B_2pin_2 (127 mg, 0.50 mmol), $[Ir(COD)OMe]_2$ (2.5 mg, 0.00375 mmol) and dtbpy (2.0 mg, 0.0075 mmol) in THF (1.25 mL). The reaction was stirred at 50 °C for 20 hours before cooling and the solvents removed. Analysis of crude ^{1}H NMR using internal standard 1,2-dimethoxyethane showed 1.1:1 *meta*:*para* borylation in 86% yield with 10% SM present. The crude product was purified by silica gel chromatography (Pet. Ether (40-60):EtOAc (17:3) to give the title compound (as a 11:10:1 mixture of *meta*:*para*:SM ratio, as determined by ^{1}H NMR) as a colourless oil (75 mg) with NMR yield used for comparison. By subtracting the resonances for the *meta* isomer, the NMR data of the *para* isomer can be assigned as follows:

Para ^{1}H NMR (500 MHz, $CDCl_3$) δ 7.67 (s, 1H), 7.65 (d, *J* = 7.6 Hz, 1H), 7.23 (d, *J* = 7.6 H, 1H), 6.40 (br s, 1H), 4.54 (d, *J* = 5.4 Hz, 2H), 2.34 (s, 3H), 1.35 (s, 12H); **^{13}C NMR** (126 MHz, $CDCl_3$) δ 156.9 (q, $^2J_{C-F}$ = 37 Hz), 137.2, 136.5, 135.3, 132.9, 128.1, 115.8 (q, $^1J_{C-F}$ = 288 Hz), 83.9, 42.1, 24.8, 18.7.

***N*-(2-chloro-5-(4,4,5,5-tetramethyl-1,3,2-dioxaborolan-2-yl)benzyl)-2,2,2-trifluoroacetamide (3c)**

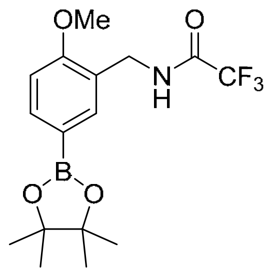

meta

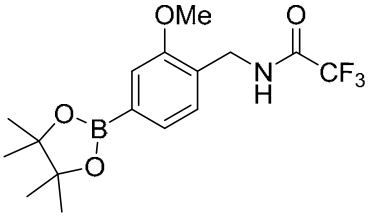

para

1a in THF:

Following general procedure F using 2,2,2-Trifluoro-*N*-(2-methoxybenzyl)acetamide (58.3 mg, 0.25 mmol), B_2pin_2 (127 mg, 0.50 mmol), $[Ir(COD)OMe]_2$ (2.5 mg, 0.00375 mmol) and **1a** (3.8 mg, 0.0075 mmol) in THF (1.25 mL). The reaction was stirred at 50 °C for 20 hours before cooling and the solvents removed. Analysis of crude ^{1}H NMR using internal standard 1,2-dimethoxyethane showed 1:1 *meta*:*para* borylation in 97% yield. The crude product was purified by silica gel chromatography (Pet. Ether (40-60):EtOAc (19:1) to give the title compound (as a 1:1 mixture of *meta*:*para* ratio, as determined by ^{1}H NMR) as a colourless oil (87.4 mg, 0.24 mmol, 97%).

Meta ^{1}H NMR (500 MHz, $CDCl_3$) δ 7.79 (dd, *J* = 1.6, 8.2 Hz, 1H), 7.71 (d, *J* = 1.6 Hz, 1H), 6.91 (d, *J* = 8.2 Hz, 1H), 6.77 (br s, 1H), 4.53 (d, *J* = 5.6 Hz, 2H), 3.90 (s, 3H), 1.33 (s, 12H); **^{13}C NMR** (126 MHz, $CDCl_3$) δ 160.4, 156.7 (q, $^2J_{C\text{-}F}$ = 37 Hz), 137.1, 136.9, 123.2, 115.9 (q, $^1J_{C\text{-}F}$ = 283 Hz), 109.9, 83.8, 55.5, 40.2, 24.8; **HRMS**: m/z: $[M]^+$ calc'd for $[C_{16}H_{20}{}^{10}BNO_4F_3]^+$ expect 158.1552; found 158.1542.

By subtracting the resonances for the *meta* isomer, the NMR data of the *para* isomer can be assigned as follows:

Para ^{1}H NMR (500 MHz, $CDCl_3$) δ 7.41 (dd, *J* = 0.7, 7.3 Hz, 1H), 7.32 (s, 1H), 7.27 (d, *J* = 7.3 Hz, 1H), 6.89 (br s, 1H), 4.53 (d, *J* = 5.6 Hz, 2H), 3.92 (s, 3H), 1.35 (s, 12H); **^{13}C NMR** (126 MHz, $CDCl_3$) δ 157.0, 156.7 (q, $^2J_{C\text{-}F}$ = 37 Hz), 129.5, 127.7, 126.9, 116.0, 115.9 (q, $^1J_{C\text{-}F}$ = 283 Hz), 84.0, 55.5, 40.2, 24.8.

Dtbpy in THF:

Following general procedure F using 2,2,2-Trifluoro-*N*-(2-methoxybenzyl)acetamide (58.3 mg, 0.25 mmol), B_2pin_2 (127 mg, 0.50 mmol), $[Ir(COD)OMe]_2$ (2.5 mg, 0.00375 mmol) and dtbpy (2.0 mg, 0.0075 mmol) in THF (1.25 mL). The reaction was stirred at 50 °C for 20 hours before cooling and the solvents removed. Analysis of crude ^{1}H NMR using internal standard 1,2-dimethoxyethane showed 1:2 *meta*:*para* borylation in 70% yield. The crude product was purified by silica gel chromatography (Pet. Ether (40-60):EtOAc (19:1) to give the title compound (as a 1:2 mixture of *meta*:*para* ratio, as determined by ^{1}H NMR) as a colourless oil (53.1 mg, 0.15 mmol, 59%).

N-(2-chloro-5-(4,4,5,5-tetramethyl-1,3,2-dioxaborolan-2-yl)benzyl)-2,2,2-trifluoroacetamide (3d)

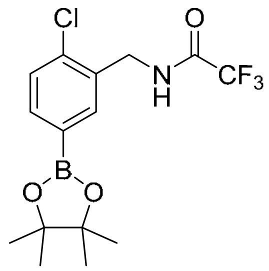

meta

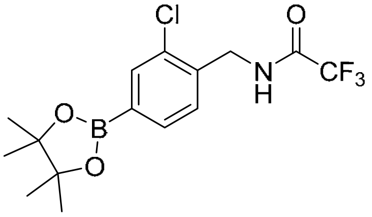

para

1a in THF:

Following general procedure F using *N*-(2-chlorobenzyl)-2,2,2-trifluoroacetamide (59.4 mg, 0.25 mmol), B_2pin_2 (127 mg, 0.50 mmol), $[Ir(COD)OMe]_2$ (2.5 mg, 0.00375 mmol) and **1a** (3.8 mg, 0.0075 mmol) in THF (1.25 mL). The reaction was stirred at 35 °C for 20 hours before cooling and the solvents removed. Analysis of crude ^{1}H NMR using internal standard 1,2-dimethoxyethane showed 10:76:15 *dimeta*:*meta*:*para* borylation in 100% yield. The crude product was purified by silica gel chromatography (Pet. Ether (40-60):EtOAc (19:1) to give the title compound in two fractions, the first as a 4.2:1 mixture of *meta*:*para* ratio, as determined by ^{1}H NMR, as a white solid (42.5 mg, 0.12 mmol, 47%) and the second as a 10:75:12:21 mixture of *dimeta*:*meta*:*para*:B_2pin_2 ratio as determined by ^{1}H NMR, as a colourless oil (56.3 mg, 0.13 mmol). This gave the combined yield of 88.8 mg, 0.24 mmol, 97% as quoted.

Meta ^{1}H NMR (600 MHz, $CDCl_3$) δ 7.80 (d, *J* = 1.2 Hz, 1H), 7.71 (dd, *J* = 1.9 Hz, 1.7 Hz, 1H), 7.41 (d, *J* = 7.9 Hz, 1H), 6.74 (br s, 1H), 4.63 (d, *J* = 5.7 Hz, 2H), 1.34 (s, 12H); **^{13}C NMR** (151 MHz, $CDCl_3$) δ 156.9 (q, $^2J_{C\text{-}F}$ = 37 Hz), 137.2, 137.1, 136.3, 132.5, 129.4, 115.7 (q, $^1J_{C\text{-}F}$ = 287 Hz), 84.3, 42.0, 24.8; **HRMS**: m/z: $[M + H]^+$ calc'd for $[C_{15}H_{19}ClBF_3NO_3]^+$ expect 364.1135; found 363.1125.

*Yield calculated from a weighted average mass of B_2pin_2, mono and diborylated products.

Dtbpy in THF:

Following general procedure F using *N*-(2-chlorobenzyl)-2,2,2-trifluoroacetamide (59.4 mg, 0.25 mmol), B_2pin_2 (127 mg, 0.50 mmol), $[Ir(COD)OMe]_2$ (2.5 mg, 0.00375 mmol) and dtbpy (2.0 mg, 0.0075 mmol) in THF (1.25 mL). The reaction was stirred at 35 °C for 20 hours before cooling and the solvents removed. Analysis of crude ^{1}H NMR using internal standard 1,2-dimethoxyethane showed 5:33:56 *dimeta*:*meta*:*para* borylation in 93% yield. The crude product was purified by silica gel chromatography (Pet. Ether (40-60):EtOAc (19:1) to give the title compound (as a 4:33:66 mixture of *dimeta*:*meta*:*para* ratio, as determined by ^{1}H NMR) as a colourless oil (64 mg, 0.18 mmol, 70%). By subtracting the resonances for the *meta* isomer, the NMR data of the *para* isomer can be assigned as follows:

Para ^{1}H NMR (600 MHz, $CDCl_3$) δ 7.82 (s, 1H), 7.68 (d, *J* = 7.3 Hz, 1H), 7.37 (d, *J* = 7.3 Hz, 1H), 6.87 (br s, 1H), 4.63 (d, *J* = 5.7 Hz, 1H), 1.34 (s, 12H); **^{13}C NMR** (151 MHz, $CDCl_3$) δ 157.1 (q, $^2J_{C\text{-}F}$ = 37 Hz), 136.0, 135.8, 133.6, 133.5, 129.9, 115.7 (q, $^1J_{C\text{-}F}$ = 287 Hz), 84.3, 41.9, 24.8.

2,2,2-Trifluoro-*N*-(3-fluoro-5-(4,4,5,5-tetramethyl-1,3,2-dioxaborolan-2-yl)benzyl)acetamide (3e)

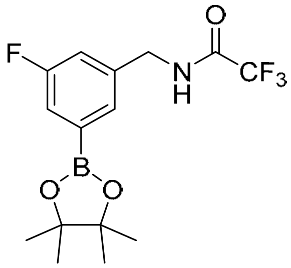

meta

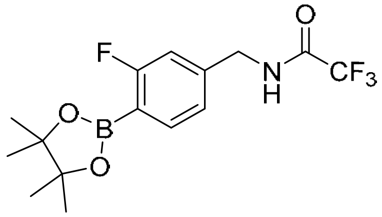

para

1a in THF:

Following general procedure F using 2,2,2-trifluoro-*N*-(3-fluorobenzyl)acetamide (55.3 mg, 0.25 mmol), B_2pin_2 (127 mg, 0.50 mmol), $[Ir(COD)OMe]_2$ (2.5 mg, 0.00375 mmol) and **1a** (3.8 mg, 0.0075 mmol) in THF (1.25 mL). The reaction was stirred at 50 °C for 20 hours before cooling and the solvents removed. Analysis of crude ^{1}H NMR using internal standard 1,2-dimethoxyethane showed 16.8:1 *meta*:*para* borylation in 71% yield. The crude product was purified by silica gel chromatography (Pet. Ether (40-60):EtOAc (9:1) to give the title compound as a white solid (60.5 mg, 0.17 mmol, 69%).

Meta ^{1}H NMR (600 MHz, $CDCl_3$) δ 7.49 (s, 1H), 7.43 (dd, *J* = 2.3, 8.6 Hz, 1H), 7.09 (dt, *J* = 9.2, 1.9 Hz, 1H), 6.74 (br s, 1H), 4.51 (d, *J* = 5.9 Hz, 2H), 1.35 (s, 12H); **^{13}C NMR** (151 MHz, $CDCl_3$) δ 162.8 (d, $^1J_{C\text{-}F}$ = 248 Hz), 157.2 (q, $^2J_{C\text{-}F}$ = 37 Hz), 137.6 (d, $^3J_{C\text{-}F}$ = 7 Hz), 129.7 (d, $^4J_{C\text{-}F}$ = 3 Hz), 121.0 (d, $^2J_{C\text{-}F}$ = 19 Hz), 117.7 (d, $^2J_{C\text{-}F}$ = 22 Hz), 115.8 (q, $^1J_{C\text{-}F}$ = 287 Hz), 84.4, 43.3 (d, $^4J_{C\text{-}F}$ = 1 Hz), 24.8; **HRMS**: m/z: $[M + H]^+$ calc'd for $[C_{15}H_{19}BF_4NO_3]^+$ expect 348.1389; found 348.1385.

Dtbpy in THF:

Following general procedure F using 2,2,2-trifluoro-*N*-(3-fluorobenzyl)acetamide (55.3 mg, 0.25 mmol), B_2pin_2 (127 mg, 0.50 mmol), $[Ir(COD)OMe]_2$ (2.5 mg, 0.00375 mmol) and dtbpy (2.0 mg, 0.0075 mmol) in THF (1.25 mL). The reaction was stirred at 50 °C for 20 hours before cooling and the solvents removed. Analysis of crude ^{1}H NMR using internal standard 1,2-dimethoxyethane showed 1.5:1 *meta*:*para* borylation in 53% yield. The crude product was subjected to silica gel chromatography (Pet. Ether (40-60):EtOAc (9:1) however significant decomposition was observed. A small *para* enriched fraction (1:2.7 *meta*:*para* isomer ration by ^{1}H NMR) was isolated as an off white solid (8 mg, 0.02 mmol, 9%). By subtracting the resonances for the *meta* isomer, the NMR data of the *para* isomer can be assigned as follows:

Para ^{1}H NMR (500 MHz, $CDCl_3$) δ 7.74 (dd, *J* = 6.2, 7.6 Hz, 1H), 7.06 (d, *J* = 1.3, 7.5 Hz, 1H), 6.96 (dd, *J* = 1.2, 9.7 Hz, 1H), 6.60 (br s, 1H), 4.54 (d, *J* = 5.8 Hz, 2H), 1.36 (s, 12H); **^{13}C NMR** (126 MHz, $CDCl_3$) δ 167.4 (d, $^1J_{C\text{-}F}$ = 303 Hz), 157.2 (q, $^2J_{C\text{-}F}$ = 37 Hz), 141.5 (d, $^3J_{C\text{-}F}$ = 10 Hz), 137.7 (d, $^3J_{C\text{-}F}$ = 8 Hz), 122.9 (d, $^4J_{C\text{-}F}$ = 3 Hz), 115.7 (q, $^1J_{C\text{-}F}$ = 287 Hz), 114.6 (d, $^2J_{C\text{-}F}$ = 25 Hz) 84.1, 43.3, 24.8.

2,2,2-trifluoro-N-(2-(hydroxymethyl)-5-(4,4,5,5-tetramethyl-1,3,2-dioxaborolan-2-yl)benzyl)acetamide (3f)

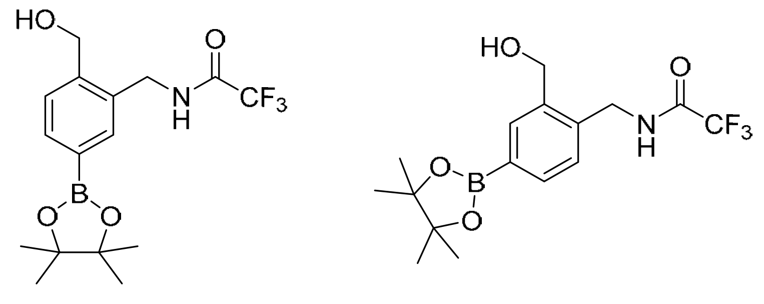

Meta ***Para***

1a in THF:

Following general procedure F using **2e** (58 mg, 0.25 mmol), B_2pin_2 (127 mg, 0.50 mmol), $[Ir(COD)OMe]_2$ (2.5 mg, 0.00375 mmol) and **1a** (3.8 mg, 0.0075 mmol) in THF (1.25 mL). Stirred in vial at 50 °C for 20 hours. Analysis of crude ^1H NMR using internal standard 1,2-dimethoxyethane showed 10.2:1 *meta*:*para* borylation in 80% yield (19% starting material remaining). The crude product was purified by silica gel chromatography (30% EtOAc in Petroleum Ether 40-60 °C), however this gave a mixture of the borylated products and starting material, which were inseparable on silica (10.5:1:2.0 mixture of *meta*:*para:*starting material ratio, as determined by ^1H NMR), as a white solid (76 mg). As the mixture contained 15% of starting material, the isolated yield was corrected by calculating the mass of the starting material in the mixture. Corrected yield for the borylated products was 68 mg, 0.19 mmol, 76%.

Meta ^1H NMR (600 MHz, $CDCl_3$) δ 7.98 (br, 1H), 7.80 (s, 1H), 7.74 (dd, *J* = 7.5, 1.0 Hz, 1H), 7.31 (d, *J* = 7.5 Hz, 1H), 4.74 (d, *J* = 3.9 Hz, 2H), 4.57 (d, *J* = 5.8 Hz, 2H), 3.17 (br, 1H), 1.33 (s, 12H); **^{13}C NMR** (151 MHz, $CDCl_3$) δ 156.7 (q, *J* = 36.9 Hz), 141.6, 137.0, 135.3, 134.5, 129.2, 115.9 (q, *J* = 287.7 Hz), 84.1, 63.9, 41.9, 24.8. **HRMS**: m/z: $[M]^+$ calc'd for $[C_{16}H_{21}BNO_4F_3]^+$ expect 359.1516 ; found 359.1504 .

Dtbpy in THF:

Following general procedure F using **2e** (58 mg, 0.25 mmol), B_2pin_2 (127 mg, 0.50 mmol), $[Ir(COD)OMe]_2$ (2.5 mg, 0.00375 mmol) and dtbpy (2.0 mg, 0.0075 mmol) in THF (1.25 mL). Stirred in vial at 50 °C for 20 hours. Analysis of crude ^1H NMR using internal standard 1,2-dimethoxyethane showed 1.4:1 *meta*:*para* borylation in 77% yield (19% starting material remaining). The crude product was purified by silica gel chromatography (30% EtOAc in Petroleum Ether 40-60 °C), however this gave a mixture of the borylated products and starting material, which were inseparable on silica (1.6:1:0.52 mixture of *meta*:*para:*starting material ratio, as determined by ^1H NMR), as a white solid (73 mg). The yield was corrected to account for the 20% of starting material. Corrected yield for the borylated products was 64 mg, 0.18 mmol, 71%. By subtracting the resonances for the *meta* isomer, the NMR data of the *para* isomer can be assigned as follows:

Para ^1H NMR (500 MHz, $CDCl_3$) δ 8.06 (br, 1H), 7.75 (dd, *J* = 7.5, 1.2 Hz, 1H), 7.72 (s, 1H), 7.37 (d, *J* = 7.5 Hz, 1H), 4.73 (m, 2H), 4.56 (d, *J* = 7.3 Hz, 2H), 1.33 (s, 12H); **^{13}C NMR** (126 MHz, $CDCl_3$) δ 156.7 (q, *J* = 36.9 Hz), 138.5, 137.7, 136.2, 135.7, 130.0, 115.9 (q, *J* = 287.6 Hz), 84.2, 64.1, 41.9, 24.8.

Methyl 4-(4,4,5,5-tetramethyl-1,3,2-dioxaborolan-2-yl)-2-((2,2,2-trifluoroacetamido)methyl)benzoate (3g)

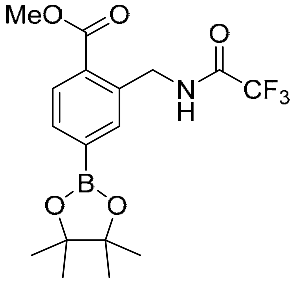

meta

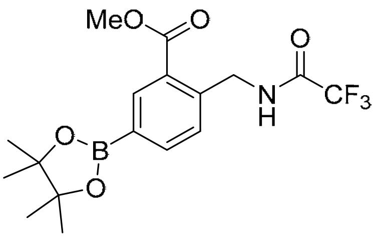

para

1a in THF:

Following general procedure methyl 2-((2,2,2-trifluoroacetamido)methyl)benzoate using (65.3 mg, 0.25 mmol), B_2pin_2 (127 mg, 0.50 mmol), $[Ir(COD)OMe]_2$ (2.5 mg, 0.00375 mmol) and **1a** (3.8 mg, 0.0075 mmol) in THF (1.25 mL). The reaction was stirred at 50 °C for 20 hours before cooling and the solvents removed. Analysis of crude ^{1}H NMR using internal standard 1,2-dimethoxyethane showed 7.3:1 *meta*:*para* borylation in 89% yield. The crude product was purified by silica gel chromatography (Pet. Ether (40-60):EtOAc (9:1) to give the title compound (as a 7.4:1 mixture of *meta*:*para* ratio, as determined by ^{1}H NMR) as a white solid (85.3 mg, 0.22 mmol, 88%).

Meta ^{1}H NMR (600 MHz, $CDCl_3$) δ 7.99 (d, *J* = 7.7 Hz, 1H), 7.94 (s, 1H), 7.85 (br s, 1H), 7.83 (d, *J* = 7.7 Hz, 1H), 4.69 (d, *J* = 6.5 Hz, 2H), 3.95 (s, 3H), 1.35 (s, 12H; **^{13}C NMR** (151 MHz, $CDCl_3$) δ 168.2, 156.6 (q, $^2J_{C\text{-}F}$ = 36 Hz), 138.2, 136.6, 134.8, 131.1, 130.3, 116.0 (q, $^1J_{C\text{-}F}$ = 289 Hz), 84.4, 52.6, 42.9, 24.8; **HRMS**: m/z: $[M + H]^+$ calc'd for $[C_{17}H_{22}BF_3NO_5]^+$ expect 387.1579; found 387.1568.

Dtbpy in THF:

Following general procedure F using methyl 2-((2,2,2-trifluoroacetamido)methyl)benzoate using (65.3 mg, 0.25 mmol), B_2pin_2 (127 mg, 0.50 mmol), $[Ir(COD)OMe]_2$ (2.5 mg, 0.00375 mmol) and dtbpy (2.0 mg, 0.0075 mmol) in THF (1.25 mL). The reaction was stirred at 50 °C for 20 hours before cooling and the solvents removed. Analysis of crude ^{1}H NMR using internal standard 1,2-dimethoxyethane showed 2.2:1 *meta*:*para* borylation in 88% yield. The crude product was purified by silica gel chromatography (Pet. Ether (40-60):EtOAc (9:1) to give the title compound (as a 2:1 mixture of *meta*:*para* ratio, as determined by ^{1}H NMR) as a colourless oil (81.2 mg, 0.21 mmol, 84%). By subtracting the resonances for the *meta* isomer, the NMR data of the *para* isomer can be assigned as follows:

Para ^{1}H NMR (600 MHz, $CDCl_3$) δ 8.42 (s, 1H), 7.96 (dd, *J* = 1.1, 7.5 Hz, 1H), 7.53 (d, *J* = 7.5 Hz, 1H), 4.68 (d, *J* = 5.3 Hz, 2H), 3.94 (s, 3H), 1.35 (s, 12H); **^{13}C NMR** (151 MHz, $CDCl_3$) δ 168.2, 156.7 (q, $^2J_{C\text{-}F}$ = 37 Hz), 140.3, 139.6, 137.4, 131.4, 128.3, 115.9 (q, $^1J_{C\text{-}F}$ = 289 Hz), 84.3, 52.4, 42.9, 24.8.

N-(3,5-bis(4,4,5,5-tetramethyl-1,3,2-dioxaborolan-2-yl)benzyl)-2,2,2-trifluoroacetamide (3h)

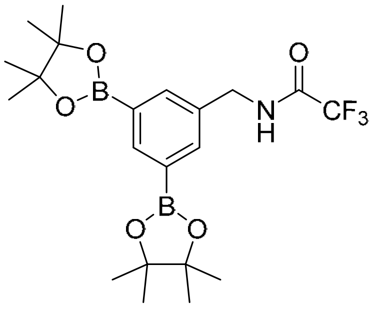

dimeta

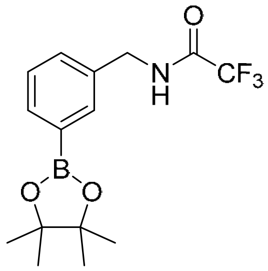

meta

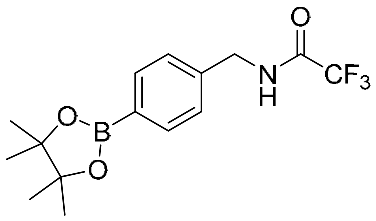

para

1a in THF:

Following general procedure F using *N*-benzyl-2,2,2-trifluoroacetamide (50.8 mg, 0.25 mmol), B_2pin_2 (254 mg, 1.0 mmol), $[Ir(COD)OMe]_2$ (2.5 mg, 0.00375 mmol) and **1a** (3.8 mg, 0.0075 mmol) in THF (1.25 mL). The reaction was stirred at 50 °C for 20 hours before cooling and the solvents removed. Analysis of crude ^{1}H NMR using internal standard 1,2-dimethoxyethane showed >20:1 *dimeta*:*para* borylation in 89% yield. The crude product was purified by silica gel chromatography (Pet. Ether (40-60):EtOAc (19:1-9:1) to give the title compound as a white solid (101 mg, 0.22 mmol, 89%).

Dimeta ^{1}H NMR (600 MHz, $CDCl_3$) δ 8.23 (s, 1H), 7.82 (s, 2H), 6.69 (br s, 1H), 4.50 (d, *J* = 5.5 Hz), 1.33 (s, 24H); **^{13}C NMR** (151 MHz, $CDCl_3$) δ 156.9 (q, $^2J_{C-F}$ = 37 Hz), 141.3, 137.4, 134.1, 115.8 (q, $^1J_{C-F}$ = 288 Hz), 84.0, 44.0, 24.8. **HRMS**: m/z: $[M + H]^+$ calc'd for $[C_{21}H_{31}{}^{10}B^{11}BF_3NO_5]^+$ expect 455.2377; found 455.2368.

Dtbpy in THF:

Following general procedure F using *N*-benzyl-2,2,2-trifluoroacetamide (50.8 mg, 0.25 mmol), B_2pin_2 (254 mg, 1.0 mmol), $[Ir(COD)OMe]_2$ (2.5 mg, 0.00375 mmol) and dtbpy (2.0 mg, 0.0075 mmol) in THF (1.25 mL). The reaction was stirred at 35 °C for 20 hours before cooling and the solvents removed. Analysis of crude ^{1}H NMR using internal standard 1,2-dimethoxyethane showed 23:31:17 *dimeta*:*meta*:*para* borylation in 86% yield. The crude product was purified by silica gel chromatography (Pet. Ether (40-60):EtOAc (19:1) to give the title compound (as a 58:27:14 mixture of *dimeta*:*meta*:*para* ratio, as determined by ^{1}H NMR) as a colourless oil (62.9 mg, 0.16 mmol, 63%). From an identical experiment a small fraction of the mono-*meta* isomer was isolated also a colourless oil (11 mg, 0.03 mmol, 13%) described below. By subtracting the resonances for the *dimeta* and *meta* isomers, the NMR data of the *para* isomer can be assigned as follows:

Meta ^{1}H NMR (500 MHz, $CDCl_3$) δ 7.79-7.77 (m, 1H), 7.72 (s, 1H), 7.42-7.39 (m, 2H), 6.51 (br s, 1H), 4.53 (d, *J* = 5.7 Hz, 2H), 1.35 (s, 12H); **^{13}C NMR** (126 MHz, $CDCl_3$) δ 157.0 (q, $^2J_{C-F}$ = 37 Hz), 135.0, 134.8, 134.4, 131.0, 128.6, 115.8 (q, $^1J_{C-F}$ = 289 Hz), 84.1, 44.0, 24.9.

Para ^{1}H NMR (500 MHz, $CDCl_3$) δ 7.80 (d, *J* = 8.1 Hz, 2H), 7.28 (d, *J* = 8.1 Hz, 2H), 6.69 (br s, 1H), 4.52 (d, *J* = 5.6 Hz, 2H), 1.34 (s, 12 H); **^{13}C NMR** (126 MHz, $CDCl_3$) δ 156.9 (q, $^2J_{C-F}$ = 37 Hz), 138.8, 135.5, 127.2, 115.8 (q, $^1J_{C-F}$ = 288 Hz), 84.1, 43.9, 24.9.

*conducting reaction at 35 °C avoided borylation of the amide nitrogen which complicated product assignment from the crude ^{1}H NMR.

(S)-*N*-(1-(3,5-bis(4,4,5,5-tetramethyl-1,3,2-dioxaborolan-2-yl)phenyl)ethyl)-2,2,2-trifluoroacetamide (3i)

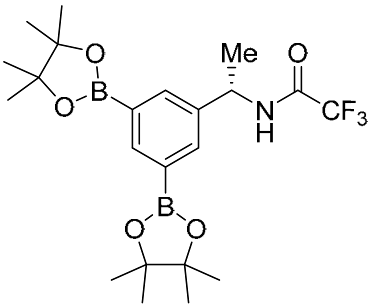

dimeta

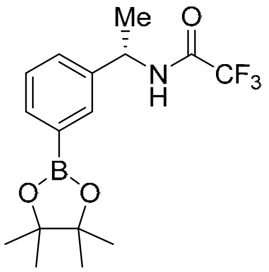

meta

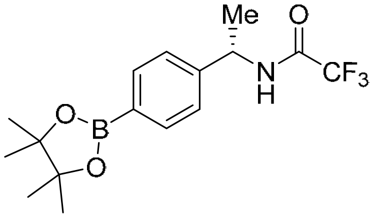

para

1A in THF:

Following general procedure F using (S)-2,2,2-trifluoro-*N*-(1-phenylethyl)acetamide (54.3 mg, 0.25 mmol), B_2pin_2 (254 mg, 1.0 mmol), $[Ir(COD)OMe]_2$ (2.5 mg, 0.00375 mmol) and **1A** (3.8 mg, 0.0075 mmol) in THF (1.25 mL). The reaction was stirred at 50 °C for 20 hours before cooling and the solvents removed. Analysis of crude ^{1}H NMR using internal standard 1,2-dimethoxyethane showed 71:20:8 *dimeta*:*meta*:*para* borylation in 99% yield. The crude product was purified by silica gel chromatography (Pet. Ether (40-60):EtOAc (19:1-9:1) to give two fractions: the title compound as a white solid (48.1 mg, 0.10 mmol, 41%) and a mixed fraction (as a 39:42:17:12 mixture of *dimeta*:*meta*:*para*:B_2pin_2 ratio, as determined by ^{1}H NMR) as a colourless oil (40.1 mg, 0.09 mmol, 38%*), the quoted yield is that of the *dimeta* product. Using a combination of 2D NMR analysis the *dimeta* and both mono borylated products were assigned as follows:

Dimeta ^{1}H NMR (600 MHz, $CDCl_3$) δ 8.24 (s, 1H), 7.84 (d, *J* = 0.7 Hz, 2H), 6.47 (br s, 1H), 5.15 (dq, *J* = 7.3, 7.0 Hz, 1H), 1.60 (d, *J* = 7.0 Hz, 3H), 1.35 (s, 24H); ^{13}**C NMR** (151 MHz, $CDCl_3$) δ 156.1 (q, $^2J_{C-F}$ = 38 Hz), 141.3, 139.4, 135.3, 115.8 (q, $^1J_{C-F}$ = 288 Hz), 84.0, 50.2, 24.9, 24.8, 21.4; **HRMS:** m/z: $[M + H]^+$ calc'd for $[C_{22}H_{33}B_2NO_5F_3]^+$ expect 470.2497; found 470.2505.

Meta ^{1}H NMR (600 MHz, $CDCl_3$) δ 7.77 (dt, *J* = 6.9, 1.5 Hz, 1H), 7.75 (br s, 1H), 7.41 (dt, *J* = 7.8, 1.6 Hz, 1H), 7.38 (t, *J* = 7.6 Hz, 1H), 6.51 (br s, 1H), 5.14 (quint, *J* = 7.0 Hz, 1H), 1.59 (d, *J* = 0.9 Hz, 3H), 1.35 (s, 12H); ^{13}**C NMR** (126 MHz, $CDCl_3$) δ 156.2 (q, $^2J_{C-F}$ = 37 Hz), 140.2, 134.7, 132.2, 129.4, 128.4, 115.7 (q, $^1J_{C-F}$ = 286 Hz), 84.0, 50.0, 24.9, 24.8, 21.2.

Para ^{1}H NMR (500 MHz, $CDCl_3$) δ 7.82 (d, *J* = 8.0 Hz, 2H), 7.32 (d, *J* = 8.0 Hz, 2H), 6.51 (br s, 1H), 5.14 (quint, *J* = 7.0 Hz, 1H), 1.57 (s, 3H), 1.34 (s, 12H); ^{13}**C NMR** (126 MHz, $CDCl_3$) δ 156.3 (q, $^2J_{C-F}$ = 38 Hz), 143.9, 139.4, 125.5, 115.8 (q, $^1J_{C-F}$ = 287 Hz), 83.9, 49.9, 50.2, 25.0, 21.0.

*Yield calculated from a weighted average mass of B_2pin_2, mono and diborylated products.

Dtbpy in THF:

Following general procedure F using (S)-2,2,2-trifluoro-*N*-(1-phenylethyl)acetamide (54.3 mg, 0.25 mmol), B_2pin_2 (254 mg, 1.0 mmol), $[Ir(COD)OMe]_2$ (2.5 mg, 0.00375 mmol) and dtbpy (2.0 mg, 0.0075 mmol) in THF (1.25 mL). The reaction was stirred at 50 °C for 20 hours before cooling and the solvents removed. Analysis of crude ^{1}H NMR using internal standard 1,2-dimethoxyethane showed 2.9:1 *dimeta*:*para* borylation in 97% yield. As all isomers could be assigned from the reaction with **1a** the mixture was not isolated and NMR yields were used for all comparisons.

2,2,2-Trifluoro-*N*-(2-fluoro-3,5-bis(4,4,5,5-tetramethyl-1,3,2-dioxaborolan-2-yl)benzyl)acetamide (3j)

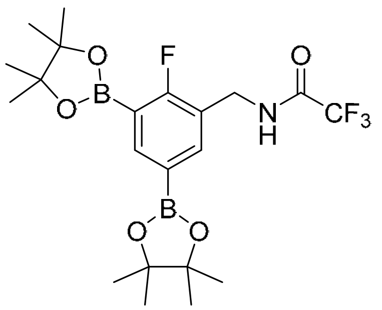

dimeta

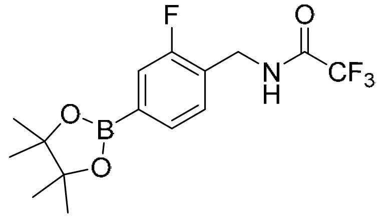

para

1A in THF:

Following general procedure F using 2,2,2-trifluoro-*N*-(2-fluorobenzyl)acetamide (55.3 mg, 0.25 mmol), B_2pin_2 (254 mg, 1.0 mmol), $[Ir(COD)OMe]_2$ (2.5 mg, 0.00375 mmol) and **1A** (3.8 mg, 0.0075 mmol) in THF (1.25 mL). The reaction was stirred at 35 °C for 20 hours before cooling and the solvents removed. Analysis of crude ^1H NMR using internal standard 1,2-dimethoxyethane showed 7.4:1 *dimeta*:*para* borylation in 99% yield. The crude product was purified by silica gel chromatography (Pet. Ether (40-60):EtOAc (19:1-17:3) to yield a clean fraction of the *dimeta* product as a white solid (75 mg, 0.16 mmol, 63%).

Dimeta ^1H NMR (600 MHz, $CDCl_3$) δ 8.17 (d, J = 6.1 Hz, 1H), 7.87 (d, J = 8.0 Hz, 1H) 6.70 (br s, 1H), 4.56 (d, J = 5.7 Hz, 2H), 1.36 (s, 12H), 1.33 (s, 12H); **^{13}C NMR** (151 MHz, $CDCl_3$) δ 167.7 (d, $^1J_{C\text{-}F}$ = 255.5 Hz), 156.9 (q, $^2J_{C\text{-}F}$ = 37 Hz), 144.3 (d, $^3J_{C\text{-}F}$ = 9 Hz), 140.7 (d, $^3J_{C\text{-}F}$ = 5.1 Hz), 121.9 (d, $^2J_{C\text{-}F}$ = 17 Hz), 115.8 (q, $^1J_{C\text{-}F}$ = 288 Hz), 84.13, 84.12, 38.4 (d, $^3J_{C\text{-}F}$ = 3 Hz), 24.81, 24.80; **HRMS**: m/z: $[M + H]^+$ calc'd for $[C_{21}H_{30}B_2F_4NO_5]^+$ expect 472.2319; found 472.2302.

*Yield calculated from a weighted average mass of mono and diborylated products.

Dtbpy in THF:

Following general procedure F using 2,2,2-trifluoro-*N*-(2-fluorobenzyl)acetamide (55.3 mg, 0.25 mmol), B_2pin_2 (254 mg, 1.0 mmol), $[Ir(COD)OMe]_2$ (2.5 mg, 0.00375 mmol) and dtbpy (2.0 mg, 0.0075 mmol) in THF (1.25 mL). The reaction was stirred at 35 °C for 20 hours before cooling and the solvents removed. Analysis of crude ^1H NMR using internal standard 1,2-dimethoxyethane showed 56:6:24 *dimeta*:*meta*:*para* borylation in 86% yield. The crude product was purified by silica gel chromatography (Pet. Ether (40-60):EtOAc (19:1-15:5) to give a *para* enriched fraction suitable for characterisation containing trace amounts of the mono *meta* products and as a 1:2 mixture of *para*:B_2pin_2 (as determined by ^1H NMR) as a colourless oil (8.1 mg) with NMR yield used for comparison. The *para* isomer can be assigned as follows:

Para ^1H NMR (600 MHz, $CDCl_3$) δ 7.56 (dd, J = 0.8, 7.4 Hz, 1H), 7.49 (d, J = 10.4 Hz, 1H), 7.33 (t, J = 7.4 Hz, 1H), 6.72 (br s, 1H), 4.59 (d, J = 6.0 Hz, 2H), 1.33 (s, 12H); **^{13}C NMR** (151 MHz, $CDCl_3$) δ 160.7 (d, $^1J_{C\text{-}F}$ = 247 Hz), 157.1 (q, $^2J_{C\text{-}F}$ = 37 Hz), 130.9 (d, $^3J_{C\text{-}F}$ = 3 Hz), 129.8 (d, $^3J_{C\text{-}F}$ = 3 Hz), 125.8 (d, $^2J_{C\text{-}F}$ = 15 Hz), 121.0 (d, $^2J_{C\text{-}F}$ = 19 Hz), 115.8 (q, $^1J_{C\text{-}F}$ = 287 Hz), 84.3, 38.1 (d, $^3J_{C\text{-}F}$ = 3 Hz), 24.8.

2,2,2-Trifluoro-*N*-((4-(4,4,5,5-tetramethyl-1,3,2-dioxaborolan-2-yl)pyridin-2-yl)methyl)acetamide (3k)

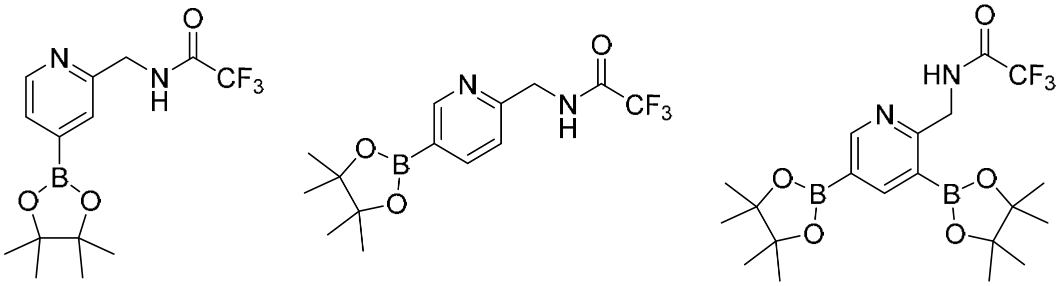

C4 **C5** **C3,5**

1a in THF:

Following general procedure F using 2,2,2-trifluoro-*N*-(pyridin-2-ylmethyl)acetamide (51.0 mg, 0.25 mmol), B_2pin_2 (95 mg, 0.375 mmol), $[Ir(COD)OMe]_2$ (2.5 mg, 0.00375 mmol) and **1a** (3.8 mg, 0.0075 mmol) in THF (1.25 mL). The reaction was stirred at 35 °C for 20 hours before cooling and the solvents removed. Analysis of crude ^{1}H NMR using internal standard 1,2-dimethoxyethane showed 8.0:1:1 C4:C5-borylation:SM in 69% yield. The crude product was unable to be purified by silica gel chromatography, however a small sample of the C4 product was isolated clean from flushing the silica with EtOAc (mixed fractions) followed by 20% MeOH in EtOAc (clean C4 fraction) as an off white solid (9.9 mg, 0.03 mmol, 12%):

C4 ^{1}H NMR (500 MHz, $CDCl_3$) δ 8.58 (dd, *J* = 0.8 4.8 Hz, 1H), 8.04 (br, s), 7.61 (s, 1H), 7.59 (d, *J* = 4.8 Hz, 1H), 4.63 (d *J* = 5.7 Hz, 2H), 1.36 (s, 12H); **^{13}C NMR** (126 MHz, $CDCl_3$) δ 157.1 (q, $^2J_{C\text{-}F}$ = 37 Hz), 152.7, 158.4, 128.0, 127.2, 115.9 (q, $^1J_{C\text{-}F}$ = 287 Hz), 84.8, 43.9, 24.9; **HRMS** m/z: $[M + H]^+$ calc'd for $[C_{14}H_{19}BF_3N_2O_3]^+$ expect 331.1435; found 331.1420.

Dtbpy in THF:

Following general procedure F using 2,2,2-trifluoro-*N*-(pyridin-2-ylmethyl)acetamide (51.0 mg, 0.25 mmol), B_2pin_2 (95 mg, 0.375 mmol), $[Ir(COD)OMe]_2$ (2.5 mg, 0.00375 mmol) and dtbpy (2.0 mg, 0.0075 mmol) in THF (1.25 mL). The reaction was stirred at 35 °C for 20 hours before cooling and the solvents removed. Analysis of crude ^{1}H NMR using internal standard 1,2-dimethoxyethane showed 30:30:16:9 C4:C5:C3,5-borylation:SM in 60% yield. The crude product was unable to be purified by silica gel chromatography and was hence characterised from the crude. By subtracting the resonances for the C4 isomer, SM, and using a combination of 2D NMR analysis the NMR data of the C5 and C3,5 isomers can be assigned as follows:

C5 ^{1}H NMR (600 MHz, $CDCl_3$) δ 8.85 (s, 1H), 8.4 (br s, 1H), 8.07 (dd, *J* = 1.6, 7.7 Hz, 1H), 7.34 (d, *J* = 7.7 Hz, 1H), 4.57 (d, *J* = 8.2 Hz, 2H), 1.32 (s, 12H); ^{13}C NMR (151 MHz, $CDCl_3$) δ ~157.2 (q, $^2J_{C\text{-}F}$ = 37 Hz), 156.4, 154.7, 143.8, 122.4, ~115.9 (q, $^1J_{C\text{-}F}$ = 287 Hz), 84.4, 42.2, 24.8.

C3,5 ^{1}H NMR (600 MHz, $CDCl_3$) δ 8.89 (d, *J* = 1.7 Hz, 1H), 8.61 (br s, 1H), 8.49 (d, *J* = 1.7 Hz, 1H), 4.85 (d, *J* = 4.7 Hz, 2H), 1.34 (s, 12H), 1.32 (s, 12H); ^{13}C NMR (151 MHz, $CDCl_3$) δ 161.1, ~157.2 (q, $^2J_{C\text{-}F}$ = 37 Hz), 156.4, 151.0, ~115.9 (q, $^1J_{C\text{-}F}$ = 287 Hz), 84.6, 84.3, 44.3, 24.8, 24.8.

_N_-(5-(4,4,5,5-tetramethyl-1,3,2-dioxaborolan-2-yl)-2-(trifluoromethyl)benzyl)methanesulfonamide (3m)

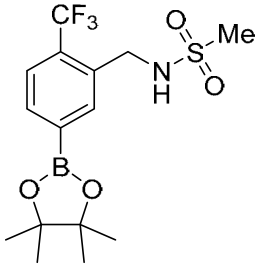

meta

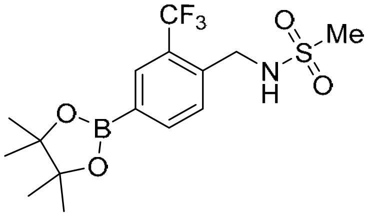

para

1A in THF:

Following general procedure F using *N*-(2-(trifluoromethyl)benzyl)methanesulfonamide (63.3 mg, 0.25 mmol), B_2pin_2 (95 mg, 0.375 mmol), $[Ir(COD)OMe]_2$ (2.5 mg, 0.00375 mmol) and **1A** (3.8 mg, 0.0075 mmol) in THF (1.25 mL). The reaction was stirred at 50 °C for 20 hours before cooling and the solvents removed. Analysis of crude ^{1}H NMR using internal standard 1,2-dimethoxyethane showed 7.5:1 *meta*:*para* borylation in 94% yield. The crude product was purified by silica gel chromatography (Pet. Ether (40-60):EtOAc (7:3) to give the title compound (as a 62:10:1 mixture of *meta*:*para* ratio with pinacol and B_2pin_2 contaminants, as determined by ^{1}H NMR) as a white solid (90 mg), due to multiple impurities, the NMR yield was used for all comparisons.

Meta ^{1}H NMR (500 MHz, $CDCl_3$) δ 8.00 (s, 1H), 7.83 (d, *J* = 7.8 Hz, 1H), 7.65 (d, *J* = 7.8 Hz, 1H), 5.93 (br s, 1H), 4.49 (d, *J* = 6.2 Hz, 2H), 2.92 (s, 3H), 1.34 (s, 12H); **^{13}C NMR** (126 MHz, $CDCl_3$) 136.8, 134.6, 133.9, 130.4 (q, $^2J_{C\text{-}F}$ = 31 Hz), 125.5 (q, $^5J_{C\text{-}F}$ = 5 Hz), 124.3 (q, $^1J_{C\text{-}F}$ = 273 Hz), 84.5, 43.9, 40.8, 24.8; **HRMS** m/z: $[M + Na]^+$ calc'd for $[C_{15}H_{21}BF_3NO_3SNa]^+$ expect 402.1134; found 402.1129.

Dtbpy in THF:

Following general procedure F using *N*-(2-(trifluoromethyl)benzyl)methanesulfonamide (63.3 mg, 0.25 mmol), B_2pin_2 (95 mg, 0.375 mmol), $[Ir(COD)OMe]_2$ (2.5 mg, 0.00375 mmol) and dtbpy (2.0 mg, 0.0075 mmol) in THF (1.25 mL). The reaction was stirred at 35 °C* for 20 hours before cooling and the solvents removed. Analysis of crude ^{1}H NMR using internal standard 1,2-dimethoxyethane showed 1:2 *meta*:*para* borylation in 72% yield. The crude product was purified by silica gel chromatography (Pet. Ether (40-60):EtOAc (7:3) to give the title compound (as a 26:52:18 mixture of *meta*:*para*:SM ratio as determined by ^{1}H NMR) as a colourless oil (91.6 mg) due to the presence of the SM the NMR yield was used for all comparisons. By subtracting the resonances for the *meta* isomer and the SM, the NMR data of the *para* isomer can be assigned as follows:

Para ^{1}H NMR (600 MHz, $CDCl_3$) δ 8.09 (s, 1H), 7.99 (d, *J* = 7.9 Hz, 1H), 7.65 (d, *J* = 7.8 Hz, 1H), 4.78 (t, *J* = 6.0, 1H), 4.51 (d, *J* = 6.0 Hz, 2H), 2.89 (s, 3H), 1.35 (s, 12H); **^{13}C NMR** (151 MHz, $CDCl_3$) δ 138.1, 137.8, 132.5 (q, $^3J_{C\text{-}F}$ = 5 Hz), 130.0, 127.5 (q, $^2J_{C\text{-}F}$ = 31 Hz), 124.5 (q, $^1J_{C\text{-}F}$ = 273 Hz), 84.4, 44.0, 41.2, 24.9.

*A lower temperature was required to avoid amide N-H borylation which confused the ratio in the crude reaction mixture.

4-Methyl-*N*-(5-(4,4,5,5-tetramethyl-1,3,2-dioxaborolan-2-yl)-2-(trifluoromethyl)benzyl)benzenesulfonamide (3n)

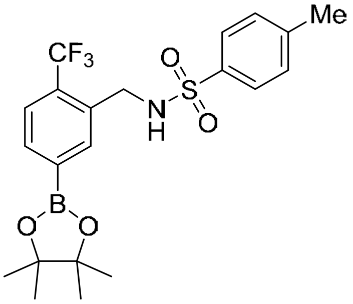

meta

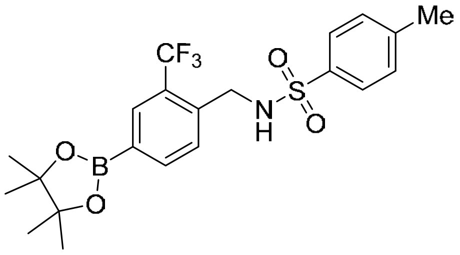

para

1A in THF:

Following general procedure F using 4-methyl-*N*-(2-(trifluoromethyl)benzyl)benzenesulfonamide (82.3 mg, 0.25 mmol), B_2pin_2 (95 mg, 0.375 mmol), $[Ir(COD)OMe]_2$ (2.5 mg, 0.00375 mmol) and **1A** (3.8 mg, 0.0075 mmol) in THF (1.25 mL). The reaction was stirred at 50 °C for 20 hours before cooling and the solvents removed. Analysis of crude ^{1}H NMR using internal standard 1,2-dimethoxyethane showed 2.5:1 *meta*:*para* borylation in 88% yield. The crude product was purified by silica gel chromatography (Pet. Ether (40-60):EtOAc (9:1) to give the title compound (as a 17:35:1 mixture of *meta*:*para* ratio as determined by ^{1}H NMR) as a white solid (93 mg), due to the presence of the SM the NMR yield was used for all comparisons. Using a combination of 2D NMR analysis the *meta* and *para* isomers are described:

Meta ^{1}H NMR (500 MHz, $CDCl_3$) δ 7.90 (s, 1H), 7.76 (d, *J* = 7.8 Hz, 1H), 7.72 (d, *J* = 8.2 Hz, 2H), 7.55 (d, *J* = 7.8 Hz, 1H), 7.25 (d, *J* = 8.2 Hz, 2H), 4.94 (t, *J* = 6.0 Hz, 1H), 4.29 (d, *J* = 6.0 Hz, 2H), 2.40 (s, 3H), 1.34 (s, 12H); **^{13}C NMR** (151 MHz, $CDCl_3$) δ 143.5, 136.9, 136.8, 134.3, 133.5, 130.2 (q, $^2J_{C\text{-}F}$ = 32 Hz), 129.7, 127.1, 125.1 (q, $^2J_{C\text{-}F}$ = 6 Hz), 124.2 (q, $^1J_{C\text{-}F}$ = 273 Hz), 84.4, 43.8, 24.8, 21.5; **HRMS** m/z: $[M + H]^+$ calc'd for $[C_{21}H_{26}BF_3NO_4S]^+$ expect 456.1622; found 456.1625.

Para ^{1}H NMR (126 MHz, $CDCl_3$) δ 8.00 (s, 1H), 7.87 (d, *J* = 7.8 Hz, 1H), 7.72 (d, *J* = 8.2 Hz, 2H), 7.55 (d, *J* = 7.8 Hz, 1H), 7.27 (d, *J* = 8.2 Hz, 2H), 5.10 (t, *J* = 5.9 Hz, 1H), 4.29 (d, *J* = 5.9 Hz, 2H), 2.40 (s, 3H), 1.33 (s, 12H); **^{13}C NMR** (151 MHz, $CDCl_3$) δ 143.6, 138.6, 137.8, 136.8, 132.0 (q, $^3J_{C\text{-}F}$ = 5 Hz), 129.8, 129.7, 127.1, 125.2 (q, $^2J_{C\text{-}F}$ = 30 Hz), 124.1 (q, $^1J_{C\text{-}F}$ = 275 Hz), 84.3, 43.7, 24.8, 21.5.

Dtbpy in THF:

Following general procedure F using 4-methyl-*N*-(2-(trifluoromethyl)benzyl)benzenesulfonamide (82.3 mg, 0.25 mmol), B_2pin_2 (95 mg, 0.375 mmol), $[Ir(COD)OMe]_2$ (2.5 mg, 0.00375 mmol) and dtbpy (2.0 mg, 0.0075 mmol) in THF (1.25 mL). The reaction was stirred at 35 °C* for 20 hours before cooling and the solvents removed. Analysis of crude ^{1}H NMR using internal standard 1,2-dimethoxyethane showed 1:2 *meta*:*para* borylation in 84% yield. As the *para* isomer could be characterised from the reaction with 1a the product was not isolated.

*A lower temperature was required to avoid amide N-H borylation which confused the ratio in the crude reaction mixture.

N-(5-(4,4,5,5-tetramethyl-1,3,2-dioxaborolan-2-yl)-2-(trifluoromethyl)benzyl)acetamide (3o)

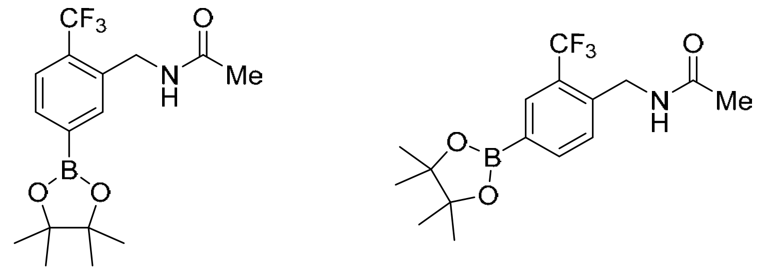

meta ***para***

1A in THF:

Following general procedure F using *N*-(2-(trifluoromethyl)benzyl)acetamide (54.3 mg, 0.25 mmol), B_2pin_2 (95 mg, 0.375 mmol), $[Ir(COD)OMe]_2$ (2.5 mg, 0.00375 mmol) and **1A** (3.8 mg, 0.0075 mmol) in THF (1.25 mL). The reaction was stirred at 50 °C for 20 hours before cooling and the solvents removed. Analysis of crude ^1H NMR using internal standard 1,2-dimethoxyethane showed 5.6:1 *meta*:*para* borylation in 91% yield. The crude product was purified by silica gel chromatography (Pet. Ether (40-60):EtOAc (7:3) to give the title compound (as a 6.4:1 mixture of *meta*:*para* ratio with pinacol and B_2pin_2 contaminants, as determined by ^1H NMR) as a white solid (62.0 mg), due to multiple impurities, the NMR yield was used for all comparisons.

Meta ^1H NMR (600 MHz, $CDCl_3$) δ 7.91 (s, 1H), 7.80 (d, *J* = 7.7 Hz, 1H), 7.62 (d, *J* = 7.7 Hz, 1H), 5.93 (br s, 1H), 4.60 (d, *J* = 5.9 Hz, 2H), 2.01 (s, 3H), 1.33 (s, 12H); ^{13}**C NMR** (151 MHz, $CDCl_3$) δ 169.9, 136.9, 135.4, 134.0, 130.5 (q, $^2J_{C-F}$ = 30 Hz), 125.2 (q, $^2J_{C-F}$ = 6 Hz), 124.3 (q, $^1J_{C-F}$ = 275 Hz), 84.3, 40.3 (q, $^3J_{C-F}$ = 2 Hz), 24.8, 23.2; **HRMS** m/z: $[M + H]^+$ calc'd for $[C_{16}H_{22}BF_3NO_3]^+$ expect 344.1645; found 344.1646.

Dtbpy in THF:

Following general procedure F using *N*-(2-(trifluoromethyl)benzyl)acetamide (54.3 mg, 0.25 mmol), B_2pin_2 (95 mg, 0.375 mmol), $[Ir(COD)OMe]_2$ (2.5 mg, 0.00375 mmol) and dtbpy (2.0 mg, 0.0075 mmol) in THF (1.25 mL). The reaction was stirred at 50 °C for 20 hours before cooling and the solvents removed. Analysis of crude ^1H NMR using internal standard 1,2-dimethoxyethane showed 1:1.1 *meta*:*para* borylation in 92% yield. The crude product was purified by silica gel chromatography (Pet. Ether (40-60):EtOAc (7:3) to give the title compound (as a 1:1.9 mixture of *meta*:*para* ratio with pinacol and B_2pin_2 as determined by ^1H NMR) as a white solid (59.7 mg), due to multiple impurities, the NMR yield was used for all comparisons. By subtracting the resonances for the *meta* isomer, the NMR data of the *para* isomer can be assigned as follows:

Para ^1H NMR (600 MHz, $CDCl_3$) δ 8.05 (s, 1H), 7.92 (d, *J* = 7.7 Hz, 1H), 7.53 (d, *J* = 7.7 Hz, 1H), 6.06 (br s, 1H), 4.60 (d, *J* = 5.8 Hz, 2H), 2.00 (s, 3H), 1.33 (s, 12H); ^{13}**C NMR** (151 MHz, $CDCl_3$) δ 170.1, 139.5, 138.6, 132.1 (q, $^3J_{C-F}$ = 5 Hz), 129.9, 127.5 (q, $^2J_{C-F}$ = 30 Hz), 124.5 (q, $^1J_{C-F}$ = 275 Hz), 84.3, 40.2, 24.8, 23.1.

tert-Butyl (5-(4,4,5,5-tetramethyl-1,3,2-dioxaborolan-2-yl)-2-(trifluoromethyl)benzyl)carbamate (3p)

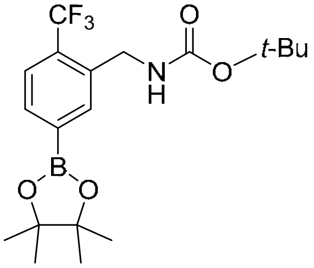

meta

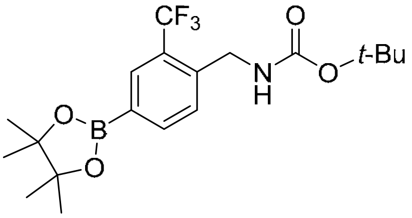

para

1a in THF:

Following general procedure F using *tert*-butyl (2-(trifluoromethyl)benzyl)carbamate (68.8 mg, 0.25 mmol), B_2pin_2 (95 mg, 0.375 mmol), $[Ir(COD)OMe]_2$ (2.5 mg, 0.00375 mmol) and **1a** (3.8 mg, 0.0075 mmol) in THF (1.25 mL). The reaction was stirred at 50 °C for 20 hours before cooling and the solvents removed. Analysis of crude ^{1}H NMR using internal standard 1,2-dimethoxyethane showed 2.8:1 *meta*:*para* borylation in 100% yield. The crude product was purified by silica gel chromatography (Pet. Ether (40-60):EtOAc (19:1) to give the title compound (as a 2.3:1 mixture of *meta*:*para* ratio as determined by ^{1}H NMR) as a white solid (80.4 mg, 0.20 mmol, 80%). Using a combination of 2D NMR analysis the *meta* and *para* isomers are described:

Meta ^{1}H NMR (500 MHz, $CDCl_3$) δ 7.93 (s, 1H), 7.78 (d, *J* = 7.7 Hz, 1H), 7.62 (d, *J* = 7.6 Hz, 1H), 4.89 (br s, 1H), 4.51 (d, *J* = 5.4 Hz, 2H), 1.47 (s, 9H), 1.34 (s, 12H); **^{13}C NMR** (151 MHz, $CDCl_3$) δ 155.8, 136.1, 135.8, 133.7, 130.3 (q, $^2J_{C\text{-}F}$ = 32 Hz), 125.1 (q, $^2J_{C\text{-}F}$ = 5 Hz), 124.4 (q, $^1J_{C\text{-}F}$ = 274 Hz), 84.3, 41.3, 28.4, 24.9; **HRMS** m/z: $[M + Na]^+$ calc'd for $[C_{19}H_{27}BF_3NO_4Na]^+$ expect 424.1883; found 424.1887.

Para ^{1}H NMR (126 MHz, $CDCl_3$) δ 8.06 (s, 1H), 7.94 (d, *J* = 7.6 Hz, 1H), 7.56 (d, *J* = 7.6 Hz, 1H), 4.92 (br s, 1H), 4.51 (d, *J* = 5.4 Hz, 2H), 1.44 (s, 9H), 1.34 (s, 12H); **^{13}C NMR** (151 MHz, $CDCl_3$) δ 155.8, 140.4, 138.9, 132.1 (q, $^3J_{C\text{-}F}$ = 5 Hz), 129.2, 127.3 (q, $^2J_{C\text{-}F}$ = 30 Hz), 124.5 (q, $^1J_{C\text{-}F}$ = 274 Hz), 84.3, 41.3, 28.4, 24.9.

Dtbpy in THF:

Following general procedure F using *tert*-butyl (2-(trifluoromethyl)benzyl)carbamate (68.8 mg, 0.25 mmol), B_2pin_2 (95 mg, 0.375 mmol), $[Ir(COD)OMe]_2$ (2.5 mg, 0.00375 mmol) and dtbpy (2.0 mg, 0.0075 mmol) in THF (1.25 mL). The reaction was stirred at 50 °C for 20 hours before cooling and the solvents removed. Analysis of crude ^{1}H NMR using internal standard 1,2-dimethoxyethane showed 1:1.6 *meta*:*para* borylation in 98% yield. As the *para* isomer could be characterised from the reaction with 1a the product was not isolated.

N-(5-(4,4,5,5-tetramethyl-1,3,2-dioxaborolan-2-yl)-2-(trifluoromethyl)benzyl)pivalamide (3q)

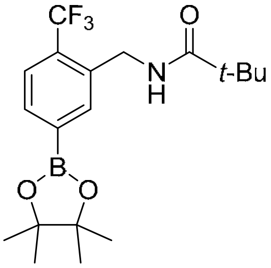

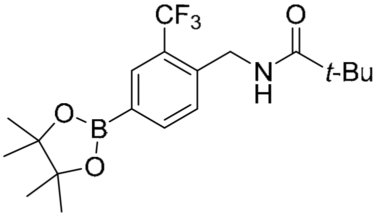

meta ***para***

1A in THF:

Following general procedure F using *N*-(2-(trifluoromethyl)benzyl)pivalamide (64.8 mg, 0.25 mmol), B_2pin_2 (95 mg, 0.375 mmol), $[Ir(COD)OMe]_2$ (2.5 mg, 0.00375 mmol) and **1A** (3.8 mg, 0.0075 mmol) in THF (1.25 mL). The reaction was stirred at 50 °C for 20 hours before cooling and the solvents removed. Analysis of crude ^{1}H NMR using internal standard 1,2-dimethoxyethane showed 3.8:1 *meta*:*para* borylation in 91% yield. The crude product was purified by silica gel chromatography (Pet. Ether (40-60):EtOAc (19:1-6:4) to give the title compound (as a 3.0:1 mixture of *meta*:*para* ratio as determined by ^{1}H NMR) as a white solid (69.1 mg, 0.18 mmol, 72%). Using a combination of 2D NMR analysis the *meta* and *para* isomers are described:

Meta ^{1}H NMR (600 MHz, $CDCl_3$) δ 7.87 (s, 1H), 7.79 (d, *J* = 7.6 Hz, 1H), 7.63 (d, *J* = 7.6 Hz, 1H), 6.00 (br s, 1H), 4.60 (d, *J* = 5.8 Hz, 2H), 1.33 (s, 12H), 1.21 (s, 9H); **^{13}C NMR** (151 MHz, $CDCl_3$) δ 178.1, 136.5, 135.6, 133.8, 130. (q, $^2J_{C-F}$ = 30 Hz), 125.3 (q, $^2J_{C-F}$ = 5 Hz), 124.4 (q, $^1J_{C-F}$ = 274 Hz), 84.3, 40.5 (q, $^3J_{C-F}$ = 2 Hz), 38.7, 27.4, 24.8; **HRMS** m/z: $[M + H]^+$ calc'd for $[C_{19}H_{28}BF_3NO_3]^+$ expect 386.2109 found 386.2091.

Para ^{1}H NMR (600 MHz, $CDCl_3$) δ 8.06 (s, 1H), 7.92 (d, *J* = 7.7 Hz, 1H), 7.50 (d, *J* = 7.7 Hz, 1H), 6.07 (br t, *J* = 5.3 Hz, 1H), 4.60 (d, *J* = 5.8 Hz, 2H), 1.34 (s, 12H), 1.18 (s, 9H); **^{13}C NMR** (151 MHz, $CDCl_3$) δ 178.2, 139.8, 138.6, 132.1 (q, $^3J_{C-F}$ = 5 Hz), 129.8, 127.5 (q, $^2J_{C-F}$ = 31 Hz), 124.5 (q, $^1J_{C-F}$ = 275 Hz), 84.2, 40.3 (q, $^3J_{C-F}$ = 2 Hz), 38.7, 27.4, 24.8.

Dtbpy in THF:

Following general procedure F using *N*-(2-(trifluoromethyl)benzyl)pivalamide (64.8 mg, 0.25 mmol), B_2pin_2 (95 mg, 0.375 mmol), $[Ir(COD)OMe]_2$ (2.5 mg, 0.00375 mmol) and dtbpy (2.0 mg, 0.0075 mmol) in THF (1.25 mL). The reaction was stirred at 50 °C for 20 hours before cooling and the solvents removed. Analysis of crude ^{1}H NMR using internal standard 1,2-dimethoxyethane showed 1:1.3 *meta*:*para* borylation in 100% yield. As the *para* isomer could be characterised from the reaction with 1a the product was not isolated.

2,2,2-trifluoro-N-(5-(4,4,5,5-tetramethyl-1,3,2-dioxaborolan-2-yl)-2 (trifluoromethyl)phenethyl)acetamide (5a)

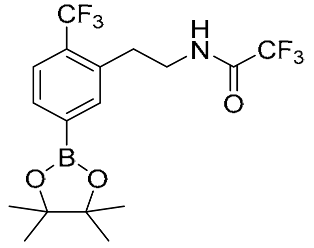

Meta

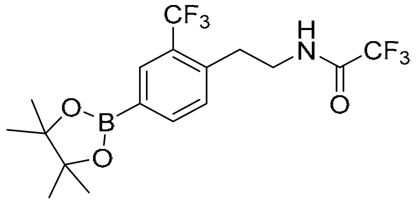

Para

1a in THF:

Following general procedure F using **4a** (71.3 mg, 0.25 mmol), B_2pin_2 (127 mg, 0.50 mmol), $[Ir(COD)OMe]_2$ (2.5 mg, 0.00375 mmol) and **1a** (3.8 mg, 0.0075 mmol) in THF (1.25 mL). Stirred in vial at 50 °C for 20 hours. Analysis of crude ^{1}H NMR using internal standard 1,2-dimethoxyethane showed 18.4:1 *meta*:*para* borylation in 83% yield. The crude product was purified by silica gel chromatography (10% EtOAc in Petroleum Ether 40-60 °C) to give the title compound (as a 19.1:1 mixture of *meta*:*para* ratio, as determined by ^{1}H NMR) as a white solid (94 mg). A small amount of B_2Pin_2 impurity was found to be inseparable from the final product on silica. Corrected yield - 89 mg, 0.22 mmol, 86%.

Meta ^{1}H NMR (600 MHz, $CDCl_3$) δ 7.77 (d, *J* = 7.1 Hz, 1H), 7.77 (s, 1H), 7.64 (d, *J* = 8.1 Hz, 1H), 6.68 (br, 1H), 3.63 (dt, *J* = 6.8, 6.5 Hz, 2H), 3.08 (t, *J* = 7.2 Hz, 2H), 1.35 (s, 12H); **^{13}C NMR** (151 MHz, $CDCl_3$) δ 157.3 (q, *J* = 37.1 Hz), 137.8, 135.2, 133.3, 131.0 (q, *J* = 29.7 Hz), 125.4 (q, *J* = 5.6 Hz), 124.4 (q, *J* = 273.8 Hz), 115.8 (q, *J* = 287.8 Hz), 84.4, 40.9, 31.6, 24.8. **HRMS** m/z: $[M + H]^+$ calc'd for $[C_{17}H_{21}BF_6NO_3]^+$ expect 412.1522; found 412.1526.

Dtbpy in THF:

Following general procedure F using **4a** (71.3 mg, 0.25 mmol), B_2pin_2 (127 mg, 0.50 mmol), $[Ir(COD)OMe]_2$ (2.5 mg, 0.00375 mmol) and dtbpy (2.0 mg, 0.0075 mmol) in THF (1.25 mL). Stirred in vial at 50 °C for 20 hours. Analysis of crude ^{1}H NMR using internal standard 1,2-dimethoxyethane showed 1.4:1 *meta*:*para* borylation in 83% yield. The crude product was purified by silica gel chromatography (10% EtOAc in Petroleum Ether 40-60 °C) gave the title compound (as a 1.3:1 mixture of *meta*:*para* ratio, as determined by ^{1}H NMR) as a colourless oil (99 mg, 0.24 mmol, 96%). By subtracting the resonances for the *meta* isomer, the NMR data of the *para* isomer can be assigned as follows:

Para ^{1}H NMR (600 MHz, $CDCl_3$) δ 8.09 (s, 1H), 7.94 (d, *J* = 7.6 Hz, 1H), 7.35 (d, *J* = 7.6 Hz, 1H), 6.59 (br, 1H), 3.64 (dt, *J* = 6.8, 6.8 Hz, 2H), 3.09 (t, *J* = 7.3 Hz, 2H), 1.35 (s, 12H); **^{13}C NMR** (151 MHz, $CDCl_3$) δ 157.3 (q, *J* = 37.1 Hz), 139.1, 138.4, 132.5 (q, *J* = 5.5 Hz), 130.8, 128.3 (q, *J* = 29.5 Hz), 124.5 (q, *J* = 273.7 Hz), 115.7 (q, *J* = 287.7 Hz), 84.3, 40.7, 31.9, 24.8 (d, *J* = 1.9 Hz).

N-(5-(4,4,5,5-tetramethyl-1,3,2-dioxaborolan-2-yl)-2-(trifluoromethyl)phenethyl)methanesulfonamide (5b)

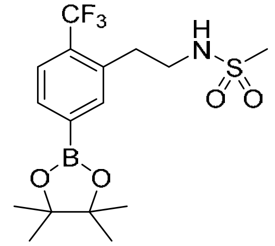

Meta

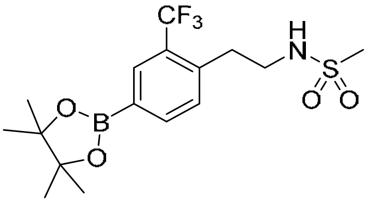

Para

1a in THF:

Following general procedure F using **4b** (66.8 mg, 0.25 mmol), B_2pin_2 (127 mg, 0.50 mmol), $[Ir(COD)OMe]_2$ (2.5 mg, 0.00375 mmol) and **1a** (3.8 mg, 0.0075 mmol) in THF (1.25 mL). Stirred in vial at 50 °C for 20 hours. Analysis of crude ^{1}H NMR using internal standard 1,2-dimethoxyethane showed 22.6:1 *meta*:*para* borylation in 94% yield (4% starting material remaining). The crude product was purified by silica gel chromatography (40% EtOAc in Petroleum Ether 40-60 °C) This gave the title compound (as a 27.1:1:1 mixture of *meta*:*para:*st.mat. ratio, as determined by ^{1}H NMR) as a colourless oil (93 mg). A corrected yield was calculated to account for the 3% starting material in the mixture. Corrected yield – 91 mg, 0.23 mmol, 93%.

Meta ^{1}H NMR (600 MHz, $CDCl_3$) δ 7.78 (s, 1H), 7.77 (d, *J* = 10.2 Hz, 1H), 7.64 (d, *J* = 7.7 Hz, 1H), 4.69 (br t, *J* = 6.2 Hz, 1H), 3.41 (dt, *J* = 6.8, 6.5 Hz, 2H), 3.08 (t, *J* = 7.6 Hz, 2H), 2.90 (s, 3H), 1.35 (s, 12H); **^{13}C NMR** (151 MHz, $CDCl_3$) δ 137.7, 135.4, 133.3, 131.1 (q, *J* = 29.7 Hz), 125.5 (q, *J* = 5.5 Hz), 124.4 (q, *J* = 274.0 Hz), 84.4, 44.2, 40.5, 33.5, 24.8. **HRMS** m/z: $[M + H]^+$ calc'd for $[C_{16}H_{24}BF_3NO_4S]^+$ expect 394.1474; found 394.1476.

Dtbpy in THF:

Following general procedure F using **4b** (66.8 mg, 0.25 mmol), B_2pin_2 (127 mg, 0.50 mmol), $[Ir(COD)OMe]_2$ (2.5 mg, 0.00375 mmol) and dtbpy (2.0 mg, 0.0075 mmol) in THF (1.25 mL). Stirred in vial at 50 °C for 20 hours. Analysis of crude ^{1}H NMR using internal standard 1,2-dimethoxyethane showed 1.3:1 *meta*:*para* borylation in 97% yield. The crude product was purified by silica gel chromatography (40% EtOAc in Petroleum Ether 40-60 °C) gave the title compound (as a 1.2:1 mixture of *meta*:*para* ratio, as determined by ^{1}H NMR) as a colourless oil (90 mg, 0.23 mmol, 92%). By subtracting the resonances for the *meta* isomer, the NMR data of the *para* isomer can be assigned as follows:

Para ^{1}H NMR (600 MHz, $CDCl_3$) δ 8.08 (s, 1H), 7.93 (d, *J* = 7.6 Hz, 1H), 7.40 (d, *J* = 7.6 Hz, 1H), 4.88 – 4.60 (br, 2H), 3.52 – 3.26 (m, 1H), 3.24 – 2.97 (m, 2H), 2.88 (s, 3H), 1.35 (s, 12H); **^{13}C NMR** (151 MHz, $CDCl_3$) δ 139.3, 138.3, 132.5 (q, *J* = 5.4 Hz), 131.1, 128.3 (q, *J* = 29.6 Hz), 124.5 (q, *J* = 273.9 Hz), 84.3, 44.0 (s), 40.5, 33.8, 24.8.

tert-butyl (5-(4,4,5,5-tetramethyl-1,3,2-dioxaborolan-2-yl)-2-(trifluoromethyl)phenethyl)carbamate (5c)

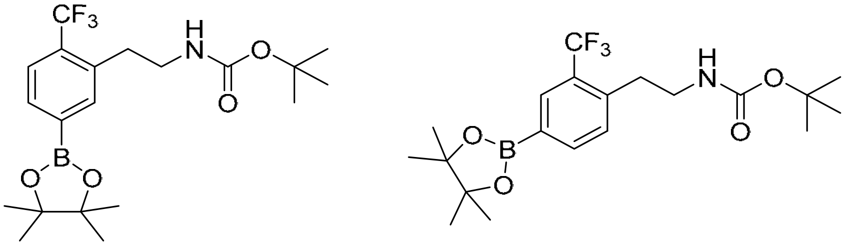

Meta ***Para***

1a in THF:

Following general procedure F using **4c** (72.3 mg, 0.25 mmol), B_2pin_2 (127 mg, 0.50 mmol), $[Ir(COD)OMe]_2$ (2.5 mg, 0.00375 mmol) and **1a** (3.8 mg, 0.0075 mmol) in THF (1.25 mL). Stirred in vial at 50 °C for 20 hours. Analysis of crude ^{1}H NMR using internal standard 1,2-dimethoxyethane showed 8.3:1 *meta*:*para* borylation in 86% yield. The crude product was purified by silica gel chromatography (10% EtOAc in Petroleum Ether 40-60 °C) gave the title compound (as a 8.8:1 mixture of *meta*:*para* ratio, as determined by ^{1}H NMR) as a white solid (91 mg, 0.22 mmol, 87%).

Meta ^{1}H NMR (600 MHz, $CDCl_3$) δ 7.78 (s, 1H), 7.73 (d, *J* = 7.8 Hz, 1H), 7.61 (d, *J* = 7.8 Hz, 1H), 4.68 (br, 1H), 3.38 (br, 2H), 2.99 (br t, *J* = 7.0 Hz, 2H), 1.44 (s, 9H), 1.34 (s, 12H); **^{13}C NMR** (151 MHz, $CDCl_3$) δ 155.8, 138.0, 136.5, 132.7, 131.1 (q, *J* = 29.8 Hz), 125.2 (q, *J* = 5.5 Hz), 124.5 (q, *J* = 274.0 Hz), 84.2, 79.2, 41.7, 32.9, 28.4, 24.8. **HRMS** m/z: $[M + H]^+$ calc'd for $[C_{20}H_{30}BF_3NO_4]^+$ expect 416.2224; found 416.2229.

Dtbpy in THF:

Following general procedure F using **4c** (72.3 mg, 0.25 mmol), B_2pin_2 (127 mg, 0.50 mmol), $[Ir(COD)OMe]_2$ (2.5 mg, 0.00375 mmol) and dtbpy (2.0 mg, 0.0075 mmol) in THF (1.25 mL). Stirred in vial at 50 °C for 20 hours. Analysis of crude ^{1}H NMR using internal standard 1,2-dimethoxyethane showed 1.4:1 *meta*:*para* borylation in 99% yield. The crude product was purified by silica gel chromatography (10% EtOAc in Petroleum Ether 40-60 °C) gave the title compound (as a 1.3:1 mixture of *meta*:*para* ratio, as determined by ^{1}H NMR) as a white solid (94 mg, 0.23 mmol, 90%). By subtracting the resonances for the *meta* isomer, the NMR data of the *para* isomer can be assigned as follows:

Para ^{1}H NMR (600 MHz, $CDCl_3$) δ 8.07 (s, 1H), 7.90 (d, *J* = 7.6 Hz, 1H), 7.37 (d, *J* = 7.4 Hz, 1H), 4.66 (br, 2H), 3.47 – 3.30 (m, 2H), 3.03 – 2.96 (m, 2H), 1.44 (s, 9H), 1.34 (s, 12H); **^{13}C NMR** (151 MHz, $CDCl_3$) δ 155.8, 140.6, 138.1, 132.3 (q, *J* = 5.2 Hz), 131.0, 128.3 (q, *J* = 29.6 Hz), 124.6 (q, *J* = 273.8 Hz), 84.2, 79.3, 41.5, 33.2, 28.4, 24.8.

2,2,2-trifluoro-N-(2-iodo-5-(4,4,5,5-tetramethyl-1,3,2-dioxaborolan-2-yl)phenethyl)acetamide (5d)

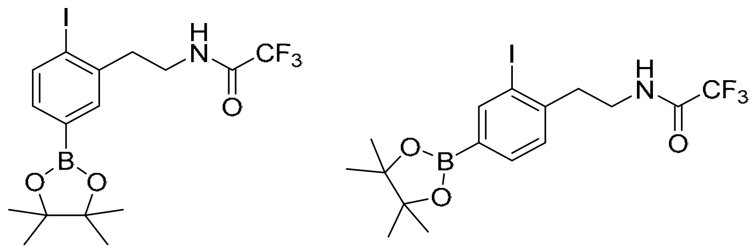

Meta ***Para***

1a in THF:

Following general procedure F using **4d** (86 mg, 0.25 mmol), B_2pin_2 (127 mg, 0.50 mmol), $[Ir(COD)OMe]_2$ (2.5 mg, 0.00375 mmol) and **1a** (3.8 mg, 0.0075 mmol) in THF (1.25 mL). Stirred in vial at 50 °C for 20 hours. Analysis of crude ^{1}H NMR using internal standard 1,2-dimethoxyethane showed 5.7:1 *meta*:*para* borylation in 85% yield (12% starting material remaining). The crude product was purified by silica gel chromatography (15% EtOAc in Petroleum Ether 40-60 ^{o}C) gave the title compound (as a 5.4:1 mixture of *meta*:*para* ratio, as determined by ^{1}H NMR) as a yellowish oil (79 mg, 0.17 mmol, 68%).

Meta ^{1}H NMR (500 MHz, $CDCl_3$) δ 7.85 (d, *J* = 7.8 Hz, 1H), 7.61 (d, *J* = 1.5 Hz, 1H), 7.33 (dd, *J* = 7.8, 1.6 Hz, 1H), 6.53 (br, 1H), 3.61 (dt, *J* = 6.8, 6.5 Hz, 2H), 3.04 (t, *J* = 7.1 Hz, 2H), 1.33 (s, 12H); **^{13}C NMR** (126 MHz, $CDCl_3$) δ 157.3 (q, *J* = 37.0 Hz), 139.8, 139.4, 136.2, 134.7, 115.8 (q, *J* = 287.9 Hz), 104.7, 84.2, 39.9, 39.2, 24.8. **HRMS** m/z: $[M + H]^+$ calc'd for $[C_{16}H_{21}BNO_3F_3I]^+$ expect 470.0611; found 470.0619.

Dtbpy in THF:

Following general procedure F using **4d** (86 mg, 0.25 mmol), B_2pin_2 (127 mg, 0.50 mmol), $[Ir(COD)OMe]_2$ (2.5 mg, 0.00375 mmol) and dtbpy (2.0 mg, 0.0075 mmol) in THF (1.25 mL). Stirred in vial at 50 °C for 20 hours. Analysis of crude ^{1}H NMR using internal standard 1,2-dimethoxyethane showed 1:1.8 *meta*:*para* borylation in 99% yield. The crude product was purified by silica gel chromatography (15% EtOAc in Petroleum Ether 40-60 ^{o}C) gave the title compound (as a 1:1.9 mixture of *meta*:*para* ratio, as determined by ^{1}H NMR) as a yellowish oil (119 mg). A small amount of B_2Pin_2 impurity was found to be inseparable from the final product on silica. Corrected yield - 113 mg, 0.24 mmol, 97%. By subtracting the resonances for the *meta* isomer, the NMR data of the *para* isomer can be assigned as follows:

Para ^{1}H NMR (500 MHz, $CDCl_3$) δ 8.26 (d, *J* = 1.0 Hz, 1H), 7.70 (dd, *J* = 7.5, 1.1 Hz, 1H), 7.19 (d, *J* = 7.5 Hz, 1H), 6.65 (br, 1H), 3.60 (dt, *J* = 6.8, 6.5 Hz, 2H), 3.03 (t, *J* = 7.1 Hz, 2H), 1.32 (s, 12H); **^{13}C NMR** (126 MHz, $CDCl_3$) δ 157.4 (q, *J* = 37.0 Hz), 145.9, 143.3, 134.9, 129.6, 115.8 (q, *J* = 287.8 Hz), 100.6, 84.2, 39.7, 39.6, 24.8.

2,2,2-trifluoro-N-(3-fluoro-5-(4,4,5,5-tetramethyl-1,3,2-dioxaborolan-2-yl)phenethyl)acetamide (5e)

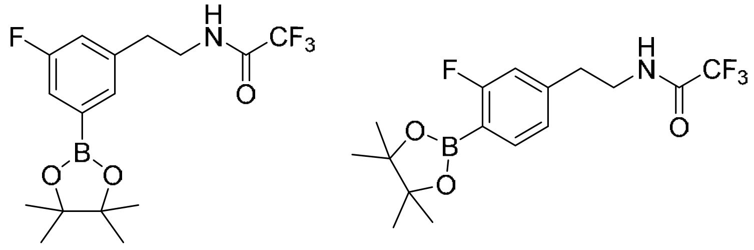

Meta ***Para***

1a in THF:

Following general procedure F using **4e** (58.8 mg, 0.25 mmol), B_2pin_2 (127 mg, 0.50 mmol), $[Ir(COD)OMe]_2$ (2.5 mg, 0.00375 mmol) and **1a** (3.8 mg, 0.0075 mmol) in THF (1.25 mL). Stirred in vial at 50 °C for 20 hours. Analysis of crude ^{1}H NMR using internal standard 1,2-dimethoxyethane showed 12.9:1 *meta*:*para* borylation in 98% yield (these numbers include some *meta* isomer that has also undergone NH borylation). The crude product was purified by silica gel chromatography (15% EtOAc in Petroleum Ether 40-60 °C) which resulted in the cleavage of the N-B bond in the NH-borylation material and gave the title compound (as a 13:1 mixture of *meta*:*para* ratio, as determined by ^{1}H NMR) as a white solid (80 mg, 0.22 mmol, 89%).

Meta ^{1}H NMR (600 MHz, $CDCl_3$) δ 7.41 (s, 1H), 7.36 (dd, *J* = 8.7, 2.3 Hz, 1H), 6.97 (d, *J* = 9.3 Hz, 1H), 6.68 (br, 1H), 3.60 (dt, *J* = 6.9, 6.5 Hz, 2H), 2.88 (t, *J* = 7.2 Hz, 2H), 1.34 (s, 12H); **^{13}C NMR** (151 MHz, $CDCl_3$) δ 162.7 (d, *J* = 247.6 Hz), 157.3 (q, *J* = 37.0 Hz), 139.5 (d, *J* = 6.6 Hz), 130.6 (d, *J* = 2.5 Hz), 119.5 (d, *J* = 19.3 Hz), 118.4 (d, *J* = 21.3 Hz), 115.7 (q, *J* = 287.7 Hz), 84.2, 40.8, 34.5, 24.8. **HRMS** m/z: $[M + H]^+$ calc'd for $[C_{16}H_{20}BF_4NO_3]^+$ expect 362.1554; found 362.1552.

Dtbpy in THF:

Following general procedure F using **4e** (58.8 mg, 0.25 mmol), B_2pin_2 (127 mg, 0.50 mmol), $[Ir(COD)OMe]_2$ (2.5 mg, 0.00375 mmol) and dtbpy (2.0 mg, 0.0075 mmol) in THF (1.25 mL). Stirred in vial at 50 °C for 20 hours. Analysis of crude ^{1}H NMR using internal standard 1,2-dimethoxyethane showed 1.4:1 *meta*:*para* borylation in 89% yield (these numbers include the *meta/para* products which have also undergone NH borylation). The crude product was purified by silica gel chromatography (15% EtOAc in Petroleum Ether 40-60 °C) which resulted in the cleavage of the N-B bond and reprotonation of the NH-borylation products and gave the title compound (as a 1.7:1 mixture of *meta*:*para* ratio, as determined by ^{1}H NMR) as a colourless oil (84 mg, 0.23 mmol, 93%). By subtracting the resonances for the *meta* isomer, the NMR data of the *para* isomer can be assigned as follows:

Para ^{1}H NMR (600 MHz, $CDCl_3$) δ 7.72 – 7.64 (m, 1H), 6.97 (d, *J* = 8.6 Hz, 1H), 6.87 (m, 1H), 6.69 (br, 1H), 3.68 – 3.49 (m, 2H), 2.88 (m, 2H), 1.35 (s, 12H); **^{13}C NMR** (151 MHz, $CDCl_3$) δ 167.4 (d, *J* = 251.7 Hz), 157.3 (q, *J* = 37.1 Hz), 143.8 (d, *J* = 8.2 Hz), 137.3 (d, *J* = 8.6 Hz), 130.6 (d, *J* = 2.6 Hz), 124.1 (d, *J* = 2.8 Hz), 115.5 (d, *J* = 24.1 Hz), 84.0, 40.6, 34.7, 24.8.

N-(2-bromo-5-(4,4,5,5-tetramethyl-1,3,2-dioxaborolan-2-yl)phenethyl)-2,2,2-trifluoroacetamide (5f)

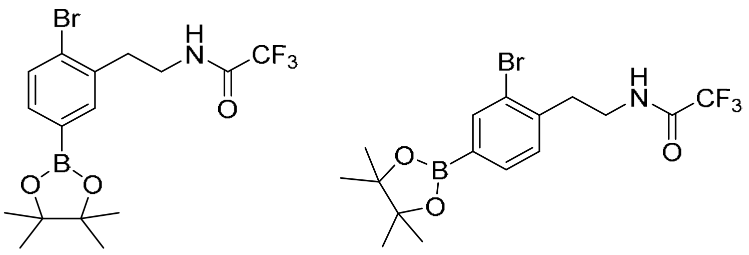

Meta ***Para***

1a in THF:

Following general procedure F using **4f** (74 mg, 0.25 mmol), B_2pin_2 (127 mg, 0.50 mmol), $[Ir(COD)OMe]_2$ (2.5 mg, 0.00375 mmol) and **1a** (3.8 mg, 0.0075 mmol) in THF (1.25 mL). Stirred in vial at 50 °C for 20 hours. Analysis of crude ^{1}H NMR using internal standard 1,2-dimethoxyethane showed 8.0:1 *meta*:*para* borylation in 93% yield. The crude product was purified by silica gel chromatography (15% EtOAc in Petroleum Ether 40-60 °C) gave the title compound (as a 7.2:1 mixture of *meta*:*para* ratio, as determined by ^{1}H NMR) as a white solid (103 mg). A small amount of B_2Pin_2 impurity was found to be inseparable from the final product on silica. Corrected yield - 96 mg, 0.23 mmol, 91%.

Meta ^{1}H NMR (600 MHz, $CDCl_3$) δ 7.64 (d, *J* = 1.4 Hz, 1H), 7.56 (d, *J* = 7.9 Hz, 1H), 7.52 (dd, *J* = 7.9, 1.5 Hz, 1H), 6.60 (br, 1H), 3.63 (dt, *J* = 6.8, 6.5 Hz, 2H), 3.05 (t, *J* = 7.1 Hz, 2H), 1.33 (s, 12H); **^{13}C NMR** (151 MHz, $CDCl_3$) δ 157.3 (q, *J* = 37.0 Hz), 137.2, 136.4, 134.8, 132.6, 128.0, 115.8 (q, *J* = 287.9 Hz), 84.1, 39.7, 34.8, 24.8. **HRMS** m/z: $[M + H]^+$ calc'd for $[C_{16}H_{21}BNO_3F_3Br]^+$ expect 422.0750; found 422.0744.

Dtbpy in THF:

Following general procedure F using **4f** (74 mg, 0.25 mmol), B_2pin_2 (127 mg, 0.50 mmol), $[Ir(COD)OMe]_2$ (2.5 mg, 0.00375 mmol) and dtbpy (2.0 mg, 0.0075 mmol) in THF (1.25 mL). Stirred in vial at 50 °C for 20 hours. Analysis of crude ^{1}H NMR using internal standard 1,2-dimethoxyethane showed 1:1.7 *meta*:*para* borylation in 92% yield. The crude product was purified by silica gel chromatography (15% EtOAc in Petroleum Ether 40-60 °C) gave the title compound (as a 1:1.8 mixture of *meta*:*para* ratio, as determined by ^{1}H NMR) as a colourless oil (103 mg). A small amount of B_2Pin_2 impurity was found to be inseparable from the final product on silica. Corrected yield - 100 mg, 0.23 mmol, 94%. By subtracting the resonances for the *meta* isomer, the NMR data of the *para* isomer can be assigned as follows:

Para ^{1}H NMR (600 MHz, $CDCl_3$) δ 7.99 (d, *J* = 0.7 Hz, 1H), 7.68 (dd, *J* = 7.5, 0.9 Hz, 1H), 7.20 (d, *J* = 7.5 Hz, 1H), 6.63 (br, 1H), 3.63 (q, *J* = 6.7 Hz, 2H), 3.05 (t, *J* = 7.0 Hz, 2H), 1.33 (s, 12H); ^{13}C NMR (151 MHz, $CDCl_3$) δ 157.3 (q, *J* = 37.0 Hz), 140.0, 139.2, 134.0, 130.5, 124.4, 115.7 (q, *J* = 287.8 Hz), 84.2, 39.5, 35.2, 24.8.

Methyl 4-(4,4,5,5-tetramethyl-1,3,2-dioxaborolan-2-yl)-2-(2-(2,2,2-trifluoroacetamido)ethyl)benzoate (5g)

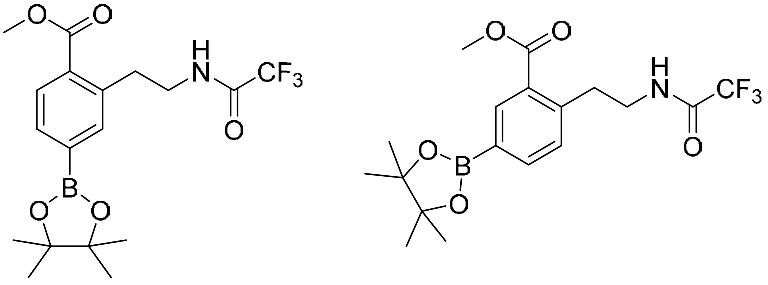

Meta ***Para***

1a in THF:

Following general procedure F using **4g** (68.8 mg, 0.25 mmol), B_2pin_2 (127 mg, 0.50 mmol), $[Ir(COD)OMe]_2$ (2.5 mg, 0.00375 mmol) and **1a** (3.8 mg, 0.0075 mmol) in THF (1.25 mL). Stirred in vial at 50 °C for 20 hours. Analysis of crude ^{1}H NMR using internal standard 1,2-dimethoxyethane showed 23:1 *meta*:*para* borylation in 97% yield (these numbers include a small amount of meta isomer that also underwent NH borylation). The crude product was purified by silica gel chromatography (15% EtOAc in Petroleum Ether 40-60 °C) which resulted in the cleavage of the N-B bond in the small amount of *N*-borylated product and gave the title compound (as a 17.8:1 mixture of *meta*:*para* ratio, as determined by ^{1}H NMR) as an off-white solid (97 mg, 0.24 mmol, 97%).

Meta ^{1}H NMR (600 MHz, $CDCl_3$) δ 8.01 (br, 1H), 7.84 (d, *J* = 8.1 Hz, 1H), 7.73 (m, 2H), 3.93 (s, 3H), 3.67 (dt, *J* = 6.3, 5.6 Hz, 2H), 3.27 – 3.10 (t, *J* = 6.4 Hz, 2H), 1.35 (s, 12H) ; ^{13}**C NMR** (151 MHz, $CDCl_3$) δ 169.2, 157.4 (q, *J* = 36.7 Hz), 138.8, 137.8, 133.1, 132.0, 129.6, 115.8 (q, *J* = 287.7 Hz), 84.3, 52.7, 42.2, 31.6, 24.9. **HRMS** m/z: $[M + H]^+$ calc'd for $[C_{18}H_{24}BF_3NO_5]^+$ expect 402.1703; found 402.1702.

Dtbpy in THF:

Following general procedure F using **4g** (68.8 mg, 0.25 mmol), B_2pin_2 (127 mg, 0.50 mmol), $[Ir(COD)OMe]_2$ (2.5 mg, 0.00375 mmol) and dtbpy (2.0 mg, 0.0075 mmol) in THF (1.25 mL). Stirred in vial at 50 °C for 20 hours. Analysis of crude ^{1}H NMR using internal standard 1,2-dimethoxyethane showed 2.4:1 *meta*:*para* borylation in 82% yield (these numbers include samll amounts of the *meta/para* products which had also undergone NH borylation). The crude product was purified by silica gel chromatography (15% EtOAc in Petroleum Ether 40-60 °C) which resulted in the cleavage of the N-B bond in the NH-borylation products and gave the title compound (as a 2.5:1 mixture of *meta*:*para* ratio, as determined by ^{1}H NMR) as an off-white solid (61 mg, 0.15 mmol, 61%). By subtracting the resonances for the *meta* isomer, the NMR data of the *para* isomer can be assigned as follows:

Para ^{1}H NMR (600 MHz, $CDCl_3$) δ 8.29 (s, 1H), 8.01 (br, 1H), 7.91 (dd, *J* = 7.6, 1.1 Hz, 1H), 7.31 (d, *J* = 7.6 Hz, 1H), 3.93 (s, 3H), 3.69 – 3.63 (m, 2H), 3.18 (m, 2H), 1.35 (s, 12H); ^{13}**C NMR** (151 MHz, $CDCl_3$) δ 169.3, 157.4 (q, *J* = 36.8 Hz), 142.9, 138.8, 136.9, 130.9, 129.4, 115.8 (q, *J* = 287.7 Hz), 84.2, 52.6, 42.0, 32.0, 24.9.

N-(2-chloro-5-(4,4,5,5-tetramethyl-1,3,2-dioxaborolan-2-yl)phenethyl)-2,2,2-trifluoroacetamide (5h)

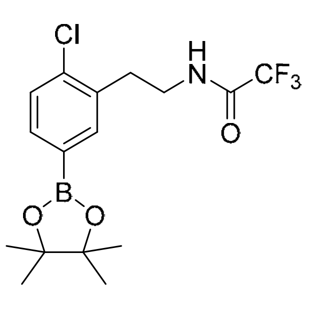

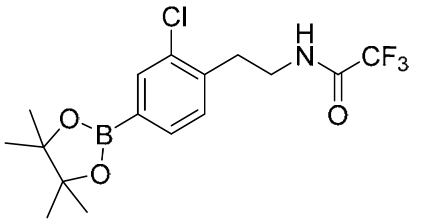

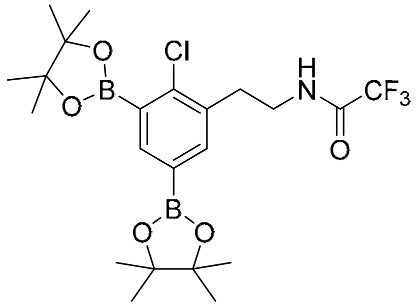

Meta ***Para*** ***Dimeta***

1a in THF:

Following general procedure F using **4h** (62.9 mg, 0.25 mmol), B_2pin_2 (127 mg, 0.50 mmol), $[Ir(COD)OMe]_2$ (2.5 mg, 0.00375 mmol) and **1a** (3.8 mg, 0.0075 mmol) in THF (1.25 mL). Stirred in vial at 50 °C for 20 hours. Analysis of crude ^{1}H NMR using internal standard 1,2-dimethoxyethane showed 7.7:0.43:1 *meta:dimeta*:*para* borylation (overall *meta*:*para* borylation 8.1:1) in 95% yield (these numbers include a small amount of the *meta* isomer that had also undergone NH borylation). The crude product was purified by silica gel chromatography (15% EtOAc in Petroleum Ether 40-60 °C) which resulted in the cleavage of the N-B bond in the small amount of the NH-borylation products and gave the title compound (as a 6.9:0.4:1 mixture of *meta:dimeta*:*para* ratio and overall *meta*:*para* ratio of 7.3:1, as determined by ^{1}H NMR) as a colourless oil (96 mg). A small amount of B_2Pin_2 impurity was found to be inseparable from the final product on silica. Corrected yield - 88 mg, 0.24 mmol, 94%).

Meta ^{1}H NMR (600 MHz, $CDCl_3$) δ 7.65 (d, *J* = 1.2 Hz, 1H), 7.61 (dd, *J* = 7.9, 1.4 Hz, 1H), 7.37 (d, *J* = 7.9 Hz, 1H), 6.70 (br, 1H), 3.62 (dt, *J* = 6.8, 6.5 Hz, 2H), 3.03 (t, *J* = 7.1 Hz, 2H), 1.33 (s, 12H); **^{13}C NMR** (151 MHz, $CDCl_3$) δ 157.3 (q, *J* = 37.0 Hz), 137.4, 137.3, 134.7, 134.7, 129.2, 115.8 (q, *J* = 287.8 Hz), 84.1, 39.7, 32.4, 24.8. **HRMS** m/z: $[M + H]^+$ calc'd for $[C_{16}H_{20}BClF_3NO_3]^+$ expect 378.1258; found 378.1253.

Dtbpy in THF:

Following general procedure F using **4h** (62.9 mg, 0.25 mmol), B_2pin_2 (127 mg, 0.50 mmol), $[Ir(COD)OMe]_2$ (2.5 mg, 0.00375 mmol) and dtbpy (2.0 mg, 0.0075 mmol) in THF (1.25 mL). Stirred in vial at 50 °C for 20 hours. Analysis of crude ^{1}H NMR using internal standard 1,2-dimethoxyethane showed 1:0.15:1.8 *meta:dimeta*:*para* borylation in 93% yield (these numbers include the *meta/para* products which have also undergone NH borylation). The crude product was purified by silica gel chromatography (15% EtOAc in Petroleum Ether 40-60 °C) which resulted in the cleavage of the N-B bond and reprotonation of the NH-borylation products and gave the title compound (as a 1:0.14:1.9 mixture of *meta:dimeta*:*para* ratio, as determined by ^{1}H NMR) as a colourless oil (100 mg). A small amount of B_2Pin_2 impurity was found to be inseparable from the final product on silica. Corrected yield - 92 mg, 0.23 mmol, 92%. By subtracting the resonances for the *meta* isomer, the NMR data of the *para* isomer can be assigned as follows:

Para ^{1}H NMR (600 MHz, $CDCl_3$) δ 7.80 (s, 1H), 7.63 (dd, *J* = 7.6, 0.6 Hz, 1H), 7.21 (d, *J* = 7.5 Hz, 1H), 6.70 (br, 1H), 3.66 – 3.57 (m, 2H), 3.04 (m, 2H), 1.33 (s, 12H); **^{13}C NMR** (151 MHz, $CDCl_3$) δ 157.4 (q, *J* = 37.0 Hz), 138.4, 135.8, 133.9, 133.4, 130.5, 115.7 (q, *J* = 287.8 Hz), 84.2, 39.5, 32.8, 24.8.

2,2,2-trifluoro-N-(2-methyl-5-(4,4,5,5-tetramethyl-1,3,2-dioxaborolan-2-yl)phenethyl)acetamide (5i)

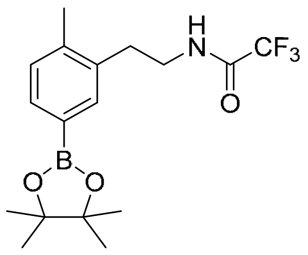

Meta

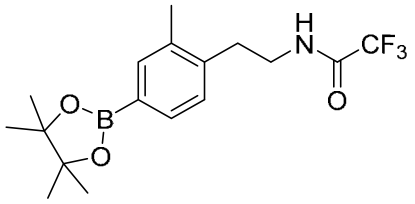

Para

1a in THF:

Following general procedure F using **4i** (58 mg, 0.25 mmol), B_2pin_2 (127 mg, 0.50 mmol), $[Ir(COD)OMe]_2$ (2.5 mg, 0.00375 mmol) and **1a** (3.8 mg, 0.0075 mmol) in THF (1.25 mL). Stirred in vial at 70 °C for 20 hours. Analysis of crude ^{1}H NMR using internal standard 1,2-dimethoxyethane showed ~7:1 *meta*:*para* borylation in 69% yield (30% starting material remaining). The crude product was purified by silica gel chromatography (20% EtOAc in Petroleum Ether 40-60 °C), however this gave a mixture of the borylated products and starting material, which were inseparable on silica (8.2:1:2.3 mixture of *meta*:*para*:starting material ratio, as determined by ^{1}H NMR), as a colourless oil (60 mg). As the mixture contained 20% of starting material, the isolated yield was corrected by calculating the mass of the starting material in the mixture. Corrected yield for the borylated products was 52 mg, 0.15 mmol, 58%.

Meta ^{1}H NMR (500 MHz, $CDCl_3$) δ 7.61 (dd, *J* = 7.5, 1.1 Hz, 1H), 7.57 (s, 1H), 7.20 (d, *J* = 7.5 Hz, 1H), 6.55 (br, 1H), 3.58 (dt, *J* = 6.8, 6.5 Hz, 2H), 2.91 (t, *J* = 7.3 Hz, 2H), 2.36 (s, 3H), 1.34 (s, 12H); **^{13}C NMR** (126 MHz, $CDCl_3$) δ 157.3 (q, *J* = 36.9 Hz), 139.8, 135.8, 135.1, 133.5, 130.2, 115.8 (q, *J* = 287.8 Hz), 83.8, 40.0, 32.1, 24.8, 19.4. **HRMS** m/z: $[M + H]^+$ calc'd for $[C_{17}H_{24}BNO_3F_3]^+$ expect 358.1801; found 358.1803.

Dtbpy in THF:

Following general procedure F using **4i** (58 mg, 0.25 mmol), B_2pin_2 (127 mg, 0.50 mmol), $[Ir(COD)OMe]_2$ (2.5 mg, 0.00375 mmol) and dtbpy (2.0 mg, 0.0075 mmol) in THF (1.25 mL). Stirred in vial at 70 °C for 20 hours. Analysis of crude ^{1}H NMR using internal standard 1,2-dimethoxyethane showed 1.2:1 *meta*:*para* borylation in 99% yield. The crude product was purified by silica gel chromatography (20% EtOAc in Petroleum Ether 40-60 °C) gave the title compound (as a 1.2:1 mixture of *meta*:*para* ratio, as determined by ^{1}H NMR) as a colourless oil (85 mg, 0.24 mmol, 96%). By subtracting the resonances for the *meta* isomer, the NMR data of the *para* isomer can be assigned as follows:

Para ^{1}H NMR (600 MHz, $CDCl_3$) δ 7.65 (s, 1H), 7.13 (d, *J* = 7.5 Hz, 1H), 6.63 (s, 1H), 3.73 – 3.44 (m, 2H), 2.97 – 2.87 (m, 2H), 2.35 (s, 3H), 1.34 (s, 12H); **^{13}C NMR** (151 MHz, $CDCl_3$) δ 157.3 (q, *J* = 36.9 Hz), 139.1, 137.0, 135.6, 132.8, 128.7, 115.8 (q, *J* = 287.8 Hz), 83.8, 39.8, 32.5, 24.8, 18.9.

2,2,2-trifluoro-N-(2-methoxy-5-(4,4,5,5-tetramethyl-1,3,2-dioxaborolan-2-yl)phenethyl)acetamide (5j)

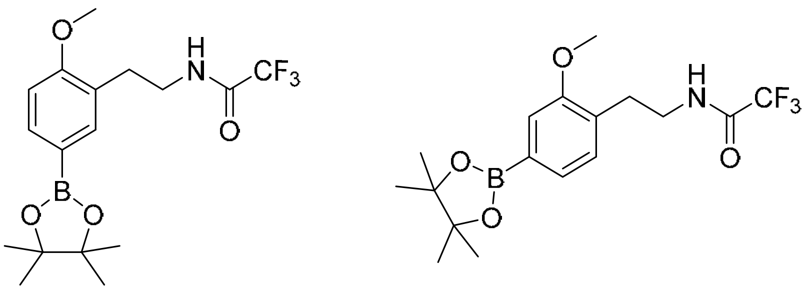

Meta ***Para***

1a in THF:

Following general procedure F using **4j** (61.8 mg, 0.25 mmol), B_2pin_2 (127 mg, 0.50 mmol), $[Ir(COD)OMe]_2$ (2.5 mg, 0.00375 mmol) and **1a** (3.8 mg, 0.0075 mmol) in THF (1.25 mL). Stirred in vial at 50 °C for 20 hours. Analysis of crude ^{1}H NMR using internal standard 1,2-dimethoxyethane showed 1.4:1 *meta*:*para* borylation in 96% yield. The crude product was purified by silica gel chromatography (10% EtOAc in Petroleum Ether 40-60 °C) gave the title compound (as a 1.6:1 mixture of *meta*:*para* ratio, as determined by ^{1}H NMR) as a colourless oil (82 mg, 0.22 mmol, 88%).

Meta ^{1}H NMR (600 MHz, $CDCl_3$) δ 7.73 (dd, J = 8.2, 1.5 Hz, 1H), 7.60 (d, J = 1.4 Hz, 1H), 7.10 (br, 1H), 6.90 (d, J = 8.2 Hz, 1H), 3.87 (s, 3H), 3.55 (dt, J = 6.4, 6.4 Hz, 2H), 2.93 (t, J = 6.4 Hz, 2H), 1.34 (s, 12H).; **^{13}C NMR** (151 MHz, $CDCl_3$) δ 159.7, 157.1 (q, J = 36.6 Hz), 137.5, 135.7, 125.9, 115.9 (q, J = 287.9 Hz), 109.9, 83.7, 41.2, 29.1, 24.8. **HRMS** m/z: $[M + H]^+$ calc'd for $[C_{17}H_{23}BF_3NO_4]^+$ expect 374.1754; found 374.1754.

Dtbpy in THF:

Following general procedure F using **4j** (61.8 mg, 0.25 mmol), B_2pin_2 (127 mg, 0.50 mmol), $[Ir(COD)OMe]_2$ (2.5 mg, 0.00375 mmol) and dtbpy (2.0 mg, 0.0075 mmol) in THF (1.25 mL). Stirred in vial at 50 °C for 20 hours. Analysis of crude ^{1}H NMR using internal standard 1,2-dimethoxyethane showed 1:2.4 *meta*:*para* borylation in 85% yield. The crude product was purified by silica gel chromatography (15% EtOAc in Petroleum Ether 40-60 °C) gave the title compound (as a 1:2.3 mixture of *meta*:*para* ratio, as determined by ^{1}H NMR) as a colourless oil (76 mg, 0.2 mmol, 82%). By subtracting the resonances for the *meta* isomer, the NMR data of the *para* isomer can be assigned as follows:

Para ^{1}H NMR (600 MHz, $CDCl_3$) δ 7.41 (d, J = 7.3 Hz, 1H), 7.31 (s, 1H), 7.18 (br, 1H), 7.16 (d, J = 7.3 Hz, 1H), 3.91 (s, 3H), 3.56 (dt, J = 6.4, 6.4 Hz, 2H), 2.93 (t, J = 6.4 Hz, 2H), 1.35 (s, 12H); **^{13}C NMR** (151 MHz, $CDCl_3$) δ 157.1 (q, J = 36.5 Hz), 156.6, 130.4, 130.0, 128.0, 115.9, 115.9 (q, J = 287.8 Hz), 84.0, 55.3, 41.2, 29.4, 24.8.

N-(2-(((tert-butyldimethylsilyl)oxy)methyl)-5-(4,4,5,5-tetramethyl-1,3,2-dioxaborolan-2-yl)phenethyl)-2,2,2-trifluoroacetamide (5k)

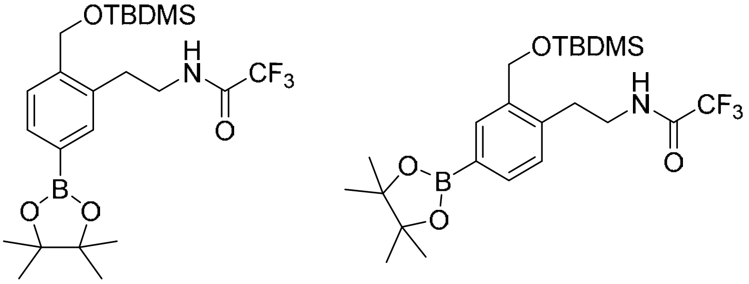

Meta ***Para***

1a in THF:

Following general procedure F using **4k** (90 mg, 0.25 mmol), B_2pin_2 (127 mg, 0.50 mmol), $[Ir(COD)OMe]_2$ (2.5 mg, 0.00375 mmol) and **1a** (3.8 mg, 0.0075 mmol) in THF (1.25 mL). Stirred in vial at 70 °C for 20 hours. Analysis of crude ^{1}H NMR using internal standard 1,2-dimethoxyethane showed ~14:1 *meta:para* borylation in 99% yield (these numbers include the *meta/para* products which have also undergone NH borylation at the higher temperature). The crude product was purified by silica gel chromatography (10% EtOAc in Petroleum Ether 40-60 °C) which resulted in the cleavage of the N-B bond of the NH-borylation products and gave the title compound (as a 15.2:1 mixture of *meta*:*para* ratio, as determined by ^{1}H NMR) as a yellowish oil (113 mg, 0.23 mmol, 93%).

Meta ^{1}H NMR (600 MHz, $CDCl_3$) δ 7.70 (dd, *J* = 7.5, 1.0 Hz, 1H), 7.61 (s, 1H), 7.39 (d, *J* = 7.6 Hz, 1H), 6.75 (br, 1H), 4.76 (s, 2H), 3.63 (dt, 6.9, 6.5 Hz, 2H), 2.96 (t, *J* = 7.2 Hz, 2H), 1.35 (s, 12H), 0.94 (s, 9H), 0.12 (s, 6H); **^{13}C NMR** (151 MHz, $CDCl_3$) δ 157.2 (q, *J* = 36.8 Hz), 141.9 (s), 135.9, 134.8, 133.5, 127.8, 115.8 (q, *J* = 287.8 Hz), 83.8, 63.8, 40.6, 30.9, 25.9, 24.8, 18.4, -5.3. **HRMS** m/z: $[M - H]^{\bullet+}$ calc'd for $[C_{23}H_{36}BNO_4F_3Si]^{\bullet+}$ expect 486.2459; found 486.2460.

Dtbpy in THF:

Following general procedure F using **4k** (90 mg, 0.25 mmol), B_2pin_2 (127 mg, 0.50 mmol), $[Ir(COD)OMe]_2$ (2.5 mg, 0.00375 mmol) and dtbpy (2.0 mg, 0.0075 mmol) in THF (1.25 mL). Stirred in vial at 70 °C for 20 hours. Analysis of crude ^{1}H NMR using internal standard 1,2-dimethoxyethane showed 1.7:1 *meta:para* borylation in 99% yield (these numbers include the *meta/para* products which have also undergone NH borylation). The crude product was purified by silica gel chromatography (10% EtOAc in Petroleum Ether 40-60 °C) which resulted in the cleavage of the N-B bond and reprotonation of the NH-borylation products and gave the title compound (as a 1.7:1 mixture of *meta*:*para* ratio, as determined by ^{1}H NMR) as a yellowish oil (119 mg, 0.24 mmol, 97%). By subtracting the resonances for the *meta* isomer, the NMR data of the *para* isomer can be assigned as follows:

Para ^{1}H NMR (600 MHz, $CDCl_3$) δ ^{1}H NMR (600 MHz, $CDCl_3$) δ 7.81 – 7.64 (m, 2H), 7.18 (d, *J* = 7.4 Hz, 1H), 6.87 (br, 1H), 4.75 (s, 2H), 3.64 (m, 2H), 3.02 (t, *J* = 7.0 Hz, 2H), 1.35 (s, 12H), 0.95 (s, 9H), 0.14 (s, 6H); **^{13}C NMR** (151 MHz, $CDCl_3$) δ 157.2 (q, *J* = 36.9 Hz), 139.9 (s), 137.7, 135.7, 134.8, 129.1, 115.7 (q, *J* = 287.8 Hz), 83.8, 64.6, 40.5, 31.0, 25.9, 24.8, 18.4, -5.3.

N-(3,5-bis(4,4,5,5-tetramethyl-1,3,2-dioxaborolan-2-yl)phenethyl)-2,2,2-trifluoroacetamide (5l)

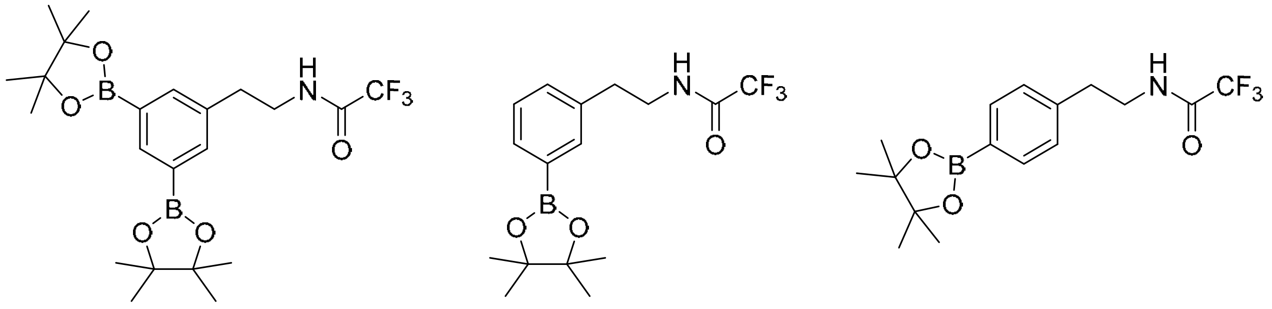

Dimeta ***Meta*** ***Para***

1a in THF:

Following general procedure F using **4l** (54.3 mg, 0.25 mmol), B_2pin_2 (127 mg, 0.50 mmol), $[Ir(COD)OMe]_2$ (2.5 mg, 0.00375 mmol) and **1a** (3.8 mg, 0.0075 mmol) in THF (1.25 mL). Stirred in vial at 50 °C for 20 hours. Analysis of crude ^1H NMR using internal standard 1,2-dimethoxyethane showed 9.2:1.3:1 *dimeta*:*meta*:*para* borylation in 99% yield (overall *meta*:*para* borylation 10.5:1). The crude product was purified by silica gel chromatography (20% EtOAc in Petroleum Ether 40-60 °C) gave the title compound (as a 17.8:2.2:1 mixture of *dimeta*:*meta*:*para* ratio and overall *meta*:*para* ratio of 20:1, as determined by ^1H NMR) as a white solid (109 mg, 0.24 mmol, 97%). The mass quoted above is the actual mass of the isolated mixture of products, whilst the amount of substance in mmol has been calculated by examining the ratios of the *dimeta* to monoborylated products and accounting for the extra mass due to the higher molecular weight of the *dimeta* product.

Dimeta ^1H NMR (600 MHz, $CDCl_3$) δ 8.17 (s, 1H), 7.73 (s, 2H), 6.49 (s, 1H), 3.61 (dt, *J* = 6.9, 6.5 Hz, 2H), 2.89 (t, *J* = 7.4 Hz, 2H), 1.33 (s, 24H); ^{13}**C NMR** (151 MHz, $CDCl_3$) δ 157.2 (q, *J* = 36.9 Hz), 139.8, 137.9, 136.1, 115.8 (q, *J* = 287.9 Hz), 83.9, 41.0, 34.6, 24.8. **HRMS** m/z: $[M + H]^+$ calc'd for $[C_{22}H_{33}B_2F_3NO_5]^+$ expect 470.2491; found 470.2495.

Dtbpy in THF:

Following general procedure F using **4l** (54.3 mg, 0.25 mmol), B_2pin_2 (127 mg, 0.50 mmol), $[Ir(COD)OMe]_2$ (2.5 mg, 0.00375 mmol) and dtbpy (2.0 mg, 0.0075 mmol) in THF (1.25 mL). Stirred in vial at 50 °C for 20 hours. Analysis of crude ^1H NMR using internal standard 1,2-dimethoxyethane showed 2:1:1.2 *dimeta*:*meta*:*para* borylation in 94% yield. The crude product was purified by silica gel chromatography (20% EtOAc in Petroleum Ether 40-60 °C) gave the title compound (as a 2.1:1:1.2 mixture of *dimeta*:*meta*:*para* ratio, as determined by ^1H NMR) as a colourless oil (92 mg, 0.23 mmol, 91%). The mass quoted above is the actual mass of the isolated mixture of products, whilst the amount of substance in mmol has been calculated by examining the ratios of the *dimeta* to monoborylated products and accouting for the extra mass due to the higher molecular weight of the *dimeta* product. By subtracting the resonances for the *dimeta* isomer, the NMR data of the *meta* and *para* isomers can be assigned as follows:

Meta ^1H NMR (600 MHz, $CDCl_3$) δ 7.70 (d, *J* = 7.3 Hz, 1H), 7.65 (s, 1H), 7.34 (t, *J* = 7.5 Hz, 1H), 7.28 (d, *J* = 8.9 Hz, 1H), 6.53 (br, 1H), 3.80 – 3.46 (m, 2H), 2.94 – 2.73 (m2H), 1.34 (s, 12H); ^{13}**C NMR** (151 MHz, $CDCl_3$) δ 157.2 (q, *J* = 36.9 Hz), 136.8, 135.0, 133.3, 131.6, 128.3, 115.8 (q, *J* = 287.8 Hz), 83.8, 40.9, 34.8, 24.8.

Para ^1H NMR (600 MHz, $CDCl_3$) δ 7.78 (d, *J* = 8.0 Hz, 2H), 7.20 (d, *J* = 8.0 Hz, 2H), 6.53 (br, 1H), 3.65 – 3.52 (m, 2H), 2.95 – 2.85 (m, 2H), 1.34 (s, 12H); ^{13}**C NMR** (151 MHz, $CDCl_3$) δ 157.2 (q, *J* = 36.9 Hz), 140.8, 135.3, 128.1, 115. 8 (q, *J* = 287.8 Hz), 83.9, 41.0, 35.1, 24.8.

2,2,2-trifluoro-N-(2-(4-(4,4,5,5-tetramethyl-1,3,2-dioxaborolan-2-yl)pyridin-2-yl)ethyl)acetamide (5m)

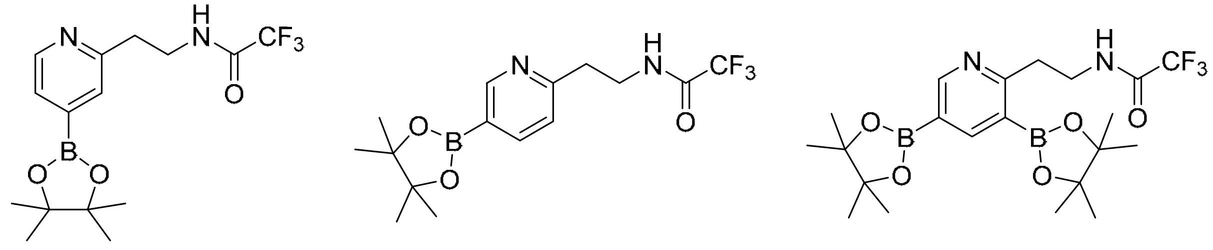

Meta ***Para*** ***Di(3,5)***

1a in THF:

Following general procedure F using **4m** (54.5 mg, 0.25 mmol), B_2pin_2 (95.2 mg, 0.375 mmol), $[Ir(COD)OMe]_2$ (2.5 mg, 0.00375 mmol) and **1a** (3.8 mg, 0.0075 mmol) in THF (1.25 mL). Stirred in vial at 35 °C for 6 hours. Analysis of crude ^1H NMR using internal standard 1,2-dimethoxyethane showed 6.1:1:0.3 *meta:para:di(3,5)* borylation (overall meta:para borylation 4.7:1) in 94% yield. Purification by silica gel chromatography proved not to be possible, leading to product decomposition. As such, the compounds were characterised from the crude, which contained pinacol borane impurity, and NMR yield was quoted in manuscript.

Meta ^1H NMR (500 MHz, $CDCl_3$) δ 8.49 (dd, *J* = 4.8, 0.9 Hz, 1H), 8.31 (br, 1H), 7.51 (s, 1H), 7.49 (d, *J* = 4.8 Hz, 1H), 3.75 – 3.67 (m, 2H), 3.04 – 2.98 (m, 2H), 1.31 (s, 12H); ^{13}**C NMR** (151 MHz, $CDCl_3$) δ ^{13}C NMR (126 MHz, $CDCl_3$) δ 158.0, 157.0 (q, *J* = 36.7 Hz), 148.3, 128.7, 126.9, 115.9 (q, *J* = 287.7 Hz), 84.6, 38.8, 35.1, 24.8. **HRMS** m/z: $[M + H]^+$ calc'd for $[C_{15}H_{21}BN_2O_3F_3]^+$ expect 345.1597; found 345.1601.

Dtbpy in THF:

Following general procedure F using **4m** (54.5 mg, 0.25 mmol), B_2pin_2 (95.2 mg, 0.375 mmol), $[Ir(COD)OMe]_2$ (2.5 mg, 0.00375 mmol) and dtbpy (2.0 mg, 0.0075 mmol) in THF (1.25 mL). Stirred in vial at 35 °C for 6 hours. Analysis of crude ^1H NMR using internal standard 1,2-dimethoxyethane showed 2:1:0.4 *meta*:*para*:*di(3,5)* borylation (overall *meta*:*para* borylation 1.4:1) in 99% yield. Purification by silica gel chromatography proved not to be possible, leading to product decomposition. As such, the compounds was characterised from the crude, which contained pinacol borane impurity. By subtracting the resonances for the *meta* isomer, the NMR data of the *para* and *di(3,5)* borylated isomer can be assigned as follows:

Para ^1H NMR (500 MHz, $CDCl_3$) δ ^1H NMR (500 MHz, $CDCl_3$) δ 8.82 (s, 1H), 8.37 (br, 1H), 7.99 (dd, *J* = 7.7, 1.8 Hz, 1H), 7.16 (d, *J* = 7.7 Hz, 1H), 3.76 – 3.64 (m, 2H), 3.07 – 2.99 (m, 2H), 1.32 (s, 12H); ^{13}**C NMR** (126 MHz, $CDCl_3$) δ 161.1, 157.0 (q, *J* = 36.7 Hz), 154.7, 143.3, 123.0, 115.9 (q, *J* = 287.7 Hz), 84.3, 38.7, 35.4, 24.8.

Di(3,5) ^1H NMR (500 MHz, $CDCl_3$) δ 8.86 (d, *J* = 1.9 Hz, 1H), 8.42 (d, *J* = 1.9 Hz, 1H), 3.78 – 3.65 (m, 2H), 3.34 – 3.29 (m, 2H), 1.32 (s, 24H); ^{13}**C NMR** (126 MHz, $CDCl_3$) δ 166.5, 157.03 (q, *J* = 36.7 Hz), 156.4, 150.9, 115.9 (q, *J* = 287.7 Hz), 84.5, 84.2, 39.4, 39.0, 24.8.

2,2,2-Trifluoro-*N*-(3-(5-(4,4,5,5-tetramethyl-1,3,2-dioxaborolan-2-yl)-2-(trifluoromethyl)phenyl)propyl)acetamide (7a)

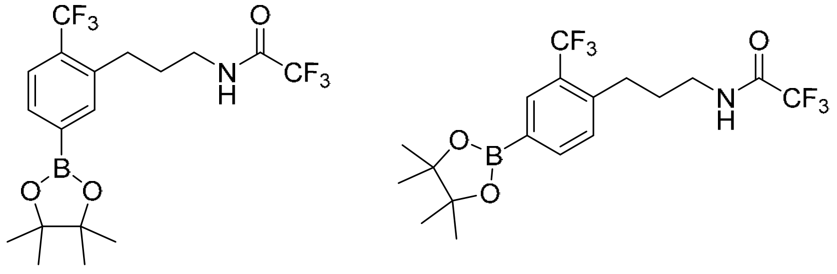

meta ***para***

1A in THF:

Following general procedure F using 2,2,2-trifluoro-*N*-(3-(2-(trifluoromethyl)phenyl)propyl)acetamide (74.8 mg, 0.25 mmol), B_2pin_2 (127 mg, 0.50 mmol), $[Ir(COD)OMe]_2$ (2.5 mg, 0.00375 mmol) and **1A** (3.8 mg, 0.0075 mmol) in THF (1.25 mL). The reaction was stirred at 50 °C for 20 hours before cooling and the solvents removed. Analysis of crude ^{1}H NMR using internal standard 1,2-dimethoxyethane showed 19.5:1 *meta*:*para* borylation in 97% yield. The crude product was purified by silica gel chromatography (Pet. Ether (40-60):EtOAc (19:1) to give the title compound (as a 19:1 mixture of *meta*:*para* ratio, as determined by ^{1}H NMR) as a white solid (103.5 mg, 0.24 mmol, 97%).

Meta ^{1}H NMR (600 MHz, $CDCl_3$) δ 7.74 (s, 1H), 7.73 (d, *J* = 8.0 Hz, 1H), 7.61 (d, *J* = 8.0 Hz, 1H), 6.50 (br s, 1H), 3.44 (dt, *J* = 6.5, 6.7 Hz, 2H), 2.84 (app t, *J* = 7.9 Hz, 2H), 1.94 (tt, *J* = 7.3, 7.9 Hz, 2H), 1.36 (s, 12H); **^{13}C NMR** (151 MHz, $CDCl_3$) δ 157.3 (q, $^2J_{C-F}$ = 37 Hz), 138.5, 137.1, 132.7, 130.6 (q, $^2J_{C-F}$ = 30 Hz), 125. 3 (q, $^3J_{C-F}$ = 6 Hz), 124.5 (q, $^1J_{C-F}$ = 274 Hz), 115.8 (q, $^1J_{C-F}$ = 288 Hz), 84.3, 39.7, 30.9, 29.7, 24.8; **HRMS** m/z: $[M + H]^+$ calc'd for $[C_{18}H_{23}BF_6NO_3]^+$ expect 425.1711; found 425.1704.

Dtbpy in THF:

Following general procedure F using 2,2,2-trifluoro-*N*-(3-(2-(trifluoromethyl)phenyl)propyl)acetamide (74.8 mg, 0.25 mmol), B_2pin_2 (127 mg, 0.50 mmol), $[Ir(COD)OMe]_2$ (2.5 mg, 0.00375 mmol) and dtbpy (2.0 mg, 0.0075 mmol) in THF (1.25 mL). The reaction was stirred at 50 °C for 20 hours before cooling and the solvents removed. Analysis of crude ^{1}H NMR using internal standard 1,2-dimethoxyethane showed 0.8:1 *meta*:*para* borylation in 96% yield. The crude product was purified by silica gel chromatography (Pet. Ether (40-60):EtOAc (19:1) to give the title compound (as a 1.2:1 mixture of *meta*:*para* ratio, as determined by ^{1}H NMR) as a colourless oil (89.0 mg, 0.21 mmol, 84%). By subtracting the resonances for the *meta* isomer, the NMR data of the *para* isomer can be assigned as follows:

Para ^{1}H NMR (600 MHz, $CDCl_3$) δ 8.05 (s, 1H), 7.90 (d, *J* = 7.7 Hz, 1H), 7.32 (d, *J* = 7.7 Hz, 1H), 6.74 (br s, 1H), 3.43 (dt, *J* = 6.8, 6.4 Hz, 2H), 2.84-2.81 (m, 2H), 1.95-1.88 (m, 2H), 1.34 (s, 12H); **^{13}C NMR** (151 MHz, $CDCl_3$) δ 157.4 (q, $^2J_{C-F}$ = 37 Hz), 142.6, 138.2, 132.3 (q, $^3J_{C-F}$ = 6 Hz), 130.3, 127.4 (q, $^2J_{C-F}$ = 30 Hz), 124.7 (q, $^1J_{C-F}$ = 274 Hz), 115.8 (q, $^1J_{C-F}$ = 288 Hz), 84.2, 39.6, 30.6, 29.8, 24.8.

N-(3-(3,5-bis(4,4,5,5-tetramethyl-1,3,2-dioxaborolan-2-yl)phenyl)propyl)-2,2,2-trifluoroacetamide (7b)

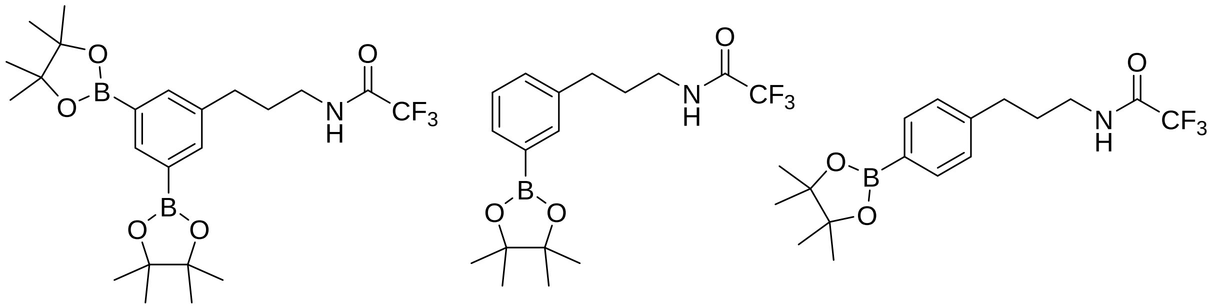

dimeta ***meta*** ***para***

1a in THF:

Following general procedure F using 2,2,2-trifluoro-*N*-(3-phenylpropyl)acetamide (57.8 mg, 0.25 mmol), B_2pin_2 (381 mg, 1.50 mmol), $[Ir(COD)OMe]_2$ (5.0 mg, 0.0075 mmol) and **1a** (7.6 mg, 0.015 mmol) in THF (1.25 mL). The reaction was stirred at 50 °C for 20 hours before cooling and the solvents removed. Analysis of crude ^{1}H NMR using internal standard 1,2-dimethoxyethane showed 74:16:6 *dimeta*:*meta*:*para* borylation in 96% yield. The crude product was purified by silica gel chromatography (Pet. Ether (40-60):EtOAc (9:1) to give a clean fraction of the title compound as a white solid (74.2 mg, 0.15 mmol, 61%), and a mixed fraction (as a 34:46:15 *dimeta*:*meta*:*para* ratio, as determined by ^{1}H NMR) as a colourless oil (38.8 mg, 0.08 mmol, 32%*). The yield quoted is that of the pure *dimeta* product isolated.

Dimeta ^{1}H NMR (500 MHz, $CDCl_3$) δ 8.14 (s, 1H), 7.71 (s, 2H), 6.27 (br s, 1H), 3.38 (dt, *J* = 6.7, 6.8 Hz, 2H), 2.69 (t, *J* = 7.8 Hz, 2H), 1.96 (quint, 7.4 Hz, 2H), 1.34 (s, 24H); **^{13}C NMR** (126 MHz, $CDCl_3$) δ 157.1 (q, $^2J_{C-F}$ = 37 Hz), 139.4, 139.1, 137.4, 155.8 (q, $^1J_{C-F}$ = 288 Hz), 83.8, 39.8, 33.0, 30.4, 24.9; **HRMS** m/z: $[M + H]^+$ calc'd for $[C_{23}H_{35}{}^{10}B^{11}BF_3NO_5]^+$ expect 483.2690; found 483.2698.

*Yield calculated from a weighted average mass of mono and diborylated products.

Dtbpy in THF:

Following general procedure F using 2,2,2-trifluoro-*N*-(3-phenylpropyl)acetamide (57.8 mg, 0.25 mmol), B_2pin_2 (274 mg, 1.0 mmol), $[Ir(COD)OMe]_2$ (2.5 mg, 0.00375 mmol) and dtbpy (2.0 mg, 0.0075 mmol) in THF (1.25 mL). The reaction was stirred at 50 °C for 20 hours before cooling and the solvents removed. Analysis of crude ^{1}H NMR using internal standard 1,2-dimethoxyethane showed 52:28:26 *dimeta*:*meta*:*para* borylation in 99% yield. The crude product was purified by silica gel chromatography (Pet. Ether (40-60):EtOAc (9:1) to give a fraction of the mono borylated products to aid characterisation (as a 1:1.5 mixture of *meta*:*para* ratio, as determined by ^{1}H NMR) as a colourless oil (32.2 mg, 0.09 mmol, 36%), the NMR yield was used for direct comparison. By using a combination of 2D NMR analysis the NMR data of the *meta* and *para* isomers can be assigned as follows:

Meta ^{1}H NMR (500 MHz, $CDCl_3$) δ 7.67 (d, *J* = 6.9 Hz, 1H), 7.62 (s, 1H), 7.31 (t, *J* = 7.1 Hz, 1H), 7.27 (dt, *J* = 7.9, 1.7 Hz), 6.32 (br s, 1H), 3.40-3.37 (m, 2H), 2.69 (t, *J* = 7.4 Hz, 2H), 1.94 (tt, *J* = 7.2, 7.7 Hz, 2H), 1.35 (s, 12H); **^{13}C NMR** (126 MHz, $CDCl_3$) δ 157.2 (q, $^2J_{C-F}$ = 37 Hz), 139.9, 134.5, 132.8, 131.3, 128.1, 115.8 (q, $^1J_{C-F}$ = 288 Hz), 83.8, 39.7, 33.0, 30.4, 24.8.

Para ^{1}H NMR (500 MHz, $CDCl_3$) δ 7.75 (d, *J* = 8.2 Hz, 2H), 7.18 (d, *J* = 8.2 Hz, 2H), 6.32 (br s, 1H), 3.39-3.35 (m, 2H), 2.69 (t, *J* = 7.4 Hz, 2H), 1.93 (tt, *J* = 6.9, 7.4 Hz, 2H), 1.34 (s, 12H); **^{13}C NMR** (126 MHz, $CDCl_3$) δ 157.2 (q, $^2J_{C-F}$ = 37 Hz), 143.9, 135.2, 127.7, 115.8 (q, $^1J_{C-F}$ = 288 Hz), 83.7, 39.5, 33.2, 30.2, 24.8.

N-(3-(2-bromo-5-(4,4,5,5-tetramethyl-1,3,2-dioxaborolan-2-yl)phenyl)propyl)-2,2,2-trifluoroacetamide (7c)

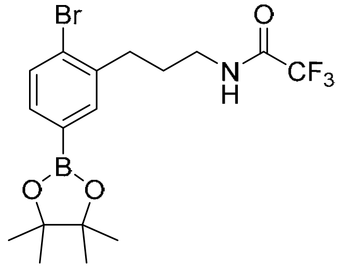

meta

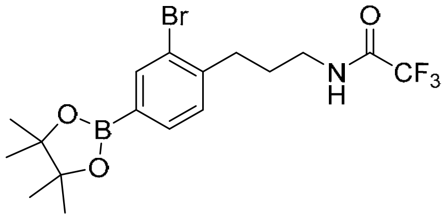

para

1a in THF:

Following general procedure F using *N*-(3-(2-bromophenyl)propyl)-2,2,2-trifluoroacetamide (77.5 mg, 0.25 mmol), B_2pin_2 (127 mg, 0.50 mmol), $[Ir(COD)OMe]_2$ (2.5 mg, 0.00375 mmol) and **1a** (3.8 mg, 0.0075 mmol) in THF (1.25 mL). The reaction was stirred at 35 °C for 20 hours before cooling and the solvents removed. Analysis of crude ^{1}H NMR using internal standard 1,2-dimethoxyethane showed 9.1:1 *meta*:*para* borylation in 98% yield. The crude product was purified by silica gel chromatography (Pet. Ether (40-60):EtOAc (19:1-8:1) to give the title compound (as a 8.5:1 mixture of *meta*:*para* ratio, as determined by ^{1}H NMR) as a colourless oil (103.0 mg, 0.24 mmol, 94%).

Meta ^{1}H NMR (600 MHz, $CDCl_3$) δ 7.63 (d, *J* = 1.1 Hz, 1H), 7.54 (d, *J* = 7.9 Hz, 1H), 7.49 (dd, *J* = 1.1, 7.9 Hz, 1H), 6.59 (s, 1H), 3.41 (dt, *J* = 6.5, 6.8 Hz, 2H), 2.81 (app t, *J* = 7.8 Hz, 2H), 1.93 (tt, *J* = 7.2, 7.7 Hz, 2H), 1.34 (s, 12H); ^{13}**C NMR** (151 MHz, $CDCl_3$) δ 157.3 (q, $^2J_{C-F}$ = 37 Hz), 139.4, 136.5, 134.2, 132.5, 127.9, 115.8 (q, $^1J_{C-F}$ = 287 Hz), 84.1, 39.5, 33.1, 29.1, 24.8; **HRMS** m/z: $[M + H]^+$ calc'd for $[C_{17}H_{23}BNO_3BrF_3]^+$ expect 436.0906; found 436.0898.

Dtbpy in THF:

Following general procedure F using *N*-(3-(2-bromophenyl)propyl)-2,2,2-trifluoroacetamide (77.5 mg, 0.25 mmol), B_2pin_2 (127 mg, 0.50 mmol), $[Ir(COD)OMe]_2$ (2.5 mg, 0.00375 mmol) and dtbpy (2.0 mg, 0.0075 mmol) in THF (1.25 mL). The reaction was stirred at 35 °C for 20 hours before cooling and the solvents removed. Analysis of crude ^{1}H NMR using internal standard 1,2-dimethoxyethane showed 1:2 *meta*:*para* borylation in 93% yield. The crude product was purified by silica gel chromatography (Pet. Ether (40-60):EtOAc (19:1-8:1) to give the title compound (as a 1:1.7 mixture of *meta*:*para* ratio, as determined by ^{1}H NMR) as a colourless oil (85.9 mg, 0.20 mmol, 79%). By subtracting the resonances for the *meta* isomer, the NMR data of the *para* isomer can be assigned as follows:

Para ^{1}H NMR (600 MHz, $CDCl_3$) δ 7.97 (s, 1H), 7.66 (d, *J* = 7.5 Hz, 1H), 7.21 (d, *J* = 7.5 Hz, 1H), 6.57 (br s, 1H), 3.41 (dt, *J* = 7.0, 6.7 Hz, 2H), 2.81 (app t, *J* = 7.8 Hz, 2H), 1.92 (tt, *J* = 7.4, 8.4 Hz, 2H), 1.34 (s, 12H) ; ^{13}**C NMR** (151 MHz, $CDCl_3$) δ 157.3 (q, $^2J_{C-F}$ = 37 Hz), 143.0, 139.2, 133.9, 129.9, 124.3, 115.8 (q, $^1J_{C-F}$ = 287 Hz), 84.1, 39.4, 33.4, 29.0, 24.8.

N-(3-(2-chloro-5-(4,4,5,5-tetramethyl-1,3,2-dioxaborolan-2-yl)phenyl)propyl)-2,2,2-trifluoroacetamide (7d)

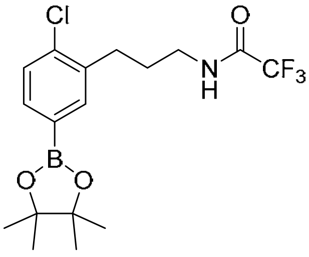

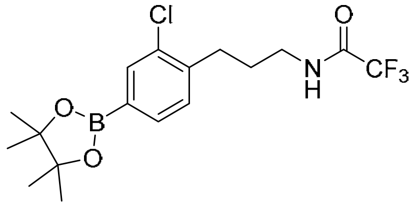

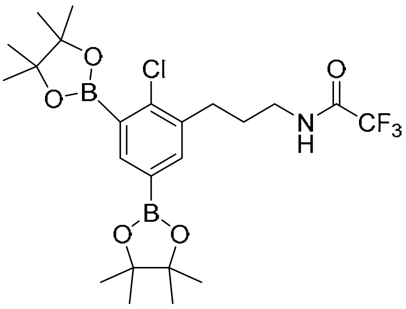

meta ***para*** ***dimeta***

1a in THF:

Following general procedure F using *N*-(3-(2-chlorophenyl)propyl)-2,2,2-trifluoroacetamide (66.4 mg, 0.25 mmol), B_2pin_2 (127 mg, 0.50 mmol), $[Ir(COD)OMe]_2$ (2.5 mg, 0.00375 mmol) and **1a** (3.8 mg, 0.0075 mmol) in THF (1.25 mL). The reaction was stirred at 50 °C for 20 hours before cooling and the solvents removed. Analysis of crude ^{1}H NMR using internal standard 1,2-dimethoxyethane showed 10:78:10 *dimeta*:*meta*:*para* borylation in 98% yield. The crude product was purified by silica gel chromatography (Pet. Ether (40-60):EtOAc (19:1-9:1) to give the title compound (as a 7:78:10 mixture of dimeta:*meta*:*para* ratio, as determined by ^{1}H NMR) as a colourless oil (97.1 mg, 0.24 mmol, 97%*).

Meta ^{1}H NMR (600 MHz, $CDCl_3$) δ 7.63 (d, *J* = 0.8 Hz, 1H), 7.58 (dd, *J* = 0.8, 78 Hz, 1H), 7.34 (d, *J* = 7.8 Hz, 1H), 6.61 (br s, 1H), 3.40 (dt, *J* = 6.7, 6.8 Hz, 2H), 2.80 (app t, *J* = 8.0 Hz, 2H), 1.93 (tt, *J* = 7.5, 7.6 Hz, 2H), 1.34 (s, 12 H); **^{13}C NMR** (151 MHz, $CDCl_3$) δ 157.3 (q, $^2J_{C-F}$ = 36 Hz), 137.6, 137.1, 136.7, 134.1, 129.1, 115.8 (q, $^1J_{C-F}$ = 289 Hz), 84.1, 39.6, 30.5, 29.0, 24.8; **HRMS** m/z: $[M + H]^+$ calc'd for $[C_{17}H_{23}BClF_3NO_3]^+$ expect 391.1448; found 391.1443.

*Yield calculated from a weighted average mass of mono and diborylated products.

Dtbpy in THF:

Following general procedure F using *N*-(3-(2-chlorophenyl)propyl)-2,2,2-trifluoroacetamide (66.4 mg, 0.25 mmol), B_2pin_2 (127 mg, 0.50 mmol), $[Ir(COD)OMe]_2$ (2.5 mg, 0.00375 mmol) and dtbpy (2.0 mg, 0.0075 mmol) in THF (1.25 mL). The reaction was stirred at 50 °C for 20 hours before cooling and the solvents removed. Analysis of crude ^{1}H NMR using internal standard 1,2-dimethoxyethane showed 4:37:55 *dimeta*:*meta*:*para* borylation in 95% yield. The crude product was purified by silica gel chromatography (Pet. Ether (40-60):EtOAc (19:1-9:1) to give the title compound (as a 1:1.6 mixture of *meta*:*para* ratio, as determined by ^{1}H NMR) as a colourless oil (94.0 mg, 0.24 mmol, 96%). By subtracting the resonances for the *meta* isomer, the NMR data of the *para* isomer can be assigned as follows:

Para ^{1}H NMR (600 MHz, $CDCl_3$) δ 7.78 (s, 1H), 7.62 (d, *J* = 7.5 Hz, 1H), 7.21 (d, *J* = 7.5 Hz, 1H), 6.55 (br s, 1H), 3.40 (dt, *J* = 6.7, 6.8 Hz, 2H), 2.80 (app t, *J* = 8.0 Hz, 2H), 1.92 (tt, *J* = 7.5, 7.6 Hz, 2H), 1.34 (s, 12 H); **^{13}C NMR** (151 MHz, $CDCl_3$) δ 157.3 (q, $^2J_{C-F}$ = 37 Hz), 141.3, 135.7, 133.6, 133.3, 129.9, 115.8 (q, $^1J_{C-F}$ = 289 Hz), 84.1, 39.4, 30.8, 28.8, 24.8.

2,2,2-Trifluoro-*N*-(3-(2-fluoro-3,5-bis(4,4,5,5-tetramethyl-1,3,2-dioxaborolan-2-yl)phenyl)propyl)acetamide (7e)

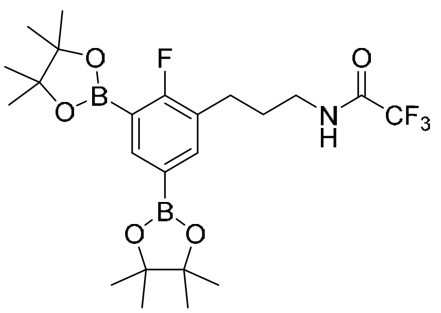

dimeta

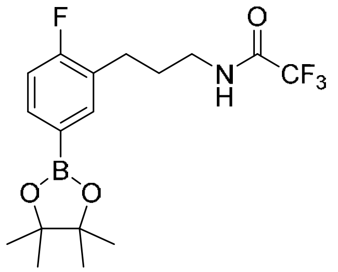

para

1a in THF:

Following general procedure F using *N*-(3-(2-fluorophenyl)propyl)-2,2,2-trifluoroacetamide (62.3 mg, 0.25 mmol), B_2pin_2 (254 mg, 1.0 mmol), $[Ir(COD)OMe]_2$ (2.5 mg, 0.00375 mmol) and **1a** (3.8 mg, 0.0075 mmol) in THF (1.25 mL). The reaction was stirred at 50 °C for 20 hours before cooling and the solvents removed. Analysis of crude with a combination of ^{1}H and ^{19}F NMRs using internal standard 1,2-dimethoxyethane showed 62:7.5:9.7 *dimeta*:*monometas*:*para* borylation in 79% yield. The crude product was purified by silica gel chromatography (Pet. Ether (40-60):EtOAc (19:1-8:1) to give the title compound as a white solid (80.3 mg, 0.10 mmol, 40%).

Dimeta ^{1}H NMR (600 MHz, $CDCl_3$) δ 8.07 (d, *J* = 5.7 Hz, 1H), 7.2 (d, *J* = 8.0 Hz, 1H), 6.44 (br s, 1H), 3.38 (q, *J* = 6.6 Hz, 2H), 2.70 (app t, *J* = 7.6 Hz, 2H), 1.92 (tt, *J* = 7.2, 7.3 Hz, 2H), 1.36 (s, 12H), 1.34 (s, 12H); **^{13}C NMR** (151 MHz, $CDCl_3$) δ 167.7 (d, $^1J_{C-F}$ = 254 Hz), 157.2 (q, $^2J_{C-F}$ = 37 Hz), 142.2 (d, $^3J_{C-F}$ = 9 Hz), 140.7 (d, $^3J_{C-F}$ = 6 Hz), 126.6 (d, $^2J_{C-F}$ = 18 Hz), 115.8 (q, $^1J_{C-F}$ = 289 Hz), 83.93, 83.91, 39.5, 29.2, 26.3 (d, $^3J_{C-F}$ = 3 Hz), 24.82, 24.80; **^{19}F NMR** (376 MHz, $CDCl_3$) δ -76.83 ($COCF_3$), -105.27 (C-F); **HRMS** m/z: $[M + H]^+$ calc'd for $[C_{23}H_{34}B_2NO_5F_4]^+$ expect 502.2559; found 502.2563.

Dtbpy in THF:

Following general procedure F using *N*-(3-(2-fluorophenyl)propyl)-2,2,2-trifluoroacetamide (62.3 mg, 0.25 mmol), B_2pin_2 (254 mg, 1.0 mmol), $[Ir(COD)OMe]_2$ (2.5 mg, 0.00375 mmol) and dtbpy (2.0 mg, 0.0075 mmol) in THF (1.25 mL). The reaction was stirred at 50 °C for 20 hours before cooling and the solvents removed. Analysis of crude ^{1}H NMR using internal standard 1,2-dimethoxyethane showed 37:18:36 *dimeta*:*monometas*:*para* in 90% yield. The crude product was purified by silica gel chromatography (Pet. Ether (40-60):EtOAc (19:1-9:1) to give a clean fraction of the *para* isomer as a white solid (20.1 mg, 0.05 mmol, 20%), the NMR was used for comparison.

Para ^{1}H NMR (600 MHz, $CDCl_3$) δ 7.51 (dd, *J* = 1.0, 7.4 Hz, 1H), 7.45 (dd, *J* = 0.7, 10.4 Hz, 1H), 7.18 (t, *J* = 7.4 Hz, 1H), 6.34 (br s, 1H), 3.37 (q, *J* = 6.6 Hz, 2H), 2.73 (t, *J* = 7.4 Hz, 2H), 1.92 (quint, *J* = 7.2 Hz, 2H), ; **^{13}C NMR** (151 MHz, $CDCl_3$) δ 160.8 (d, $^1J_{C-F}$ = 245.2 Hz), 157.2 (q, $^2J_{C-F}$ = 37.3 Hz), 130.7 (d, $^3J_{C-F}$ = 3.5 Hz), 121.0 (d, $^2J_{C-F}$ = ~18 Hz), 130.2 (d, $^3J_{C-F}$ = 4.1 Hz), 121.1 (d, $^2J_{C-F}$ = 20.3 Hz), 115.8 (q, $^1J_{C-F}$ = 288.1 Hz), 84.1, 39.3, 29.1, 38.1 (d, $^3J_{C-F}$ = 2.0 Hz), 24.8; **^{19}F NMR** (376 MHz, $CDCl_3$) δ-76.83 ($COCF_3$), -121.36 (C-F).

2,2,2-Trifluoro-*N*-(3-(3-fluoro-5-(4,4,5,5-tetramethyl-1,3,2-dioxaborolan-2-yl)phenyl)propyl)acetamide (7f)

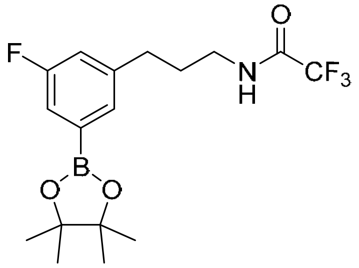

meta

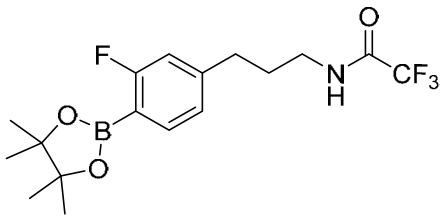

para

1a in THF:

Following general procedure F using *N*-(3-(3-fluorophenyl)propyl)-2,2,2-trifluoroacetamide (62.3 mg, 0.25 mmol), B_2pin_2 (127 mg, 0.50 mmol), $[Ir(COD)OMe]_2$ (2.5 mg, 0.00375 mmol) and **1a** (3.8 mg, 0.0075 mmol) in THF (1.25 mL). The reaction was stirred at 35 °C for 20 hours before cooling and the solvents removed. Analysis of crude ^{1}H NMR using internal standard 1,2-dimethoxyethane showed 13.0:1 *meta*:*para* borylation in 97% yield. The crude product was purified by silica gel chromatography (Pet. Ether (40-60):EtOAc (19:1-9:1) to give the title compound (as a 13.1:1 mixture of *meta*:*para* ratio, as determined by ^{1}H NMR) as a colourless oil (86.9 mg, 0.23 mmol, 93%).

Meta ^{1}H NMR (600 MHz, $CDCl_3$) δ 7.39 (s, 1H), 7.32 (dd, *J* = 2.4, 8.8 Hz, 1H), 6.96 (dt, *J* = 9.7, 1.7 Hz, 1H), 6.58 (br s, 1H), 3.38 (dt, *J* = 6.8, 6.6 Hz, 2H), 2.67 (t, *J* = 7.8 Hz, 2H), 1.93 (tt, *J* = 7.4, 7.5 Hz, 2H), 1.34 (s, 12H); **^{13}C NMR** (151 MHz, $CDCl_3$) δ 162.6 (d, $^{1}J_{C-F}$ = 248 Hz), 157.3 (q, $^{2}J_{C-F}$ = 37 Hz), 142.7 (d, $^{3}J_{C-F}$ = 6 Hz), 130.1 (d, $^{4}J_{C-F}$ = 2 Hz), 118.8 (d, $^{2}J_{C-F}$ = 20 Hz), 118.0 (d, $^{2}J_{C-F}$ = 22 Hz), 115.8 (q, $^{1}J_{C-F}$ = 287 Hz), 84.1, 39.6, 32.6 (d, $^{4}J_{C-F}$ = 1 Hz), 30.2, 24.8; **HRMS** m/z: $[M + H]^+$ calc'd for $[C_{17}H_{23}BF_4NO_3]^+$ expect 376.1707; found 376.1713.

Dtbpy in THF:

Following general procedure F using *N*-(3-(3-fluorophenyl)propyl)-2,2,2-trifluoroacetamide (62.3 mg, 0.25 mmol), B_2pin_2 (127 mg, 0.50 mmol), $[Ir(COD)OMe]_2$ (2.5 mg, 0.00375 mmol) and dtbpy (2.0 mg, 0.0075 mmol) in THF (1.25 mL). The reaction was stirred at 35 °C for 20 hours before cooling and the solvents removed. Analysis of crude ^{1}H NMR using internal standard 1,2-dimethoxyethane showed 1.4:1 *meta*:*para* borylation in 92% yield. The crude product was purified by silica gel chromatography (Pet. Ether (40-60):EtOAc (19:1-9:1) to give the title compound (as a 1.3:1 mixture of *meta*:*para* ratio, as determined by ^{1}H NMR) as a colourless oil (51.4 mg, 0.14 mmol, 55%). By subtracting the resonances for the *meta* isomer, the NMR data of the *para* isomer can be assigned as follows:

Para ^{1}H NMR (600 MHz, $CDCl_3$) δ 7.67 (t, *J* = 7.2 Hz, 1H), 6.97 (d, *J* = 7.2 Hz, 1H), 6.86 (d, *J* = 10.2 Hz, 1H), 6.42 (br s, 1H), 3.38 (dt, *J* = 6.8, 6.6 Hz, 2H), 2.68 (t, *J* = 7.6 Hz, 2H), 1.92 (tt, *J* = 7.5, 8.6 Hz, 2H), 1.35 (s, 12H); **^{13}C NMR** (151 MHz, $CDCl_3$) δ167.4 (d, $^{1}J_{C-F}$ = 252 Hz), 157.3 (q, $^{2}J_{C-F}$ = 37 Hz), 146.9 (d, $^{3}J_{C-F}$ = 8 Hz), 137.1 (d, $^{4}J_{C-F}$ = 9 Hz), 123.7 (d, $^{4}J_{C-F}$ = 3 Hz), 115.8 (q, $^{1}J_{C-F}$ = 287 Hz), 115.0 (d, $^{2}J_{C-F}$ = 23 Hz), 83.8, 39.4, 32.8 (d, $^{4}J_{C-F}$ = 1 Hz), 29.9, 24.8.

Methyl 4-(4,4,5,5-tetramethyl-1,3,2-dioxaborolan-2-yl)-2-(3-(2,2,2-trifluoroacetamido)propyl)benzoate (7g)

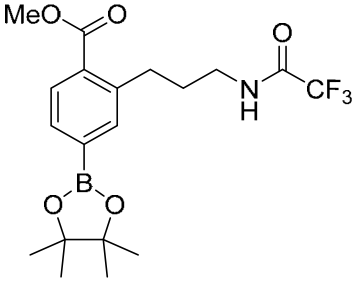

meta

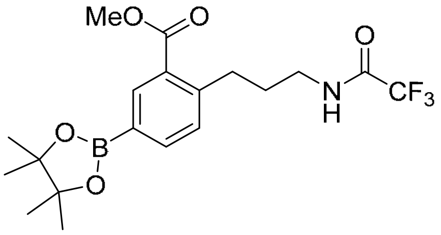

para

1a in THF:

Following general procedure F using methyl 2-(3-(2,2,2-trifluoroacetamido)propyl)benzoate (57.9 mg, 0.20 mmol), B_2pin_2 (101 mg, 0.40 mmol), $[Ir(COD)OMe]_2$ (2.0 mg, 0.0030 mmol) and **1a** (3.0 mg, 0.0060 mmol) in THF (1.0 mL). The reaction was stirred at 50 °C for 20 hours before cooling and the solvents removed. Analysis of crude ^{1}H NMR using internal standard 1,2-dimethoxyethane showed >20:1 *meta*:*para* borylation in 100% yield. The crude product was purified by silica gel chromatography (Pet. Ether (40-60):EtOAc (9:1-8-1) to give the title compound (as a 20:1 mixture of *meta*:*para* ratio, as determined by ^{1}H NMR) as a colourless oil (60.3 mg, 0.145 mmol, 73%).

Meta ^{1}H NMR (600 MHz, $CDCl_3$) δ 7.90 (d, *J* = 8.1 Hz, 1H), 7.71 (d, *J* = 8.1 Hz, 1H), 7.70 (s, 1H), 7.61 (br s, 1H), 3.91 (s, 3H), 3.37 (dt, *J* = 6.2, 5.6 Hz, 2H), 3.00 (t, *J* = 7.6 Hz, 2H), 1.95 (tt, *J* = 6.2, 7.2 Hz, 2H), 1.36 (s, 12H); **^{13}C NMR** (151 MHz, $CDCl_3$) δ 168.3, 157.4 (q, $^2J_{C\text{-}F}$ = 37 Hz), 142.1, 137.4, 132.5, 131.2, 129.9, 116.0 (q, $^1J_{C\text{-}F}$ = 288 Hz), 84.2, 52.2, 39.1, 30.7, 30.4, 24.8; **HRMS** m/z: $[M + H]^+$ calc'd for $[C_{19}H_{25}BF_3NO_5]^+$ expect 415.1778; found 415.1775.

Dtbpy in THF:

Following general procedure F using using methyl 2-(3-(2,2,2-trifluoroacetamido)propyl)benzoate (57.9 mg, 0.20 mmol), B_2pin_2 (101 mg, 0.40 mmol), $[Ir(COD)OMe]_2$ (2.0 mg, 0.0030 mmol) and dtbpy (1.6 mg, 0.0060 mmol) in THF (1.0 mL). The reaction was stirred at 50 °C for 20 hours before cooling and the solvents removed. Analysis of crude ^{1}H NMR using internal standard 1,2-dimethoxyethane showed 3:1 *meta*:*para* borylation in 76% yield. The crude product was purified by silica gel chromatography (Pet. Ether (40-60):EtOAc (9:1-8:1) to give the title compound (as a 2.7:1 mixture of *meta*:*para* ratio, as determined by ^{1}H NMR) as a colourless oil (43.0 mg, 0.10 mmol, 69%).

By subtracting the resonances for the *meta* isomer, the NMR data of the *para* isomer can be assigned as follows:

Para ^{1}H NMR (500 MHz, $CDCl_3$) δ 8.35 (d, *J* = 1.1 Hz, 1H), 7.88 (dd, *J* = 1.2, 7.6 Hz, 1H), 7.67 (br s, 1H), 7.28 (d, *J* = 7.8 Hz, 1H), 3.91 (s, 3H), 3.35 (dt, *J* = 6.3, 4.5 Hz, 2H), 3.00 (t, *J* = 7.6 Hz, 2H), 1.96-1.91 (m, 2H), 1.35 (s, 12H); **^{13}C NMR** (126 MHz, $CDCl_3$) δ 168.4, 157.4 (q, $^2J_{C\text{-}F}$ = 37 Hz), 146.3, 138.7, 137.3, 130.6, 126.6, 116.0 (q, $^1J_{C\text{-}F}$ = 288 Hz), 84.1, 52.2, 39.0, 30.9, 30.3, 24.9.

N-(3-(2,5-difluoro-3-(4,4,5,5-tetramethyl-1,3,2-dioxaborolan-2-yl)phenyl)propyl)-2,2,2-trifluoroacetamide (7h)

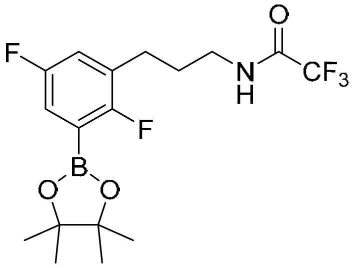

meta

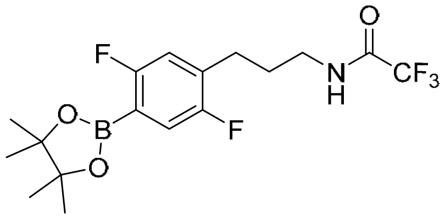

para

1A in THF:

Following general procedure F using *N*-(3-(2,5-difluorophenyl)propyl)-2,2,2-trifluoroacetamide (66.8 mg, 0.25 mmol), B_2pin_2 (127 mg, 0.50 mmol), $[Ir(COD)OMe]_2$ (2.5 mg, 0.00375 mmol) and **1A** (3.8 mg, 0.0075 mmol) in THF (1.25 mL). The reaction was stirred at 35 °C for 20 hours before cooling and the solvents removed. Analysis of crude ^{1}H NMR using internal standard 1,2-dimethoxyethane showed 5.2:1 *meta*:*para* borylation in 99% yield. The crude product was purified by silica gel chromatography (Pet. Ether (40-60):EtOAc (19:1-9:1) to give the title compound (as a 5.7:1 mixture of *meta*:*para* ratio, as determined by ^{1}H NMR) as a colourless oil (80.0 mg, 0.20 mmol, 81%).

Meta ^{1}H NMR (600 MHz, $CDCl_3$) δ 7.26 (dt, *J* = 4.2, 3.4 Hz, 1H), 6.97 (ddd, *J* = 3.4, 5.3 8.2 Hz, 1H), 6.50 (br s, 1H), 3.39 (q, *J* = 6.6 Hz, 2H), 2.67 (t, *J* = 7.6 Hz, 2H), 1.91 (tt, *J* = 7.2, 7.4 Hz, 2H), 1.36 (s, 12H); **^{13}C NMR** (151 MHz, $CDCl_3$) δ 161.0 (dd, $^1J_{C-F}$ = 246 Hz, $^4J_{C-F}$ = 2 Hz), 158.3 (dd, $^1J_{C-F}$ = 243 Hz , $^4J_{C-F}$ = 2 Hz), 157.3 (q, $^2J_{C-F}$ = 37 Hz), 129.2 (dd, $^2J_{C-F}$ = 21 Hz, $^3J_{C-F}$ = 7 Hz), 120.2 (dd, , $^2J_{C-F}$ = 22 Hz , $^3J_{C-F}$ = 9 Hz), 120.1 (dd, $^2J_{C-F}$ = 24 Hz, $^3J_{C-F}$ = 6 Hz), 115.8 (q, $^2J_{C-F}$ = 289 Hz), 84.3, 39.3, 28.9, 26.4, 24.8; **HRMS** m/z: $[M + H]^+$ calc'd for $[C_{17}H_{22}BF_5NO_3]^+$ expect 394.1607; found 394.1597.

Dtbpy in THF:

Following general procedure F using using *N*-(3-(2,5-difluorophenyl)propyl)-2,2,2-trifluoroacetamide (66.8 mg, 0.25 mmol), B_2pin_2 (127 mg, 0.50 mmol), $[Ir(COD)OMe]_2$ (2.5 mg, 0.00375 mmol) and dtbpy (2.0 mg, 0.0075 mmol) in THF (1.25 mL). The reaction was stirred at 35 °C for 20 hours before cooling and the solvents removed. Analysis of crude ^{1}H NMR using internal standard 1,2-dimethoxyethane showed 0.9:1 *meta*:*para* borylation in 97% yield. The crude product was purified by silica gel chromatography (Pet. Ether (40-60):EtOAc (19:1-9:1) to give the title compound (as a 0.9:1 mixture of *meta*:*para* ratio, as determined by ^{1}H NMR) as a colourless oil (79.7 mg, 0.20 mmol, 81%). By subtracting the resonances for the *meta* isomer, the NMR data of the *para* isomer can be assigned as follows:

Para ^{1}H NMR (600 MHz, $CDCl_3$) δ 7.36 (dd, *J* = 4.5, 9.7 Hz, 1H), 6.86 (dd, *J* = 5.7, 8.7 Hz, 1H), 6.50 (br s, 1H), 3.40-3.36 (m, 2H), 2.68 (dt, *J* = 8.0, 7.8 Hz, 2H), 1.94-1.88 (m, 2H), 1.35 (s, 12H); **^{13}C NMR** (151 MHz, $CDCl_3$) δ 162.8 (dd, $^1J_{C-F}$ = 248 Hz, $^4J_{C-F}$ = 1 Hz), 157.3 (q, $^2J_{C-F}$ = 38 Hz), 156.8 (dd, $^1J_{C-F}$ = 241 Hz , $^4J_{C-F}$ = 2 Hz), 132.9 (dd, $^2J_{C-F}$ = 19 Hz, $^3J_{C-F}$ = 9 Hz), 122.2 (dd, $^2J_{C-F}$ = 24 Hz, $^3J_{C-F}$ = 9 Hz), 116.8 (dd, $^2J_{C-F}$ = 27 Hz, $^3J_{C-F}$ = 5 Hz), 115.8 (q, $^2J_{C-F}$ = 289 Hz), 84.2, 39.2, 28.8, 26.2, 24.7.

2,2,2-Trifluoro-*N*-(3-(2-methyl-5-(4,4,5,5-tetramethyl-1,3,2-dioxaborolan-2-yl)phenyl)propyl)acetamide (7i)

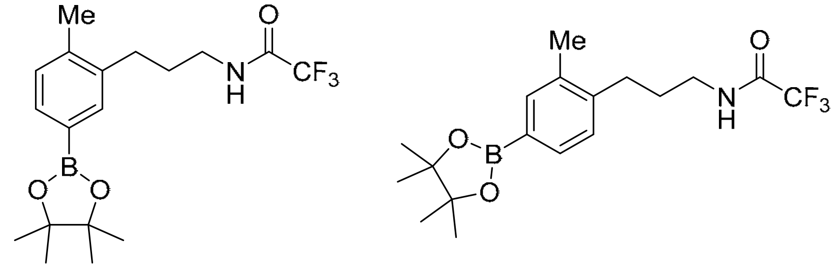

meta ***para***

1A in THF:

Following general procedure F using 2,2,2-trifluoro-N-(3-(o-tolyl)propyl)acetamide (61.3 mg, 0.25 mmol), B_2pin_2 (127 mg, 0.50 mmol), $[Ir(COD)OMe]_2$ (2.5 mg, 0.00375 mmol) and **1A** (3.8 mg, 0.0075 mmol) in THF (1.25 mL). The reaction was stirred at 50 °C for 20 hours before cooling and the solvents removed. Analysis of crude ^{1}H NMR using internal standard 1,2-dimethoxyethane showed 13.0:1 *meta*:*para* borylation in 86% yield. The crude product was purified by silica gel chromatography (Pet. Ether (40-60):EtOAc (91:1-8:1) to give the title compound (as a 10.5:1 mixture of *meta*:*para* ratio, as determined by ^{1}H NMR) as a white solid (74.7 mg, 0.20 mmol, 81%).

Meta ^{1}H NMR (500 MHz, $CDCl_3$) δ 7.58 (d, *J* = 7.3 Hz, 1H), 7.55 (s, 1H), 7.16 (d, *J* = 7.4 Hz, 1H), 6.41 (br s, 1H), 3.42 (dt, *J* = 6.6, 6.8 Hz, 2H), 2.68 (app t, 7.6 Hz, 2H), 2.32 (s, 3H), 1.89 (tt, *J* = 7.4, 7.6 Hz, 2H), 1.34 (s, 12H); **^{13}C NMR** (126 MHz, $CDCl_3$) δ 157.2 (q, $^2J_{C\text{-}F}$ = 37 Hz), 139.3, 138.2, 135.1, 133.0, 130.0, 115.8 (q, $^1J_{C\text{-}F}$ = 287 Hz), 83.7, 39.9, 30.4, 29.3, 24.8, 19.5; **HRMS** m/z: $[M + H]^+$ calc'd for $[C_{18}H_{26}BF_3NO_3]^+$ expect 372.1958; found 372.1959.

Dtbpy in THF:

Following general procedure F using 2,2,2-trifluoro-N-(3-(o-tolyl)propyl)acetamide (61.3mg, 0.25 mmol), B_2pin_2 (127 mg, 0.50 mmol), $[Ir(COD)OMe]_2$ (2.5 mg, 0.00375 mmol) and dtbpy (2.0 mg, 0.0075 mmol) in THF (1.25 mL). The reaction was stirred at 50 °C for 20 hours before cooling and the solvents removed. Analysis of crude ^{1}H NMR using internal standard 1,2-dimethoxyethane showed 1.6:1 *meta*:*para* borylation in 54% yield. The crude product was purified by silica gel chromatography (Pet. Ether (40-60):EtOAc (9:1:8:1) to give the title compound (as a 1.5:1 mixture of *meta*:*para* ratio, as determined by ^{1}H NMR) as a colourless oil (46.0 mg, 0.12 mmol, 50%). By subtracting the resonances for the *meta* isomer, the NMR data of the *para* isomer can be assigned as follows:

Para ^{1}H NMR (600 MHz, $CDCl_3$) δ 7.61 (s, 1H), 7.59 (d, *J* = 7.4 Hz, 1H), 7.13 (d, *J* = 7.4 Hz, 1H), 6.31 (br s, 1H), 3.44-3.40 (m, 2H), 2.68 (app t, 7.4 Hz, 2H), 2.31 (s, 3H), 1.88 (tt, *J* = 7.4, 7.6 Hz, 2H), 1.34 (s, 12H); **^{13}C NMR** (151 MHz, $CDCl_3$) δ 157.2 (q, $^2J_{C\text{-}F}$ = 37 Hz), 142.2, 136.9, 135.1, 132.7, 128.2, 115.8 (q, $^1J_{C\text{-}F}$ = 287 Hz), 83.7, 39.8, 30.6, 29.1, 24.8, 19.0.

2,2,2-Trifluoro-*N*-(3-(2-methoxy-5-(4,4,5,5-tetramethyl-1,3,2-dioxaborolan-2-yl)phenyl)propyl)acetamide (7j)

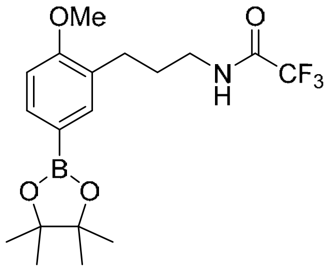

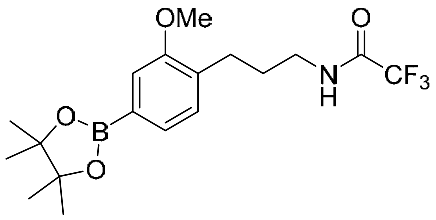

meta ***para***

1a in THF:

Following general procedure F using 2,2,2-trifluoro-N-(3-(2-methoxyphenyl)propyl)acetamide (65.3 mg, 0.25 mmol), B_2pin_2 (127 mg, 0.50 mmol), $[Ir(COD)OMe]_2$ (2.5 mg, 0.00375 mmol) and **1a** (3.8 mg, 0.0075 mmol) in THF (1.25 mL). The reaction was stirred at 70 °C for 20 hours before cooling and the solvents removed. Analysis of crude ^{1}H NMR using internal standard 1,2-dimethoxyethane showed 1.3:1 *meta*:*para* borylation in 65% yield. The crude product was purified by silica gel chromatography (Pet. Ether (40-60):EtOAc (19:1-9:1) to give the title compound (as a 1.3:1 mixture of *meta*:*para* ratio, as determined by ^{1}H NMR) as a colourless oil (61.7 mg, 0.16 mmol, 64%).

Meta ^{1}H NMR (600 MHz, $CDCl_3$) δ 7.69 (dd, *J* = 1.6, 8.1 Hz, 1H), 7.58 (d, *J* = 1.6 Hz, 1H), 6.88 (d, *J* = 8.1 Hz, 1H), 6.79 (br s, 1H), 3.87 (s, 3H), 3.30 (quint, *J* = 6.5 Hz, 2H), 2.71 (t, 7.3 Hz, 2H), 1.88 (dt, *J* = 7.0, 6.8 Hz, 2H), 1.34 (s, 12H); **^{13}C NMR** (151 MHz, $CDCl_3$) δ 159.8, 157.0 (q, $^2J_{C\text{-}F}$ = 37 Hz), 136.8, 135.0, 128.1, 116.0 (q, $^1J_{C\text{-}F}$ = 288 Hz), 109.9, 83.6, 55.4, 38.8, 29.1, 26.4, 24.8; **HRMS** m/z: $[M]^+$ calc'd for $[C_{16}H_{20}{}^{10}BNO_4F_3]^+$ expect 388.1907; found 388.1915.

Para ^{1}H NMR (600 MHz, $CDCl_3$) δ 7.40 (d, *J* = 7.4 Hz, 1H), 7.30 (s, 1H), 7.15 (d, *J* = 7.4 Hz, 1H), 6.89 (br s, 1H), 3.90 (s, 3H), 3.29 (quint, *J* = 6.5 Hz, 2H), 2.74 (t, 7.3 Hz, 2H), 1.87 (dt, *J* = 7.0, 6.8 Hz, 2H), 1.35 (s, 12H); **^{13}C NMR** (151 MHz, $CDCl_3$) δ 156.9, 157.0 (q, $^2J_{C\text{-}F}$ = 37 Hz), 132.3, 129.7, 127.9, 116.1, 115.9 (q, $^1J_{C\text{-}F}$ = 288 Hz), 83.8, 55.5, 38.5, 29.1, 26.5, 24.8.

dtbpy in THF:

Following general procedure F using 2,2,2-trifluoro-N-(3-(2-methoxyphenyl)propyl)acetamide (65.3 mg, 0.25 mmol), B_2pin_2 (127 mg, 0.50 mmol), $[Ir(COD)OMe]_2$ (2.5 mg, 0.00375 mmol) and dtbpy (2.0 mg, 0.0075 mmol) in THF (1.25 mL). The reaction was stirred at 70 °C for 20 hours before cooling and the solvents removed. Analysis of crude ^{1}H NMR using internal standard 1,2-dimethoxyethane showed 1:2.0 *meta*:*para* borylation in 91% yield.

2,2,2-Trifluoro-*N*-(3-(4'-methyl-4-(4,4,5,5-tetramethyl-1,3,2-dioxaborolan-2-yl)-[1,1'-biphenyl]-2-yl)propyl)acetamide (7k)

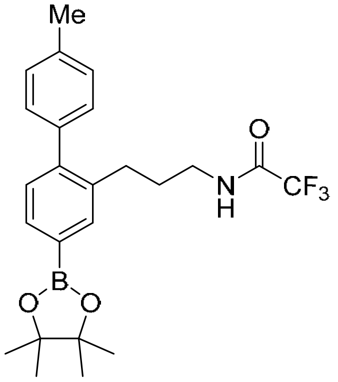

meta

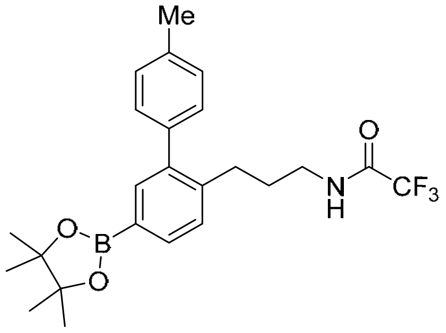

para

1a in THF:

Following general procedure F using 2,2,2-trifluoro-*N*-(3-(4'-methyl-[1,1'-biphenyl]-2-yl)propyl)acetamide (80.3 mg, 0.25 mmol), B_2pin_2 (127 mg, 0.50 mmol), $[Ir(COD)OMe]_2$ (2.5 mg, 0.00375 mmol) and **1a** (3.8 mg, 0.0075 mmol) in THF (1.25 mL). The reaction was stirred at 50 °C for 20 hours before cooling and the solvents removed. Analysis of crude ^{1}H NMR using internal standard 1,2-dimethoxyethane showed 10.4:1 *meta*:*para* borylation in 99% yield. The crude product was purified by silica gel chromatography (Pet. Ether (40-60):EtOAc (19:1-9:1) to give the title compound (as a 9.5:1 mixture of *meta*:*para* ratio, as determined by ^{1}H NMR) as a white solid (102.4 mg, 0.23 mmol, 92%).

Meta ^{1}H NMR (600 MHz, $CDCl_3$) δ 7.74 (s, 1H), 7.73 (d, *J* = 7.6 Hz, 1H), 7.26-7.25 (m, 3H), 7.20 (d, *J* = 7.9 Hz, 2H), 5.87 (br s, 1H), 3.14 (dt, *J* = 6.3, 6.4 Hz, 2H), 2.73 (t, *J* = 7.6 Hz, 2H), 2.42 (s, 3H), 1.72 (tt, *J* 6.8, 7.4 Hz, 2H), 1.38 (s, 12H); **^{13}C NMR** (151 MHz, $CDCl_3$) δ 157.1 (q, $^2J_{C\text{-}F}$ = 37 Hz), 144.8, 138.3, 137.6, 137.2, 135.9, 132.7, 129.8, 129.1, 128.9, 115.7 (q, $^1J_{C\text{-}F}$ = 288 Hz), 83.9, 39.0, 30.4, 29.5, 24.9, 21.1; **HRMS** m/z: $[M]^+$ calc'd for $[C_{24}H_{29}BF_3NO_3]^+$ expect 466.2229; found 446.2217.

Dtbpy in THF:

Following general procedure F using 2,2,2-trifluoro-*N*-(3-(4'-methyl-[1,1'-biphenyl]-2-yl)propyl)acetamide (80.3 mg, 0.25 mmol), B_2pin_2 (127 mg, 0.50 mmol), $[Ir(COD)OMe]_2$ (2.5 mg, 0.00375 mmol) and dtbpy (2.0 mg, 0.0075 mmol) in THF (1.25 mL). The reaction was stirred at 50 °C for 20 hours before cooling and the solvents removed. Analysis of crude ^{1}H NMR using internal standard 1,2-dimethoxyethane showed 1:1 *meta*:*para* borylation in 100% yield. The crude product was purified by silica gel chromatography (Pet. Ether (40-60):EtOAc (19:1-9:1) to give the title compound (as a 1:1.1 mixture of *meta*:*para* ratio, as determined by ^{1}H NMR) as a colourless oil (95.0 mg, 0.21 mmol, 85%). By subtracting the resonances for the *meta* isomer, the NMR data of the *para* isomer can be assigned as follows:

Para ^{1}H NMR (500 MHz, $CDCl_3$) δ 7.74 (dd, *J* = 0.7, 7.4 Hz, 1H), 7.68 (s, 1H), 7.28 (d, *J* = 7.4 Hz, 1H), 7.23-7.18 (2 x d, *J* = ~7.9 Hz, 2 x 2H), 5.78 (br s, 1H), 3.14 (dt, *J* = 6.3, 6.4 Hz, 2H), 2.71 (t, *J* = 7.6 Hz, 2H), 2.40 (s, 3H), 1.70 (tt, *J* 6.8, 7.4 Hz, 2H), 1.34 (s, 12H); **^{13}C NMR** (126 MHz, $CDCl_3$) δ 157.1 (q, $^2J_{C\text{-}F}$ = 37 Hz), 141.5, 141.3, 138.3, 136.9, 136.7, 134.0, 129.1, 129.0, 128.8, 115.7 (q, $^1J_{C\text{-}F}$ = 288 Hz), 83.8, 38.9, 30.2, 29.9, 24.9, 21.1.

2,2,2-Trifluoro-*N*-(3-(4-(4,4,5,5-tetramethyl-1,3,2-dioxaborolan-2-yl)pyridin-2-yl)propyl)acetamide (7l)

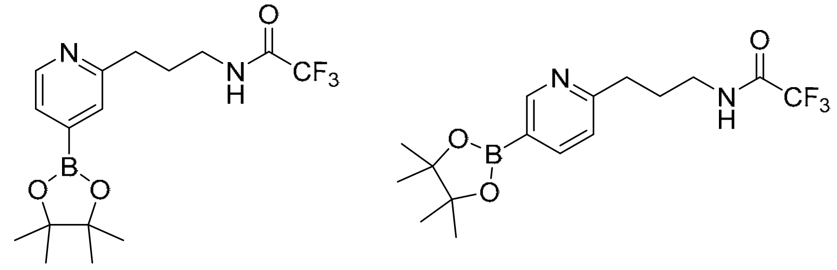

meta ***para***

1a in THF:

Following general procedure F using 2,2,2-trifluoro-*N*-(3-(pyridin-2-yl)propyl)acetamide (58.1 mg, 0.25 mmol), B_2pin_2 (127 mg, 0.50 mmol), $[Ir(COD)OMe]_2$ (2.5 mg, 0.00375 mmol) and **1a** (3.8 mg, 0.0075 mmol) in THF (1.25 mL). The reaction was stirred at rt for 15 hours before cooling and the solvents removed. Analysis of crude ^{1}H NMR using internal standard 1,2-dimethoxyethane showed 7.8:1 *meta*:*para* borylation in 71% yield. The crude product was unable to be purified by silica gel chromatography and was hence characterised from the crude mixture.

Meta ^{1}H NMR (500 MHz, $CDCl_3$) δ 8.67 (br s, 1H), 8.43 (dd, *J* = 0.7, 5.0 Hz, 1H), 7.48 (br s, 1H) 7.45 (dd, *J* = 0.6, 4.8 Hz, 1H), 3.36-3.30 (m, 2H), 2.84 (t, *J* = 6.9 Hz, 2H), 1.96 (tt, *J* = 6.2, 6.6 Hz, 2H) 1.28 (s, 12H); **^{13}C NMR** (126 MHz, $CDCl_3$) δ 159.7, 157.4 (q, $^2J_{C-F}$ = 30 Hz), 147.6, 128.5, 126.6, 115.9 (q, $^1J_{C-F}$ = 287 Hz), 84.6, 39.7, 34.8, 27.6, 24.7; **HRMS** m/z: $[M + H]^+$ calc'd for $[C_{16}H_{23}BN_2O_3F_3]^+$ expect 359.1574; found 359.1579.

Dtbpy in THF:

Following general procedure F using 2,2,2-trifluoro-*N*-(3-(pyridin-2-yl)propyl)acetamide (58.1 mg, 0.25 mmol), B_2pin_2 (127 mg, 0.50 mmol), $[Ir(COD)OMe]_2$ (2.5 mg, 0.00375 mmol) and dtbpy (2.0 mg, 0.0075 mmol) in THF (1.25 mL). The reaction was stirred at rt for 22 hours before cooling and the solvents removed. Analysis of crude ^{1}H NMR using internal standard 1,2-dimethoxyethane showed 2.3:1 *meta*:*para* borylation in 70% yield. The crude product was unable to be purified by silica gel chromatography and was hence characterised from the crude mixture.

By subtracting the resonances for the *meta* isomer, the NMR data of the *para* isomer can be assigned as follows:

Para ^{1}H NMR (400 MHz, $CDCl_3$) δ 9.10 (br s, 1H), 8.80 (s, 1H), 7.98 (dd, *J* = 1.1, 7.7 Hz, 1H), 7.16 (d, *J* = 7.7 Hz, 1H), 3.40-3.56 (m, 2H), 2.93-2.90 (m, 2H), 2.00 (tt, *J* = 5.9, 6.0 Hz, 2H), 1.32 (s, 12H); **^{13}C NMR** (100 MHz, $CDCl_3$) δ 163.0, 157.4 (q, $^2J_{C-F}$ = 30 Hz), 154.3, 143.6, 122.6, 115.9 (q, $^1J_{C-F}$ = 287 Hz), 84.3, 40.0, 35.5, 27.0, 24.8.

Borylation of Longer Chain Substrates

_N_-(4-(3,5-bis(4,4,5,5-tetramethyl-1,3,2-dioxaborolan-2-yl)phenyl)butyl)-2,2,2-trifluoroacetamide (8)

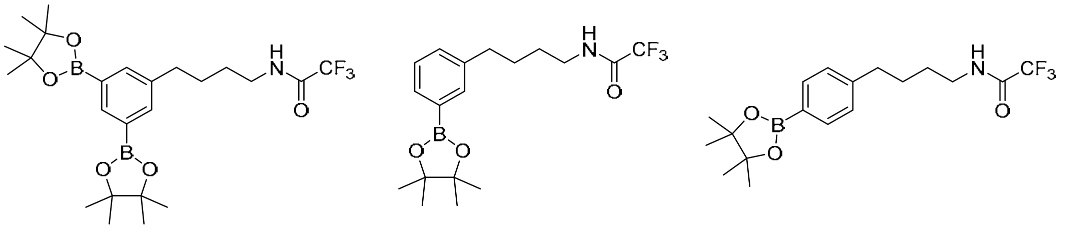

dimeta ***meta*** ***para***

1a in THF:

Following general procedure F using 2,2,2-trifluoro-*N*-(4-phenylbutyl)acetamide (61.3 mg, 0.25 mmol), B_2pin_2 (381 mg, 1.50 mmol), $[Ir(COD)OMe]_2$ (5.0 mg, 0.0075 mmol) and **1a** (7.6 mg, 0.015 mmol) in THF (1.25 mL). The reaction was stirred at 50 °C for 20 hours before cooling and the solvents removed. Analysis of crude ^1H NMR using internal standard 1,2-dimethoxyethane showed 22:49:12 *dimeta*:*meta*:*para* borylation in 83% yield. The crude product was purified by silica gel chromatography (Pet. Ether (40-60):EtOAc (9:1) to give a clean fraction of the *dimeta* compound as a white solid (22.1 mg, 0.04 mmol, 18%), and a mixed fraction (as a 4:73:18 *dimeta*:*meta*:*para* ratio, as determined by ^1H NMR) as a colourless oil (68.7 mg, 0.19 mmol, 74%). This gave a combined yield of 92%. (Ratio is different to value in manuscript due to increased B_2pin_2 used in scale up for characterisation).

Dimeta ^1H NMR (500 MHz, $CDCl_3$) δ 8.13 (t, *J* = 1.0 Hz, 1H), 7.70 (d, *J* = 1.0 Hz, 2H), 6.26 (br s, 1H), 3.36 (dt, *J* = 6.6, 7.0 Hz, 2H), 2.66 (t, *J* = 7.5 Hz, 2H), 1.73-1.67 (m, 2H), 1.63-1.57 (m, 2H), 1.34 (s, 24H); **^{13}C NMR** (126 MHz, $CDCl_3$) δ 157.1 (q, $^2J_{C\text{-}F}$ = 36 Hz), 140.1, 139.1, 137.6, 155.8 (q, $^1J_{C\text{-}F}$ = 289 Hz), 83.8, 39.9, 35.2, 28.6, 28.5, 24.9; **HRMS** m/z: $[M + H]^+$ calc'd for $[C_{24}H_{37}B_2NO_5F_3]^+$ expect 498.2810; found 498.2813.

Dtbpy in THF:

Following general procedure F using 2,2,2-trifluoro-*N*-(3-phenylpropyl)acetamide (57.8 mg, 0.25 mmol), B_2pin_2 (274 mg, 1.0 mmol), $[Ir(COD)OMe]_2$ (2.5 mg, 0.00375 mmol) and dtbpy (2.0 mg, 0.0075 mmol) in THF (1.25 mL). The reaction was stirred at 50 °C for 20 hours before cooling and the solvents removed. Analysis of crude ^1H NMR using internal standard 1,2-dimethoxyethane showed 43:27:31 *dimeta*:*meta*:*para* borylation in 100% yield. The crude product was purified by silica gel chromatography (Pet. Ether (40-60):EtOAc (9:1) to give a fraction of the mono borylated products to aid characterisation (as a 1:1:0.2 mixture of *meta*:*para* ratio, as determined by ^1H NMR) as a colourless oil (45 mg), the NMR yield was used for direct comparison. By using a combination of 2D NMR analysis the NMR data of the *meta* and *para* isomers can be assigned as follows:

Meta ^1H NMR (500 MHz, $CDCl_3$) δ 7.65 (d, *J* = 7.0 Hz, 1H), 7.61 (s, 1H), 7.29 (t, *J* = 7.3 Hz, 1H), 7.27 (dt, *J* = 7.6, 1.5 Hz), 6.35 (br s, 1H), 3.36 (dt, *J* = 6.2, 7.9 Hz, 2H), 2.65 (t, *J* = 7.6 Hz, 2H), 1.71-1.64 (m, 2H), 1.63-1.16 (m, 2H), 1.35 (s, 12H); **^{13}C NMR** (126 MHz, $CDCl_3$) δ 157.2 (q, $^2J_{C\text{-}F}$ = 36 Hz), 140.9, 134.6, 132.5, 131.4, 127.9, 115.8 (q, $^1J_{C\text{-}F}$ = 287 Hz), 83.8, 39.8, 35.2, 28.5, 28.4, 24.8.

Para ^1H NMR (500 MHz, $CDCl_3$) δ 7.74 (d, *J* = 7.9 Hz, 2H), 7.18 (d, *J* = 7.9 Hz, 2H), 6.35 (br s, 1H), 3.36 (dt, *J* = 6.2, 7.9 Hz, 2H), 2.65 (t, *J* = 7.6 Hz, 2H), 1.71-1.64 (m, 2H), 1.63-1.16 (m, 2H), 1.34 (s, 12H); **^{13}C**

NMR (126 MHz, $CDCl_3$) δ 157.2 (q, $^2J_{C-F}$ = 36 Hz), 145.0, 135.0, 127.8, 115.8 (q, $^1J_{C-F}$ = 287 Hz), 83.7, 39.8, 35.4, 28.4, 28.1, 24.8.

N-(5-(3,5-bis(4,4,5,5-tetramethyl-1,3,2-dioxaborolan-2-yl)phenyl)pentyl)-2,2,2-trifluoroacetamide (9)

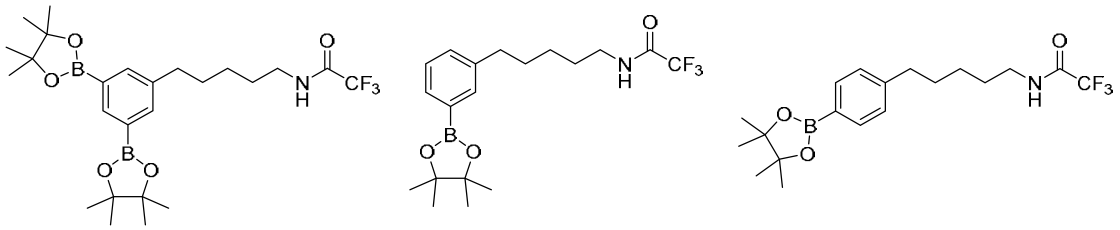

1b in THF:

Following general procedure F using 2,2,2-trifluoro-*N*-(5-phenylpentyl)acetamide (38.9 mg, 0.15 mmol), B_2pin_2 (229 mg, 0.90 mmol), $[Ir(COD)OMe]_2$ (3.0 mg, 0.0045 mmol) and **1b** (4.8 mg, 0.009 mmol) in THF (0.75 mL). The reaction was stirred at 50 °C for 20 hours before cooling and the solvents removed. Analysis of crude ^{1}H NMR using internal standard 1,2-dimethoxyethane showed 56:27:13 *dimeta*:*meta*:*para* borylation in 96% yield. The crude product was purified by silica gel chromatography (Pet. Ether (40-60):EtOAc (9:1) to give a clean fraction of the title compound as a white solid (27.9 mg, 0.05 mmol, 36%), and a mixed fraction (as a 37:38:22 *dimeta*:*meta*:*para* ratio, as determined by ^{1}H NMR) as a colourless oil (32.6 mg, 0.075 mmol, 50%*). This gave a combined yield of 86%. (Ratio is different to value in manuscript due to increased B_2pin_2 used in scale up for characterisation).

Dimeta ^{1}H NMR (500 MHz, $CDCl_3$) δ 8.12 (br s, 1H), 7.70 (d, *J* = 1.0 Hz, 2H), 6.29 (br s, 1H), 3.35 (dt, *J* = 6.6, 6.9 Hz, 2H), 2.62 (app t, *J* = 7.9 Hz, 2H), 1.70-1.64 (m, 2H), 1.63-1.57 (m, 2H), 1.40-1.34 (m, 2H), 1.34 (s, 24H); ^{13}**C NMR** (126 MHz, $CDCl_3$) δ 157.1 (q, $^2J_{C-F}$ = 36 Hz), 140.6, 139.0, 137.6, 155.9 (q, $^1J_{C-F}$ = 284 Hz), 83.7, 39.9, 35.6, 31.0, 28.8, 26.4, 24.9; **HRMS** m/z: $[M + H]^+$ calc'd for $[C_{25}H_{39}B_2NO_5F_3]^+$ expect 512.2966; found 512.2962.

*Yield calculated from a weighted average mass of mono and diborylated products.

Dtbpy in THF:

Following general procedure F using 2,2,2-trifluoro-*N*-(3-phenylpropyl)acetamide (39.8 mg, 0.15 mmol), B_2pin_2 (164 mg, 0.6 mmol), $[Ir(COD)OMe]_2$ (1.5 mg, 0.00225 mmol) and dtbpy (1.2 mg, 0.0045 mmol) in THF (1.25 mL). The reaction was stirred at 50 °C for 20 hours before cooling and the solvents removed. Analysis of crude ^{1}H NMR using internal standard 1,2-dimethoxyethane showed 7:41:20 *dimeta*:*meta*:*para* borylation in 67% yield. The crude product was purified by silica gel chromatography (Pet. Ether (40-60):EtOAc (9:1) to give a fraction of the mono borylated products to aid characterisation (as a 2:1 mixture of *meta*:*para* ratio, as determined by ^{1}H NMR) as a colourless oil (25.5 mg, 0.07 mmol, 44%), the NMR yield was used for direct comparison. By using a combination of 2D NMR analysis the NMR data of the *meta* and *para* isomers can be assigned as follows:

Meta ^{1}H NMR (500 MHz, $CDCl_3$) δ 7.64 (dt, *J* = 7.0, 1.3 Hz, 1H), 7.61 (s, 1H), 7.29 (t, *J* = 7.2 Hz, 1H), 7.26 (dt, *J* = 7.6, 1.6 Hz), 6.34 (br s, 1H), 3.35 (dt, *J* = 6.2, 6.9 Hz, 2H), 2.62 (t, *J* = 7.6 Hz, 2H), 1.69-1.57 (m, 4H), 1.40-1.34 (m, 2H), 1.35 (s, 12H); ^{13}**C NMR** (126 MHz, $CDCl_3$) δ 157.2 (q, $^2J_{C-F}$ = 37 Hz), 141.4, 134.7, 132.3, 131.4, 127.9, 115.9 (q, $^1J_{C-F}$ = 288 Hz), 83.8, 39.9, 35.6, 31.0, 28.8, 26.3, 24.9.

Para ^{1}H NMR (500 MHz, $CDCl_3$) δ 7.73 (d, *J* = 7.9 Hz, 2H), 7.18 (d, *J* = 7.9 Hz, 2H), 6.34 (br s, 1H), 3.33 (dt, *J* = 5.5, 6.6 Hz, 2H), 2.63 (t, *J* = 7.7 Hz, 2H), 1.69-1.57 (m, 4H), 1.40-1.34 (m, 2H), 1.34 (s, 12H); ^{13}**C**

NMR (126 MHz, $CDCl_3$) δ 157.2 (q, $^2J_{C-F}$ = 37 Hz), 145.6, 134.9, 127.8, 115.9 (q, $^1J_{C-F}$ = 288 Hz), 83.7, 39.9, 35.8, 30.7, 28.8, 26.2, 24.9.

_N_-(6-(3,5-bis(4,4,5,5-tetramethyl-1,3,2-dioxaborolan-2-yl)phenyl)hexyl)-2,2,2-trifluoroacetamide (10)

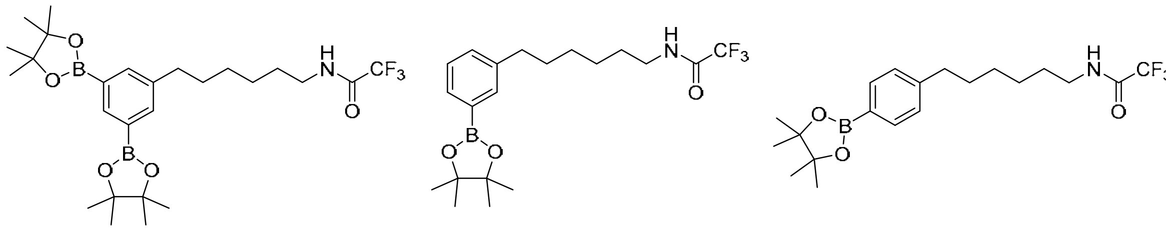

1b in THF:

Following general procedure F using 2,2,2-trifluoro-*N*-(4-phenylbutyl)acetamide (68.3 mg, 0.25 mmol), B_2pin_2 (381 mg, 1.50 mmol), $[Ir(COD)OMe]_2$ (5.0 mg, 0.0075 mmol) and **1b** (8.0 mg, 0.015 mmol) in THF (1.25 mL). The reaction was stirred at 50 °C for 20 hours before cooling and the solvents removed. Analysis of crude ^{1}H NMR using internal standard 1,2-dimethoxyethane showed 66:20:14 *dimeta*:*meta*:*para* borylation in 99% yield . The crude product was purified by silica gel chromatography (Pet. Ether (40-60):EtOAc (9:1) to give a clean fraction of the title compound as a white solid (54.3 mg, 0.10 mmol, 41%), and a mixed fraction (as a 35:33:22:11 *dimeta*:*meta*:*para*:B_2pin_2 ratio, as determined by ^{1}H NMR) as a colourless oil (59.5 mg, 0.12 mmol, 50%*). This gave a combined yield of 91%. (Ratio is different to value in manuscript due to increased B_2pin_2 used in scale up for characterisation).

Dimeta ^{1}H NMR (500 MHz, $CDCl_3$) δ 8.11 (s, 1H), 7.70 (d, *J* = 1.0 Hz, 2H), 6.35 (br s, 1H), 3.34 (dt, *J* = 6.8, 6.7 Hz, 2H), 2.60 (app t, *J* = 7.8 Hz, 2H), 1.65-1.54 (m, 4H), 1.38-1.34 (m, 4H), 1.34 (s, 24H); **^{13}C NMR** (126 MHz, $CDCl_3$) δ 157.1 (q, $^2J_{C-F}$ = 37 Hz), 141.1, 138.9, 137.6, 155.9 (q, $^1J_{C-F}$ = 288 Hz), 83.7, 40.0, 35.6, 31.4, 28.9, 28.8, 26.5, 24.9; **HRMS** m/z: $[M + H]^+$ calc'd for $[C_{26}H_{14}B_2NO_5F_3]^+$ expect 526.3123; found 526.3134.

*Yield calculated from a weighted average mass of B_2pin_2, mono and diborylated products.

Dtbpy in THF:

Following general procedure F using 2,2,2-trifluoro-*N*-(3-phenylpropyl)acetamide (57.8 mg, 0.25 mmol), B_2pin_2 (274 mg, 1.0 mmol), $[Ir(COD)OMe]_2$ (2.5 mg, 0.00375 mmol) and dtbpy (2.0 mg, 0.0075 mmol) in THF (1.25 mL). The reaction was stirred at 50 °C for 20 hours before cooling and the solvents removed. Analysis of crude ^{1}H NMR using internal standard 1,2-dimethoxyethane showed 31:37:39 *dimeta*:*meta*:*para* borylation in 97% yield. The crude product was purified by silica gel chromatography (Pet. Ether (40-60):EtOAc (9:1) to give a fraction of the mono borylated products to aid characterisation (as a 2:1 mixture of *meta*:*para* ratio, as determined by ^{1}H NMR) as a colourless oil (27.0 mg, 0.07 mmol, 27%) the NMR yield was used for direct comparison. By using a combination of 2D NMR analysis the NMR data of the *meta* and *para* isomers can be assigned as follows:

Meta ^{1}H NMR (500 MHz, $CDCl_3$) δ 7.63 (dt, *J* = 6.7, 1.2 Hz, 1H), 7.62(s, 1H), 7.29 (t, *J* = 7.5 Hz, 1H), 7.27 (dt, *J* = 7.7, 1.6 Hz), 6.34 (br s, 1H), 3.34 (dt, *J* = 6.9, 6.7 Hz, 2H), 2.61 (t, *J* = 7.8 Hz, 2H), 1.66-1.54 (m, 4H), 1.37-1.34 (m, 4H), 1.35 (s, 12H); **^{13}C NMR** (126 MHz, $CDCl_3$) δ 157.2 (q, $^2J_{C-F}$ = 37 Hz), 141.7, 134.7, 132.2, 131.4, 127.9, 115.9 (q, $^1J_{C-F}$ = 288 Hz), 83.8, 40.0, 35.7, 31.3, 28.9, 28.8, 26.5, 24.9.

Para ^{1}H NMR (500 MHz, $CDCl_3$) δ 7.73 (d, *J* = 8.0 Hz, 2H), 7.18 (d, *J* = 8.0 Hz, 2H), 6.34 (br s, 1H), 3.36-3.32 (m, 2H), 2.62 (t, *J* = 7.5 Hz, 2H), 1.66-1.54 (m, 4H), 1.37-1.34 (m, 4H), 1.34 (s, 12H); **^{13}C NMR** (126

MHz, $CDCl_3$) δ 157.2 (q, $^{2}J_{C-F}$ = 37 Hz), 145.9, 134.9, 127.7, 115.9 (q, $^{1}J_{C-F}$ = 288 Hz), 83.7, 40.0, 36.0, 31.0, 28.9, 28.7, 26.5, 24.9.

Analysis of sulfonate 1a, 1b, 1i and 1j ligands on longer chain substrates - 7b, 8, 9 and 10:

n=3 (7b):

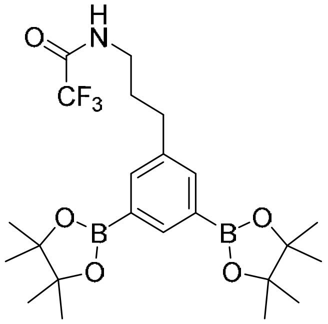

Ligand	*dimeta*	*meta*	*para*	NMR yield / %	*m/p*
**1a**	29.2	32	4.2	65	14.6
**1b**	38.6	38.2	13.6	90	5.6
**1i**	18.2	49.3	20.0	88	3.4
**1j**	25.1	38.6	27.1	91	2.4

n=4 (8):

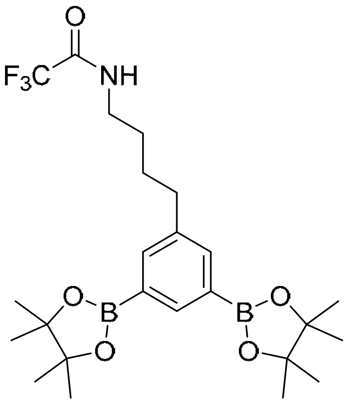

Ligand	*dimeta*	*meta*	*Para*	NMR yield / %	*m/p*
**1a**	13.8	48.9	8.4	71	7.5
**1b**	30.8	42.0	27.2	100	5.0
**1i**	12.7	54.0	23.7	88	2.8
**1j**	31.3	42.5	27.7	91	2.7

n=5 (9):

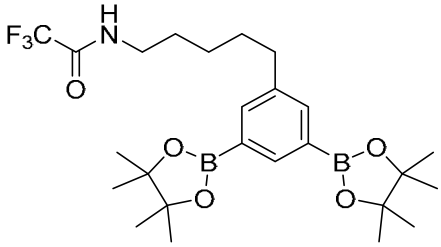

Ligand	*dimeta*	*meta*	*Para*	NMR yield / %	*m/p*
**1a**	3.6	32.8	6.4	43	5.7
**1b**	19.5	45.8	9.8	75	6.7
**1i**	32.0	40.9	21.0	94	3.5
**1j**	33.5	37.1	22.2	93	3.2

n=6 (10):

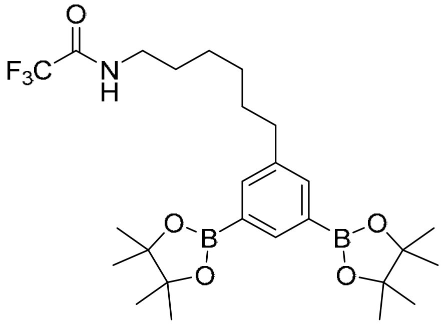

Ligand	*dimeta*	*meta*	*Para*	NMR yield / %	*m/p*
**1a**	3.8	34.7	8.9	47	4.3
**1b**	58.7	26.6	14.9	100	5.7
**1i**	23.2	51.9	23.5	99	3.2
**1j**	33.0	45.6	21.7	100	3.6

Borylation of conformationally restricted substrates (Figure 3a)

N-(2,3-dihydro-1H-inden-1-yl)-2,2,2-trifluoroacetamide (11a)

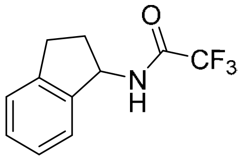

Following general procedure A using 2,3-dihydro-1H-inden-1-amine (240 mg, 1.8 mmol), stirred for 2 h and purified by washing with Pet. Ether (40-60) to yield a pale brown powder (354 mg, 1.5 mmol, 86%).

^{1}H NMR (600 MHz, $CDCl_3$) δ 7.32-7.25 (m, 4H), 6.55 (br s, 1H), 5.49 (q, J = 7.6 Hz, 1H), 3.06 (ddd, J = 4.3, 8.7, 16.0), 2.94 (dt, J = 16.0, 8.2 Hz, 1H), 2.69-2.63 (m, 1H), 1.96-1.90 (m, 1H); **^{13}C NMR** (151 MHz, $CDCl_3$) δ 157.1 (q, $^2J_{C\text{-}F}$ = 37 Hz), 143.5, 141.0, 128.7, 127.1, 125.0, 124.0, 115.9 (q, $^1J_{C\text{-}F}$ = 290 Hz), 55.2, 33.1; **HRMS** m/z: $[M + H]^+$ calc'd for $[C_{13}H_{17}F_3NO]^+$ expect 260.1262; found 260.1258.

2,2,2-Trifluoro-*N*-(6-(4,4,5,5-tetramethyl-1,3,2-dioxaborolan-2-yl)-2,3-dihydro-1H-inden-1-yl)acetamide (11)

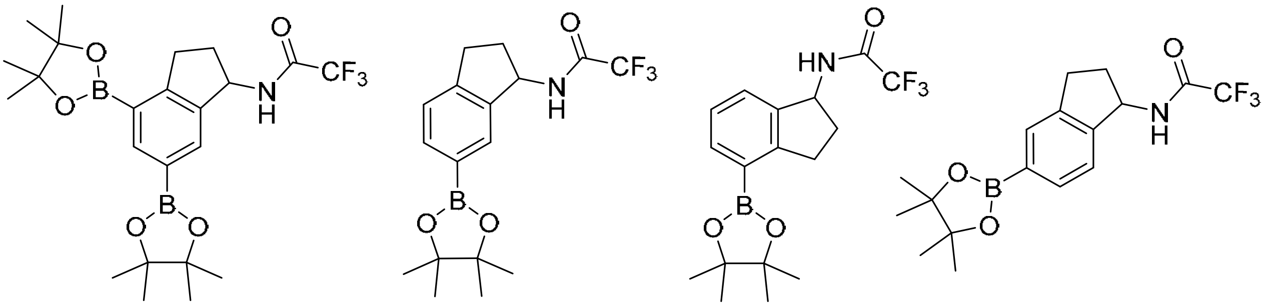

dimeta ***meta 1*** ***meta 2*** ***para***

1a in THF:

Following general procedure F using *N*-(2,3-dihydro-1H-inden-1-yl)-2,2,2-trifluoroacetamide (**11a**) (57.3 mg, 0.25 mmol), B_2pin_2 (95 mg, 0.375 mmol), $[Ir(COD)OMe]_2$ (2.5 mg, 0.00375 mmol) and **1a** (3.8 mg, 0.0075 mmol) in THF (1.25 mL). The reaction was stirred at 50 °C for 20 hours before cooling and the solvents removed. Analysis of crude ^{1}H NMR using internal standard 1,2-dimethoxyethane showed 11:18:20:30* *dimeta*:*meta*1:*meta*2:*para* borylation in 79% yield. The crude product was purified by silica gel chromatography (Pet. Ether (40-60):EtOAc (19:1-9:1) to give the title compound in 2 fractions: as pale brown oil containing the *meta* 2 product and SM in a 3:1 ratio, and as a colourless oil containing the *dimeta, meta 1* and *para* products in a 1:2:2.8 ratio. NMR yields have been used for clarity.

Dimeta ^{1}H NMR (600 MHz, $CDCl_3$) δ 8.22 (s, 1H), 7.80 (s, 1H), 6.45 (br d, J = 8.1 Hz, 1H), 5.43 (q, J = 7.4 Hz, 1H), 3.31 (ddd, J = 4.9, 8.9, 17.7 Hz, 1H), 3.09 (dt, J = 17.7, 8.0 Hz, 1H), 2.65-2.58 (m, 1H), 1.92-1.86 (m, 1H); 1.35 (s, 24H); **^{13}C NMR** (151 MHz, $CDCl_3$) δ 156.9 (q, $^2J_{C\text{-}F}$ = 37 Hz), 154.2, 142.6, 139.6, 132.9, 115.9 (q, $^1J_{C\text{-}F}$ = 288 Hz), 84.0, 83.7, 55.2, 33.3, 31.6, 24.9, 24.8.

Meta 1 ^{1}H NMR (500 MHz, $CDCl_3$) δ 7.76 (d, J = 7.6 Hz, 1H), 7.72 (s, 1H), 7.30 (d, J = 7.6 Hz, 1H), 6.50 (br d, J = 5.7 Hz, 1H), 5.46 (q, J = 7.6 Hz, 1H), 3.08-3.01 (m, 1H), 2.96-2.89 (m, 1H), 2.69-2.61 (m, 1H), 1.95-1.87 (m, 1H); 1.35 (s, 12 H); **^{13}C NMR** (126 MHz, $CDCl_3$) δ 157.0 (q, $^2J_{C\text{-}F}$ = 37 Hz), 147.2, 140.2, 135.5, 130.2, 124.6, 115.9 (q, $^1J_{C\text{-}F}$ = 288 Hz), 84.0, 55.2, 33.5, 30.4, 24.8; **HRMS** m/z: $[M + H]^+$ calc'd for $[C_{17}H_{22}BF_3NO_3]^+$ expect 356.1645; found 356.1630.

Meta 2 ^{1}H NMR (600 MHz, $CDCl_3$) δ 7.76 (d, *J* = 7.4 Hz, 1H), 7.39 (d, *J* = 7.4 Hz, 1H), 7.27 (t, *J* = 7.6 Hz, 1H), 6.40 (br d, *J* = 7.4 Hz, 1H), 5.49 (q, *J* = 7.5 Hz, 1H), 3.32 (ddd, *J* = 4.7, 8.8, 17.4 Hz, 1H), 3.09 (dt, *J* = 17.2, 8.4 Hz, 1H), 2.67-2.61 (m, 1H), 1.93-1.87 (m, 1H), 1.34 (s, 12H); **^{13}C NMR** (126 MHz, $CDCl_3$) δ 156.9 (q, $^2J_{C-F}$ = 37 Hz), 150.6, 140.4, 135.9, 126.8, 126.5, 115.9 (q, $^1J_{C-F}$ = 289 Hz), 83.7, 55.2, 33.2, 31.4, 25.0.

Para ^{1}H NMR (500 MHz, $CDCl_3$) δ 7.74 (s, 1H), 7.70 (d, *J* = 7.5 Hz, 1H), 7.29 (d, *J* = 7.5 Hz, 1H), 6.50 (br d, *J* = 5.7 Hz, 1H), 5.50 (q, *J* = 7.6 Hz, 1H), 3.08-3.01 (m, 1H), 2.96-2.89 (m, 1H), 2.69-2.61 (m, 1H), 1.95-1.87 (m, 1H); 1.35 (s, 12 H); **^{13}C NMR** (126 MHz, $CDCl_3$) δ 157.0 (q, $^2J_{C-F}$ = 37 Hz), 144.1, 142.8, 133.7, 131.4, 123.5, 115.9 (q, $^1J_{C-F}$ = 288 Hz), 84.0, 55.4, 33.3, 33.0, 24.9.

Dtbpy in THF:

Following general procedure F using *N*-(2,3-dihydro-1H-inden-1-yl)-2,2,2-trifluoroacetamide (57.3 mg, 0.25 mmol), B_2pin_2 (95 mg, 0.375 mmol), $[Ir(COD)OMe]_2$ (2.5 mg, 0.00375 mmol) and dtbpy (2.0 mg, 0.0075 mmol) in THF (1.25 mL). The reaction was stirred at 50 °C for 20 hours before cooling and the solvents removed. Analysis of crude ^{1}H NMR using internal standard 1,2-dimethoxyethane showed 29:16:6:33 *dimeta*:*meta*1:*meta*2:*para* borylation in 84% yield.

2,2,2-trifluoro-N-(2-phenylcyclohexyl)acetamide (12a)

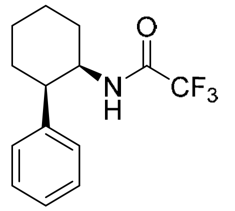

Cis-12a

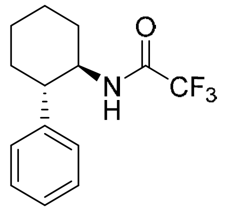

Trans-12a

2-Phenylcyclohexanone (1.31 g; 7.5 mmol; 1eq) was dissolved in a mixture of methanol (20 ml) and water (3 ml). Then ammonium formate (4.73 g; 75 mmol; 10eq) was added. Upon complete dissolution 10% Pd/C (0.3 g ;0.28 mmol) was added and the mixture was stirred at room temperature for 16 hours. The mixture was then filtered through celite, filter cake washed with methanol and the volatiles were removed *in vacuo*. The crude was treated with concentrated HCl_{aq} (4 ml) and water (30 ml) and extracted with diethyl ether (2x 30 ml). The aqueous was treated with 10% $NaOH_{aq}$ until pH basic and extracted with diethyl ether (3x 30 ml). Organics evaporated to give a yellowish oil which was retaken into DCM (30 ml), cooled to 0 °C and TFAA (1.25 ml; 9 mmol; 1.2eq) and triethylamine (2.1 ml; 15 mmol; 2eq) were added slowly. Stirred at room temperature for 2 hours. The mixture was washed with sat. $NaHCO_3$ (30 ml), the organic layer was dried with $MgSO_4$ and concentrated to give an orange oil which solidifies upon staying. The crude was then purified by column chromatography (SiO_2, 5% EtOAc in Petroleum Ether 40-60 °C) to give the *cis* isomer (1.18 g; 4.35 mmol) and the trans isomer (291 mg; 1.07 mmol) as white solids with overall yield of 72% over two steps.

Cis ^{1}H NMR (600 MHz, $CDCl_3$) δ 7.33 (t, J = 7.6 Hz, 2H), 7.24 (t, J = 7.4 Hz, 1H), 7.20 (d, J = 7.5 Hz, 2H), 6.26 (s, 1H), 4.41 (dd, J = 8.2, 3.8 Hz, 1H), 3.04 (dt, J = 12.4, 3.6 Hz, 1H), 2.12 – 2.03 (m, 1H), 2.00 – 1.90 (m, 2H), 1.81 – 1.66 (m, 3H), 1.56 – 1.46 (m, 1H), 1.46 – 1.36 (m, 1H); **^{13}C NMR** (151 MHz, $CDCl_3$) δ 156.4 (q, J = 36.5 Hz), 141.6, 128.6, 127.1, 127.0, 115.7 (q, J = 288.4 Hz), 50.8, 44.7, 30.2, 25.4, 25.2, 20.5. **HRMS** m/z: $[M + H]^+$ calc'd for $[C_{14}H_{17}F_3NO]^+$ expect 272.1257; found 272.1268.

Trans ^{1}H NMR (600 MHz, $CDCl_3$) δ 7.31 (dd, J = 10.4, 4.7 Hz, 2H), 7.23 (dd, J = 10.5, 4.3 Hz, 1H), 7.20 – 7.15 (m, 2H), 4.05 (qd, J = 11.6, 3.9 Hz, 1H), 2.51 (td, J = 11.7, 3.6 Hz, 1H), 2.25 – 2.15 (m, 1H), 2.02 – 1.95 (m, 1H), 1.94 – 1.81 (m, 2H), 1.61 (ddd, J = 25.9, 13.1, 3.5 Hz, 1H), 1.56 – 1.47 (m, 1H), 1.44 – 1.32 (m, 2H); **^{13}C NMR** (151 MHz, $CDCl_3$) δ 156.3 (q, J = 36.6 Hz), 142.2, 128.7, 127.2, 127.0, 115.6 (q, J =

288.2 Hz), 53.6, 50.2, 34.6, 33.0, 25.9, 25.1. **HRMS** m/z: $[M + H]^+$ calc'd for $[C_{14}H_{17}F_3NO]^+$ expect 272.1257; found 272.1267.

(cis)-2,2,2-trifluoro-N-(2-(3-(4,4,5,5-tetramethyl-1,3,2-dioxaborolan-2-yl)phenyl)cyclohexyl)acetamide (cis-12)

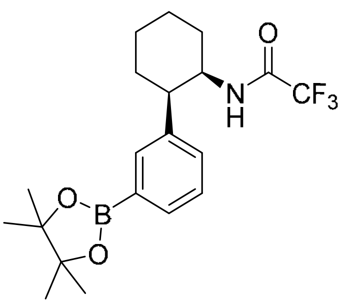

Meta

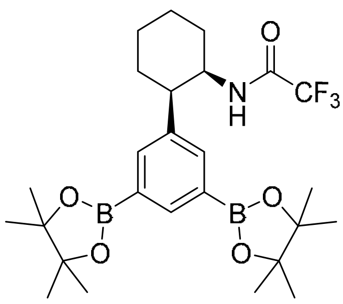

Dimeta

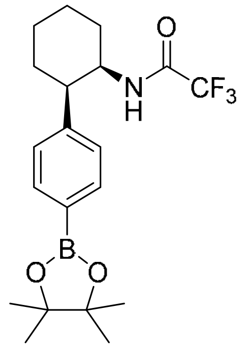

Para

1a in THF:

Following general procedure F using *cis*-**12a** (67.8 mg, 0.25 mmol), B_2pin_2 (127 mg, 0.50 mmol), $[Ir(COD)OMe]_2$ (2.5 mg, 0.00375 mmol) and **1a** (3.8 mg, 0.0075 mmol) in THF (1.25 mL). Stirred in vial at 50 °C for 20 hours. Analysis of crude ^{1}H NMR using internal standard 1,2-dimethoxyethane showed 3.4:0.4:1 *meta:dimeta*:*para* borylation (overall *meta*:*para* ratio of 3.8:1) in 64% yield (with 36% unreacted starting material). The crude product was purified by silica gel chromatography (10% EtOAc in Petroleum Ether 40-60 °C) and the monoborylated products were partially separated from the *dimeta* product. Mono borylated products were isolated as colourless oil (52 mg) with 3.7:1 *meta:para* ratio (as determined by ^{1}H NMR). A small amount of B_2Pin_2 impurity was found to be inseparable from the final product on silica. Corrected yield - 50 mg, 0.13 mmol, 52%.

Meta ^{1}H NMR (600 MHz, $CDCl_3$) δ 7.68 (d, J = 7.2 Hz, 1H), 7.63 (s, 1H), 7.32 (t, J = 7.5 Hz, 1H), 7.28 (d, J = 8.6 Hz, 1H), 6.28 (d, J = 7.0 Hz, 1H), 4.36 (dd, J = 8.0, 3.6 Hz, 1H), 3.03 (dt, J = 12.2, 3.3 Hz, 1H), 2.10 – 2.02 (m, 1H), 1.99 – 1.88 (m, 2H), 1.86 – 1.65 (m, 3H), 1.54 – 1.29 (m, 2H), 1.34 (s, 12H); **^{13}C NMR** (151 MHz, $CDCl_3$) δ 156.4 (q, J = 36.5 Hz), 140.9, 133.6, 133.4, 130.1, 128.0, 115.7 (q, J = 288.4 Hz), 83.8, 50.8, 44.9, 30.3, 25.5, 25.3, 24.8, 20.5. **HRMS** m/z: $[M + H]^+$ calc'd for $[C_{20}H_{28}BF_3NO_3]^+$ expect 398.2109; found 398.2109.

Dtbpy in THF:

Following general procedure F using *cis*-**12a** (67.8 mg, 0.25 mmol), B_2pin_2 (127 mg, 0.50 mmol), $[Ir(COD)OMe]_2$ (2.5 mg, 0.00375 mmol) and dtbpy (2.0 mg, 0.0075 mmol) in THF (1.25 mL). Stirred in vial at 50 °C for 20 hours. Analysis of crude ^{1}H NMR using internal standard 1,2-dimethoxyethane showed 1:2.5:1.5 *meta:dimeta*:*para* borylation (overall *meta*:*para* ratio of 2.3:1) in 99% yield. The crude product was purified by silica gel chromatography (10% EtOAc in Petroleum Ether 40-60 °C) and gave the title compound (as a 1:2.5:1.5 *meta:dimeta*:*para* ratio or overall *meta:para* ratio of 2.3:1, as determined by ^{1}H NMR) as a colourless oil (101.4 mg, 0.22 mmol, 88%). The mass quoted above is the actual mass of the isolated mixture of products, whilst the amount of substance in mmol has been calculated by examining the ratios of the *dimeta* to monoborylated products and accounting for the extra mass due to the higher molecular weight of the *dimeta* product. By subtracting the resonances for the *meta* isomer, the NMR data of the *dimeta* and *para* isomer can be assigned as follows:

Dimeta ^{1}H NMR (600 MHz, $CDCl_3$) δ 8.15 (s, 1H), 7.71 (s, 2H), 6.35 (d, J = 8.2 Hz, 1H), 4.33 (dd, J = 8.1, 3.6 Hz, 1H), 3.03 (dt, J = 12.2, 3.3 Hz, 1H), 2.10 – 2.02 (m, 1H), 1.99 – 1.88 (m, 2H), 1.86 – 1.65 (m, 3H), 1.54 – 1.29 (m, 2H), 1.32 (s, 24H); **^{13}C NMR** (151 MHz, $CDCl_3$) δ 156.4 (q, J = 36.5 Hz), 140.2, 140.1, 136.5, 115.7 (q, J = 288.6 Hz), 83.7, 50.8, 45.2, 30.3, 25.6, 25.4, 24.7, 20.4. **HRMS** m/z: $[M + H]^+$ calc'd for $[C_{26}H_{39}B_2F_3NO_5]^+$ expect 524.2961; found 524.2979.

Para ^{1}H NMR (600 MHz, $CDCl_3$) δ 7.76 (d, J = 8.1 Hz, 2H), 7.20 (d, J = 8.0 Hz, 2H), 6.24 (d, J = 6.7 Hz, 1H), 4.40 (dd, J = 8.0, 3.7 Hz, 1H), 3.03 (dt, J = 12.2, 3.3 Hz, 1H), 2.10 – 2.02 (m, 1H), 1.99 – 1.88 (m, 2H), 1.86 – 1.65 (m, 3H), 1.54 – 1.29 (m, 2H), 1.34 (s, 12H); **^{13}C NMR** (151 MHz, $CDCl_3$) δ 156.4 (q, J = 36.5 Hz), 144.8, 135.1, 126.6, 115.7 (q, J = 288.4 Hz), 83.8, 50.7, 44.8, 30.2, 25.5, 25.2, 24.8, 20.5.

(trans)-2,2,2-trifluoro-N-(2-(3-(4,4,5,5-tetramethyl-1,3,2-dioxaborolan-2-yl)phenyl)cyclohexyl)acetamide (trans-12)

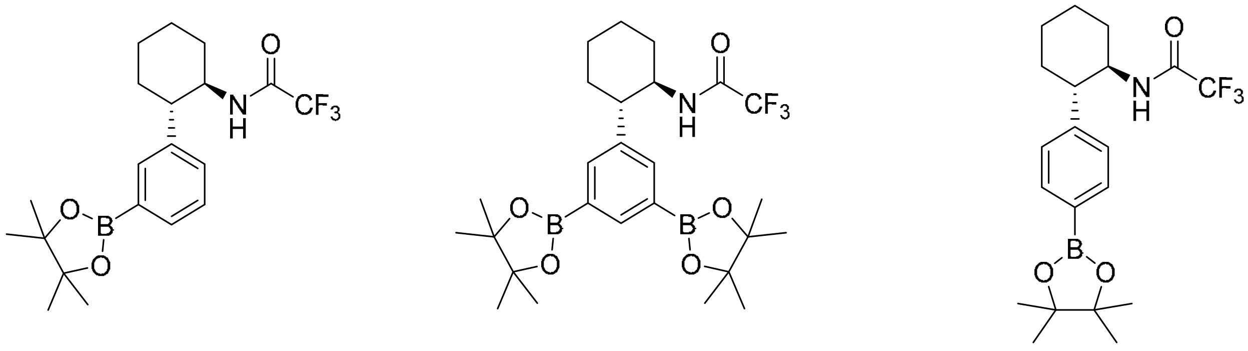

Meta ***Dimeta*** ***Para***

1a in THF:

Following general procedure F using *trans*-**12a** (67.8 mg, 0.25 mmol), B_2pin_2 (127 mg, 0.50 mmol), $[Ir(COD)OMe]_2$ (2.5 mg, 0.00375 mmol) and **1a** (3.8 mg, 0.0075 mmol) in THF (1.25 mL). Stirred in vial at 50 °C for 20 hours. Analysis of crude ^{1}H NMR using internal standard 1,2-dimethoxyethane showed 11.2:3.9:1 *meta:dimeta:para* borylation (overall *meta:para* ratio of 15.1:1) in 91% yield (with 9% unreacted starting material). The crude product was purified by silica gel chromatography (10% EtOAc in Petroleum Ether 40-60 °C) and the monoborylated products were partially separated from the *dimeta* product. Mono borylated products were isolated as colourless oil (52 mg) with 14:1 *meta:para* ratio (as determined by ^{1}H NMR). A small amount of B_2Pin_2 impurity was found to be inseparable from the final product on silica. Corrected yield - 60 mg, 0.15 mmol, 60%.

Meta ^{1}H NMR (600 MHz, $CDCl_3$) δ 7.67 (d, J = 6.9 Hz, 1H), 7.59 (s, 1H), 7.35 – 7.27 (m, 1H), 6.02 (s, 1H), 4.02 (qd, J = 11.6, 3.9 Hz, 1H), 2.53 (td, J = 11.7, 3.6 Hz, 1H), 2.22 (d, J = 12.3 Hz, 1H), 1.97 (d, J = 13.6 Hz, 1H), 1.90 – 1.79 (m, 2H), 1.62 (ddd, J = 25.9, 13.0, 3.5 Hz, 1H), 1.49 (dt, J = 28.4, 14.2 Hz, 1H), 1.40 – 1.35 (m, 1H), 1.39 – 1.29 (m, 2H), 1.34 (s, 12H); **^{13}C NMR** (151 MHz, $CDCl_3$) δ 156.2 (q, J = 36.6 Hz), 141.3, 134.2, 133.5, 129.5, 128.2, 115.6 (q, J = 288.3 Hz), 83.8, 53.6, 50.0, 34.5, 33.0, 25.9, 25.1, 24.8. **HRMS** m/z: $[M + H]^+$ calc'd for $[C_{20}H_{28}BF_3NO_3]^+$ expect 398.2109; found 398.2108.

Dtbpy in THF:

Following general procedure F usin *trans*-**12a** (67.8 mg, 0.25 mmol), B_2pin_2 (127 mg, 0.50 mmol), $[Ir(COD)OMe]_2$ (2.5 mg, 0.00375 mmol) and dtbpy (2.0 mg, 0.0075 mmol) in THF (1.25 mL). Stirred in vial at 50 °C for 20 hours. Analysis of crude ^{1}H NMR using internal standard 1,2-dimethoxyethane showed 1:1.4:1.3 *meta:dimeta:para* borylation (overall *meta:para* ratio of 1.8:1) in 98% yield. The

crude product was purified by silica gel chromatography (10% EtOAc in Petroleum Ether 40-60 ^{o}C) gave the title compound (as a 1:1.3:1.2 *meta:dimeta:para* ratio or overall *meta:para* ratio of 1.9:1, as determined by ^{1}H NMR) as a white solid (104.3 mg, 0.24 mmol, 94%). The mass quoted above is the actual mass of the isolated mixture of products, whilst the amount of substance in mmol has been calculated by examining the ratios of the *dimeta* to monoborylated products and accounting for the extra mass due to the higher molecular weight of the *dimeta* product. By subtracting the resonances for the *meta* isomer, the NMR data of the *dimeta* and *para* isomer can be assigned as follows:

Dimeta ^{1}H NMR (600 MHz, $CDCl_3$) δ 8.14 (s, 1H), 7.70 (s, 2H), 6.14 (d, J = 8.4 Hz, 1H), 4.02 (qd, J = 11.6, 3.9 Hz, 1H), 2.53 (td, J = 11.7, 3.6 Hz, 1H), 2.22 (d, J = 12.3 Hz, 1H), 1.97 (d, J = 13.6 Hz, 1H), 1.90 – 1.79 (m, 2H), 1.62 (ddd, J = 25.9, 13.0, 3.5 Hz, 1H), 1.49 (dt, J = 28.4, 14.2 Hz, 1H), 1.40 – 1.35 (m, 1H), 1.39 – 1.29 (m, 2H), 1.34 (s, 12H); **^{13}C NMR** (151 MHz, $CDCl_3$) δ 156.2 (q, J = 36.6 Hz), 140.4, 140.1, 136.5, 115.6 (q, J = 288.2 Hz), 83.8, 53.9, 49.8, 34.1, 32.9, 25.9, 25.1, 24.8. **HRMS** m/z: $[M + H]^+$ calc'd for $[C_{26}H_{39}B_2F_3NO_5]^+$ expect 524.2961; found 524.2978.

Para ^{1}H NMR (600 MHz, $CDCl_3$) δ 7.74 (d, J = 8.0 Hz, 2H), 7.18 (d, J = 8.0 Hz, 2H), 6.00 (d, J = 7.8 Hz, 1H), 4.02 (qd, J = 11.6, 3.9 Hz, 1H), 2.53 (td, J = 11.7, 3.6 Hz, 1H), 2.22 (d, J = 12.3 Hz, 1H), 1.97 (d, J = 13.6 Hz, 1H), 1.90 – 1.79 (m, 2H), 1.62 (ddd, J = 25.9, 13.0, 3.5 Hz, 1H), 1.49 (dt, J = 28.4, 14.2 Hz, 1H), 1.40 – 1.35 (m, 1H), 1.39 – 1.29 (m, 2H), 1.33 (s, 12H); **^{13}C NMR** (151 MHz, $CDCl_3$) δ 156.2 (q, J = 36.6 Hz), 145.5, 135.2, 126.6, 115.6 (q, J = 288.2 Hz), 83.7, 53.3, 50.2, 34.7, 32.9, 25.8, 25.1, 24.9.

Borylation of Acetanilides (Figure 3b)

N-(3,5-bis(4,4,5,5-tetramethyl-1,3,2-dioxaborolan-2-yl)phenyl)acetamide

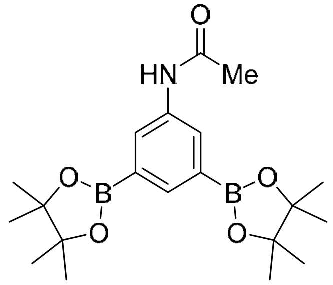

1a in THF:

Following general procedure F using acetanilide (33.8 mg, 0.25 mmol), B_2pin_2 (191 mg, 0.75 mmol), $[Ir(COD)OMe]_2$ (2.5 mg, 0.00375 mmol) and **1a** (3.8 mg, 0.0075 mmol) in THF (1.25 mL). The reaction was stirred at 50 °C for 20 hours before cooling and the solvents removed. Analysis of crude ^{1}H NMR using internal standard 1,2-dimethoxyethane and comparison to separately prepared mono borylated samples (see above) showed 14:48:5:5 *dimeta*:*meta*:*para*:*ortho* borylation in 77% yield. Whilst largely inseparable, the crude product was able to be purified to some extent by silica gel chromatography (Pet. Ether (40-60):EtOAc (8:2-6:4) to give an enriched (~3:1 diborylated:monoborylated) fraction of the title compound as a white solid (9.5 mg) used for characterisation of the diborylated product. The mono borylated isomers were identified in the crude NMR by comparison to separately prepared mono-borylated samples (see below):

Dimeta ^{1}H NMR (500 MHz, $CDCl_3$) δ 8.02 (s, 1H), 7.98 (s, 2H), 7.13 (br s, 1H), 3.39 (s, 3H), 1.33 (s, 24H); **^{13}C NMR** (126 MHz, $CDCl_3$) δ 168.0, 137.2, 136.7, 129.2, 83.9, 24.9, 24.5; **HRMS** m/z: $[M + H]^+$ calc'd for $[C_{20}H_{32}NO_5B_2]^+$ expect 388.2467; found 388.2469.

Dtbpy in THF:

Following general procedure using acetanilide (33.8 mg, 0.25 mmol), B_2pin_2 (274 mg, 1.0 mmol), $[Ir(COD)OMe]_2$ (2.5 mg, 0.00375 mmol) and dtbpy (2.0 mg, 0.0075 mmol) in THF (1.25 mL). The reaction was stirred at 50 °C for 20 hours before cooling and the solvents removed. Analysis of crude ^{1}H NMR using internal standard 1,2-dimethoxyethane and comparison to separately prepared mono borylated samples (see above) showed 39:26:27 *dimeta*:*meta*:*para* borylation in 93% yield.

N-(4-(4,4,5,5-tetramethyl-1,3,2-dioxaborolan-2-yl)phenyl)acetamide

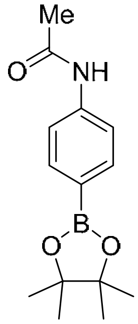

To a stirred solution of 4-(4,4,5,5-tetramethyl-1,3,2-dioxaborolan-2-yl)aniline (219 mg, 1.0 mmol) in CH_2Cl_2 was added triethylamine (0.41 ml, 3.0 mmol) and acetyl chloride (0.14 ml, 2.0 mmol) at 0 ^{o}C. After stirring at room temperature for 3 h, water was added and the organic layer washed with brine,

dried (Na_2SO_4) and evaporated *in vacuo*. The crude residue was purified on silica (eluting with Et_2O) to give the title compound as a white solid (165 mg, 0.63 mmol, 63%).

^{1}H NMR (500 MHz, $CDCl_3$) δ 7.87 (br s, 1H), 7.74 (d, *J* = 8.1 Hz, 2H), 7.52 (d, *J* = 8.1 Hz, 2H), 2.14 (s, 3H), 1.33 (s, 12H); **^{13}C NMR** (126 MHz, $CDCl_3$) δ 168.8, 140.7, 135.8, 118.7, 83.7, 24.7, 24.7; **HRMS**: m/z: $[M + H]^+$ calc'd for $[C_{14}H_{21}NO_3B]^+$ expect 262.1614; found 262.1624.

N-(3-(4,4,5,5-tetramethyl-1,3,2-dioxaborolan-2-yl)phenyl)acetamide

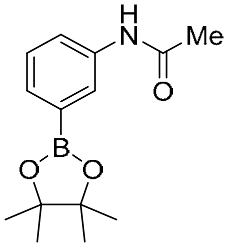

To a stirred solution of 3-(4,4,5,5-tetramethyl-1,3,2-dioxaborolan-2-yl)aniline (219 mg, 1.0 mmol) in CH_2Cl_2 was added triethylamine (0.41 ml, 3.0 mmol) and acetyl chloride (0.14 ml, 2.0 mmol) at 0 °C. After stirring at room temperature for 3 h, water was added and the organic layer washed with brine, dried (Na_2SO_4) and evaporated *in vacuo*. The crude residue was purified on silica (eluting with Et_2O) to give the title compound as a white solid (110 mg, 0.42 mmol, 42%).

^{1}H NMR (500 MHz, $CDCl_3$) δ 7.87 (br d, *J* = 6.5 Hz, 1H), 7.63 (s, 1H), 7.54 (d, *J* = 7.4 Hz, 1H), 7.34 (t, *J* = 7.5 Hz, 1H), 7.31 (br s, 1H) 2.16 (s, 3H), 1.33 (s, 12H); **^{13}C NMR** (126 MHz, $CDCl_3$) δ 168.6, 137.4, 130.6, 128.6, 125.8, 123.1, 83.9, 24.9, 24.6.

Data is in accordance with those previously reported.[14]

N-(2-(4,4,5,5-tetramethyl-1,3,2-dioxaborolan-2-yl)phenyl)acetamide

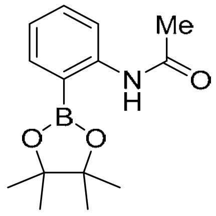

To a stirred solution of 2-(4,4,5,5-tetramethyl-1,3,2-dioxaborolan-2-yl)aniline (219 mg, 1.0 mmol) in CH_2Cl_2 was added triethylamine (0.41 ml, 3.0 mmol) and acetyl chloride (0.14 ml, 2.0 mmol) at 0 °C. After stirring at room temperature for 3 h, water was added and the organic layer washed with brine, dried (Na_2SO_4) and evaporated *in vacuo*. The crude residue was purified on silica (eluting with Et_2O) to give the title compound as a white solid (22 mg, 0.084 mmol, 8%). Apparent diacetylation accounted for a large amount of the by-product in this case.

^{1}H NMR (500 MHz, $CDCl_3$) δ 10.35 (br s, 1H), 7.96 (br s, 1H), 7.72 (dd, *J* = 1.4, 7.2 Hz, 1H), 7.34 (t, *J* = 7.8 Hz, 1H), 7.09 (t, *J* = 7.4 Hz, 1H), 1.99 (s, 3H), 1.37 (s, 12H); **^{13}C NMR** (126 MHz, $CDCl_3$) δ 168.8, 135.2, 131.2, 124.0, 118.1, 83.2, 25.3, 23.6; **HRMS**: m/z: $[M + Na]^+$ calc'd for $[C_{14}H_{20}NO_3BNa]^+$ expect 284.1428; found 284.1427.

N-(2-chloro-5-(4,4,5,5-tetramethyl-1,3,2-dioxaborolan-2-yl)phenyl)acetamide

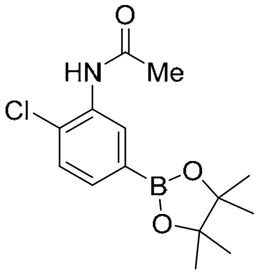

meta

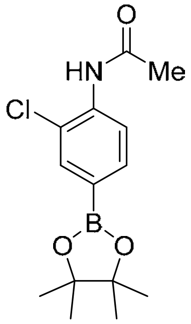

para

1a in THF:

Following general procedure F using 2-chloroacetanilide (42.4 mg, 0.25 mmol), B_2pin_2 (127 mg, 0.50 mmol), $[Ir(COD)OMe]_2$ (2.5 mg, 0.00375 mmol) and **1a** (3.8 mg, 0.0075 mmol) in THF (1.25 mL). The reaction was stirred at 50 °C for 20 hours before cooling and the solvents removed. Analysis of crude ^{1}H NMR using internal standard 1,2-dimethoxyethane showed 1:1.1 *meta*:*para* borylation in 74% yield. The crude product was purified by silica gel chromatography (Pet. Ether (40-60):EtOAc (19:1-6:4) to give the title compound (as a 1:1.3:0.4 mixture of *meta*:*para*:SM ratio, as determined by ^{1}H NMR) as a colourless oil. NMR yields have been used for all comparisons.

Meta ^{1}H NMR (600 MHz, $CDCl_3$) δ 8.62 (s, 1H), 7.56 (br s, 1H), 7.46 (d, *J* = 7.9 Hz, 1H), 7.35 (d, *J* = 7.9 Hz, 1H), 2.23 (s, 3H), 1.32 (s, 12H); ^{13}C NMR (151 MHz, $CDCl_3$) δ 168.1, 134.0, 131.1, 128.5, 128.4, 126.4, 84.1, 24.8; HRMS: m/z: $[M+H]^+$ calc'd for $[C_{14}H_{20}{}^{10}BNO_3Cl]^+$ expect 295.1256; found 295.1257.

Para ^{1}H NMR (600 MHz, $CDCl_3$) δ 8.40 (br d, *J* = 8.0 Hz, 1H), 7.79 (s, 1H), 7.75 (br s, 1H), 7.68 (d, *J* = 8.0 Hz, 1H), 2.24 (s, 3H), 1.31 (s, 12H); ^{13}C NMR (151 MHz, $CDCl_3$) δ 168.3, 137.0, 135.1, 134.3, 121.8, 120.3, 84.1, 24.8, 24.8.

N-(2-bromo-5-(4,4,5,5-tetramethyl-1,3,2-dioxaborolan-2-yl)phenyl)acetamide

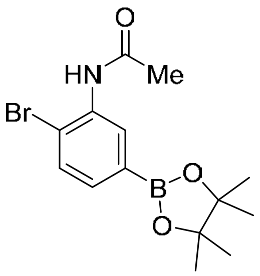

meta

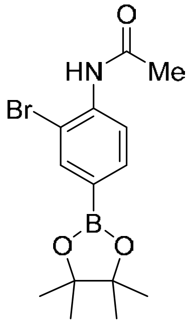

para

1a in THF:

Following general procedure F using 2-bromoacetanilide (53.5 mg, 0.25 mmol), B_2pin_2 (127 mg, 0.50 mmol), $[Ir(COD)OMe]_2$ (2.5 mg, 0.00375 mmol) and **1a** (3.8 mg, 0.0075 mmol) in THF (1.25 mL). The reaction was stirred at 70 °C for 20 hours before cooling and the solvents removed. Analysis of crude ^{1}H NMR using internal standard 1,2-dimethoxyethane showed 1:1.4 *meta*:*para* borylation in 65% yield. The crude product was purified by silica gel chromatography (Pet. Ether (40-60):EtOAc (19:1-6:4) to give the title compound (as a 1:1.7:1.3 mixture of *meta*:*para*:SM ratio, as determined by ^{1}H NMR) as a colourless oil. NMR yields have been used for all comparisons.

Meta ^{1}H NMR (500 MHz, $CDCl_3$) δ 8.55 (s, 1H), 7.52 (br s, 1H ,overlapped with d, J = 7.8 Hz, 1H), 7.37 (d, J = 7.8 Hz, 1H), 2.20 (s, 3H), 1.30 (s, 12H); **^{13}C NMR** (126 MHz, $CDCl_3$) δ 168.1, 135.1, 131.8 131.6, 128.8, 117.5 84.4, 24.9, 24.8; **HRMS** m/z: $[M+H]^+$ calc'd for $[C_{14}H_{20}{}^{10}BNO_3Br]^+$ expect 339.0756; found 339.0752.

Para ^{1}H NMR (500 MHz, $CDCl_3$) δ 8.35 (br d, J = 7.9 Hz, 1H), 7.94 (d, J = 1.2 Hz, 1H), 7.72 (br s, 1H), 7.70 (dd, J = 1.2, 7.9 Hz, 1H), 2.22 (s, 3H), 1.31 (s, 12H); **^{13}C NMR** (126 MHz, $CDCl_3$) δ 168.3, 138.5, 138.0, 135.0, 120.8, 112.7, 84.1, 24.9, 24.8.

NMR Titrations (Figure 3c)

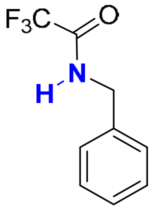

2h

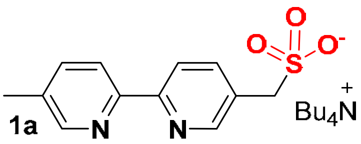

1a

The ^{1}H-NMR of a solution of **2h** (0.010 mmol) in the given deuterated solvent (0.75 ml) was recorded (**2h** concentration 0.0133 M) and the chemical shift of the NH proton observed. Increasing amounts of **1a** were added to the sample (the concentration of 1a being determined in the sample relative to **2h**), and the downfield shift of the NH proton was measured for each concentration of **1a**. The resulting points were fitted (http://app.supramolecular.org/bindfit/), using the assumption of 1:1 binding, using a Nelder-Mead algorithm. http://supramolecular.org/.

Solvent: d_3-MeCN

Conc. **2h** (M)	Conc. **1a** (M)	δ NH Proton
1.33E-02	0	8.002
1.33E-02	0.011039	8.142
1.33E-02	0.023275	8.27
1.33E-02	0.041363	8.428
1.33E-02	0.071288	8.63
1.33E-02	0.098021	8.778
1.33E-02	0.122227	8.891

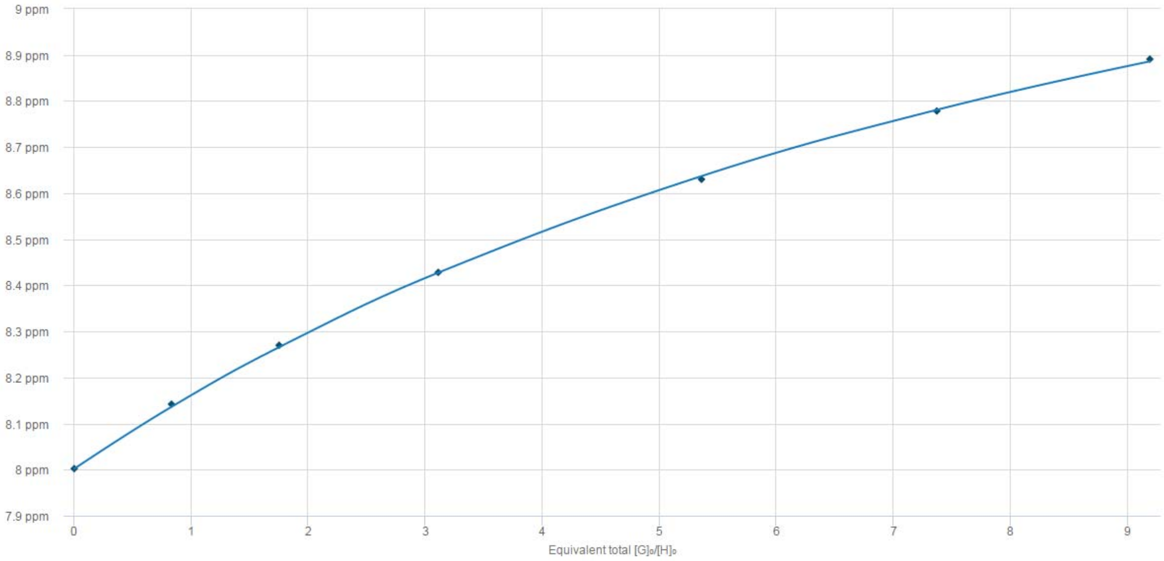

Calculated K_a = 7.56 M^{-1} ± 1.75%

Solvent: d_8-THF

Conc. **2h** (M)	Conc. **1a** (M)	δ NH Proton
1.33E-02	0	8.881
1.33E-02	0.0108129	9.104
1.33E-02	0.022211	9.257
1.33E-02	0.040432	9.422
1.33E-02	0.06384	9.568
1.33E-02	0.08911	9.674
1.33E-02	0.119168	9.77

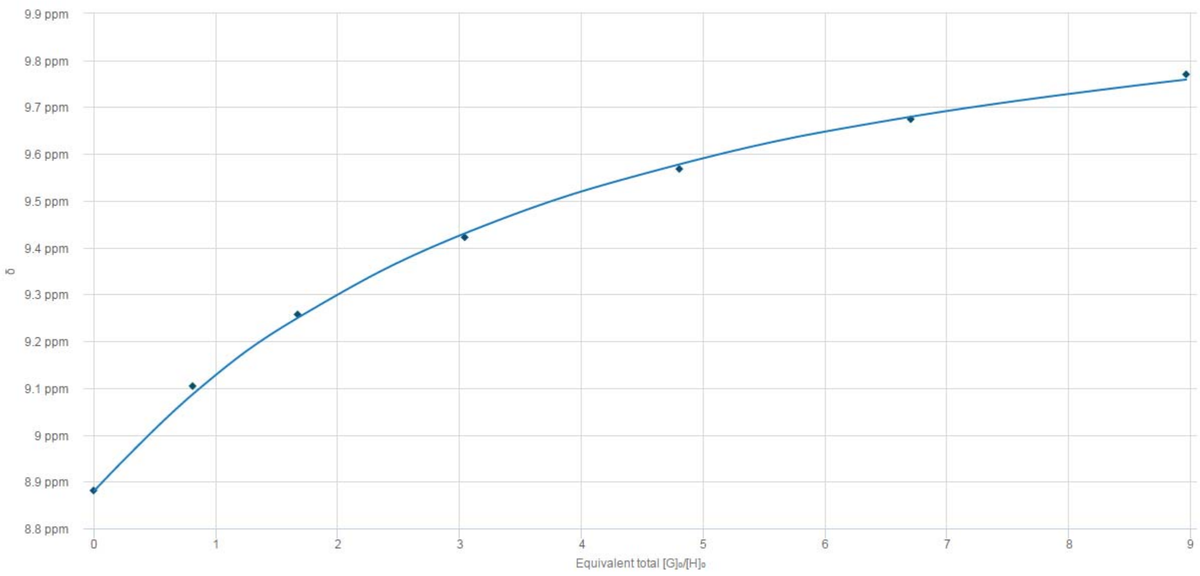

Calculated K_a = 24.21 M^{-1} ± 5.44%

Solvent: d_6-Benzene (in this case concentration of 0.0066 M was used for **2h**).

Conc. **2h** (M)	Conc. **1a** (M)	δ NH Proton
6.66E-03	0	5.55
6.66E-03	0.0060606	6.965
6.66E-03	0.0135864	7.875
6.66E-03	0.0215784	8.445
6.66E-03	0.0301032	8.856
6.66E-03	0.0380952	9.176
6.66E-03	0.0510822	9.504

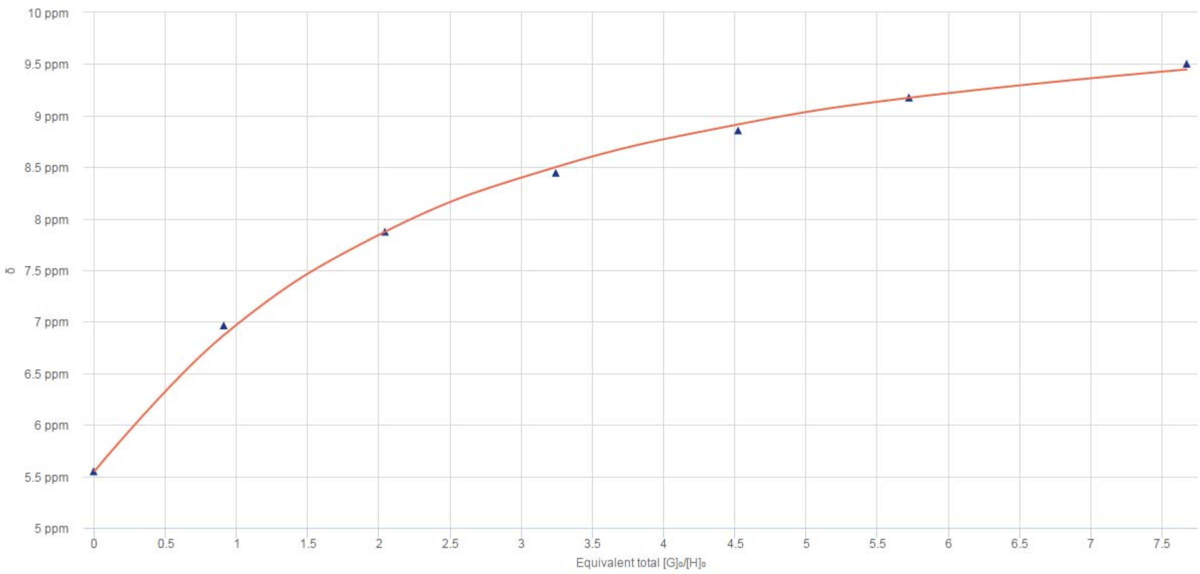

Calculated K_a = 87.59 M^{-1} ± 7.68%

Substrate **2h** was titrated with 5,5'-dimethylbipyridine as a control in order to rule out possible interaction of bipyridine nitrogens with the amide NH on the substrate.

Titration in d_3-MeCN and d_8-THF at same concentrations as used below for d_6-Benzene showed no appreciable shift of NH proton. In d_6-Benzene, some shift was observed and the K_a was calculated to be <1 M^{-1} compared with 88 M^{-1} for titration with **1a**. These control titrations suggest that it is indeed the sulfonate portion of 1a that is responsible for the interaction observed.

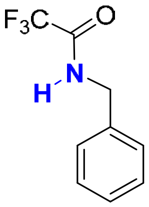

2h

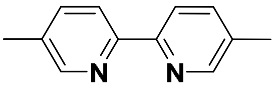

1j

Solvent: d_6-Benzene.

Conc. **2h** (M)	Conc. **1j** (M)	δ NH Proton
1.33E-02	0	5.56
1.33E-02	0.026733	5.72
1.33E-02	0.055993	5.88
1.33E-02	0.080465	6
1.33E-02	0.109326	6.15
1.33E-02	0.136325	6.3
1.33E-02	0.188328	6.53
1.33E-02	0.244853	6.77

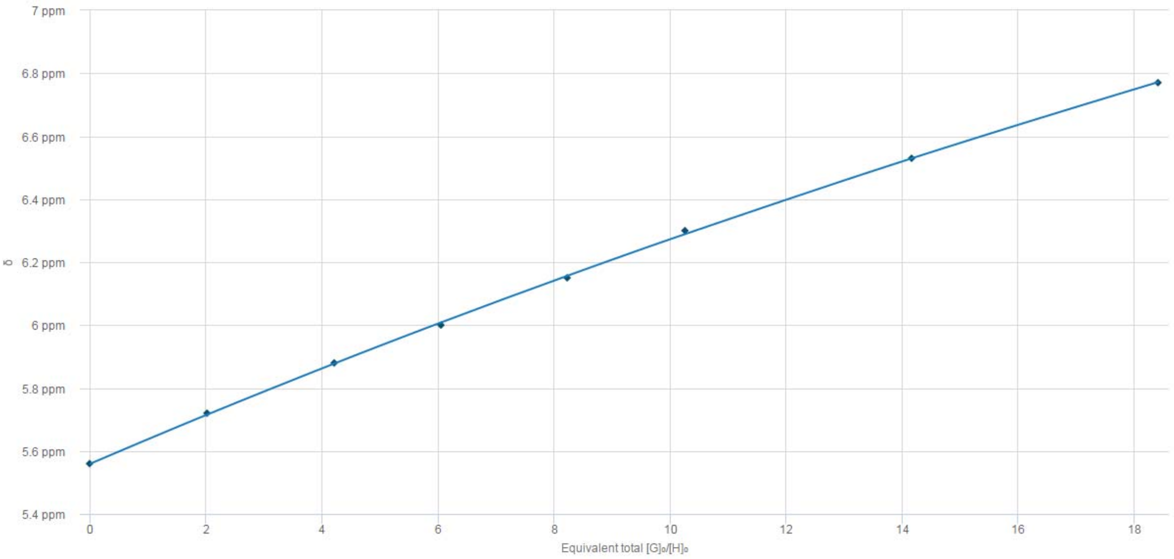

Calculated K_a = 0.84 M^{-1} ± 1.09%

Synthesis and borylations of *N*-Ethyl substrates (Figure 3d)

_N_-ethyl-2,2,2-trifluoro-N-(2-(trifluoromethyl)benzyl)acetamide (13)

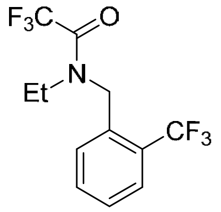

2,2,2-Trifluoro-N-(2-(trifluoromethyl)benzyl)acetamide (1.36g, 5.0 mmol) was dissolved in THF (10 mL) and cooled to 0 °C. NaH (60%, 260 mg, 6.5 mmol) was added in portions over 1 min and the reaction stirred for 30 mins at the same temperature before the addition of iodoethane (0.60 mL, 7.5 mmol) dropwise over 1 min. The reaction was allowed to warm to rt then heated to 60 °C for 8 h. Water (1 mL) was added to quench and the solvents removed *in vacuo*. Water (10 mL) was added to the residue and the aqueous phase extracted with Et_2O (3 x 20 mL) and washed with brine. The combined organics were dried ($MgSO_4$) and concentrated *in vacuo*. The crude oil was purified by silica gel chromatography with Pet. Ether (40-60):Et_2O (19:1) to yield a colourless oil (702 mg, 2.35 mg, 47%). In CDCl3 at 298K ^{1}H-NMR showed a mixture of rotamers. Heating to 120°C in DMSO was required for these to coalesce.

^{1}H NMR (500 MHz, DMSO-d_6 – 120 °C) δ 7.77 (d, *J* = 7.7 Hz, 1H), 7.70 (t, *J* = 7.6 Hz, 1H), 7.54 (t, *J* = 7.6 Hz, 1H), 7.37 (d, *J* = 7.6 Hz, 1H), 4.87 (s, 2H), 3.51 (q, *J* = 7.0 Hz, 2H), 1.18 (t, *J*= 7.1 Hz, 3H); **^{13}C NMR** (126 MHz, DMSO-d_6 – 120 °C) δ 156.9 (q, $^2J_{C-F}$ = 36 Hz), 134.6, 133.1, 128.4, 128.2, 127.3 (q, $^2J_{C-F}$ = 30 Hz), 126.6 (q, $^3J_{C-F}$ = 6 Hz), 124.7 (q, $^1J_{C-F}$ = 275 Hz), 116.8 (q, $^1J_{C-F}$ = 289 Hz), 46.6, 43.0, 13.1; **HRMS** m/z: $[M + H]^+$ calc'd for $[C_{12}H_{12}F_6NO]^+$ expect 300.0823; found 300.0826.

N-(2-bromophenethyl)-N-ethyl-2,2,2-trifluoroacetamide (14)

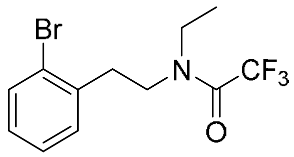

N-(2-bromophenethyl)-2,2,2-trifluoroacetamide (**4f**) (888 mg; 3 mmol; 1eq) was dissolved in dry THF (12 ml) in a flame dried flask under argon atmosphere. The solution was cooled to 0 °C and NaH (60% dispersion in mineral oil) (240 mg; 6 mmol; 2eq) was added. Reaction was stirred at RT for 1 hour and iodoethane (0.36 ml; 4.5 mmol; 1.5eq) was added. The reaction mixture was then refluxed for 16 hours. Reaction was then quenched with water (20 ml) at 0 °C. Reaction mixture was then extracted with ethyl acetate (3x20 ml). Organics were combined, washed with brine (50 ml), dried over $MgSO_4$, filtered and concentrated to give the crude product as a yellow oil. The crude was then purified by column chromatography (SiO_2, 4% EtOAc in Petroleum Ether 40-60 °C) to give the final product as a light yellow oil (676 mg; 70%).

^{1}H NMR (500 MHz, DMSO-d$_6$, 120 °C) δ 7.60 (d, J = 7.8 Hz, 1H), 7.41 – 7.30 (m, 2H), 7.19 (ddd, J = 8.8, 6.2, 3.0 Hz, 1H), 3.66 (t, J = 8.3 Hz, 2H), 3.52 – 3.39 (br, 2H), 3.08 (t, J = 8.3 Hz, 2H), 1.19 (t, J = 7.1 Hz, 3H); **^{13}C NMR** (126 MHz, DMSO-d$_6$, 120 °C) δ 156.1 (q, J = 34.9 Hz), 138.0, 133.0, 131.4, 129.0, 128.2, 124.2, 116.8 (q, J = 288.7 Hz), 46.9, 42.7, 33.5, 13.4. **HRMS**: m/z: $[M + H]^+$ calc'd for $[C_{12}H_{14}BrF_3NO]^+$ expect 324.0205; found 324.0204.

N-(3-(2-Bromophenyl)propyl)-N-ethyl-2,2,2-trifluoroacetamide (15)

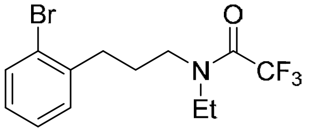

A flask was charged with (7c) (900 mg, 2.90 mmol) in THF (10 mL) and cooled to 0 °C before the portion wise addition of NaH (174 mg, 4.35 mmol, 60% on mineral oil). The suspension was stirred under argon for 15 mins at this temperature before the addition of iodoethane (0.50 mL, 5.80 mmol). The reaction was heated to 60 °C for 14 h before being quenched with water (0.5 mL), solvents removed *in vacuo* and the residue extracted with Et_2O (2 x 20 mL), dried ($MgSO_4$) and solvents removed *in vacuo*. The crude product was purified by silica gel chromatography (Pet. Ether (40-60):EtOAc (20:1) to give **15** as a colourless oil (730 mg, 2.16 mmol, 74%).

^{1}H NMR (500 MHz, DMSO-d_6, 120 °C) δ 7.57 (dd, J = 0.8, 7.9 Hz, 1H), 7.35 (dd, J = 1.6, 7.8 Hz, 1H), 7.31 (dt, J = 0.8, 7.2 Hz, 1H), 7.14 (dt, J = 1.8, 7.8 Hz, 1H), 3.50-3.46 (m, 4H), 2.78 (t, J = 7.8 Hz, 2H), 1.95 (quint, J = 7.6 Hz, 2H), 1.19 (t, J = 7.1 Hz, 3H); **^{13}C NMR** (126 MHz, DMSO-d_6, 120 °C) δ 156.1 (q, $^2J_{C-F}$ = 35 Hz), 140.6, 132.9, 130.7, 128.3, 128.0, 124.0, 116.9 (q, $^1J_{C-F}$ = 289 Hz), 46.5, 42.3, 33.0, 28.0, 13.3; **HRMS**: m/z: $[M]^+$ calc'd for $[C_{13}H_{16}NOF_3Br]^+$ expect 338.0367; found 338.0374.

Substrates **13**, **14** and **15** were submitted to borylation with ligand **1a** and in all cases regioselectivity was found to be poor, implicating the crucial aspect of hydrogen bonding in the transition state for high *meta*-selectivity to be achieved. Procedures and characterisation data for the borylation of these compounds is given below.

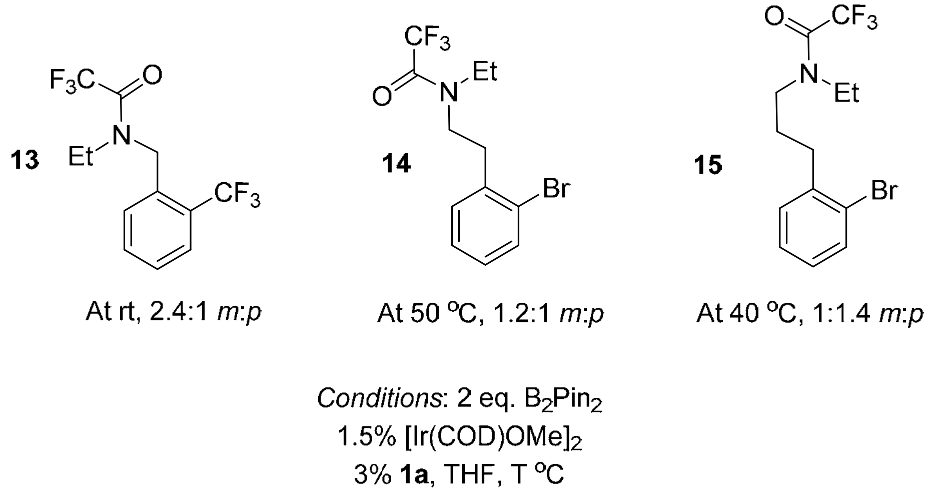

N-ethyl-2,2,2-trifluoro-N-(5-(4,4,5,5-tetramethyl-1,3,2-dioxaborolan-2-yl)-2-(trifluoromethyl)benzyl)acetamide (13a)

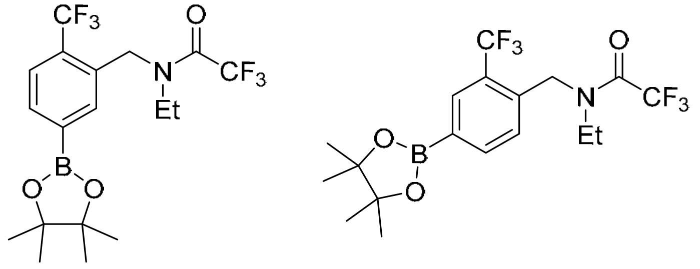

meta ***para***

1a in THF:

Following general procedure F using *N*-ethyl-2,2,2-trifluoro-N-(2-(trifluoromethyl)benzyl)acetamide (**13**) (149.5 mg, 0.50 mmol), B_2pin_2 (254 mg, 1.0 mmol), $[Ir(COD)OMe]_2$ (5.0 mg, 0.0075 mmol) and **1a** (7.6 mg, 0.015 mmol) in THF (2.5 mL). The reaction was stirred at rt °C for 20 hours before cooling and the solvents removed. Analysis of crude ^{1}H NMR using internal standard 1,2-dimethoxyethane showed 2.4:1 *meta*:*para* borylation in 98% yield as a mixture of rotamers. The crude product was purified by silica gel chromatography (Pet. Ether (40-60):EtOAc (19:1-9:1) to give the title compound (as a 1.5:1:0.8 mixture of *meta*:*para*:B_2pin_2 ratio, as determined by ^{1}H NMR) as a colourless oil (180 mg). As the B_2Pin_2 impurity was found to be inseparable from the final product on silica, corrected yield is calculated as 0.36 mmol, 71%. This sample required heating to 120 °C in DMSO for the rotamers to coalesce. However, it was subsequently then possible to deconvolute *meta*:*para* ratio in the rt $CDCl_3$ spectrum.

Meta ^{1}H NMR (500 MHz, DMSO-d_6 – 120 °C) δ 7.83 (d, J = 7.7 Hz, 1H), 7.76 (d, J = 7.7 Hz, 1H), 4.88 (s, 1H), 3.50 (q, J = 6.8 Hz, 2H), 1.35 (s, 12H), 1.18 (t, J = 6.8 Hz, 3H); **^{13}C NMR** (126 MHz, DMSO-d_6 – 120 °C) δ 156.9 (q, $^2J_{C\text{-}F}$ = 36 Hz), 134.1, 133.73, 133.67, 129.6 (q, $^2J_{C\text{-}F}$ = 31 Hz), 125.7 (q, $^3J_{C\text{-}F}$ = 6 Hz), 124.6 (q, $^1J_{C\text{-}F}$ = 274 Hz), 116.8 (q, $^1J_{C\text{-}F}$ = 288 Hz), 84.7, 46.6, 24.9, 13.2; **HRMS** m/z: $[M + H]^+$ calc'd for $[C_{18}H_{23}BNO_3F_6]^+$ expect 426.1675; found 426.1678.

Para ^{1}H NMR (500 MHz, DMSO-d_6 – 120 °C) δ 8.01 (s, 1H), 7.97 (d, J = 7.5 Hz, 1H), 7.39 (d, J = 7.5 Hz, 1H), 4.88 (s, 2H), 3.51 (q, J = 6.8 Hz, 2H), 1.36 (s, 12H), 1.18 (t, J = 6.8 Hz, 3H); **^{13}C NMR** (126 MHz, DMSO-d_6 – 120 °C) δ 156.9 (q, $^2J_{C\text{-}F}$ = 36 Hz), 138.9, 137.7, 131.9 (q, $^2J_{C\text{-}F}$ = 6 Hz), 127.7, 126.9 (q, $^2J_{C\text{-}F}$ = 31 Hz), 124.6 (q, $^1J_{C\text{-}F}$ = 274 Hz), 116.8 (q, $^1J_{C\text{-}F}$ = 288 Hz), 84.7, 46.8, 43.1, 24.9, 13.2.

N-(2-bromo-5-(4,4,5,5-tetramethyl-1,3,2-dioxaborolan-2-yl)phenethyl)-N-ethyl-2,2,2-trifluoroacetamide (14a)

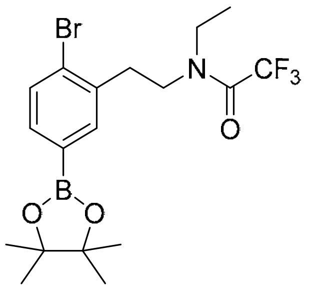

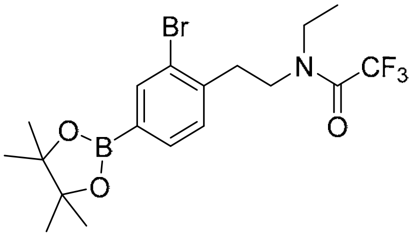

Meta ***Para***

1a in THF:

Following general procedure F using **14** (61.8 mg, 0.25 mmol), B_2pin_2 (127 mg, 0.50 mmol), $[Ir(COD)OMe]_2$ (2.5 mg, 0.00375 mmol) and **1a** (3.8 mg, 0.0075 mmol) in THF (1.25 mL). Stirred in vial at 50 °C for 20 hours. Analysis of crude ^1H NMR using internal standard 1,2-dimethoxyethane showed 1.2:1 *meta*:*para* borylation in 99% yield. The crude product was purified by silica gel chromatography (10% EtOAc in Petroleum Ether 40-60 °C) gave the title compound (as a 1.2:1 mixture of *meta*:*para* ratio, as determined by ^1H NMR) as a colourless oil (124 mg). A small amount of B_2Pin_2 impurity was found to be inseparable from the final product on silica. Corrected yield - 101 mg, 0.22 mmol, 89%.

Meta ^1H NMR (500 MHz, DMSO-d6, 120 °C) δ 7.65 – 7.59 (m, 2H), 7.47 (d, J = 7.9 Hz, 1H), 3.69 - 3.62 (m, 2H), 3.46 (br, 2H), 3.09 (t, J = 7.6 Hz, 2H), 1.34 (s, 12H), 1.21 – 1.15 (m, 3H); ^{13}C NMR (126 MHz, DMSO-d6, 120 °C) δ 156.2 (q, J = 35.6 Hz), 137.5, 137.3, 134.8, 134.1, 128.0, 116.9 (q, J = 288.7 Hz), 84.5, 46.8, 42.8, 34.0, 25.0, 13.5. HRMS: m/z: $[M + H]^+$ calc'd for $[C_{18}H_{25}BBrF_3NO_3]^+$ expect 450.1057; found 450.1058.

Para ^1H NMR (500 MHz, DMSO-d6, 120 °C) δ 7.85 (s, 1H), 7.65 – 7.59 (m, 1H), 7.37 (d, J = 7.5 Hz, 1H), 3.69 - 3.62 (m, 2H), 3.46 (br, 2H), 3.09 (t, J = 7.6 Hz, 2H), 1.34 (s, 12H), 1.21 – 1.15 (m, 3H); ^{13}C NMR (126 MHz, DMSO-d6, 120 °C) δ 156.2 (q, J = 35.6 Hz), 141.1, 138.7, 132.7, 131.2, 124.3, 116.9 (q, J = 288.7 Hz), 84.6, 47.0, 42.8, 34.0, 25.0, 13.5.

_N_-(3-(2-Bromo-5-(4,4,5,5-tetramethyl-1,3,2-dioxaborolan-2-yl)phenyl)propyl)-N-ethyl-2,2,2-trifluoroacetamide (15a)

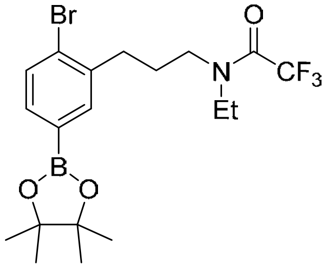

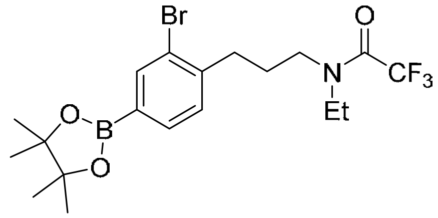

meta ***para***

Following general procedure F using *N*-(3-(2-Bromophenyl)propyl)-N-ethyl-2,2,2-trifluoroacetamide (169 mg, 0.50 mmol), B_2pin_2 (190 mg, 0.75 mmol), $[Ir(COD)OMe]_2$ (5.0 mg, 0.0075 mmol) and 5,5'-dimethyl-2,2'-dipyridyl (2.8 mg, 0.015 mmol) in THF (2.5 mL). The reaction was stirred at 40 °C for 20 hours before cooling and the solvents removed. Analysis of crude ^1H NMR using internal standard 1,2-dimethoxyethane showed 1:1.4 *meta*:*para* borylation in 98% yield. The crude product was purified by

silica gel chromatography (Pet. Ether (40-60):EtOAc (19:1-9:1) to give the title compound as a colourless oil (191 mg, 0.37 mmol, 74%*) as a 1:1.6 *meta*:*para* mixture containing 17% B_2pin_2.

*Adjusted yield accounting for B_2pin_2

Meta ^{1}H NMR (500 MHz, DMSO-d_6, 120 °C) δ 7.65 (s, 1H), 7.58 (d, J = 7.9 Hz, 1H), 7.44 (dd, J = 7.9, 1.2 Hz, 1H), 3.53 - 3.44 (m, 4H), 2.79 (t, J = 7.8 Hz, 2H), 1.94 (quint, J = 7.6 Hz, 2H), 1.33 (s), 1.19 (t, J = 7.1 Hz, 3H); ^{13}C NMR (126 MHz, DMSO-d6, 120 °C) δ 156.0 (q, J = 34.8 Hz), 140.1, 136.5, 133.9, 132.5, 127.6, 116.9 (q, J = 288.7 Hz), 84.3, 46.5, 42.4, 32.9, 28.0, 24.9, 13.3. HRMS: m/z: $[M + H]^+$ calc'd for $[C_{19}H_{27}BBrF_3NO_3]^+$ expect 464.1219; found 464.1201.

Para ^{1}H NMR (500 MHz, DMSO-d_6, 120 °C) δ 7.84 (s, 1H), 7.62 (d, J = 7.5 Hz, 1H), 7.36 (d, J = 7.5 Hz, 1H), 3.53 - 3.44 (m, 4H), 2.79 (t, J = 7.8 Hz, 2H), 1.94 (quint, J = 7.6 Hz, 2H), 1.33 (s), 1.19 (t, J = 7.1 Hz, 3H); ^{13}C NMR (126 MHz, DMSO-d6, 120 °C) δ 156.0 (q, J = 34.8 Hz), 143.7, 138.5, 134.1, 130.4, 124.0, 116.9 (q, J = 288.7 Hz), 84.3, 46.5, 42.4, 33.2, 28.0, 24.9, 13.3.

Synthesis of Bipyridine Ligands

General procedure A for the preparation benzyl substituted bipyridines from 4,4'-dimethyl-2,2'-bipyridine:

Di-isopropylamine (1.05 eq) was dissolved in THF (0.5 M) and cooled to 0 °C before the dropwise addition of *n*-BuLi (0.95 eq). The resulting solution was stirred for 30 mins at 0 °C before the quick addition of a solution of 4,4'-dimethyl-2,2'-bipyridine (1 eq) in THF (0.14 M). The reaction was warmed to RT and stirred for 2 hours before cooling to – 78 °C and the appropriate electrophile (1.1 – 2.5 eq dissolved in THF if required) added quickly. The reaction was quenched at the specified temperature after a specified time (water or EtOH). Ethyl acetate was added and the aqueous layer separated and further extracted with 2 portions of ethyl acetate. The organic layers were combined, dried ($MgSO_4$) and solvents removed *in vacuo*. The crude product was subjected to column chromatography.

General procedure B for the preparation benzyl substituted bipyridines from 5,5'-dimethyl-2,2'-bipyridine:

Di-isopropylamine (1.2 eq) was dissolved in THF (3.3 M) and cooled to – 78 °C before the dropwise addition of *n*-BuLi (1.1 eq). The resulting solution was stirred for 30 mins at – 78 °C before the quick addition of a solution of 5,5'-dimethyl-2,2'-bipyridine (1 eq) in THF (0.18 M) and stirred for 2 hours at this temperature. The appropriate electrophile (1.1 – 2.5 M) was added in a drop-wise fashion and the reaction was quenched at the specified temperature after a specified time (water of EtOH). Ethyl acetate was added and the aqueous layer separated and further extracted with 2 portions of ethyl acetate. The organic layers were combined, dried ($MgSO_4$) and solvents removed *in vacuo*. The crude product was subjected to column chromatography.

Tetrabutylammonium (5'-methyl-[2,2'-bipyridin]-5-yl)methanesulfonate (1a)

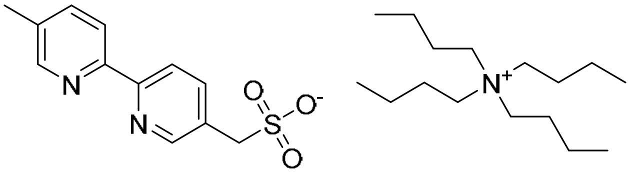

Prepared according our previous publication.[1]

Tetrabutylammonium 2-(5'-methyl-[2,2'-bipyridin]-5-yl)ethane-1-sulfonate (1b)

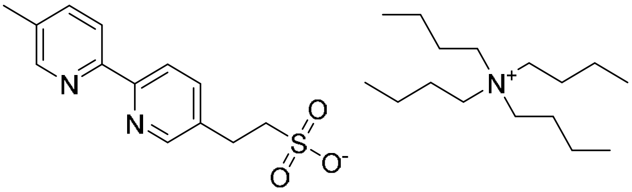

Prepared according our previous publication[1]

((5'-methyl-[2,2'-bipyridin]-5-yl)methyl)diphenylphosphine oxide (1c)

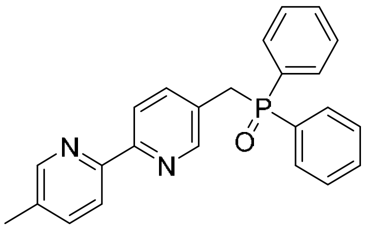

Following general procedure B with 5,5'-dimethyl-2,2'-bipyridine (307 mg, 1.69 mmol), diphenylphosphine chloride (0.37 mL, 2.0 mmol) in THF (2 mL), stirred at - 78 °C for 10 mins. The cooling bath was removed and the mixture stirred for a further 5 min before quenching with water (2 mL) and further warming to rt. The mixture was extracted with EtOAc as described and crude purified by column chromatography (CH_2Cl_2:MeOH (39:1)) to yield 1c as a white solid (250 mg, 0.65 mmol, 38%).

^{1}H NMR (600 MHz, $CDCl_3$) δ 8.47 (br s, 1H), 8.34 (br s, 1H), 8.21 (d, *J* = 8.8 Hz, 1H), 8.19 (d, *J* = 8.8 Hz, 1H), 7.75-7.71 (m, 4H), 7.67 (dt, *J* = 8.1, 2.1 Hz, 1H), 7.59 (dd, *J* = 1.7, 7.8 Hz, 1H), 7.53 (dt, *J* = 1.2, 7.5 Hz, 2H), 7.48-7.45 (m, 4H), 3.69 (d, *J* = 13.6 Hz, 2H), 2.38 (s, 3H); **^{13}C NMR** (151 MHz, $CDCl_3$) δ 154.9 (d, $^{4}J_{C-P}$ = 3.0 Hz), 153.3, 150.0 (d, $^{3}J_{C-P}$ = 6.7 Hz), 149.6, 138.4 (d, $^{3}J_{C-P}$ = 4.8 Hz), 137.4, 133.3, 132.1 (d, $^{4}J_{C-P}$ = 2.8 Hz), 131.8 (d, $^{1}J_{C-P}$ = 99.9 Hz), 131.0 (d, $^{2}J_{C-P}$ = 9.3 Hz), 128.7 (d, $^{2}J_{C-P}$ = 11.3 Hz), 127.2 (d, $^{3}J_{C-P}$ = 8.5 Hz), 120.5, 120.4 (d, $^{4}J_{C-P}$ = 2.4 Hz), 35.2 (d, $^{1}J_{C-P}$ = 66.5 Hz), 18.3; **HRMS** m/z: $[M + H]^+$ calc'd for $[C_{24}H_{22}N_2OP]^+$ expect 385.1460; found 385.1453.

(2-(5'-methyl-[2,2'-bipyridin]-5-yl)ethyl)diphenylphosphine oxide (1d)

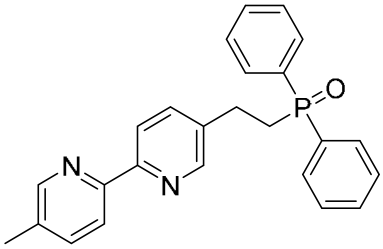

Diphenyl phosphine oxide (152 mg, 0.75 mmol) was dissolved in THF (5 mL), cooled to 0 °C and NaH (50 mg, 1.25 mmol, 1.7 eq) added portionwise. The resulting mixture was warmed to rt and stirred for 1 h before the dropwise addition of bromide 5-(2-bromoethyl)-5'-methyl-2,2'-bipyridine[1] (220 mg, 0.79 mmol) in THF (5 mL). The reaction was quenched with water (0.5 mL) and the solvents removed in *vacuo*. The crude product was purified by silica gel chromatography (CH_2Cl_2 : MeOH (39:1)) to yield phosphine oxide 1d as a white, powdery solid (144 mg, 0.36 mmol, 48%).

^{1}H NMR (600 MHz, $CDCl_3$) δ 8.48 (d, *J* = 1.9 Hz, 1H), 846 (d, *J* = 1.9 Hz, 1H), 8.23 (d, *J* = 7.9 Hz, 1H), 8.22 (d, *J* = 7.9 Hz, 1H), 7.79-7.76 (m, 4H), 7.61 (ddd, *J* = 1.6, 5.5, 7.7 Hz, 2H), 7.55-7.52 (m, 2H), 7.50-7.47 (m, 4H), 3.02-2.98 (m, 2H), 2.63-2.58 (m, 2H), 2.38 (s, 3H); **^{13}C NMR** (151 MHz, $CDCl_3$) δ 154.6, 153.4, 149.6, 148.9, 137.4, 136.4, 136.2 (d, $^{2}J_{C-P}$ = 14.7 Hz), 133.3, 132.5 (d, $^{1}J_{C-P}$ = 99.3 Hz), 131.9 (d, $^{4}J_{C-P}$ = 2.7 Hz), 130.7 (d, $^{3}J_{C-P}$ = 9.3 Hz), 128.8 (d, $^{3}J_{C-P}$ = 11.8 Hz), 120.5, 120.4, 31.5 (d, $^{1}J_{C-P}$ = 69.6 Hz), 24.8 (d, $^{2}J_{C-P}$ = 2.8 Hz), 18.3; **HRMS** m/z: $[M + H]^+$ calc'd for $[C_{25}H_{24}N_2OP]^+$ expect 399.1616; found 399.1604.

5-Methyl-5'-((phenylsulfinyl)methyl)-2,2'-bipyridine (1e)

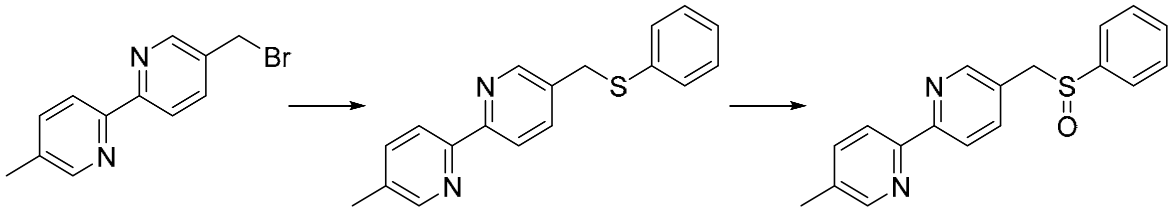

To 5-(2-bromoethyl)-5'-methyl-2,2'-bipyridine[1] (262 mg, 1.0 mmol) added PhSH (110 mg, 1.0 mmol) and Na_2CO_3 (159 mg, 1.5 mmol) in MeOH. After 30 mins solvents were removed and the residue redissolved in CH_2Cl_2. The organic layer was washed with 5% aq NaOH then brine, dried ($MgSO_4$), the solvent evaporated and the product taken directly through to the next step.

*m*CPBA (dried on high vac at rt for 30 mins, assume 85%) (101 mg, 0.5 mmol) was added to the sulphide redissolved in CH_2Cl_2 (3 mL) and cooled to 0 °C. The mixture was stirred for 1 h at the same temperature before the solvents were removed *in vacuo* and the product purified by silica gel chromatography with EtOAc to yield 1e as a white solid (92 mg, 0.3 mmol, 30%).

^{1}H NMR (500 MHz, $CDCl_3$) δ 8.52 (br s, 1H), 8.33 (d, *J* = 8.2 Hz, 1H), 8.28 (d, *J* = 1.9 Hz, 1H), 8.26 (d, *J* = 8.1 Hz, 1H), 7.70-7.61 (m, 5H), 7.50-7.46 (m, 2H), 4.36 (s, 2H), 2.41 (s, 3H); **^{13}C NMR** (126 MHz, $CDCl_3$) δ 156.2, 152.6, 150.7, 149.4, 139.0, 137.9, 137.5, 134.2, 129.2, 128.6, 124.1, 121.1, 120.6, 60.0, 18.4; **HRMS** m/z: $[M + Na]^+$ calc'd for $[C_{18}H_{17}N_2OSNa]^+$ expect 331.0870; found 331.0868.

5-Methyl-5'-(2-(phenylsulfinyl)ethyl)-2,2'-bipyridine (1f)

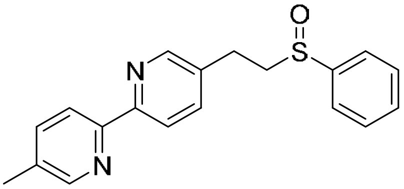

To a solution of di-idopropylamine (0.09 ml, 0.66 mmol) in THF (5 mL) cooled to -78 °C was added nBuLi (0.41 mL, 0.66 mmol) dropwise over 5 mins and stirred for 15 mins at the same temperature. Methylphenyl sulfoxide (200 mg, 0.62 mmol) in THF (3 mL) was added over 5 mins and the reaction was warmed to 0 °C over 1 h. The mixture was cooled again to -78 °C and 5-(bromomethyl)-5'-methyl-2,2'-bipyridine[1] (163 mg, 0.62 mmol) in THF (20 mL) was added dropwise over 15 mins and stirred at the same temperature for 1 h before warming to rt and quenching with water (10 mL). EtOAc (20 mL) was added and the aqueous separated and extracted further with EtOAc (2 x 20 mL). The combined organic layers were dried ($MgSO_4$), concentrated *in vacuo* and purified by column chromatography (CH_2Cl_2:MeOH (19:1)) to yield 1f as an off white solid (80 mg, 0.25 mmol, 40%).

^{1}H NMR (600 MHz, $CDCl_3$) δ 8.44 (br s, 1H), 8.24 (d, *J* = 8.1 Hz, 1H), 8.19 (d, *J* = 8.1 Hz, 1H), 7.61-7.60 (m, 2H), 7.57 (dt, *J* = 2.3, 8.0 Hz, 2H), 7.51-7.45 (m, 3H), 7.13 (br d, *J* = 2.8 Hz, 2H), 3.16-3.06 (m, 2H), 3.03-2.98 (m, 1H), 2.89 (ddd, 5.3, 10.2, 14.5 Hz, 1H), 2.33 (s, 3H); **^{13}C NMR** (151 MHz, $CDCl_3$) δ 154.9, 153.2, 149.6, 149.2, 143.2, 137.4, 136.9, 134.0, 133.4, 131.1, 129.3, 124.0, 120.6, 120.5, 57.4, 25.0, 18.3; **HRMS** m/z: $[M + H]^+$ calc'd for $[C_{19}H_{19}N_2OS]^+$ expect 323.1209; found 323.1212.

2-(5'-Methyl-[2,2'-bipyridin]-5-yl)acetic acid

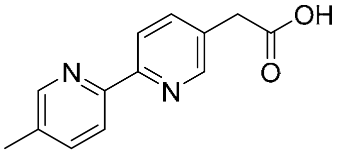

Di-isopropylamine (1.61 mL, 13.0 mmol) was dissolved in THF (5 mL) and cooled to – 78 °C before the dropwise addition of *n*-BuLi (7.04 mL 11.3 mmol). The resulting solution was stirred for 30 mins at – 78 °C before the quick addition of a solution of 5,5'-dimethyl-2,2'-bipyridine (2.0 g, 10.9 mmol) in THF (25 mL) and stirred for 2 hours at this temperature. CO_2 was bubbled through at the same temperature for 1 h and the reaction warmed to rt with CO_2 still being bubbled through. Water (ca. 5 drops) was added to quench the reaction and solvents were removed *in vacuo.* The residue was taken up in 1M NaOH (50 mL), washed with hexane (50 mL), and the organic phase extracted with 1M NaOH (2 x 50 mL). The acidity of the combined aqueous phases was adjusted to pH = 5 by the addition of 3M HCl, and the solvents removed *in vacuo* once more. The resulting solid was extracted into MeOH (50 mL), dried *in vacuo* and recrystallised from EtOH with addition of Pet. Ether (40-60) to yield a white solid (1.39 g, 6.09 mmol, 56%).

^{1}H NMR (600 MHz, DMSO-d_6) δ 8.52 (d, *J* = 1.9 Hz, 1H), 8.50 (d, *J* = 1.9 Hz, 1H), 8.28 (d, *J* = 8.1 Hz, 1H), 8.25 (d, *J* = 8.1 Hz, 1H), 7.80 (d, *J* = 2.1, 8.1 Hz, 1H), 7.73 (d, *J* = 2.0, 8.1 Hz, 1H), 3.68 (s, 2H), 2.34 (s, 3H); **^{13}C NMR** (151 MHz, DMSO-d_6) δ 172.7, 154.2, 153.2, 150.4, 149.9, 138.6, 138.0, 133.9, 131.6, 120.3, 120.0, 38.1, 18.3.

Preparation adapted from and data in accordance with those previously reported.[15]

***N,N*-diethyl-2-(5'-methyl-[2,2'-bipyridin]-5-yl)acetamide (1g)**

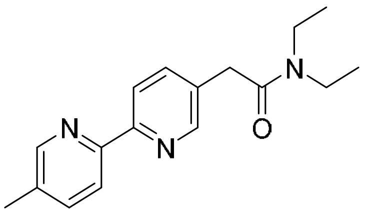

2-(5'-Methyl-[2,2'-bipyridin]-5-yl)acetic acid (228 mg, 1.0 mmol) was dissolved in DMF (2 mL), followed by the addition of EDCl.HCl (211 mg, 1.1 mmol) and HOBt (149 mg, 1.1 mmol) and the mixture stirred at rt for 30 mins. Diethylamine (0.11 mL, 1.1 mmol) was added followed by DIPEA (0.37 mmol, 2.1 mmol) and the reaction stirred for a further 16 h at rt. Water (2 mL) was added and the aqueous extracted with EtOAc (3 x 10 mL). The combined organic layers were washed with water (2 x 5 mL), dried ($MgSO_4$) solvents removed *in vacuo* and the crude purified by column chromatography (CH_2Cl_2:MeOH (49:1)) to yield 1g as white crystals (124 mg, 0.44 mmol, 44%).

^{1}H NMR (600 MHz, $CDCl_3$) δ 8.49 (d, *J* = 1.9 Hz, 1H), 8.44 (d, *J* = 1.5 Hz, 1H), 8.27 (d, *J* = 8.1 Hz, 1H), 8.21 (d, *J* = 8.1 Hz, 1H), 7.71 (d, *J* = 2.2, 8.2 Hz, 1H), 7.56 (dd, *J* = 1.6, 8.1 Hz, 1H), 3.68 (s, 2H), 3.36 (q, *J* = 7.2 Hz, 2H), 3.29 (q, *J* = 7.2 Hz, 2H), 2.33 (s, 3H), 1.11 (t, *J* = 7.2 Hz, 3H), 1.08 (t, *J* = 7.2 Hz, 3H); **^{13}C NMR** (151 MHz, $CDCl_3$) δ 169.0, 154.9, 153.4, 149.6, 149.3, 137.39, 137.36, 133.2, 1301.0, 120.5, 120.4, 42.4,

40.3, 37.5, 18.3, 14.4, 12.9; **HRMS** m/z: [M + H]$^+$ calc'd for [$C_{17}H_{22}N_3O$]$^+$ expect 284.1753; found 284.1763.

N,N-diethyl-3-(5'-methyl-[2,2'-bipyridin]-5-yl)propanamide (1h)

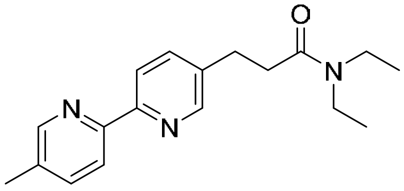

Following general procedure B with 5,5'-dimethyl-2,2'-bipyridine (307 mg, 1.69 mmol), 2-bromo-*N*,*N*-diethylacetamide[16] (322 mg, 1.66 mmol)in THF (2 mL), stirred at - 78 °C for 30 mins and water (4 mL) added at this temperature and the reaction allowed to warm to rt with rapid stirring. The aqueous was instead extracted in CH_2Cl_2 and concentrated to ca. 3 mL. Pet. Ether (40-60) was added and the precipate removed by filtration. The filtrate was further dried ($MgSO_4$) and solvents removed *in vacuo* and purified by column chromatography (CH_2Cl_2:Acetone (8:2-6:4)) to yield 1h as a yellow oil (110 mg, 0.37 mmol, 26%).

^{1}H NMR (600 MHz, $CDCl_3$) δ 8.53 (d, *J* = 1.8 Hz, 1H), 8.48 (d, *J* = 1.6 Hz, 1H), 8.25 (d, *J* = 8.1 Hz, 1H), 8.23 (d, *J* = 8.1 Hz, 1H), 7.68 (d, *J* = 2.3, 8.2 Hz, 1H), 7.61 (dd, *J* = 1.7, 8.1 Hz, 1H), 3.68 (s, 2H), 3.37 (q, *J* = 7.2 Hz, 2H), 3.23 (q, *J* = 7.2 Hz, 2H), 3.04 (t, *J* = 7.8 Hz, 2H), 2.63 (t, *J* = 7.8 Hz, 2H), 2.38 (s, 3H), 1.10 (t, *J* = 7.2 Hz, 6H); **^{13}C NMR** (151 MHz, $CDCl_3$) δ 154.3, 153.6, 149.6, 149.2, 137.5, 137.1, 136.8, 133.2, 120.5, 120.4, 41.9, 40.3, 34.5, 28.4, 18.4, 14.3, 13.1; **HRMS** m/z: [M + H]$^+$ calc'd for [$C_{18}H_{24}N_3O$]$^+$ expect 289.1909; found 289.1906.

Tetrabutylammonium (4'-methyl-[2,2'-bipyridin]-4-yl)methanesulfonate (1i)

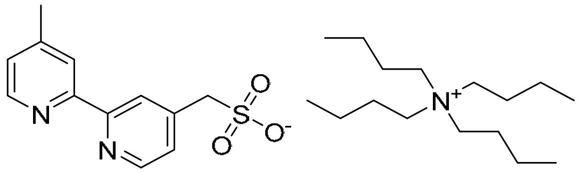

Prepared according our previous publication.[1]

Tetrabutylammonium 2-(4'-methyl-[2,2'-bipyridin]-4-yl)ethane-1-sulfonate (1j)

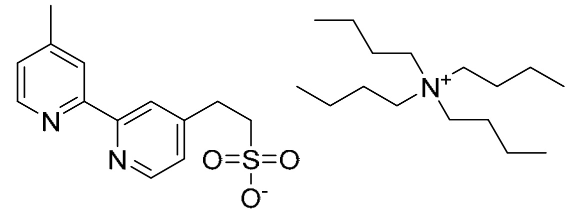

Prepared according our previous publication.[1]

((4'-Methyl-[2,2'-bipyridin]-4-yl)methyl)diphenylphosphine oxide (1k)

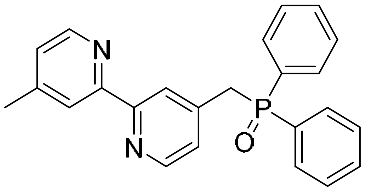

Following general procedure A with 4,4'-dimethyl-2,2'-bipyridine (307 mg, 1.69 mmol), diphenylphosphinic chloride (0.38 mL, 2.0 mmol) in THF (2 mL), stirred at - 78 °C for 30 mins then warmed to rt and stirred for a further 18 h. The reaction was quenched with water (5 mL) and purified by column chromatography (CH_2Cl_2:Acetone:MeOH (1:1:0-10:10:1)) to yield 1k as a white solid (73 mg, 0.19 mmol, 12%).

^{1}H NMR (600 MHz, $CDCl_3$) δ 8.49 (d, *J* = 5.2 Hz, 1H), 8.48 (d, *J* = 5.2 Hz, 1H), 8.14 (s, 1H), 8.11 (s, 1H), 7.75-7.71 (m, 4H), 7.51 (dt, *J* = 1.2, 7.3 Hz, 2H), 7.47-7.44 (m, 4H), 7.21 (dt, *J* = 5.1, 2.3 Hz, 2H) 7.11 (d, *J* = 5.0 Hz, 1H), 3.73 (d, *J* = 13.7 Hz, 2H), 2.41 (s, 3H); **^{13}C NMR** (151 MHz, $CDCl_3$) δ 156.0, 155.4, 149.0 (d, $^{4}J_{C-P}$ = 2.1 Hz), 148.8, 148.2, 141.8 (d, $^{3}J_{C-P}$ = 7.6 Hz), 132.1 (d, $^{4}J_{C-P}$ = 2.5 Hz), 131.7 (d, $^{1}J_{C-P}$ = 99.9 Hz), 131.0 (d, $^{2}J_{C-P}$ = 9.1 Hz), 128.7 (d, $^{2}J_{C-P}$ = 11.9 Hz), 125.0 (d, $^{3}J_{C-P}$ = 4.9 Hz), 124.7, 122.9 (d, $^{3}J_{C-P}$ = 5.6 Hz), 122.0, 37.9 (d, $^{1}J_{C-P}$ = 63.8 Hz), 21.2; **HRMS** m/z: $[M + H]^+$ calc'd for $[C_{24}H_{22}N_2OP]^+$ expect 385.1460; found 385.1464.

(2-(4'-methyl-[2,2'-bipyridin]-4-yl)ethyl)diphenylphosphine oxide (1l)

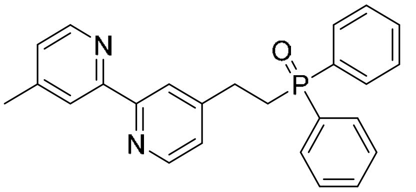

Diphenyl phosphine oxide (310 mg, 1.12 mmol) was dissolved in THF (5 mL), cooled to 0 °C and NaH (76 mg, 1.90 mmol, 1.7 eq) added portionwise. The resulting mixture was warmed to rt and stirred for 1 h before the dropwise addition of bromide 4-(2-bromoethyl)-4'-methyl-2,2'-bipyridine[1] (310 mg, 1.12 mmol) in THF (5 mL). The reaction was quenched with water (0.5 mL) and the solvents removed in *vacuo*. The crude product was purified by silica gel chromatography (CH_2Cl_2 : MeOH (39:1)) to yield phosphine oxide 1l as a white, powdery solid (390 mg, 0.98 mmol, 87%).

^{1}H NMR (600 MHz, $CDCl_3$) δ 8.52 (d, *J* = 5.0 Hz, 1H), 8.50 (d, *J* = 5.0 Hz, 1H), 8.22 (br s, 1H), 8.20 (br s, 1H), 7.79-7.75 (m, 4H), 7.52 (dt, *J* = 1.4, 7.5 Hz, 2H), 7.48-7.45 (m, 4H), 7.13 (app dd, *J* = 1.1, 5.0 Hz) , 3.03-2.99 (m, 2H), 2.68-2.63 (m, 2H), 2.42 (s, 3H); **^{13}C NMR** (151 MHz, $CDCl_3$) δ 156.2, 155.5, 151.1 (d, $^{2}J_{C-P}$ = 15.5 Hz), 149.3, 148.7, 148.5, 132.4 (d, $^{1}J_{C-P}$ = 99.5 Hz), 132.0 (d, $^{4}J_{C-P}$ = 2.7 Hz), 130.8 (d, $^{3}J_{C-P}$ = 9.3 Hz), 128.8 (d, $^{3}J_{C-P}$ = 11.6 Hz), 124.8, 123.7, 122.2, 120.6, 30.6 (d, $^{1}J_{C-P}$ = 70.5 Hz), 27.2 (d, $^{2}J_{C-P}$ = 2.8 Hz), 21.2; **HRMS** m/z: $[M + H]^+$ calc'd for $[C_{25}H_{24}N_2OP]^+$ expect 399.1616; found 399.1608.

4-Methyl-4'-((phenylthio)methyl)-2,2'-bipyridine

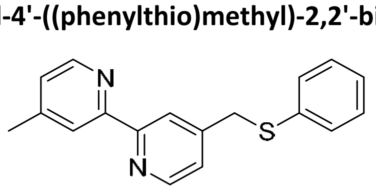

Following general procedure A using 4,4'-dimethyl-2,2'-bipyridine (250 mg, 1.36 mmol), phenyldisulfide (620 mg, 2.84 mmol) in THF (3 mL), stirred at -78 °C for 1 h before warming to rt and

stirred for a further 2 h before cooling to 0 °C and quenching with water (1 mL). Purification by column chromatography (CH_2Cl_2:MeOH (95:5)) yielded the title compound as an off white solid (181 mg, 0.62 mmol, 37%).

^{1}H NMR (600 MHz, $CDCl_3$) δ 8.52 (d, *J* = 4.8 Hz, 1H), 8.49 (d, *J* = 4.8 Hz, 1H), 8.33 (s, 1H), 8.19 (s, 1H), 7.27 (d, *J* = 7.8 Hz, 2H), 7.20-7.17 (m, 3H), 7.13 (t, *J* = 7.4 Hz, 1H), 7.07 (d, *J* = 4.8 Hz, 1H), 4.09 (s, 2H), 2.36 (s, 3H) ; **^{13}C NMR** (151 MHz, $CDCl_3$) δ 156.4, 155.6, 149.2, 149.0, 148.0, 147.8, 135.2, 130.2, 128.97, 126.8, 124.8, 123.5, 122.0, 121.3, 38.2, 21.2; **HRMS** m/z: $[M + H]^+$ calc'd for $[C_{18}H_{17}N_2S]^+$ expect 293.1104; found 293.1111.

4-Methyl-4'-((phenylsulfinyl)methyl)-2,2'-bipyridine (1m)

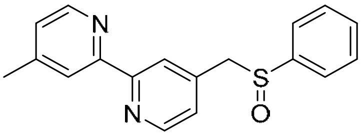

4-Methyl-4'-((phenylthio)methyl)-2,2'-bipyridine (132 mg, 0.45 mmol) was dissolved in CH_2Cl_2 (6 mL) and cooled to 0 °C. mCPBA (dried under vacuum for 1 hour assume ~89%) (111 mg, 0.45 mmol) was added in portions and the resulting solution stirred for 2 hours at the same temperature. The reaction was concentrated *in vacuo* and purified by silica gel chromatography (EtOAc:MeOH (10:0-9:1) to yield bipyridine 1m as a white powder (66 mg 0.21 mmol, 39%).

^{1}H NMR (600 MHz, $CDCl_3$) δ 8.56 (d, *J* = 5.0 Hz, 1H), 8.51 (d, *J* = 5.0 Hz, 1H), 8.21 (s, 1H), 8.09 (s, 1H), 7.46-7.45 (m, 5H), 7.15 (br d, *J* = 4.4 Hz, 1H), 6.98 (dd, *J* = 1.5, 5.0 Hz, 1H), 4.10 (d, *J* = 13.1 Hz, 1H), 4.08 (d, *J* = 13.1 Hz, 1H), 2.45 (s, 3H) ; **^{13}C NMR** (151 MHz, $CDCl_3$) δ 156.5, 155.2, 149.1, 149.0, 148.2, 142.4, 139.2, 131.5, 129.1, 125.0, 124.9, 124.2, 122.5, 122.0, 62.6, 21.3; **HRMS** m/z: $[M + H]^+$ calc'd for $[C_{18}H_{17}N_2SO]^+$ expect 309.1053; found 309.1065.

4-Methyl-4'-(2-(phenylthio)ethyl)-2,2'-bipyridine

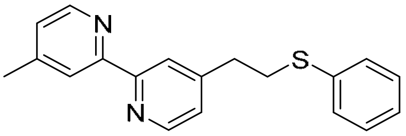

To 4-(2-bromoethyl)-4'-methyl-2,2'-bipyridine[1] (277 mg, 1.0 mmol) dissolved in MeOH (3 mL) was added thiobenzene (110 mg, 1.0 mmol) and Na_2CO_3 (159 mg, 1.5 mmol) and the reaction stirred for 18 hours at rt. The solvent was subsequently blown off with air and the solid re-dissolved in CH_2Cl_2 (3 mL) and washed with 5% NaOH (2 mL), dried ($MgSO_4$) and solvent removed *in vacuo*. The crude product was purified by silica gel chromatography (CH_2Cl_2 : MeOH (19 : 1)) to yield the title compound as a white crystalline solid (212 mg, 0.73 mmol, 73%).

^{1}H NMR (600 MHz, $CDCl_3$) δ 8.57 (d, *J* = 5.0 Hz, 1H), 8.53 (d, *J* = 4.9 Hz, 1H), 8.24 (d, *J* = 8.9 Hz, 2H), 7.38 (dd, *J* = 0.8, 7.4 Hz, 2H), 7.30 (t, *J* = 7.7 Hz, 2H), 7.20 (t, *J* = 7.0 Hz, 1H), 7.13 (br d, *J* = 2.8 Hz, 2H), 3.23 (app t, *J* = 7.9 Hz, 2H), 3.00 (app t, *J* = 7.6 Hz, 2H), 2.43 (s, 3H); **^{13}C NMR** (151 MHz, $CDCl_3$) δ 156.4, 155.8, 150.0, 149.2, 148.9, 148.2, 135.6, 129.8, 129.0, 126.4, 124.8, 123.9, 122.1, 121.2, 35.2, 34.1, 21.2; **HRMS** m/z: $[M + H]^+$ calc'd for $[C_{19}H_{19}N_2S]^+$ expect 307.1260; found 307.1263.

4-Methyl-4'-(2-(phenylsulfinyl)ethyl)-2,2'-bipyridine (1n)

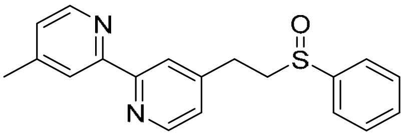

4-Methyl-4'-(2-(phenylthio)ethyl)-2,2'-bipyridine (310 mg, 1.0 mmol) was dissolved in CH_2Cl_2 (4 mL) and cooled to 0 °C. mCPBA (dried under vacuum for 1 hour assume ~89%) (201 mg, 1.0 mmol) was added in portions and the resulting solution stirred for 2 hours at the same temperature. The reaction was concentrated *in vacuo* and purified by silica gel chromatography (EtOAc) to yield 1n as a white powder (210 mg 0.66 mmol, 65%).

^{1}H NMR (600 MHz, $CDCl_3$) δ 8.57 (d, *J* = 4.9 Hz, 1H), 8.51 (d, *J* = 5.0 Hz, 1H), 8.21 (d, *J* = 4.8 Hz, 1H) 8.65-8.63 (m, 2H), 8.55-8.49 (m, 3H), 7.15-7.13 (m, 2H), 3.22-3.16 (m, 2H), 3.11-3.06 (m, 1H), 2.97-2.91 (m, 1H), 2.43 (s, 3H); **^{13}C NMR** (151 MHz, $CDCl_3$) δ 156.5, 155.5, 149.4, 148.89, 148.88, 148.3, 143.1, 131.2, 129.4, 124.9, 124.0, 123.9, 122.1, 121.1, 56.5, 27.4, 21.2; **HRMS** m/z: $[M + H]^+$ calc'd for $[C_{19}H_{19}N_2OS]^+$ expect 323.1209; found 323.1213.

_N,N_-diethyl-2-(4'-methyl-[2,2'-bipyridin]-4-yl)acetamide (1o)

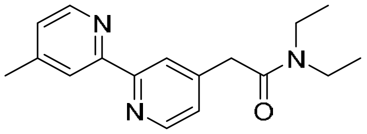

Following general procedure A with 4,4'-dimethyl-2,2'-bipyridine (768 mg, 4.17 mmol), diethylcarbomyl chloride (0.80 mL, 6.0 mmol) in THF (5 mL), stirred at - 78 °C for 30 mins then quenched by the addition of EtOH (3 mL) at – 78 °C and then allowed to warm to rt with rapid stirring. Water (3 mL) was added and the aqueous extracted with EtOAc as described and purified by column chromatography (CH_2Cl_2:MeOH (39:1)) to yield 1o as a pale yellow oil (345 mg, 1.22 mmol, 30%).

^{1}H NMR (600 MHz, $CDCl_3$) δ 8.61 (d, *J* = 5.0 Hz, 1H), 8.52 (d, *J* = 5.0 Hz, 1H), 8.27 (d, *J* = 0.7 Hz, 1H), 8.23 (s, 1H), 7.28 (d, *J* = 1.5, 5.0 Hz, 1H), 7.13 (dd, *J* = 0.7, 4.8 Hz, 1H), 3.77 (s, 2H), 3.41 (q, *J* = 7.1 Hz, 2H), 3.33 (q, *J* = 7.1 Hz, 2H), 2.43 (s, 3H), 1.16 (t, *J* = 7.1 Hz, 3H), 1.14 (t, *J* = 7.1 Hz, 3H); **^{13}C NMR** (151 MHz, $CDCl_3$) δ 168.5, 156.4, 155.7, 149.3, 148.9, 148.2, 145.6, 124.7, 124.1, 122.1, 121.8, 42.5, 40.3, 40.1, 21.2, 14.4, 12.9; **HRMS** m/z: $[M + H]^+$ calc'd for $[C_{17}H_{22}N_3O]^+$ expect 284. 1757; found 284.1757.

_N,N_-diethyl-3-(4'-methyl-[2,2'-bipyridin]-4-yl)propanamide (1p)

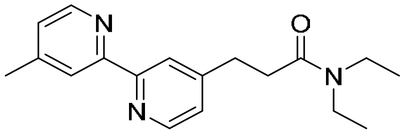

Following general procedure A with 4,4'-dimethyl-2,2'-bipyridine (250 mg, 1.40 mmol), 2-bromo-*N*,*N*-diethylacetamide[16] (588 mg, 2.0 mmol) in THF (2 mL), stirred at - 78 °C for 30 mins then warmed to rt and stirred for a further 18 h. The reaction was quenched with water (5 mL) and purified by column chromatography (CH_2Cl_2:MeOH (19:1)) to yield 1p as a yellow oil (176 mg, 0.59 mmol, 44%).

^{1}H NMR (600 MHz, $CDCl_3$) δ 8.51 (d, *J* = 4.9 Hz, 1H), 8.48 (d, *J* = 4.9 Hz, 1H), 8.22 (s, 1H), 8.17 (s, 1H), 7.16 (dd, *J* = 1.6, 4.9 Hz, 1H), 7.08 (dd, *J* = 0.7, 4.9 Hz, 1H), 3.33 (q, *J* = 6.8 Hz, 2H), 3.21 (q, *J* = 7.3 Hz, 2H), 3.03 (t, *J* = 8.1 Hz, 2H), 2.63(t, *J* = 7.5 Hz, 2H), 2.38 (s, 3H), 1.07 (t, *J* = 7.3 Hz, 3H), 1.06 (t, *J* = 7.3 Hz, 3H); **^{13}C NMR** (151 MHz, $CDCl_3$) δ 170.4, 156.2, 155.9, 151.7, 149.1, 148.8, 148.1, 124.6, 124.1, 122.0, 121.0, 41.8, 40.2, 33.6, 30.9, 21.1, 14.3, 13.0; **HRMS** m/z: $[M + H]^+$ calc'd for $[C_{18}H_{24}N_3O]^+$ expect 289.1909; found 289.1919.

References:

[1] H. J. Davis, M. T. Mihai, R. J. Phipps, *J. Am. Chem. Soc.* **2016**, *138*, 12759-12762.

[2] A. Augurusa, M. Mehta, M. Perez, J. Zhu, D. W. Stephan, *Chem. Commun.* **2016**, *52*, 12195-12198.

[3] T. Nakamura, K. Tateishi, S. Tsukagoshi, S. Hashimoto, S. Watanabe, V. A. Soloshonok, J. L. Aceña, O. Kitagawa, *Tetrahedron* **2012**, *68*, 4013-4017.

[4] M. Miura, C.-G. Feng, S. Ma, J.-Q. Yu, *Org. Lett.* **2013**, *15*, 5258-5261.

[5] J. Liu, X.-R. Zhu, J. Ren, W.-D. Chen, B.-B. Zeng, *Synlett* **2013**, *24*, 2740-2742.

[6] H. Chen, X. Han, N. Qin, L. Wei, Y. Yang, L. Rao, B. Chi, L. Feng, Y. Ren, J. Wan, *Bioorganic & Medicinal Chemistry* **2016**, *24*, 1225-1230.

[7] P. Jakubec, D. M. Cockfield, P. S. Hynes, E. Cleator, D. J. Dixon, *Tetrahedron: Asymmetry* **2011**, *22*, 1147-1155.

[8] W. A. Nack, G. He, S.-Y. Zhang, C. Lu, G. Chen, *Org. Lett.* **2013**, *15*, 3440-3443.

[9] R. Geyer, P. Igel, M. Kaske, S. Elz, A. Buschauer, *MedChemComm* **2014**, *5*, 72-81.

[10] B. Anxionnat, D. Gomez Pardo, G. Ricci, J. Cossy, *Org. Lett.* **2011**, *13*, 4084-4087.

[11] W. Zhou, J. Xu, L. Zhang, N. Jiao, *Org. Lett.* **2010**, *12*, 2888-2891.

[12] H. Dong, M. Shen, J. E. Redford, B. J. Stokes, A. L. Pumphrey, T. G. Driver, *Org. Lett.* **2007**, *9*, 5191-5194.

[13] J.-J. Dai, W.-M. Zhang, Y.-J. Shu, Y.-Y. Sun, J. Xu, Y.-S. Feng, H.-J. Xu, *Chem. Commun.* **2016**, *52*, 6793-6796.

[14] J. Lu, Z.-Z. Guan, J.-W. Gao, Z.-H. Zhang, *Applied Organometallic Chemistry* **2011**, *25*, 537-541.

[15] A. Beyeler, P. Belser, L. D. Cola, *Angew. Chem. Int. Ed.* **1997**, *36*, 2779-2781.

[16] S. L. Riches, C. Saha, N. F. Filgueira, E. Grange, E. M. McGarrigle, V. K. Aggarwal, *J. Am. Chem. Soc.* **2010**, *132*, 7626-7630.

^{1}H NMR (500 MHz, $CDCl_3$) 2,2,2-Trifluoro-*N*-(5-(4,4,5,5-tetramethyl-1,3,2-dioxaborolan-2-yl)-2-(trifluoromethyl)benzyl)acetamide (3a) – Crude after reaction with 1a

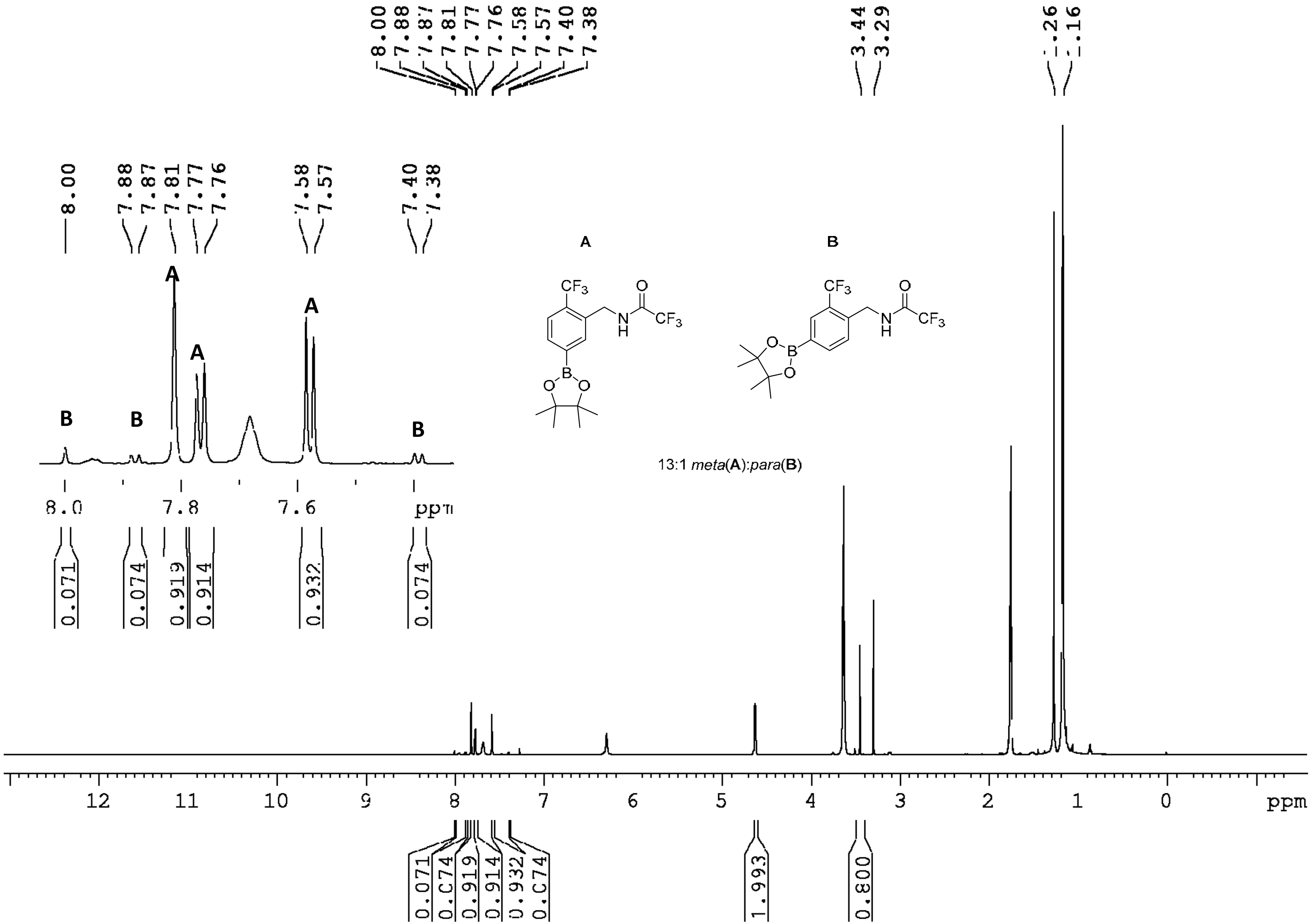

^{1}H NMR (500 MHz, $CDCl_3$) 2,2,2-Trifluoro-*N*-(5-(4,4,5,5-tetramethyl-1,3,2-dioxaborolan-2-yl)-2-(trifluoromethyl)benzyl)acetamide (3a) – >20:1 *meta* fraction after purification from reaction with 1a

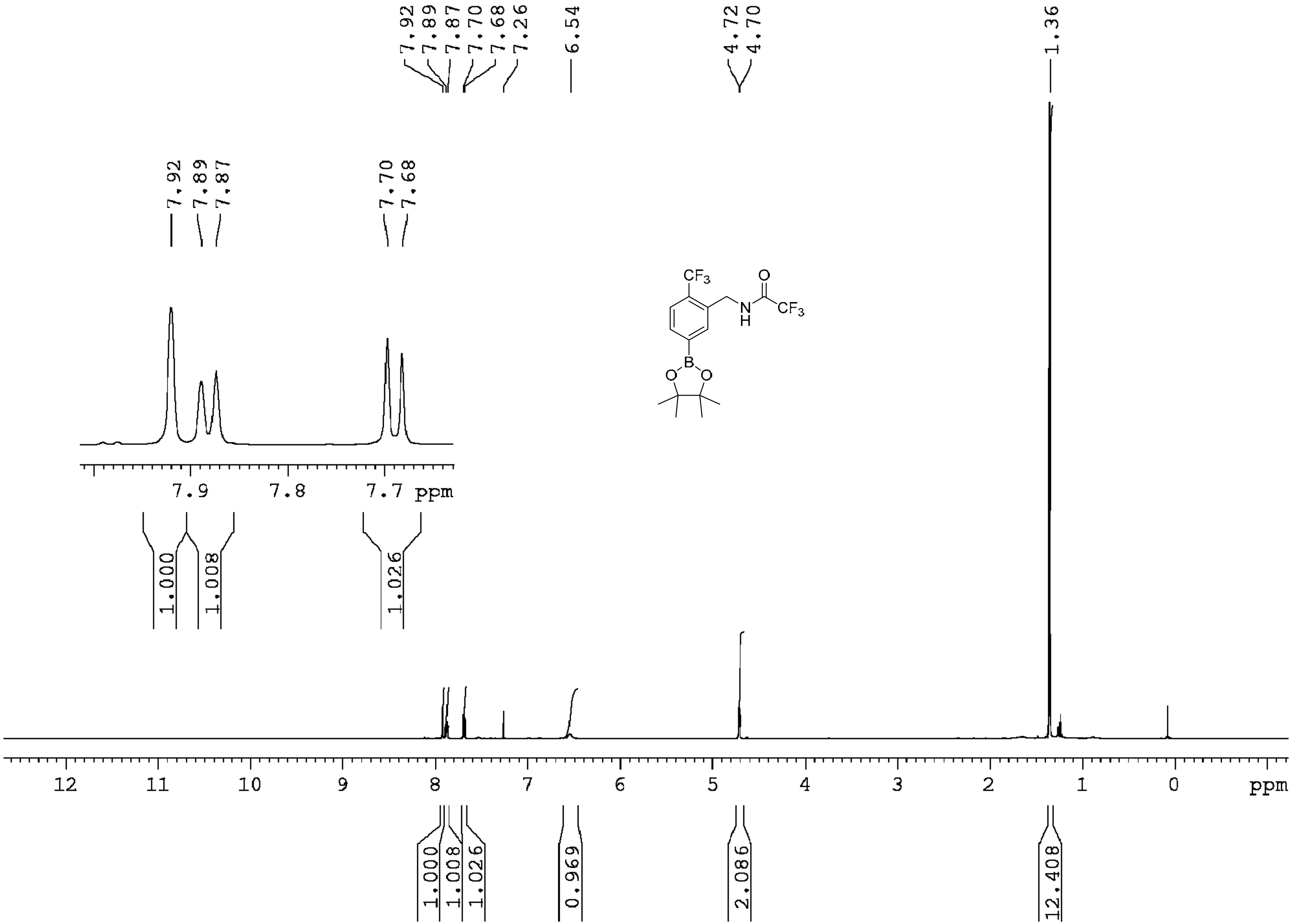

^{13}C NMR (126 MHz, $CDCl_3$) 2,2,2-Trifluoro-*N*-(5-(4,4,5,5-tetramethyl-1,3,2-dioxaborolan-2-yl)-2-(trifluoromethyl)benzyl)acetamide (3a) – Purified after reaction with 1a

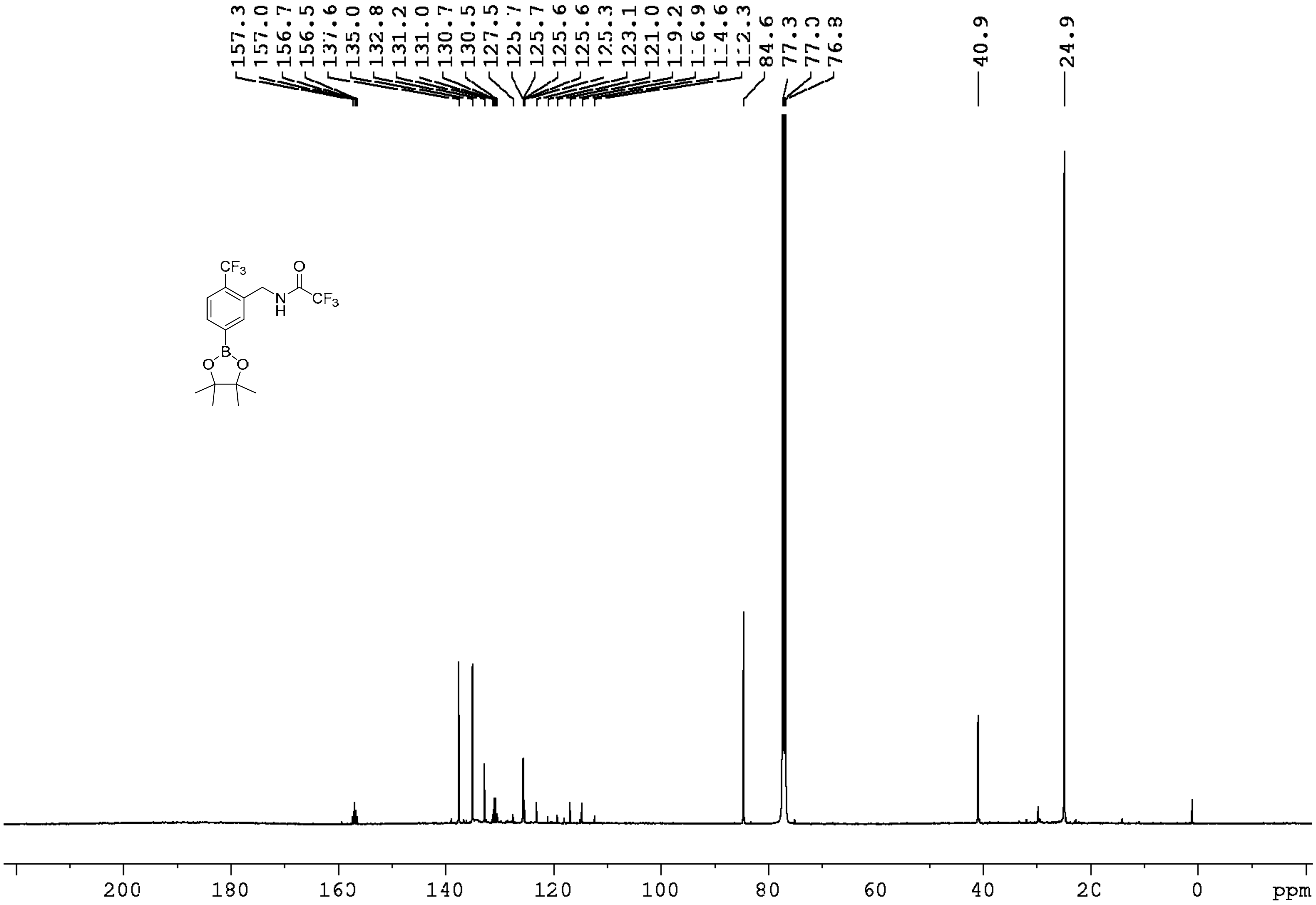

^{1}H NMR (600 MHz, $CDCl_3$) 2,2,2-Trifluoro-*N*-(5-(4,4,5,5-tetramethyl-1,3,2-dioxaborolan-2-yl)-2-(trifluoromethyl)benzyl)acetamide (3a) – Purified after reaction with dtbpy

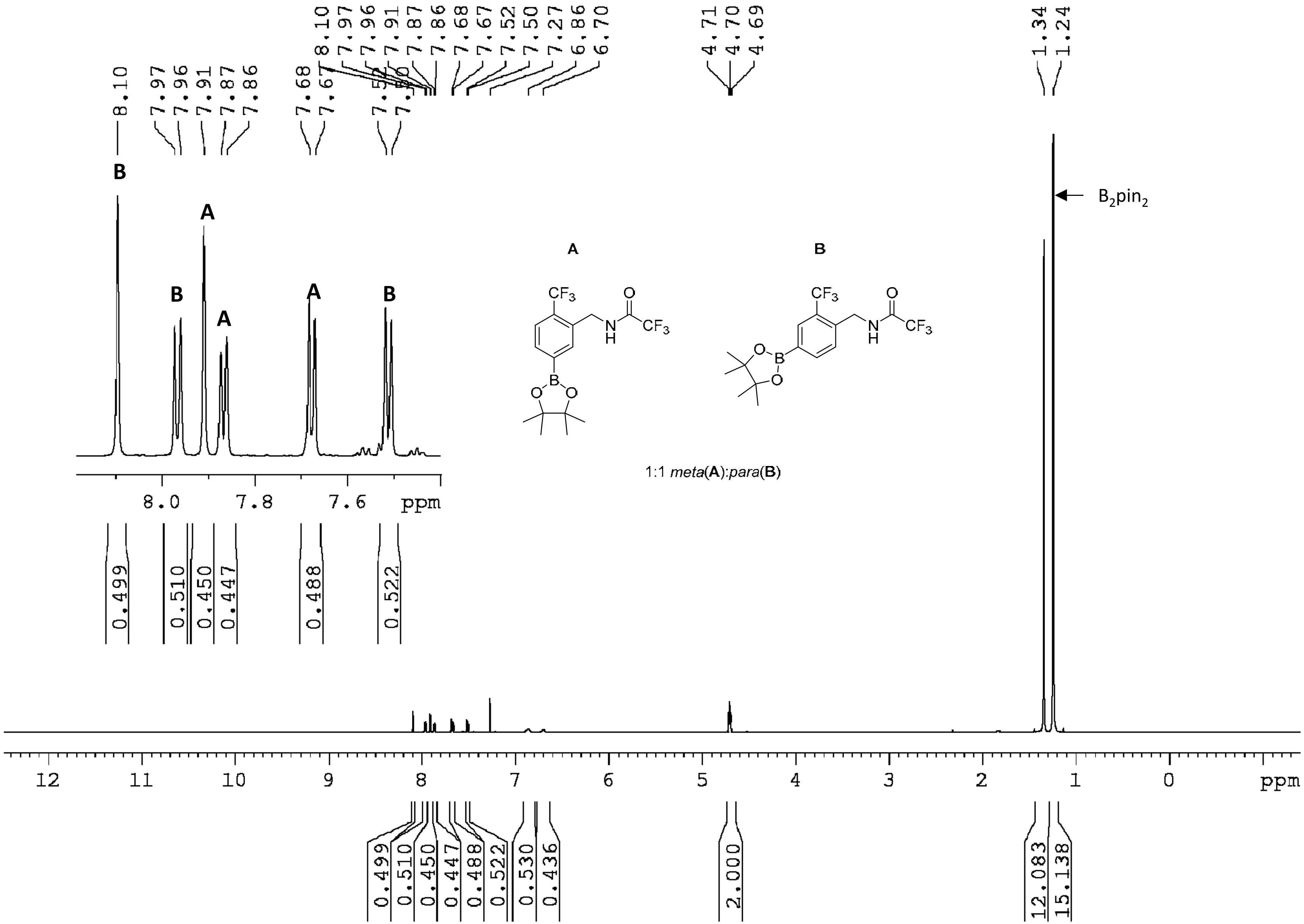

^{13}C NMR (151 MHz, $CDCl_3$) 2,2,2-Trifluoro-*N*-(5-(4,4,5,5-tetramethyl-1,3,2-dioxaborolan-2-yl)-2-(trifluoromethyl)benzyl)acetamide (3a) – Purified after reaction with dtbpy

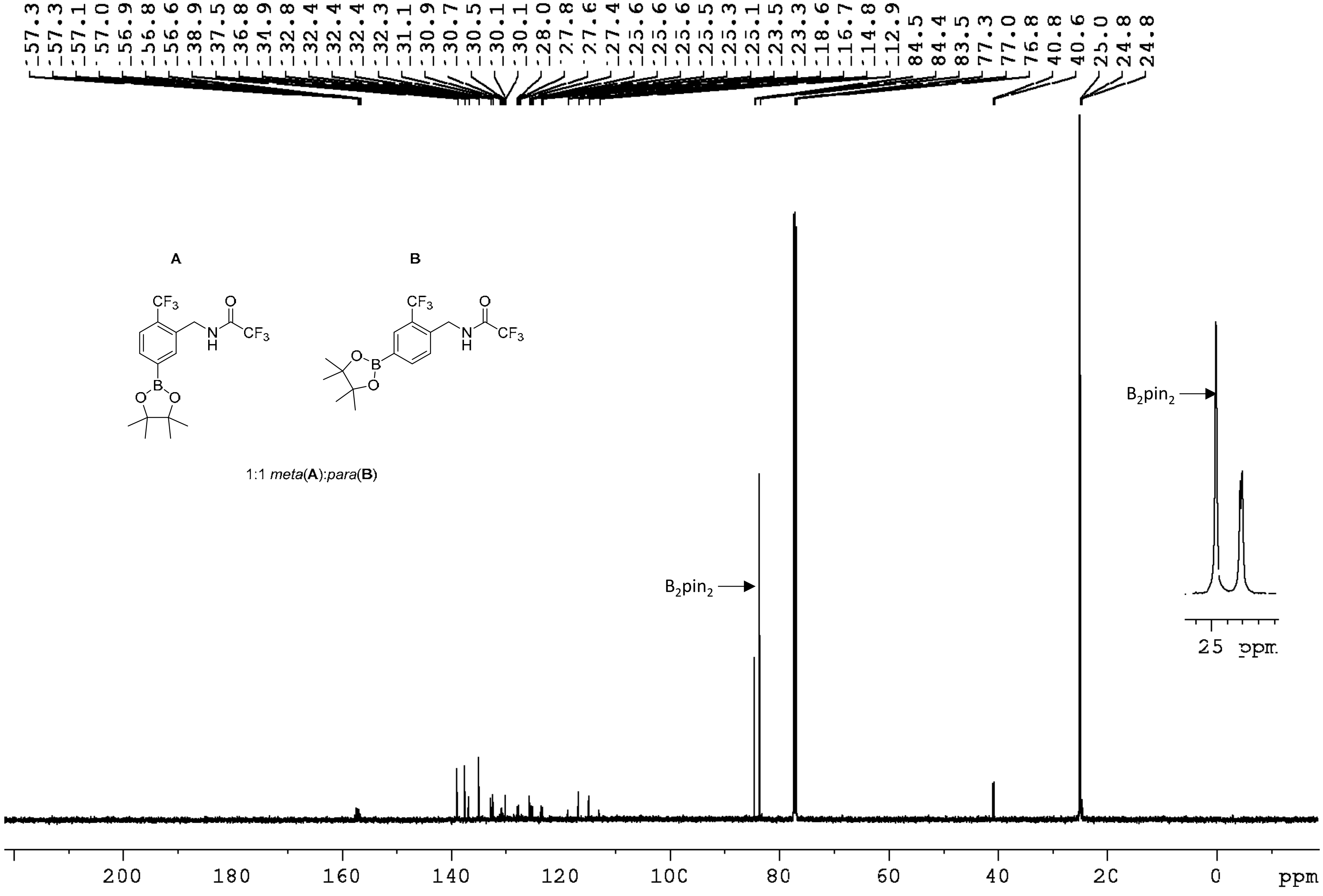

^{1}H NMR (600 MHz, $CDCl_3$) *N*-(2-methyl-5-(4,4,5,5-tetramethyl-1,3,2-dioxaborolan-2-yl)benzyl)-2,2,2-trifluoroacetamide (3b) – Crude after reaction with 1a

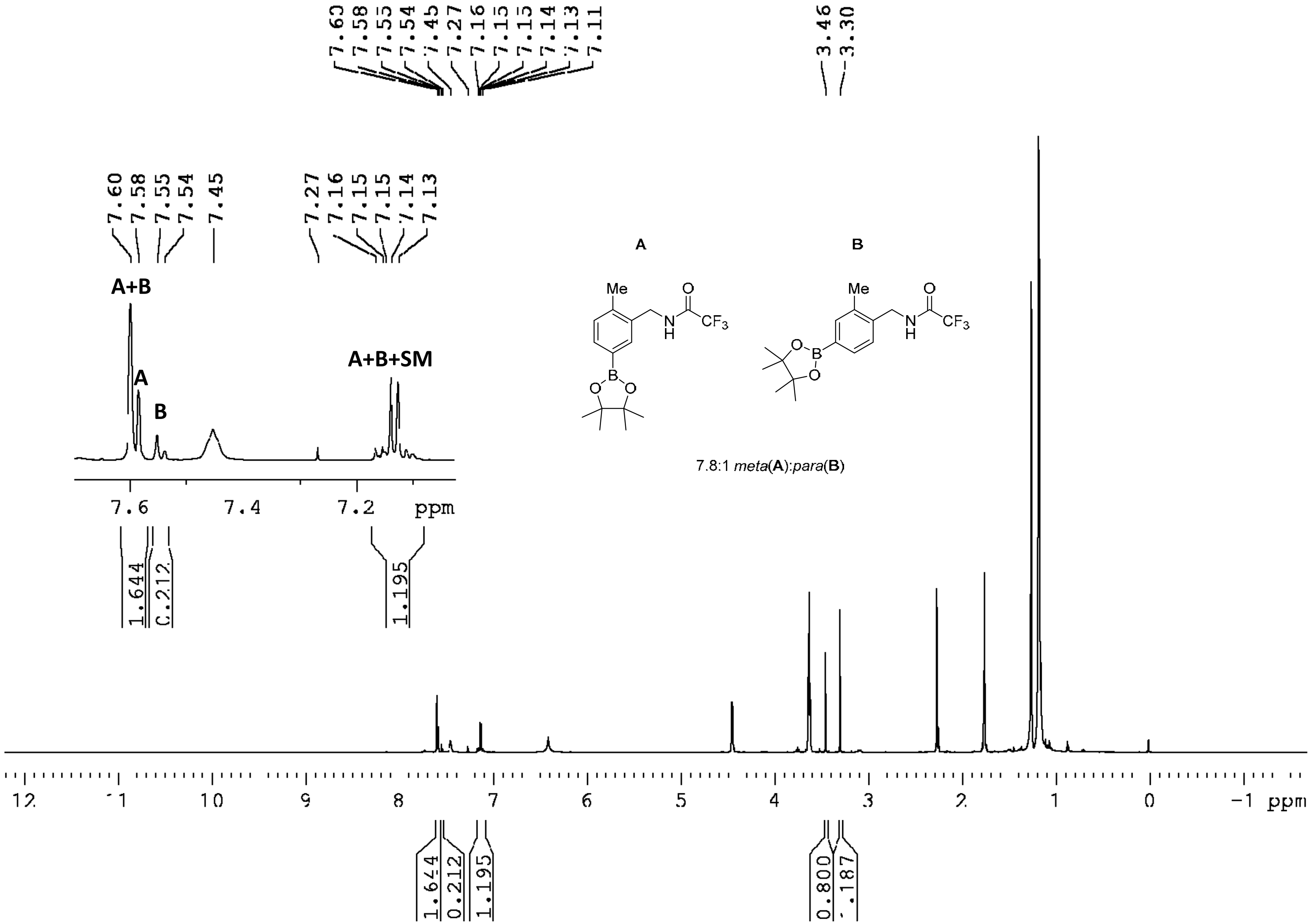

^{1}H NMR (600 MHz, $CDCl_3$) *N*-(2-methyl-5-(4,4,5,5-tetramethyl-1,3,2-dioxaborolan-2-yl)benzyl)-2,2,2-trifluoroacetamide (3b) – Purified after reaction with 1a

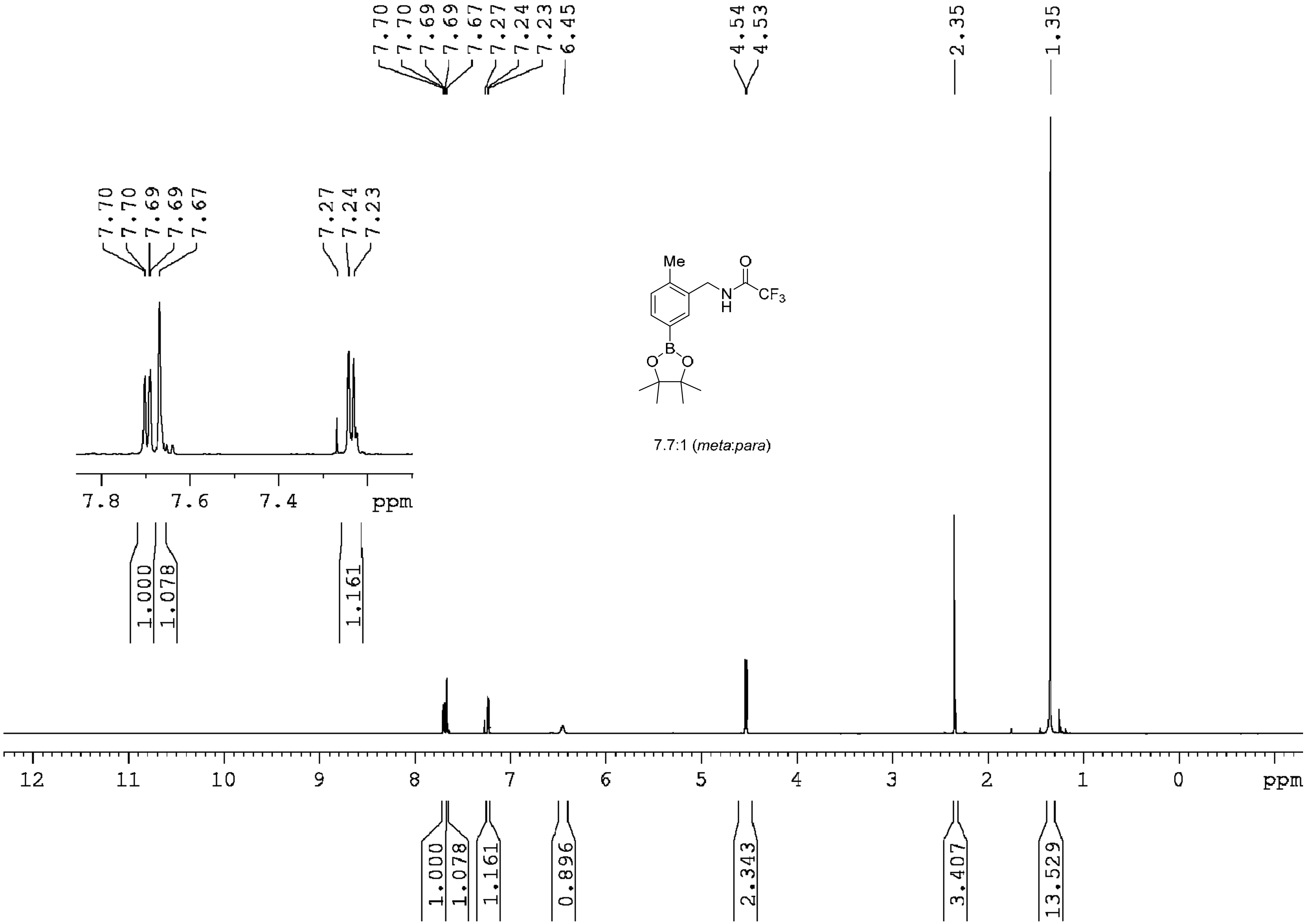

^{13}C NMR (151 MHz, $CDCl_3$) *N*-(2-methyl-5-(4,4,5,5-tetramethyl-1,3,2-dioxaborolan-2-yl)benzyl)-2,2,2-trifluoroacetamide (3b) – 1a

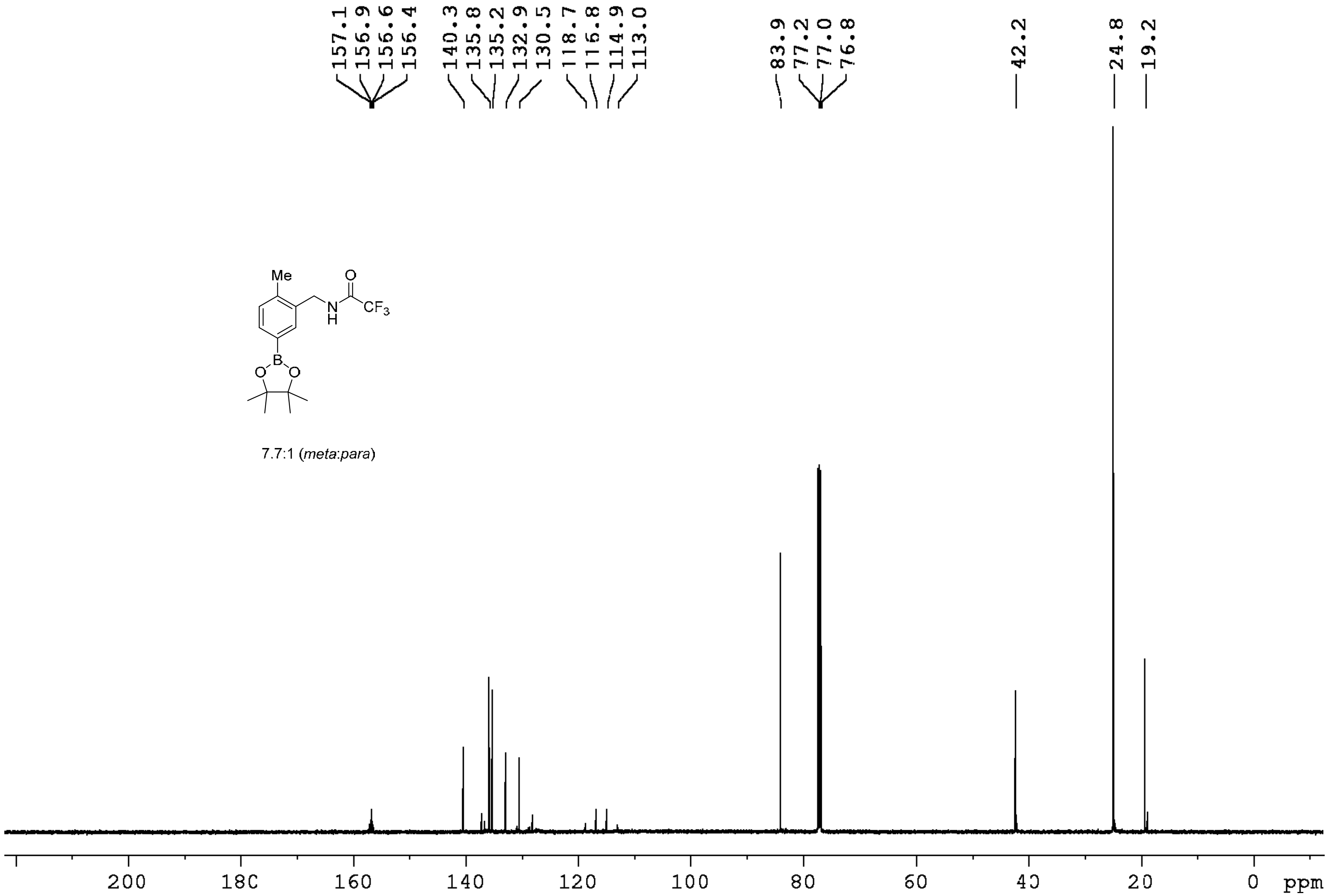

7.7:1 (*meta*:*para*)

^{1}H NMR (500 MHz, $CDCl_3$) *N*-(2-methyl-5-(4,4,5,5-tetramethyl-1,3,2-dioxaborolan-2-yl)benzyl)-2,2,2-trifluoroacetamide (3b) – Purified after reaction with dtbpy

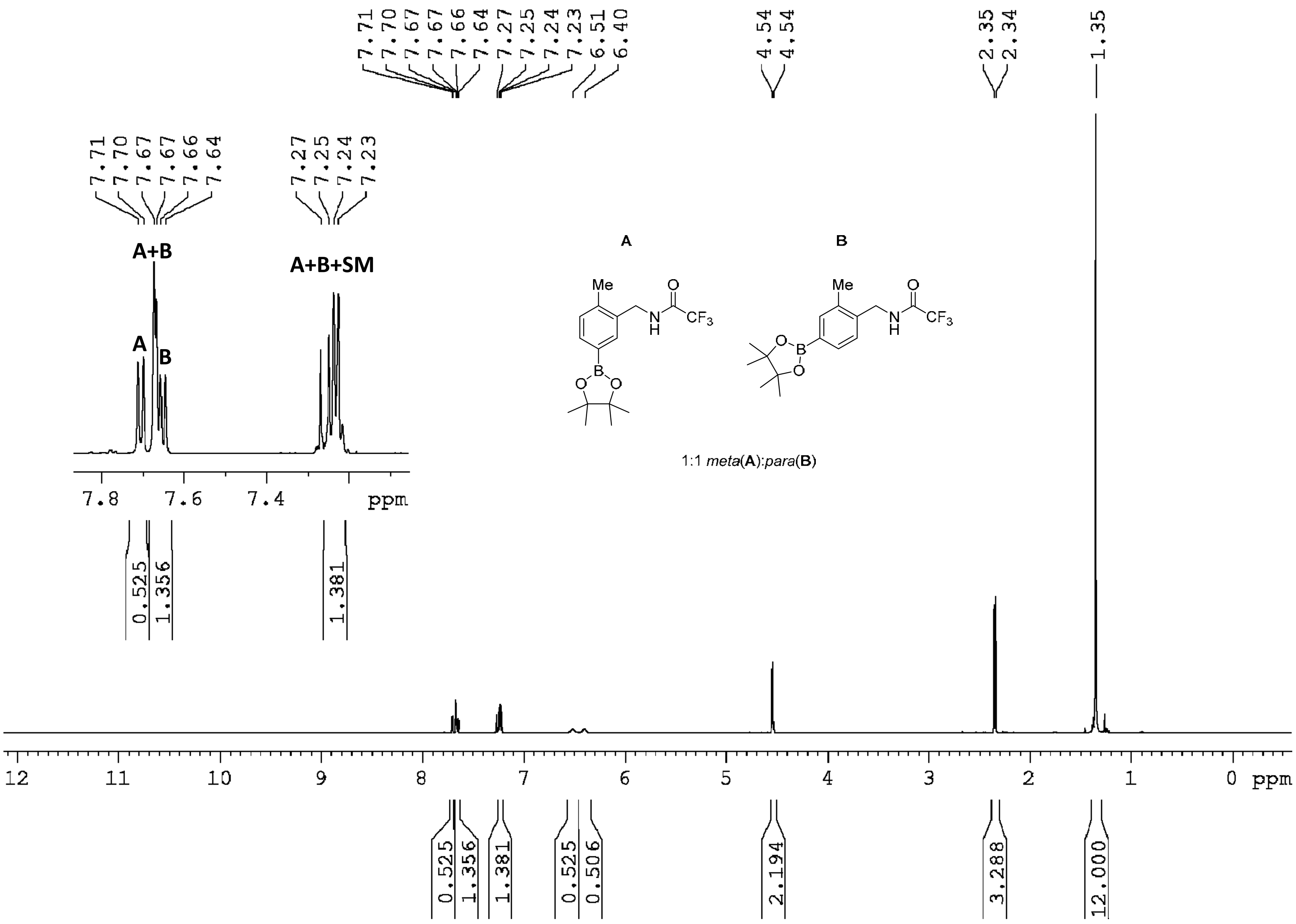

^{13}C NMR (126 MHz, $CDCl_3$) *N*-(2-methyl-5-(4,4,5,5-tetramethyl-1,3,2-dioxaborolan-2-yl)benzyl)-2,2,2-trifluoroacetamide (3b) – purified after reaction with dtbpy

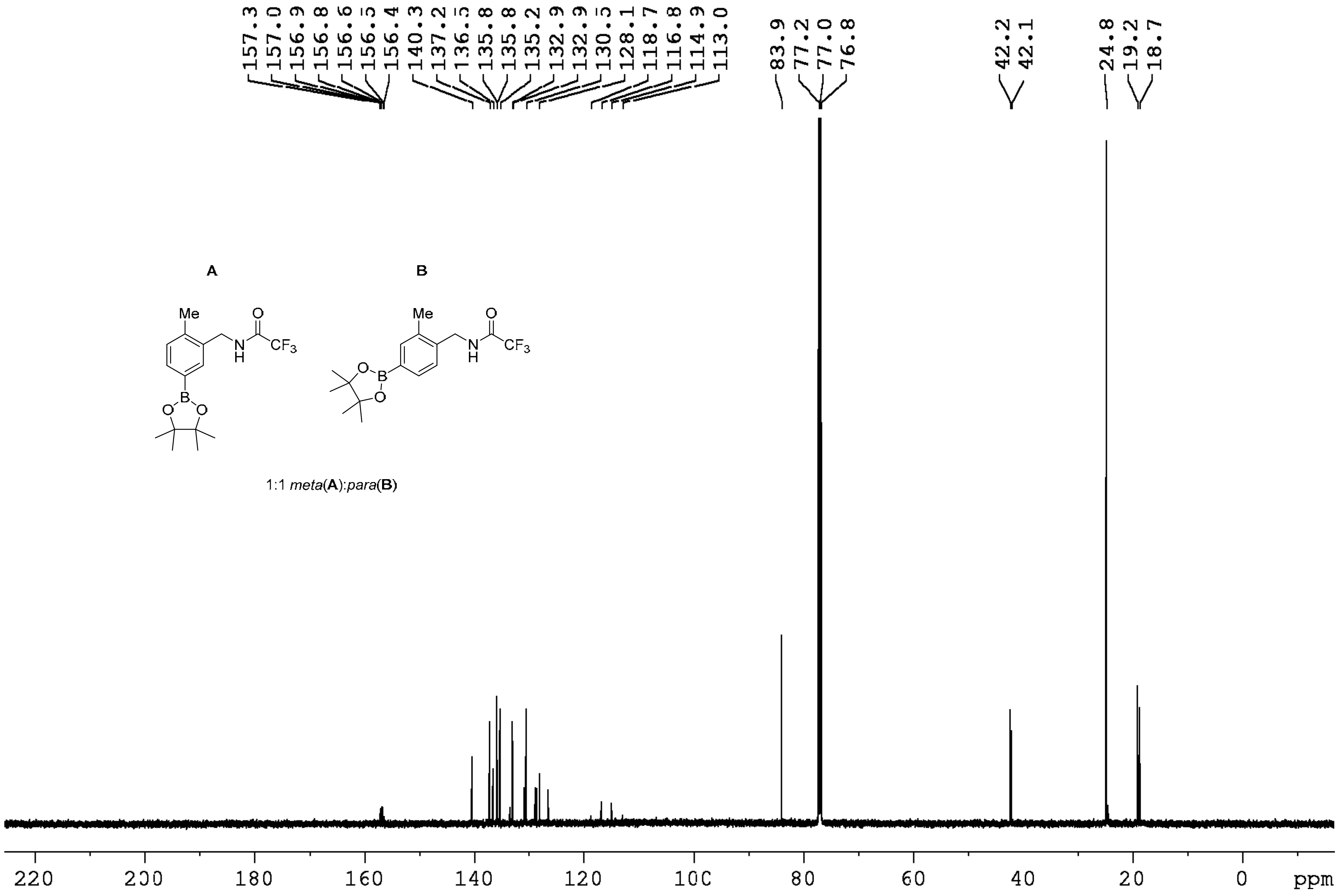

^{1}H NMR (500 MHz, $CDCl_3$) 2,2,2-Trifluoro-*N*-(2-methoxy-5-(4,4,5,5-tetramethyl-1,3,2-dioxaborolan-2-yl)benzyl)acetamide (3c) – Purified after reaction with 1a

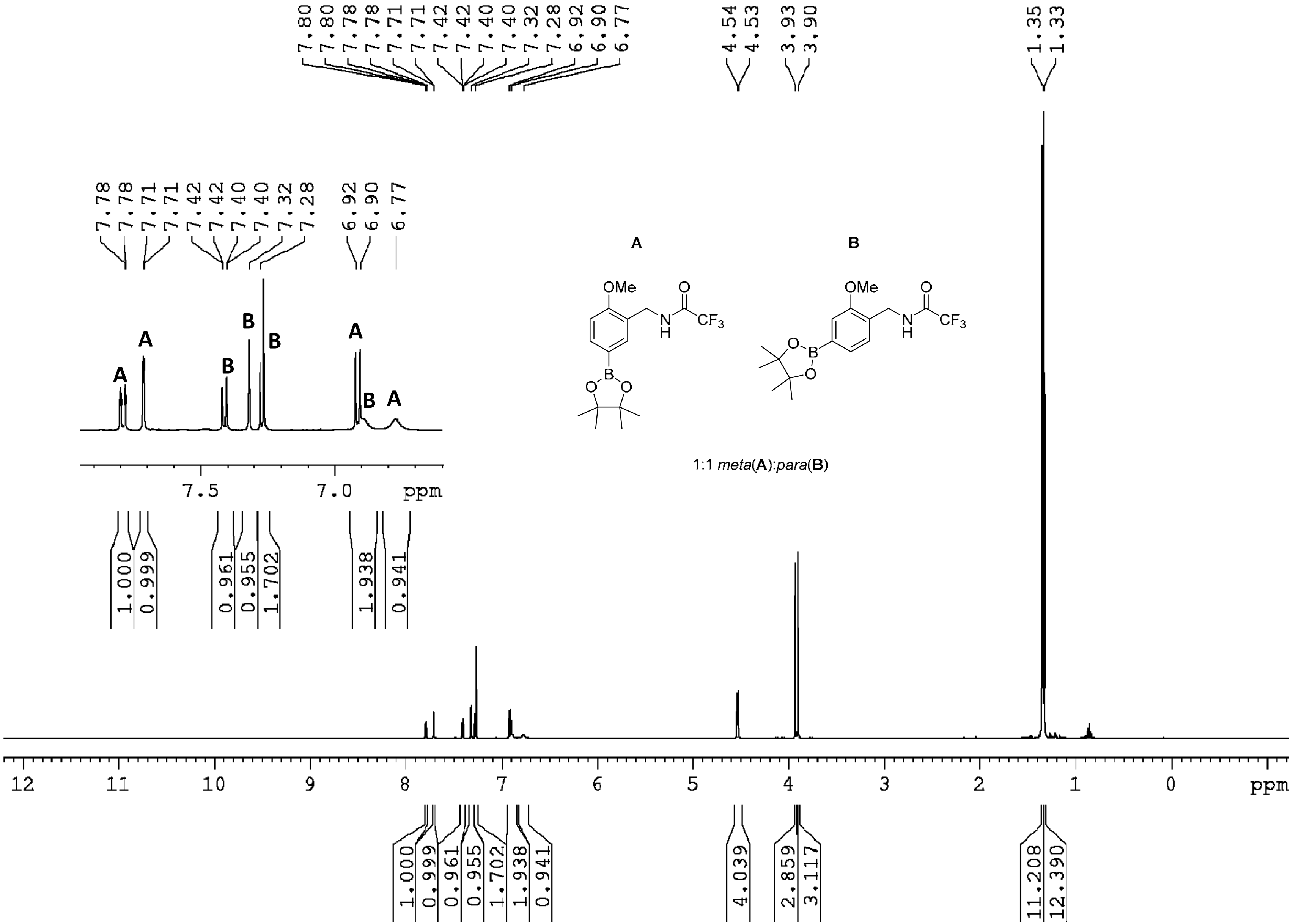

^{13}C NMR (126 MHz, $CDCl_3$) 2,2,2-Trifluoro-*N*-(2-methoxy-5-(4,4,5,5-tetramethyl-1,3,2-dioxaborolan-2-yl)benzyl)acetamide (3c) – Purified after reaction with 1a

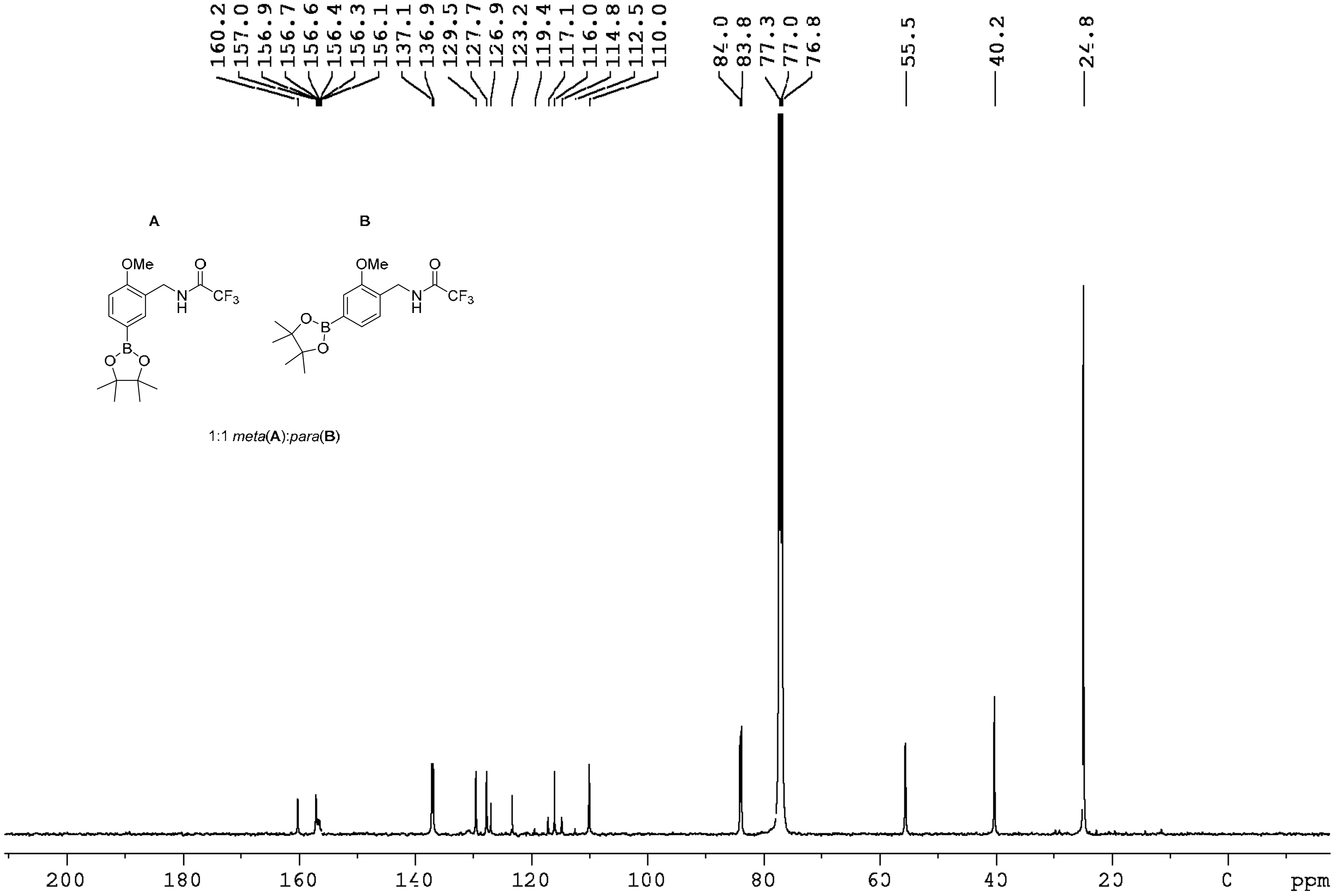

^{1}H NMR (600 MHz, $CDCl_3$) *N*-(2-chloro-5-(4,4,5,5-tetramethyl-1,3,2-dioxaborolan-2-yl)benzyl)-2,2,2-trifluoroacetamide (3d) – Crude after reaction with 1a

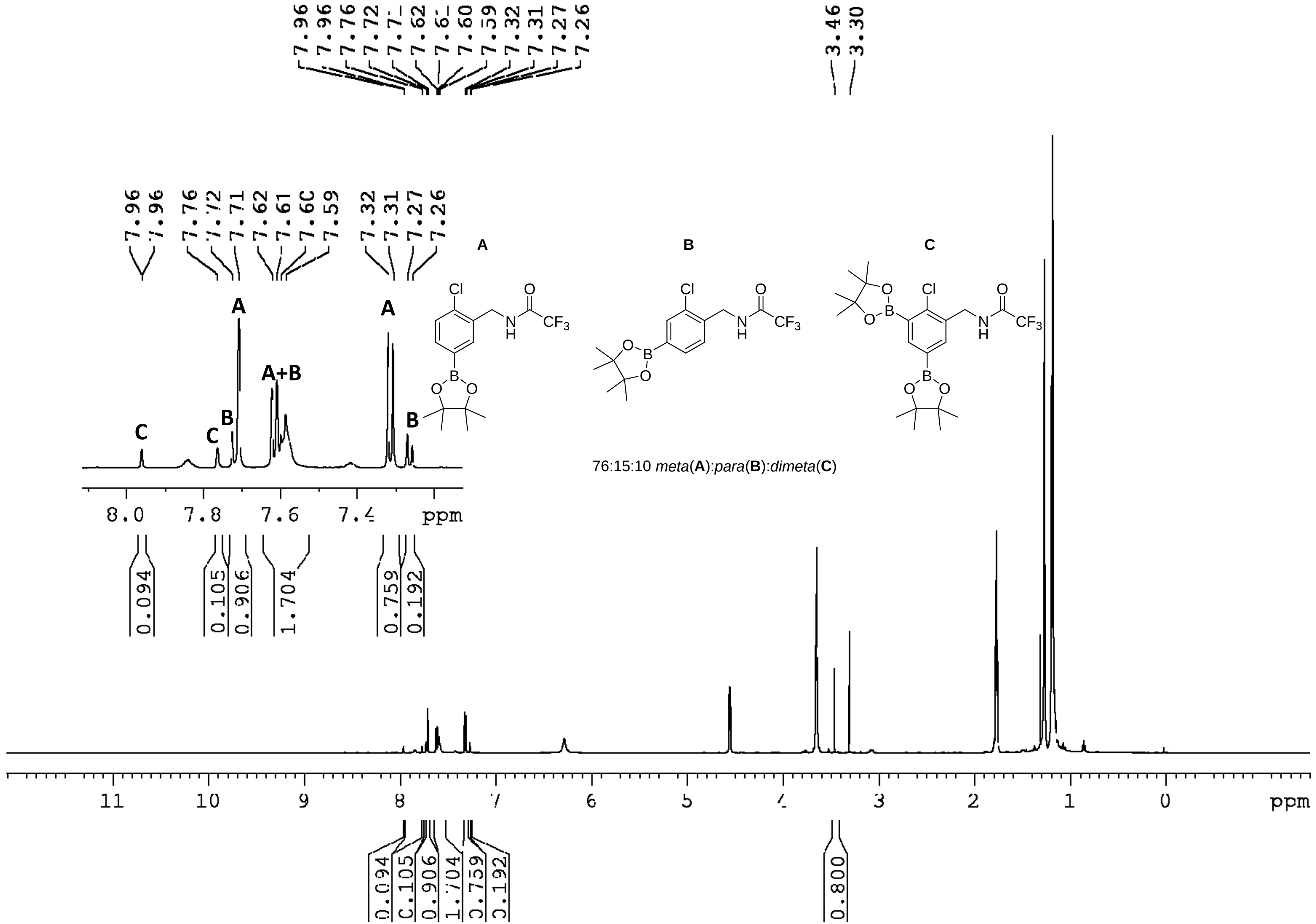

^{1}H NMR (500 MHz, $CDCl_3$) *N*-(2-chloro-5-(4,4,5,5-tetramethyl-1,3,2-dioxaborolan-2-yl)benzyl)-2,2,2-trifluoroacetamide (3d) – Clean fraction of monoborylated isomers for characterisation after reaction 1a

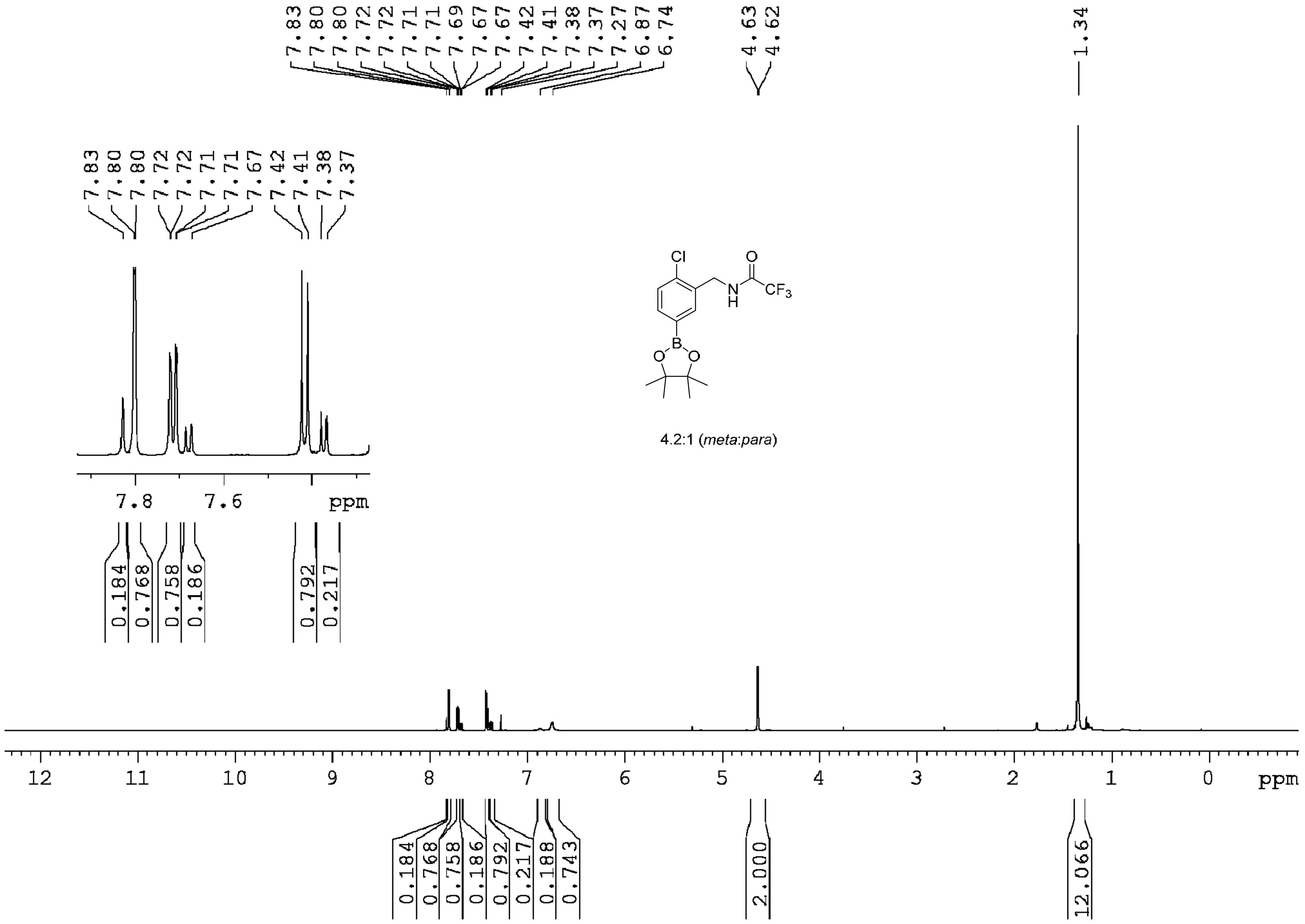

^{13}C NMR (126 MHz, $CDCl_3$) *N*-(2-chloro-5-(4,4,5,5-tetramethyl-1,3,2-dioxaborolan-2-yl)benzyl)-2,2,2-trifluoroacetamide (3d) –Clean fraction of monoborylated isomers for characterisation after reaction 1a

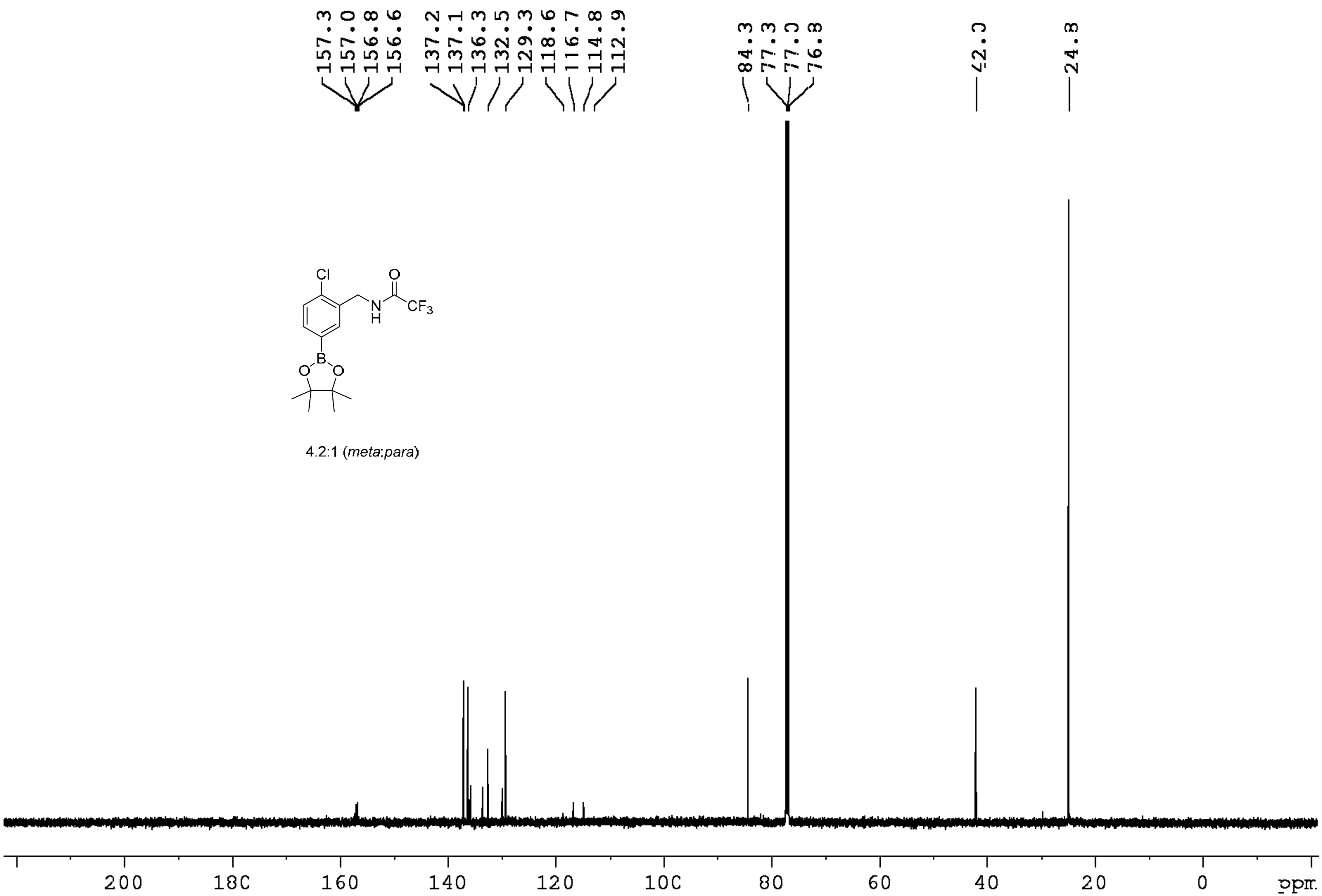

4.2:1 (*meta*:*para*)

^{1}H NMR (600 MHz, $CDCl_3$) *N*-(2-chloro-5-(4,4,5,5-tetramethyl-1,3,2-dioxaborolan-2-yl)benzyl)-2,2,2-trifluoroacetamide (3d) – Purified after reaction with dtbpy

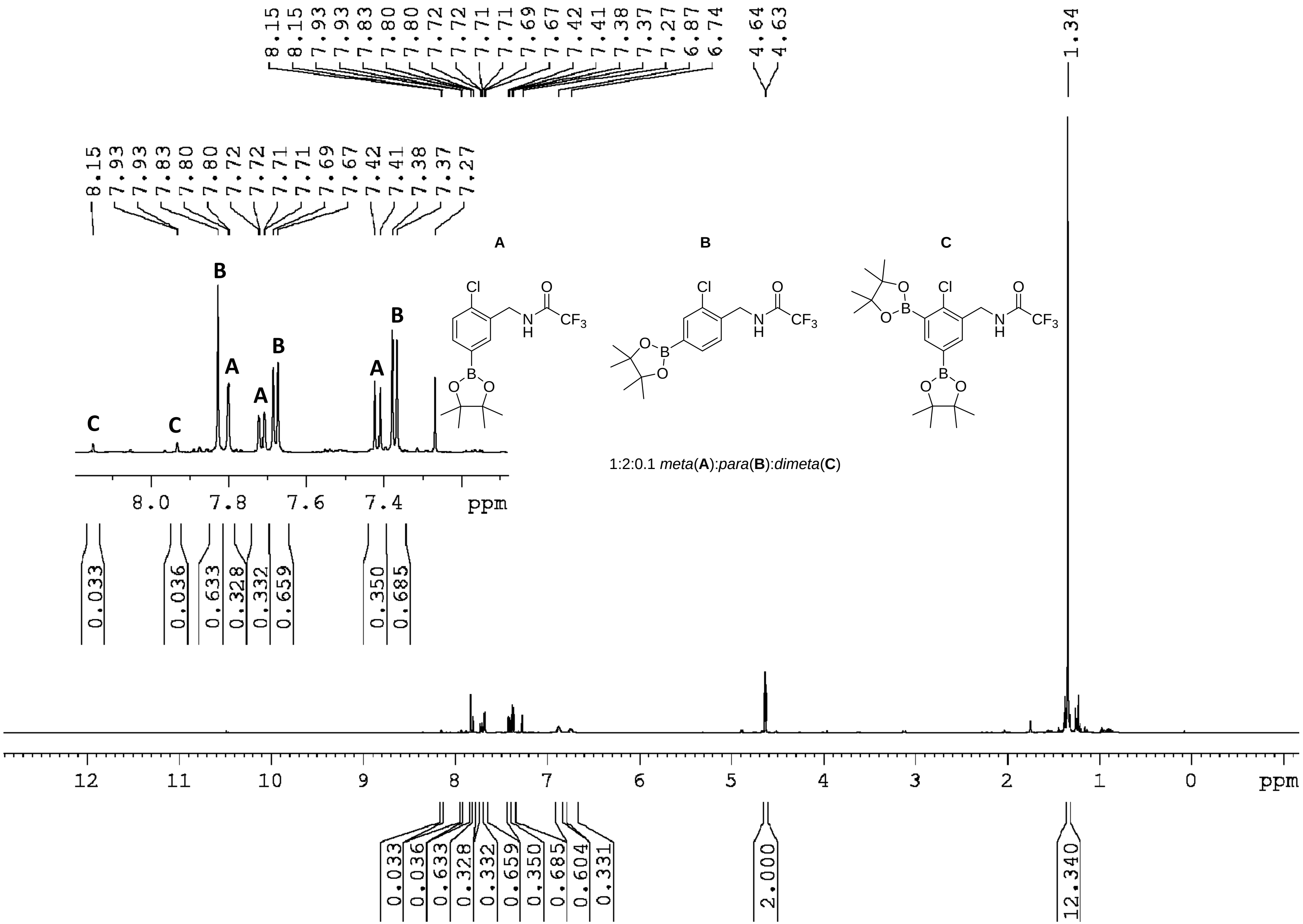

^{13}C NMR (151 MHz, $CDCl_3$) *N*-(2-chloro-5-(4,4,5,5-tetramethyl-1,3,2-dioxaborolan-2-yl)benzyl)-2,2,2-trifluoroacetamide (3d) – Purified after reaction with dtbpy

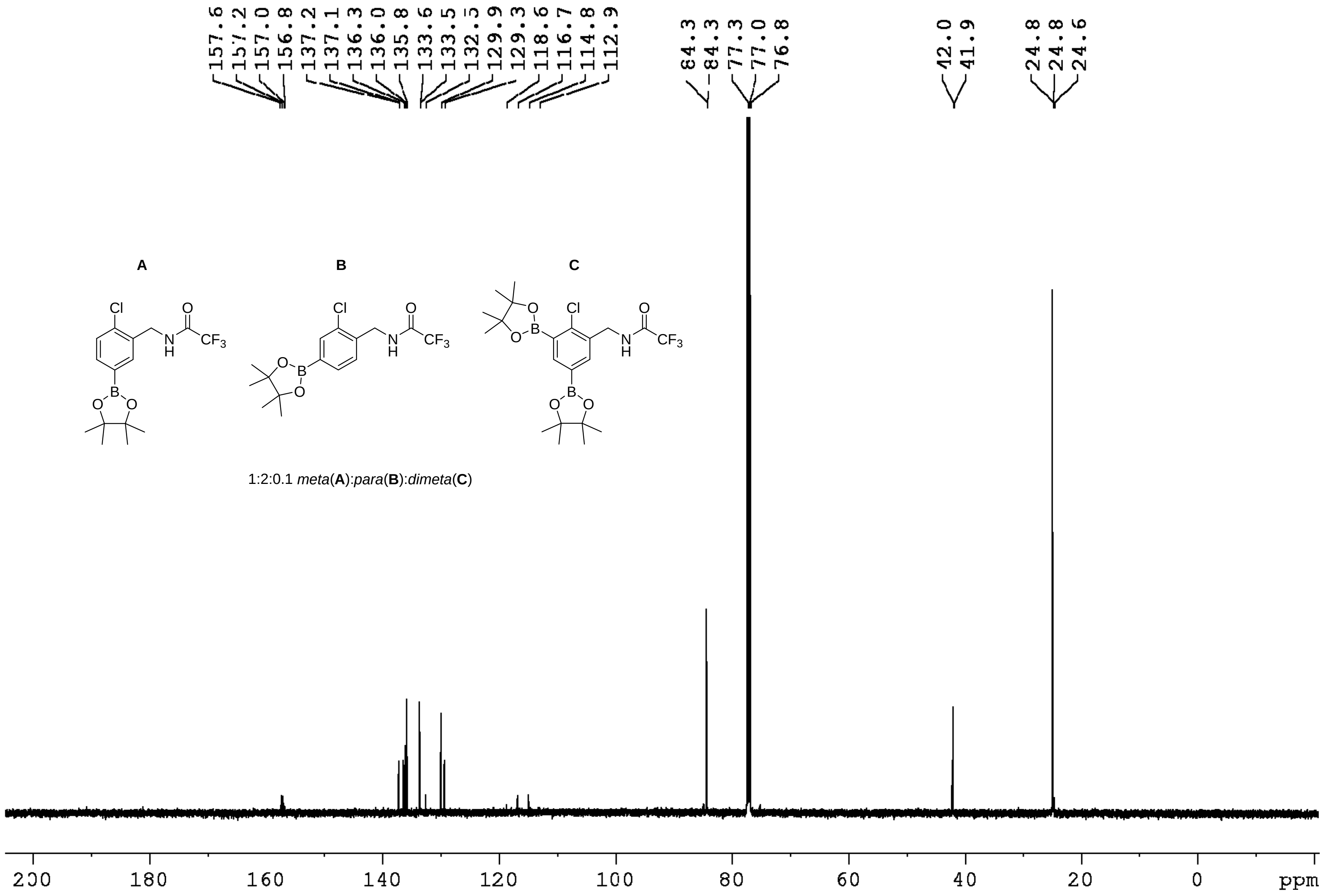

^{1}H NMR (600 MHz, $CDCl_3$) 2,2,2-Trifluoro-*N*-(3-fluoro-5-(4,4,5,5-tetramethyl-1,3,2-dioxaborolan-2-yl)benzyl)acetamide (3e) – Crude after reaction with 1a

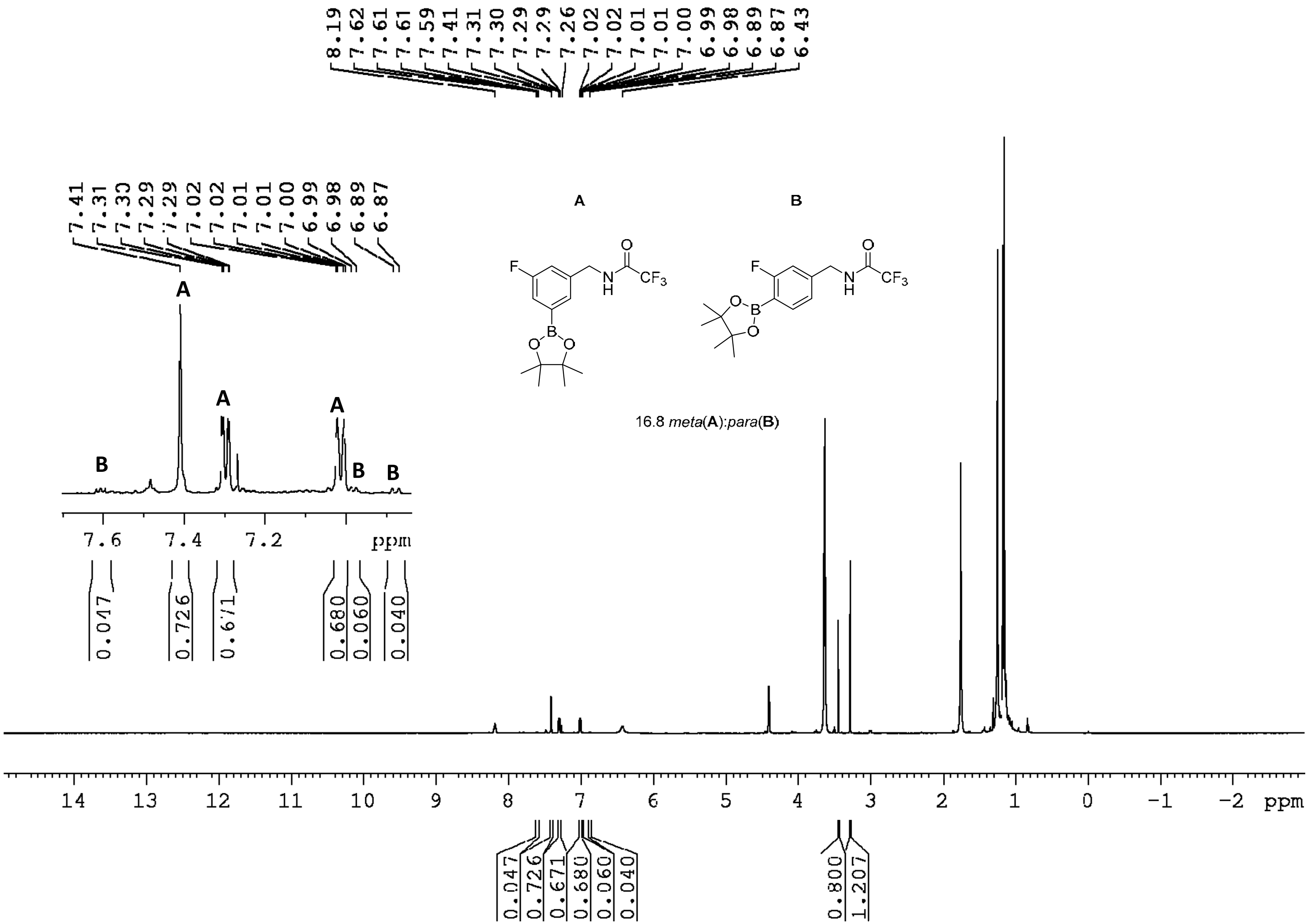

^{1}H NMR (600 MHz, $CDCl_3$) 2,2,2-Trifluoro-*N*-(3-fluoro-5-(4,4,5,5-tetramethyl-1,3,2-dioxaborolan-2-yl)benzyl)acetamide (3e) – Clean fraction of *meta* product after purification after reaction with 1a

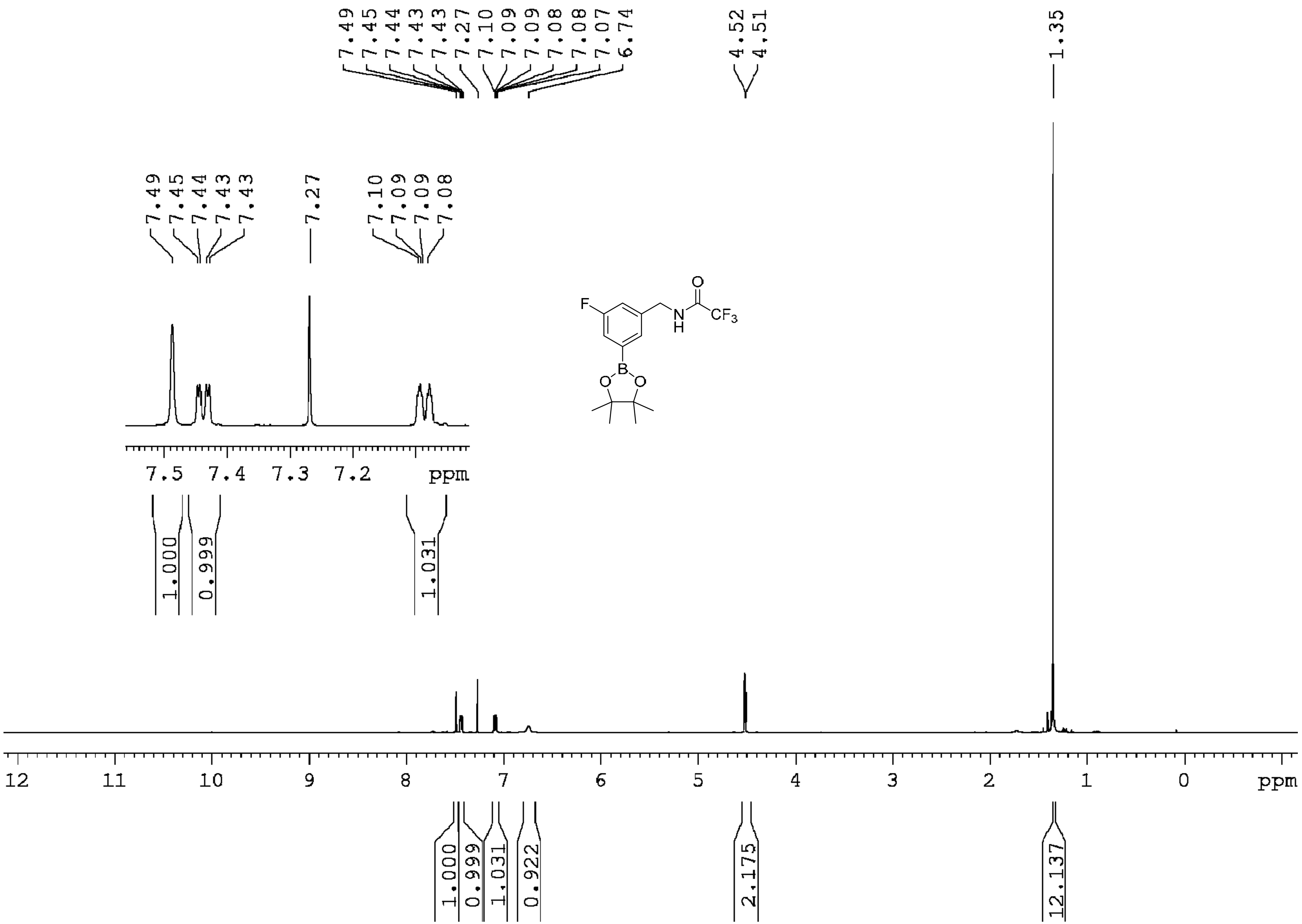

^{13}C NMR (151 MHz, $CDCl_3$) 2,2,2-Trifluoro-*N*-(3-fluoro-5-(4,4,5,5-tetramethyl-1,3,2-dioxaborolan-2-yl)benzyl)acetamide (3e) – Clean fraction of *meta* product after purification after reaction with 1a

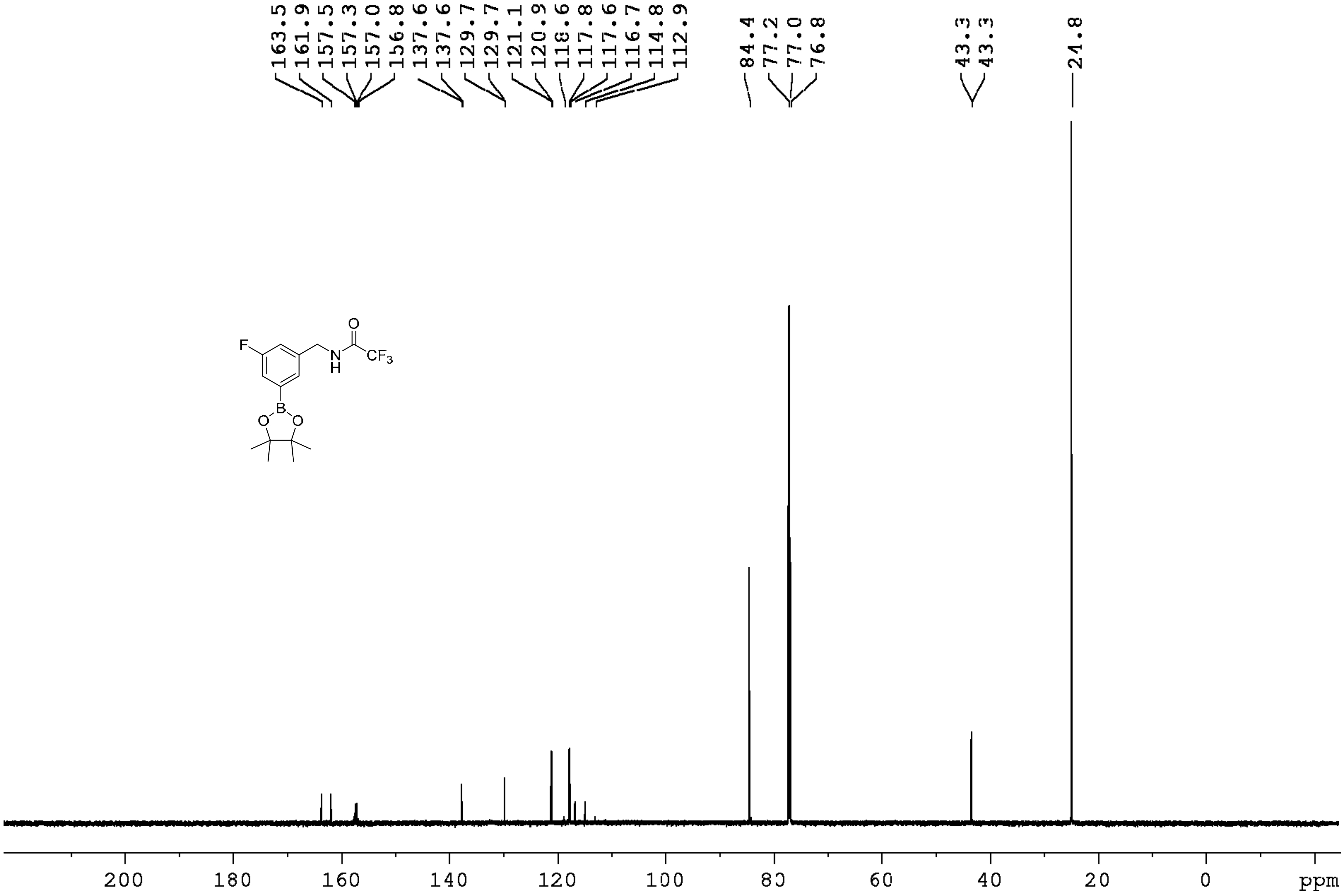

^{1}H NMR (500 MHz, $CDCl_3$) 2,2,2-Trifluoro-*N*-(3-fluoro-5-(4,4,5,5-tetramethyl-1,3,2-dioxaborolan-2-yl)benzyl)acetamide (3e) – Purified after reaction with dtbpy

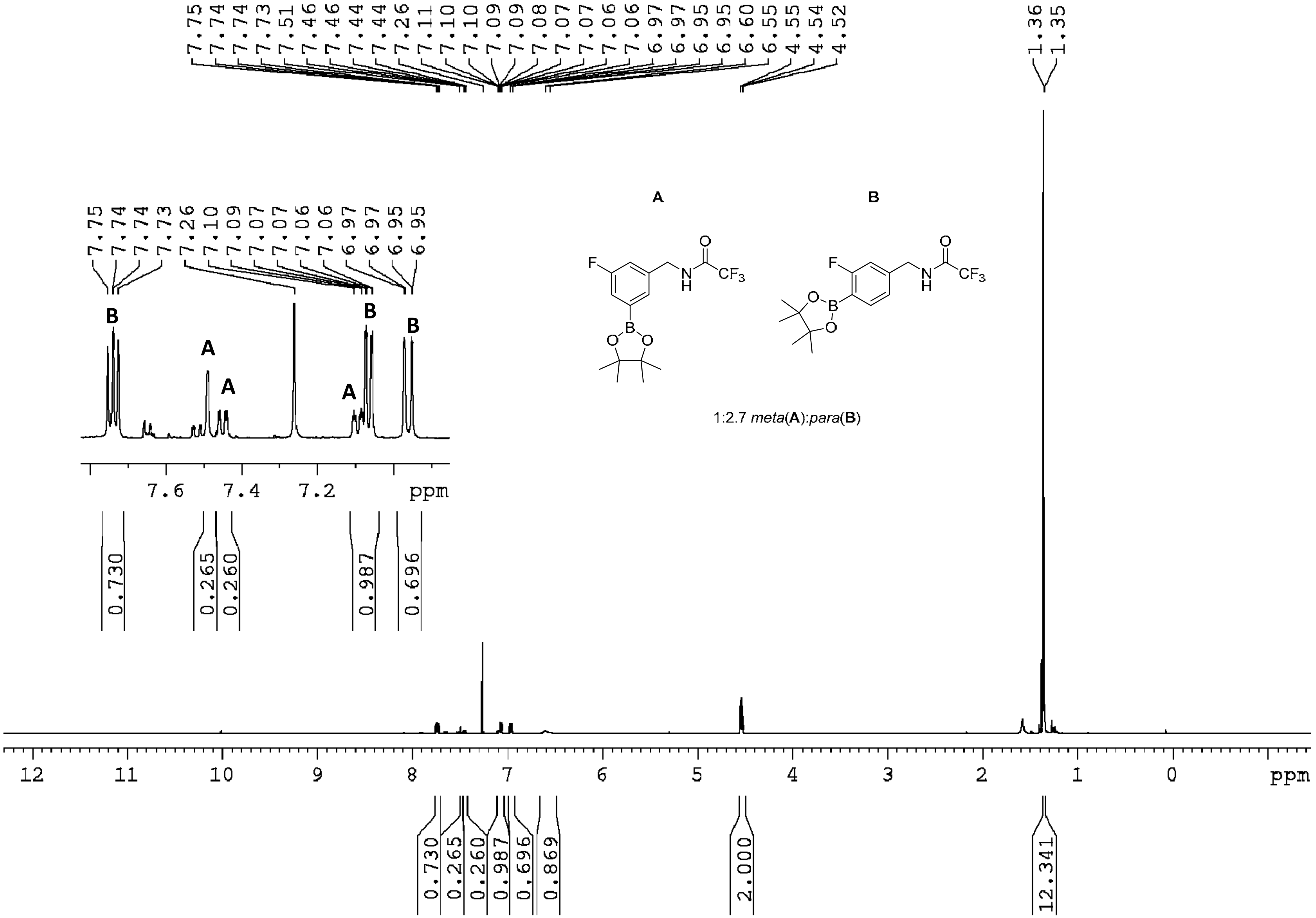

^{13}C NMR (126 MHz, $CDCl_3$) 2,2,2-Trifluoro-*N*-(3-fluoro-5-(4,4,5,5-tetramethyl-1,3,2-dioxaborolan-2-yl)benzyl)acetamide (3e) – Purified after reaction with dtbpy

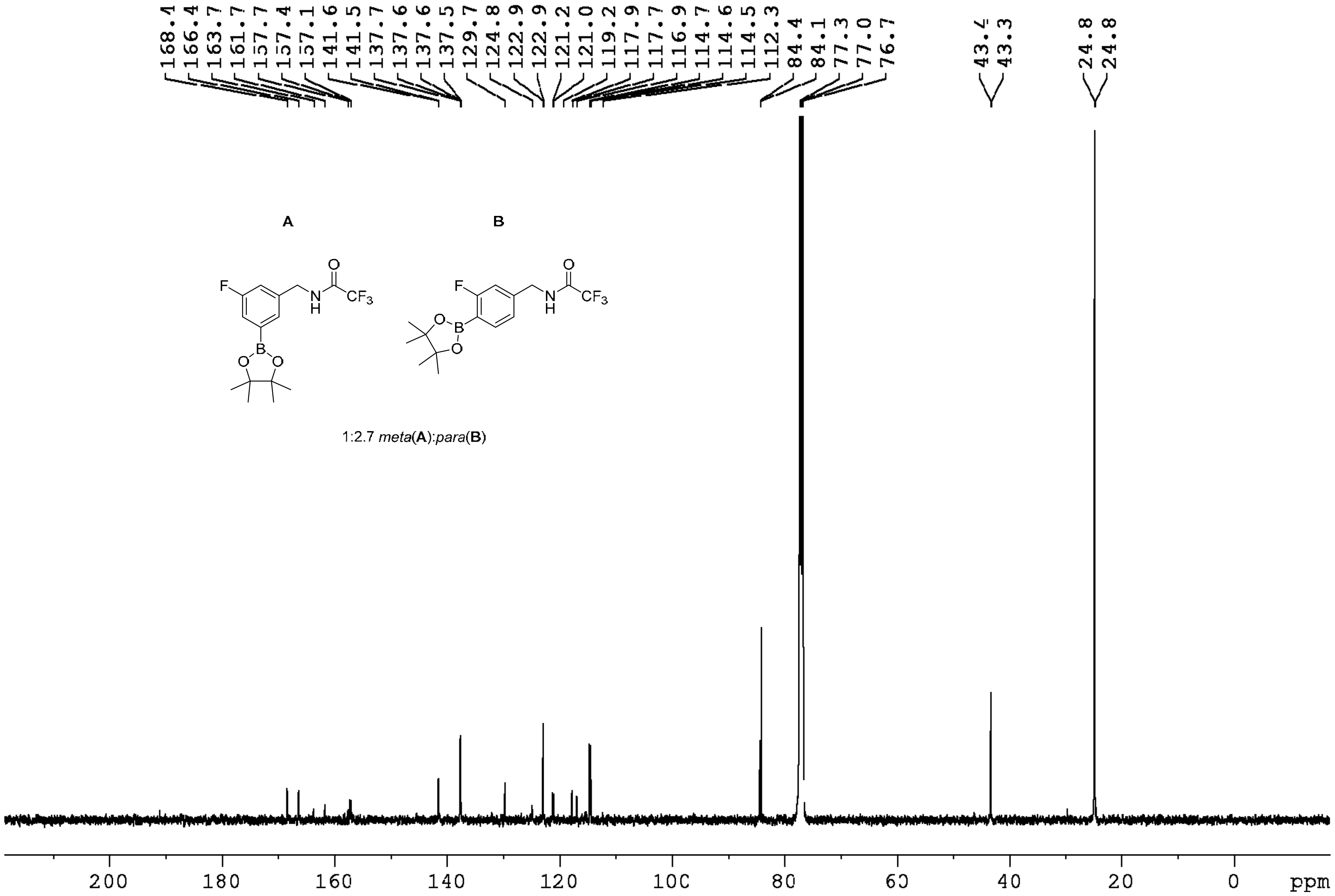

^{1}H NMR (500 MHz, $CDCl_3$) of 2,2,2-trifluoro-N-(2-(hydroxymethyl)-5-(4,4,5,5-tetramethyl-1,3,2-dioxaborolan-2-yl)benzyl)acetamide (3f) – Dtbpy – Purified

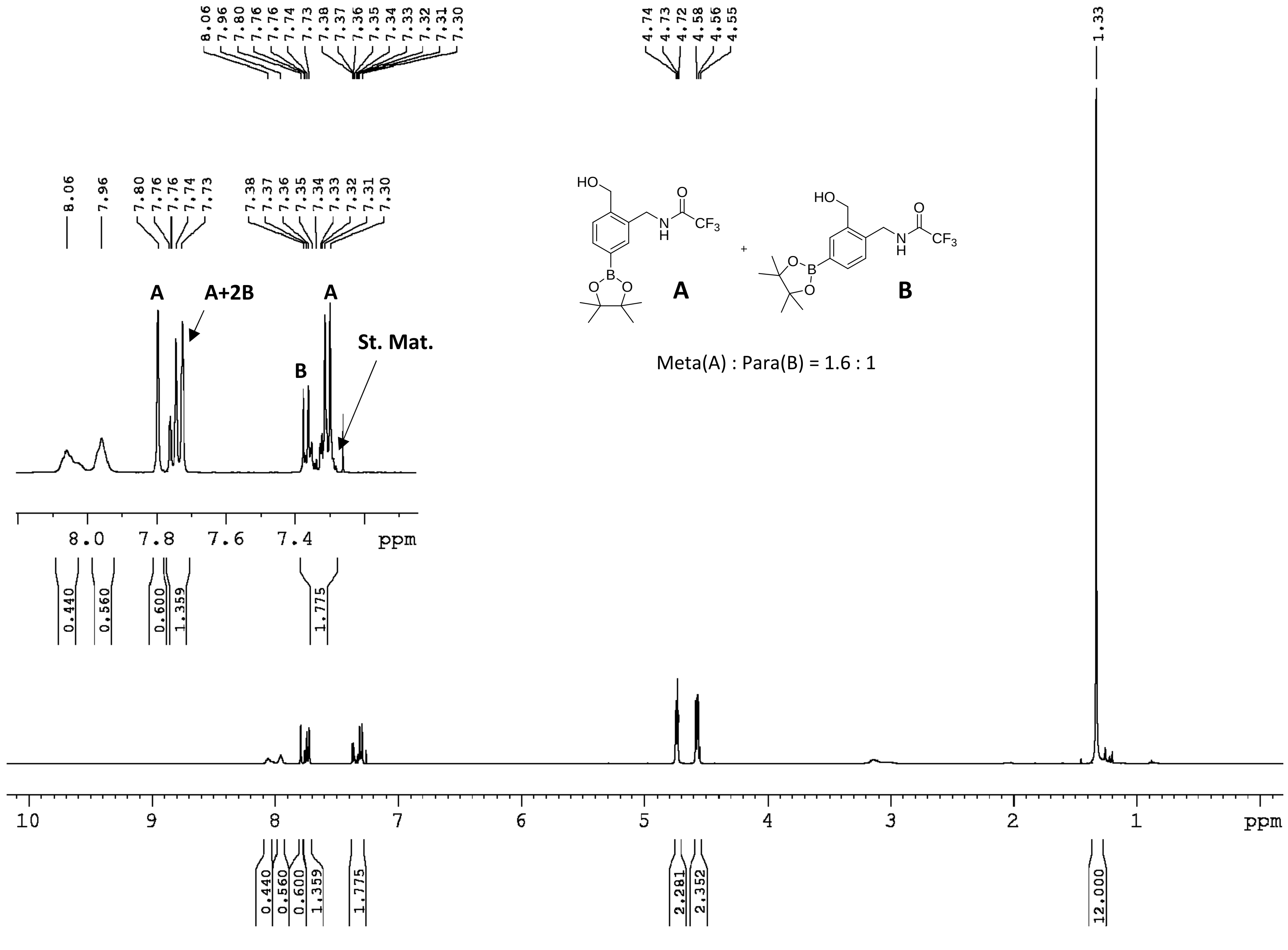

^{13}C NMR (126 MHz, $CDCl_3$) of 2,2,2-trifluoro-N-(2-(hydroxymethyl)-5-(4,4,5,5-tetramethyl-1,3,2-dioxaborolan-2-yl)benzyl)acetamide (3f) – Dtbpy – Purified

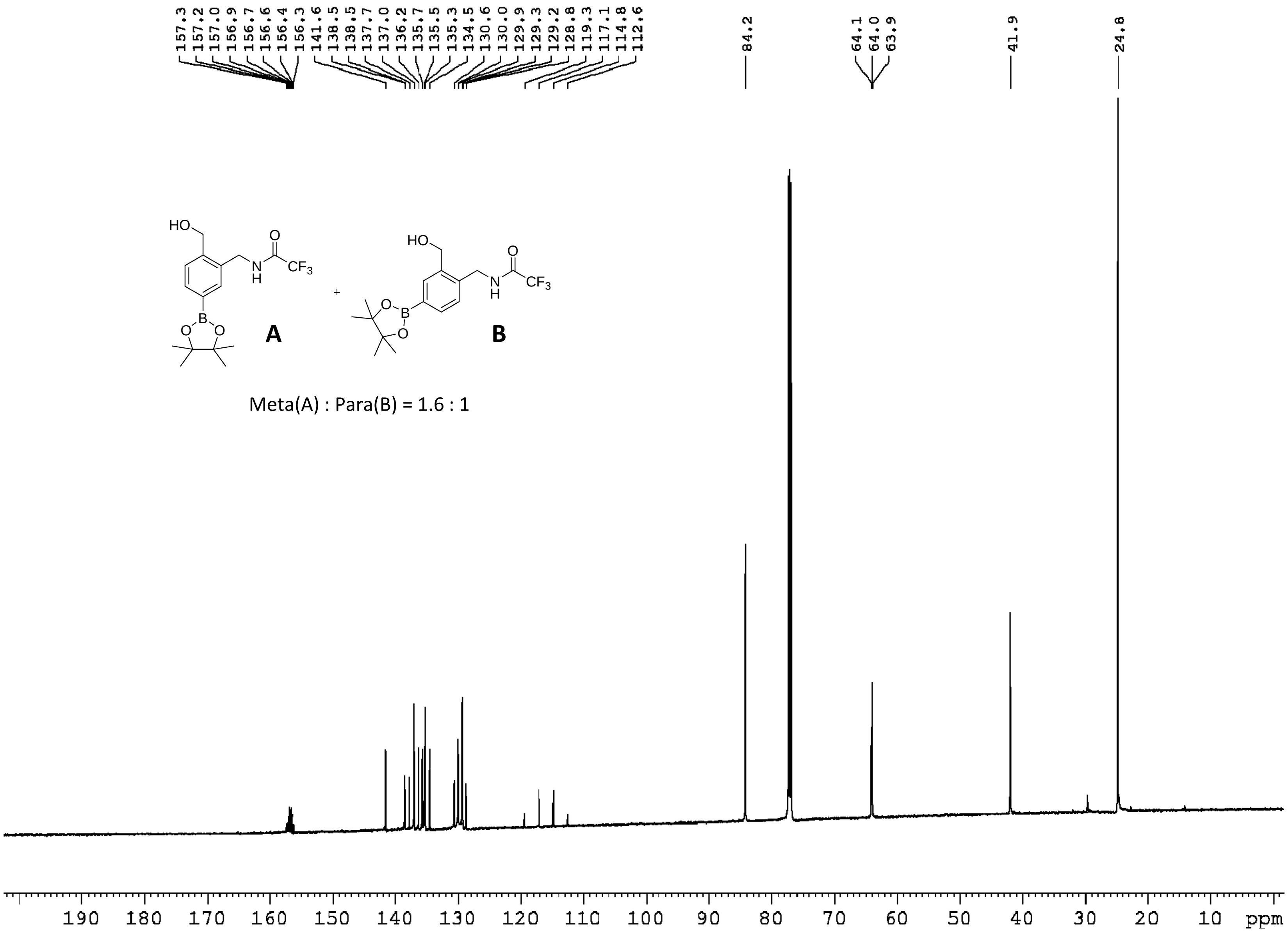

^{1}H NMR (600 MHz, $CDCl_3$) of 2,2,2-trifluoro-N-(2-(hydroxymethyl)-5-(4,4,5,5-tetramethyl-1,3,2-dioxaborolan-2-yl)benzyl)acetamide (3f) – Ligand 1a – Crude mixture

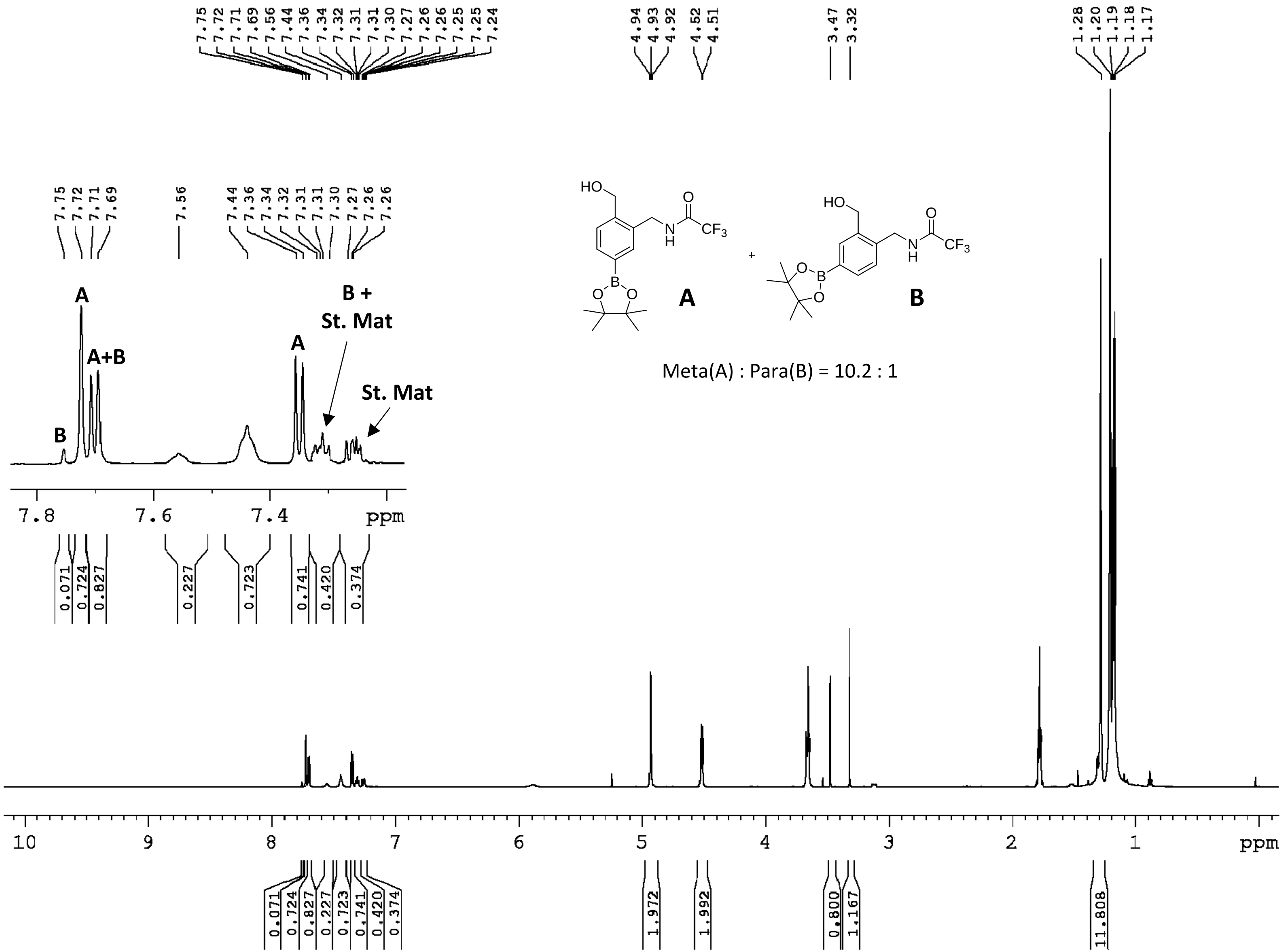

Meta(A) : Para(B) = 10.2 : 1

^{1}H NMR (600 MHz, $CDCl_3$) of 2,2,2-trifluoro-N-(2-(hydroxymethyl)-5-(4,4,5,5-tetramethyl-1,3,2-dioxaborolan-2-yl)benzyl)acetamide (3f) – Ligand 1a – Purified

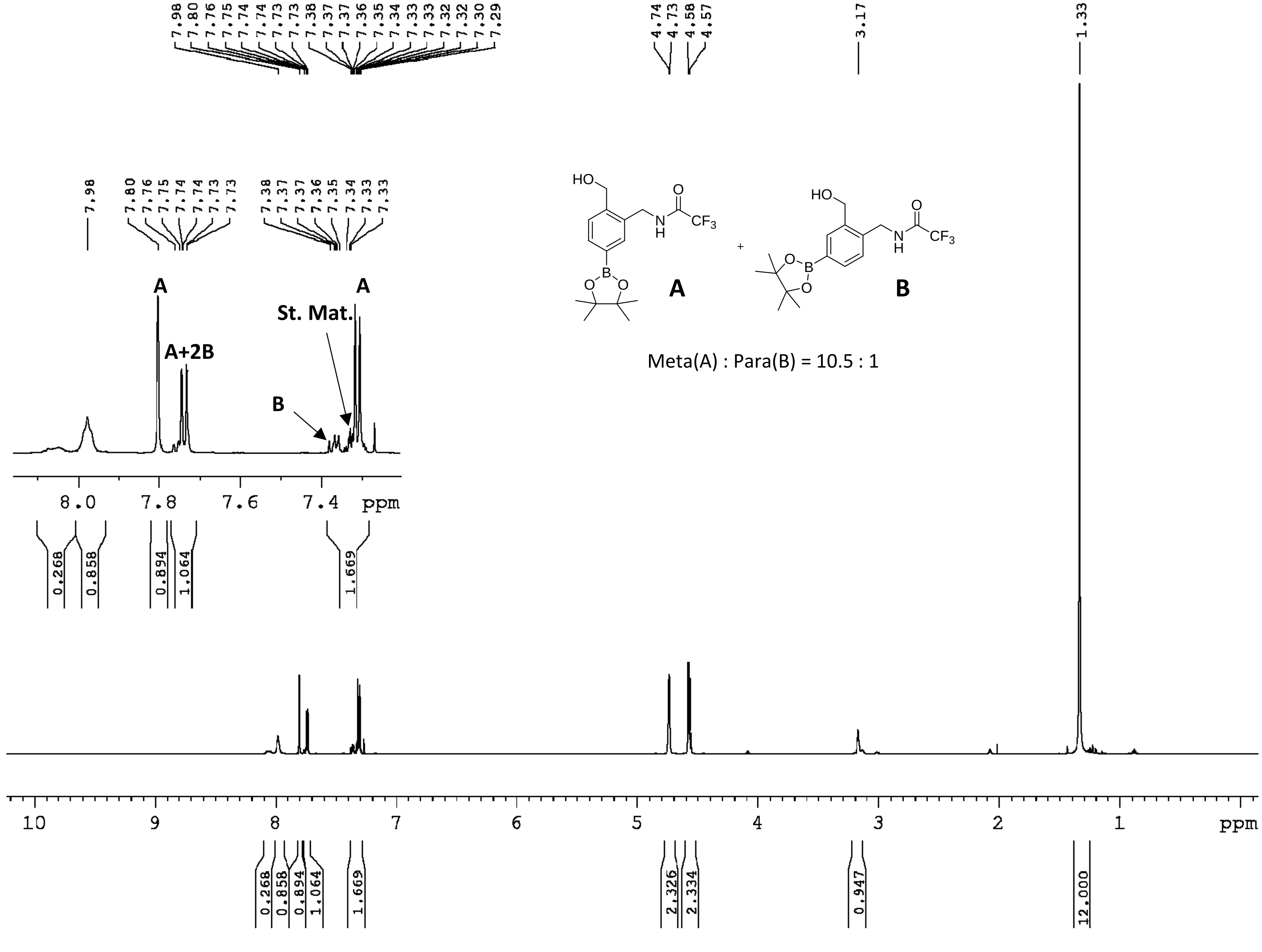

^{13}C NMR (151 MHz, $CDCl_3$) of 2,2,2-trifluoro-N-(2-(hydroxymethyl)-5-(4,4,5,5-tetramethyl-1,3,2-dioxaborolan-2-yl)benzyl)acetamide (3f) – Ligand 1a – Purified

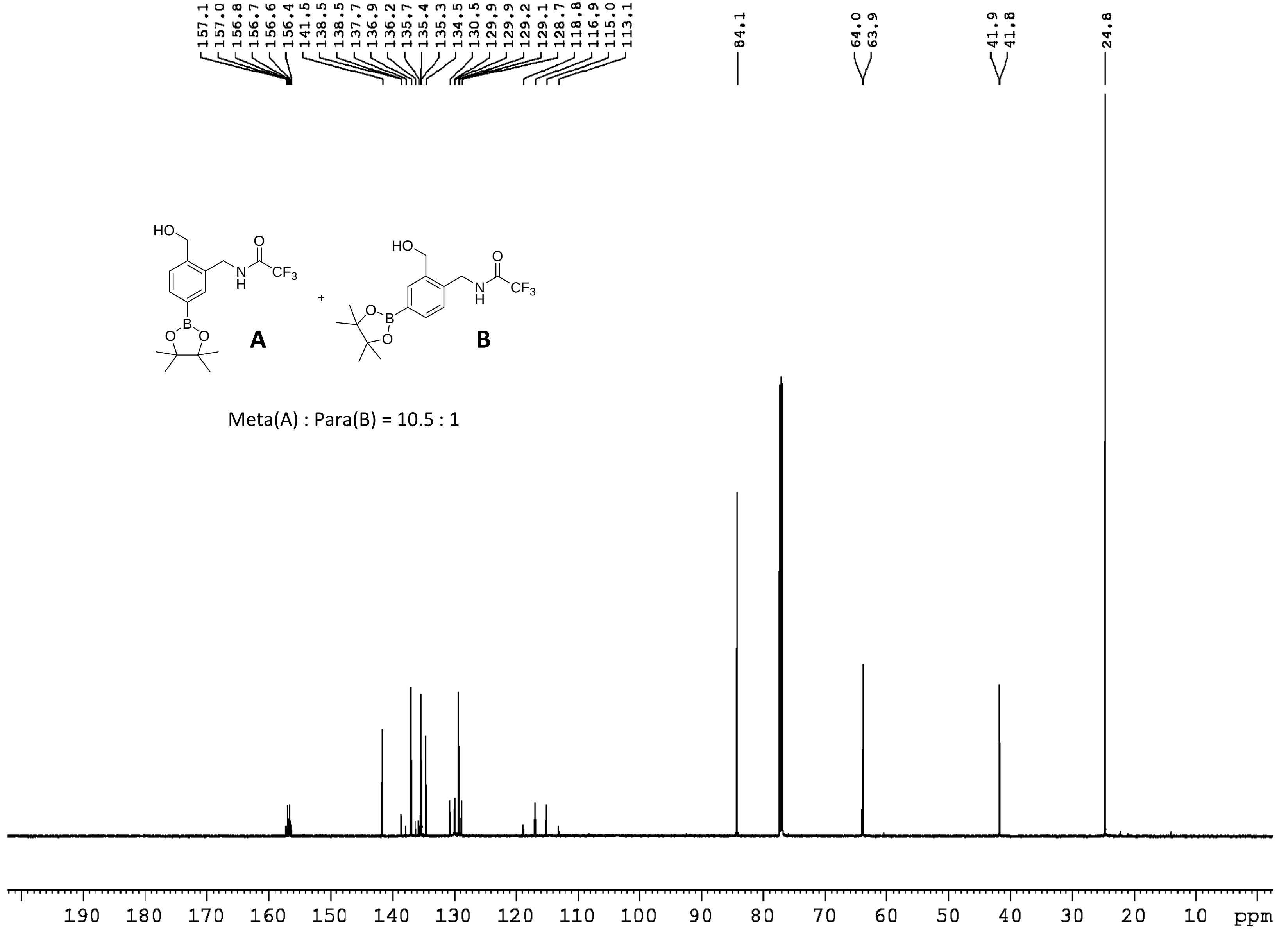

^{1}H NMR (600 MHz, $CDCl_3$) Methyl 4-(4,4,5,5-tetramethyl-1,3,2-dioxaborolan-2-yl)-2-((2,2,2-trifluoroacetamido)methyl)benzoate (3g) – Crude after reaction with 1a

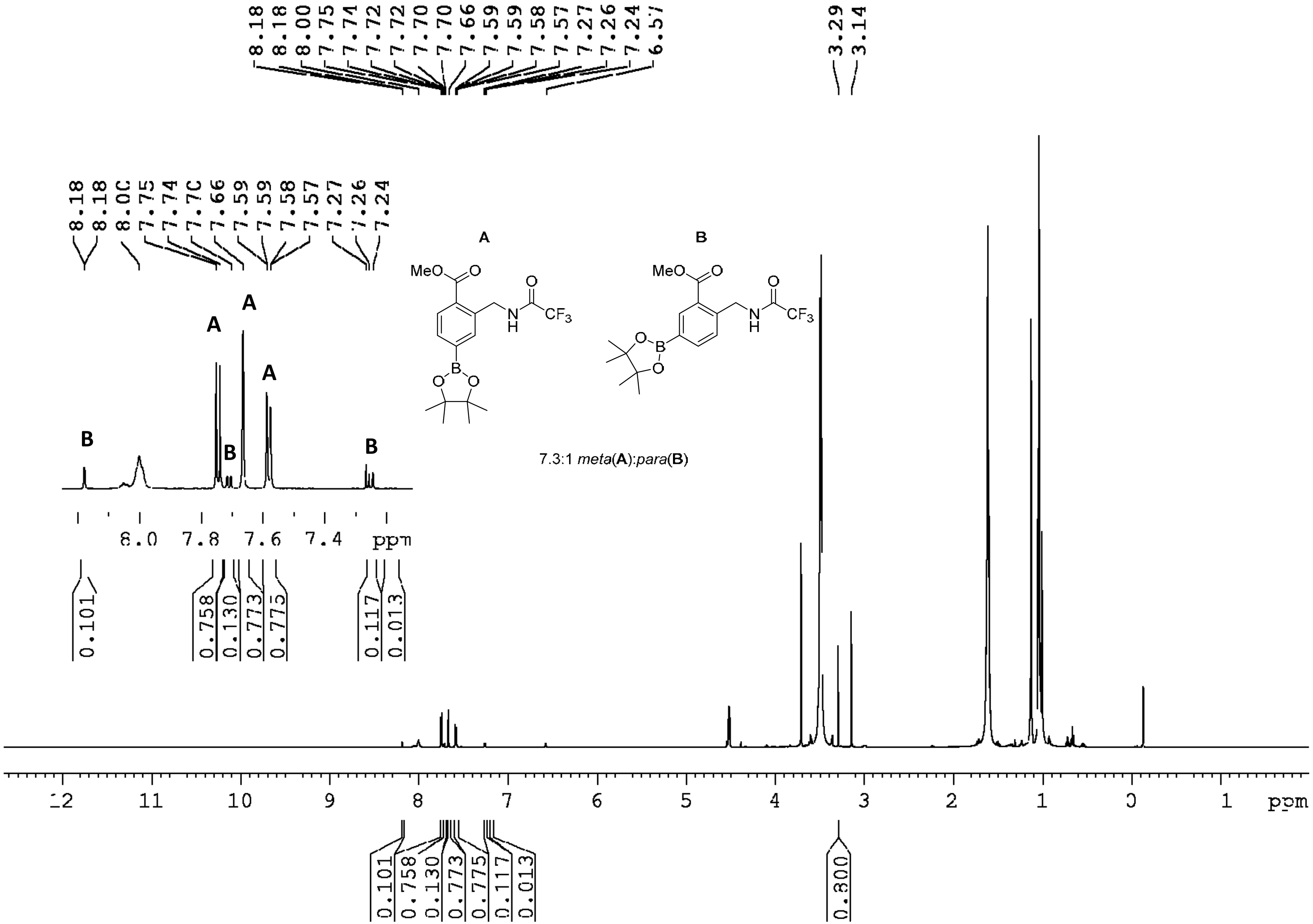

^{1}H NMR (600 MHz, $CDCl_3$) Methyl 4-(4,4,5,5-tetramethyl-1,3,2-dioxaborolan-2-yl)-2-((2,2,2-trifluoroacetamido)methyl)benzoate (3g) – Purified after reaction with 1a

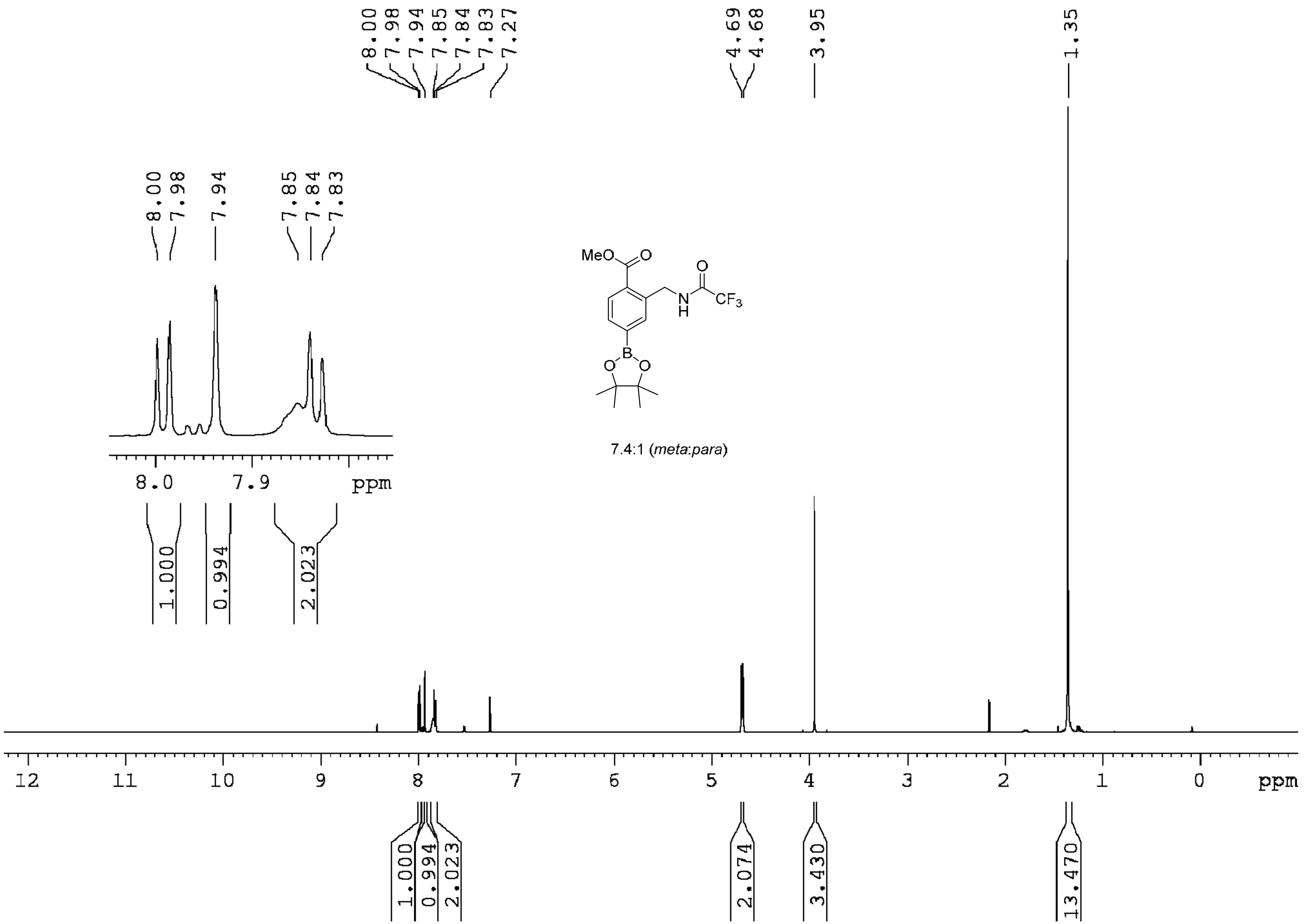

^{13}C NMR (151 MHz, $CDCl_3$) Methyl 4-(4,4,5,5-tetramethyl-1,3,2-dioxaborolan-2-yl)-2-((2,2,2-trifluoroacetamido)methyl)benzoate (3g) – Purified after reactino with 1a

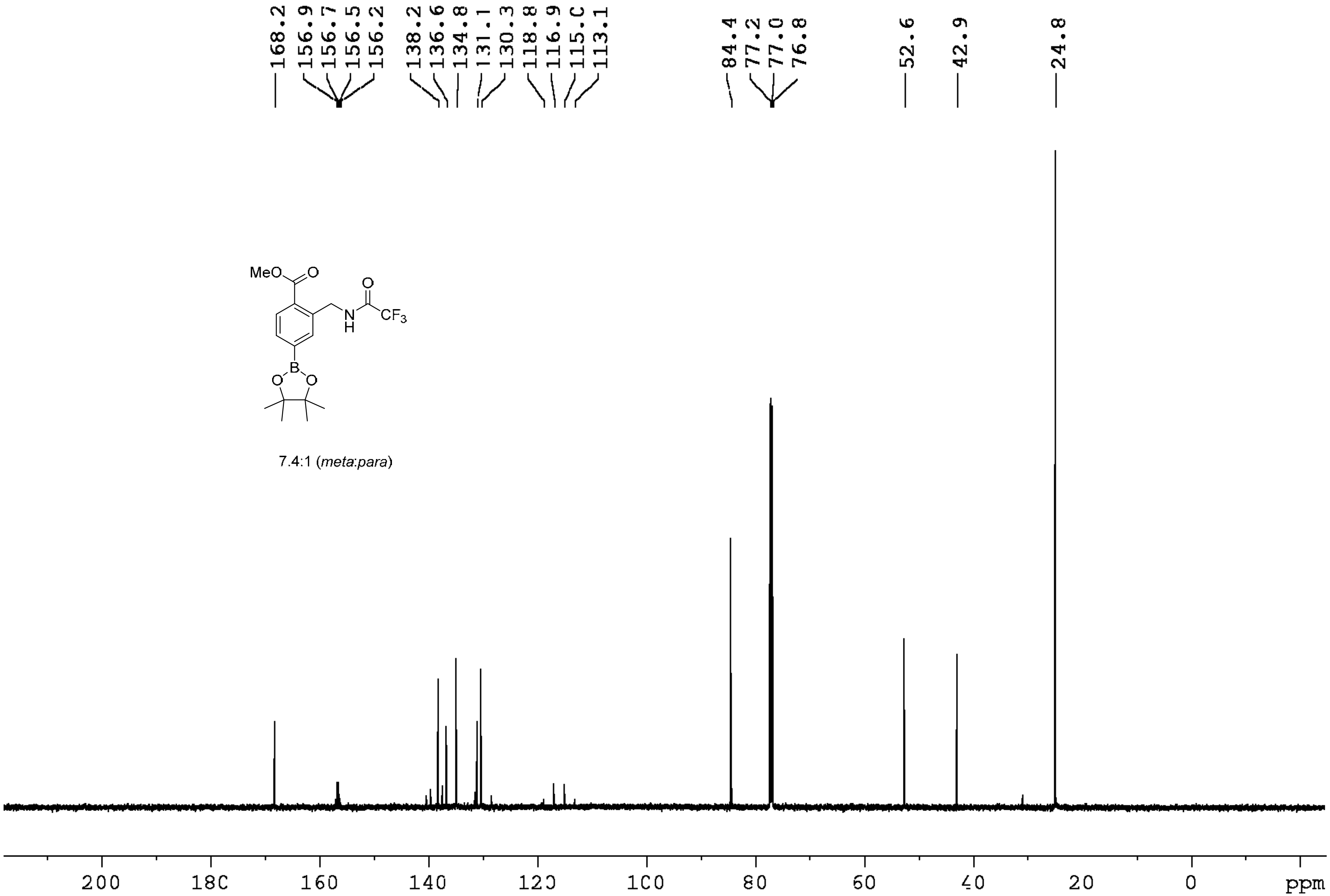

^{1}H NMR (600 MHz, $CDCl_3$) Methyl 4-(4,4,5,5-tetramethyl-1,3,2-dioxaborolan-2-yl)-2-((2,2,2-trifluoroacetamido)methyl)benzoate (3g) – Purified after reaction with dtbpy

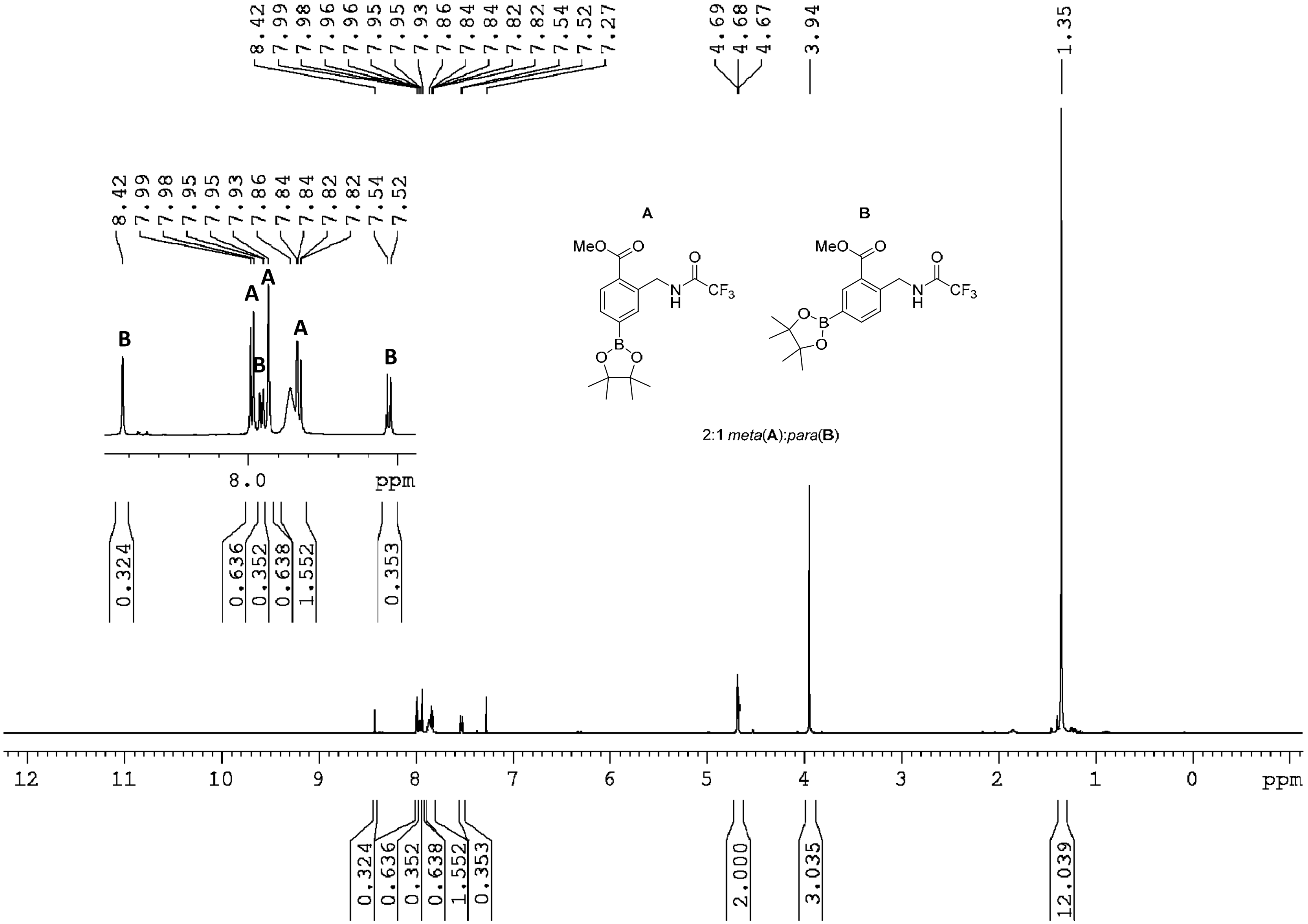

^{13}C NMR (151 MHz, $CDCl_3$) Methyl 4-(4,4,5,5-tetramethyl-1,3,2-dioxaborolan-2-yl)-2-((2,2,2-trifluoroacetamido)methyl)benzoate (3g) – Purified after reaction with dtbpy

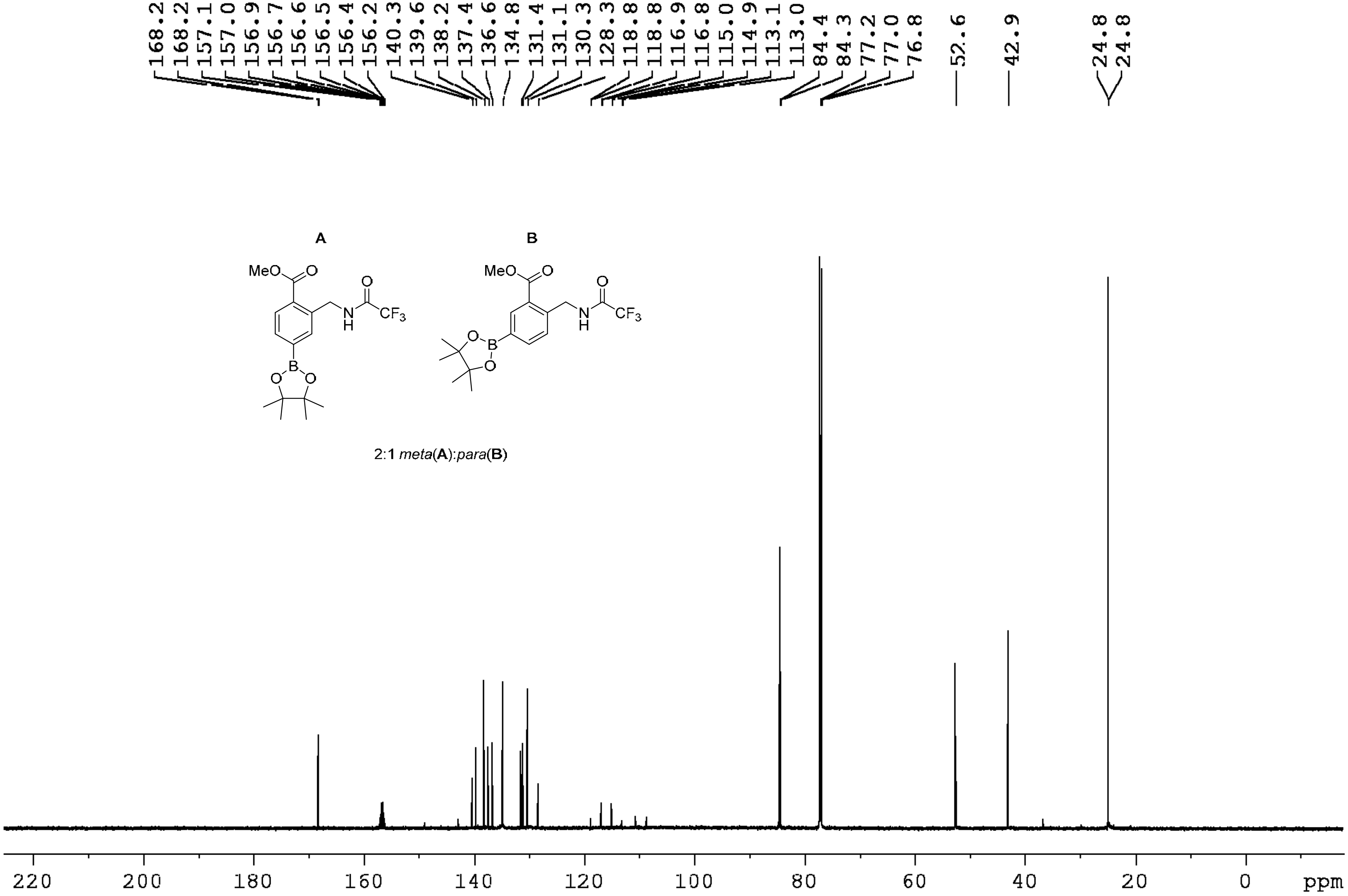

^{1}H NMR (600 MHz, $CDCl_3$) *N*-(3,5-bis(4,4,5,5-tetramethyl-1,3,2-dioxaborolan-2-yl)benzyl)-2,2,2-trifluoroacetamide (3h) – Crude after reaction with 1a

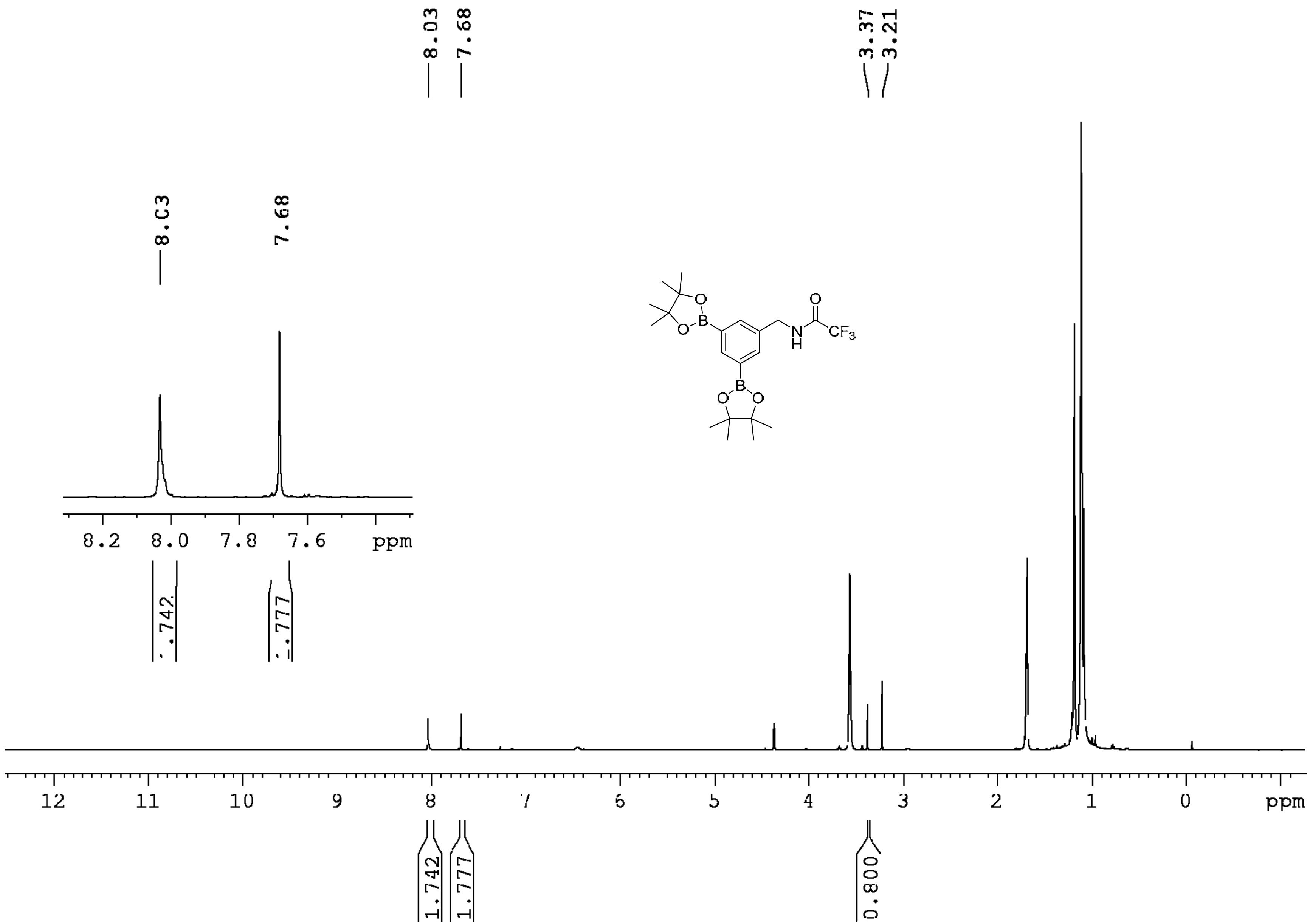

^{1}H NMR (600 MHz, $CDCl_3$) *N*-(3,5-bis(4,4,5,5-tetramethyl-1,3,2-dioxaborolan-2-yl)benzyl)-2,2,2-trifluoroacetamide (3h) – Purified after reaction with 1a

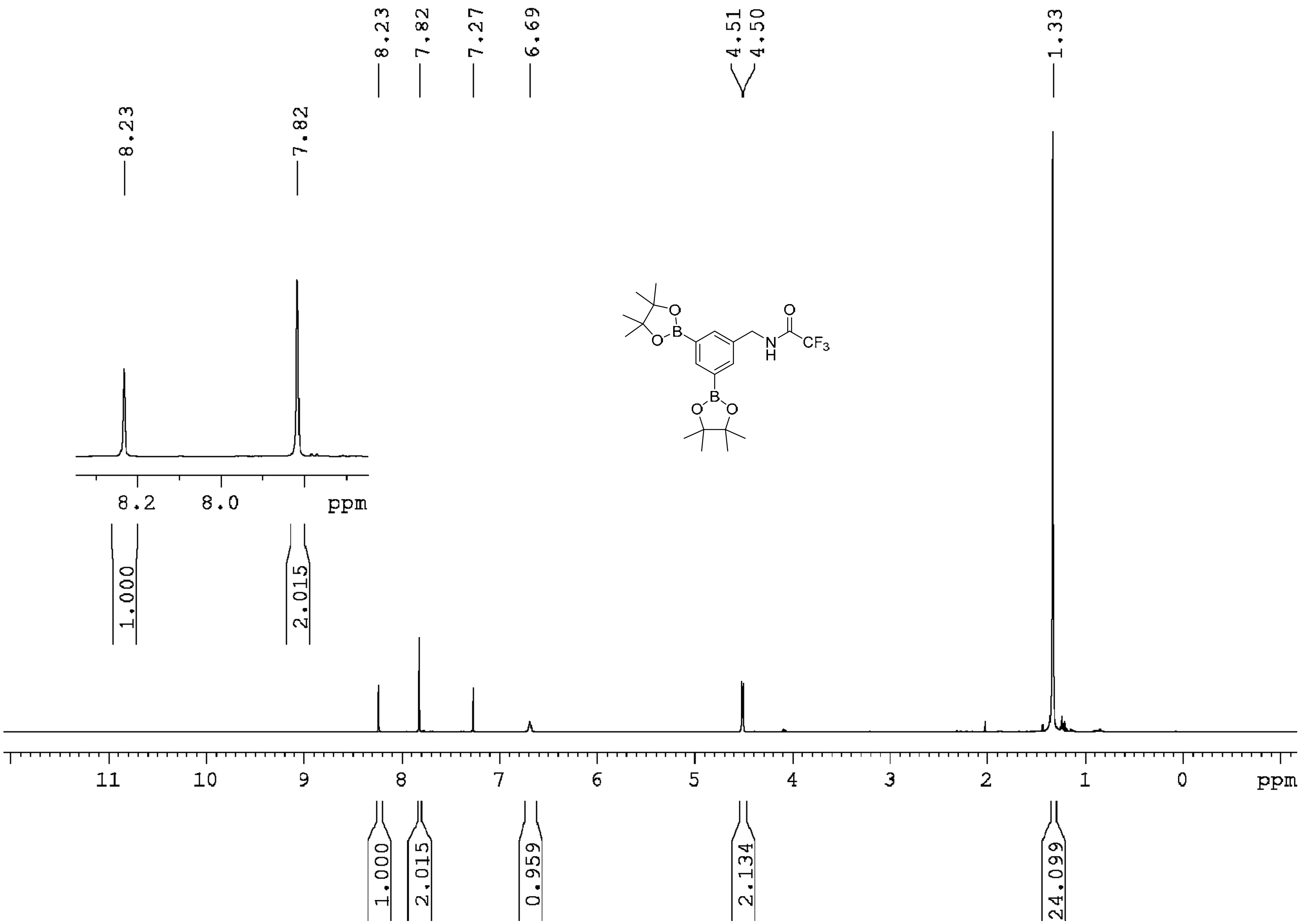

^{13}C NMR (151 MHz, $CDCl_3$) *N*-(3,5-bis(4,4,5,5-tetramethyl-1,3,2-dioxaborolan-2-yl)benzyl)-2,2,2-trifluoroacetamide (3h) – Purified after reaction with 1a

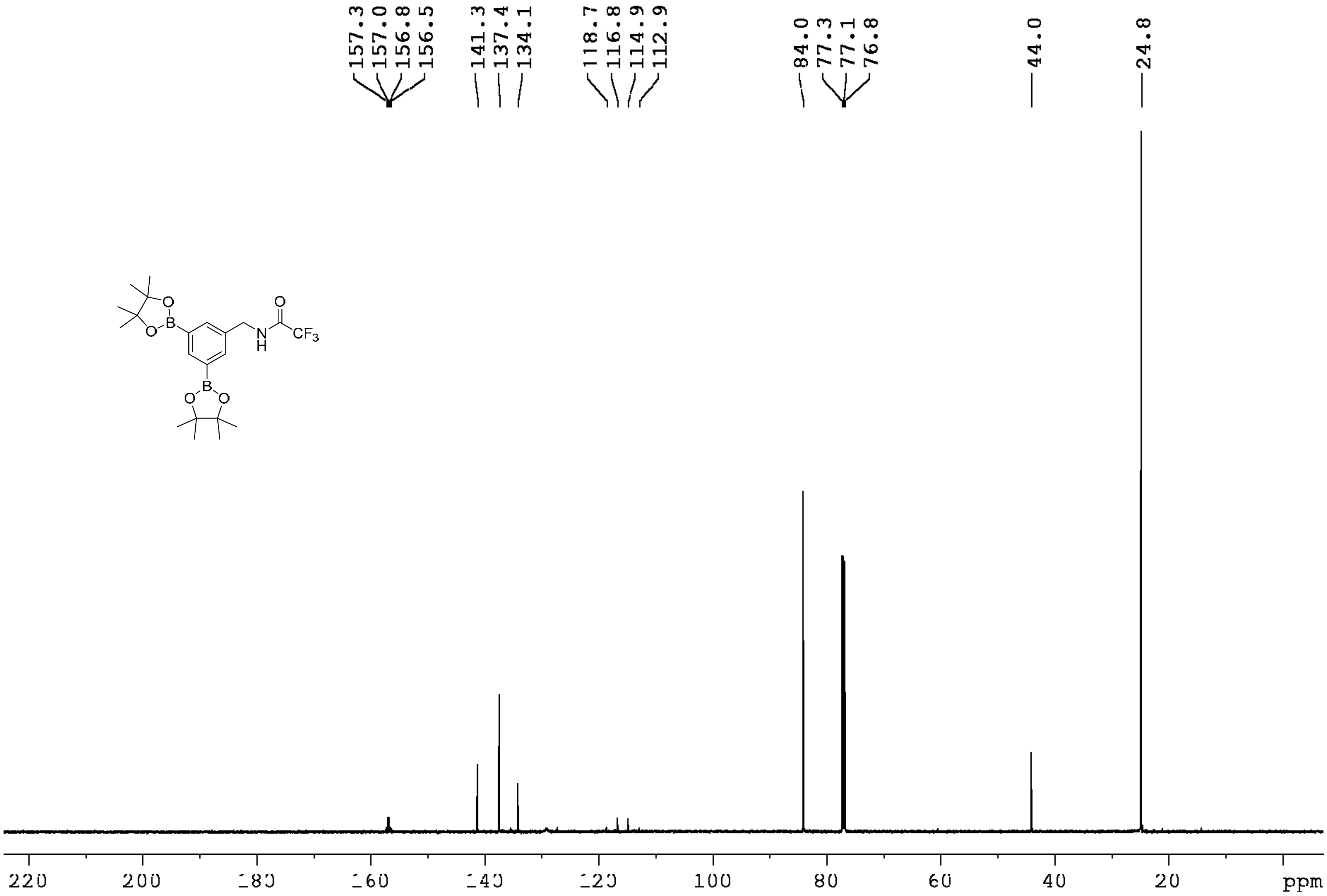

^{1}H NMR (500 MHz, $CDCl_3$) 2,2,2-Trifluoro-*N*-(3-(4,4,5,5-tetramethyl-1,3,2-dioxaborolan-2-yl)benzyl)acetamide (3h) – Mono *meta* fraction isolated from reaction with dtbpy

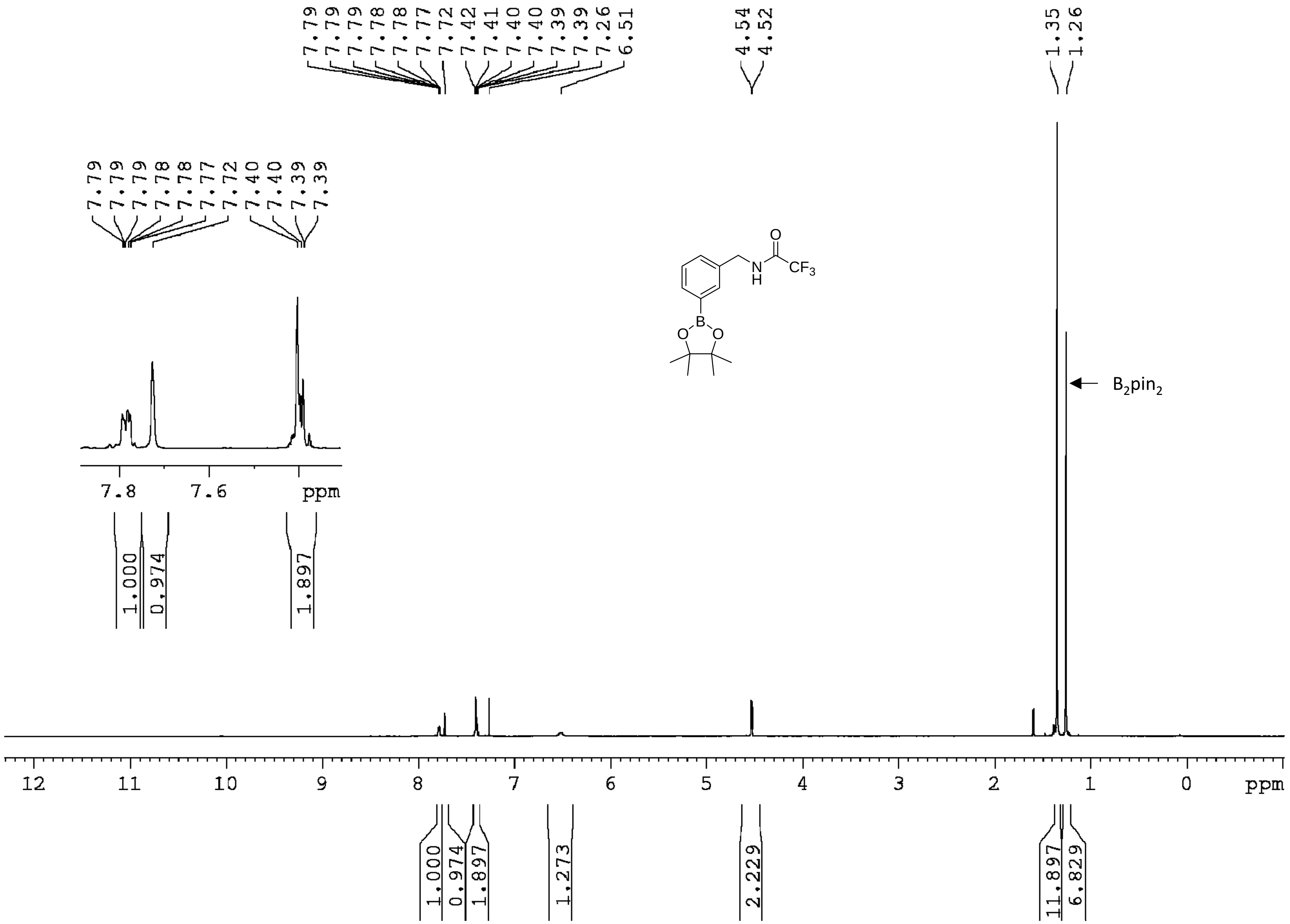

^{13}C NMR (126 MHz, $CDCl_3$) 2,2,2-Trifluoro-*N*-(3-(4,4,5,5-tetramethyl-1,3,2-dioxaborolan-2-yl)benzyl)acetamide (3h) – Mono *meta* fraction isolated from reaction with dtbpy

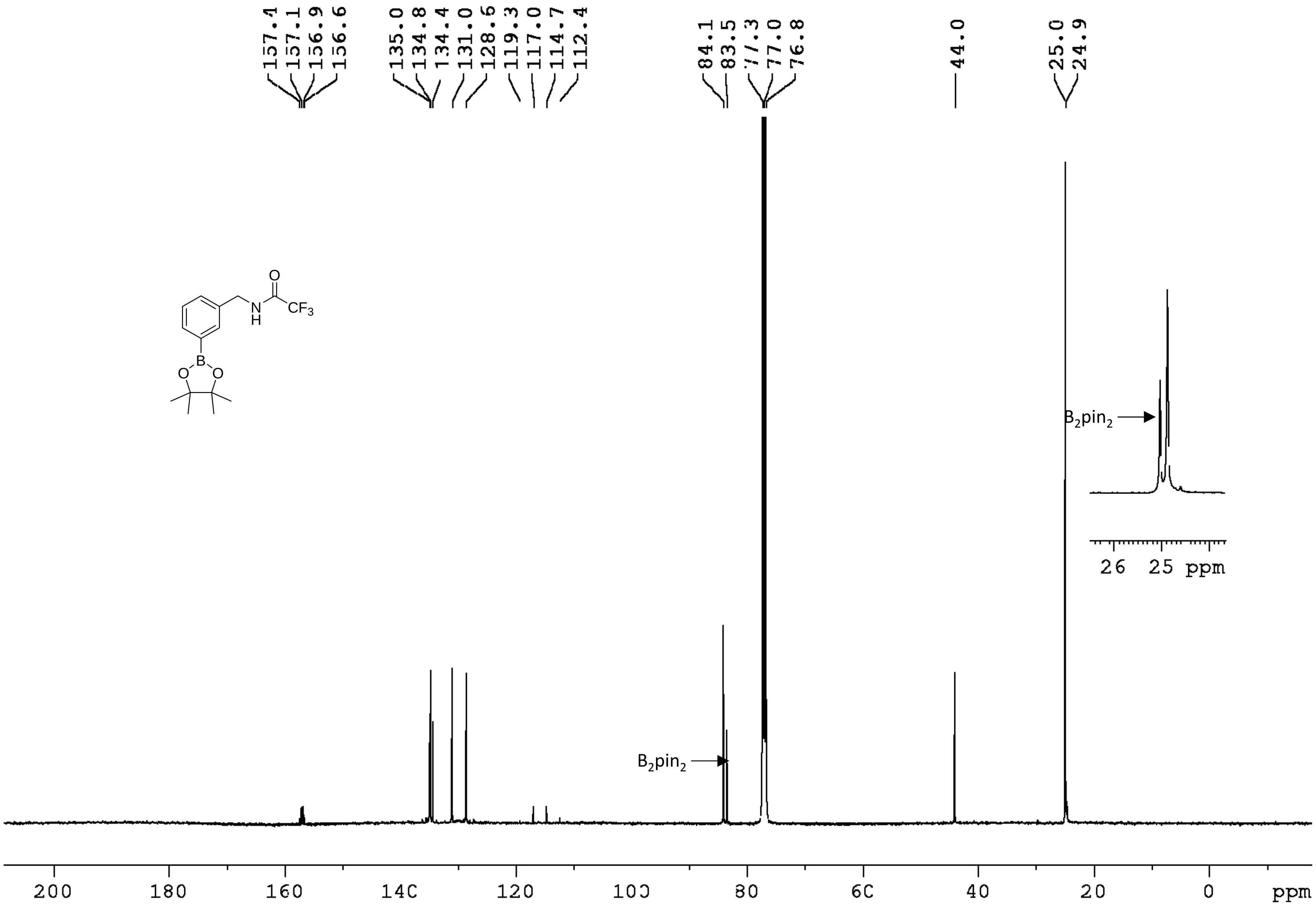

^{1}H NMR (500 MHz, $CDCl_3$) 2,2,2-Trifluoro-*N*-(5-(4,4,5,5-tetramethyl-1,3,2-dioxaborolan-2-yl)-2-(trifluoromethyl)benzyl)acetamide (3h) – Mixture of isomers after purification after reaction with dtbpy

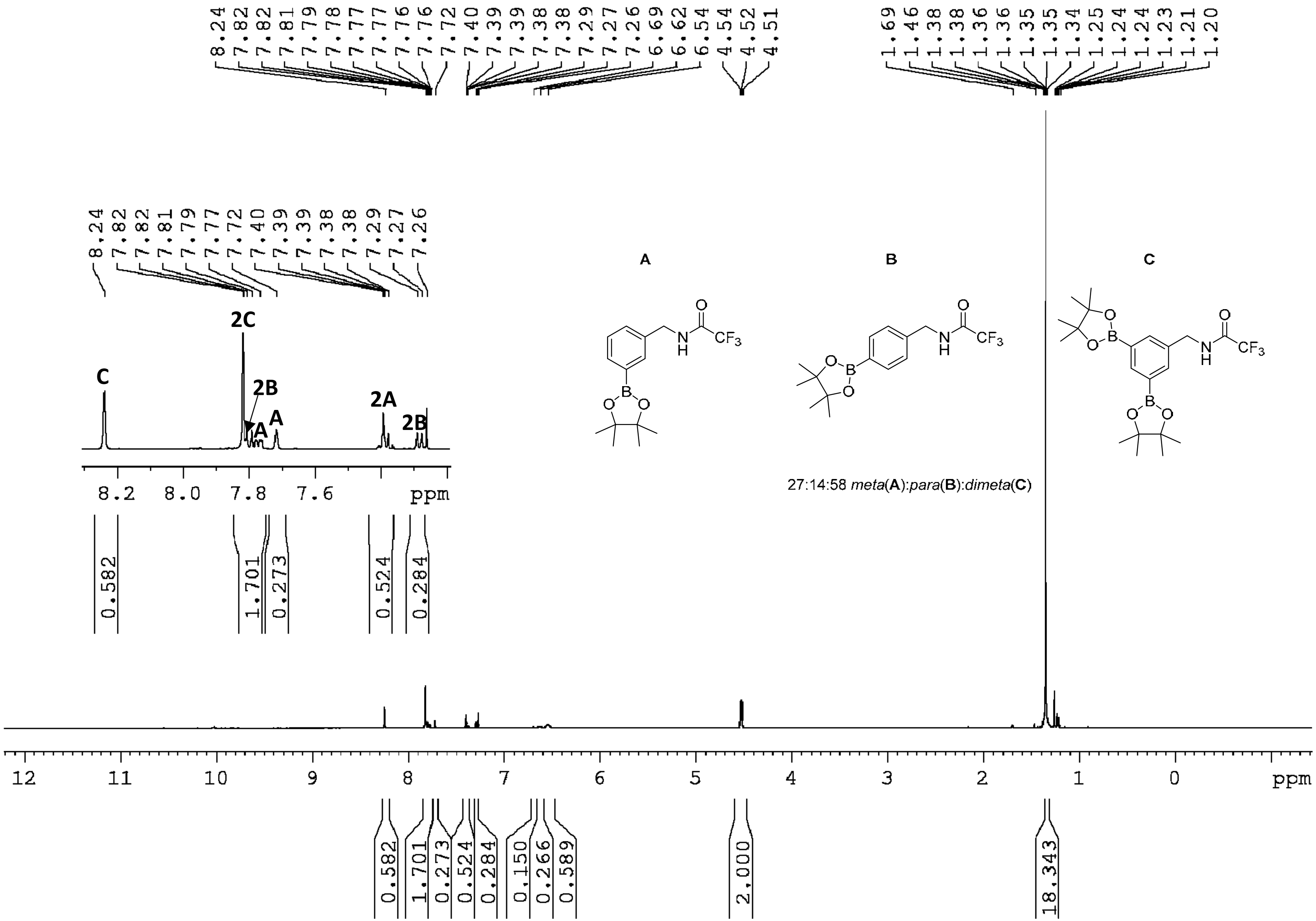

^{13}C NMR (126 MHz, $CDCl_3$) 2,2,2-Trifluoro-*N*-(5-(4,4,5,5-tetramethyl-1,3,2-dioxaborolan-2-yl)-2-(trifluoromethyl)benzyl)acetamide (3h) – Mixture of isomers after purification after reaction with dtbpy

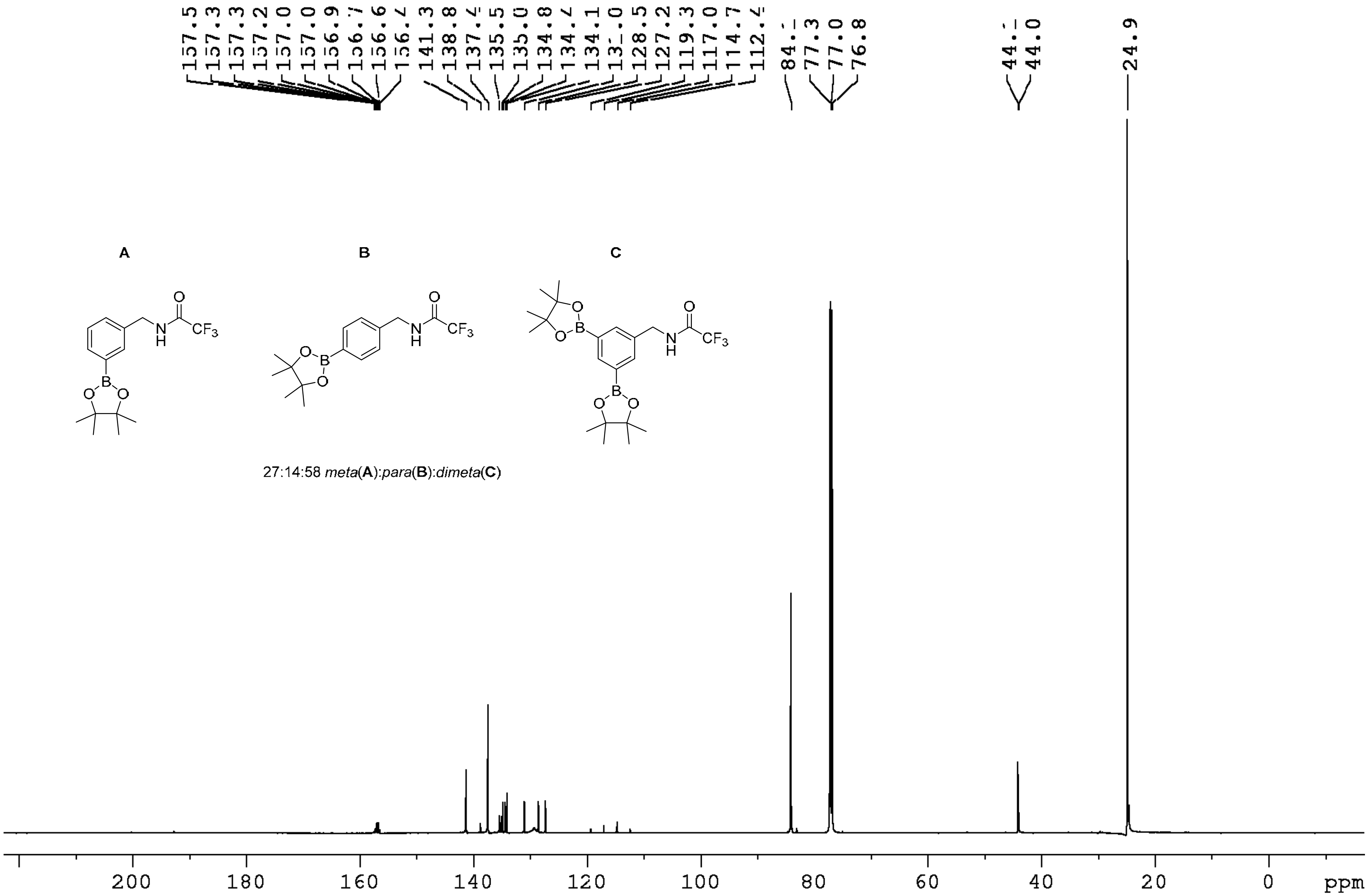

^{1}H NMR (600 MHz, $CDCl_3$) (S)-*N*-(1-(3,5-bis(4,4,5,5-tetramethyl-1,3,2-dioxaborolan-2-yl)phenyl)ethyl)-2,2,2-trifluoroacetamide (3i) – Crude after reaction with 1a

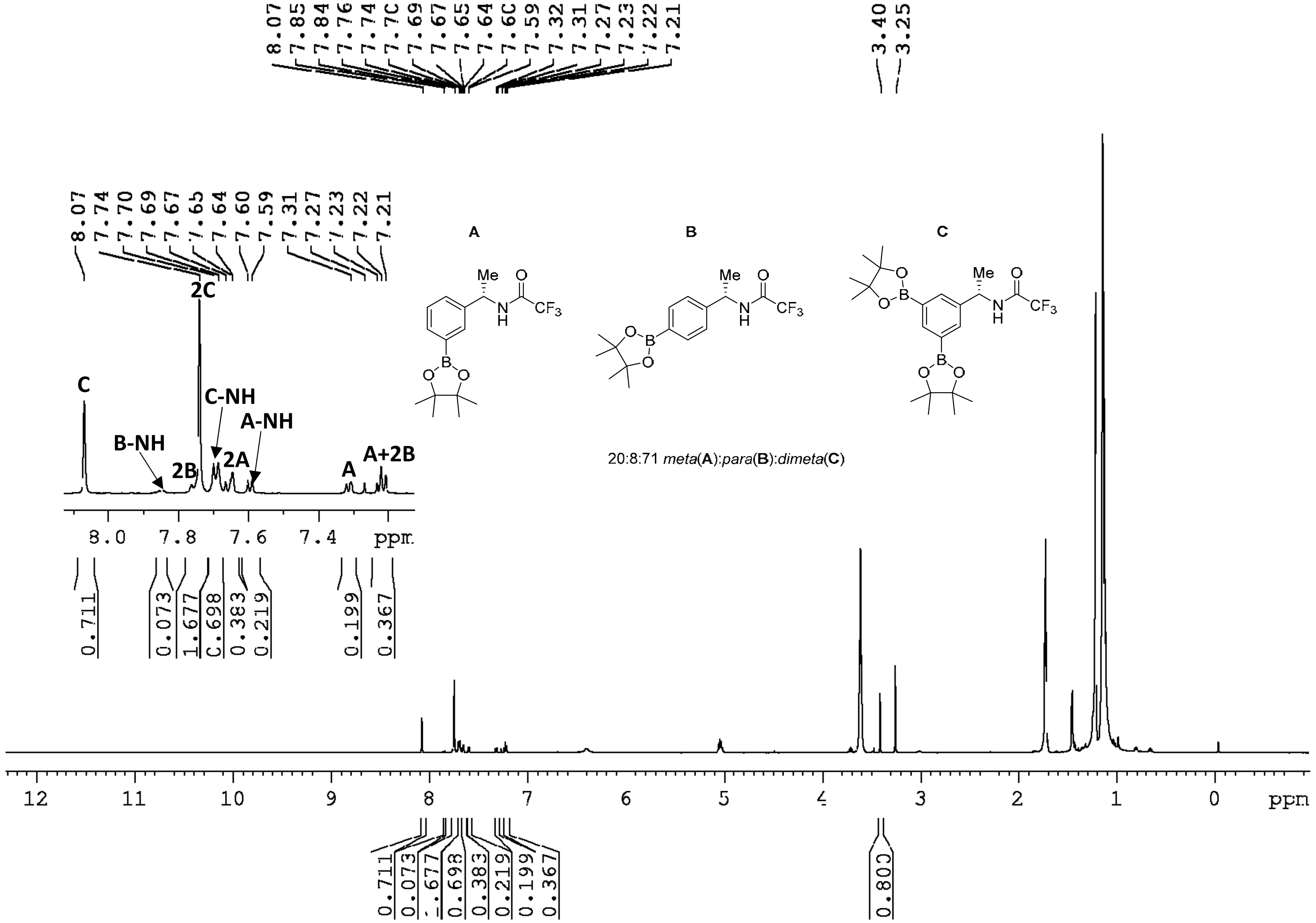

^{1}H NMR (600 MHz, $CDCl_3$) (S)-*N*-(1-(3,5-bis(4,4,5,5-tetramethyl-1,3,2-dioxaborolan-2-yl)phenyl)ethyl)-2,2,2-trifluoroacetamide (3i) – *Dimeta* fraction isolated after purification after reaction with 1a

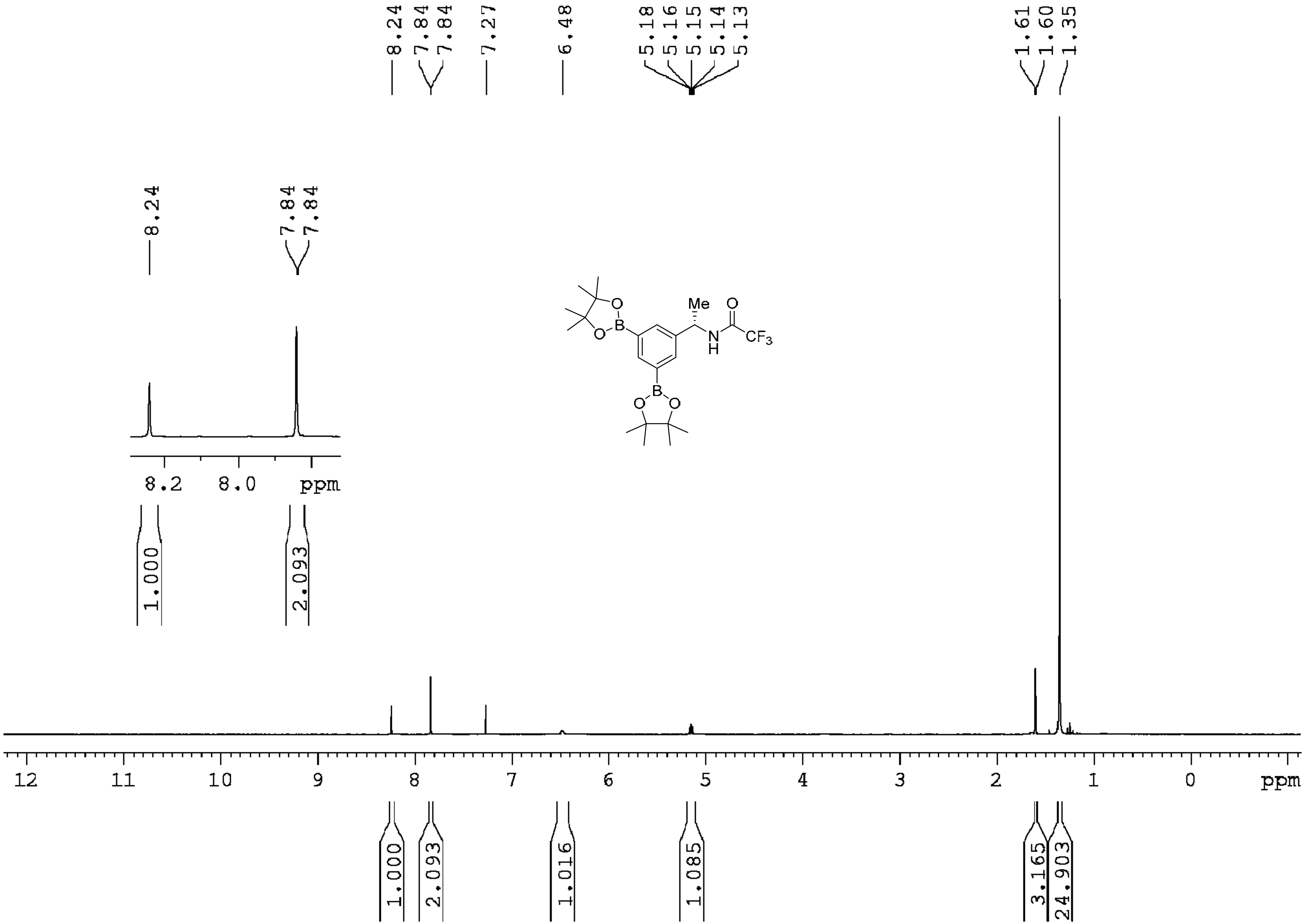

^{13}C NMR (151 MHz, $CDCl_3$) (S)-*N*-(1-(3,5-bis(4,4,5,5-tetramethyl-1,3,2-dioxaborolan-2-yl)phenyl)ethyl)-2,2,2-trifluoroacetamide (3i) – *Dimeta* fraction isolated after purification after reaction with 1a

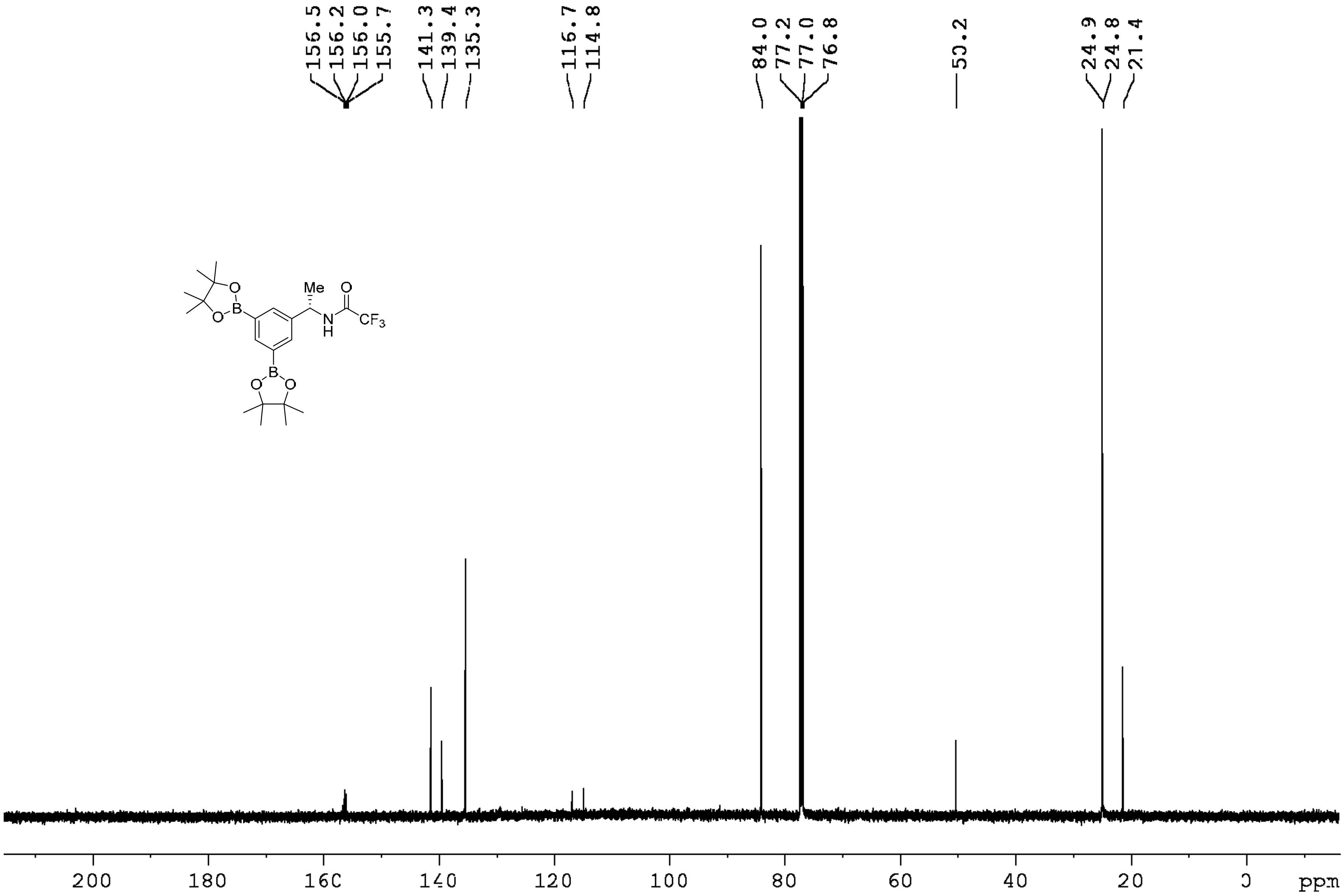

^{1}H NMR (500 MHz, $CDCl_3$) (S)-*N*-(1-(3,5-bis(4,4,5,5-tetramethyl-1,3,2-dioxaborolan-2-yl)phenyl)ethyl)-2,2,2-trifluoroacetamide (3i) – Mixed fraction of isomers after purification after reaction with 1a

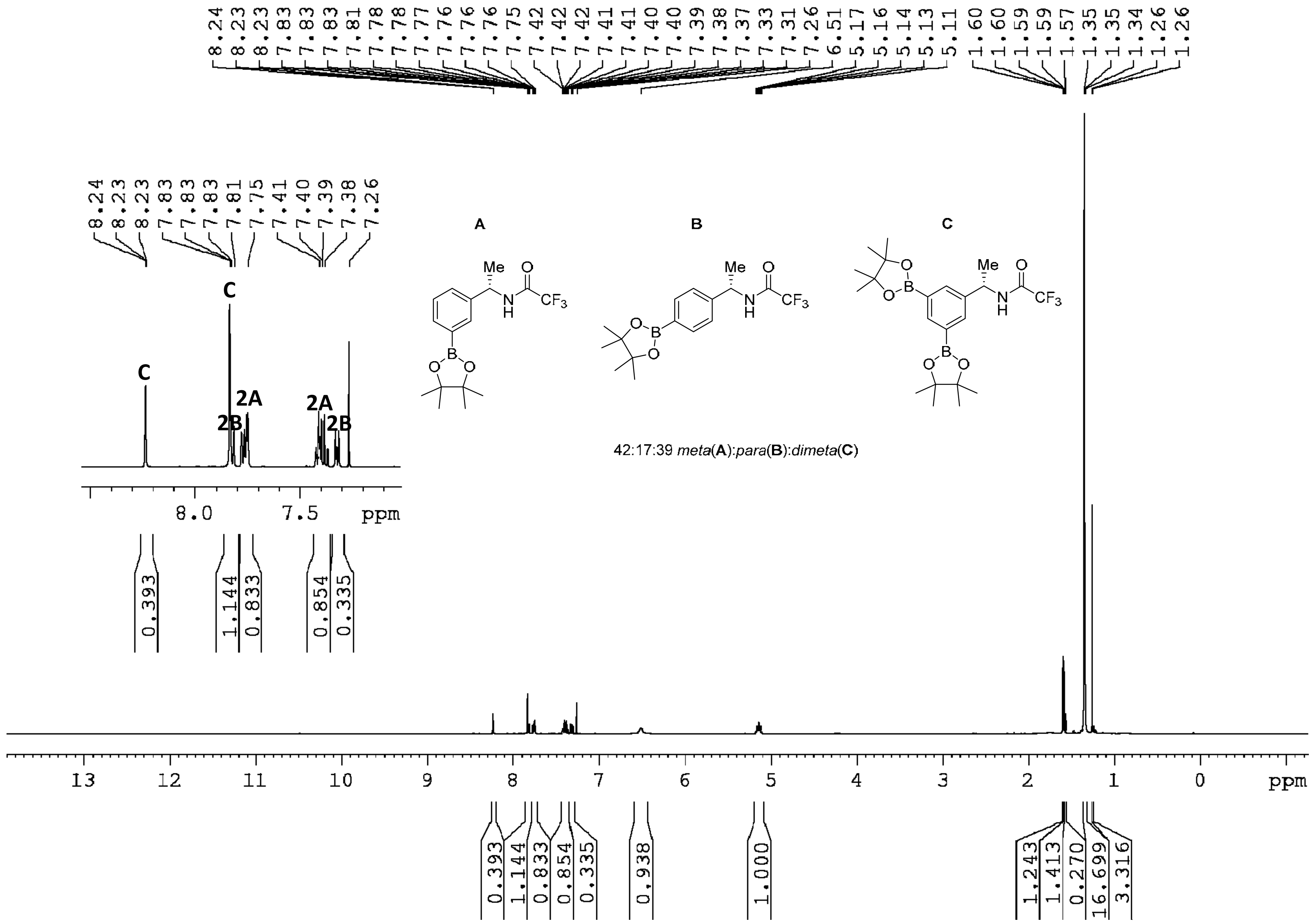

^{13}C NMR (126 MHz, $CDCl_3$) (S)-*N*-(1-(3,5-bis(4,4,5,5-tetramethyl-1,3,2-dioxaborolan-2-yl)phenyl)ethyl)-2,2,2-trifluoroacetamide (3i) - Mixed fraction of isomers after purification after reaction with 1a

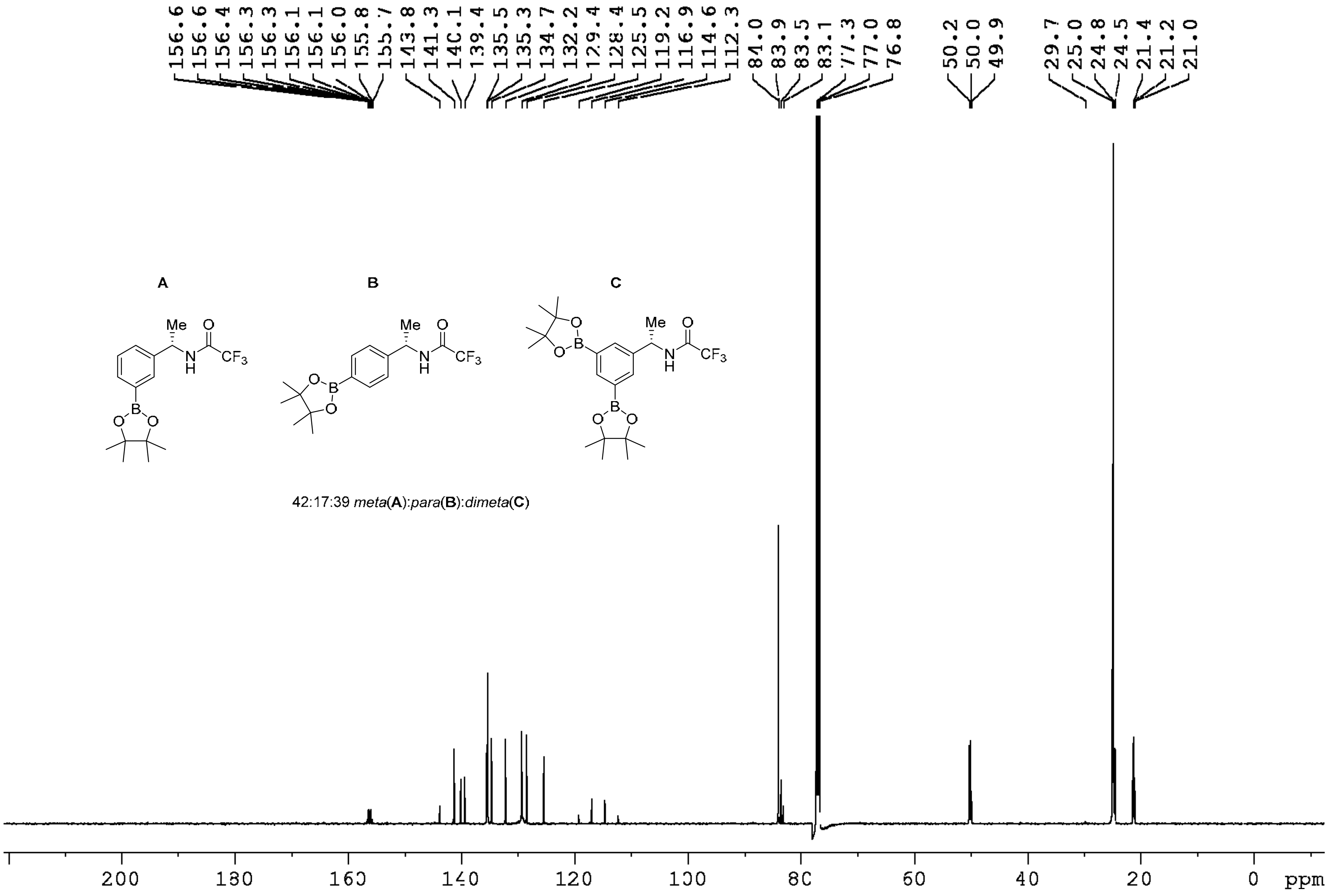

^{1}H NMR (600 MHz, $CDCl_3$) 2,2,2-Trifluoro-*N*-(2-fluoro-3,5-bis(4,4,5,5-tetramethyl-1,3,2-dioxaborolan-2-yl)benzyl)acetamide (3j) – Crude after reaction with 1a

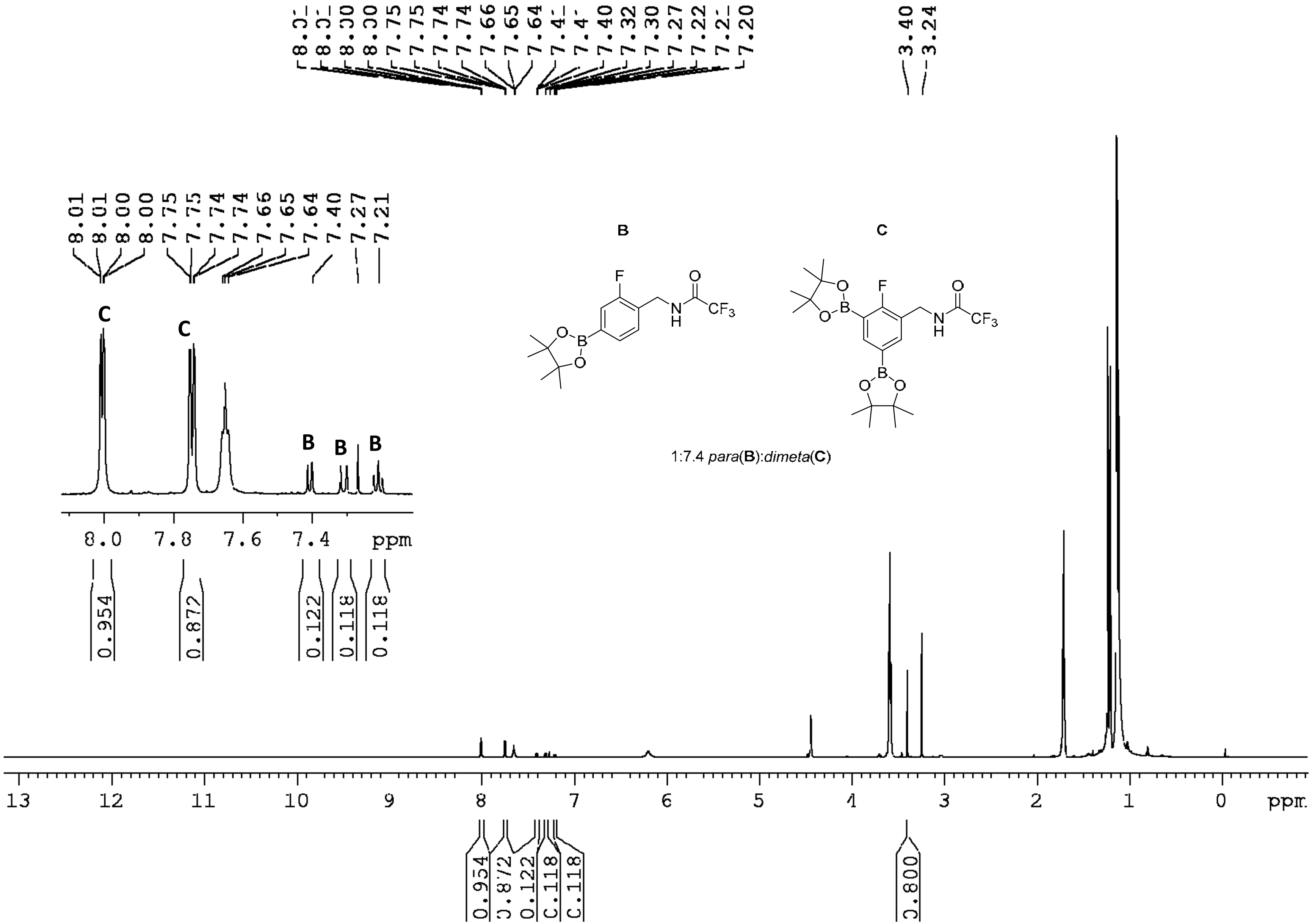

^{1}H NMR (600 MHz, $CDCl_3$) 2,2,2-Trifluoro-*N*-(2-fluoro-3,5-bis(4,4,5,5-tetramethyl-1,3,2-dioxaborolan-2-yl)benzyl)acetamide (3j) – Purified *dimeta* fraction after reaction with 1a

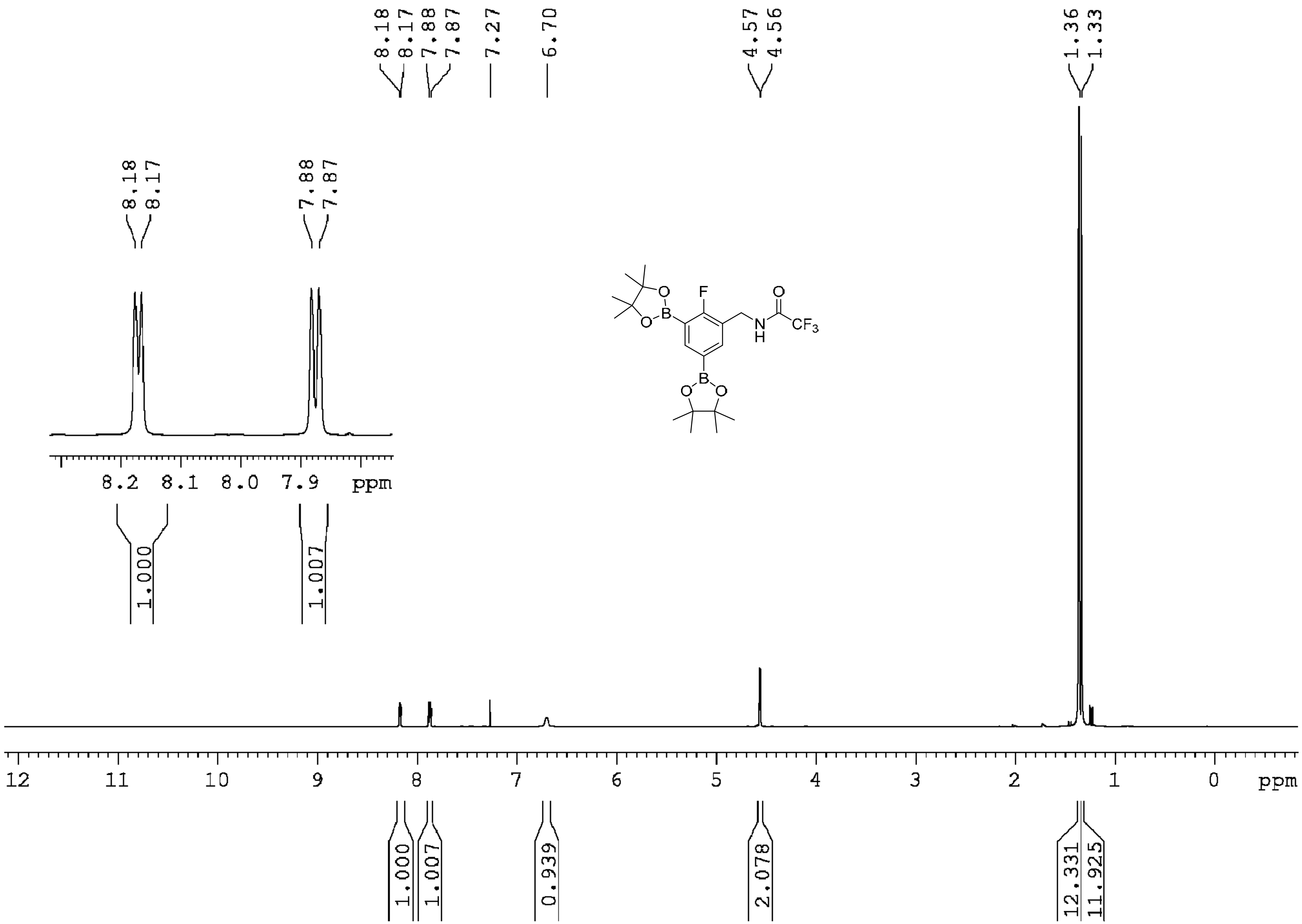

^{13}C NMR (151 MHz, $CDCl_3$) 2,2,2-Trifluoro-*N*-(2-fluoro-3,5-bis(4,4,5,5-tetramethyl-1,3,2-dioxaborolan-2-yl)benzyl)acetamide (3j) – Purified *dimeta* fraction after reaction with 1a

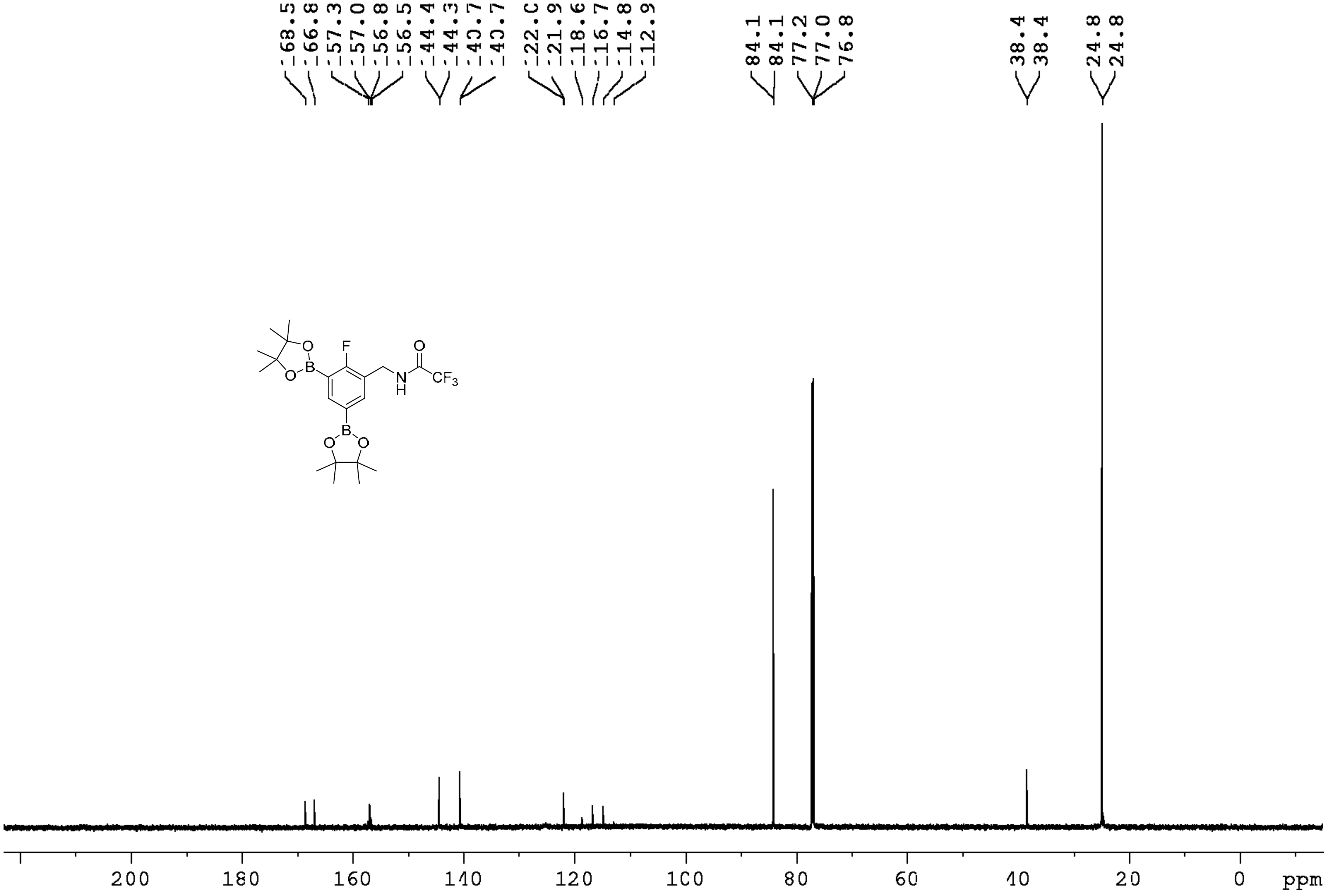

^{1}H NMR (600 MHz, $CDCl_3$) 2,2,2-Trifluoro-*N*-(2-fluoro-4-(4,4,5,5-tetramethyl-1,3,2-dioxaborolan-2-yl)benzyl)acetamide (3j) – Purified *para* enriched fraction after reaction with dtbpy

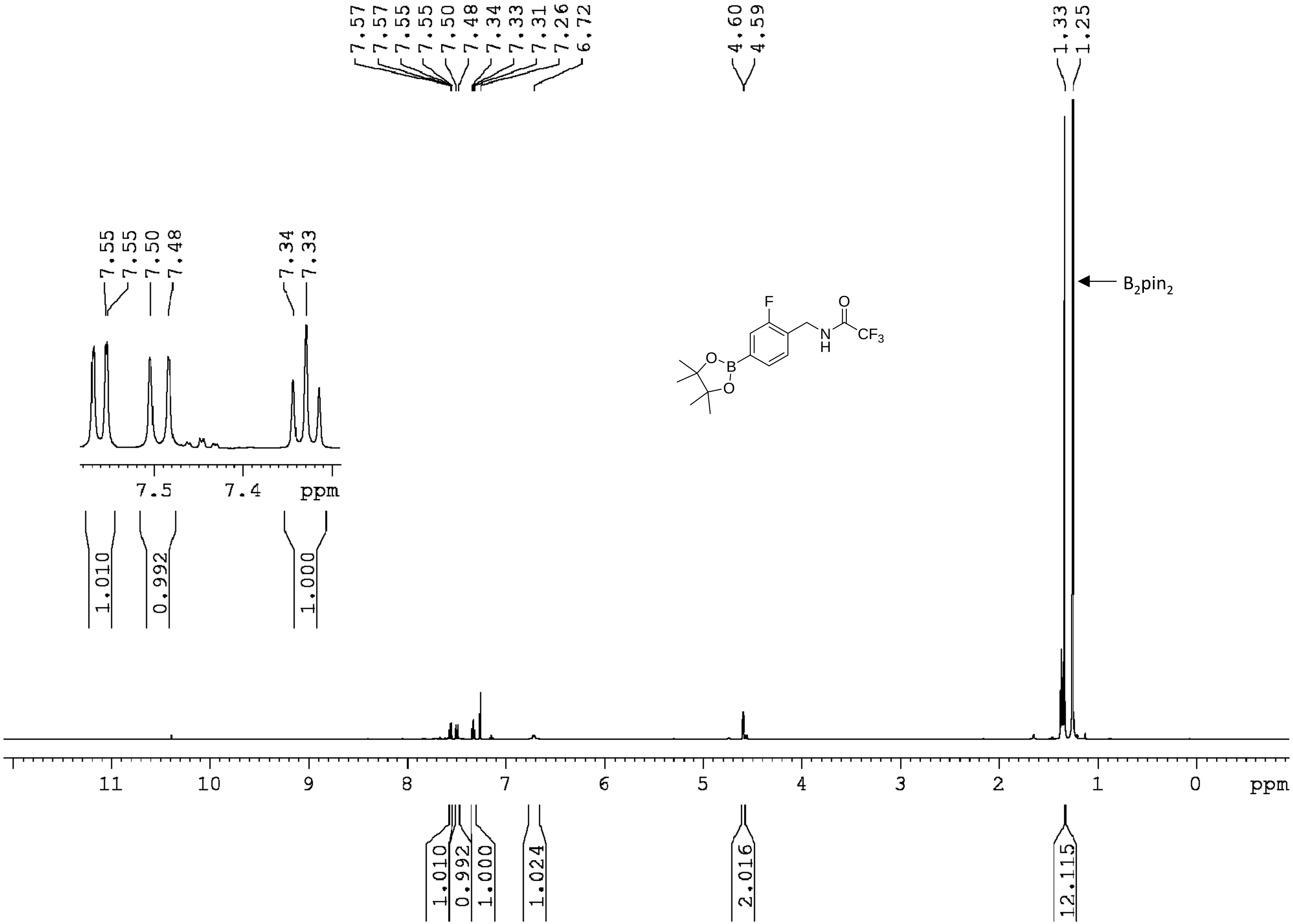

^{13}C NMR (151 MHz, $CDCl_3$) 2,2,2-Trifluoro-*N*-(2-fluoro-4-(4,4,5,5-tetramethyl-1,3,2-dioxaborolan-2-yl)benzyl)acetamide (3j) - Purified *para* enriched fraction after reaction with dtbpy

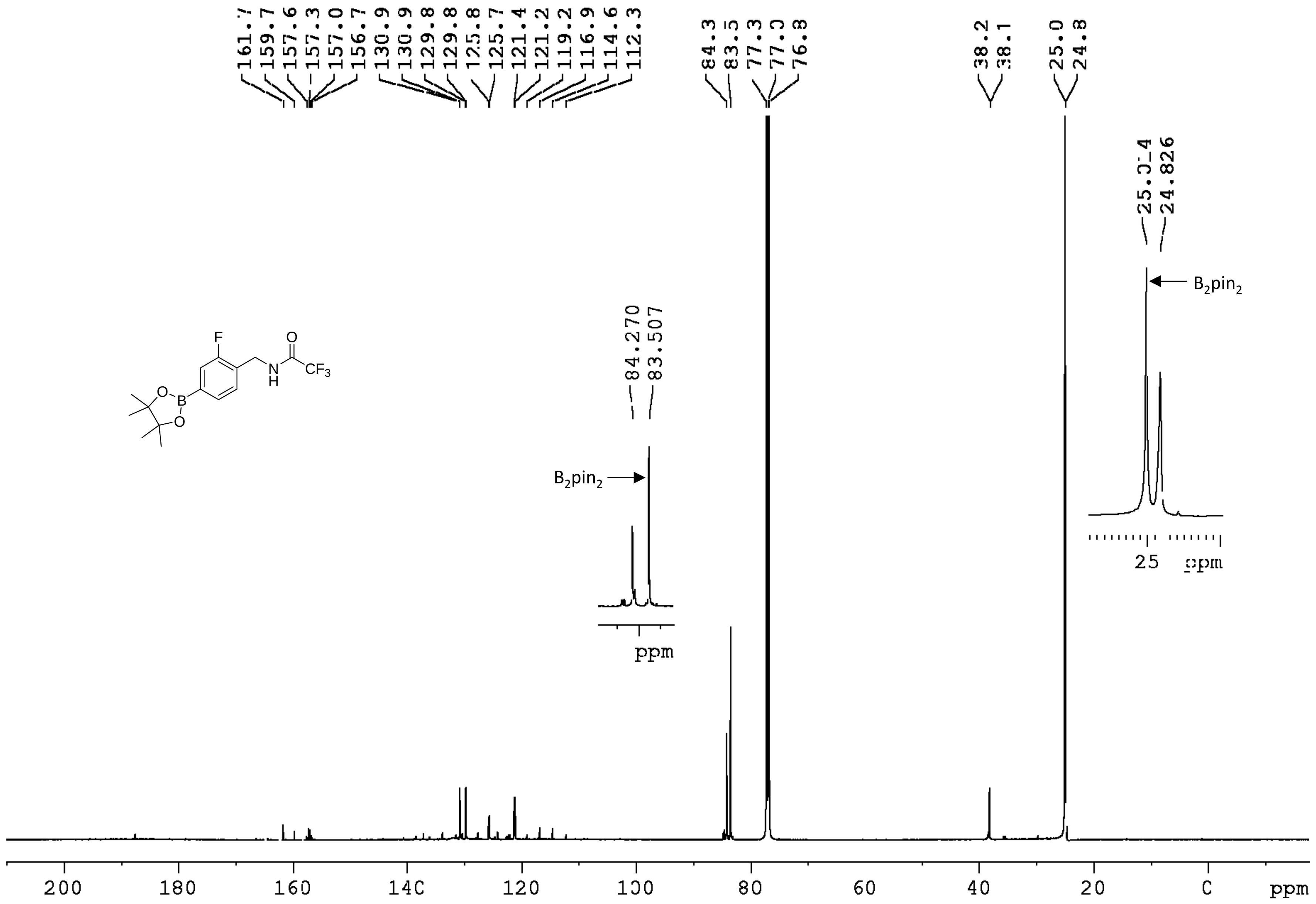

^{1}H NMR (600 MHz, $CDCl_3$) 2,2,2-Trifluoro-*N*-((4-(4,4,5,5-tetramethyl-1,3,2-dioxaborolan-2-yl)pyridin-2-yl)methyl)acetamide (3k) – Crude after reaction with 1a

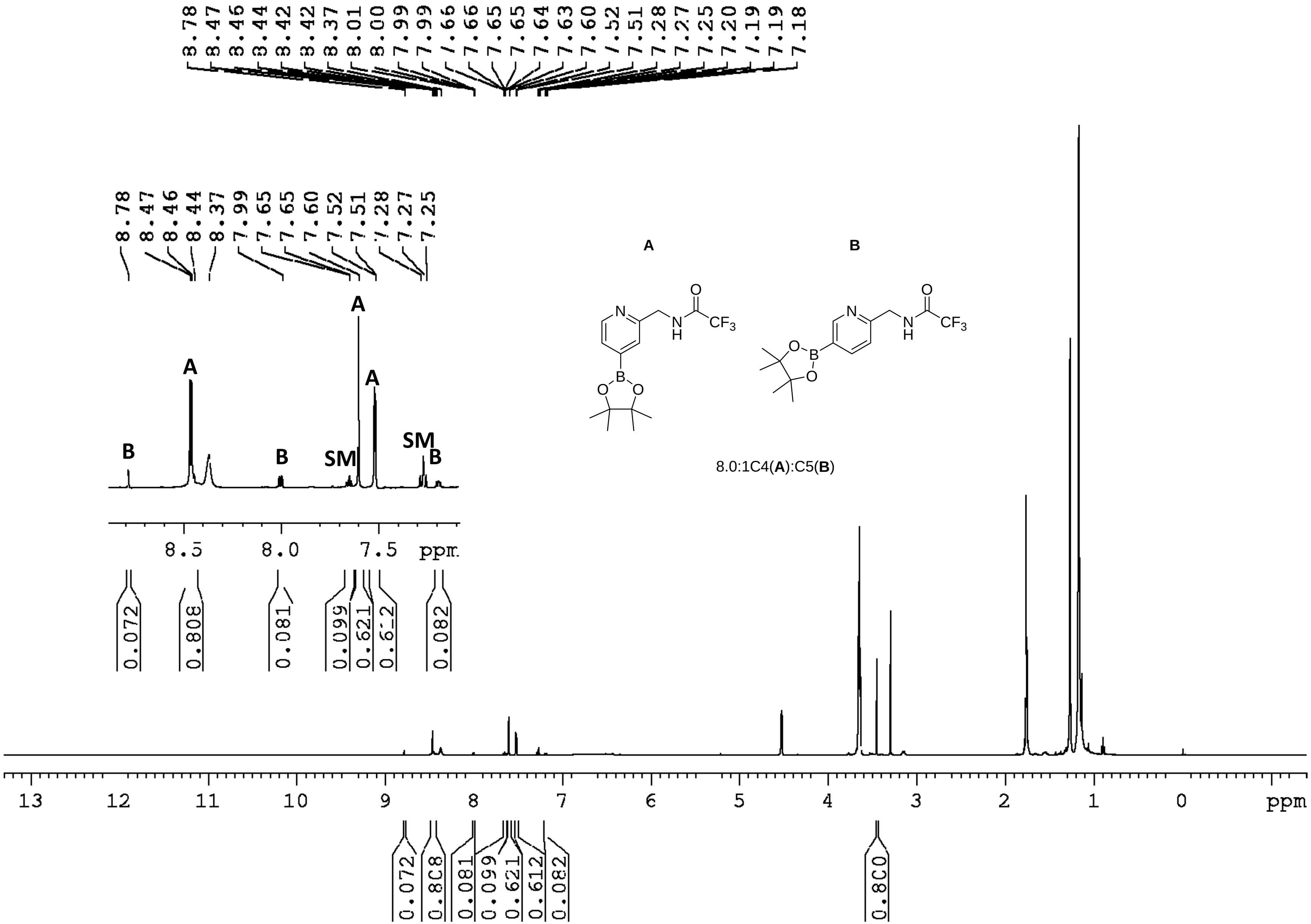

^{1}H NMR (600 MHz, $CDCl_3$) 2,2,2-Trifluoro-*N*-((4-(4,4,5,5-tetramethyl-1,3,2-dioxaborolan-2-yl)pyridin-2-yl)methyl)acetamide (3k) – Clean fraction of *meta* product after attempted purification after reaction with 1a

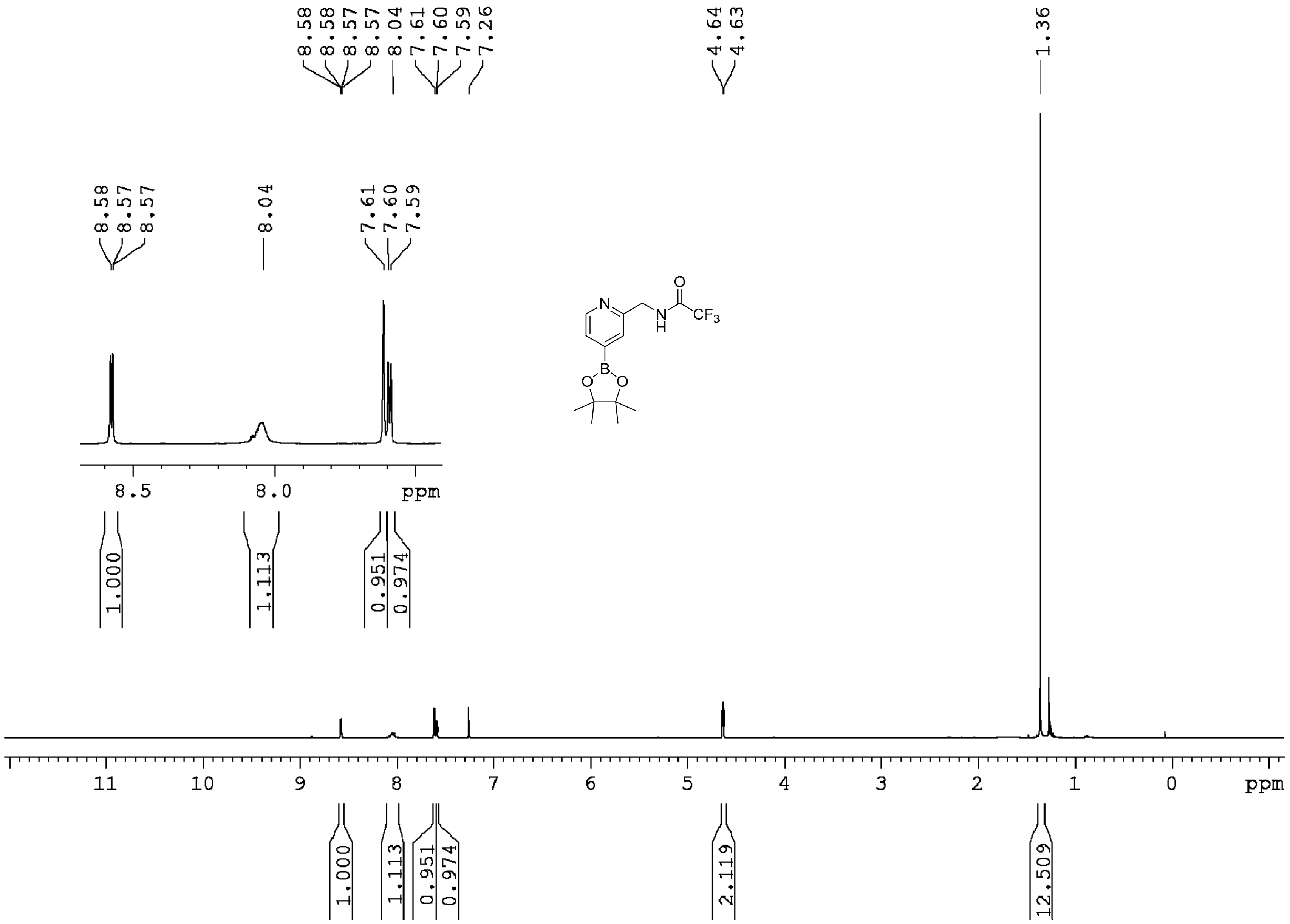

^{13}C NMR (126 MHz, $CDCl_3$2,2,2-Trifluoro-*N*-((4-(4,4,5,5-tetramethyl-1,3,2-dioxaborolan-2-yl)pyridin-2-yl)methyl)acetamide (3k) – Clean fraction of *meta* product after attempted purification after reaction with 1a

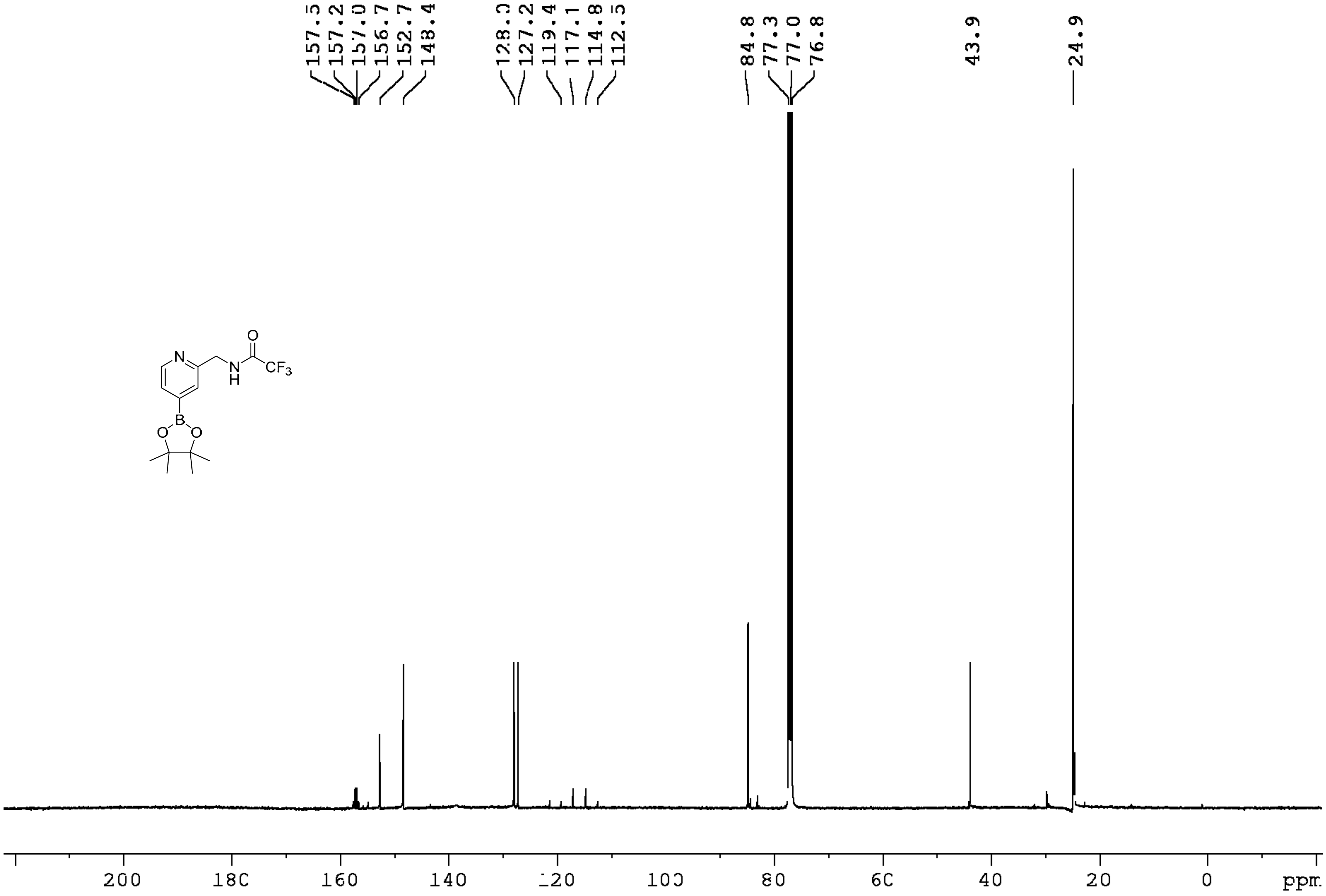

^{1}H NMR (600 MHz, $CDCl_3$) 2,2,2-Trifluoro-*N*-((4-(4,4,5,5-tetramethyl-1,3,2-dioxaborolan-2-yl)pyridin-2-yl)methyl)acetamide (3k) – Crude after reaction with dtbpy

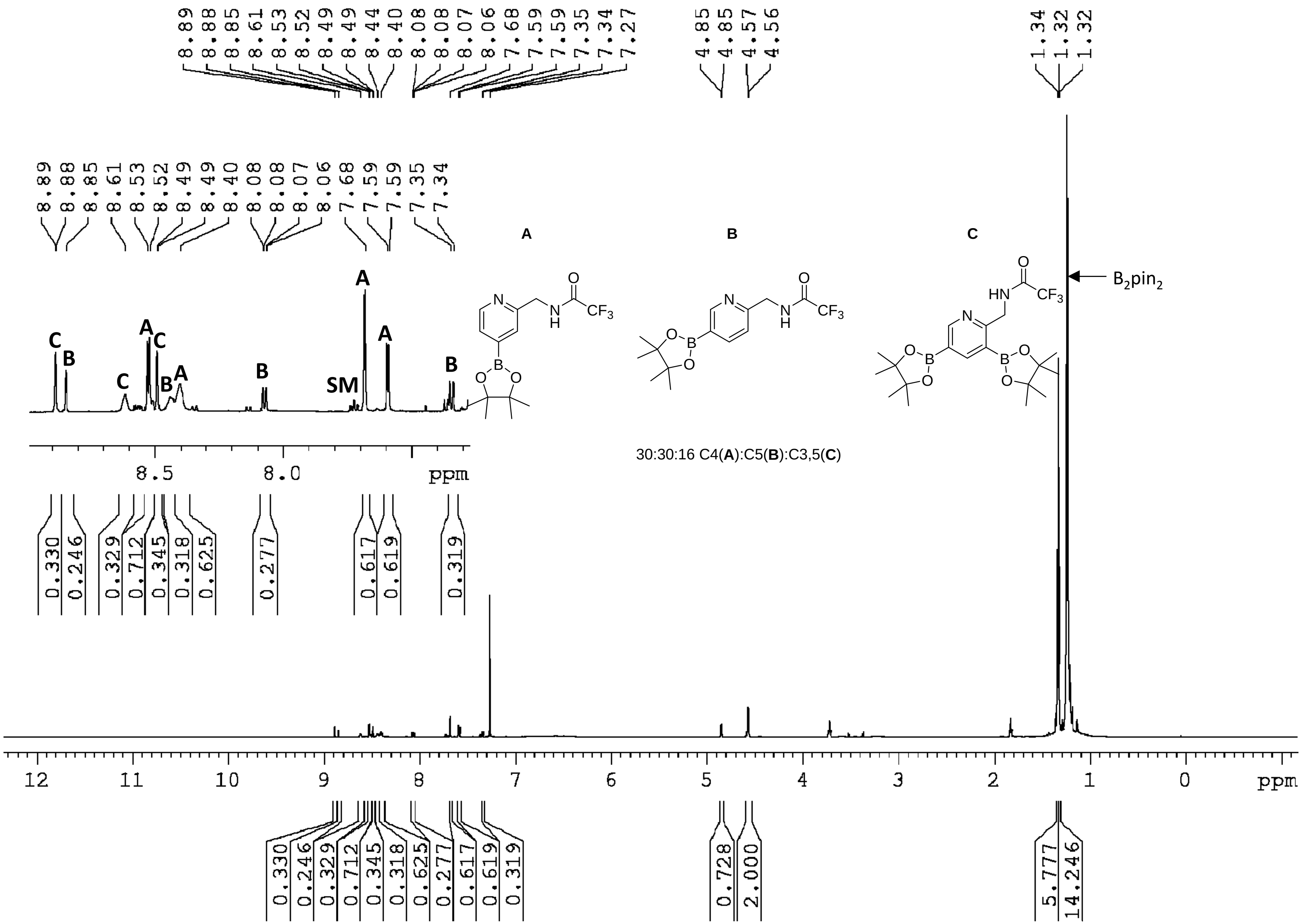

^{13}C NMR (151 MHz, CDCl$_3$) 2,2,2-Trifluoro-*N*-((4-(4,4,5,5-tetramethyl-1,3,2-dioxaborolan-2-yl)pyridin-2-yl)methyl)acetamide (3k) – Crude after reaction with dtbpy

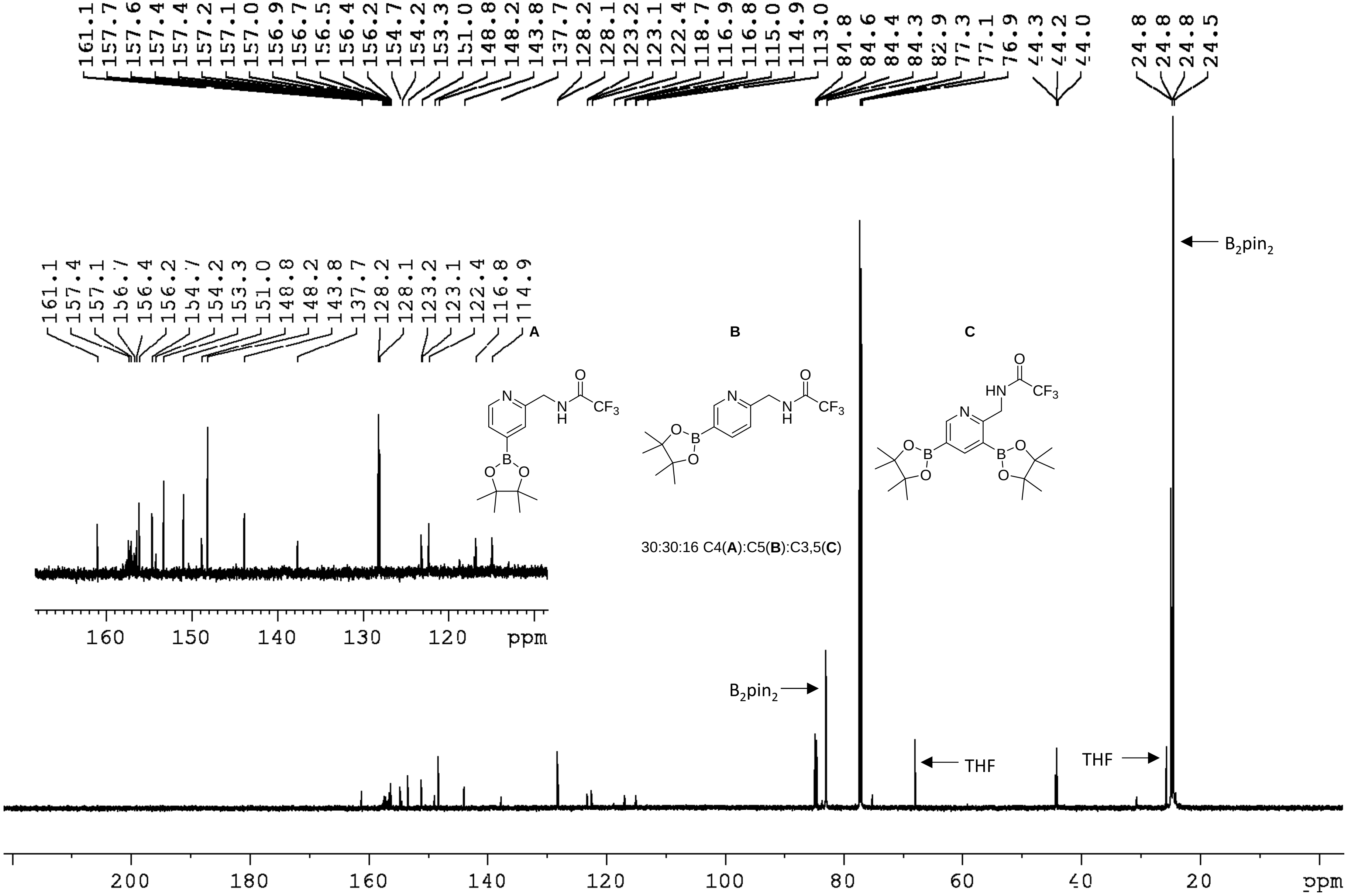

^{1}H NMR (500 MHz, $CDCl_3$) *N*-(5-(4,4,5,5-tetramethyl-1,3,2-dioxaborolan-2-yl)-2-(trifluoromethyl)benzyl)methanesulfonamide (3m) – Crude after reaction with 1a

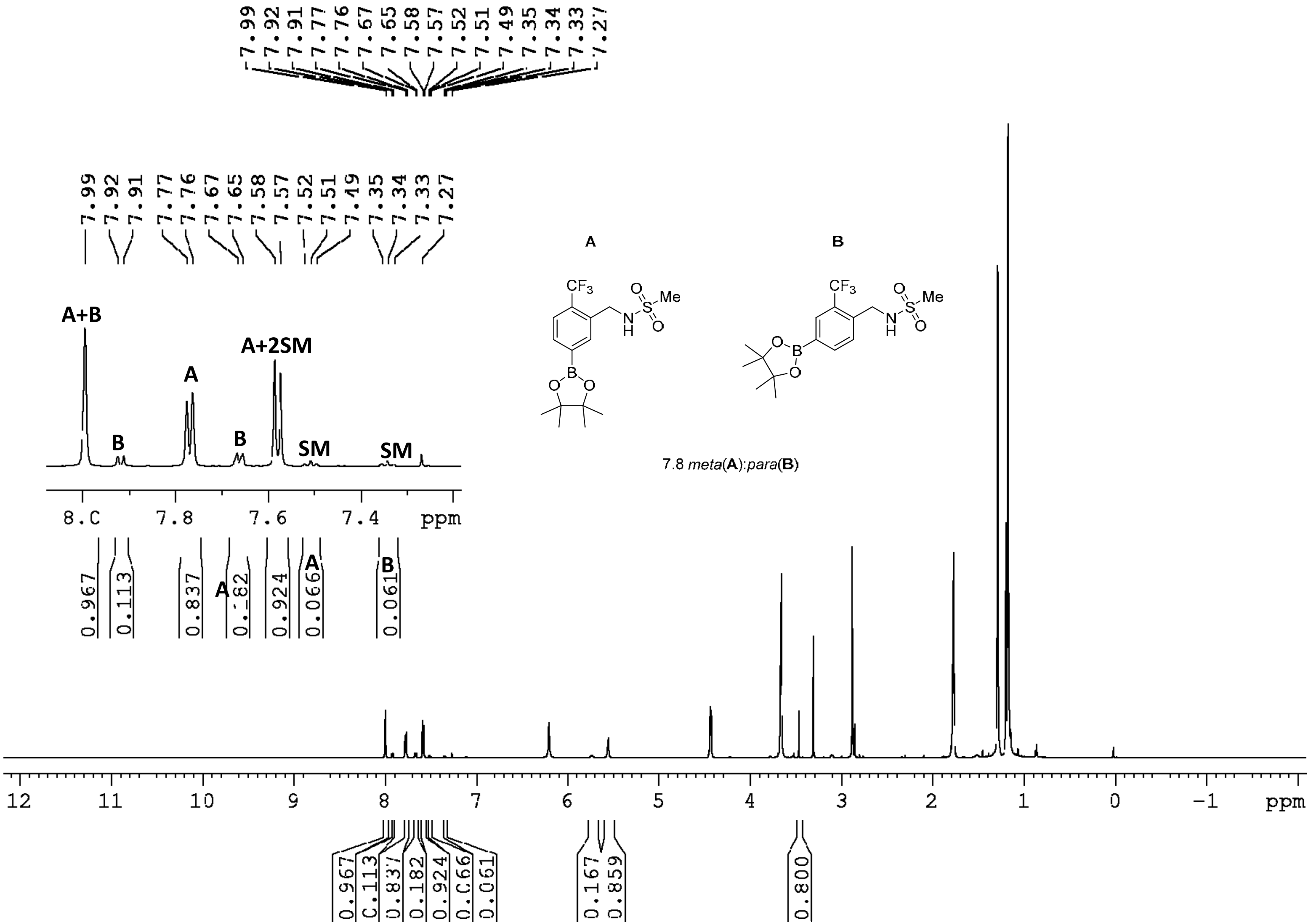

^{1}H NMR (500 MHz, $CDCl_3$) *N*-(5-(4,4,5,5-tetramethyl-1,3,2-dioxaborolan-2-yl)-2-(trifluoromethyl)benzyl)methanesulfonamide (2m) – Purified after reaction with 1a

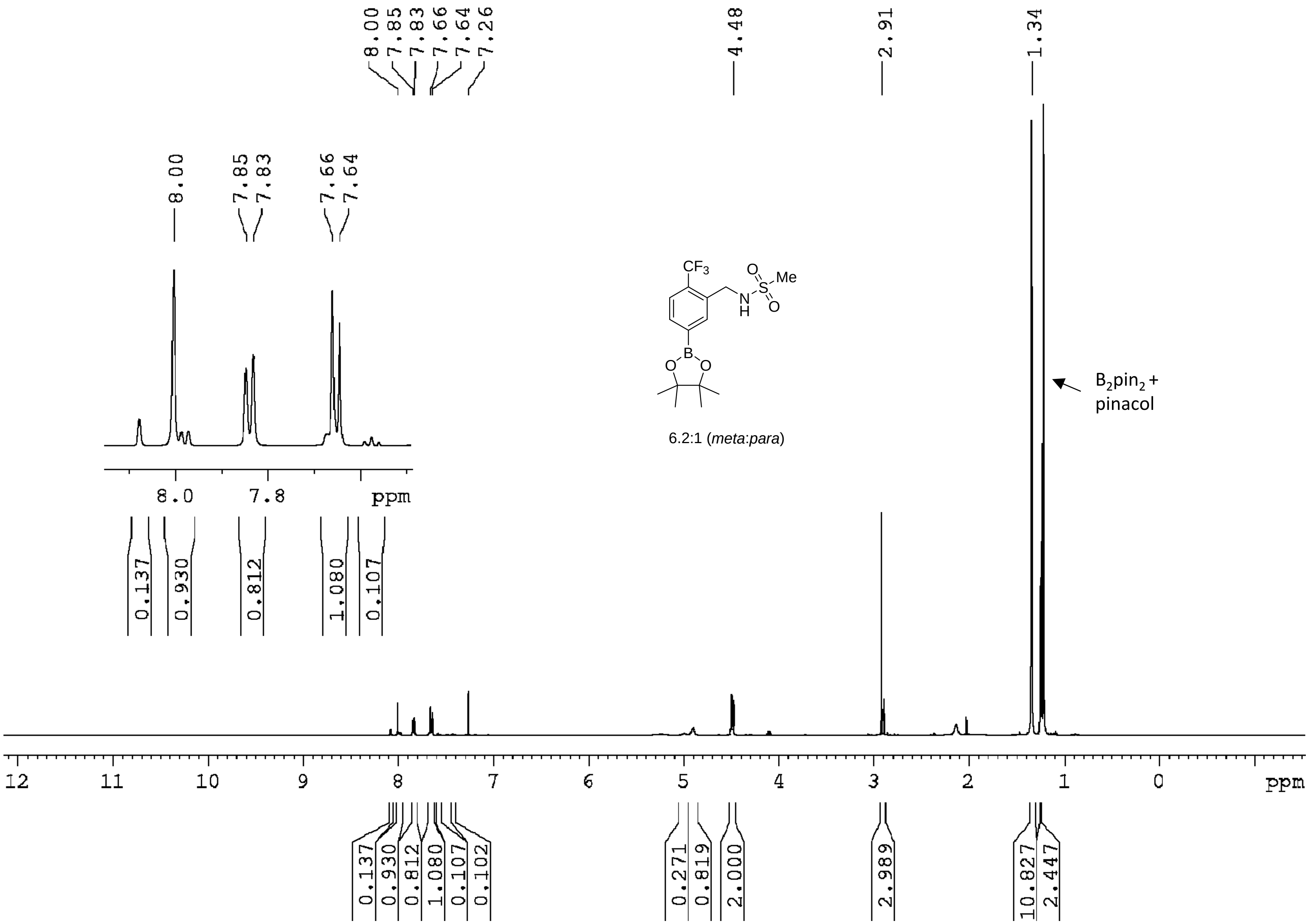

^{13}C NMR (126 MHz, $CDCl_3$) *N*-(5-(4,4,5,5-tetramethyl-1,3,2-dioxaborolan-2-yl)-2-(trifluoromethyl)benzyl)methanesulfonamide (2m) – Purified after reaction with 1a

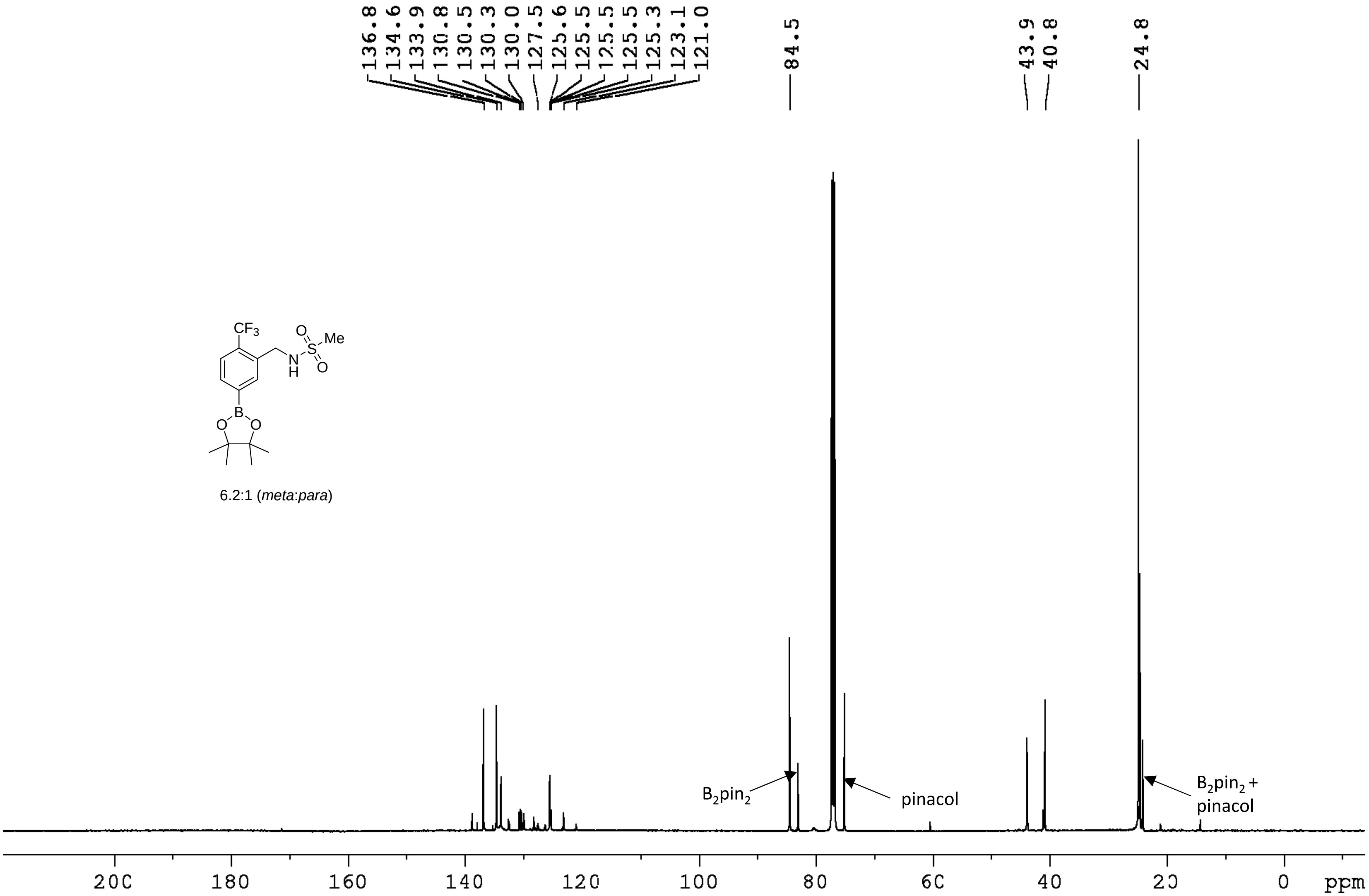

^{1}H NMR (500 MHz, $CDCl_3$) *N*-(5-(4,4,5,5-tetramethyl-1,3,2-dioxaborolan-2-yl)-2-(trifluoromethyl)benzyl)methanesulfonamide (3m) – Purified after reaction with dtbpy

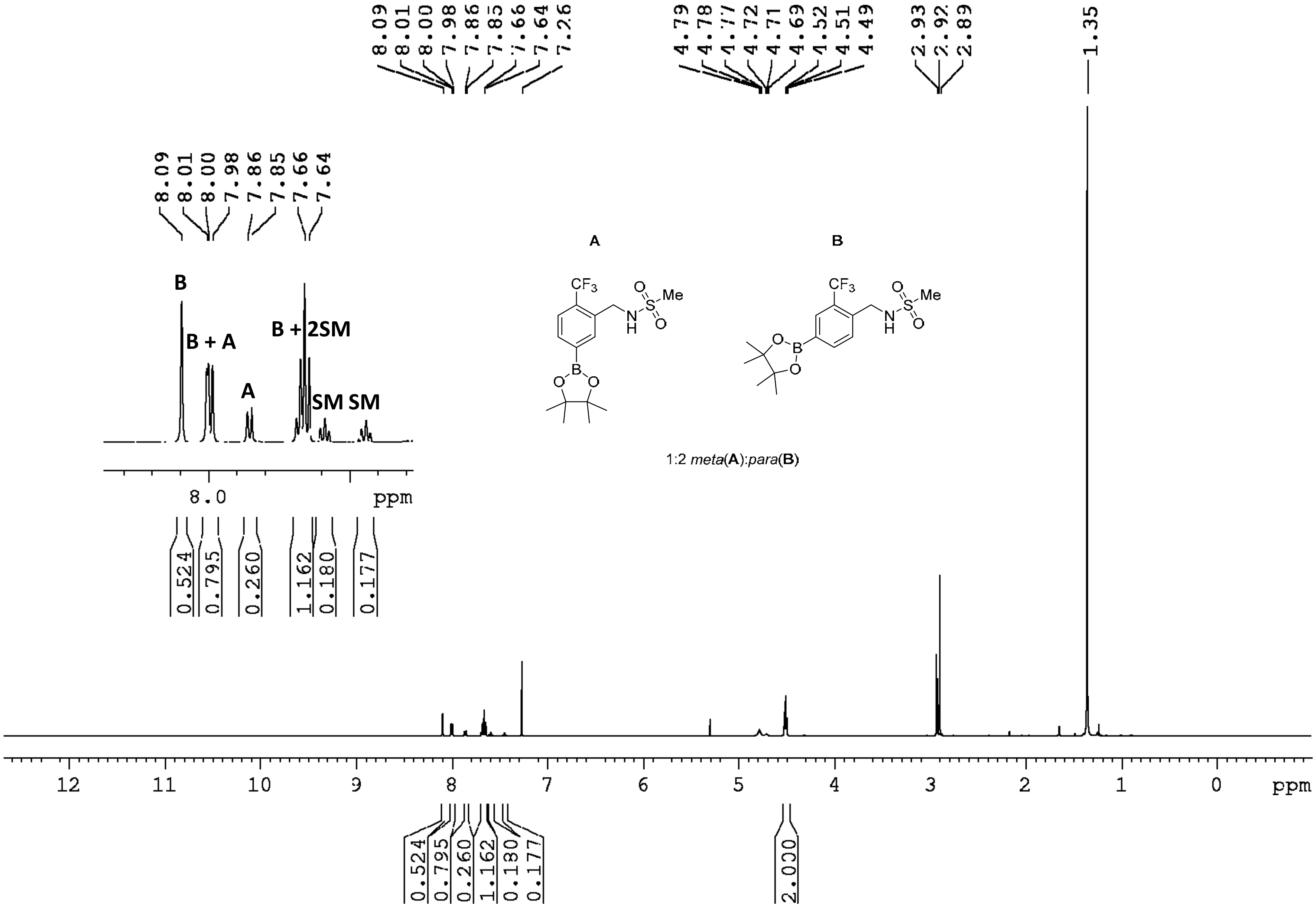

^{13}C NMR (126 MHz, $CDCl_3$) *N*-(5-(4,4,5,5-tetramethyl-1,3,2-dioxaborolan-2-yl)-2-(trifluoromethyl)benzyl)methanesulfonamide (3m) – Purified after reaction with dtbpy

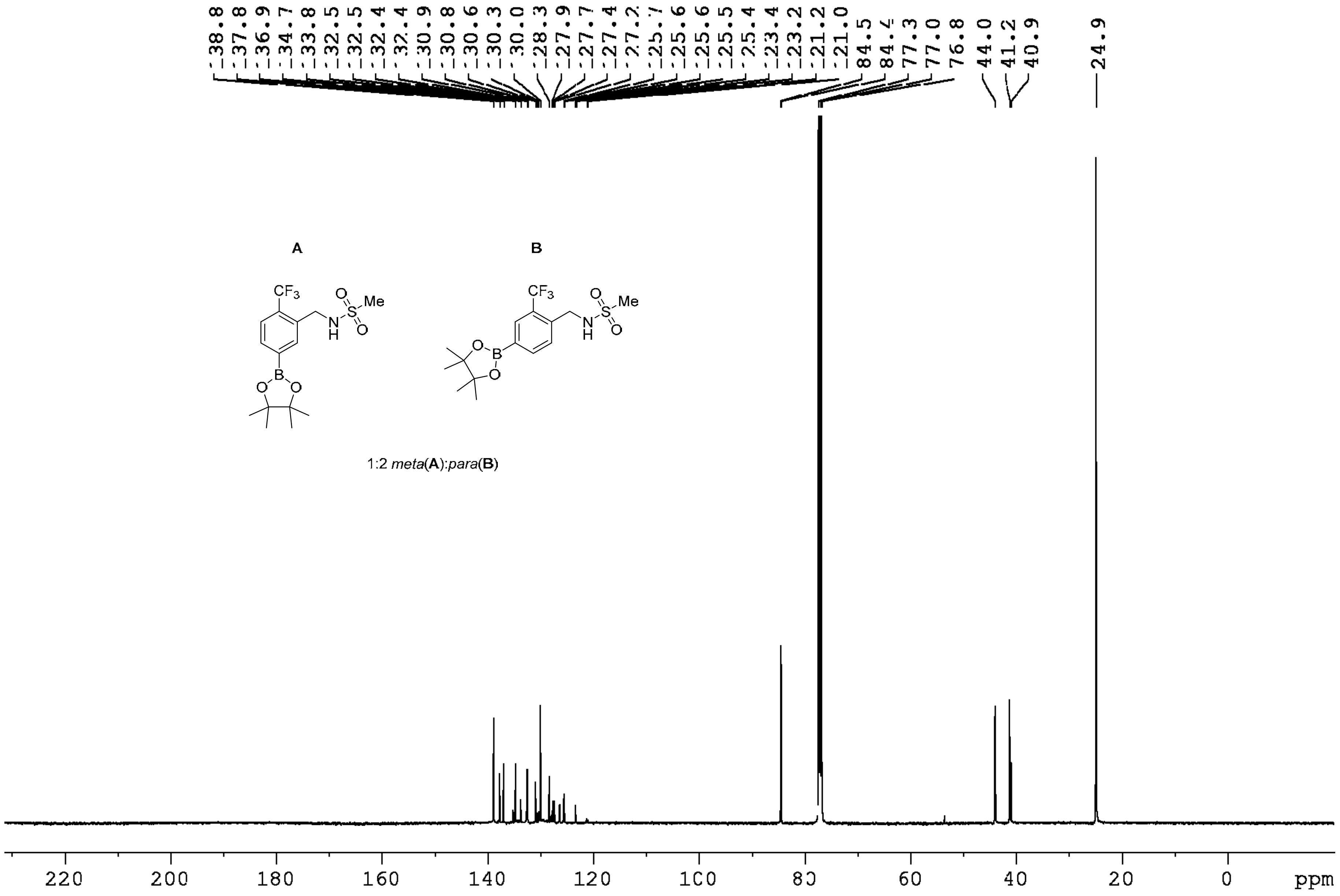

^{1}H NMR (500 MHz, $CDCl_3$) 4-Methyl-*N*-(5-(4,4,5,5-tetramethyl-1,3,2-dioxaborolan-2-yl)-2-(trifluoromethyl)benzyl)benzenesulfonamide (3n) – Crude after reaction with 1a

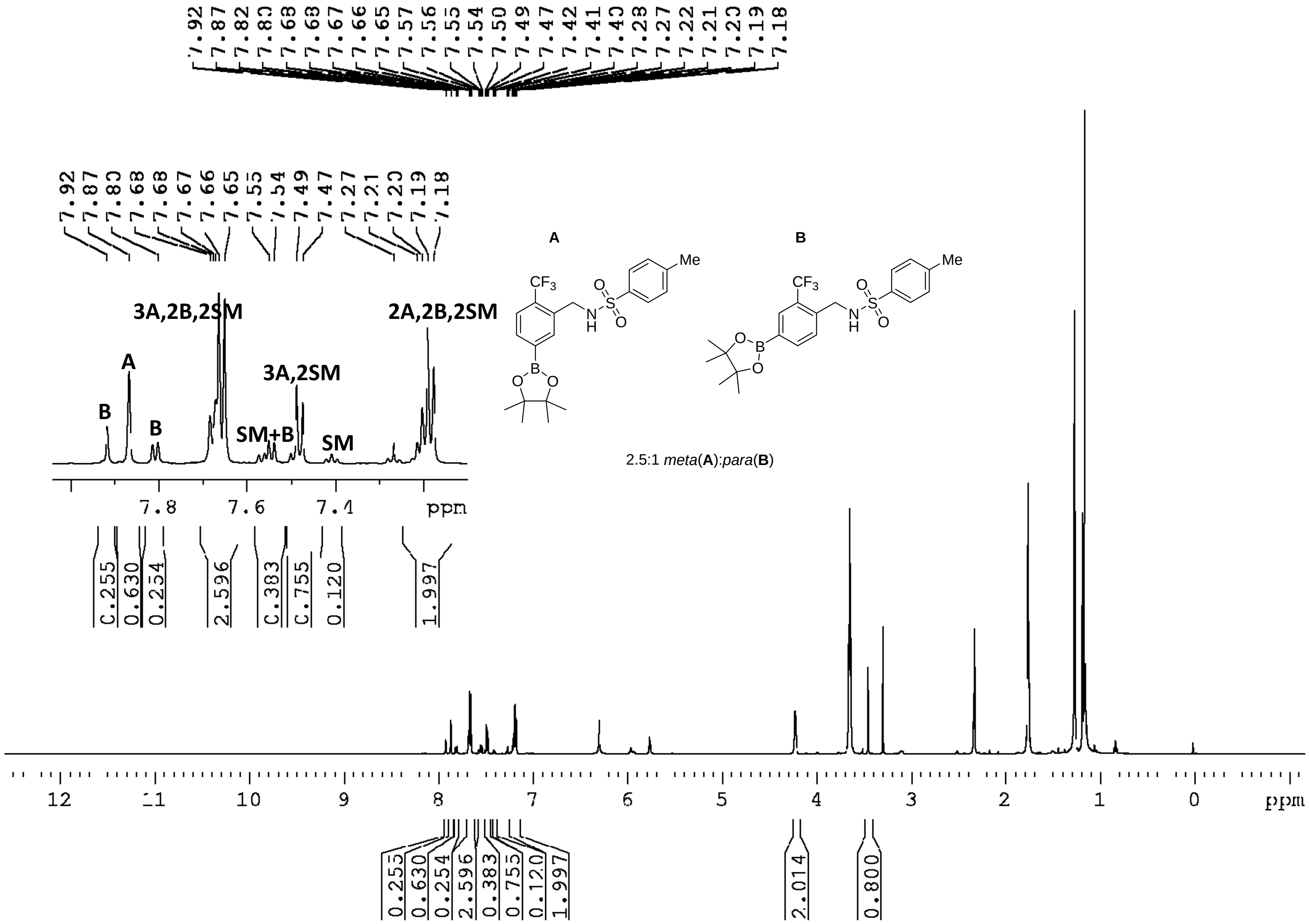

^{1}H NMR (500 MHz, $CDCl_3$) 4-Methyl-*N*-(5-(4,4,5,5-tetramethyl-1,3,2-dioxaborolan-2-yl)-2-(trifluoromethyl)benzyl)benzenesulfonamide (3n) – Purified after reaction with 1a

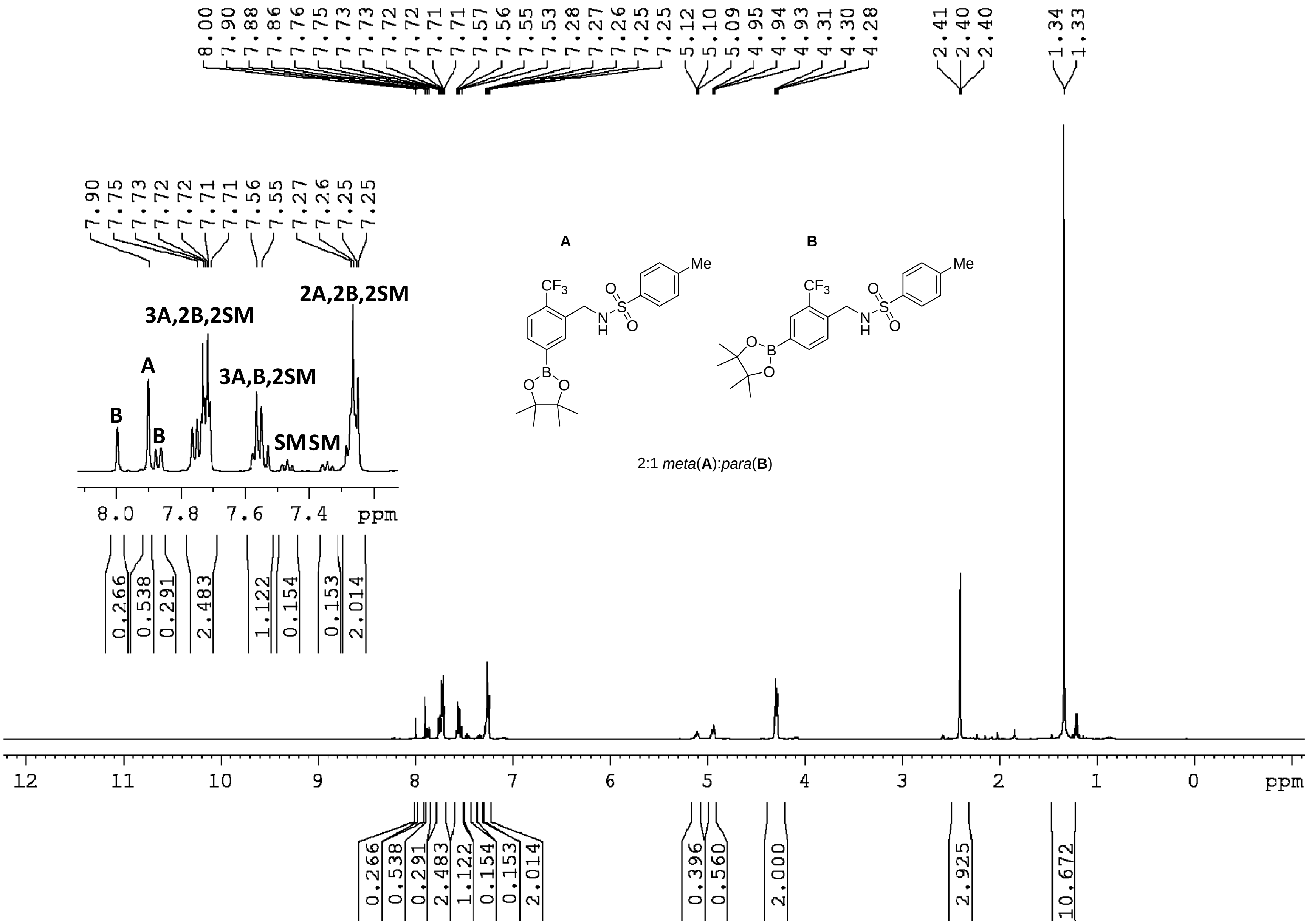

^{13}C NMR (126 MHz, $CDCl_3$) 4-Methyl-*N*-(5-(4,4,5,5-tetramethyl-1,3,2-dioxaborolan-2-yl)-2-(trifluoromethyl)benzyl)benzenesulfonamide (3n) – Purified after reaction with 1a

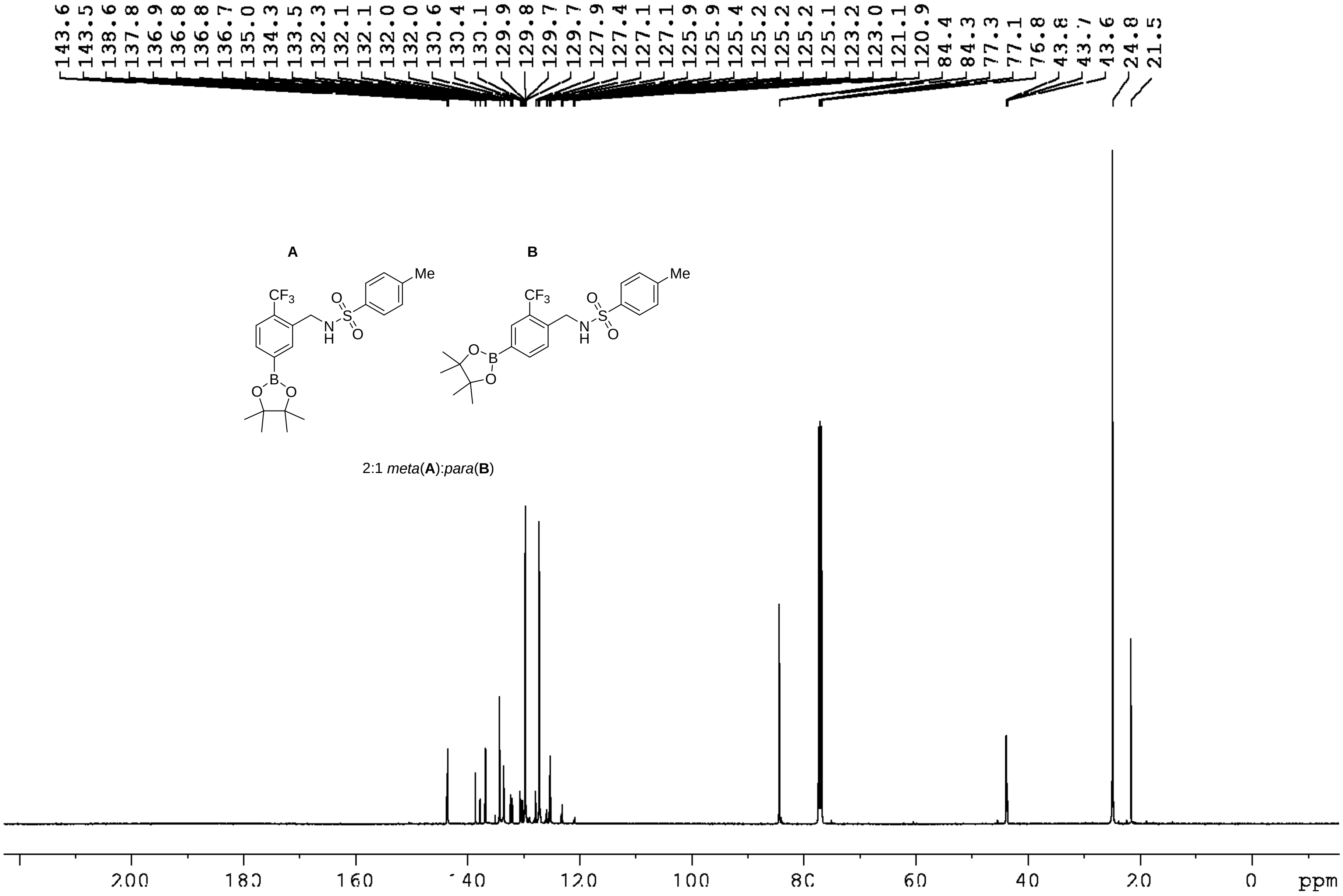

^{1}H NMR (600 MHz, $CDCl_3$) *N*-(5-(4,4,5,5-tetramethyl-1,3,2-dioxaborolan-2-yl)-2-(trifluoromethyl)benzyl)acetamide (3o) – Crude after reaction with 1a

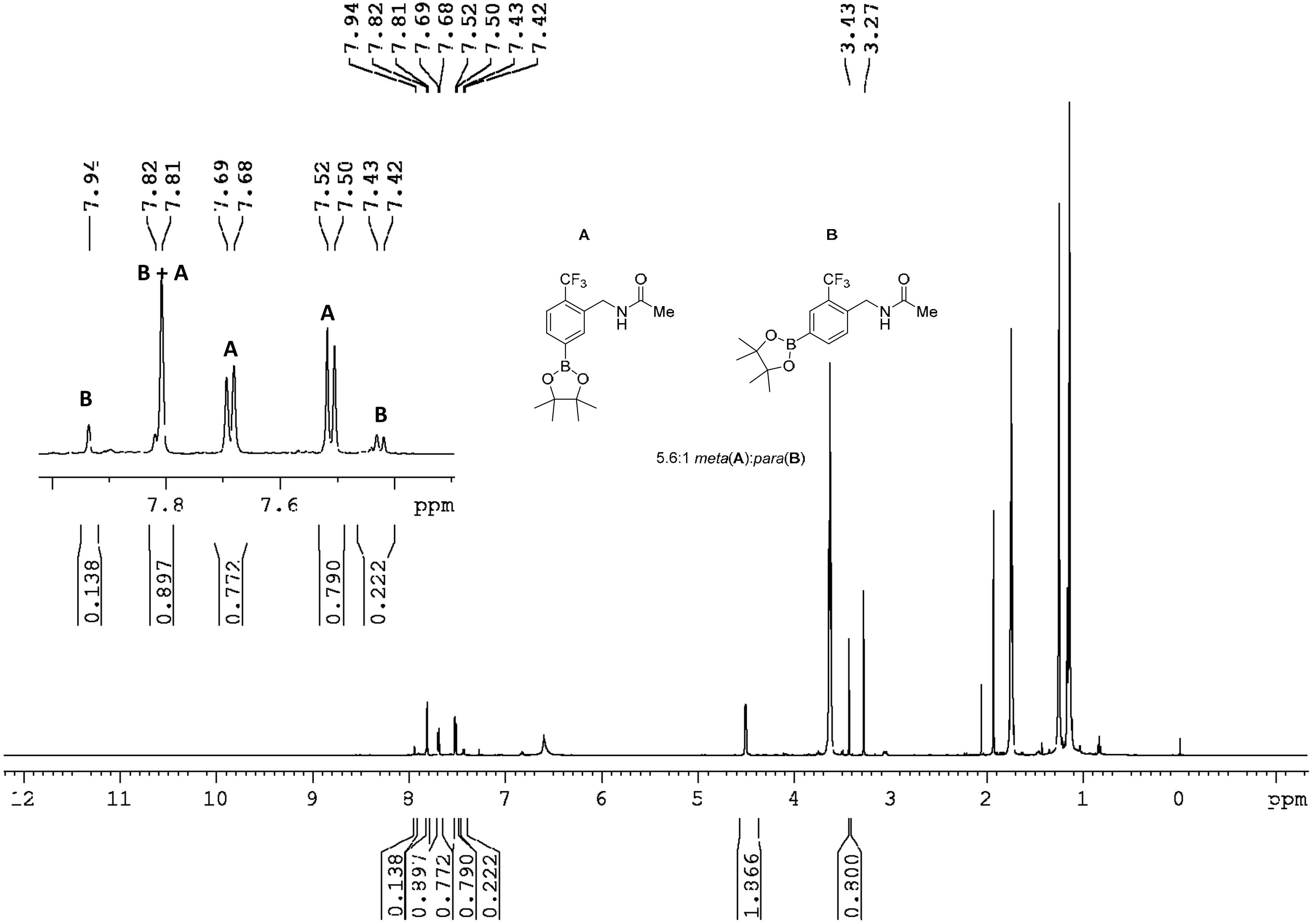

^{1}H NMR (600 MHz, $CDCl_3$) *N*-(5-(4,4,5,5-tetramethyl-1,3,2-dioxaborolan-2-yl)-2-(trifluoromethyl)benzyl)acetamide (3o) – Purified after reaction with 1a

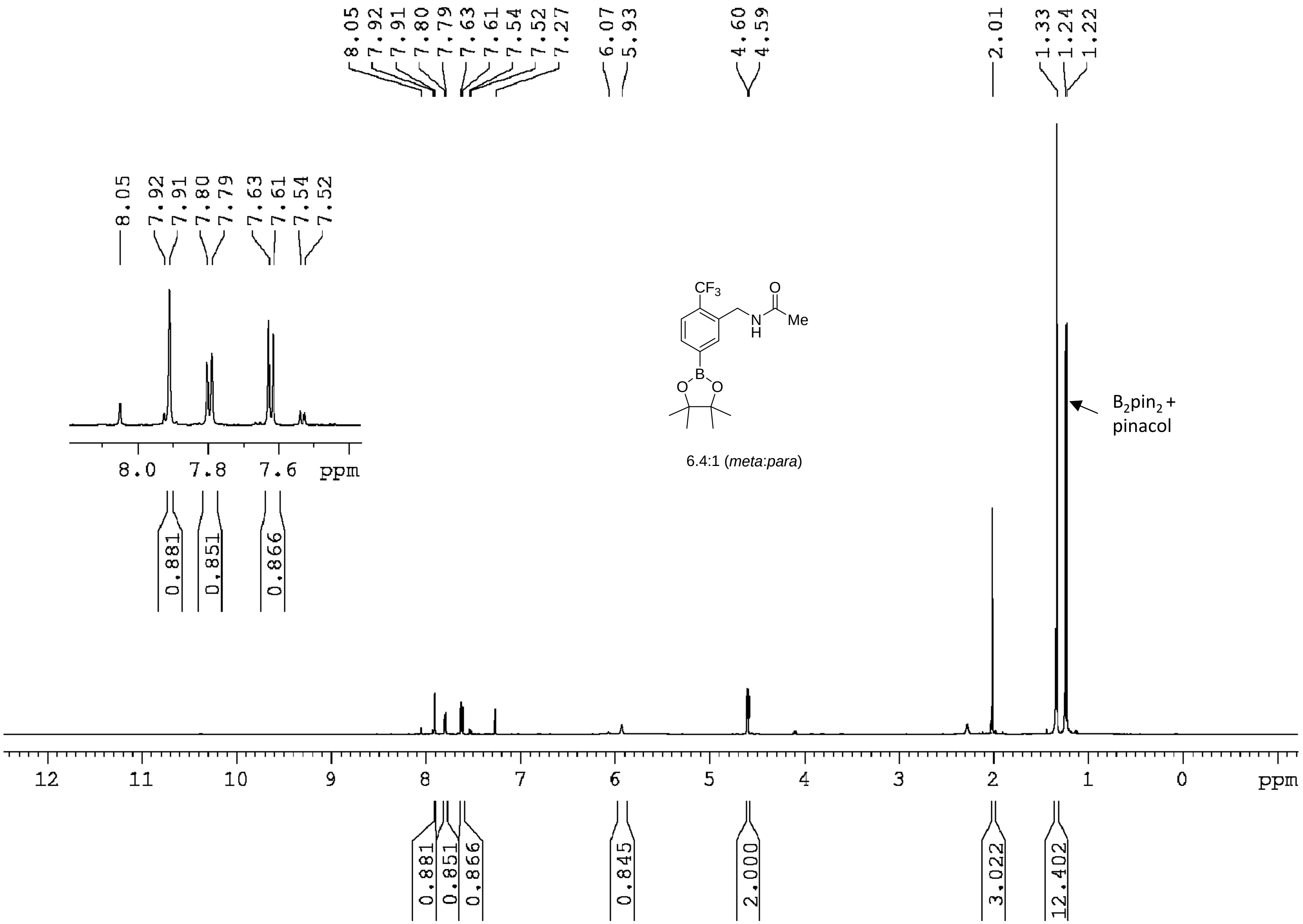

^{13}C NMR (151 MHz, $CDCl_3$) *N*-(5-(4,4,5,5-tetramethyl-1,3,2-dioxaborolan-2-yl)-2-(trifluoromethyl)benzyl)acetamide (3o) – Purified after reaction with 1a

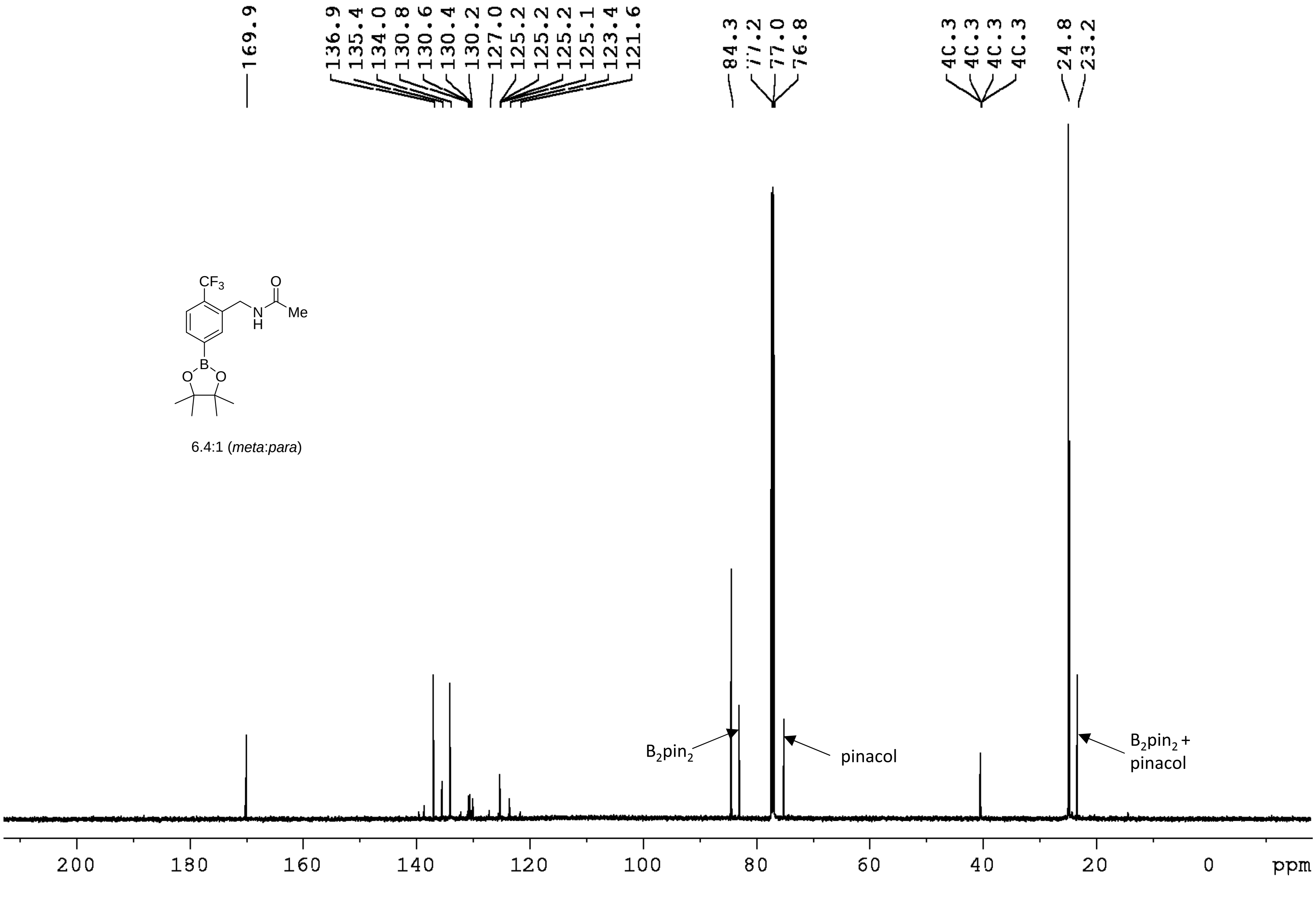

^{1}H NMR (600 MHz, $CDCl_3$) *N*-(5-(4,4,5,5-tetramethyl-1,3,2-dioxaborolan-2-yl)-2-(trifluoromethyl)benzyl)acetamide (3o) – Purified after reaction with dtbpy

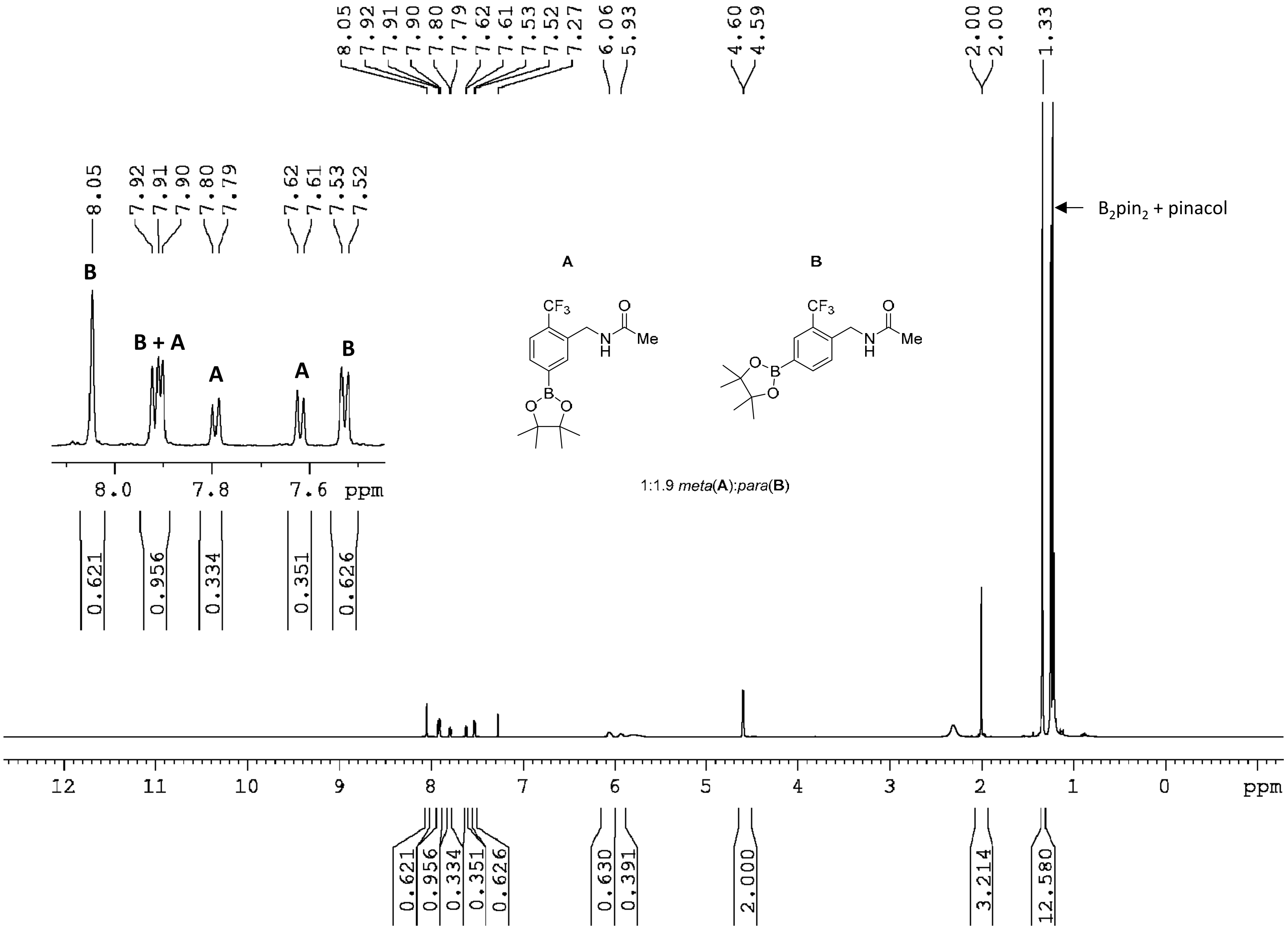

^{13}C NMR (151 MHz, $CDCl_3$) *N*-(5-(4,4,5,5-tetramethyl-1,3,2-dioxaborolan-2-yl)-2-(trifluoromethyl)benzyl)acetamide (3o) – Purified after reaction with dtbpy

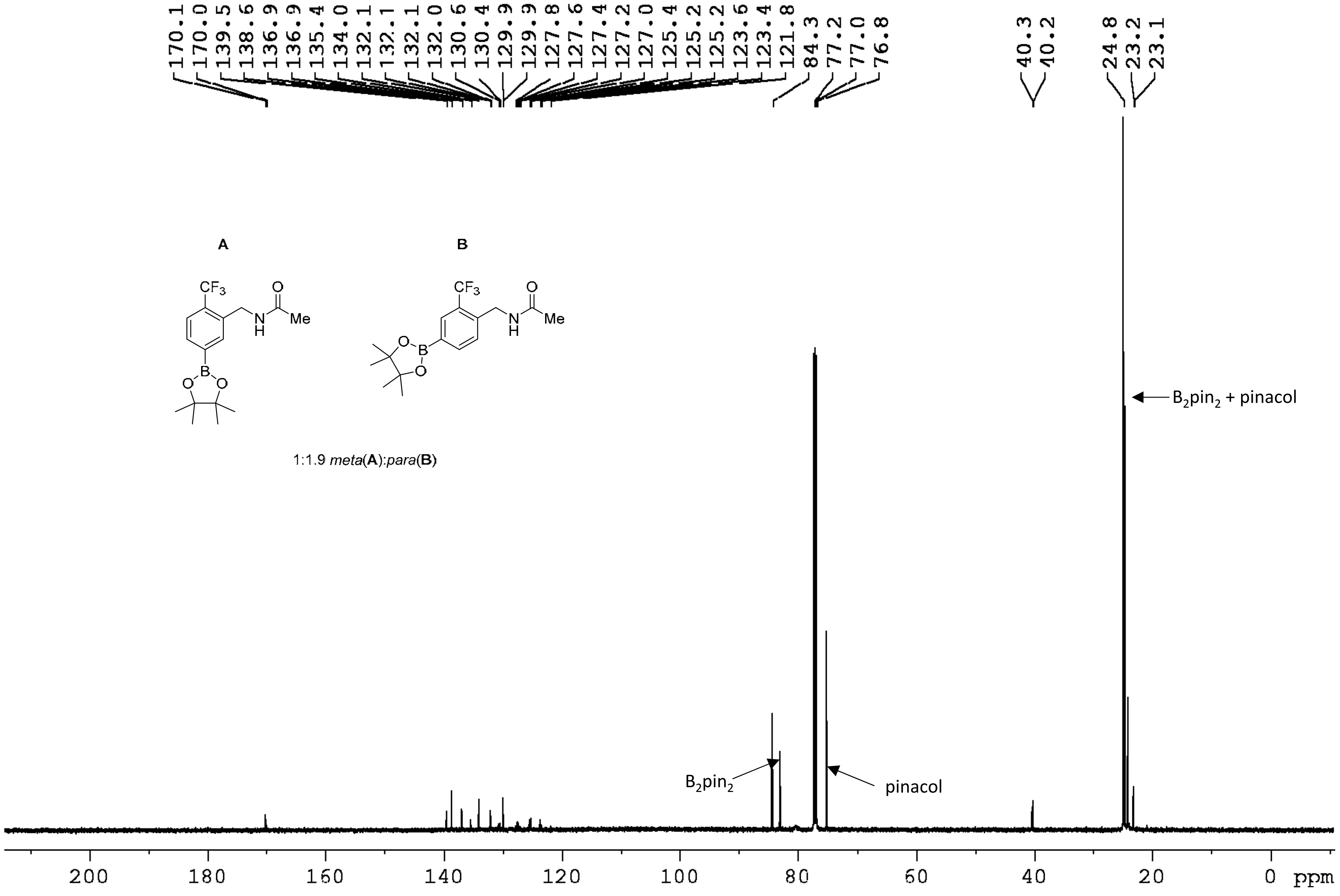

^{1}H NMR (600 MHz, $CDCl_3$) *tert*-Butyl (5-(4,4,5,5-tetramethyl-1,3,2-dioxaborolan-2-yl)-2-(trifluoromethyl)benzyl)carbamate (3p) – Crude after reaction with 1a

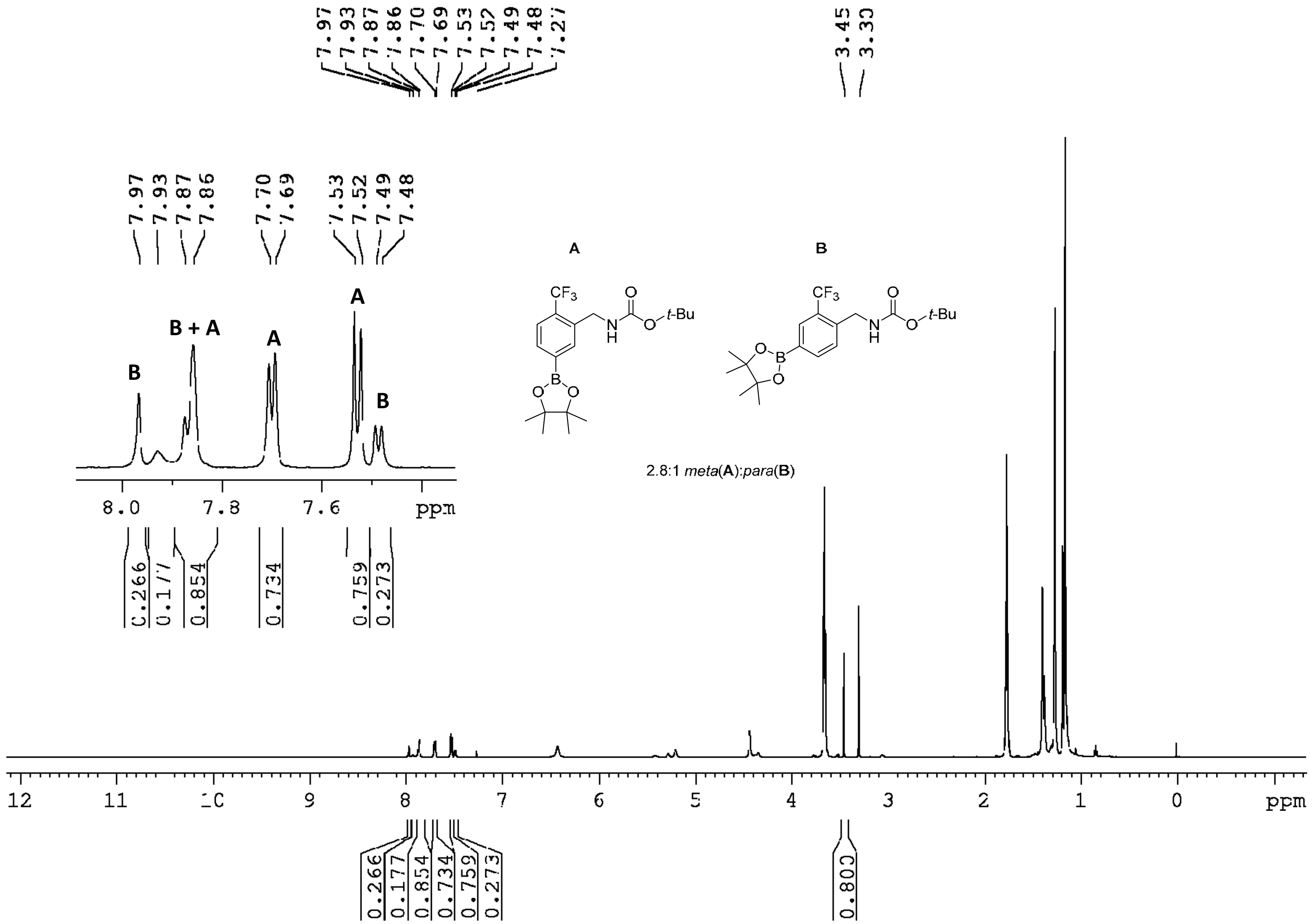

^{1}H NMR (500 MHz, $CDCl_3$) *tert*-Butyl (5-(4,4,5,5-tetramethyl-1,3,2-dioxaborolan-2-yl)-2-(trifluoromethyl)benzyl)carbamate (3p) – Purified after reaction 1a

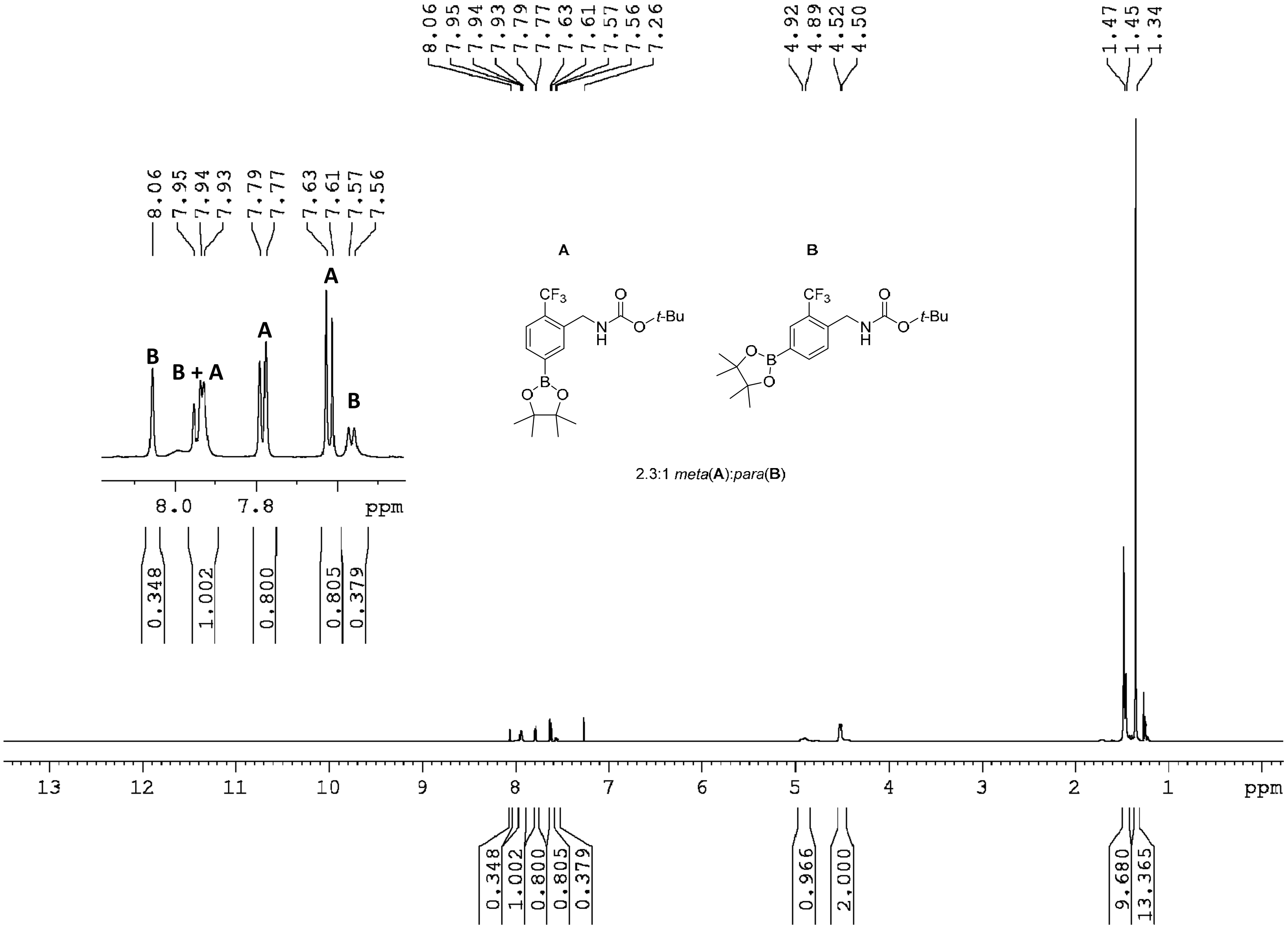

^{13}C NMR (126 MHz, $CDCl_3$) *tert*-Butyl (5-(4,4,5,5-tetramethyl-1,3,2-dioxaborolan-2-yl)-2-(trifluoromethyl)benzyl)carbamate (3p) – Purified after reaction with 1a

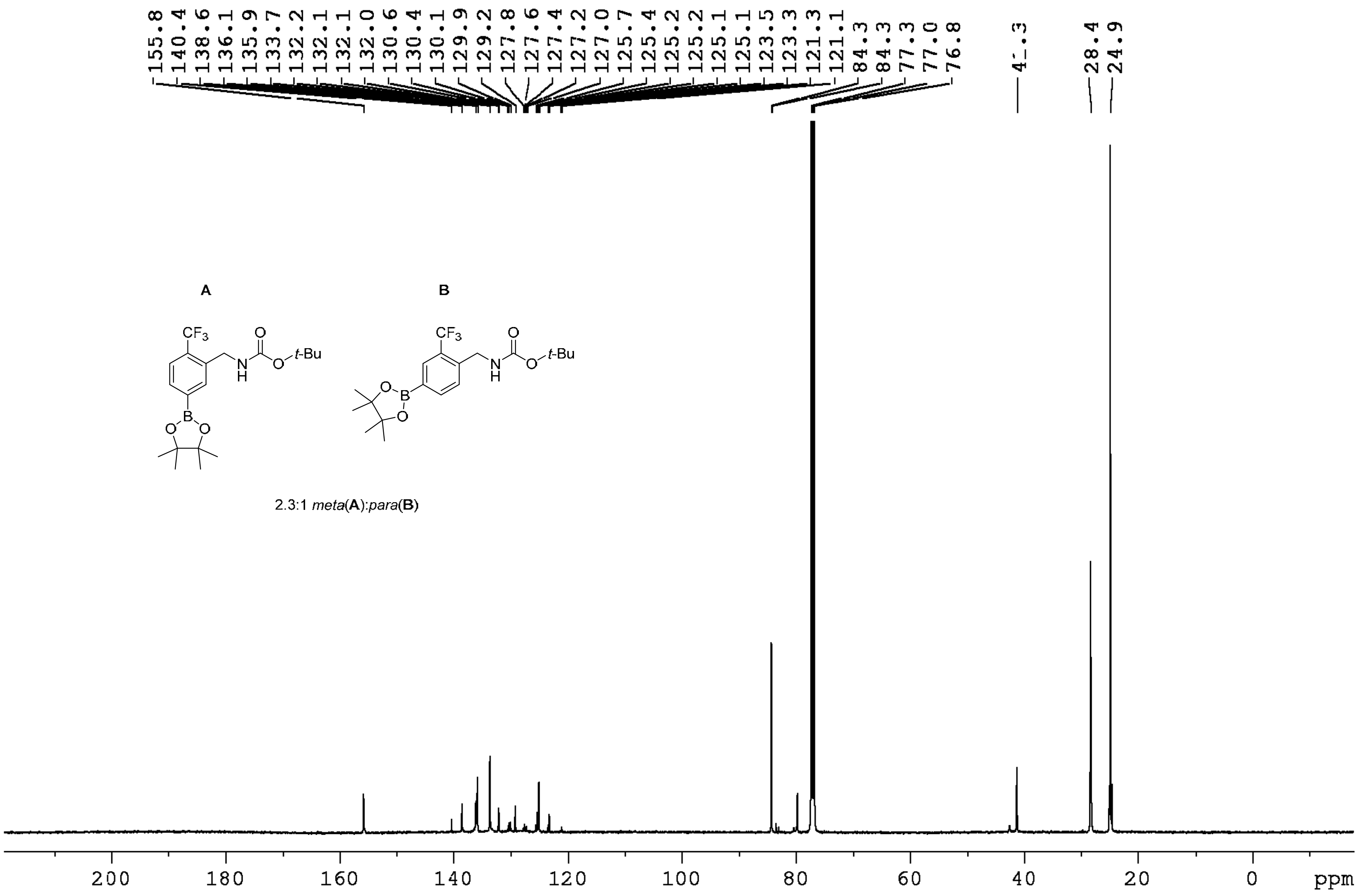

^{1}H NMR (600 MHz, $CDCl_3$) *N*-(5-(4,4,5,5-tetramethyl-1,3,2-dioxaborolan-2-yl)-2-(trifluoromethyl)benzyl)pivalamide (3q) – Crude after reaction with 1a

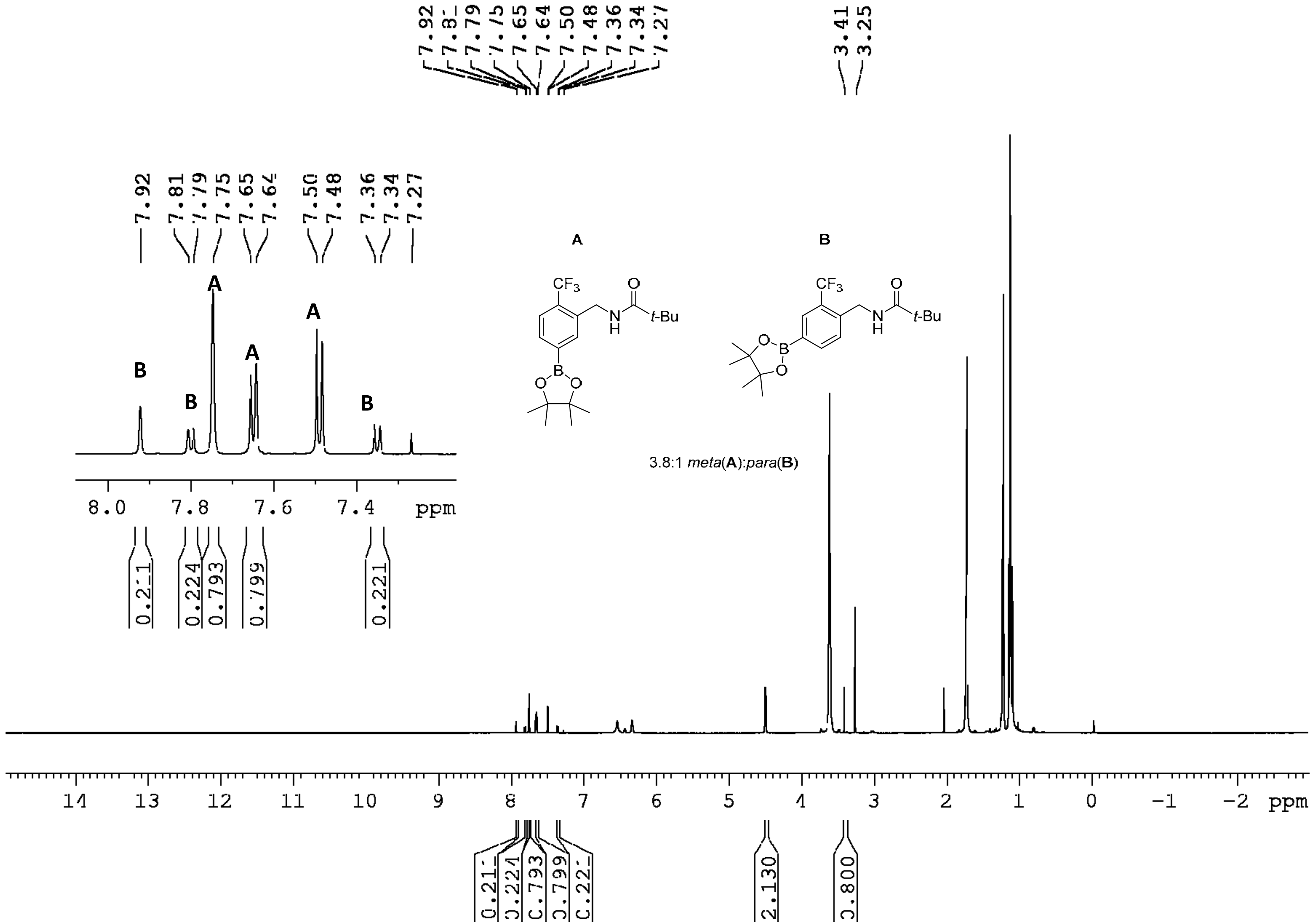

^{1}H NMR (600 MHz, $CDCl_3$) *N*-(5-(4,4,5,5-tetramethyl-1,3,2-dioxaborolan-2-yl)-2-(trifluoromethyl)benzyl)pivalamide (3q) – Purified after reaction with 1a

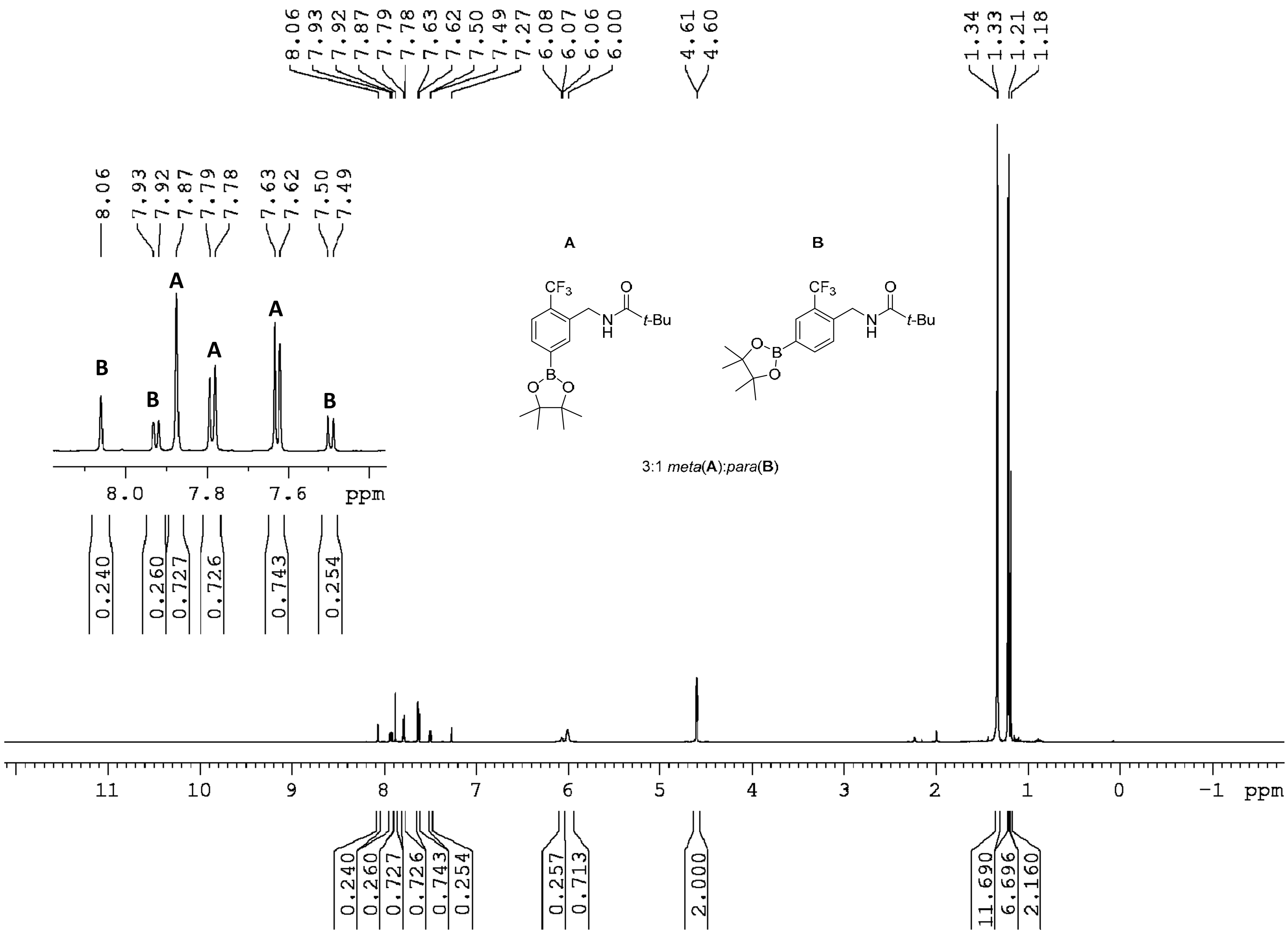

^{13}C NMR (151 MHz, $CDCl_3$) *N*-(5-(4,4,5,5-tetramethyl-1,3,2-dioxaborolan-2-yl)-2-(trifluoromethyl)benzyl)pivalamide (3q) – Purified after reaction with 1a

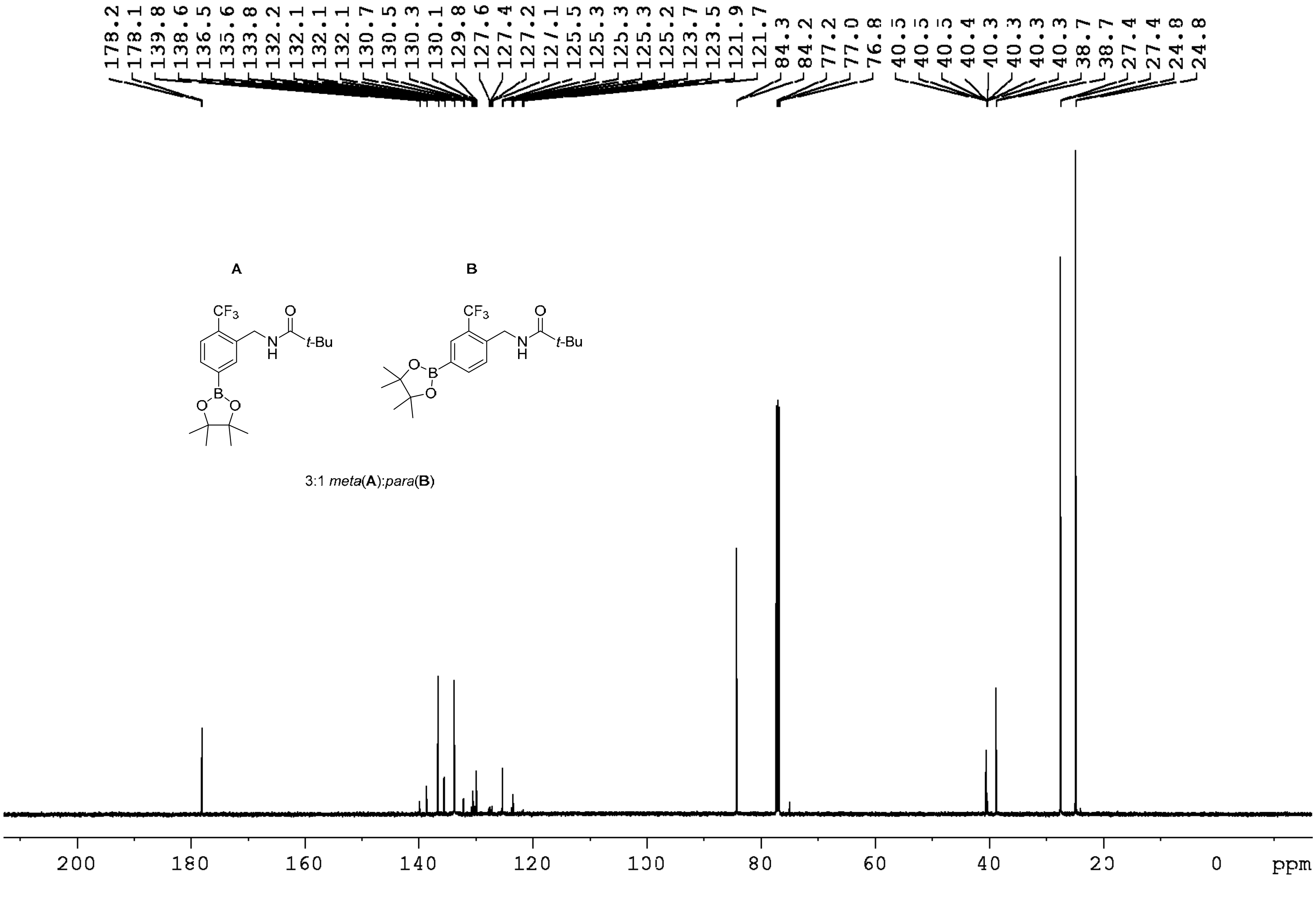

^{1}H NMR (600 MHz, $CDCl_3$) of 2,2,2-trifluoro-N-(5-(4,4,5,5-tetramethyl-1,3,2-dioxaborolan-2-yl)-2 (trifluoromethyl)phenethyl)acetamide (5a) – Dtbpy – Purified

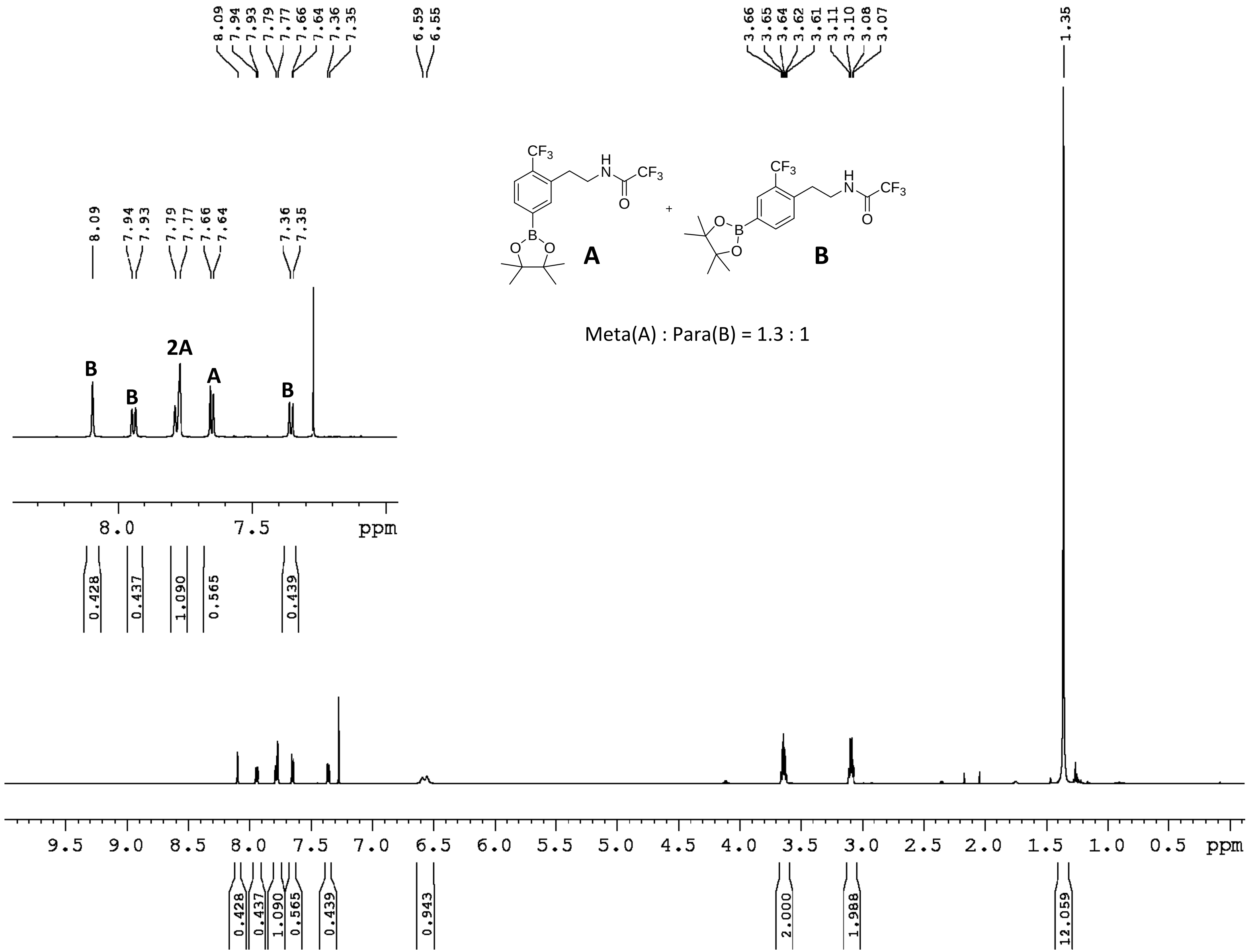

^{13}C NMR (151 MHz, $CDCl_3$) of 2,2,2-trifluoro-N-(5-(4,4,5,5-tetramethyl-1,3,2-dioxaborolan-2-yl)-2 (trifluoromethyl)phenethyl)acetamide (5a) – Dtbpy – Purified

A

B

Meta(A) : Para(B) = 1.3 : 1

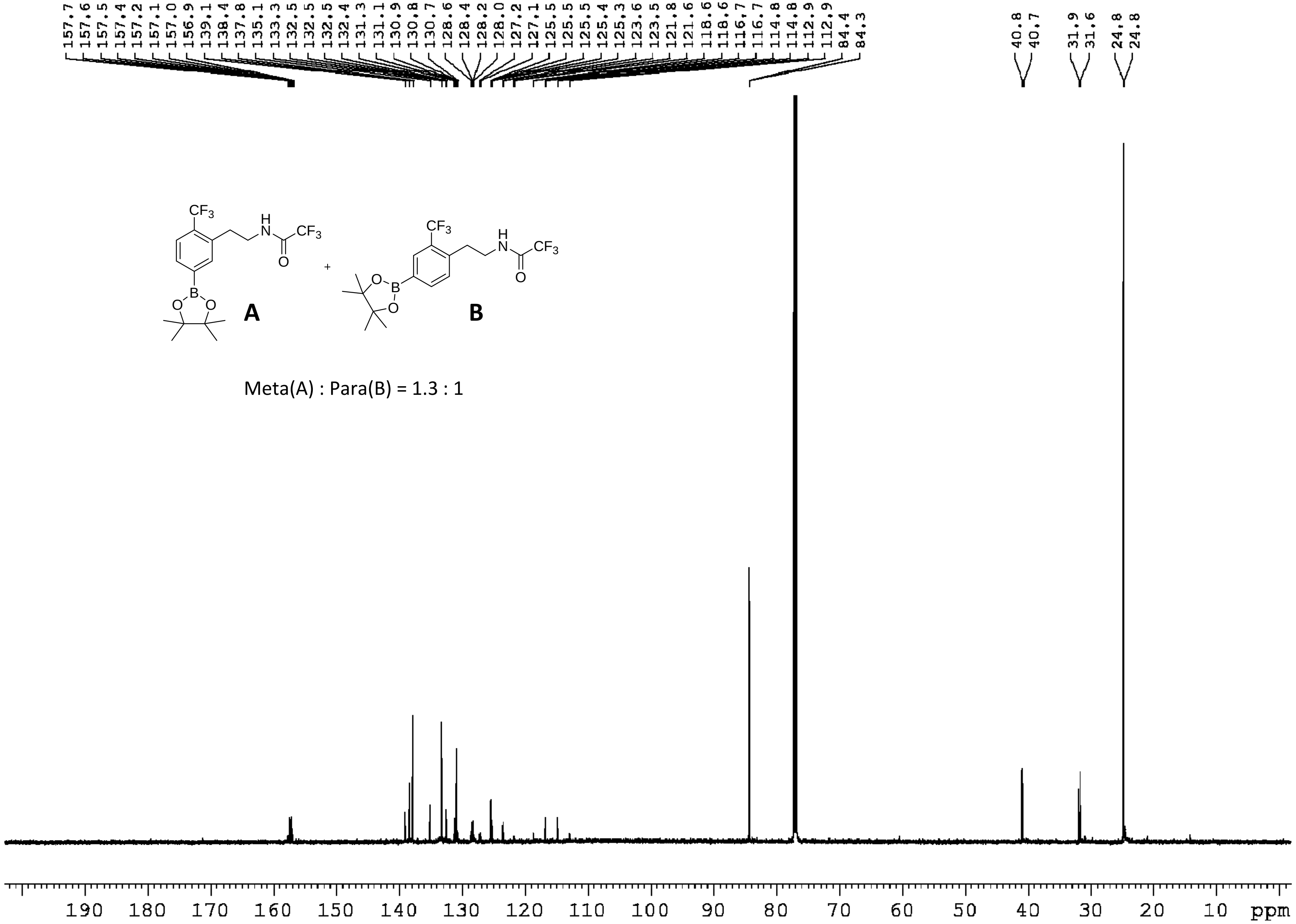

^{1}H NMR (600 MHz, $CDCl_3$) of 2,2,2-trifluoro-N-(5-(4,4,5,5-tetramethyl-1,3,2-dioxaborolan-2-yl)-2 (trifluoromethyl)phenethyl)acetamide (5a) – Ligand 1a – Crude mixture

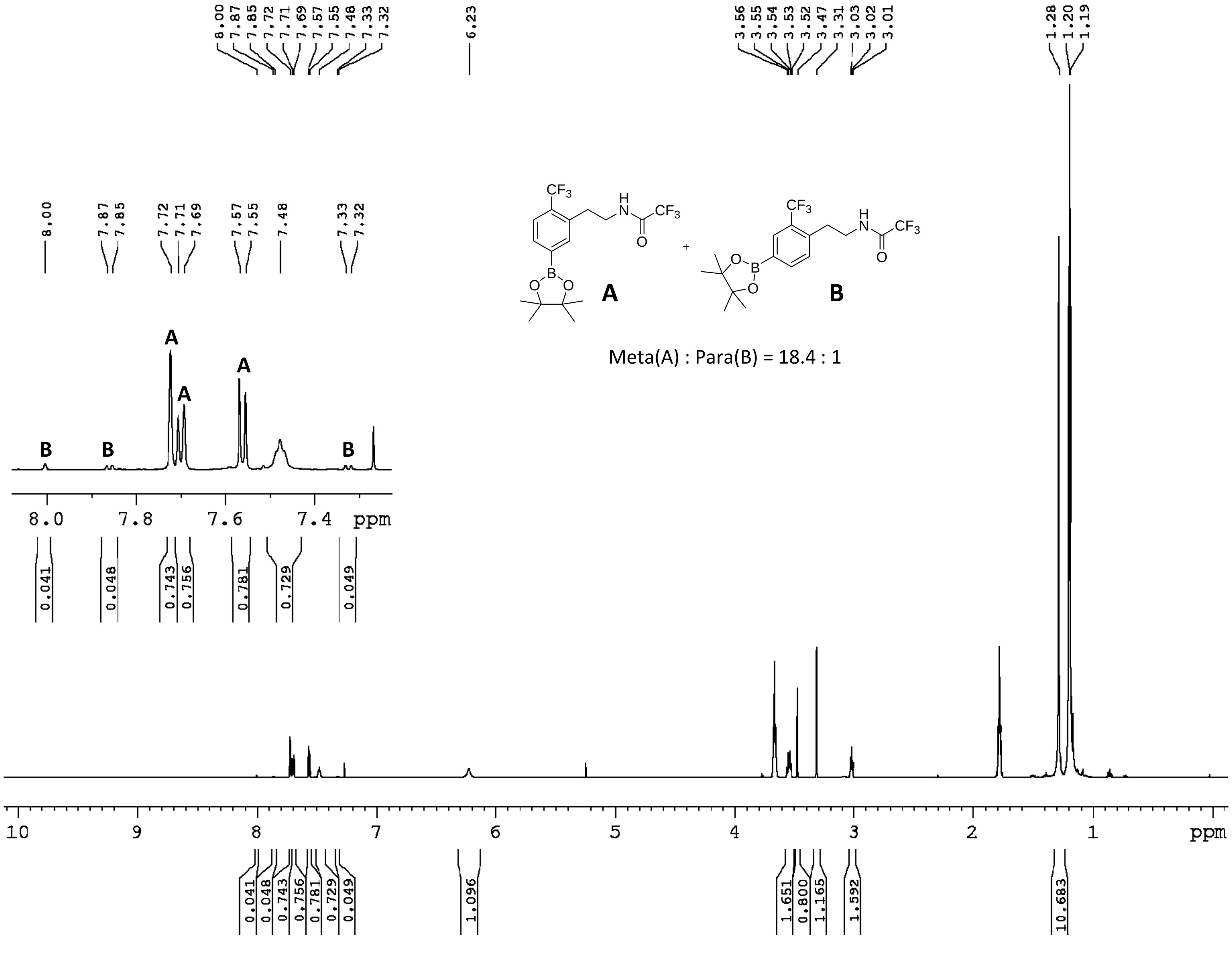

^{1}H NMR (600 MHz, $CDCl_3$) of methyl 2,2,2-trifluoro-N-(5-(4,4,5,5-tetramethyl-1,3,2-dioxaborolan-2-yl)-2 (trifluoromethyl)phenethyl)acetamide (5a) – Ligand 1a – Purified

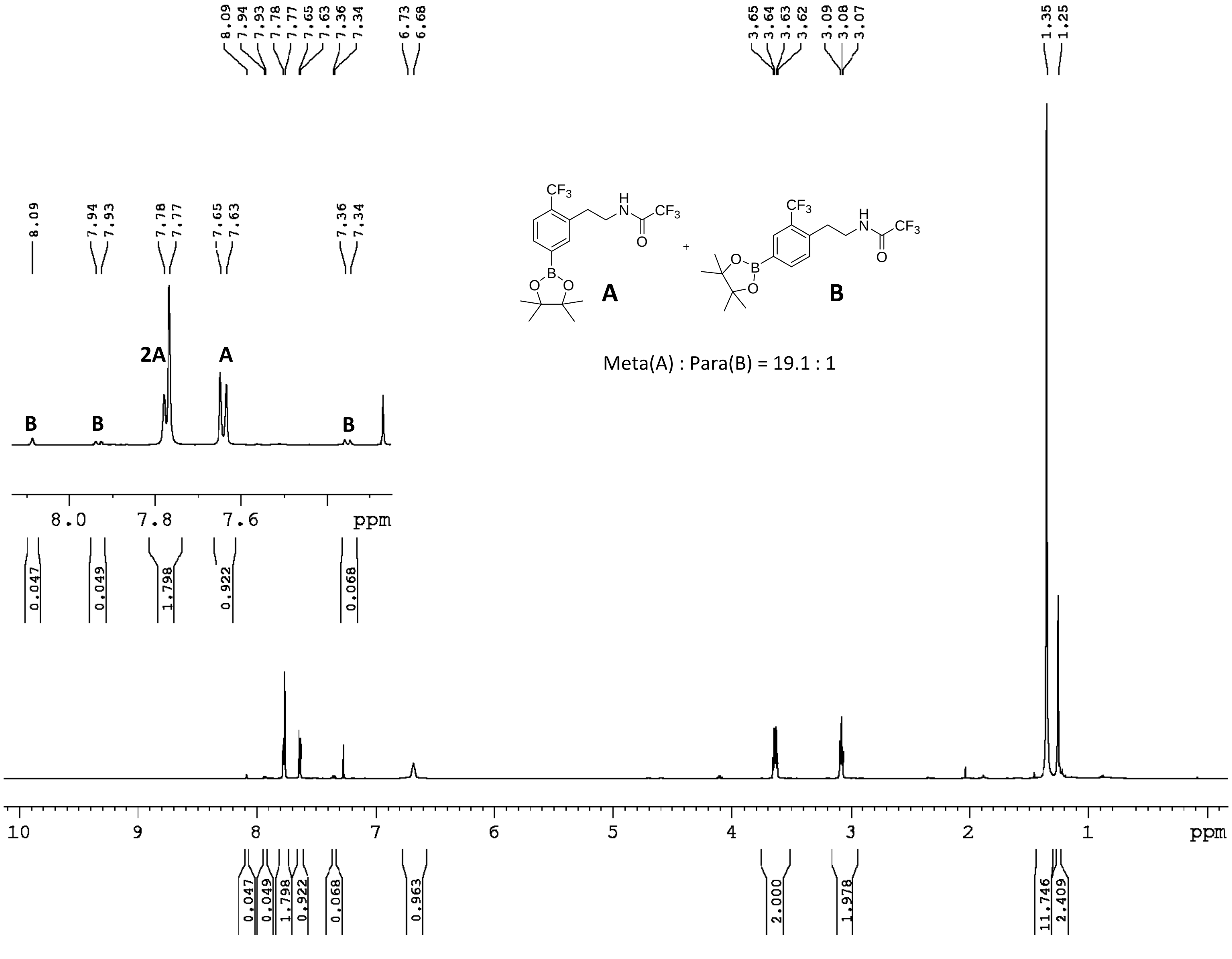

^{13}C NMR (151 MHz, $CDCl_3$) of methyl 2,2,2-trifluoro-N-(5-(4,4,5,5-tetramethyl-1,3,2-dioxaborolan-2-yl)-2 (trifluoromethyl)phenethyl)acetamide (5a) – Ligand 1a – Purified

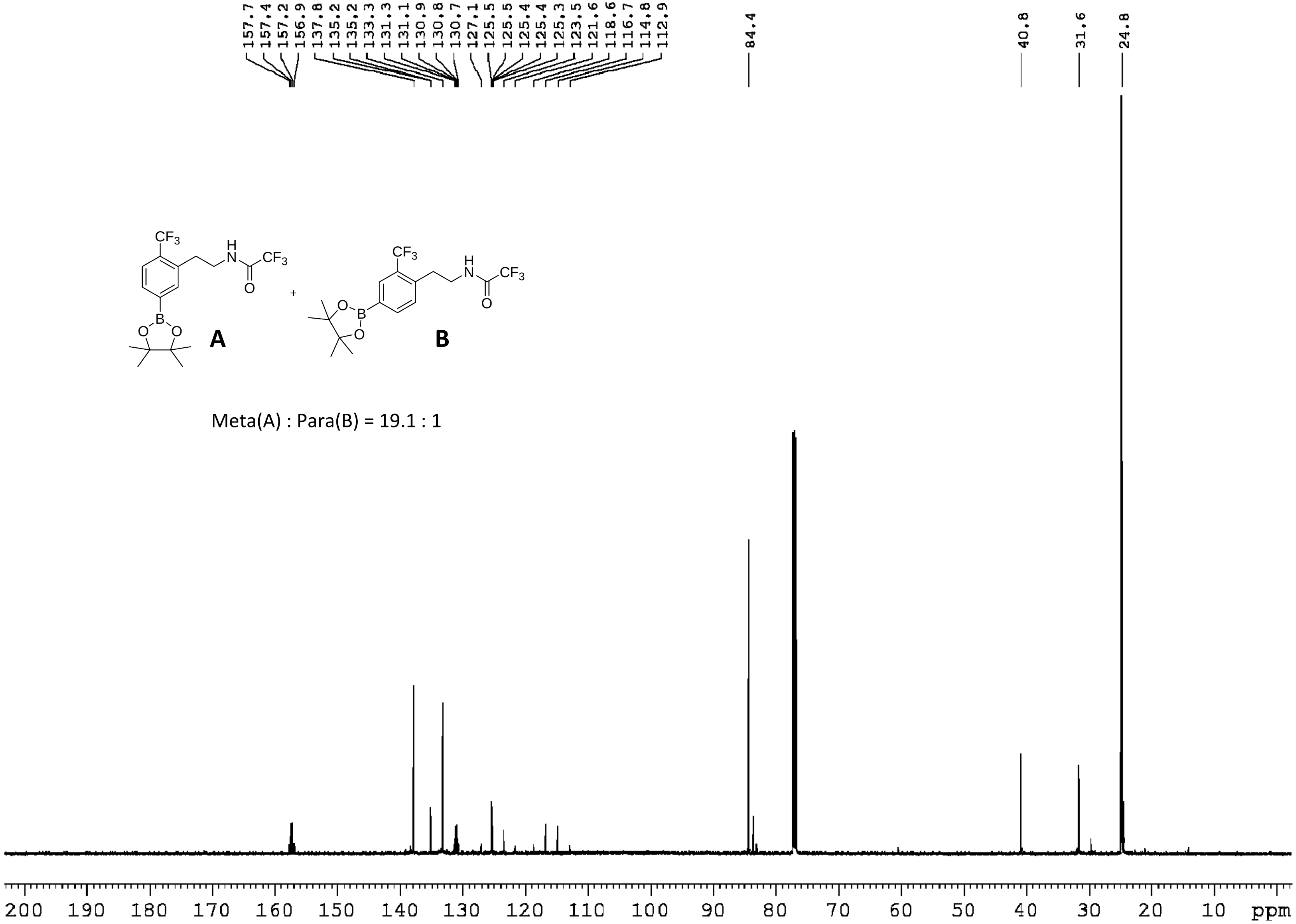

Meta(A) : Para(B) = 19.1 : 1

^{1}H NMR (600 MHz, $CDCl_3$) of N-(5-(4,4,5,5-tetramethyl-1,3,2-dioxaborolan-2-yl)-2-(trifluoromethyl)phenethyl)methanesulfonamide (5b) – Dtbpy – Purified

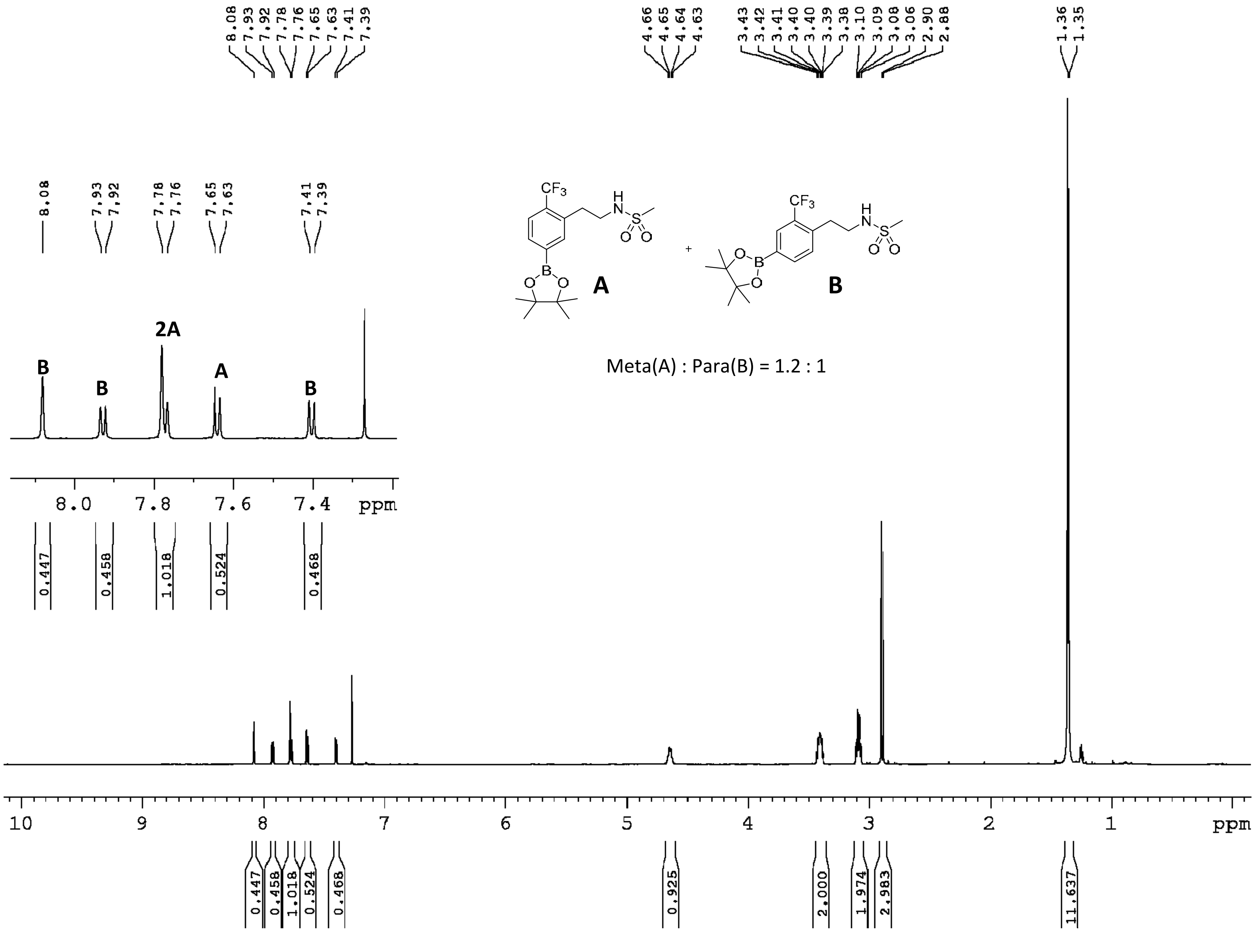

^{13}C NMR (151 MHz, $CDCl_3$) of N-(5-(4,4,5,5-tetramethyl-1,3,2-dioxaborolan-2-yl)-2-(trifluoromethyl)phenethyl)methanesulfonamide (5b) – Dtbpy – Purified

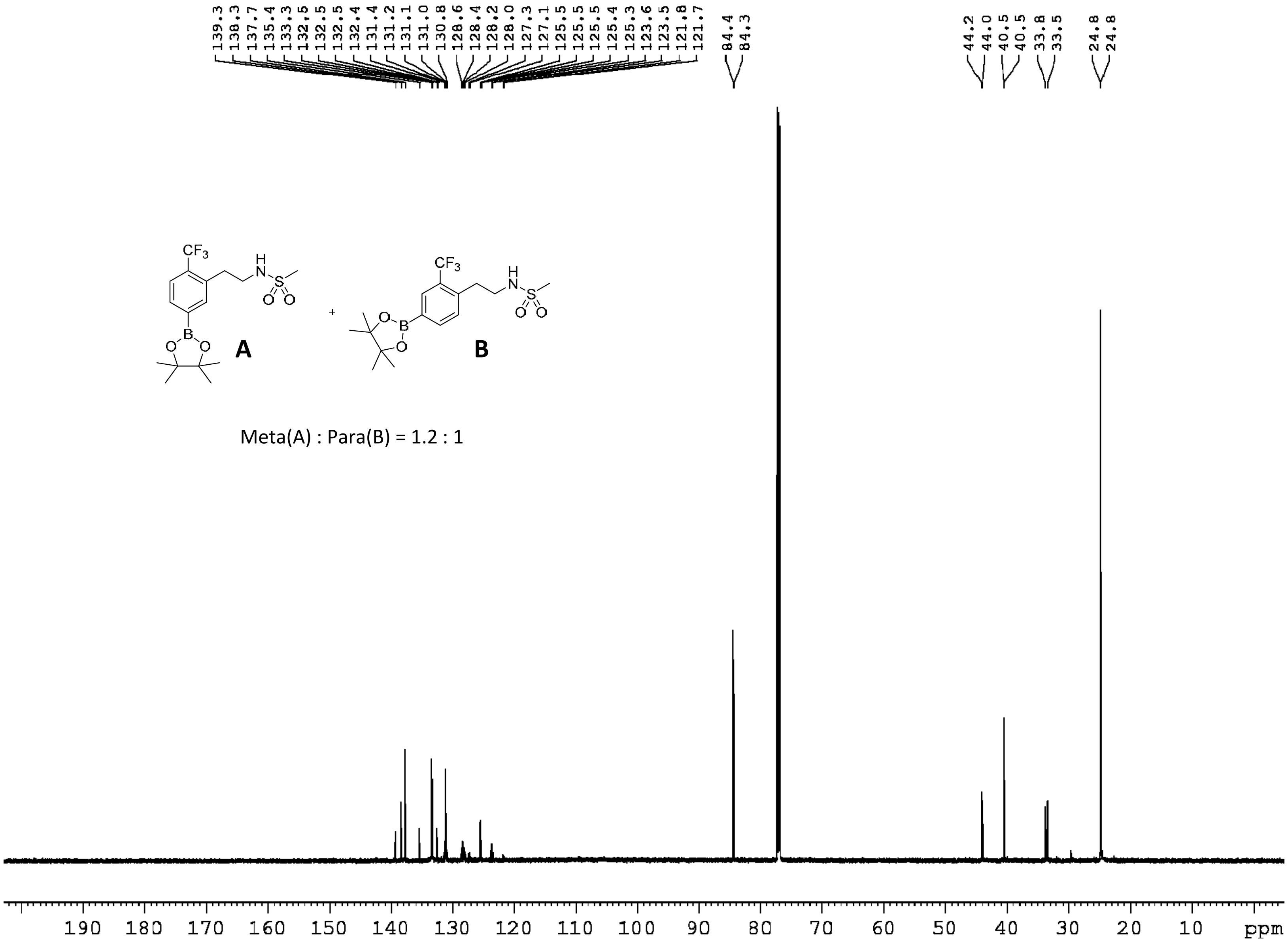

A

B

Meta(A) : Para(B) = 1.2 : 1

^{1}H NMR (600 MHz, $CDCl_3$) of N-(5-(4,4,5,5-tetramethyl-1,3,2-dioxaborolan-2-yl)-2-(trifluoromethyl)phenethyl)methanesulfonamide (5b) – Ligand 1a – Crude mixture

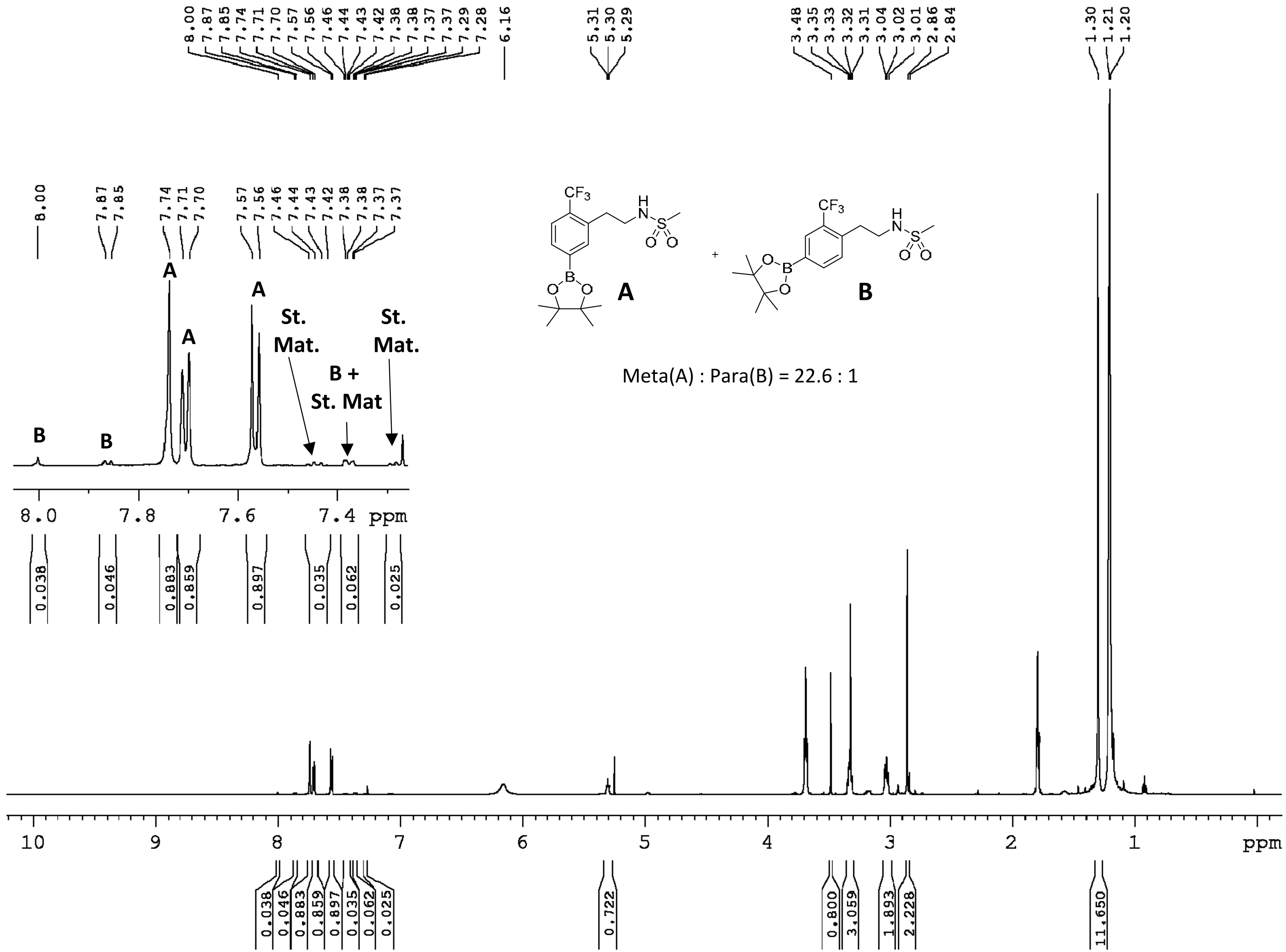

^{1}H NMR (600 MHz, $CDCl_3$) of N-(5-(4,4,5,5-tetramethyl-1,3,2-dioxaborolan-2-yl)-2-(trifluoromethyl)phenethyl)methanesulfonamide (5b) – Ligand 1a – Purified

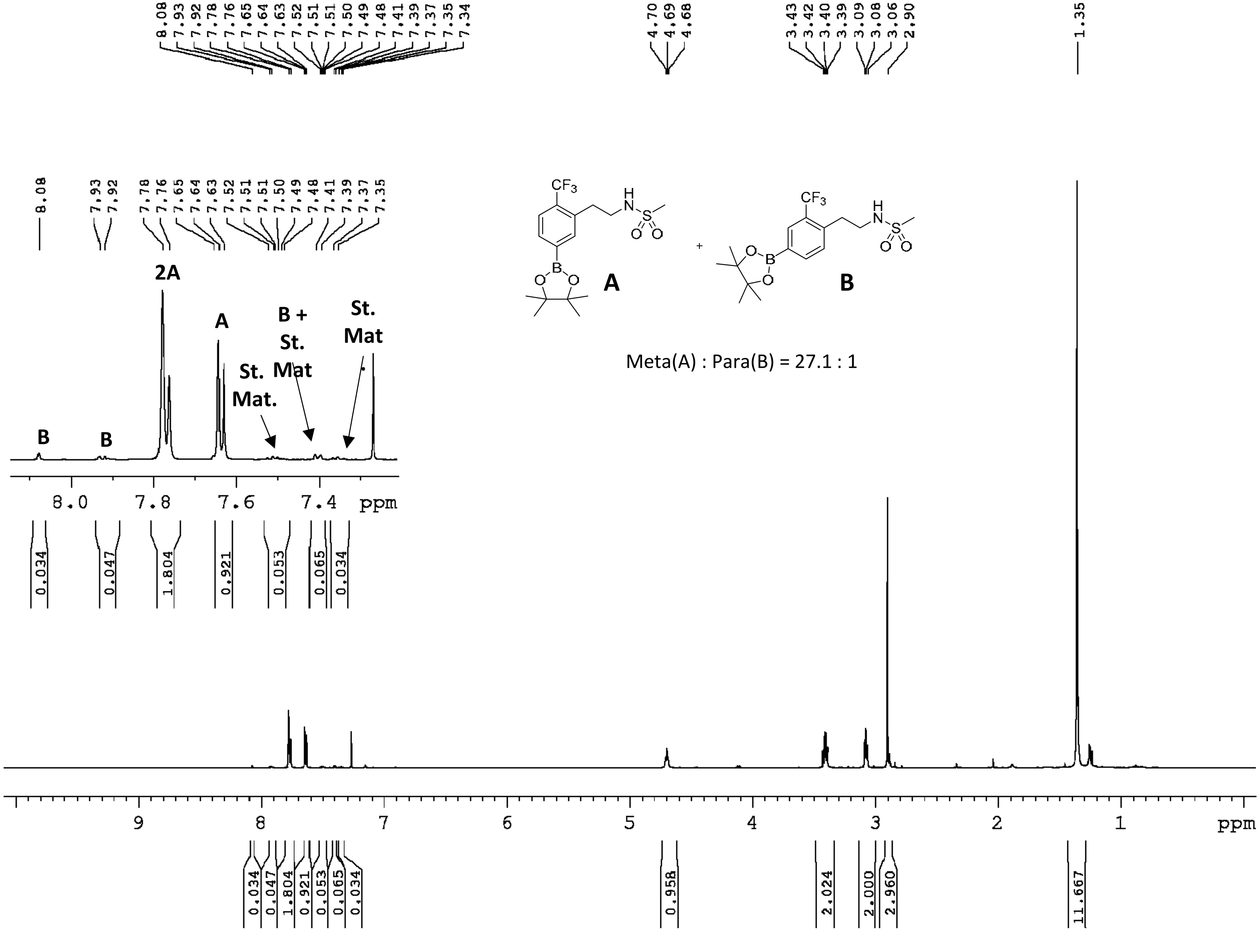

^{13}C NMR (151 MHz, $CDCl_3$) of N-(5-(4,4,5,5-tetramethyl-1,3,2-dioxaborolan-2-yl)-2-(trifluoromethyl)phenethyl)methanesulfonamide (5b) – Ligand 1a – Purified

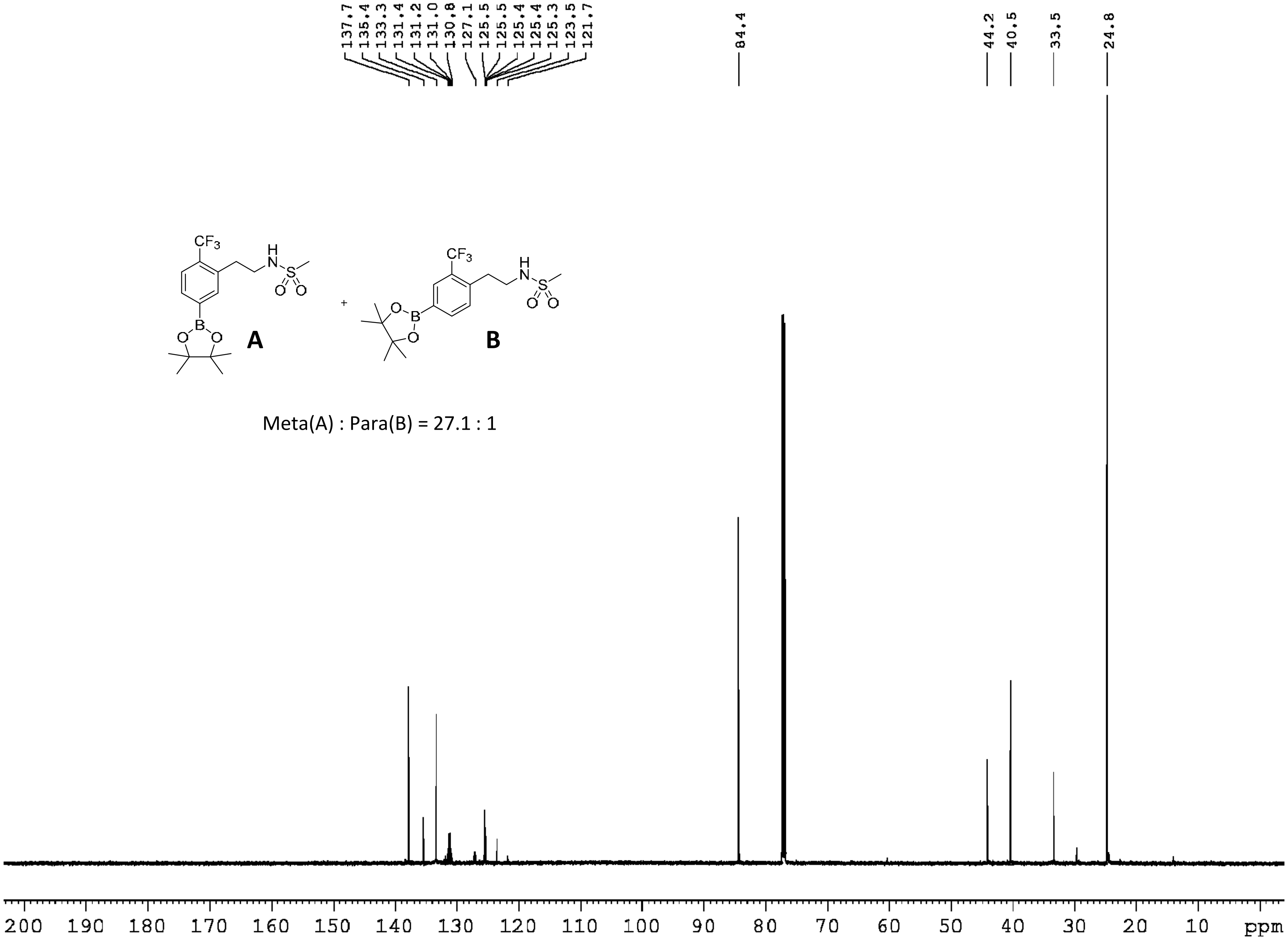

^{1}H NMR (600 MHz, $CDCl_3$) of tert-butyl (5-(4,4,5,5-tetramethyl-1,3,2-dioxaborolan-2-yl)-2-(trifluoromethyl)phenethyl)carbamate (5c) – Dtbpy – Purified

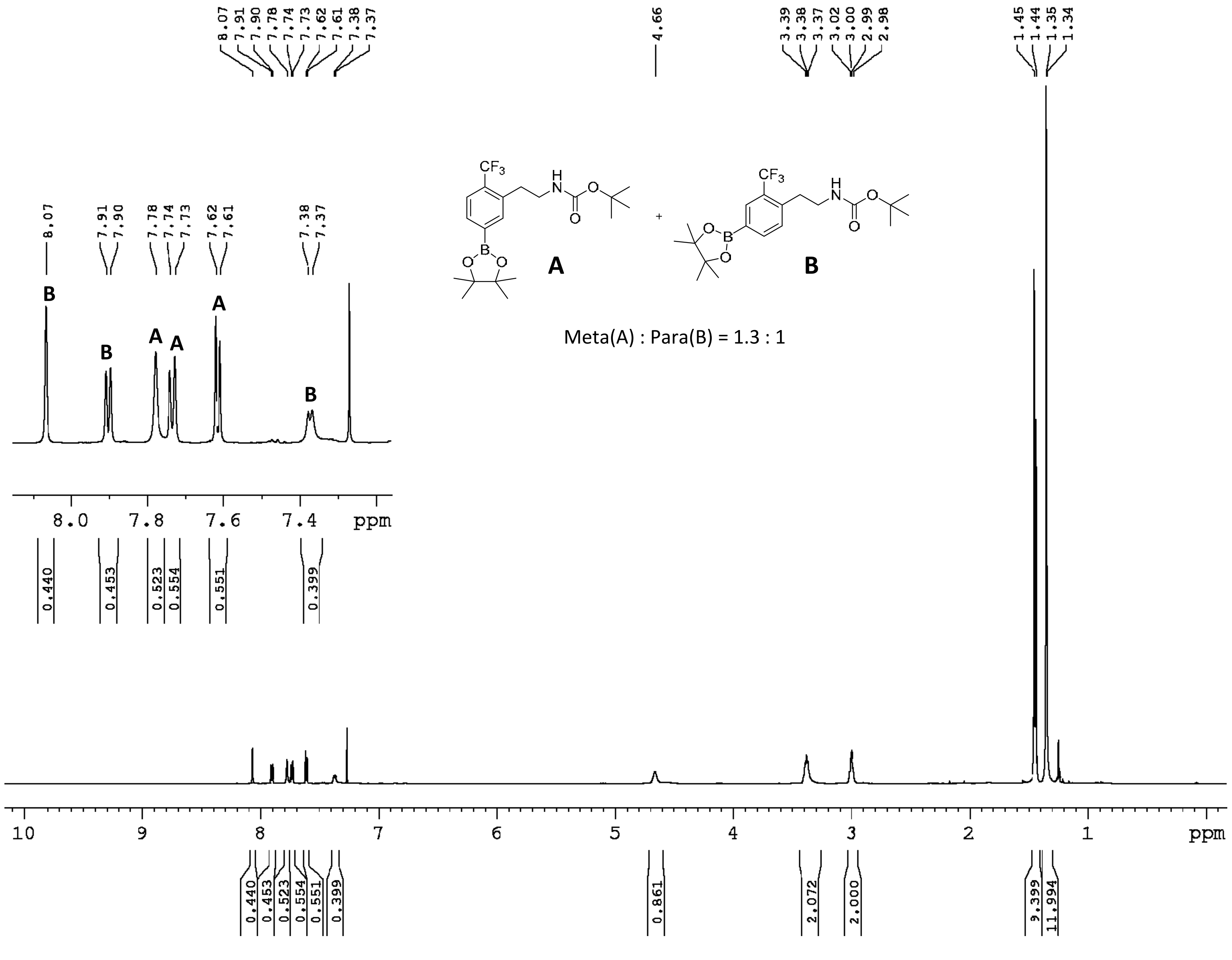

^{13}C NMR (151 MHz, $CDCl_3$) of tert-butyl (5-(4,4,5,5-tetramethyl-1,3,2-dioxaborolan-2-yl)-2-(trifluoromethyl)phenethyl)carbamate (5c) – Dtbpy – Purified

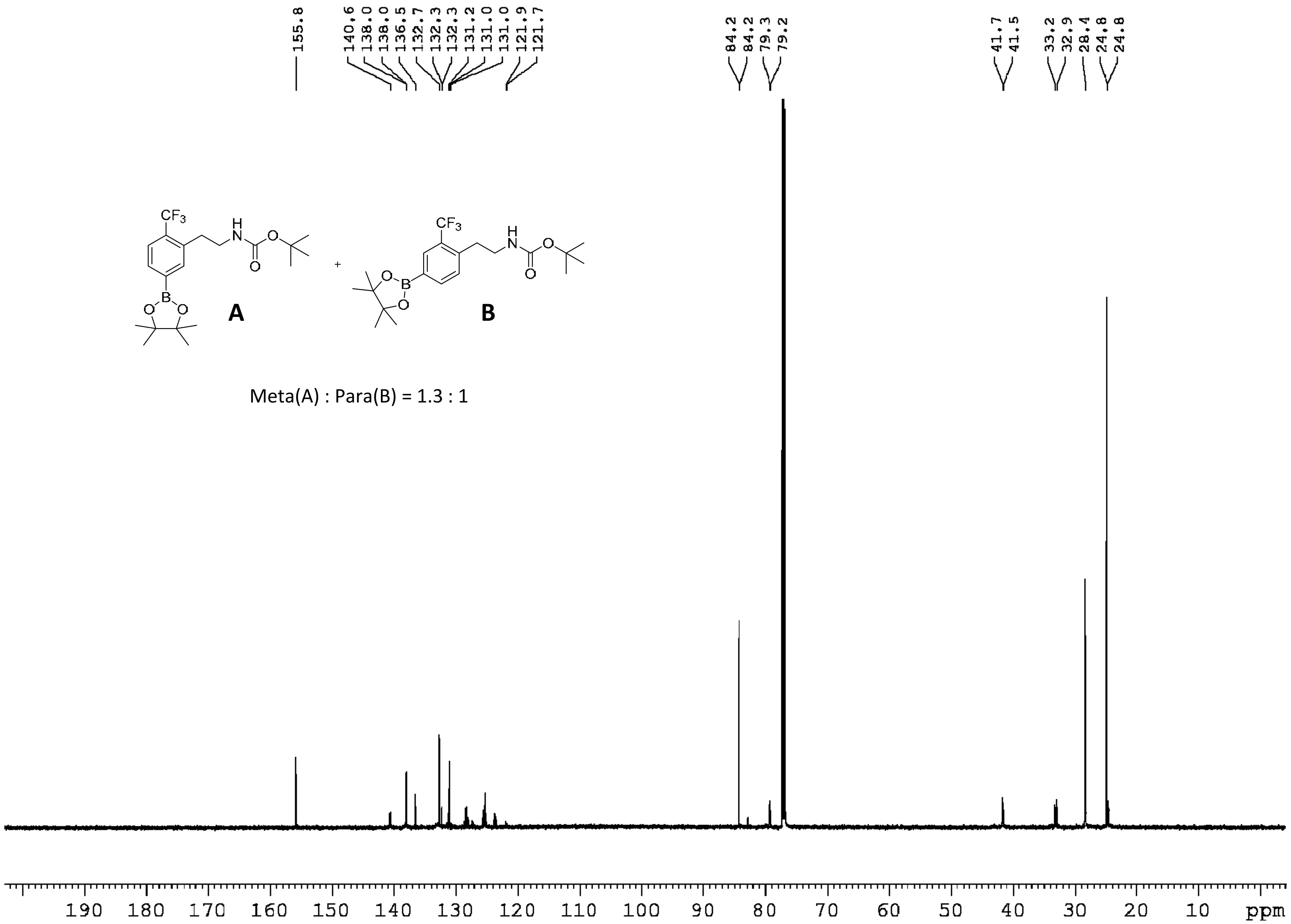

^{1}H NMR (600 MHz, $CDCl_3$) of tert-butyl (5-(4,4,5,5-tetramethyl-1,3,2-dioxaborolan-2-yl)-2-(trifluoromethyl)phenethyl)carbamate (5c) – Ligand 1a – Crude mixture

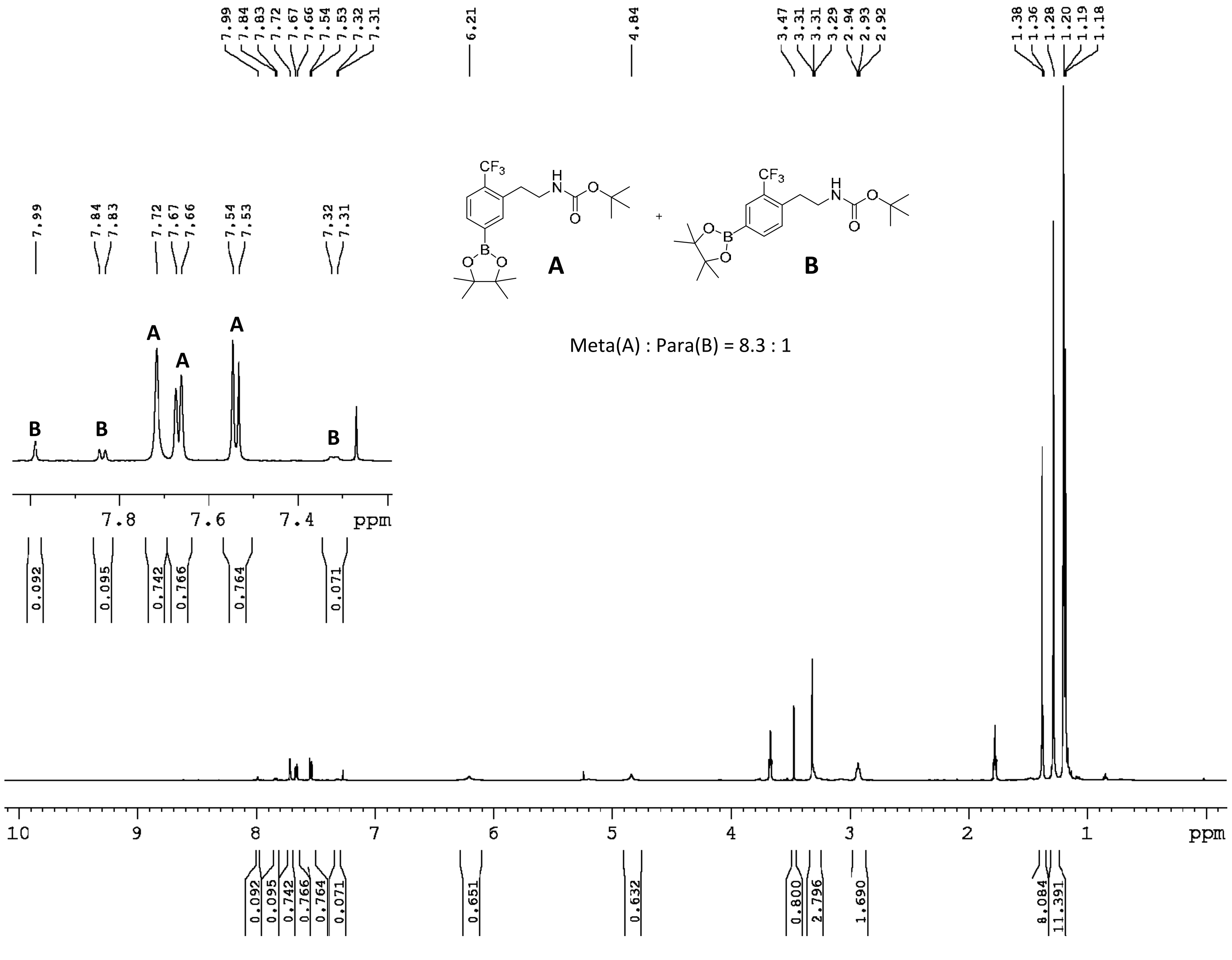

^{1}H NMR (600 MHz, $CDCl_3$) of tert-butyl (5-(4,4,5,5-tetramethyl-1,3,2-dioxaborolan-2-yl)-2-(trifluoromethyl)phenethyl)carbamate (5c) – Ligand 1a – Purified

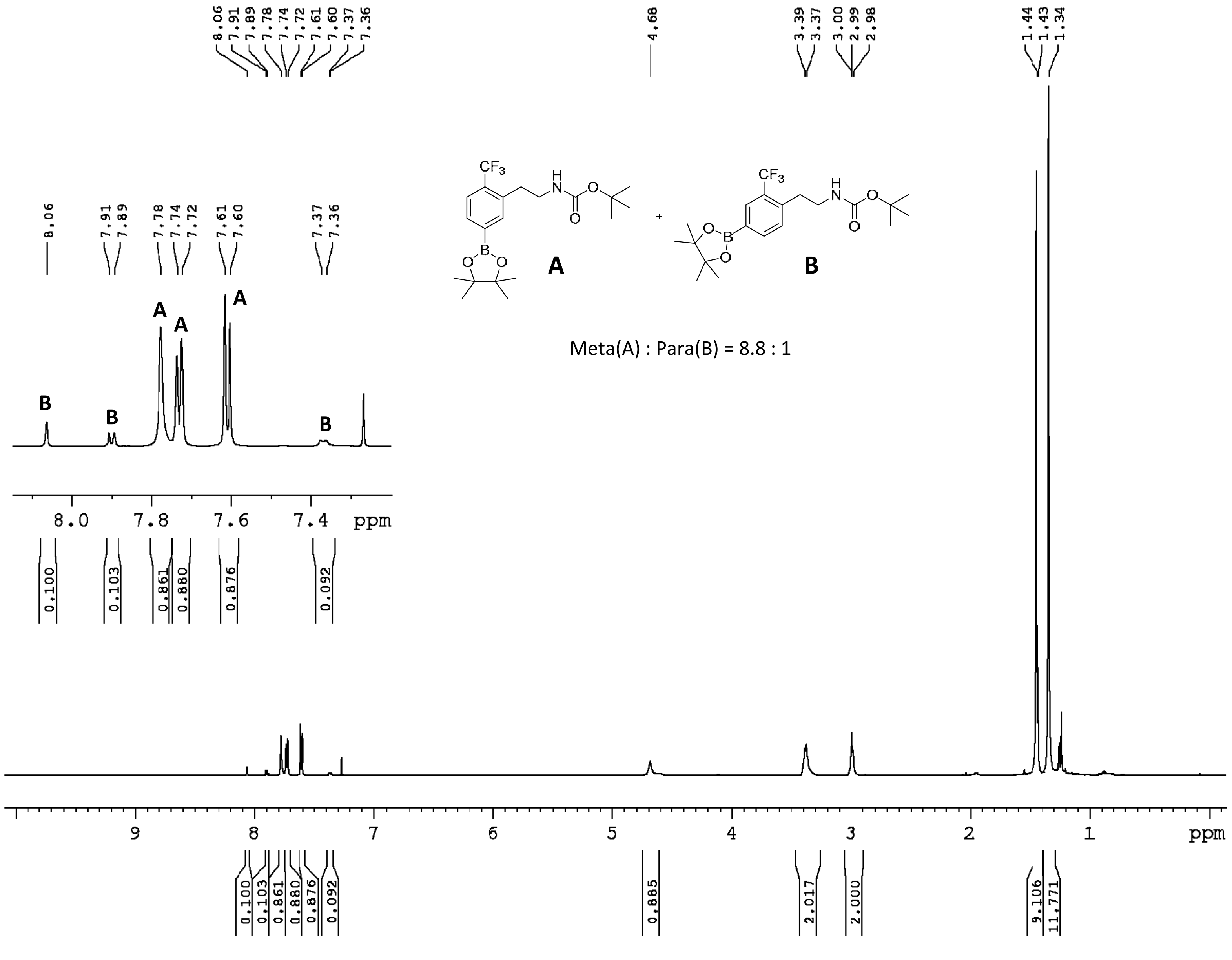

^{13}C NMR (151 MHz, $CDCl_3$) of tert-butyl (5-(4,4,5,5-tetramethyl-1,3,2-dioxaborolan-2-yl)-2-(trifluoromethyl)phenethyl)carbamate (5c) – Ligand 1a – Purified

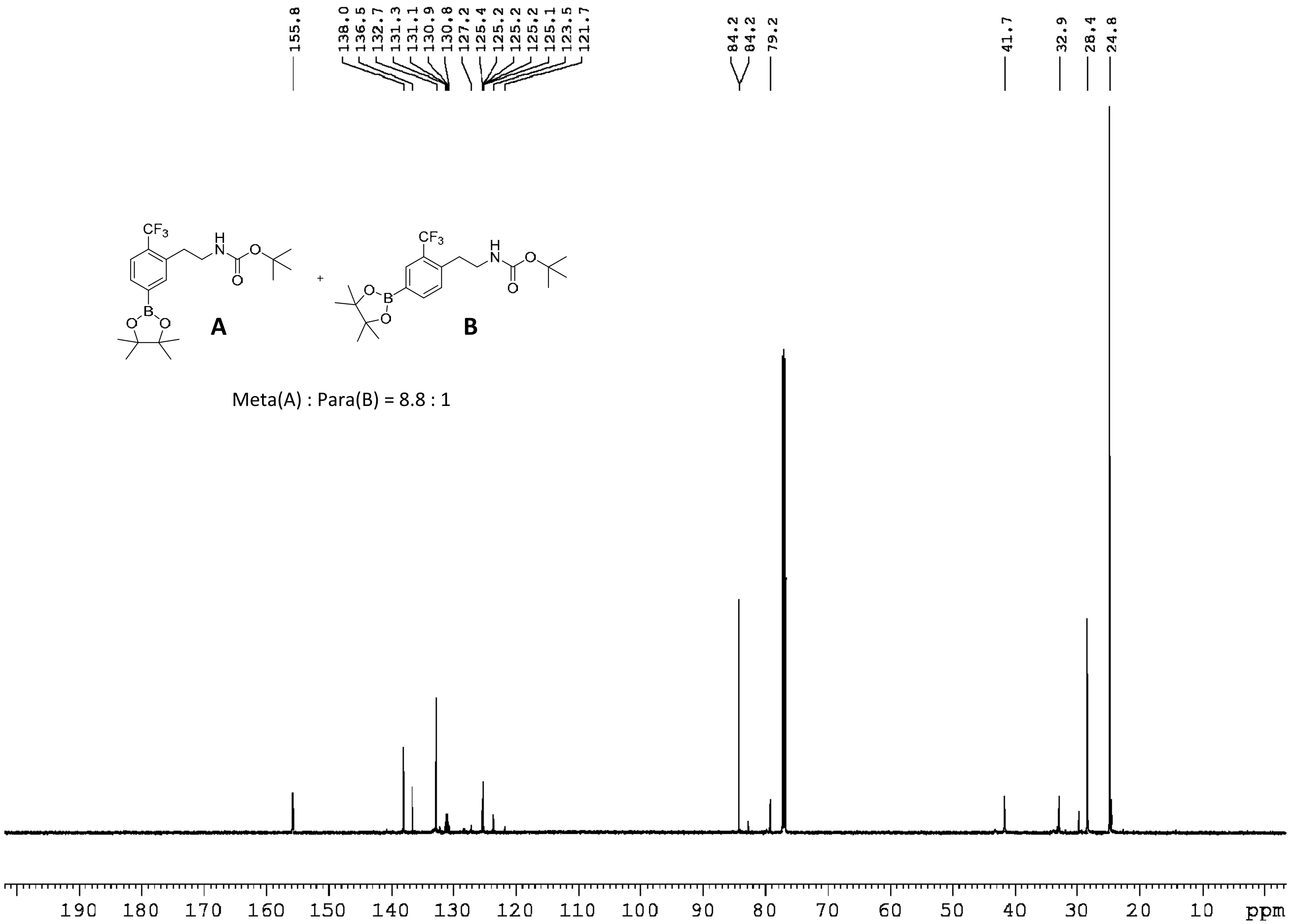

^{1}H NMR (500 MHz, $CDCl_3$) of 2,2,2-trifluoro-N-(2-iodo-5-(4,4,5,5-tetramethyl-1,3,2-dioxaborolan-2-yl)phenethyl)acetamide (5d) – Dtbpy – Purified

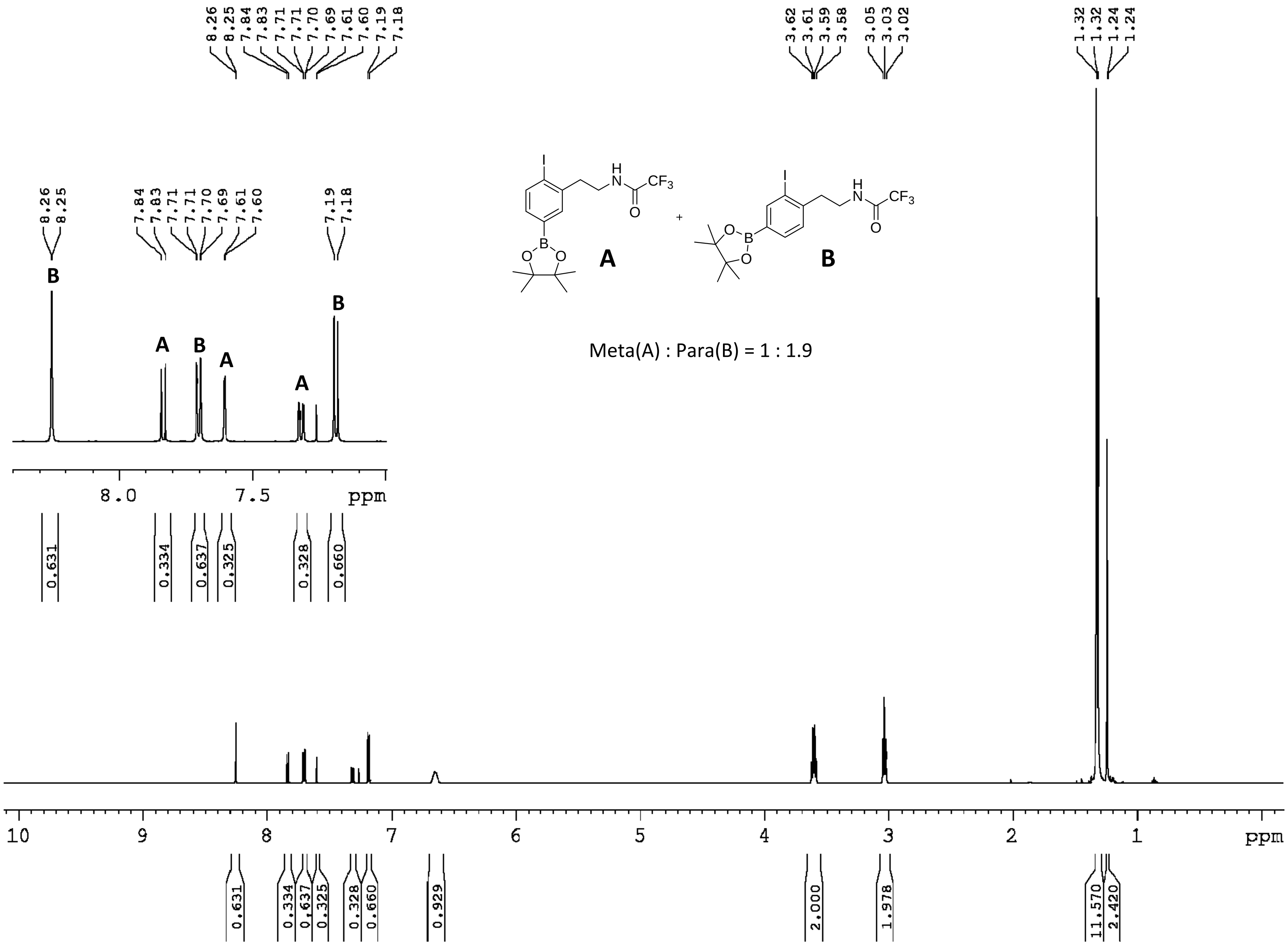

^{13}C NMR (126 MHz, $CDCl_3$) of 2,2,2-trifluoro-N-(2-iodo-5-(4,4,5,5-tetramethyl-1,3,2-dioxaborolan-2-yl)phenethyl)acetamide (5d) – Dtbpy – Purified

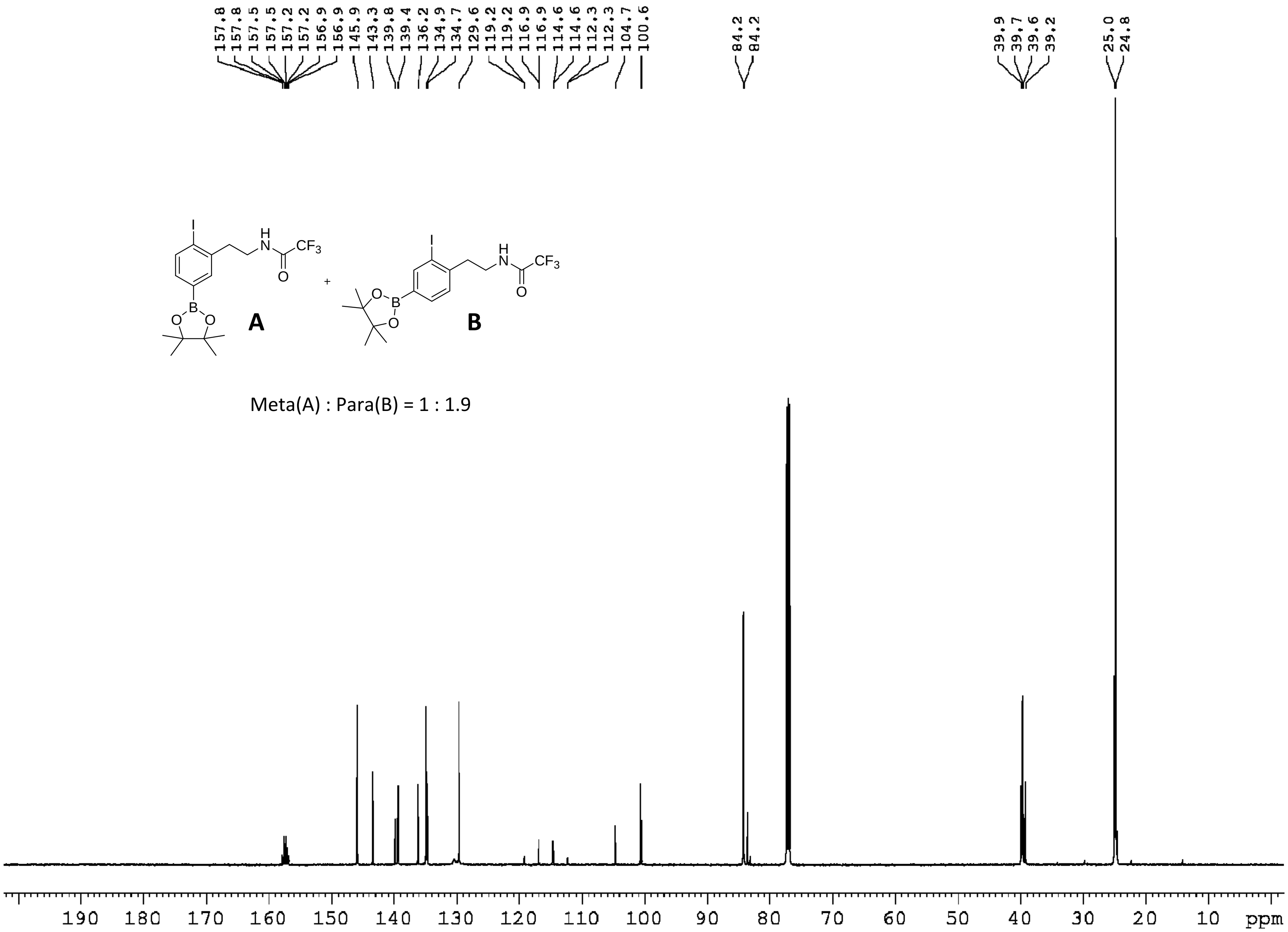

^{1}H NMR (500 MHz, $CDCl_3$) of 2,2,2-trifluoro-N-(2-iodo-5-(4,4,5,5-tetramethyl-1,3,2-dioxaborolan-2-yl)phenethyl)acetamide (5d) – Ligand 1a – Crude mixture

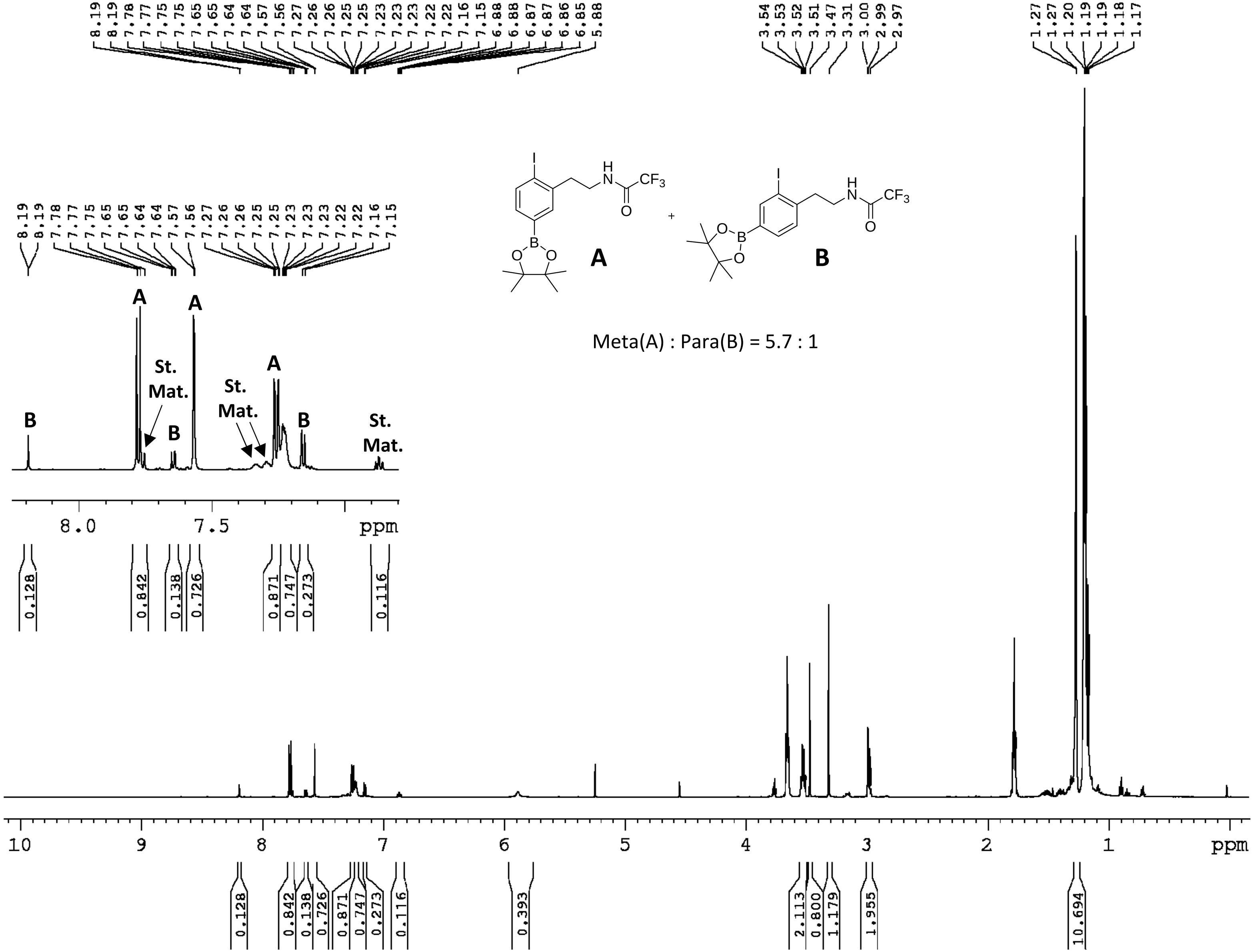

^{1}H NMR (500 MHz, $CDCl_3$) of 2,2,2-trifluoro-N-(2-iodo-5-(4,4,5,5-tetramethyl-1,3,2-dioxaborolan-2-yl)phenethyl)acetamide (5d) – Ligand 1a – Purified

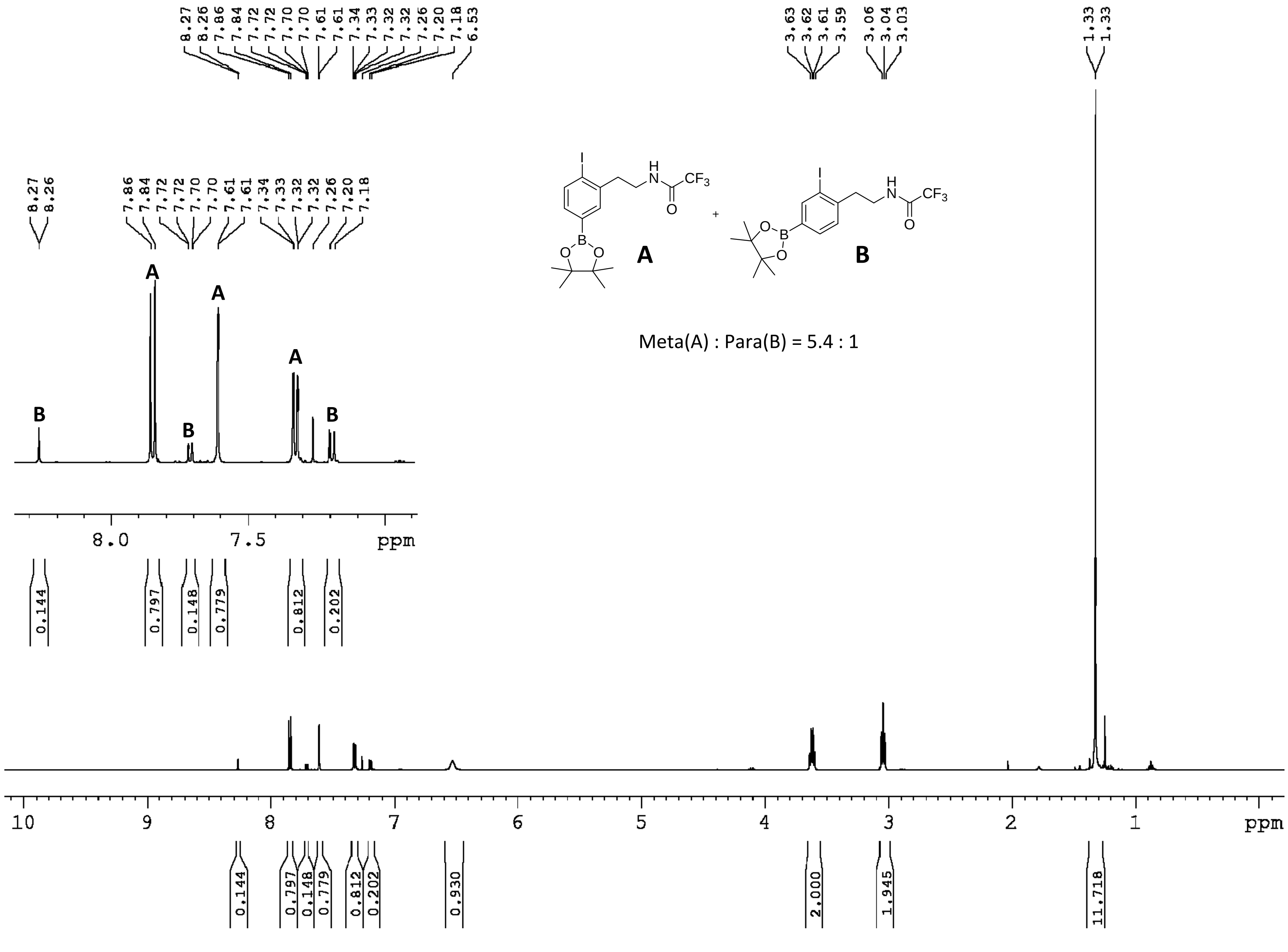

^{13}C NMR (126 MHz, $CDCl_3$) of 2,2,2-trifluoro-N-(2-iodo-5-(4,4,5,5-tetramethyl-1,3,2-dioxaborolan-2-yl)phenethyl)acetamide (5d) – Ligand 1a – Purified

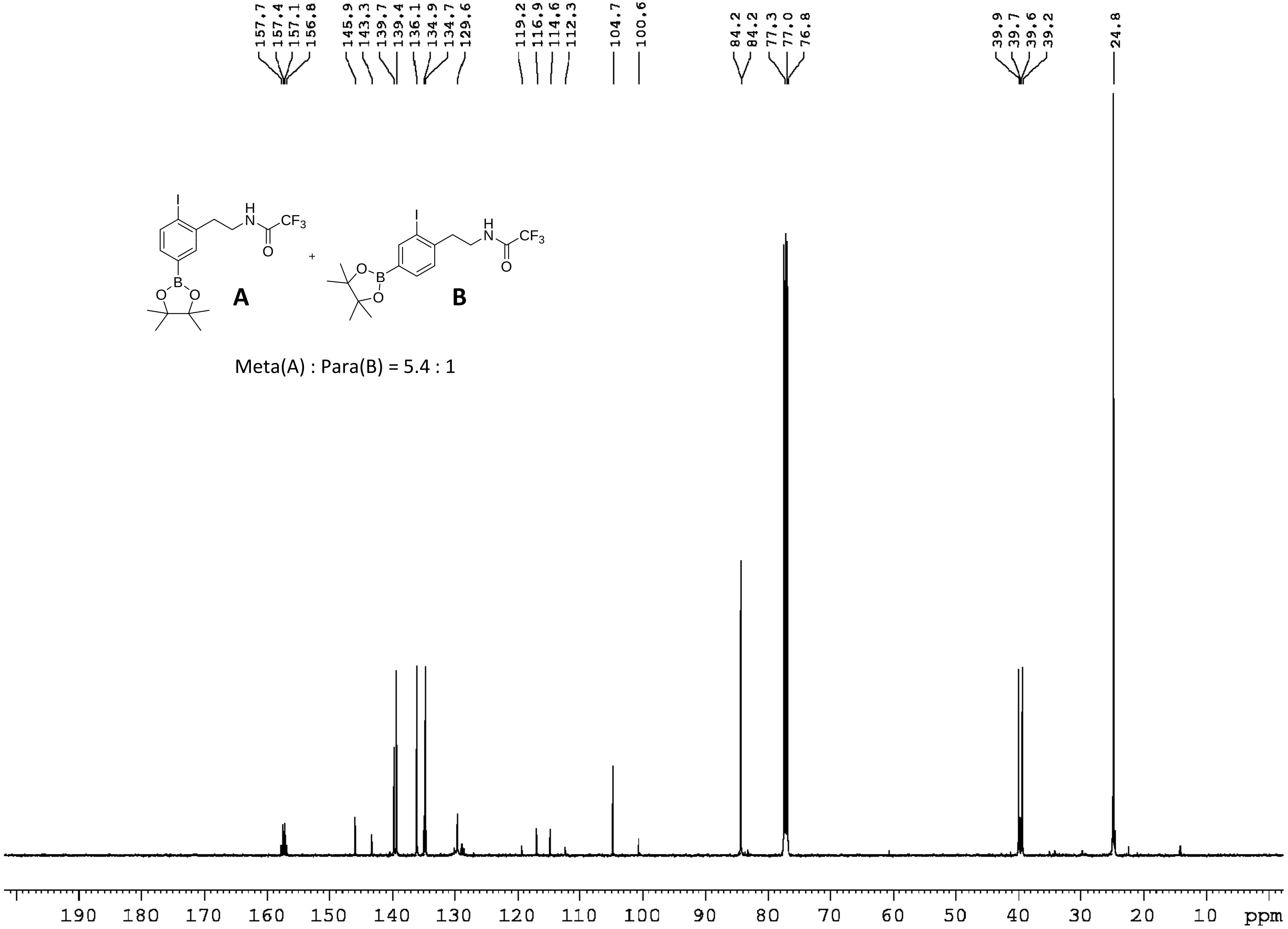

^{1}H NMR (600 MHz, $CDCl_3$) of 2,2,2-trifluoro-N-(3-fluoro-5-(4,4,5,5-tetramethyl-1,3,2-dioxaborolan-2-yl)phenethyl)acetamide (5e) – Dtbpy – Purified

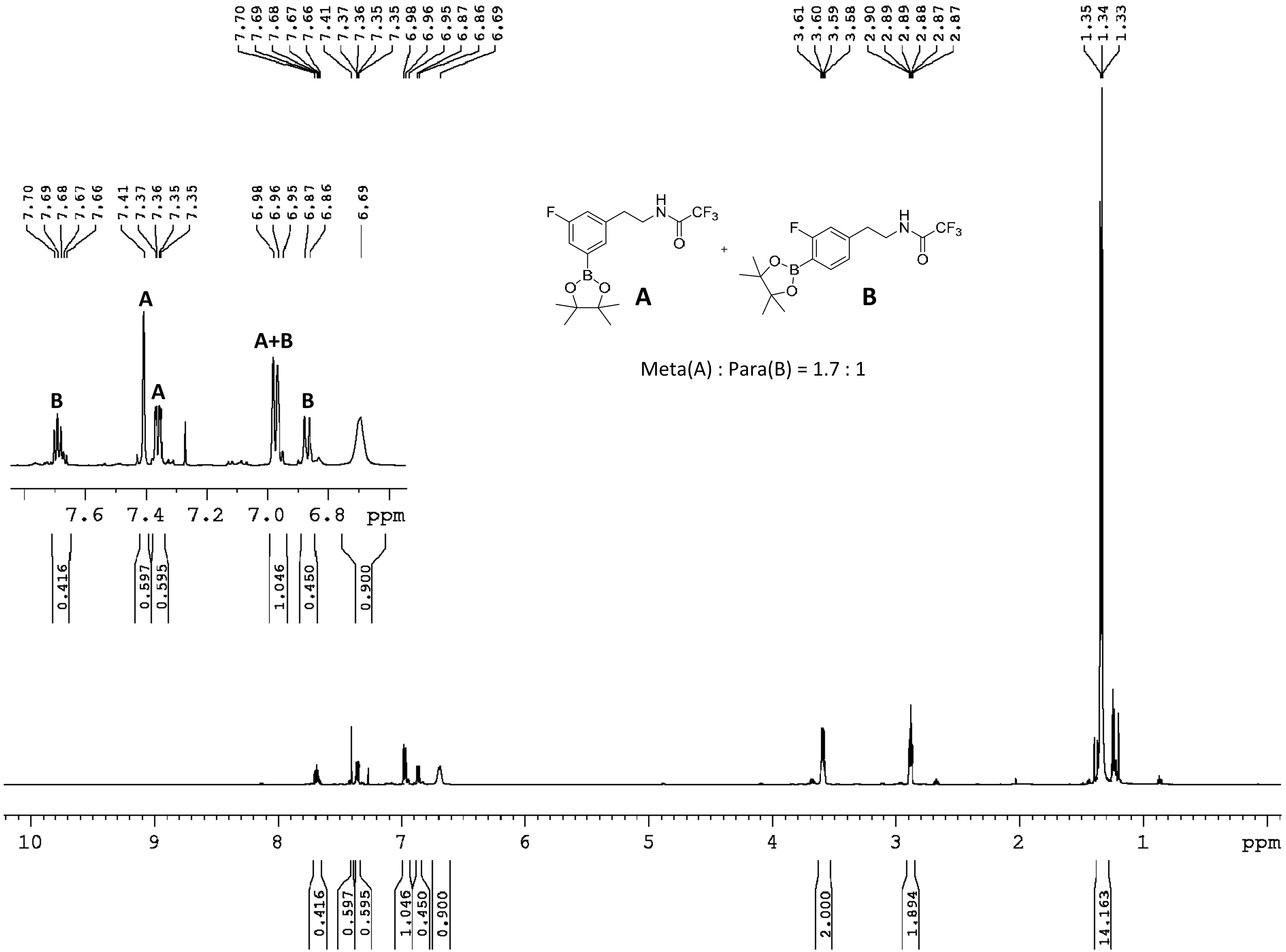

^{13}C NMR (151 MHz, $CDCl_3$) of 2,2,2-trifluoro-N-(3-fluoro-5-(4,4,5,5-tetramethyl-1,3,2-dioxaborolan-2-yl)phenethyl)acetamide (5e) – Dtbpy – Purified

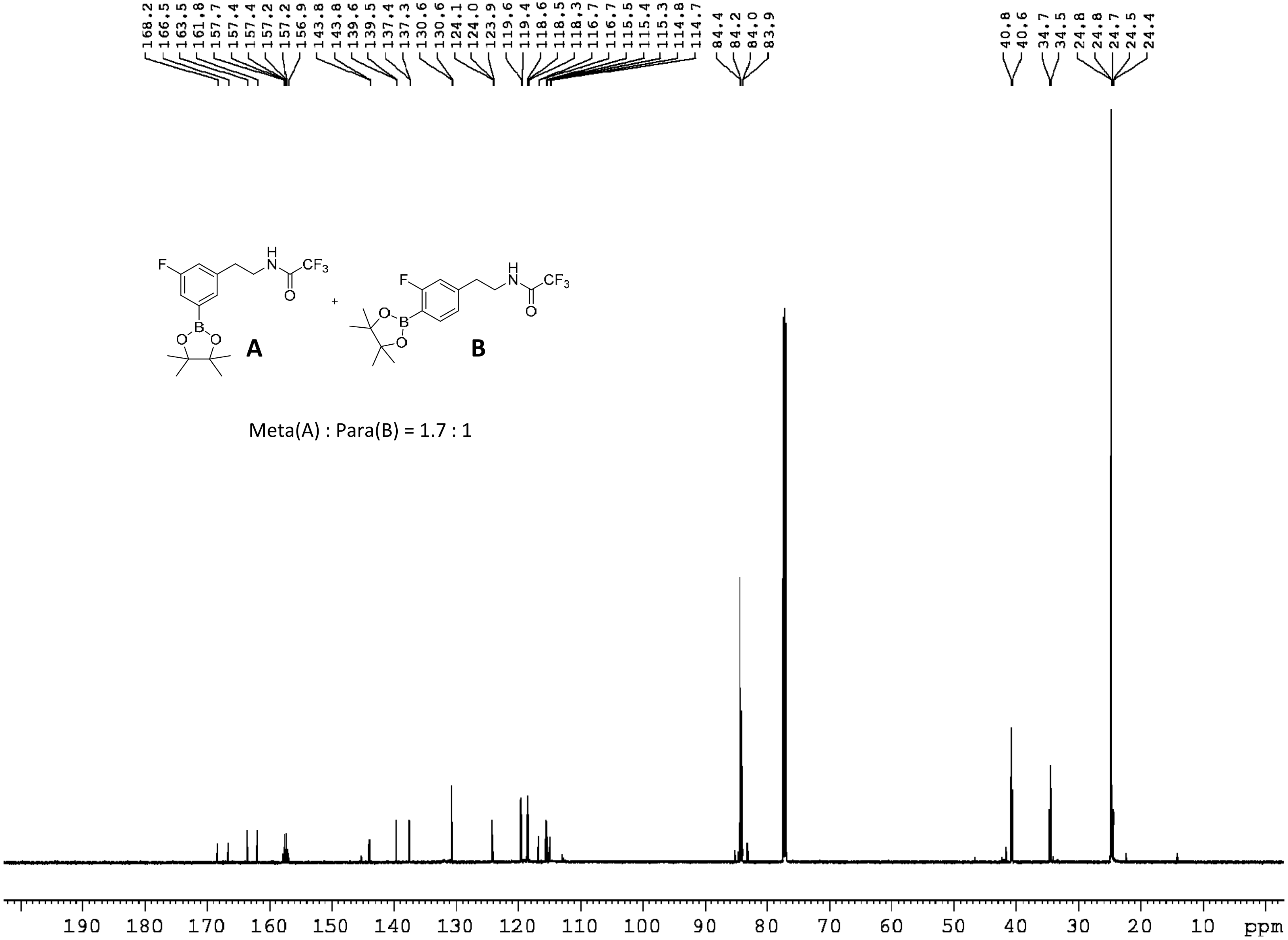

^{1}H NMR (600 MHz, $CDCl_3$) of 2,2,2-trifluoro-N-(3-fluoro-5-(4,4,5,5-tetramethyl-1,3,2-dioxaborolan-2-yl)phenethyl)acetamide (5e) – Ligand 1a – Crude mixture (X* signifies a product which has also undergone NH borylation)

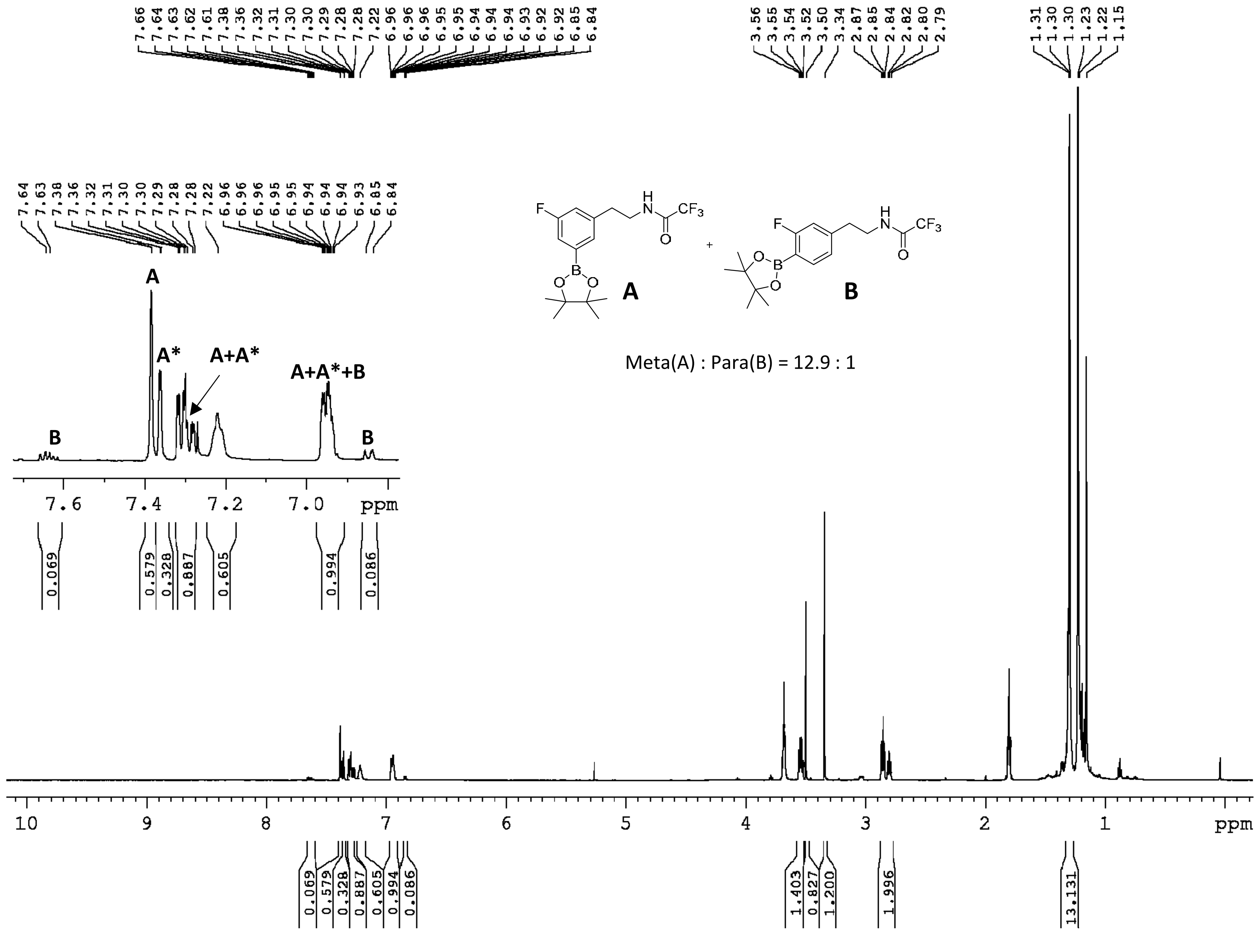

Meta(A) : Para(B) = 12.9 : 1

^{1}H NMR (600 MHz, $CDCl_3$) of 2,2,2-trifluoro-N-(3-fluoro-5-(4,4,5,5-tetramethyl-1,3,2-dioxaborolan-2-yl)phenethyl)acetamide (5e) – Ligand 1a – Purified

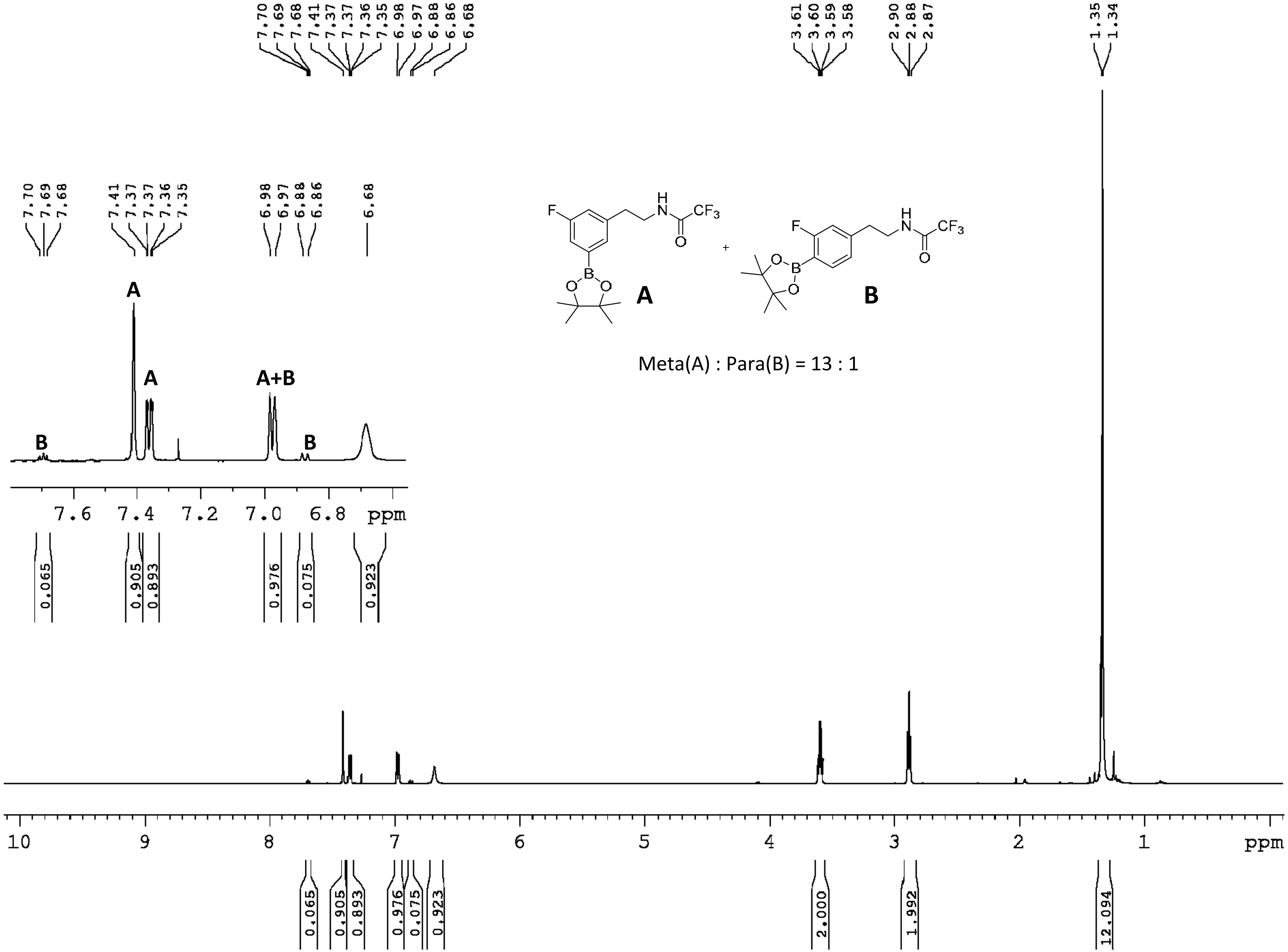

^{13}C NMR (151 MHz, $CDCl_3$) of 2,2,2-trifluoro-N-(3-fluoro-5-(4,4,5,5-tetramethyl-1,3,2-dioxaborolan-2-yl)phenethyl)acetamide (5e) – Ligand 1a – Purified

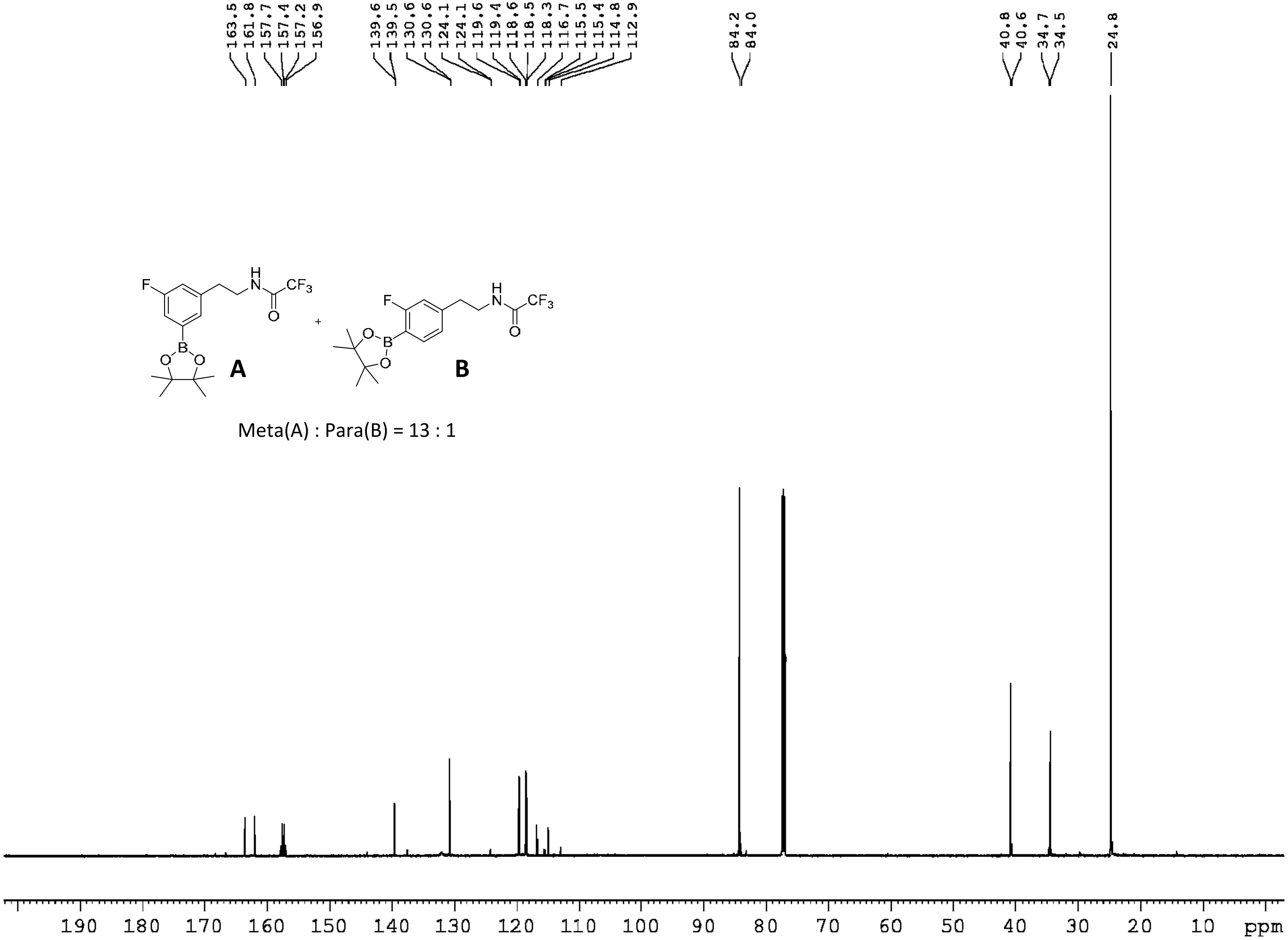

^{1}H NMR (600 MHz, $CDCl_3$) of N-(2-bromo-5-(4,4,5,5-tetramethyl-1,3,2-dioxaborolan-2-yl)phenethyl)-2,2,2-trifluoroacetamide (5f) – Dtbpy – Purified

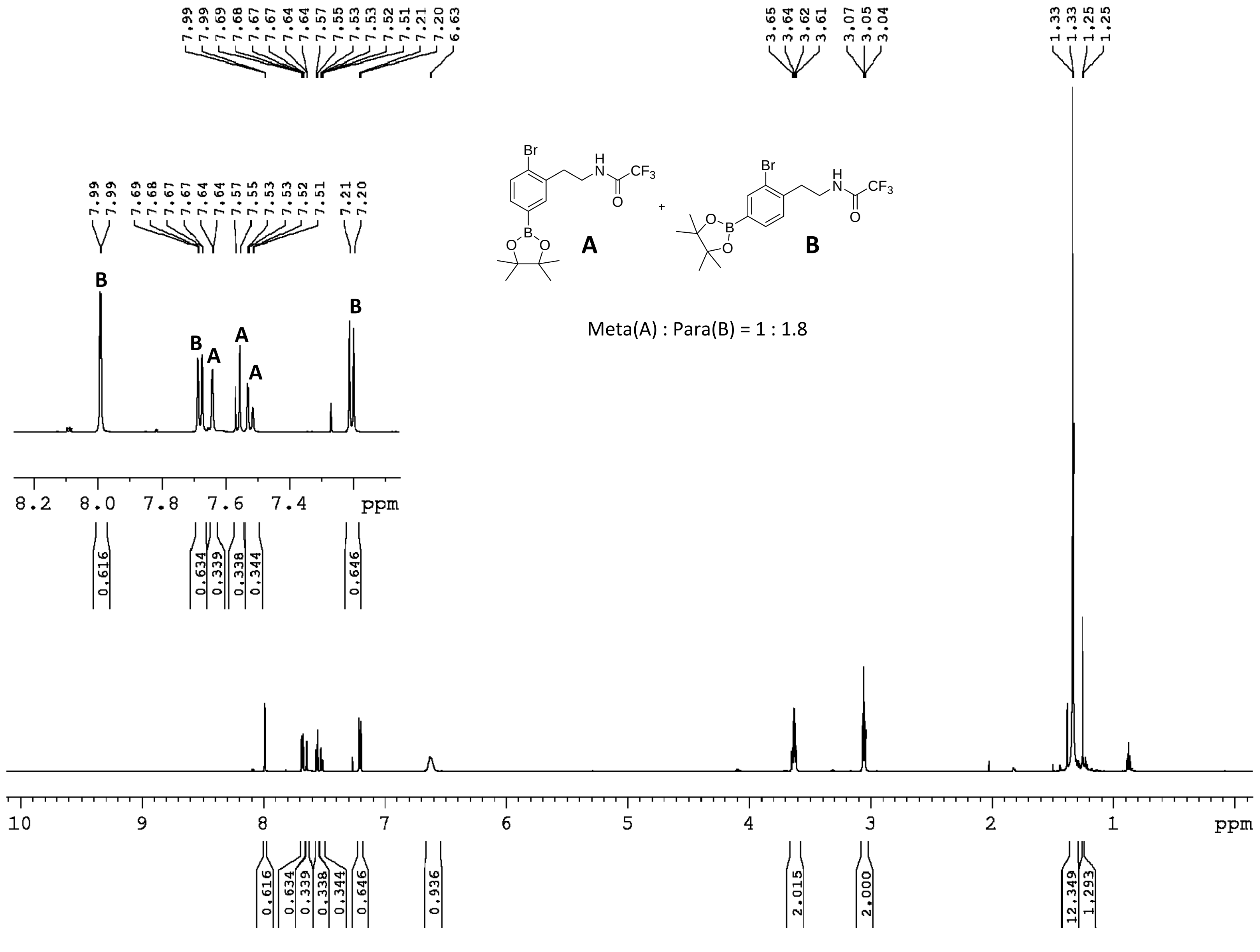

^{13}C NMR (151 MHz, $CDCl_3$) of N-(2-bromo-5-(4,4,5,5-tetramethyl-1,3,2-dioxaborolan-2-yl)phenethyl)-2,2,2-trifluoroacetamide (5f) – Dtbpy – Purified

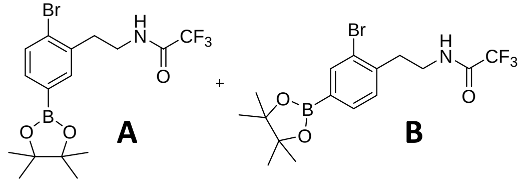

Meta(A) : Para(B) = 1 : 1.8

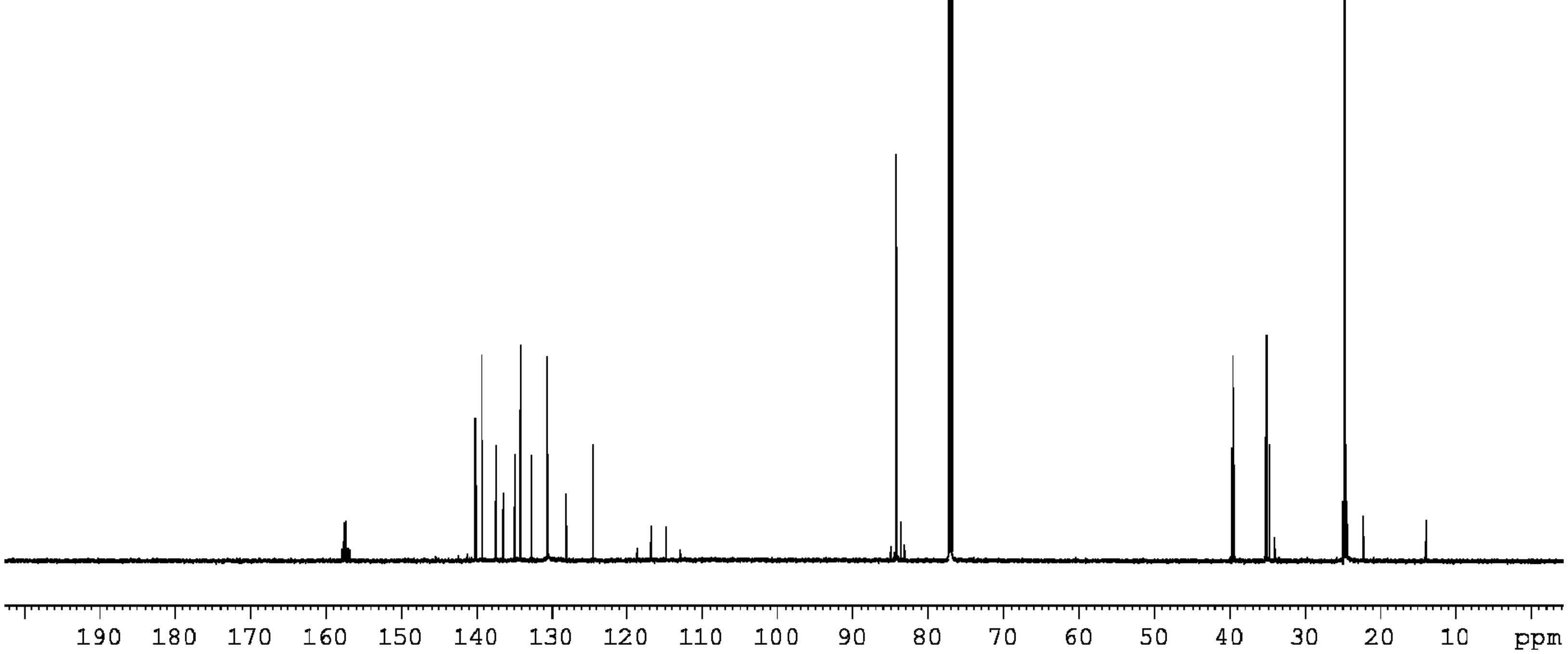

^{1}H NMR (600 MHz, $CDCl_3$) of N-(2-bromo-5-(4,4,5,5-tetramethyl-1,3,2-dioxaborolan-2-yl)phenethyl)-2,2,2-trifluoroacetamide (5f) – Ligand 1a – Crude mixture

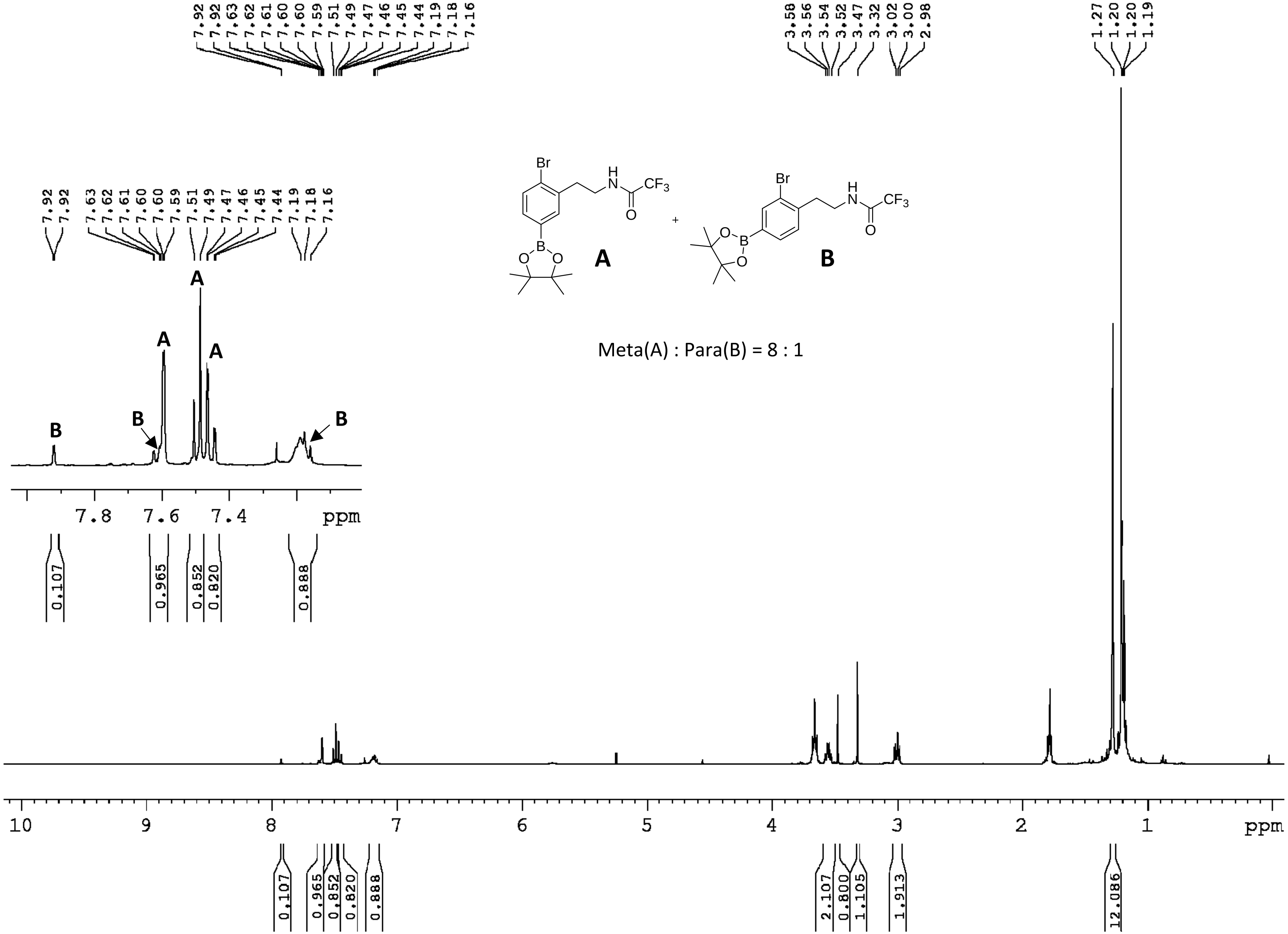

^{1}H NMR (600 MHz, $CDCl_3$) of N-(2-bromo-5-(4,4,5,5-tetramethyl-1,3,2-dioxaborolan-2-yl)phenethyl)-2,2,2-trifluoroacetamide (5f) – Ligand 1a – Purified

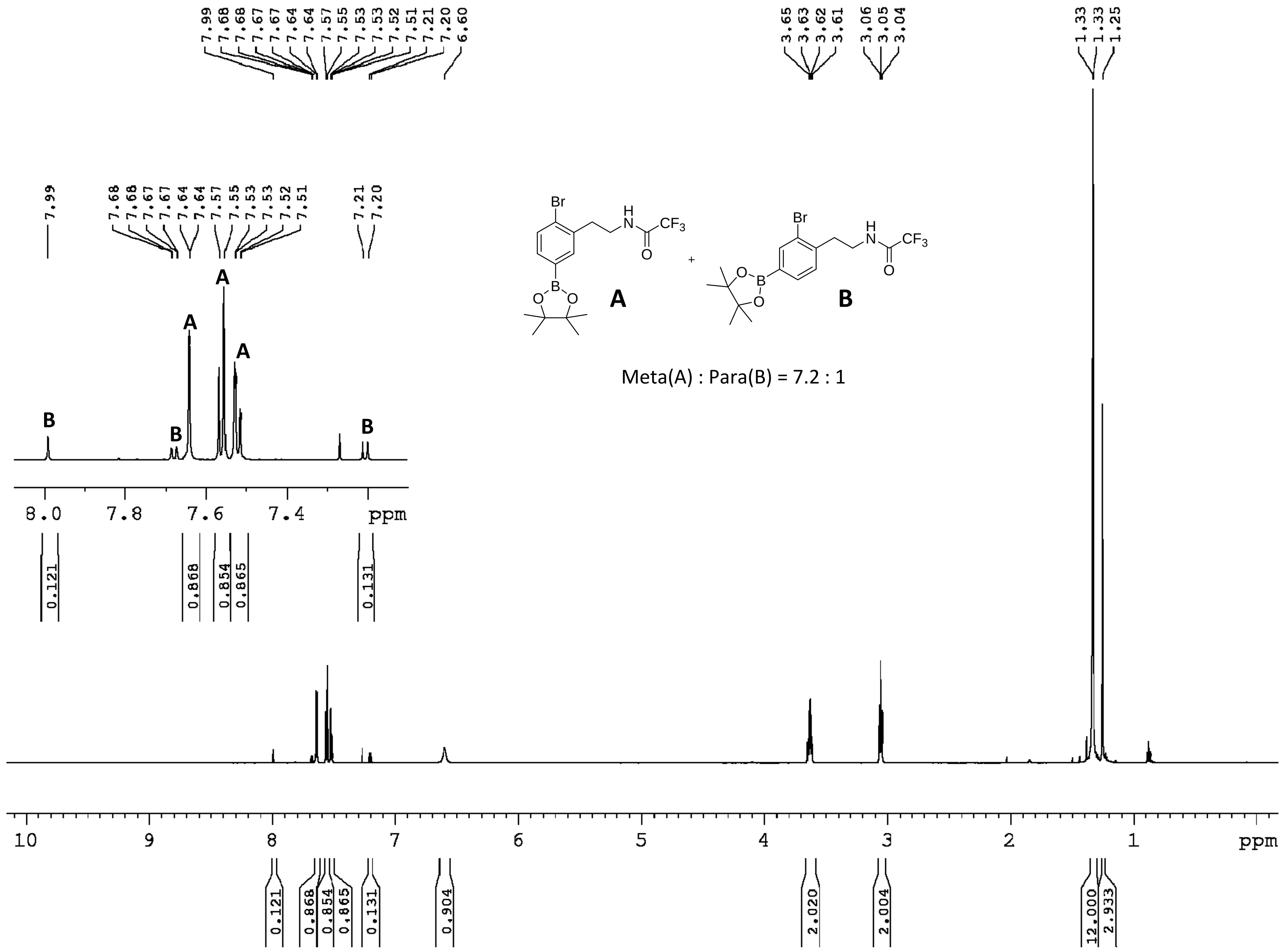

^{13}C NMR (151 MHz, $CDCl_3$) of N-(2-bromo-5-(4,4,5,5-tetramethyl-1,3,2-dioxaborolan-2-yl)phenethyl)-2,2,2-trifluoroacetamide (5f) – Ligand 1a – Purified

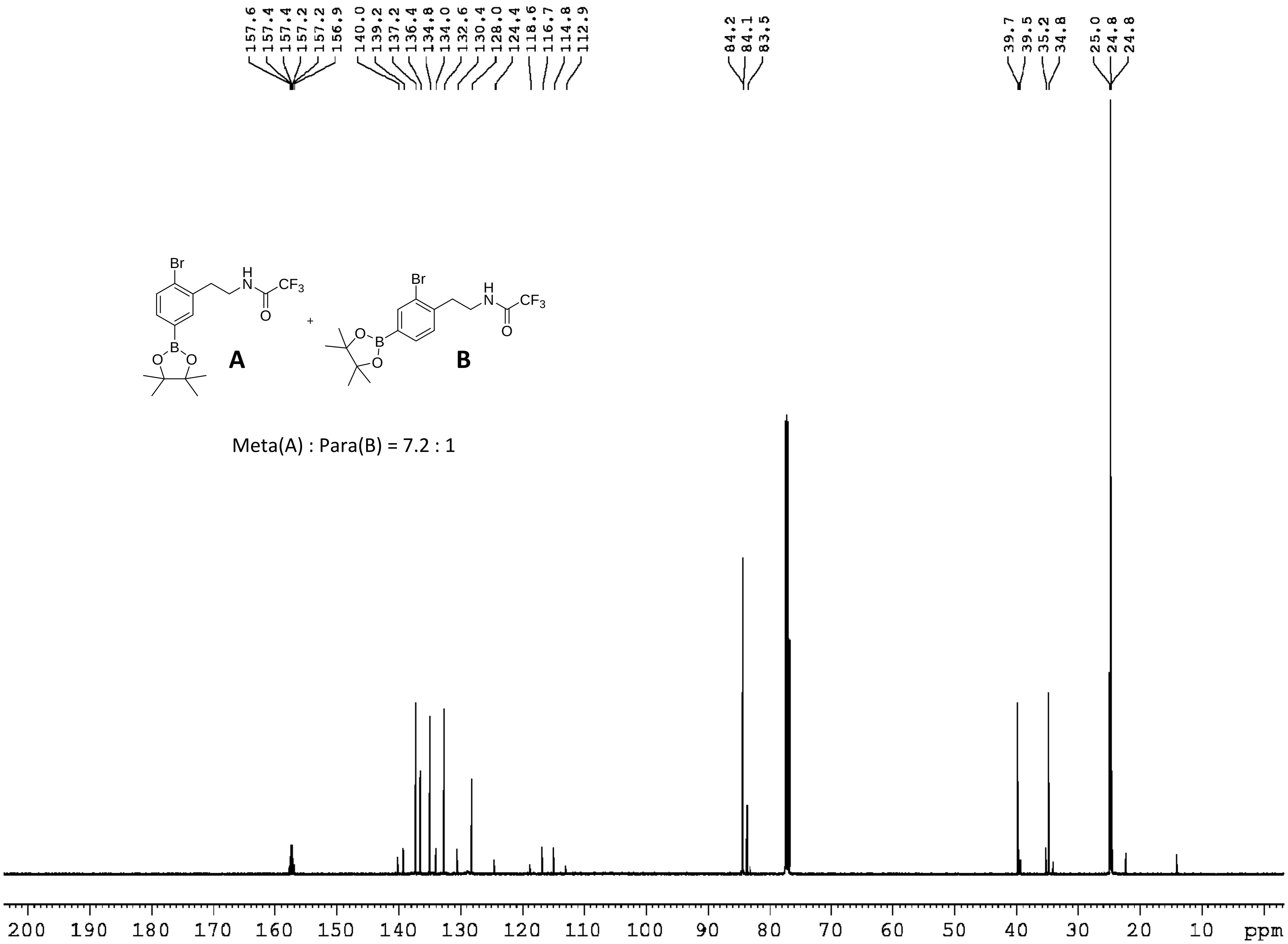

^{1}H NMR (600 MHz, $CDCl_3$) of methyl 4-(4,4,5,5-tetramethyl-1,3,2-dioxaborolan-2-yl)-2-(2-(2,2,2-trifluoroacetamido)ethyl)benzoate (5g) – Dtbpy – Purified

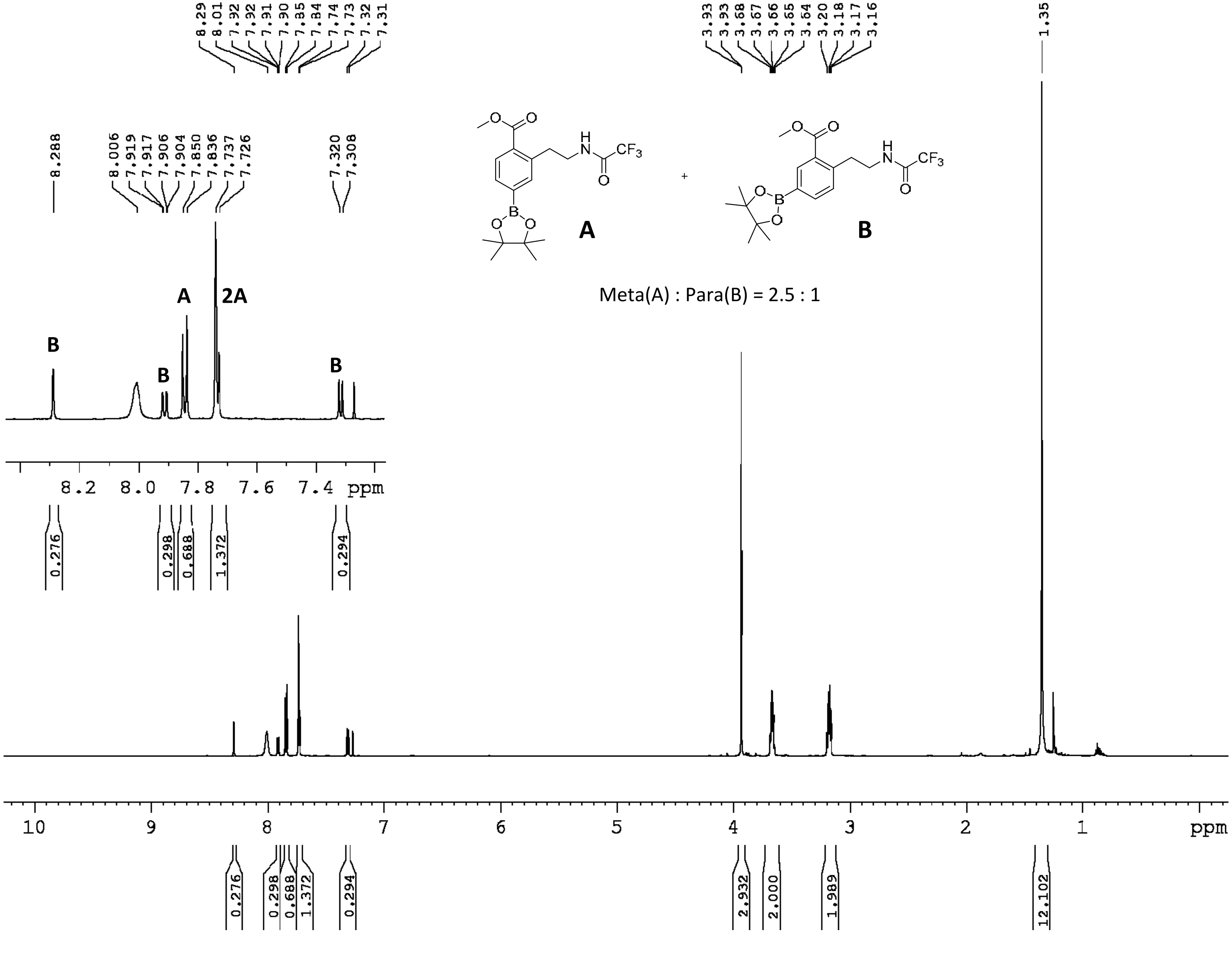

^{13}C NMR (151 MHz, $CDCl_3$) of methyl 4-(4,4,5,5-tetramethyl-1,3,2-dioxaborolan-2-yl)-2-(2-(2,2,2-trifluoroacetamido)ethyl)benzoate (5g) – Dtbpy – Purified

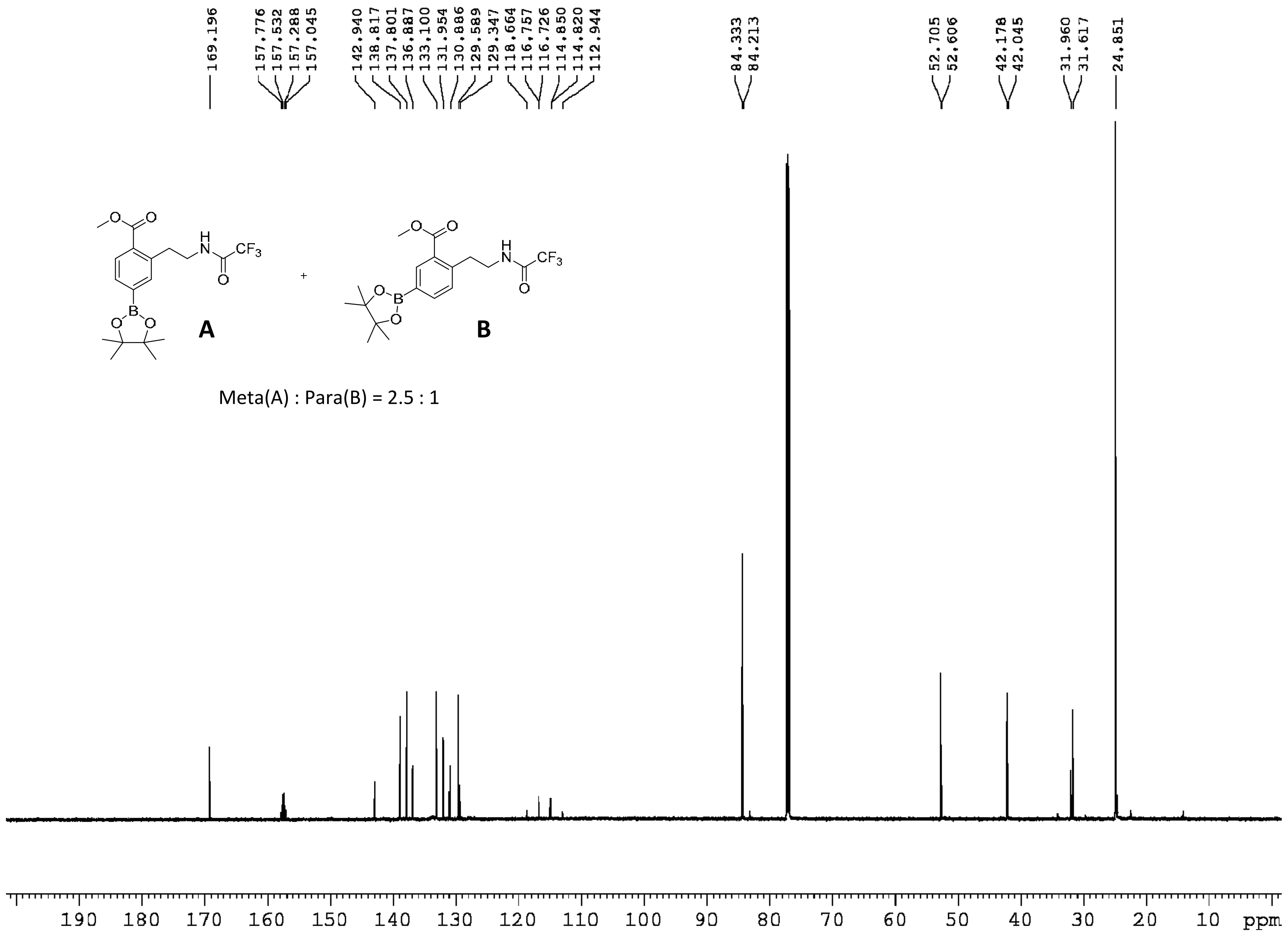

^{1}H NMR (600 MHz, $CDCl_3$) of methyl 4-(4,4,5,5-tetramethyl-1,3,2-dioxaborolan-2-yl)-2-(2-(2,2,2-trifluoroacetamido)ethyl)benzoate (5g) – Ligand 1a – Crude mixture (X* signifies a product which has also undergone NH borylation)

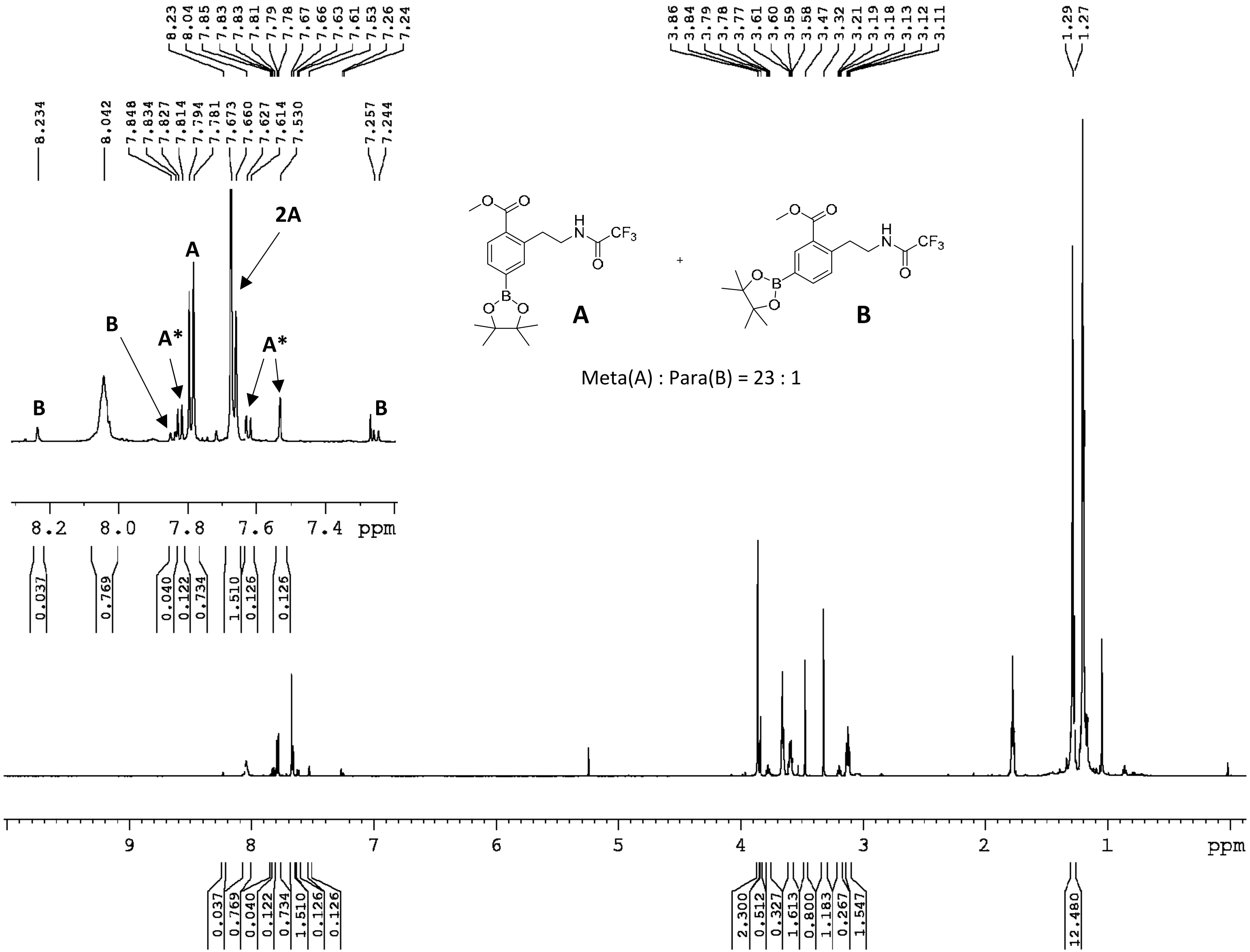

^{1}H NMR (600 MHz, $CDCl_3$) of methyl 4-(4,4,5,5-tetramethyl-1,3,2-dioxaborolan-2-yl)-2-(2-(2,2,2-trifluoroacetamido)ethyl)benzoate (5g) – Ligand 1a – Purified

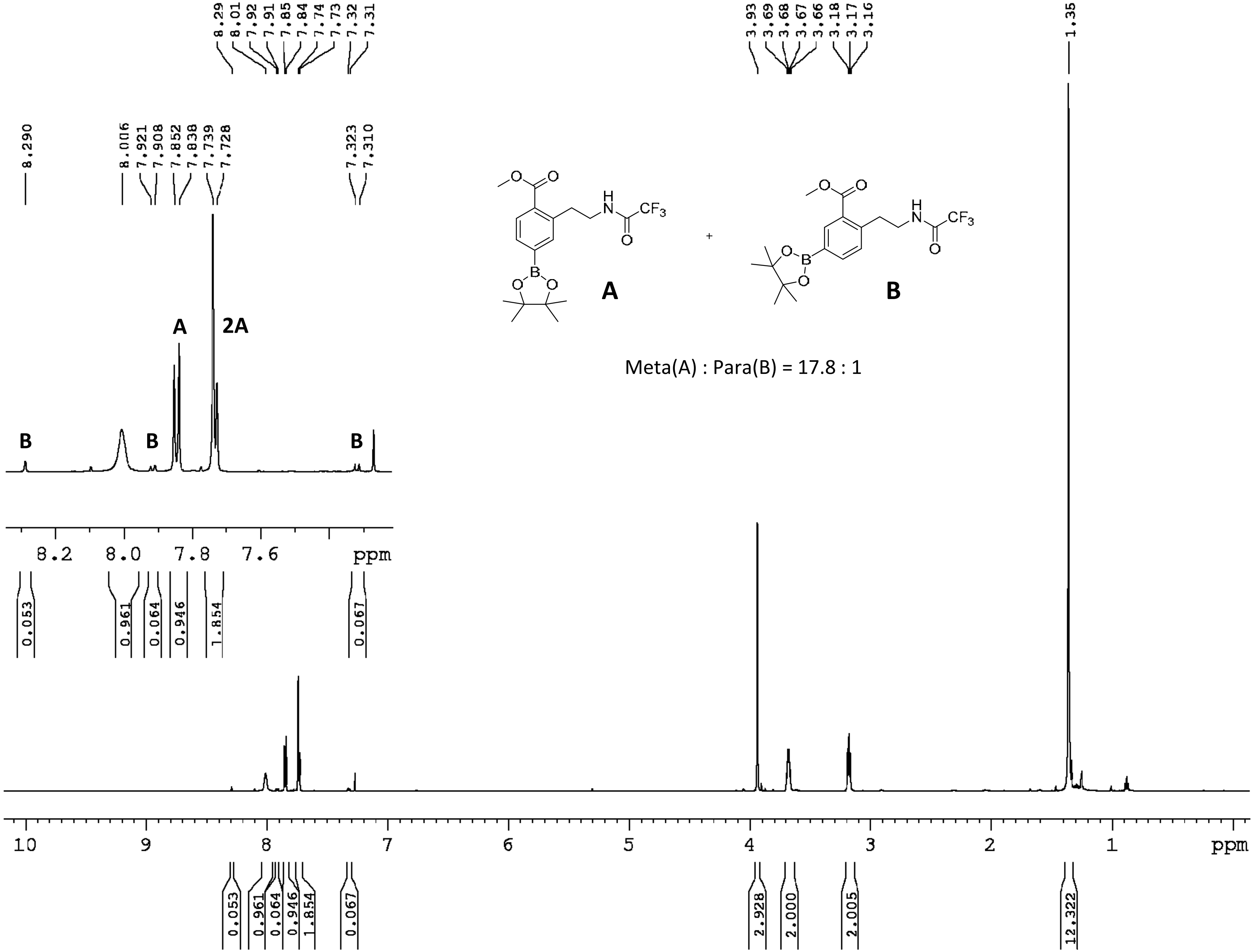

^{13}C NMR (151 MHz, $CDCl_3$) of methyl 4-(4,4,5,5-tetramethyl-1,3,2-dioxaborolan-2-yl)-2-(2-(2,2,2-trifluoroacetamido)ethyl)benzoate (5g) – Ligand 1a – Purified

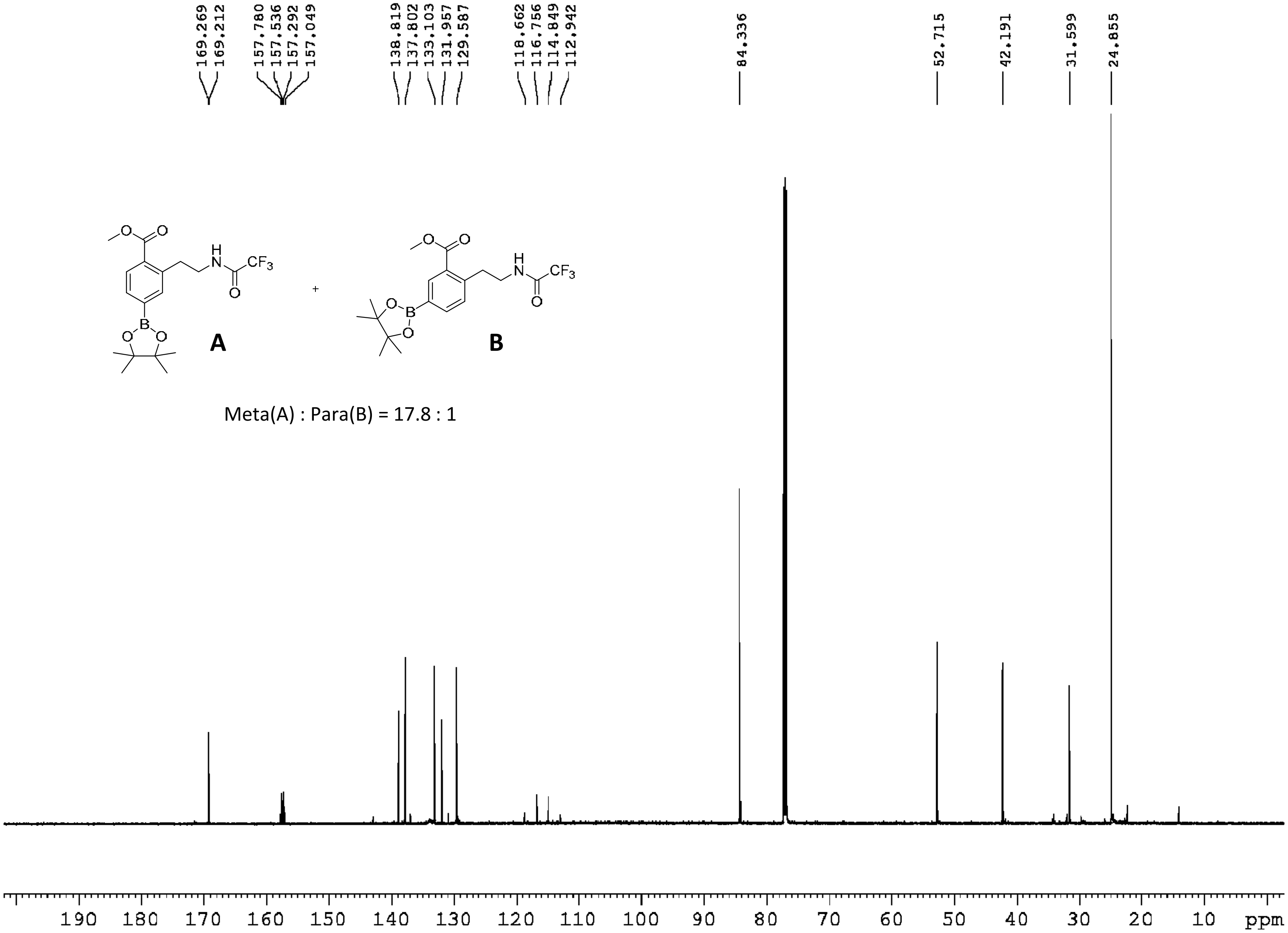

^{1}H NMR (600 MHz, $CDCl_3$) of N-(2-chloro-5-(4,4,5,5-tetramethyl-1,3,2-dioxaborolan-2-yl)phenethyl)-2,2,2-trifluoroacetamide (5h) – Dtbpy – Purified

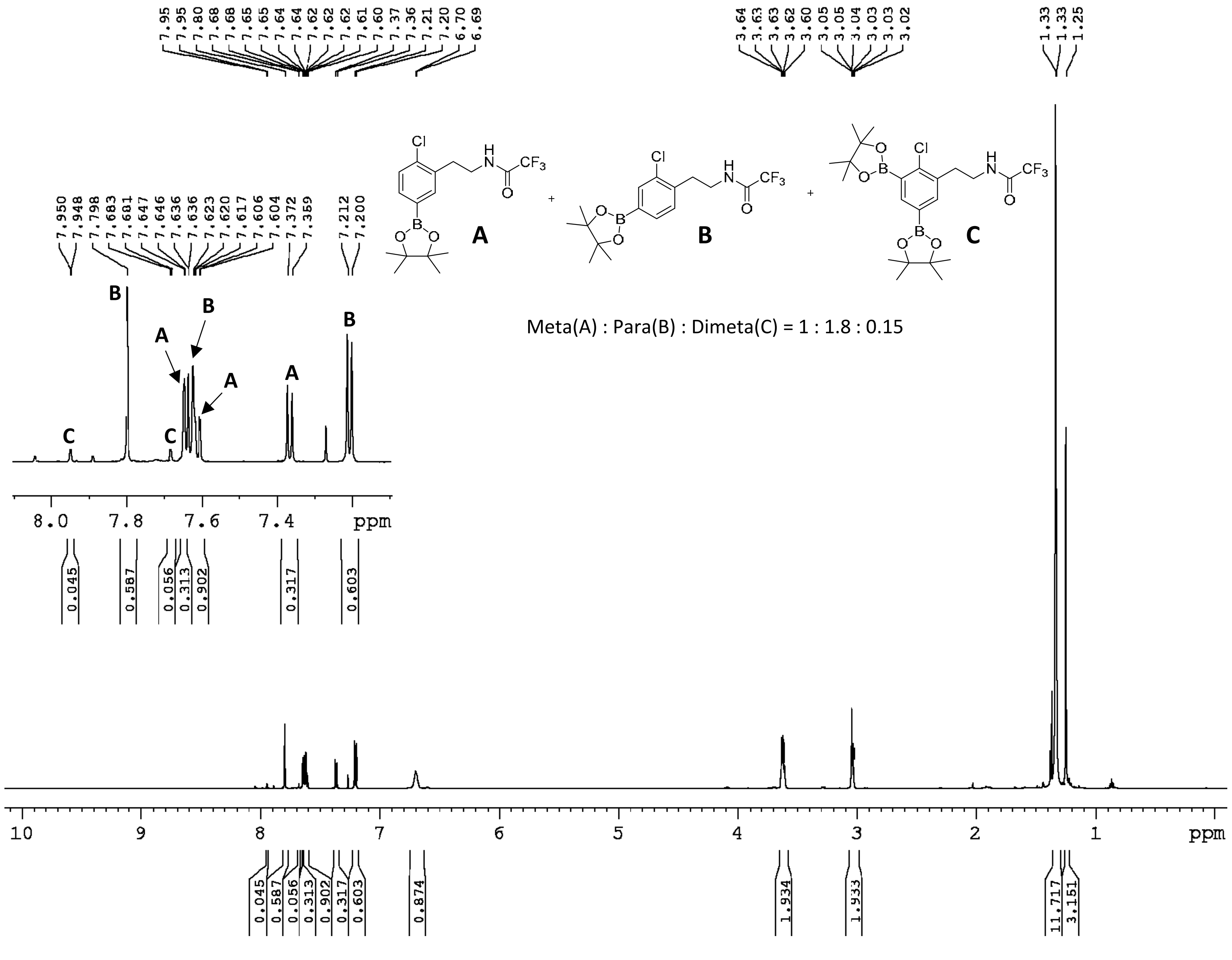

^{13}C NMR (151 MHz, $CDCl_3$) of N-(2-chloro-5-(4,4,5,5-tetramethyl-1,3,2-dioxaborolan-2-yl)phenethyl)-2,2,2-trifluoroacetamide (5h) – Dtbpy – Purified

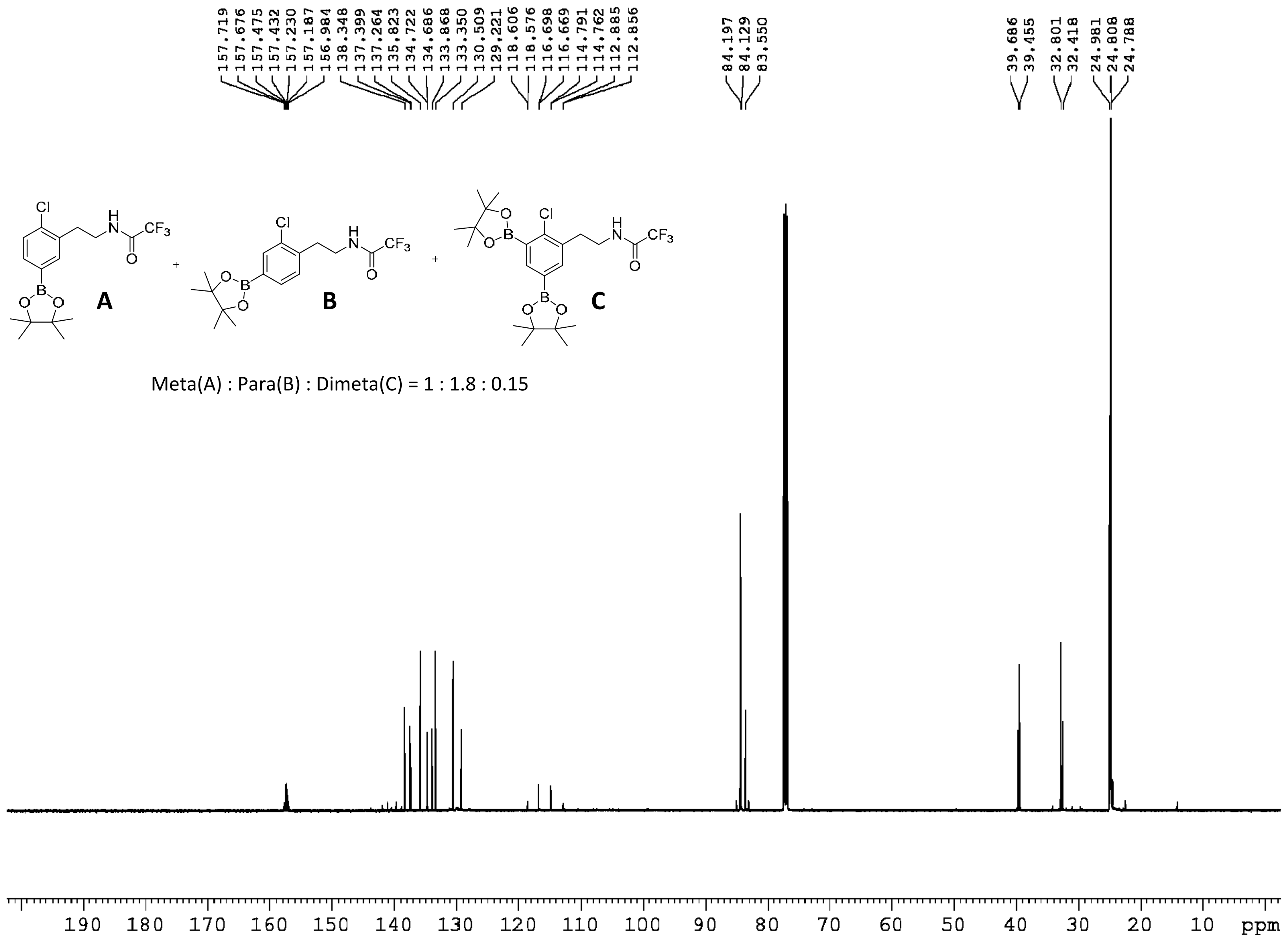

^{1}H NMR (600 MHz, $CDCl_3$) of N-(2-chloro-5-(4,4,5,5-tetramethyl-1,3,2-dioxaborolan-2-yl)phenethyl)-2,2,2-trifluoroacetamide (5h) – Ligand 1a – Crude mixture (X* signifies a product which has also undergone NH borylation)

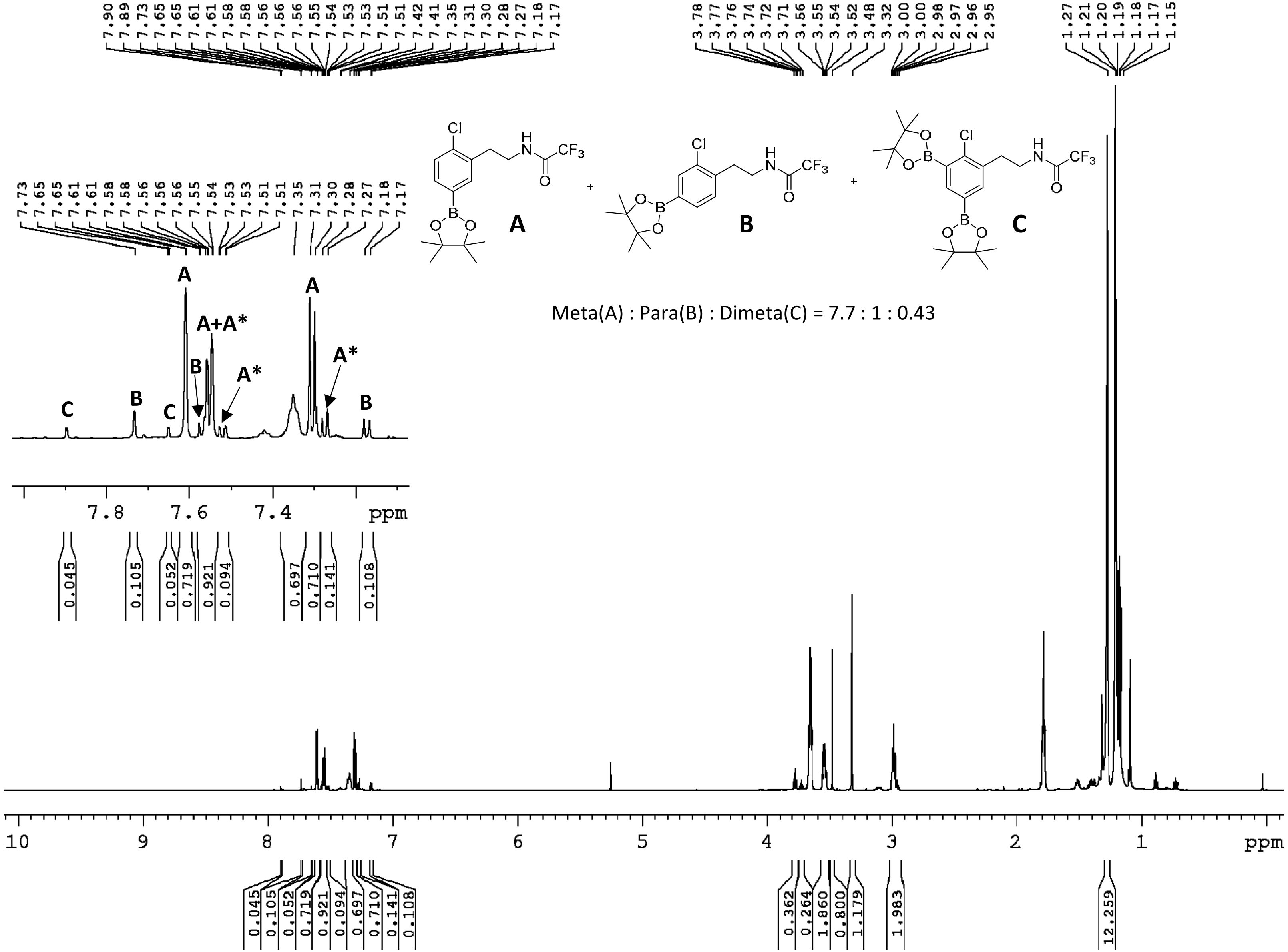

^{1}H NMR (600 MHz, $CDCl_3$) of N-(2-chloro-5-(4,4,5,5-tetramethyl-1,3,2-dioxaborolan-2-yl)phenethyl)-2,2,2-trifluoroacetamide (5h) – Ligand 1a – Purified

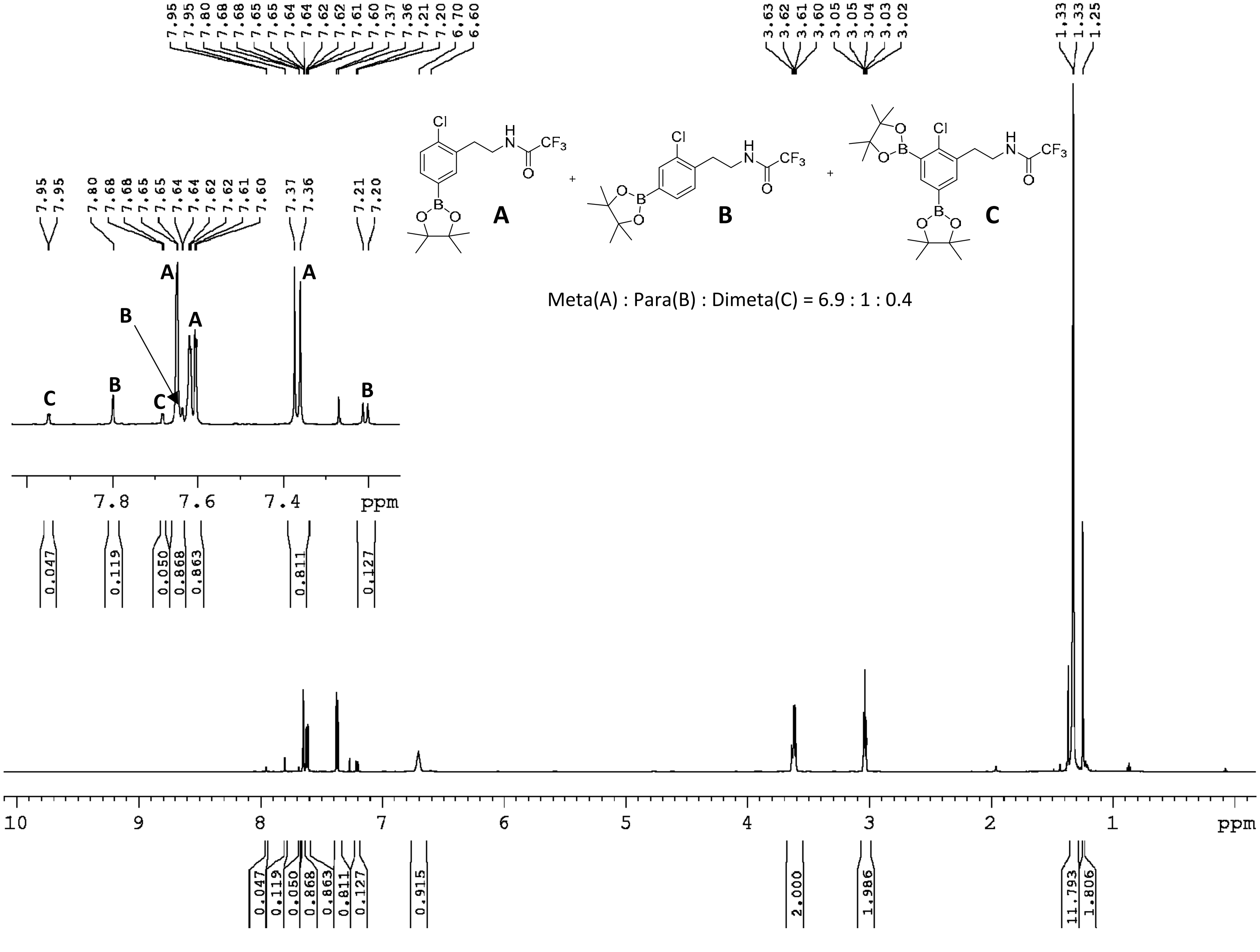

^{13}C NMR (151 MHz, $CDCl_3$) of N-(2-chloro-5-(4,4,5,5-tetramethyl-1,3,2-dioxaborolan-2-yl)phenethyl)-2,2,2-trifluoroacetamide (5h) – Ligand 1a – Purified

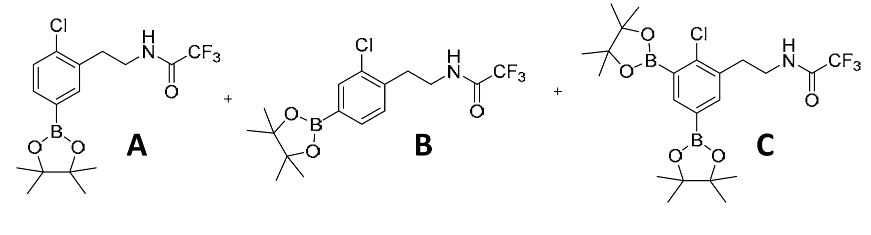

Meta(A) : Para(B) : Dimeta(C) = 6.9 : 1 : 0.4

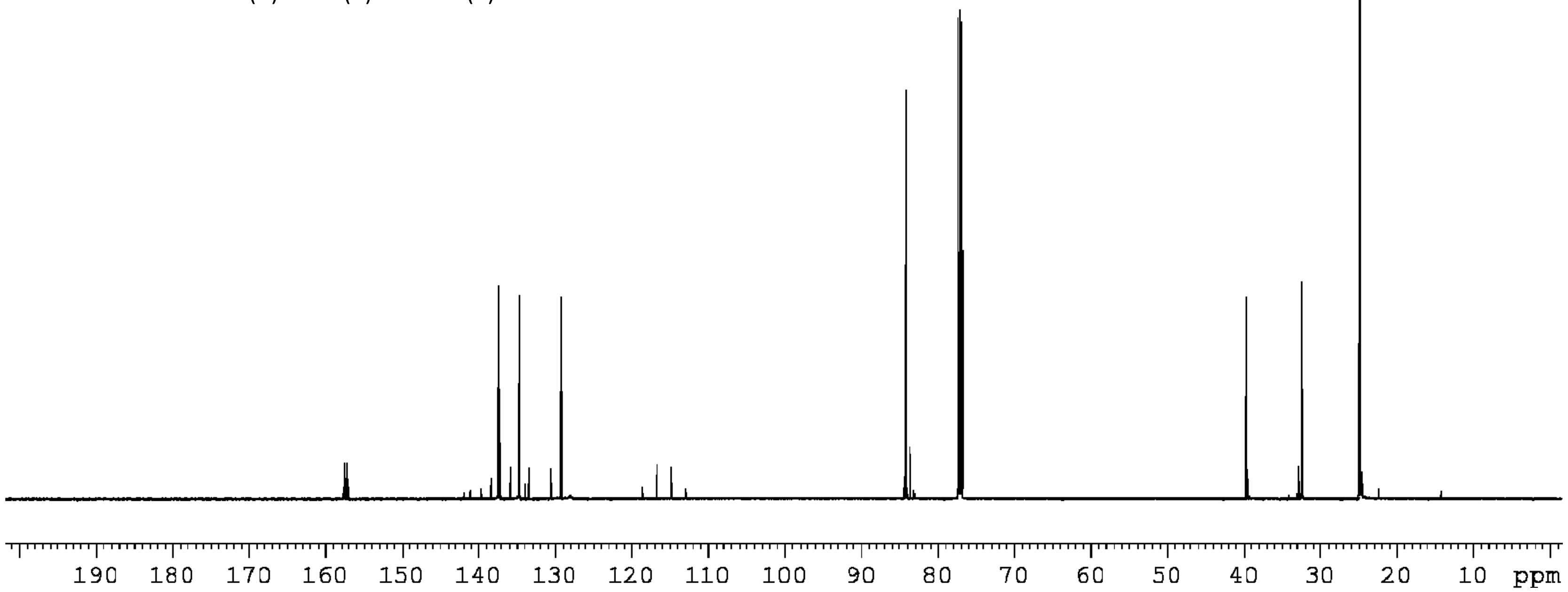

^{1}H NMR (600 MHz, $CDCl_3$) of 2,2,2-trifluoro-N-(2-methyl-5-(4,4,5,5-tetramethyl-1,3,2-dioxaborolan-2-yl)phenethyl)acetamide (5i) – Dtbpy – Purified

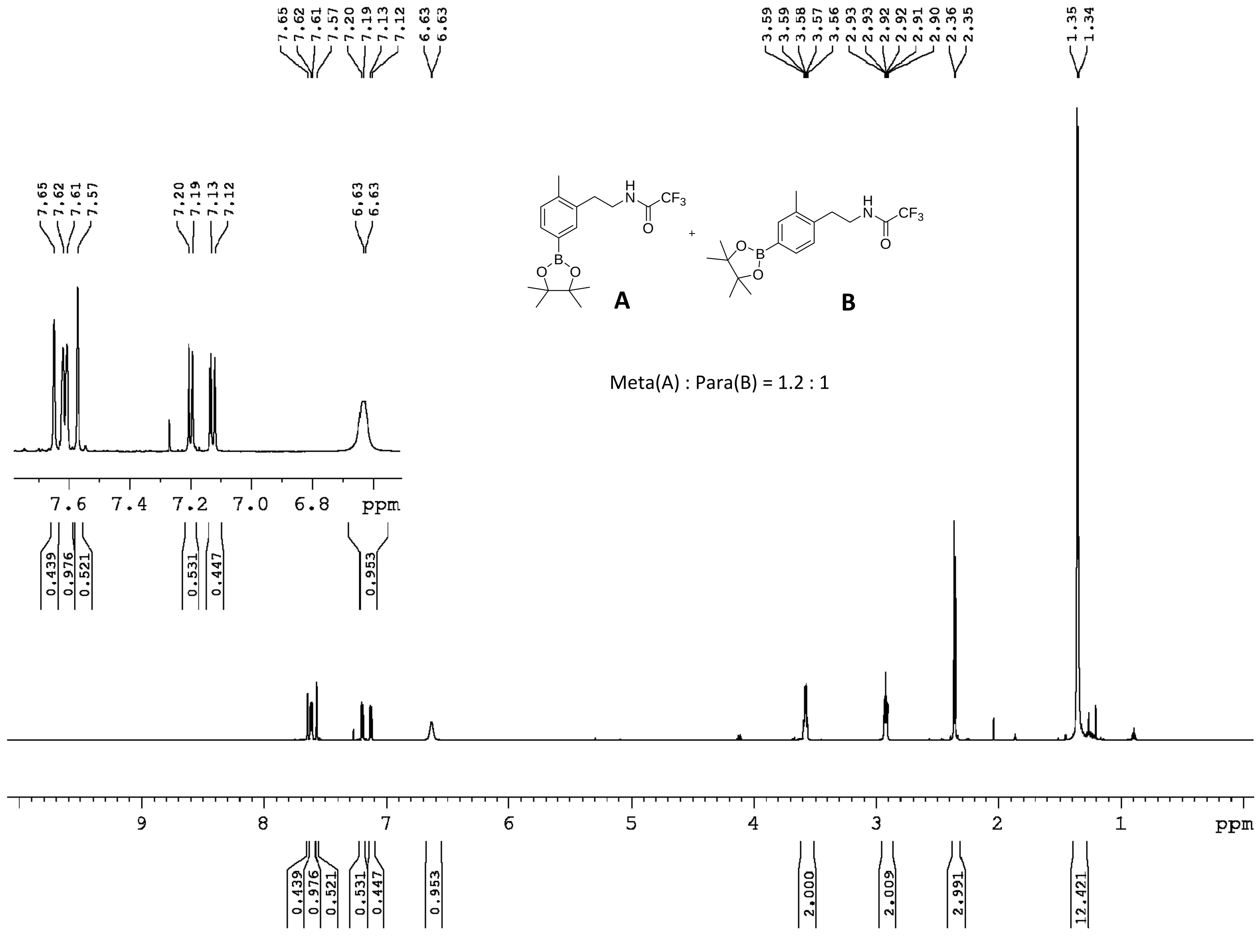

^{13}C NMR (151 MHz, $CDCl_3$) of 2,2,2-trifluoro-N-(2-methyl-5-(4,4,5,5-tetramethyl-1,3,2-dioxaborolan-2-yl)phenethyl)acetamide (5i) – Dtbpy – Purified

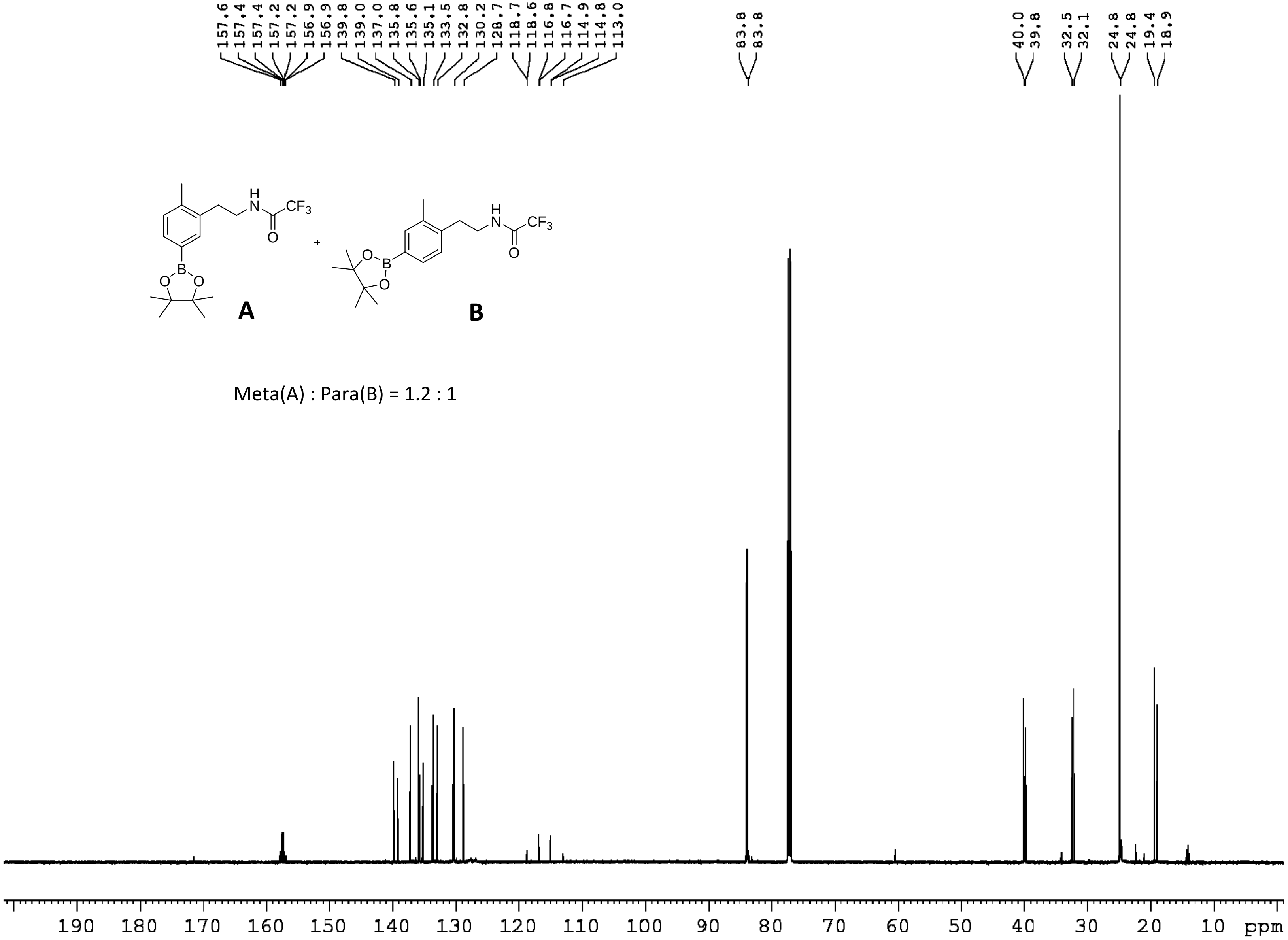

^{1}H NMR (500 MHz, $CDCl_3$) of 2,2,2-trifluoro-N-(2-methyl-5-(4,4,5,5-tetramethyl-1,3,2-dioxaborolan-2-yl)phenethyl)acetamide (5i) – Ligand 1a – Crude mixture

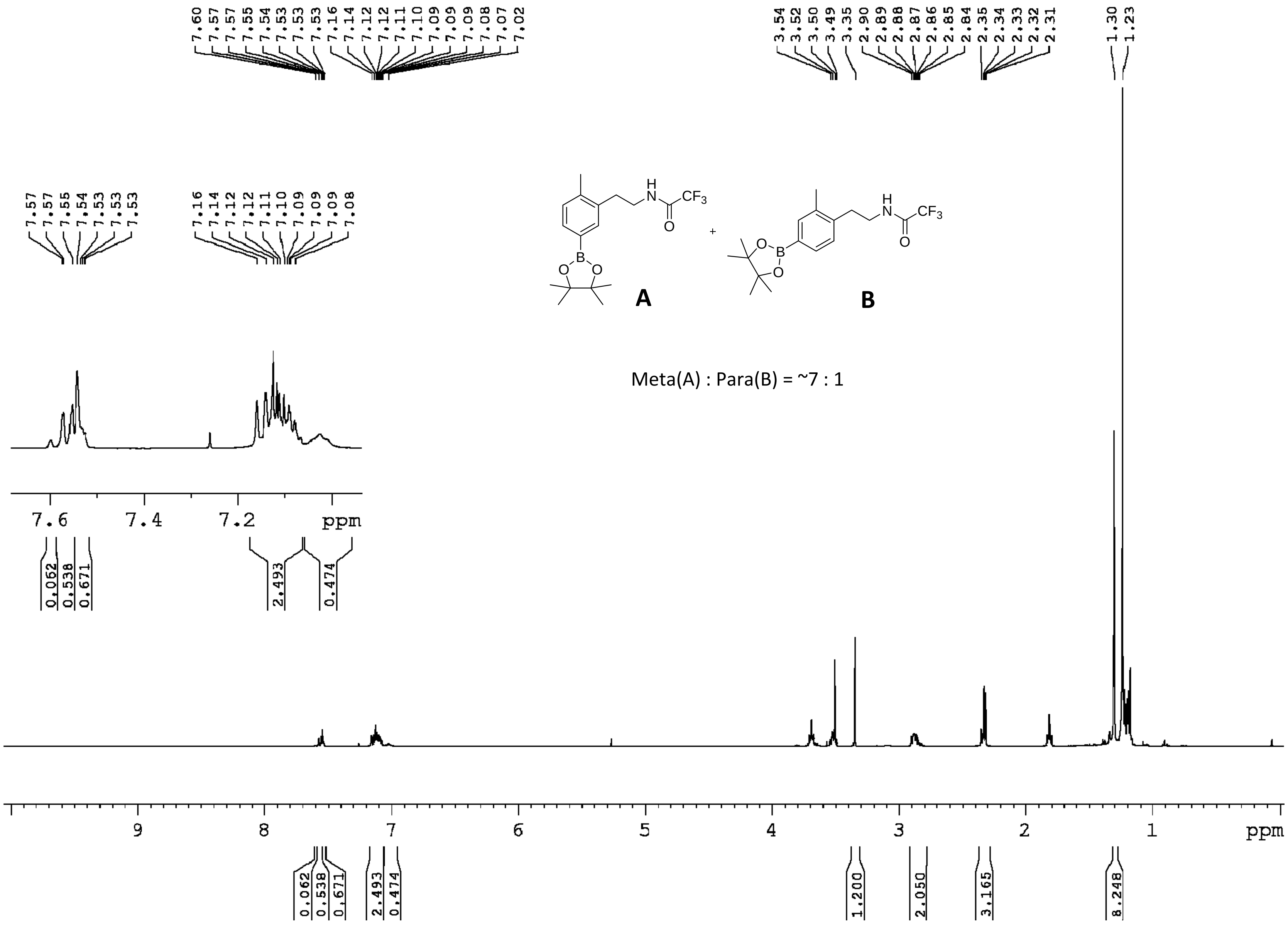

^{1}H NMR (500 MHz, $CDCl_3$) of 2,2,2-trifluoro-N-(2-methyl-5-(4,4,5,5-tetramethyl-1,3,2-dioxaborolan-2-yl)phenethyl)acetamide (5i) – Ligand 1a – Purified

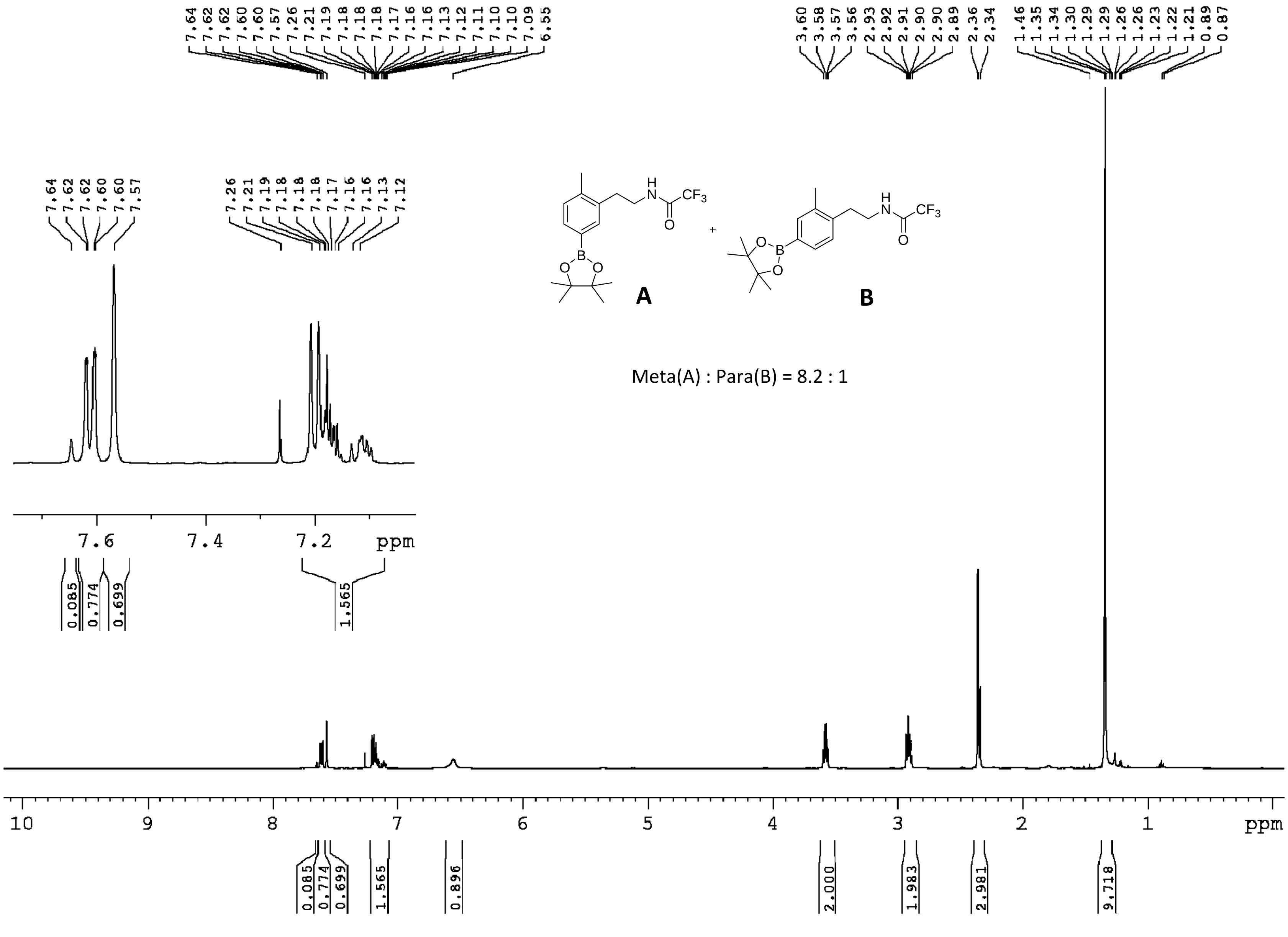

^{13}C NMR (126 MHz, $CDCl_3$) of 2,2,2-trifluoro-N-(2-methyl-5-(4,4,5,5-tetramethyl-1,3,2-dioxaborolan-2-yl)phenethyl)acetamide (5i) – Ligand 1a – Purified

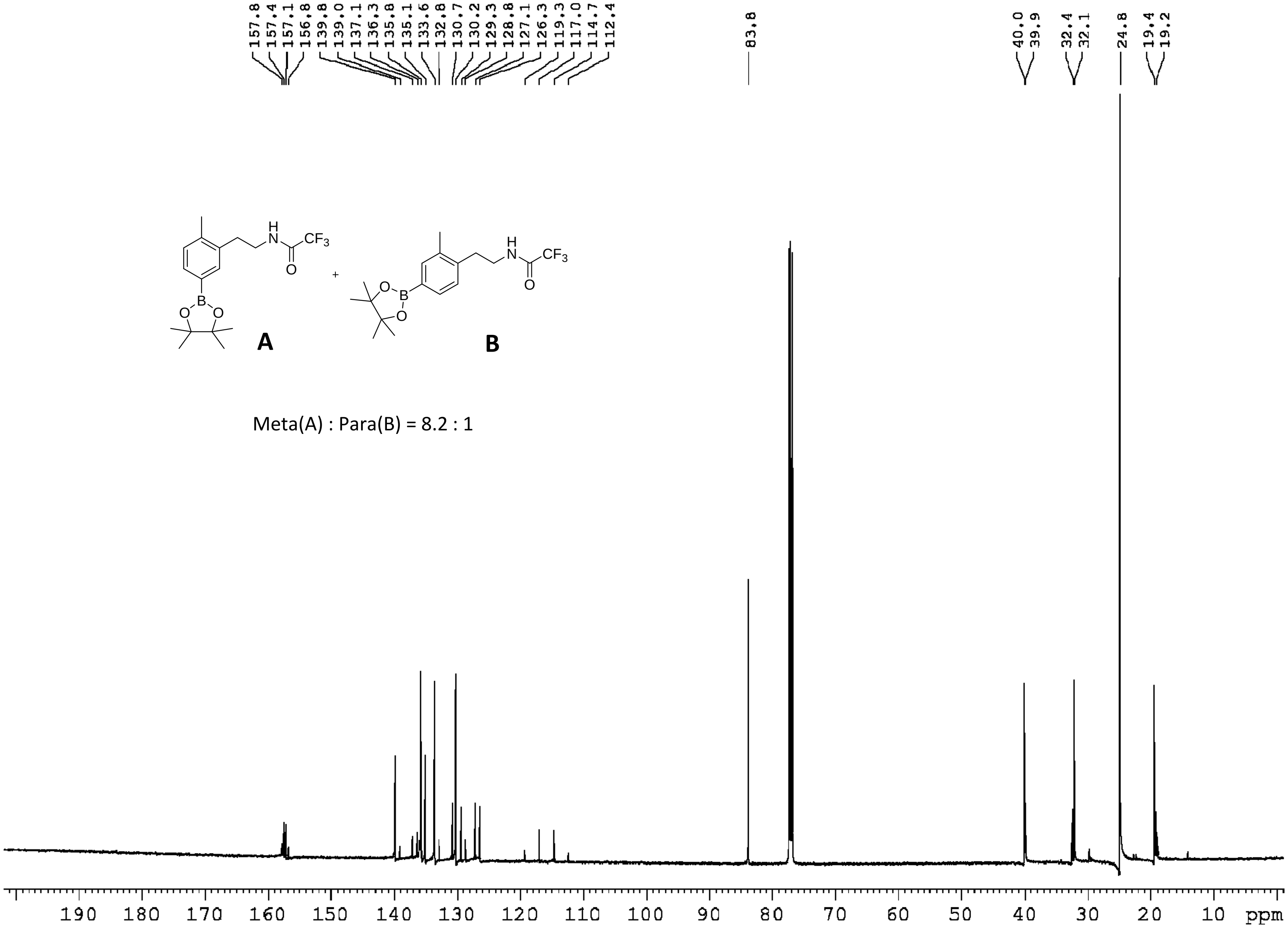

^{1}H NMR (600 MHz, $CDCl_3$) of 2,2,2-trifluoro-N-(2-methoxy-5-(4,4,5,5-tetramethyl-1,3,2-dioxaborolan-2-yl)phenethyl)acetamide (5j) – Dtbpy – Purified

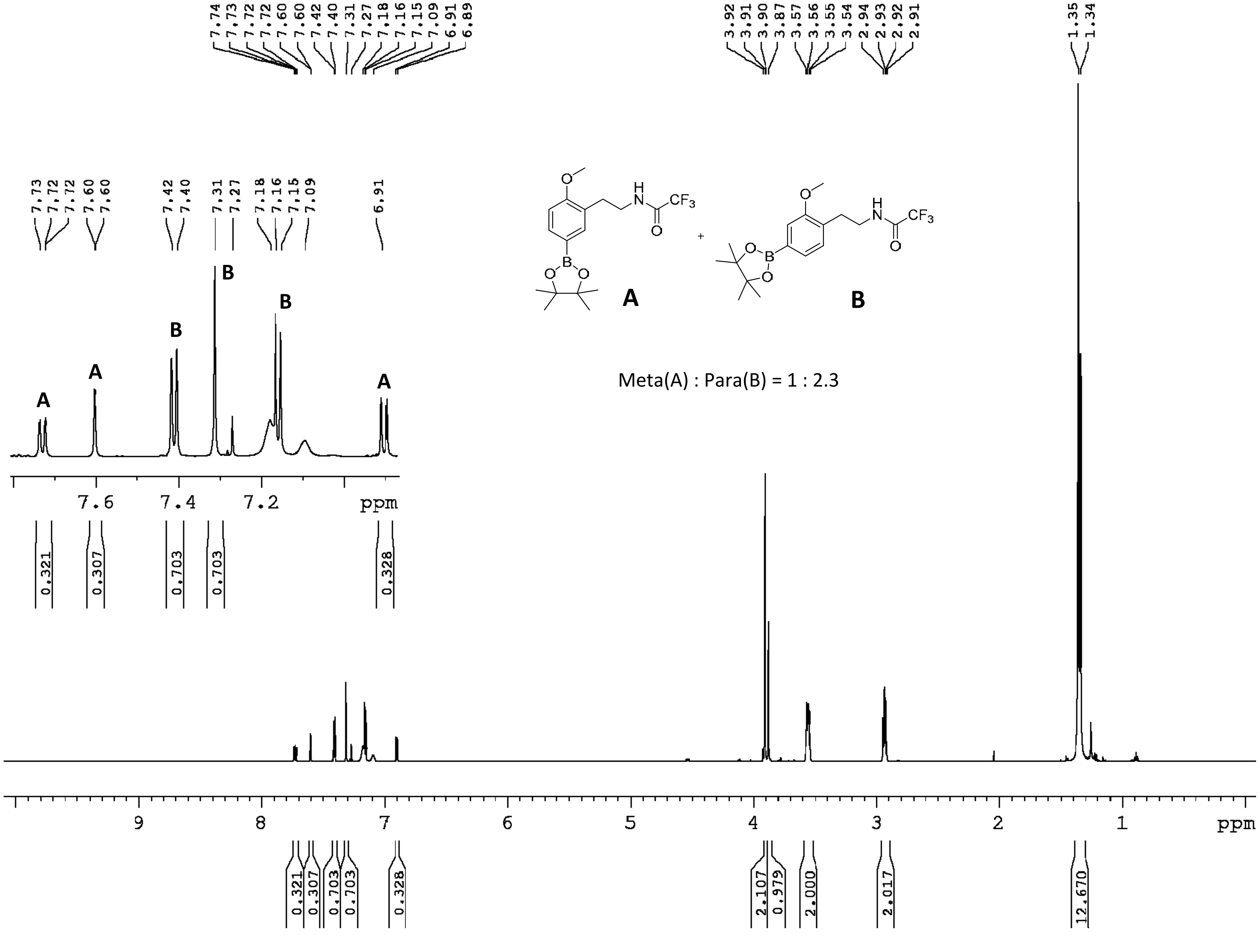

^{13}C NMR (151 MHz, $CDCl_3$) of 2,2,2-trifluoro-N-(2-methoxy-5-(4,4,5,5-tetramethyl-1,3,2-dioxaborolan-2-yl)phenethyl)acetamide (5j) – Dtbpy – Purified

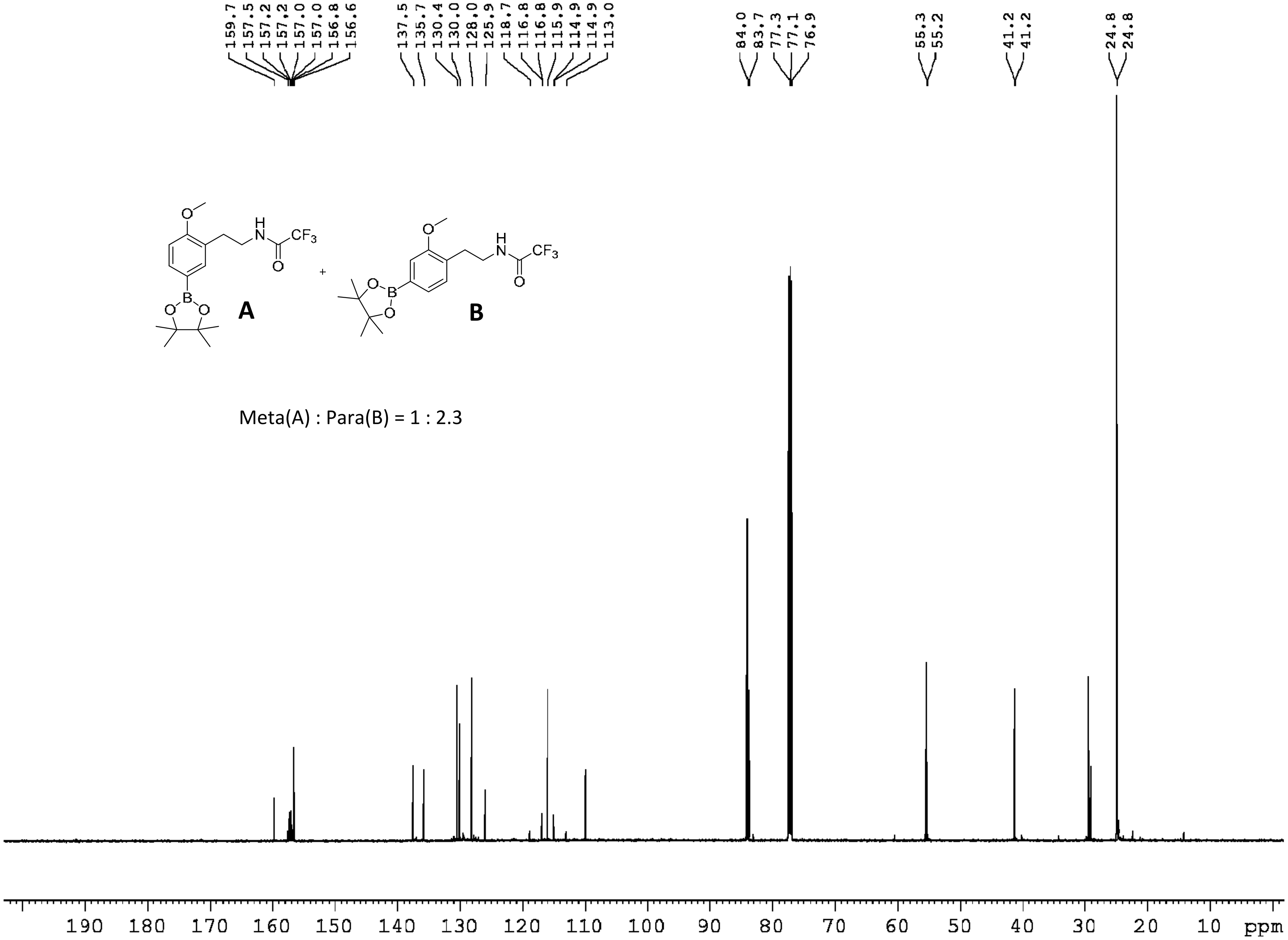

^{1}H NMR (500 MHz, $CDCl_3$) of 2,2,2-trifluoro-N-(2-methoxy-5-(4,4,5,5-tetramethyl-1,3,2-dioxaborolan-2-yl)phenethyl)acetamide (5j) – Ligand 1a – Crude mixture

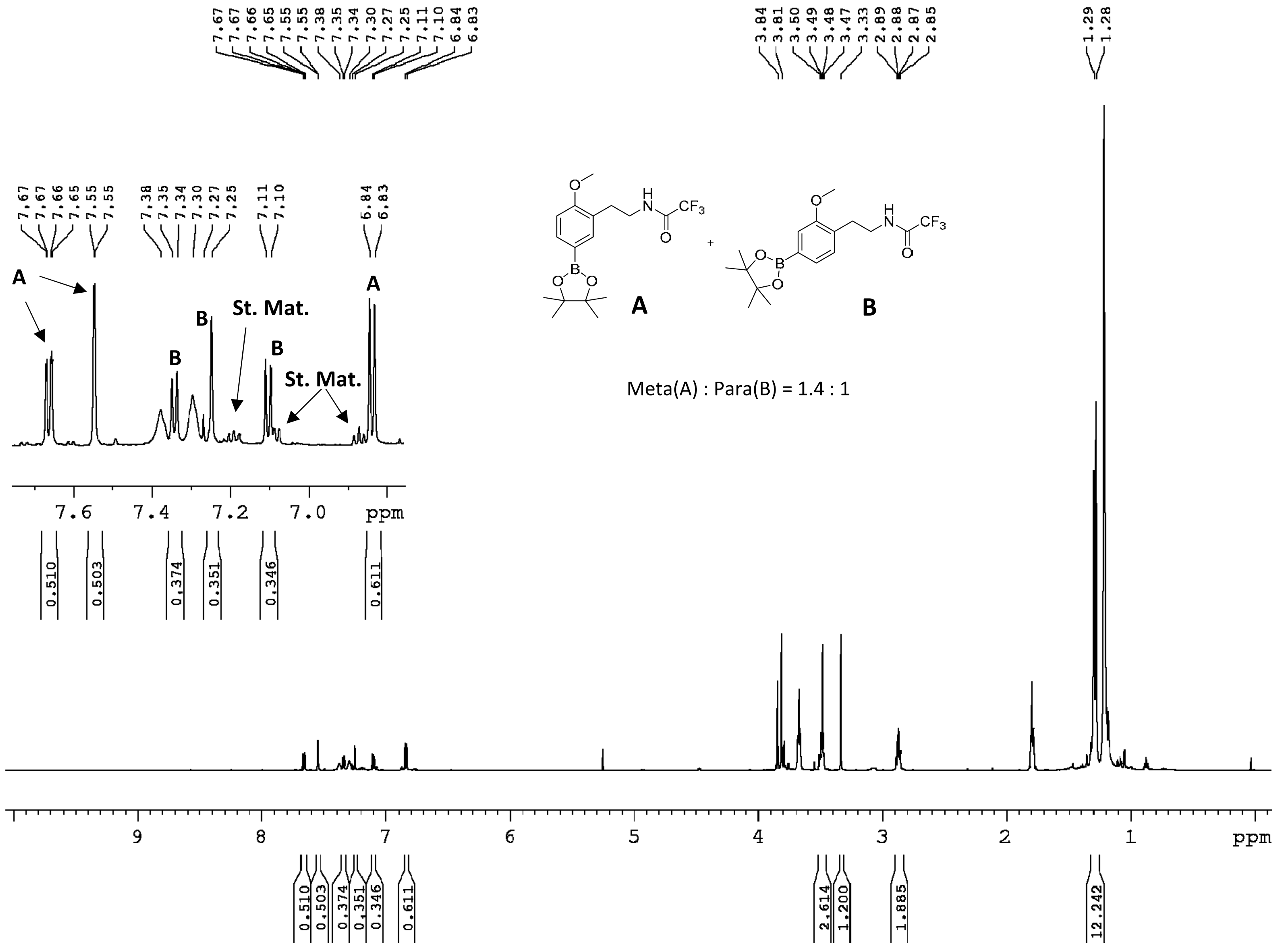

^{1}H NMR (500 MHz, $CDCl_3$) of 2,2,2-trifluoro-N-(2-methoxy-5-(4,4,5,5-tetramethyl-1,3,2-dioxaborolan-2-yl)phenethyl)acetamide (5j) – Ligand 1a – Purified

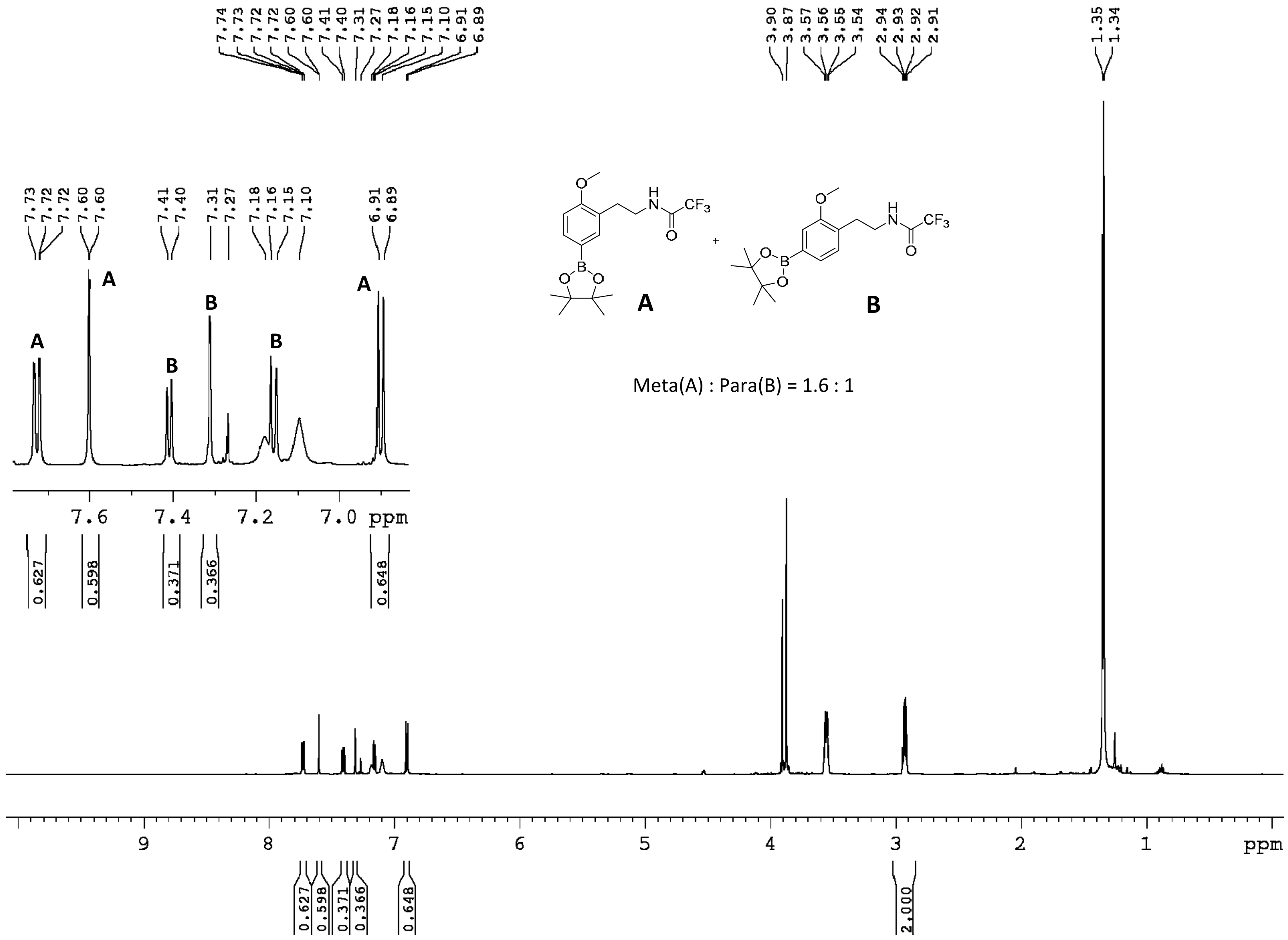

^{13}C NMR (126 MHz, $CDCl_3$) of 2,2,2-trifluoro-N-(2-methoxy-5-(4,4,5,5-tetramethyl-1,3,2-dioxaborolan-2-yl)phenethyl)acetamide (5j) – Ligand 1a – Purified

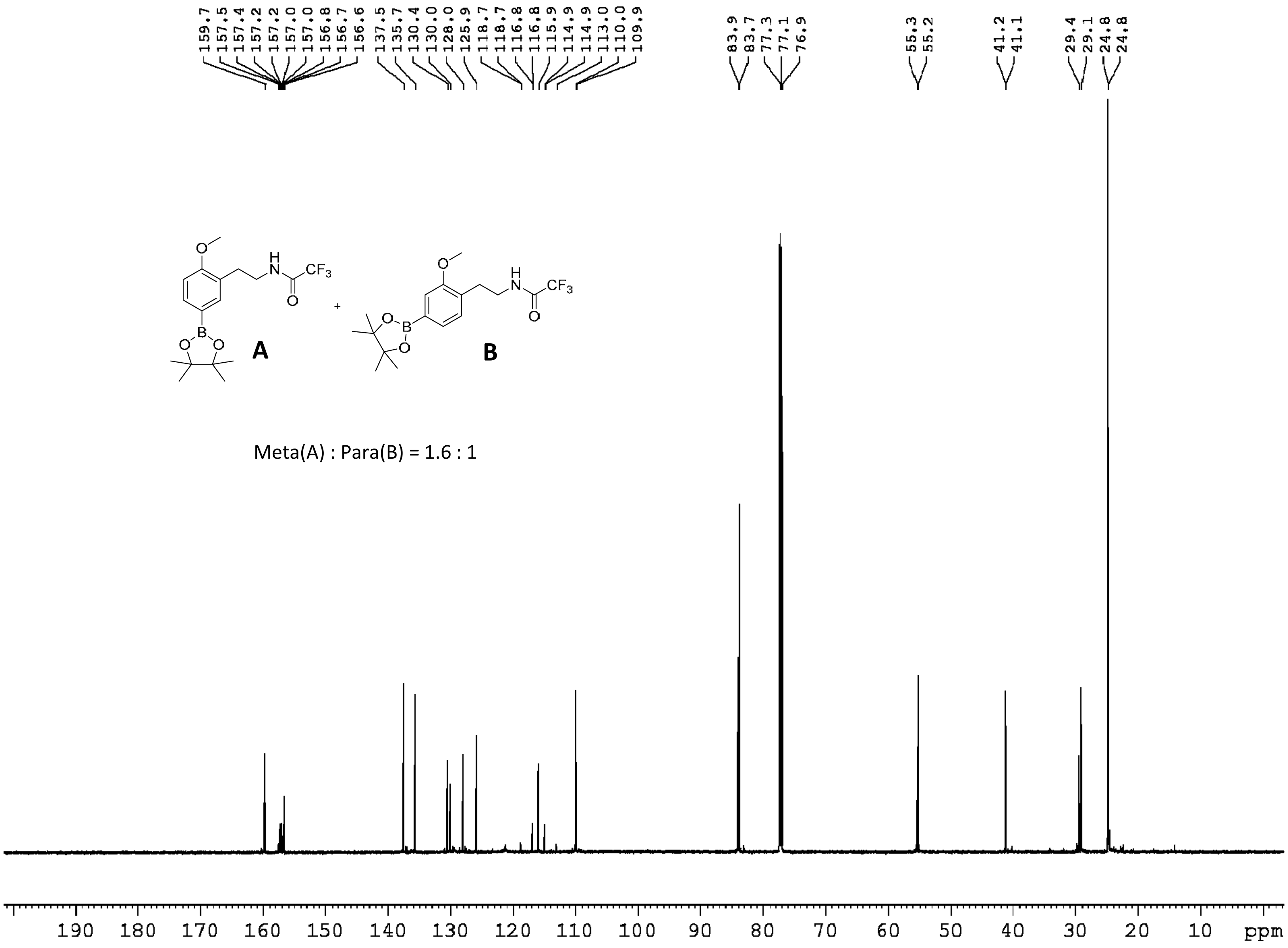

^{1}H NMR (600 MHz, $CDCl_3$) of N-(2-(((tert-butyldimethylsilyl)oxy)methyl)-5-(4,4,5,5-tetramethyl-1,3,2-dioxaborolan-2-yl)phenethyl)-2,2,2-trifluoroacetamide (5k) – Dtbpy – Purified

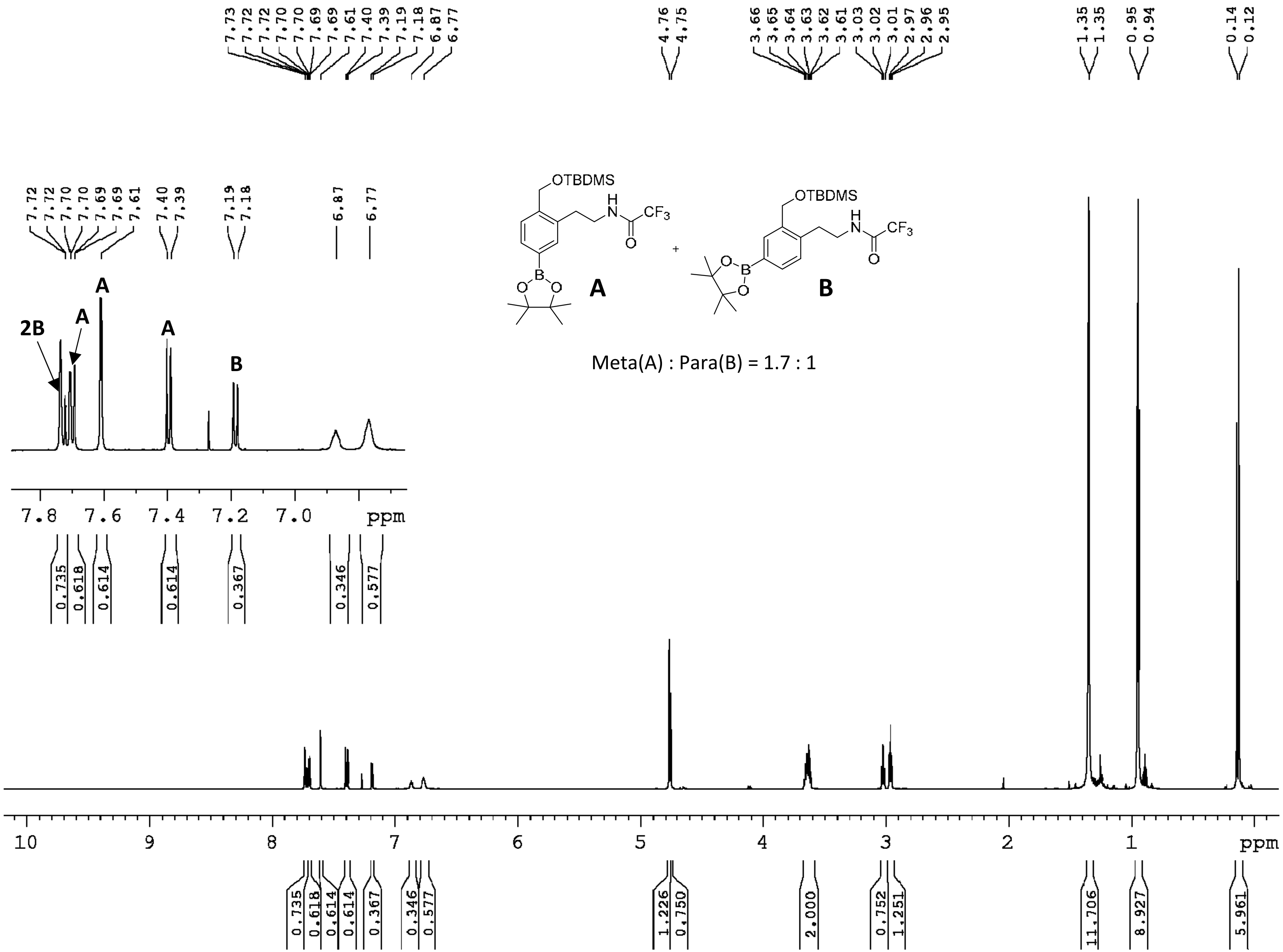

^{13}C (151 MHz, $CDCl_3$) NMR of N-(2-(((tert-butyldimethylsilyl)oxy)methyl)-5-(4,4,5,5-tetramethyl-1,3,2-dioxaborolan-2-yl)phenethyl)-2,2,2-trifluoroacetamide (5k) – Dtbpy – Purified

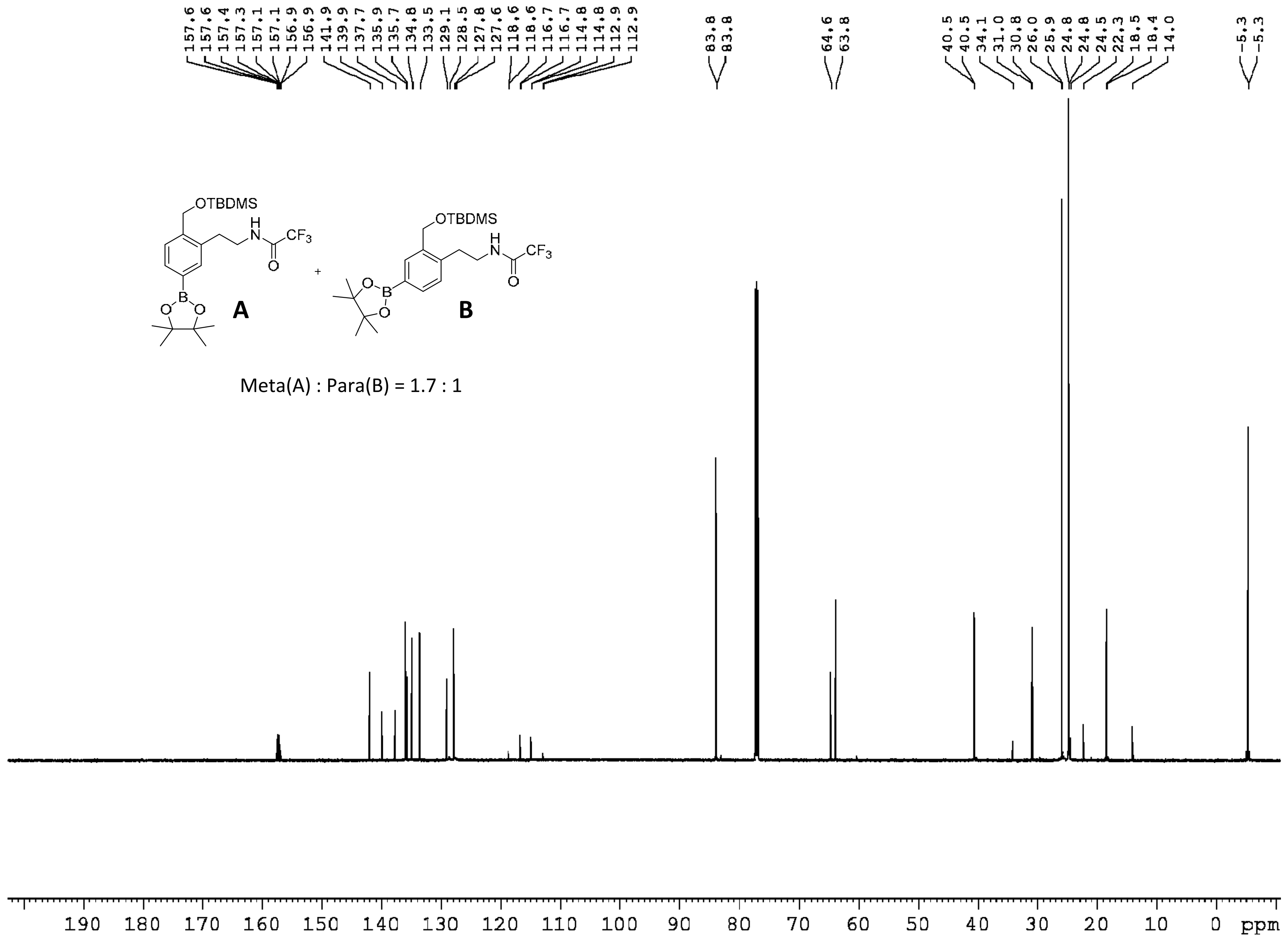

^{1}H NMR (600 MHz, $CDCl_3$) of N-(2-(((tert-butyldimethylsilyl)oxy)methyl)-5-(4,4,5,5-tetramethyl-1,3,2-dioxaborolan-2-yl)phenethyl)-2,2,2-trifluoroacetamide (5k) – Ligand 1a – Crude mixture (X* signifies a product which has also undergone NH borylation)

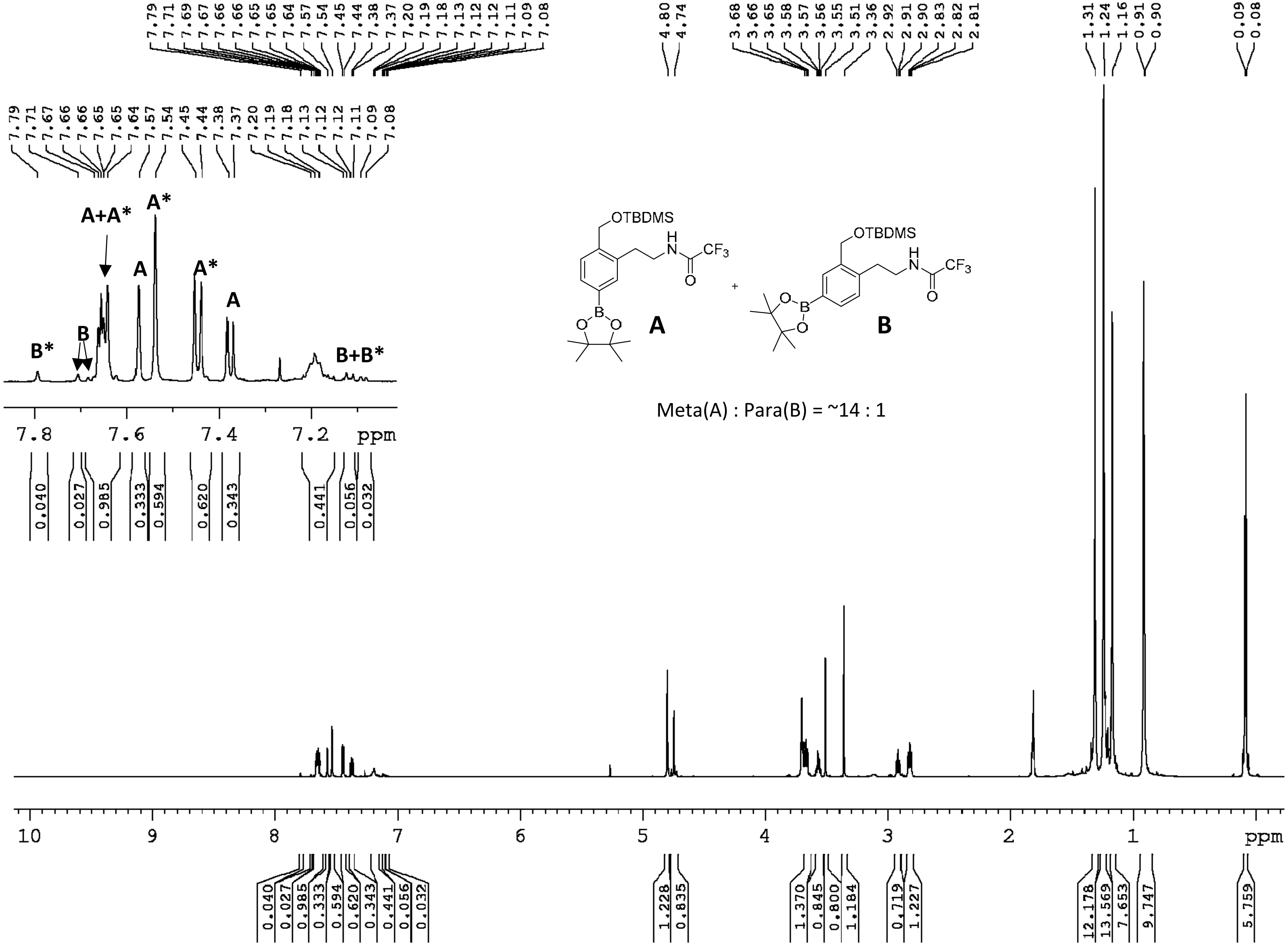

^{1}H NMR (600 MHz, $CDCl_3$) of N-(2-(((tert-butyldimethylsilyl)oxy)methyl)-5-(4,4,5,5-tetramethyl-1,3,2-dioxaborolan-2-yl)phenethyl)-2,2,2-trifluoroacetamide (5k) – Ligand 1a – Purified

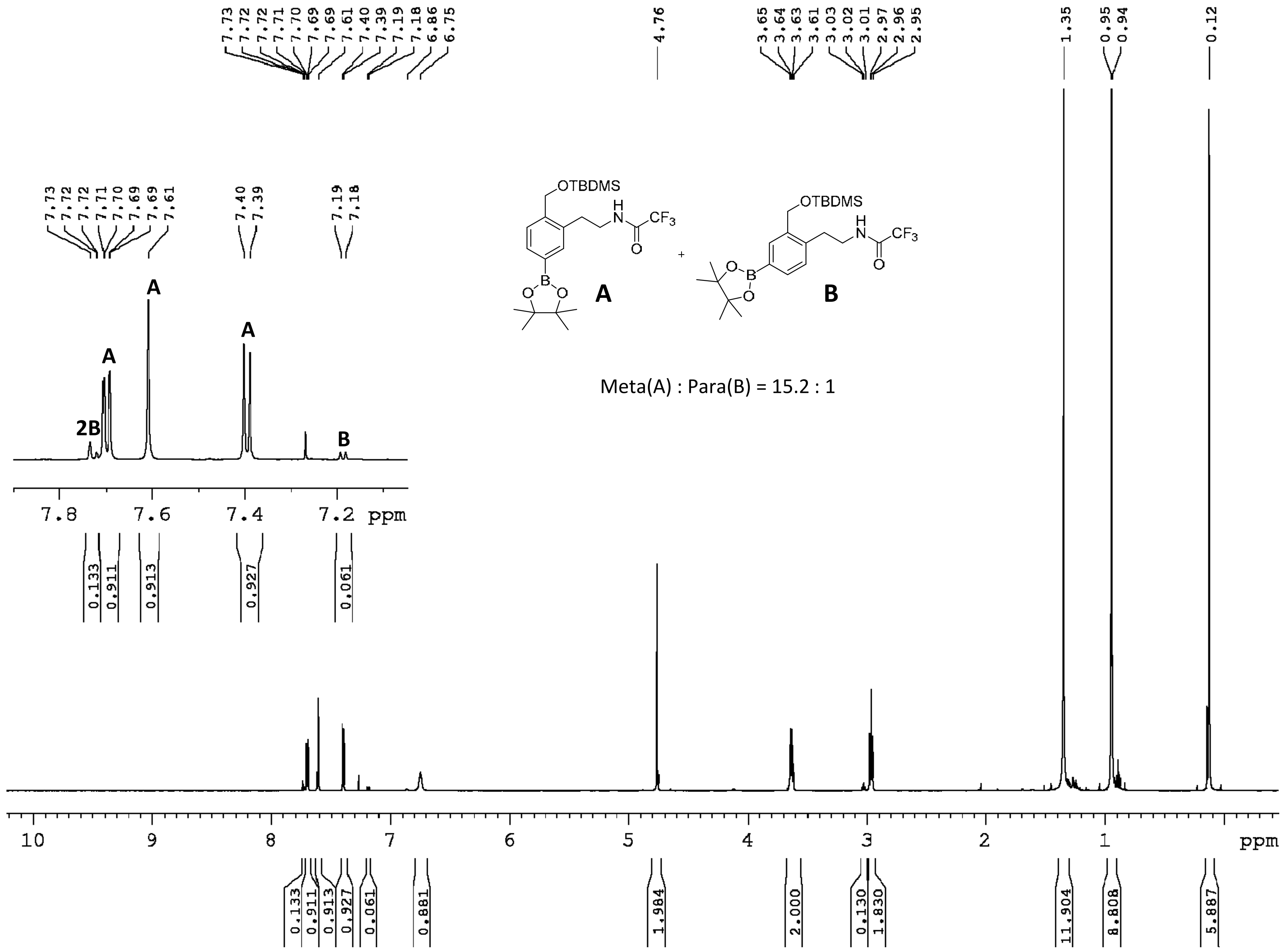

^{13}C NMR (151 MHz, $CDCl_3$) of N-(2-(((tert-butyldimethylsilyl)oxy)methyl)-5-(4,4,5,5-tetramethyl-1,3,2-dioxaborolan-2-yl)phenethyl)-2,2,2-trifluoroacetamide (5k) – Ligand 1a – Purified

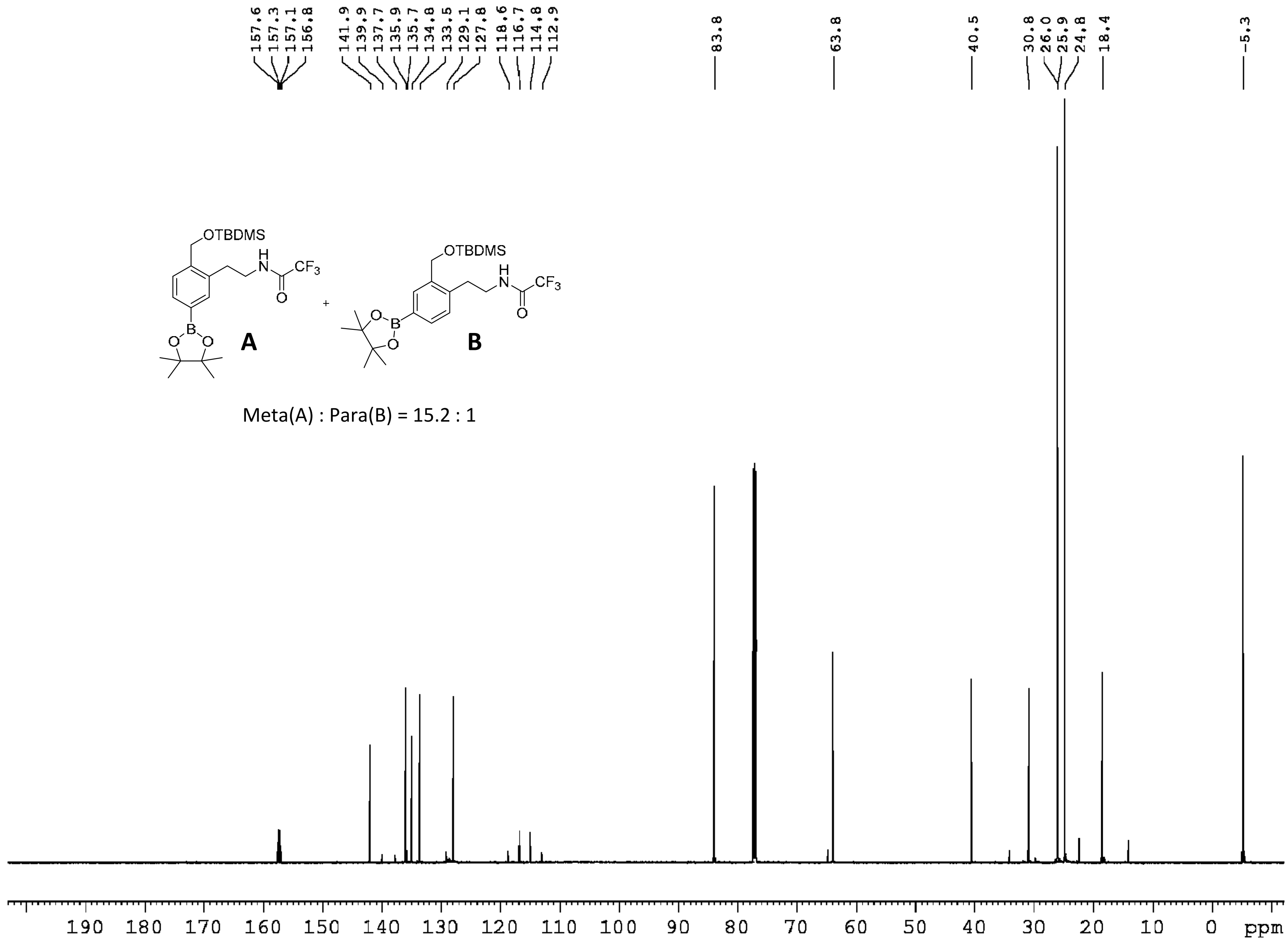

^{1}H NMR (600 MHz, $CDCl_3$) of N-(3,5-bis(4,4,5,5-tetramethyl-1,3,2-dioxaborolan-2-yl)phenethyl)-2,2,2-trifluoroacetamide (5l) – Dtbpy – Purified

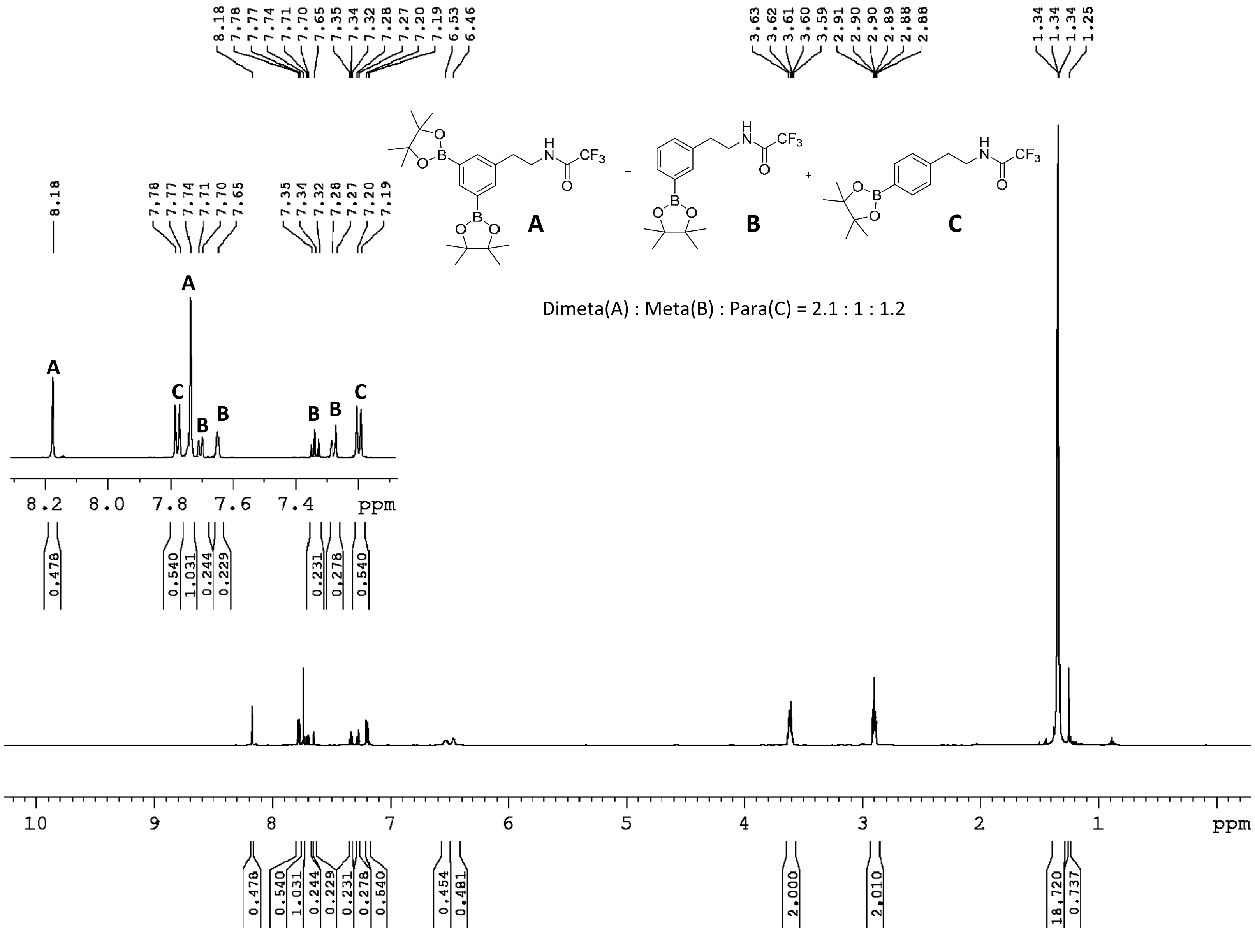

^{13}C NMR (151 MHz, $CDCl_3$) of N-(3,5-bis(4,4,5,5-tetramethyl-1,3,2-dioxaborolan-2-yl)phenethyl)-2,2,2-trifluoroacetamide (5l) – Dtbpy – Purified

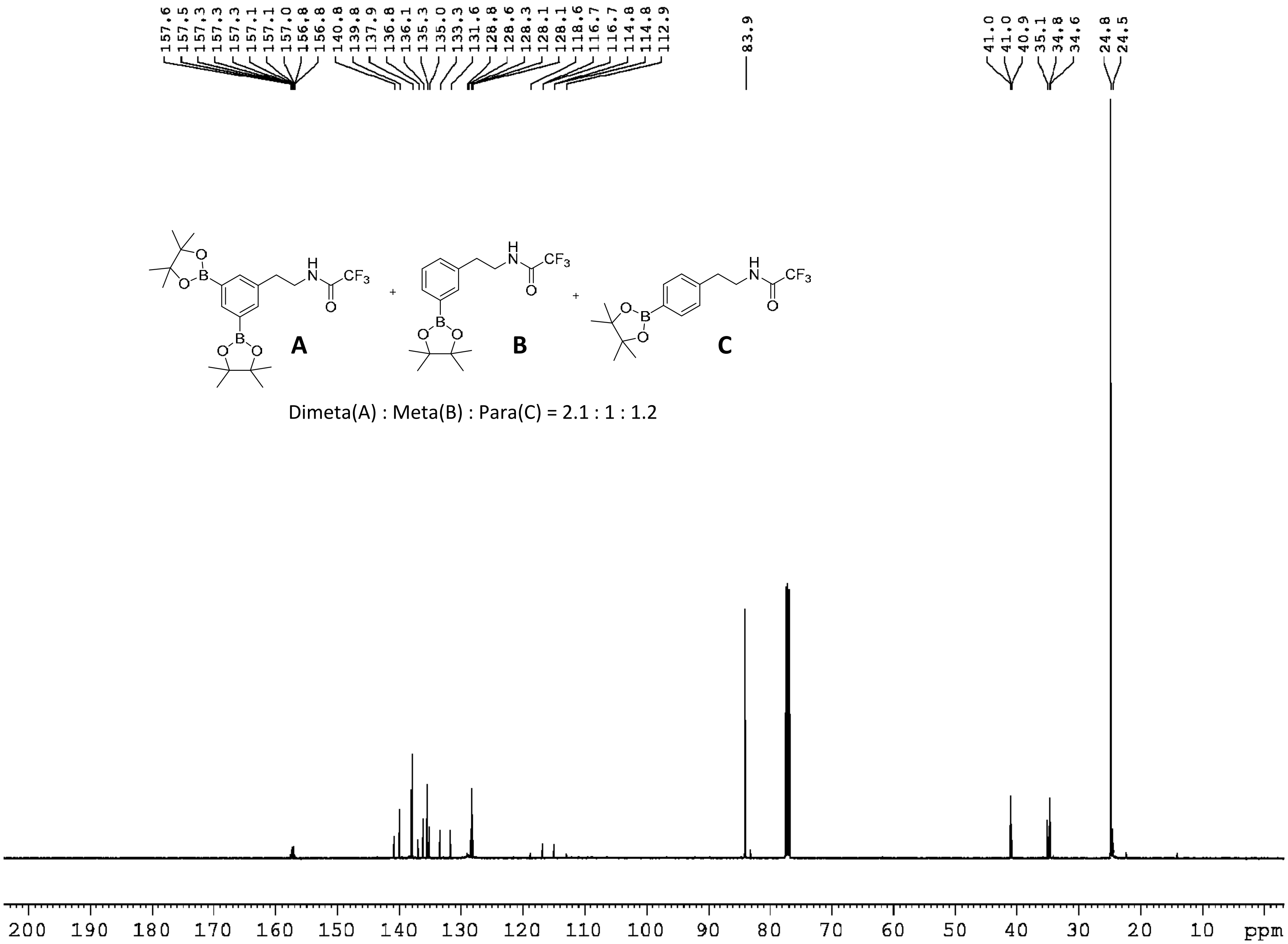

^{1}H NMR (600 MHz, $CDCl_3$) of N-(3,5-bis(4,4,5,5-tetramethyl-1,3,2-dioxaborolan-2-yl)phenethyl)-2,2,2-trifluoroacetamide (5l) – Ligand 1a – Crude mixture

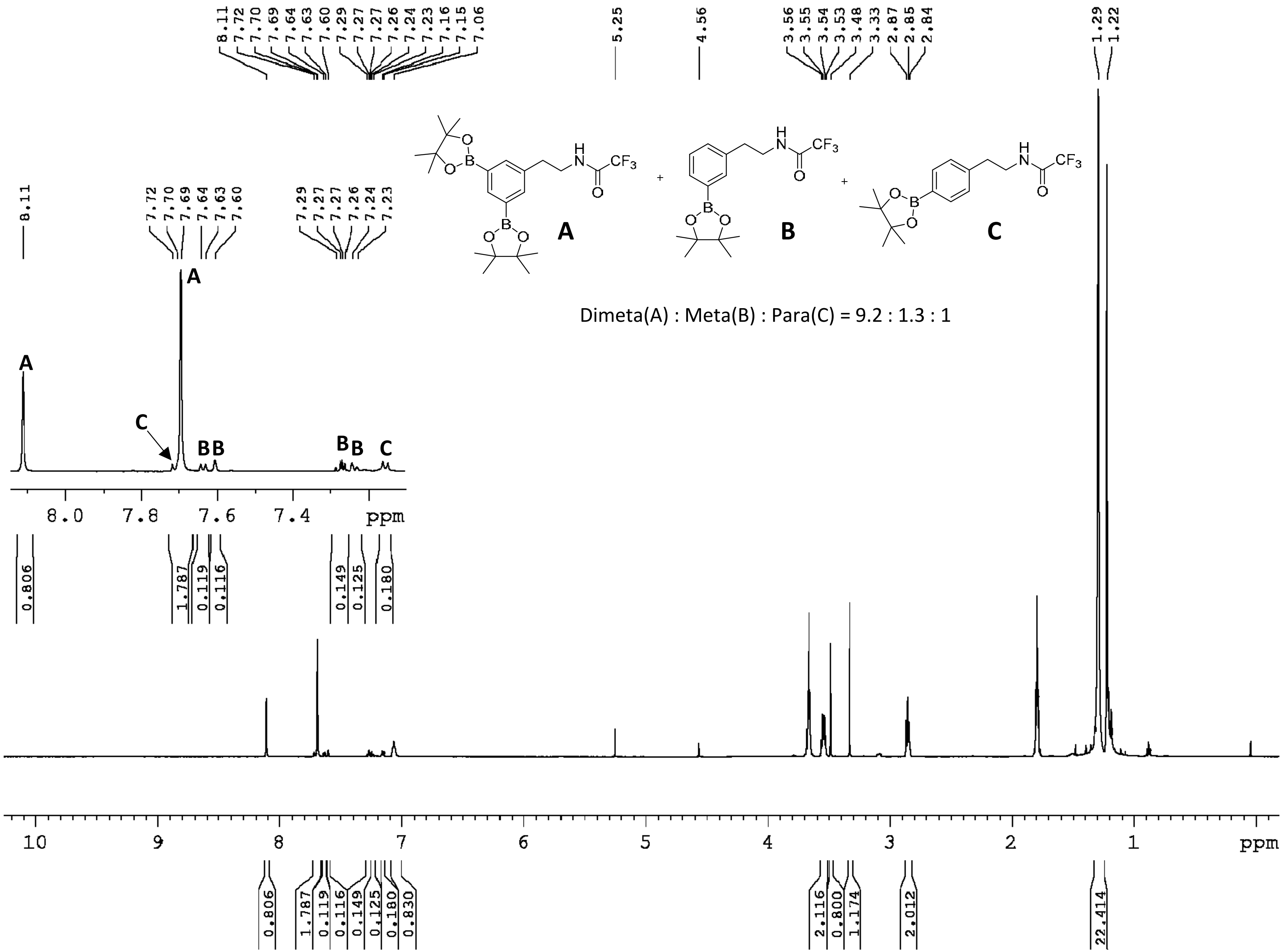

^{1}H NMR (600 MHz, $CDCl_3$) of N-(3,5-bis(4,4,5,5-tetramethyl-1,3,2-dioxaborolan-2-yl)phenethyl)-2,2,2-trifluoroacetamide (5l) – Ligand 1a – Purified

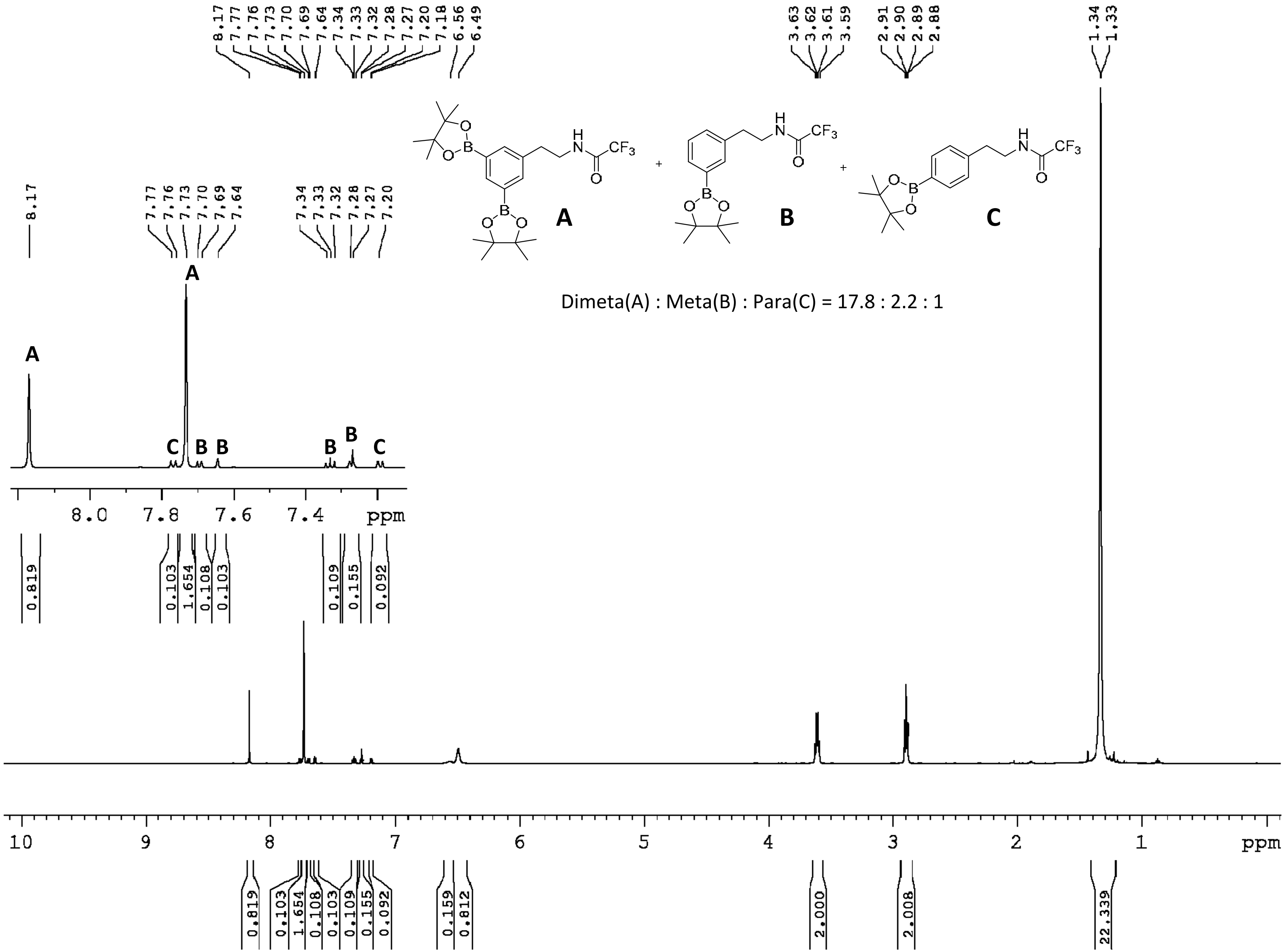

^{13}C NMR (151 MHz, $CDCl_3$) of N-(3,5-bis(4,4,5,5-tetramethyl-1,3,2-dioxaborolan-2-yl)phenethyl)-2,2,2-trifluoroacetamide (5l) – Ligand 1a – Purified

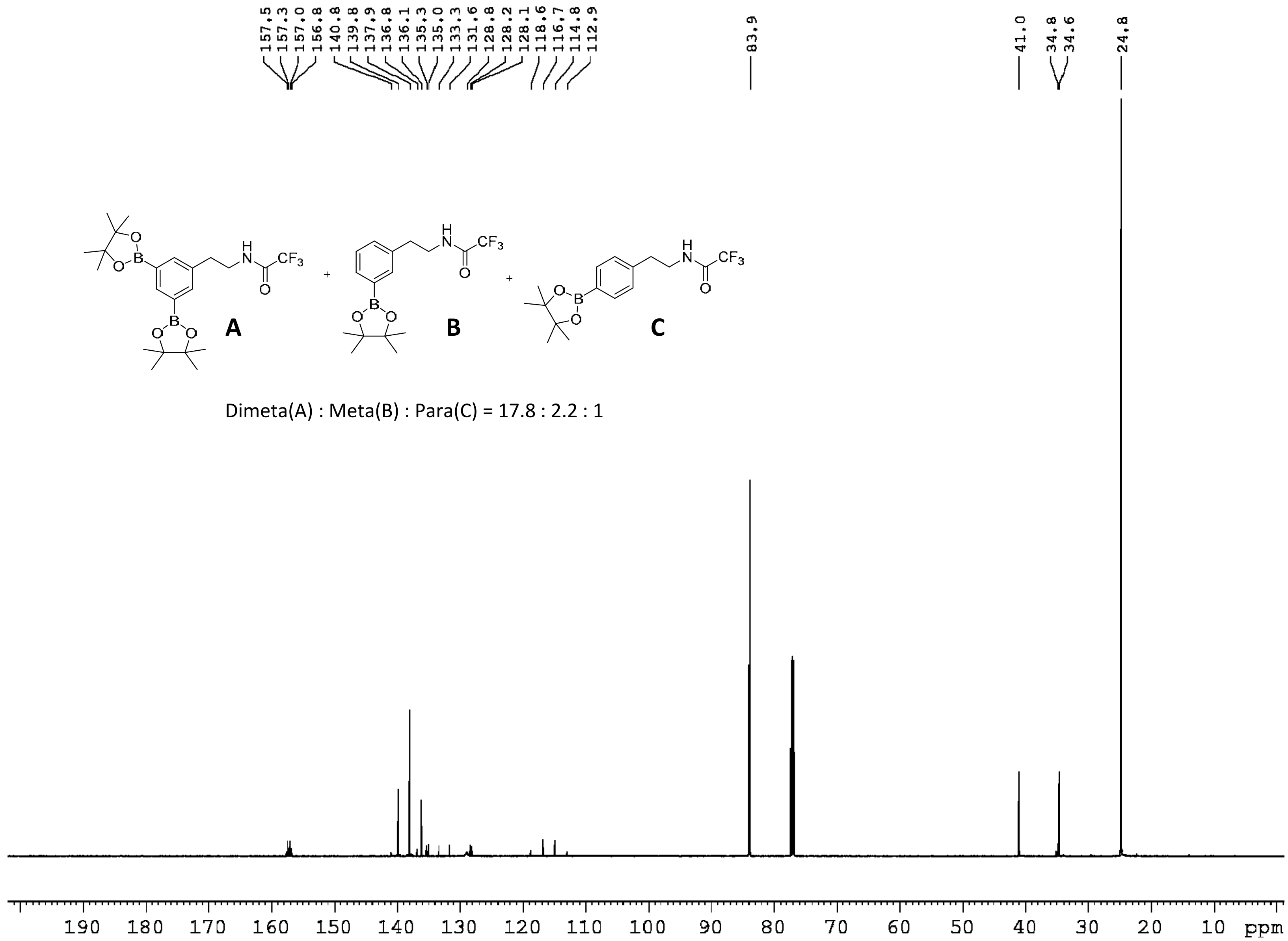

^{1}H NMR (500 MHz, $CDCl_3$) of 2,2,2-trifluoro-N-(2-(4-(4,4,5,5-tetramethyl-1,3,2-dioxaborolan-2-yl)pyridin-2-yl)ethyl)acetamide (5m) – Dtbpy – Crude mixture

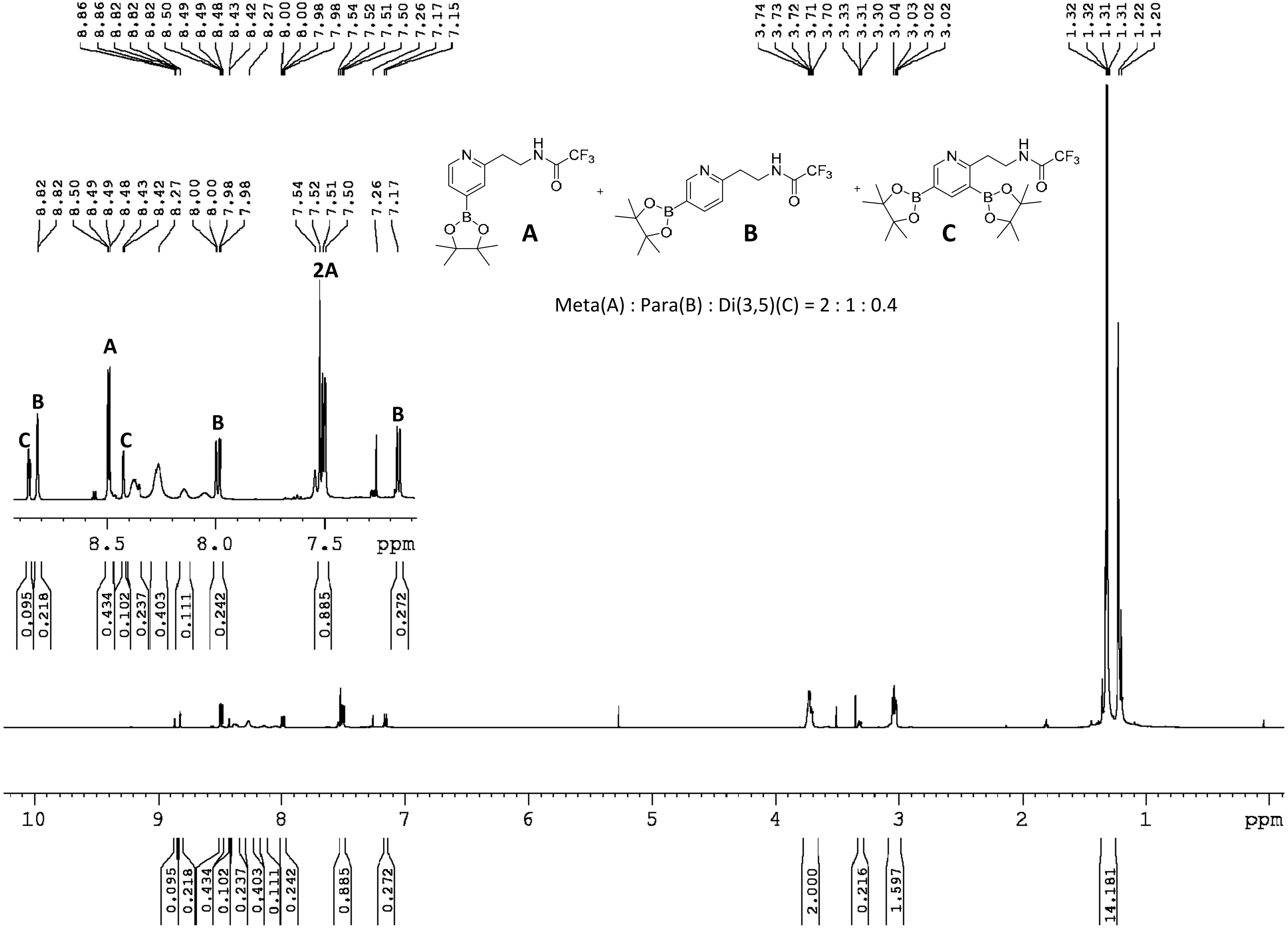

^{13}C NMR (126 MHz, $CDCl_3$) of 2,2,2-trifluoro-N-(2-(4-(4,4,5,5-tetramethyl-1,3,2-dioxaborolan-2-yl)pyridin-2-yl)ethyl)acetamide (5m) – Dtbpy – Crude mixture

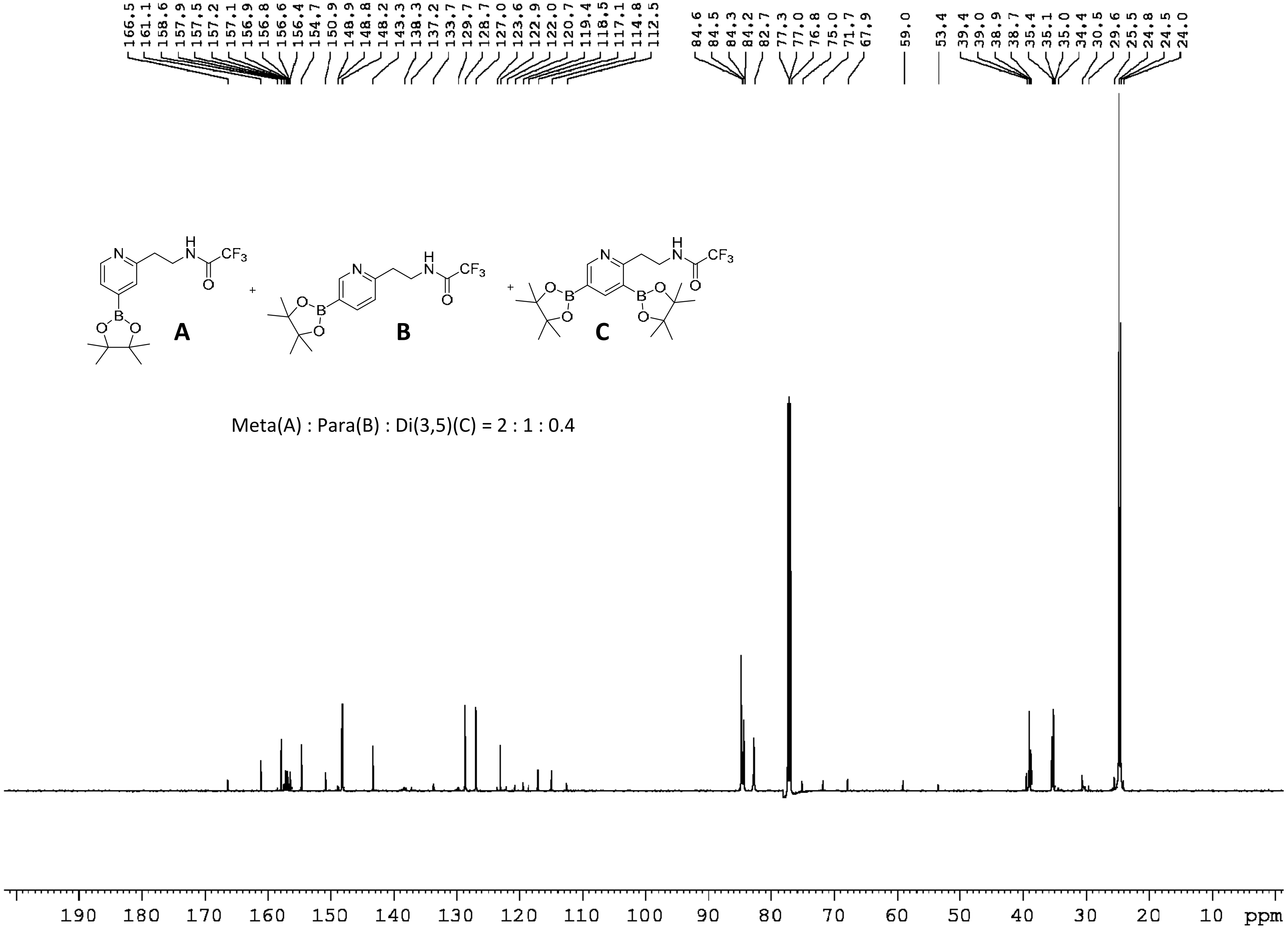

^{1}H NMR (500 MHz, $CDCl_3$) of 2,2,2-trifluoro-N-(2-(4-(4,4,5,5-tetramethyl-1,3,2-dioxaborolan-2-yl)pyridin-2-yl)ethyl)acetamide (5m) – Ligand 1a – Crude mixture

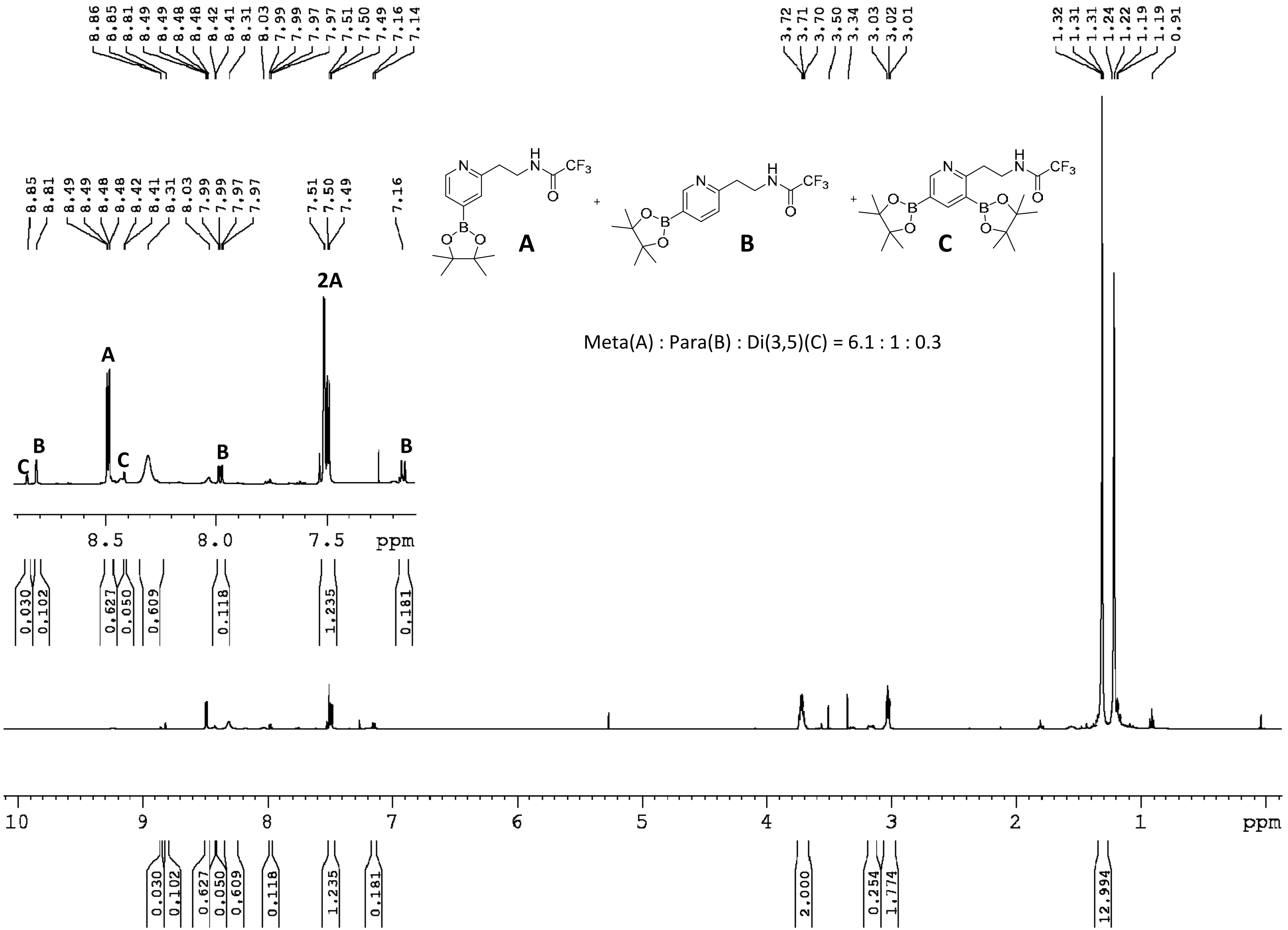

^{13}C NMR (126 MHz, $CDCl_3$) of 2,2,2-trifluoro-N-(2-(4-(4,4,5,5-tetramethyl-1,3,2-dioxaborolan-2-yl)pyridin-2-yl)ethyl)acetamide (5m) – Ligand 1a – Crude mixture

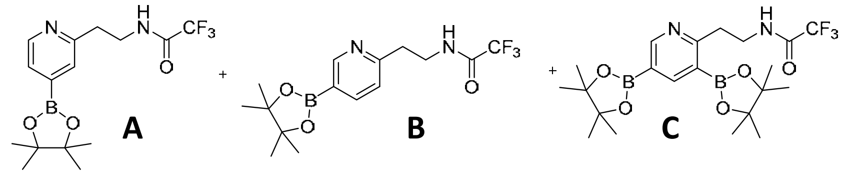

Meta(A) : Para(B) : Di(3,5)(C) = 6.1 : 1 : 0.3

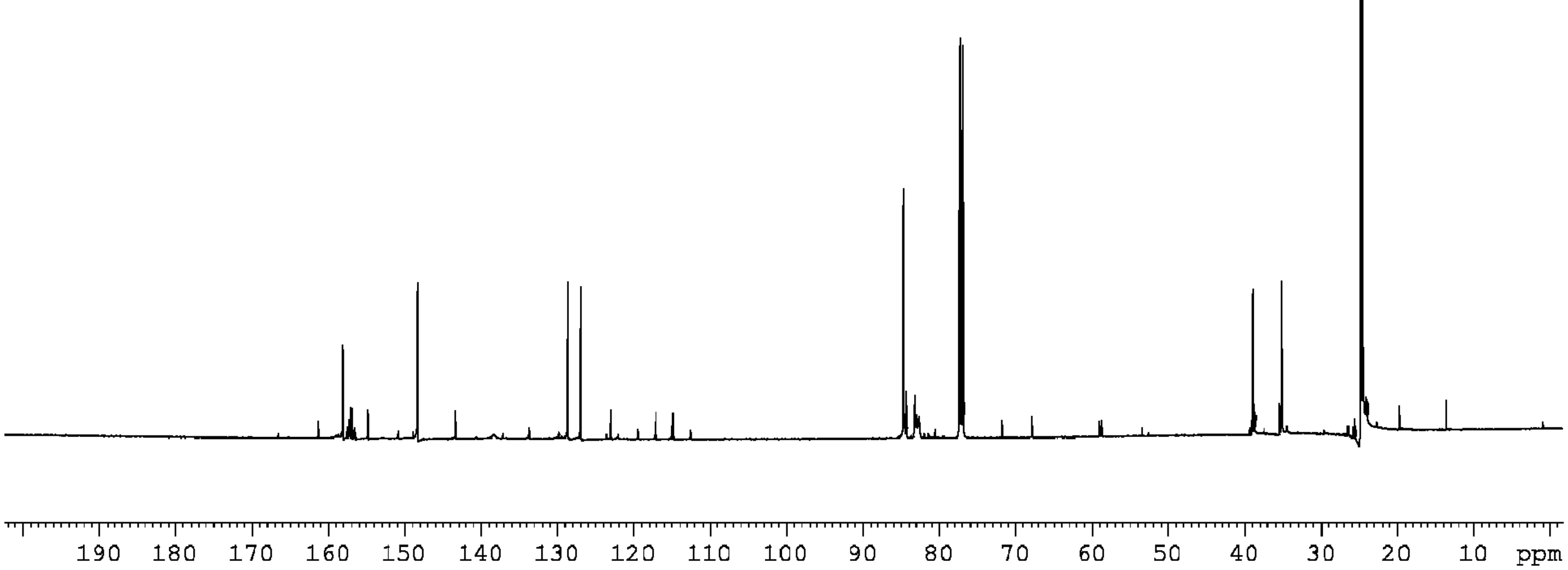

^{1}H NMR (600 MHz, $CDCl_3$) 2,2,2-Trifluoro-*N*-(3-(5-(4,4,5,5-tetramethyl-1,3,2-dioxaborolan-2-yl)-2-(trifluoromethyl)phenyl)propyl)acetamide (7a) – Crude after reaction with 1a

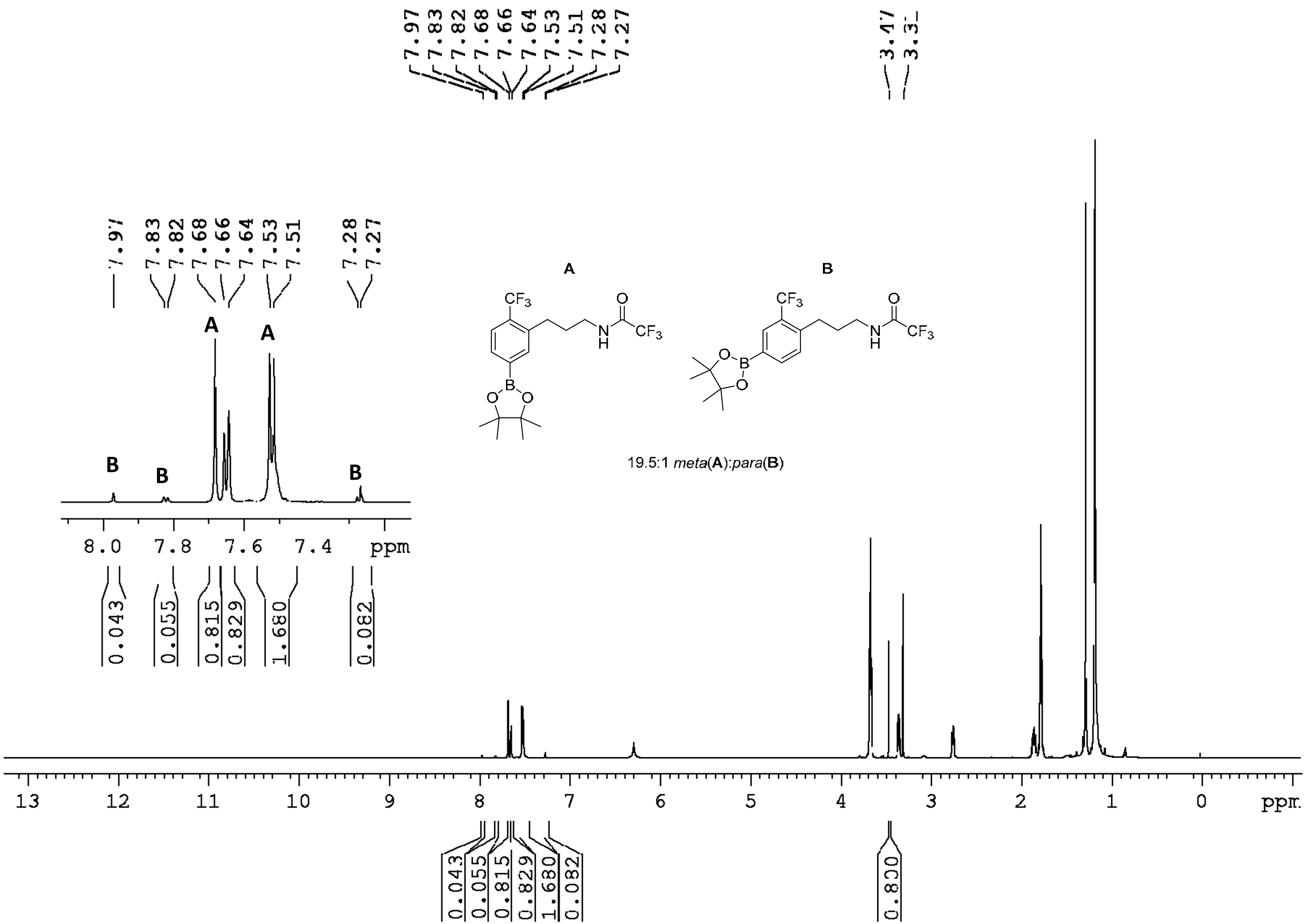

^{1}H NMR (600 MHz, $CDCl_3$) 2,2,2-Trifluoro-*N*-(3-(5-(4,4,5,5-tetramethyl-1,3,2-dioxaborolan-2-yl)-2-(trifluoromethyl)phenyl)propyl)acetamide (7a) – Purified after reaction with 1a

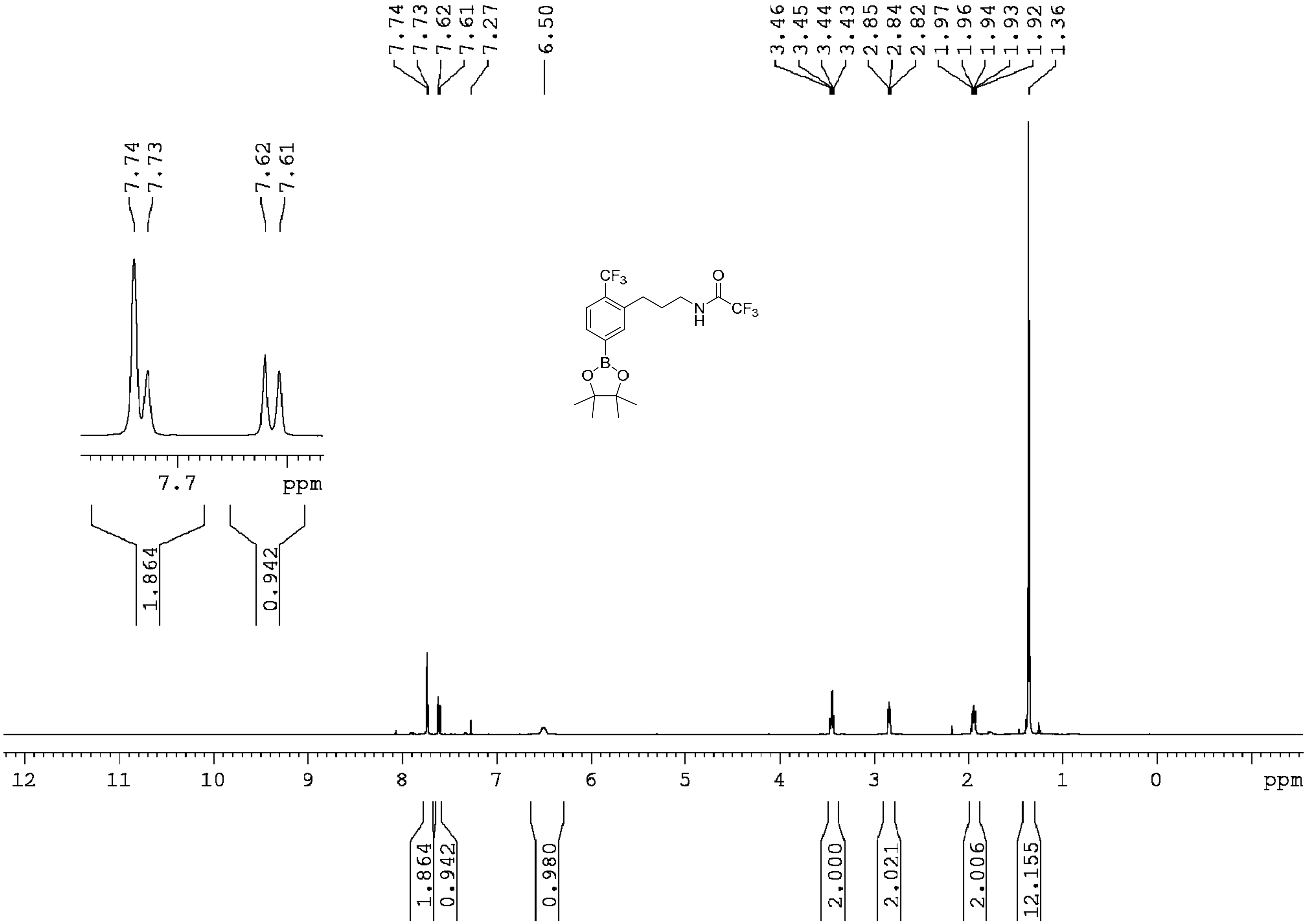

^{13}C NMR (151 MHz, $CDCl_3$ 2,2,2-Trifluoro-*N*-(3-(5-(4,4,5,5-tetramethyl-1,3,2-dioxaborolan-2-yl)-2-(trifluoromethyl)phenyl)propyl)acetamide (7a) – Purified after reaction with 1a

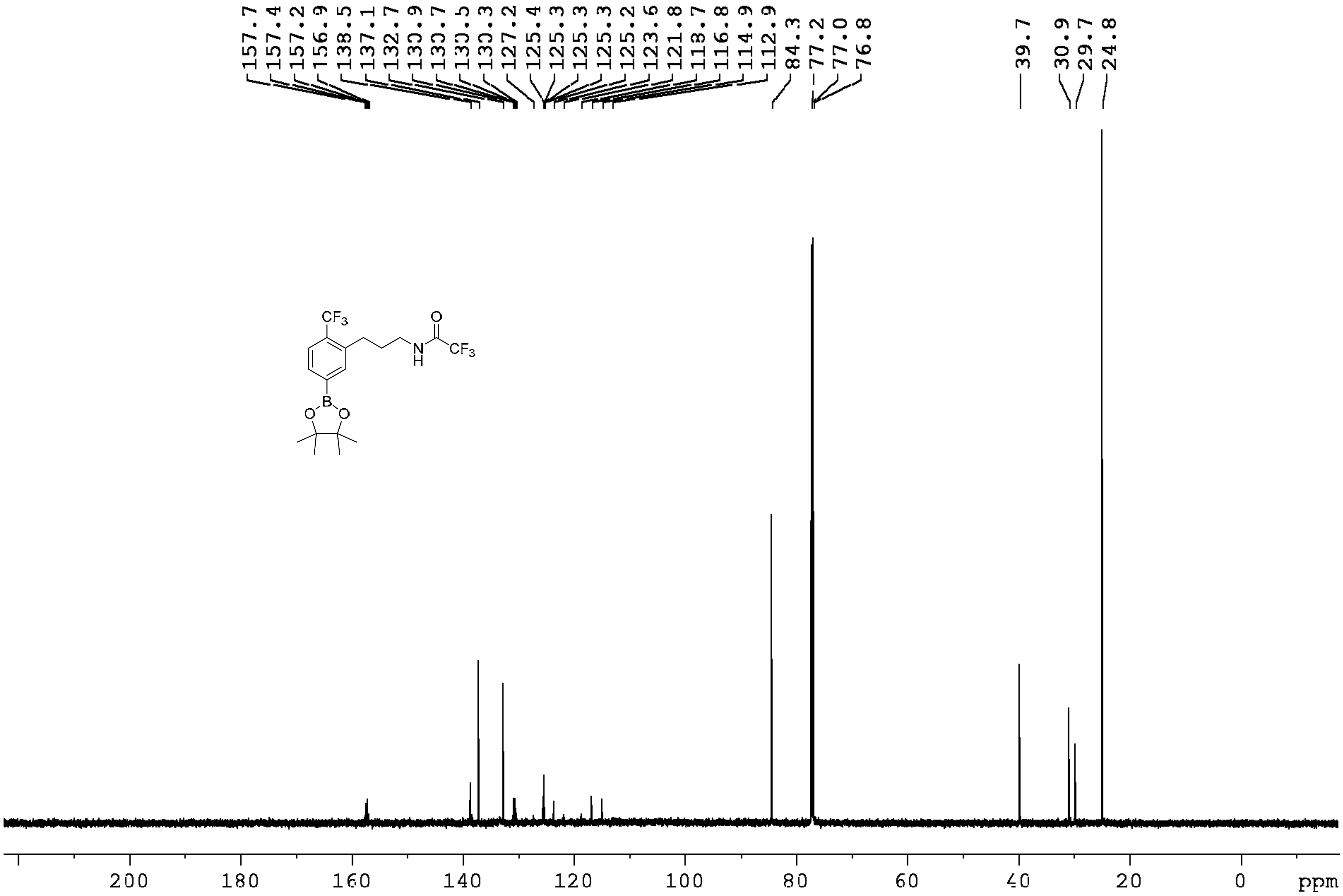

^{1}H NMR (600 MHz, $CDCl_3$) 2,2,2-Trifluoro-*N*-(3-(5-(4,4,5,5-tetramethyl-1,3,2-dioxaborolan-2-yl)-2-(trifluoromethyl)phenyl)propyl)acetamide (7a) – Purified after reaction with dtbpy

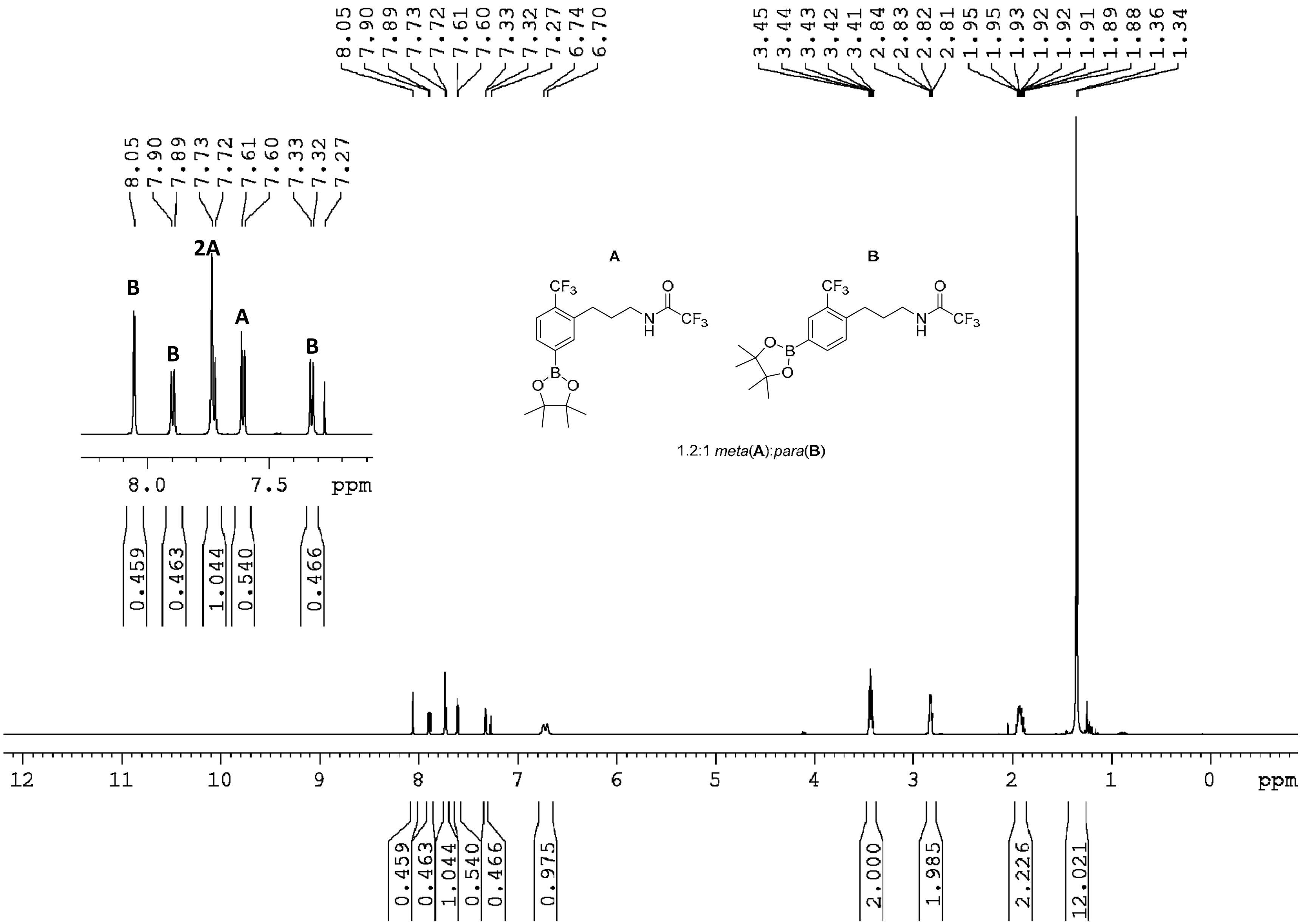

^{13}C NMR (151 MHz, $CDCl_3$ 2,2,2-Trifluoro-*N*-(3-(5-(4,4,5,5-tetramethyl-1,3,2-dioxaborolan-2-yl)-2-(trifluoromethyl)phenyl)propyl)acetamide (7a) – Purified after reaction with dtbpy

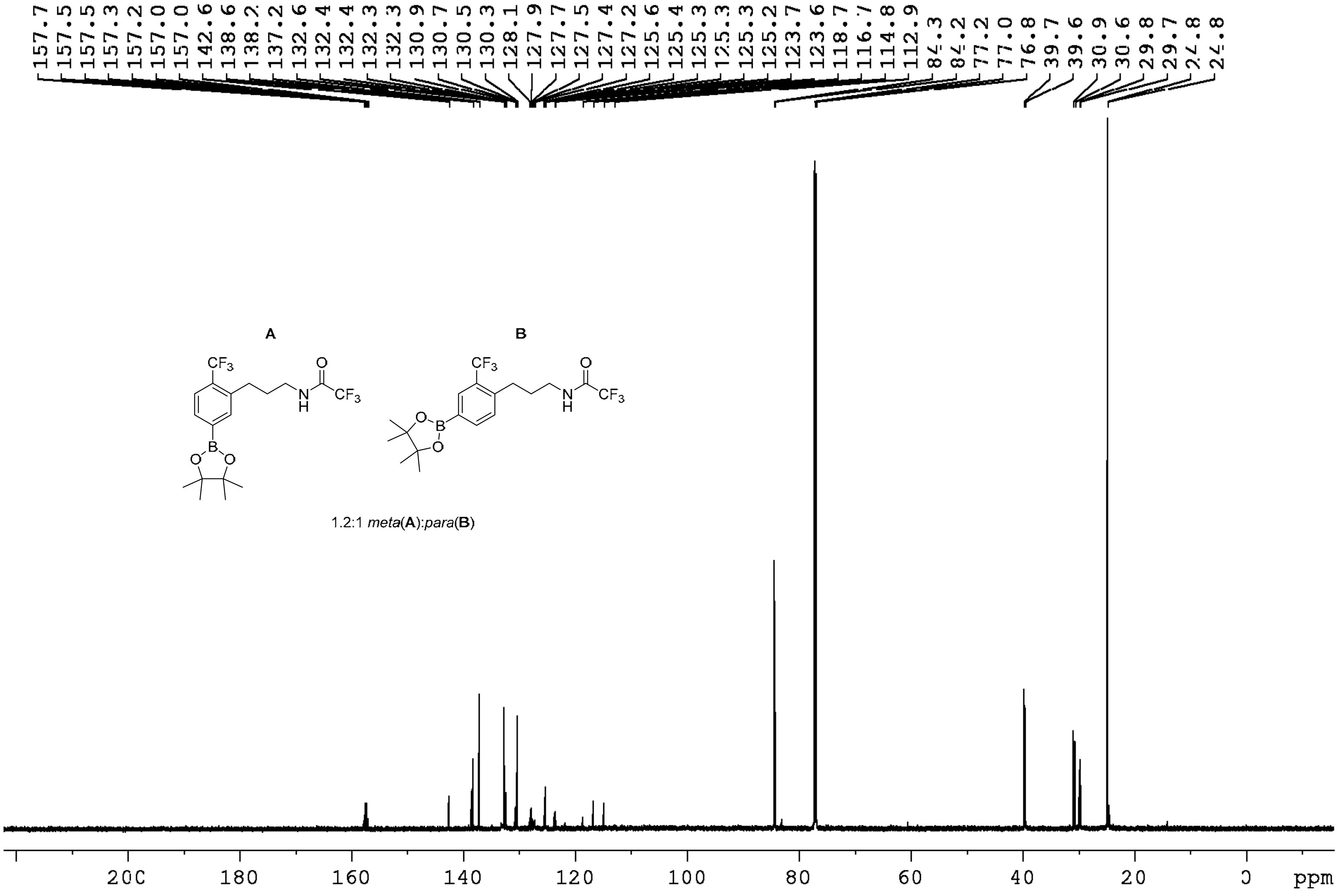

^{1}H NMR (500 MHz, $CDCl_3$) *N*-(3-(3,5-bis(4,4,5,5-tetramethyl-1,3,2-dioxaborolan-2-yl)phenyl)propyl)-2,2,2-trifluoroacetamide (7b) – Crude after reaction with 1a

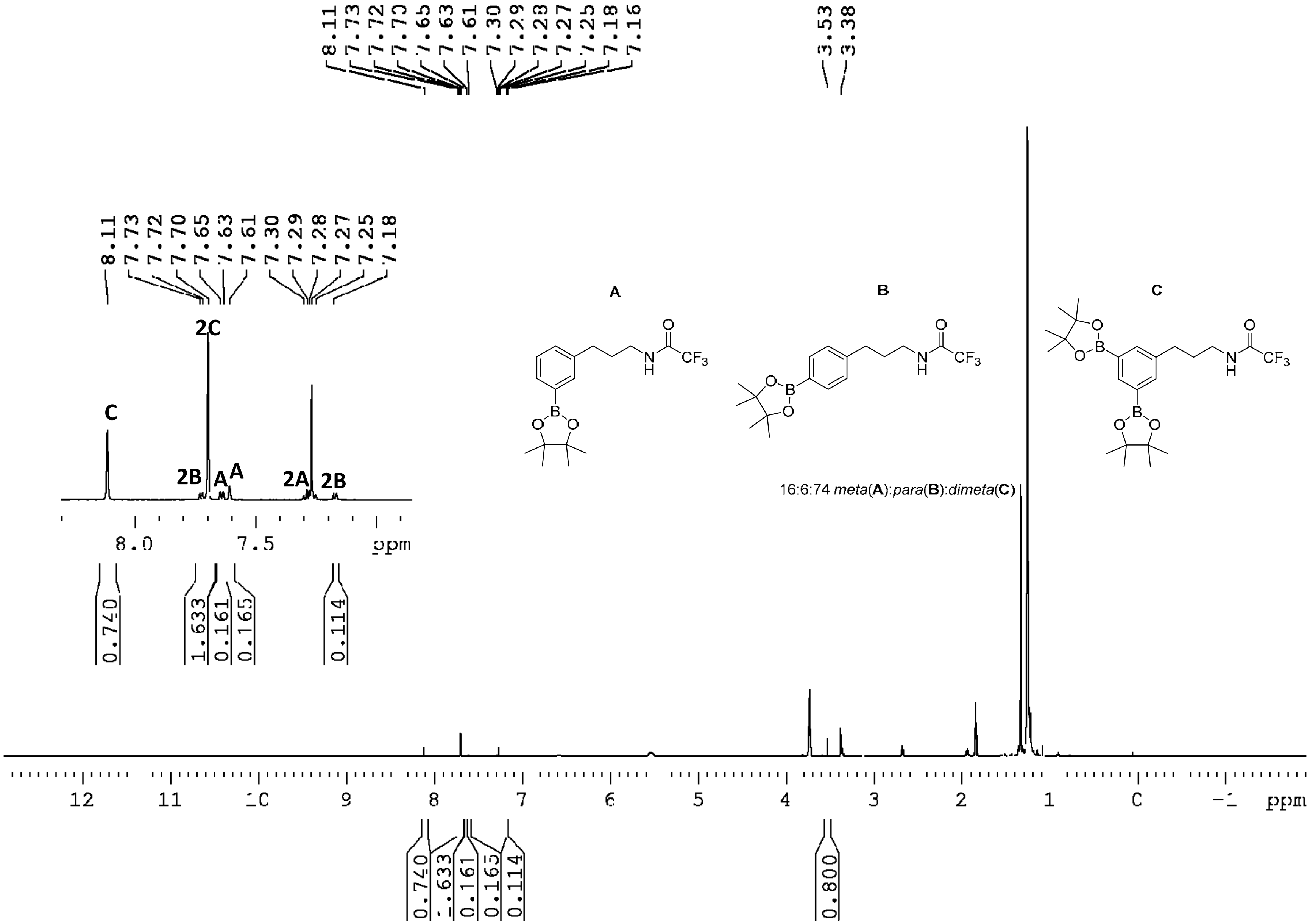

^{1}H NMR (500 MHz, $CDCl_3$) *N*-(3-(3,5-bis(4,4,5,5-tetramethyl-1,3,2-dioxaborolan-2-yl)phenyl)propyl)-2,2,2-trifluoroacetamide (7b) – Purified *dimeta* fraction after reaction with 1a

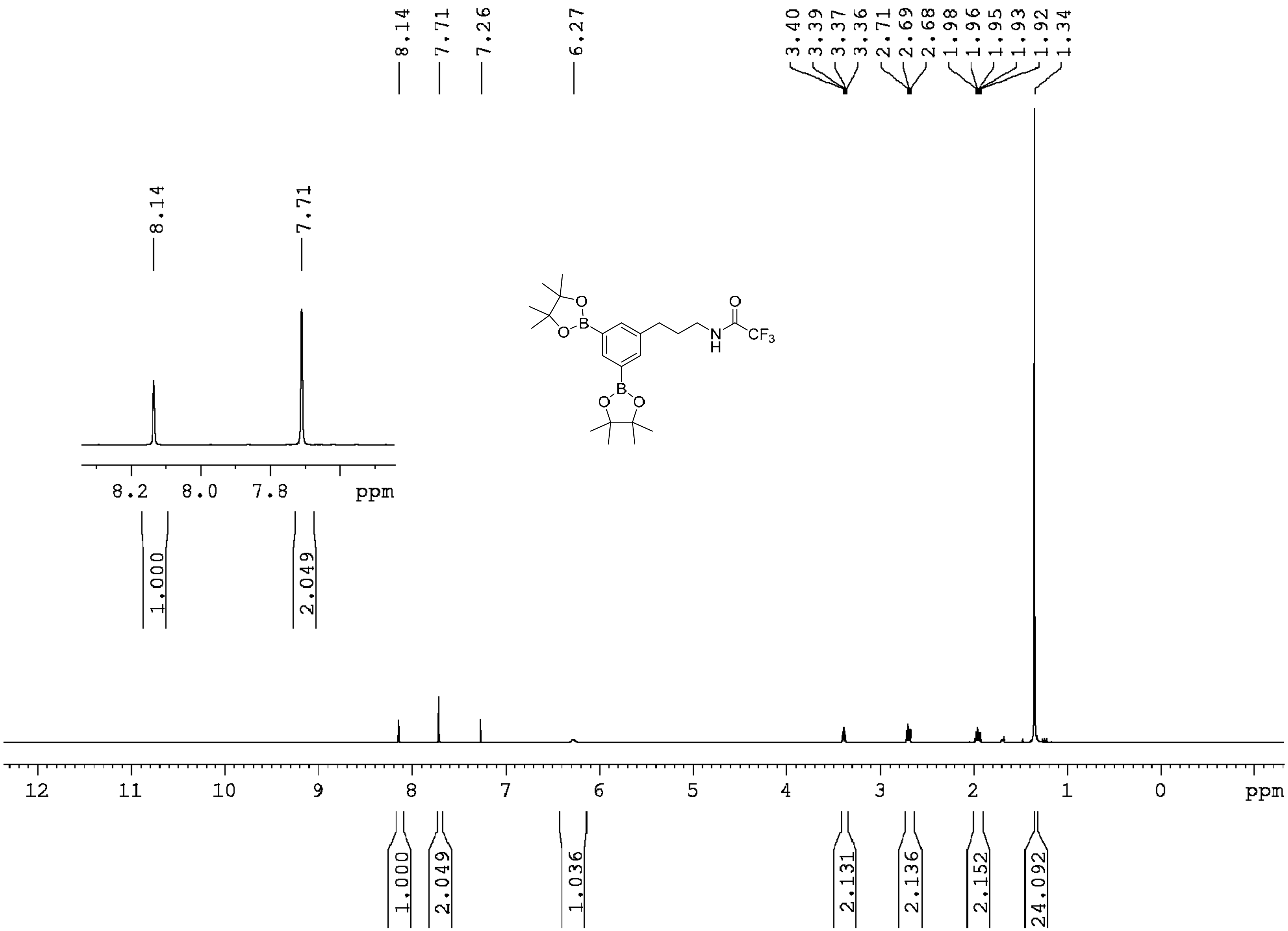

^{13}C NMR (126 MHz, $CDCl_3$) *N*-(3-(3,5-bis(4,4,5,5-tetramethyl-1,3,2-dioxaborolan-2-yl)phenyl)propyl)-2,2,2-trifluoroacetamide (7b) – Purified *dimeta* fraction after reaction with 1a

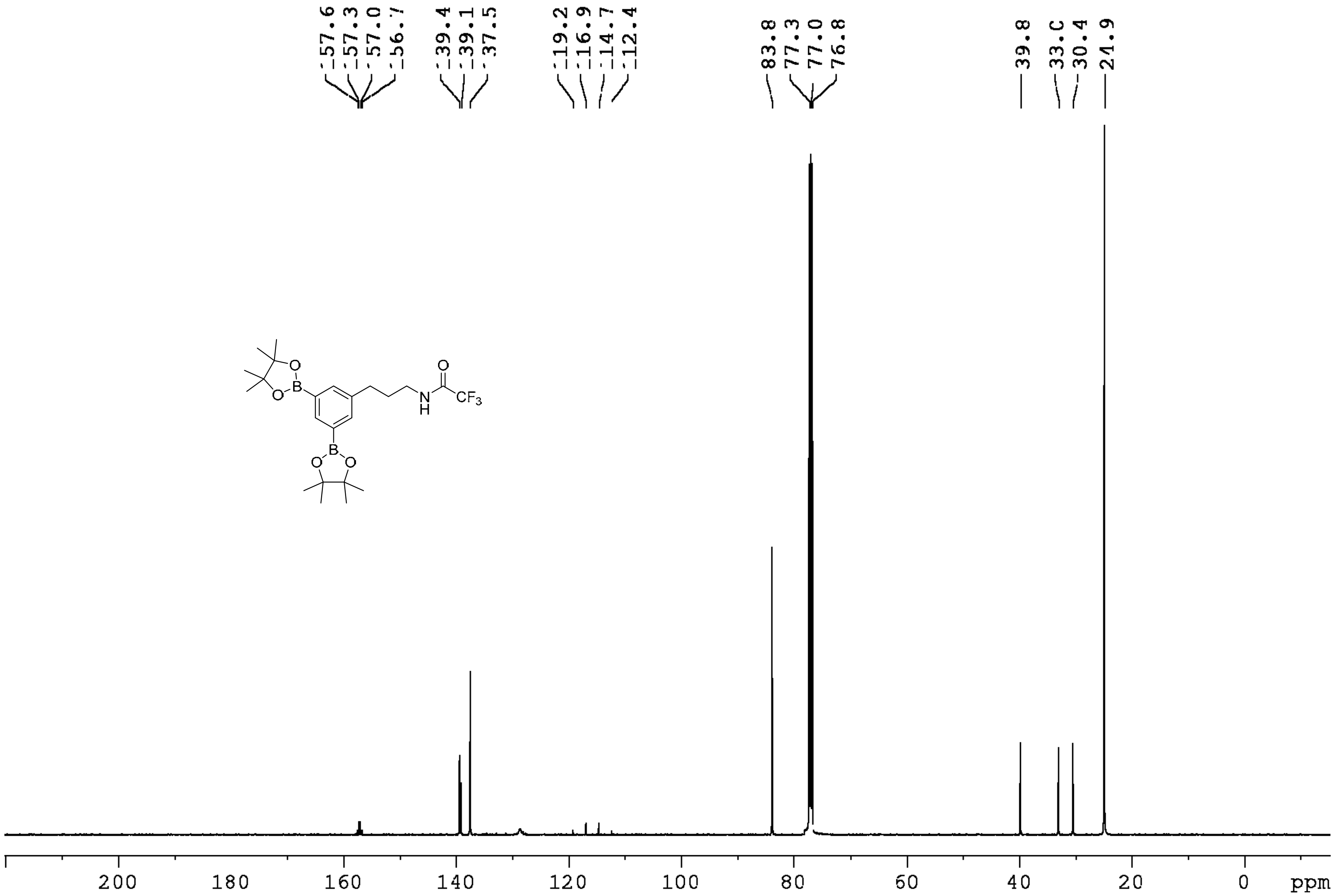

^{1}H NMR (500 MHz, $CDCl_3$) 2,2,2-Trifluoro-*N*-(3-(3-(4,4,5,5-tetramethyl-1,3,2-dioxaborolan-2-yl)phenyl)propyl)acetamide (7b) – Purified fraction of mono borylated products after reaction with dtbpy

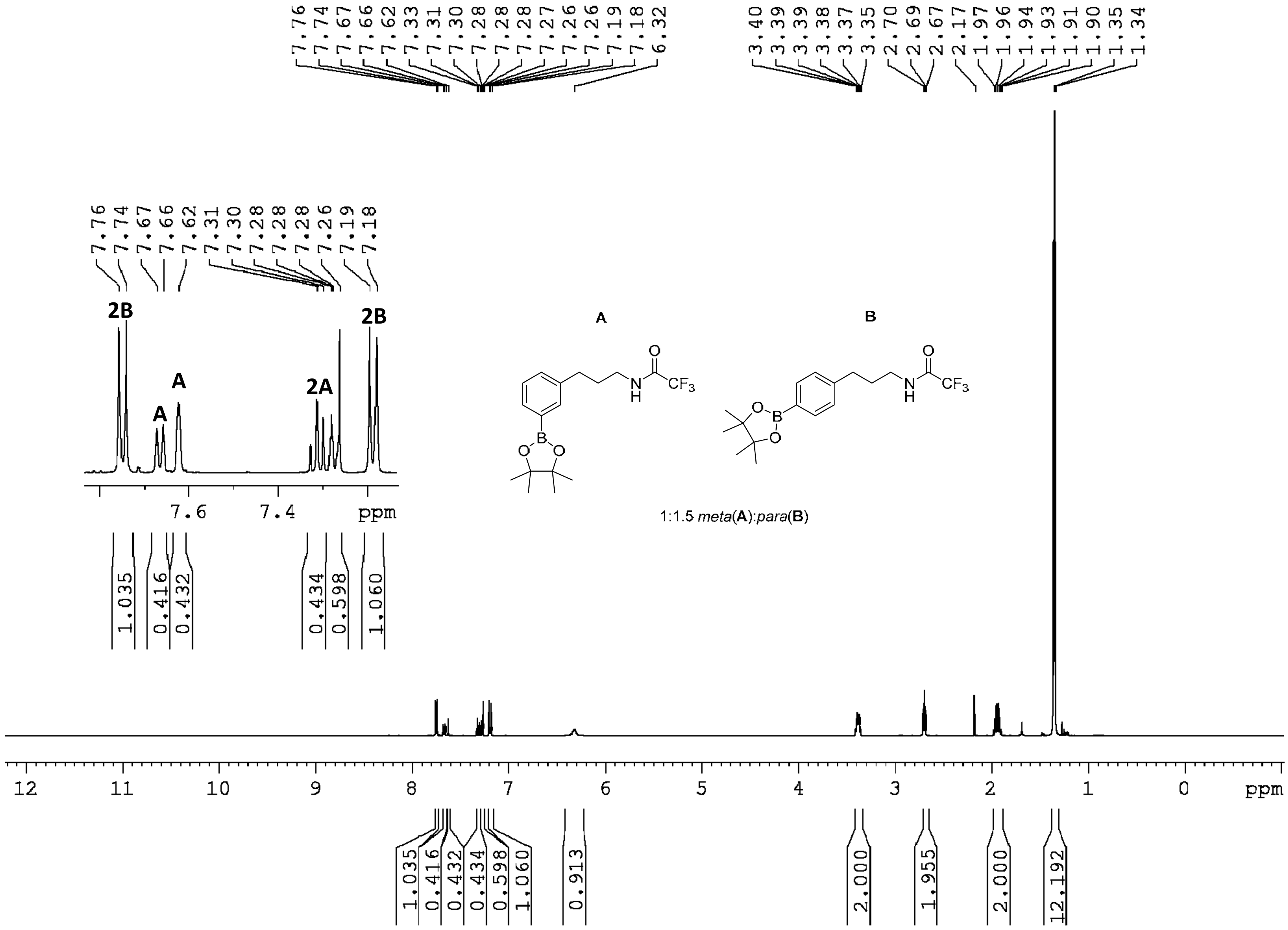

^{13}C NMR (126 MHz, $CDCl_3$) 2,2,2-Trifluoro-*N*-(3-(3-(4,4,5,5-tetramethyl-1,3,2-dioxaborolan-2-yl)phenyl)propyl)acetamide (7b) – Purified fraction of mono borylated products after reaction with dtbpy

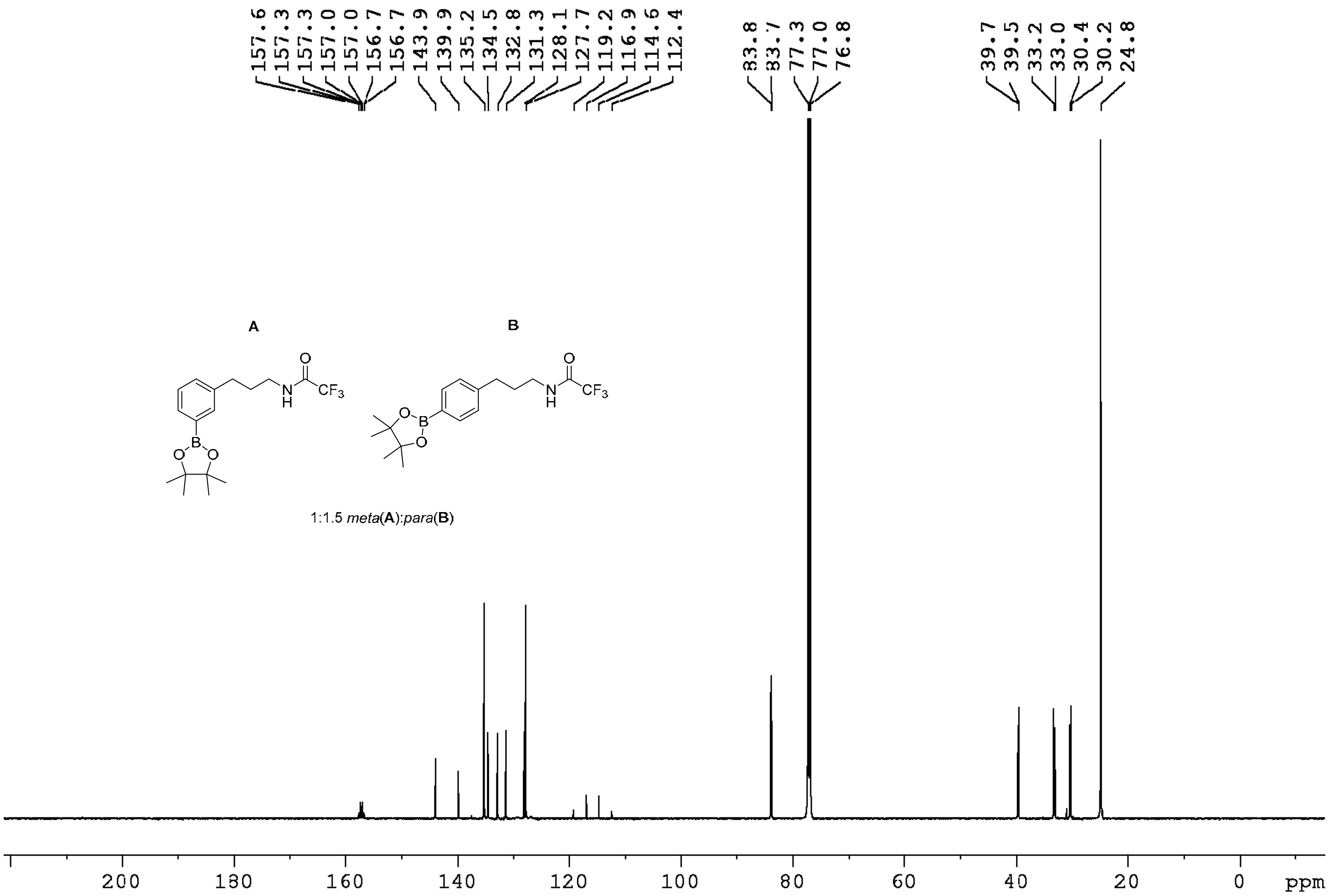

^{1}H NMR (600 MHz, $CDCl_3$) *N*-(3-(2-bromo-5-(4,4,5,5-tetramethyl-1,3,2-dioxaborolan-2-yl)phenyl)propyl)-2,2,2-trifluoroacetamide (7c) – Crude after reaction with 1a

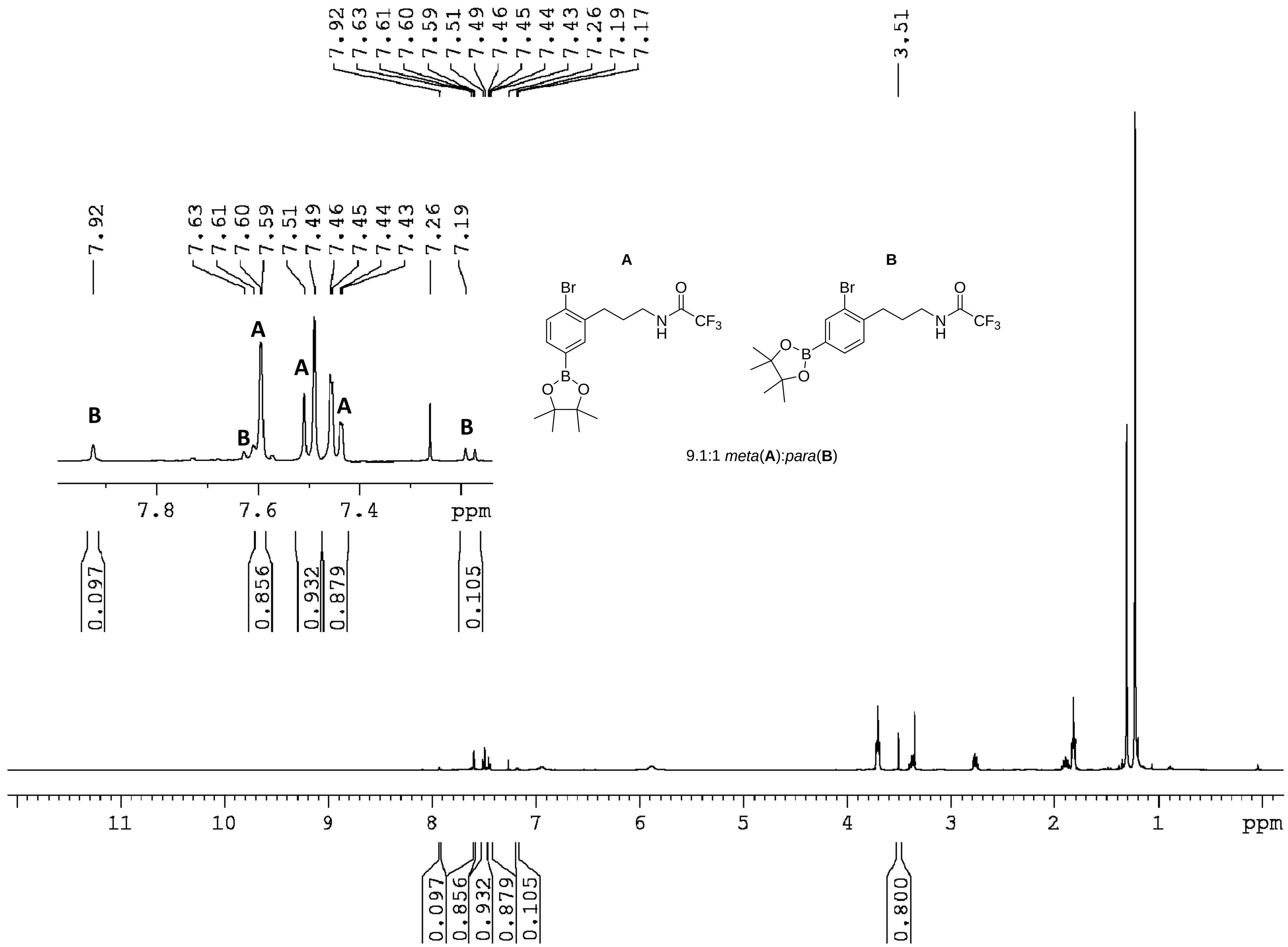

^{1}H NMR (600 MHz, $CDCl_3$) *N*-(3-(2-bromo-5-(4,4,5,5-tetramethyl-1,3,2-dioxaborolan-2-yl)phenyl)propyl)-2,2,2-trifluoroacetamide (7c) – Purified after reaction with 1a

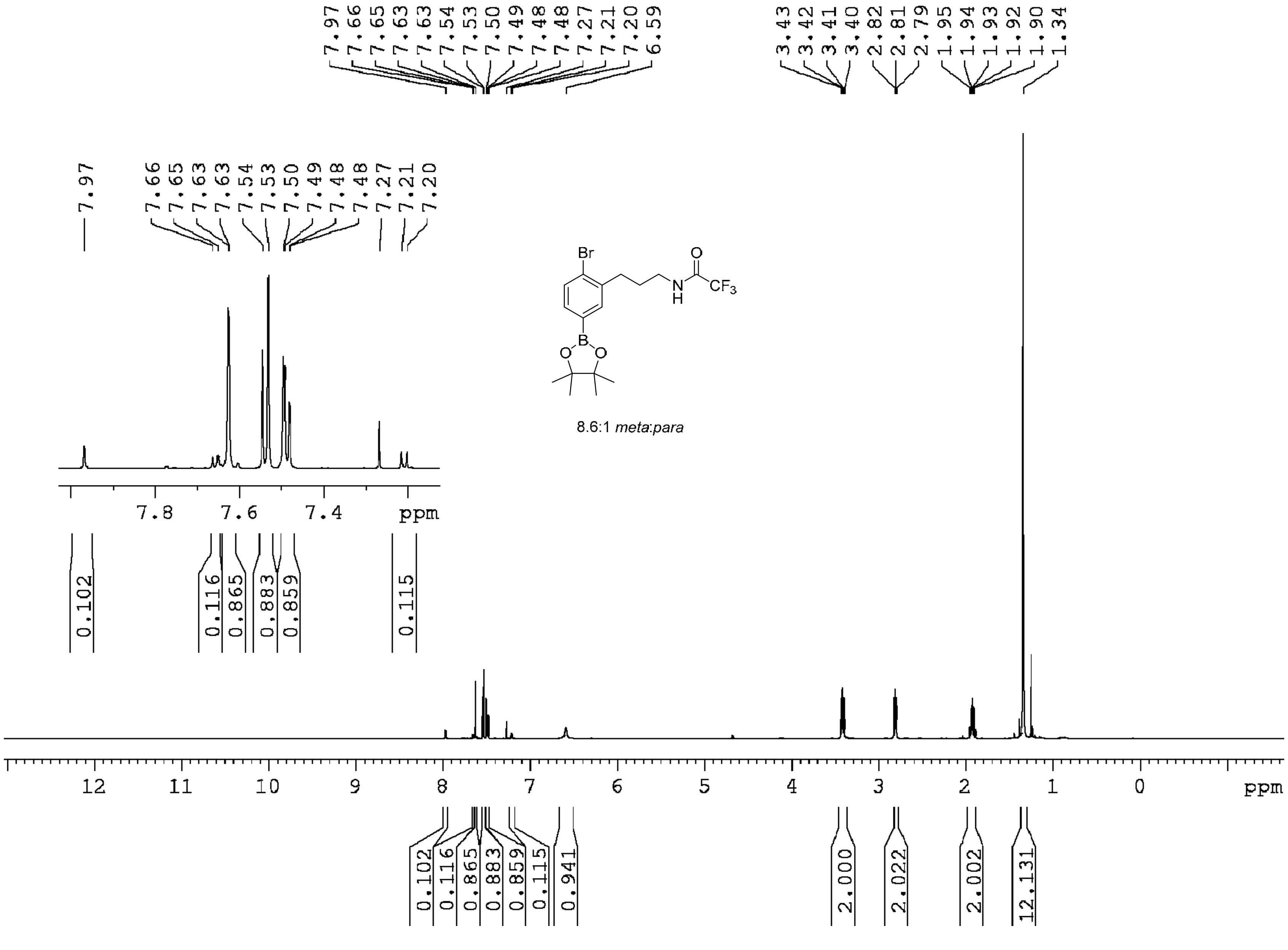

^{13}C NMR (151 MHz, $CDCl_3$) *N*-(3-(2-bromo-5-(4,4,5,5-tetramethyl-1,3,2-dioxaborolan-2-yl)phenyl)propyl)-2,2,2-trifluoroacetamide (7c) – Purified after reaction with 1a

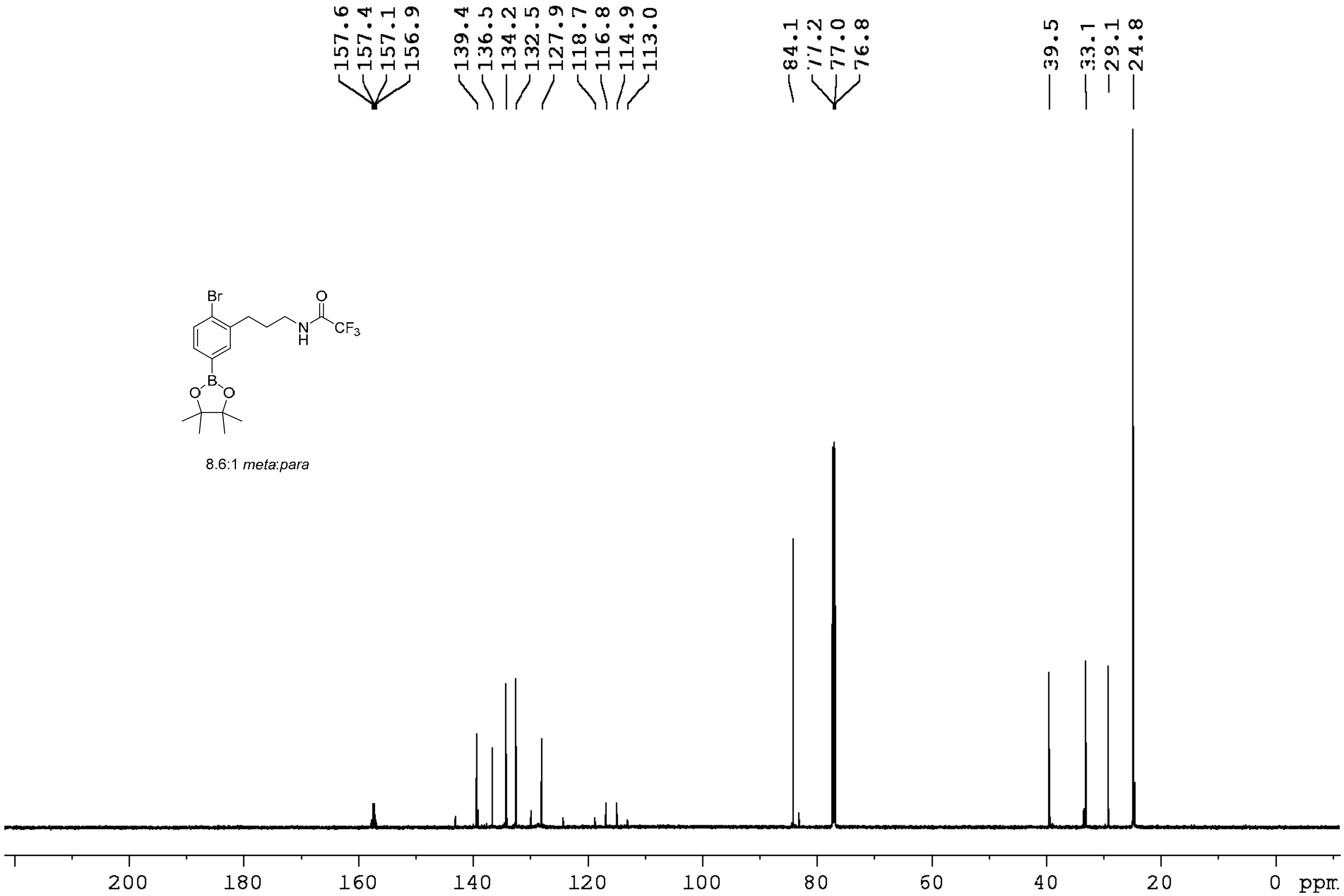

^{1}H NMR (600 MHz, $CDCl_3$) *N*-(3-(2-bromo-5-(4,4,5,5-tetramethyl-1,3,2-dioxaborolan-2-yl)phenyl)propyl)-2,2,2-trifluoroacetamide (7c) – Purified after reaction with dtbpy

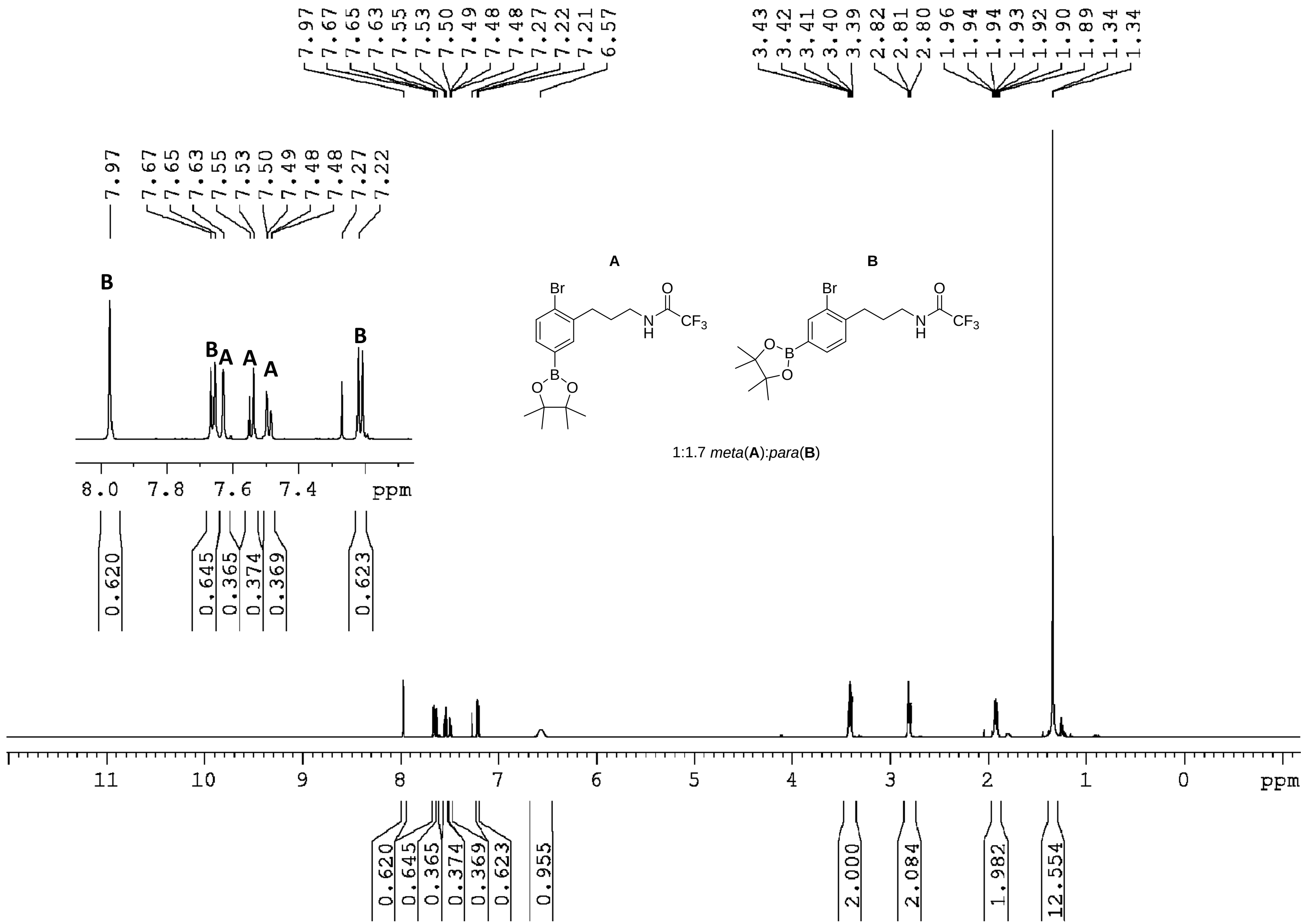

^{13}C NMR (151 MHz, $CDCl_3$) *N*-(3-(2-bromo-5-(4,4,5,5-tetramethyl-1,3,2-dioxaborolan-2-yl)phenyl)propyl)-2,2,2-trifluoroacetamide (7c) – Purified after reaction with dtbpy

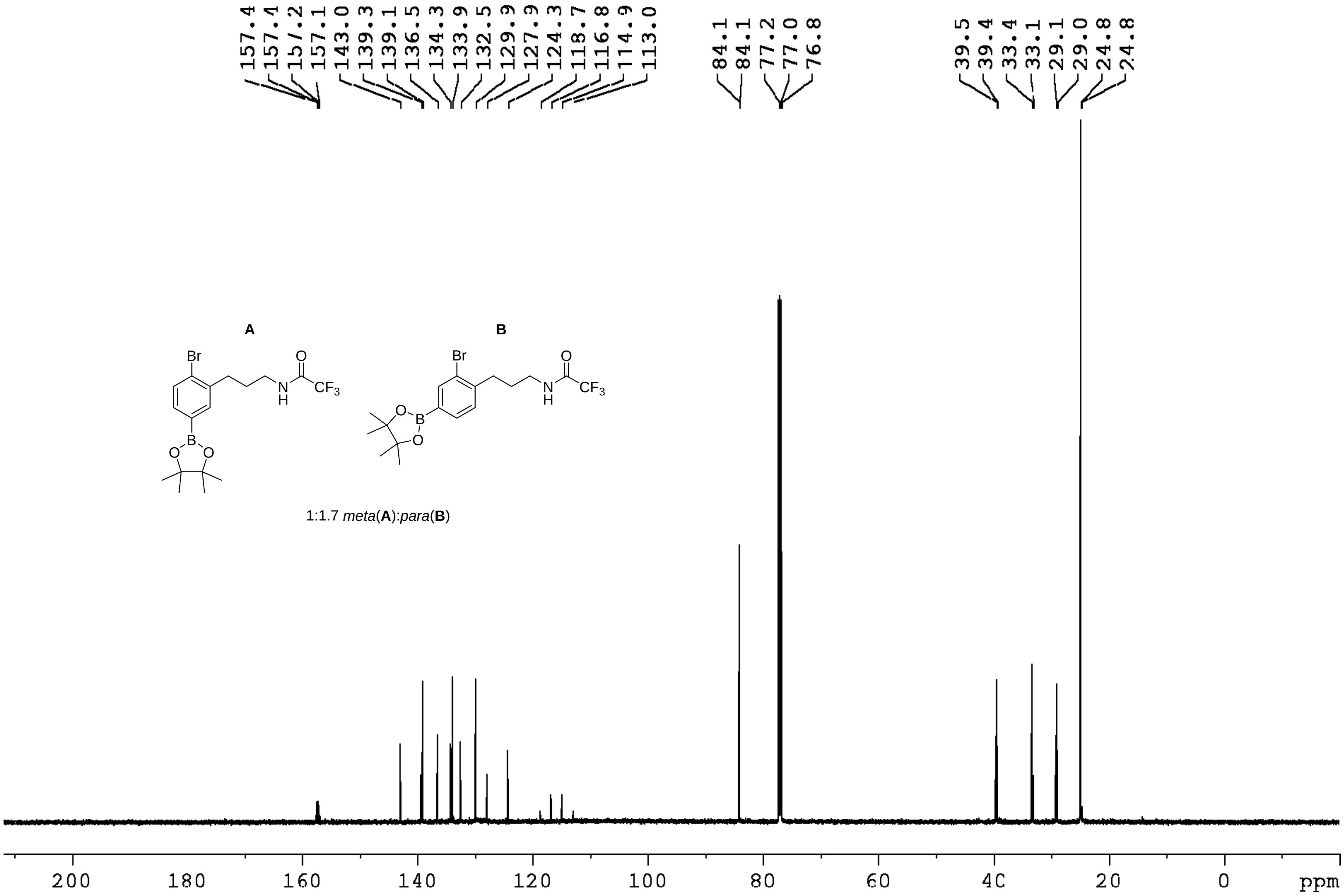

^{1}H NMR (600 MHz, $CDCl_3$) *N*-(3-(2-chloro-5-(4,4,5,5-tetramethyl-1,3,2-dioxaborolan-2-yl)phenyl)propyl)-2,2,2-trifluoroacetamide (7d) – Crude after reaction with 1a

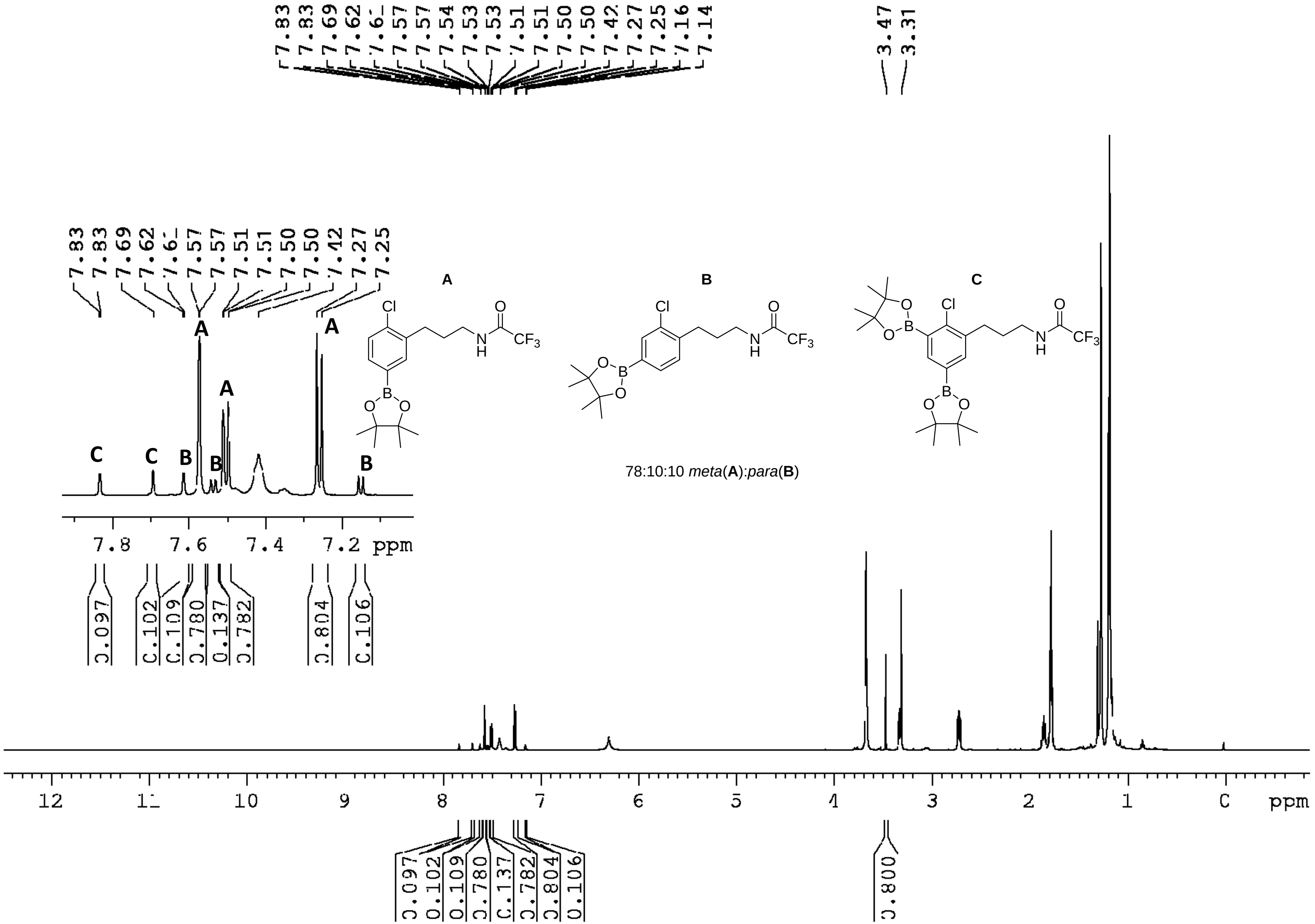

^{1}H NMR (600 MHz, $CDCl_3$) *N*-(3-(2-chloro-5-(4,4,5,5-tetramethyl-1,3,2-dioxaborolan-2-yl)phenyl)propyl)-2,2,2-trifluoroacetamide (7d) – Purified after reaction with 1a

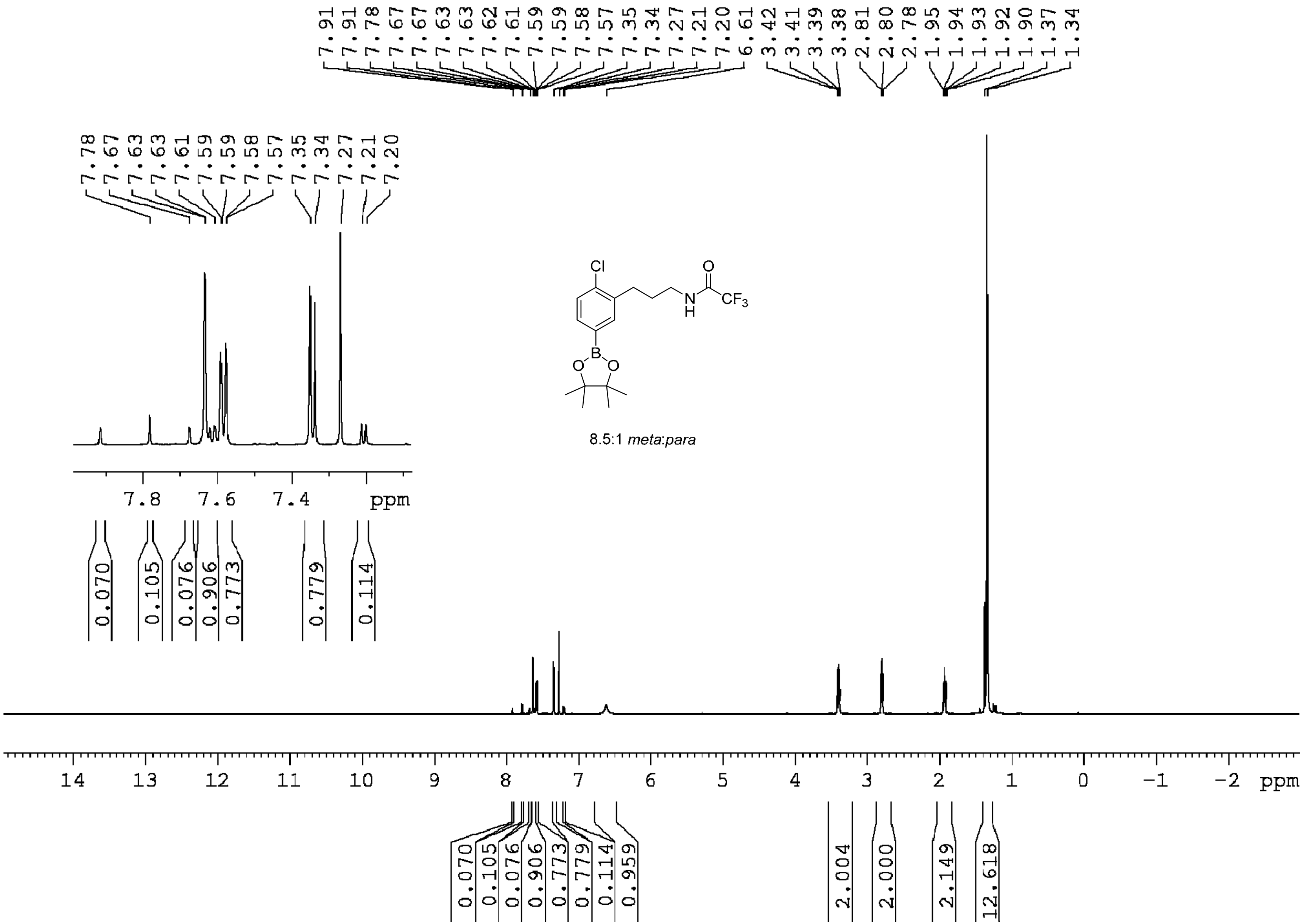

^{13}C NMR (151 MHz, CDCl$_3$) *N*-(3-(2-chloro-5-(4,4,5,5-tetramethyl-1,3,2-dioxaborolan-2-yl)phenyl)propyl)-2,2,2-trifluoroacetamide (7d) – Purified after reaction with 1a

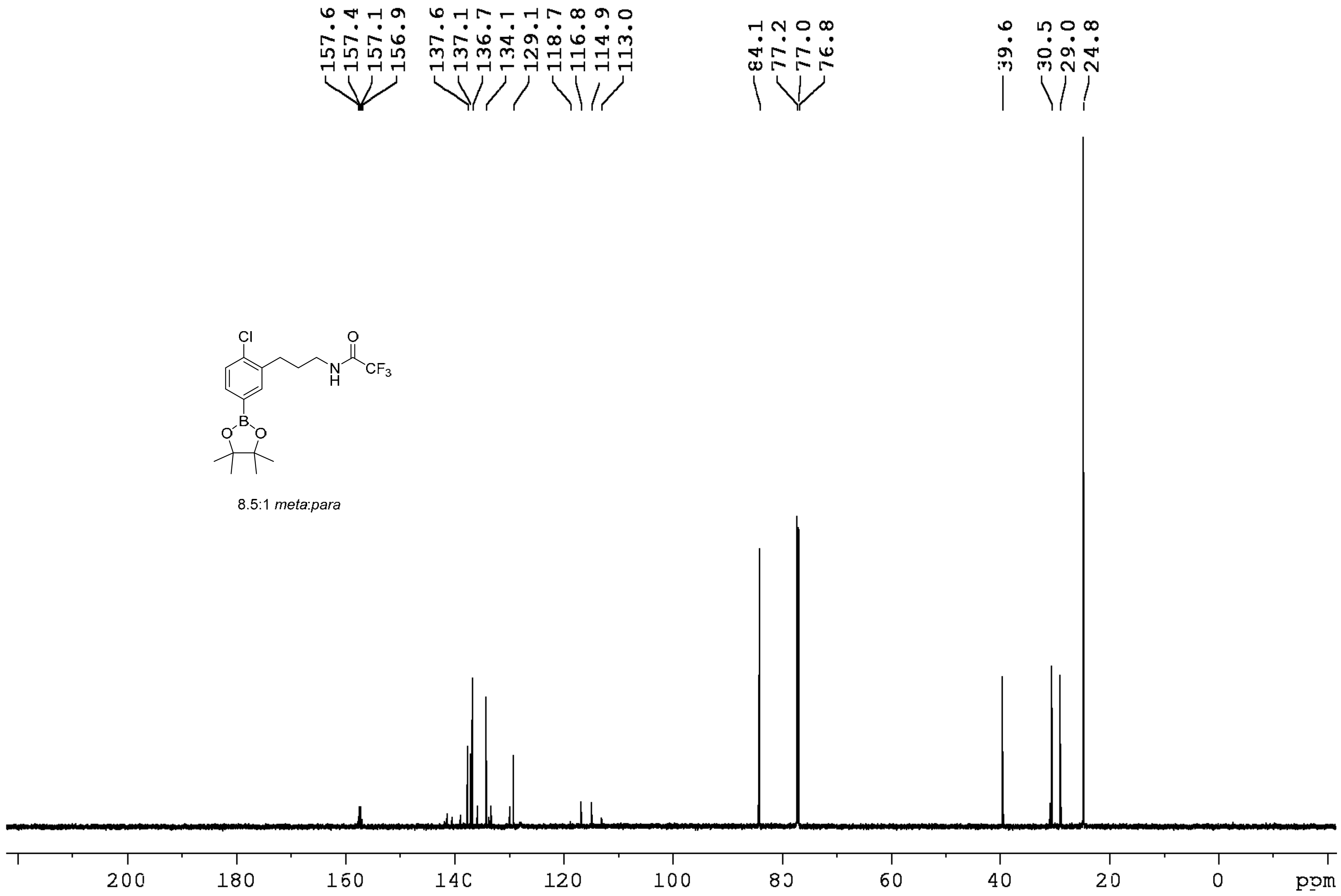

^{1}H NMR (600 MHz, CDCl$_3$) *N*-(3-(2-chloro-5-(4,4,5,5-tetramethyl-1,3,2-dioxaborolan-2-yl)phenyl)propyl)-2,2,2-trifluoroacetamide (7d) – Purified after reaction with dtbpy

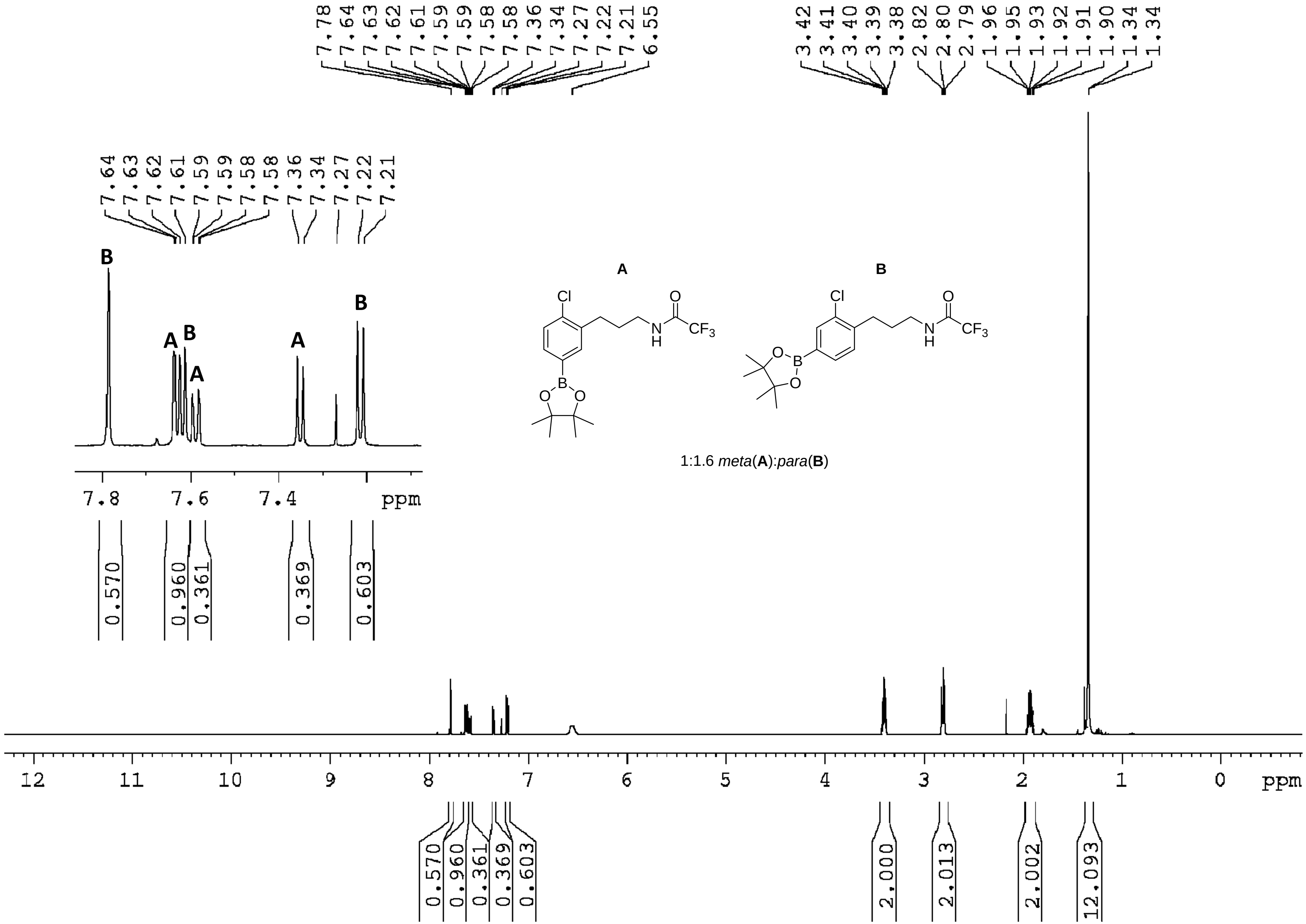

^{13}C NMR (151 MHz, $CDCl_3$) *N*-(3-(2-chloro-5-(4,4,5,5-tetramethyl-1,3,2-dioxaborolan-2-yl)phenyl)propyl)-2,2,2-trifluoroacetamide (7d) – Purified after reaction with dtbpy

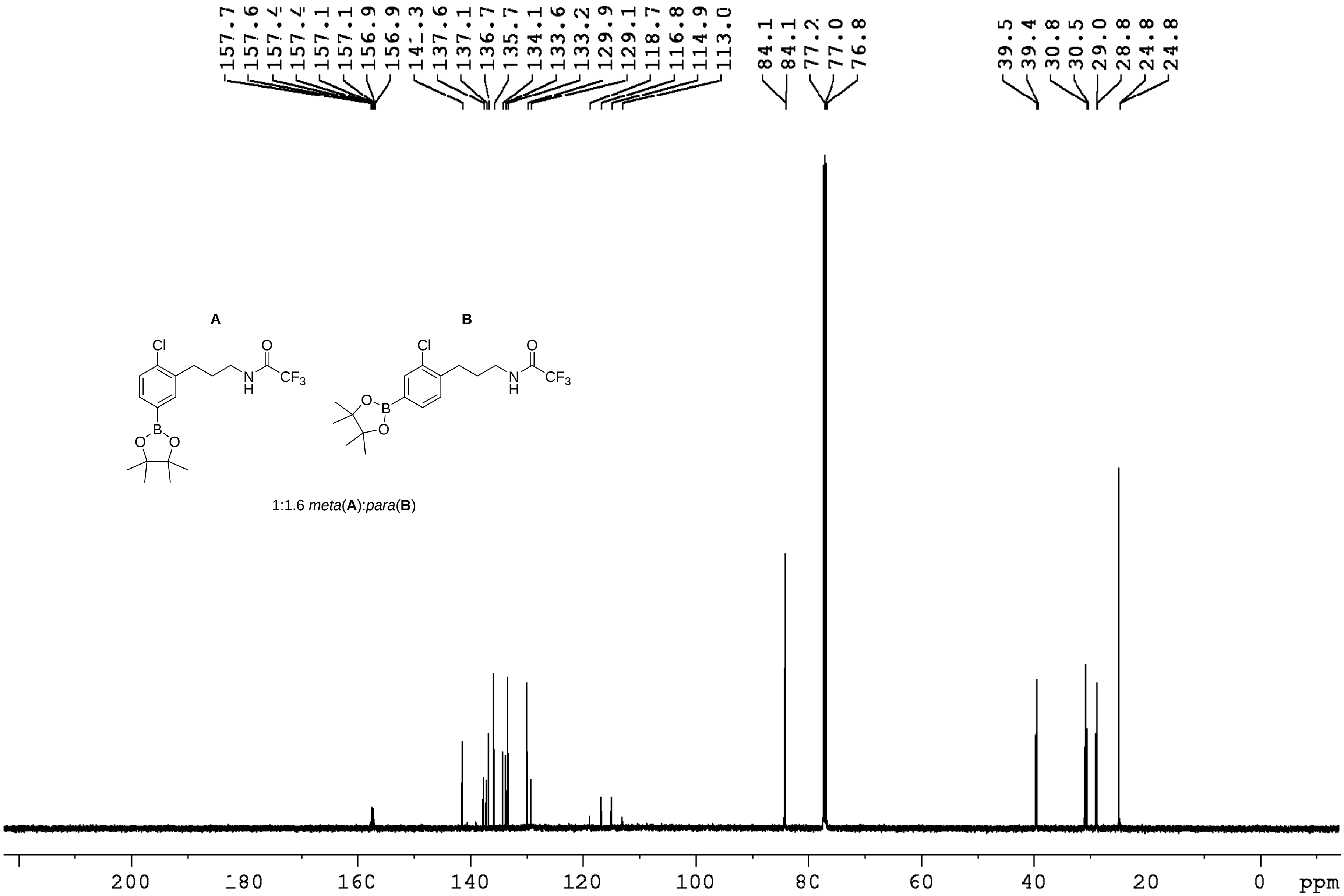

^{1}H NMR (600 MHz, $CDCl_3$) 2,2,2-Trifluoro-*N*-(3-(2-fluoro-3,5-bis(4,4,5,5-tetramethyl-1,3,2-dioxaborolan-2-yl)phenyl)propyl)acetamide (7e) – Crude after reaction with 1a

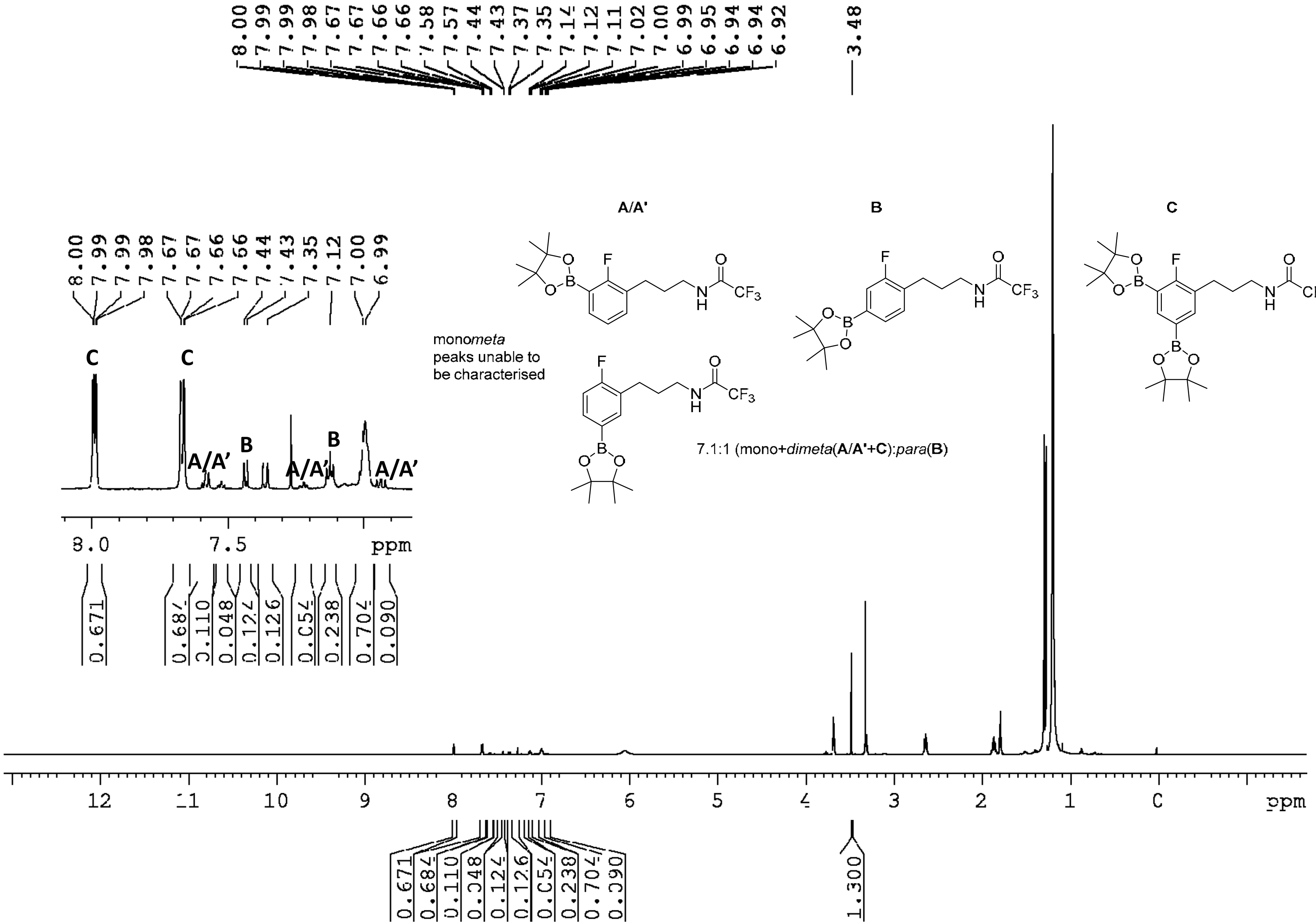

^{19}F NMR (376 MHz, $CDCl_3$) 2,2,2-Trifluoro-*N*-(3-(2-fluoro-3,5-bis(4,4,5,5-tetramethyl-1,3,2-dioxaborolan-2-yl)phenyl)propyl)acetamide (7e) – Crude after reaction with 1a calibrated to match ^{1}H NMR containing internal standard 1,2-dimethoxyethane

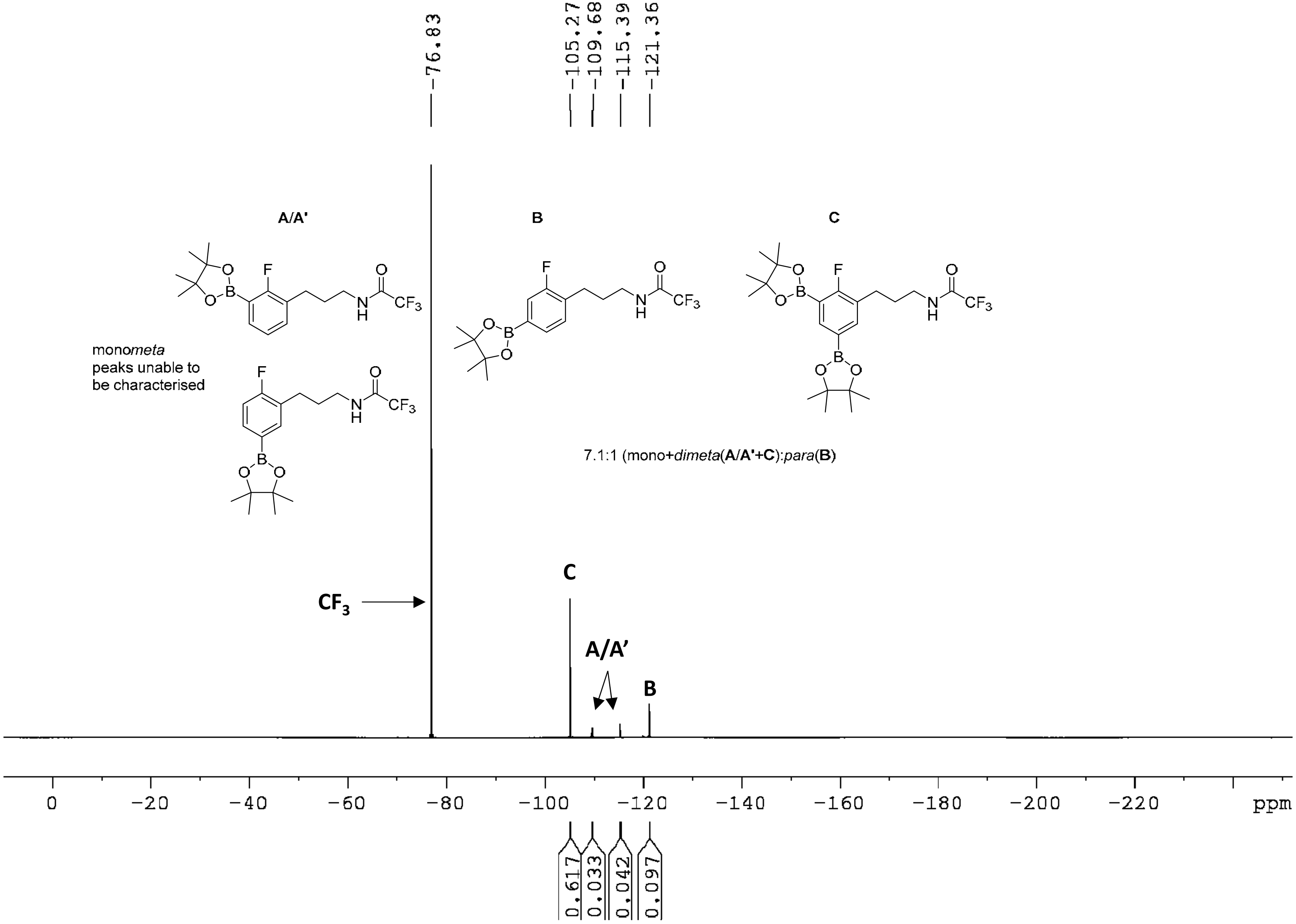

^{1}H NMR (600 MHz, $CDCl_3$) 2,2,2-Trifluoro-*N*-(3-(2-fluoro-3,5-bis(4,4,5,5-tetramethyl-1,3,2-dioxaborolan-2-yl)phenyl)propyl)acetamide (7e) – *Dimeta* fraction purified from reaction with 1a

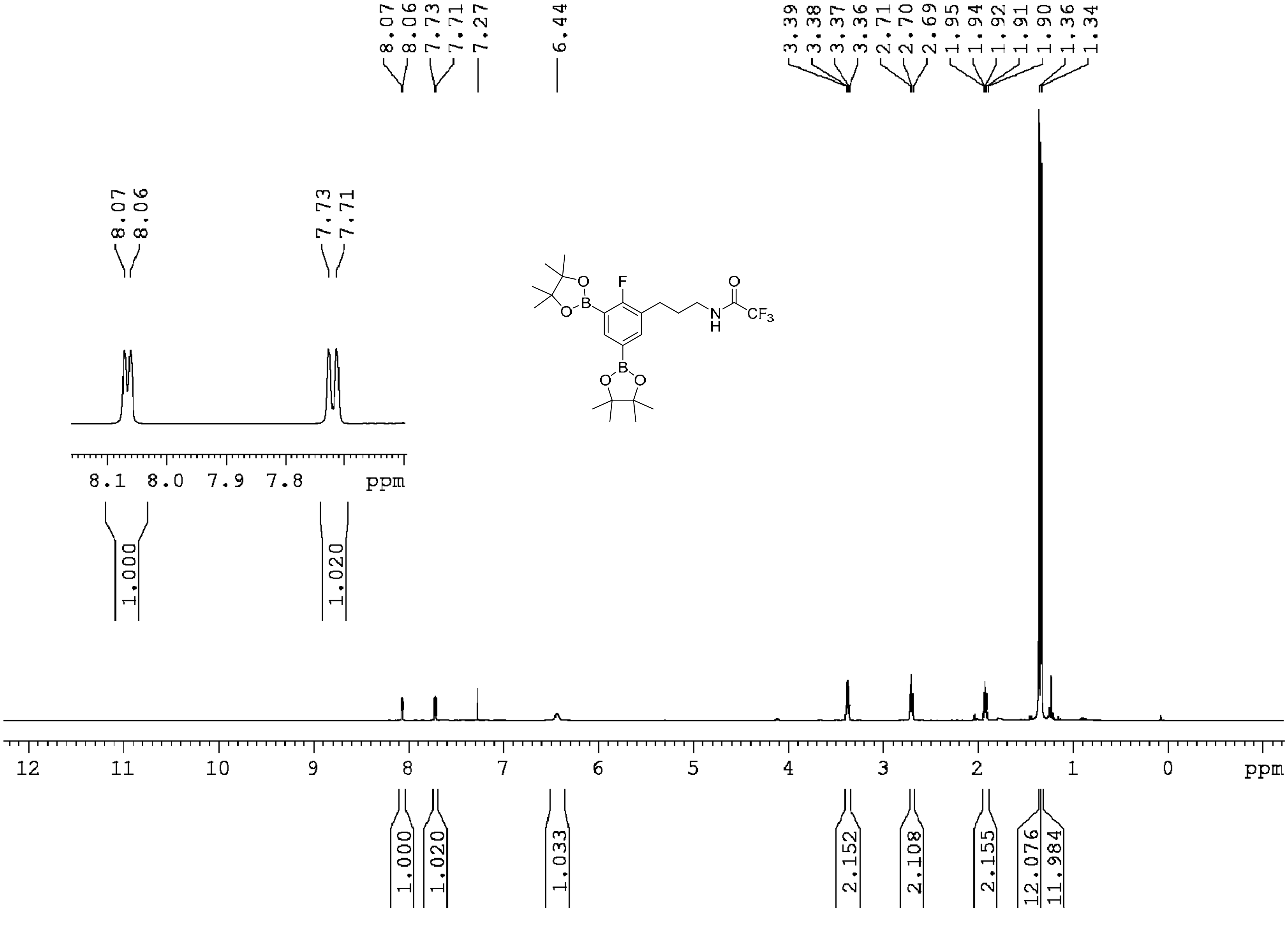

^{13}C NMR (151 MHz, $CDCl_3$) 2,2,2-Trifluoro-*N*-(3-(2-fluoro-3,5-bis(4,4,5,5-tetramethyl-1,3,2-dioxaborolan-2-yl)phenyl)propyl)acetamide (7e) – *Dimeta* fraction purified from reaction with 1a

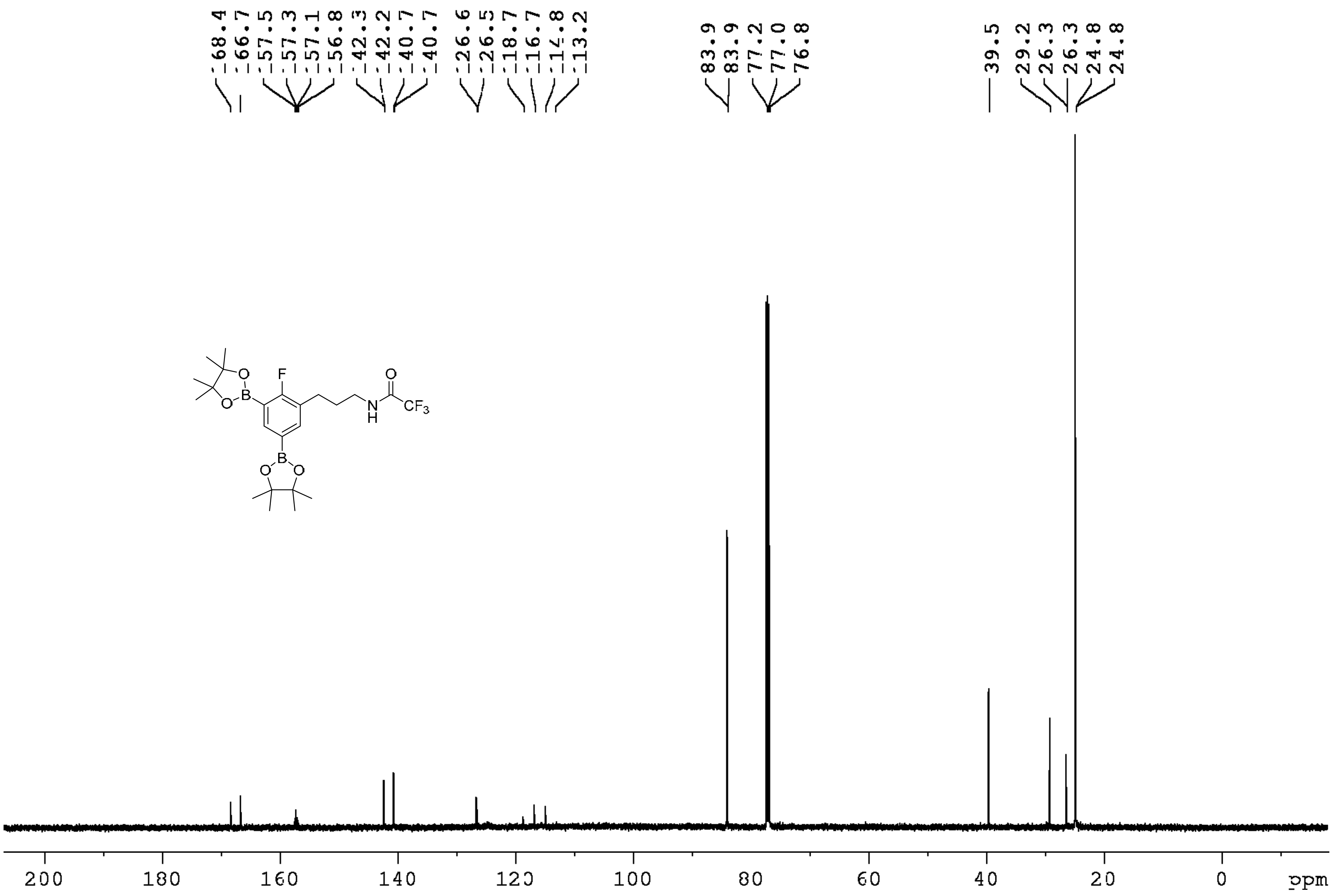

^{1}H NMR (600 MHz, CDCl$_3$) 2,2,2-Trifluoro-*N*-(3-(2-fluoro-3,5-bis(4,4,5,5-tetramethyl-1,3,2-dioxaborolan-2-yl)phenyl)propyl)acetamide (7e) – *para* fraction isolated from reaction with dtbpy

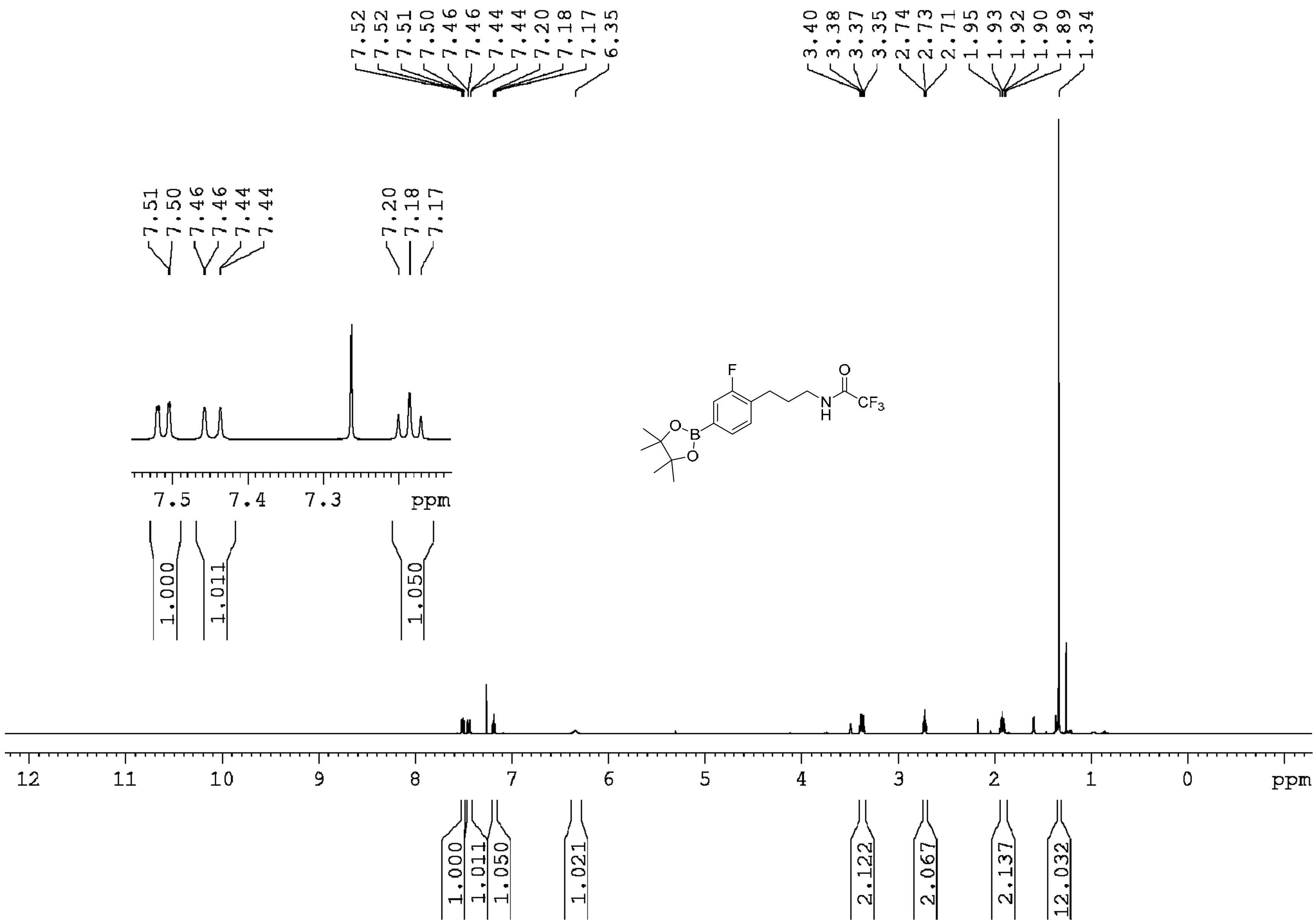

^{13}C NMR (151 MHz, $CDCl_3$) 2,2,2-Trifluoro-*N*-(3-(2-fluoro-3,5-bis(4,4,5,5-tetramethyl-1,3,2-dioxaborolan-2-yl)phenyl)propyl)acetamide (7e) – *para* fraction isolated from reaction with dtbpy

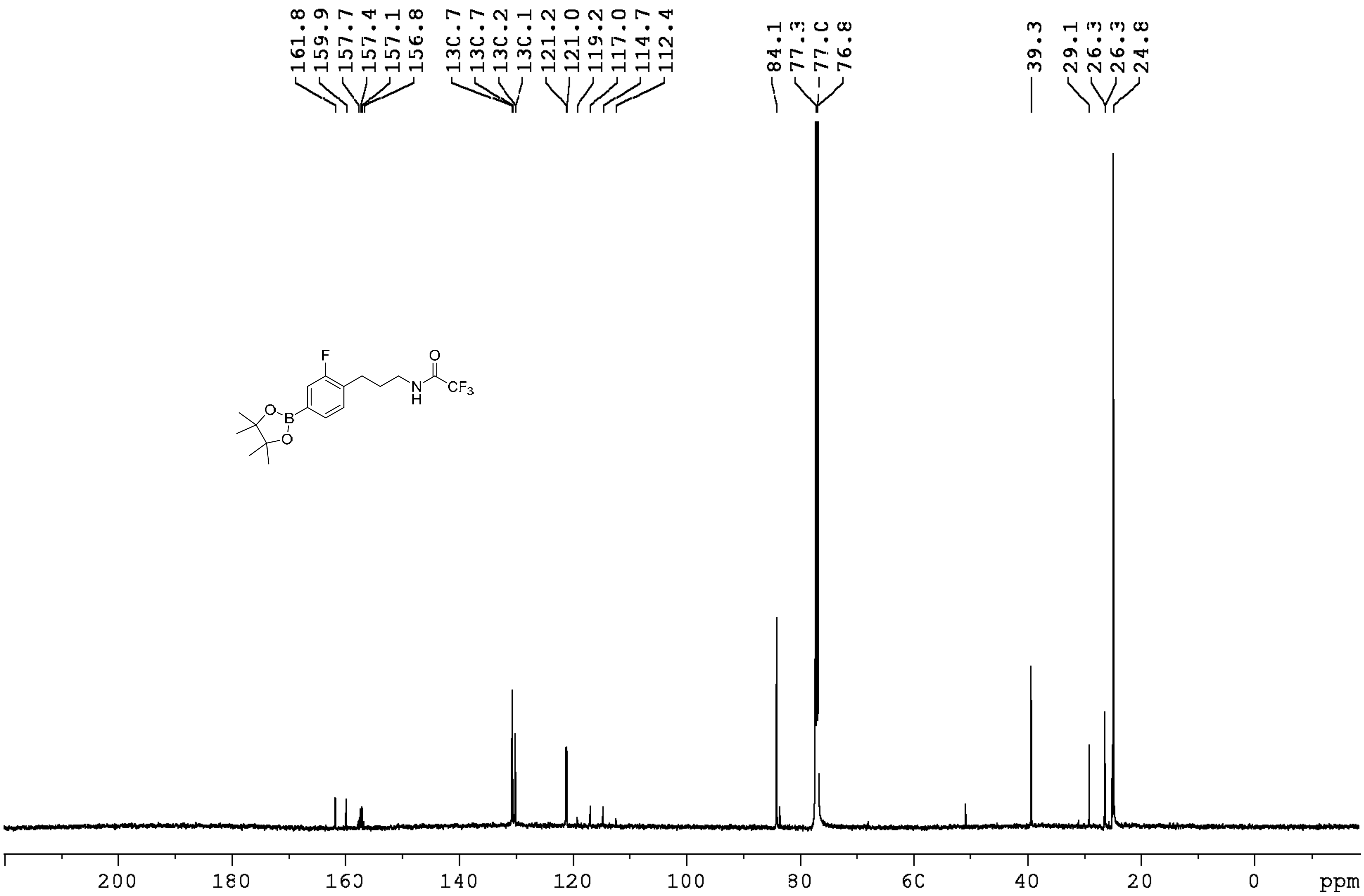

^{1}H NMR (600 MHz, $CDCl_3$) 2,2,2-Trifluoro-*N*-(3-(3-fluoro-5-(4,4,5,5-tetramethyl-1,3,2-dioxaborolan-2-yl)phenyl)propyl)acetamide (7f) – Crude after reaction with 1a

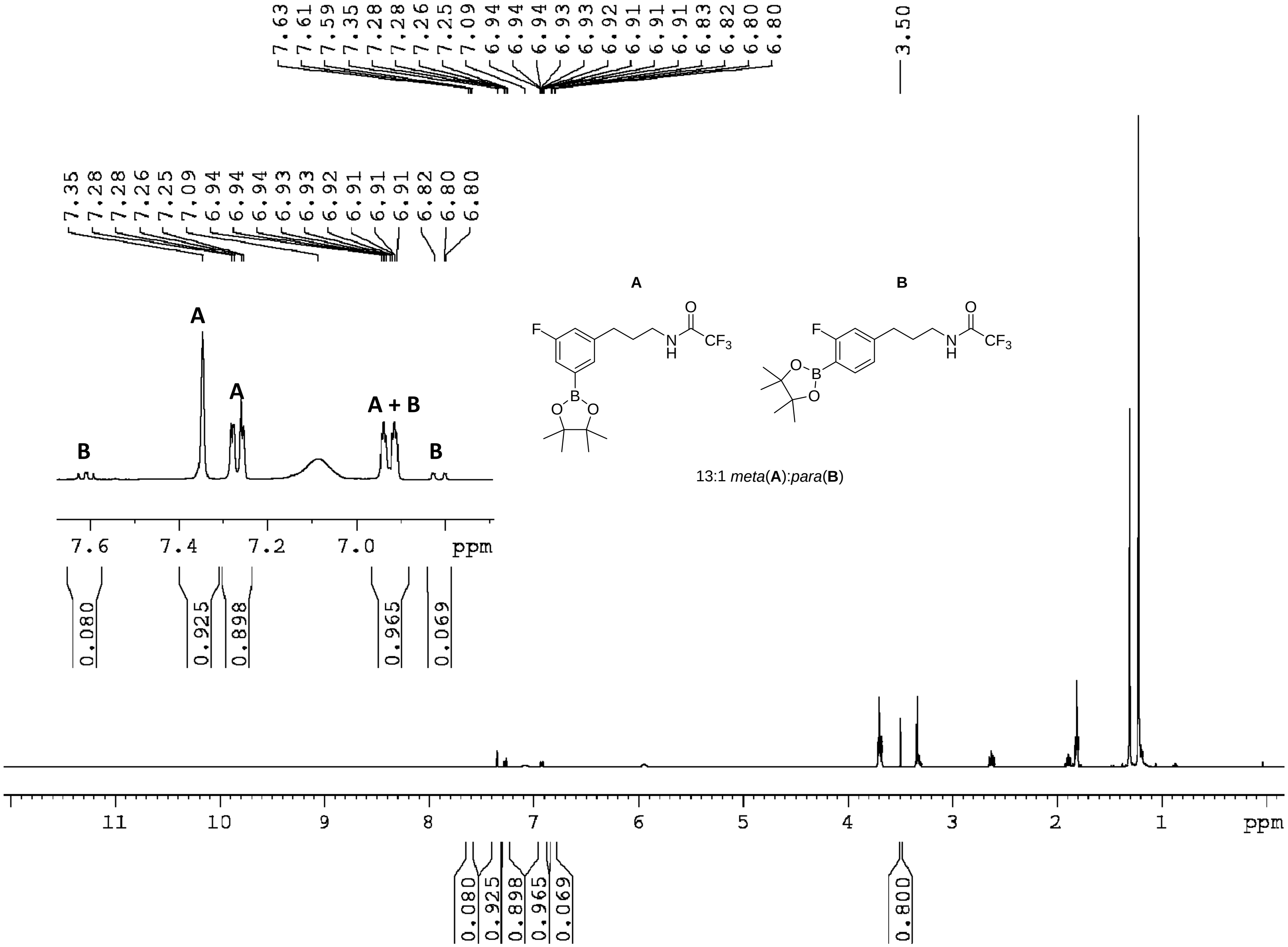

^{1}H NMR (600 MHz, $CDCl_3$) 2,2,2-Trifluoro-*N*-(3-(3-fluoro-5-(4,4,5,5-tetramethyl-1,3,2-dioxaborolan-2-yl)phenyl)propyl)acetamide (7f) – Purified after reaction with 1a

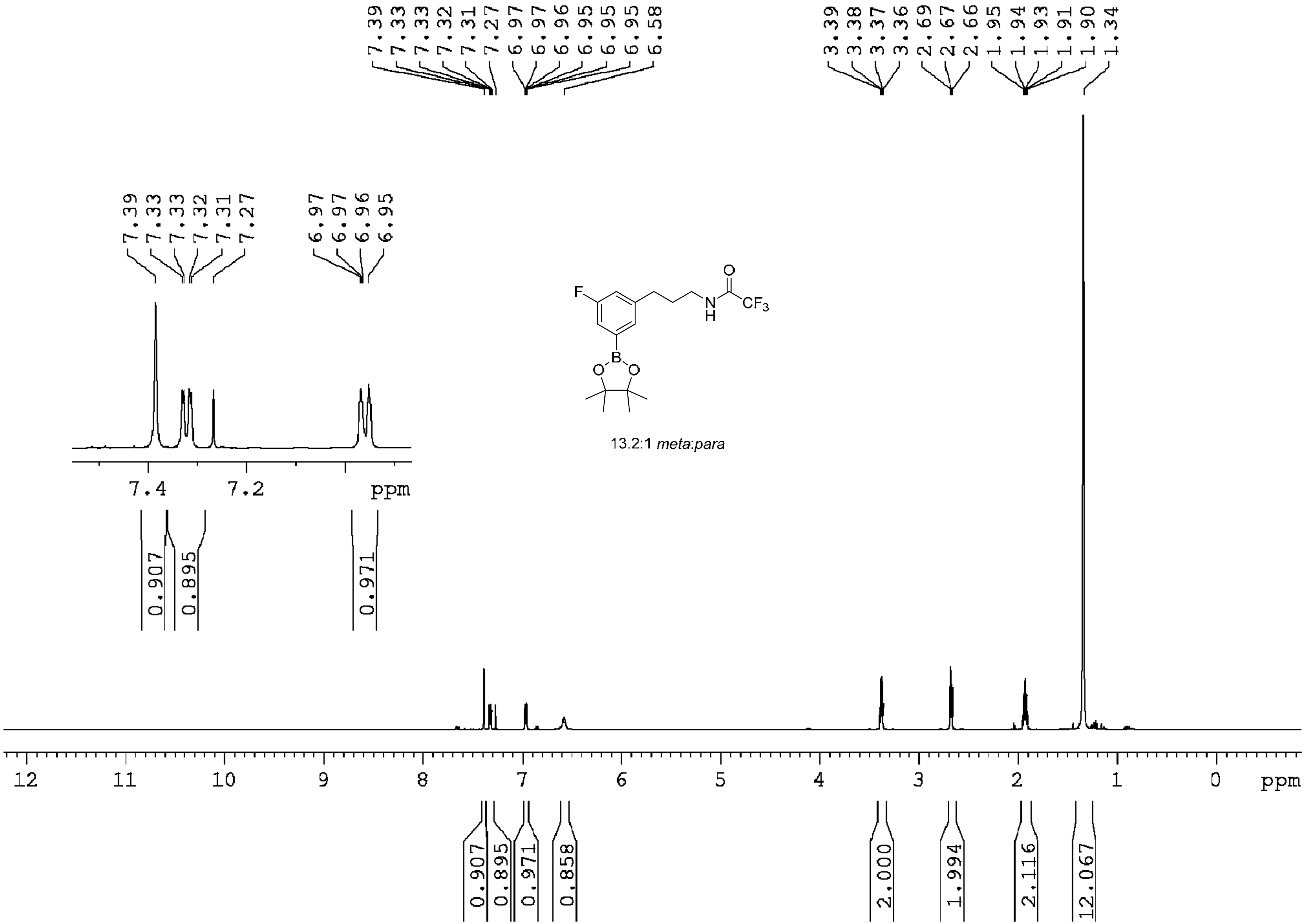

^{13}C NMR (151 MHz, $CDCl_3$) 2,2,2-Trifluoro-*N*-(3-(3-fluoro-5-(4,4,5,5-tetramethyl-1,3,2-dioxaborolan-2-yl)phenyl)propyl)acetamide (7f) – Purified after reaction with 1a

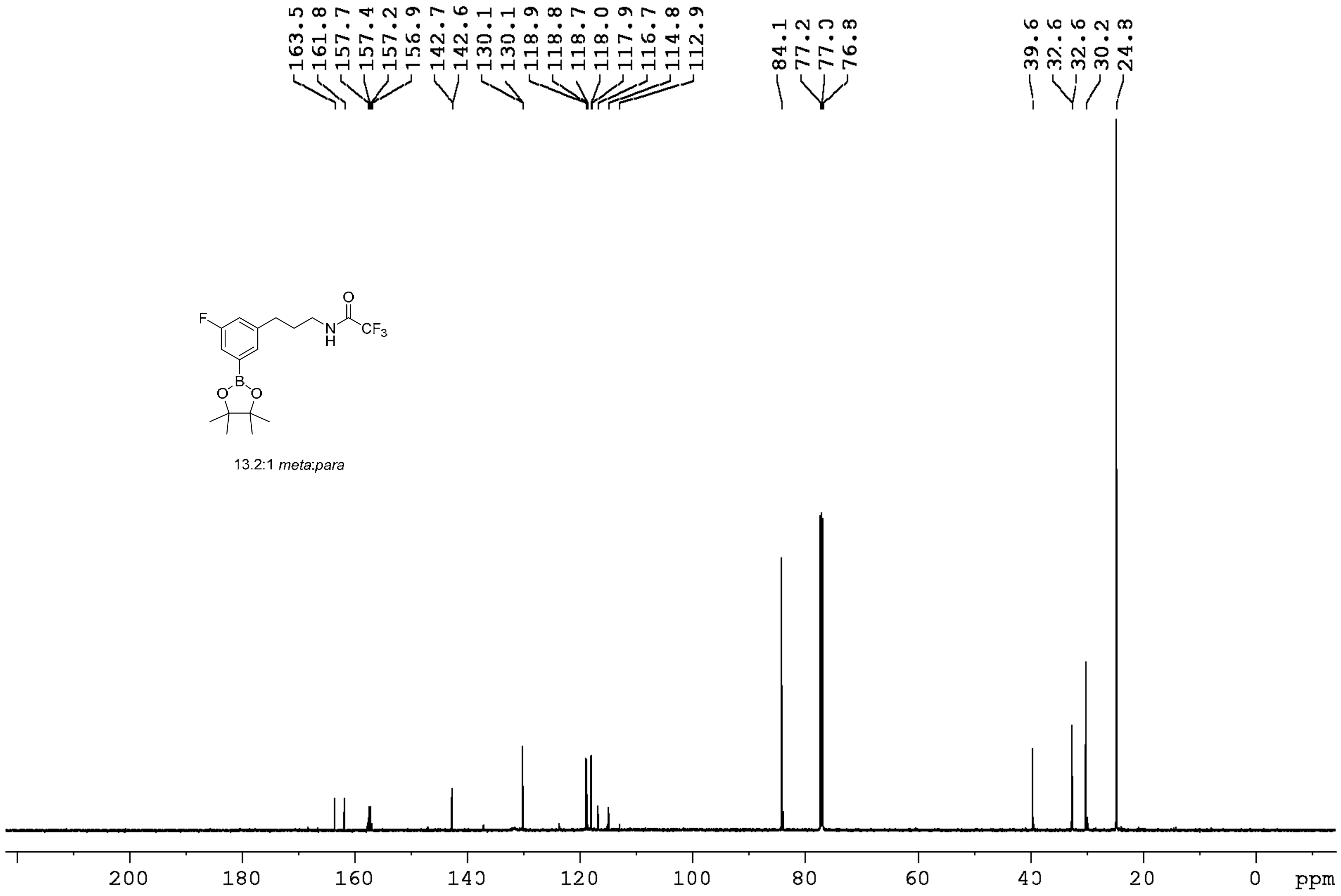

13.2:1 *meta*:*para*

^{1}H NMR (600 MHz, $CDCl_3$) 2,2,2-Trifluoro-*N*-(3-(3-fluoro-5-(4,4,5,5-tetramethyl-1,3,2-dioxaborolan-2-yl)phenyl)propyl)acetamide (7f) – Purified after reaction with dtbpy

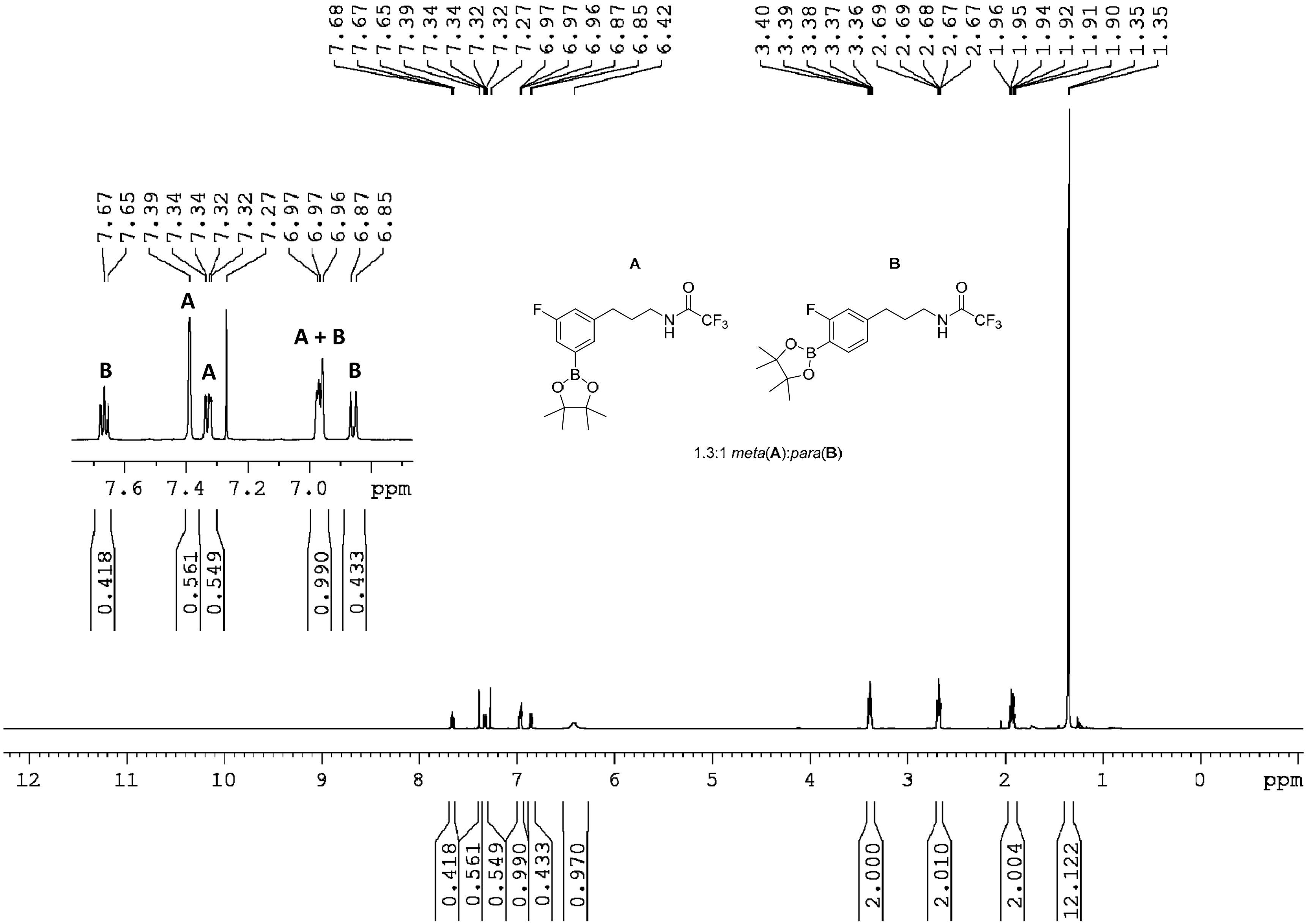

^{13}C NMR (151 MHz, CDCl$_3$) 2,2,2-Trifluoro-*N*-(3-(3-fluoro-5-(4,4,5,5-tetramethyl-1,3,2-dioxaborolan-2-yl)phenyl)propyl)acetamide (7f) – Purified after reaction with dtbpy

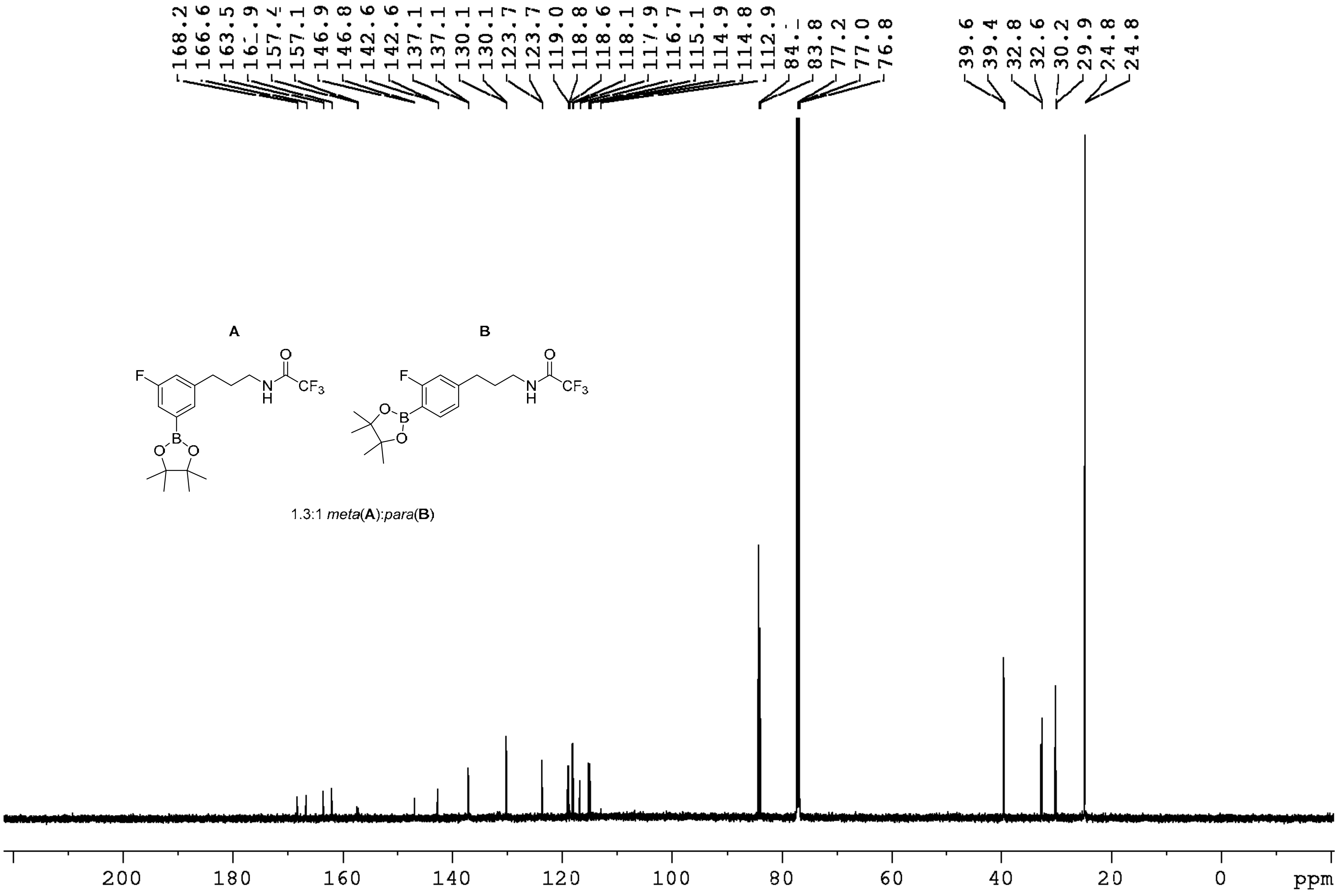

^{1}H NMR (600 MHz, $CDCl_3$) Methyl 4-(4,4,5,5-tetramethyl-1,3,2-dioxaborolan-2-yl)-2-(3-(2,2,2-trifluoroacetamido)propyl)benzoate (7g) – Crude after reaction with 1a

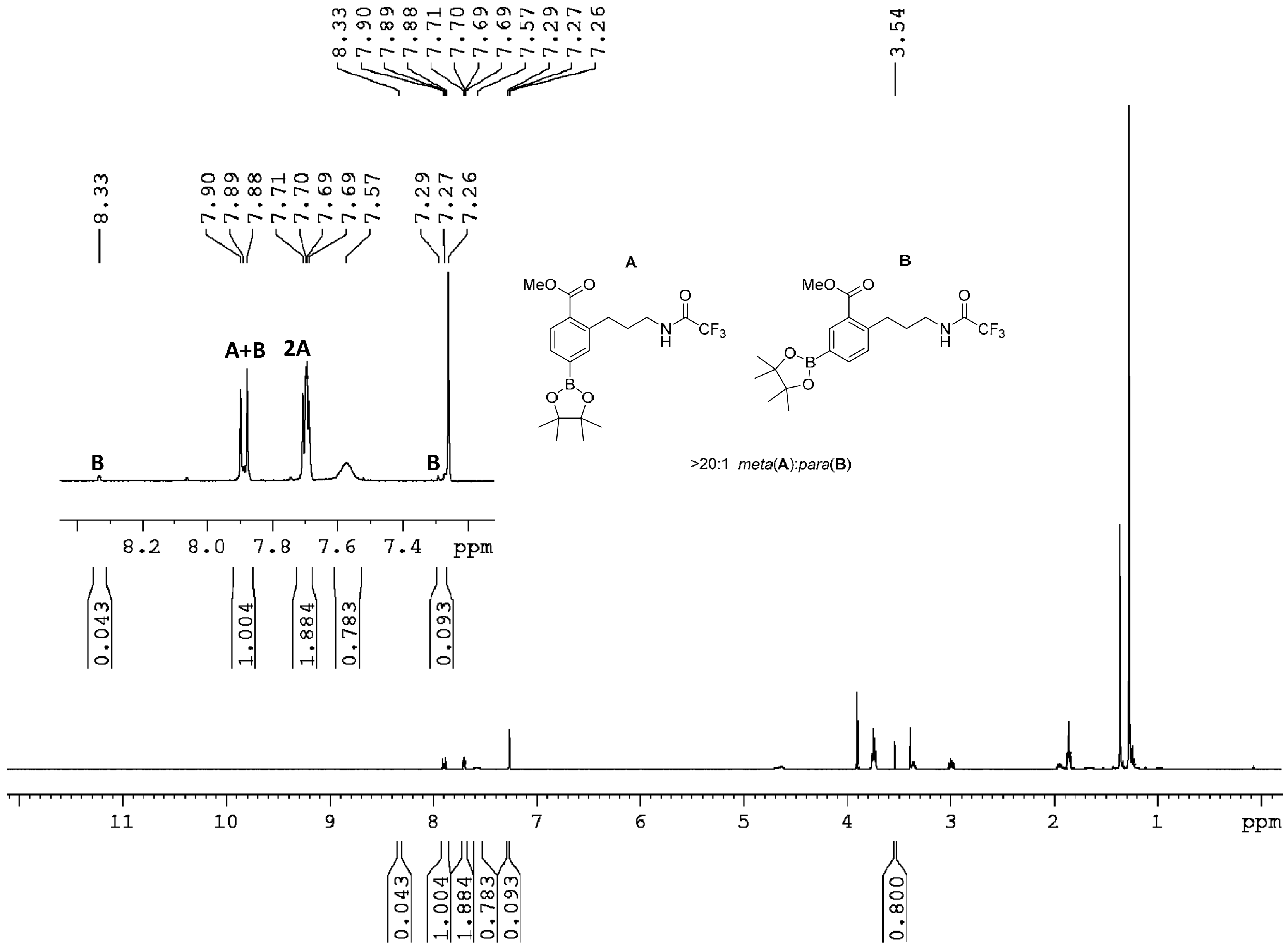

^{1}H NMR (600 MHz, $CDCl_3$) Methyl 4-(4,4,5,5-tetramethyl-1,3,2-dioxaborolan-2-yl)-2-(3-(2,2,2-trifluoroacetamido)propyl)benzoate (7g) – Purified after reaction with 1a

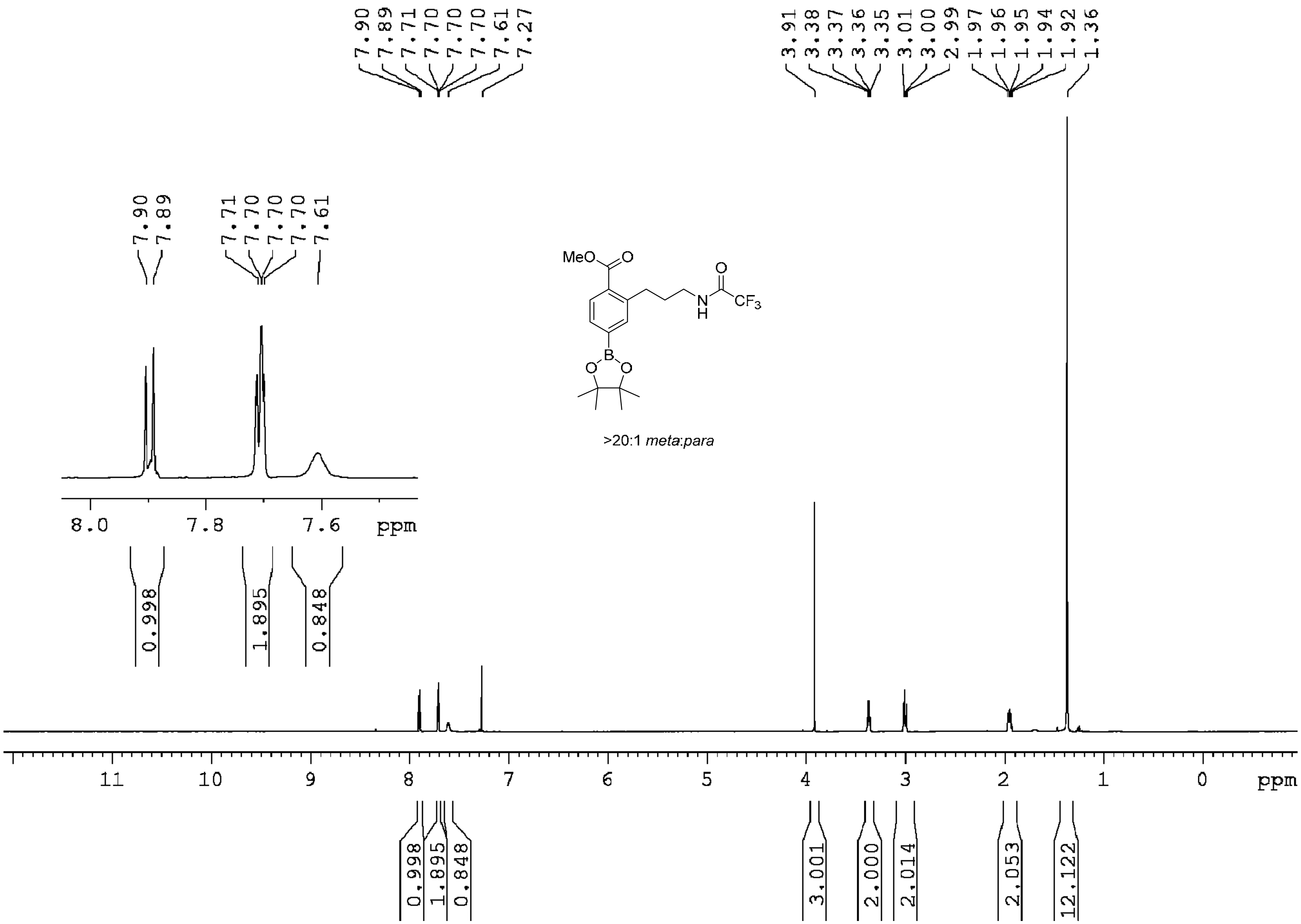

^{13}C NMR (151 MHz, $CDCl_3$) Methyl 4-(4,4,5,5-tetramethyl-1,3,2-dioxaborolan-2-yl)-2-(3-(2,2,2-trifluoroacetamido)propyl)benzoate (7g) – Purified after reaction with 1a

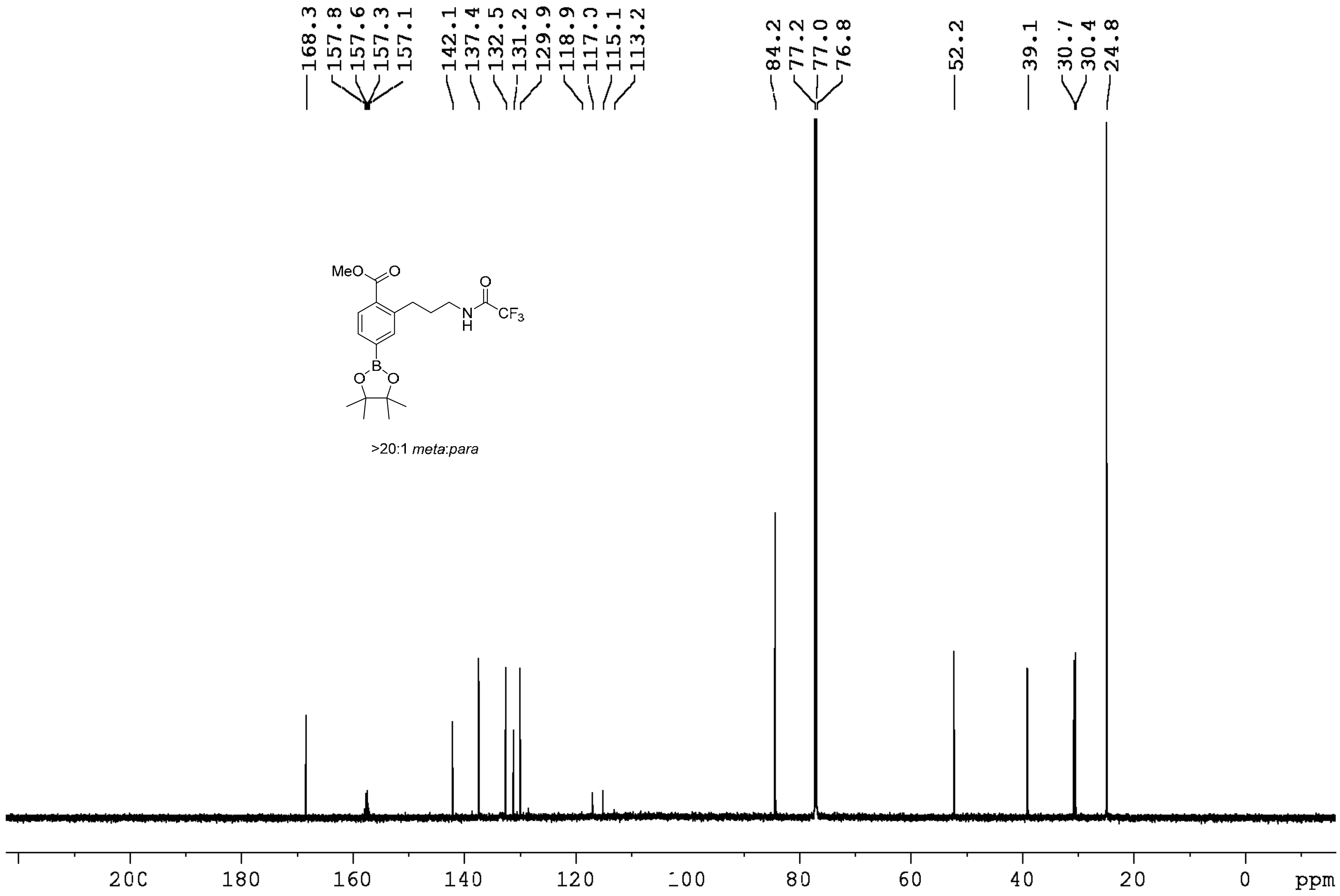

^{1}H NMR (500 MHz, $CDCl_3$) Methyl 4-(4,4,5,5-tetramethyl-1,3,2-dioxaborolan-2-yl)-2-(3-(2,2,2-trifluoroacetamido)propyl)benzoate (7g) – Purified after reaction with dtbpy

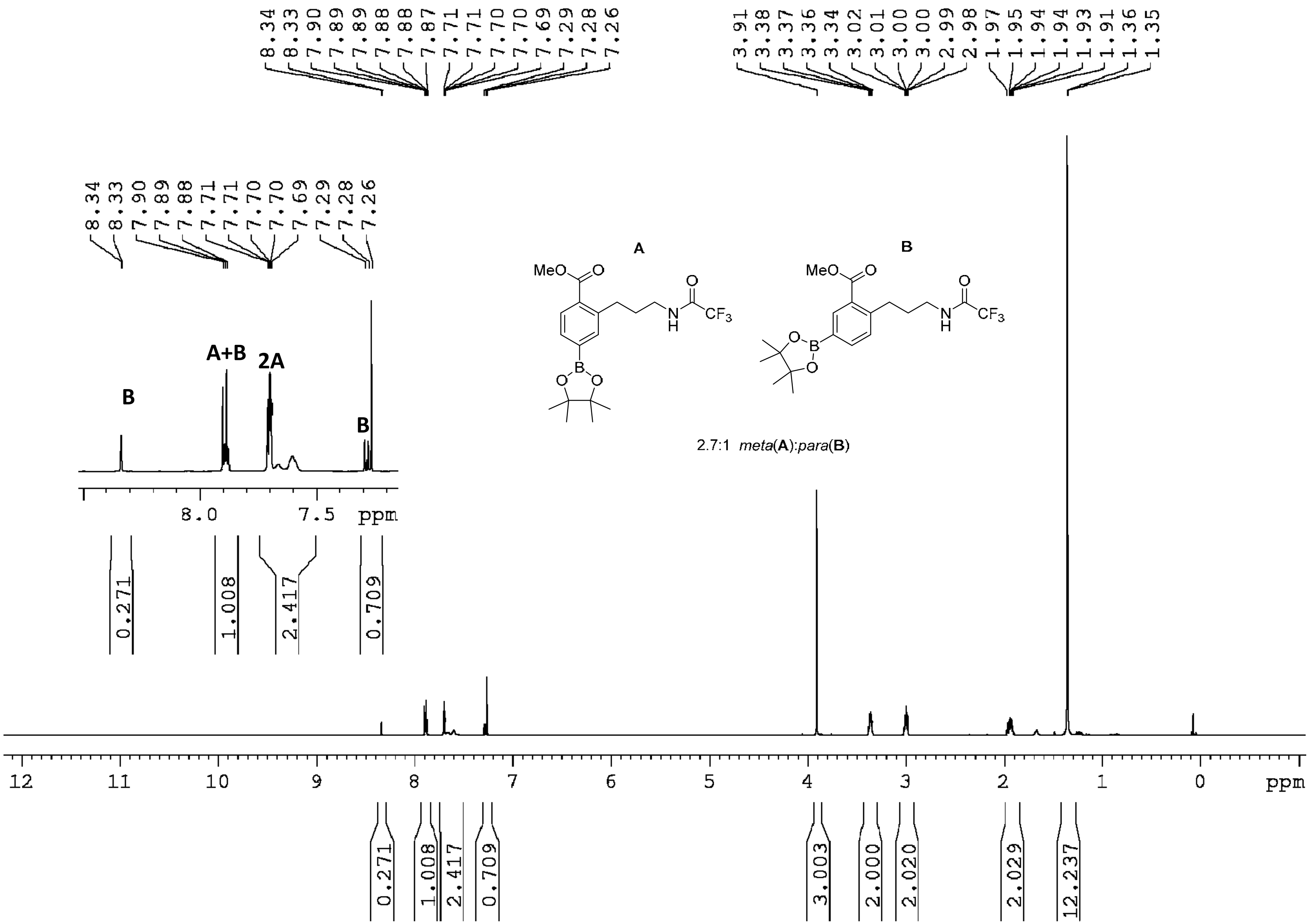

^{13}C NMR (126 MHz, $CDCl_3$) Methyl 4-(4,4,5,5-tetramethyl-1,3,2-dioxaborolan-2-yl)-2-(3-(2,2,2-trifluoroacetamido)propyl)benzoate (7g) – Purified after reaction with dtbpy

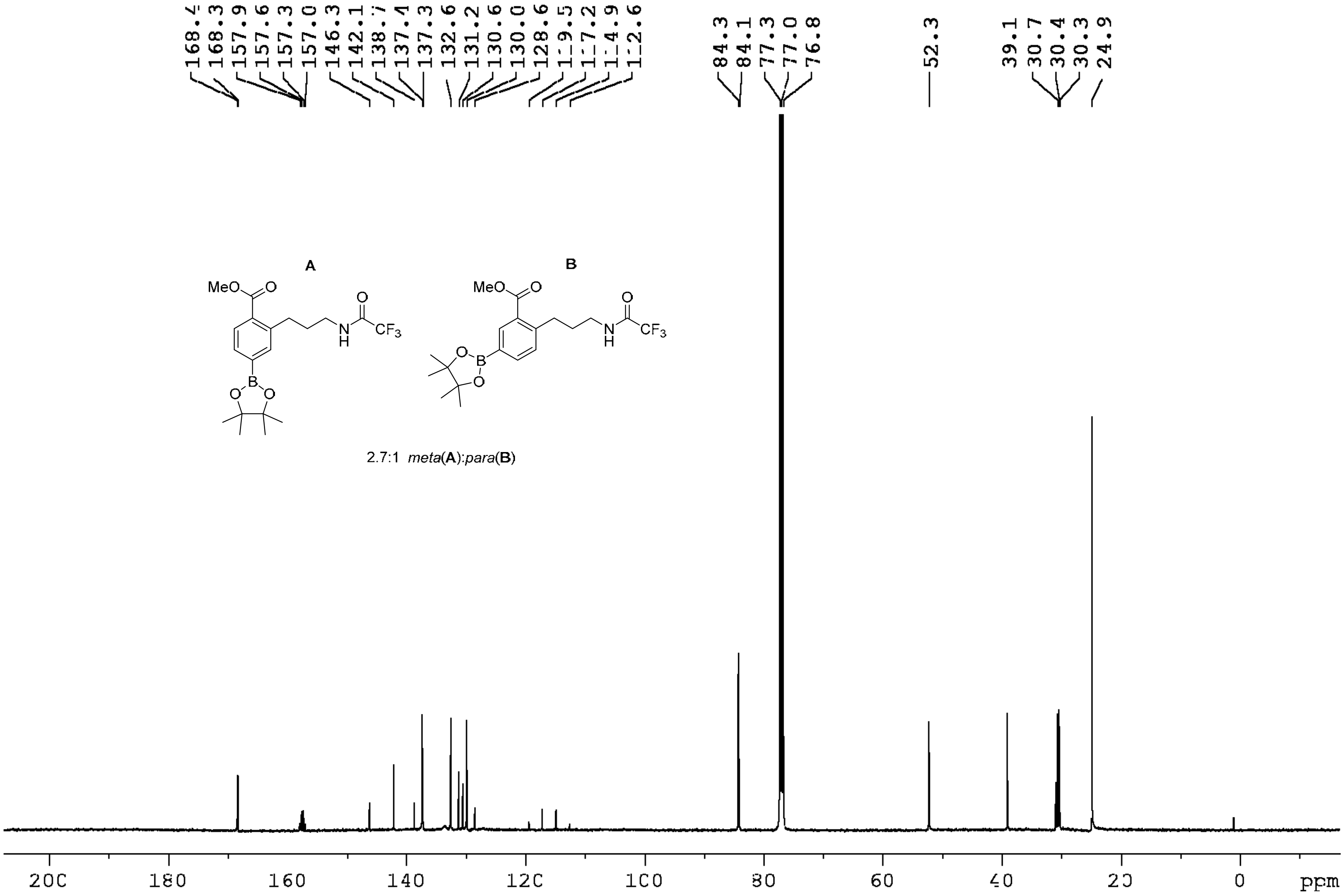

^{1}H NMR (600 MHz, $CDCl_3$) *N*-(3-(2,5-difluoro-3-(4,4,5,5-tetramethyl-1,3,2-dioxaborolan-2-yl)phenyl)propyl)-2,2,2-trifluoroacetamide (7h) – Crude after reaction with 1a

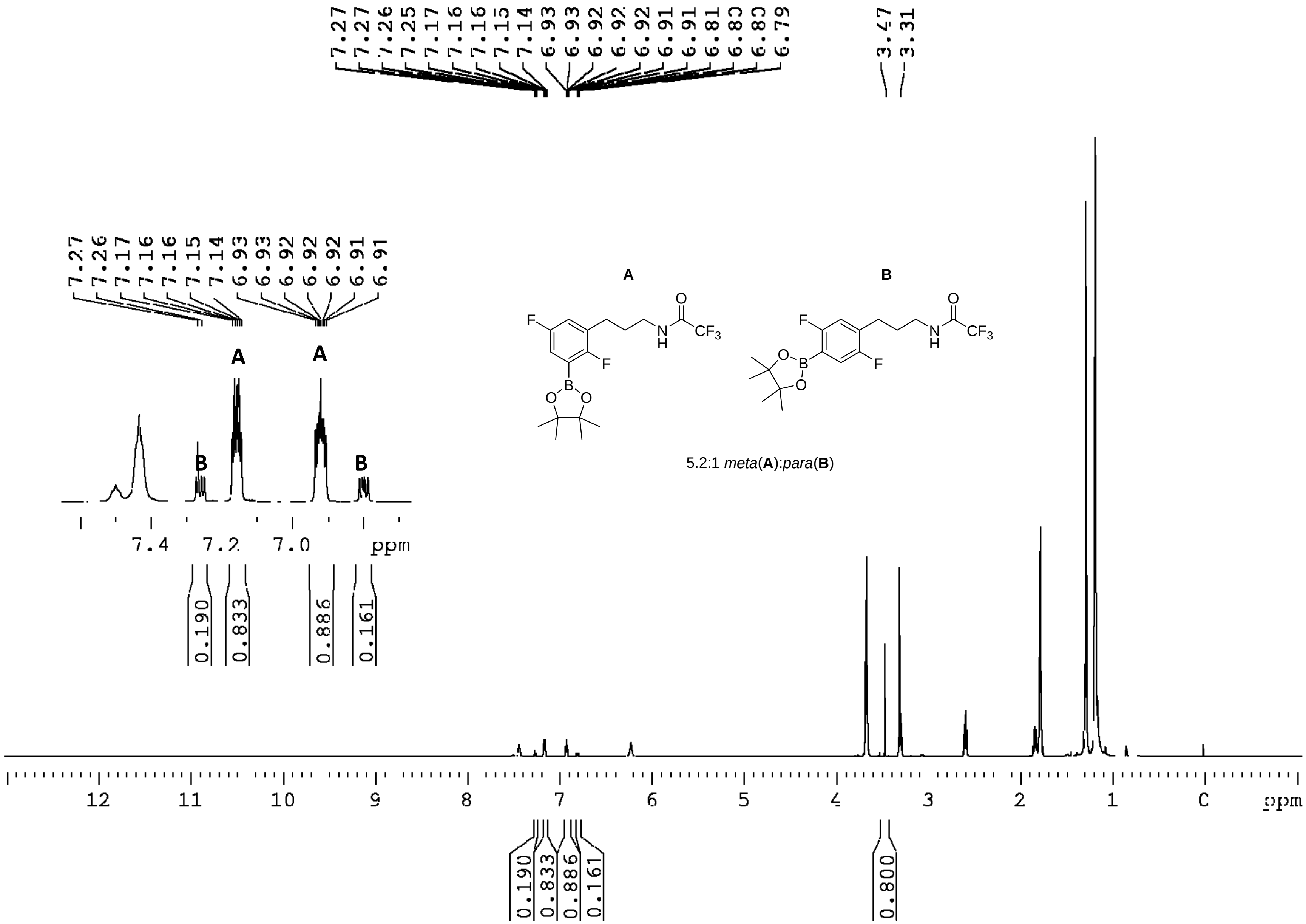

^{1}H NMR (600 MHz, $CDCl_3$) *N*-(3-(2,5-difluoro-3-(4,4,5,5-tetramethyl-1,3,2-dioxaborolan-2-yl)phenyl)propyl)-2,2,2-trifluoroacetamide (7h) – Purified after reaction with 1a

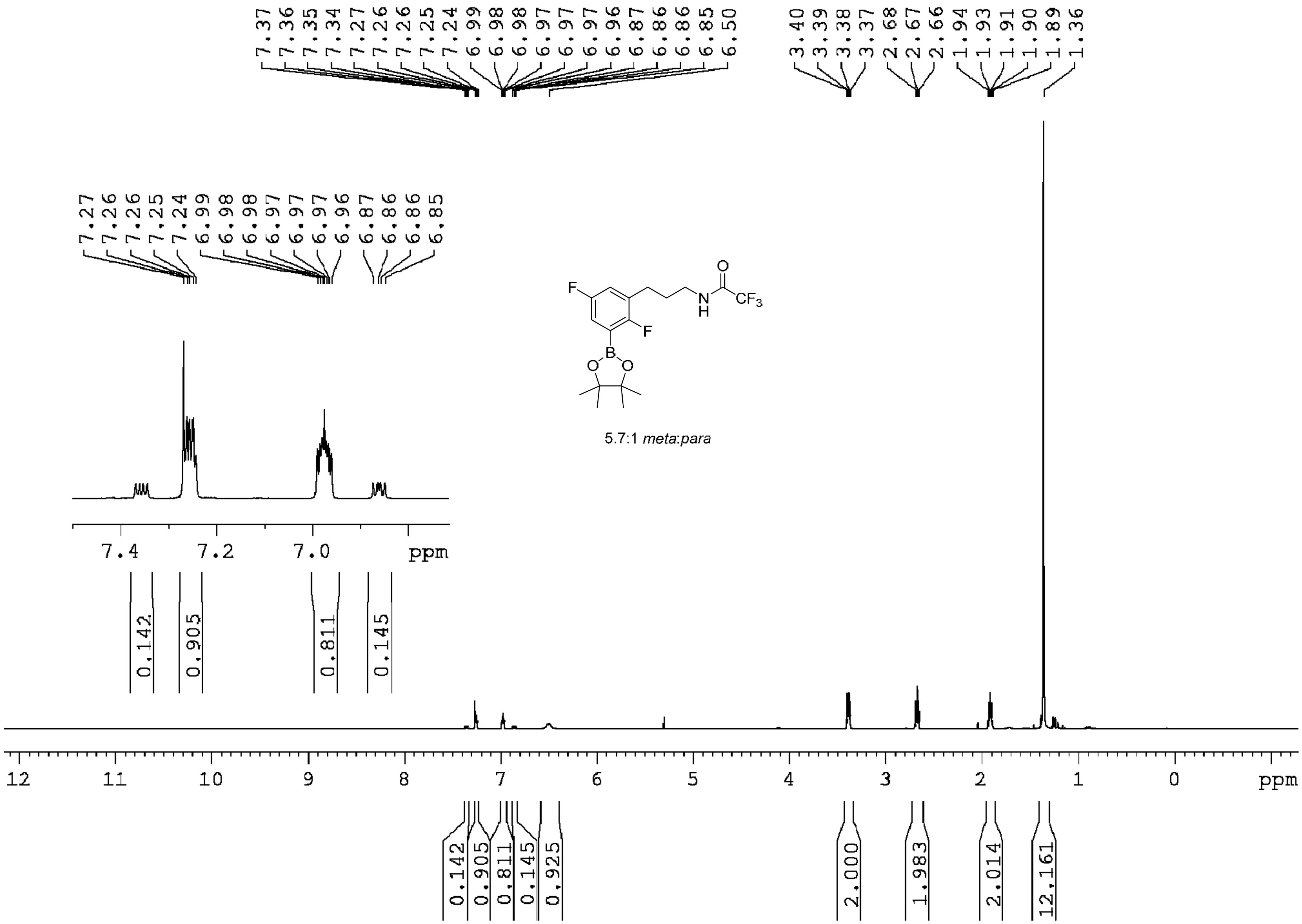

^{13}C NMR (151 MHz, $CDCl_3$) *N*-(3-(2,5-difluoro-3-(4,4,5,5-tetramethyl-1,3,2-dioxaborolan-2-yl)phenyl)propyl)-2,2,2-trifluoroacetamide (7h) – Purified after reaction with 1a

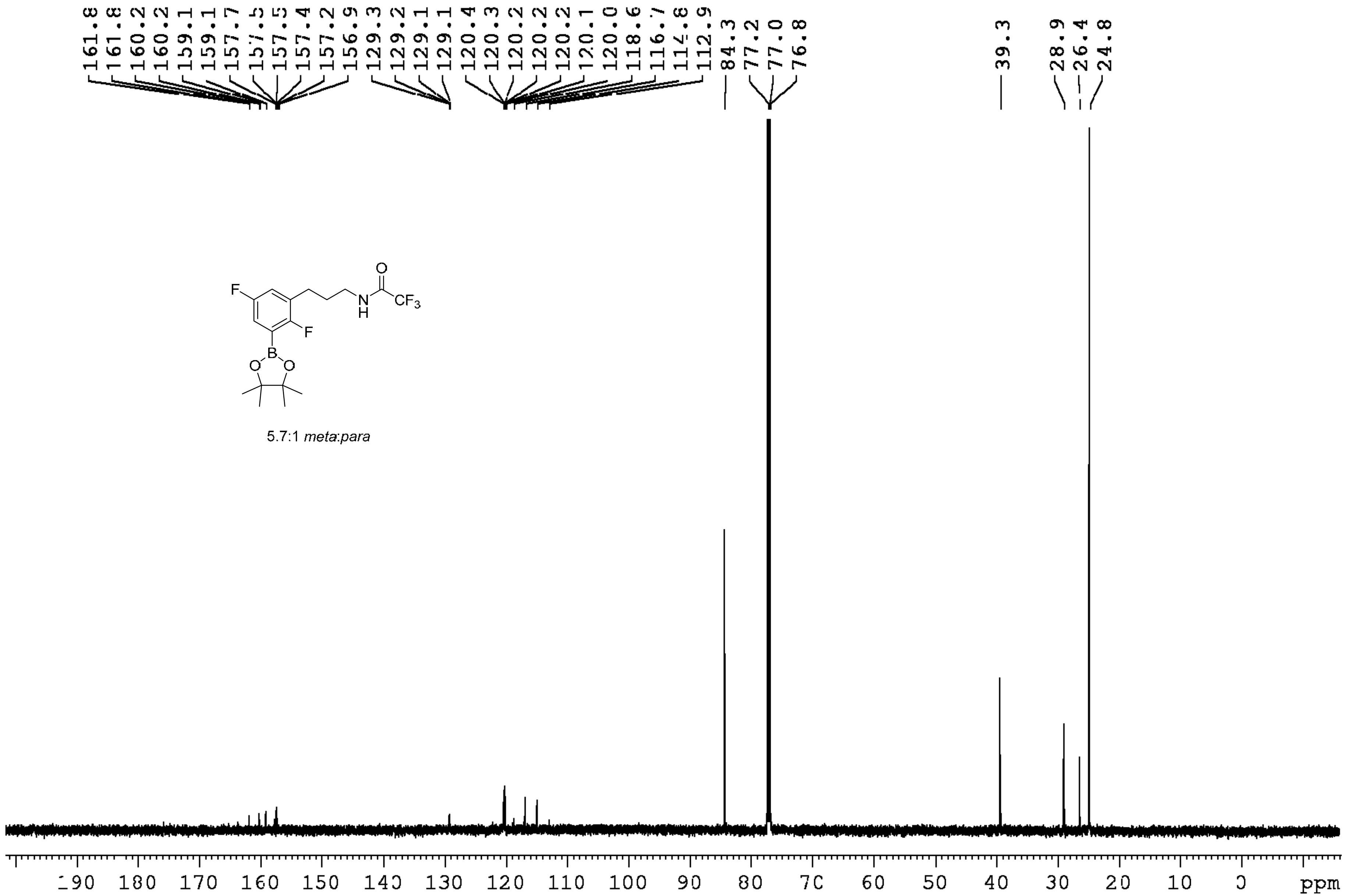

^{1}H NMR (600 MHz, CDCl$_3$) *N*-(3-(2,5-difluoro-3-(4,4,5,5-tetramethyl-1,3,2-dioxaborolan-2-yl)phenyl)propyl)-2,2,2-trifluoroacetamide (7h) – Purified after reaction with dtbpy

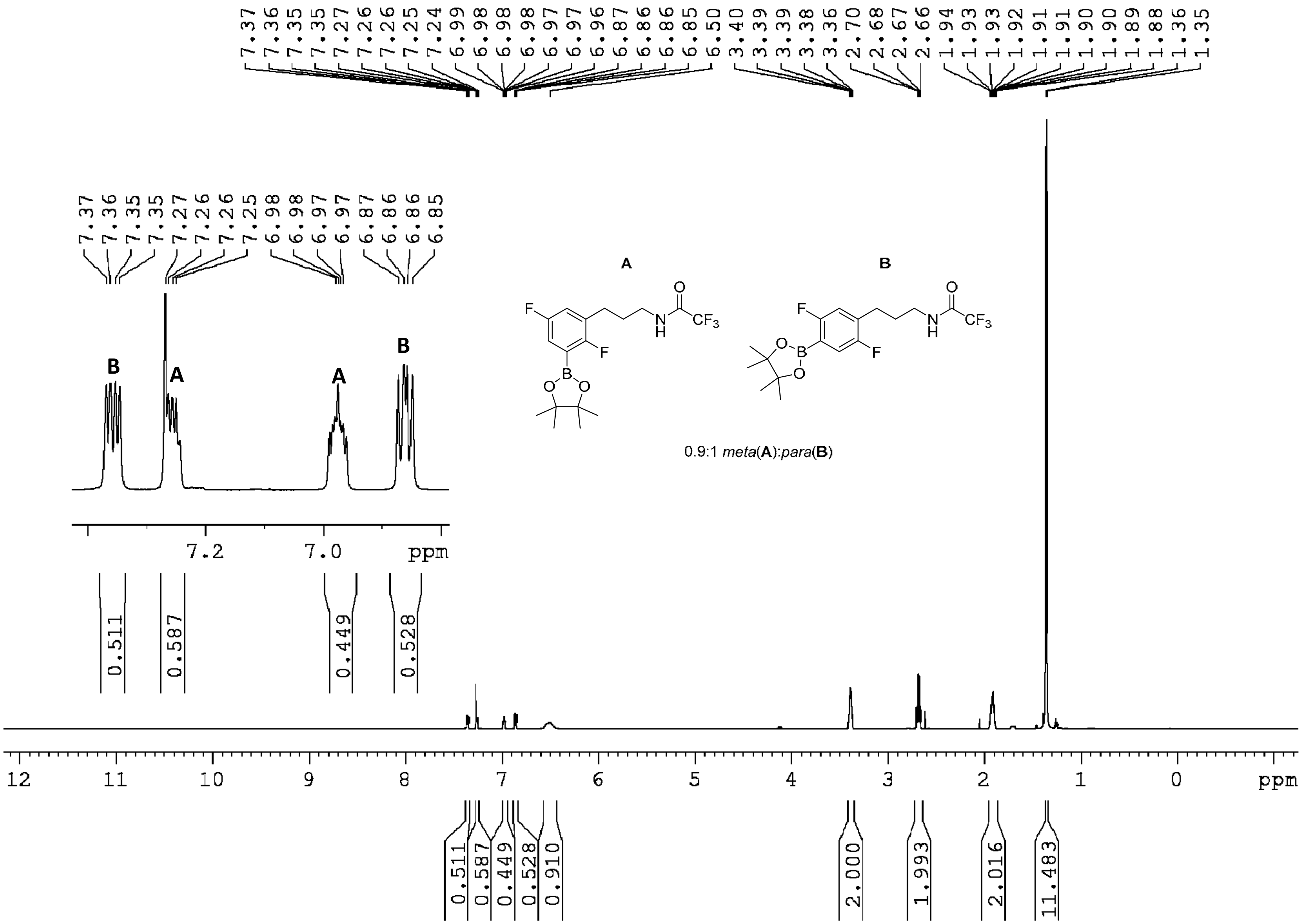

^{13}C NMR (151 MHz, $CDCl_3$) *N*-(3-(2,5-difluoro-3-(4,4,5,5-tetramethyl-1,3,2-dioxaborolan-2-yl)phenyl)propyl)-2,2,2-trifluoroacetamide (7h) – Purified after reaction with dtbpy

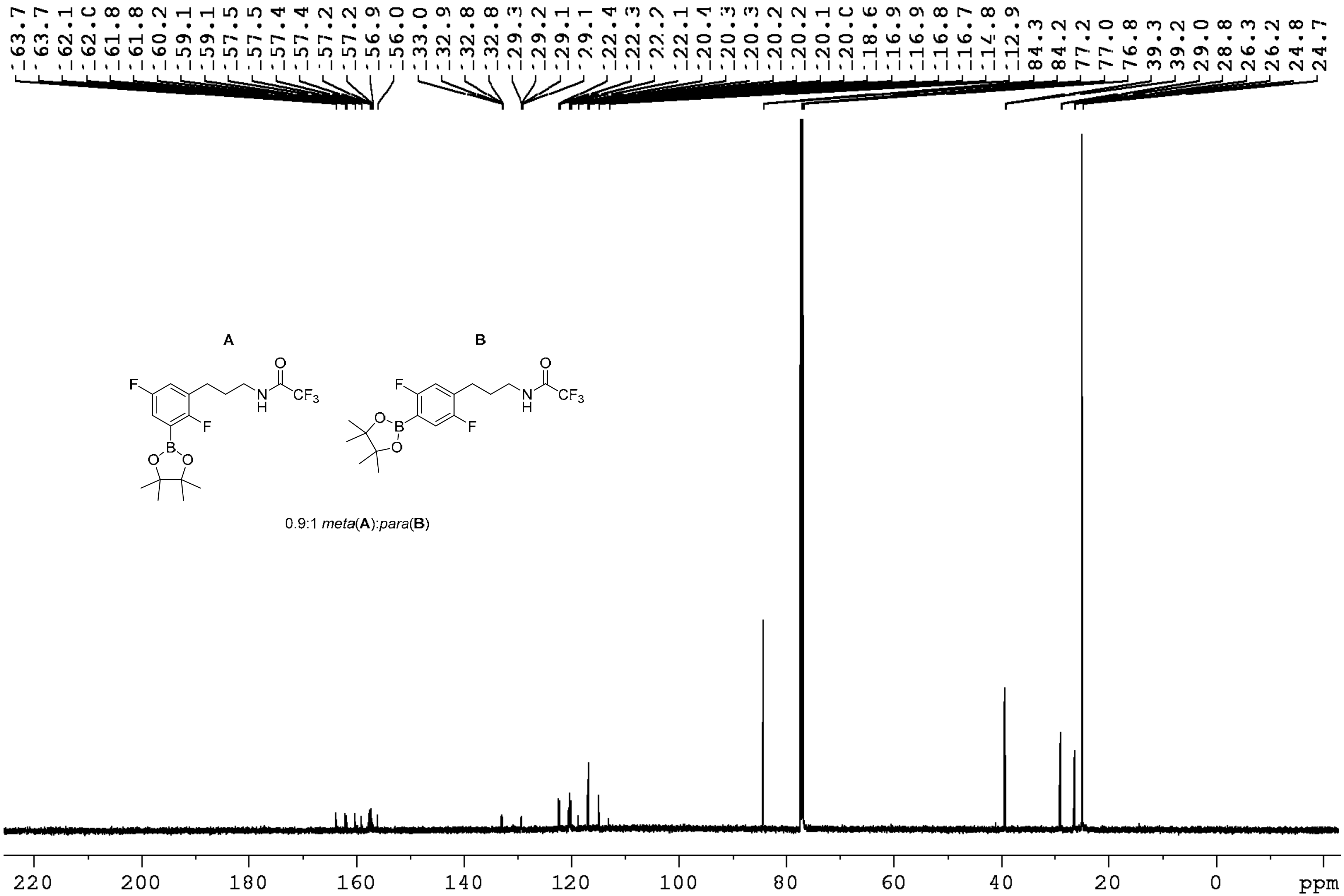

^{1}H NMR (500 MHz, $CDCl_3$) 2,2,2-Trifluoro-*N*-(3-(2-methyl-5-(4,4,5,5-tetramethyl-1,3,2-dioxaborolan-2-yl)phenyl)propyl)acetamide (7i) – Crude after reaction with 1a

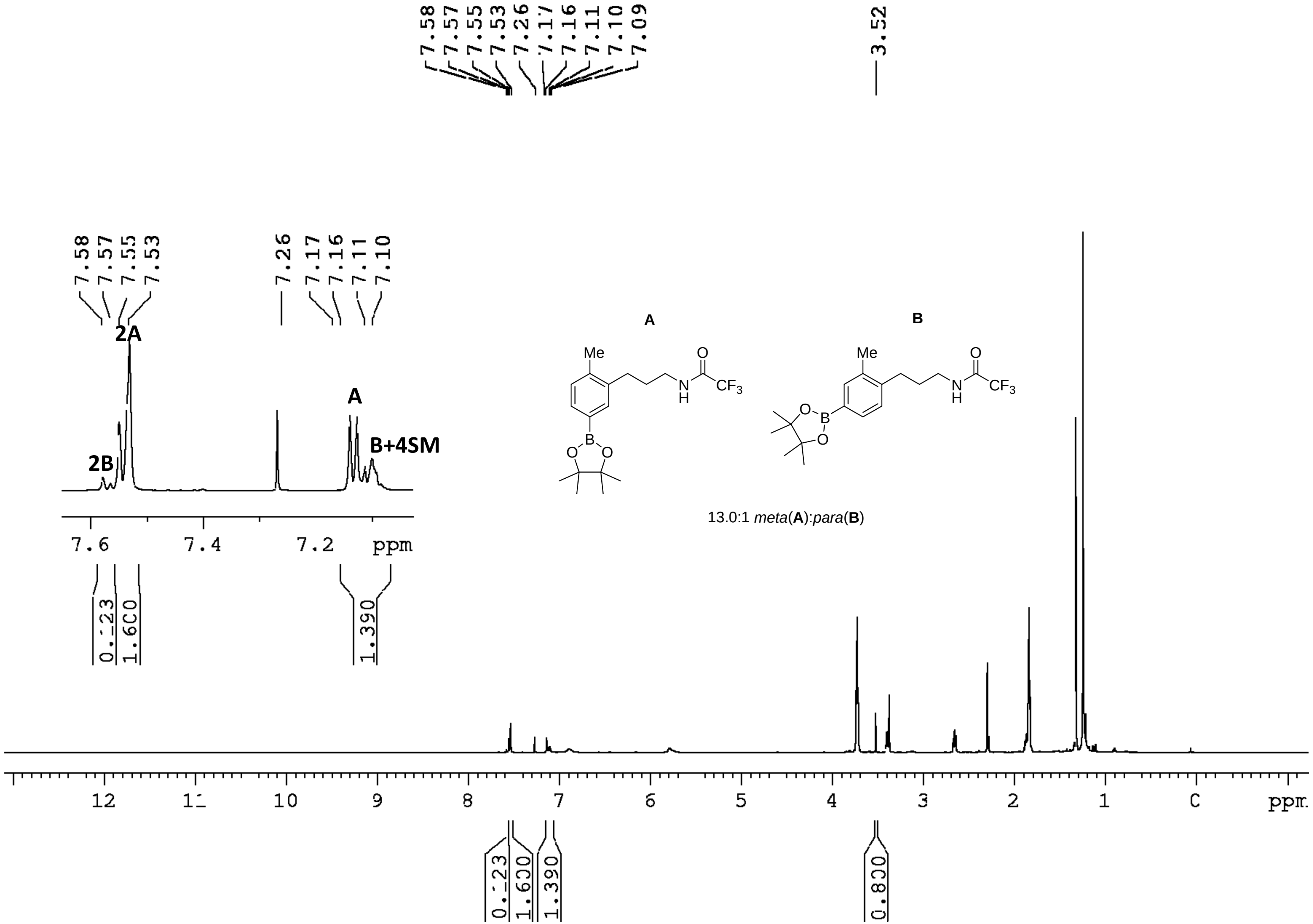

^{1}H NMR (500 MHz, $CDCl_3$) 2,2,2-Trifluoro-*N*-(3-(2-methyl-5-(4,4,5,5-tetramethyl-1,3,2-dioxaborolan-2-yl)phenyl)propyl)acetamide (7i) – Purified after reaction with 1a

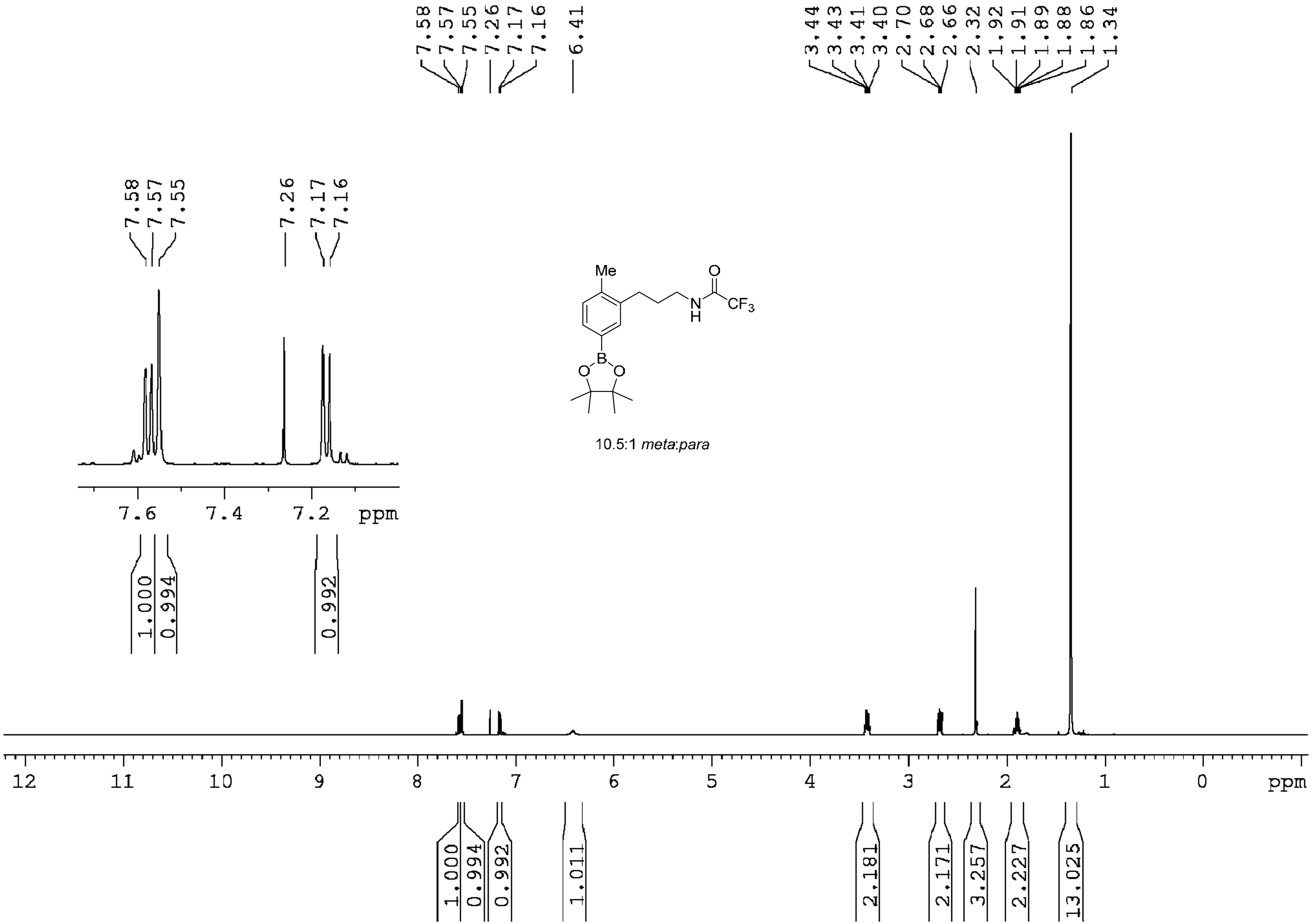

10.5:1 *meta:para*

^{13}C NMR (126 MHz, CDCl$_3$) 2,2,2-Trifluoro-*N*-(3-(2-methyl-5-(4,4,5,5-tetramethyl-1,3,2-dioxaborolan-2-yl)phenyl)propyl)acetamide (7i) – Purified after reaction with 1a

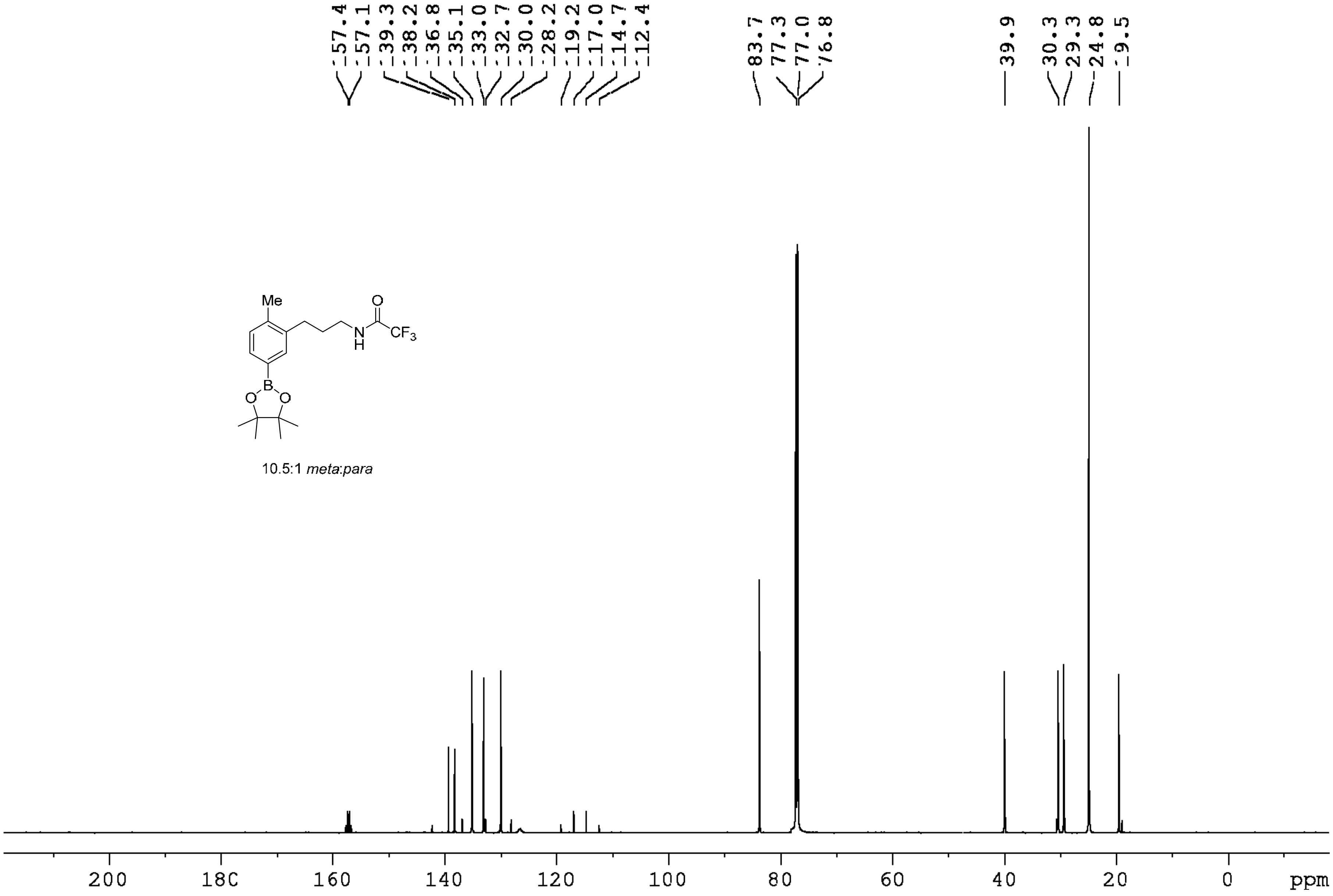

10.5:1 *meta*:*para*

^{1}H NMR (500 MHz, $CDCl_3$) 2,2,2-Trifluoro-*N*-(3-(2-methyl-5-(4,4,5,5-tetramethyl-1,3,2-dioxaborolan-2-yl)phenyl)propyl)acetamide (7i) – Purified after reaction with dtbpy

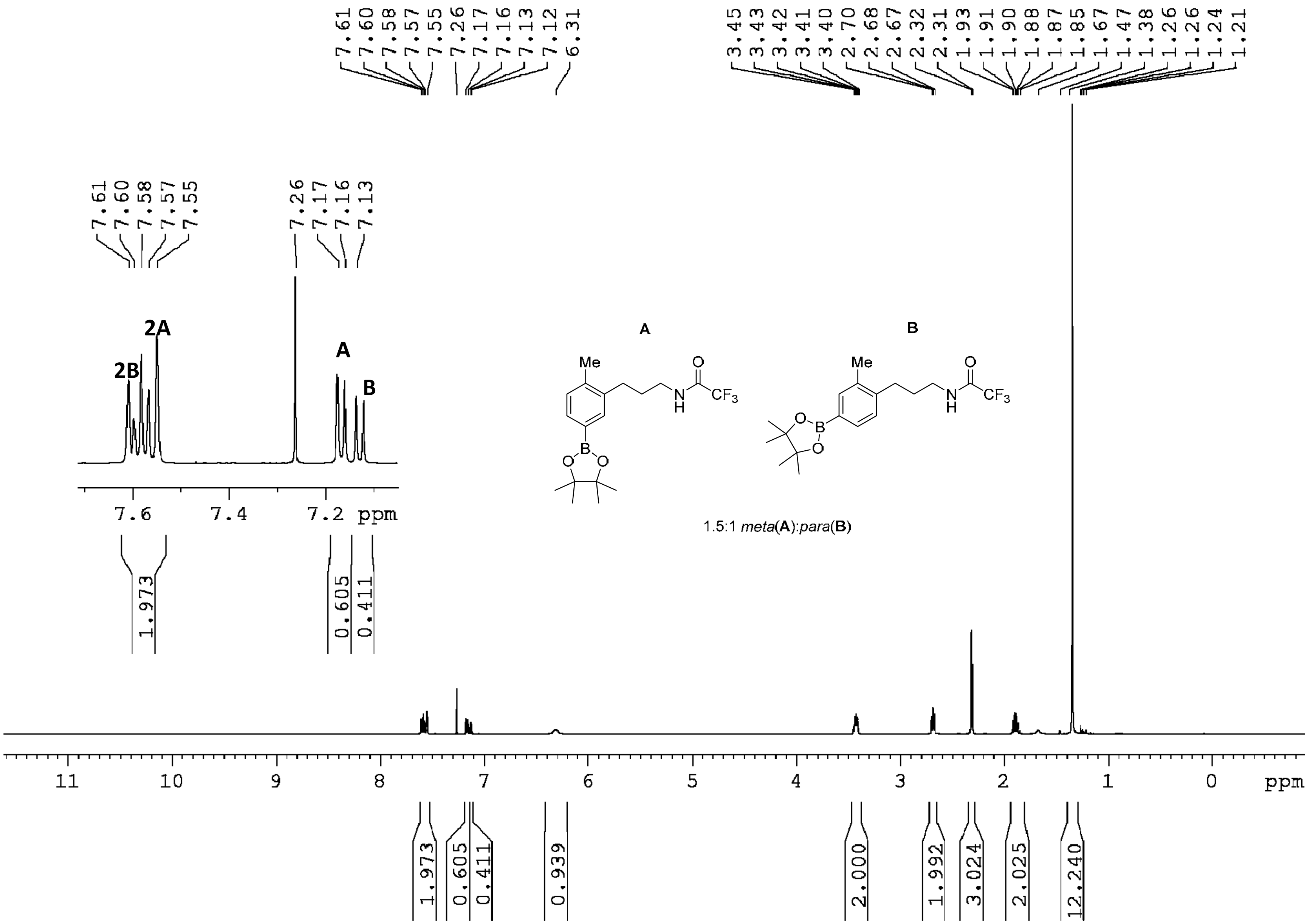

^{13}C NMR (126 MHz, CDCl$_3$) 2,2,2-Trifluoro-*N*-(3-(2-methyl-5-(4,4,5,5-tetramethyl-1,3,2-dioxaborolan-2-yl)phenyl)propyl)acetamide (7i) – Purified after reaction with dtbpy

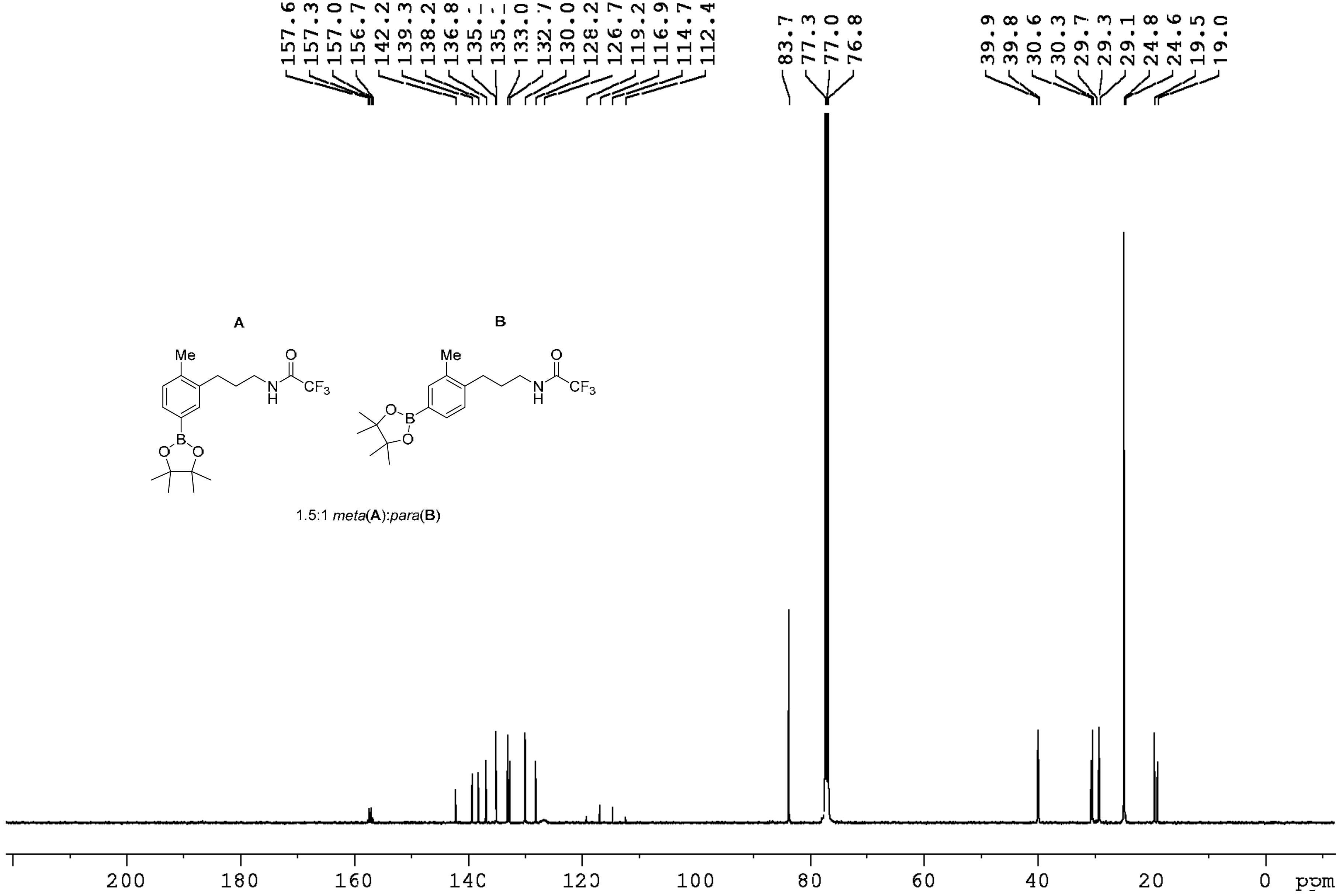

^{1}H NMR (600 MHz, $CDCl_3$) 2,2,2-Trifluoro-*N*-(3-(2-methoxy-5-(4,4,5,5-tetramethyl-1,3,2-dioxaborolan-2-yl)phenyl)propyl)acetamide (7j) – Purified after reaction with 1a

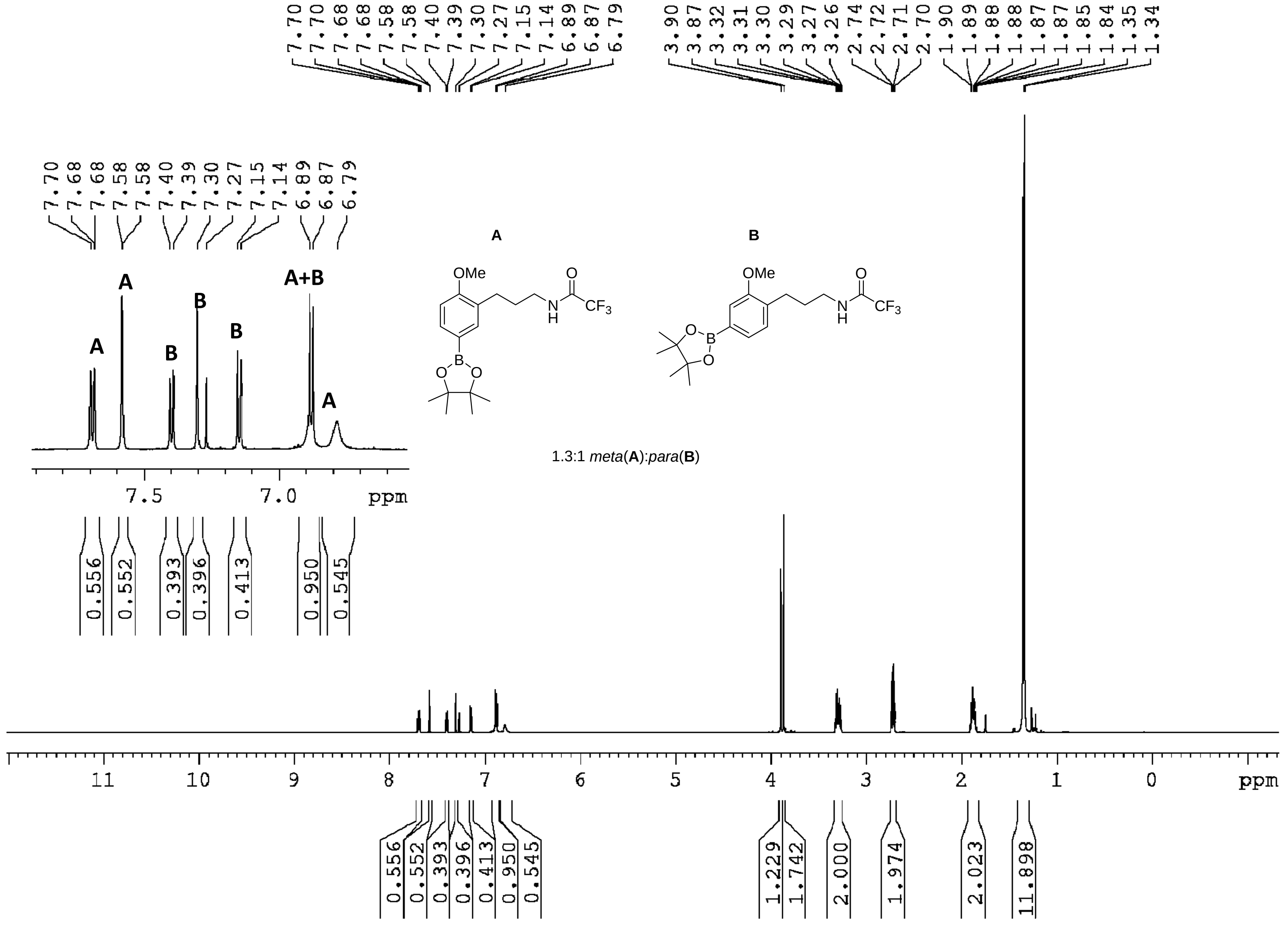

^{13}C NMR (151 MHz, $CDCl_3$) 2,2,2-Trifluoro-*N*-(3-(2-methoxy-5-(4,4,5,5-tetramethyl-1,3,2-dioxaborolan-2-yl)phenyl)propyl)acetamide (7j) – Purified after reaction with 1a

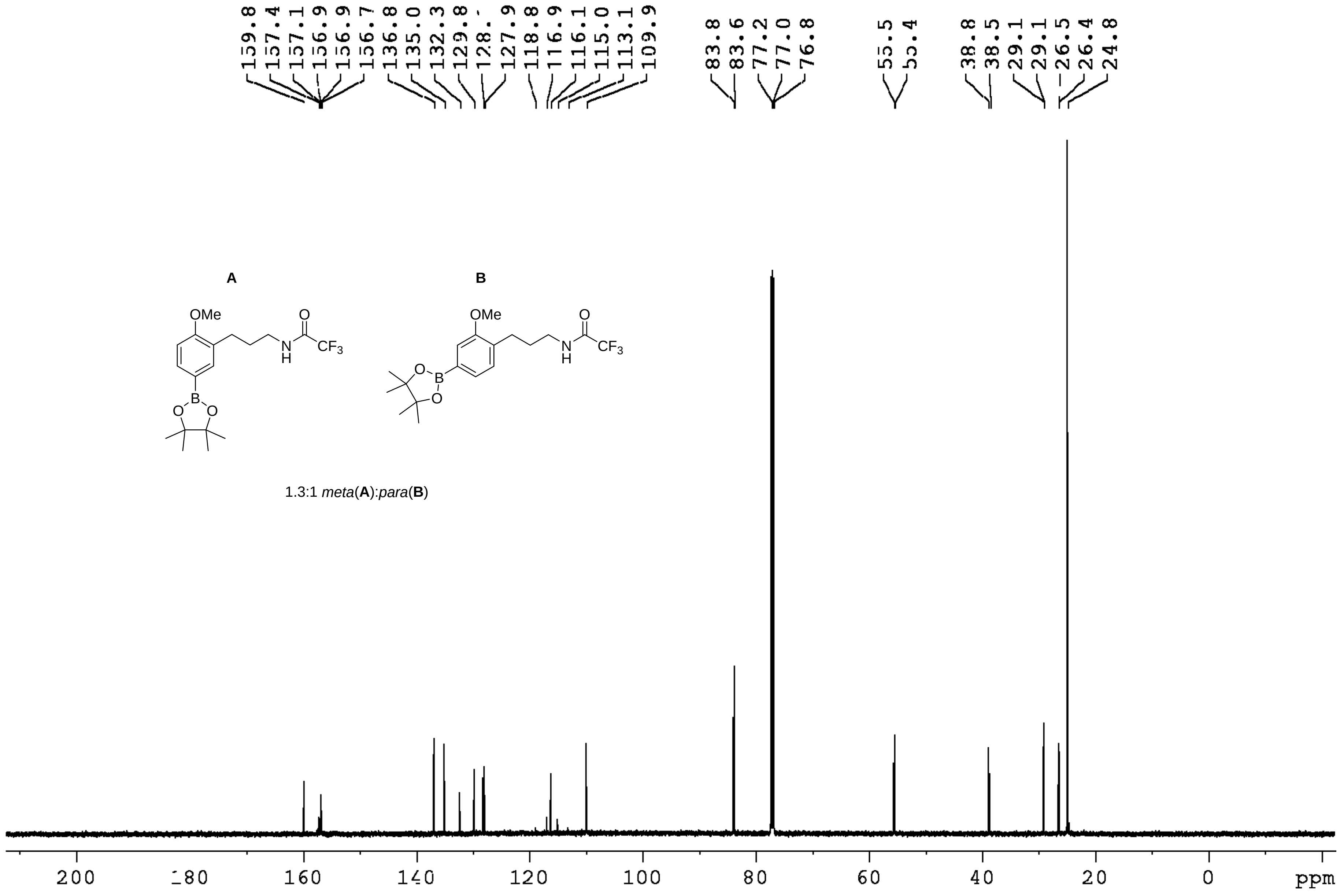

^{1}H NMR (600 MHz, $CDCl_3$) 2,2,2-Trifluoro-*N*-(3-(4'-methyl-4-(4,4,5,5-tetramethyl-1,3,2-dioxaborolan-2-yl)-[1,1'-biphenyl]-2-yl)propyl)acetamide (7k) – Crude after reaction with 1a

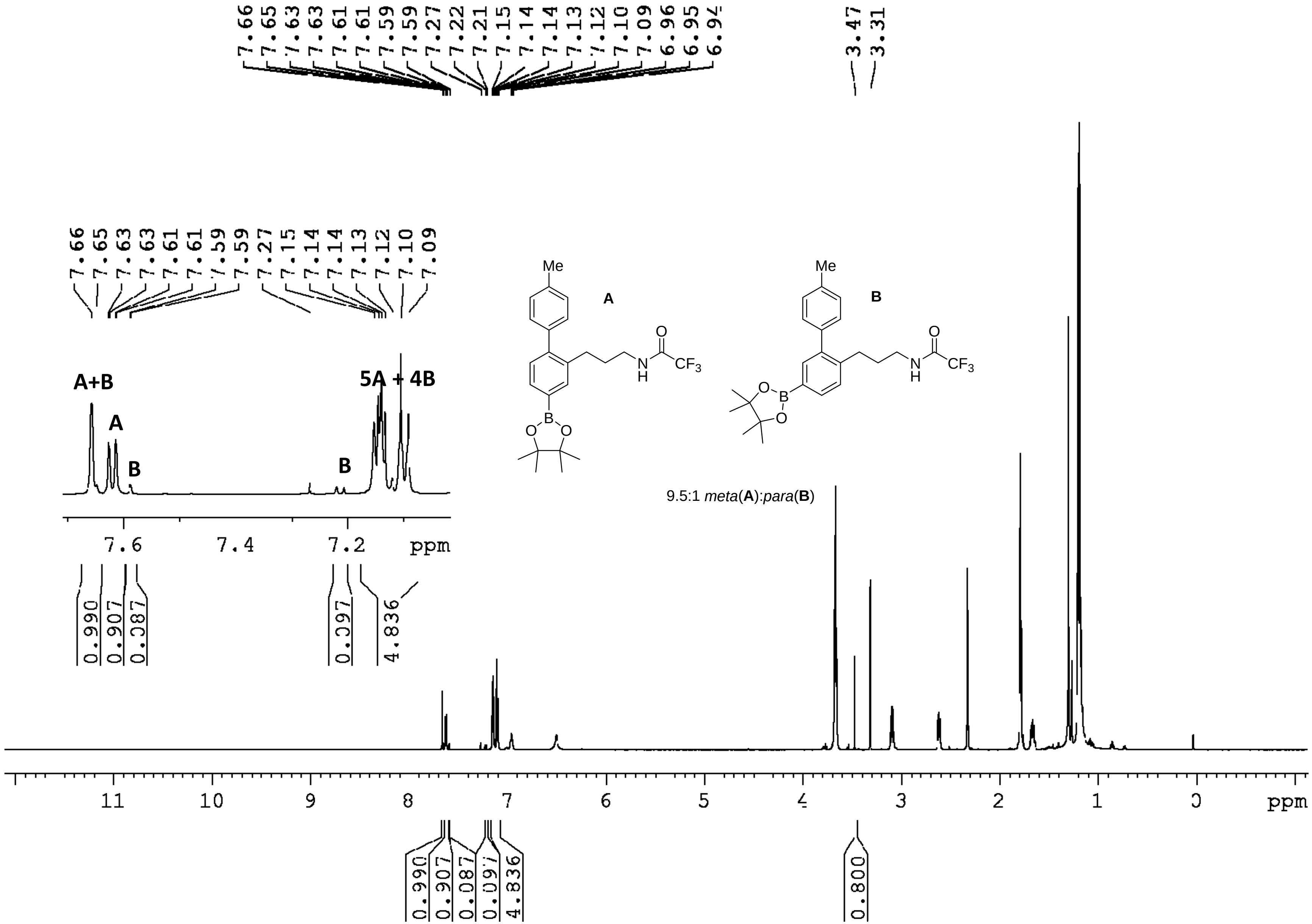

^{1}H NMR (600 MHz, $CDCl_3$) 2,2,2-Trifluoro-*N*-(3-(4'-methyl-4-(4,4,5,5-tetramethyl-1,3,2-dioxaborolan-2-yl)-[1,1'-biphenyl]-2-yl)propyl)acetamide (7k) – Purified after reaction with 1a

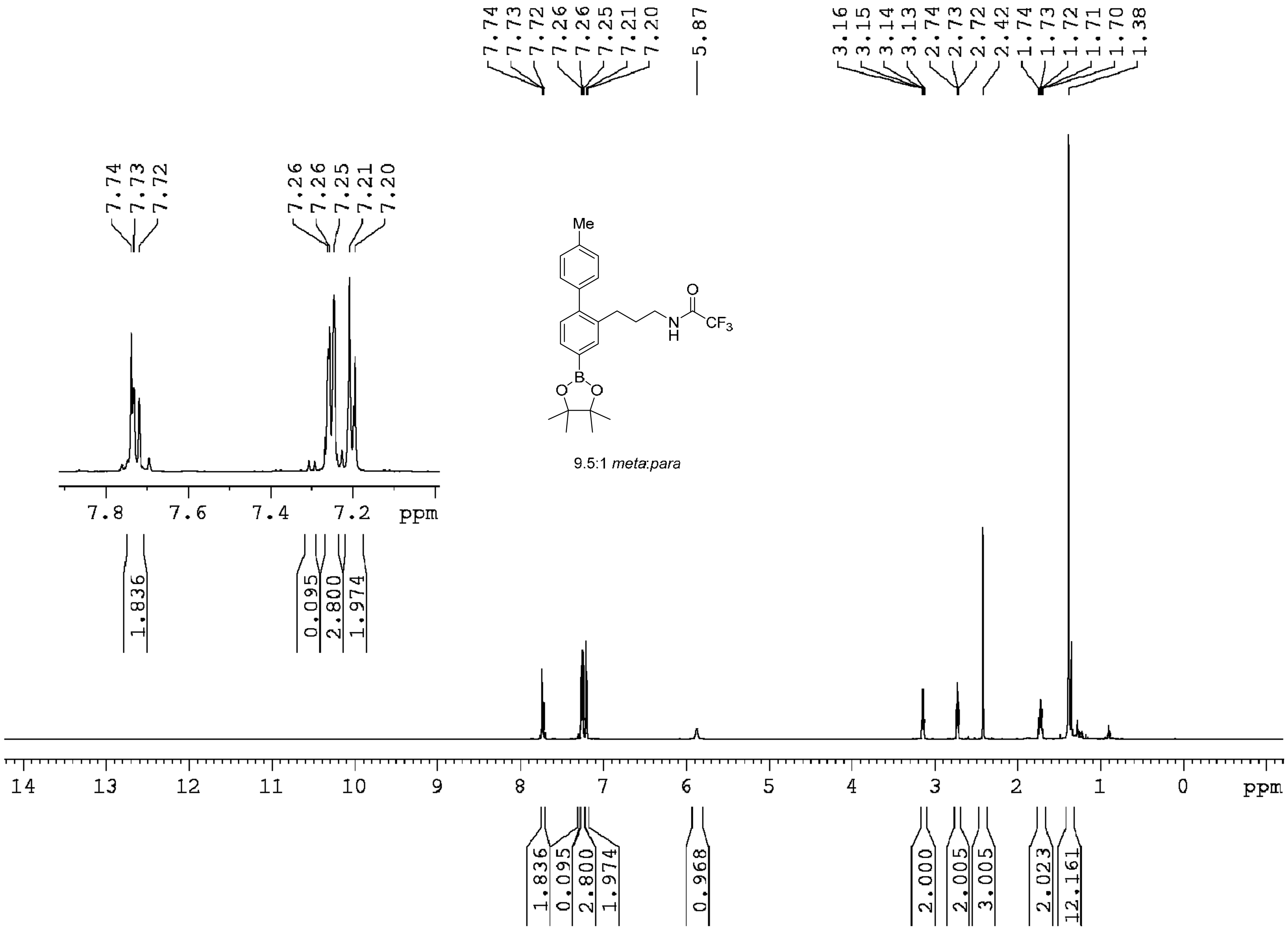

^{13}C NMR (151 MHz, $CDCl_3$) 2,2,2-Trifluoro-*N*-(3-(4'-methyl-4-(4,4,5,5-tetramethyl-1,3,2-dioxaborolan-2-yl)-[1,1'-biphenyl]-2-yl)propyl)acetamide (7k) – Purified after reaction with 1a

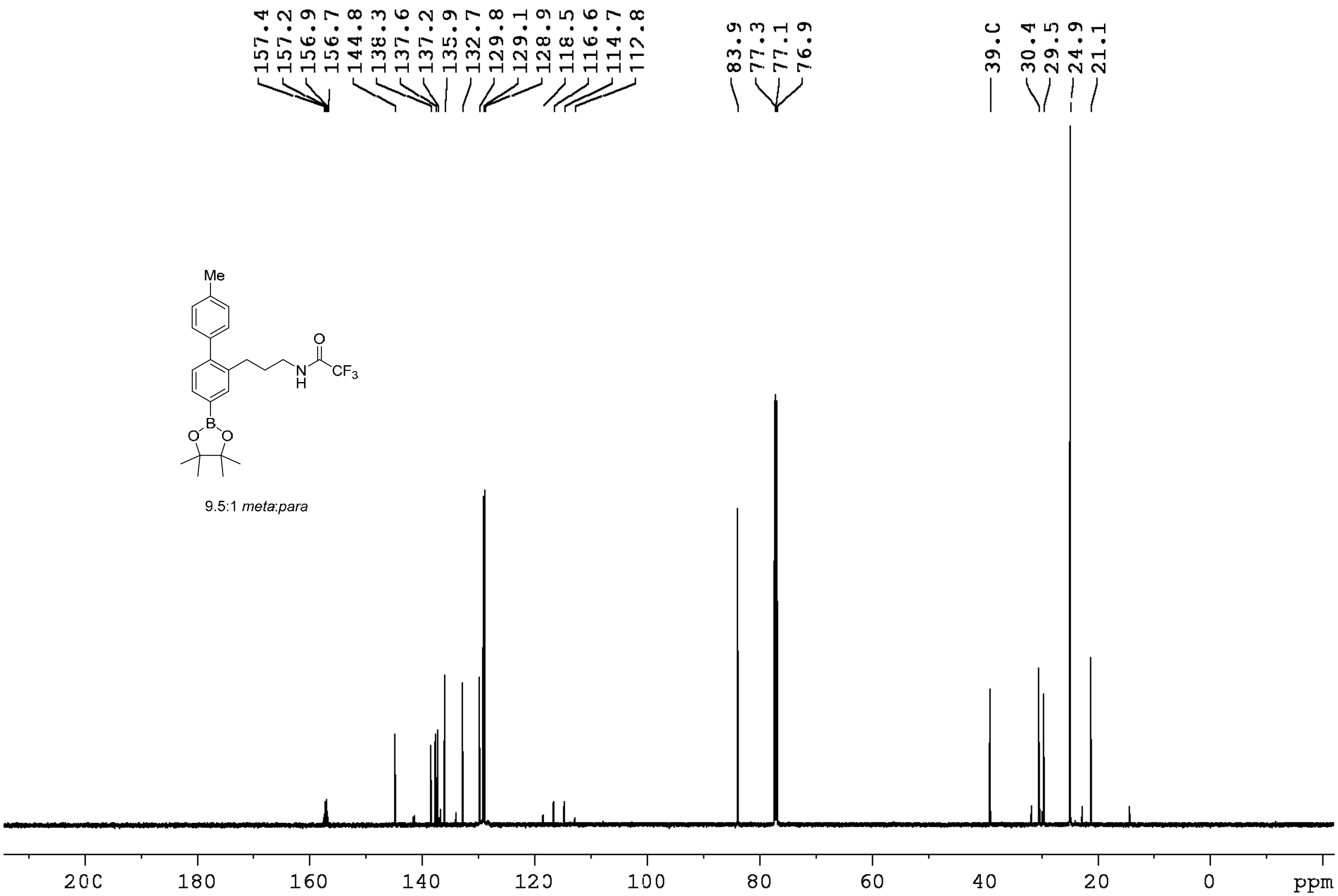

^{1}H NMR (500 MHz, $CDCl_3$) 2,2,2-Trifluoro-*N*-(3-(4'-methyl-4-(4,4,5,5-tetramethyl-1,3,2-dioxaborolan-2-yl)-[1,1'-biphenyl]-2-yl)propyl)acetamide (7k) – Purified after reaction with dtbpy

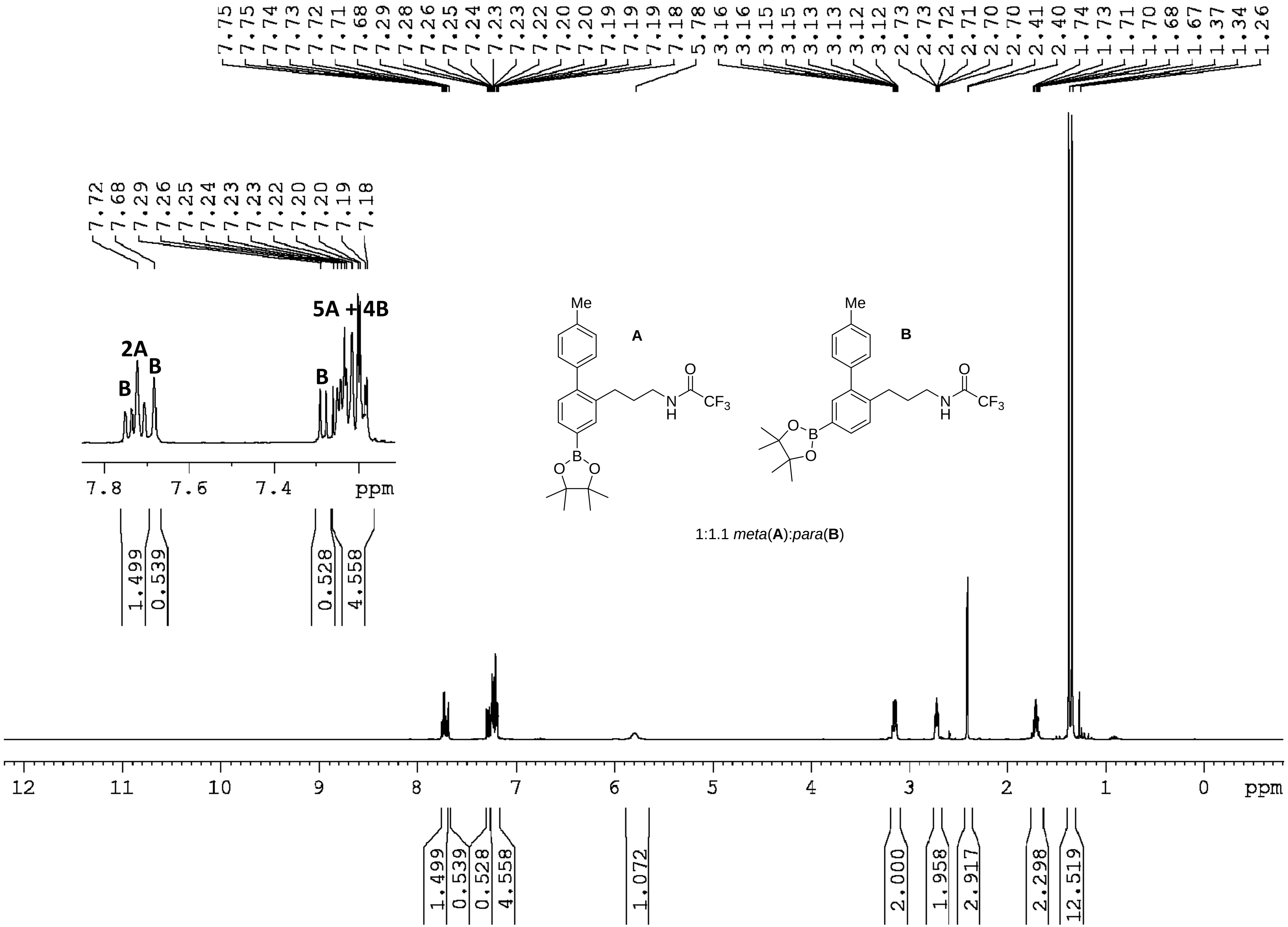

^{13}C NMR (126 MHz, $CDCl_3$) 2,2,2-Trifluoro-*N*-(3-(4'-methyl-4-(4,4,5,5-tetramethyl-1,3,2-dioxaborolan-2-yl)-[1,1'-biphenyl]-2-yl)propyl)acetamide (7k) – Purified after reaction with dtbpy

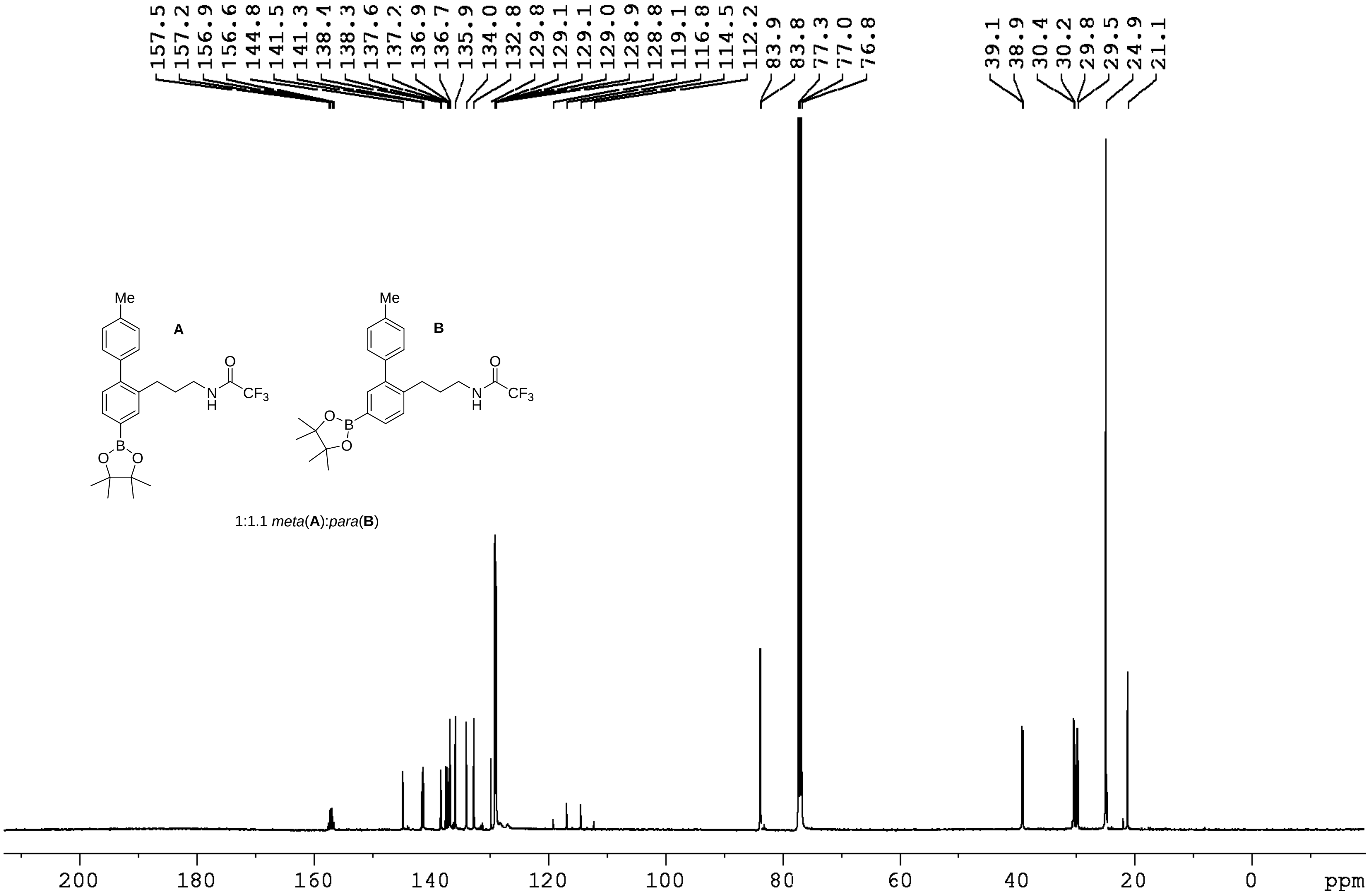

^{1}H NMR (500 MHz, $CDCl_3$) 2,2,2-Trifluoro-*N*-(3-(4-(4,4,5,5-tetramethyl-1,3,2-dioxaborolan-2-yl)pyridin-2-yl)propyl)acetamide (7l) – Crude after reaction with 1a

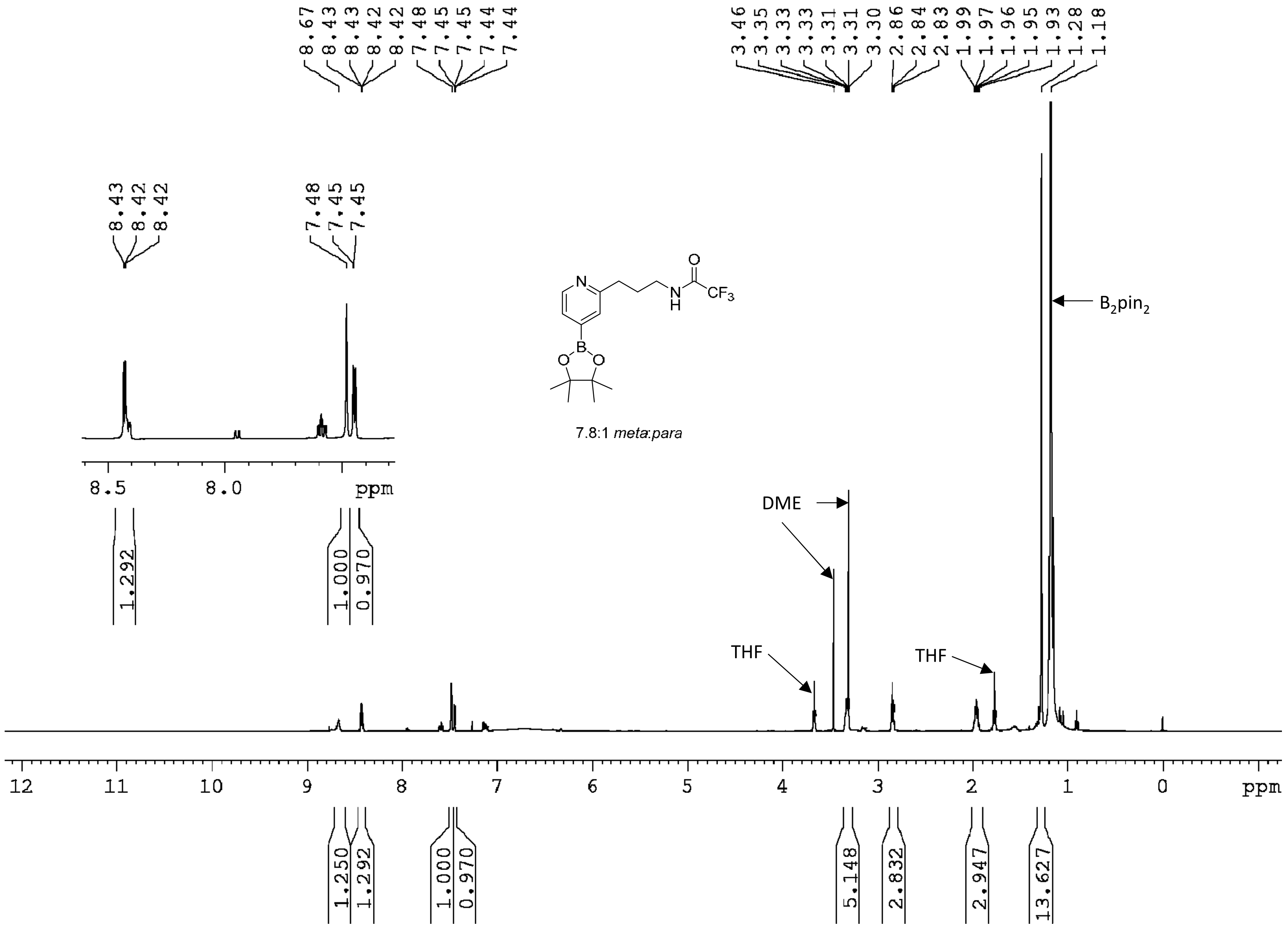

^{13}C NMR (126 MHz, $CDCl_3$) 2,2,2-Trifluoro-*N*-(3-(4-(4,4,5,5-tetramethyl-1,3,2-dioxaborolan-2-yl)pyridin-2-yl)propyl)acetamide (7l) – Crude after reaction with 1a

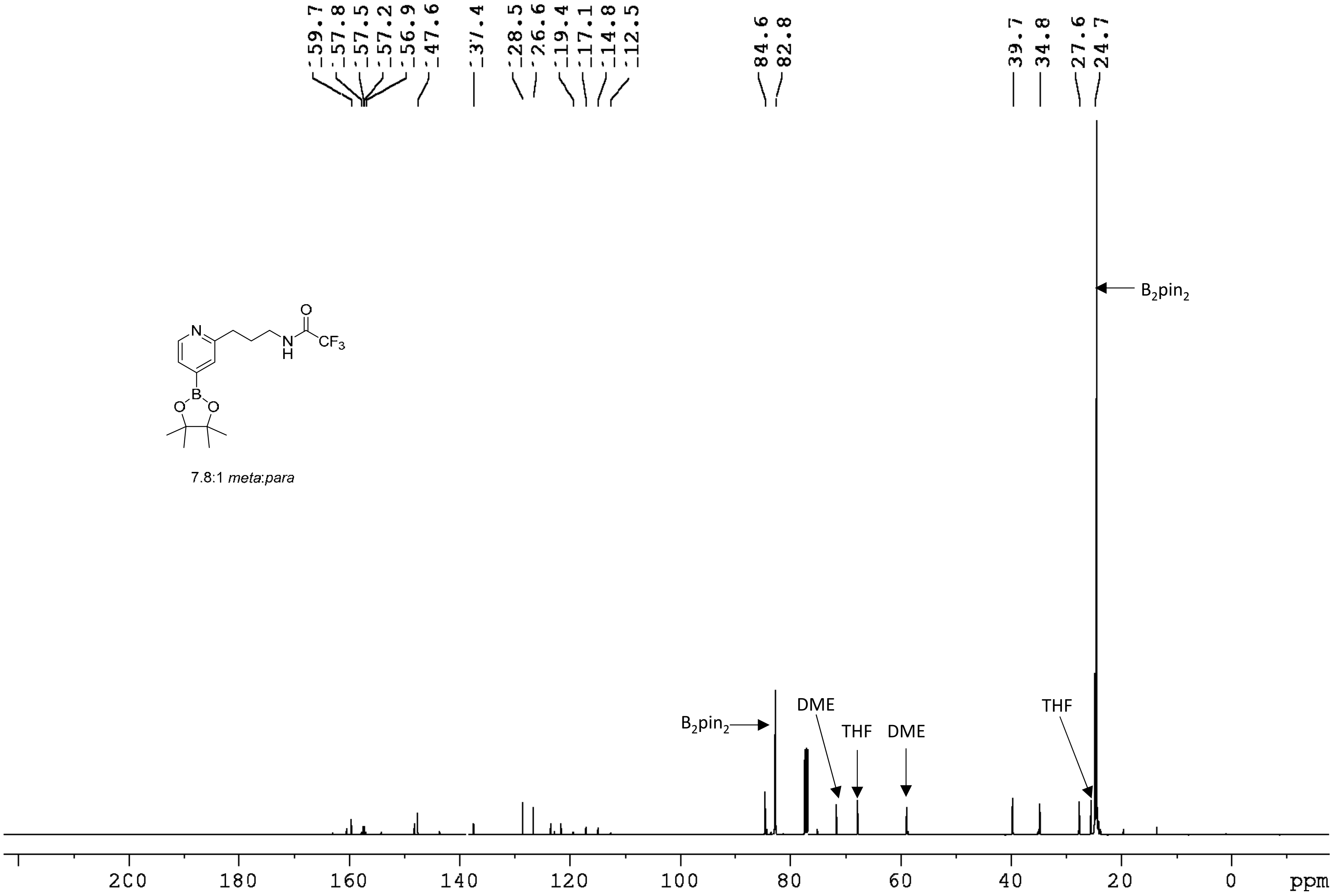

^{1}H NMR (400 MHz, $CDCl_3$) 2,2,2-Trifluoro-*N*-(3-(4-(4,4,5,5-tetramethyl-1,3,2-dioxaborolan-2-yl)pyridin-2-yl)propyl)acetamide (7l) – Crude after reaction with dtbpy

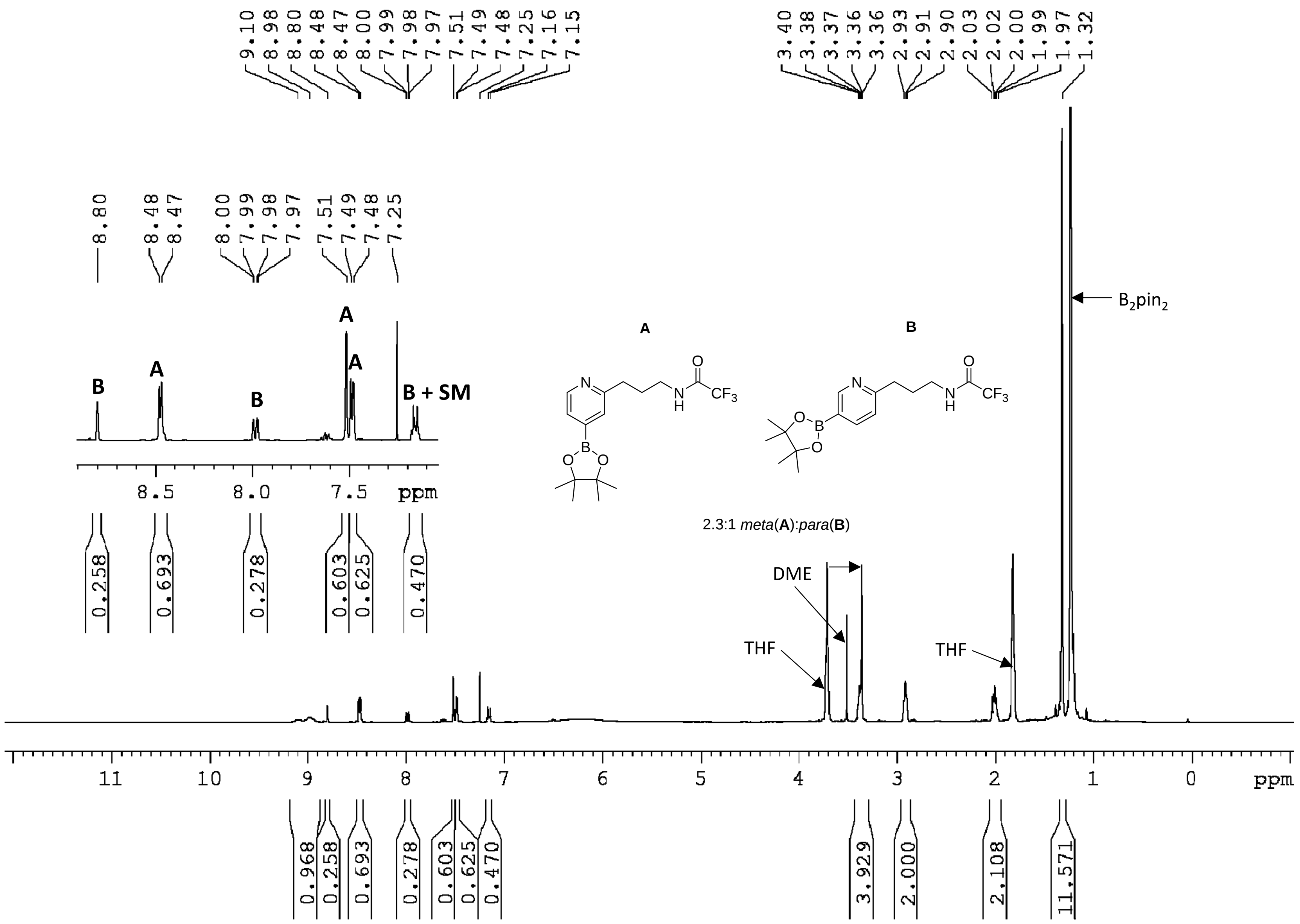

^{13}C NMR (100 MHz, $CDCl_3$) 2,2,2-Trifluoro-*N*-(3-(4-(4,4,5,5-tetramethyl-1,3,2-dioxaborolan-2-yl)pyridin-2-yl)propyl)acetamide (7l) – Crude after reaction with dtbpy

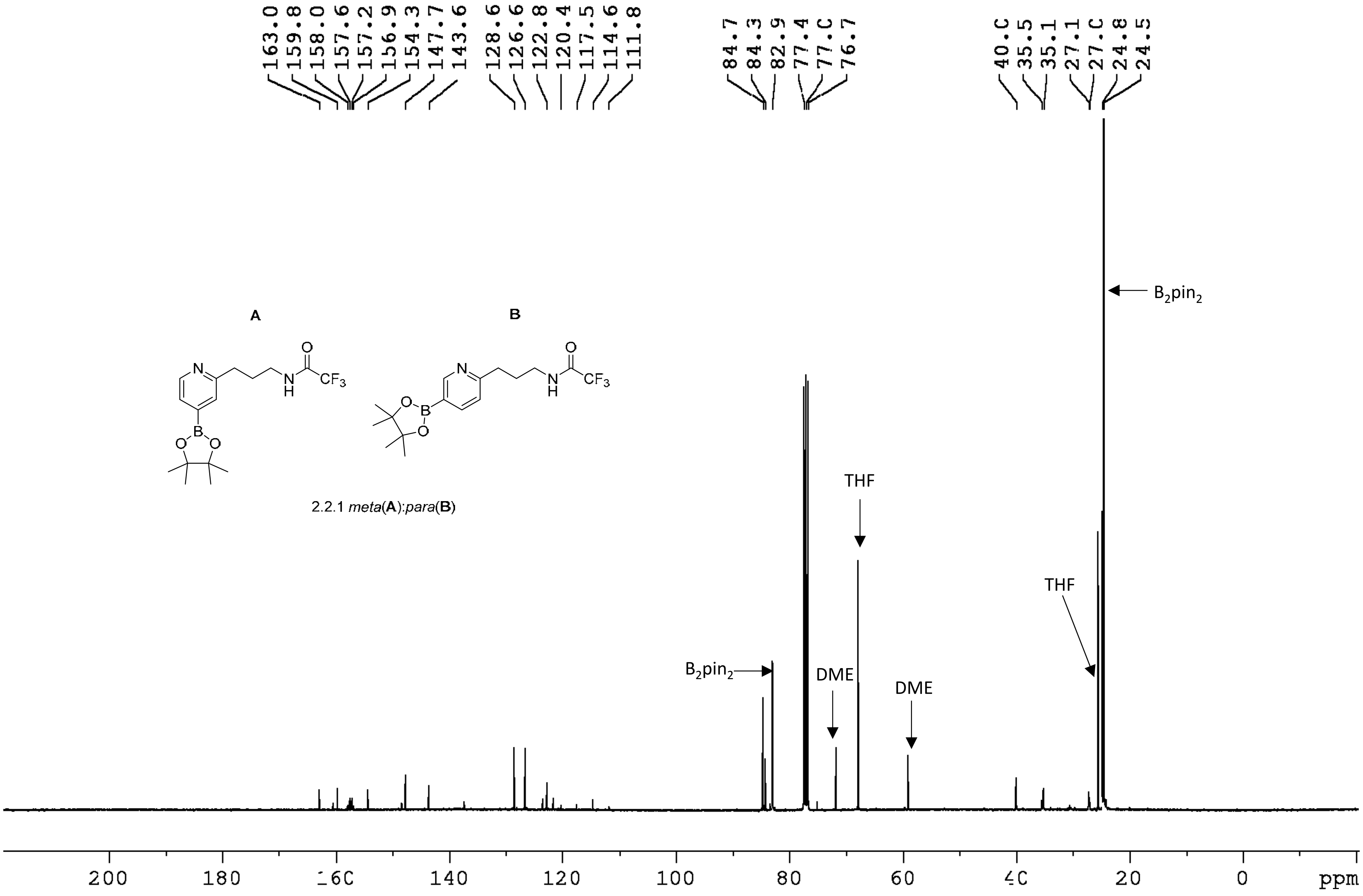

^{1}H NMR (500 MHz, CDCl$_3$) *N*-(4-(3,5-bis(4,4,5,5-tetramethyl-1,3,2-dioxaborolan-2-yl)phenyl)butyl)-2,2,2-trifluoroacetamide (10) – Crude after reaction with 1a

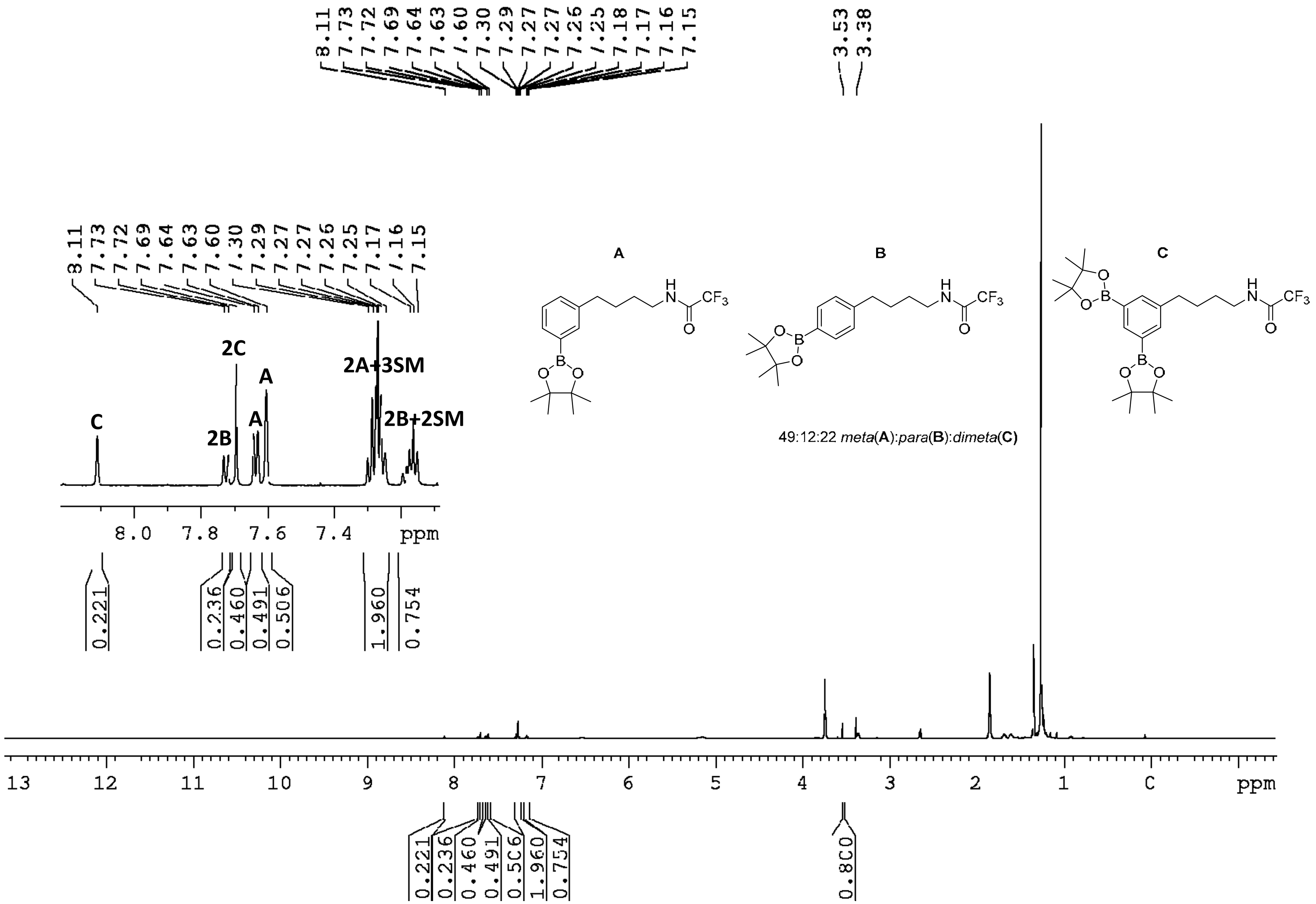

^{1}H NMR (500 MHz, $CDCl_3$) *N*-(4-(3,5-bis(4,4,5,5-tetramethyl-1,3,2-dioxaborolan-2-yl)phenyl)butyl)-2,2,2-trifluoroacetamide (10) – *Dimeta* fraction purified from reaction with 1a

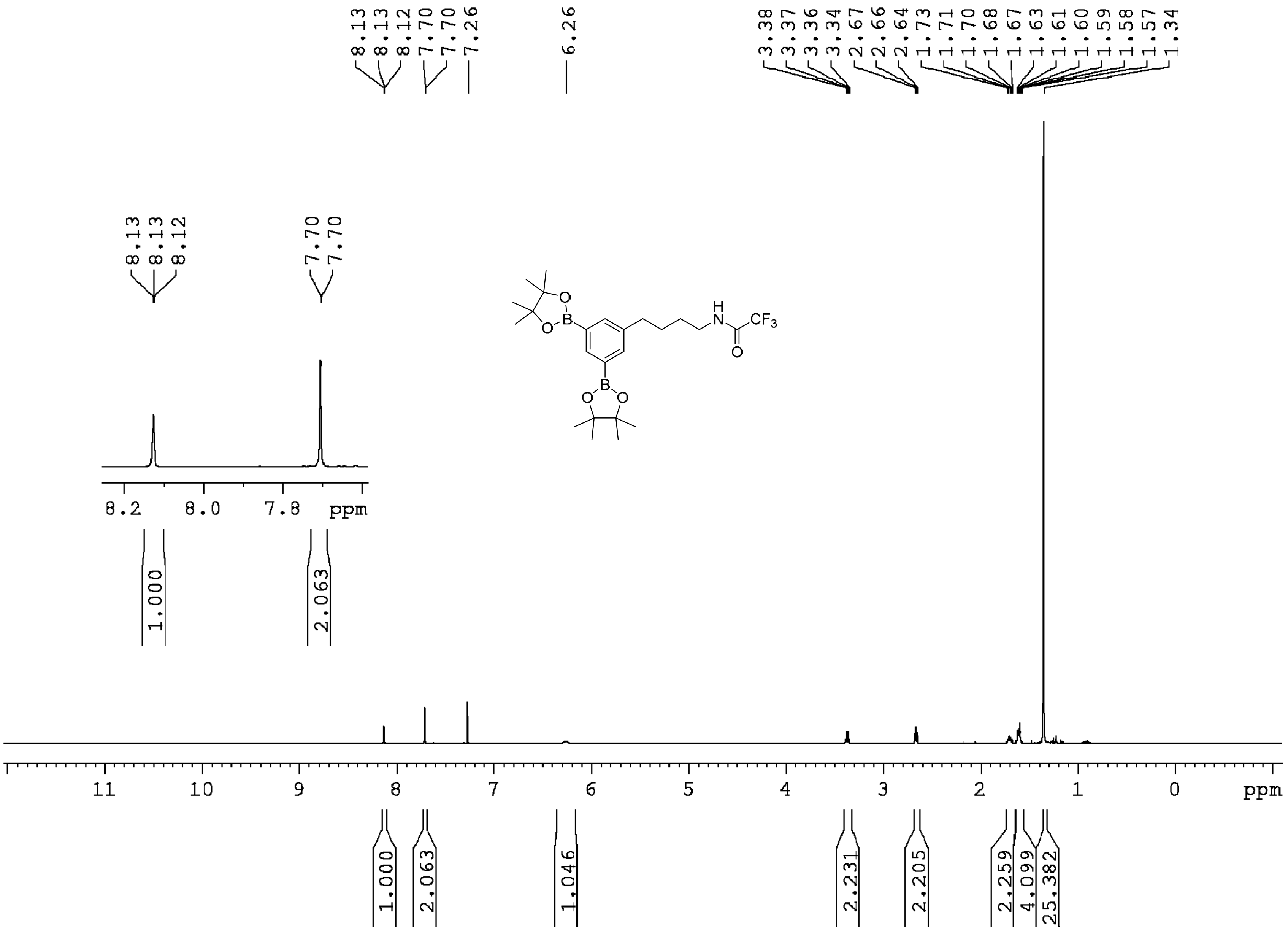

^{13}C NMR (126 MHz, $CDCl_3$) *N*-(4-(3,5-bis(4,4,5,5-tetramethyl-1,3,2-dioxaborolan-2-yl)phenyl)butyl)-2,2,2-trifluoroacetamide (10) – *Dimeta* fraction purified from reaction with 1a

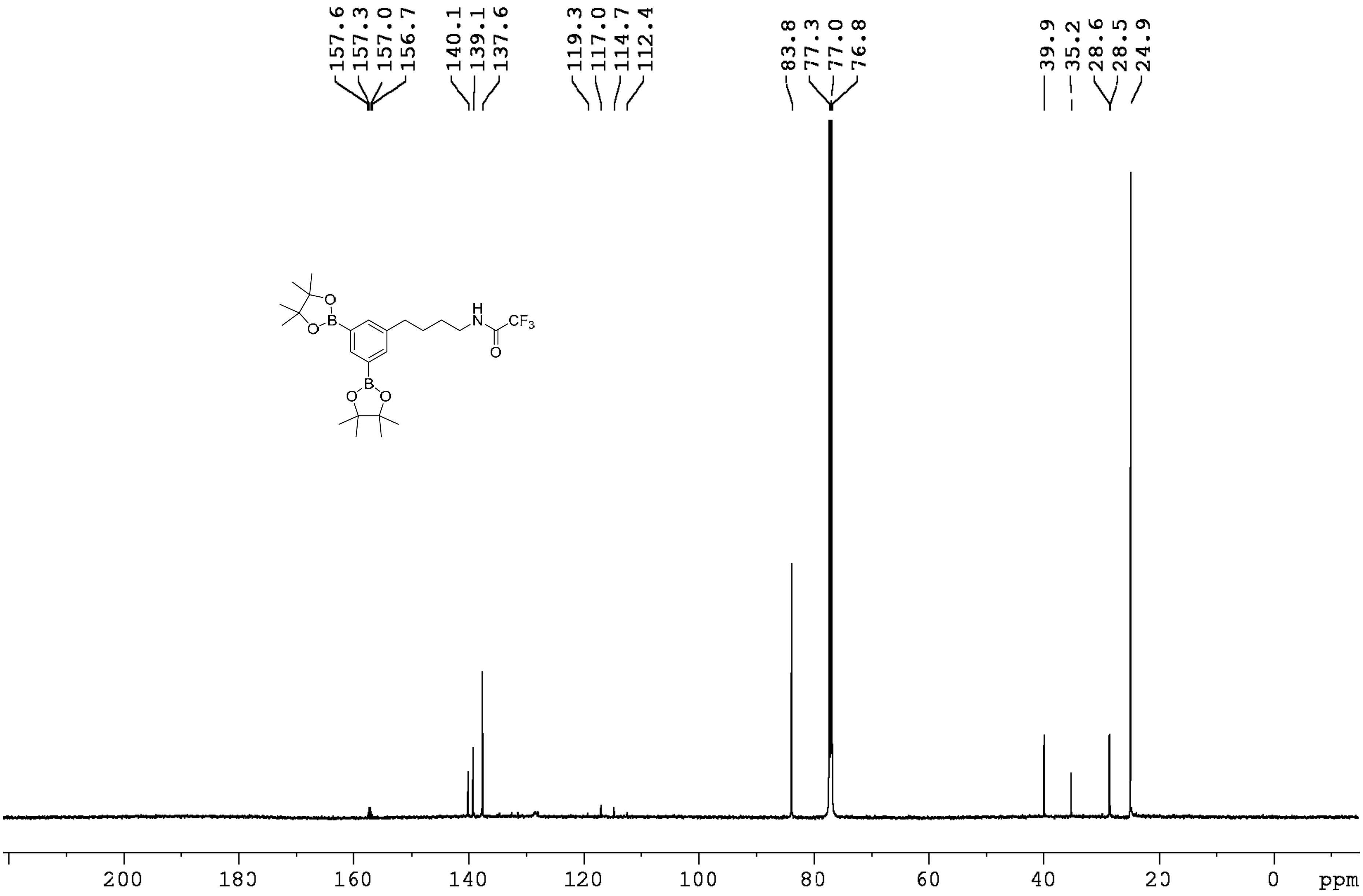

^{1}H NMR (500 MHz, CDCl$_3$) 2,2,2-Trifluoro-*N*-(4-(3-(4,4,5,5-tetramethyl-1,3,2-dioxaborolan-2-yl)phenyl)butyl)acetamide (10) – Monoborylated products isolated from reaction with dtbpy

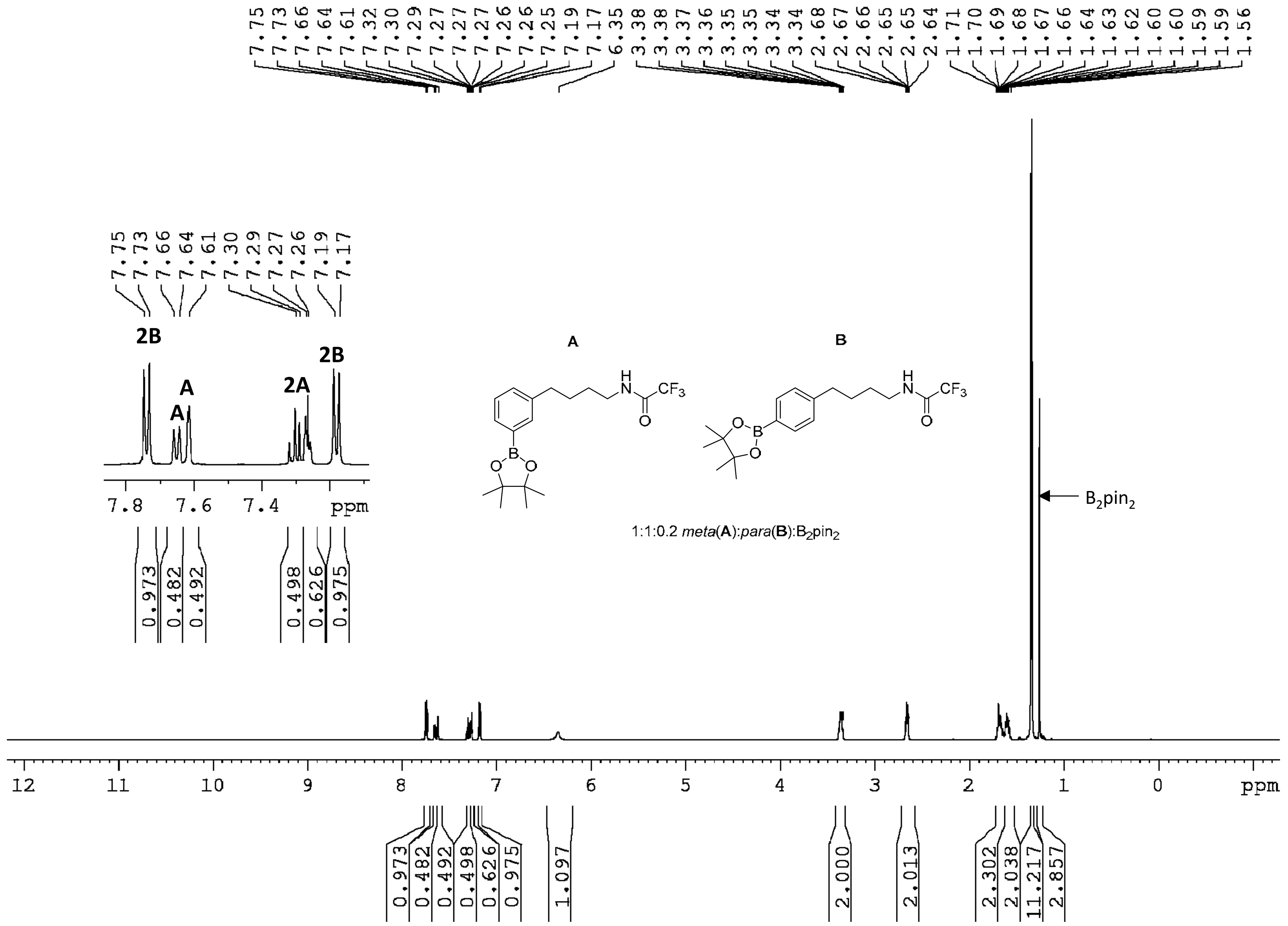

^{13}C NMR (126 MHz, $CDCl_3$) 2,2,2-Trifluoro-*N*-(4-(3-(4,4,5,5-tetramethyl-1,3,2-dioxaborolan-2-yl)phenyl)butyl)acetamide (10) – Monoborylated products isolated from reaction with dtbpy

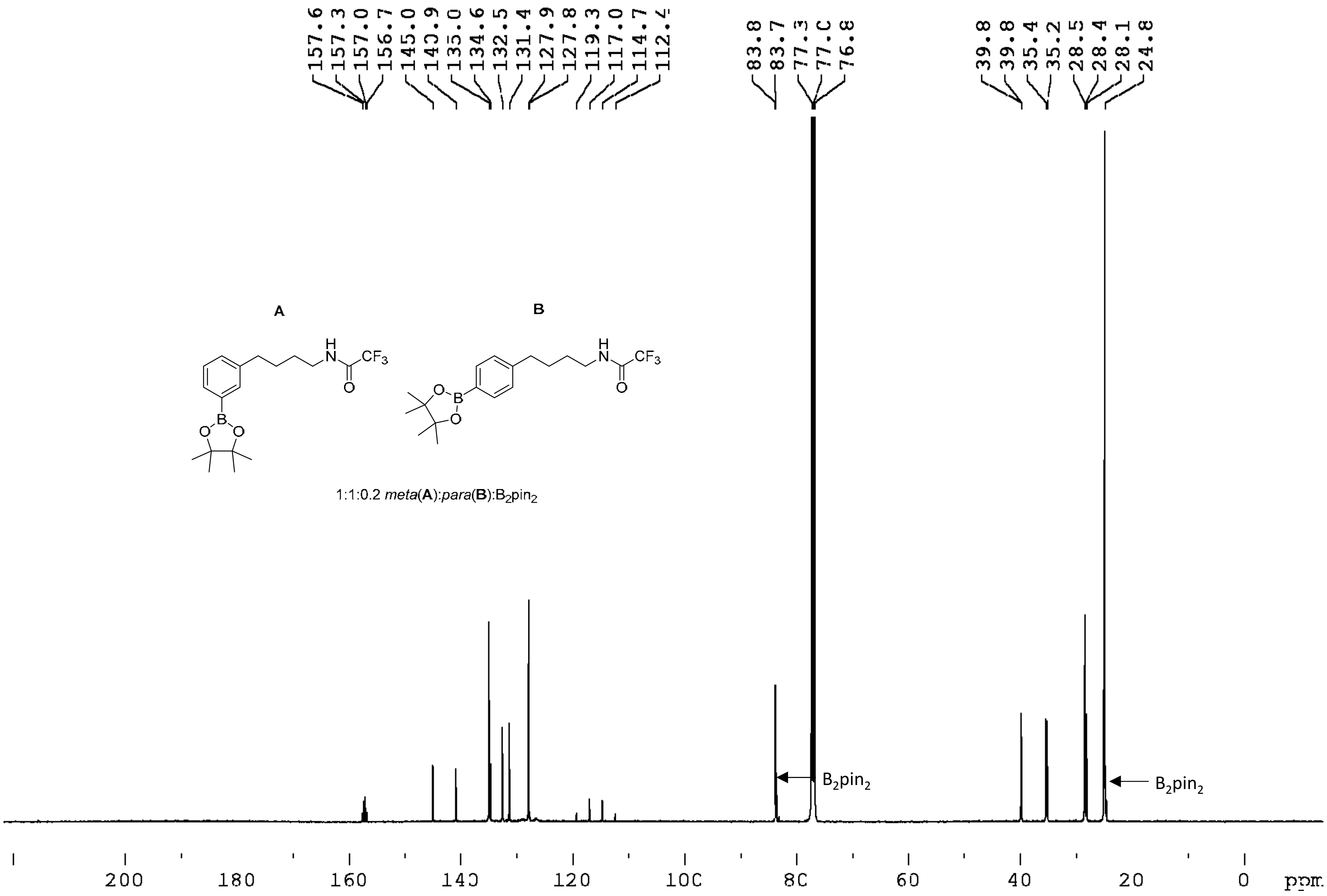

^{1}H NMR (500 MHz, $CDCl_3$) *N*-(5-(3,5-bis(4,4,5,5-tetramethyl-1,3,2-dioxaborolan-2-yl)phenyl)pentyl)-2,2,2-trifluoroacetamide (11) – Crude after reaction with 1b

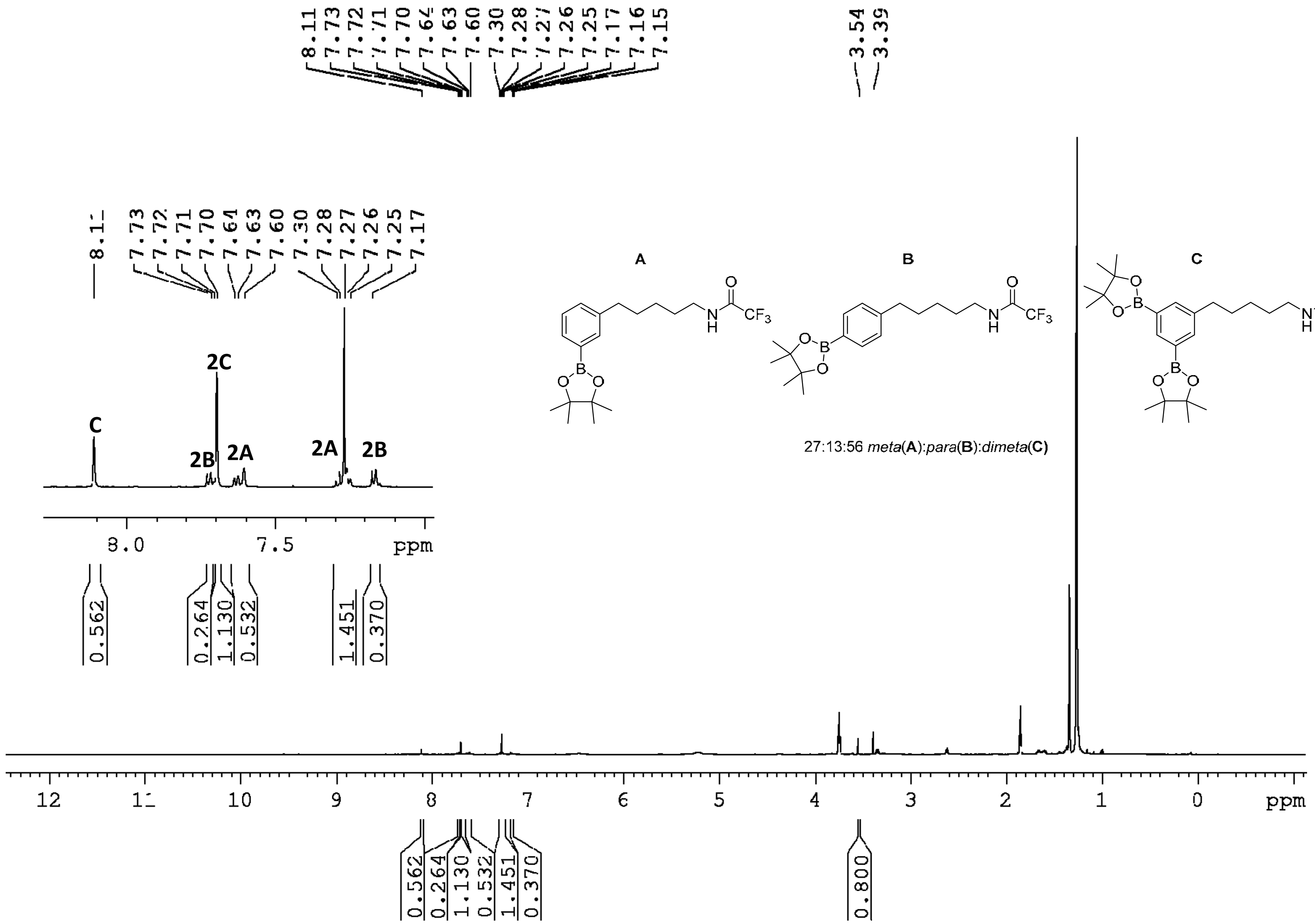

^{1}H NMR (500 MHz, $CDCl_3$) *N*-(5-(3,5-bis(4,4,5,5-tetramethyl-1,3,2-dioxaborolan-2-yl)phenyl)pentyl)-2,2,2-trifluoroacetamide (11) – *Dimeta* fraction purified from reaction with 1b

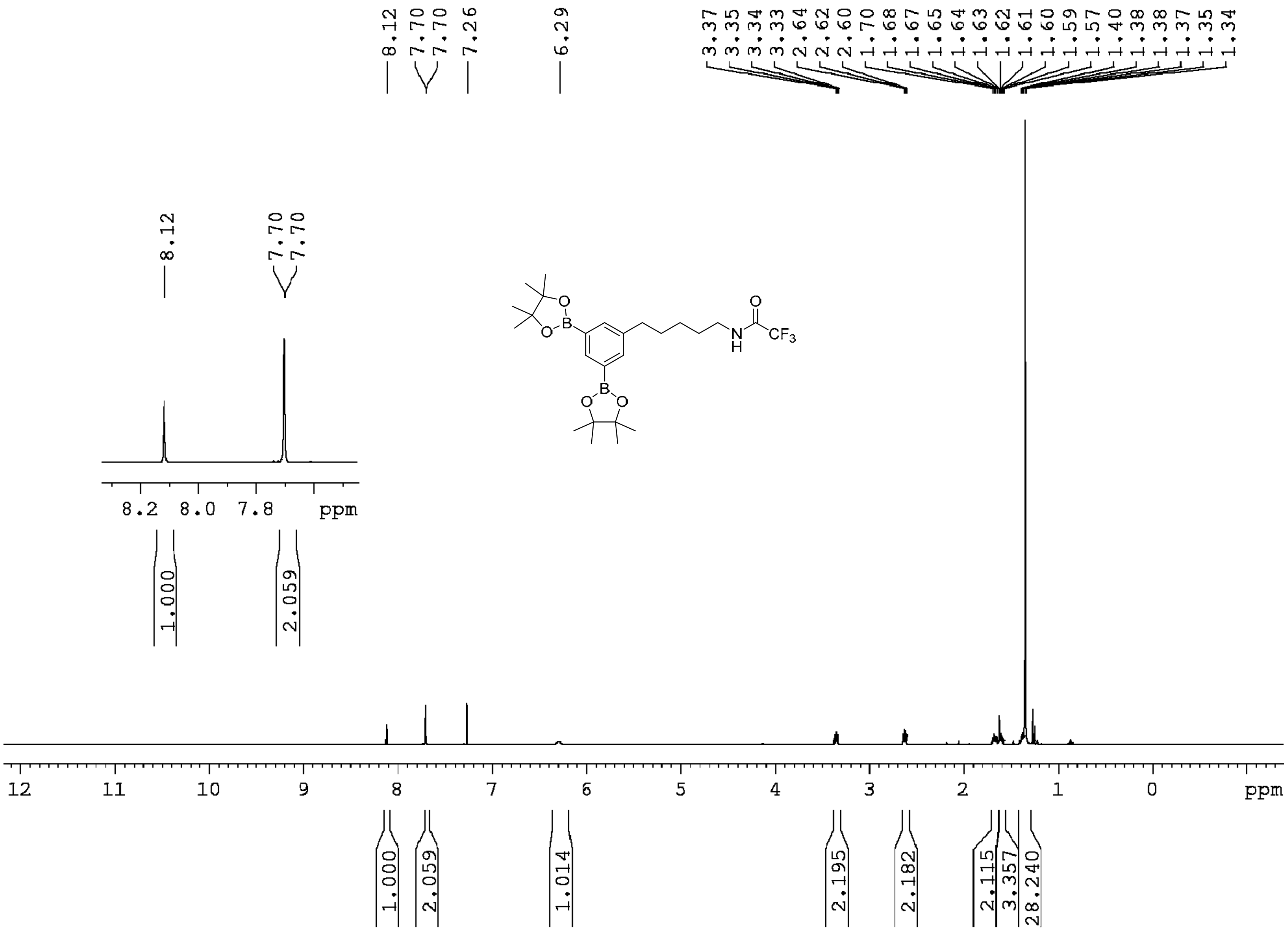

^{13}C NMR (126 MHz, $CDCl_3$) *N*-(5-(3,5-bis(4,4,5,5-tetramethyl-1,3,2-dioxaborolan-2-yl)phenyl)pentyl)-2,2,2-trifluoroacetamide (11) – *Dimeta* fraction purified from reaction with 1b

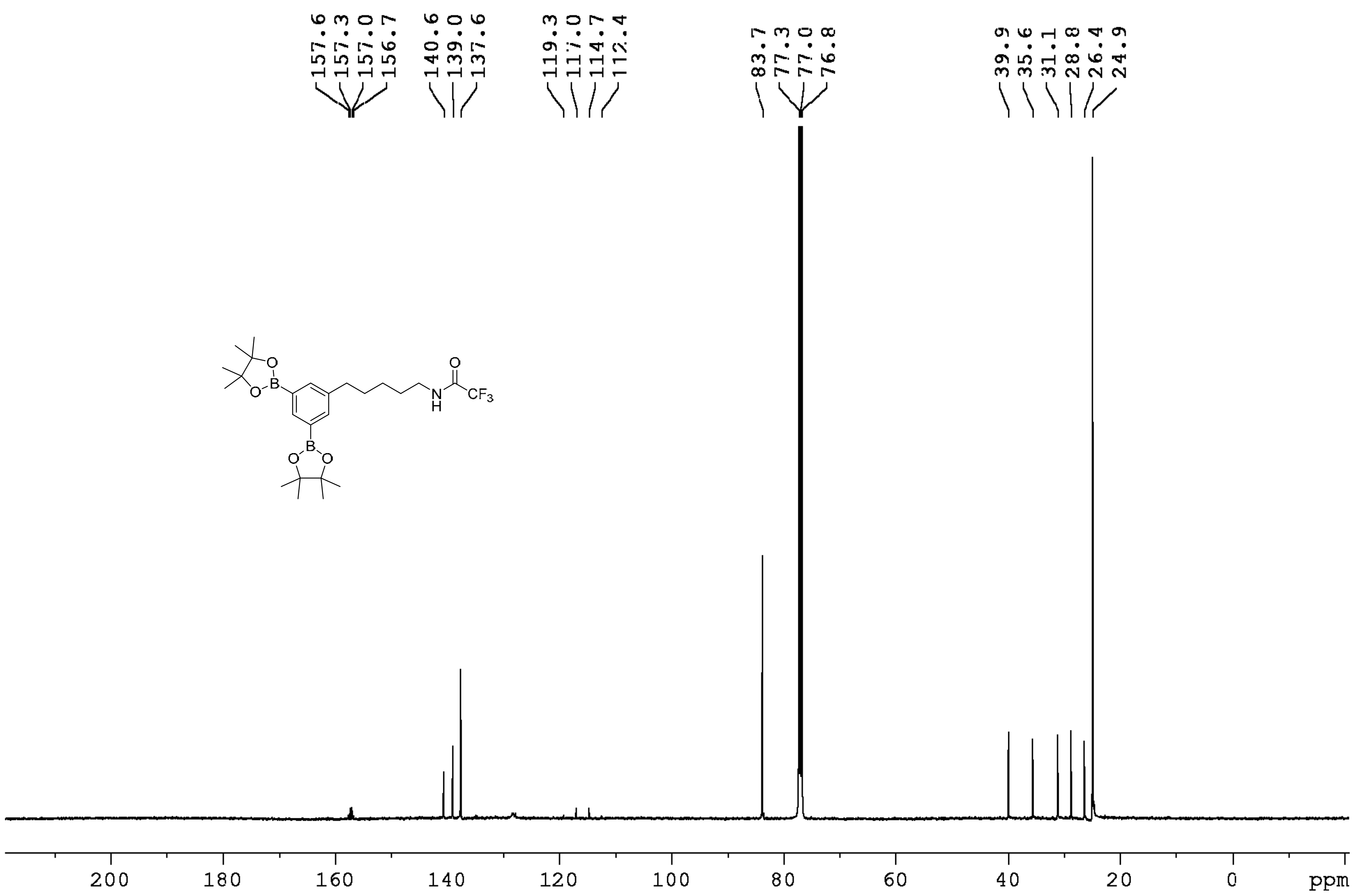

^{1}H NMR (500 MHz, $CDCl_3$) 2,2,2-Trifluoro-*N*-(5-(3-(4,4,5,5-tetramethyl-1,3,2-dioxaborolan-2-yl)phenyl)pentyl)acetamide (11) – Monoborylated products isolated from reaction with dtbpy

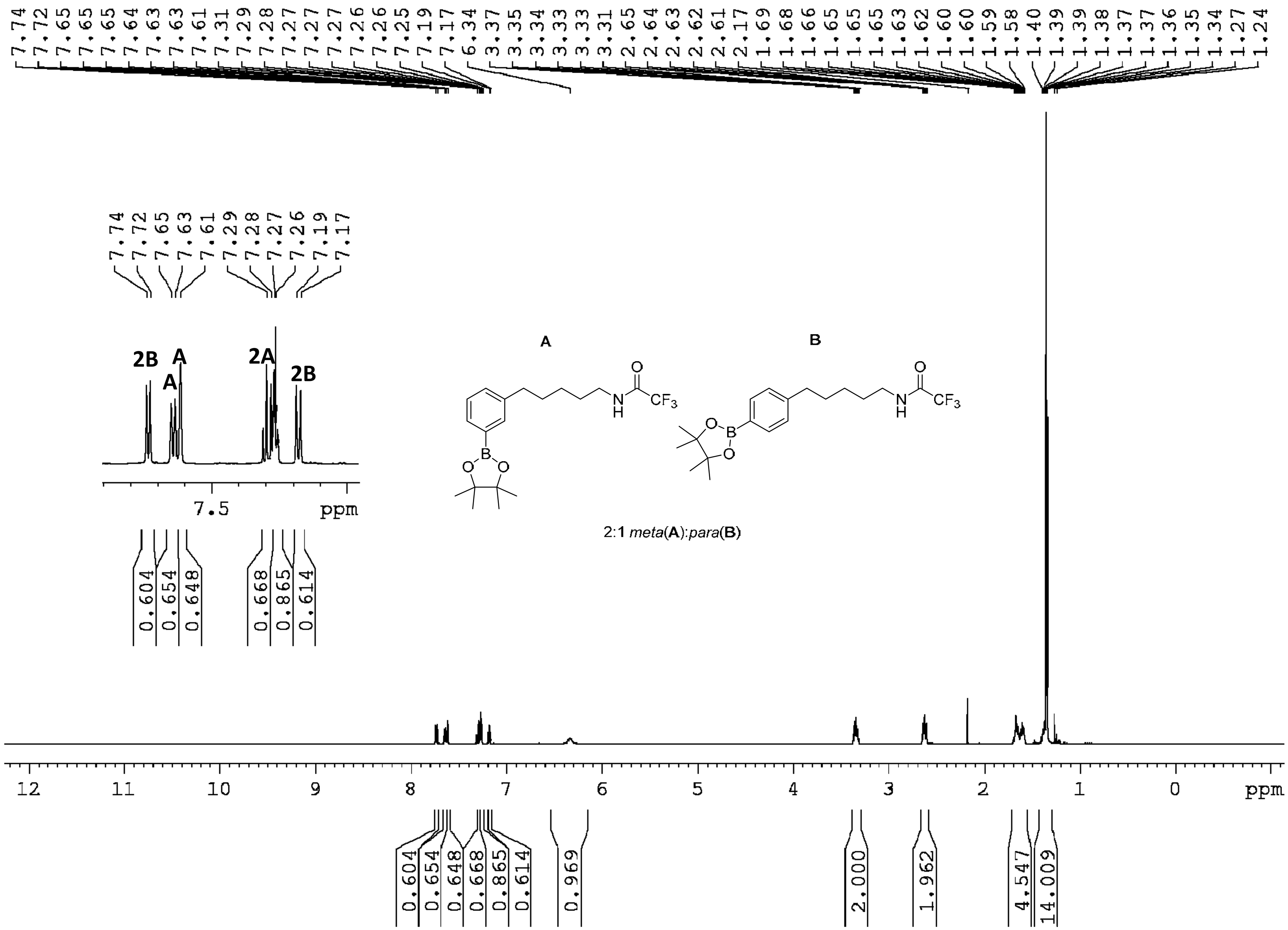

2:1 *meta*(**A**):*para*(**B**)

^{13}C NMR (126 MHz, $CDCl_3$) 2,2,2-Trifluoro-*N*-(5-(3-(4,4,5,5-tetramethyl-1,3,2-dioxaborolan-2-yl)phenyl)pentyl)acetamide (11) – Monoborylated products isolated from reaction with dtbpy

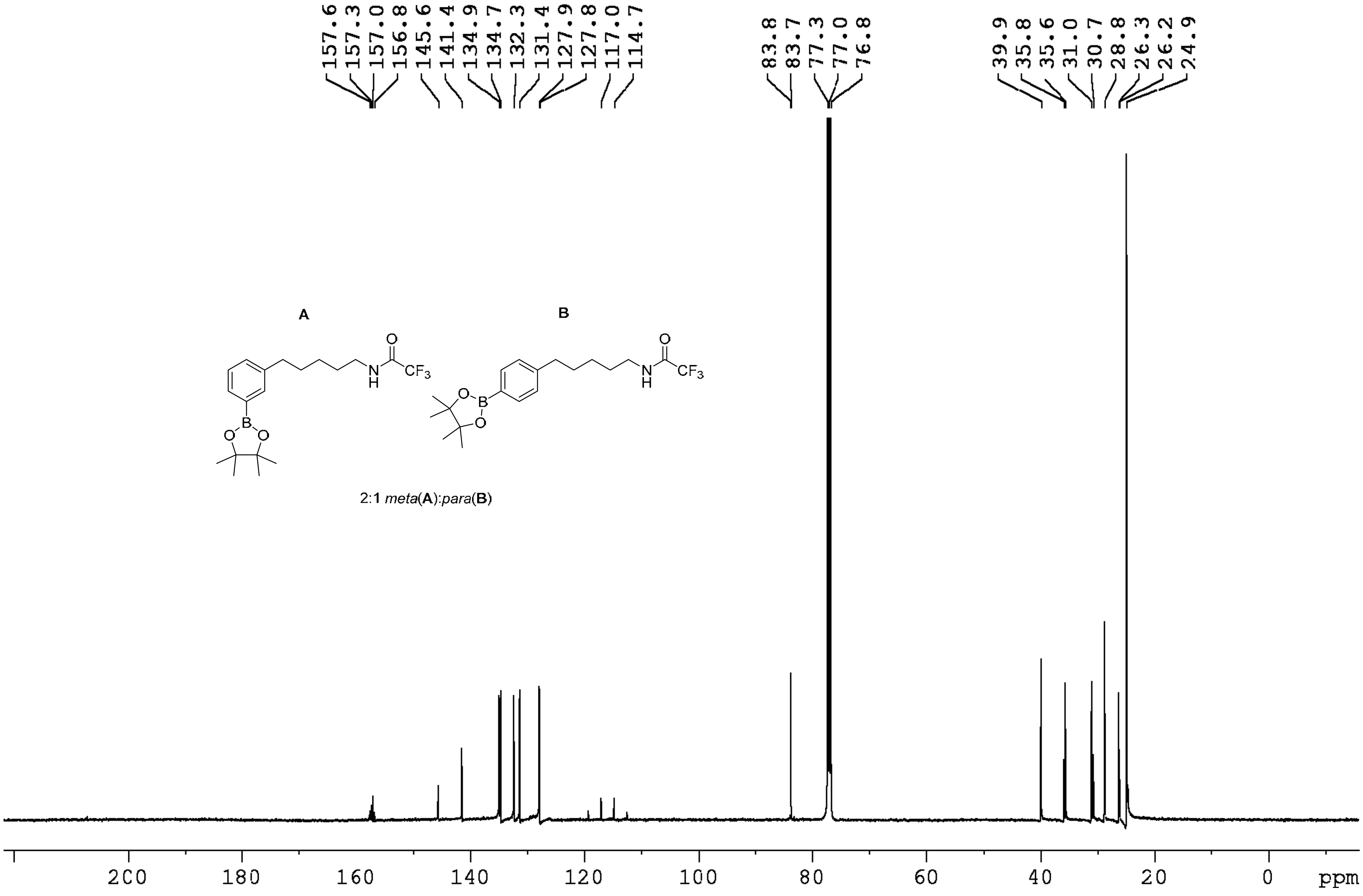

^{1}H NMR (500 MHz, $CDCl_3$) *N*-(6-(3,5-bis(4,4,5,5-tetramethyl-1,3,2-dioxaborolan-2-yl)phenyl)hexyl)-2,2,2-trifluoroacetamide (12) – Crude after reaction with 1b

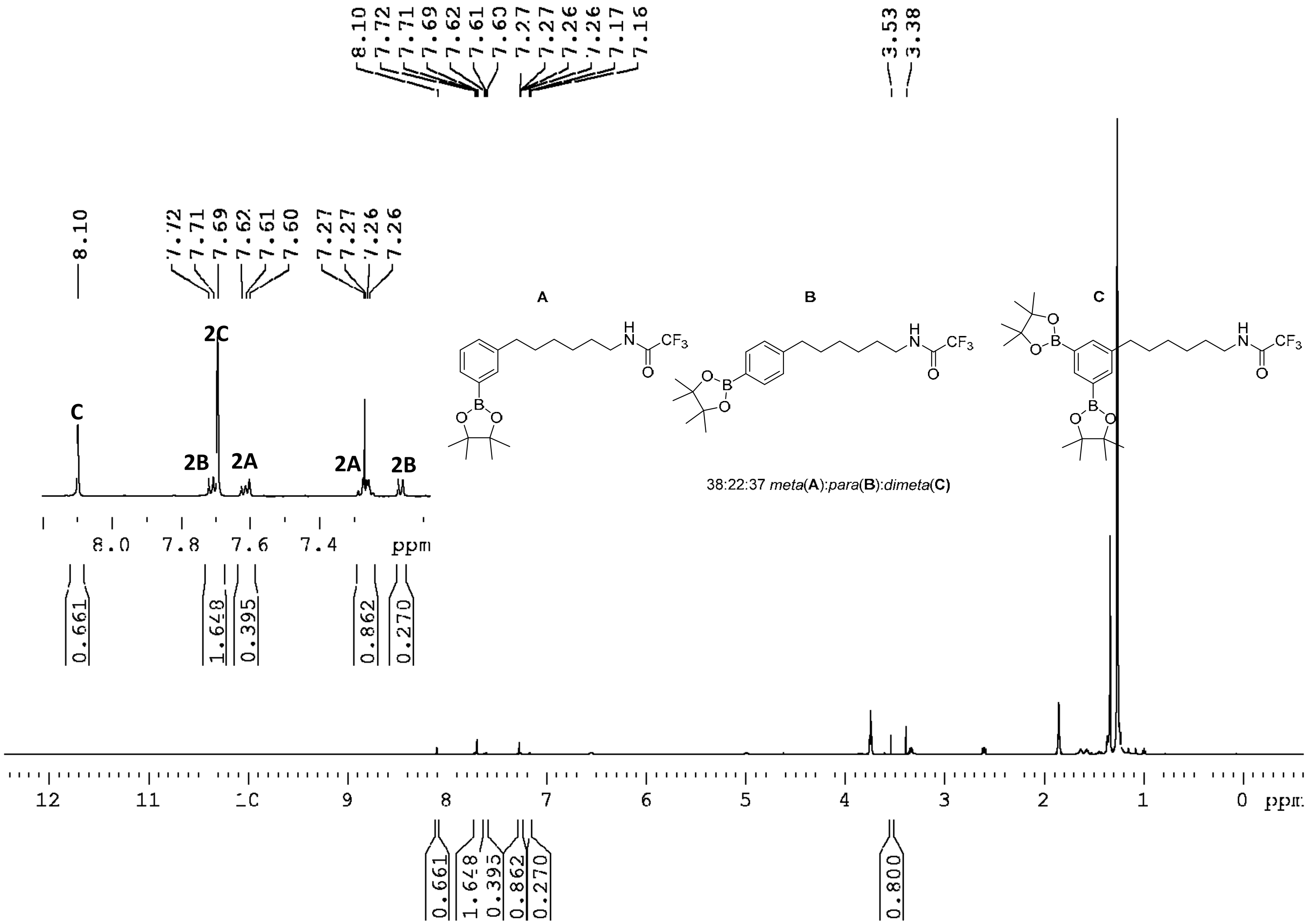

^{1}H NMR (500 MHz, $CDCl_3$) *N*-(6-(3,5-bis(4,4,5,5-tetramethyl-1,3,2-dioxaborolan-2-yl)phenyl)hexyl)-2,2,2-trifluoroacetamide (12)– *Dimeta* fraction purified from reaction with 1b

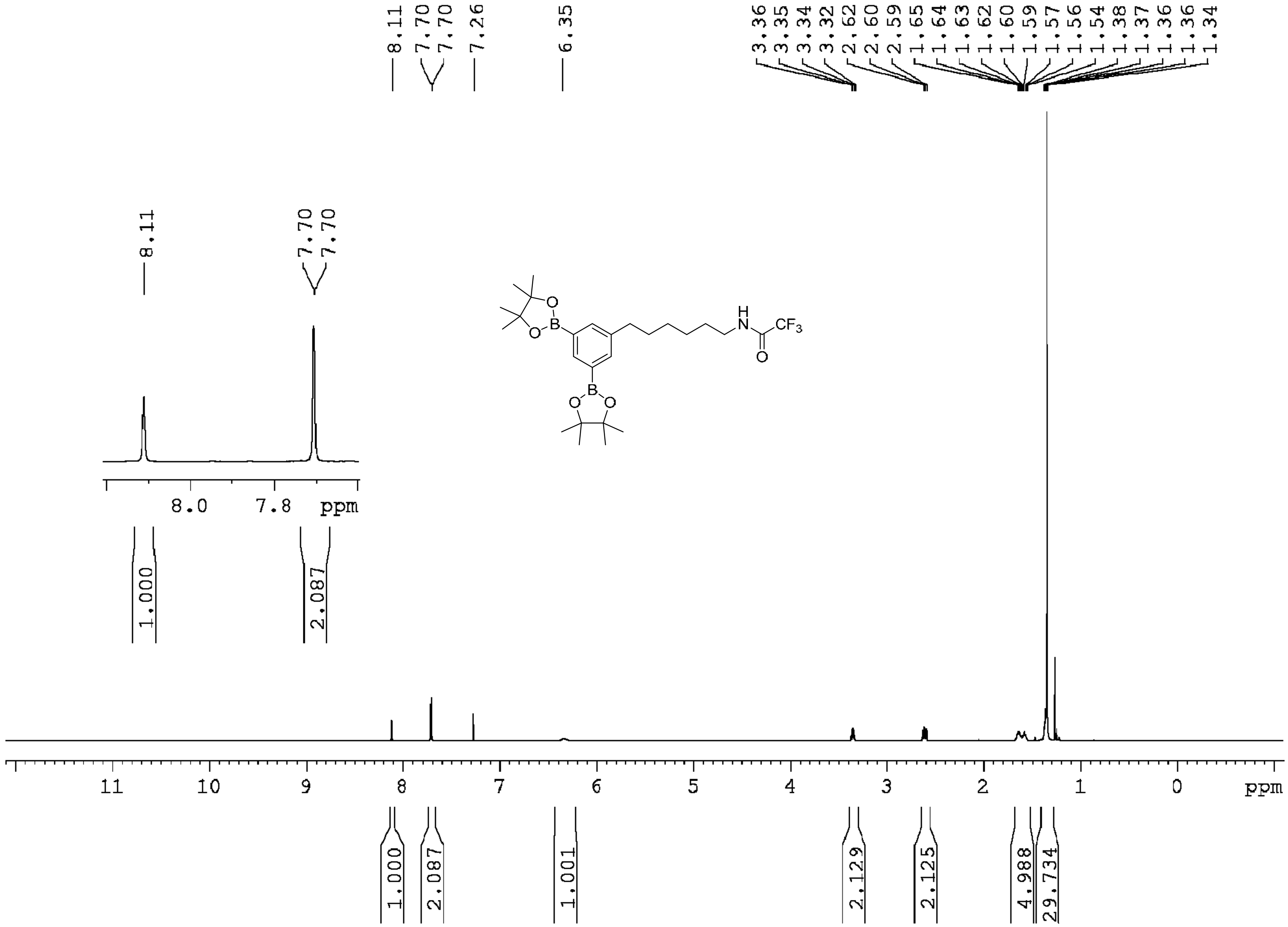

^{13}C NMR (126 MHz, $CDCl_3$) *N*-(6-(3,5-bis(4,4,5,5-tetramethyl-1,3,2-dioxaborolan-2-yl)phenyl)hexyl)-2,2,2-trifluoroacetamide (12)– *Dimeta* fraction purified from reaction with 1b

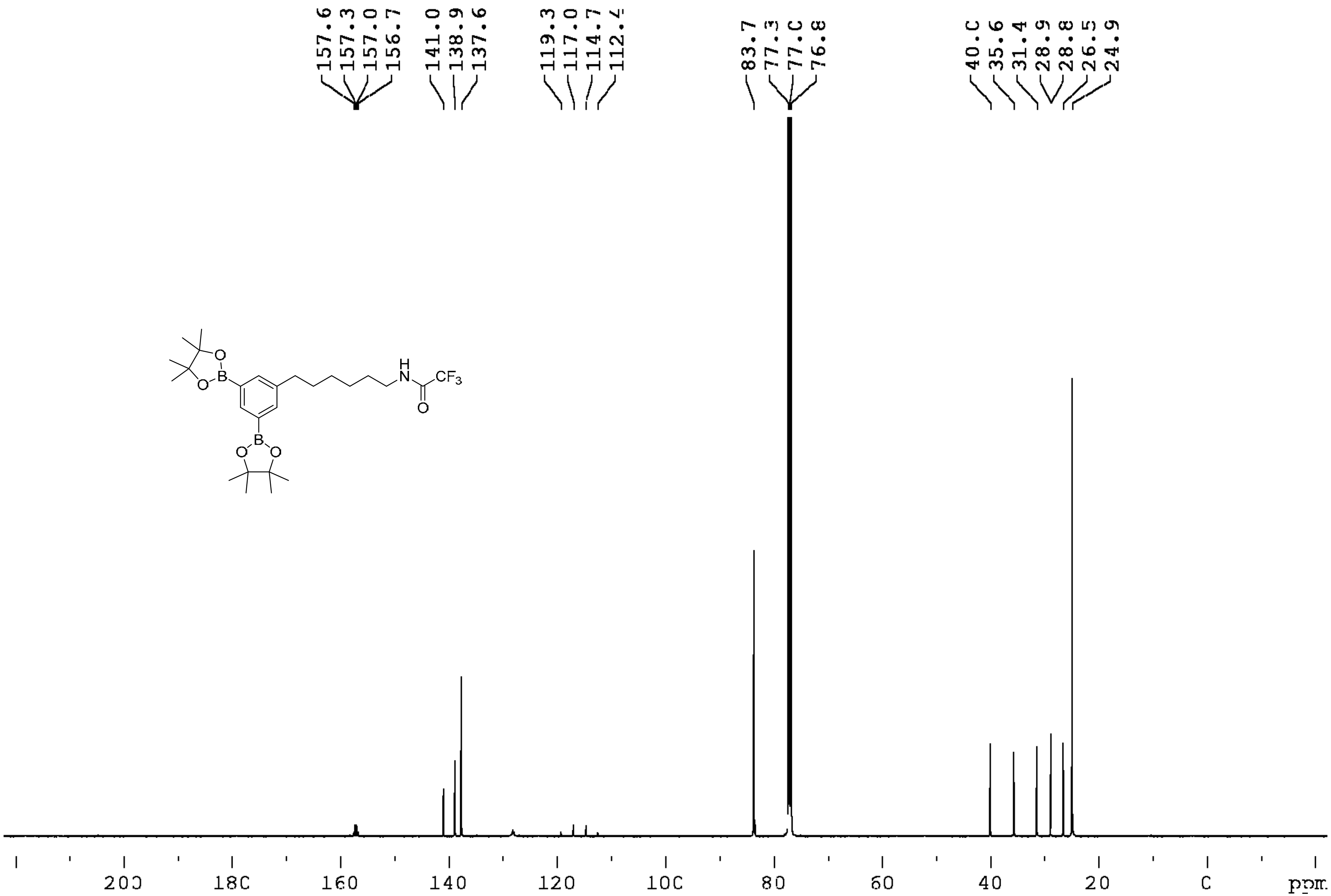

^{1}H NMR (500 MHz, $CDCl_3$) 2,2,2-Trifluoro-*N*-(6-(3-(4,4,5,5-tetramethyl-1,3,2-dioxaborolan-2-yl)phenyl)hexyl)acetamide (12) – Monoborylated products isolated from reaction with dtbpy

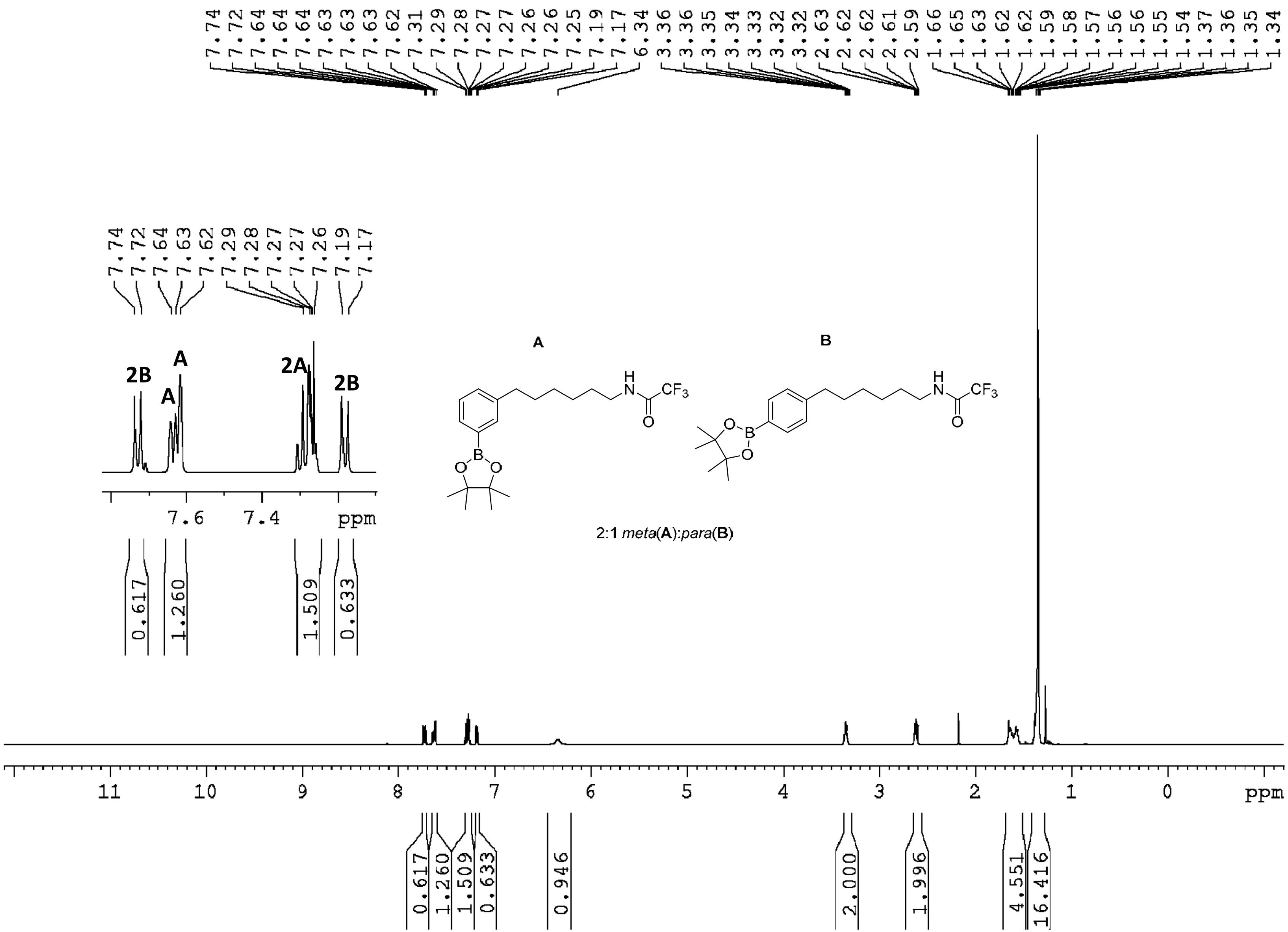

^{13}C NMR (126 MHz, $CDCl_3$) 2,2,2-Trifluoro-*N*-(6-(3-(4,4,5,5-tetramethyl-1,3,2-dioxaborolan-2-yl)phenyl)hexyl)acetamide (12)– Monoborylated products isolated from reaction with dtbpy

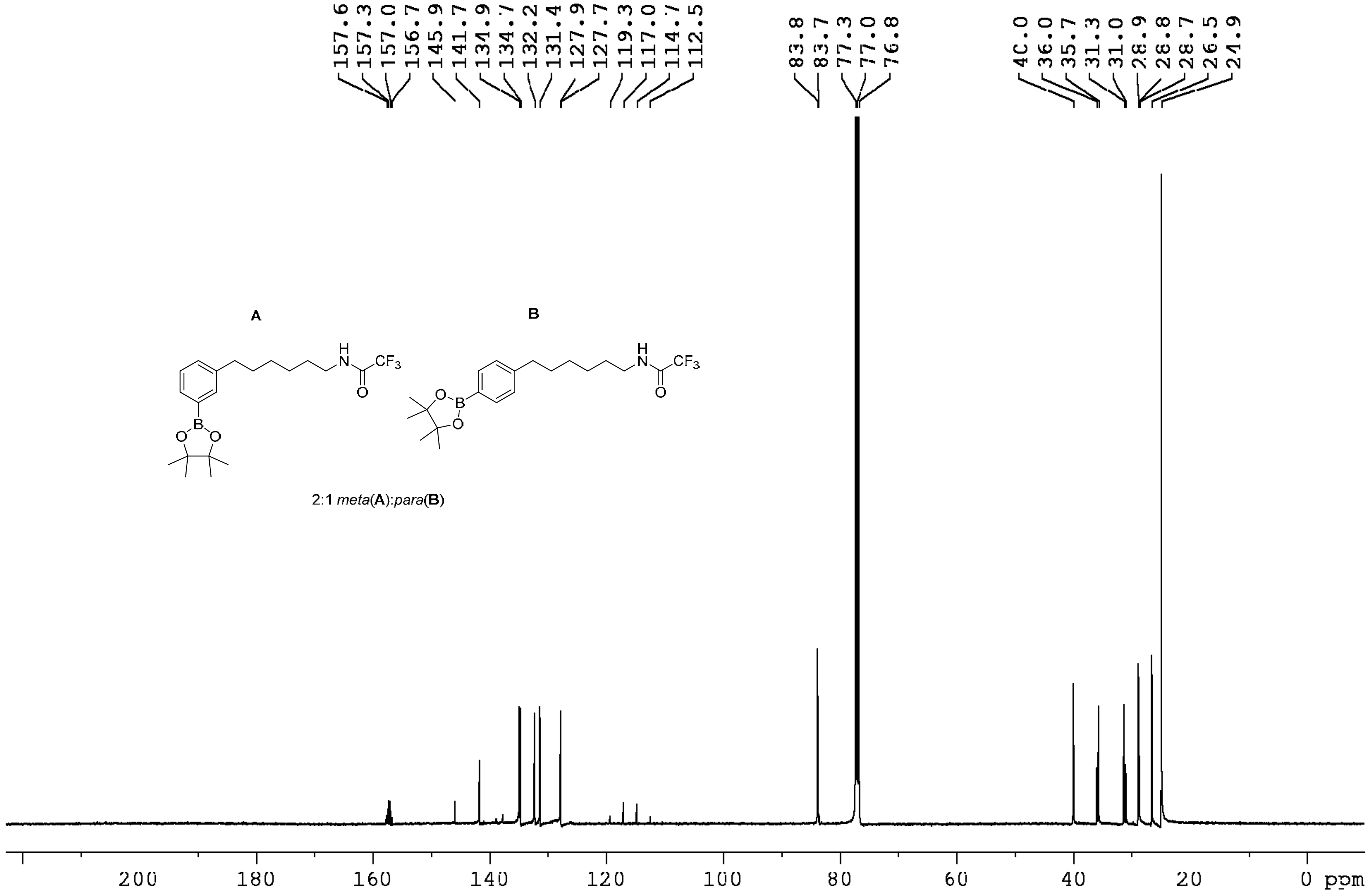

^{1}H NMR (500 MHz, $CDCl_3$) 2,2,2-Trifluoro-*N*-(6-(4,4,5,5-tetramethyl-1,3,2-dioxaborolan-2-yl)-2,3-dihydro-1H-inden-1-yl)acetamide (8a) – Crude after reaction with 1a

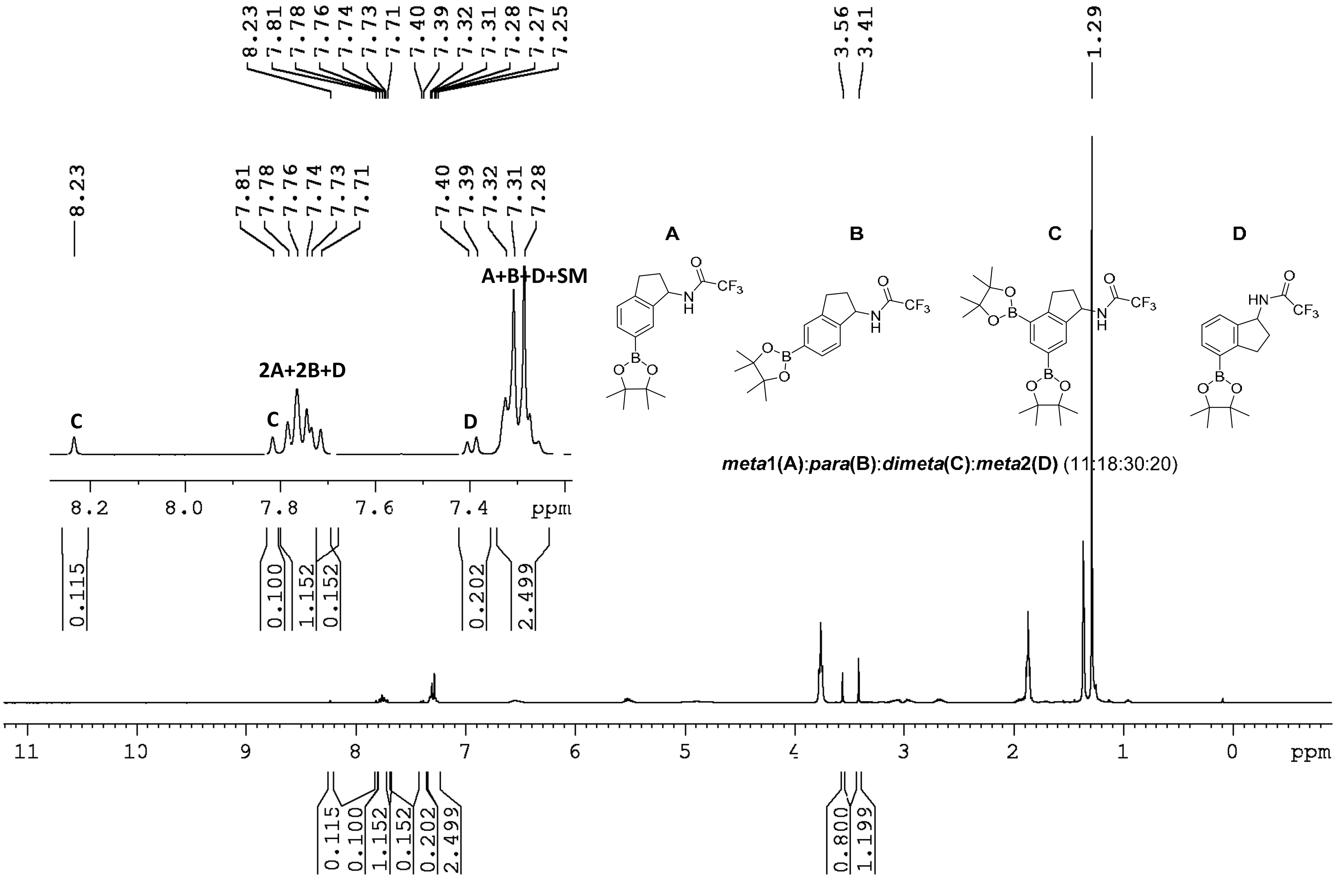

^{1}H NMR (600 MHz, $CDCl_3$) 2,2,2-Trifluoro-*N*-(4-(4,4,5,5-tetramethyl-1,3,2-dioxaborolan-2-yl)-2,3-dihydro-1H-inden-1-yl)acetamide (8a) – Purfied after reaction with 1a

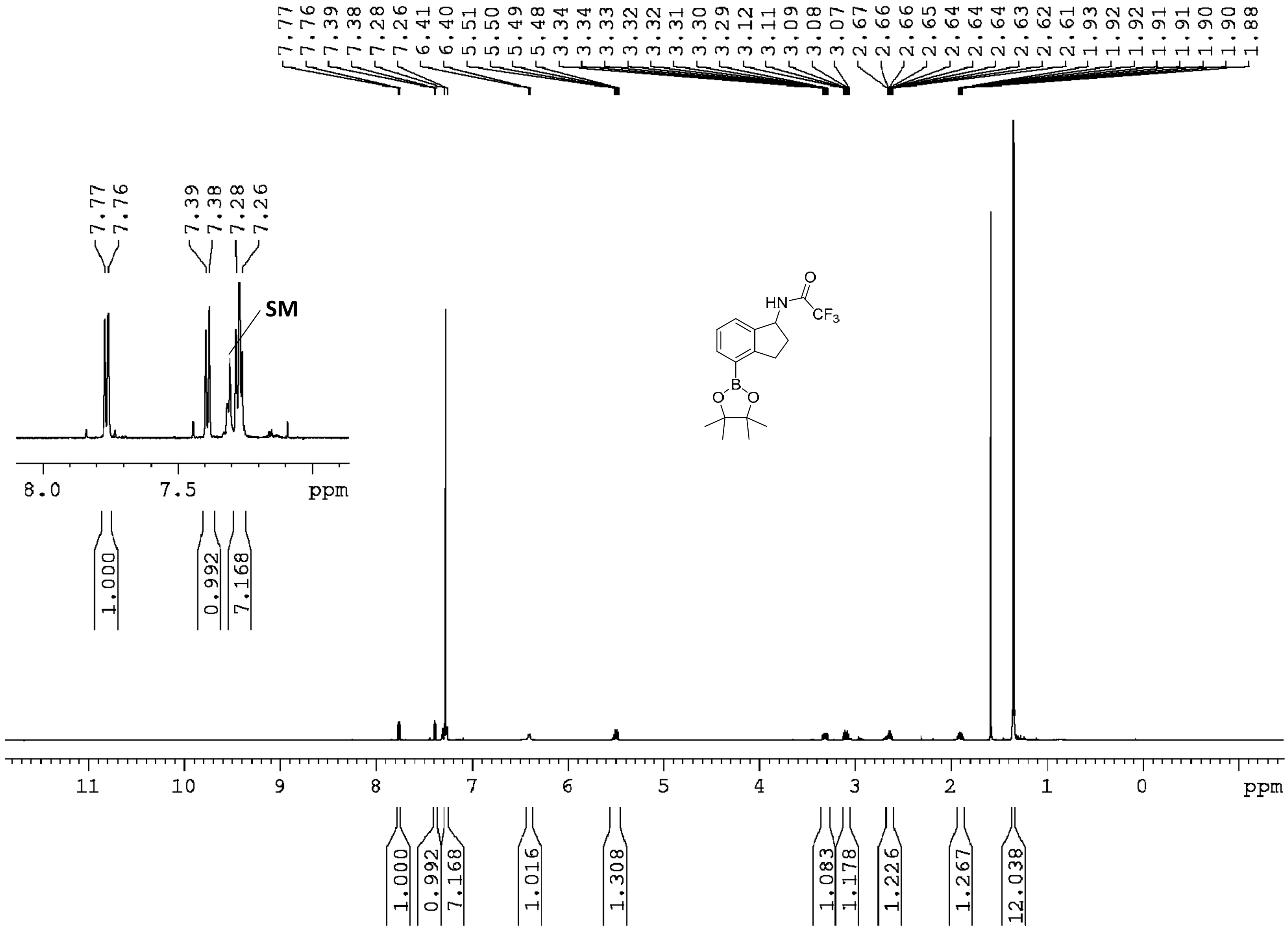

^{13}C NMR (126 MHz, $CDCl_3$) 2,2,2-Trifluoro-*N*-(4-(4,4,5,5-tetramethyl-1,3,2-dioxaborolan-2-yl)-2,3-dihydro-1H-inden-1-yl)acetamide (8a) – Purified after reaction with 1a

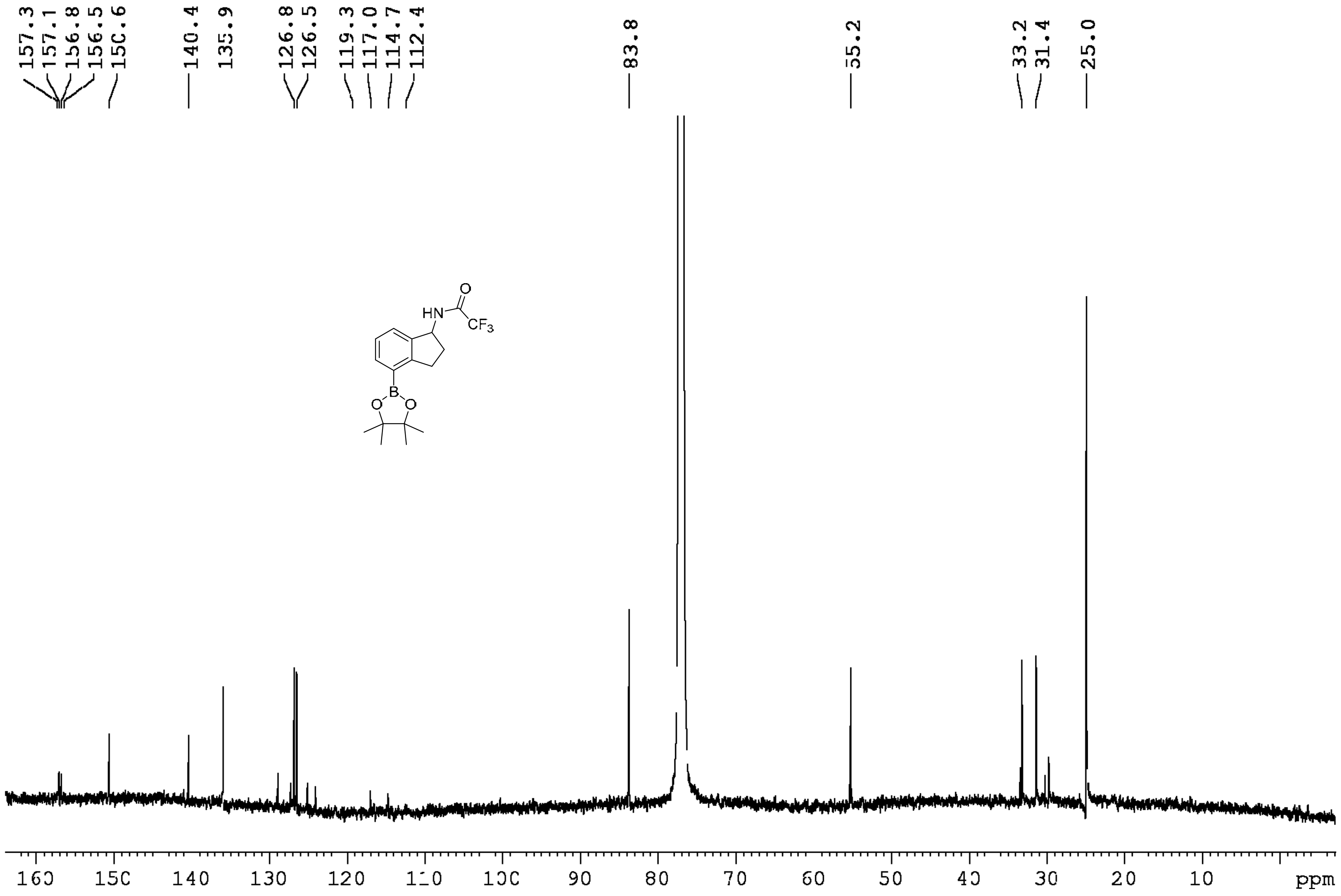

^{1}H NMR (500 MHz, $CDCl_3$) 2,2,2-Trifluoro-*N*-(6-(4,4,5,5-tetramethyl-1,3,2-dioxaborolan-2-yl)-2,3-dihydro-1H-inden-1-yl)acetamide (8a) – Purfied after reaction with 1a

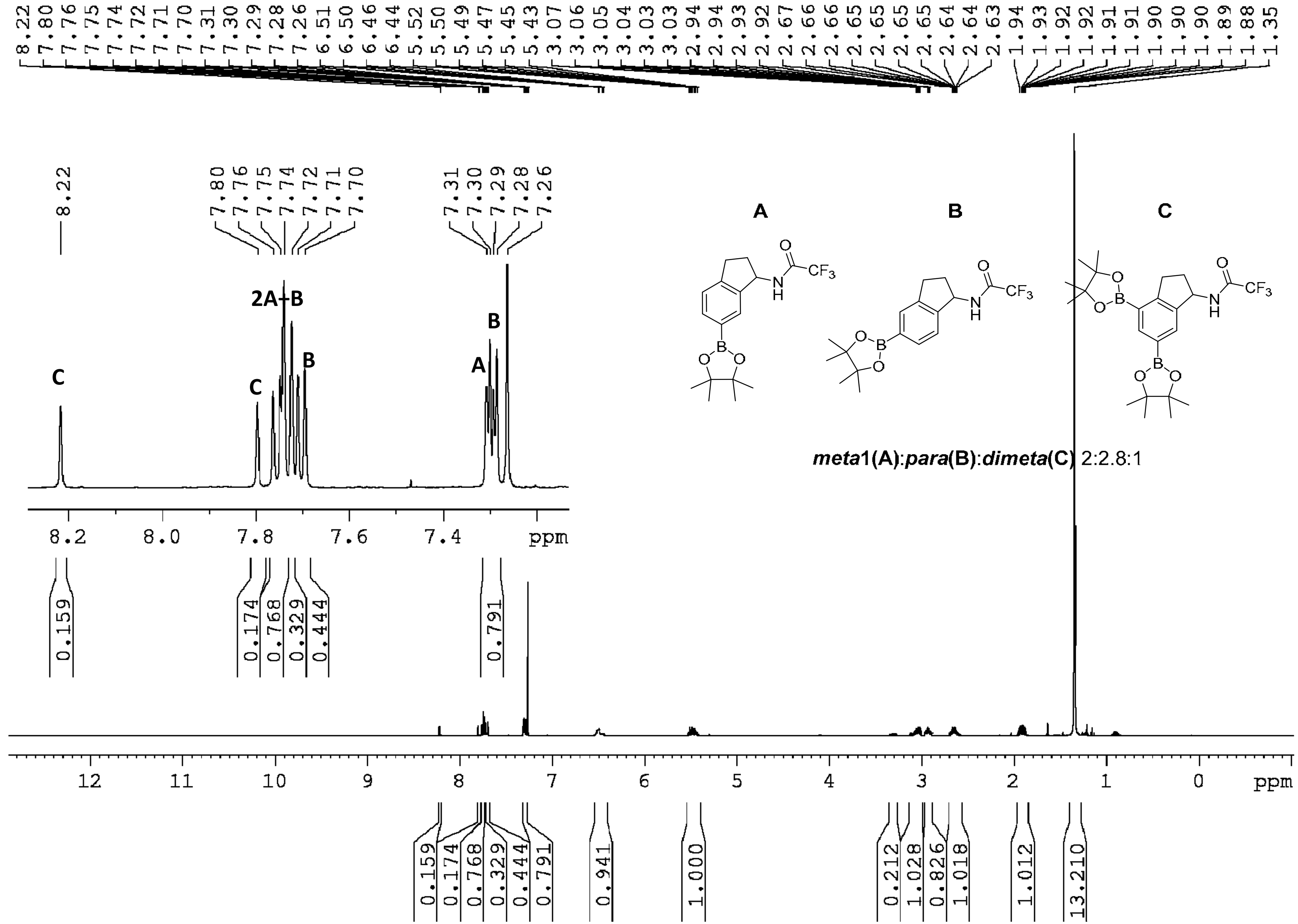

^{13}C NMR (125 MHz, CDCl$_3$) 2,2,2-Trifluoro-*N*-(6-(4,4,5,5-tetramethyl-1,3,2-dioxaborolan-2-yl)-2,3-dihydro-1H-inden-1-yl)acetamide (8a) – Purfied after reaction with 1a

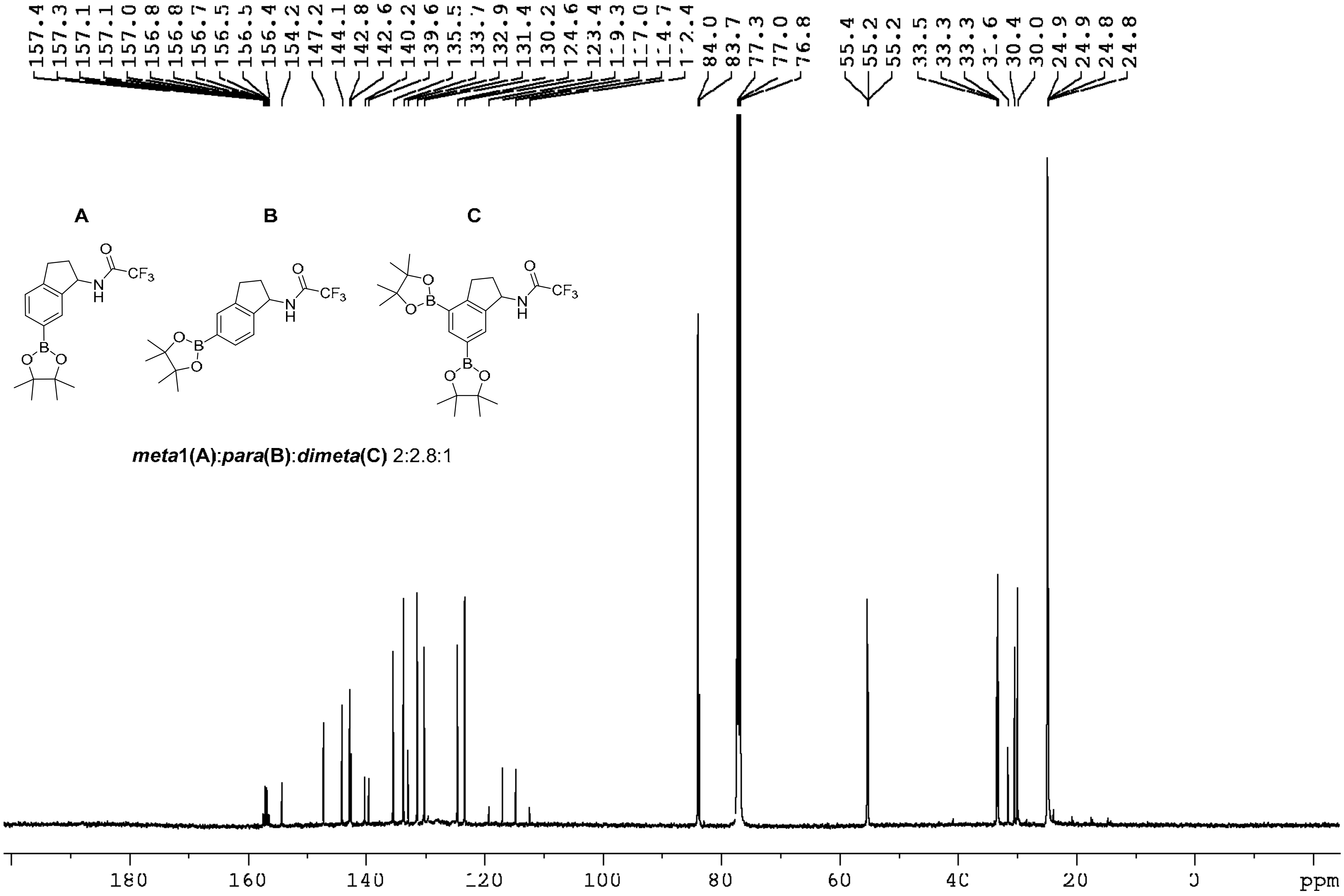

^{1}H NMR (600 MHz, $CDCl_3$) of (cis)-2,2,2-trifluoro-N-(2-(3-(4,4,5,5-tetramethyl-1,3,2-dioxaborolan-2-yl)phenyl)cyclohexyl)acetamide (*cis*-9a) – Dtbpy – Purified

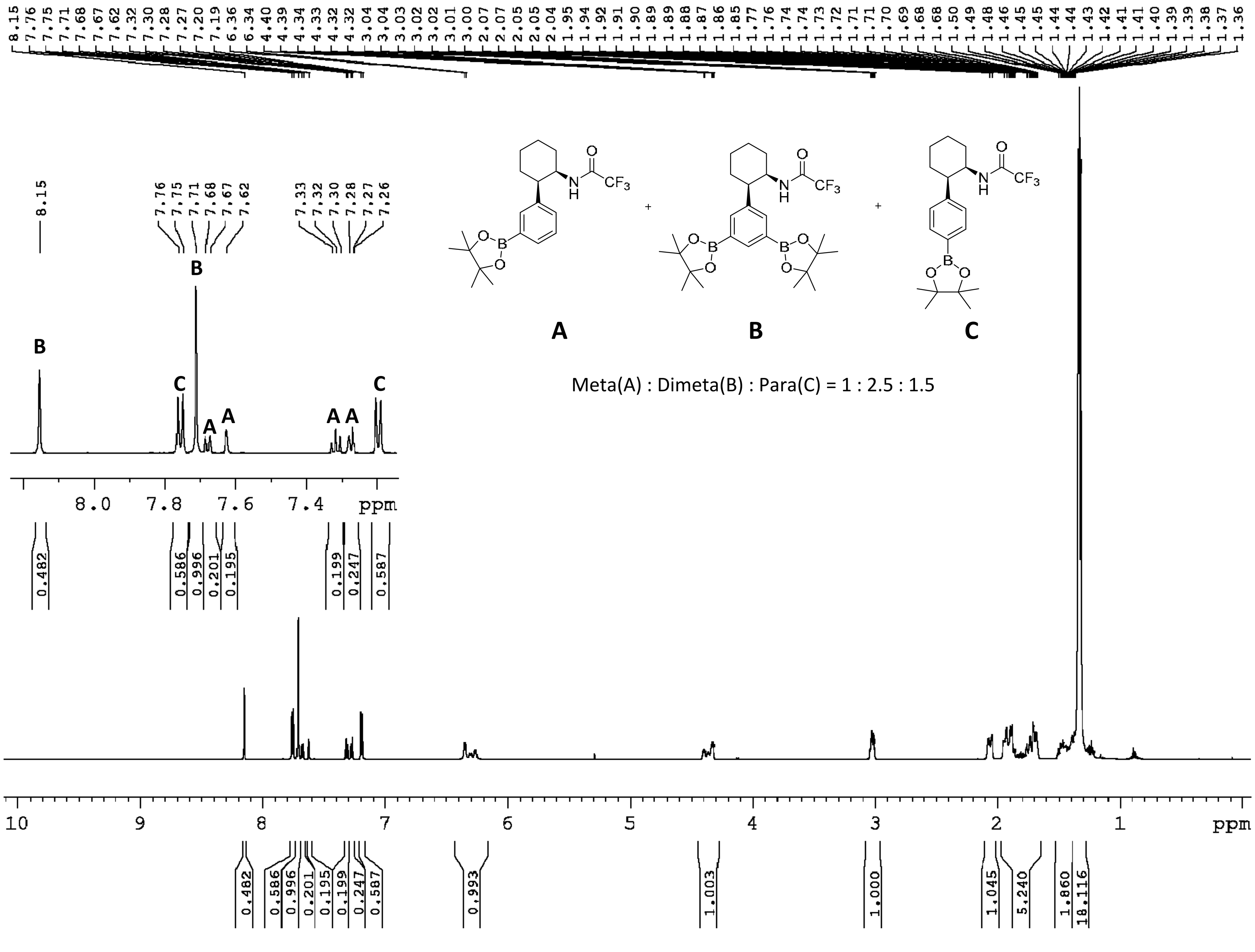

^{13}C NMR (151 MHz, $CDCl_3$) of (cis)-2,2,2-trifluoro-N-(2-(3-(4,4,5,5-tetramethyl-1,3,2-dioxaborolan-2-yl)phenyl)cyclohexyl)acetamide (*cis*-9a) – Dtbpy – Purified

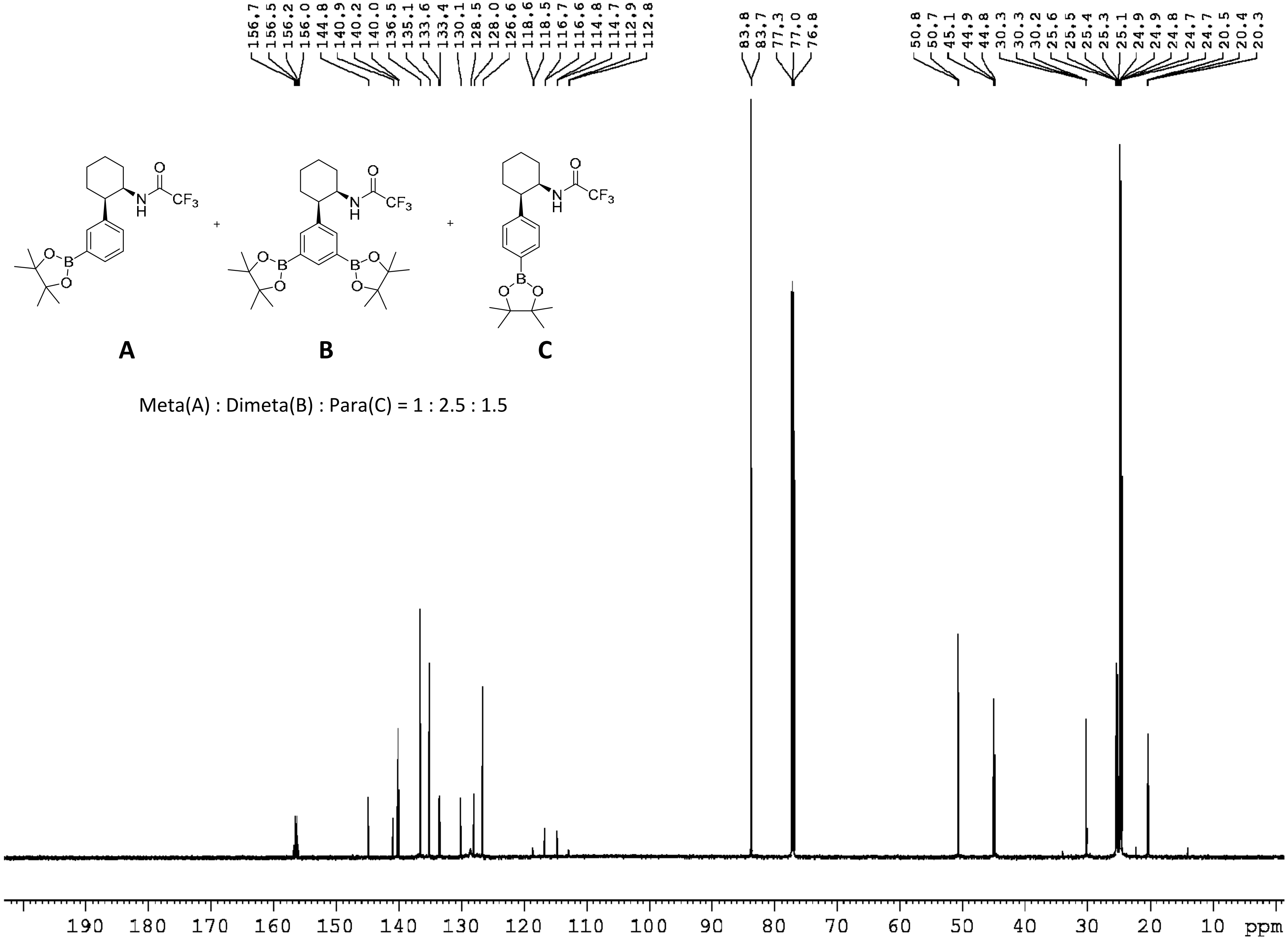

^{1}H NMR (500 MHz, $CDCl_3$) of (cis)-2,2,2-trifluoro-N-(2-(3-(4,4,5,5-tetramethyl-1,3,2-dioxaborolan-2-yl)phenyl)cyclohexyl)acetamide (*cis*-9a) – Ligand 1a – Crude mixture

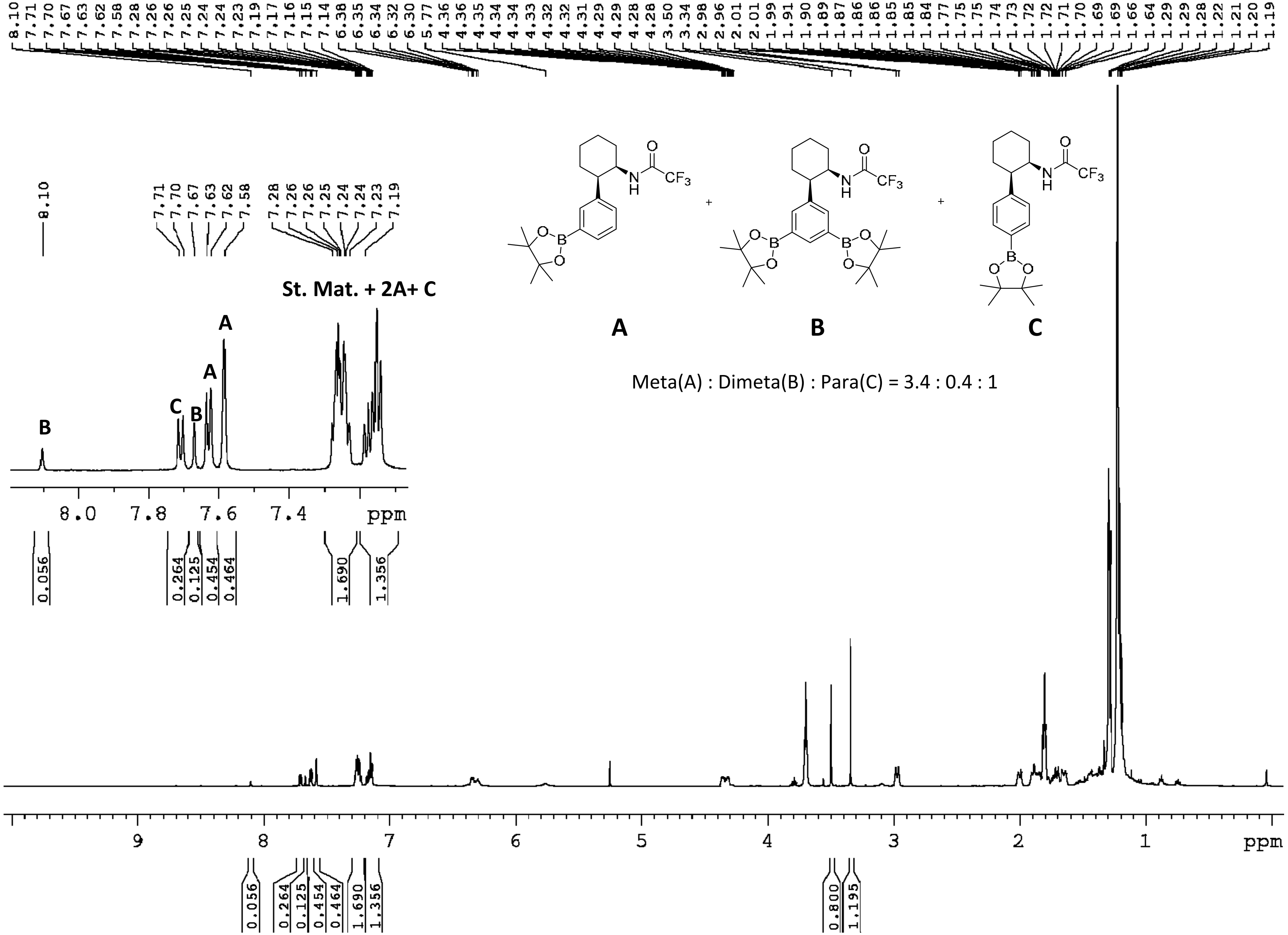

^{1}H NMR (500 MHz, $CDCl_3$) of (cis)-2,2,2-trifluoro-N-(2-(3-(4,4,5,5-tetramethyl-1,3,2-dioxaborolan-2-yl)phenyl)cyclohexyl)acetamide (*cis*-9a) – Ligand 1a – Purified mono-borylated products

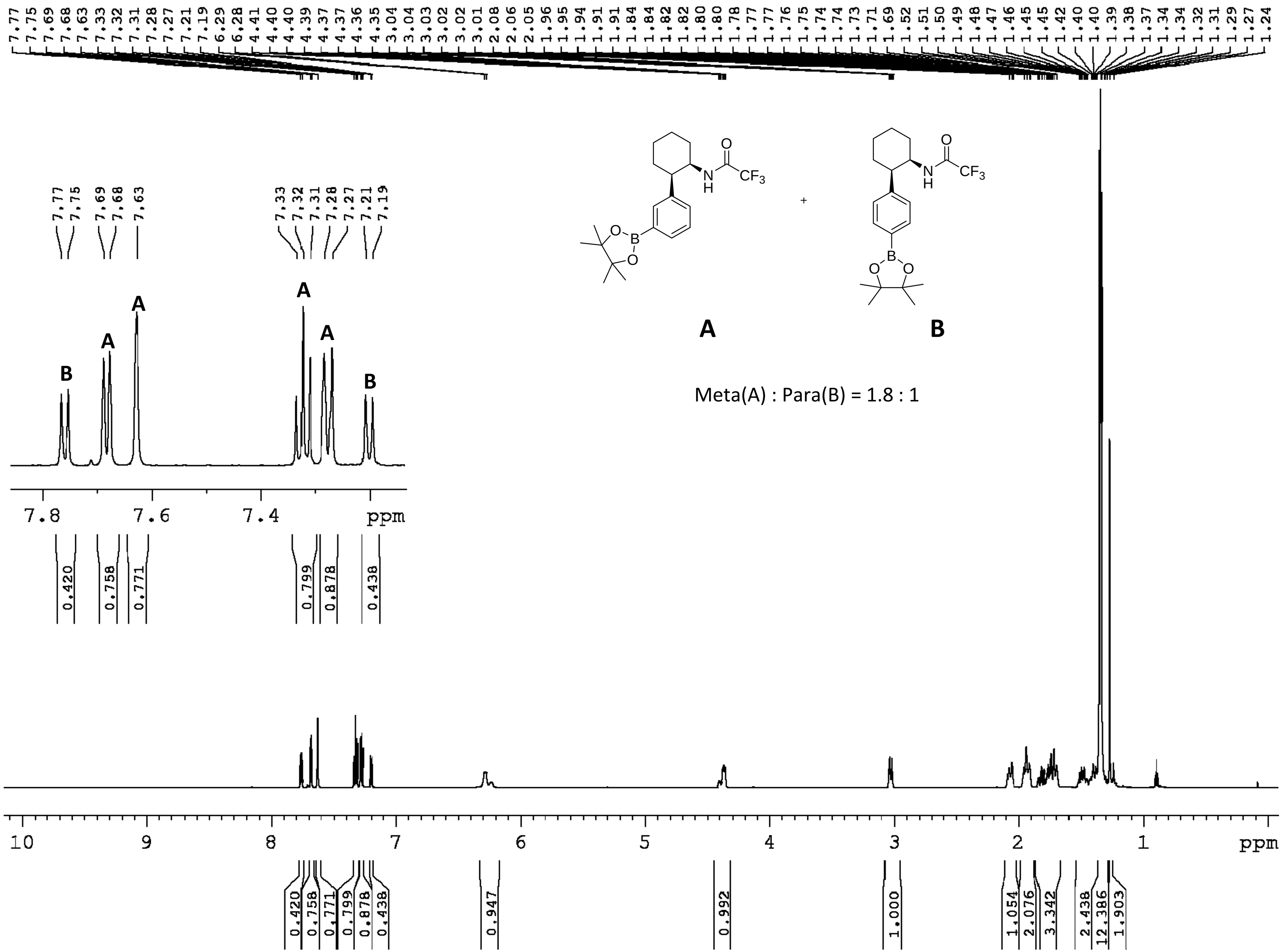

^{13}C NMR (126 MHz, $CDCl_3$) of (cis)-2,2,2-trifluoro-N-(2-(3-(4,4,5,5-tetramethyl-1,3,2-dioxaborolan-2-yl)phenyl)cyclohexyl)acetamide (*cis*-9a) – Ligand 1a – Purified mono-borylated products

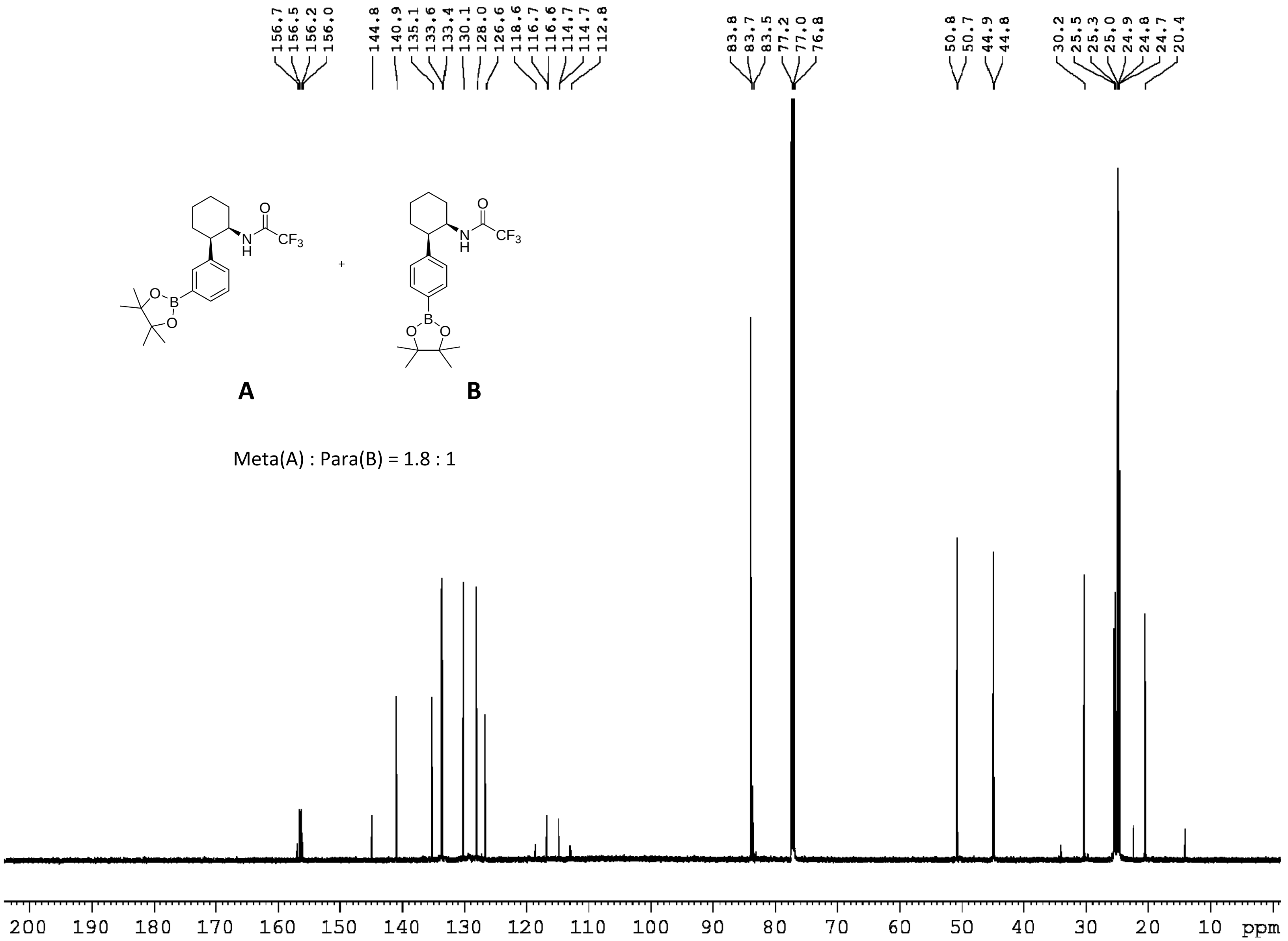

^{1}H NMR (600 MHz, $CDCl_3$) of (trans)-2,2,2-trifluoro-N-(2-(3-(4,4,5,5-tetramethyl-1,3,2-dioxaborolan-2-yl)phenyl)cyclohexyl)acetamide (*trans*-9a) – Dtbpy – Purified

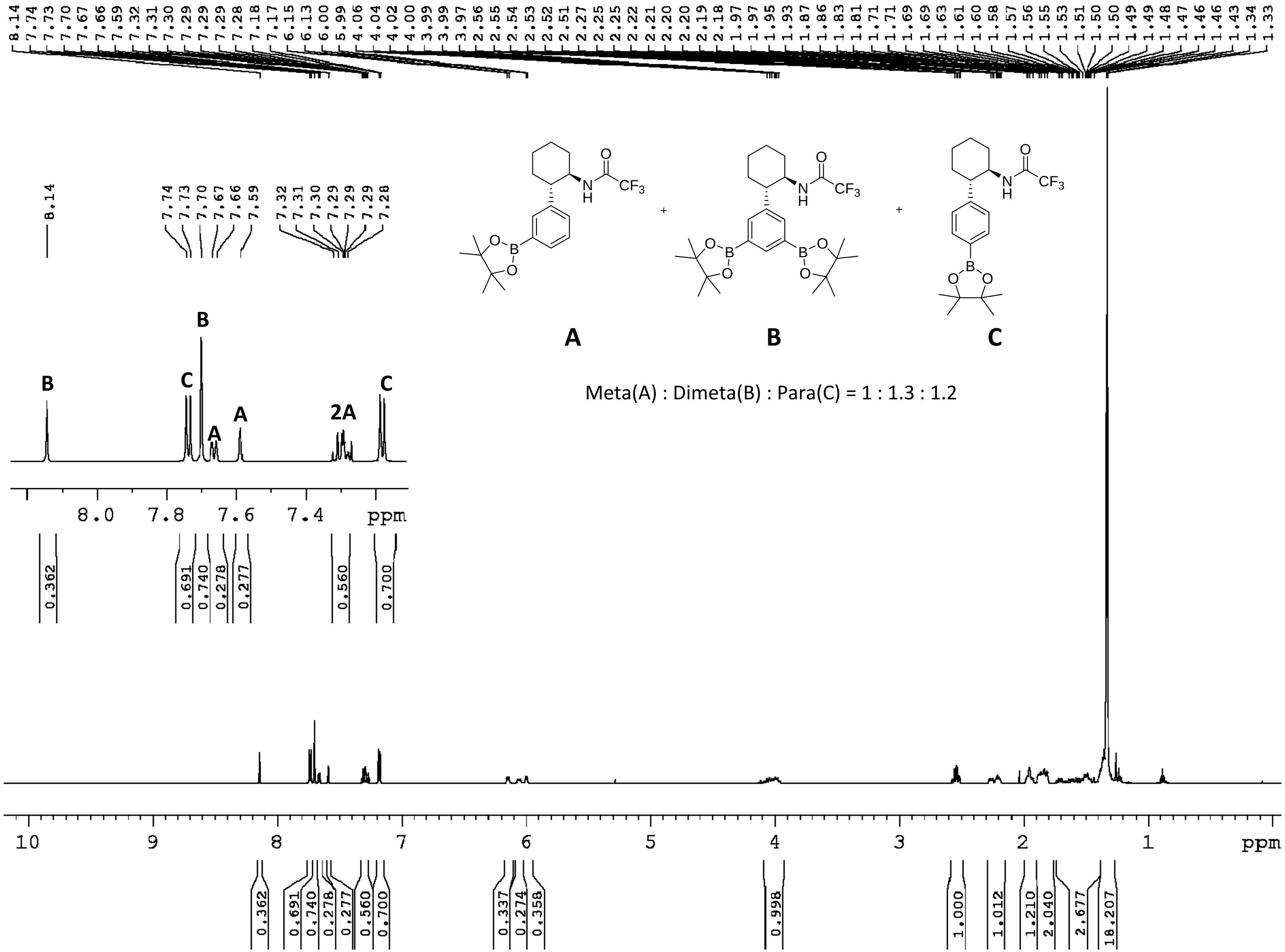

^{13}C NMR (151 MHz, $CDCl_3$) of (trans)-2,2,2-trifluoro-N-(2-(3-(4,4,5,5-tetramethyl-1,3,2-dioxaborolan-2-yl)phenyl)cyclohexyl)acetamide (*trans*-9a) – Dtbpy – Purified

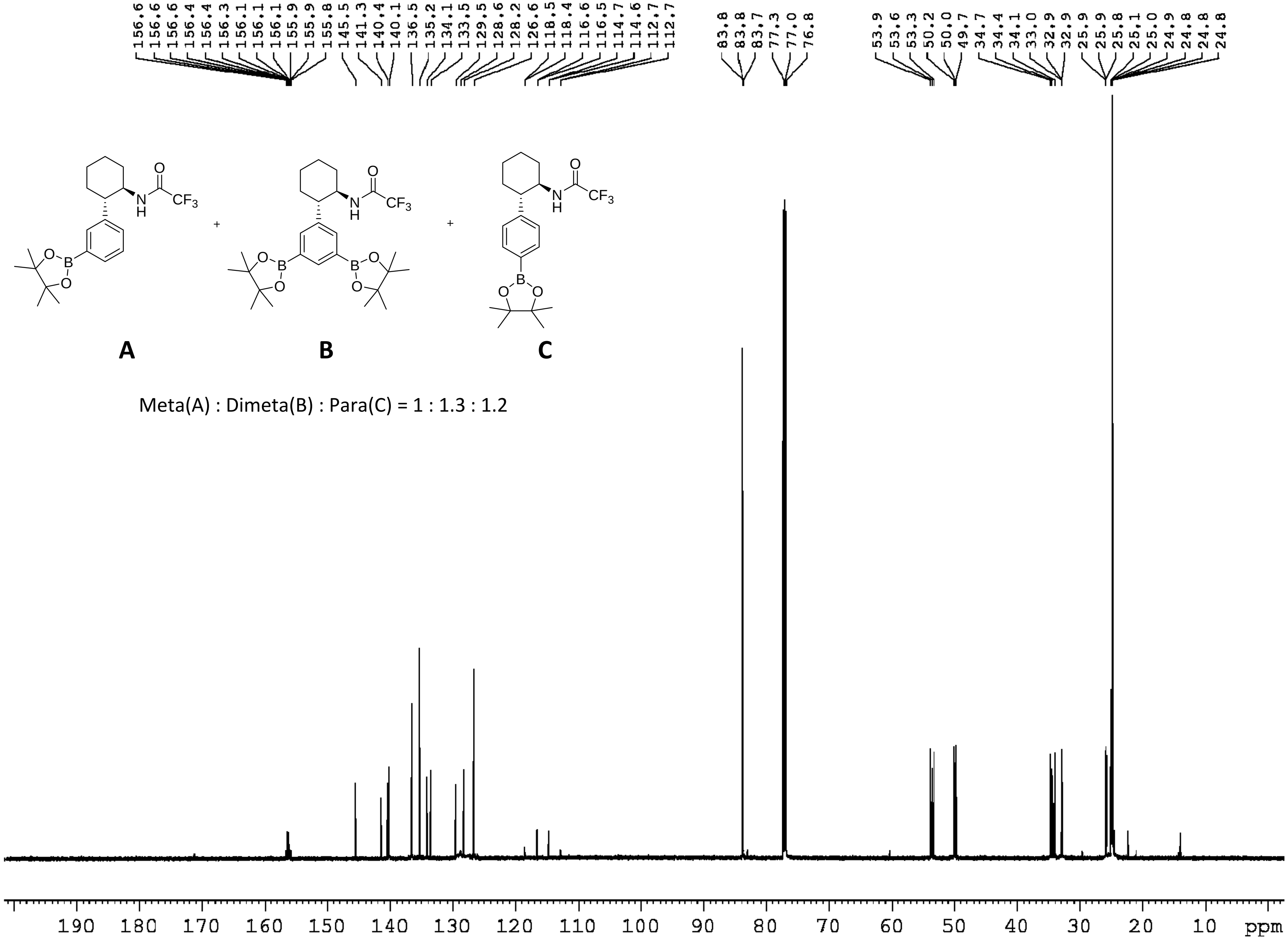

^{1}H NMR (500 MHz, $CDCl_3$) of (trans)-2,2,2-trifluoro-N-(2-(3-(4,4,5,5-tetramethyl-1,3,2-dioxaborolan-2-yl)phenyl)cyclohexyl)acetamide (*trans*-9a) – Ligand 1a – Crude mixture

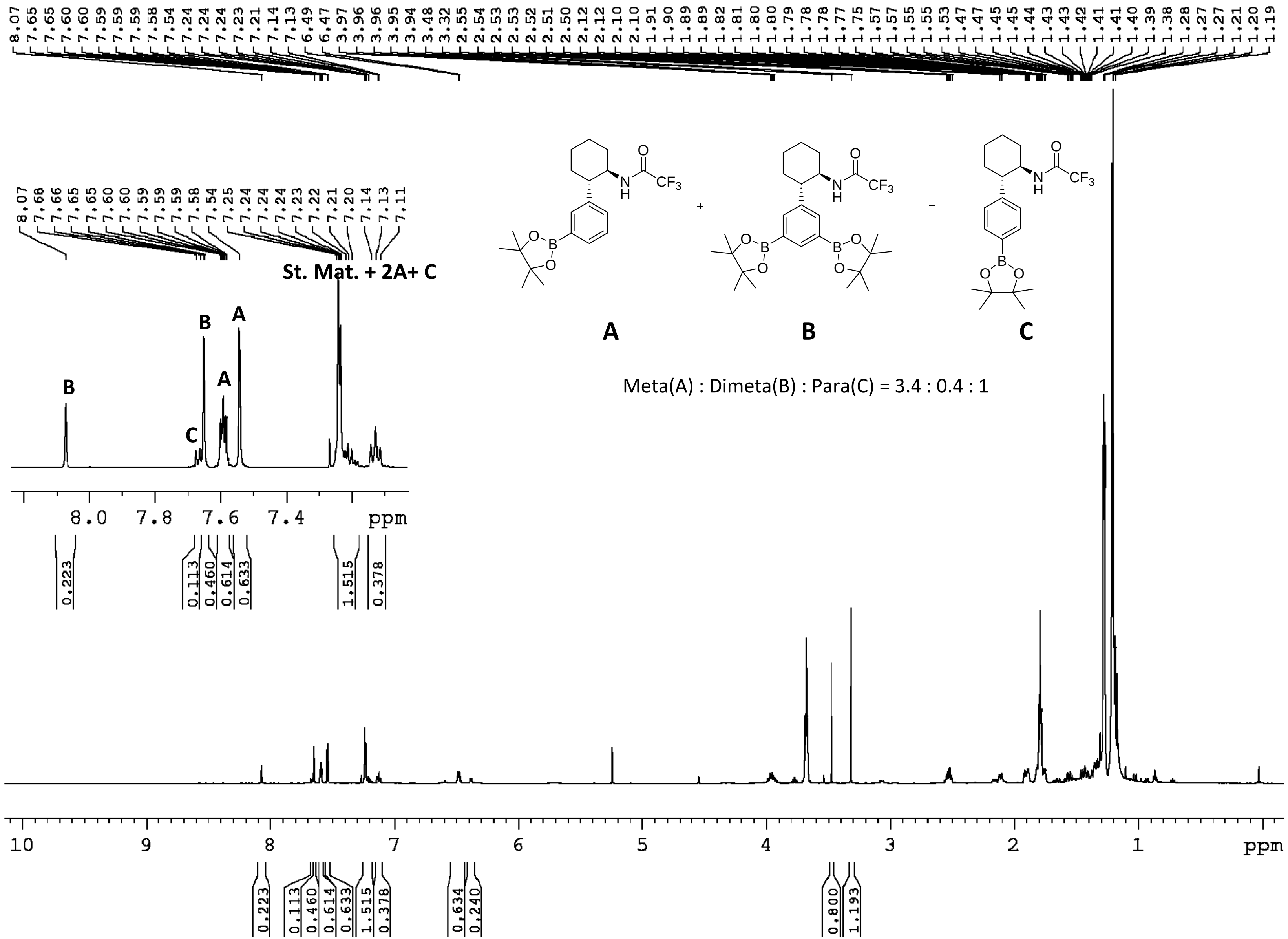

^{1}H NMR (500 MHz, $CDCl_3$) of (trans)-2,2,2-trifluoro-N-(2-(3-(4,4,5,5-tetramethyl-1,3,2-dioxaborolan-2-yl)phenyl)cyclohexyl)acetamide (*trans*-9a) – Ligand 1a – Purified monoborylated products

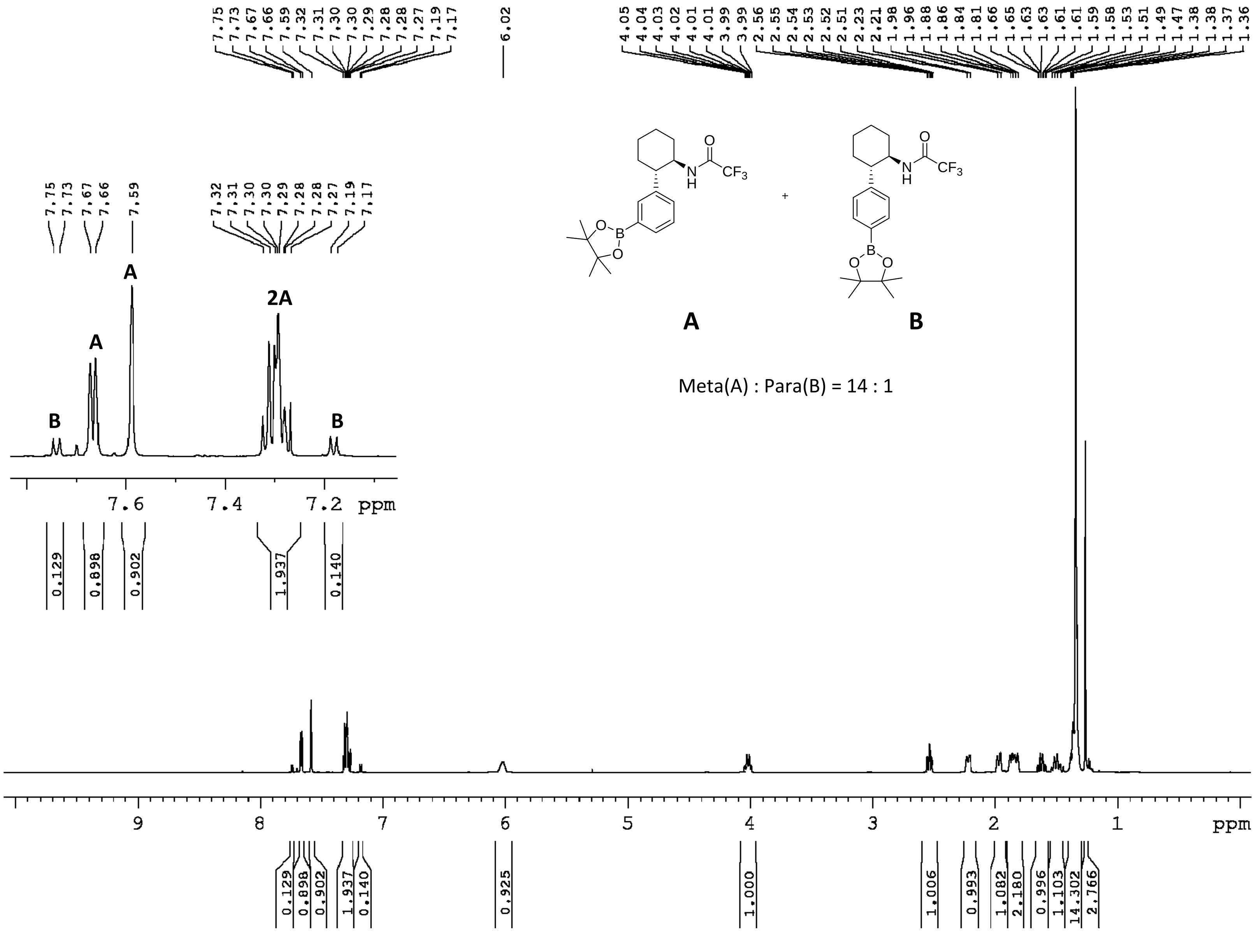

^{13}C NMR (126 MHz, $CDCl_3$) of (trans)-2,2,2-trifluoro-N-(2-(3-(4,4,5,5-tetramethyl-1,3,2-dioxaborolan-2-yl)phenyl)cyclohexyl)acetamide (*trans*-9a) – Ligand 1a – Purified monoborylated products

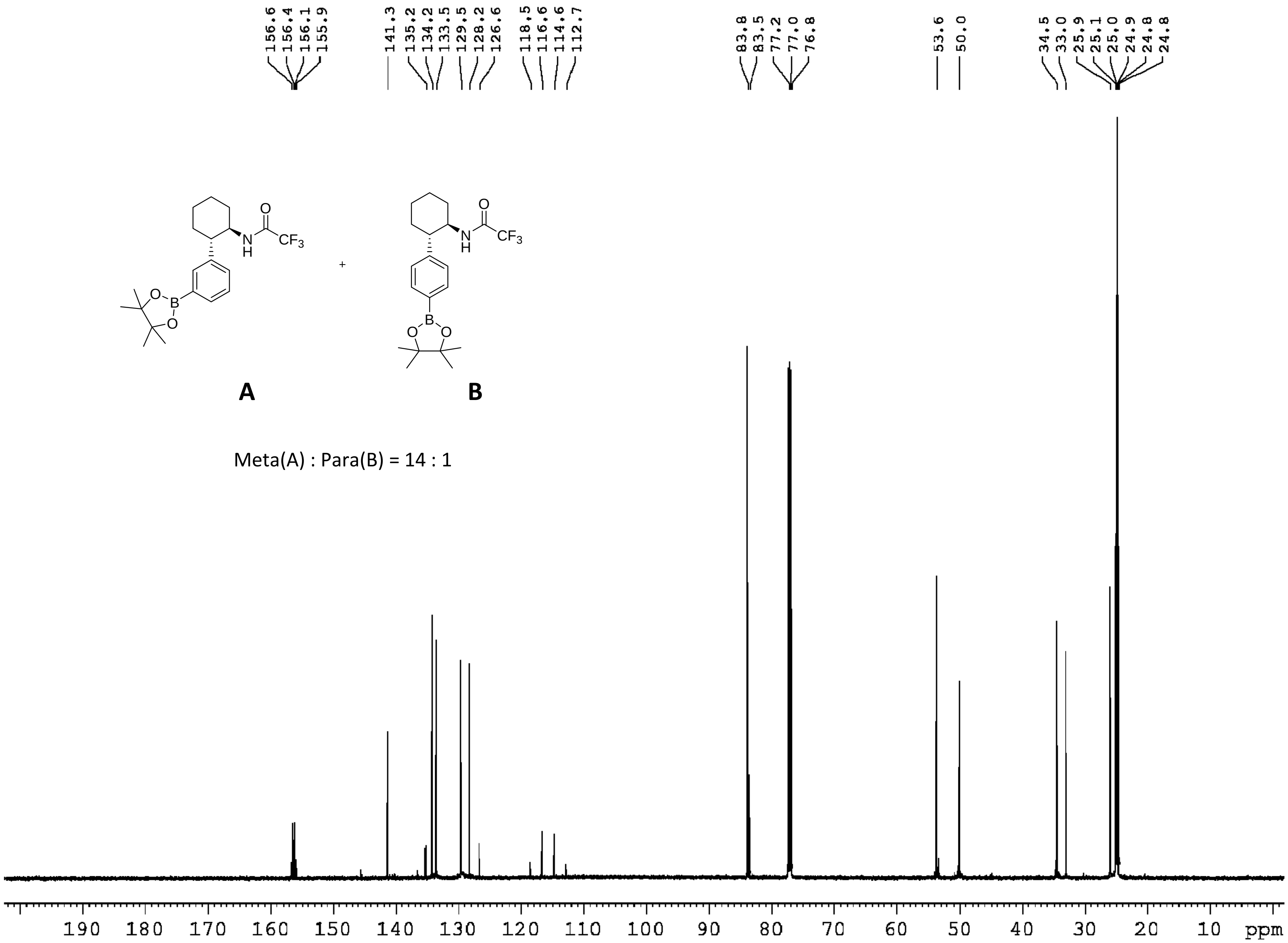

^{1}H NMR (600 MHz, $CDCl_3$) *N*-(2-bromo-5-(4,4,5,5-tetramethyl-1,3,2-dioxaborolan-2-yl)phenyl)acetamide – Crude after reaction with 1a

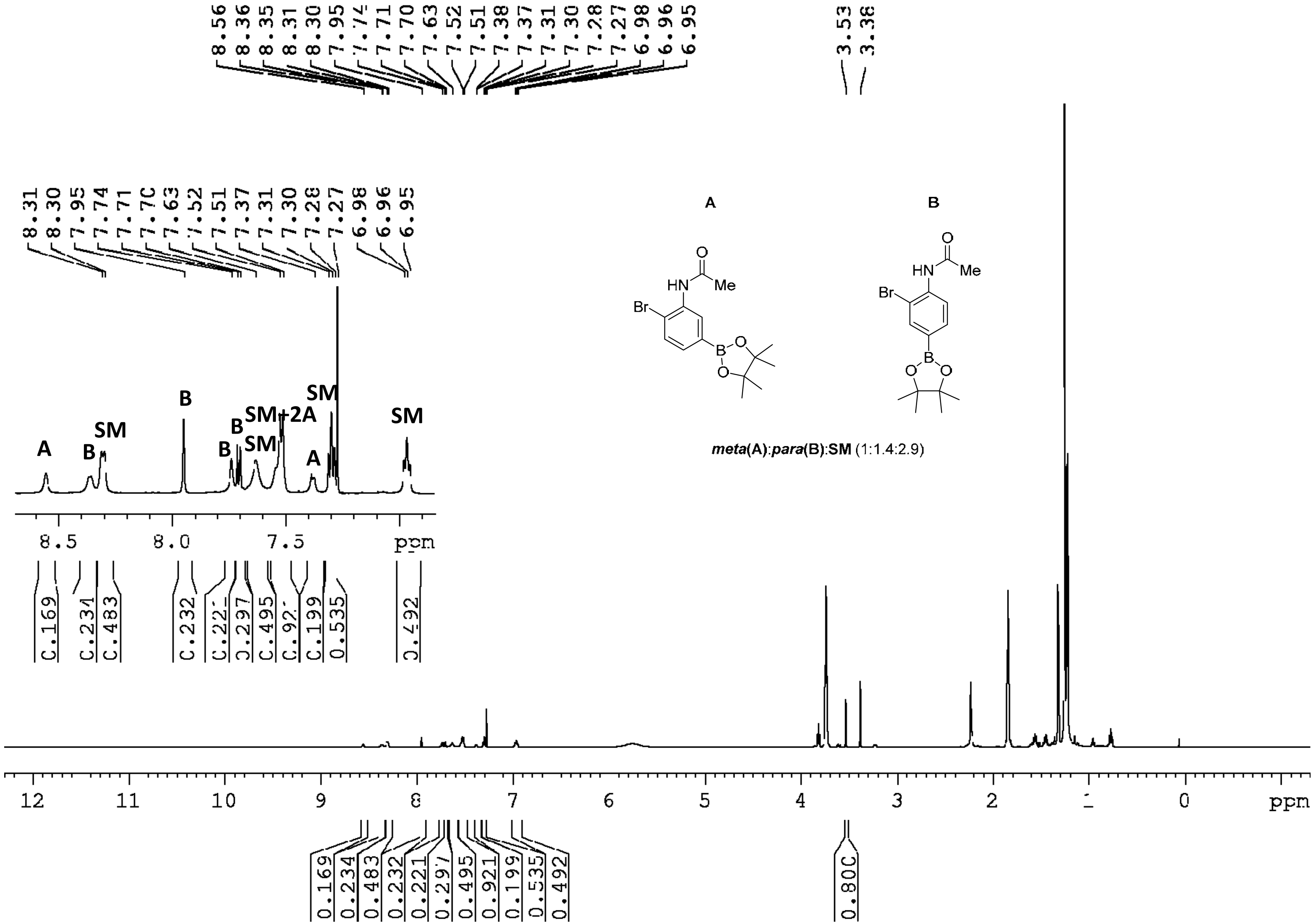

^{1}H NMR (500 MHz, CDCl$_3$) *N*-(2-bromo-5-(4,4,5,5-tetramethyl-1,3,2-dioxaborolan-2-yl)phenyl)acetamide – Purfied after reaction with 1a

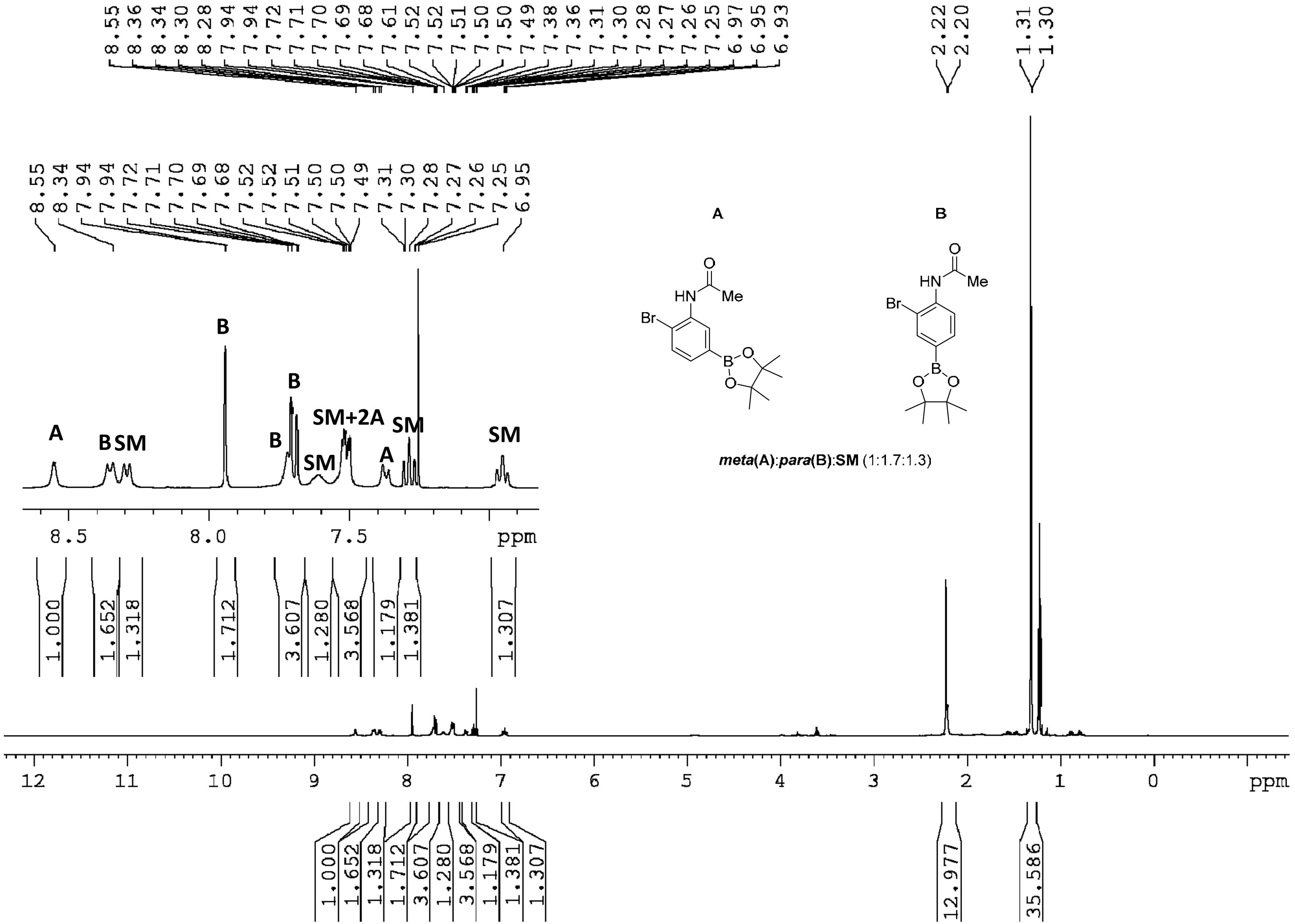

^{13}C NMR (125 MHz, $CDCl_3$) *N*-(2-bromo-5-(4,4,5,5-tetramethyl-1,3,2-dioxaborolan-2-yl)phenyl)acetamide – Purfied after reaction with 1a

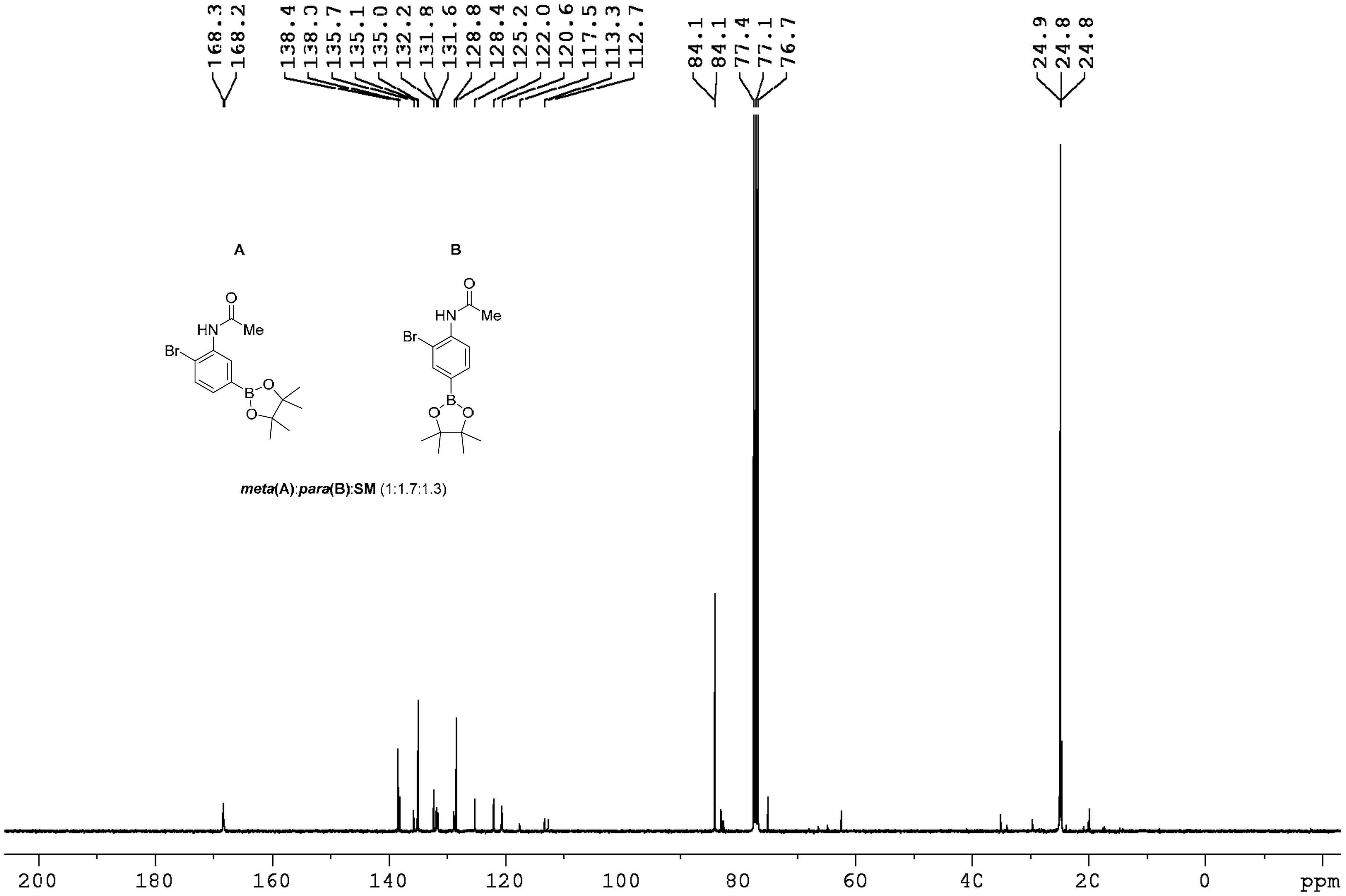

^{1}H NMR (400 MHz, $CDCl_3$) *N*-(2-chloro-5-(4,4,5,5-tetramethyl-1,3,2-dioxaborolan-2-yl)phenyl)acetamide – Crude after reaction with 1a

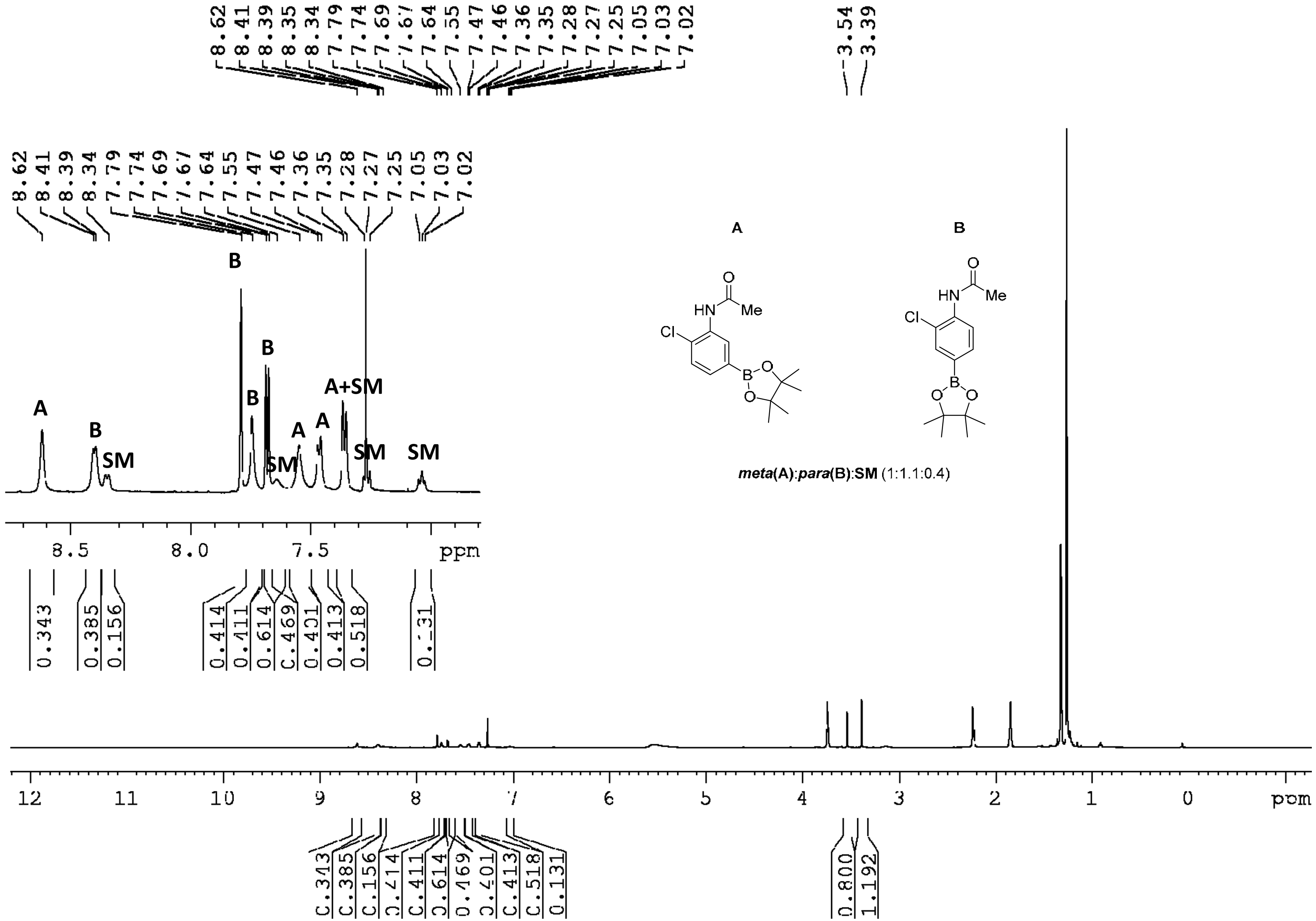

^{1}H NMR (600 MHz, $CDCl_3$) *N*-(2-chloro-5-(4,4,5,5-tetramethyl-1,3,2-dioxaborolan-2-yl)phenyl)acetamide – Purfied after reaction with 1a

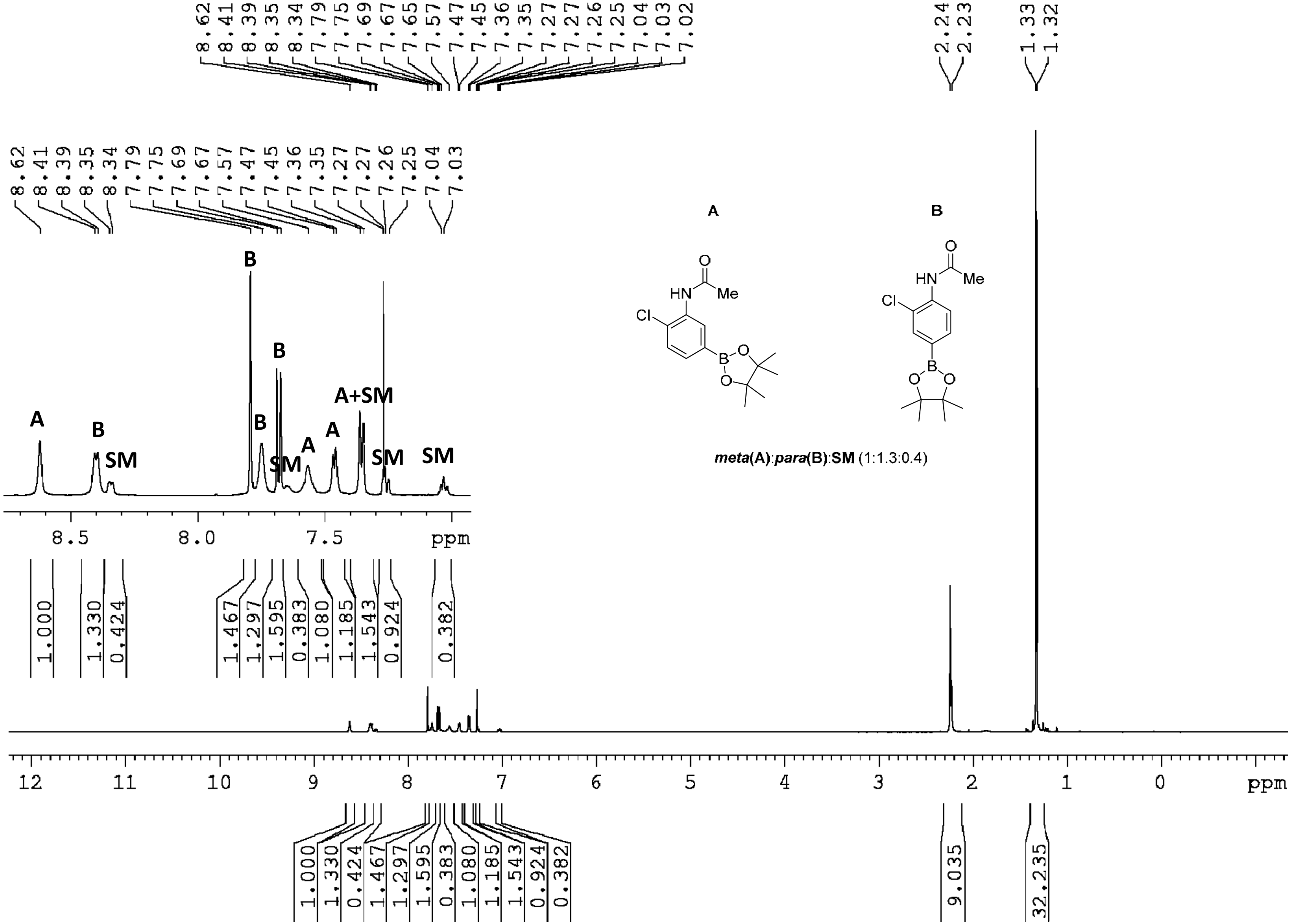

^{13}C NMR (151 MHz, $CDCl_3$) *N*-(2-chloro-5-(4,4,5,5-tetramethyl-1,3,2-dioxaborolan-2-yl)phenyl)acetamide – Purfied after reaction with 1a

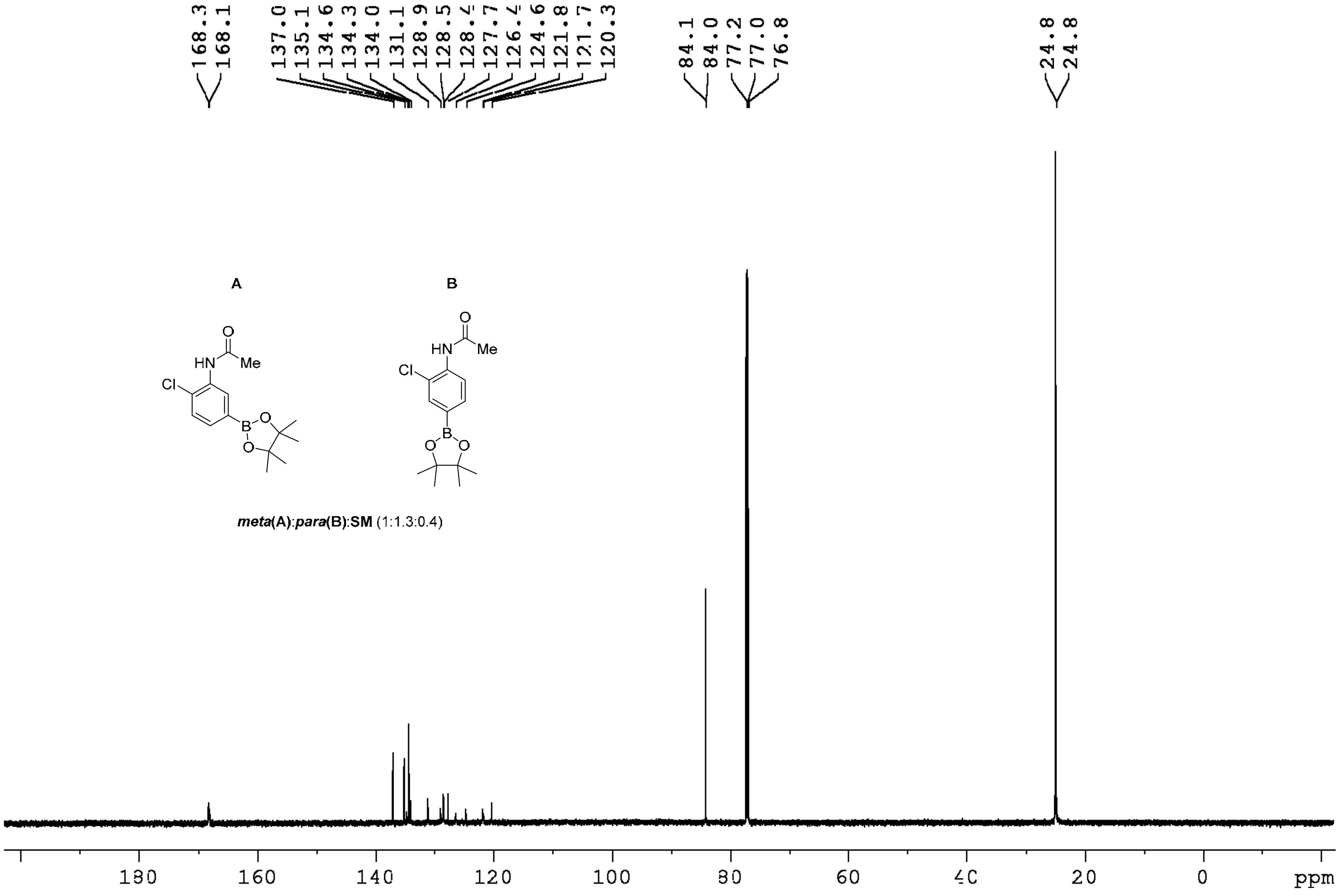

^{1}H NMR (600 MHz, $CDCl_3$) *N*-ethyl-2,2,2-trifluoro-N-(5-(4,4,5,5-tetramethyl-1,3,2-dioxaborolan-2-yl)-2-(trifluoromethyl)benzyl)acetamide (13a) – Crude as a mixture of rotamers after reaction with 1a

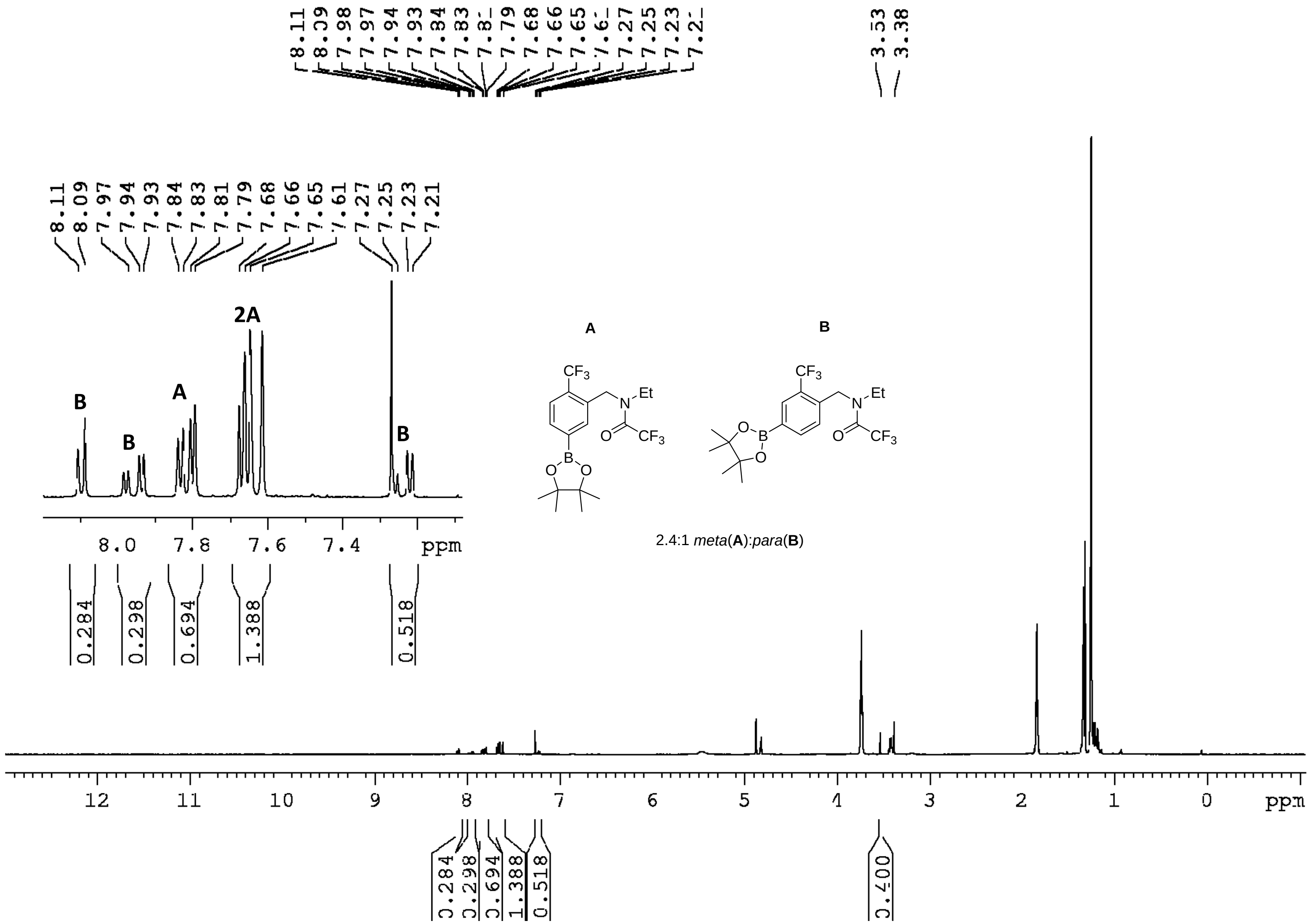

^{1}H NMR (600 MHz, $CDCl_3$) *N*-ethyl-2,2,2-trifluoro-N-(5-(4,4,5,5-tetramethyl-1,3,2-dioxaborolan-2-yl)-2-(trifluoromethyl)benzyl)acetamide (13a) – Purified as a mixture of rotamers after reaction with 1a (to show new product ratio)

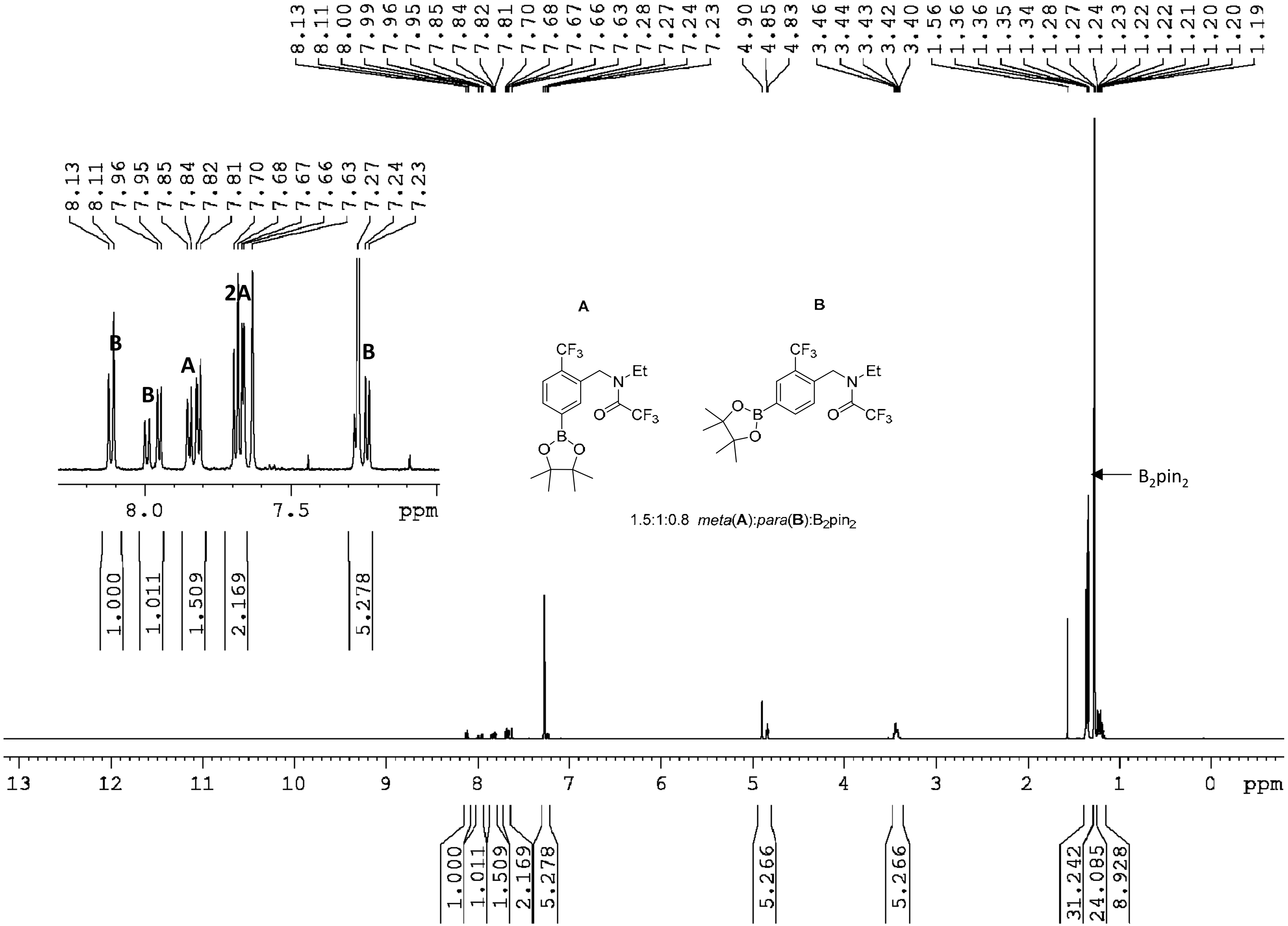

^{1}H NMR (500 MHz, DMSO-d_6 – 120 °C) *N*-ethyl-2,2,2-trifluoro-N-(5-(4,4,5,5-tetramethyl-1,3,2-dioxaborolan-2-yl)-2-(trifluoromethyl)benzyl)acetamide (13a) – Purified after reaction with 1a heated to remove rotamers

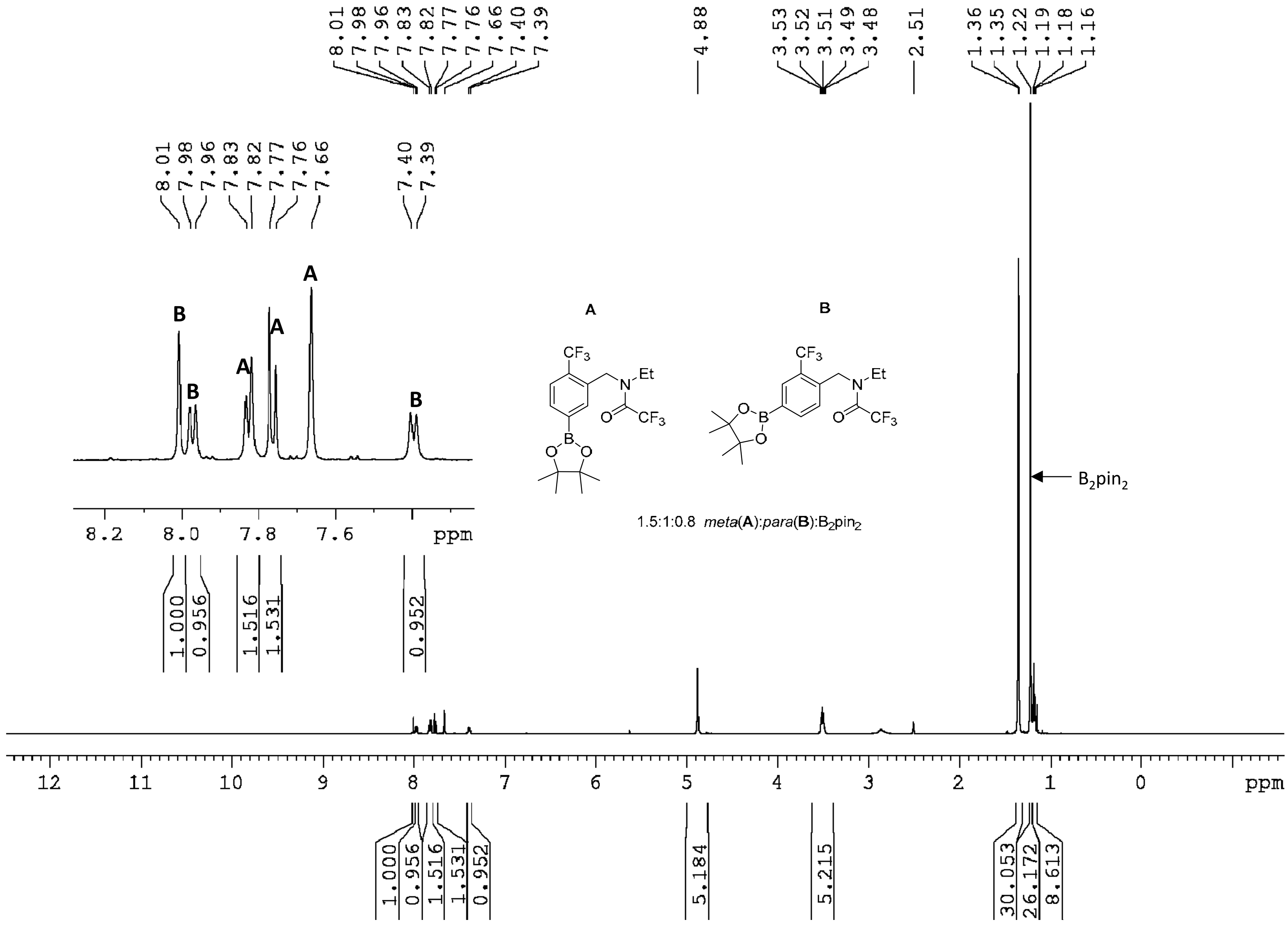

^{13}C NMR (126 MHz, DMSO-d_6 – 120 °C) *N*-ethyl-2,2,2-trifluoro-N-(5-(4,4,5,5-tetramethyl-1,3,2-dioxaborolan-2-yl)-2-(trifluoromethyl)benzyl)acetamide (13a) – Purified after reaction with 1a – heated to remove rotamers

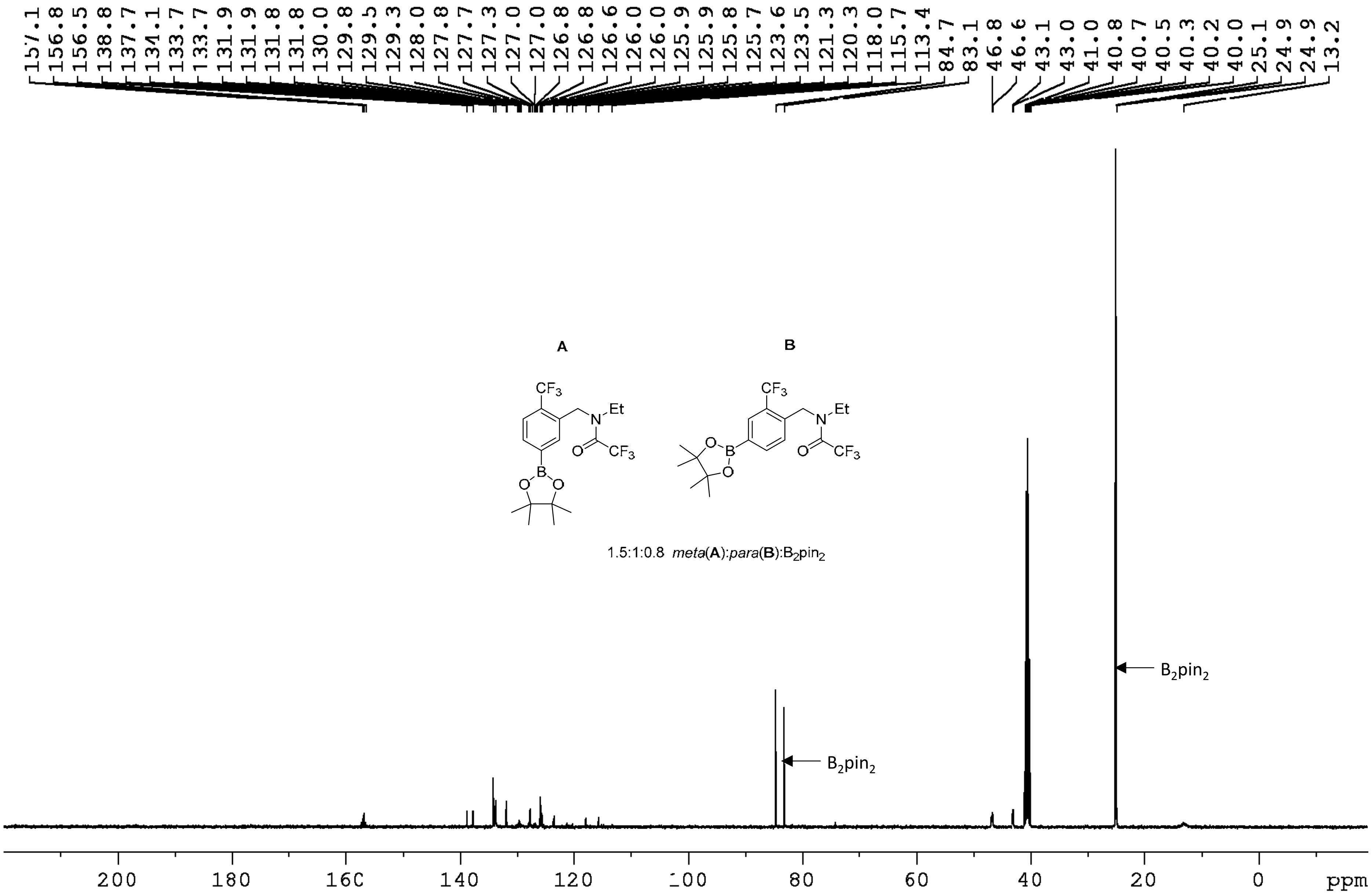

¹H NMR (500 MHz, $CDCl_3$) of N-(2-bromo-5-(4,4,5,5-tetramethyl-1,3,2-dioxaborolan-2-yl)phenethyl)-N-ethyl-2,2,2-trifluoroacetamide (14a) – Ligand 1a – Crude mixture

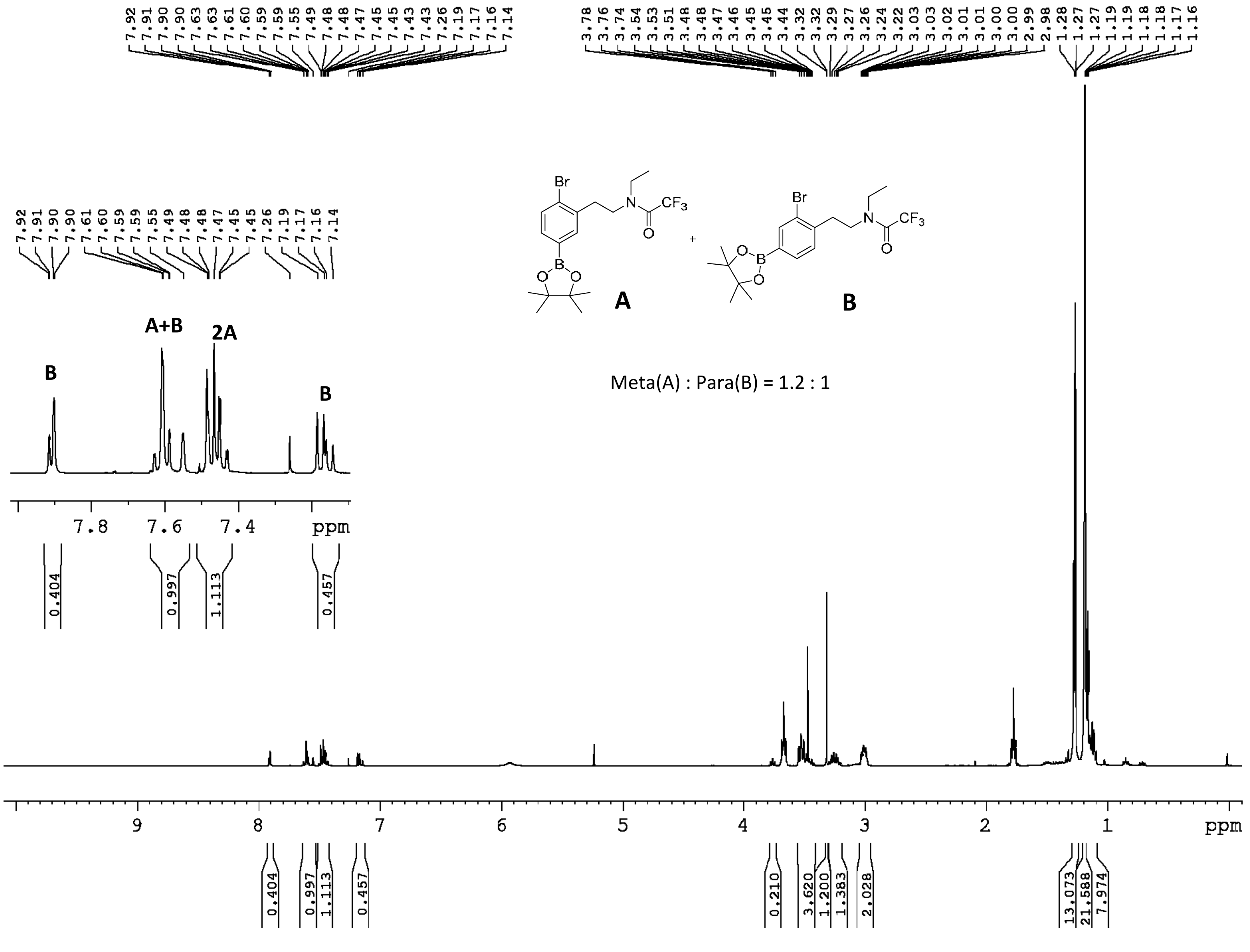

^{1}H NMR (500 MHz, DMSO-d6, 120 ^{o}C) of N-(2-bromo-5-(4,4,5,5-tetramethyl-1,3,2-dioxaborolan-2-yl)phenethyl)-N-ethyl-2,2,2-trifluoroacetamide (14a) – Ligand 1a – Purified

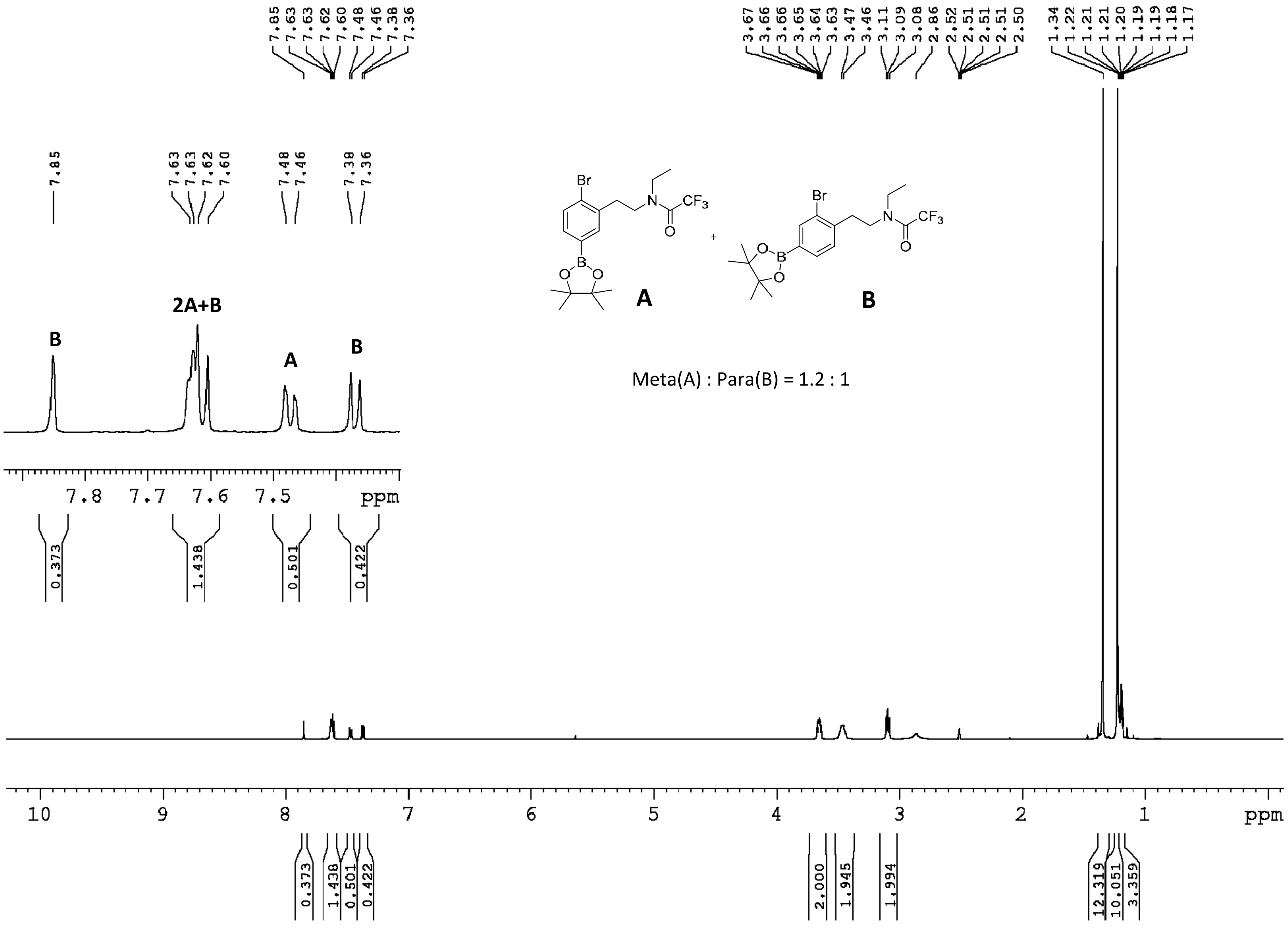

^{13}C NMR (126 MHz, DMSO-d6, 120 ^{o}C) of N-(2-bromo-5-(4,4,5,5-tetramethyl-1,3,2-dioxaborolan-2-yl)phenethyl)-N-ethyl-2,2,2-trifluoroacetamide (14a) – Ligand 1a – Purified

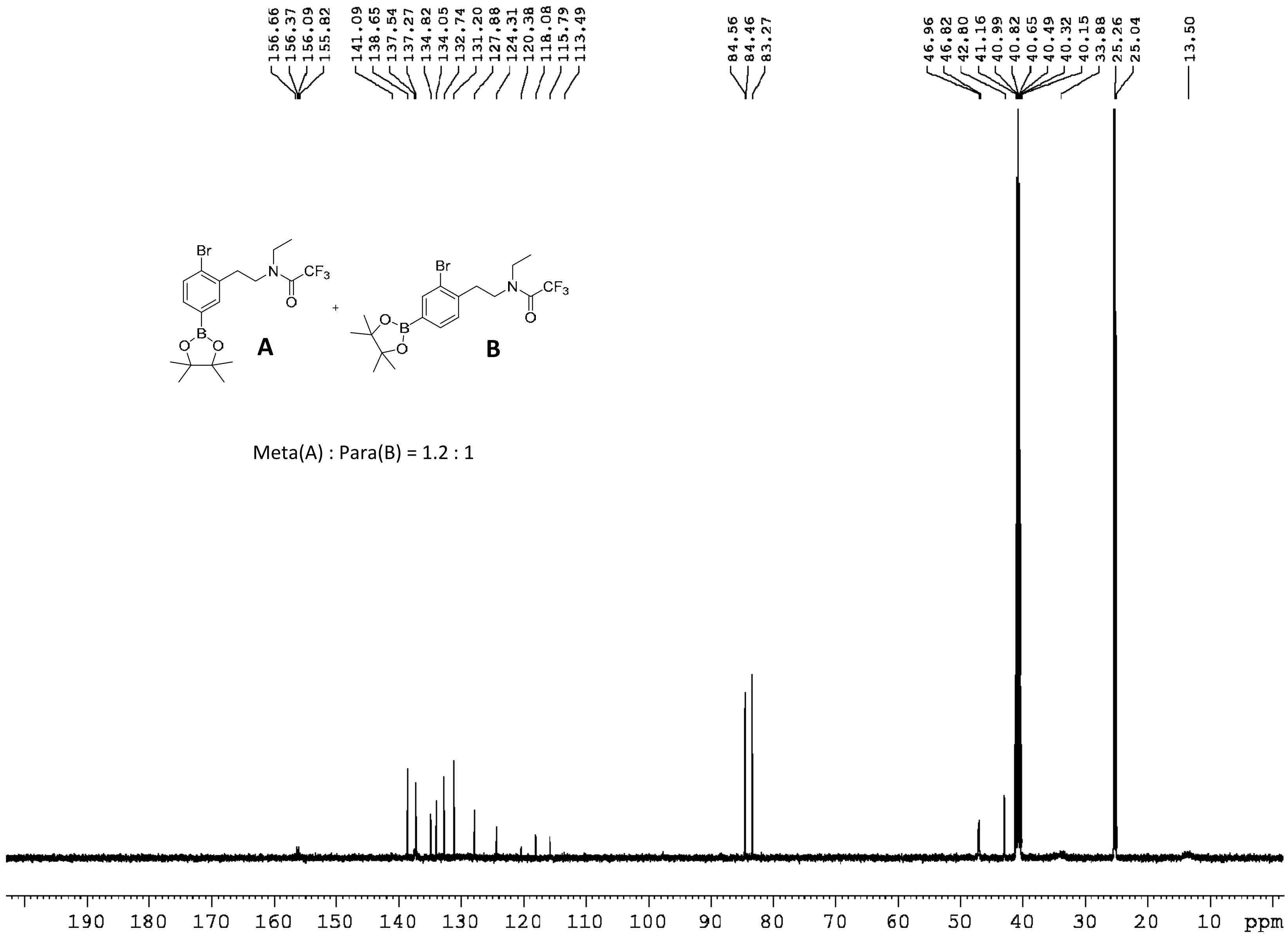

^{1}H NMR (500 MHz, DMSO-d6, 120 °C) of N-(3-(2-bromo-5-(4,4,5,5-tetramethyl-1,3,2-dioxaborolan-2-yl)phenyl)propyl)-N-ethyl-2,2,2-trifluoroacetamide (15a) – 5,5'-dimethyl-2,2'-dipyridyl – Purified

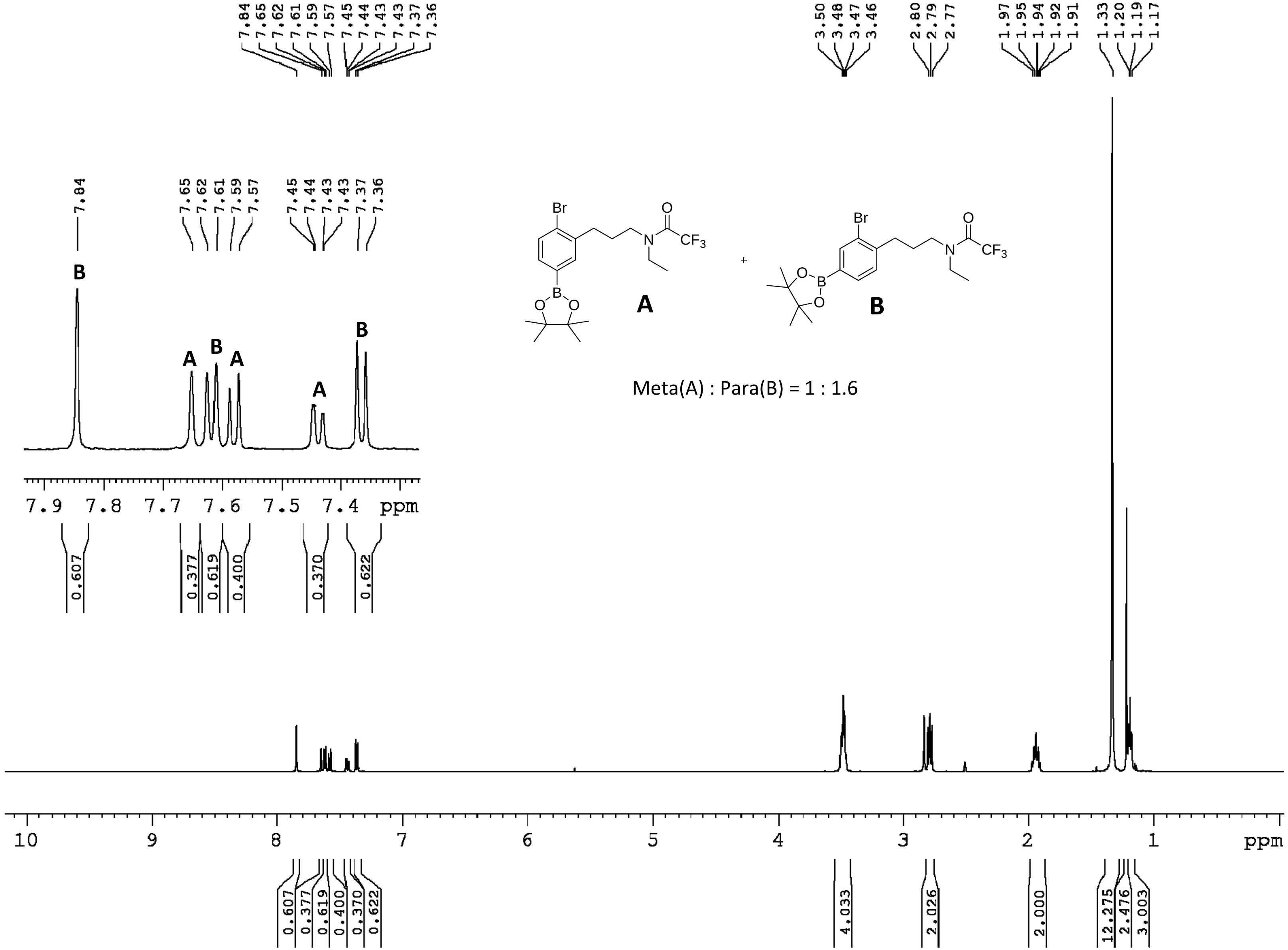

^{13}C NMR (126 MHz, DMSO-d6, 120 °C) of N-(3-(2-bromo-5-(4,4,5,5-tetramethyl-1,3,2-dioxaborolan-2-yl)phenyl)propyl)-N-ethyl-2,2,2-trifluoroacetamide (15a) – 5,5'-dimethyl-2,2'-dipyridyl – Purified

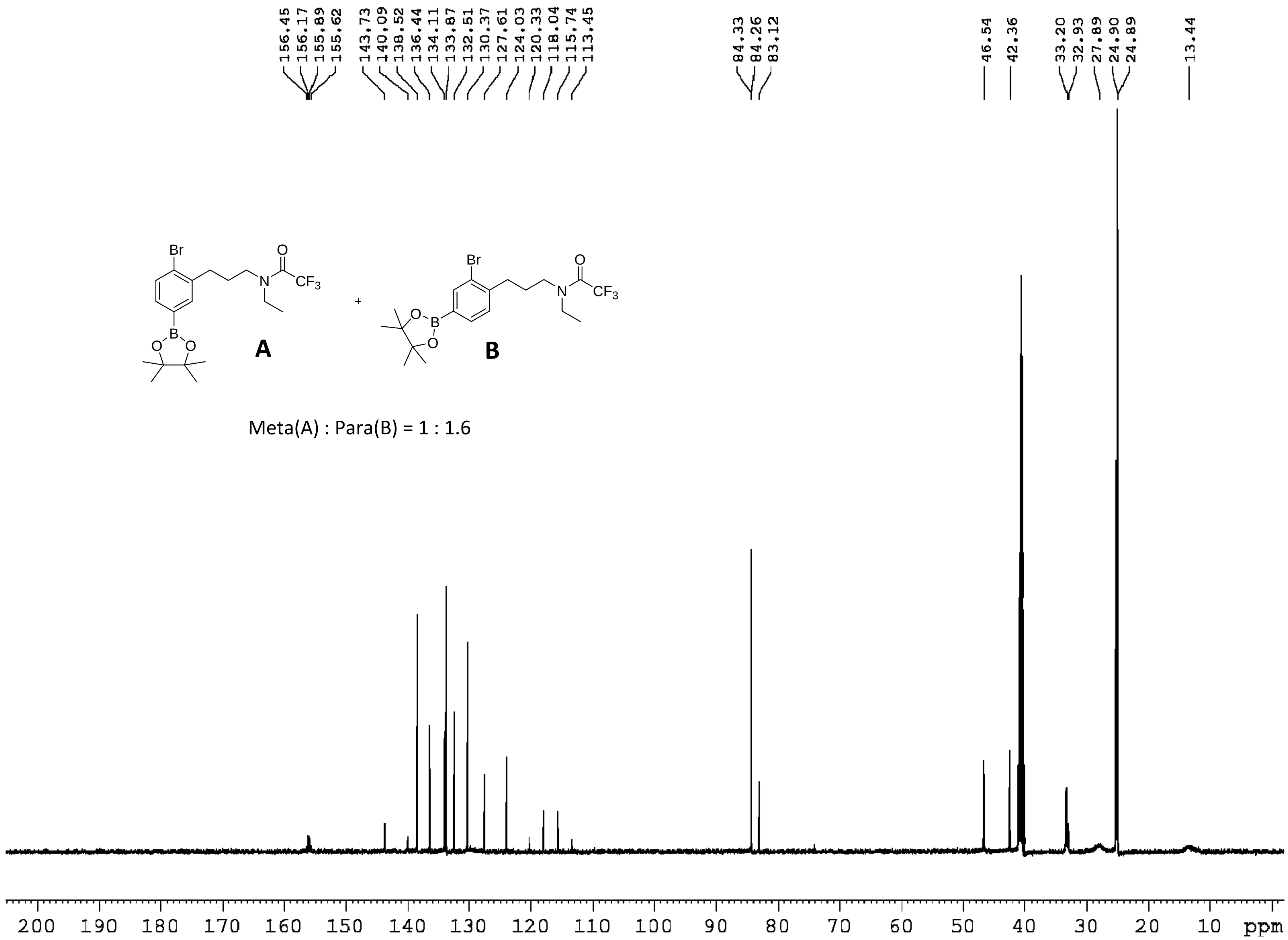

^{1}H NMR (600 MHz, $CDCl_3$) *N*-(3,5-bis(4,4,5,5-tetramethyl-1,3,2-dioxaborolan-2-yl)phenyl)acetamide – Crude mixture of borylated products from reaction with 1a assigned by comparison to separately characterised samples

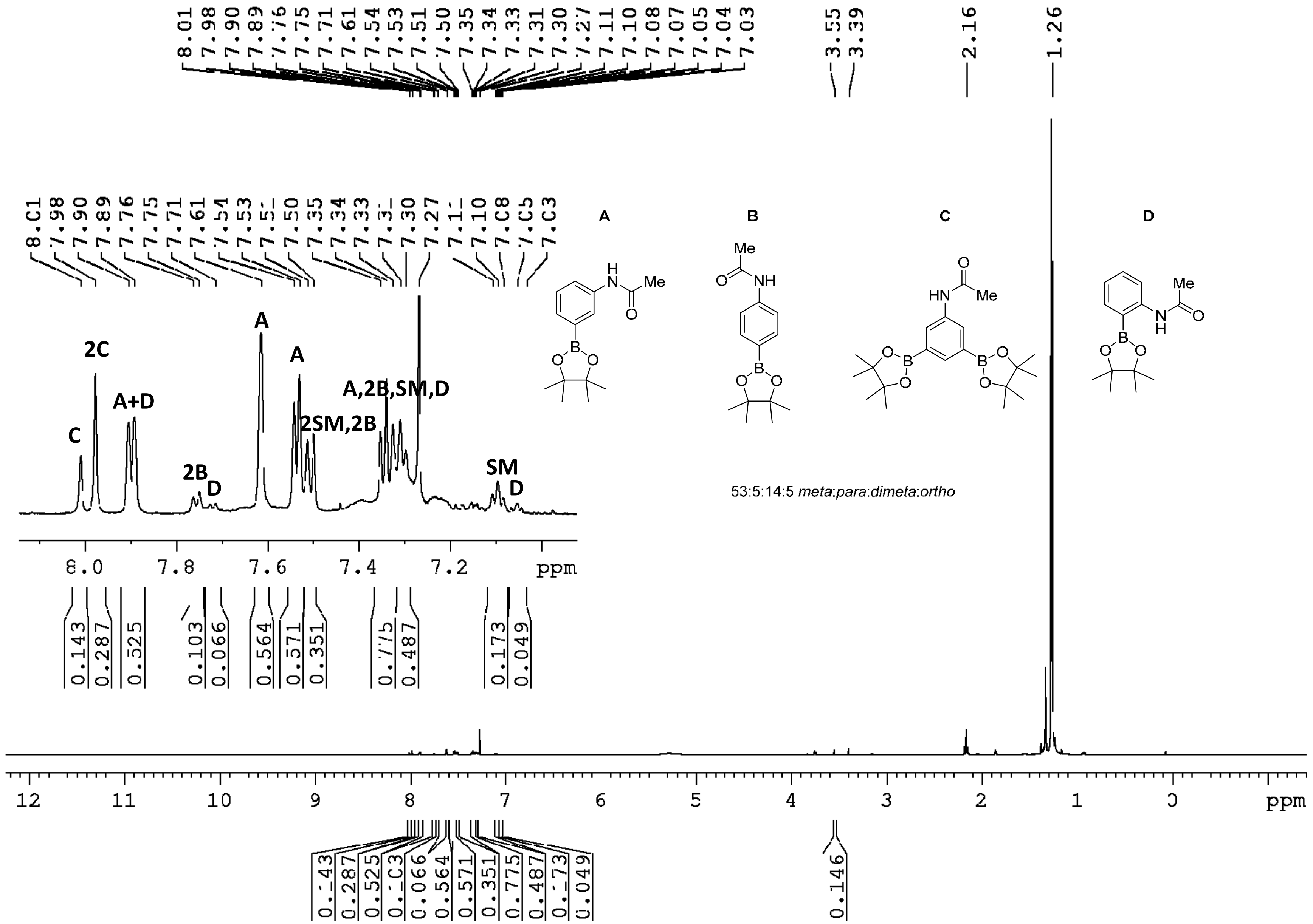

^{1}H NMR (400 MHz, $CDCl_3$) *N*-(3,5-bis(4,4,5,5-tetramethyl-1,3,2-dioxaborolan-2-yl)phenyl)acetamide – Crude mixture of borylated products from reaction with dtbpy assigned by comparison to separately characterised samples

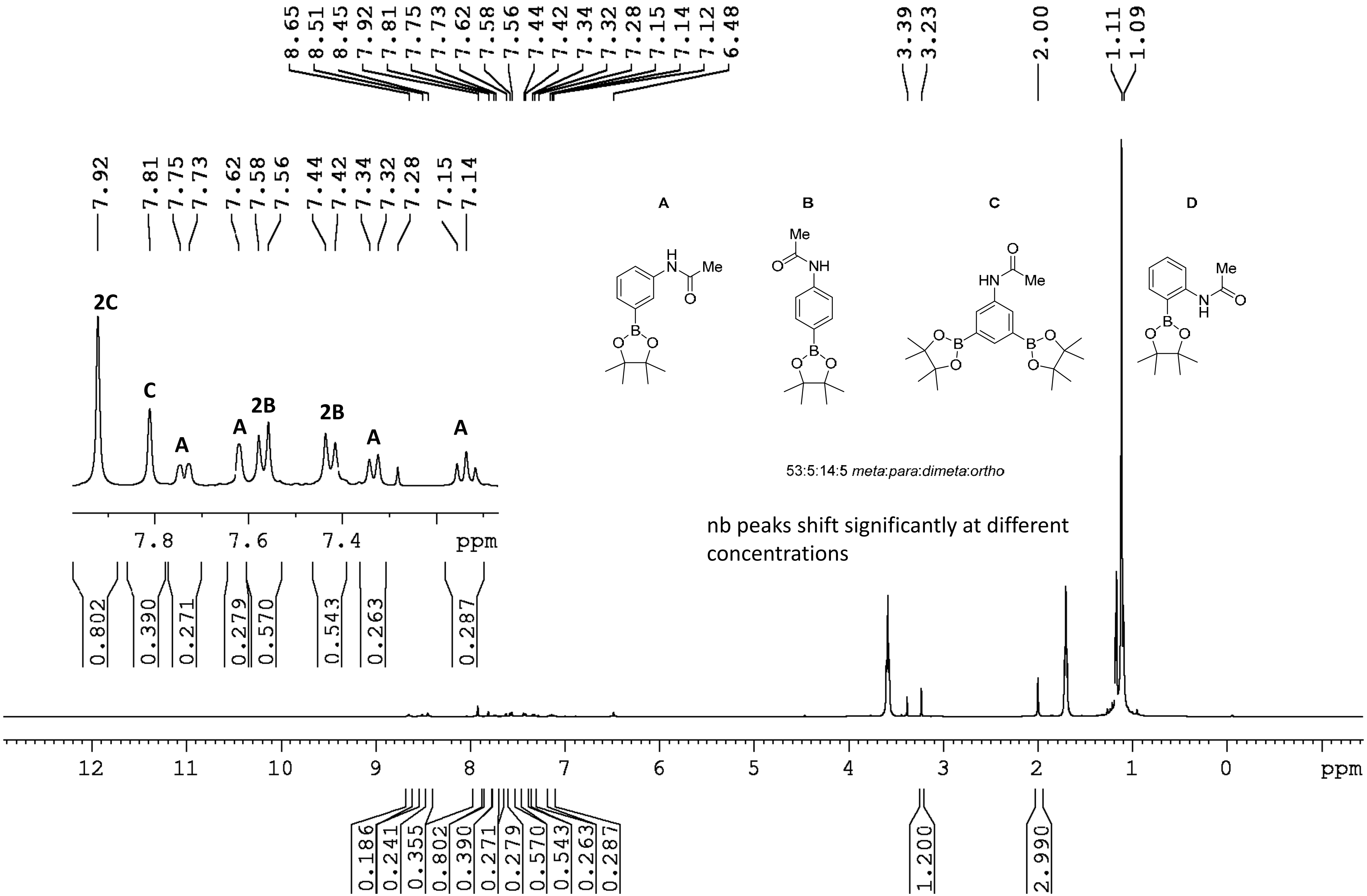

^{1}H NMR (500 MHz, $CDCl_3$) *N*-(3,5-bis(4,4,5,5-tetramethyl-1,3,2-dioxaborolan-2-yl)phenyl)acetamide – Diborylated fraction isolated from reaction of acetanilide with 1a

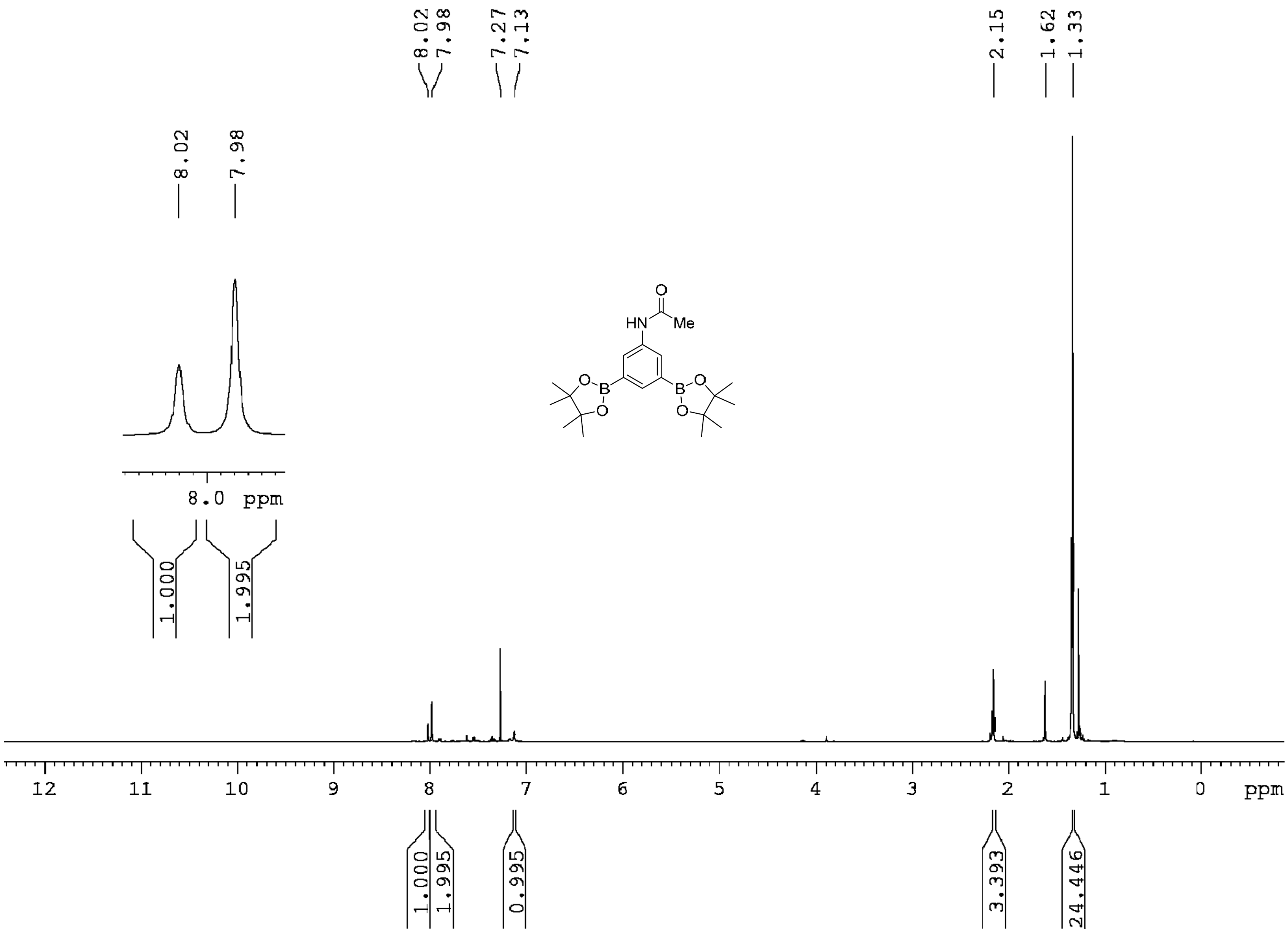

^{13}C NMR (126 MHz, CDCl$_3$) *N*-(3,5-bis(4,4,5,5-tetramethyl-1,3,2-dioxaborolan-2-yl)phenyl)acetamide – Diborylated fraction isolated from reaction of acetanilide with 1a

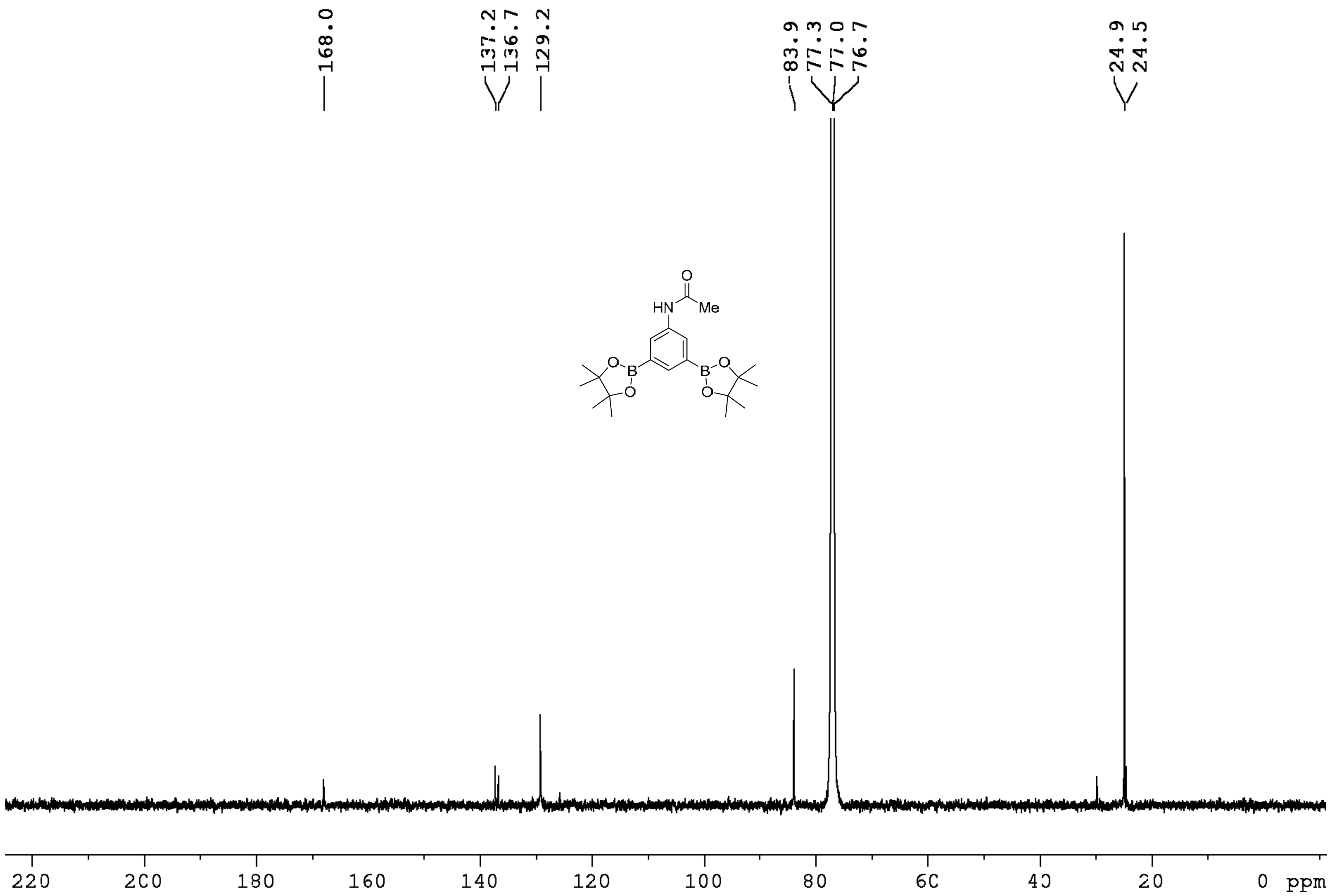

^{1}H NMR (500 MHz, $CDCl_3$) *N*-(4-(4,4,5,5-tetramethyl-1,3,2-dioxaborolan-2-yl)phenyl)acetamide

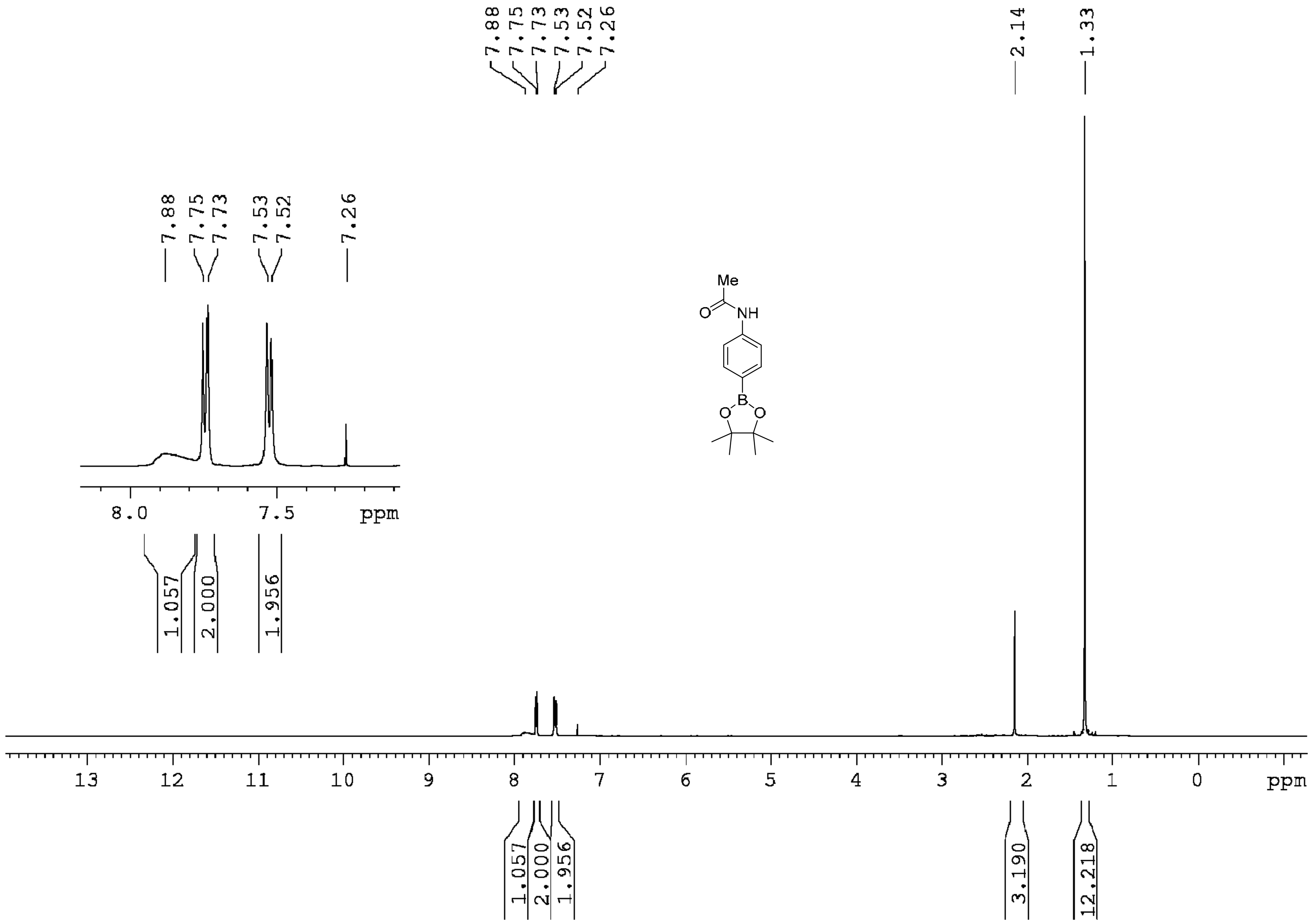

^{13}C NMR (126 MHz, CDCl$_3$) *N*-(4-(4,4,5,5-tetramethyl-1,3,2-dioxaborolan-2-yl)phenyl)acetamide

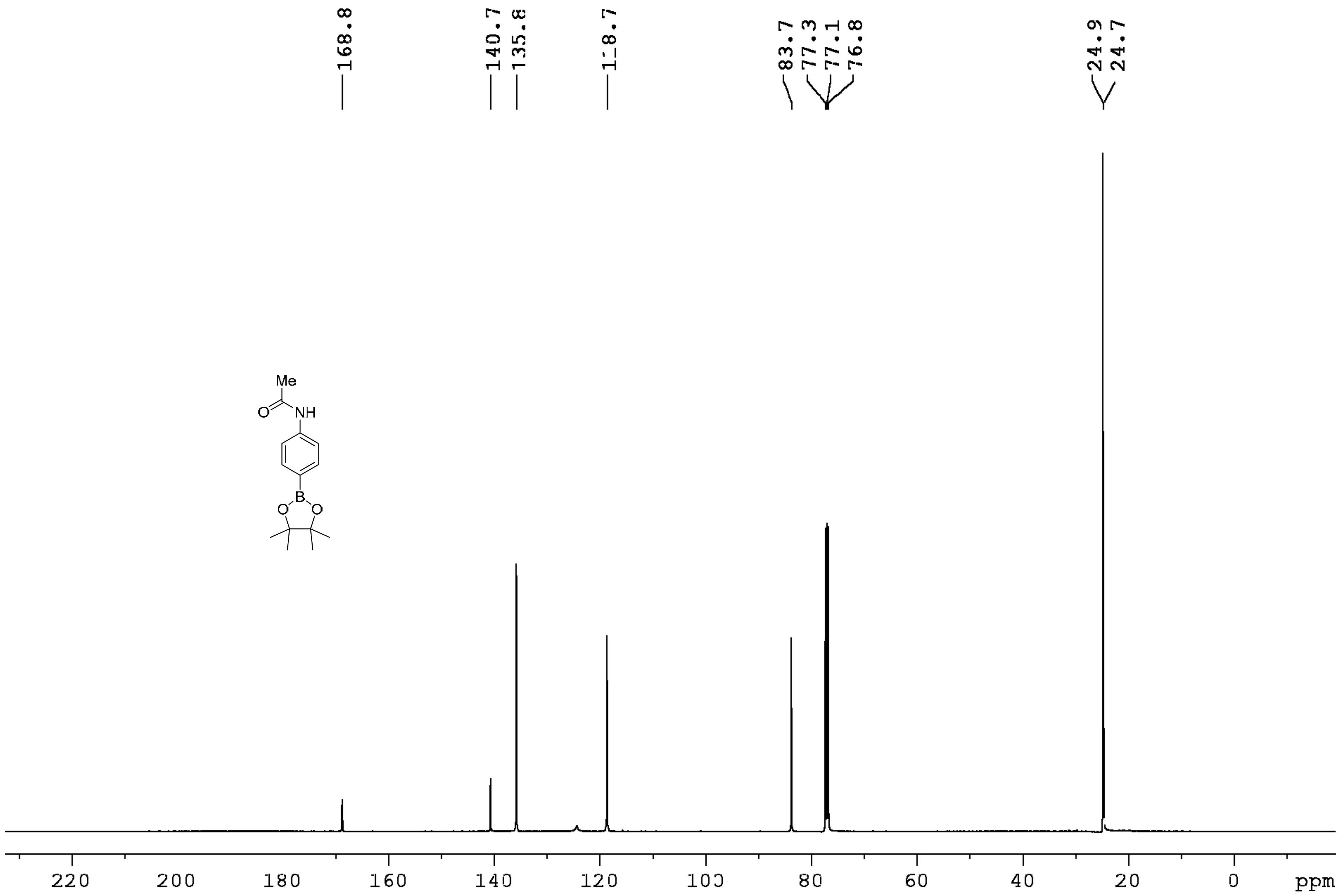

^{1}H NMR (500 MHz, $CDCl_3$) *N*-(3-(4,4,5,5-tetramethyl-1,3,2-dioxaborolan-2-yl)phenyl)acetamide

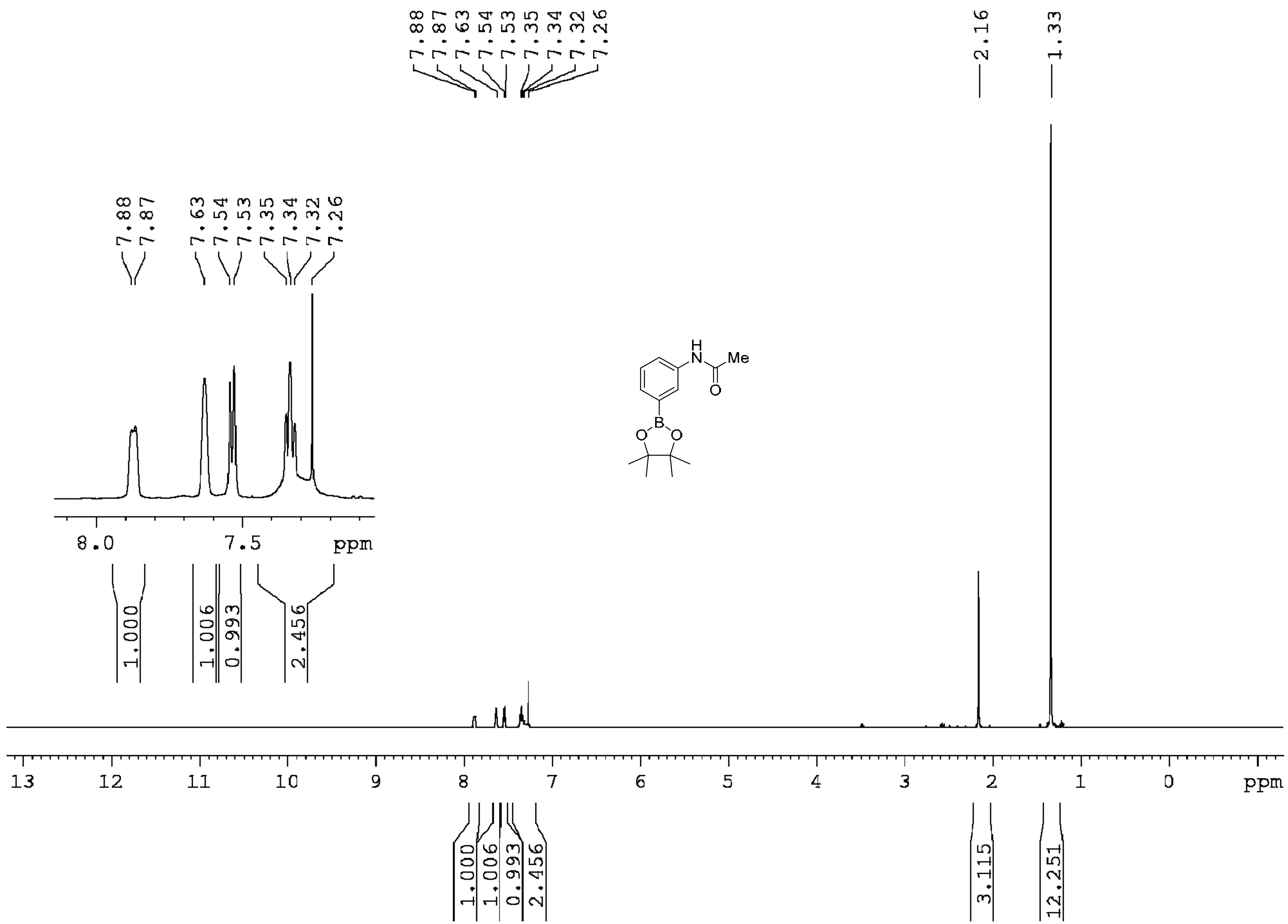

^{13}C NMR (126 MHz, $CDCl_3$) *N*-(3-(4,4,5,5-tetramethyl-1,3,2-dioxaborolan-2-yl)phenyl)acetamide

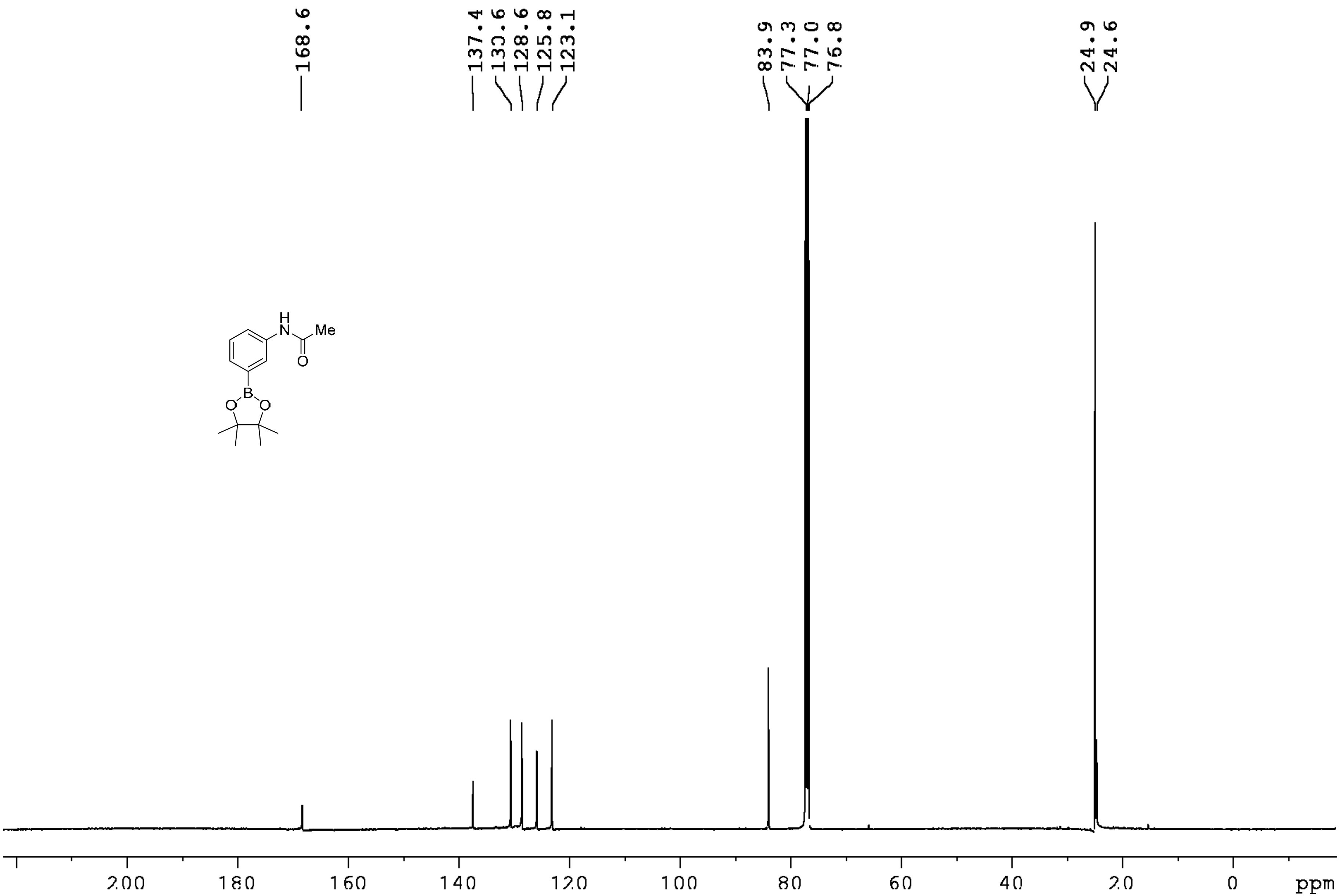

^{1}H NMR (500 MHz, $CDCl_3$) *N*-(2-(4,4,5,5-tetramethyl-1,3,2-dioxaborolan-2-yl)phenyl)acetamide

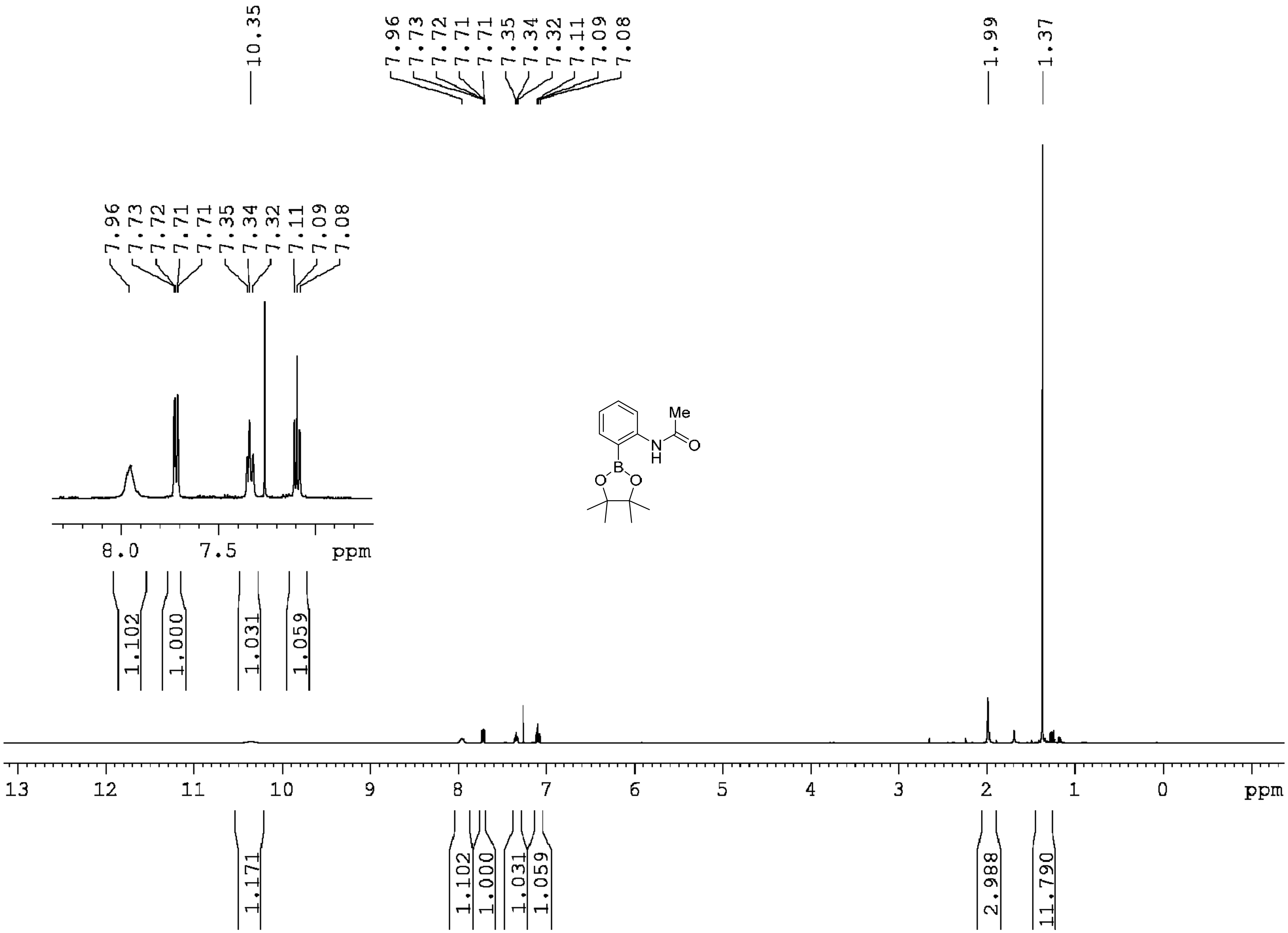

^{13}C NMR (126 MHz, CDCl$_3$) *N*-(3-(4,4,5,5-tetramethyl-1,3,2-dioxaborolan-2-yl)phenyl)acetamide

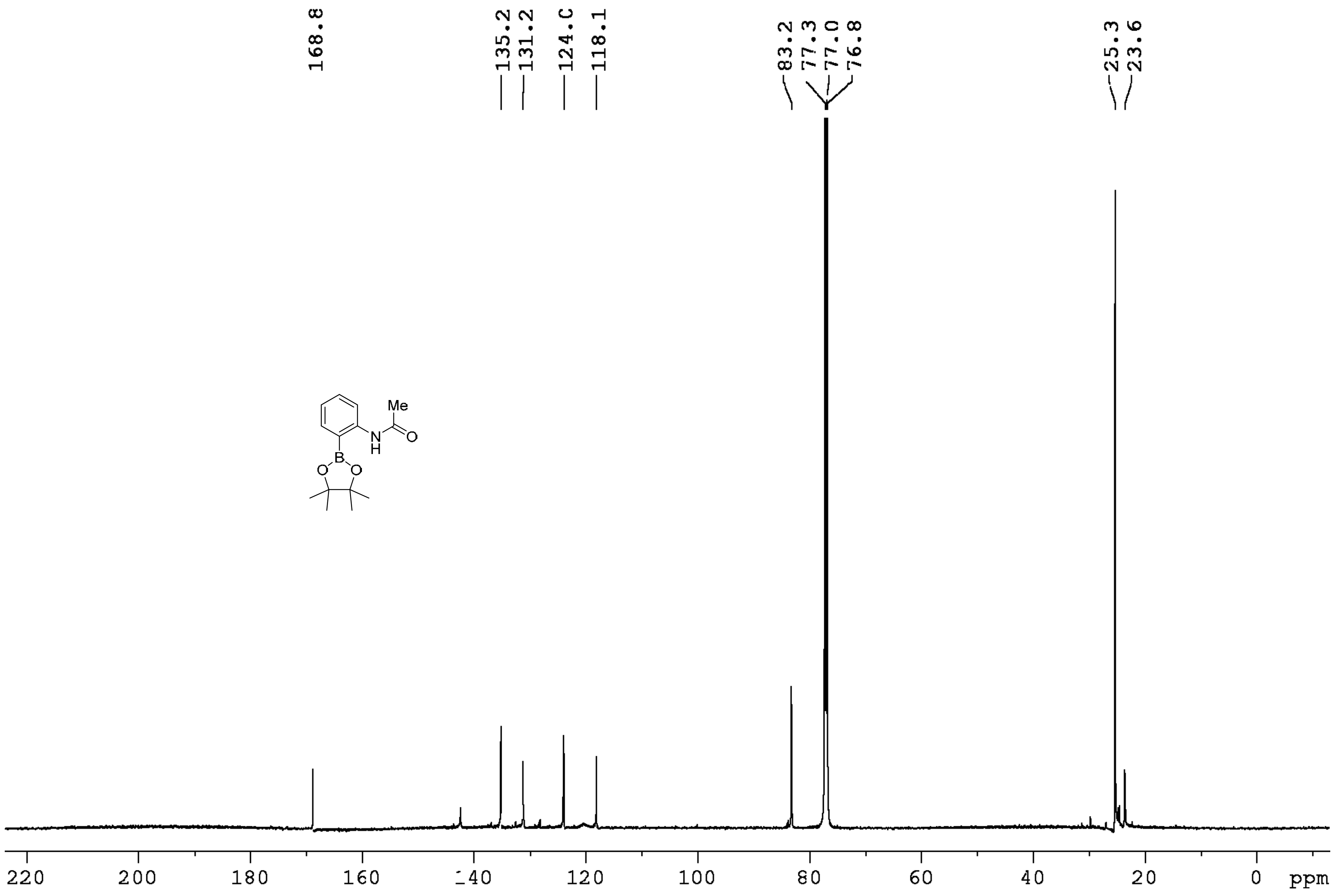

Ligand NMRs

^{1}H NMR (600 MHz, $CDCl_3$) Tetrabutylammonium 2-(5'-methyl-[2,2'-bipyridin]-5-yl)ethane-1-sulfonate (1b)

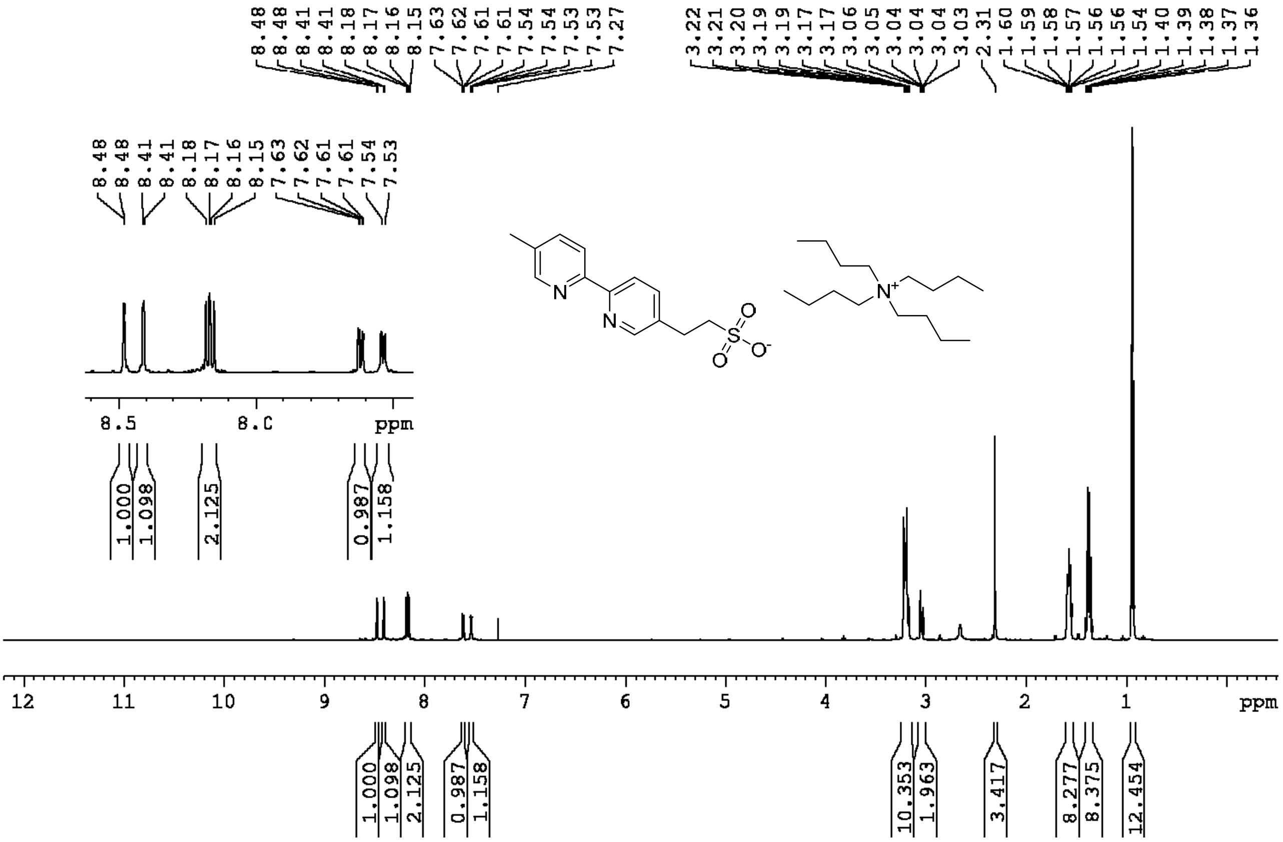

^{13}C NMR (151 MHz, $CDCl_3$) Tetrabutylammonium 2-(5'-methyl-[2,2'-bipyridin]-5-yl)ethane-1-sulfonate (1b)

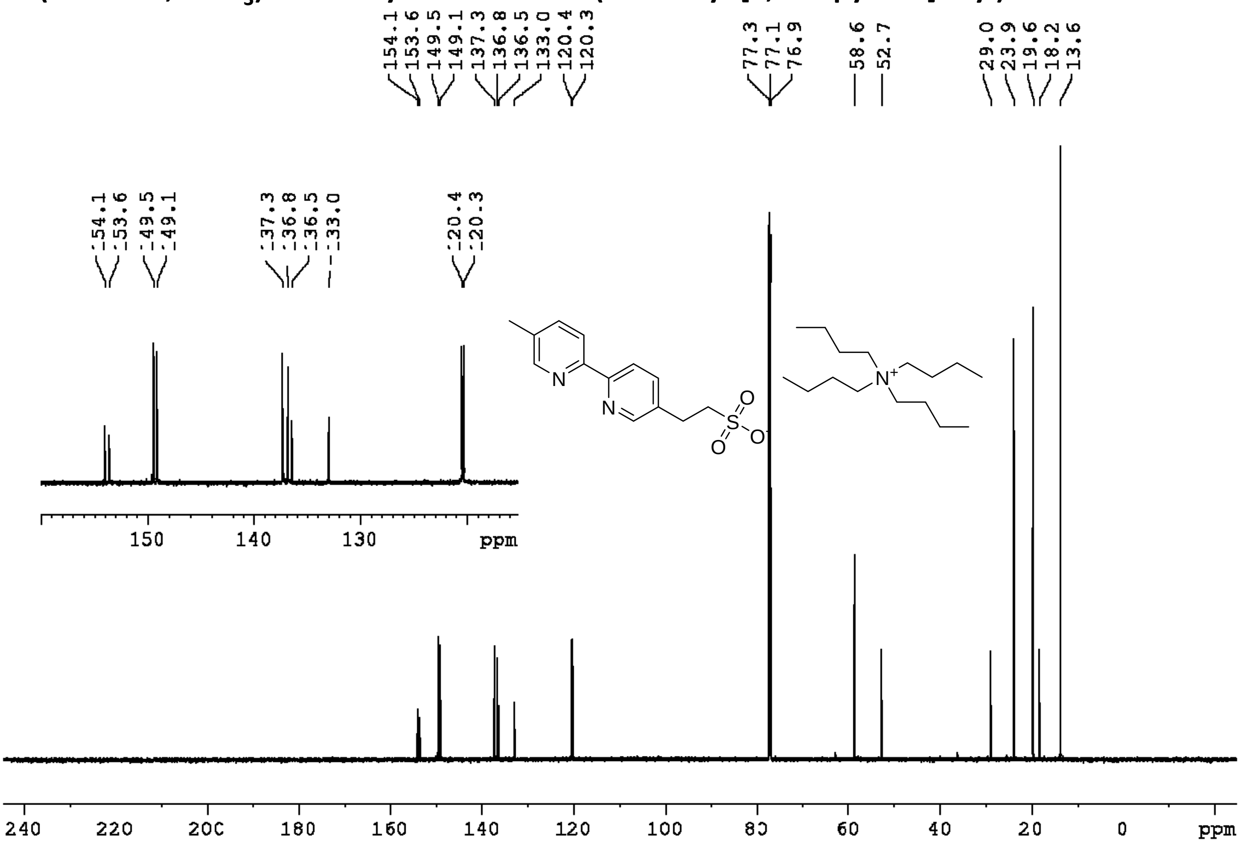

^{1}H NMR (600 MHz, $CDCl_3$) ((5'-methyl-[2,2'-bipyridin]-5-yl)methyl)diphenylphosphine oxide (1c)

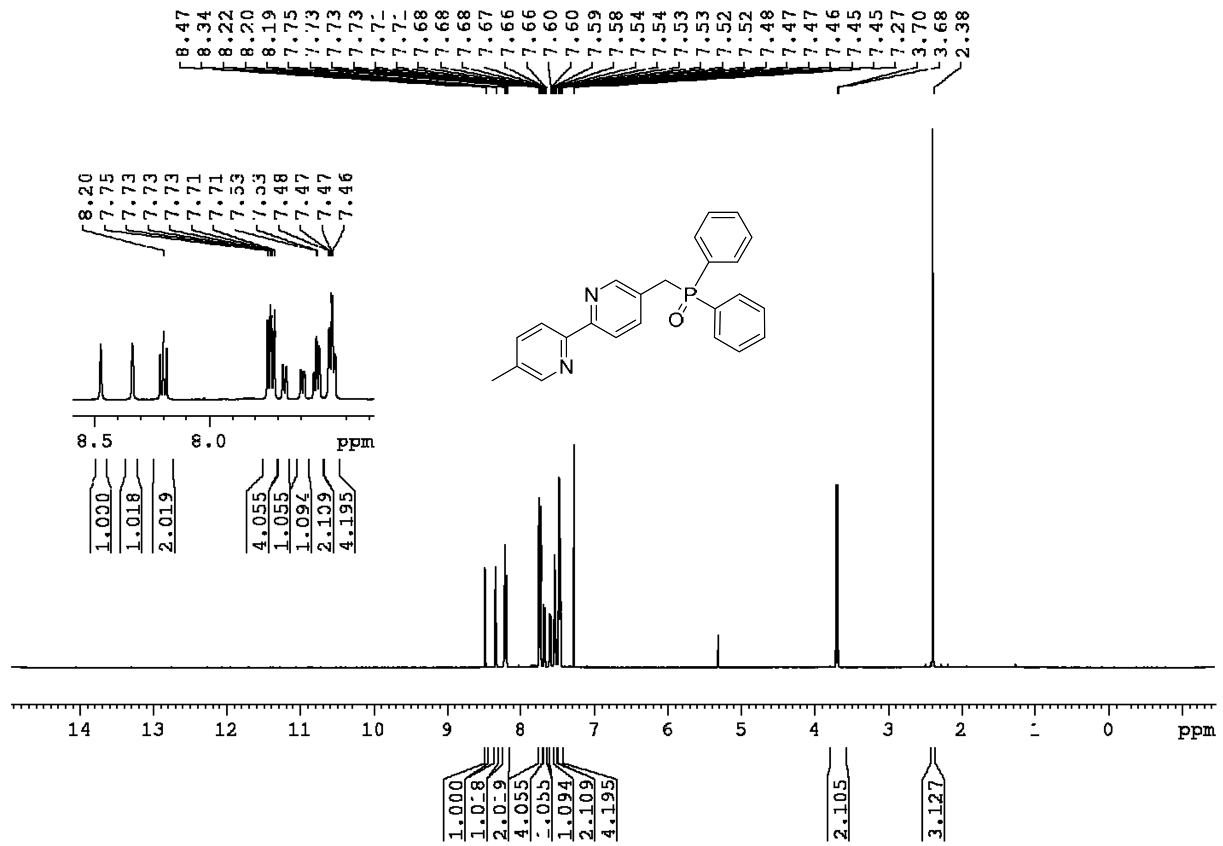

^{13}C NMR (151 MHz, $CDCl_3$) ((5'-methyl-[2,2'-bipyridin]-5-yl)methyl)diphenylphosphine oxide (1c)

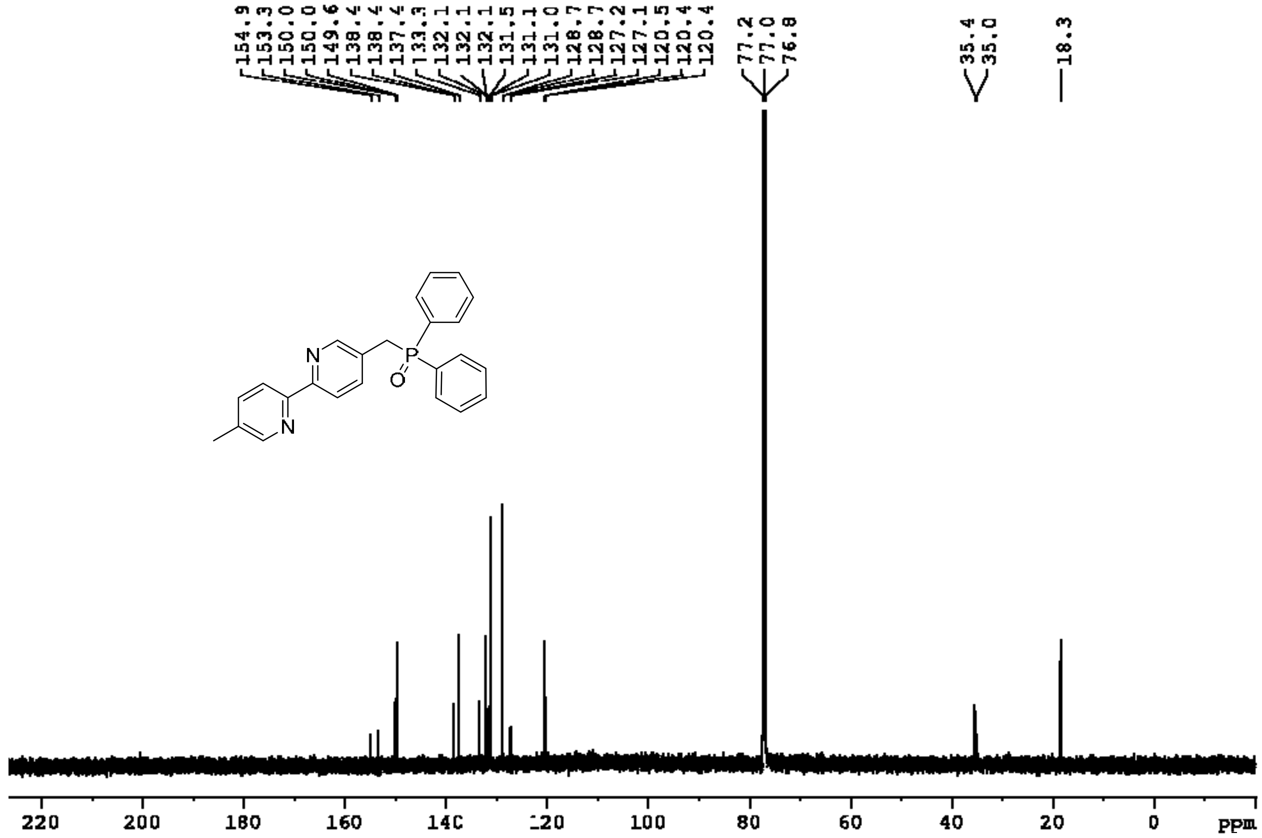

^{1}H NMR (600 MHz, CDCl$_3$) (2-(5'-methyl-[2,2'-bipyridin]-5-yl)ethyl)diphenylphosphine oxide (1d)

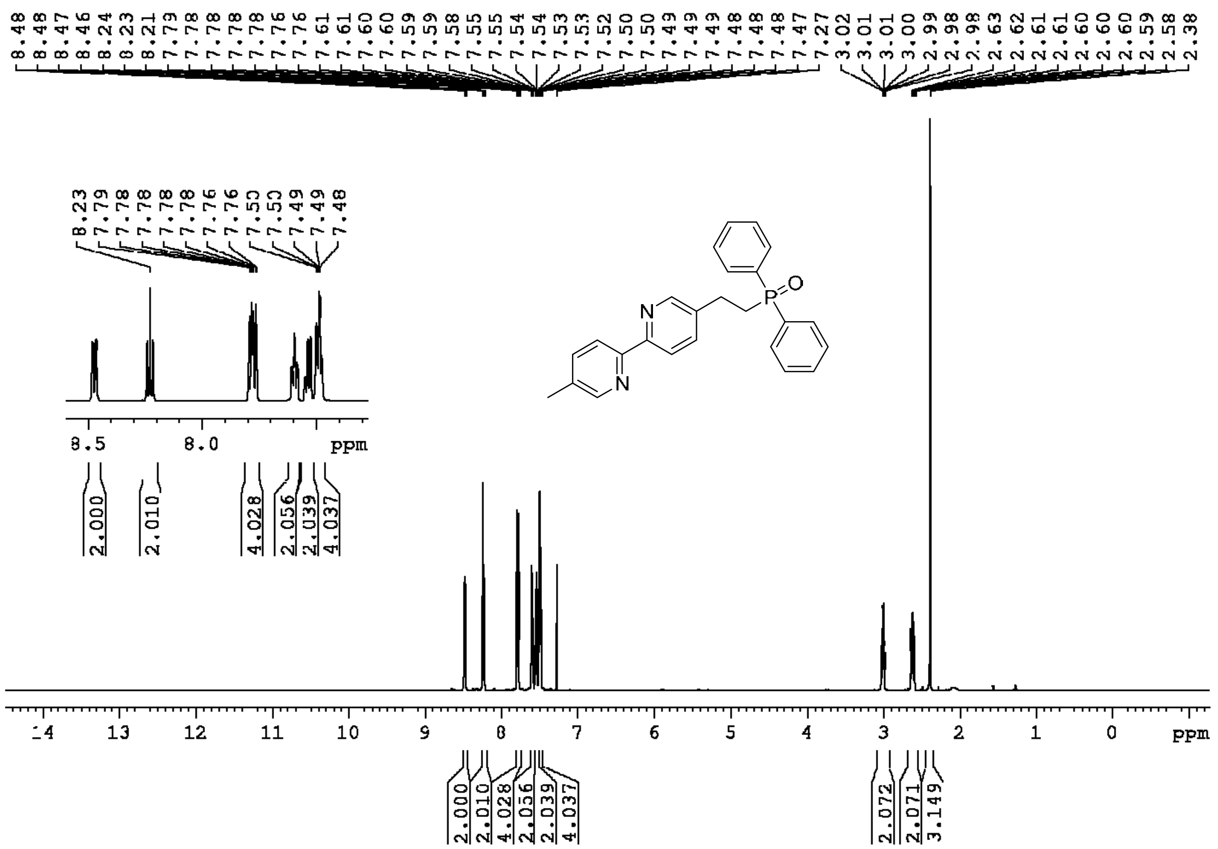

^{13}C NMR (151 MHz, CDCl$_3$) (2-(5'-methyl-[2,2'-bipyridin]-5-yl)ethyl)diphenylphosphine oxide (1d)

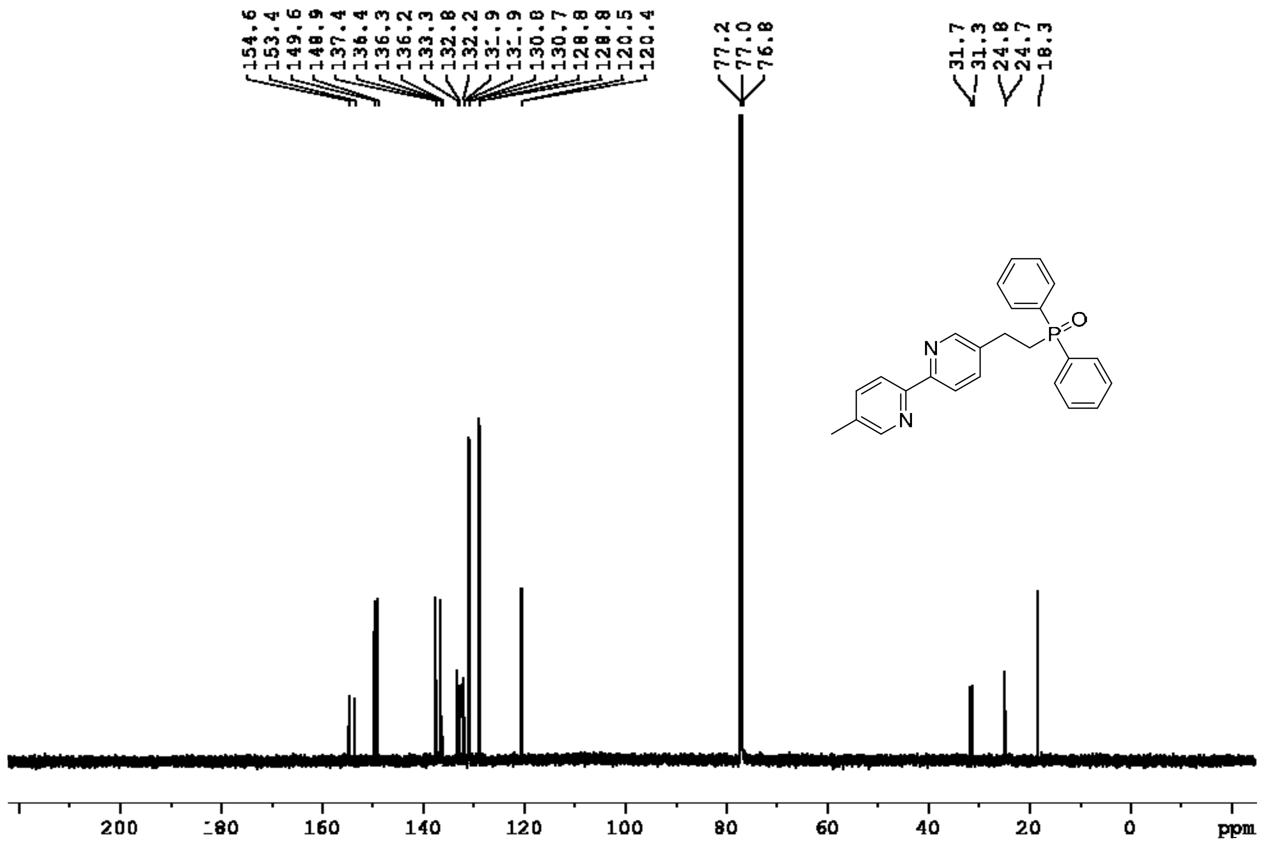

^{1}H NMR (500 MHz, $CDCl_3$) 4-Methyl-5'-((phenylsulfinyl)methyl)-2,2'-bipyridine (1e)

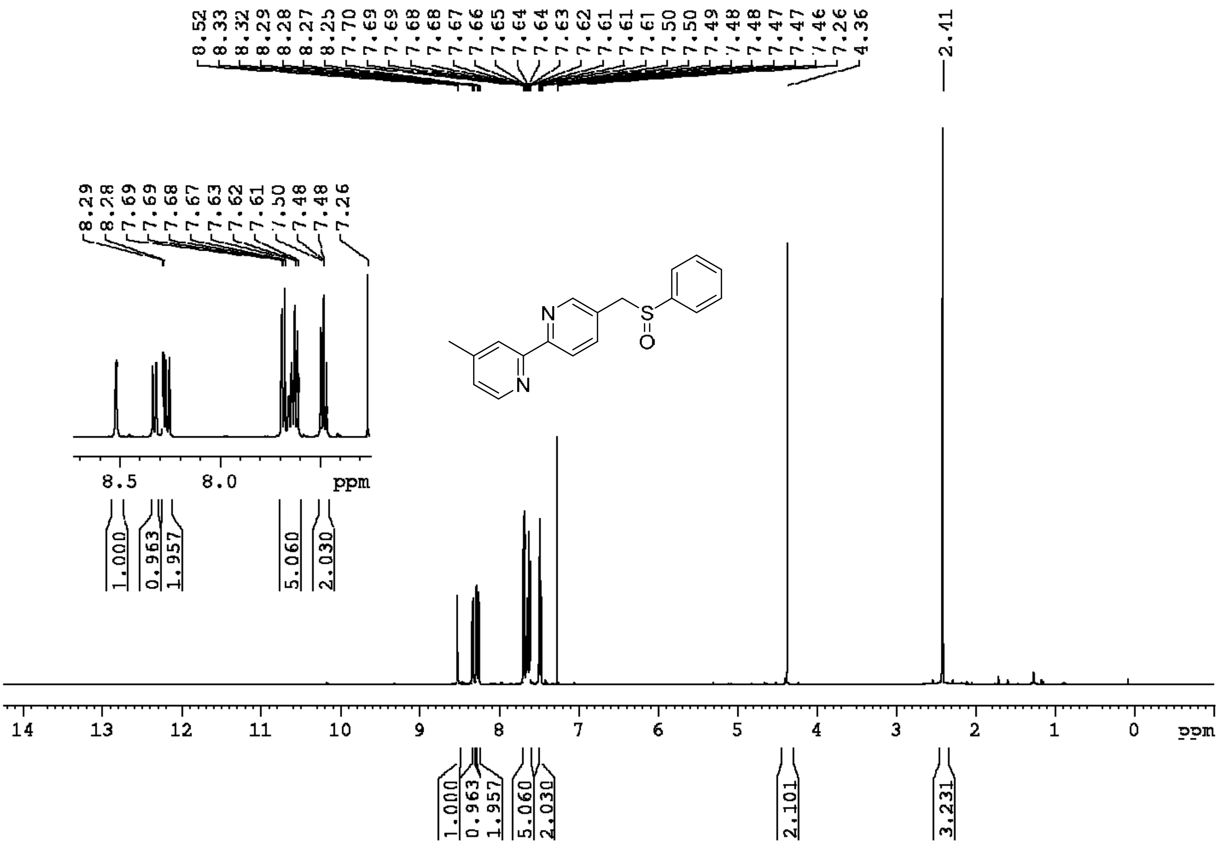

^{13}C NMR (126 MHz, $CDCl_3$) 4-Methyl-5'-((phenylsulfinyl)methyl)-2,2'-bipyridine (1e)

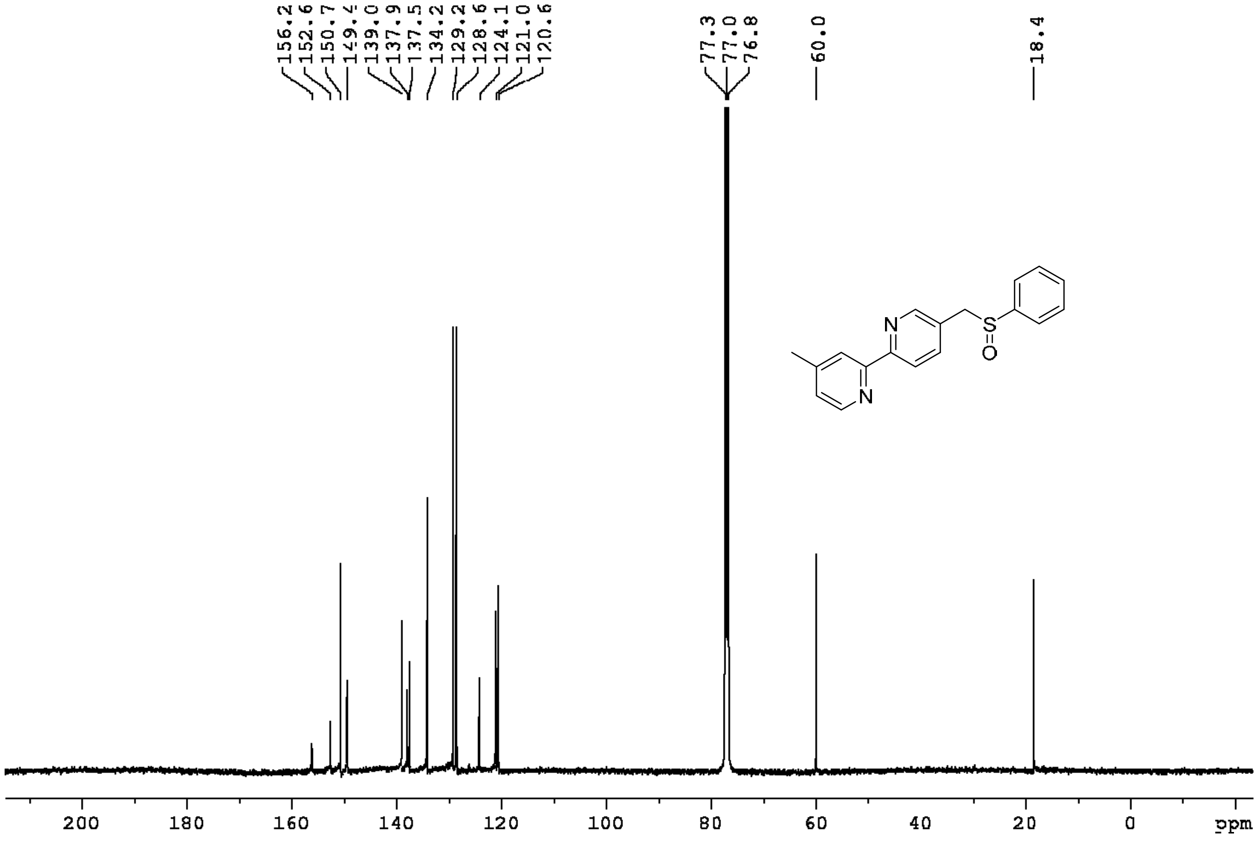

^{1}H NMR (600 MHz, $CDCl_3$) 5-methyl-5'-(2-(phenylsulfinyl)ethyl)-2,2'-bipyridine (1f)

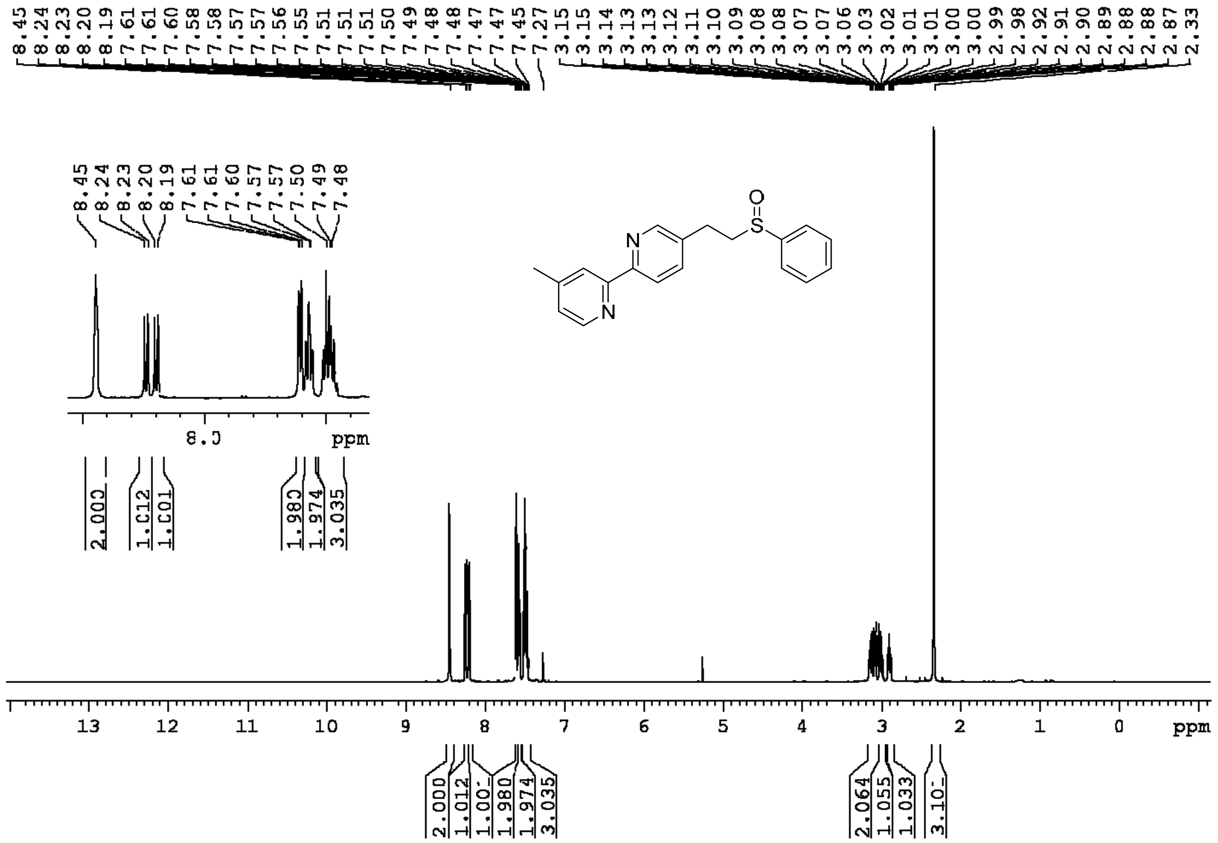

^{13}C NMR (151 MHz, $CDCl_3$) 5-methyl-5'-(2-(phenylsulfinyl)ethyl)-2,2'-bipyridine (1f)

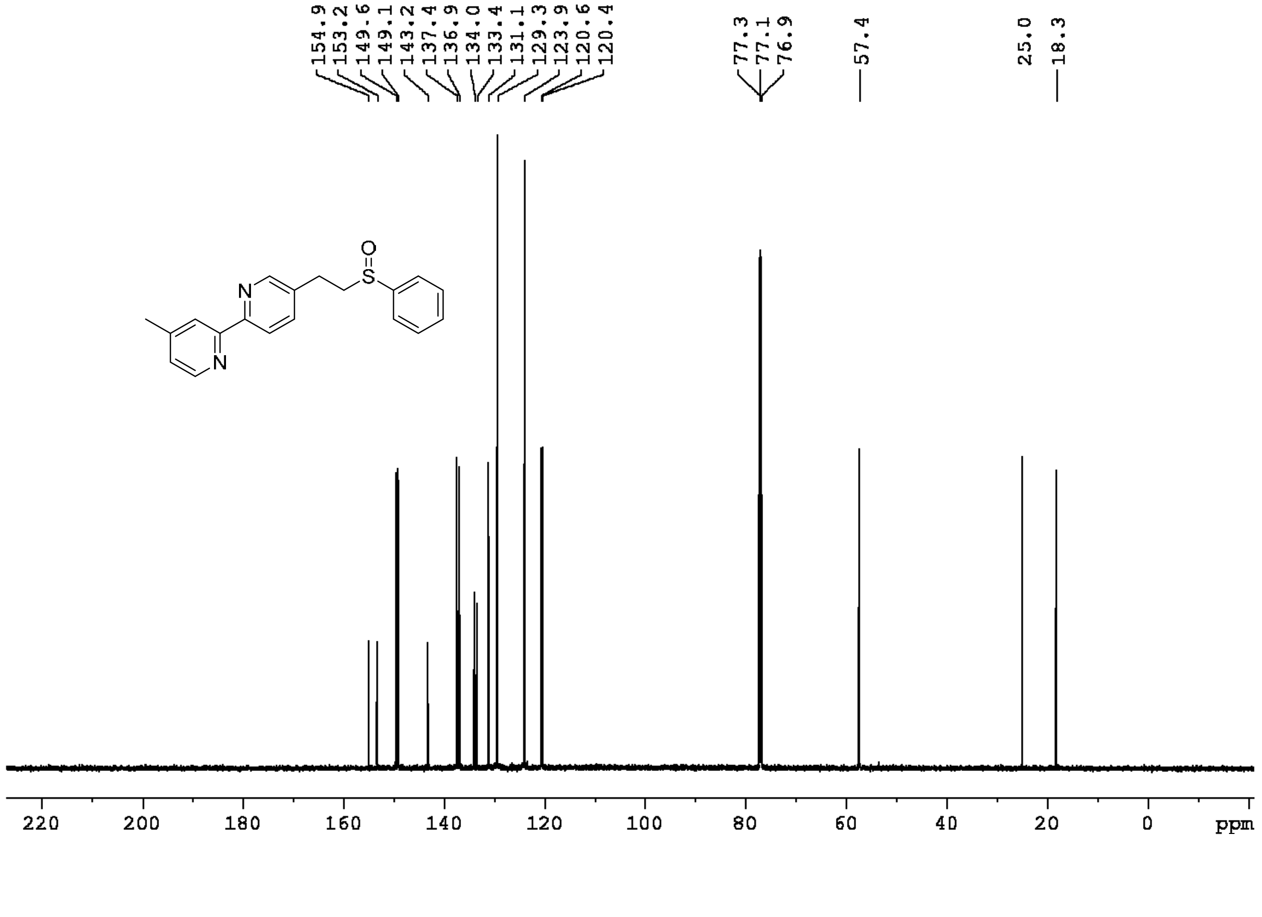

^{1}H NMR (600 MHz, DMSO-d_6) 2-(5'-Methyl-[2,2'-bipyridin]-5-yl)acetic acid

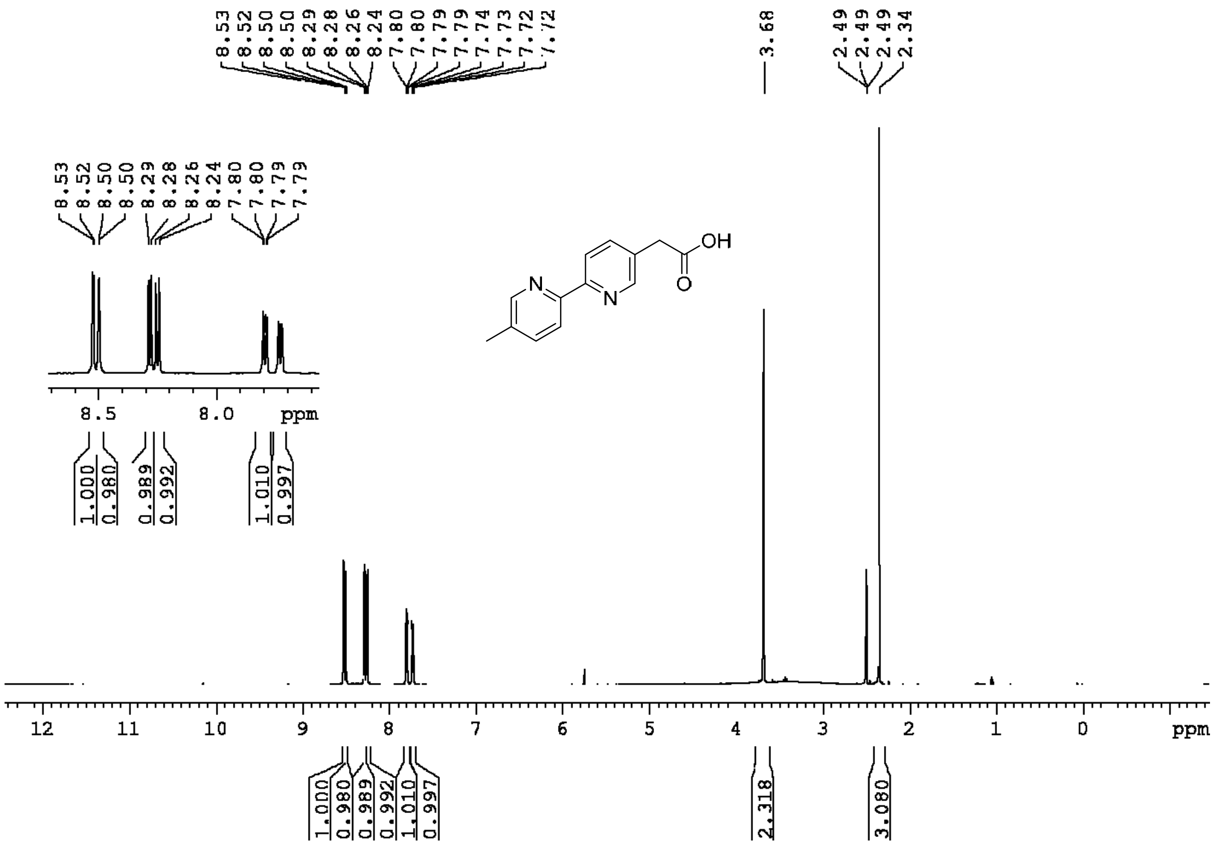

^{13}C NMR (151 MHz, DMSO-d_6) 2-(5'-Methyl-[2,2'-bipyridin]-5-yl)acetic acid

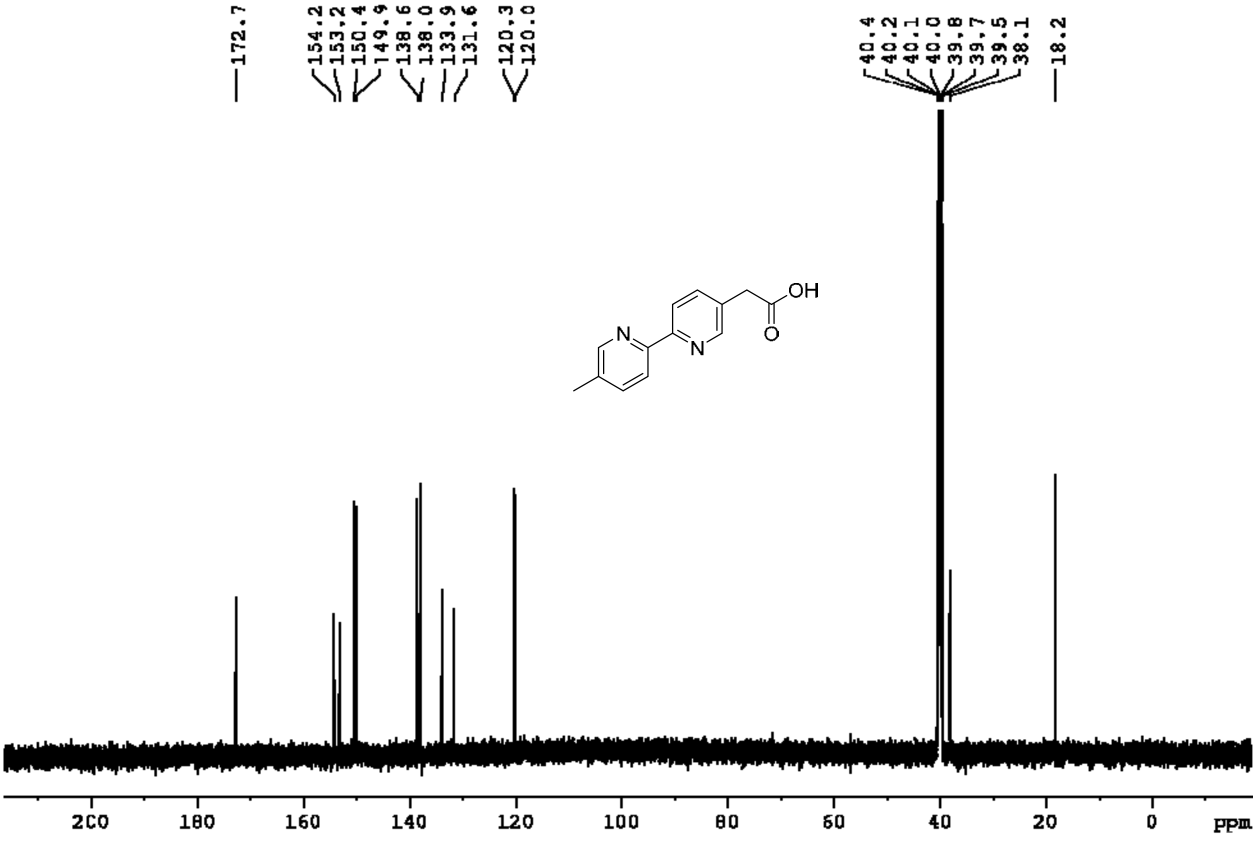

^{1}H NMR (600 MHz, CDCl$_3$) *N,N*-diethyl-2-(5'-methyl-[2,2'-bipyridin]-5-yl)acetamide (1g)

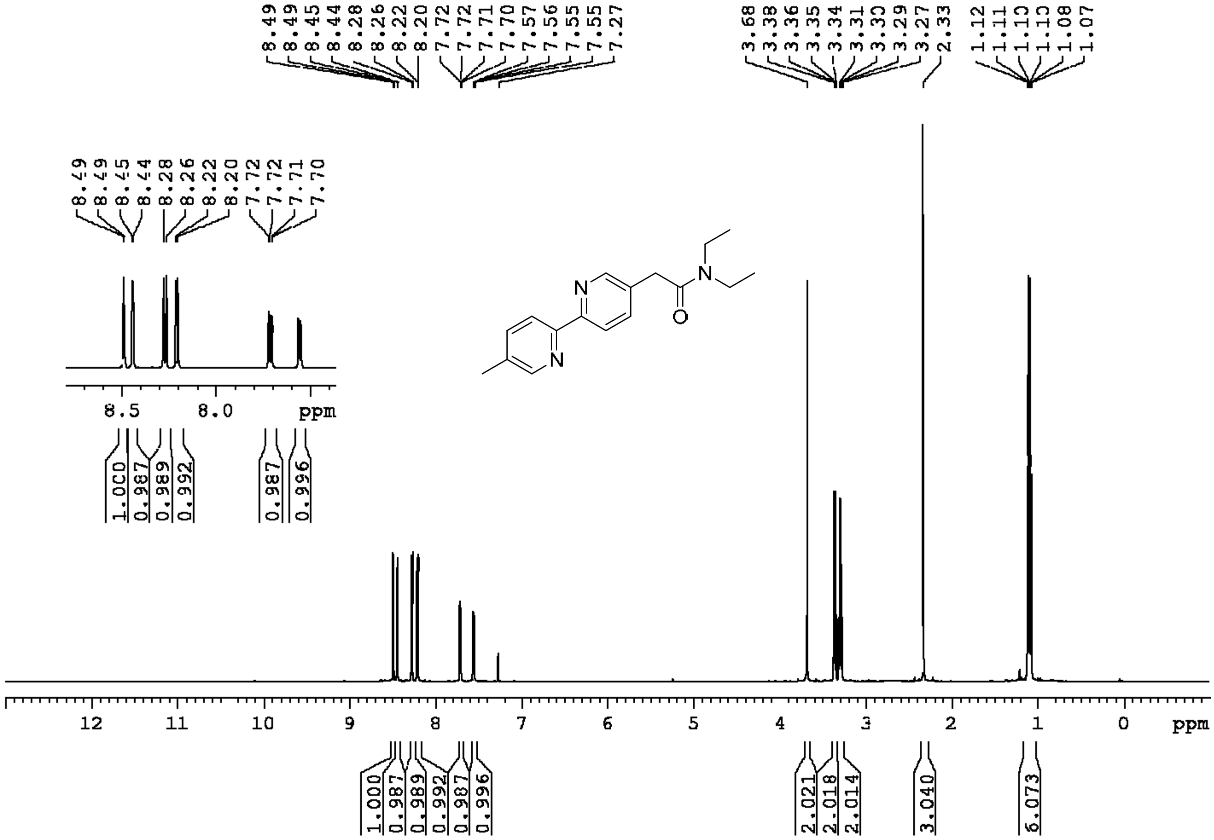

^{13}C NMR (151 MHz, CDCl$_3$) *N,N*-diethyl-2-(5'-methyl-[2,2'-bipyridin]-5-yl)acetamide (1g)

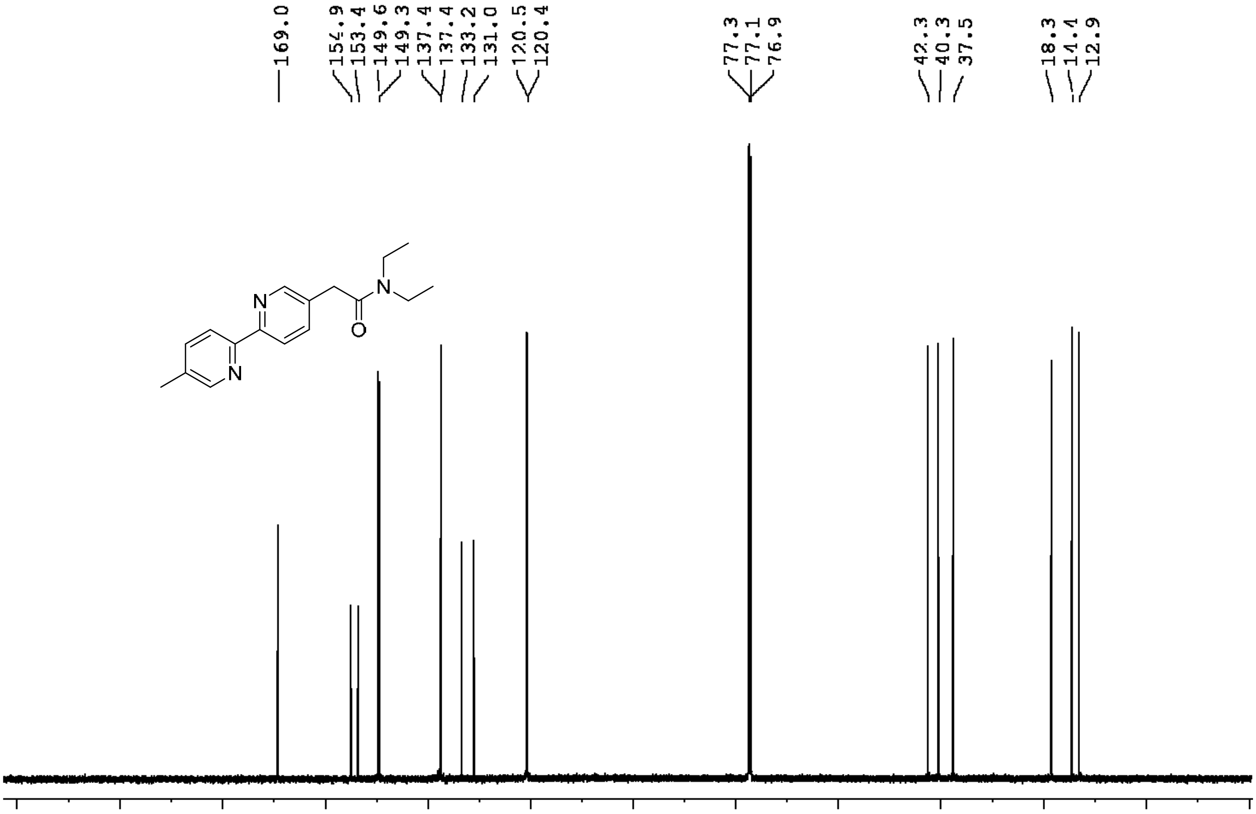

^{1}H NMR (600 MHz, $CDCl_3$) *N,N*-diethyl-3-(4'-methyl-[2,2'-bipyridin]-4-yl)propanamide (1h)

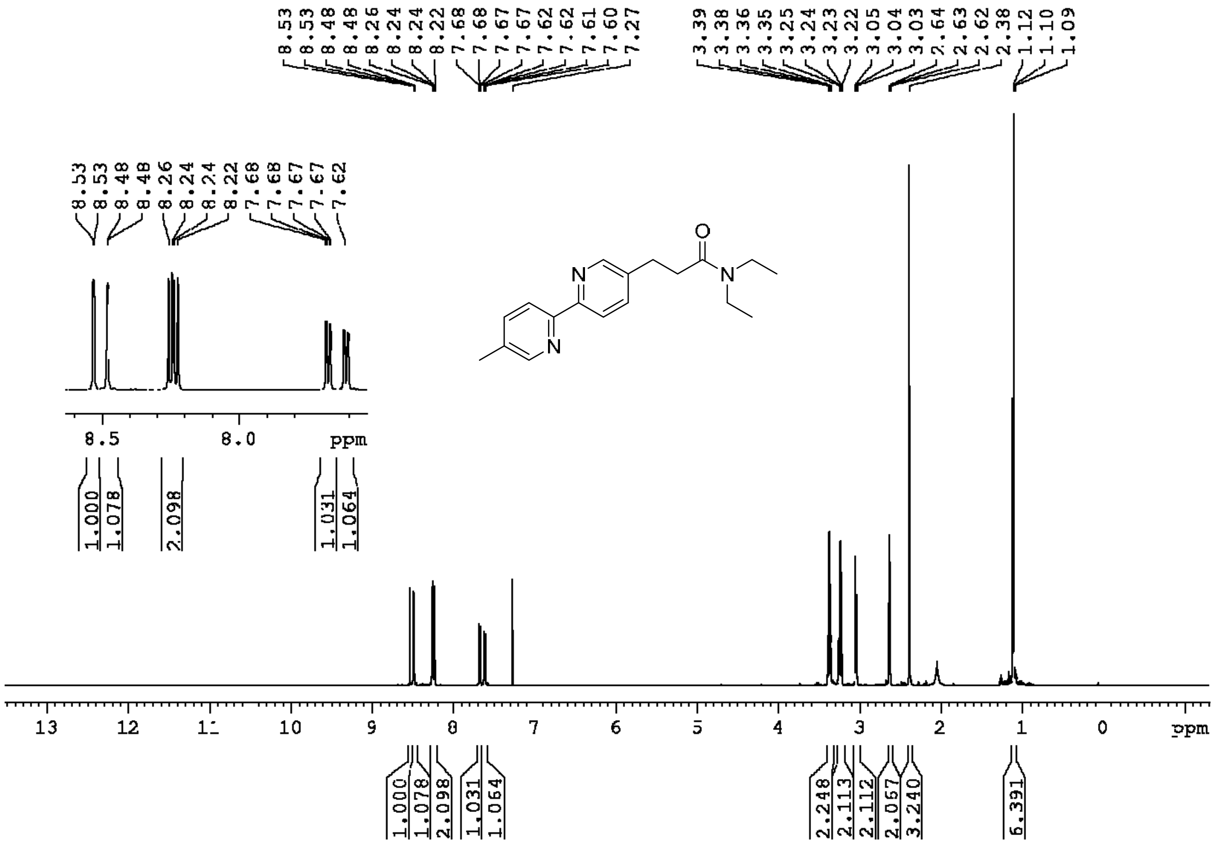

^{13}C NMR (151 MHz, $CDCl_3$) *N,N*-diethyl-3-(4'-methyl-[2,2'-bipyridin]-4-yl)propanamide (1h)

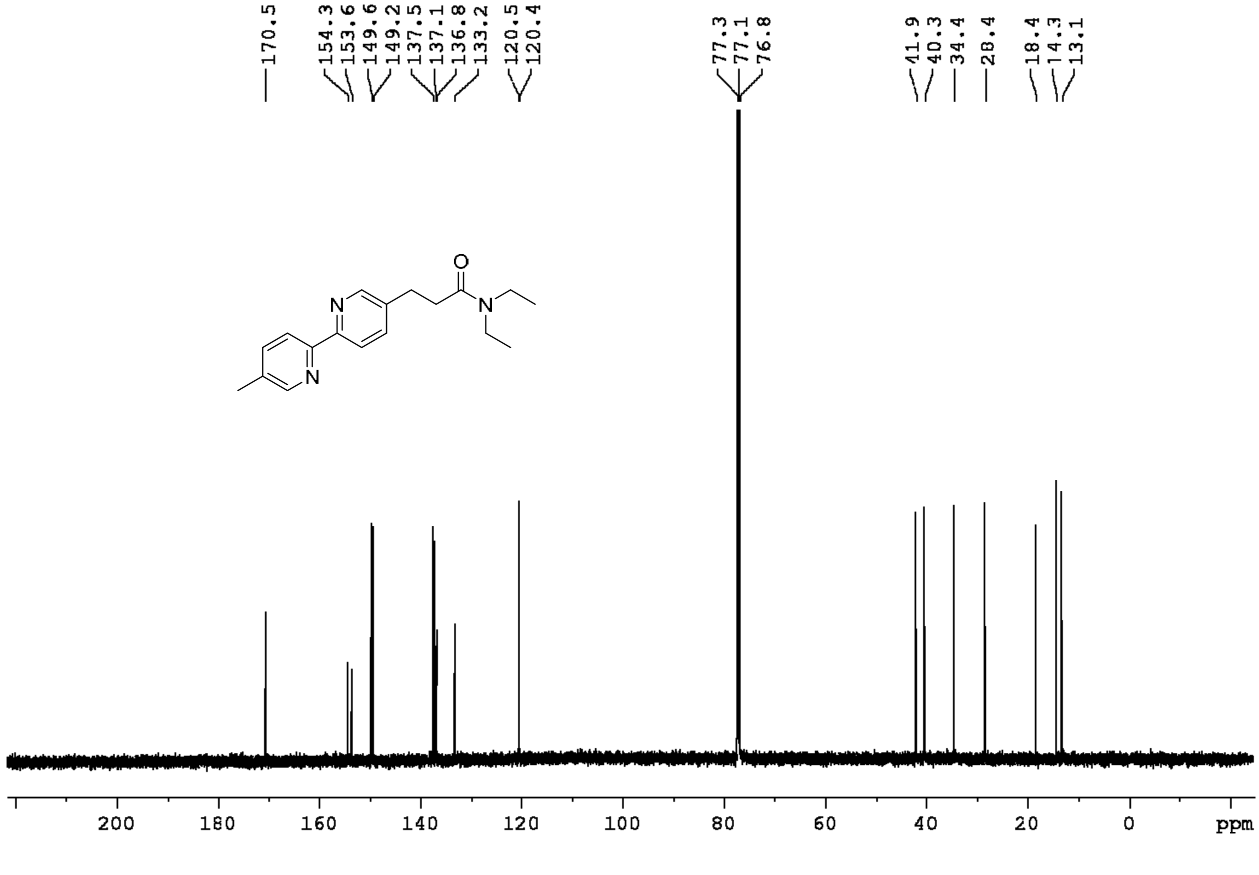

^{1}H NMR (600 MHz, $CDCl_3$) Tetrabutylammonium (4'-methyl-[2,2'-bipyridin]-4-yl)methanesulfonate (1i)

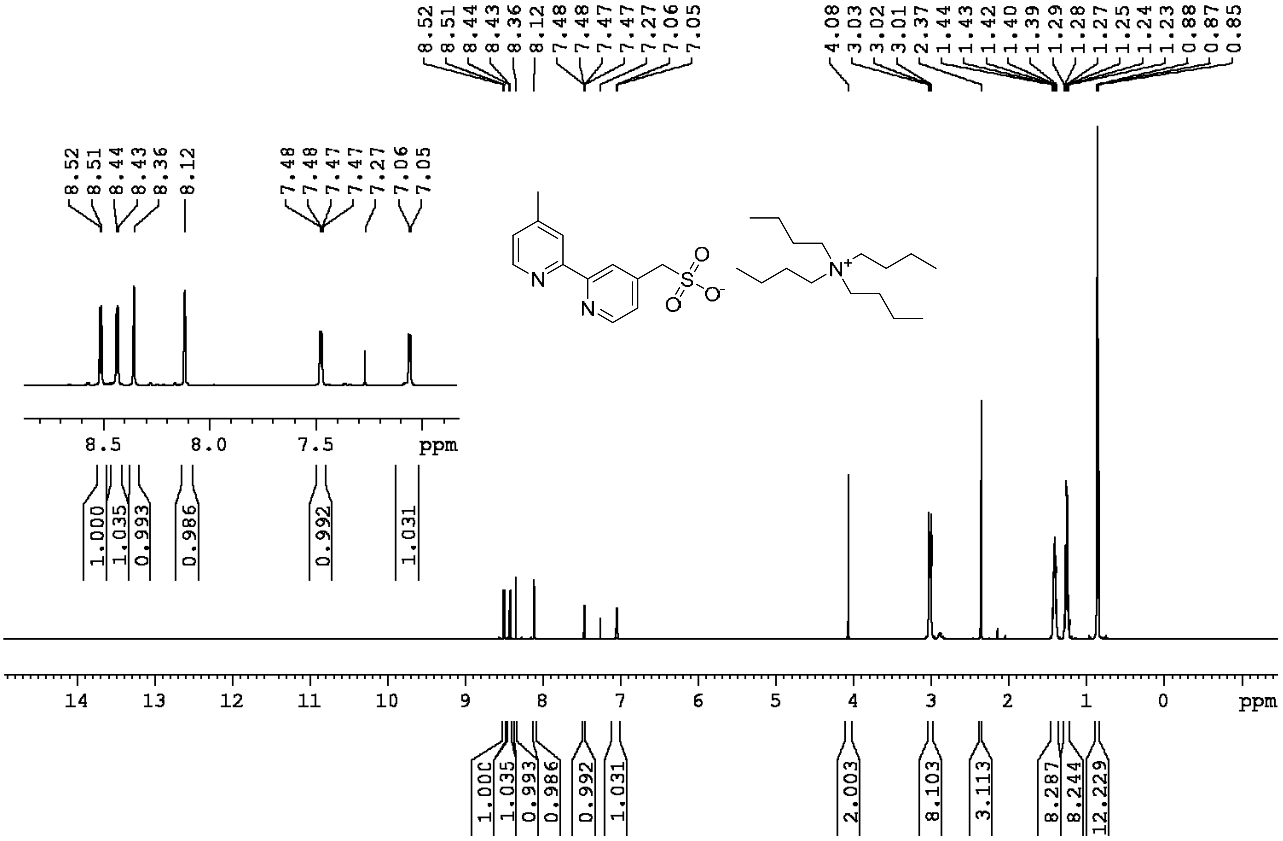

^{13}C NMR (151 MHz, $CDCl_3$) Tetrabutylammonium (4'-methyl-[2,2'-bipyridin]-4-yl)methanesulfonate (1i)

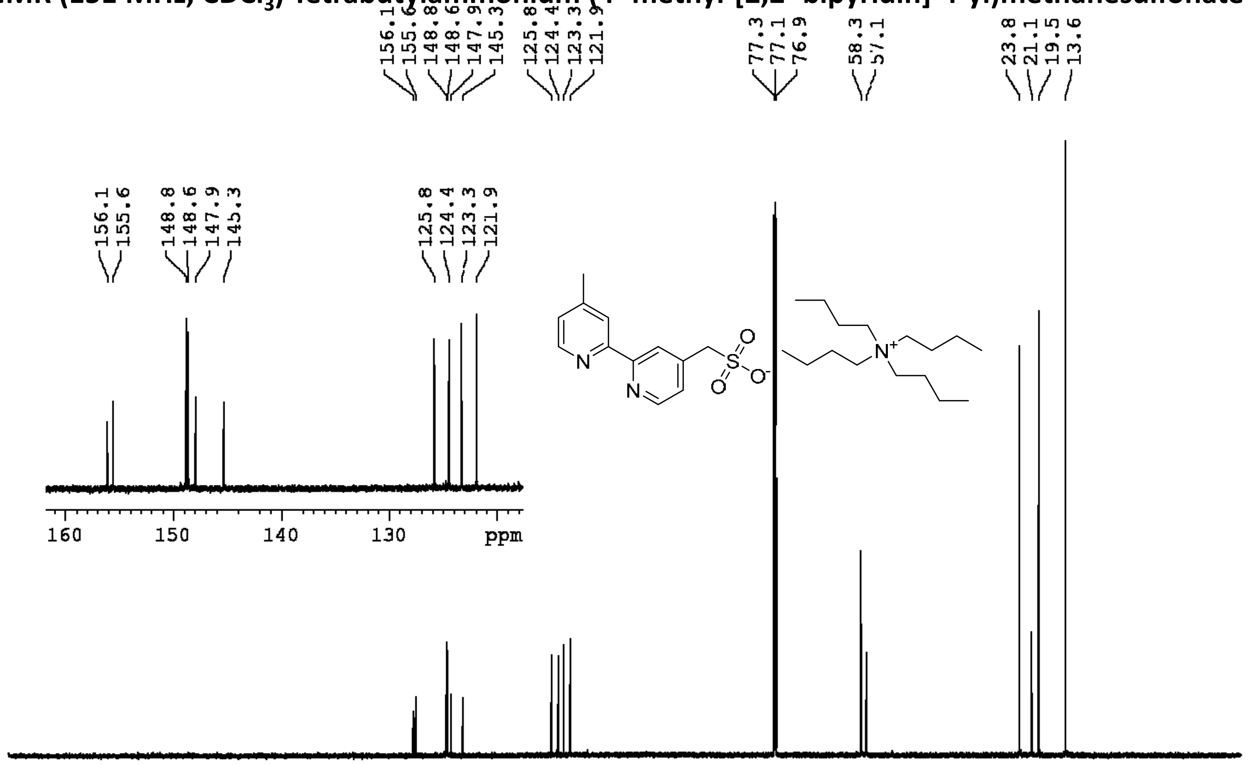

^{1}H NMR (600 MHz, $CDCl_3$) Tetrabutylammonium 2-(4'-methyl-[2,2'-bipyridin]-4-yl)ethane-1-sulfonate (1j)

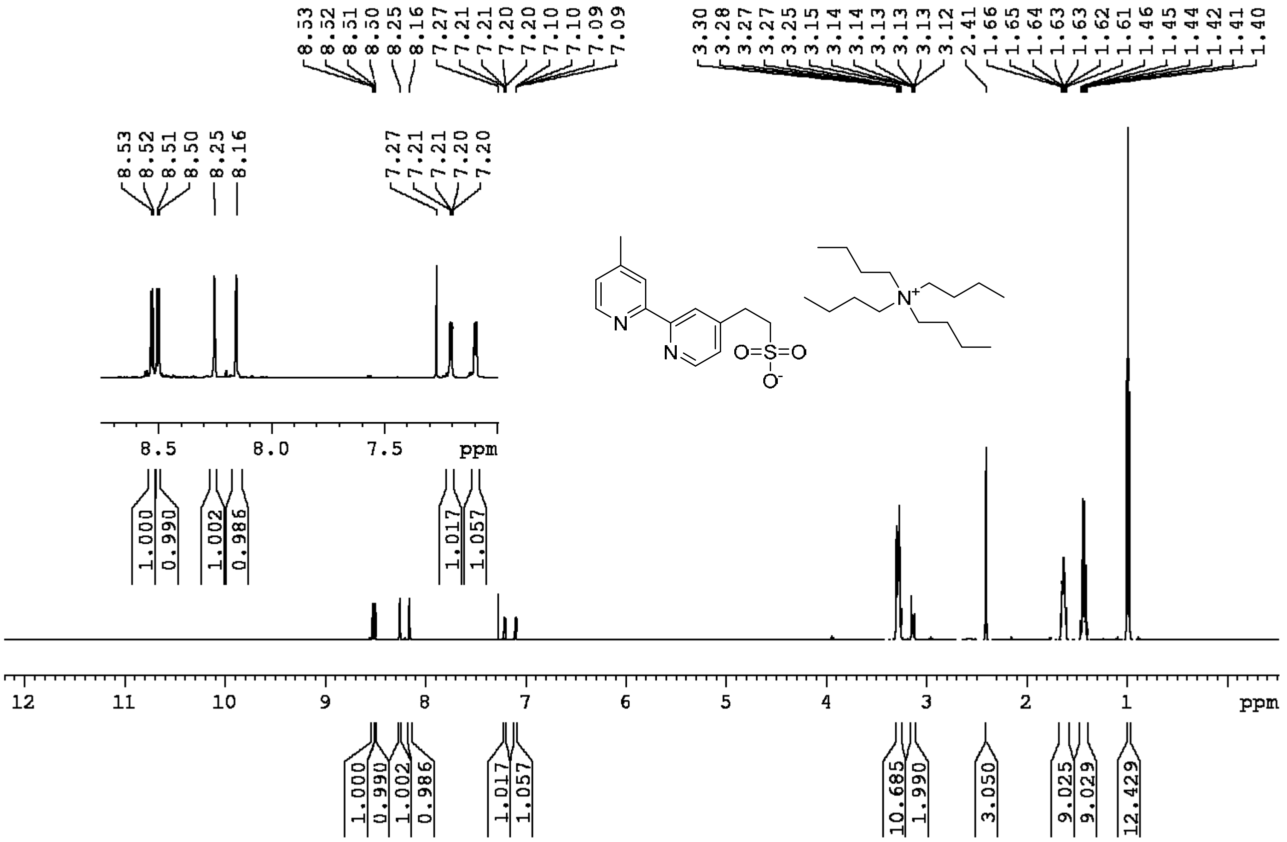

^{13}C NMR (151 MHz, $CDCl_3$) Tetrabutylammonium 2-(4'-methyl-[2,2'-bipyridin]-4-yl)ethane-1-sulfonate (1j)

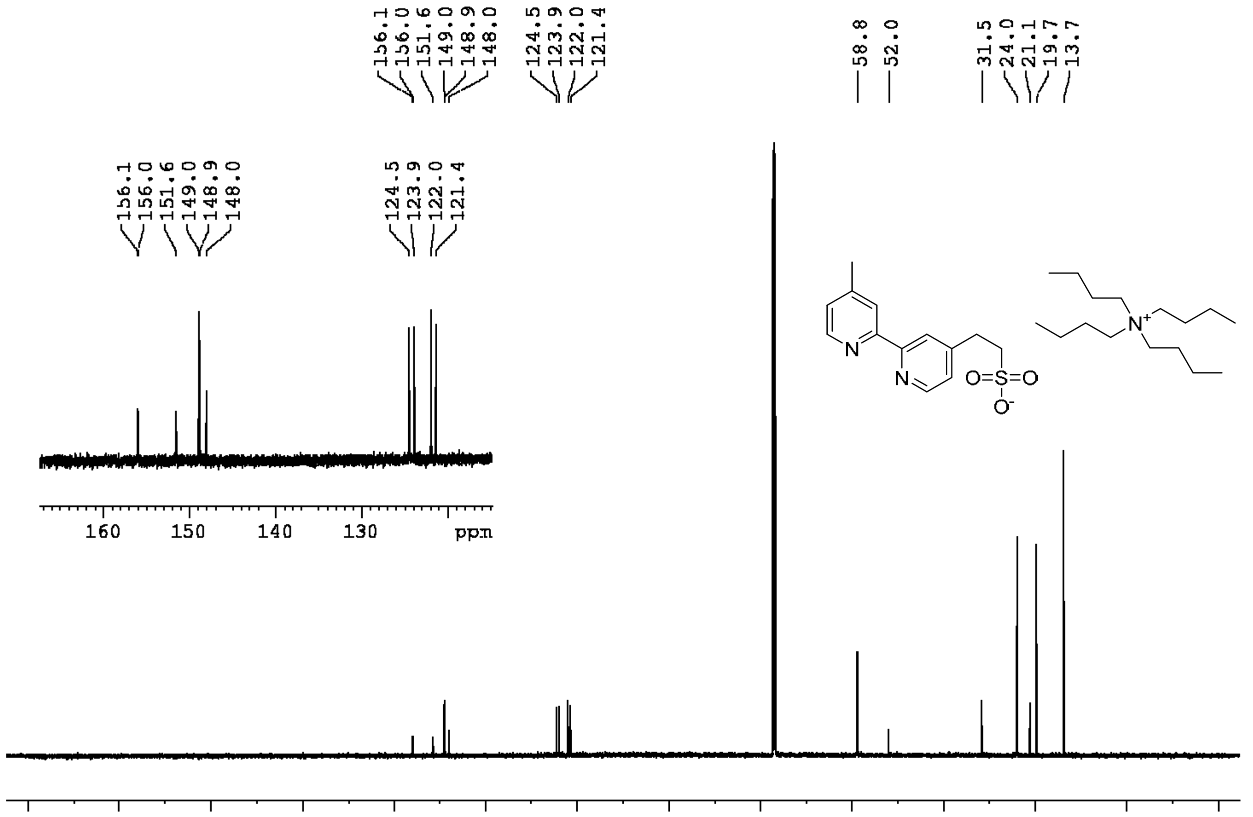

^{1}H NMR (600 MHz, $CDCl_3$) ((4'-Methyl-[2,2'-bipyridin]-4-yl)methyl)diphenylphosphine oxide (1k)

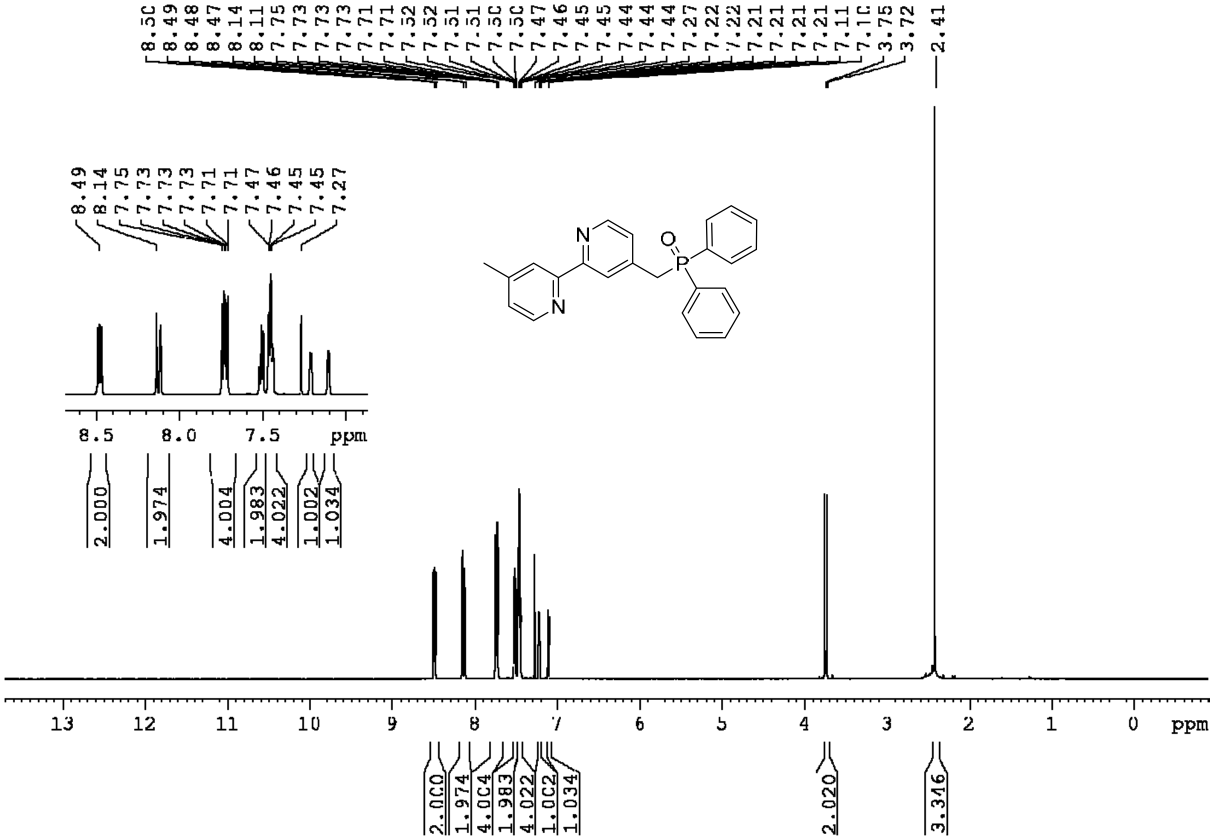

^{13}C NMR (151 MHz, $CDCl_3$) ((4'-Methyl-[2,2'-bipyridin]-4-yl)methyl)diphenylphosphine oxide (1k)

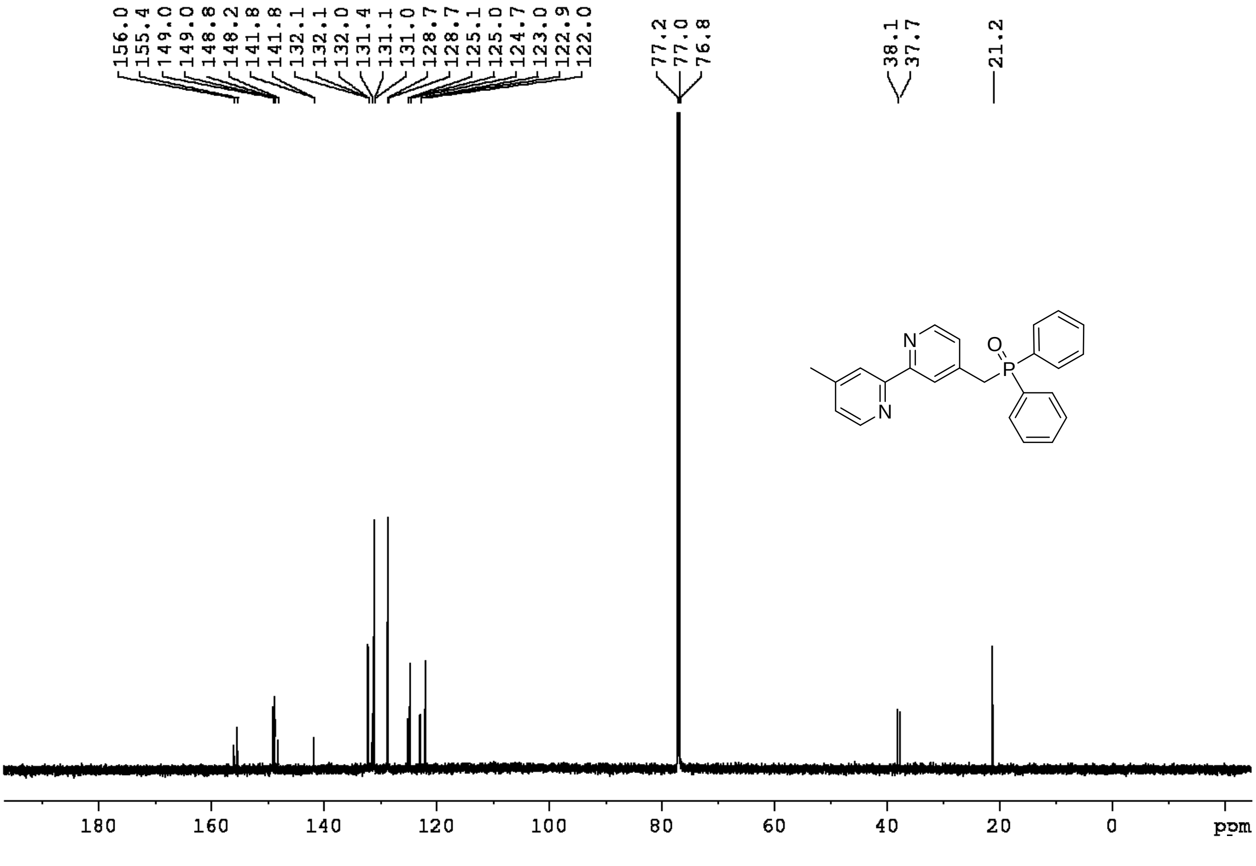

^{1}H NMR (600 MHz, $CDCl_3$) (2-(4'-methyl-[2,2'-bipyridin]-4-yl)ethyl)diphenylphosphine oxide (1l)

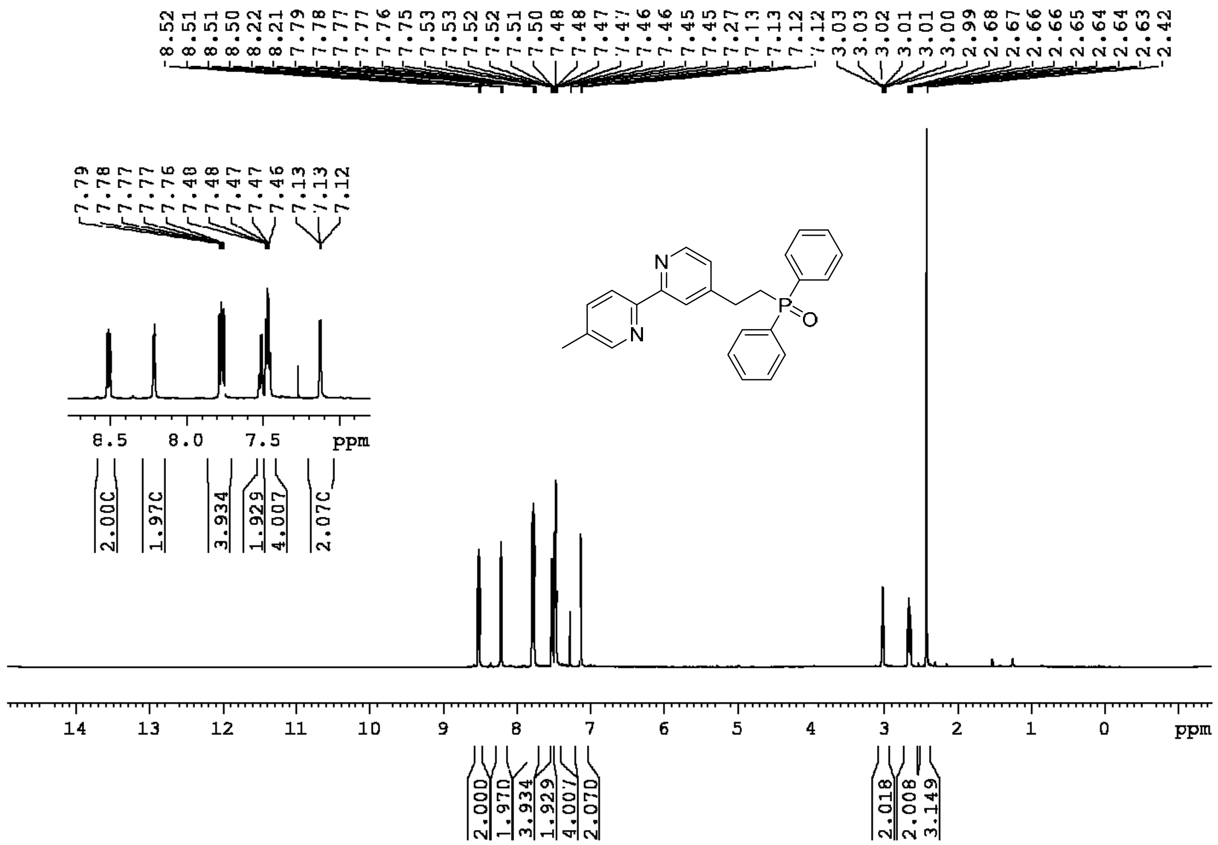

^{13}C NMR (151 MHz, $CDCl_3$) (2-(4'-methyl-[2,2'-bipyridin]-4-yl)ethyl)diphenylphosphine oxide (1l)

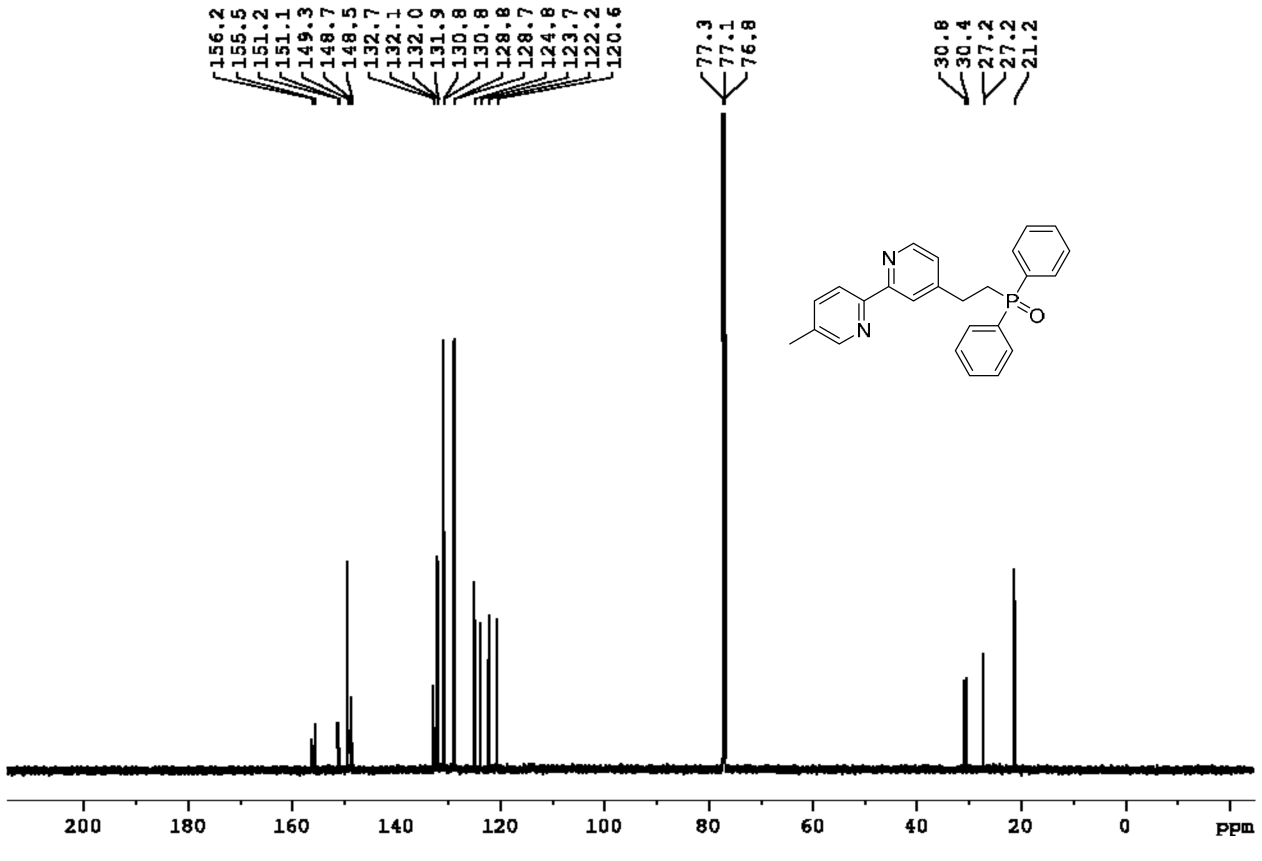

^{1}H NMR (600 MHz, $CDCl_3$) 4-Methyl-4'-((phenylthio)methyl)-2,2'-bipyridine

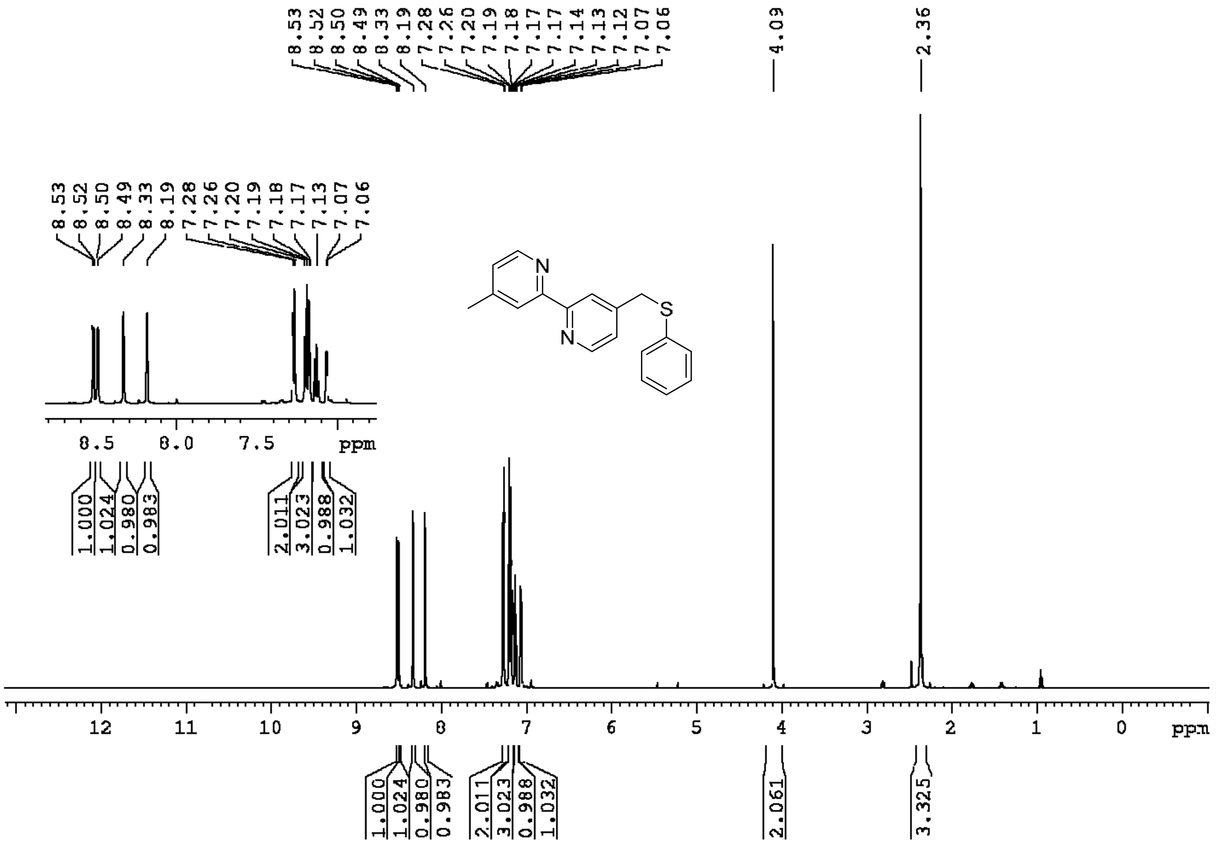

^{13}C NMR (151 MHz, $CDCl_3$) 4-Methyl-4'-((phenylthio)methyl)-2,2'-bipyridine

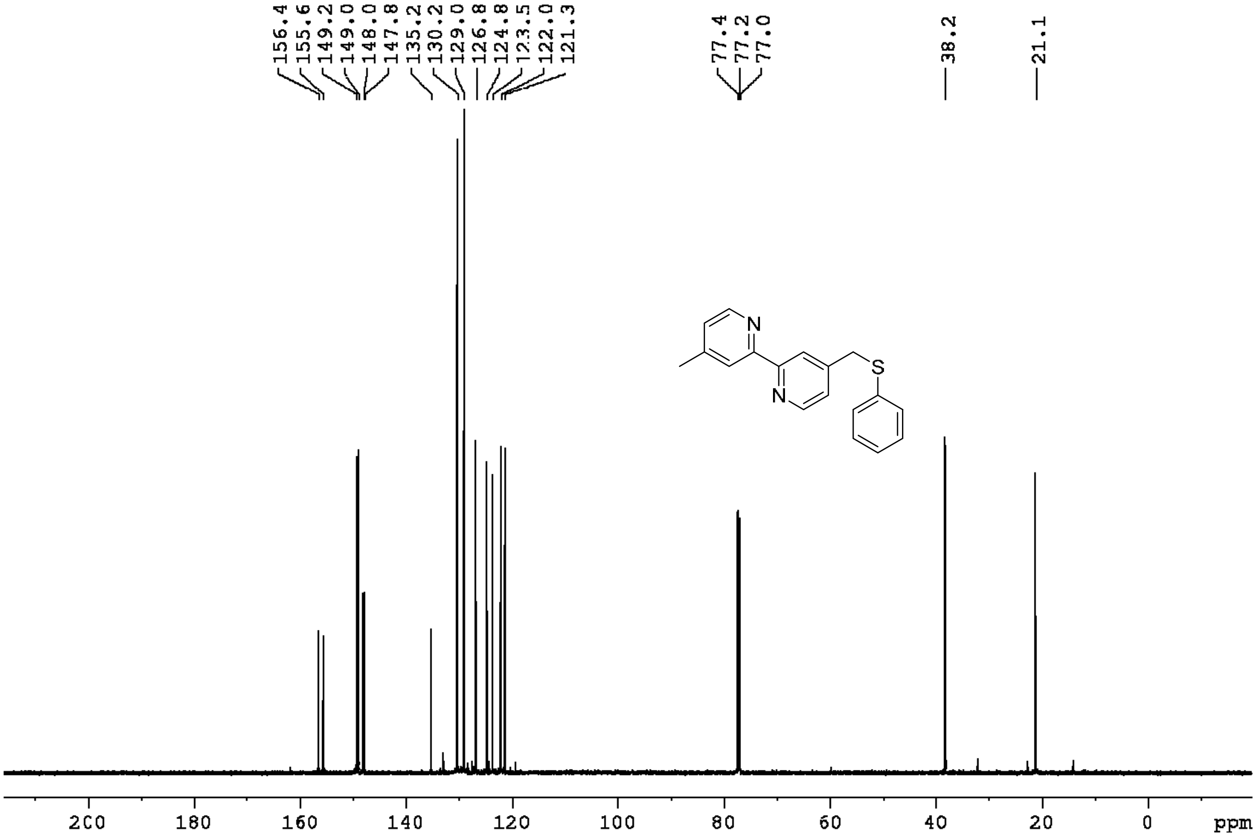

^{1}H NMR (600 MHz, $CDCl_3$) 4-Methyl-4'-((phenylsulfinyl)methyl)-2,2'-bipyridine (1m)

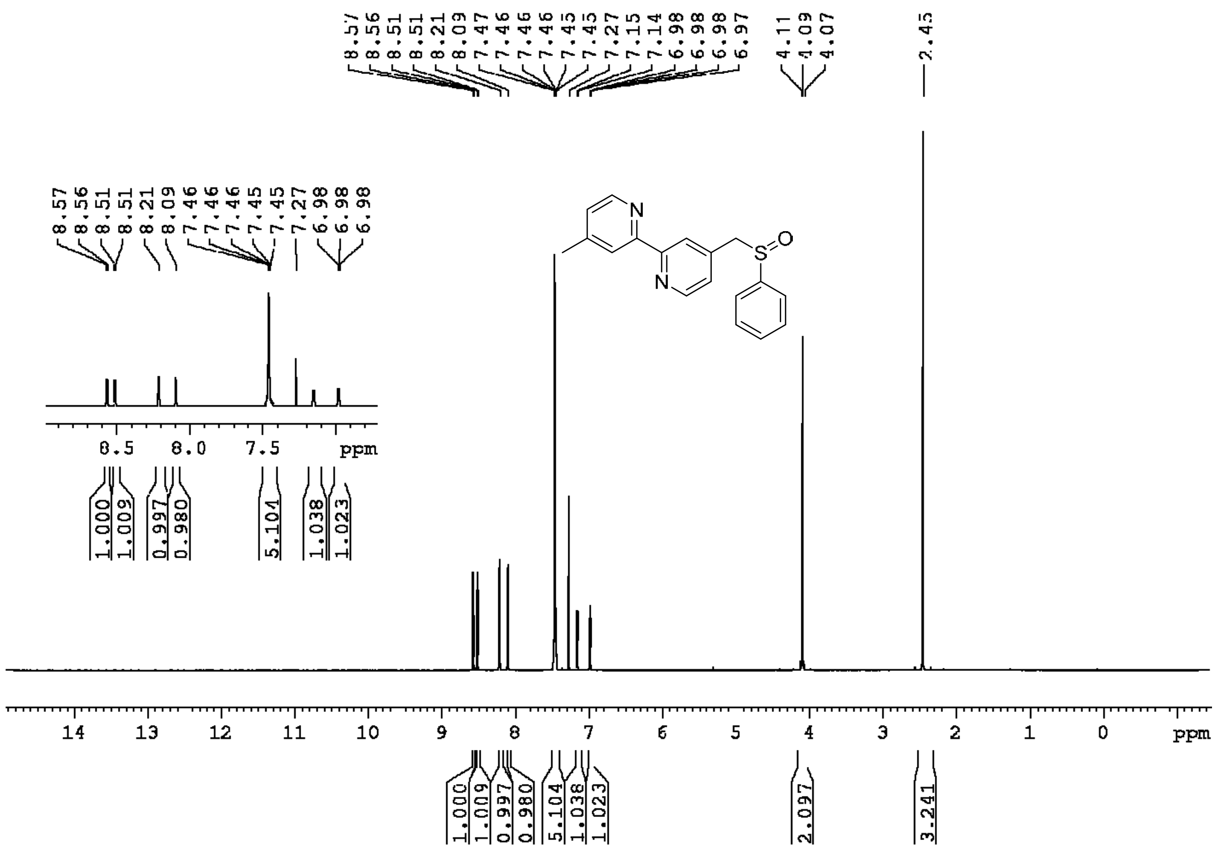

^{13}C NMR (151 MHz, $CDCl_3$) 4-Methyl-4'-((phenylsulfinyl)methyl)-2,2'-bipyridine (1m)

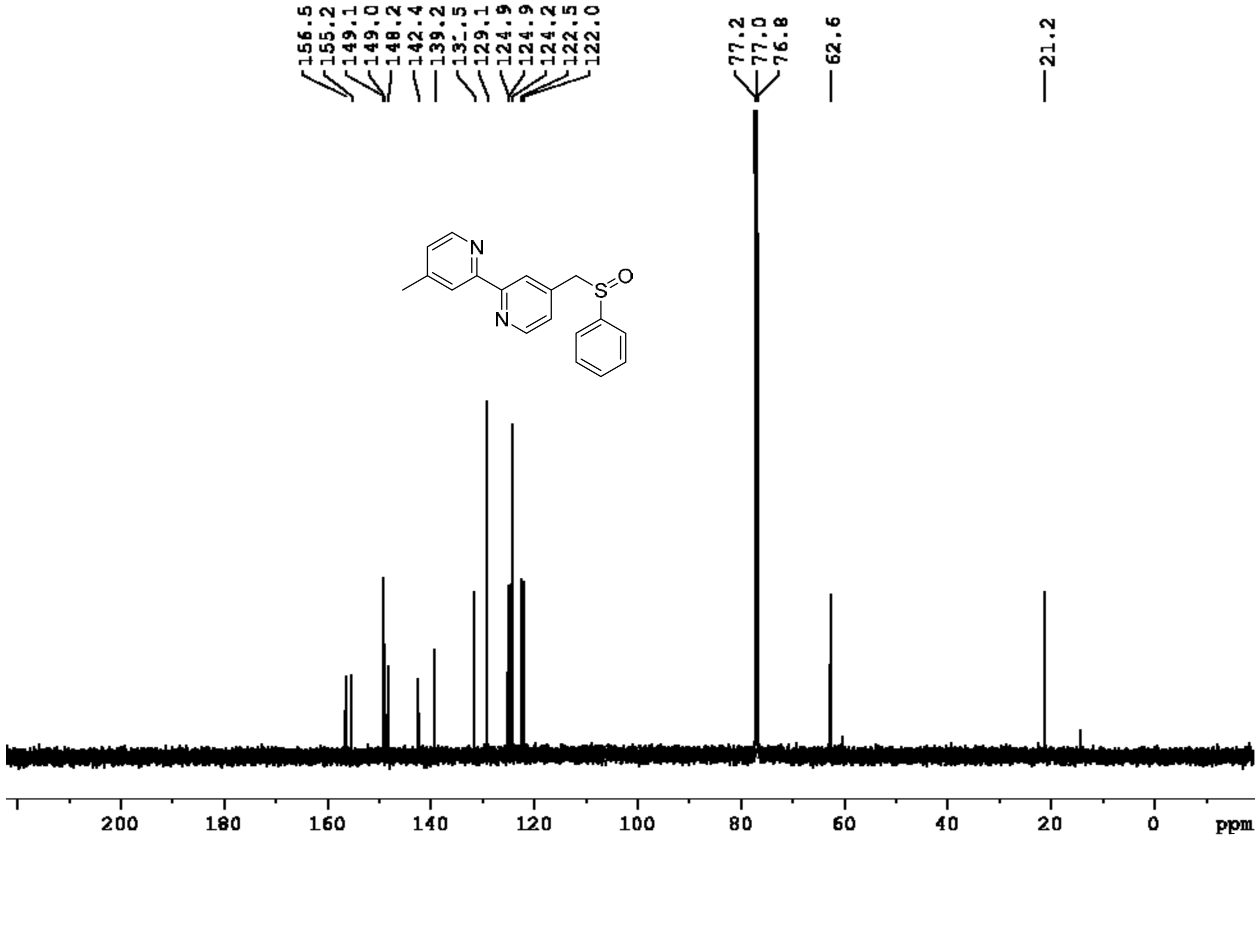

^{1}H NMR (600 MHz, $CDCl_3$) 4-Methyl-4'-(2-(phenylthio)ethyl)-2,2'-bipyridine

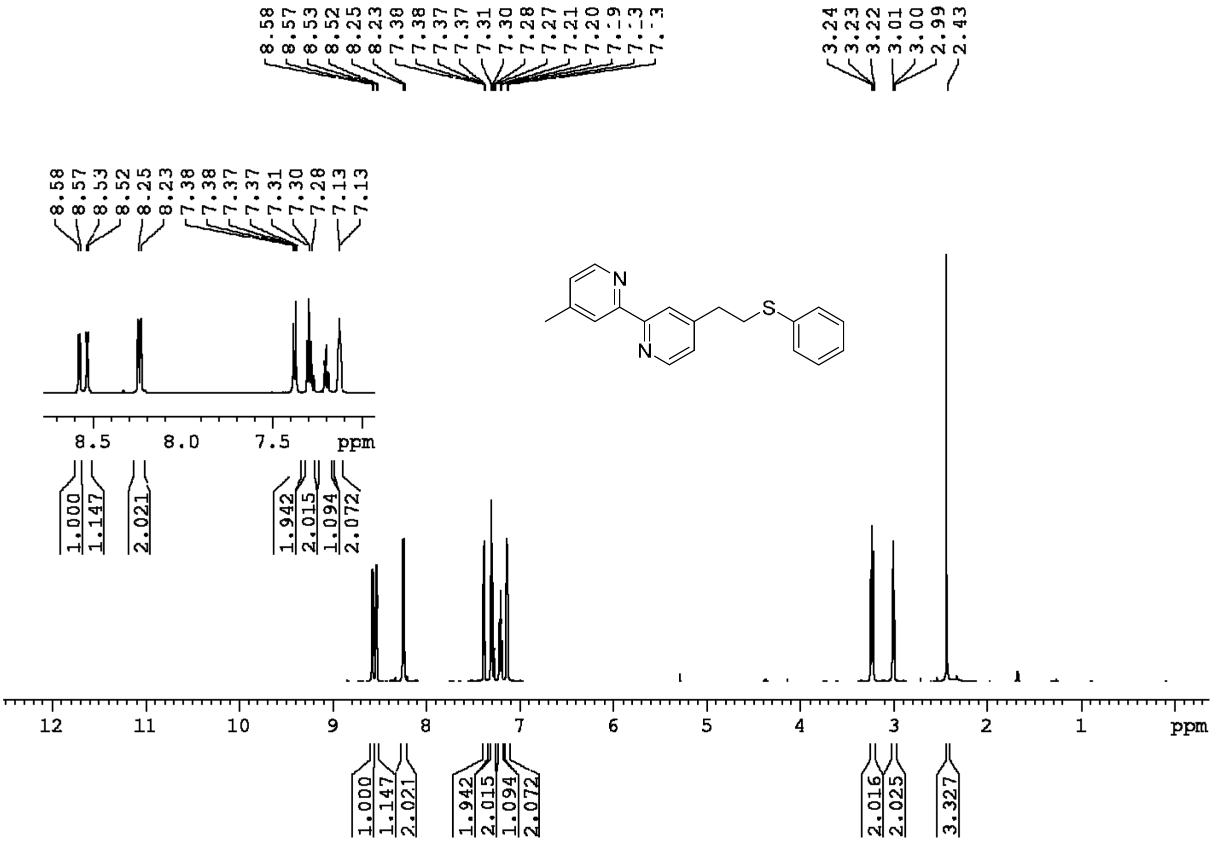

^{13}C NMR (151 MHz, $CDCl_3$) 4-Methyl-4'-(2-(phenylthio)ethyl)-2,2'-bipyridine

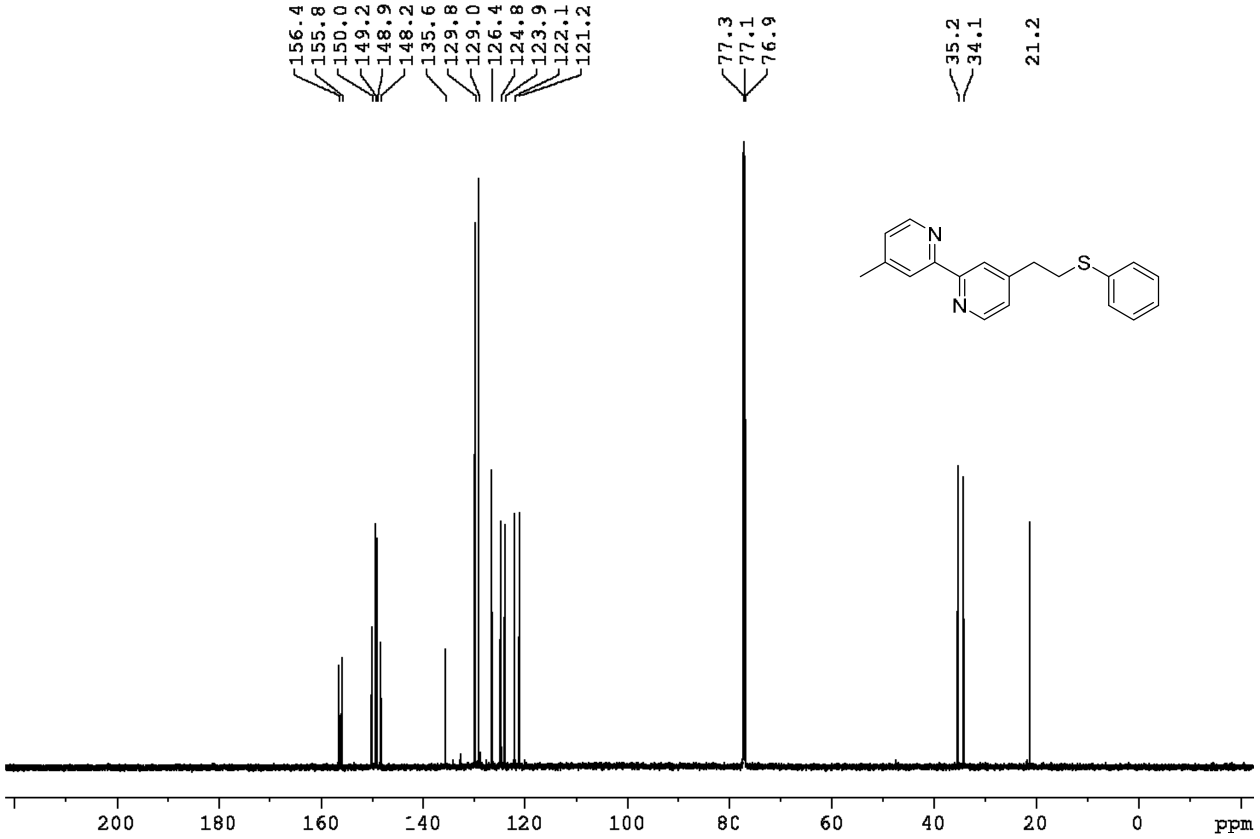

^{1}H NMR (600 MHz, $CDCl_3$) 4-Methyl-4'-(2-(phenylsulfinyl)ethyl)-2,2'-bipyridine (1n)

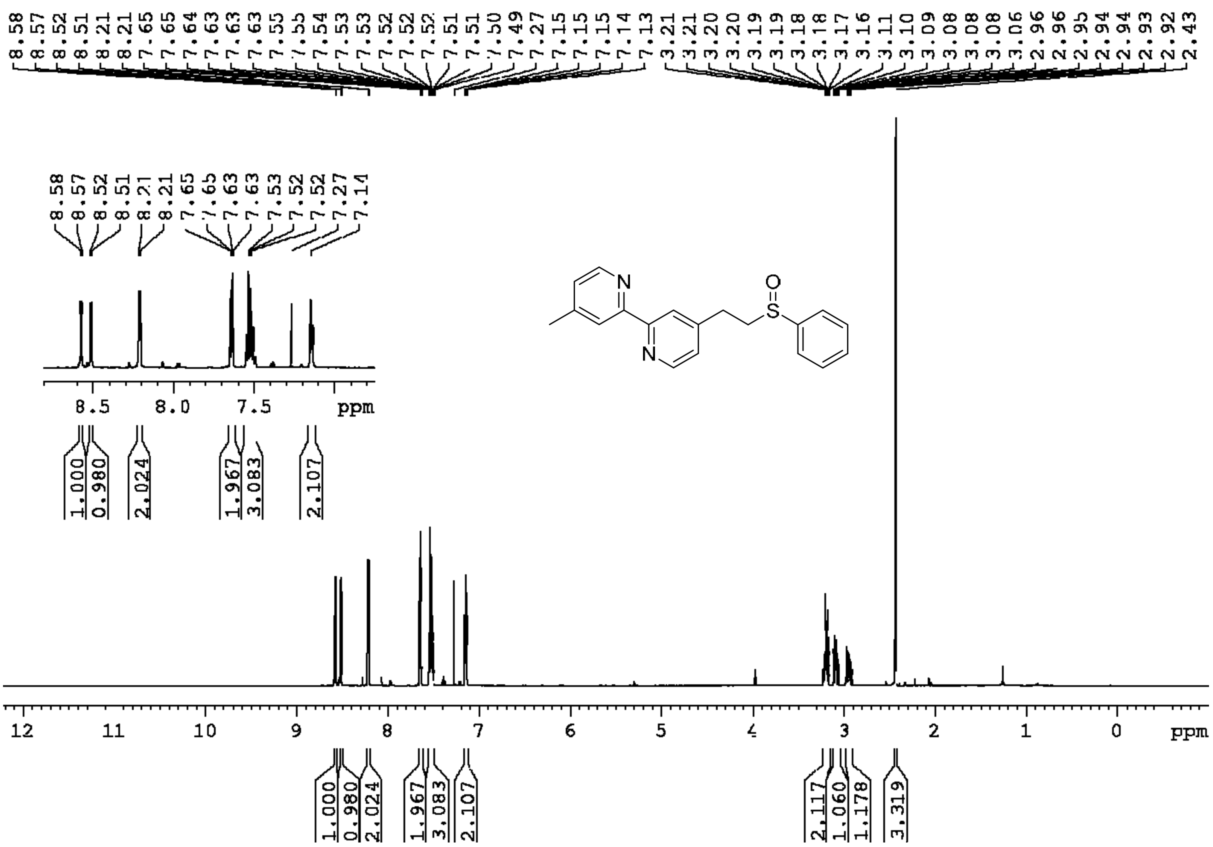

^{13}C NMR (151 MHz, $CDCl_3$) 4-Methyl-4'-(2-(phenylsulfinyl)ethyl)-2,2'-bipyridine (1n)

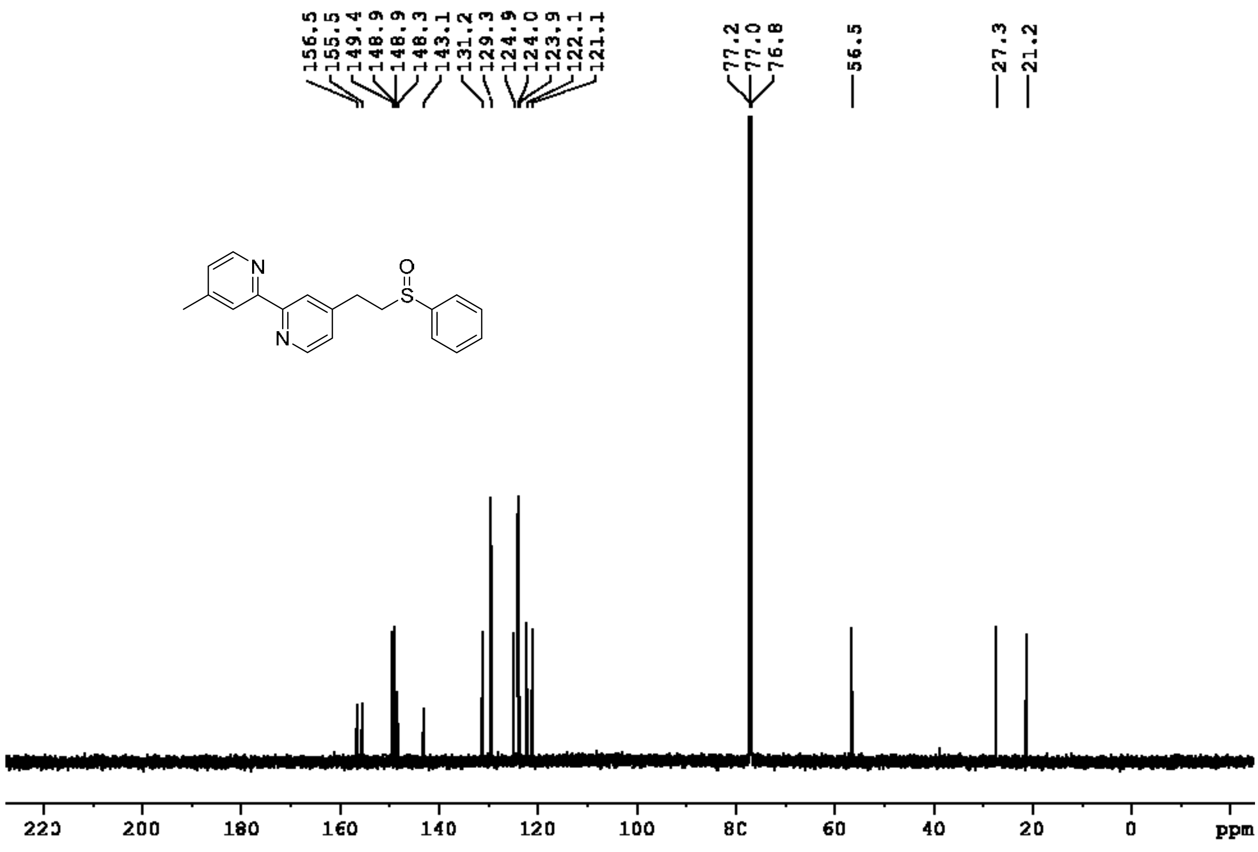

^{1}H NMR (600 MHz, $CDCl_3$) *N,N*-diethyl-2-(4'-methyl-[2,2'-bipyridin]-4-yl)acetamide (1o)

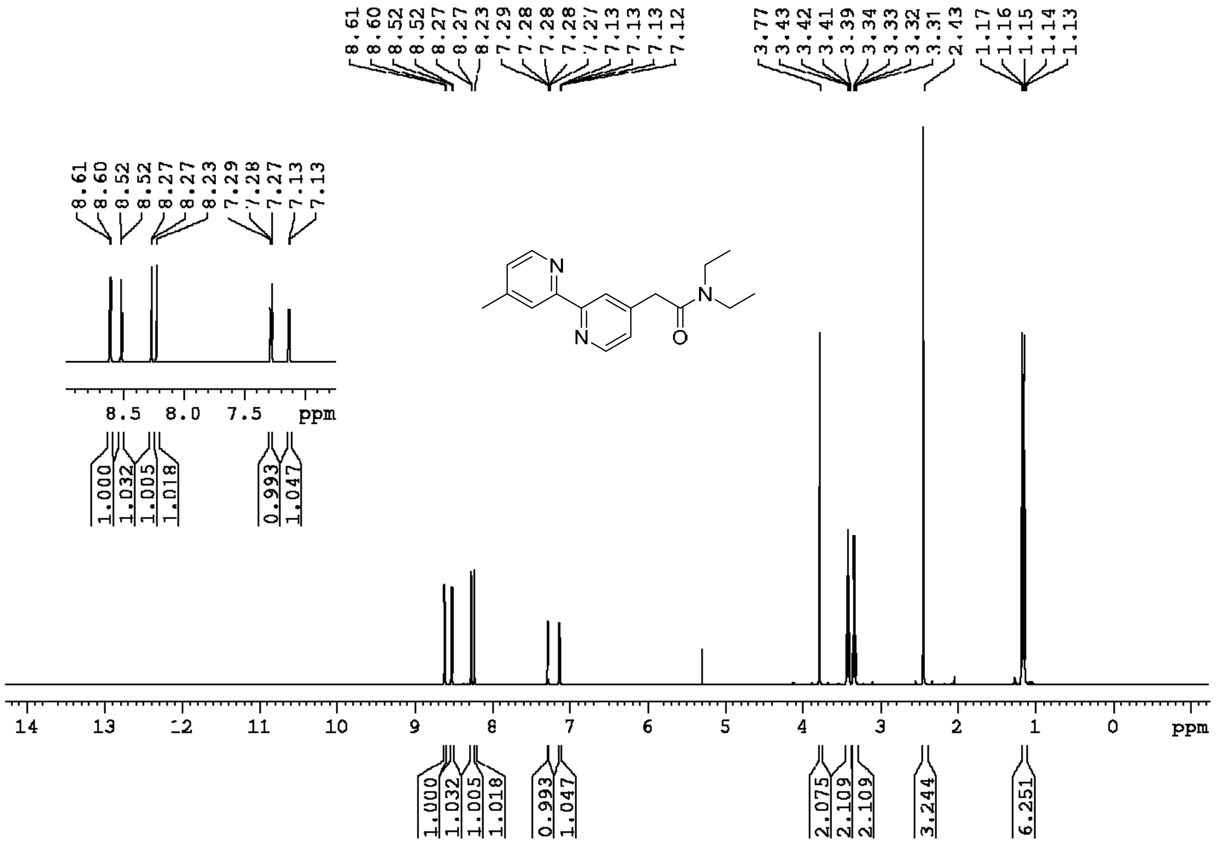

^{13}C NMR (151 MHz, $CDCl_3$) *N,N*-diethyl-2-(4'-methyl-[2,2'-bipyridin]-4-yl)acetamide (1o)

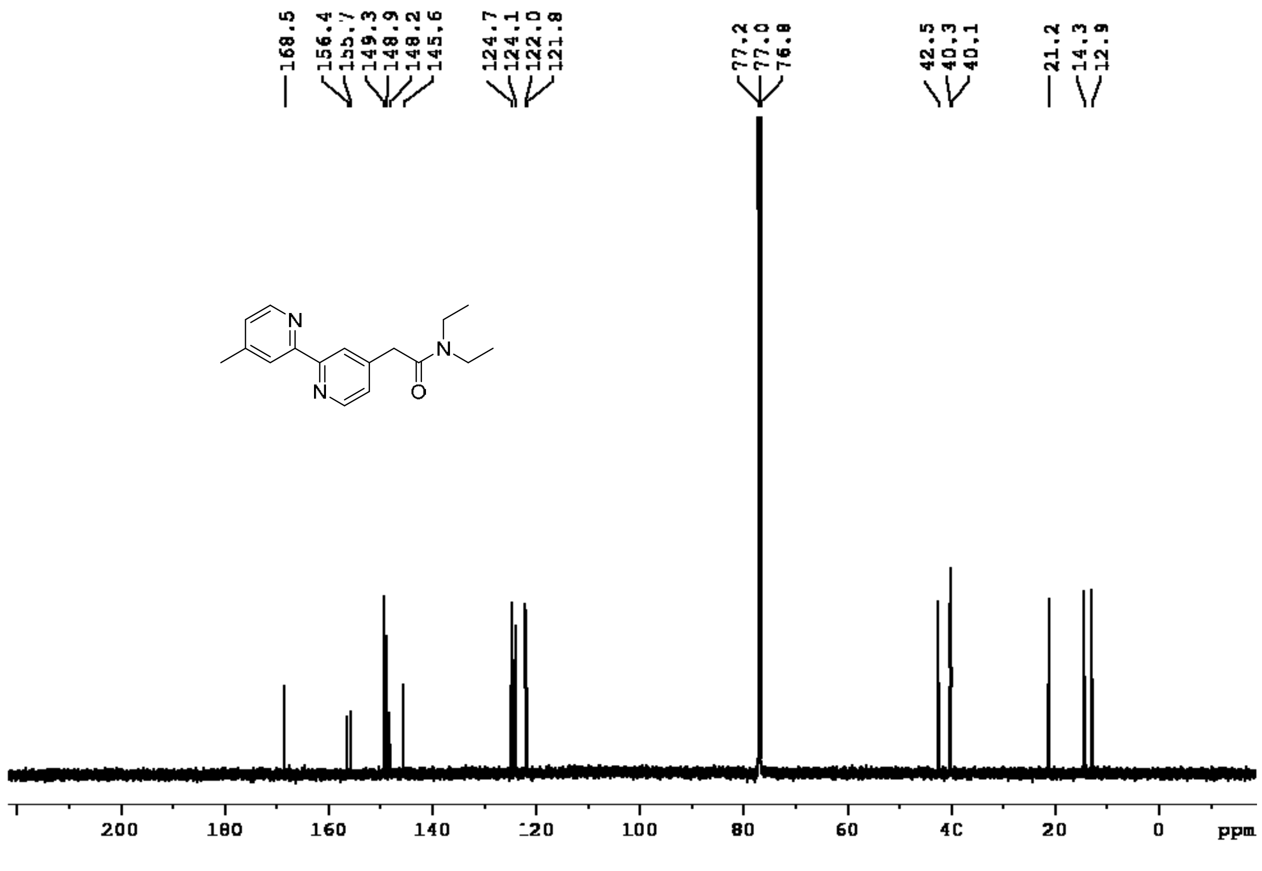

^{1}H NMR (600 MHz, $CDCl_3$) *N,N*-diethyl-3-(4'-methyl-[2,2'-bipyridin]-4-yl)propanamide (1p)

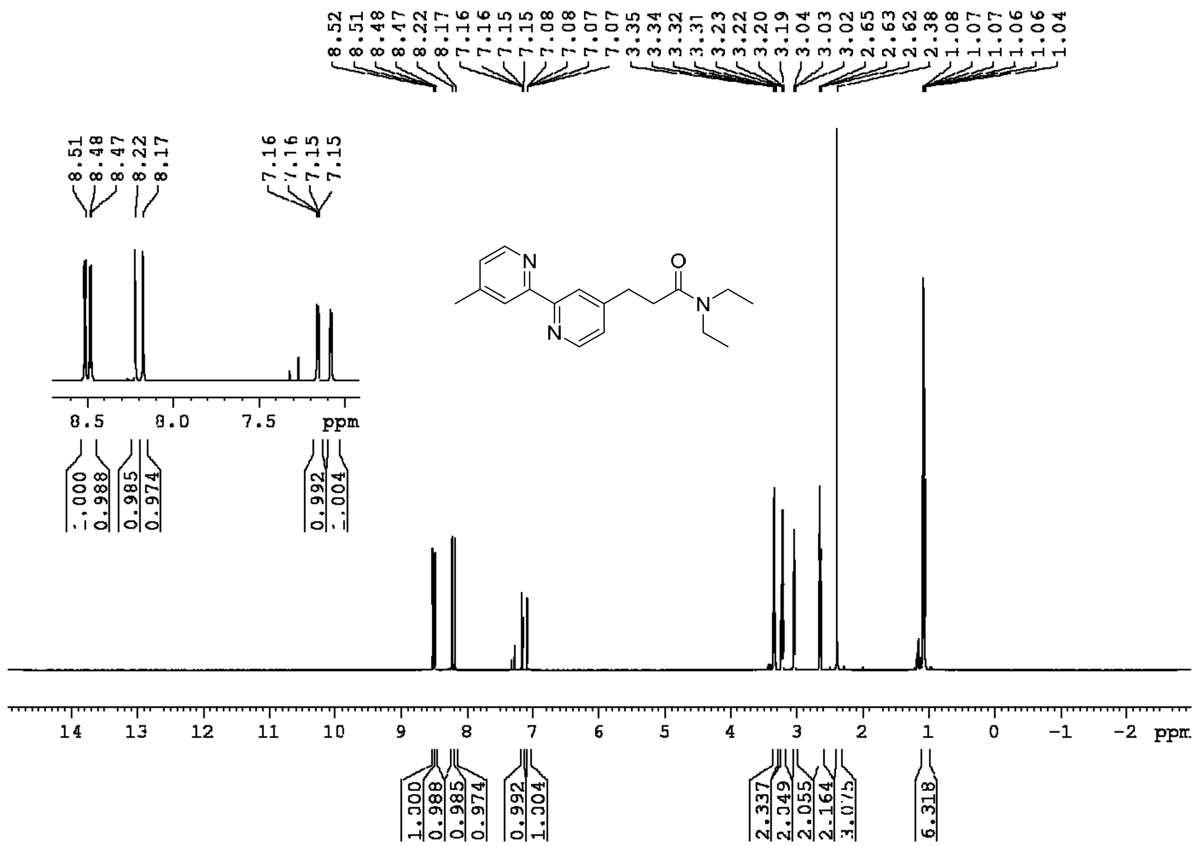

^{13}C NMR (151 MHz, $CDCl_3$) *N,N*-diethyl-3-(4'-methyl-[2,2'-bipyridin]-4-yl)propanamide (1p)

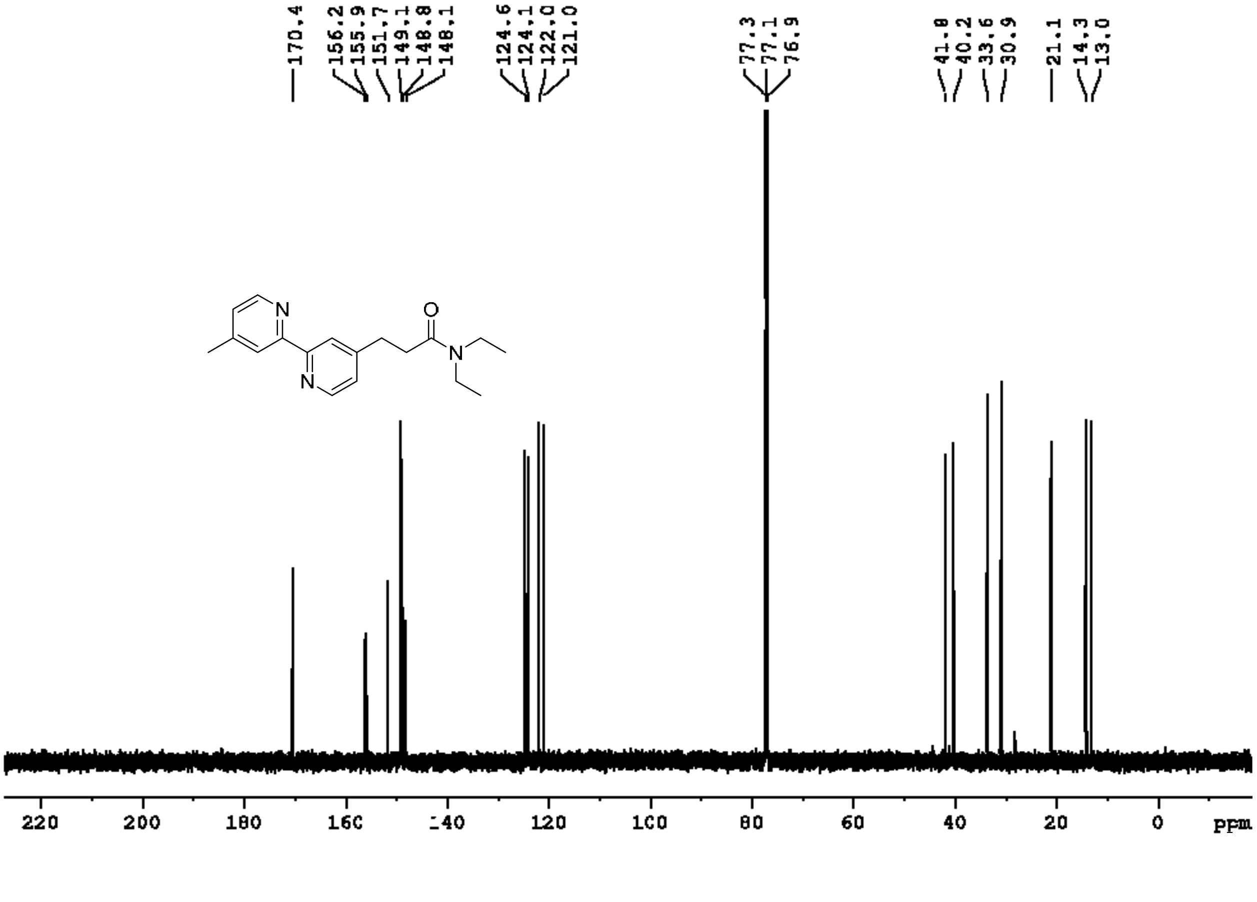

Substrate NMRs

^{1}H-NMR (600 MHz, $CDCl_3$) 2,2,2-Trifluoro-*N*-(2-(trifluoromethyl)benzyl)acetamide (2a)

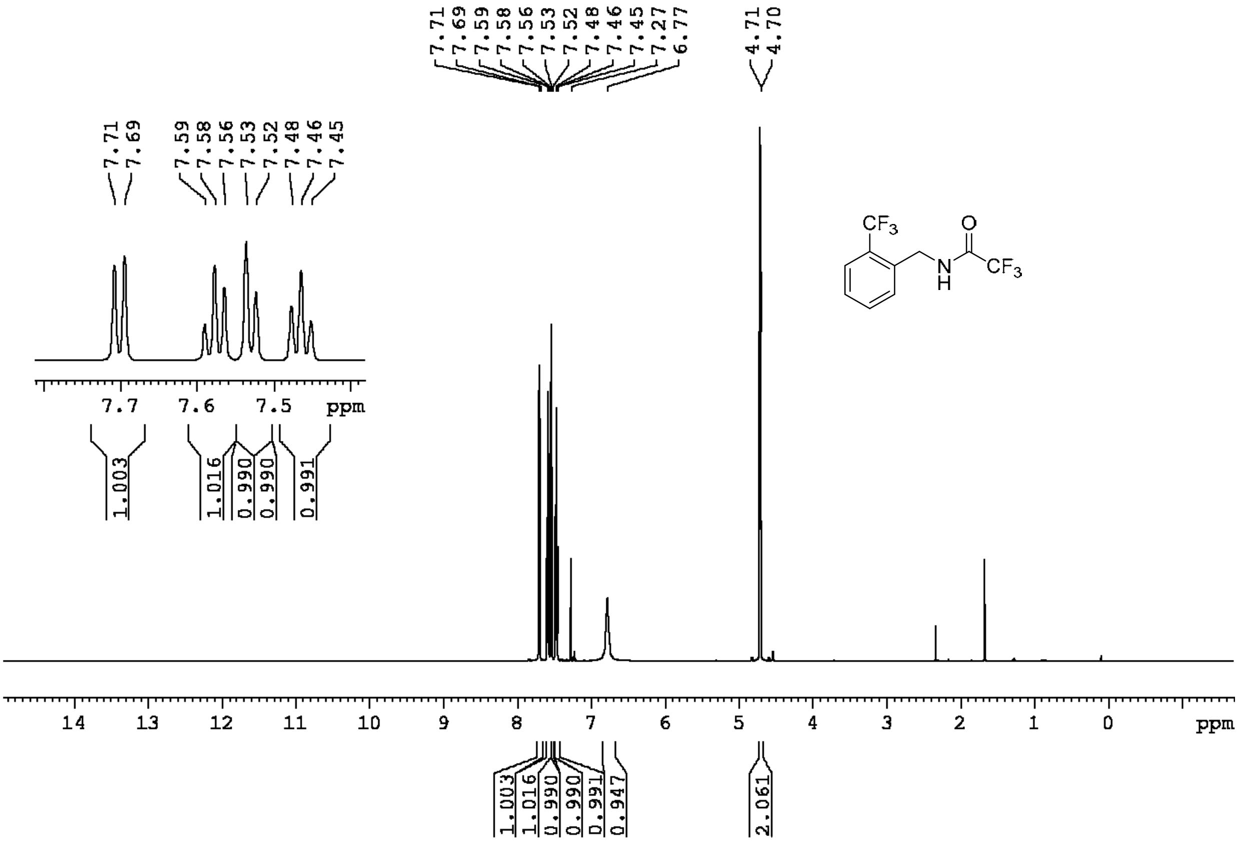

^{13}C-NMR (151 MHz, $CDCl_3$) 2,2,2-Trifluoro-*N*-(2-(trifluoromethyl)benzyl)acetamide (2a)

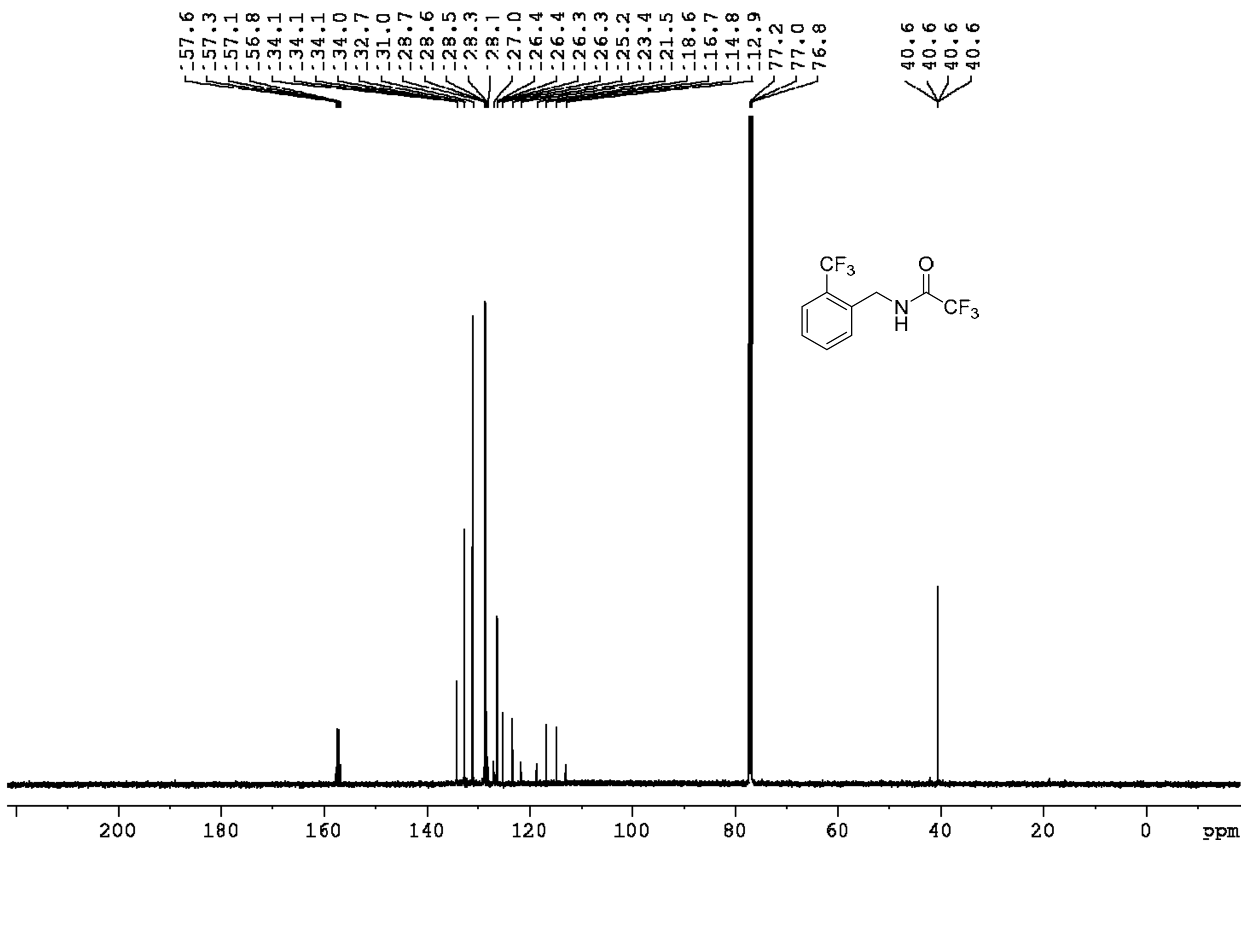

^{1}H-NMR (600 MHz, $CDCl_3$) 2,2,2-Trifluoro-*N*-(2-methylbenzyl)acetamide (2b)

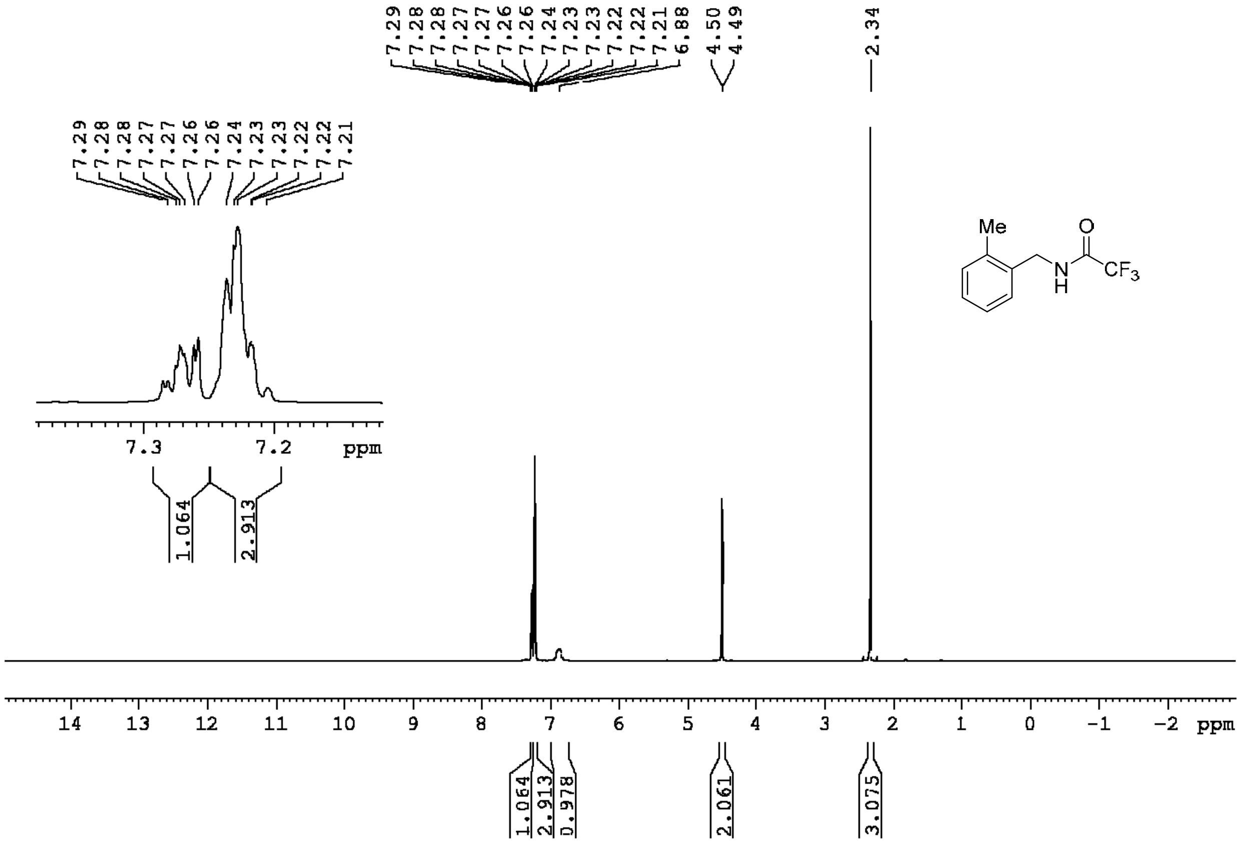

^{13}C-NMR (151 MHz, $CDCl_3$) 2,2,2-Trifluoro-*N*-(2-methylbenzyl)acetamide (2b)

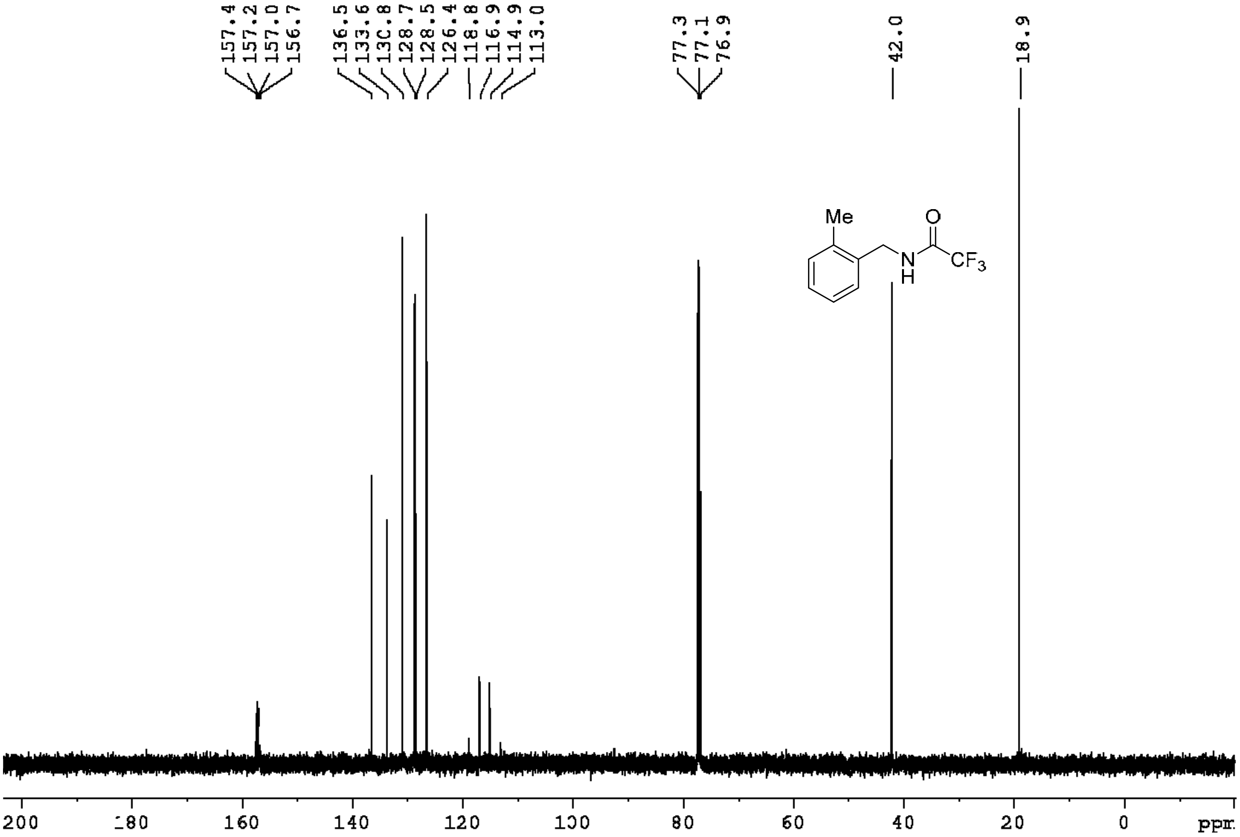

^{1}H-NMR (600 MHz, $CDCl_3$) 2,2,2-Trifluoro-*N*-(2-methoxybenzyl)acetamide (2c)

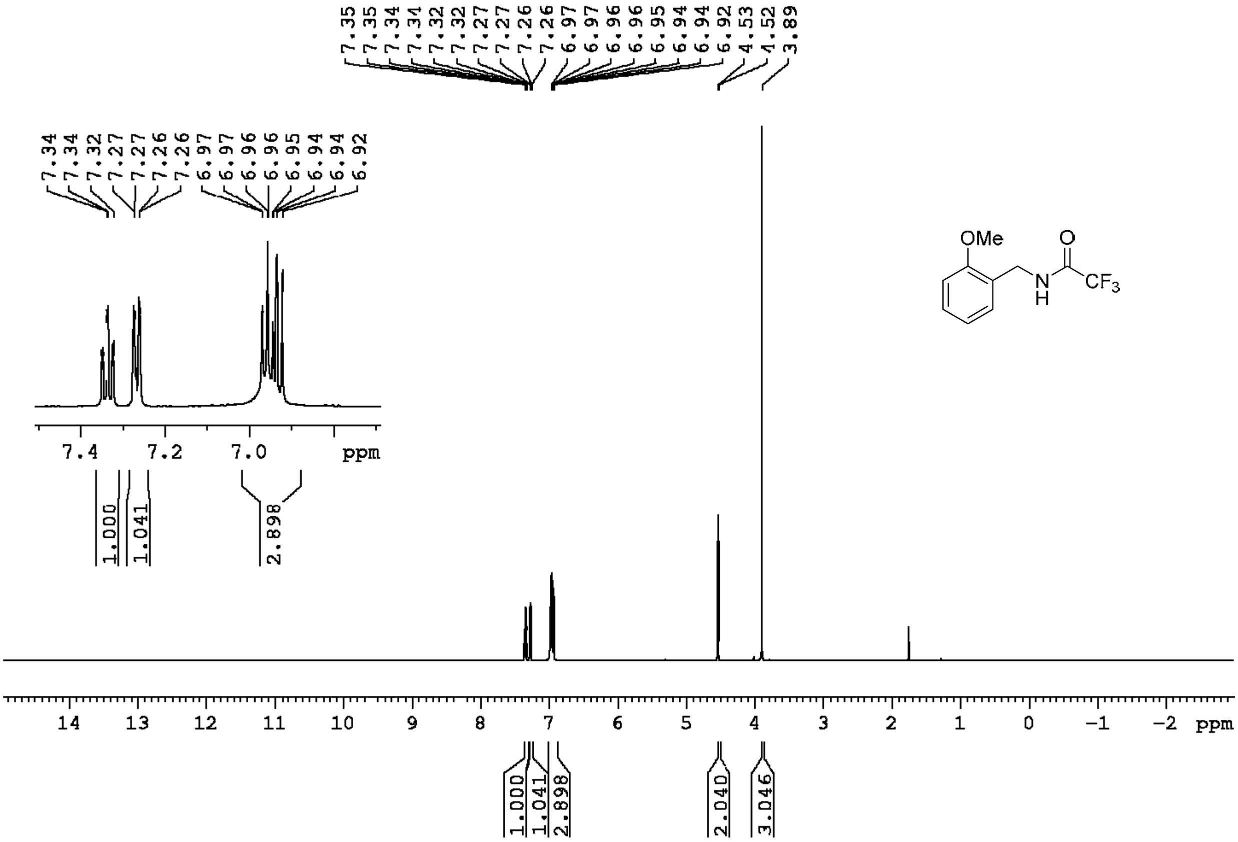

^{13}C-NMR (151 MHz, $CDCl_3$) 2,2,2-Trifluoro-*N*-(2-methoxybenzyl)acetamide (2c)

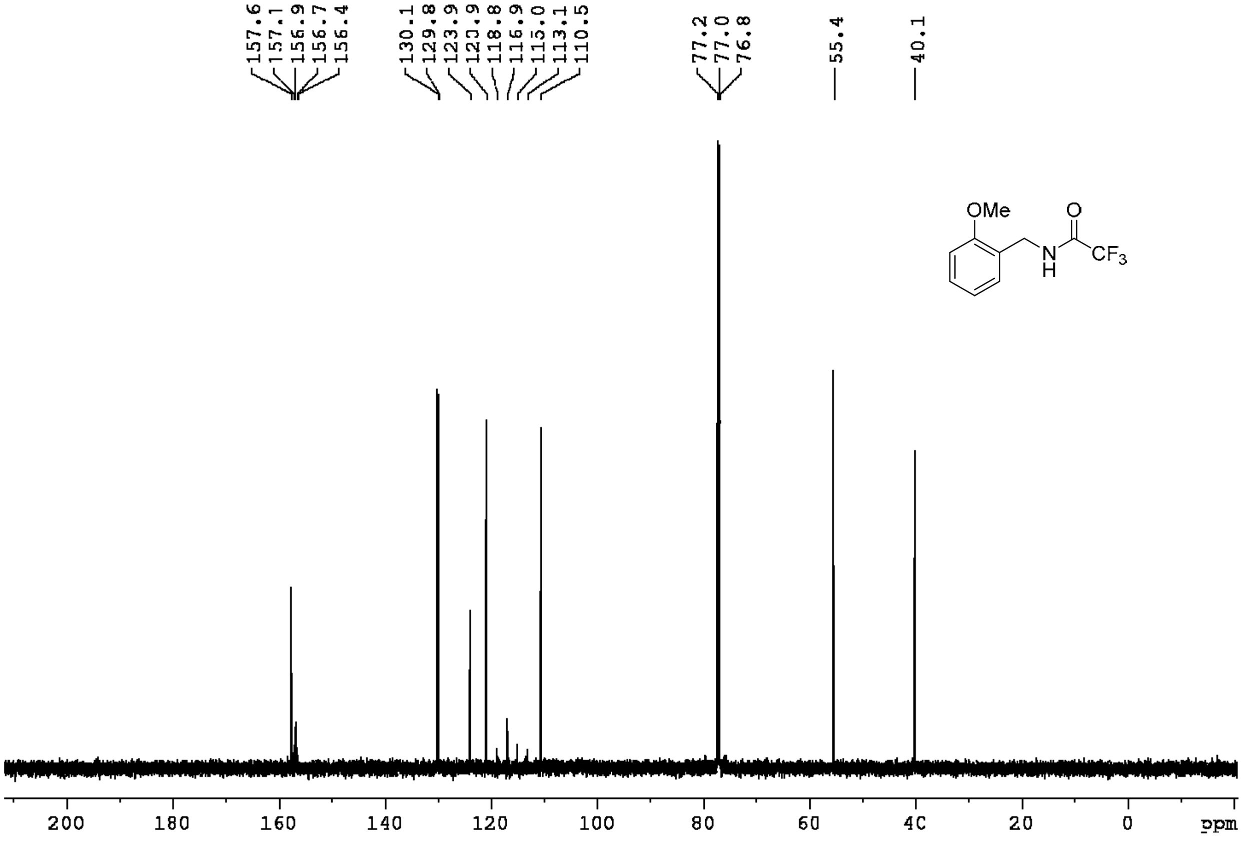

^{1}H-NMR (600 MHz, $CDCl_3$) *N*-(2-chlorobenzyl)-2,2,2-trifluoroacetamide (2d)

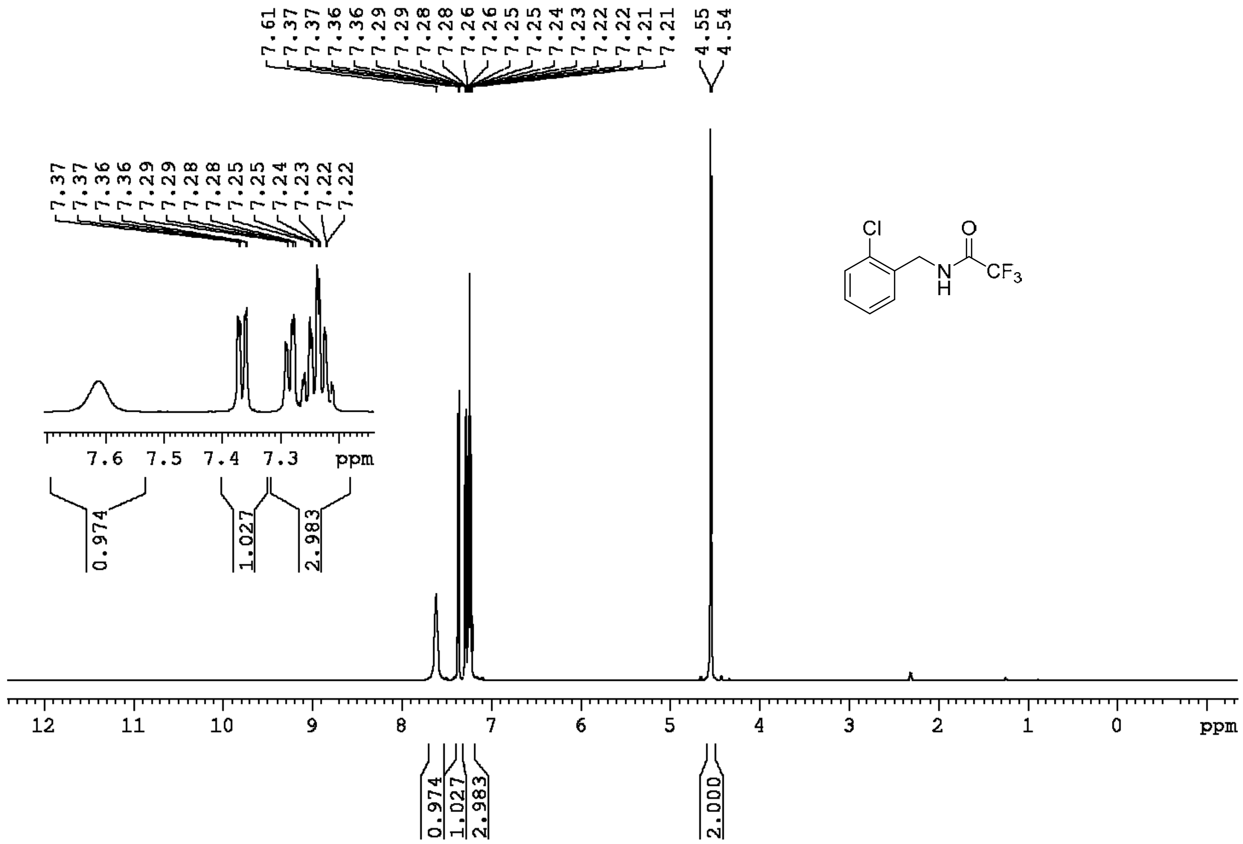

^{13}C-NMR (151 MHz, $CDCl_3$) *N*-(2-chlorobenzyl)-2,2,2-trifluoroacetamide (2d)

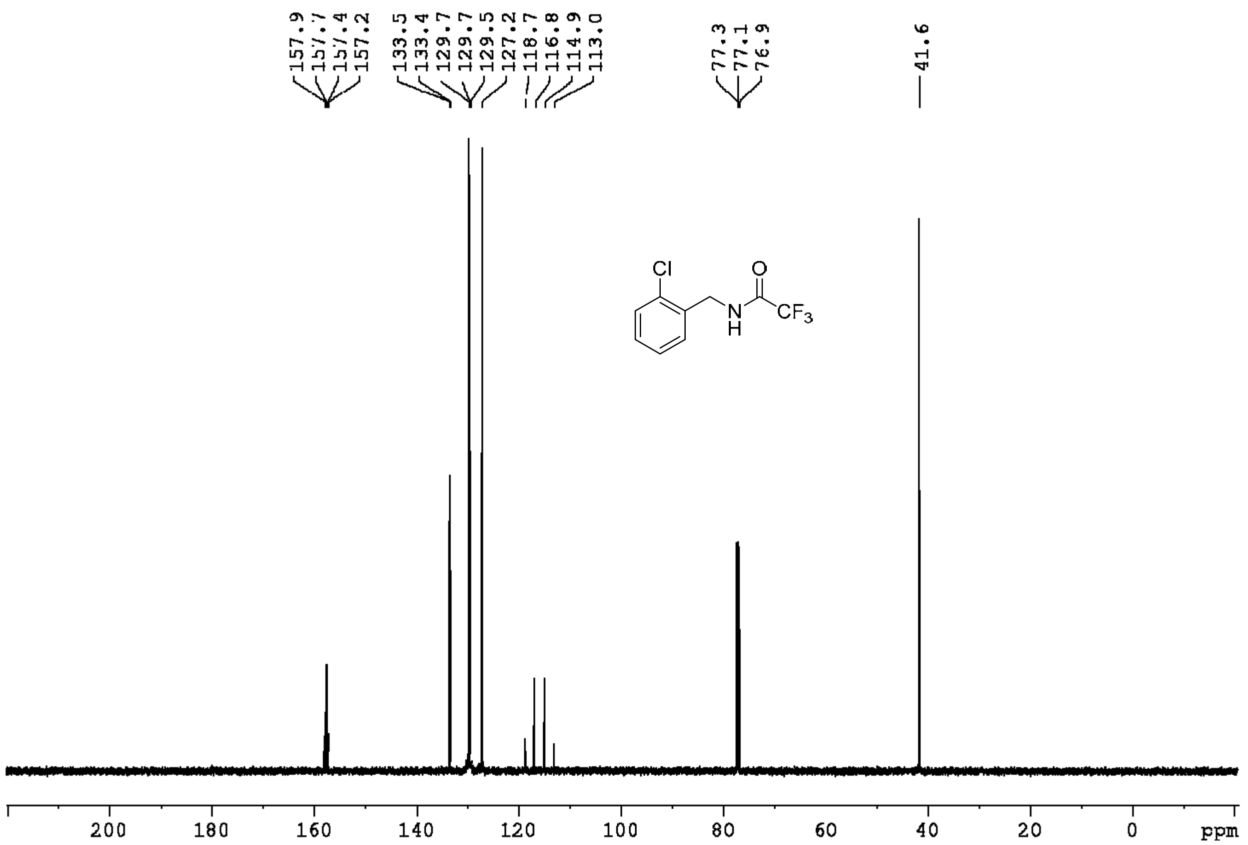

^{1}H-NMR (600 MHz, $CDCl_3$) 2,2,2-Trifluoro-*N*-(3-fluorobenzyl)acetamide (2e)

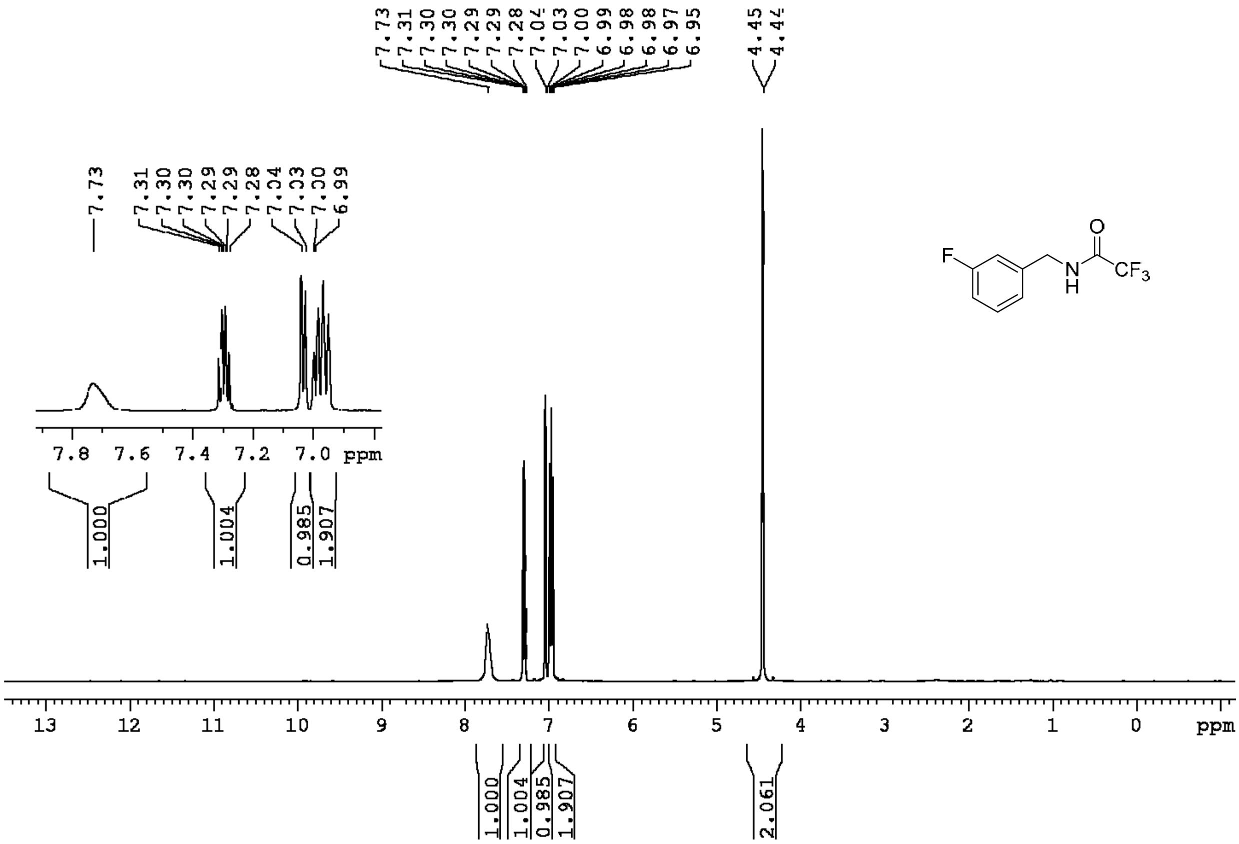

^{13}C-NMR (151 MHz, $CDCl_3$) 2,2,2-Trifluoro-*N*-(3-fluorobenzyl)acetamide (2e)

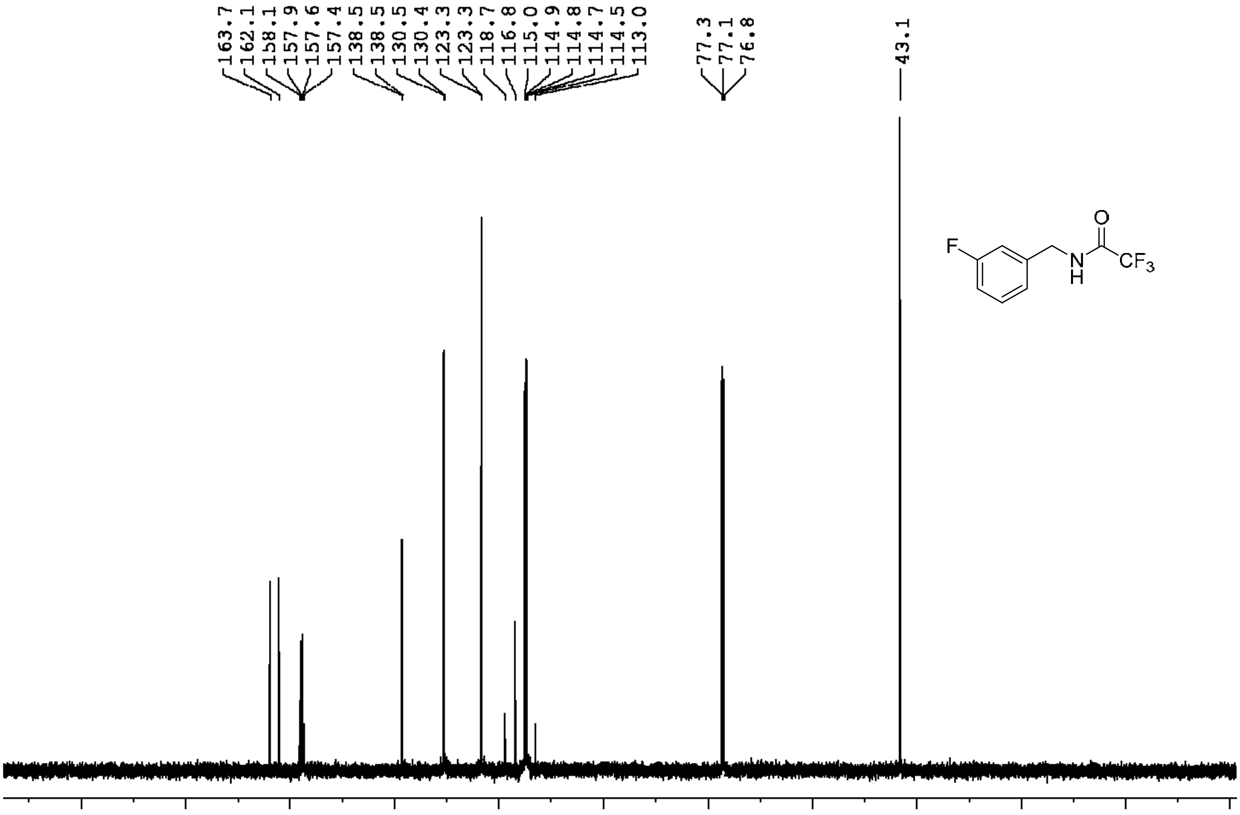

^{1}H NMR (600 MHz, $CDCl_3$) of 2,2,2-trifluoro-N-(2-(hydroxymethyl)benzyl)acetamide (2f)

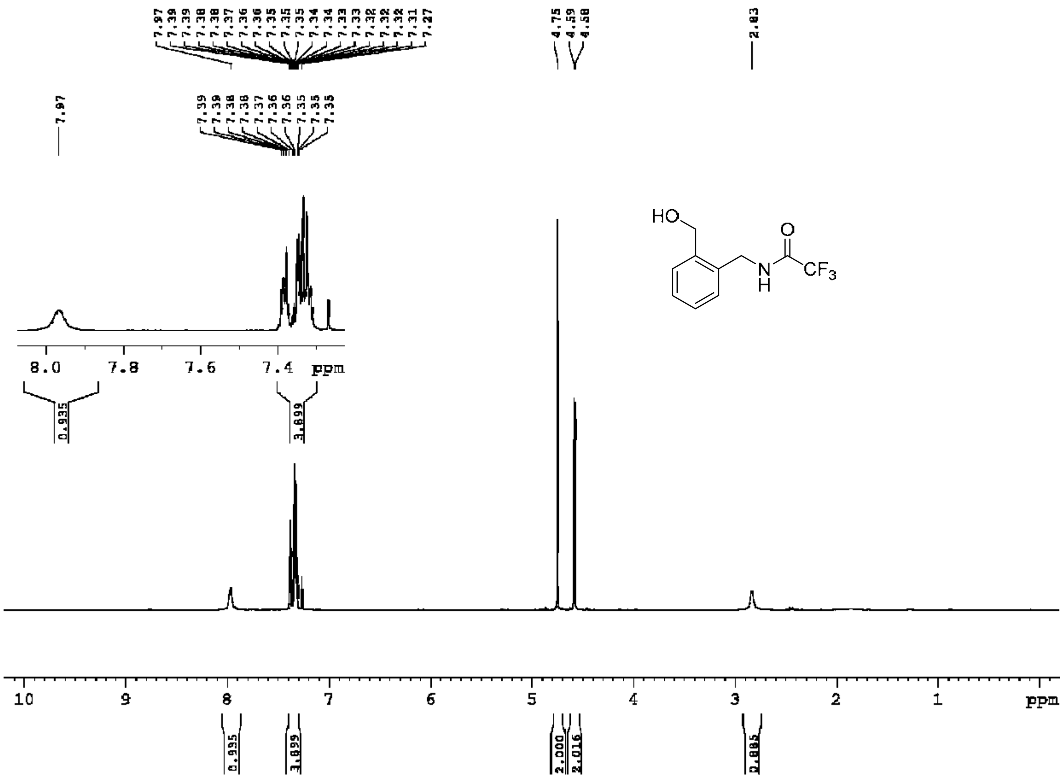

^{13}C NMR (151 MHz, $CDCl_3$) of 2,2,2-trifluoro-N-(2-(hydroxymethyl)benzyl)acetamide (2f)

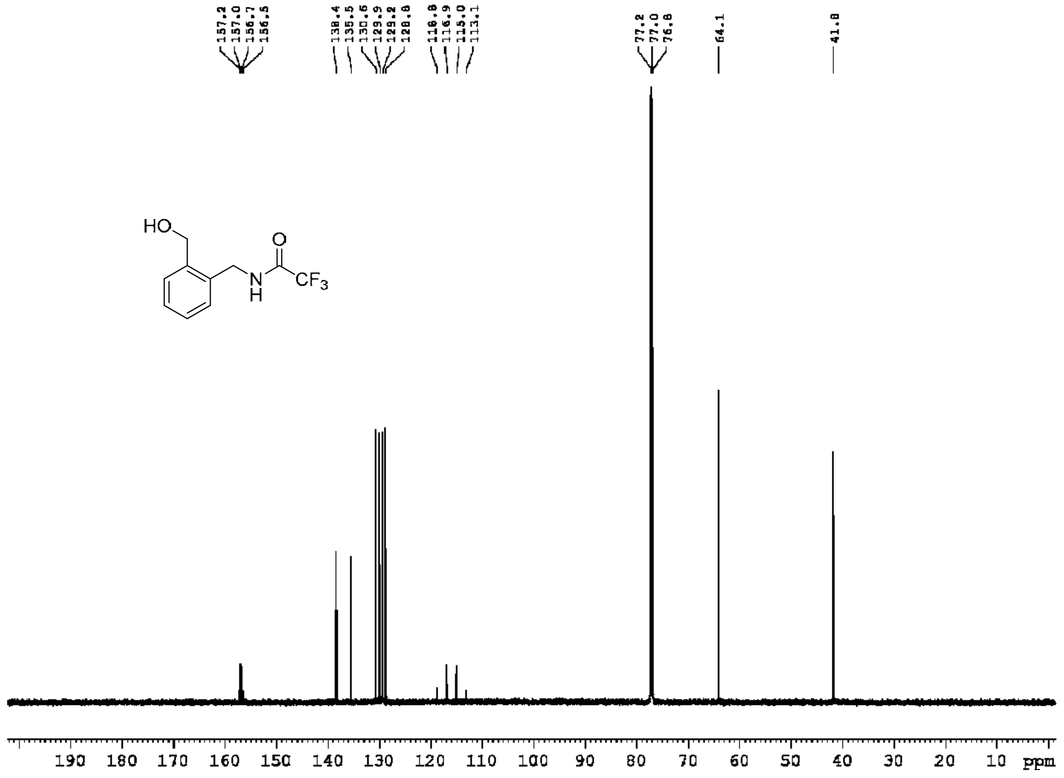

^{1}H-NMR (600 MHz, $CDCl_3$) Methyl 2-((2,2,2-trifluoroacetamido)methyl)benzoate (2g)

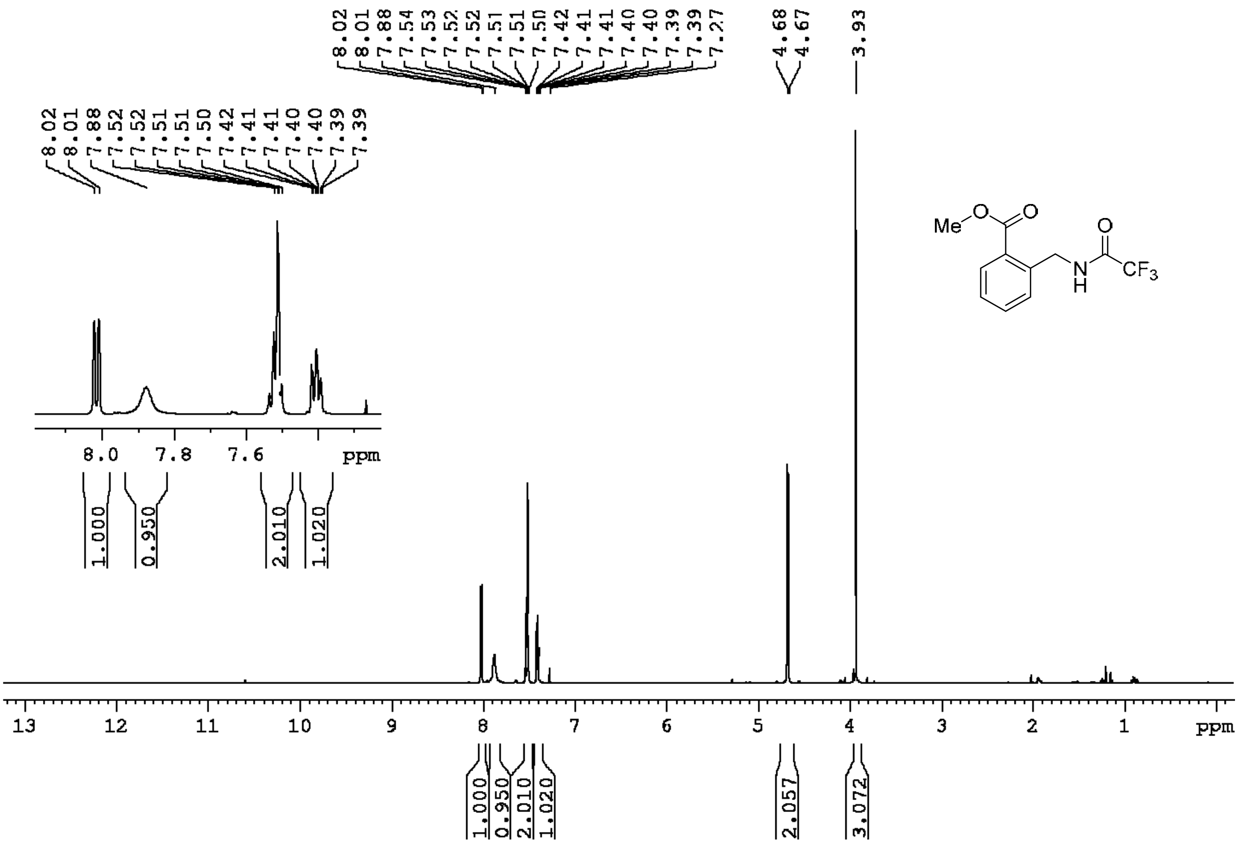

^{13}C-NMR (151 MHz, $CDCl_3$) Methyl 2-((2,2,2-trifluoroacetamido)methyl)benzoate (2g)

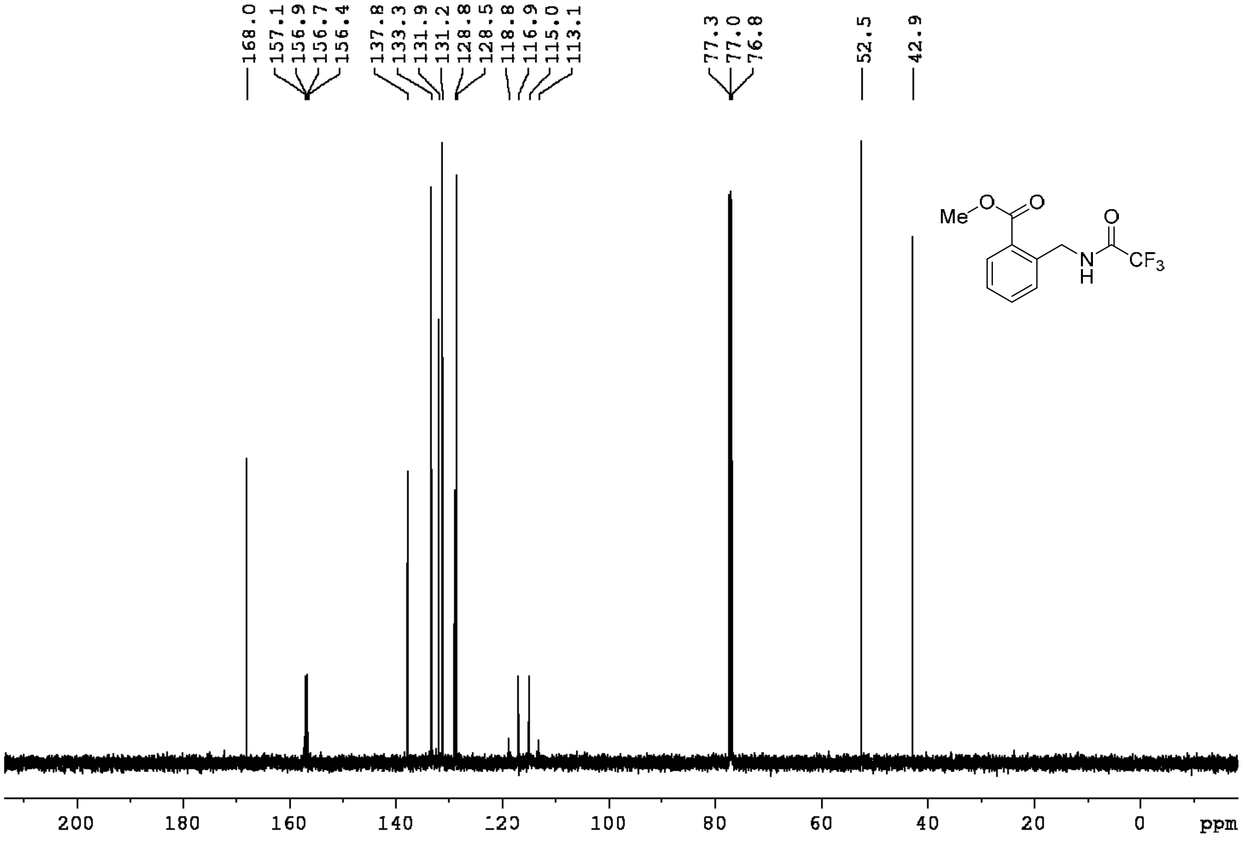

^{1}H-NMR (600 MHz, $CDCl_3$) *N*-benzyl-2,2,2-trifluoroacetamide (2h)

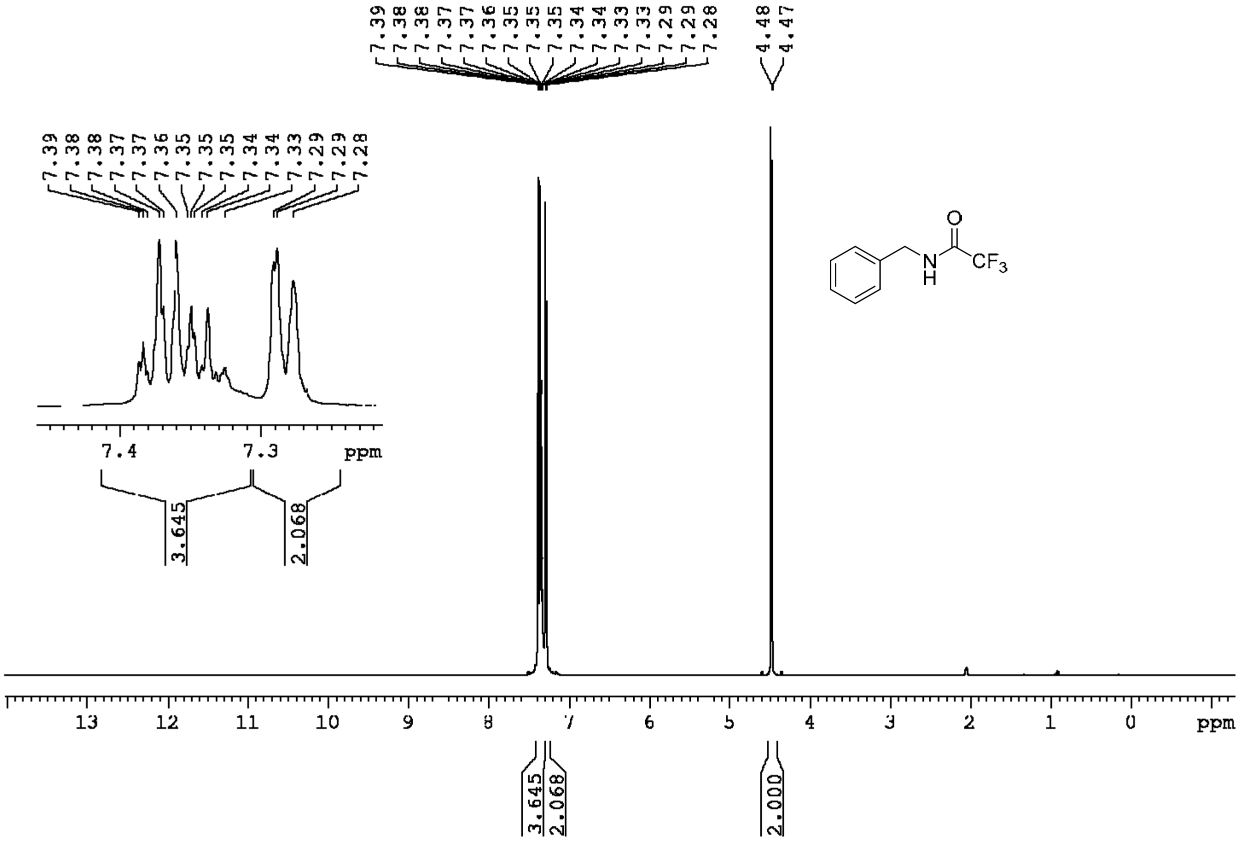

^{13}C-NMR (151 MHz, $CDCl_3$) *N*-benzyl-2,2,2-trifluoroacetamide (2h)

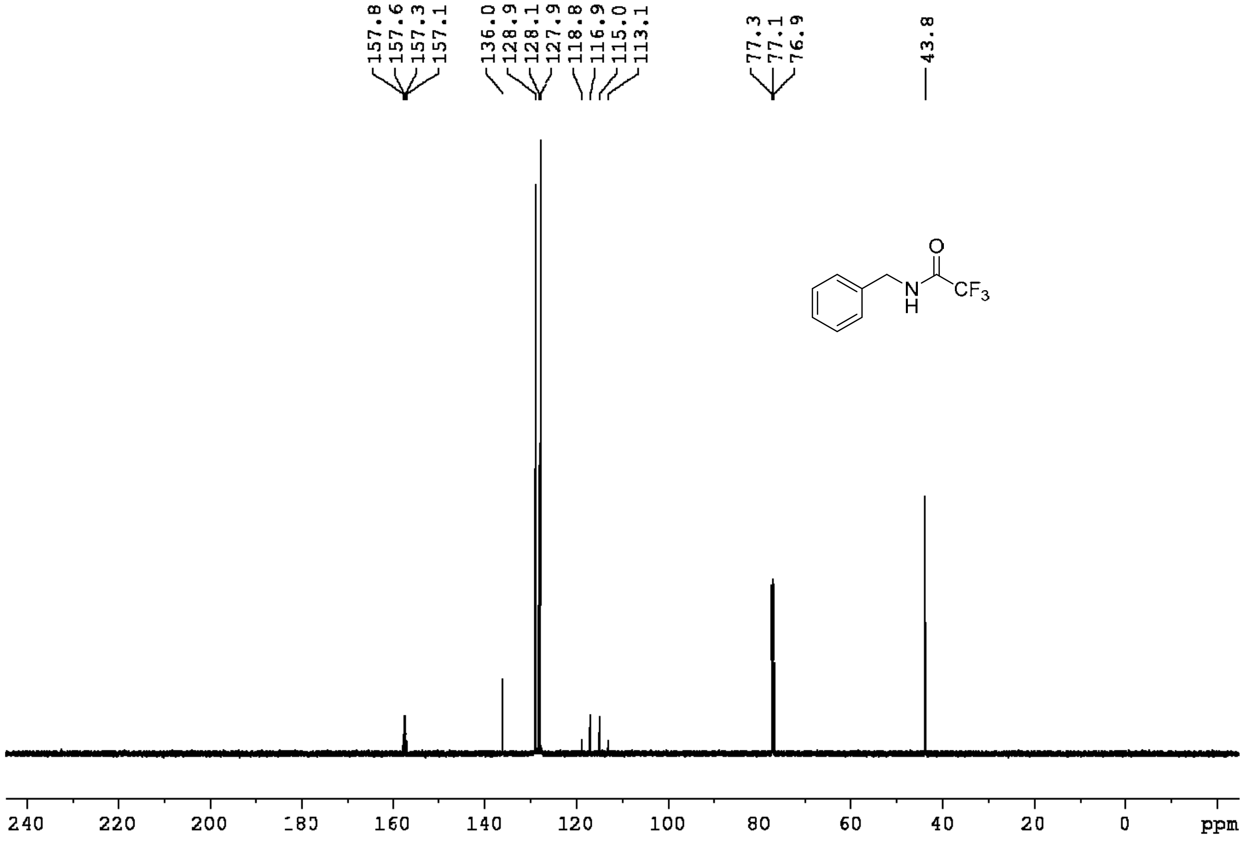

^{1}H-NMR (600 MHz, $CDCl_3$) (S)-2,2,2-Trifluoro-*N*-(1-phenylethyl)acetamide (2i)

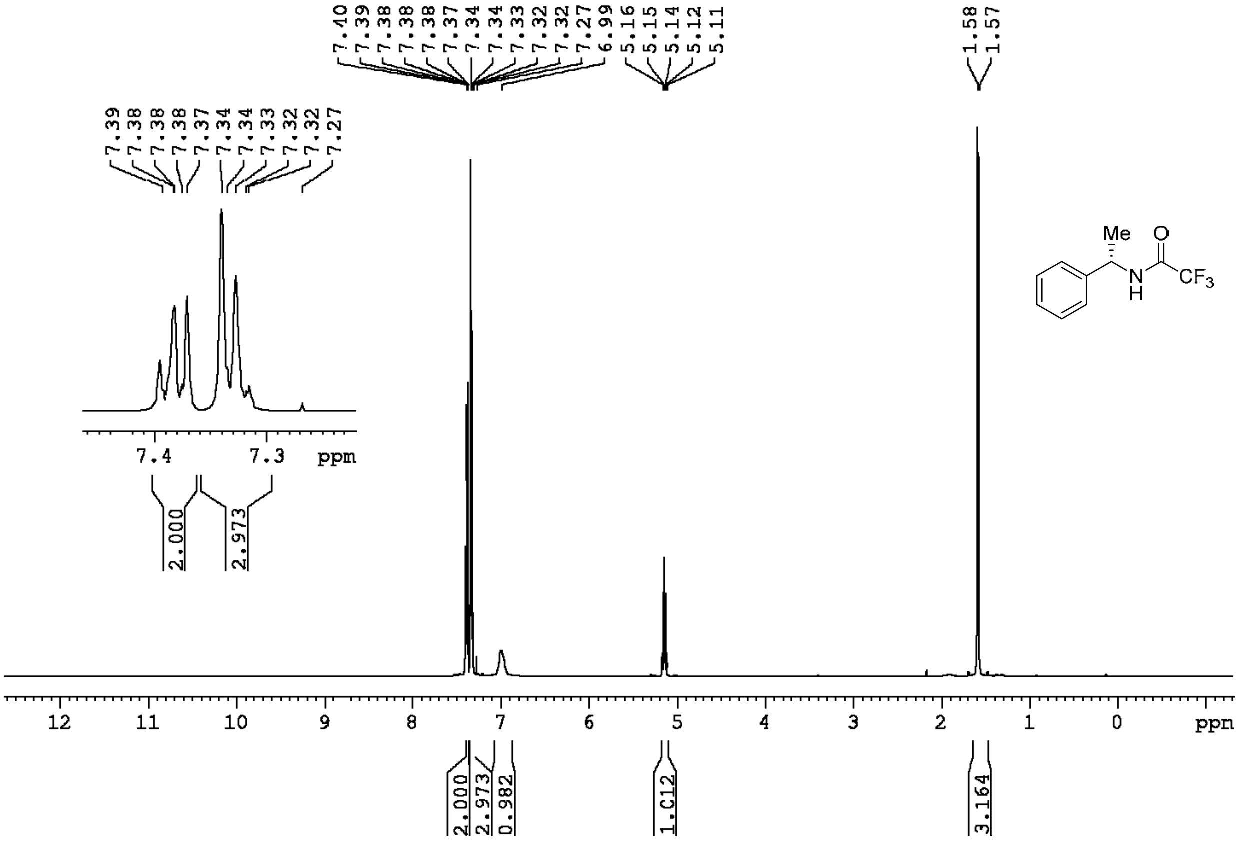

^{13}C-NMR (151 MHz, $CDCl_3$) (S)-2,2,2-Trifluoro-*N*-(1-phenylethyl)acetamide (2i)

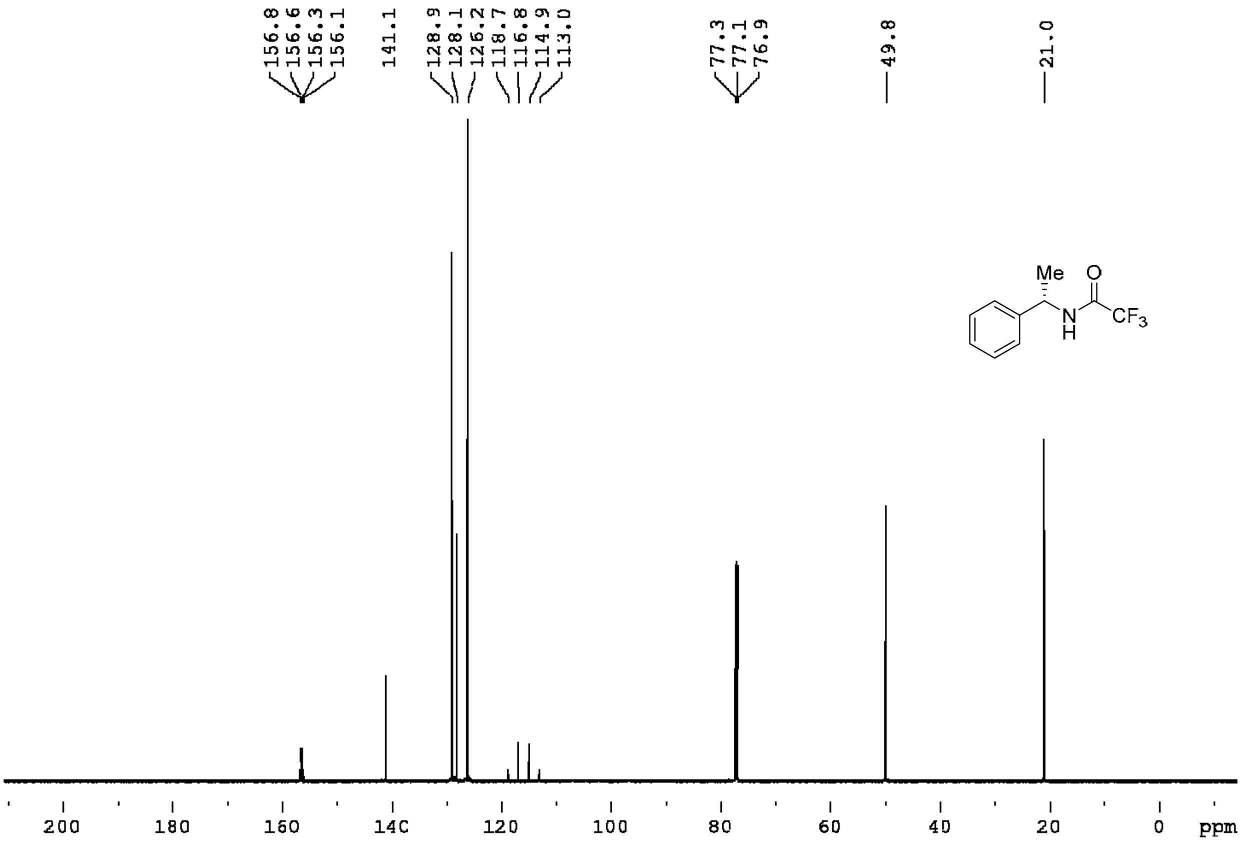

^{1}H-NMR (600 MHz, $CDCl_3$) *N*-(2-fluorobenzyl)-2,2,2-trifluoroacetamide (2j)

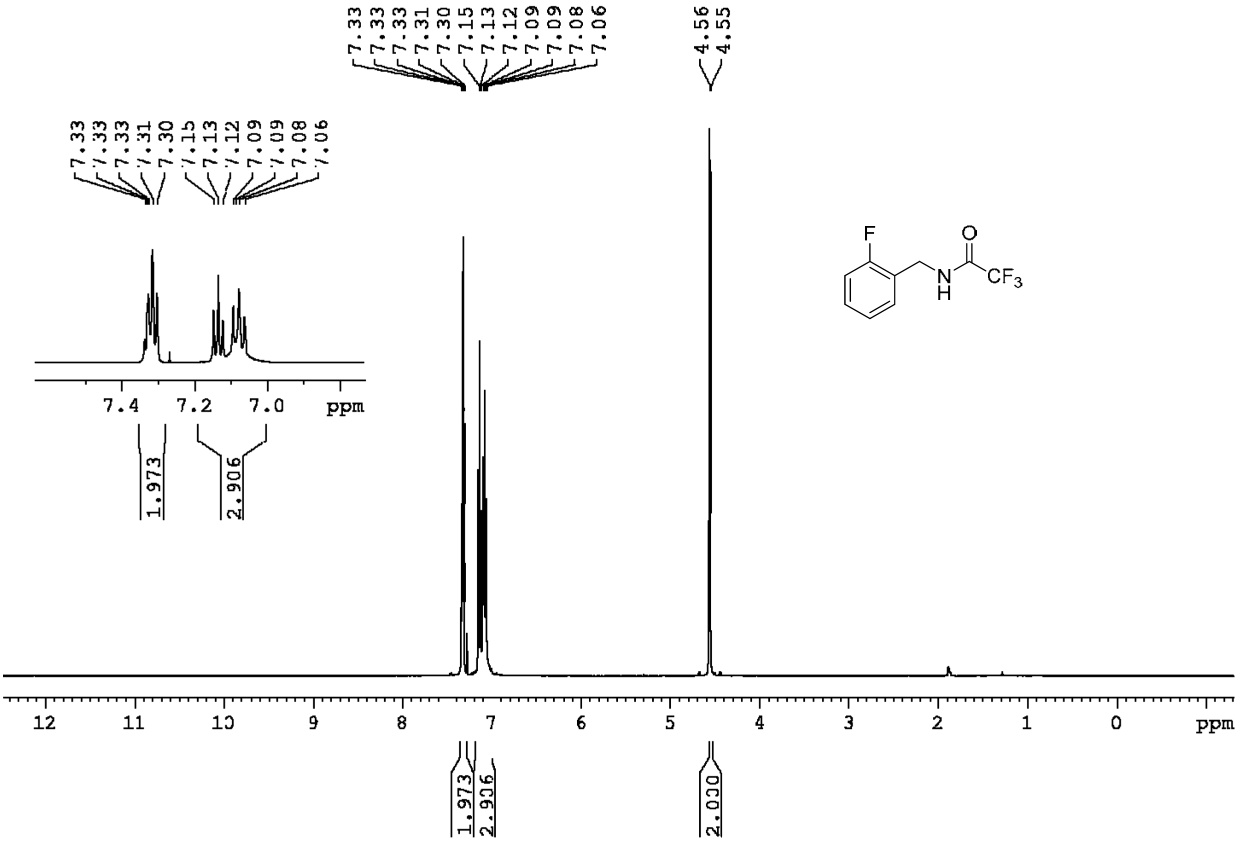

^{13}C-NMR (151 MHz, $CDCl_3$) *N*-(2-fluorobenzyl)-2,2,2-trifluoroacetamide (2j)

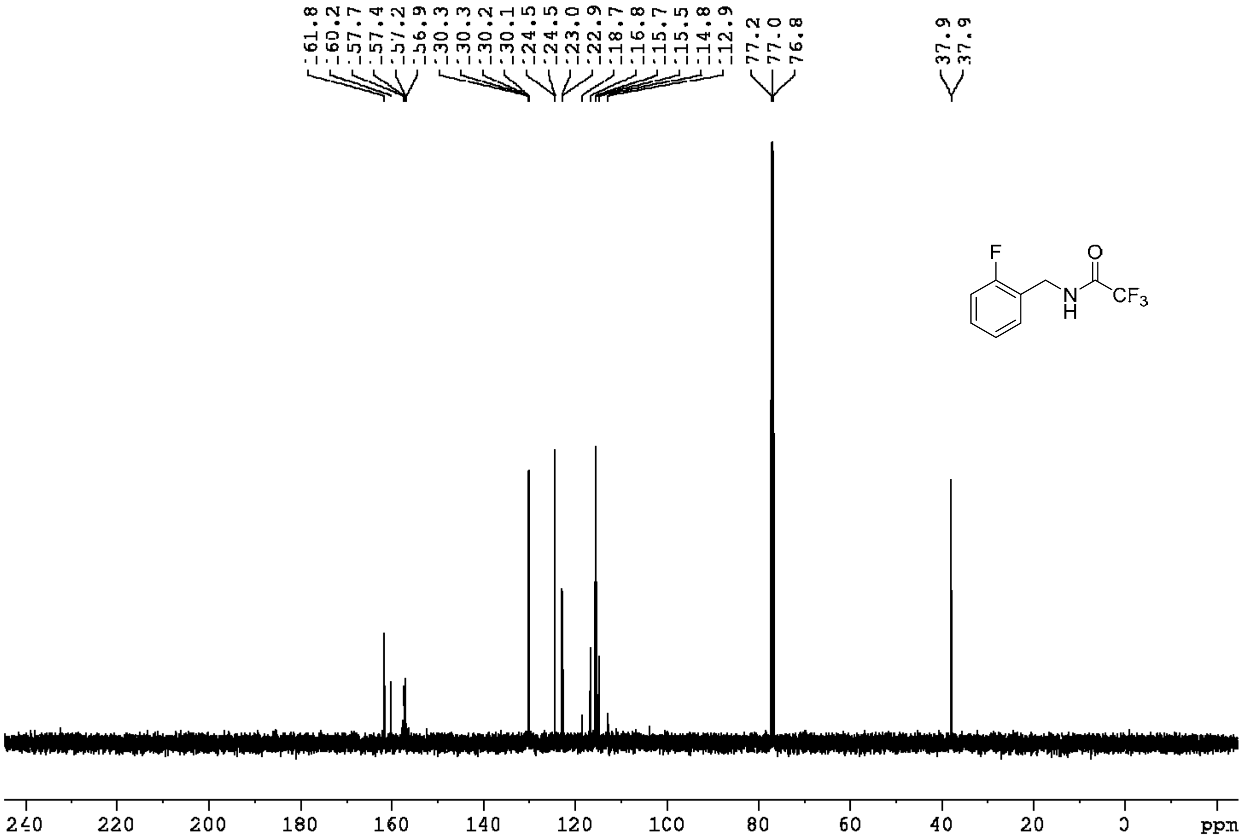

^{1}H-NMR (600 MHz, $CDCl_3$) 2,2,2-Trifluoro-*N*-(pyridin-2-ylmethyl)acetamide (2k)

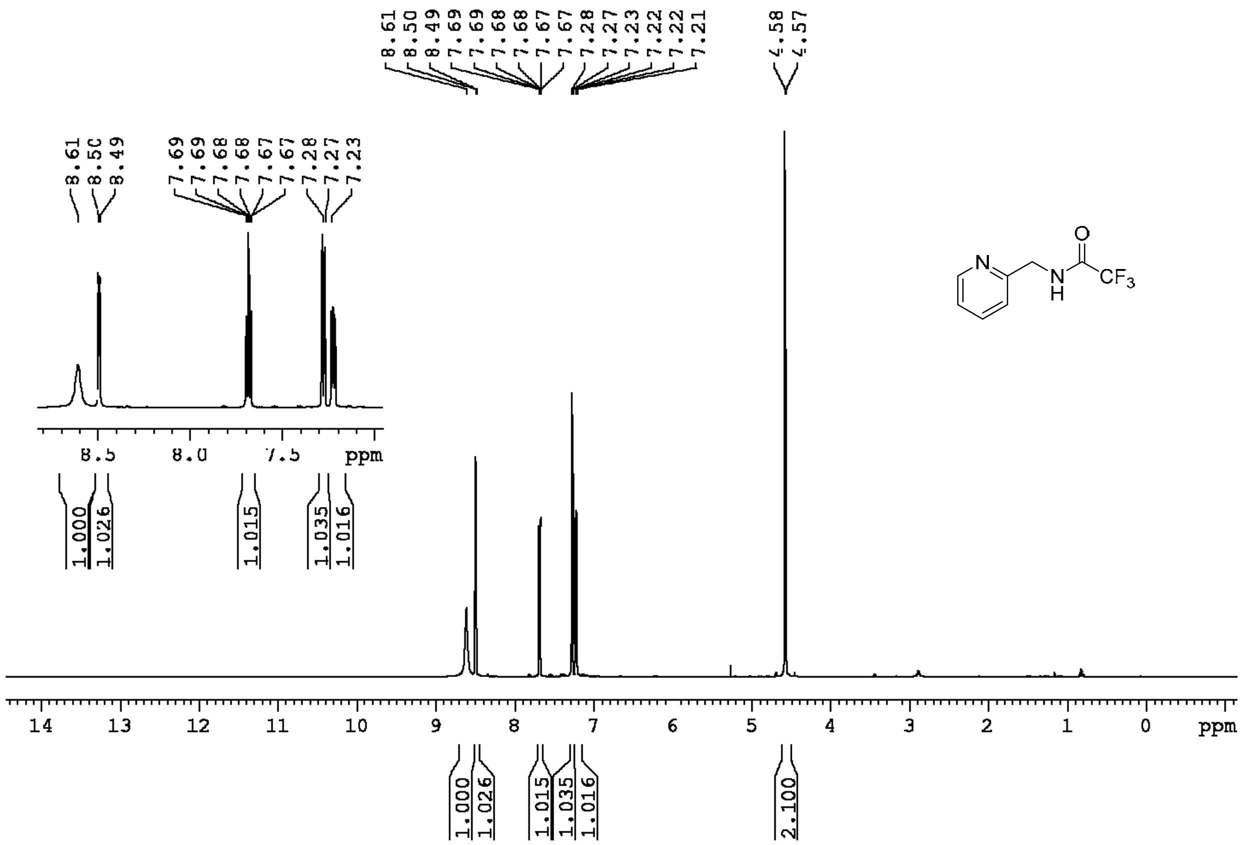

^{13}C-NMR (151 MHz, $CDCl_3$) 2,2,2-Trifluoro-*N*-(pyridin-2-ylmethyl)acetamide (2k)

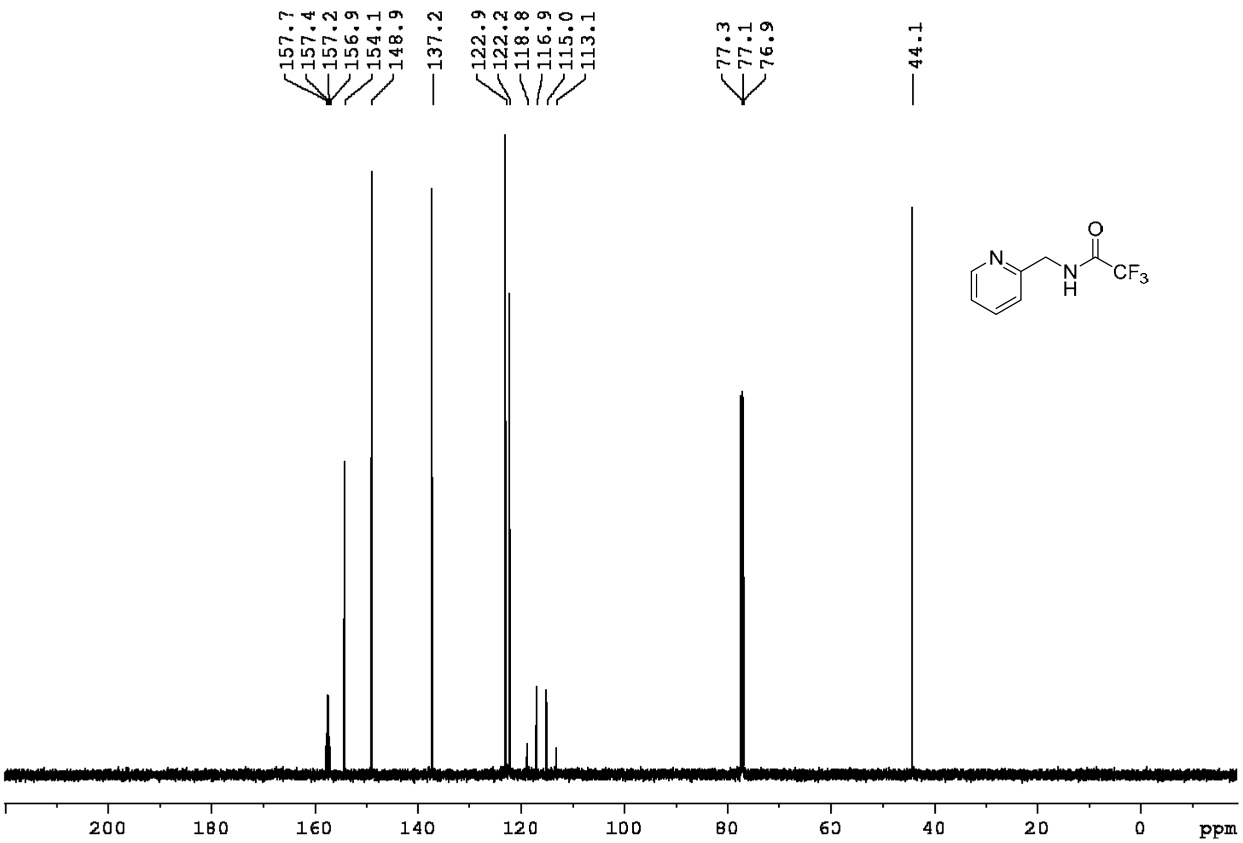

^{1}H-NMR (600 MHz, $CDCl_3$) *N*-(2-(trifluoromethyl)benzyl)methanesulfonamide (2m)

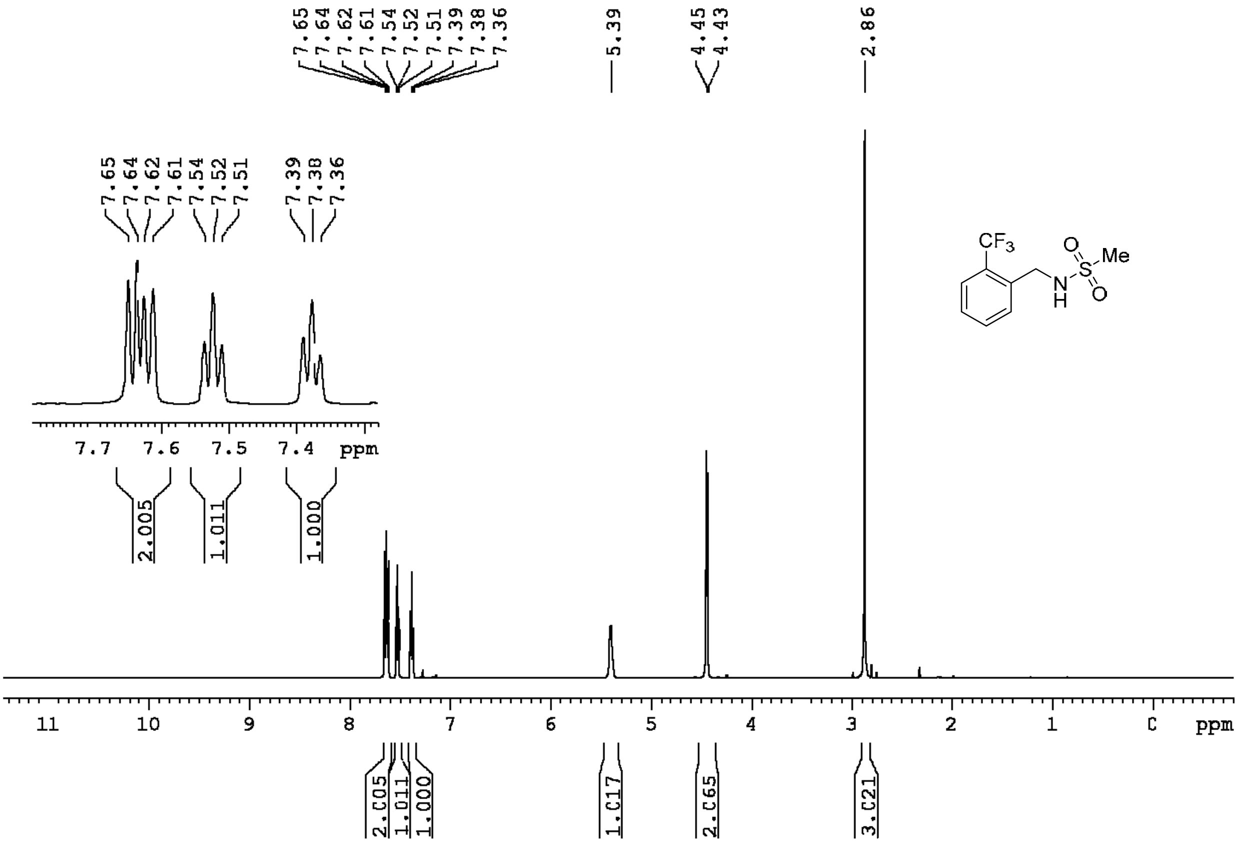

^{13}C-NMR (151 MHz, $CDCl_3$) *N*-(2-(trifluoromethyl)benzyl)methanesulfonamide (2m)

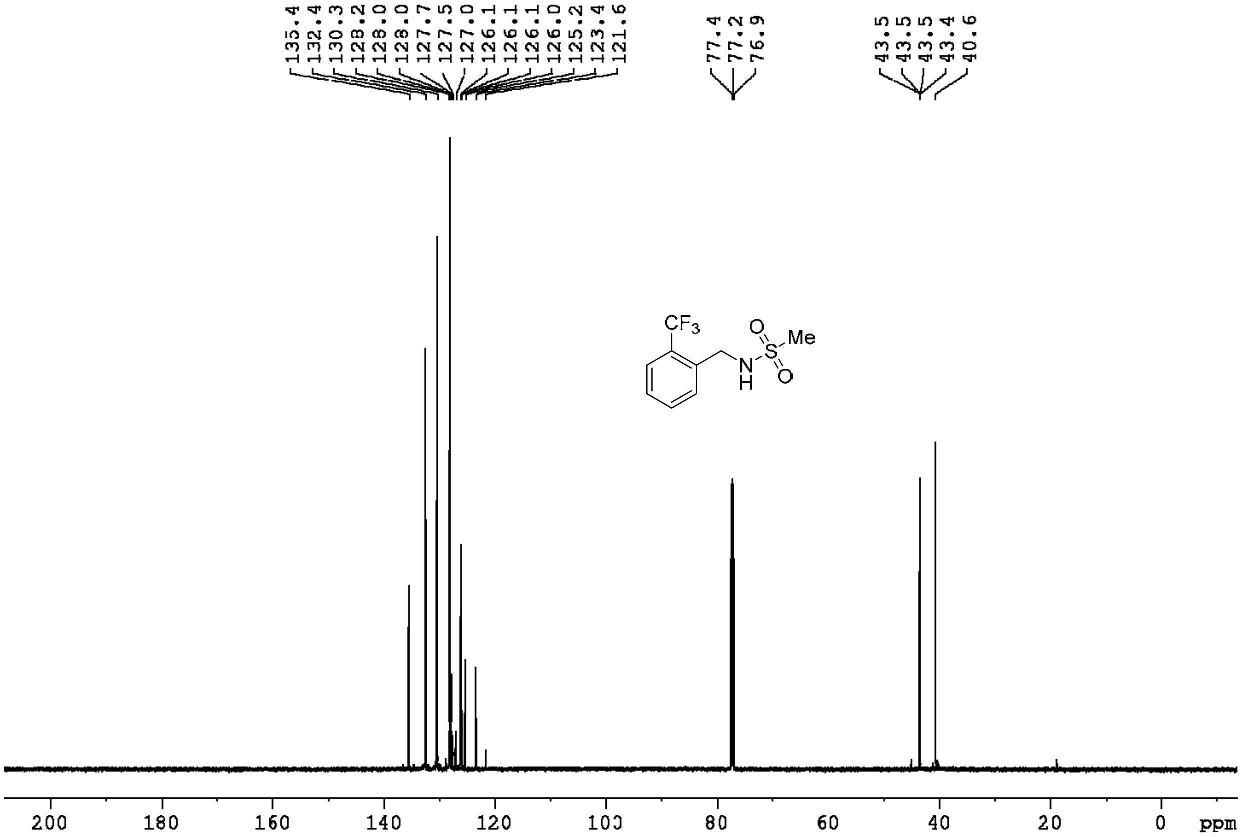

^{1}H-NMR (600 MHz, $CDCl_3$) 4-Methyl-*N*-(2-(trifluoromethyl)benzyl)benzenesulfonamide (2n)

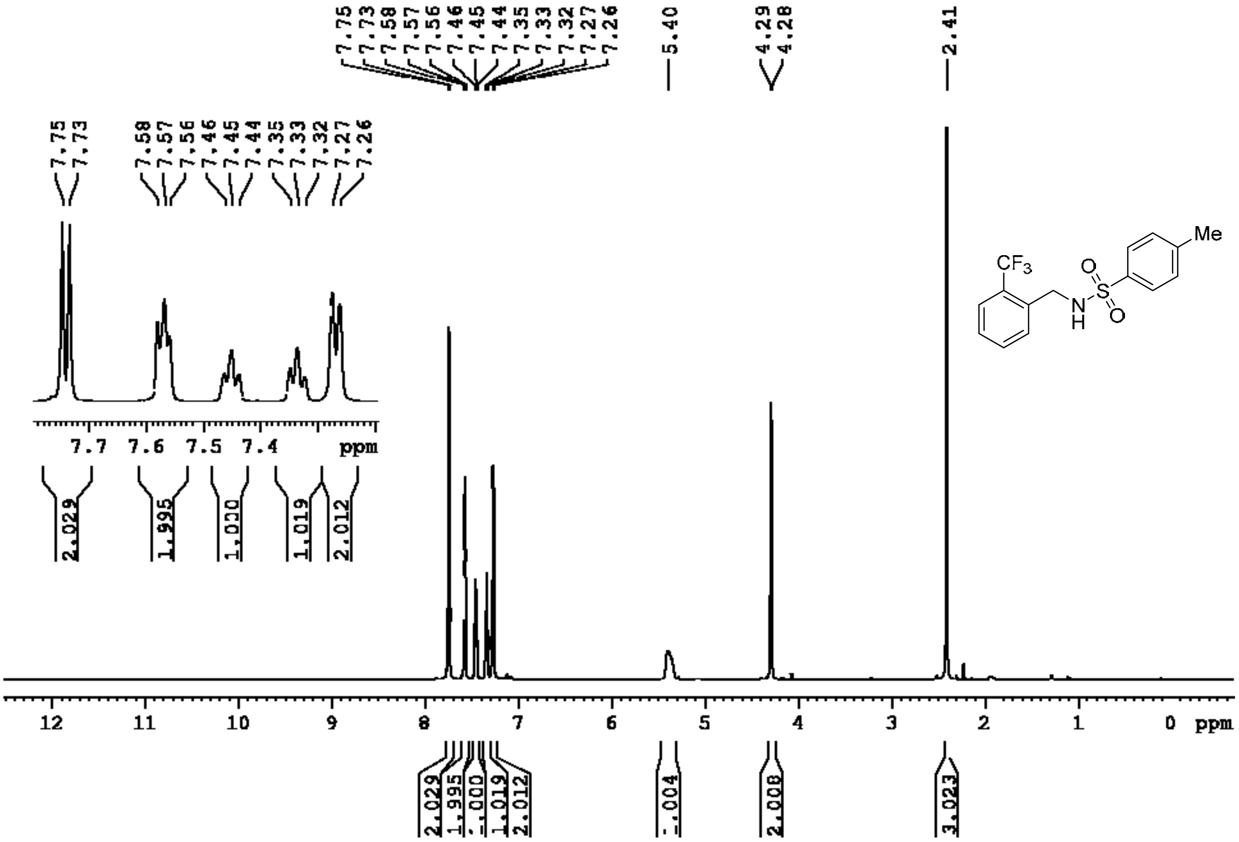

^{13}C-NMR (151 MHz, $CDCl_3$) 4-Methyl-*N*-(2-(trifluoromethyl)benzyl)benzenesulfonamide (2n)

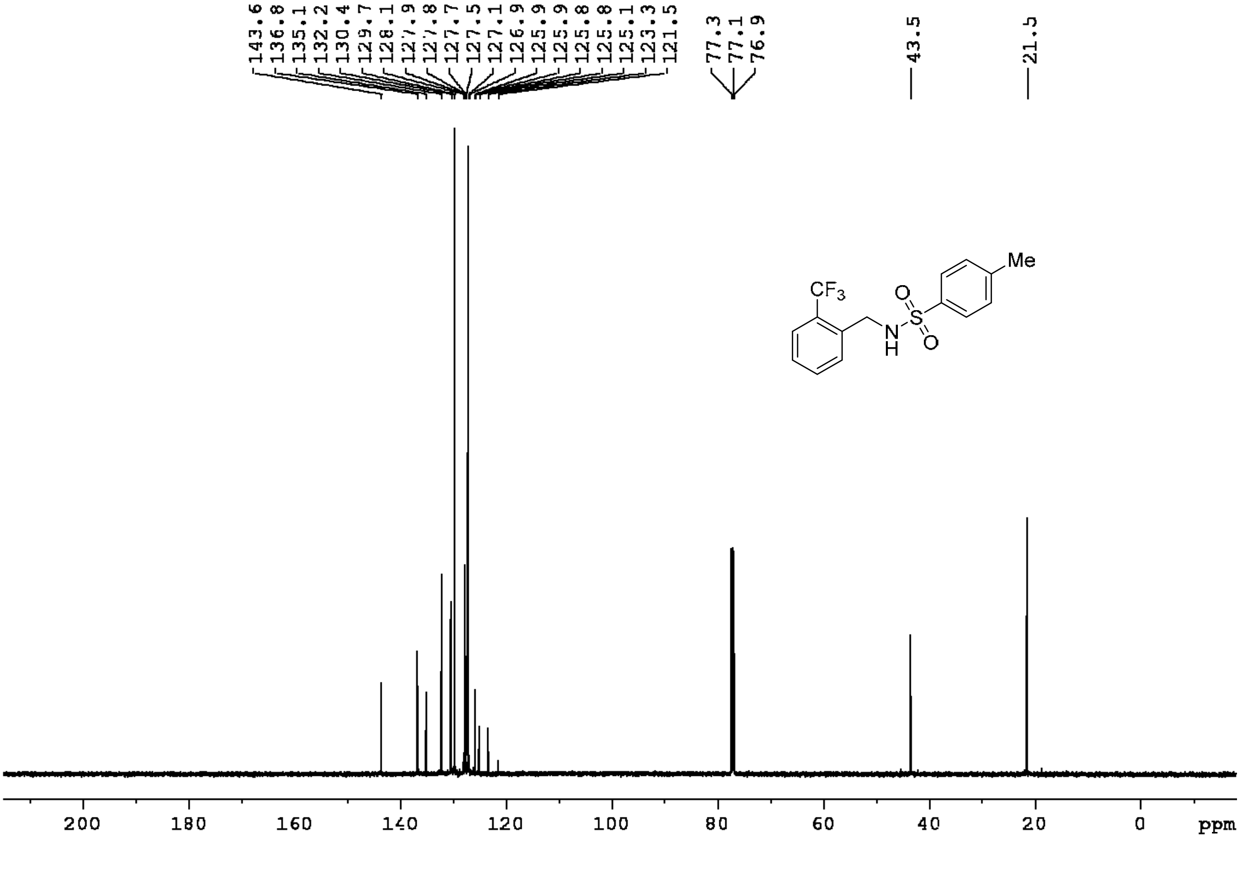

^{1}H-NMR (600 MHz, $CDCl_3$) *N*-(2-(trifluoromethyl)benzyl)acetamide (2o)

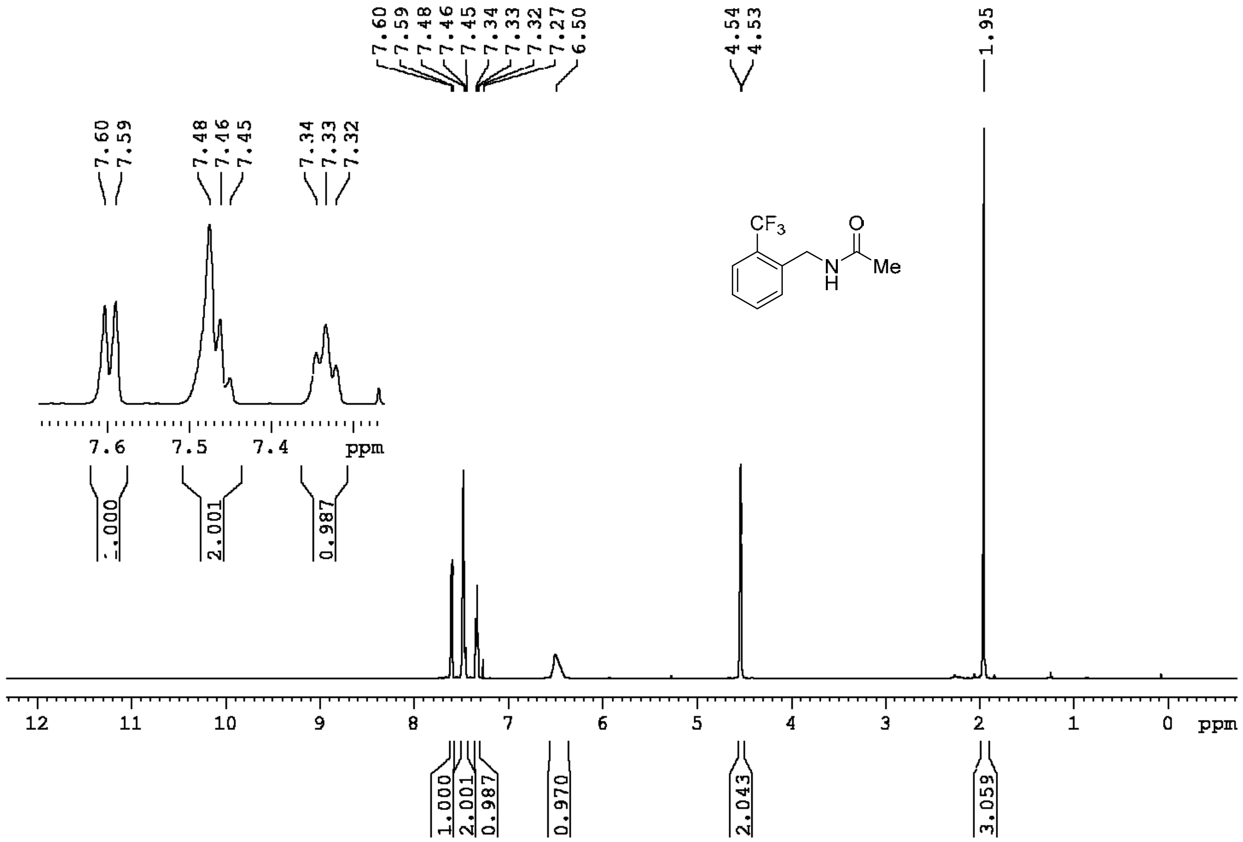

^{13}C-NMR (151 MHz, $CDCl_3$) *N*-(2-(trifluoromethyl)benzyl)acetamide (2o)

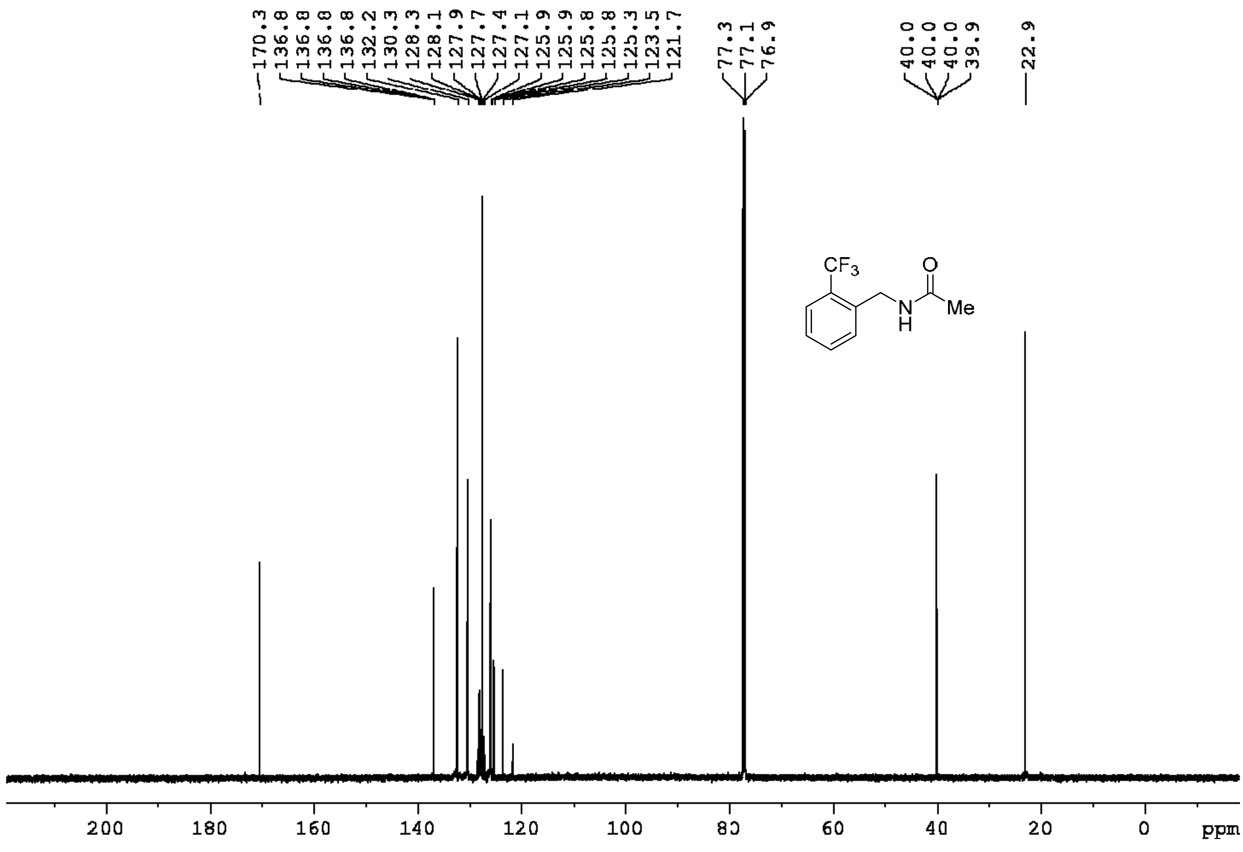

^{1}H-NMR (600 MHz, CDCl$_3$) *tert*-Butyl (2-(trifluoromethyl)benzyl)carbamate (2p)

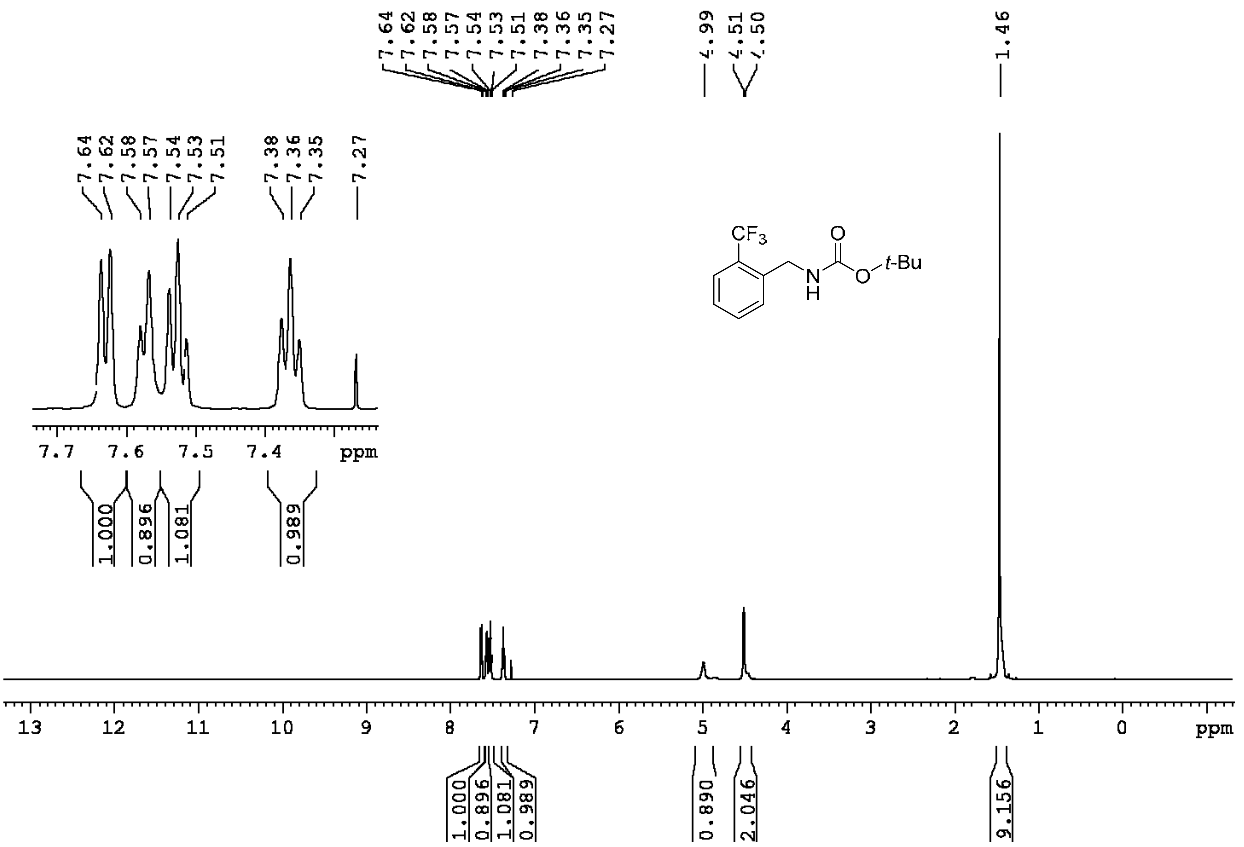

^{13}C-NMR (151 MHz, CDCl$_3$) *tert*-Butyl (2-(trifluoromethyl)benzyl)carbamate (2p)

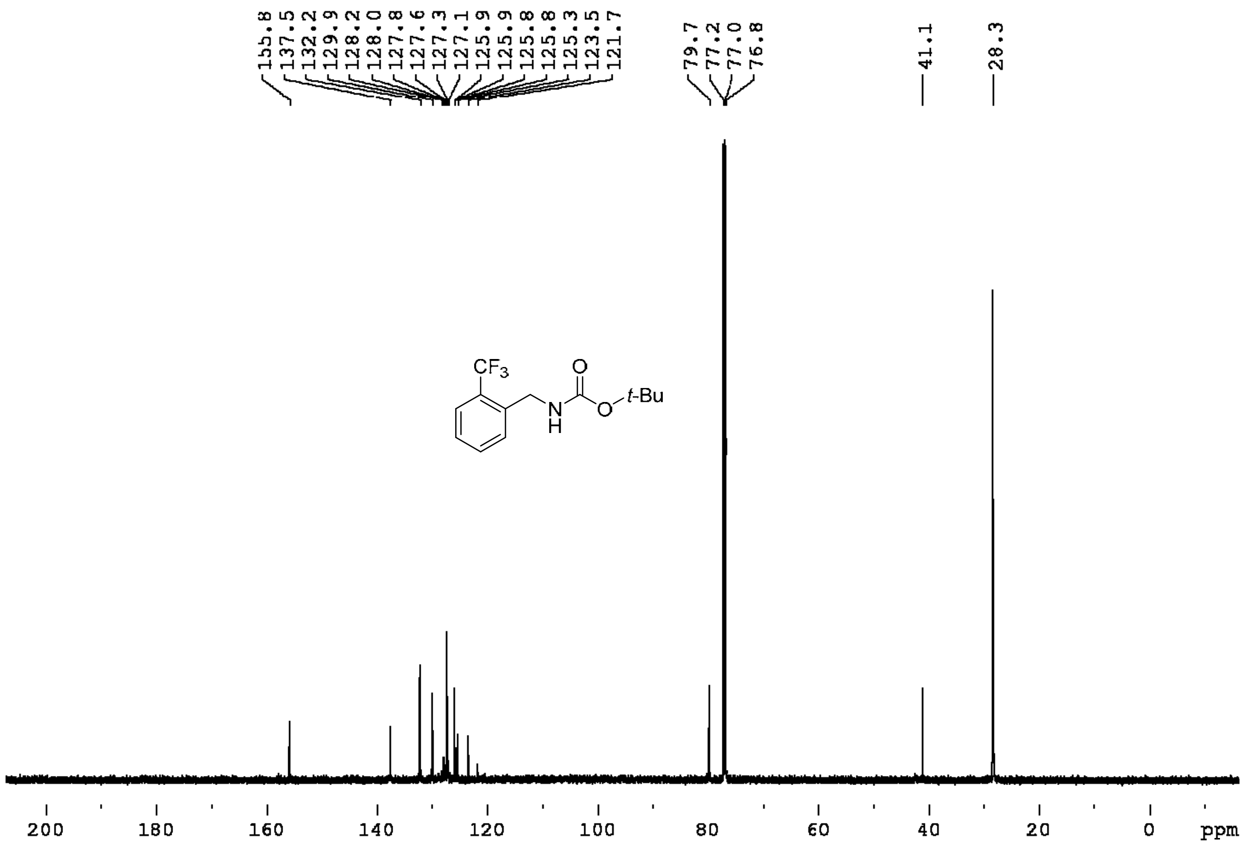

^{1}H-NMR (600 MHz, $CDCl_3$) *N*-(2-(trifluoromethyl)benzyl)acetamide (2q)

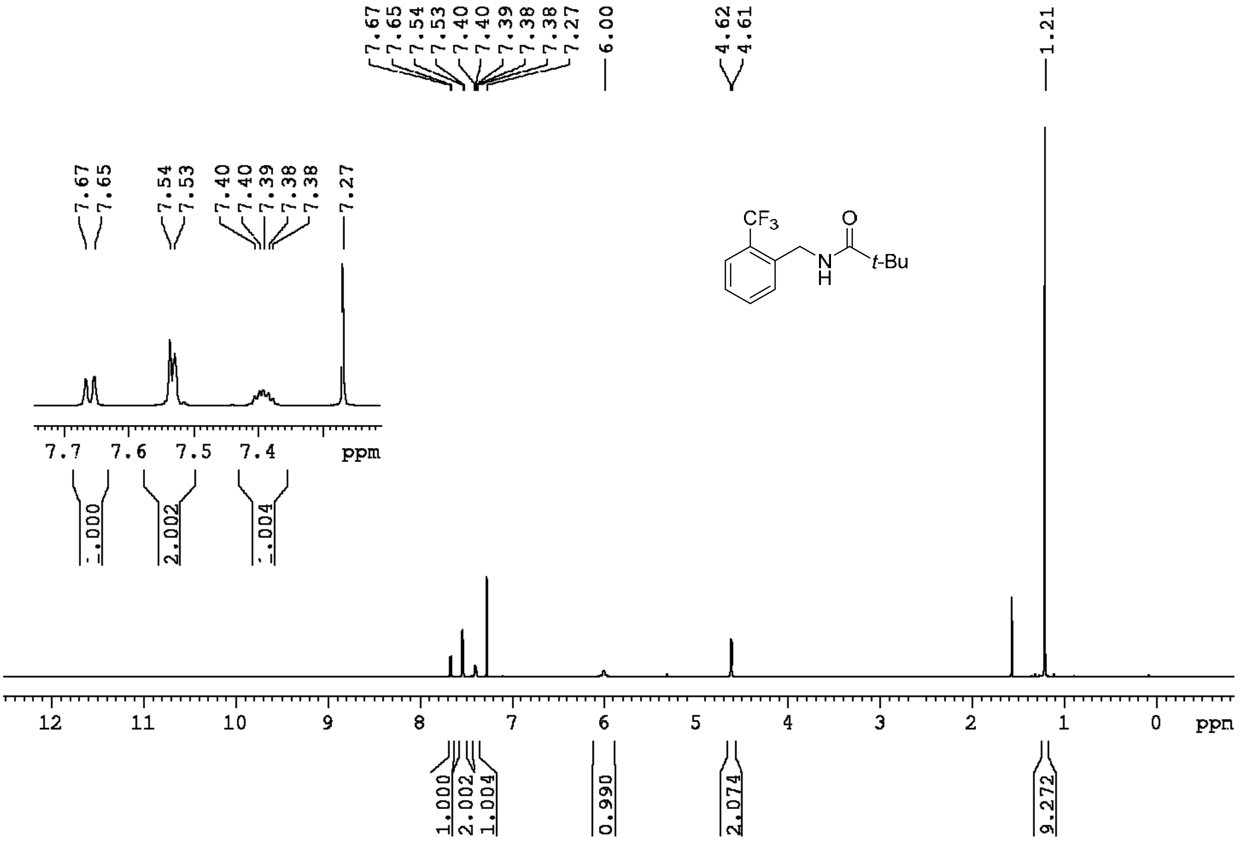

^{13}C-NMR (151 MHz, $CDCl_3$) *N*-(2-(trifluoromethyl)benzyl)acetamide (2q)

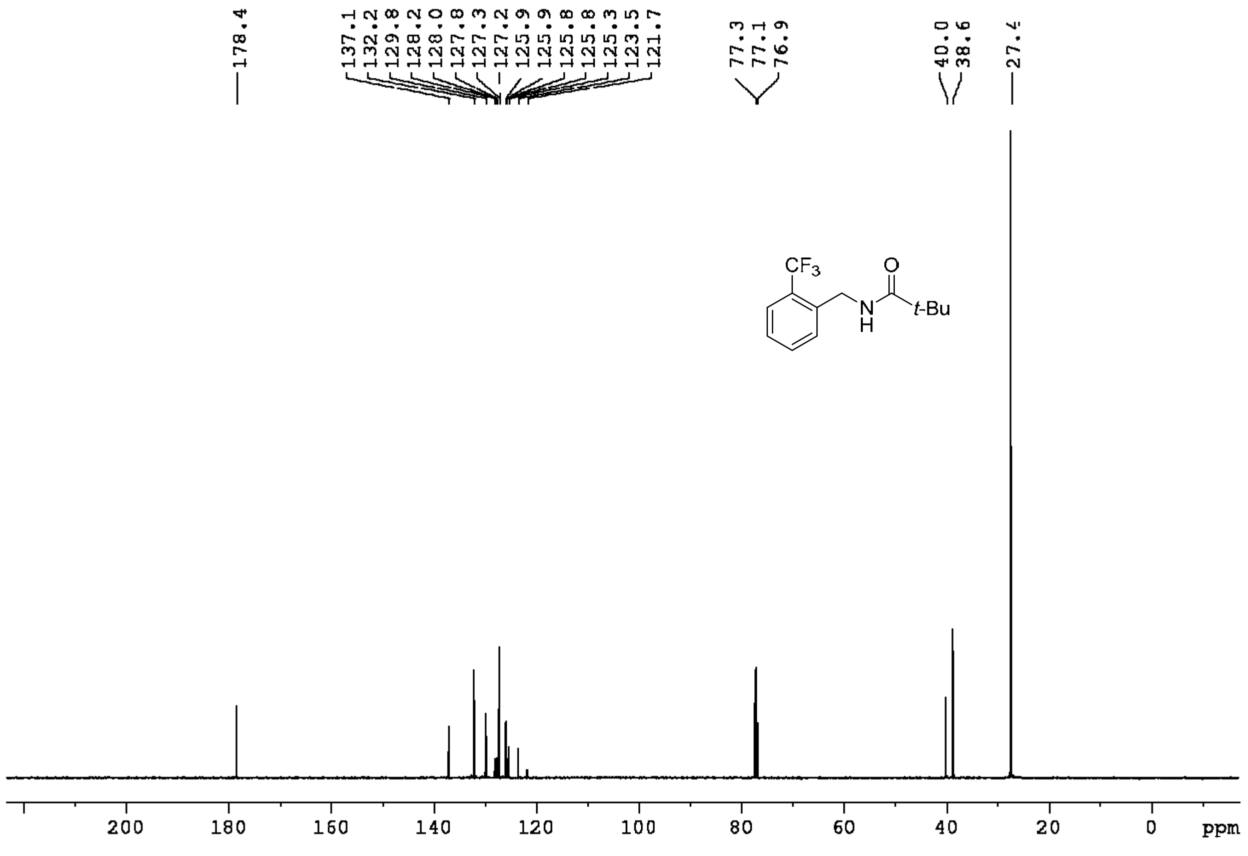

^{1}H NMR (600 MHz, $CDCl_3$) of (E)-1-(2-nitrovinyl)-2-(trifluoromethyl)benzene

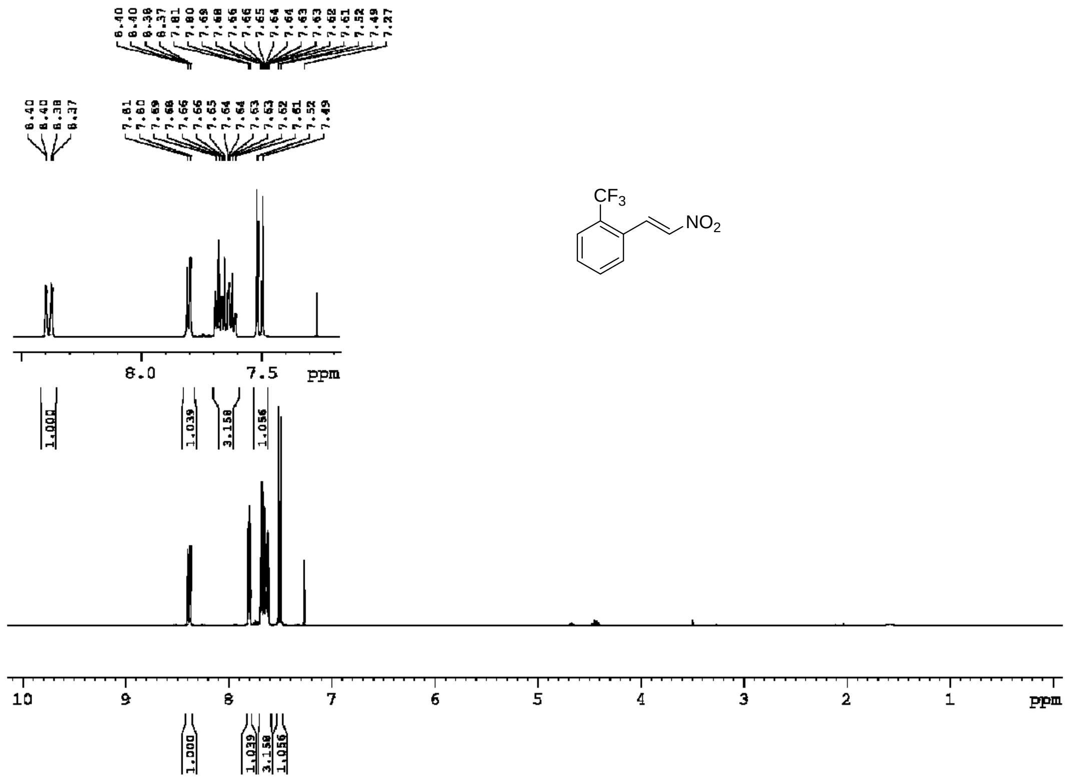

^{13}C NMR (151 MHz, $CDCl_3$) of (E)-1-(2-nitrovinyl)-2-(trifluoromethyl)benzene

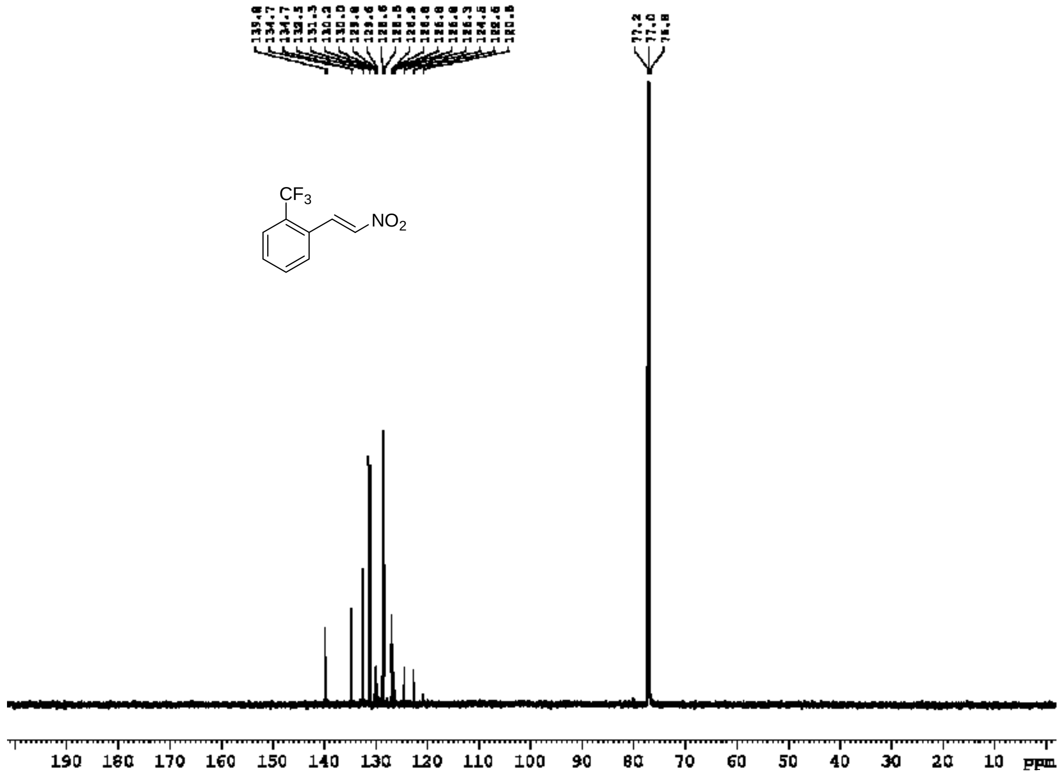

^{1}H NMR (600 MHz, $CDCl_3$) of 2,2,2-trifluoro-N-(2-(trifluoromethyl)phenethyl)acetamide (4a)

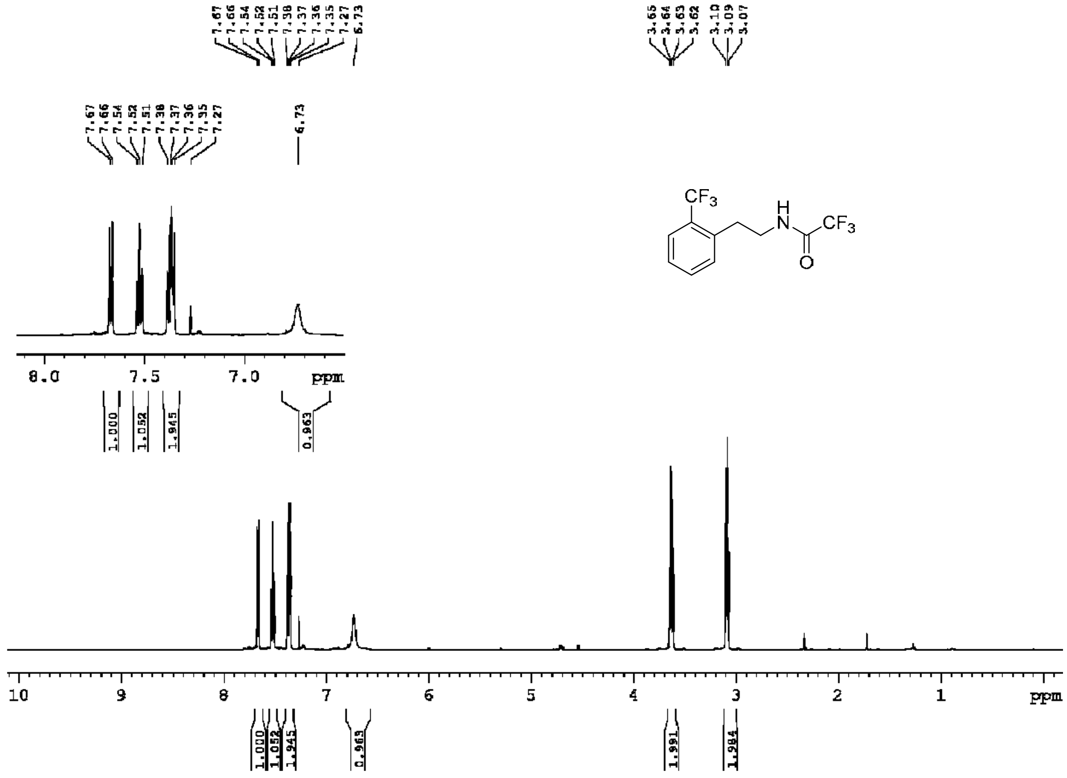

^{13}C NMR (151 MHz, $CDCl_3$) of 2,2,2-trifluoro-N-(2-(trifluoromethyl)phenethyl)acetamide (4a)

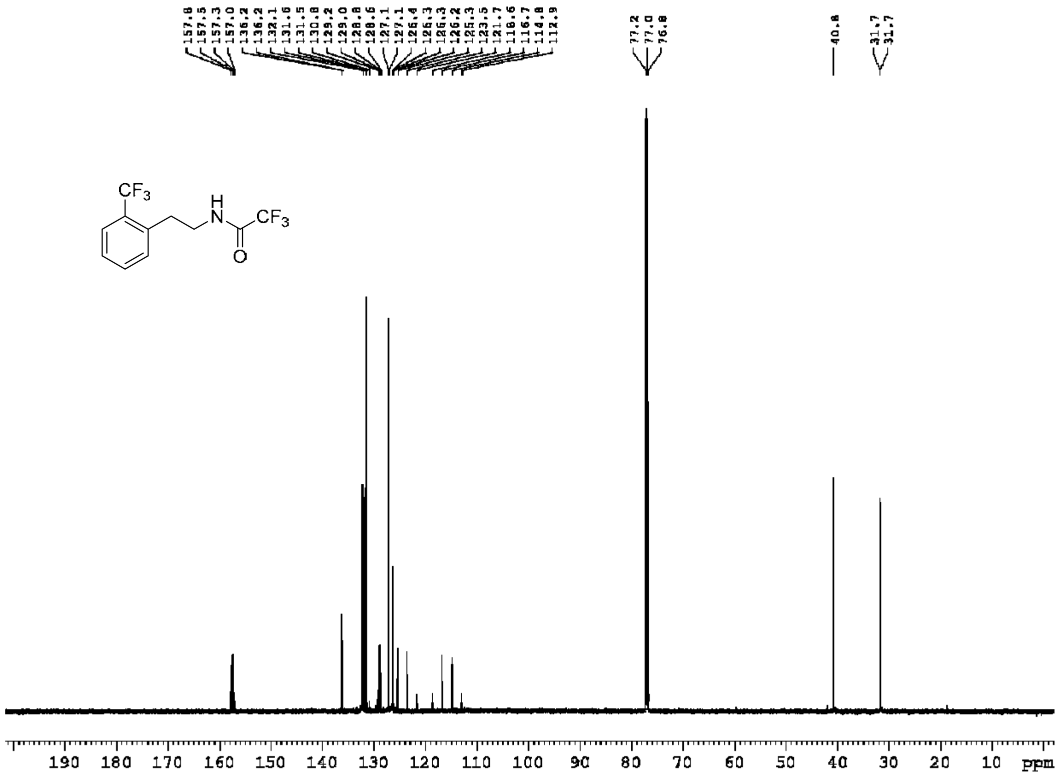

^{1}H NMR (600 MHz, $CDCl_3$) of N-(2-(trifluoromethyl)phenethyl)methanesulfonamide (4b)

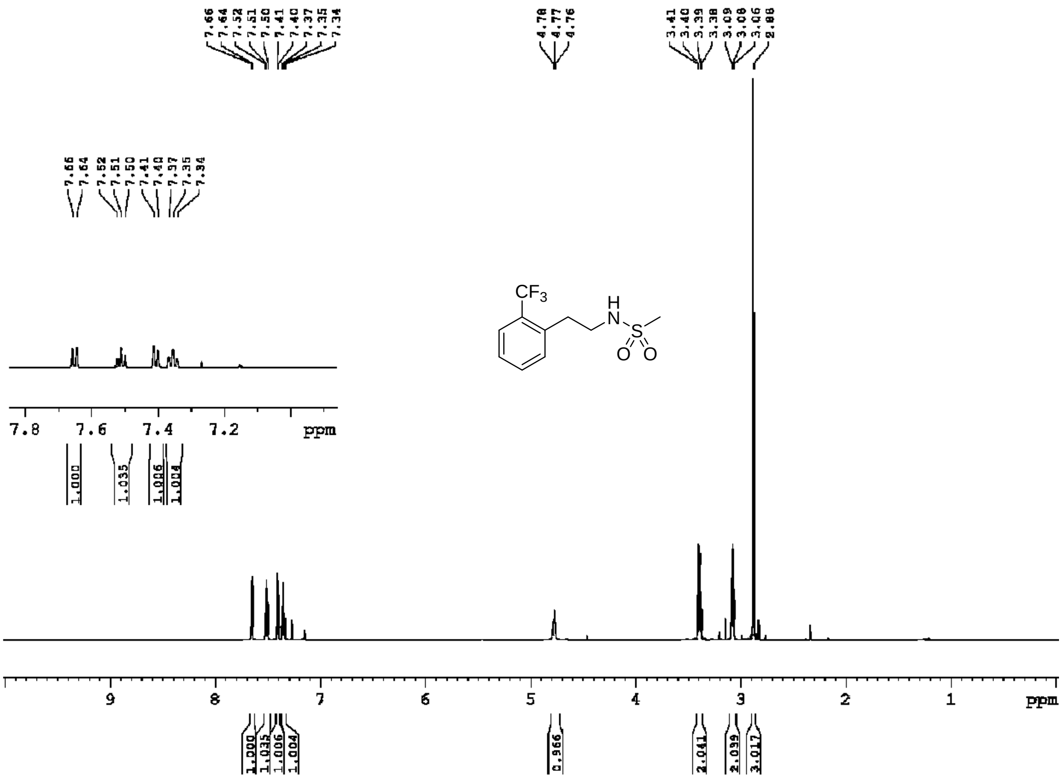

^{13}C NMR (151 MHz, $CDCl_3$) of N-(2-(trifluoromethyl)phenethyl)methanesulfonamide (4b)

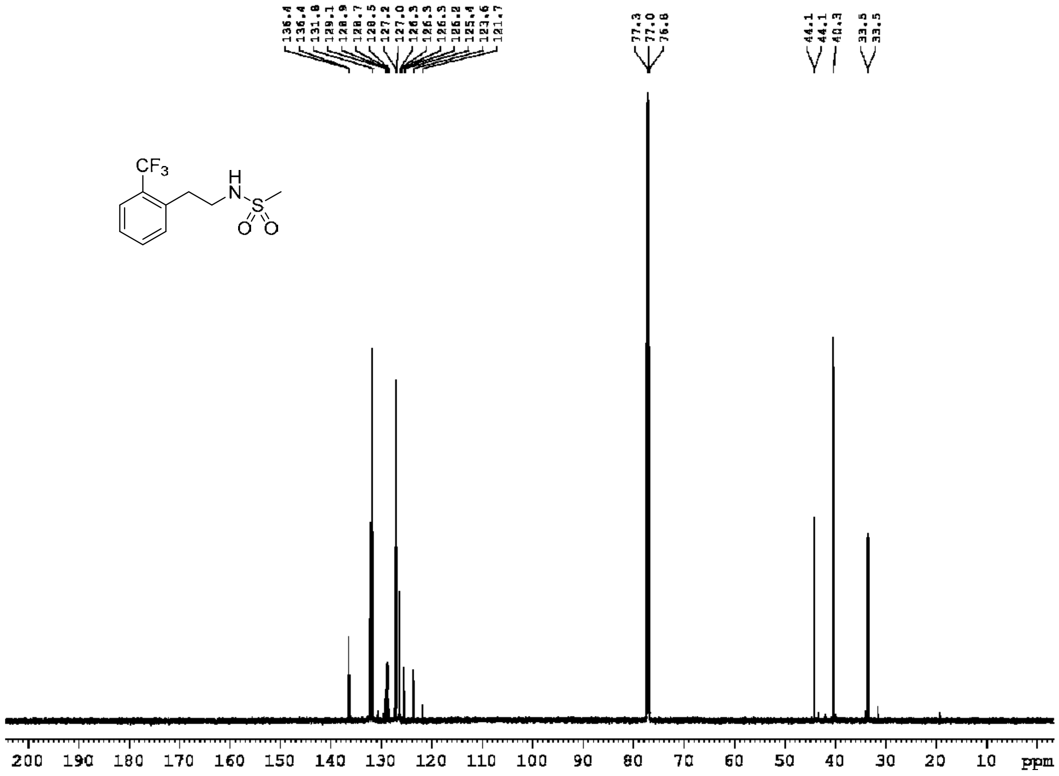

^{1}H NMR (600 MHz, $CDCl_3$) of tert-butyl (2-(trifluoromethyl)phenethyl)carbamate (4c)

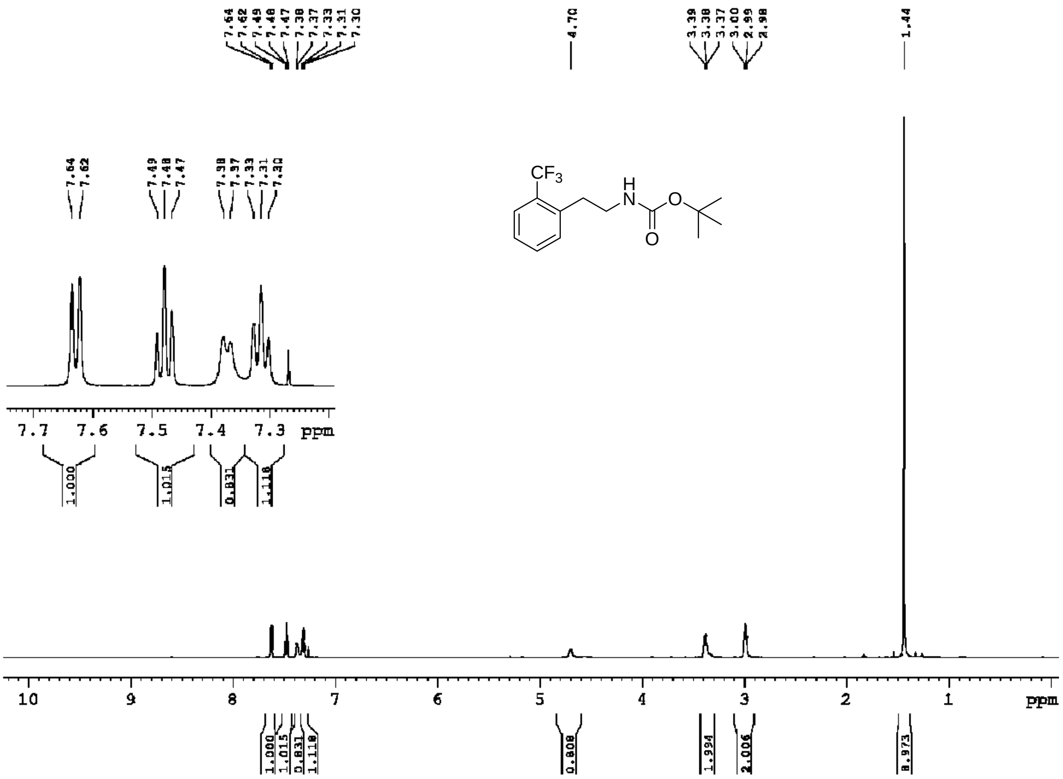

^{13}C NMR (151 MHz, $CDCl_3$) of tert-butyl (2-(trifluoromethyl)phenethyl)carbamate (4c)

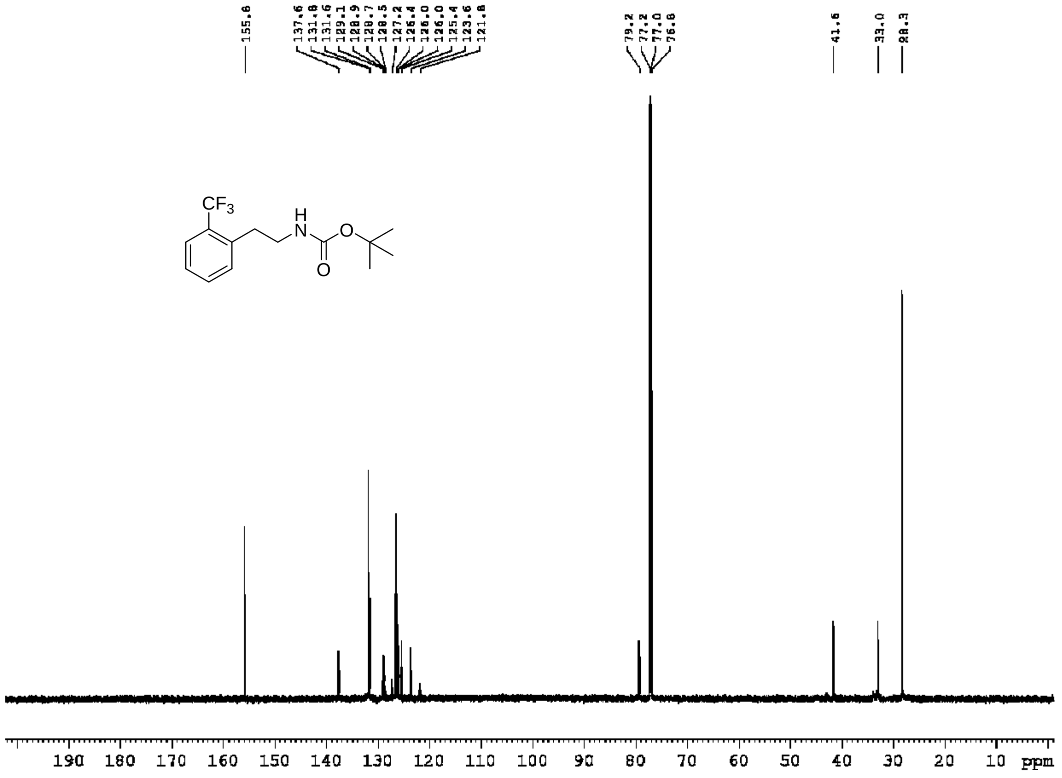

^{1}H NMR (600 MHz, $CDCl_3$) of 2,2,2-trifluoro-N-(2-iodophenethyl)acetamide (4d)

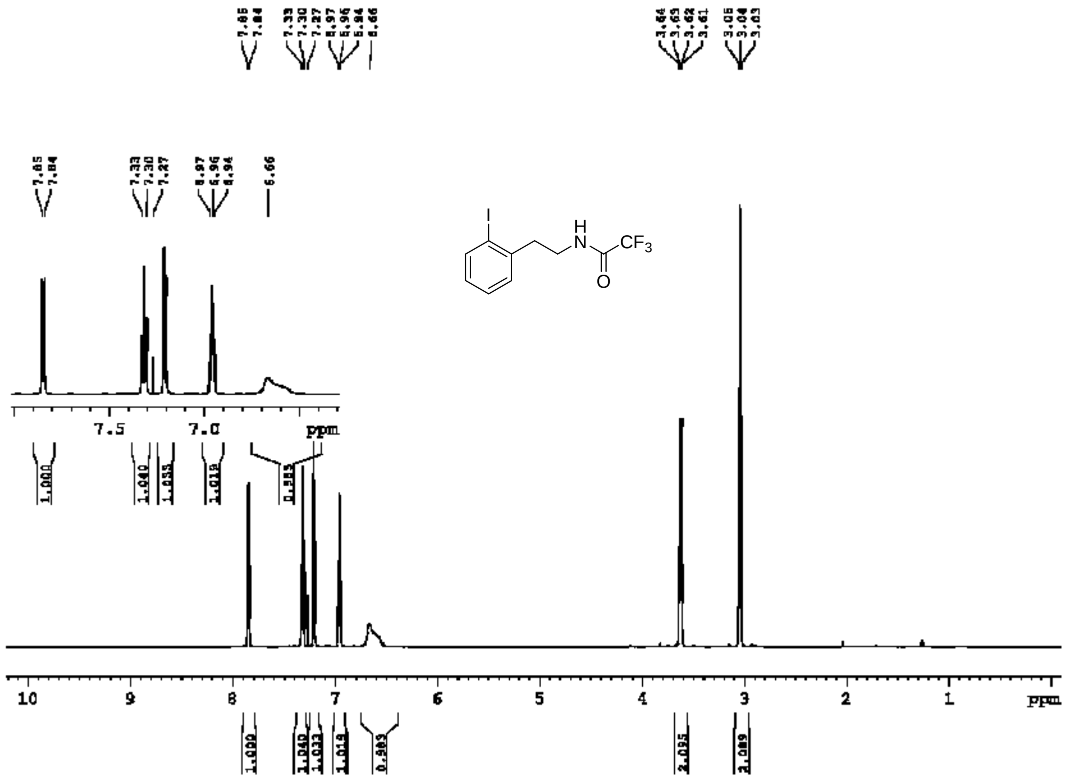

^{13}C NMR (151 MHz, $CDCl_3$) of 2,2,2-trifluoro-N-(2-iodophenethyl)acetamide (4d)

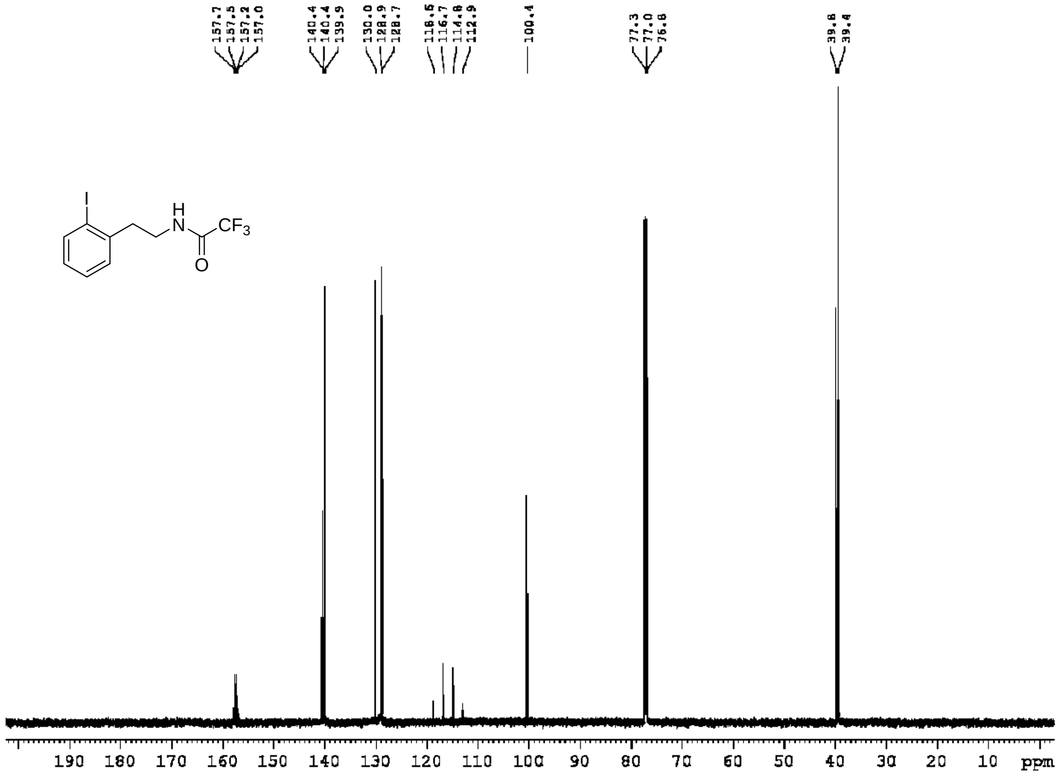

^{1}H NMR (600 MHz, $CDCl_3$) of (E)-1-fluoro-3-(2-nitrovinyl)benzene

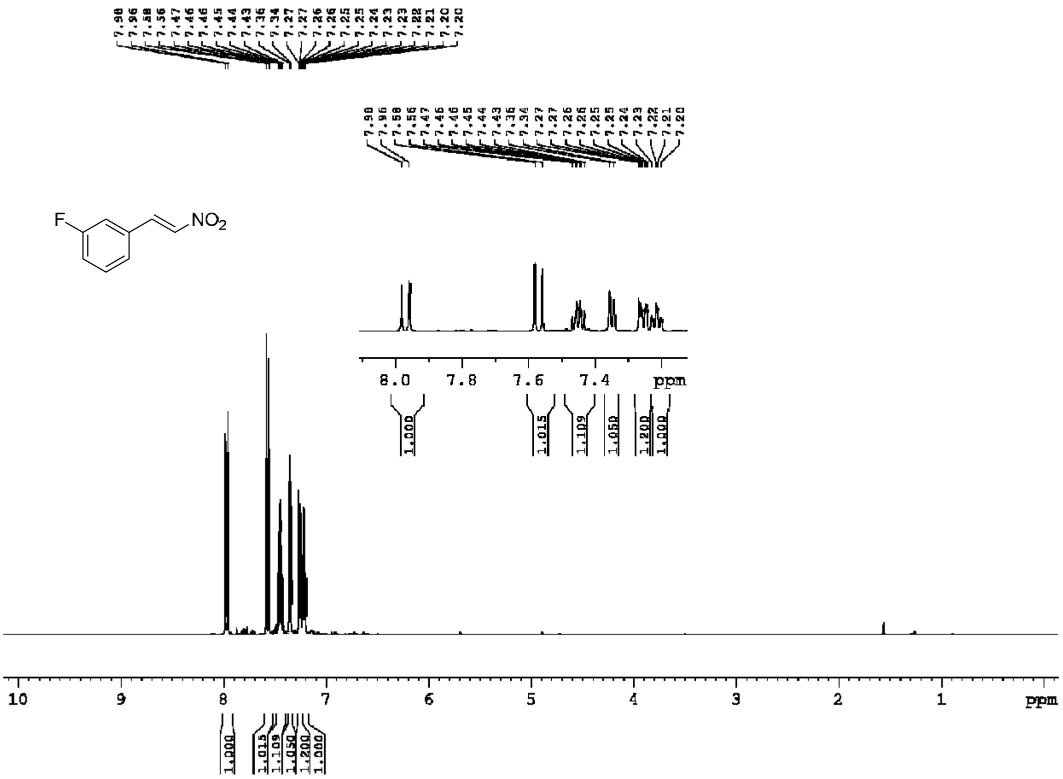

^{13}C NMR (151 MHz, $CDCl_3$) of (E)-1-fluoro-3-(2-nitrovinyl)benzene

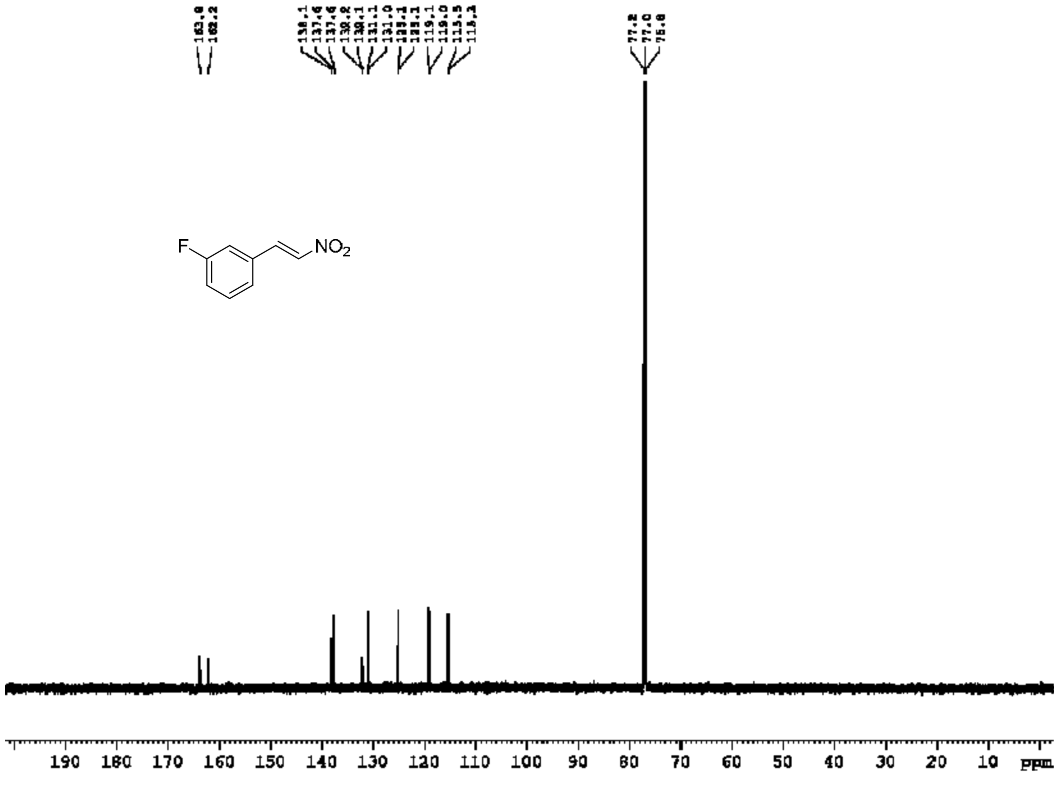

^{1}H NMR (600 MHz, $CDCl_3$) of 2,2,2-trifluoro-N-(3-fluorophenethyl)acetamide (4e)

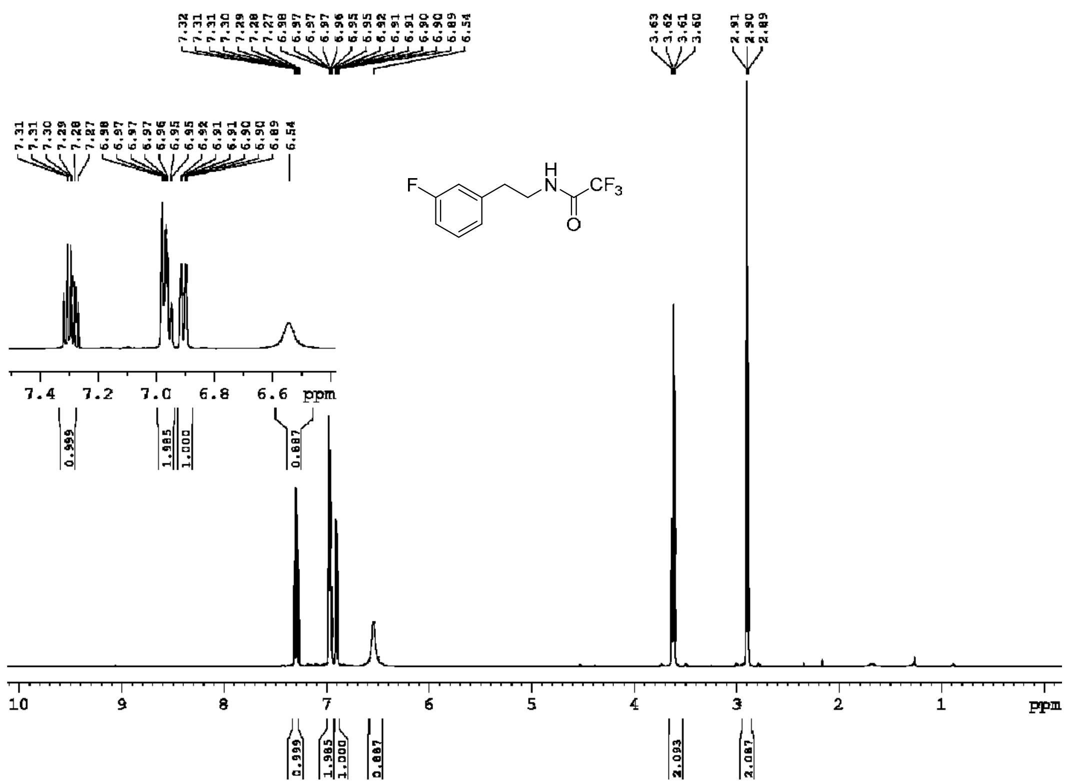

^{13}C NMR (151 MHz, $CDCl_3$) of 2,2,2-trifluoro-N-(3-fluorophenethyl)acetamide (4e)

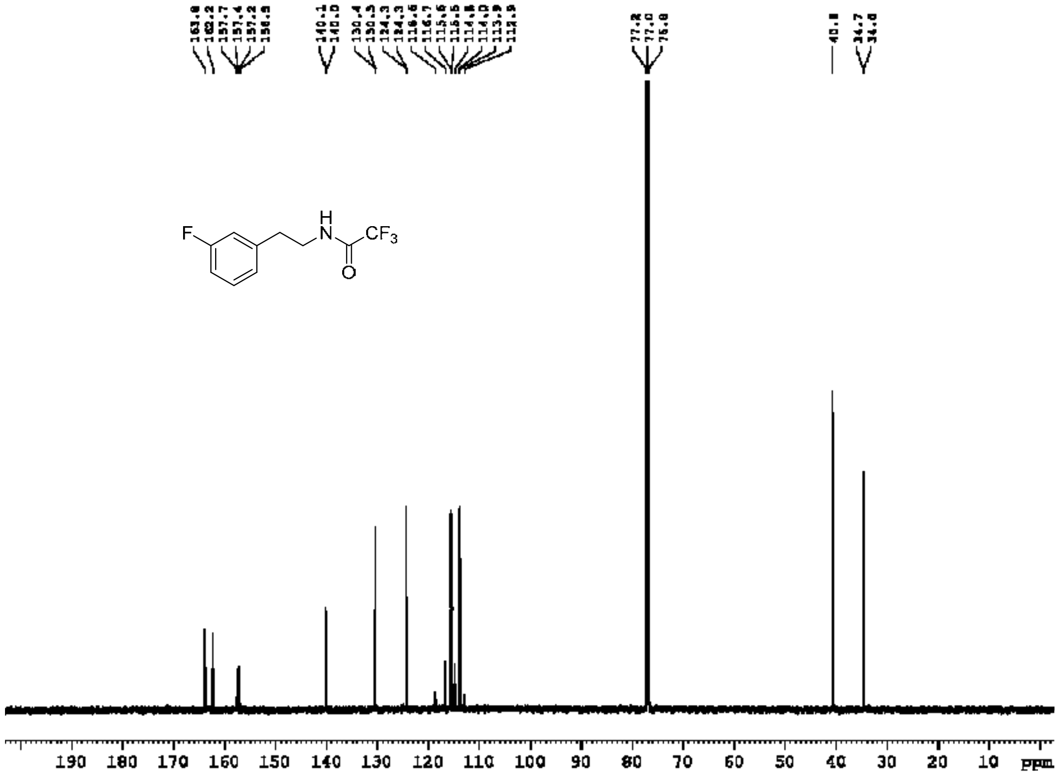

^1H NMR (600 MHz, $CDCl_3$) of N-(2-bromophenethyl)-2,2,2-trifluoroacetamide (4f)

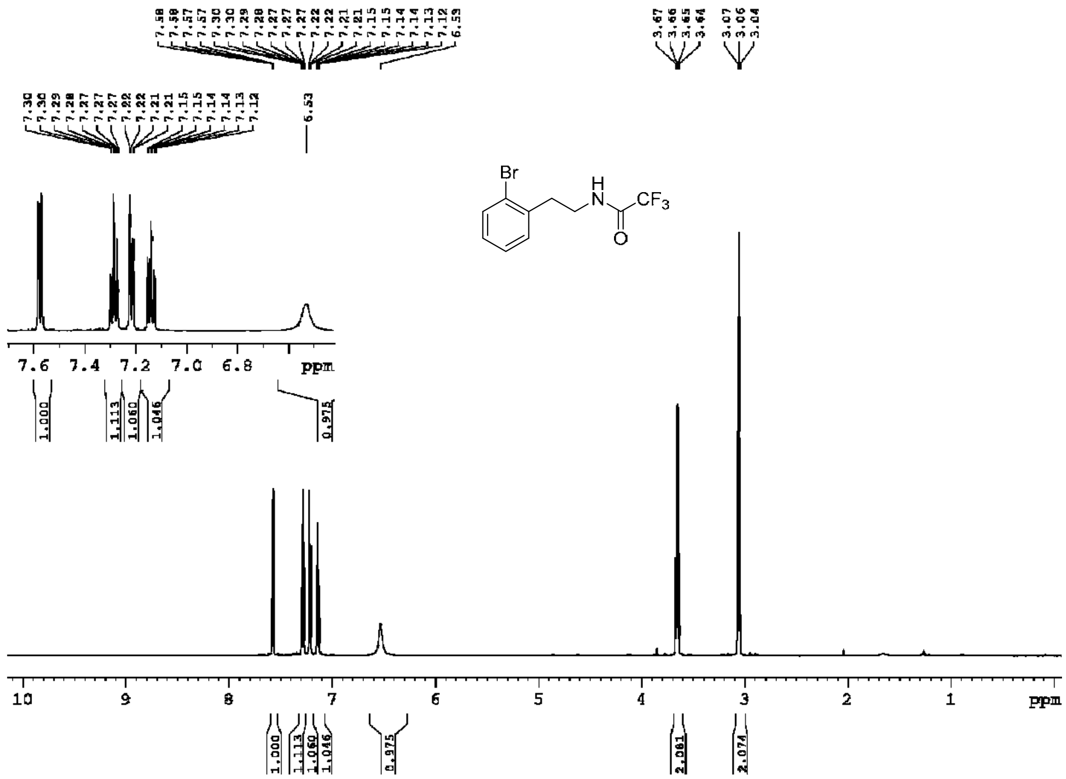

^{13}C NMR (151 MHz, $CDCl_3$) of N-(2-bromophenethyl)-2,2,2-trifluoroacetamide (4f)

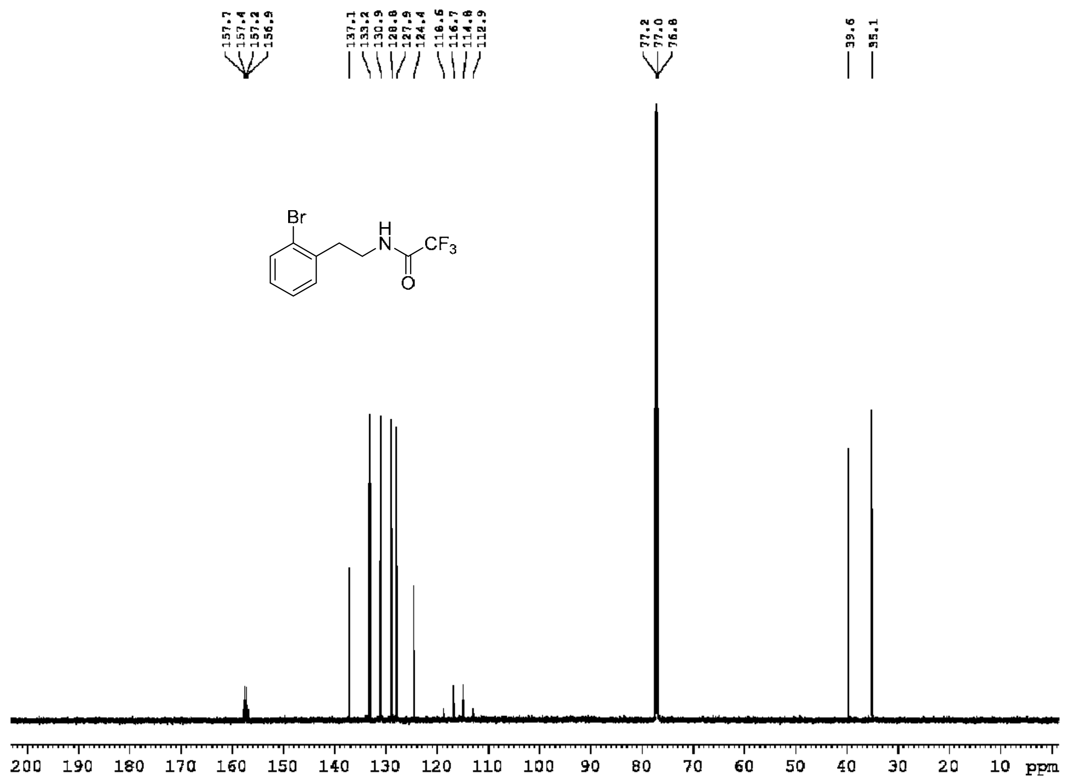

^{1}H NMR (600 MHz, $CDCl_3$) of methyl 2-(2-(2,2,2-trifluoroacetamido)ethyl)benzoate (4g)

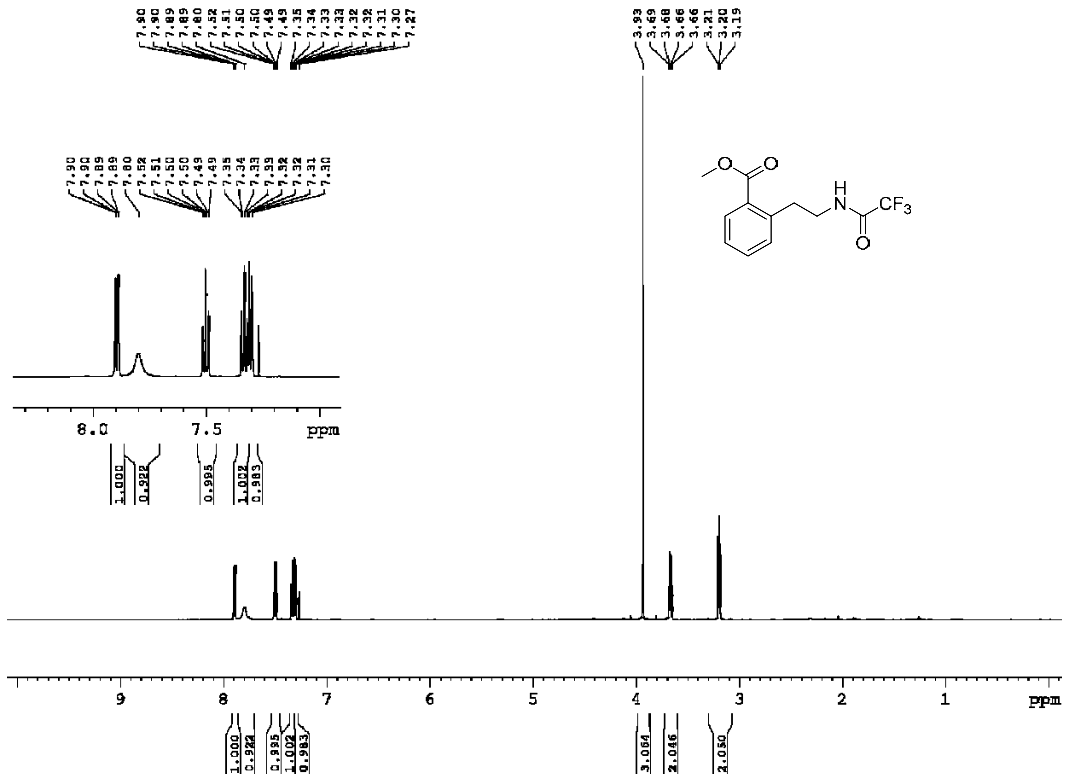

^{13}C NMR (151 MHz, $CDCl_3$) of methyl 2-(2-(2,2,2-trifluoroacetamido)ethyl)benzoate (4g)

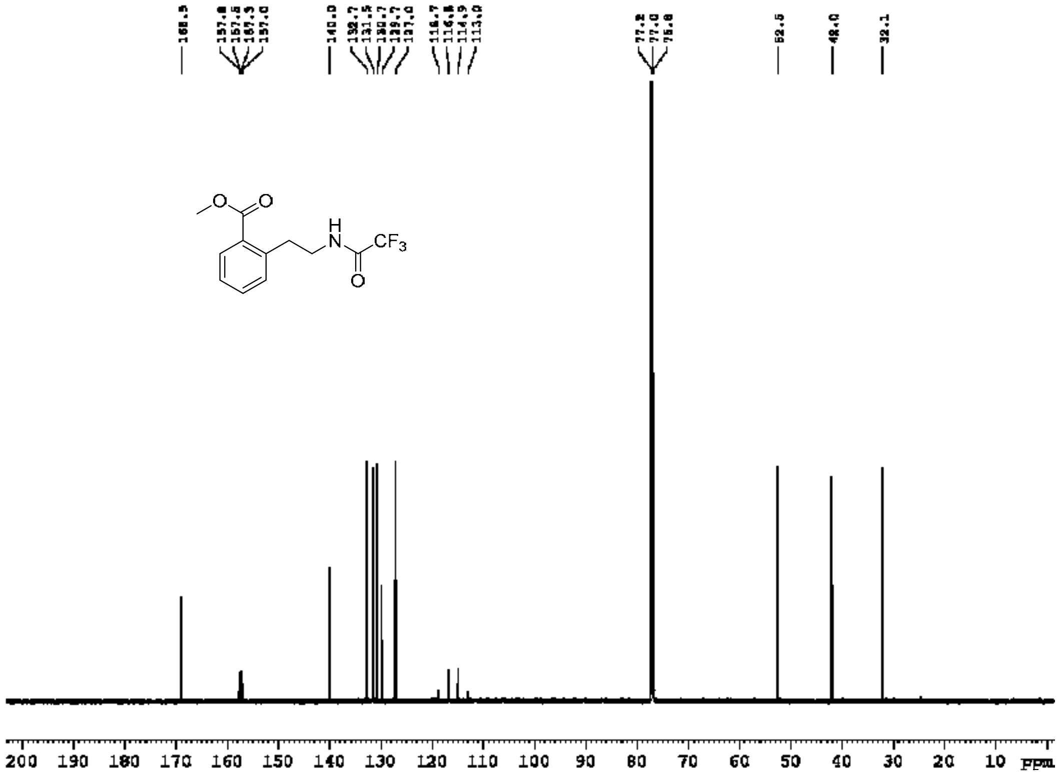

^{1}H NMR (600 MHz, $CDCl_3$) of (E)-1-chloro-2-(2-nitrovinyl)benzene

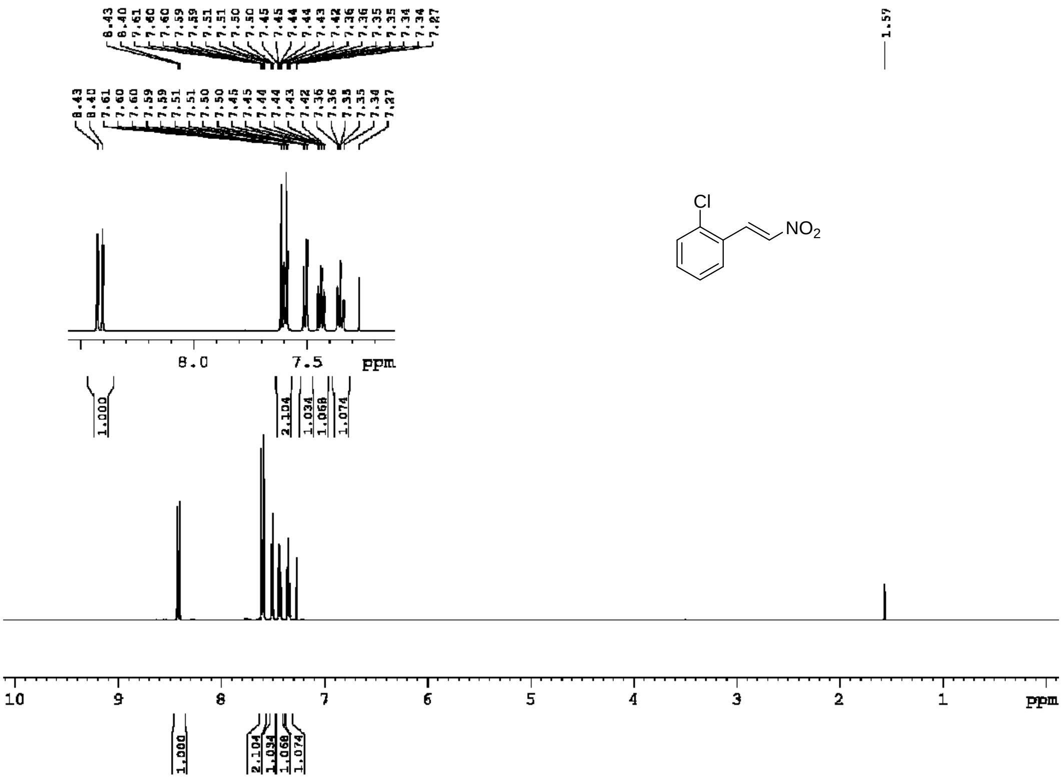

^{13}C NMR (151 MHz, $CDCl_3$) of (E)-1-chloro-2-(2-nitrovinyl)benzene

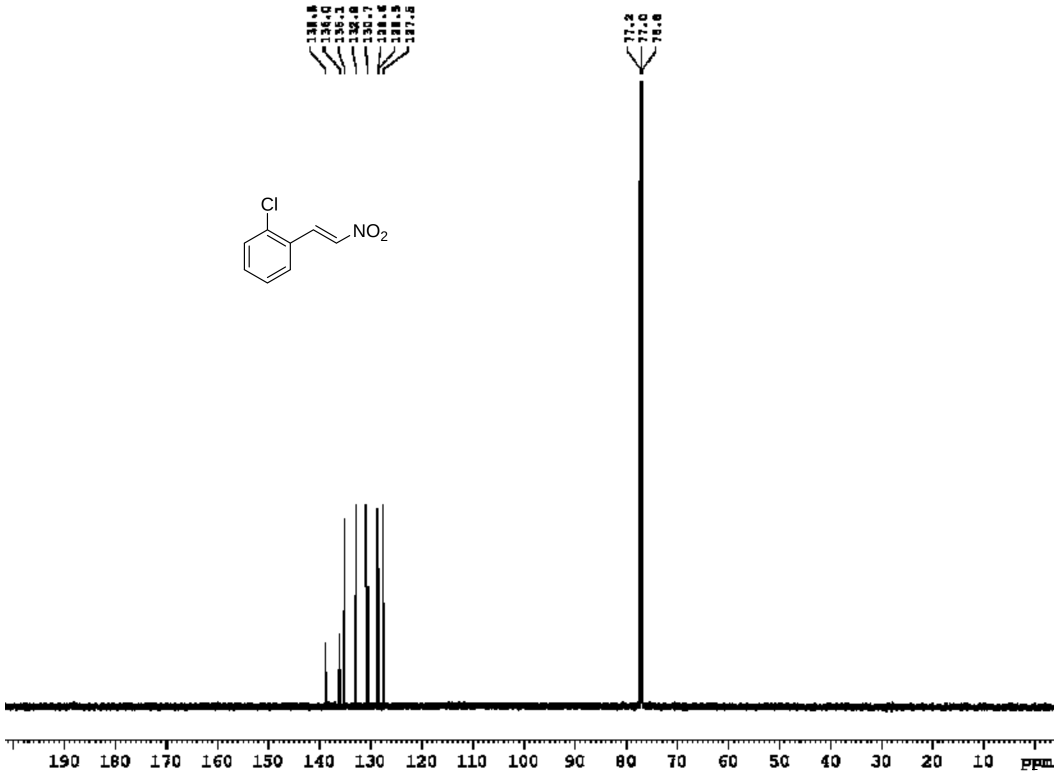

^{1}H NMR (600 MHz, $CDCl_3$) of N-(2-chlorophenethyl)-2,2,2-trifluoroacetamide (4h)

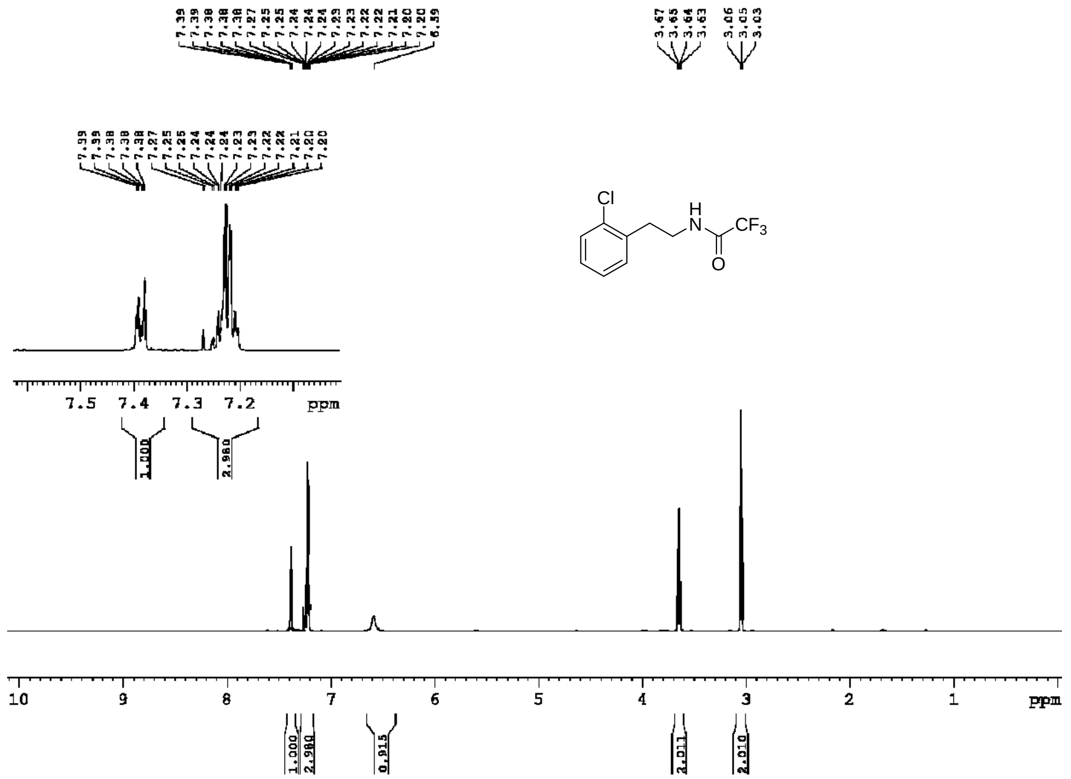

^{13}C NMR (151 MHz, $CDCl_3$) of N-(2-chlorophenethyl)-2,2,2-trifluoroacetamide (4h)

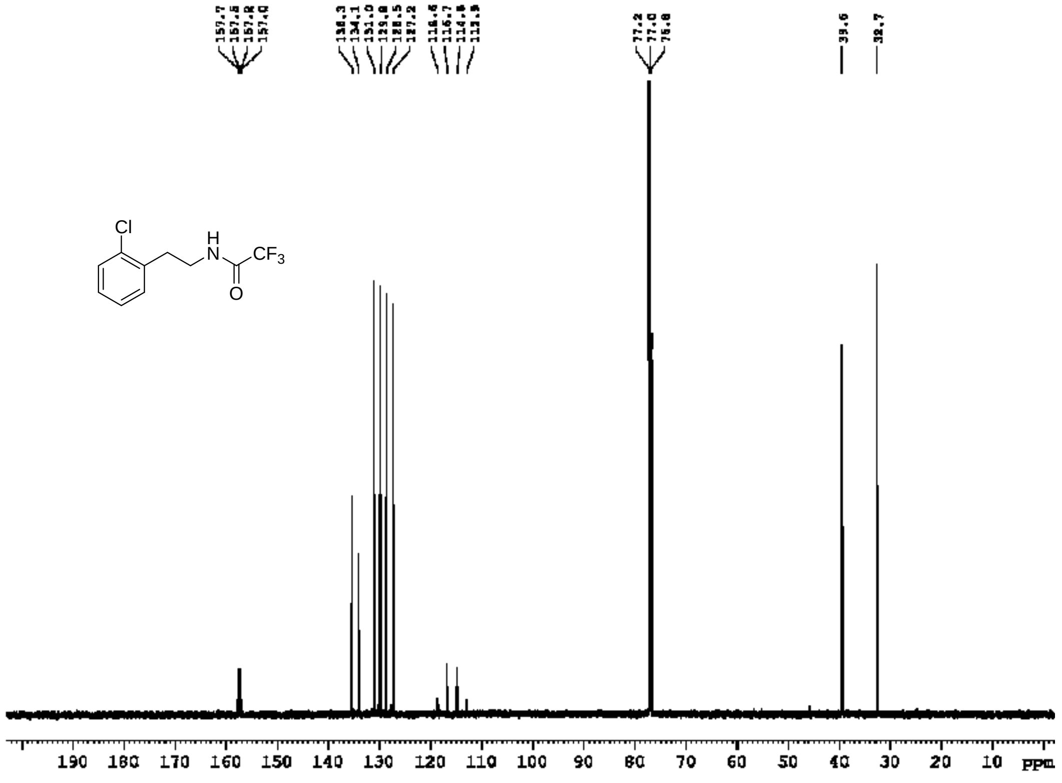

^1H NMR (600 MHz, $CDCl_3$) of 2,2,2-trifluoro-N-(2-methylphenethyl)acetamide (4i)

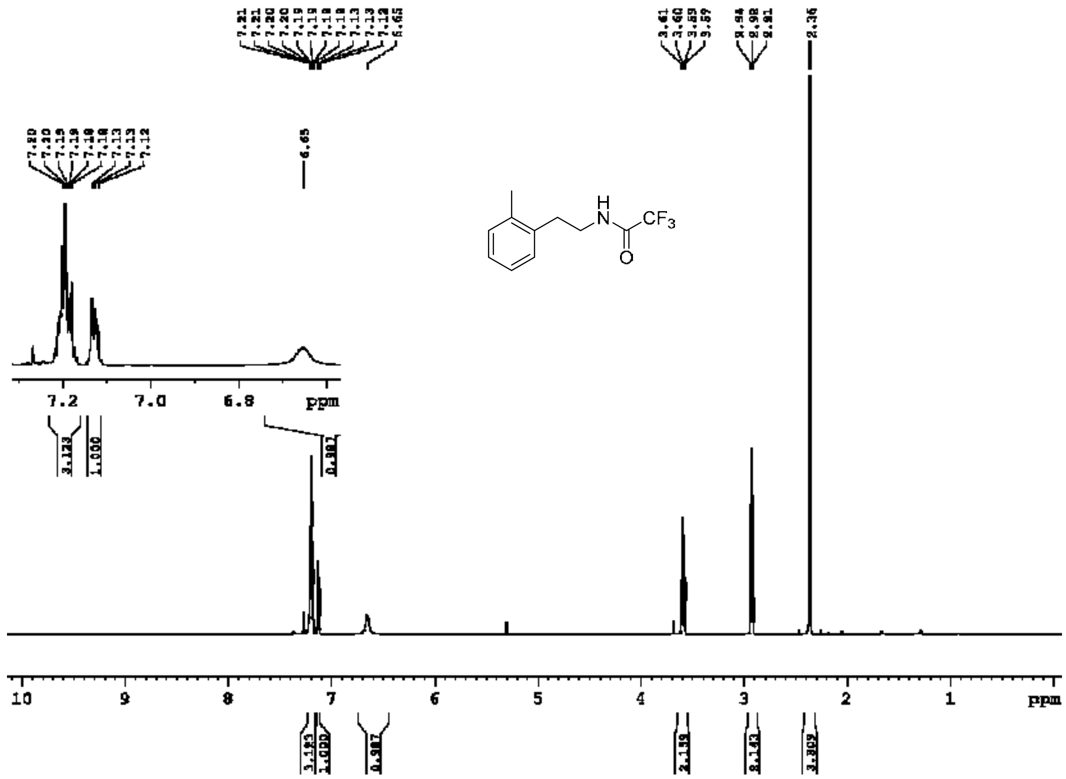

^{13}C NMR (151 MHz, $CDCl_3$) of 2,2,2-trifluoro-N-(2-methylphenethyl)acetamide (4i)

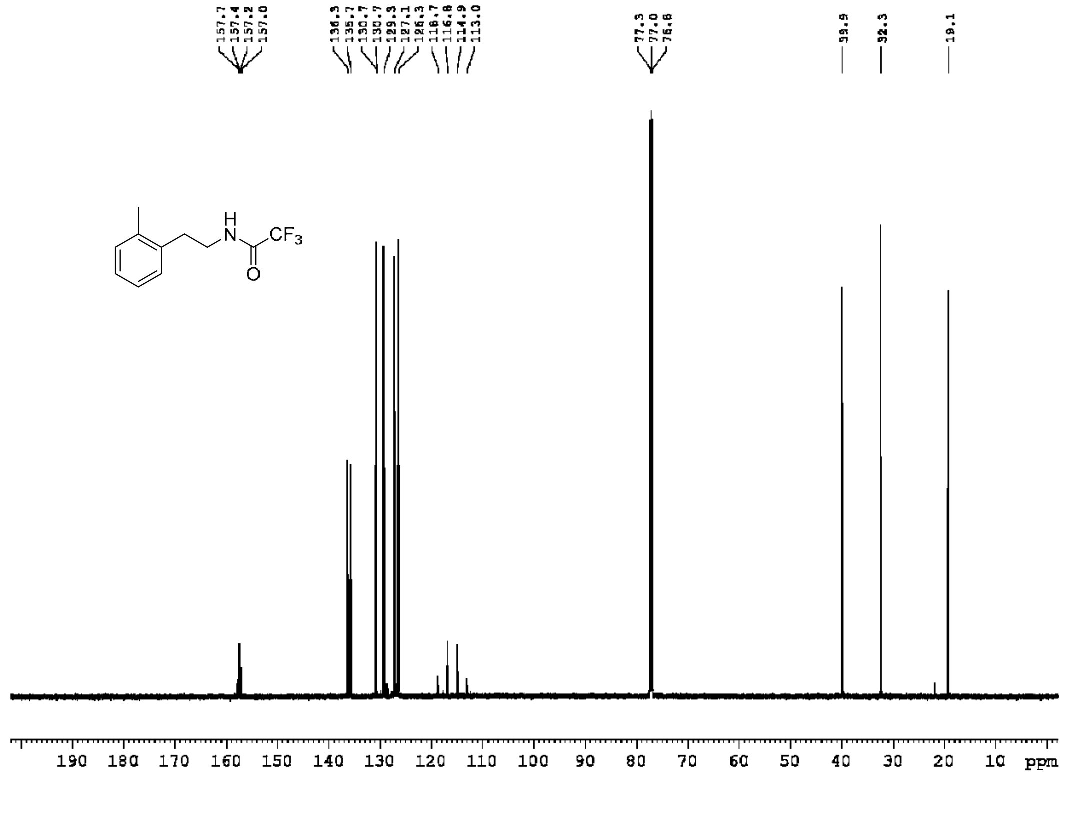

^{1}H NMR (600 MHz, $CDCl_3$) of 2,2,2-trifluoro-N-(2-methoxyphenethyl)acetamide (4j)

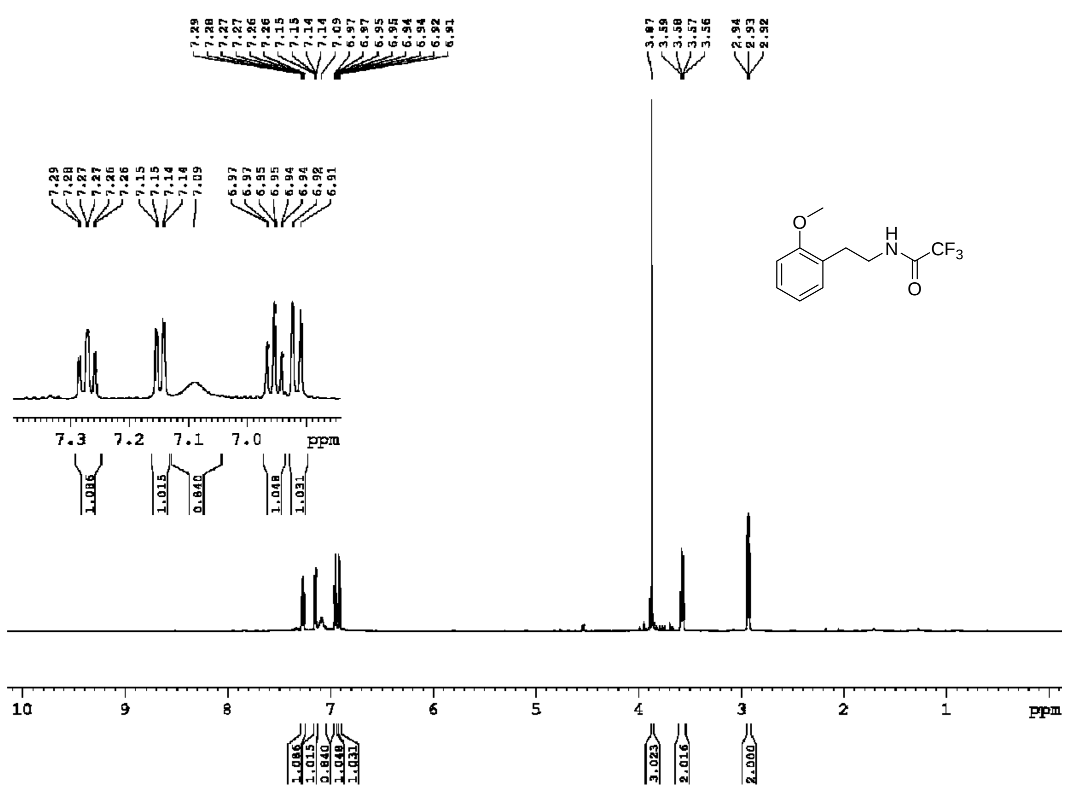

^{13}C NMR (151 MHz, $CDCl_3$) of 2,2,2-trifluoro-N-(2-methoxyphenethyl)acetamide (4j)

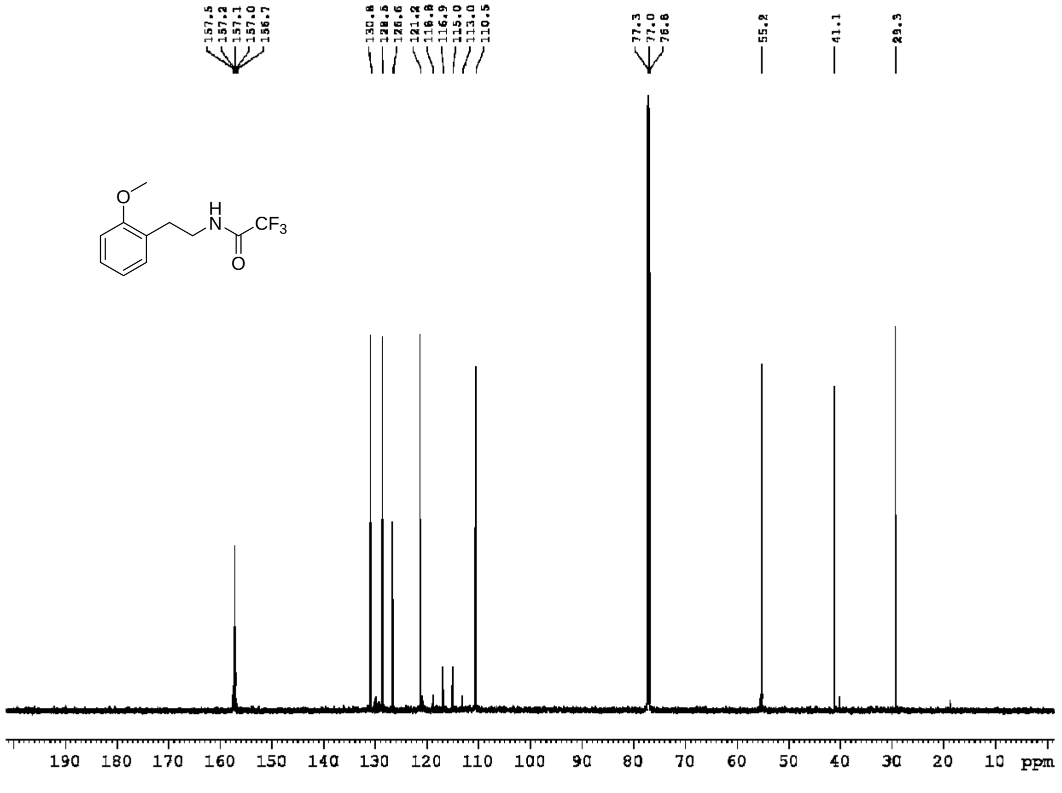

^{1}H NMR (600 MHz, $CDCl_3$) of N-(2-(((tert-butyldimethylsilyl)oxy)methyl)phenethyl)-2,2,2-trifluoroacetamide (4k)

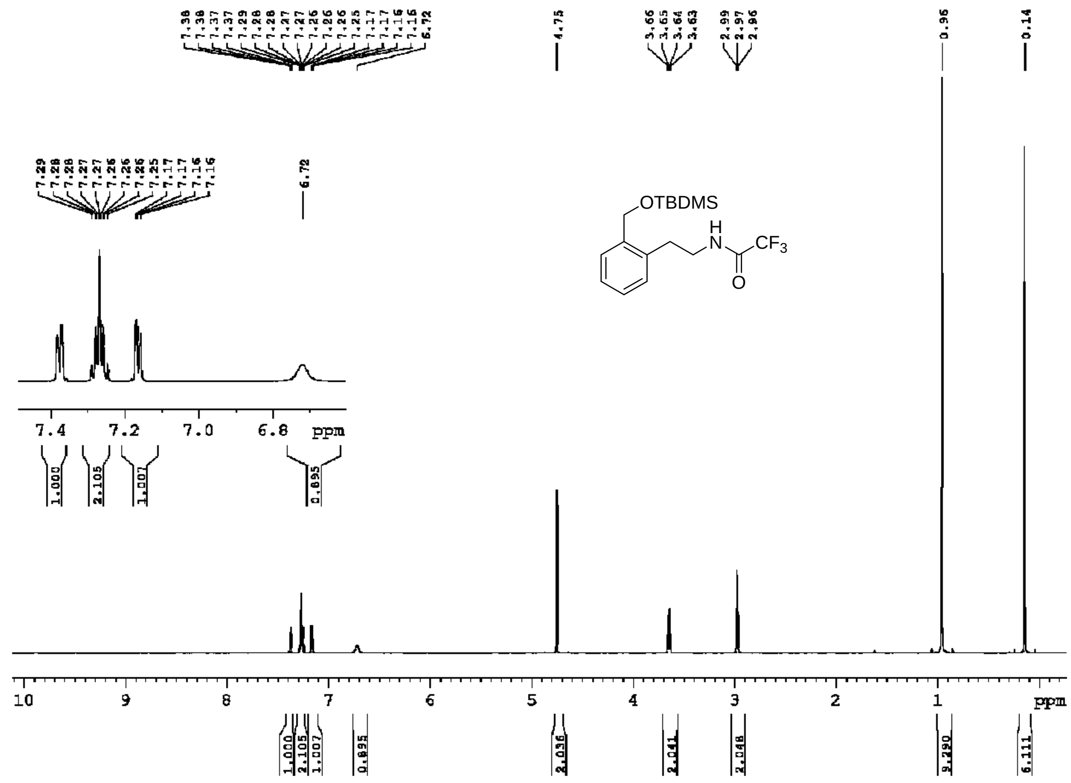

^{13}C NMR (151 MHz, $CDCl_3$) of N-(2-(((tert-butyldimethylsilyl)oxy)methyl)phenethyl)-2,2,2-trifluoroacetamide (4k)

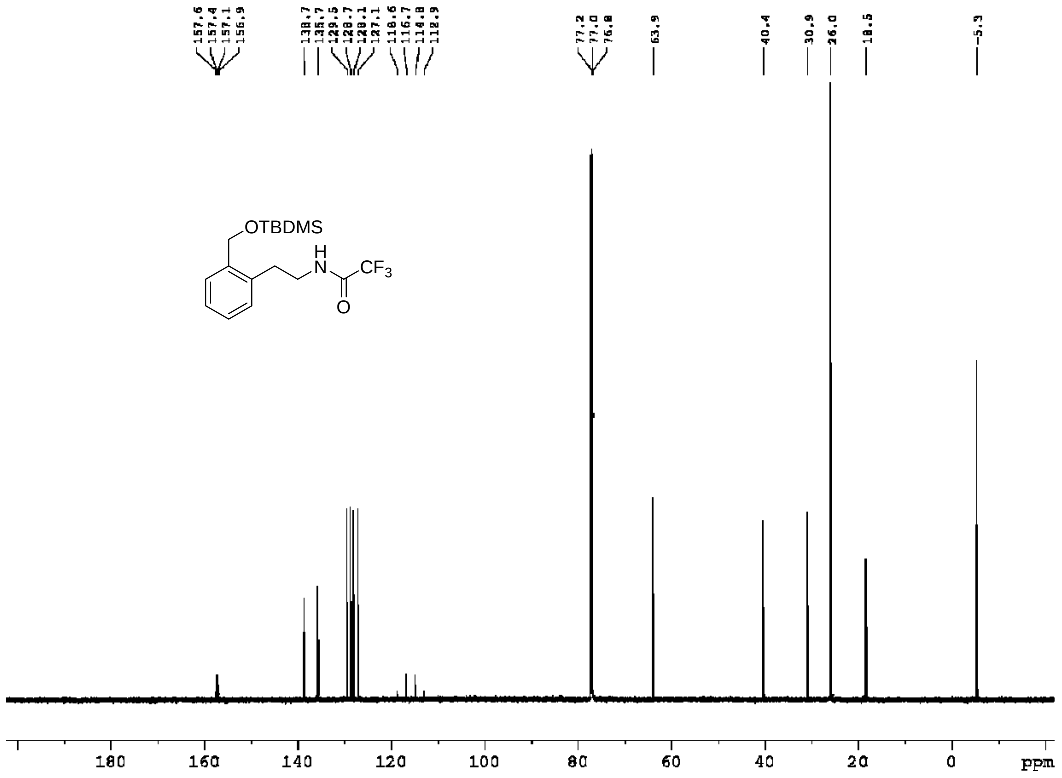

^{1}H NMR (600 MHz, $CDCl_3$) of 2,2,2-trifluoro-N-phenethylacetamide (4l)

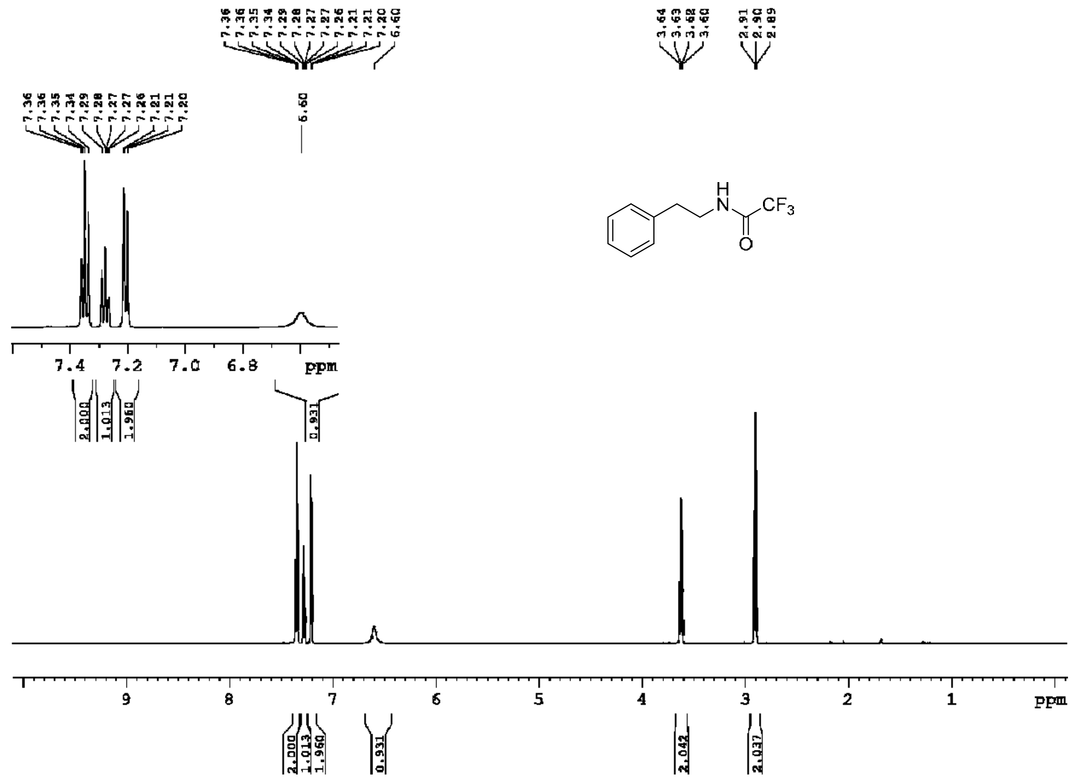

^{13}C NMR (151 MHz, $CDCl_3$) of 2,2,2-trifluoro-N-phenethylacetamide (4l)

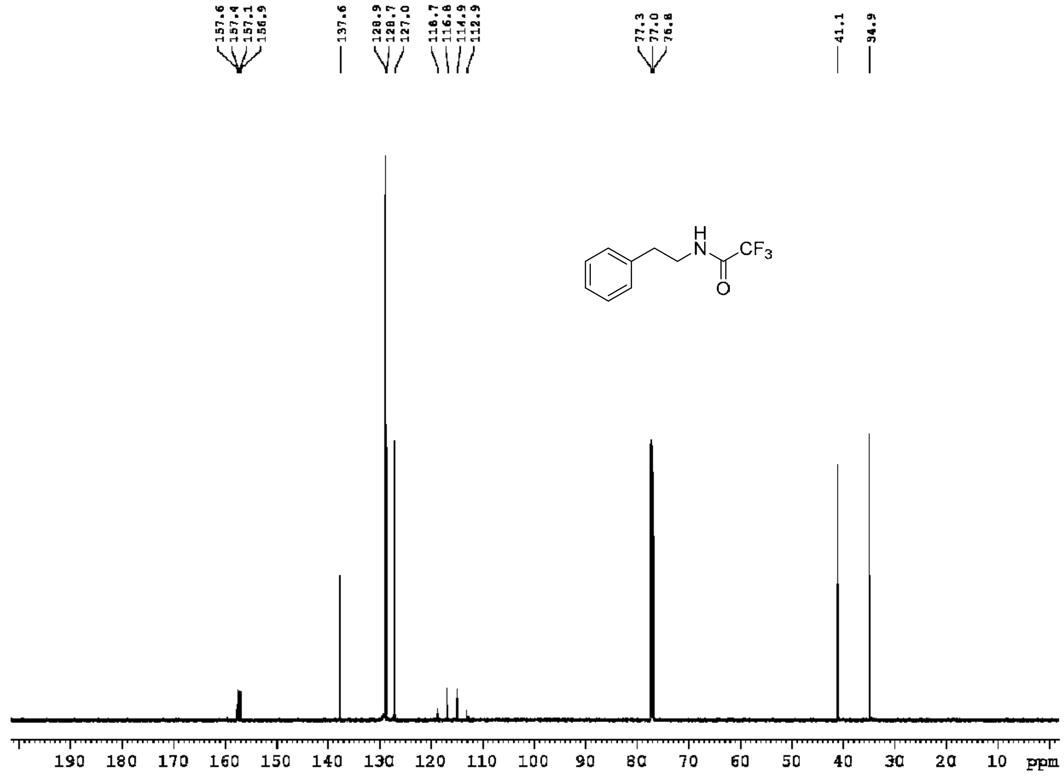

^{1}H NMR (600 MHz, $CDCl_3$) of 2,2,2-trifluoro-N-(2-(pyridin-2-yl)ethyl)acetamide (4m)

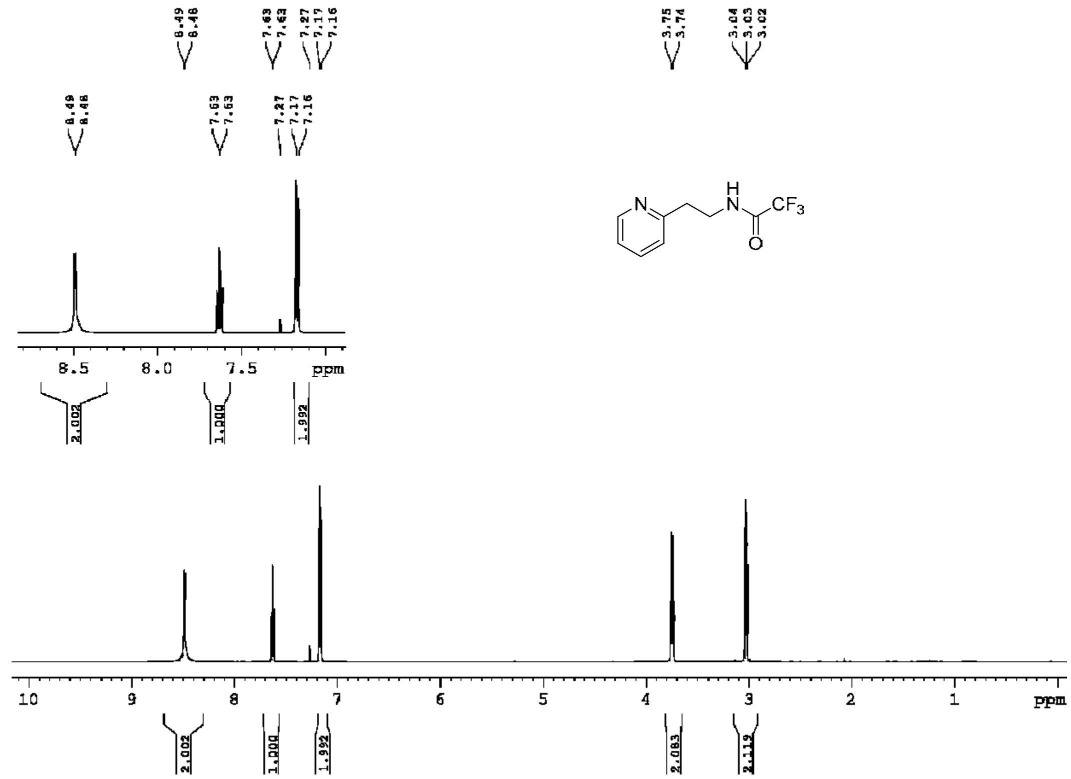

^{13}C NMR (151 MHz, $CDCl_3$) of 2,2,2-trifluoro-N-(2-(pyridin-2-yl)ethyl)acetamide (4m)

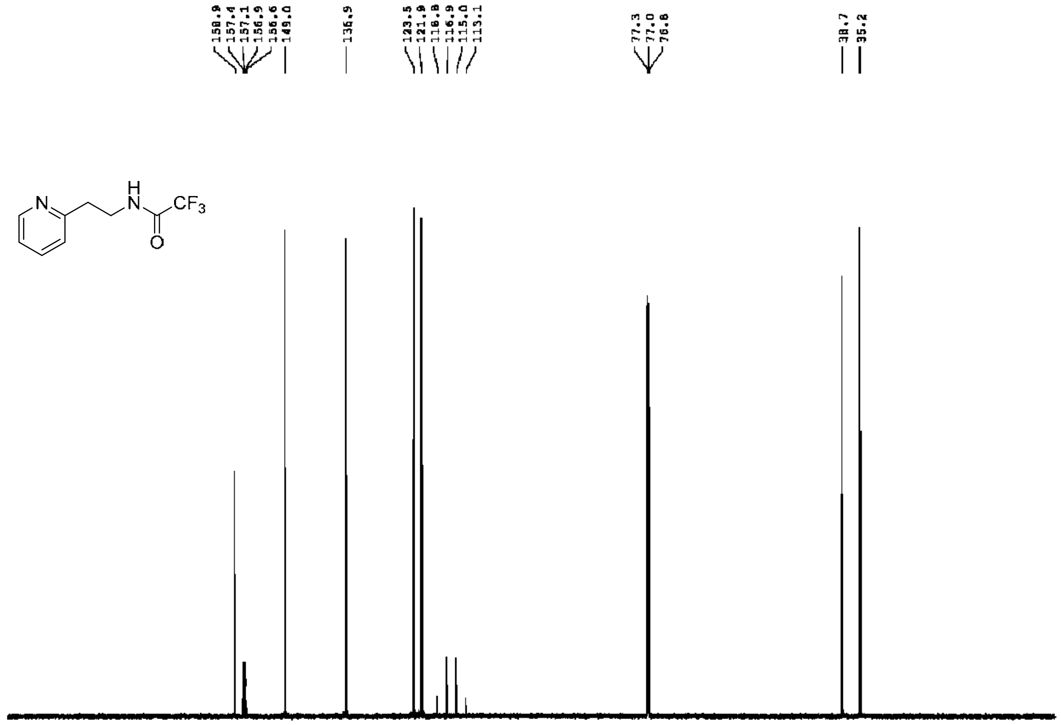

^{1}H-NMR (600 MHz, $CDCl_3$) 3-(2-(Trifluoromethyl)phenyl)propanenitrile

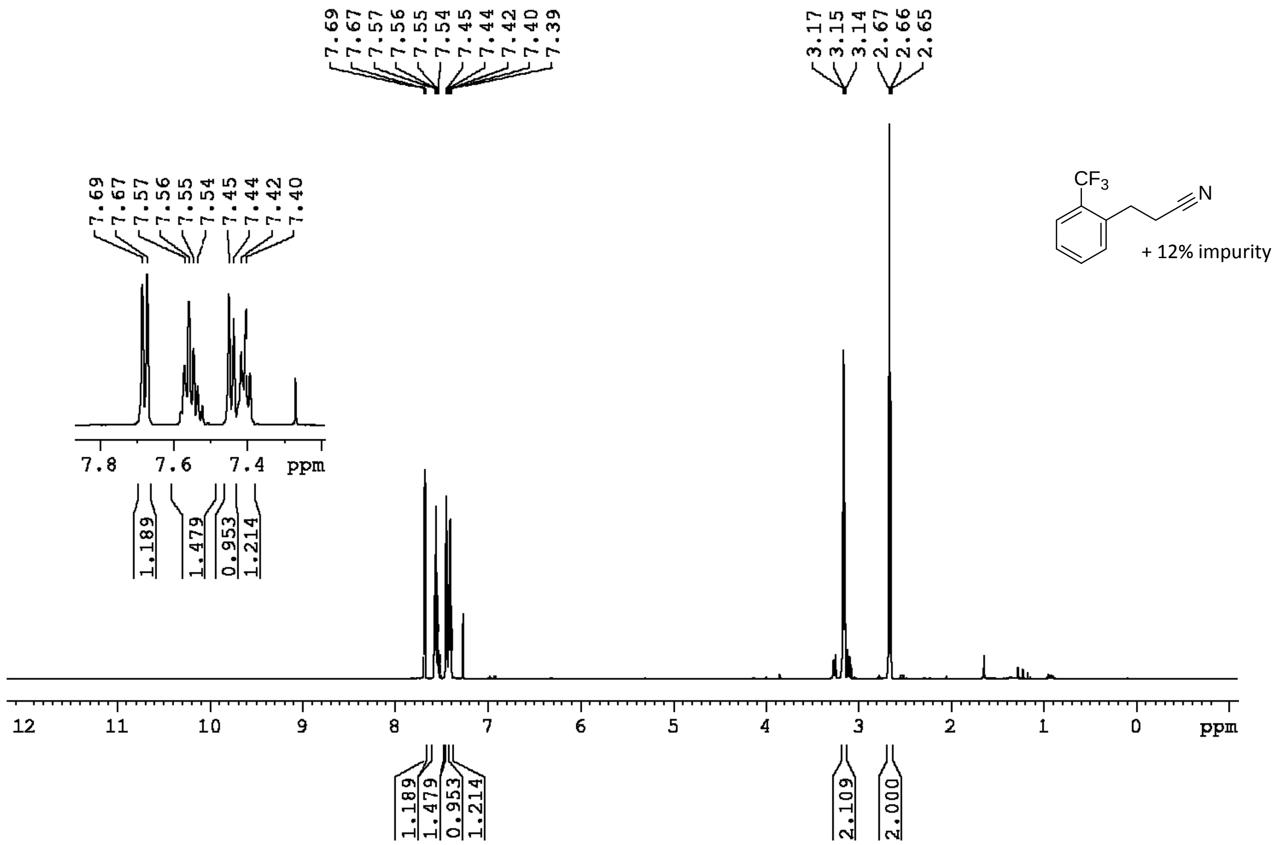

^{13}C-NMR (151 MHz, $CDCl_3$) 3-(2-(Trifluoromethyl)phenyl)propanenitrile

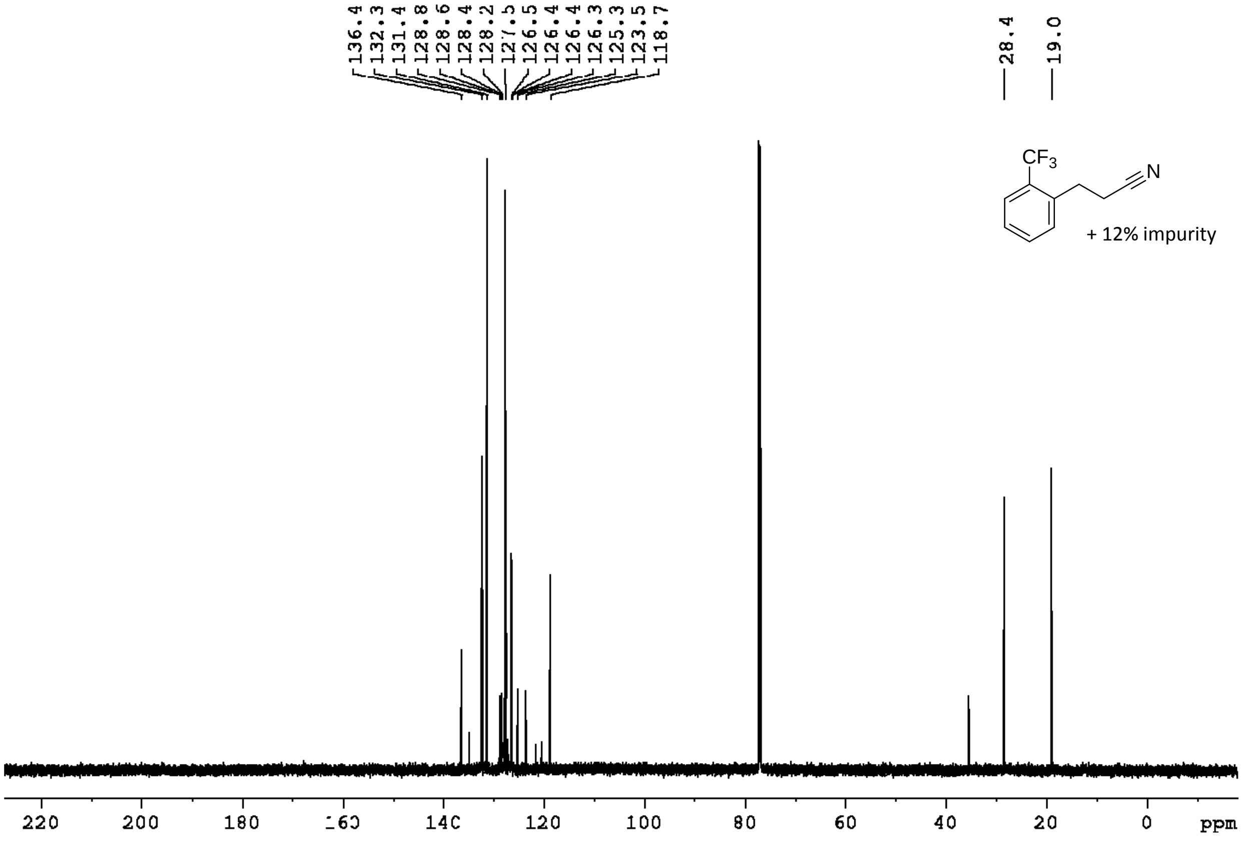

^{1}H-NMR (600 MHz, $CDCl_3$) 3-(2-(Trifluoromethyl)phenyl)propan-1-amine

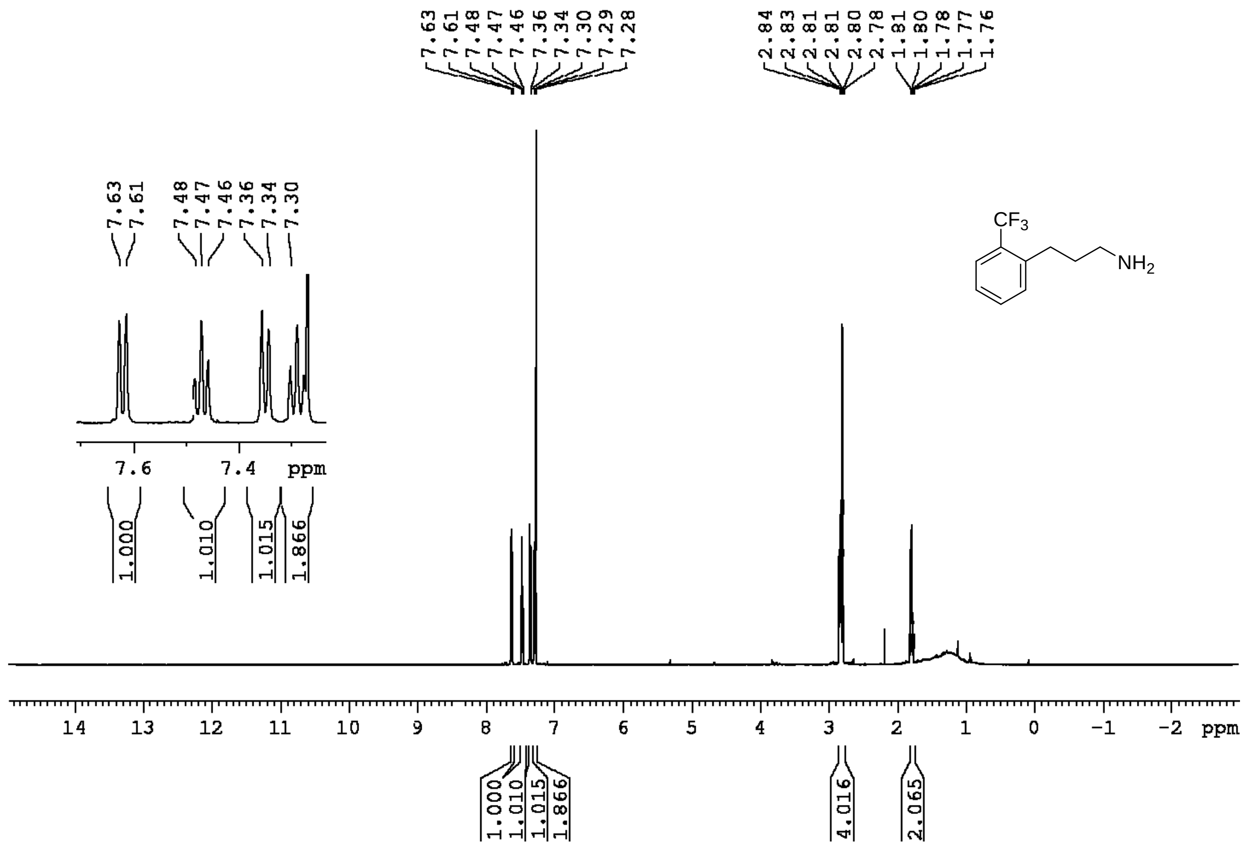

^{13}C-NMR (151 MHz, $CDCl_3$) 3-(2-(Trifluoromethyl)phenyl)propan-1-amine

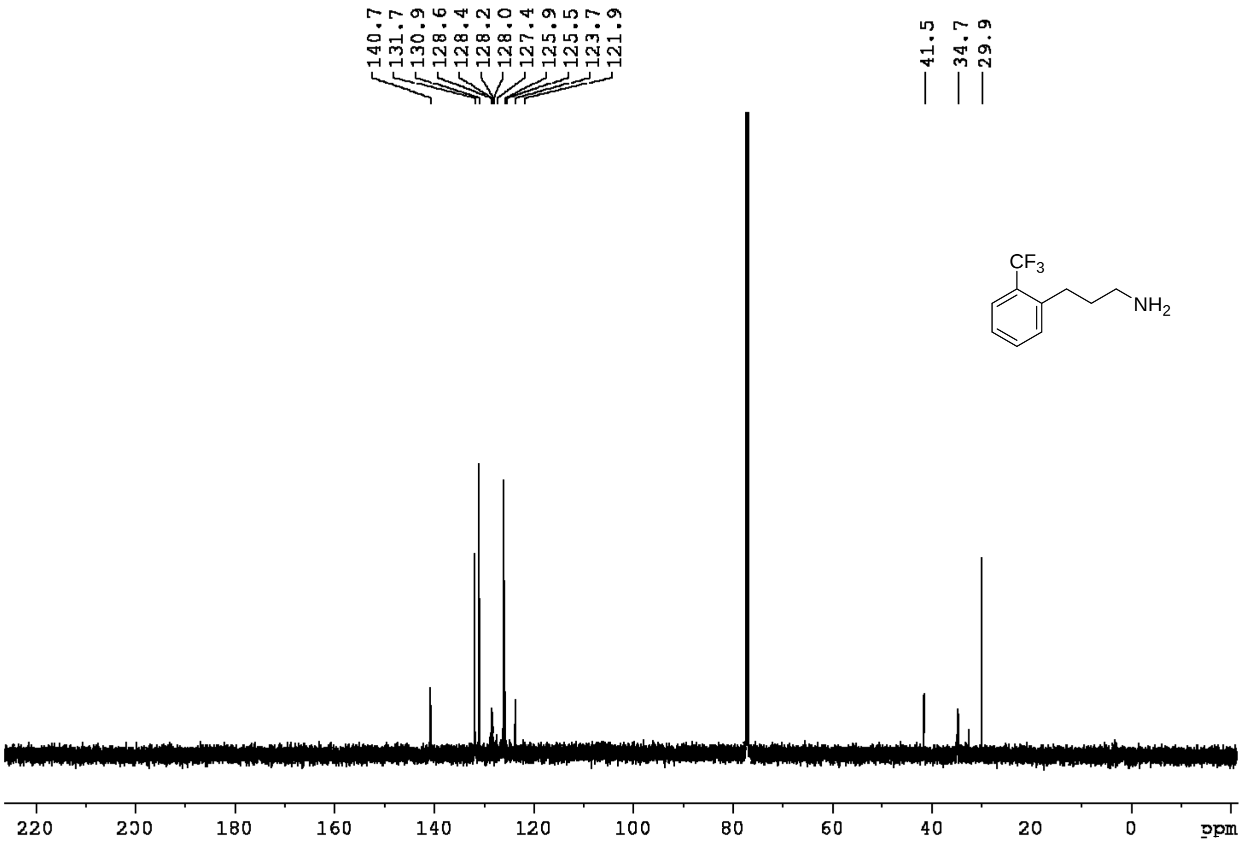

^{1}H-NMR (600 MHz, $CDCl_3$) 2,2,2-Trifluoro-*N*-(3-(2-(trifluoromethyl)phenyl)propyl)acetamide (6a)

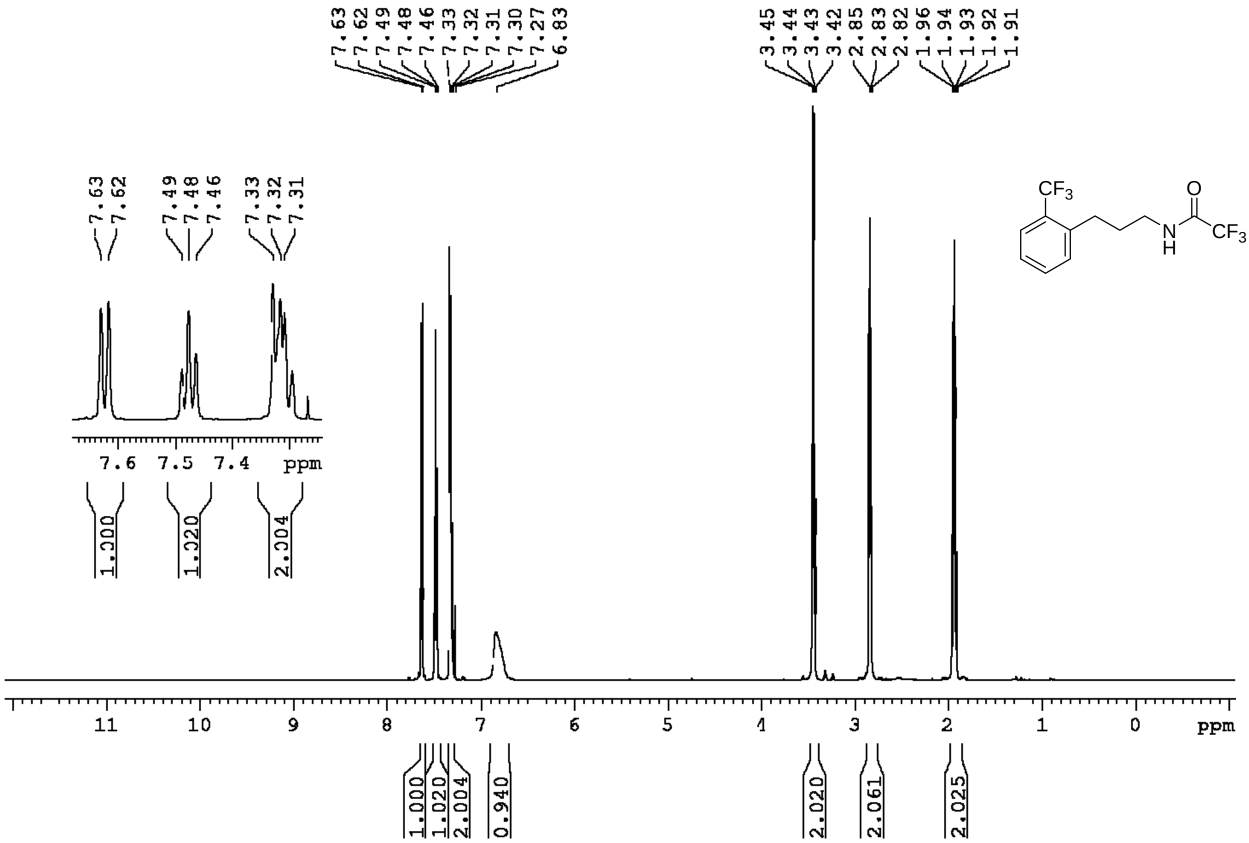

^{13}C-NMR (151 MHz, $CDCl_3$) 2,2,2-Trifluoro-*N*-(3-(2-(trifluoromethyl)phenyl)propyl)acetamide (6a)

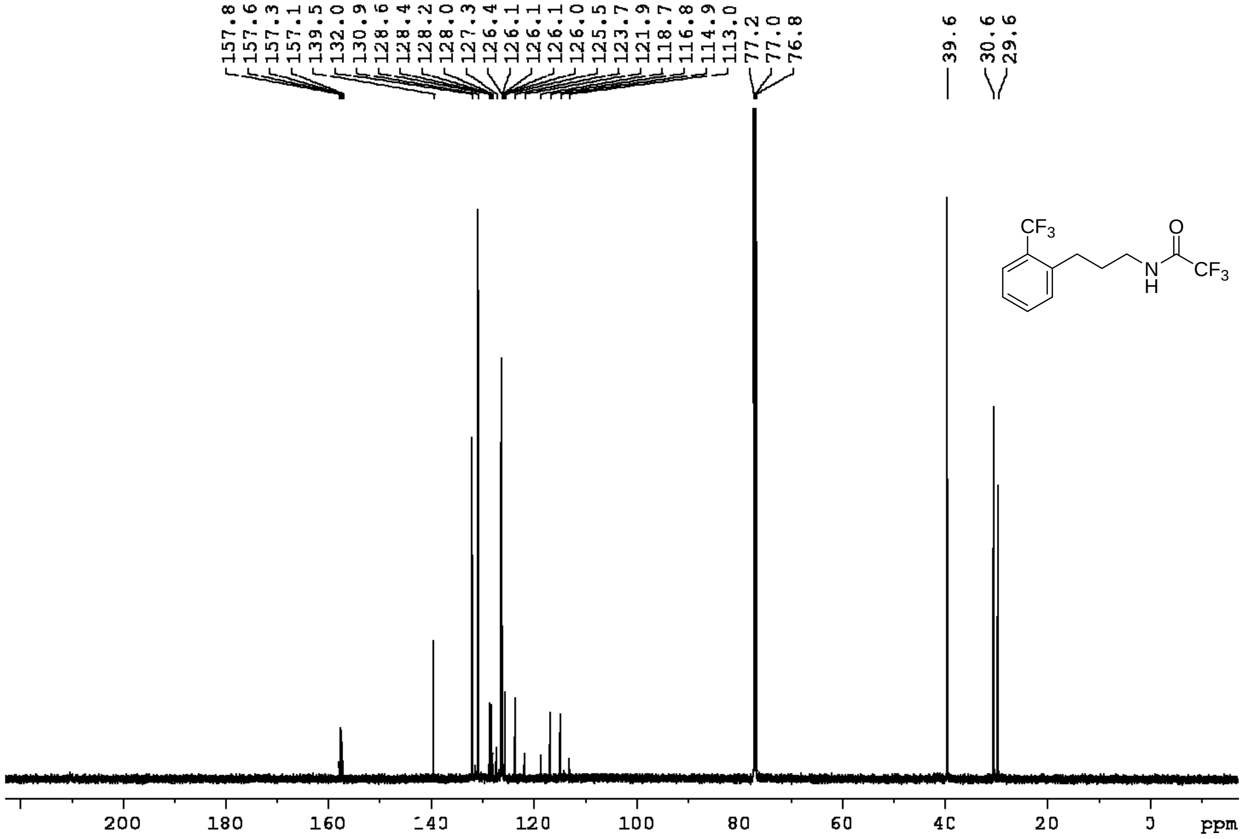

^{1}H-NMR (600 MHz, $CDCl_3$) 2,2,2-Trifluoro-*N*-(3-phenylpropyl)acetamide (6b)

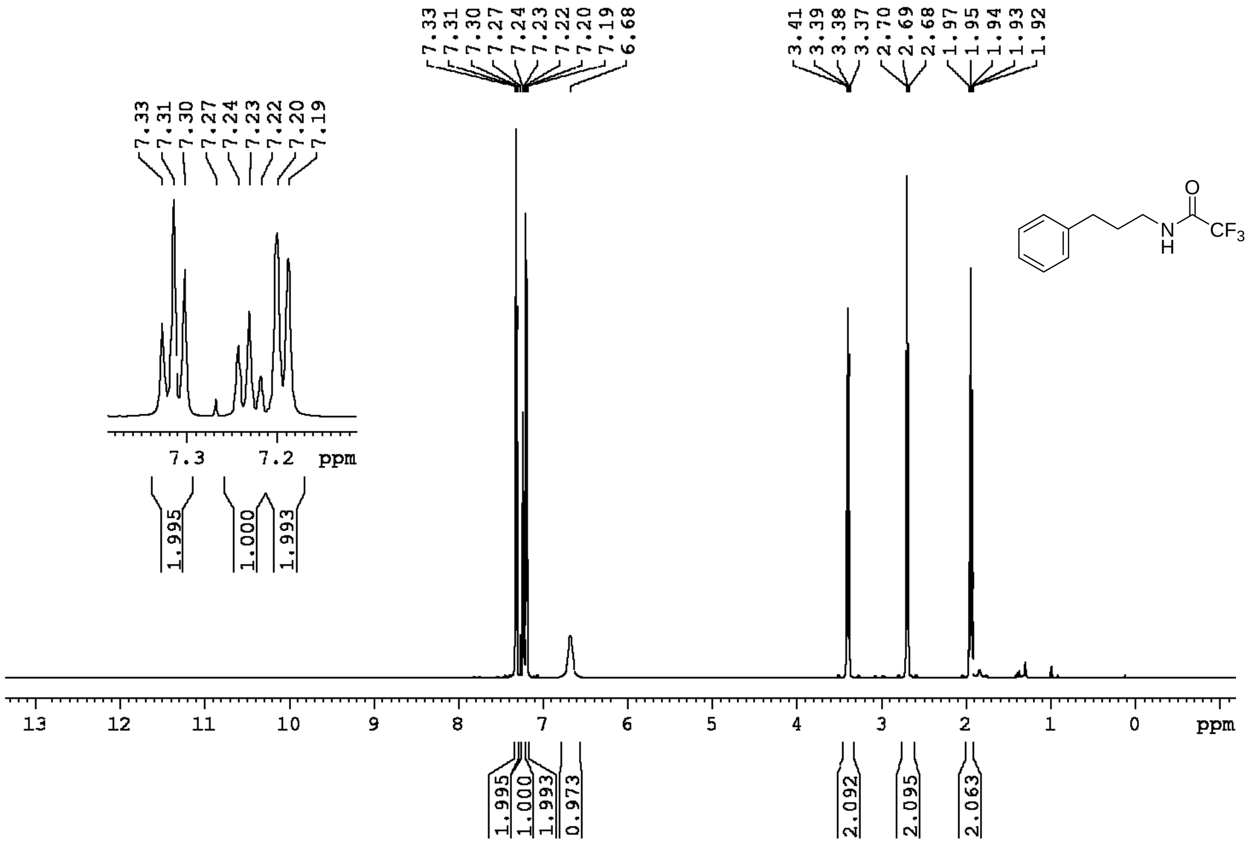

^{13}C-NMR (151 MHz, $CDCl_3$) 2,2,2-Trifluoro-*N*-(3-phenylpropyl)acetamide (6b)

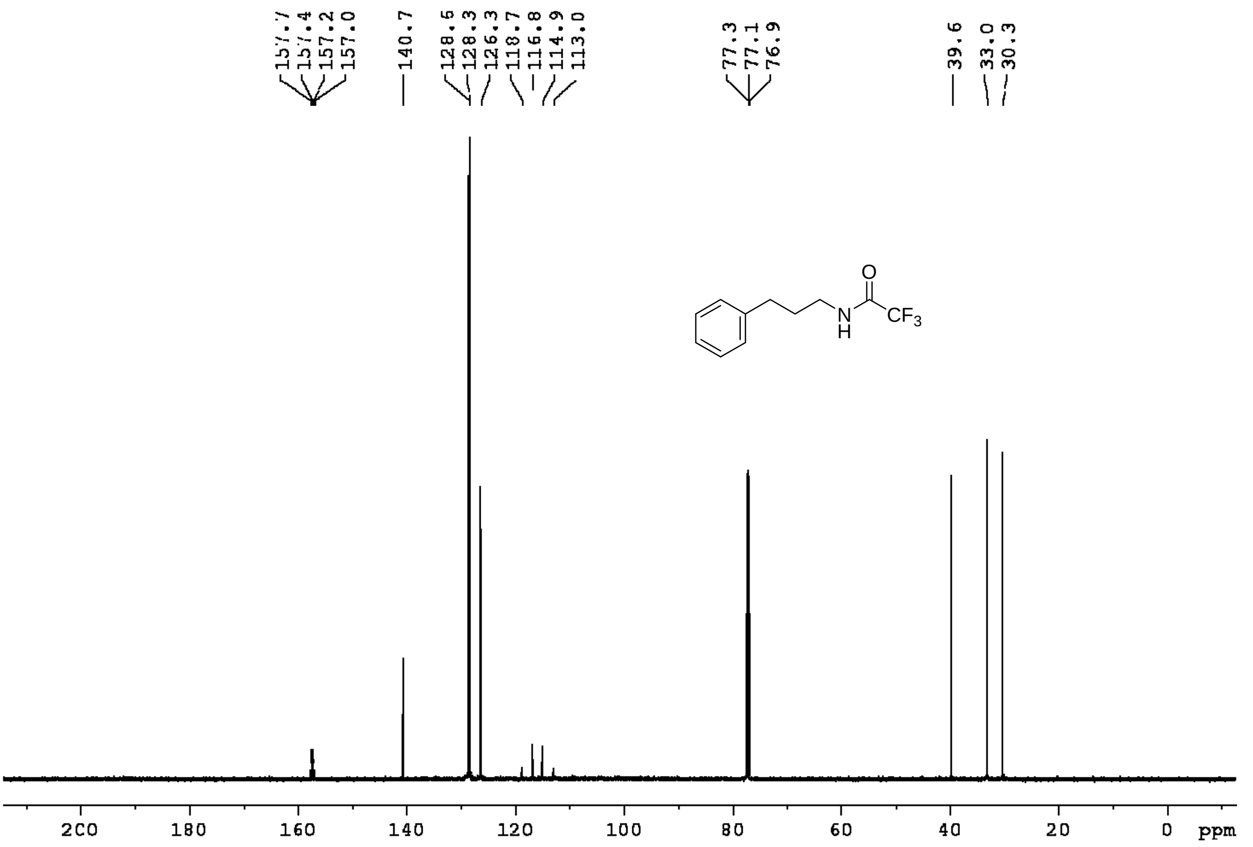

^{1}H-NMR (600 MHz, $CDCl_3$) *N*-(3-(2-bromophenyl)propyl)-2,2,2-trifluoroacetamide (6c)

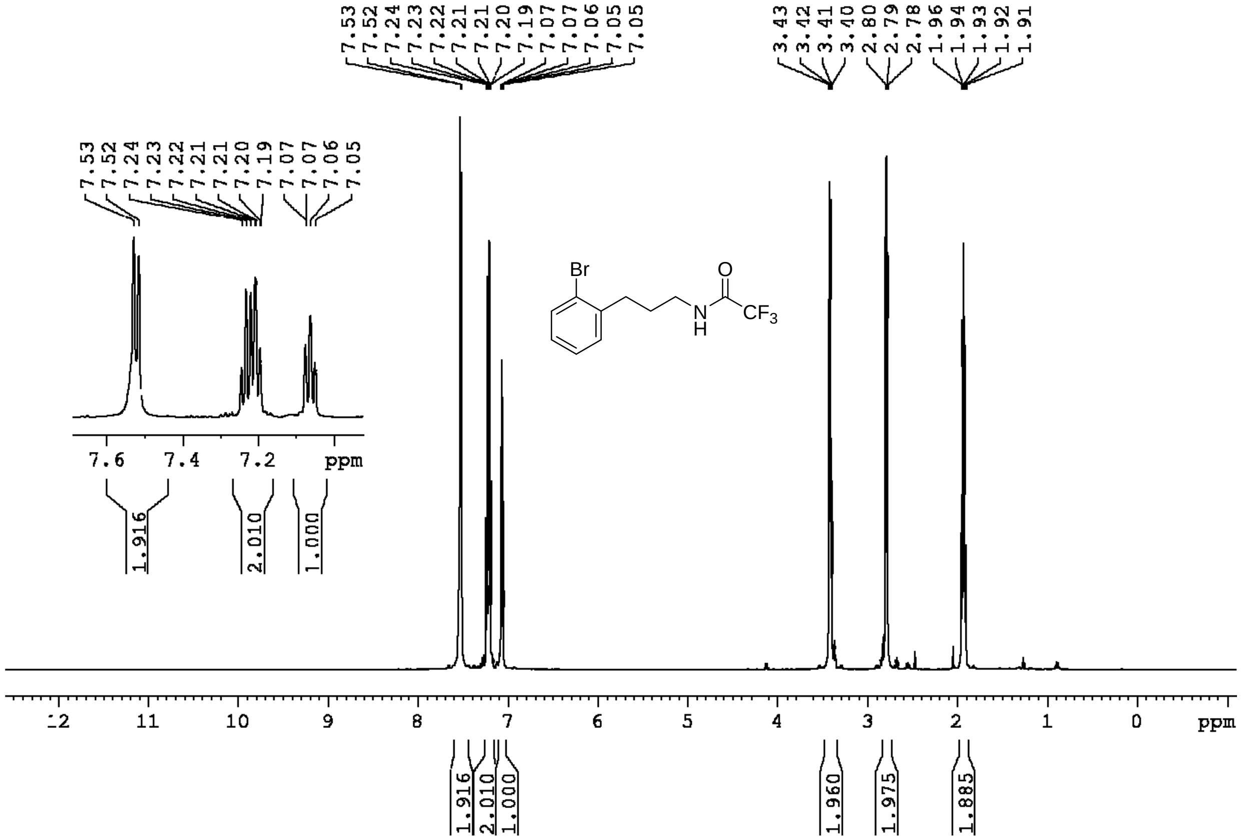

^{13}C-NMR (151 MHz, $CDCl_3$) *N*-(3-(2-bromophenyl)propyl)-2,2,2-trifluoroacetamide (6c)

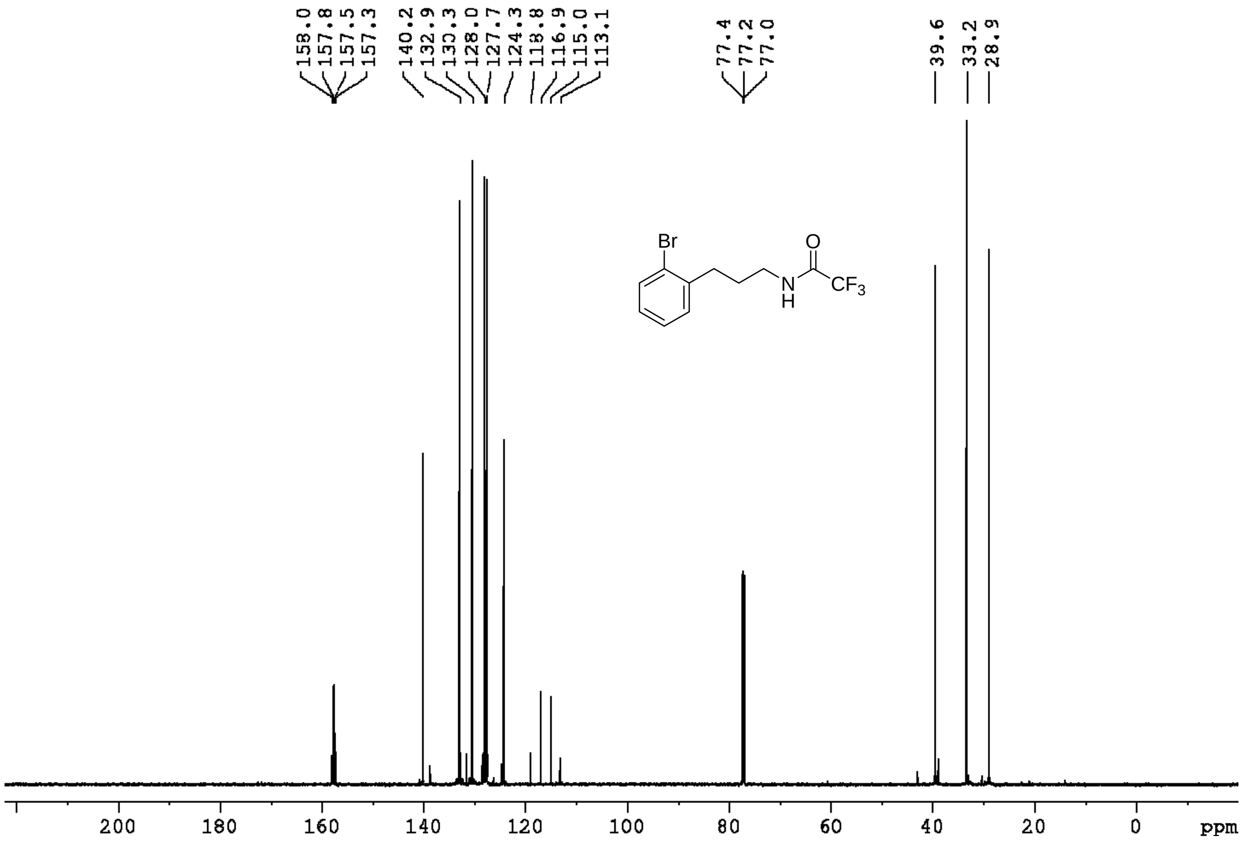

^{1}H-NMR (600 MHz, $CDCl_3$) 3-(2-Chlorophenyl)propanenitrile

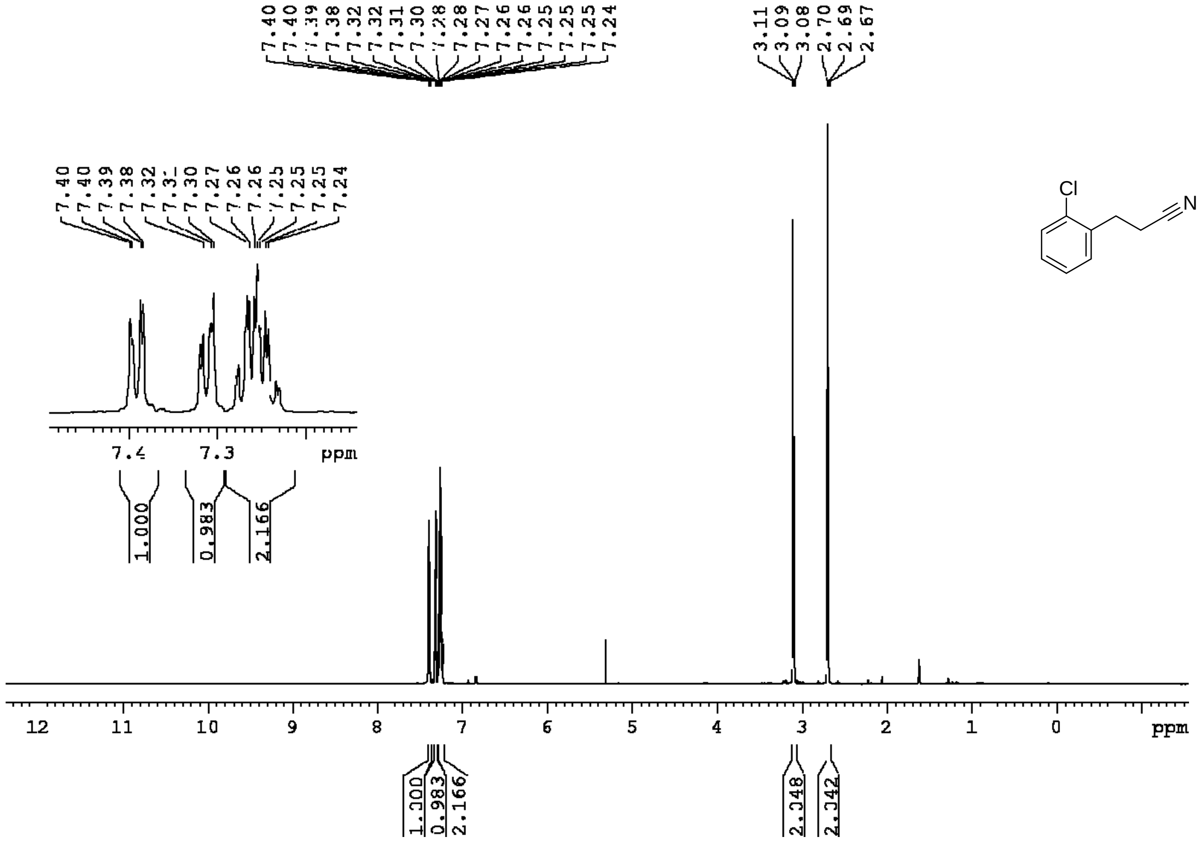

^{13}C-NMR (151 MHz, $CDCl_3$) 3-(2-Chlorophenyl)propanenitrile

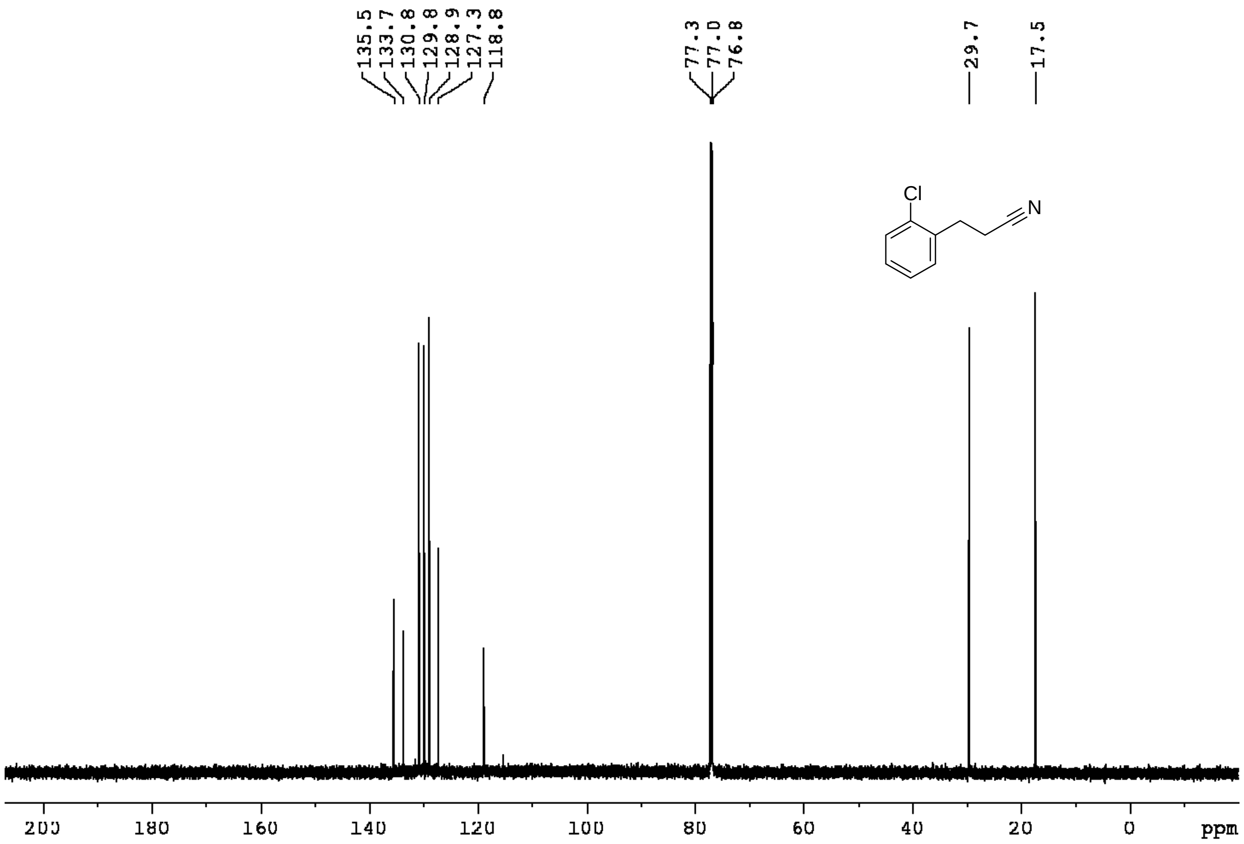

^{1}H-NMR (600 MHz, CDCl$_3$) *N*-(3-(2-chlorophenyl)propyl)-2,2,2-trifluoroacetamide (6d)

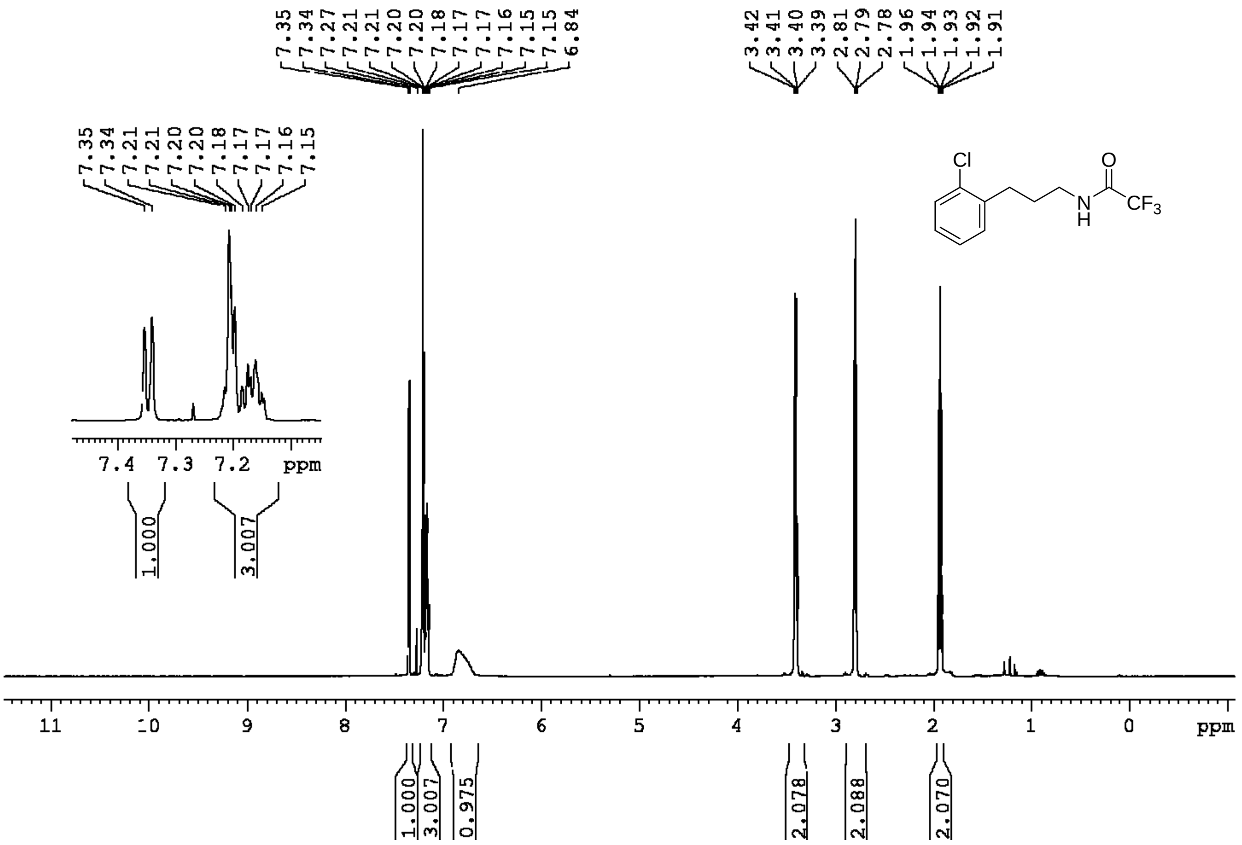

^{13}C-NMR (151 MHz, CDCl$_3$) *N*-(3-(2-chlorophenyl)propyl)-2,2,2-trifluoroacetamide (6d)

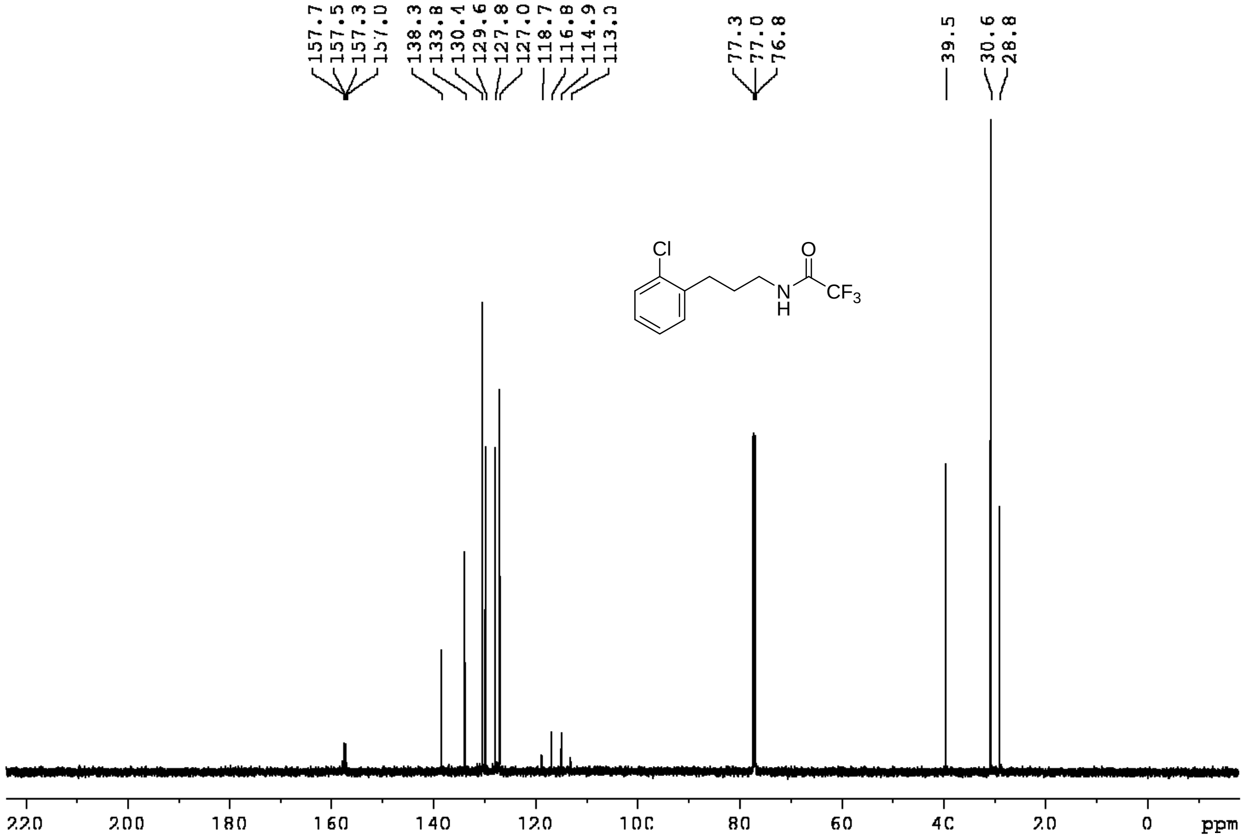

^{1}H-NMR (600 MHz, $CDCl_3$) 3-(3-Fluorophenyl)acrylonitrile

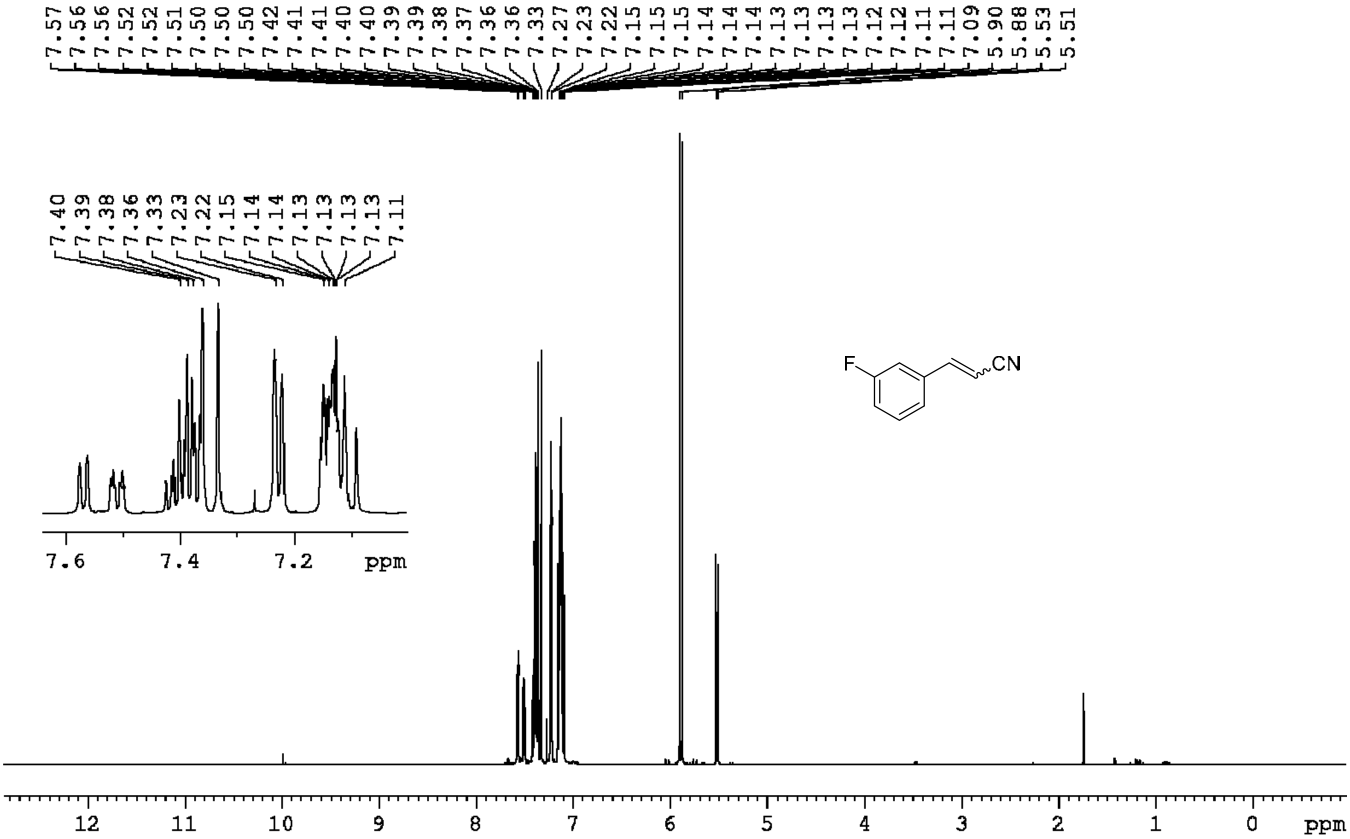

^{13}C-NMR (151 MHz, $CDCl_3$) 3-(3-Fluorophenyl)acrylonitrile

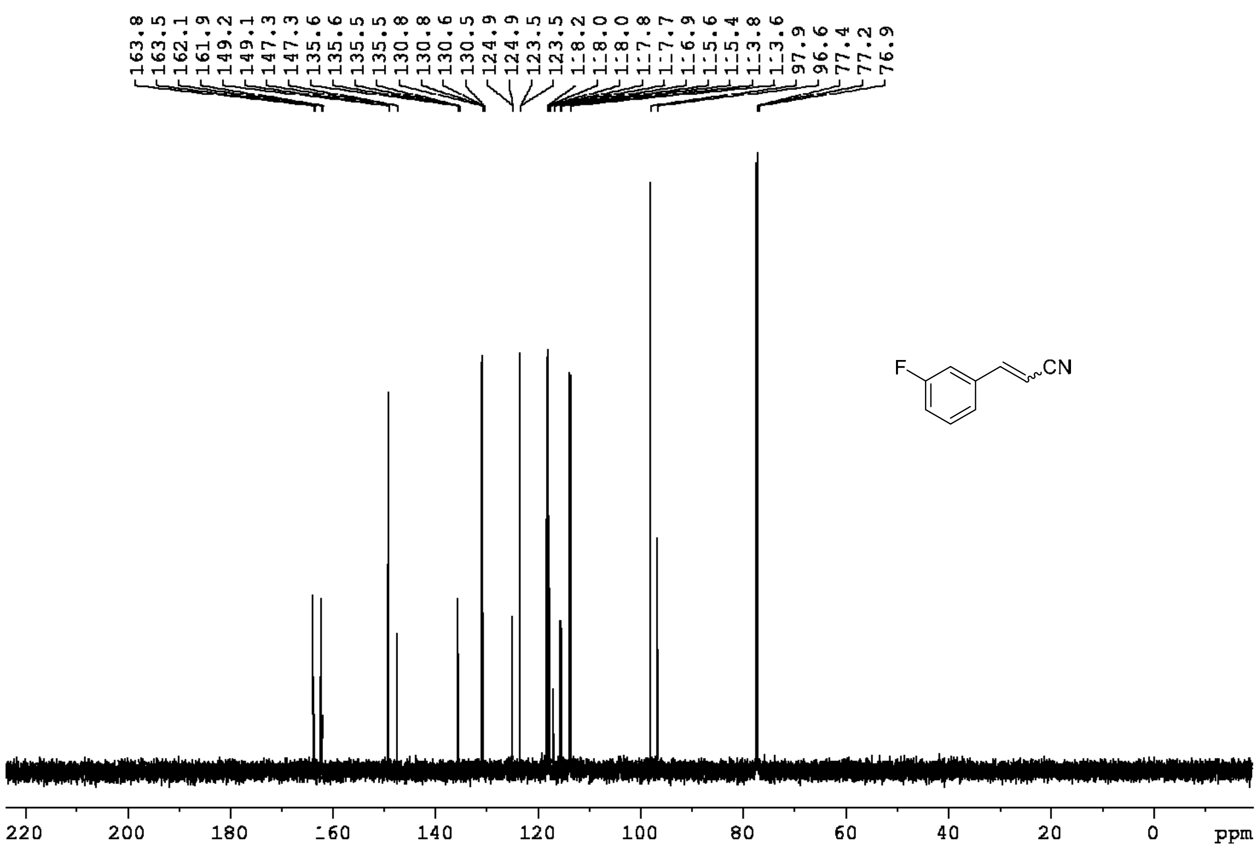

^{1}H-NMR (600 MHz, $CDCl_3$) 3-(2-Fluorophenyl)acrylonitrile

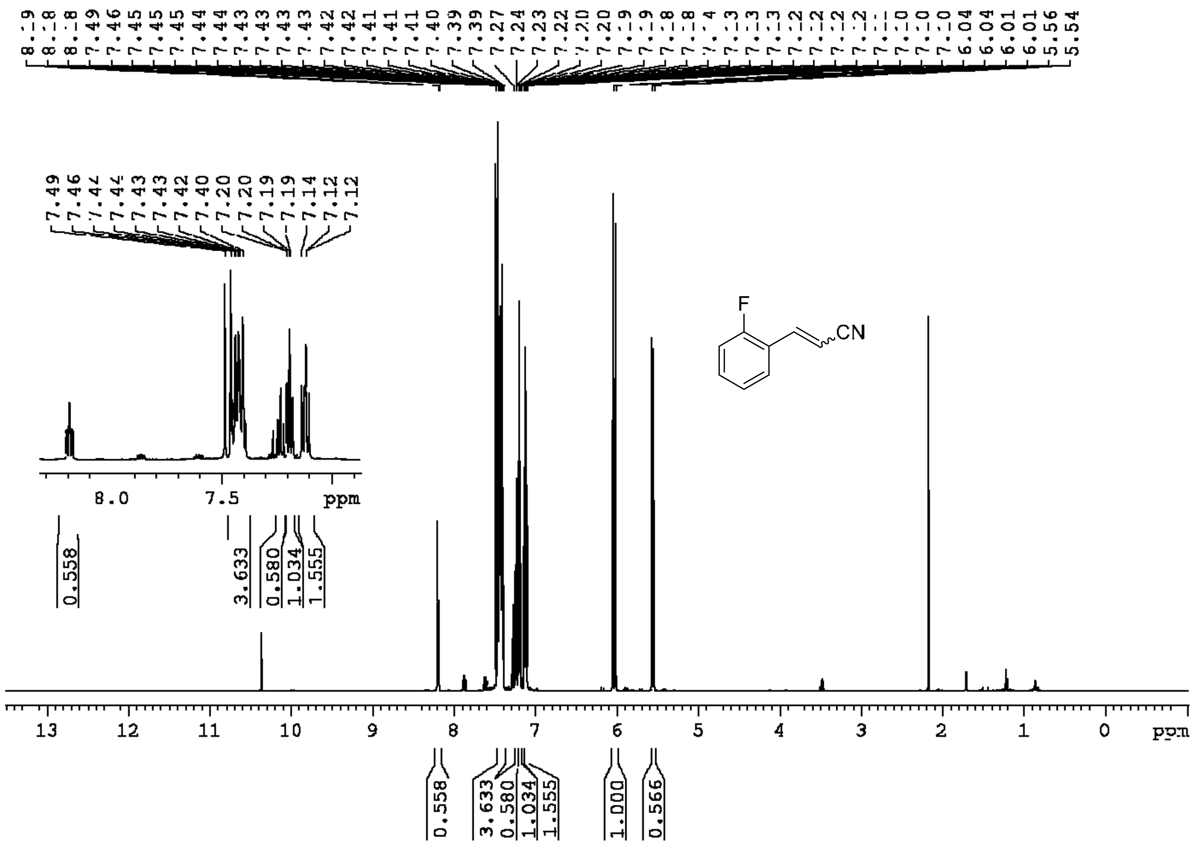

^{13}C-NMR (151 MHz, $CDCl_3$) 3-(2-Fluorophenyl)acrylonitrile

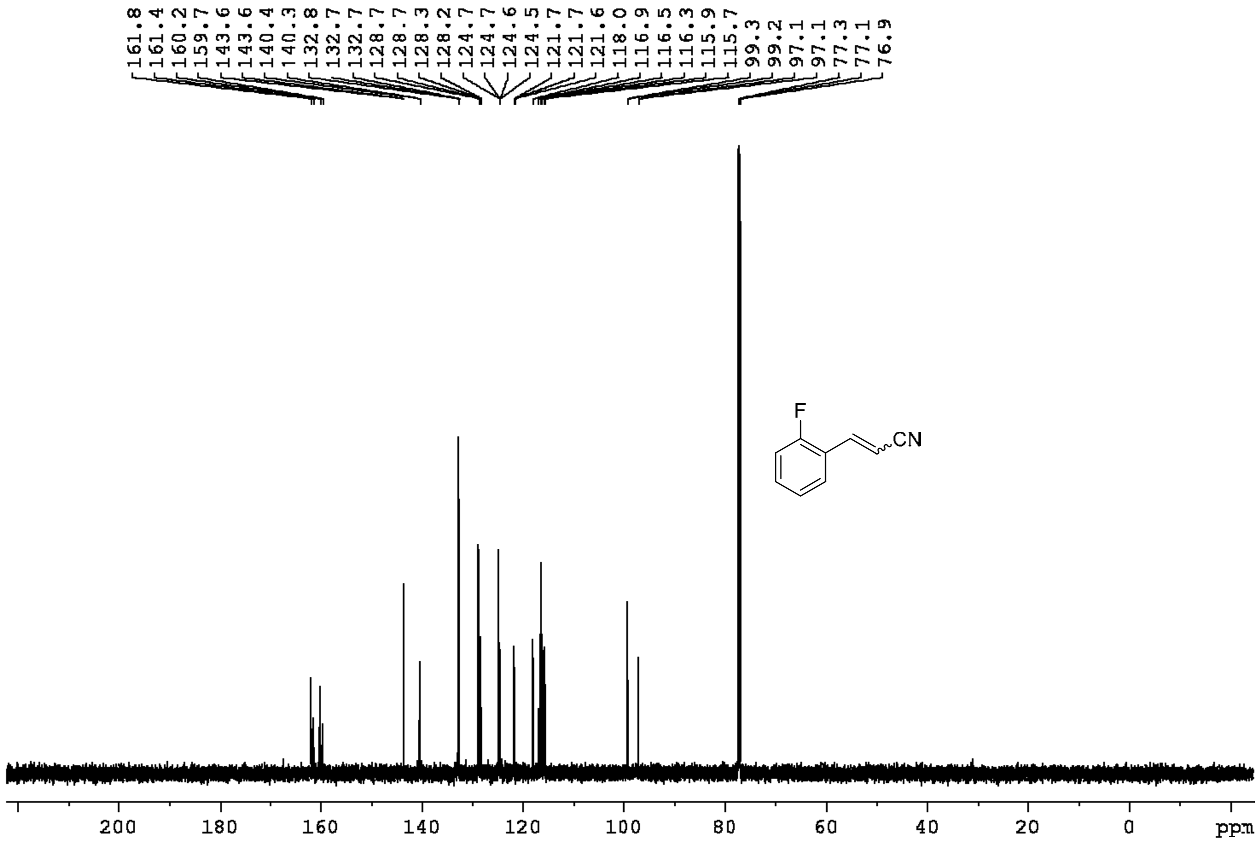

^{1}H-NMR (600 MHz, $CDCl_3$) *N*-(3-(2-fluorophenyl)propyl)-2,2,2-trifluoroacetamide (6e)

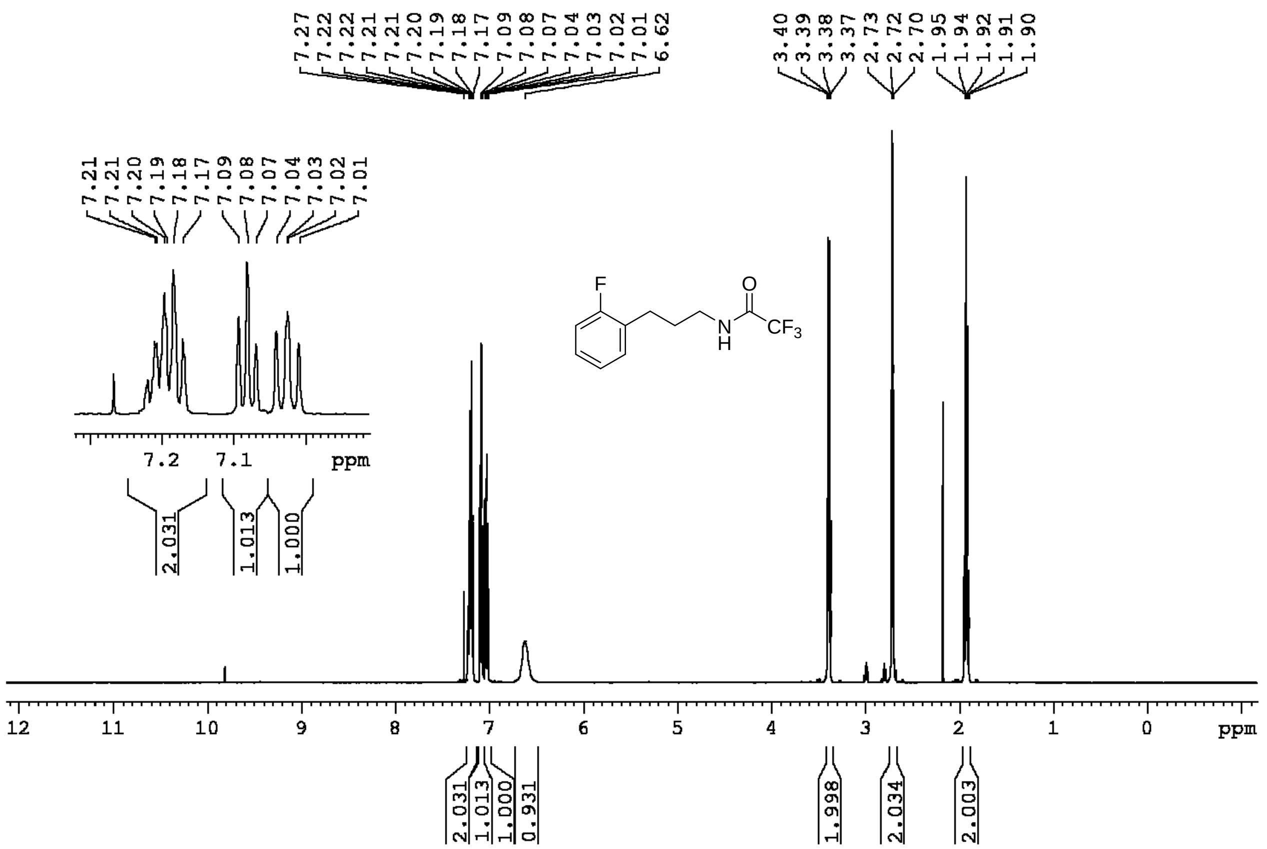

^{13}C-NMR (151 MHz, $CDCl_3$) *N*-(3-(2-fluorophenyl)propyl)-2,2,2-trifluoroacetamide (6e)

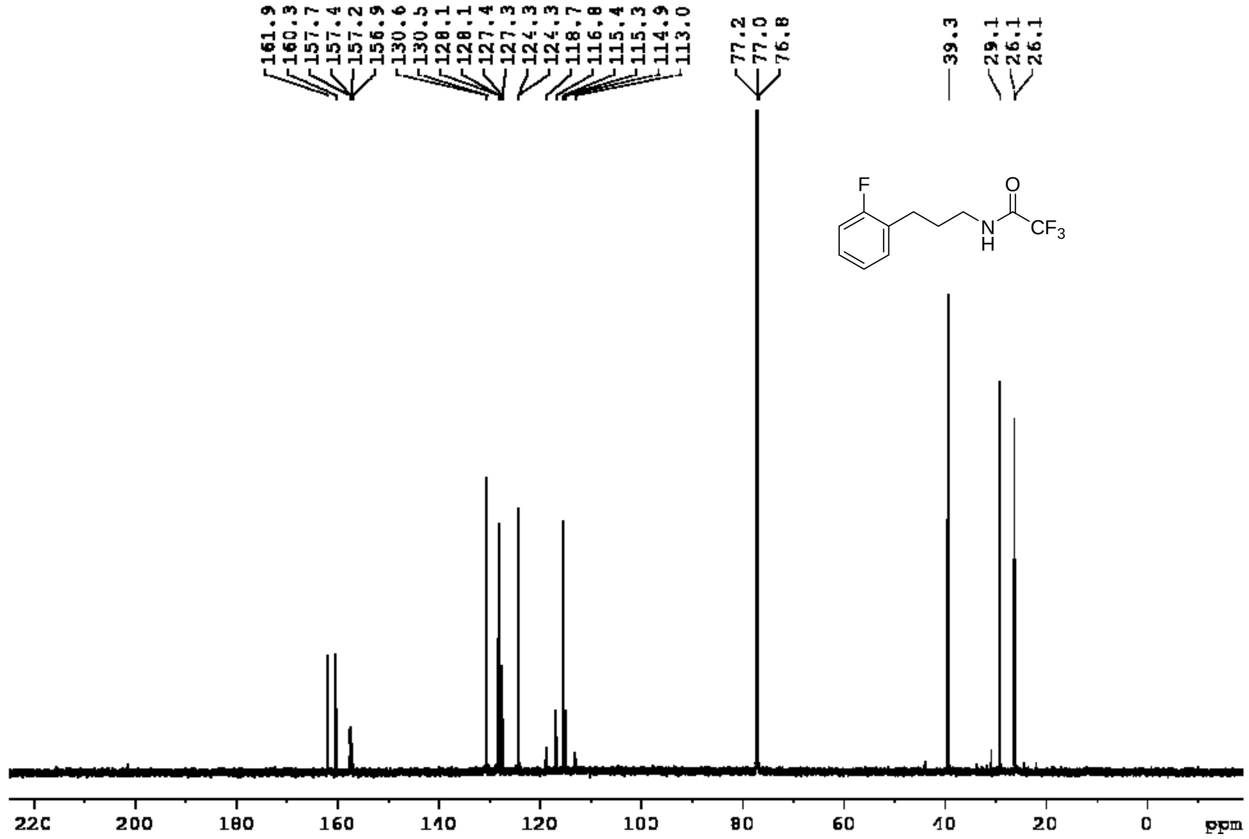

^{1}H-NMR (600 MHz, $CDCl_3$) *N*-(3-(3-fluorophenyl)propyl)-2,2,2-trifluoroacetamide (6f)

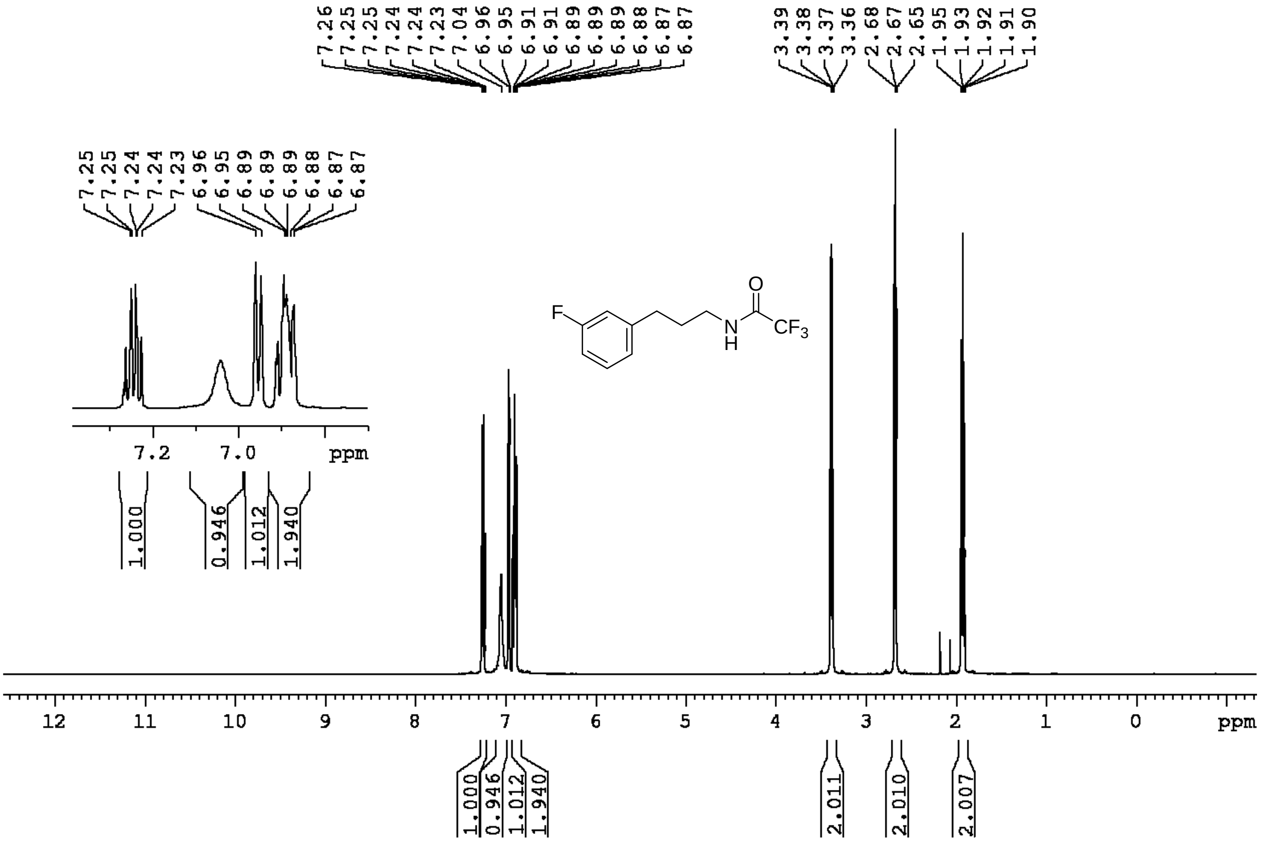

^{13}C-NMR (151 MHz, $CDCl_3$) *N*-(3-(3-fluorophenyl)propyl)-2,2,2-trifluoroacetamide (6f)

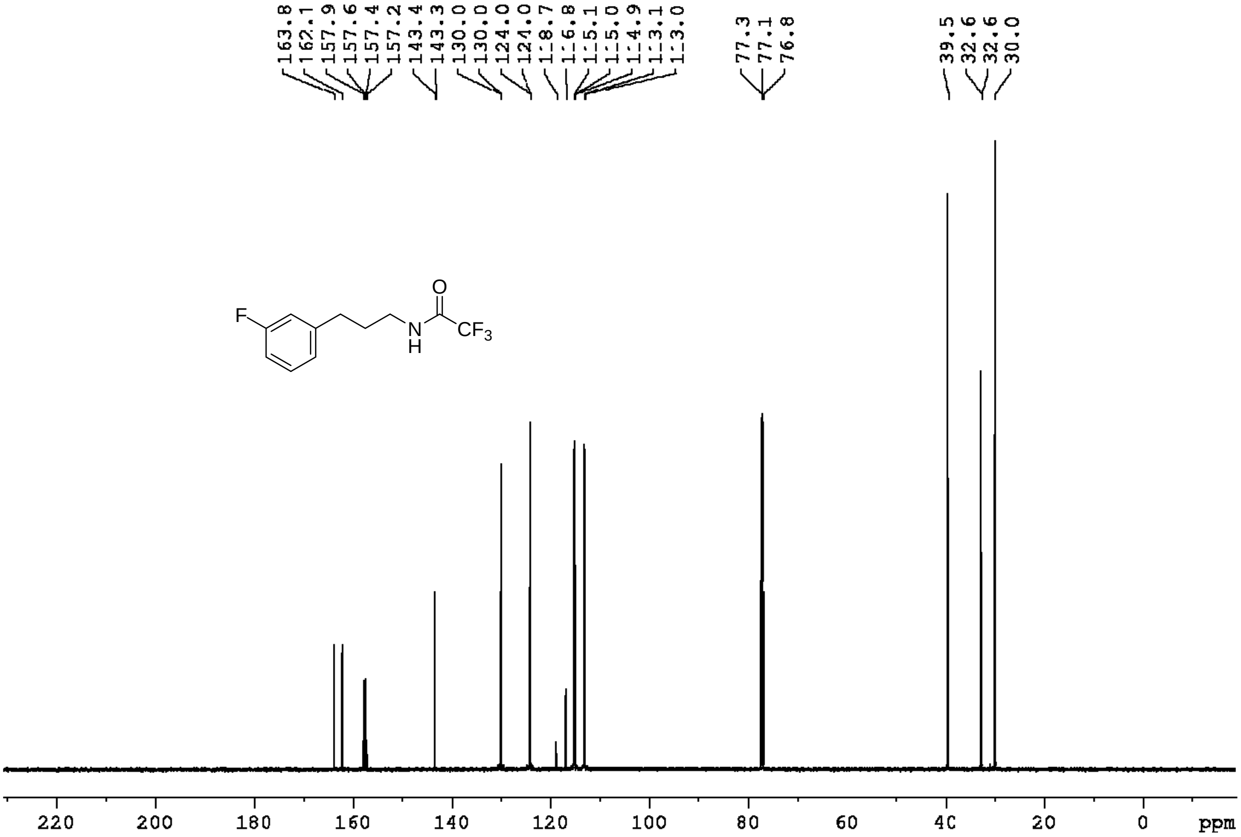

^{1}H-NMR (400 MHz, $CDCl_3$) Methyl 2-(2-cyanovinyl)benzoate

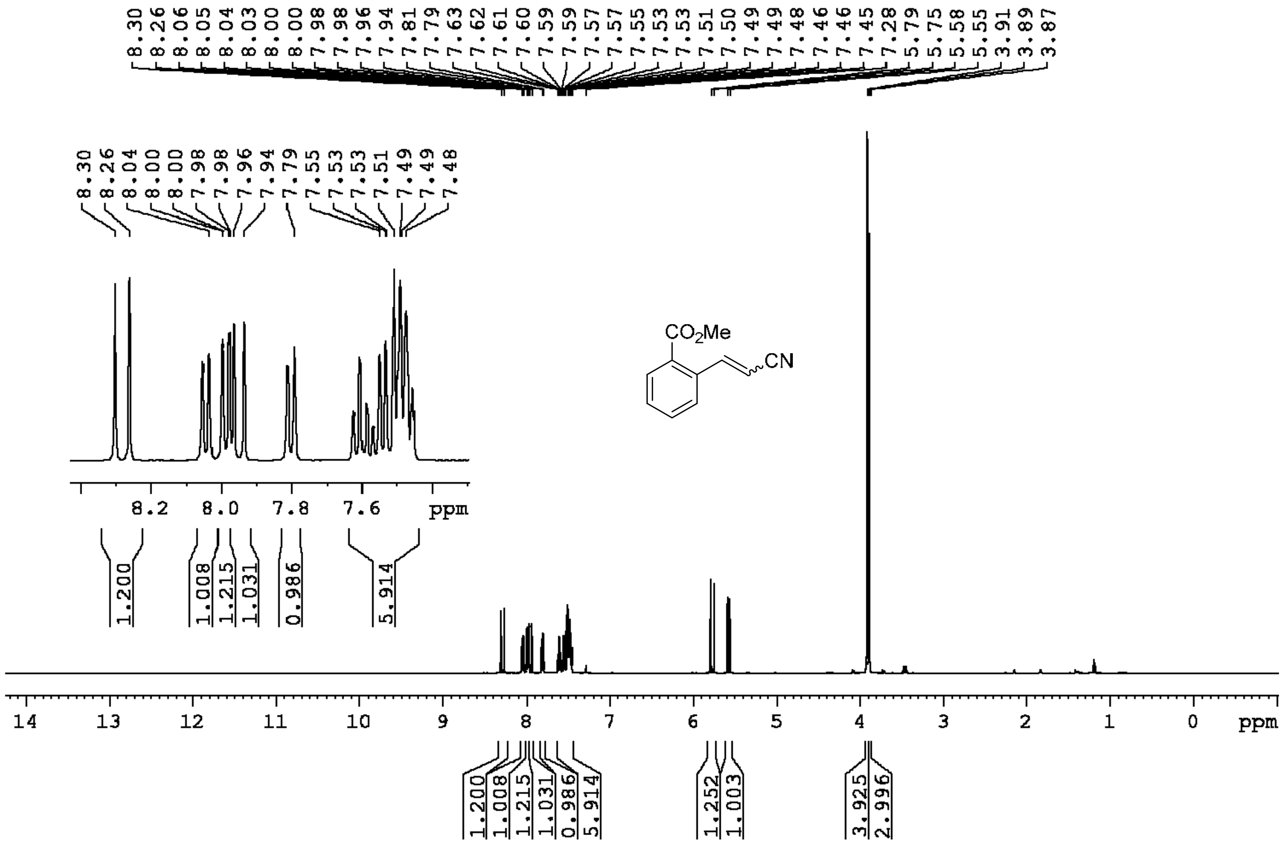

^{13}C-NMR (100 MHz, $CDCl_3$) Methyl 2-(2-cyanovinyl)benzoate

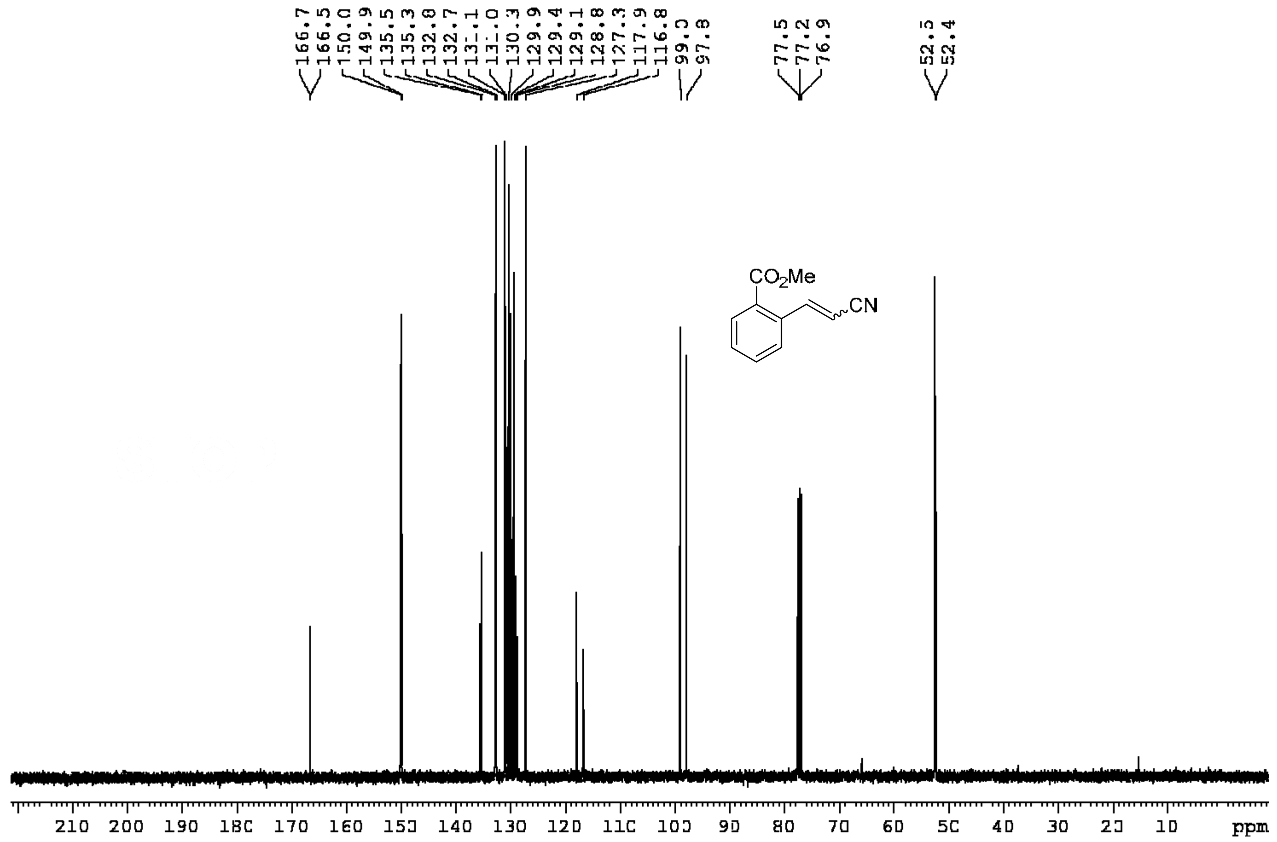

^{1}H-NMR (600 MHz, $CDCl_3$) Methyl 2-(3-(2,2,2-trifluoroacetamido)propyl)benzoate (6g)

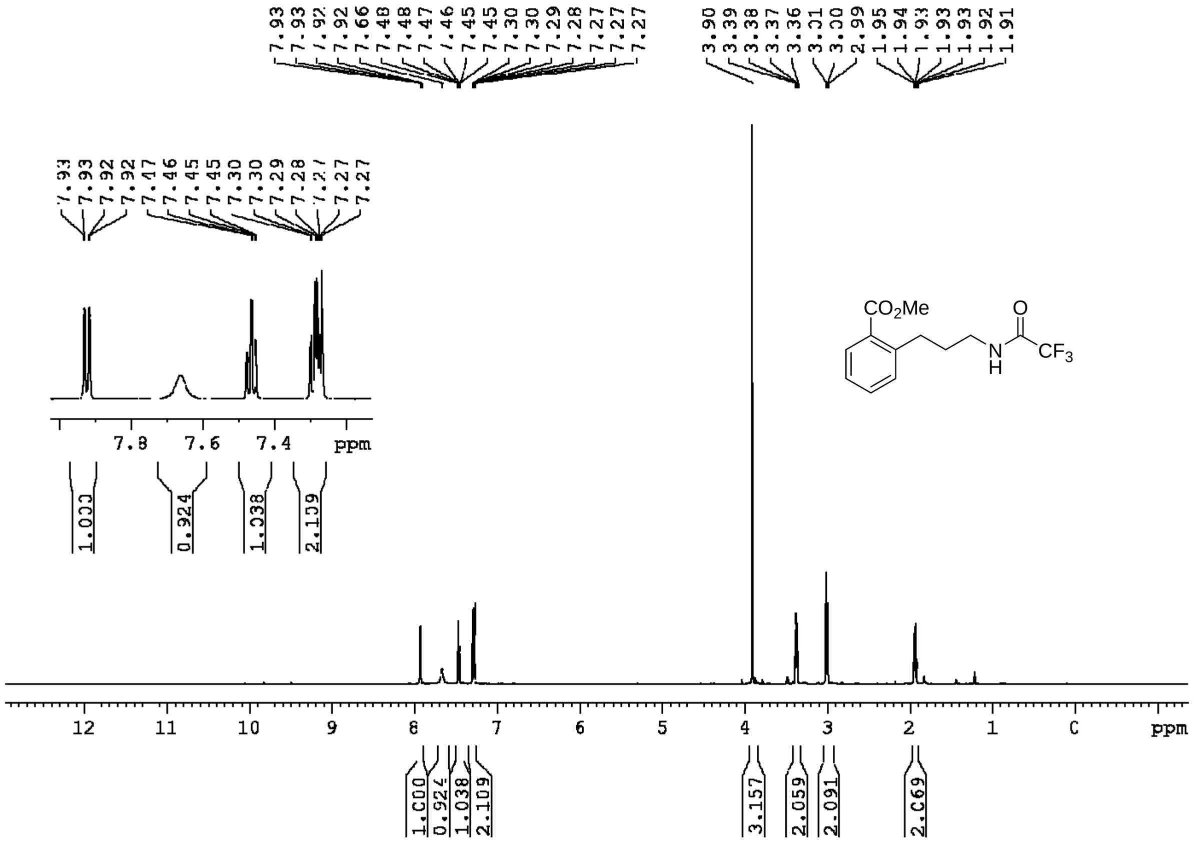

^{13}C-NMR (151 MHz, $CDCl_3$) Methyl 2-(3-(2,2,2-trifluoroacetamido)propyl)benzoate (6g)

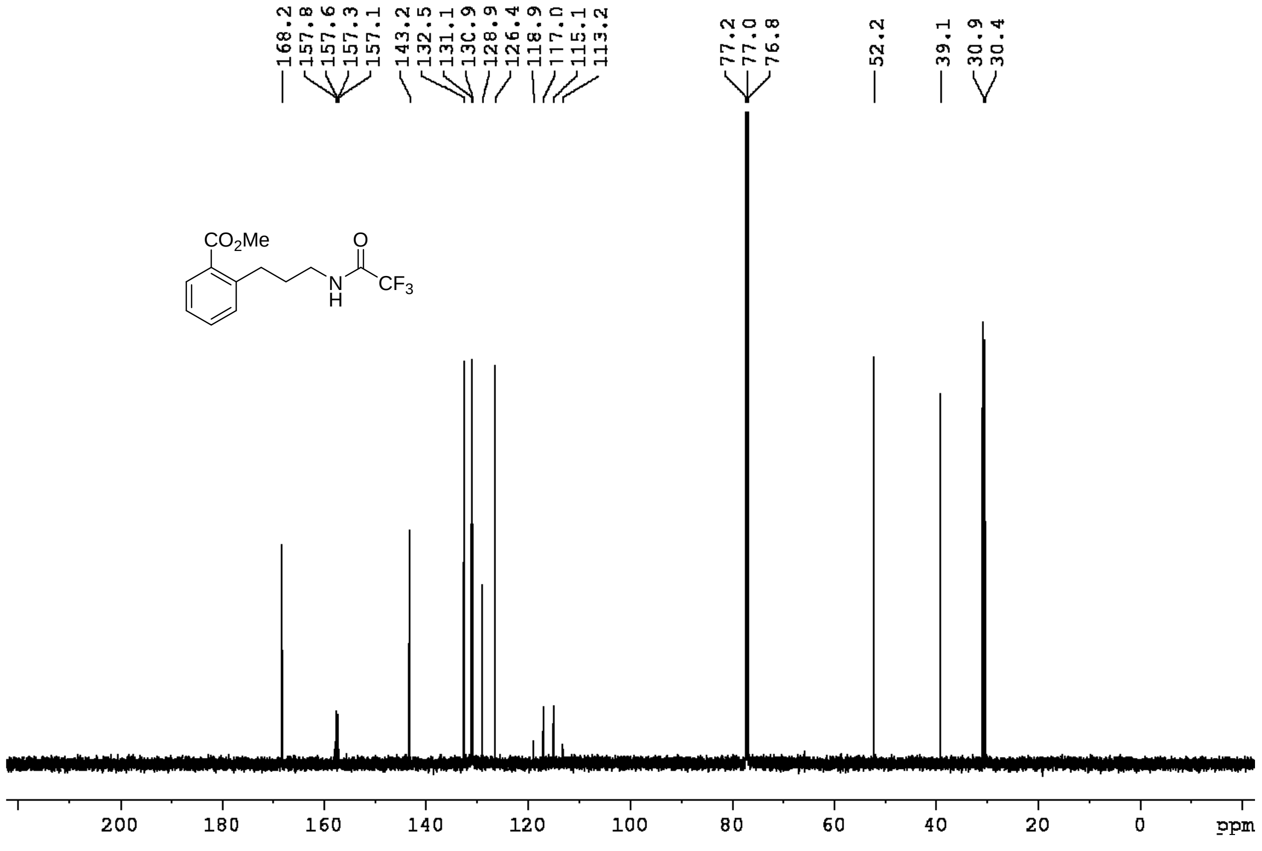

^{1}H-NMR (600 MHz, $CDCl_3$) *N*-(3-(2,5-difluorophenyl)propyl)-2,2,2-trifluoroacetamide (6h)

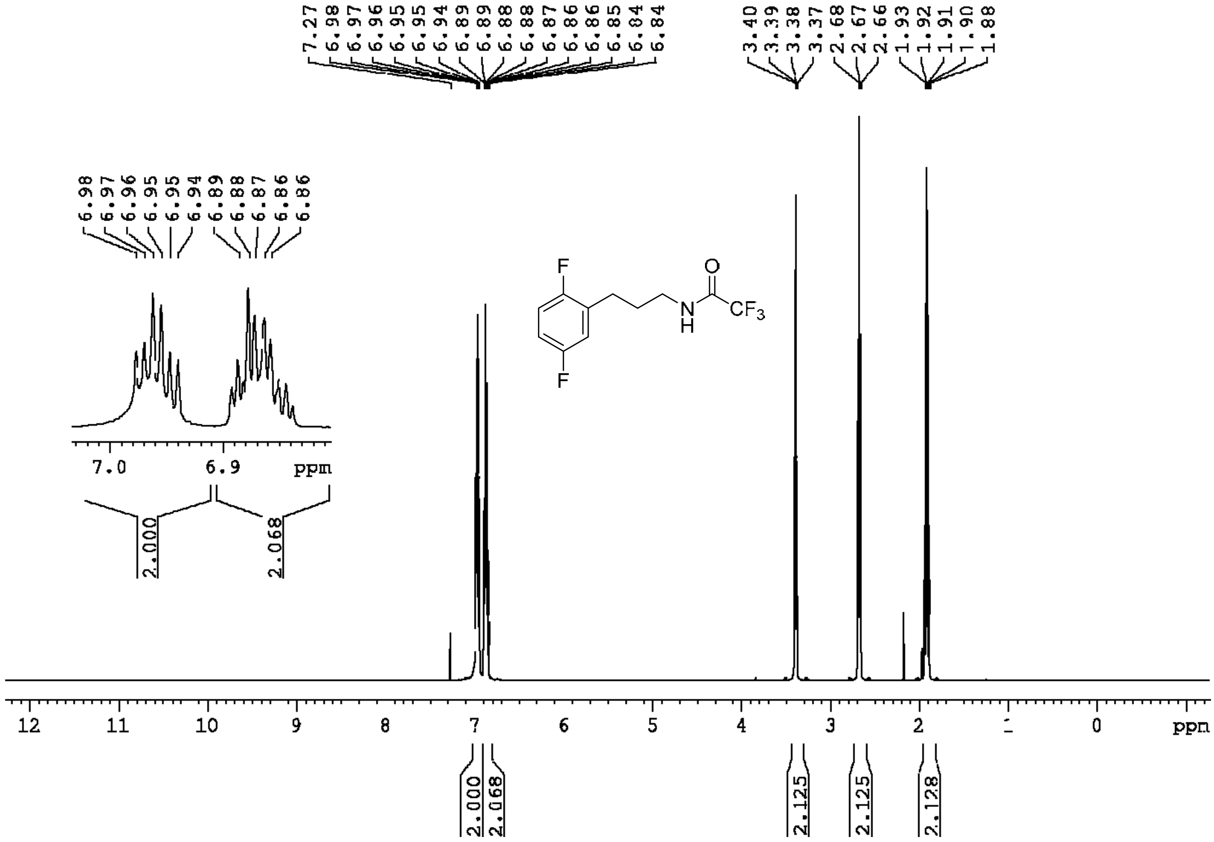

^{13}C-NMR (151 MHz, $CDCl_3$) *N*-(3-(2,5-difluorophenyl)propyl)-2,2,2-trifluoroacetamide (6h)

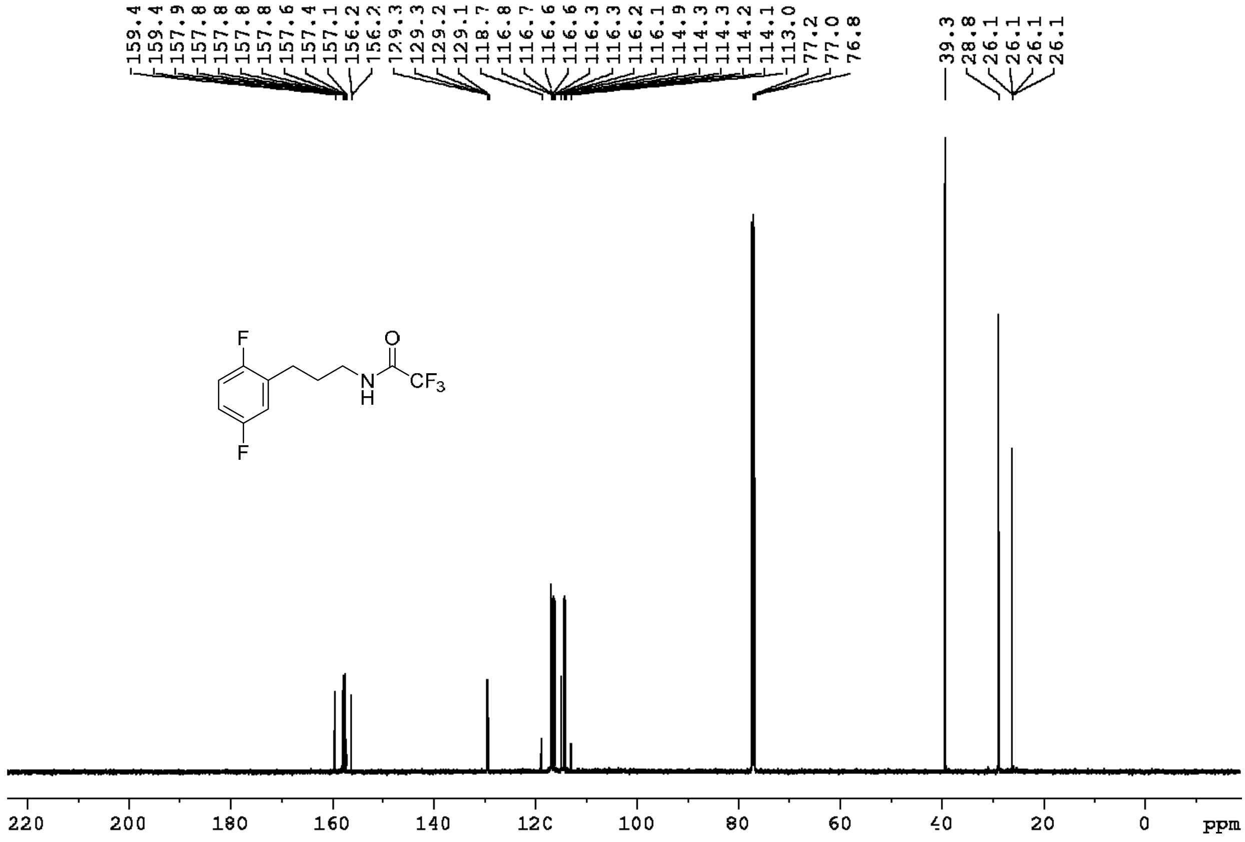

^{1}H-NMR (400 MHz, $CDCl_3$) 3-(o-tolyl)acrylonitrile

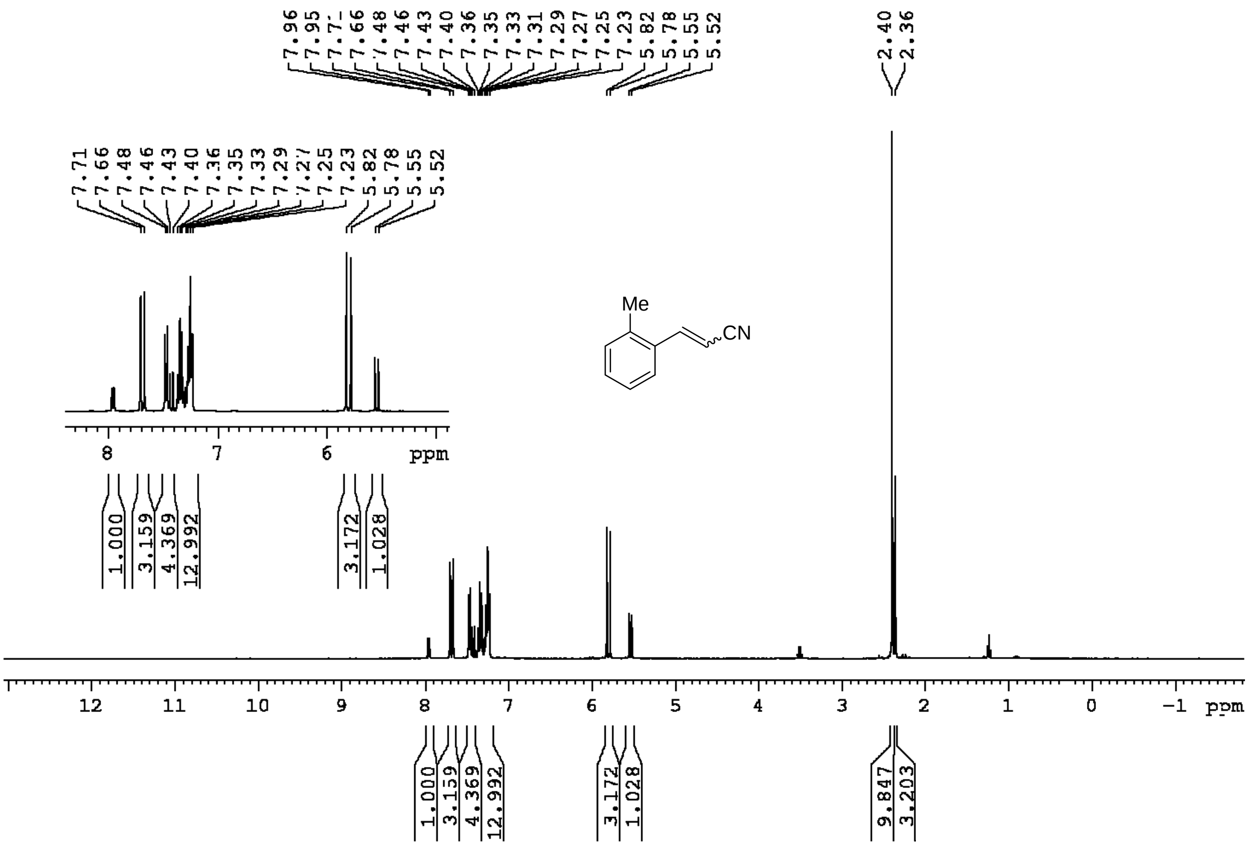

^{13}C-NMR (100 MHz, $CDCl_3$) 3-(o-tolyl)acrylonitrile

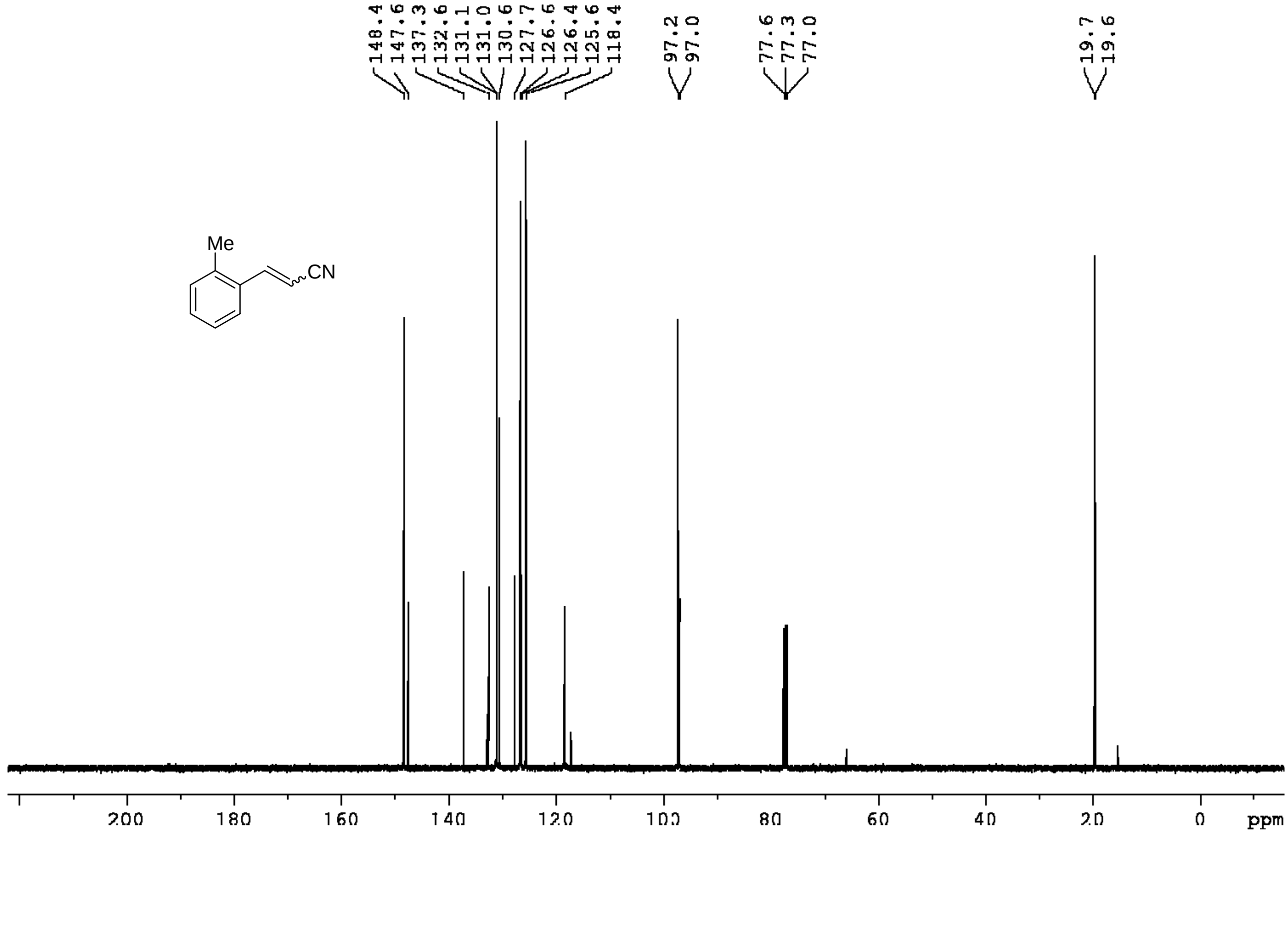

^{1}H-NMR (600 MHz, $CDCl_3$) 2,2,2-Trifluoro-N-(3-(o-tolyl)propyl)acetamide (6i)

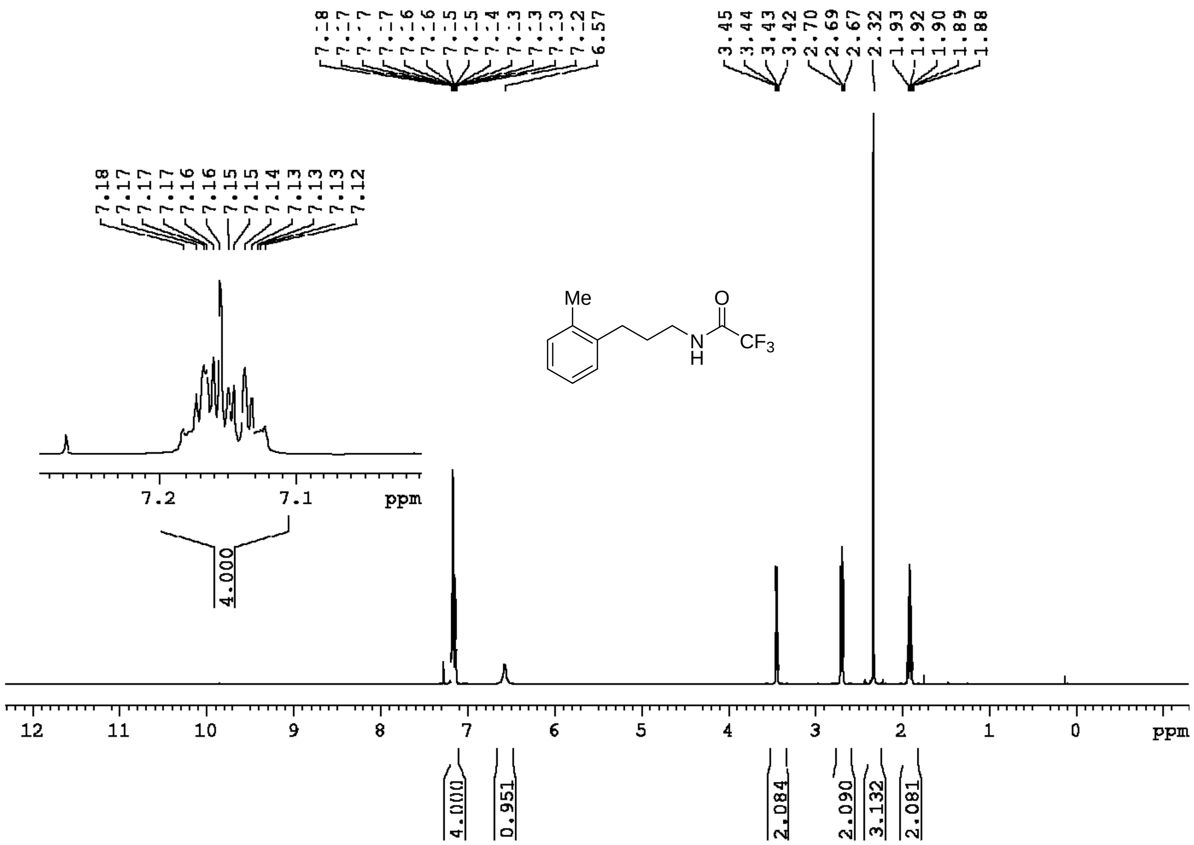

^{13}C-NMR (600 MHz, $CDCl_3$) 2,2,2-Trifluoro-N-(3-(o-tolyl)propyl)acetamide (6i)

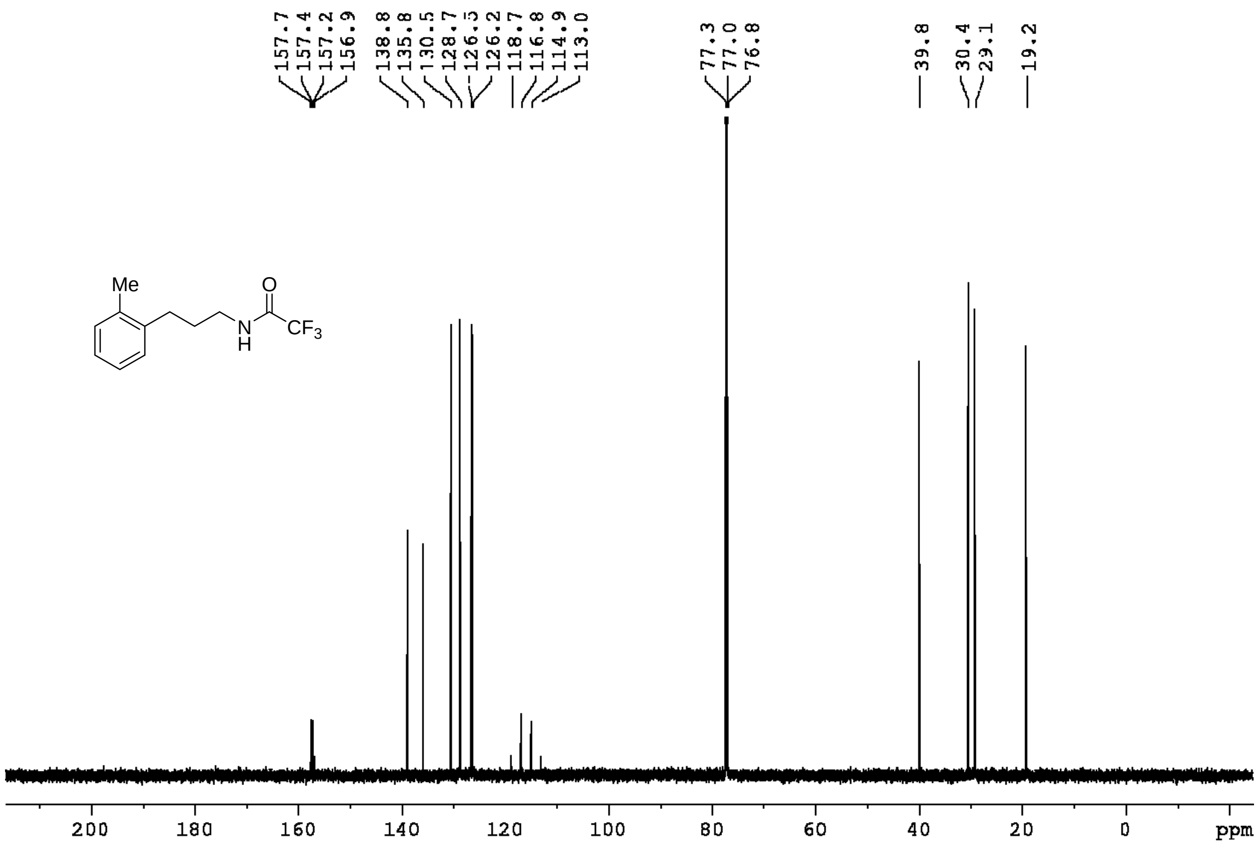

^{1}H-NMR (600 MHz, $CDCl_3$) 2,2,2-Trifluoro-*N*-(2-methoxybenzyl)acetamide (6j)

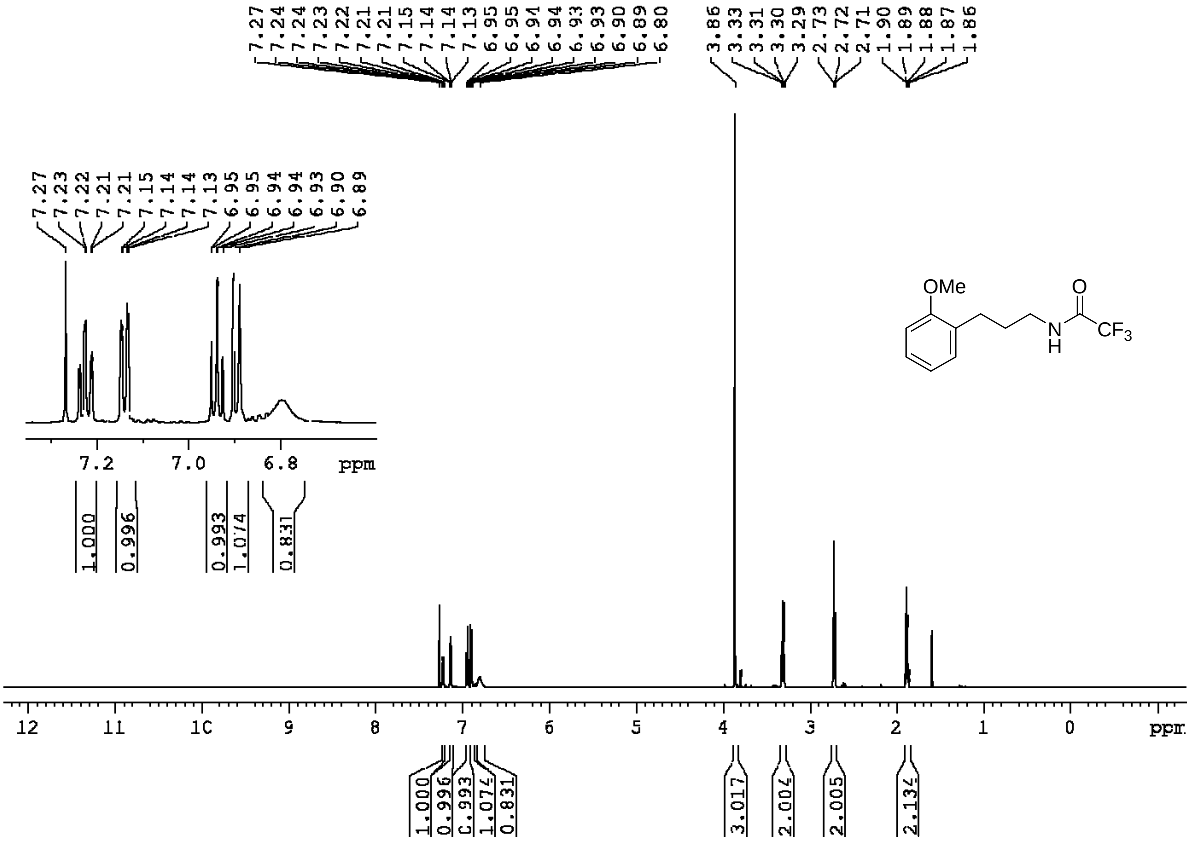

^{13}C-NMR (151 MHz, $CDCl_3$) 2,2,2-Trifluoro-*N*-(2-methoxybenzyl)acetamide (6j)

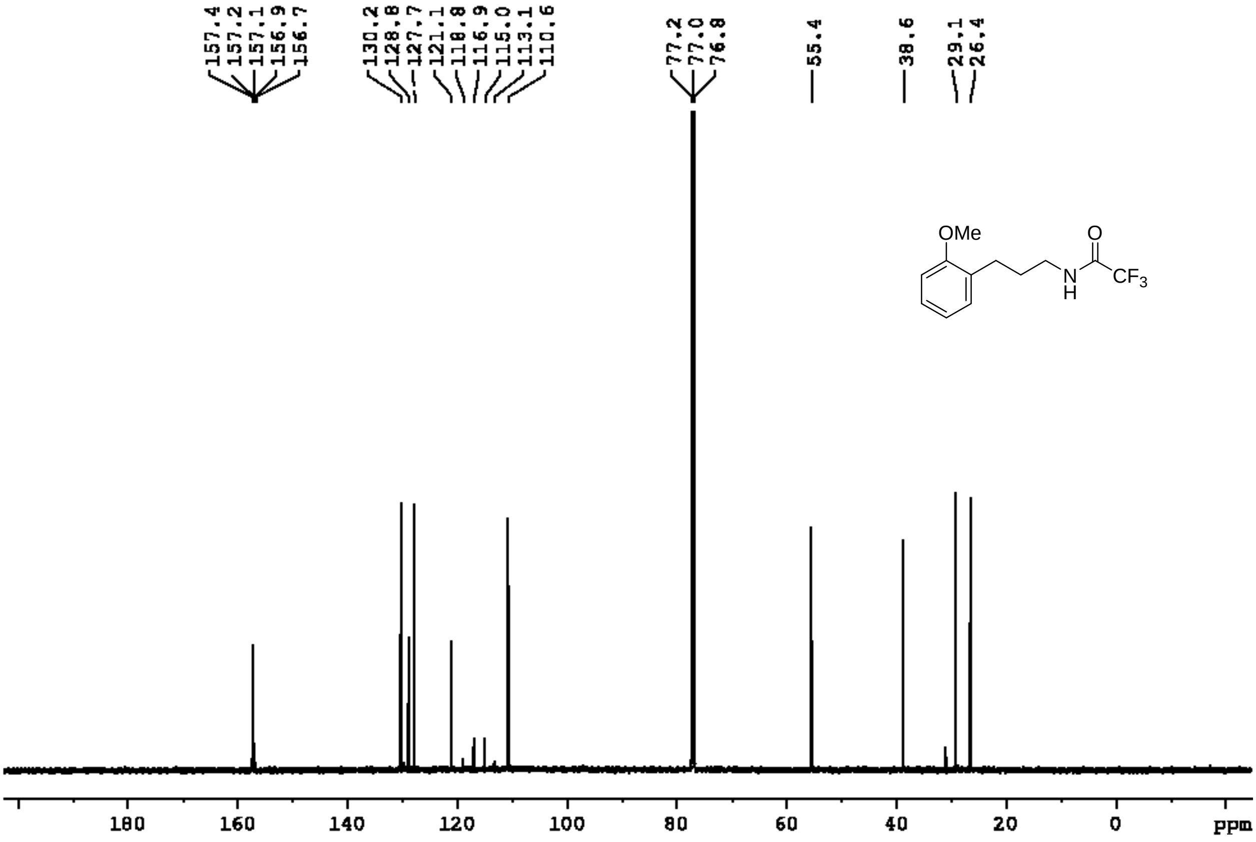

^{1}H-NMR (600 MHz, $CDCl_3$) 2,2,2-Trifluoro-*N*-(3-(4'-methyl-[1,1'-biphenyl]-2-yl)propyl)acetamide (6k)

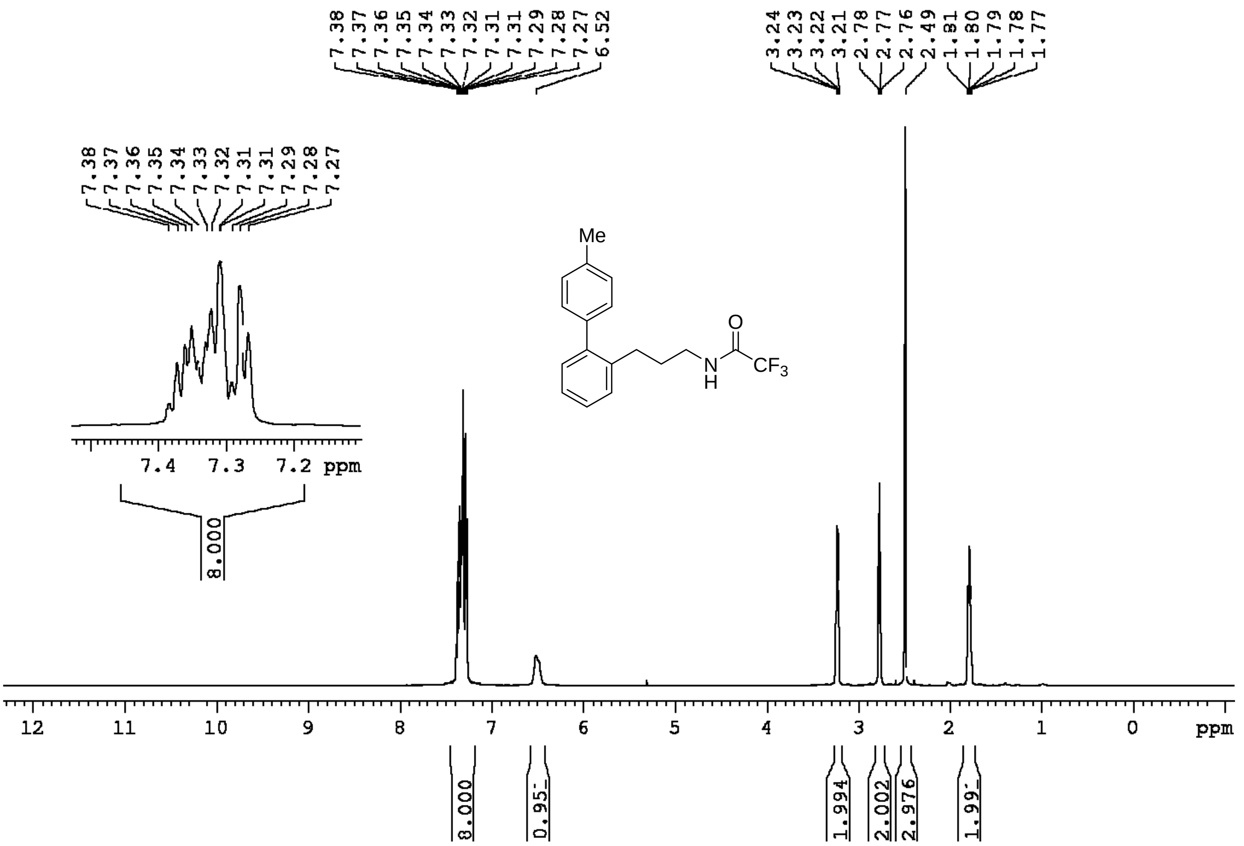

^{13}C-NMR (151 MHz, $CDCl_3$) 2,2,2-Trifluoro-*N*-(3-(4'-methyl-[1,1'-biphenyl]-2-yl)propyl)acetamide (6k)

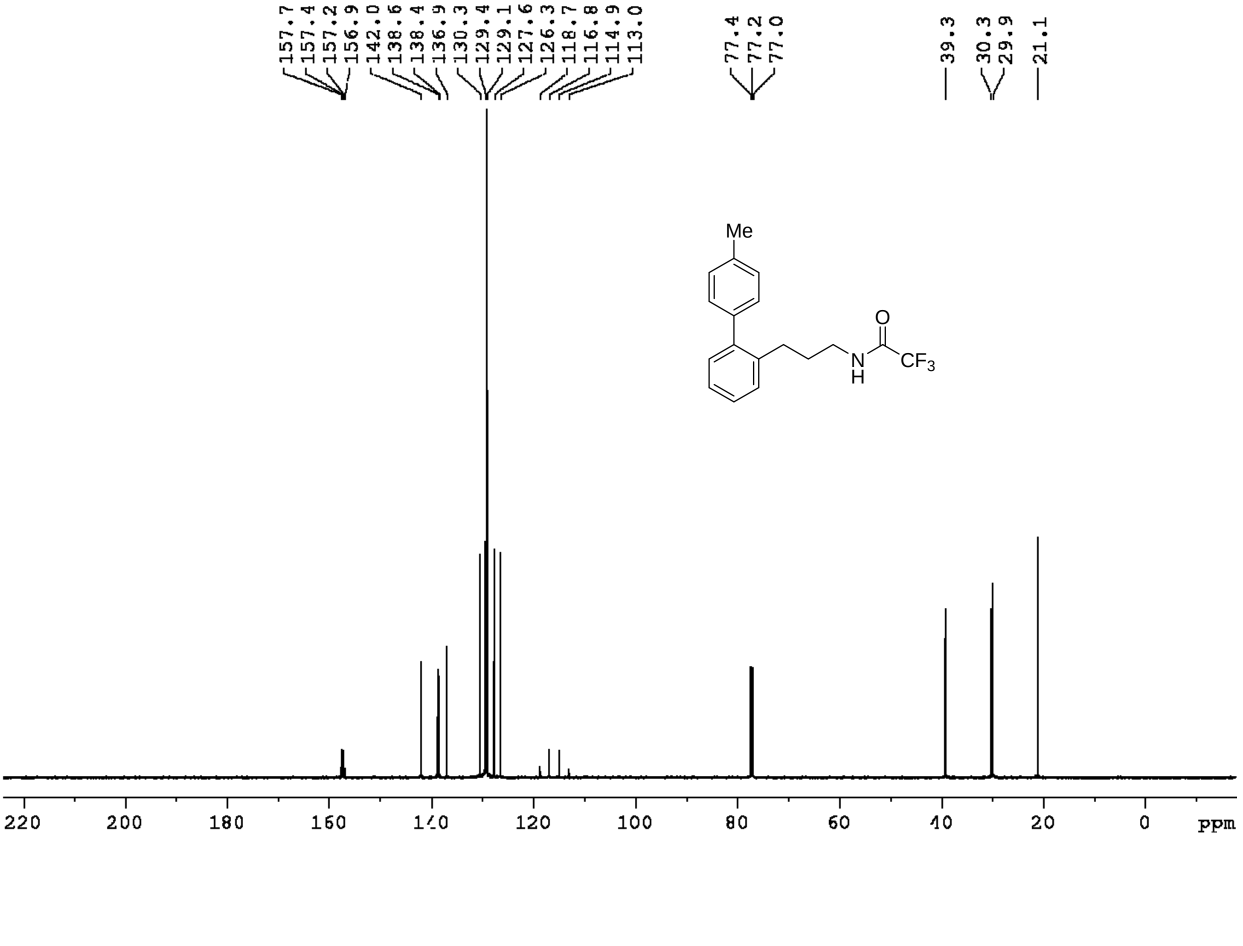

^{1}H-NMR (600 MHz, $CDCl_3$) 2,2,2-Trifluoro-*N*-(3-(pyridin-2-yl)propyl)acetamide (6l)

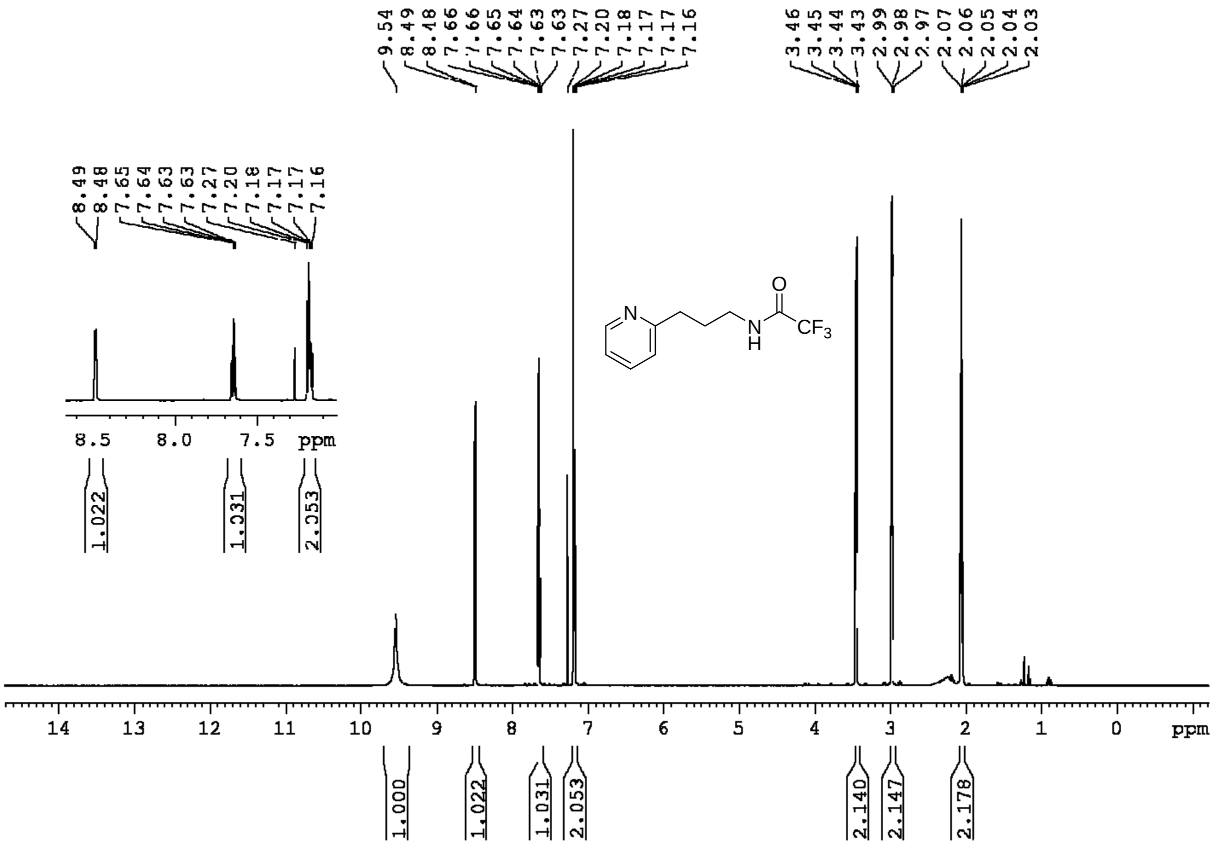

^{13}C-NMR (151 MHz, $CDCl_3$) 2,2,2-Trifluoro-*N*-(3-(pyridin-2-yl)propyl)acetamide (6l)

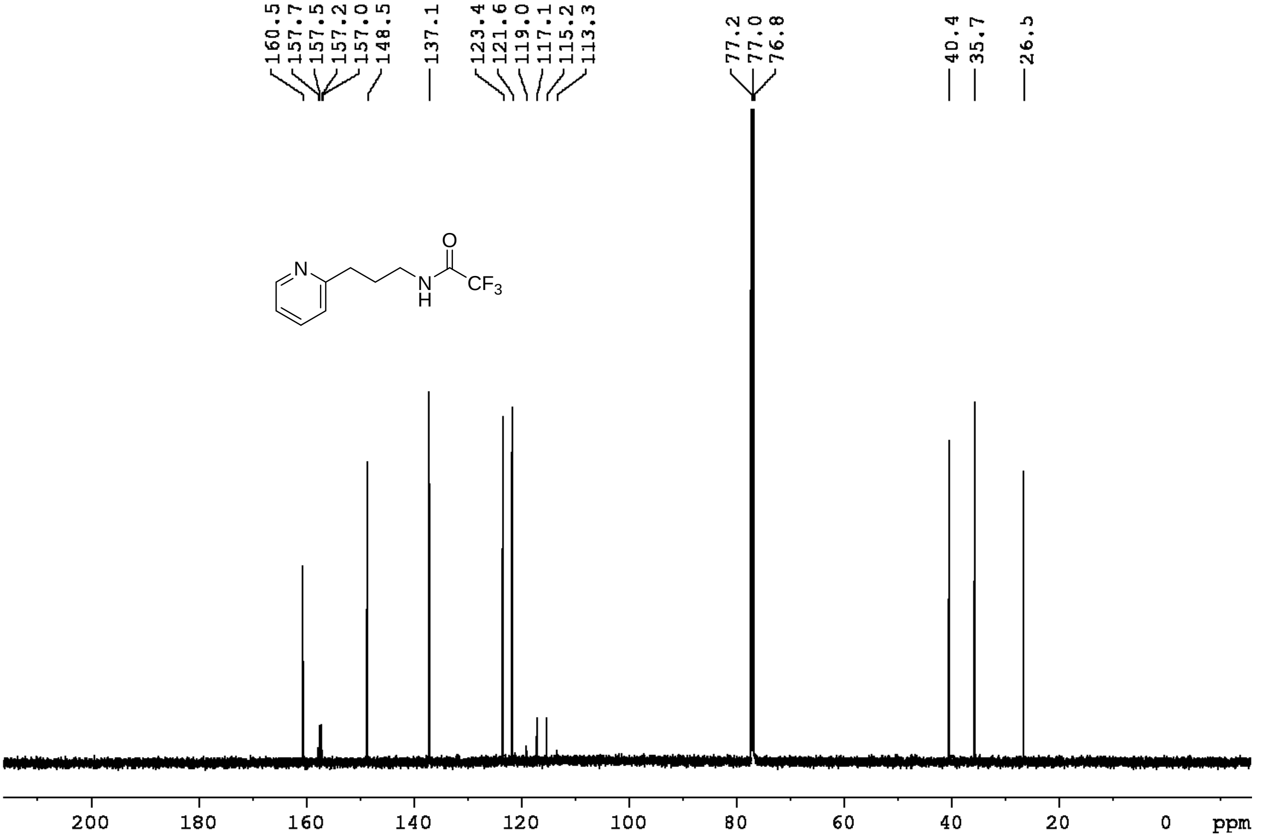

^{1}H-NMR (600 MHz, $CDCl_3$) 2,2,2-Trifluoro-*N*-(4-phenylbutyl)acetamide (8a)

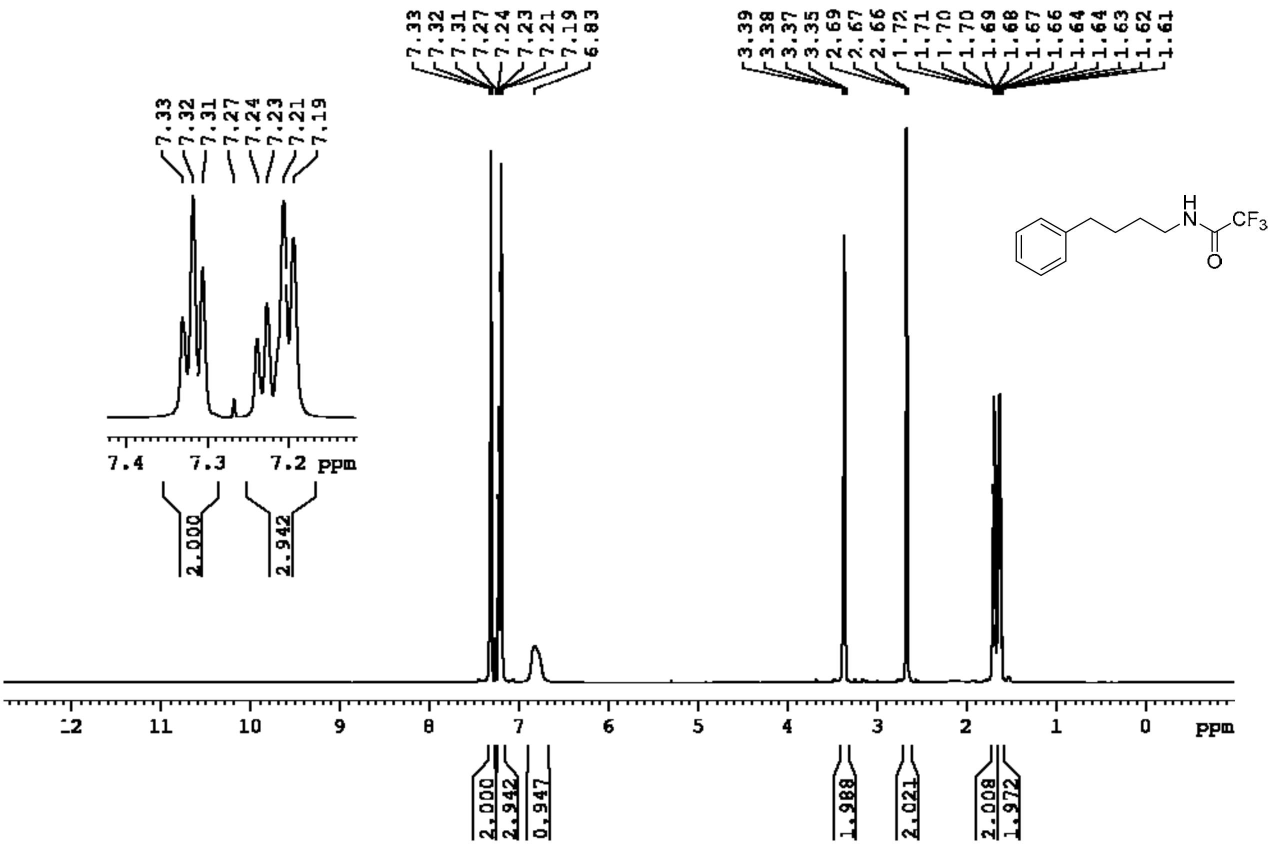

^{13}C-NMR (151 MHz, $CDCl_3$) 2,2,2-Trifluoro-*N*-(4-phenylbutyl)acetamide (8a)

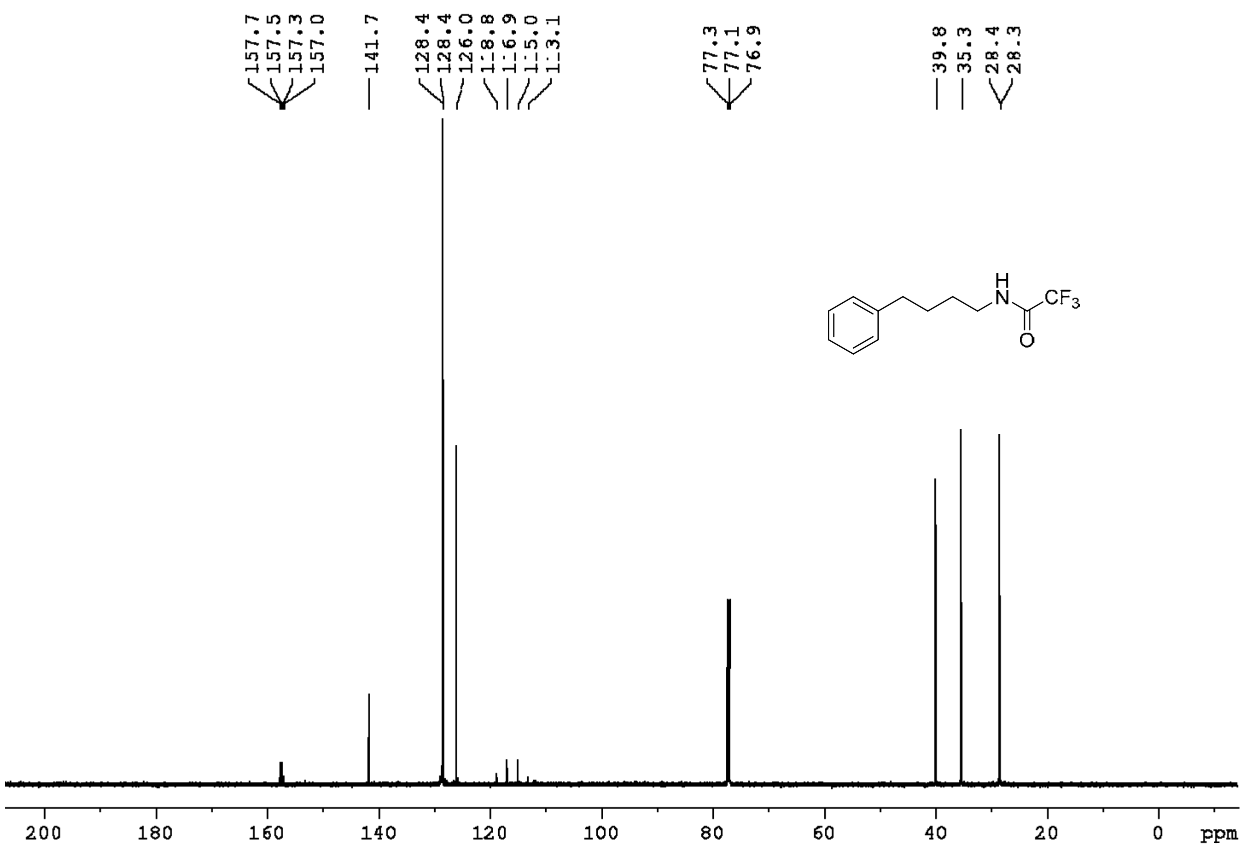

^{1}H-NMR (600 MHz, $CDCl_3$) 2,2,2-Trifluoro-*N*-(4-phenylbutyl)acetamide

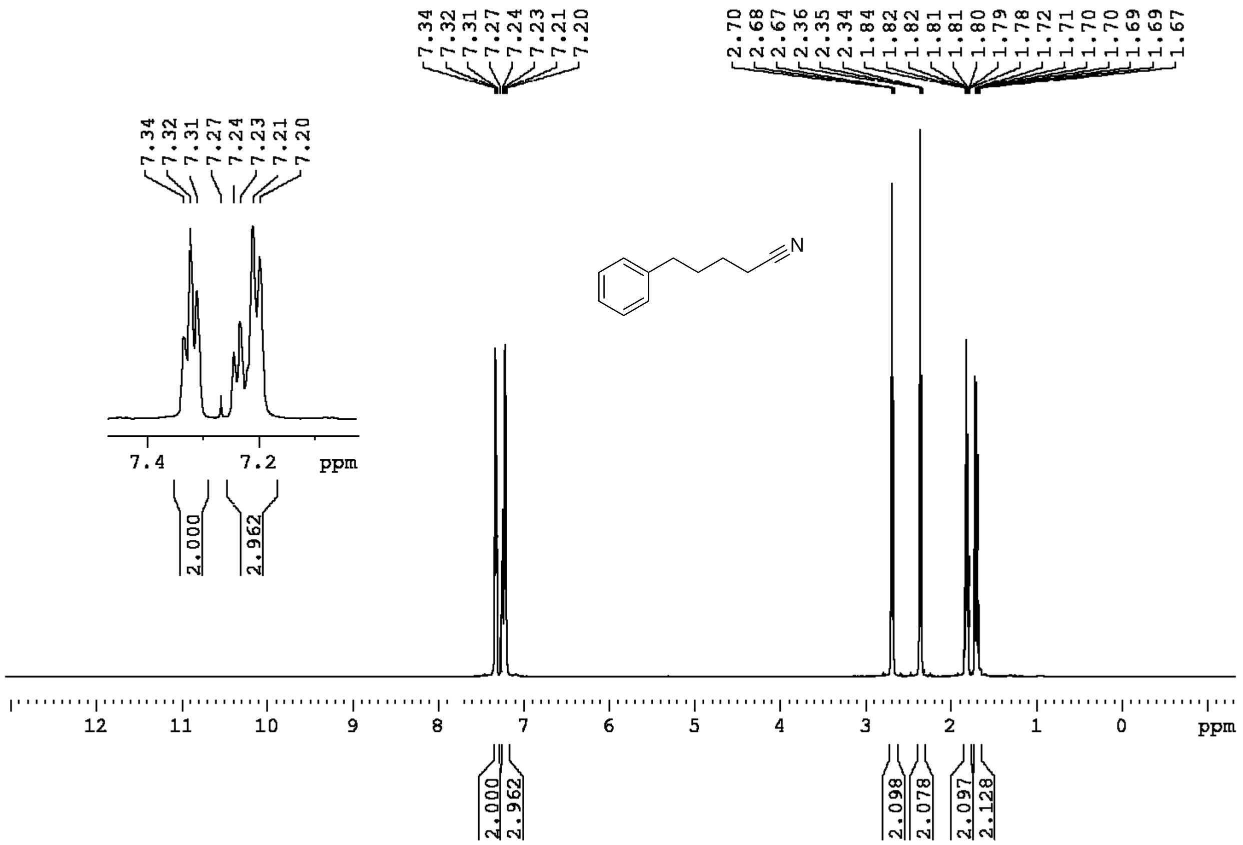

^{13}C-NMR (151 MHz, $CDCl_3$) 2,2,2-Trifluoro-*N*-(4-phenylbutyl)acetamide

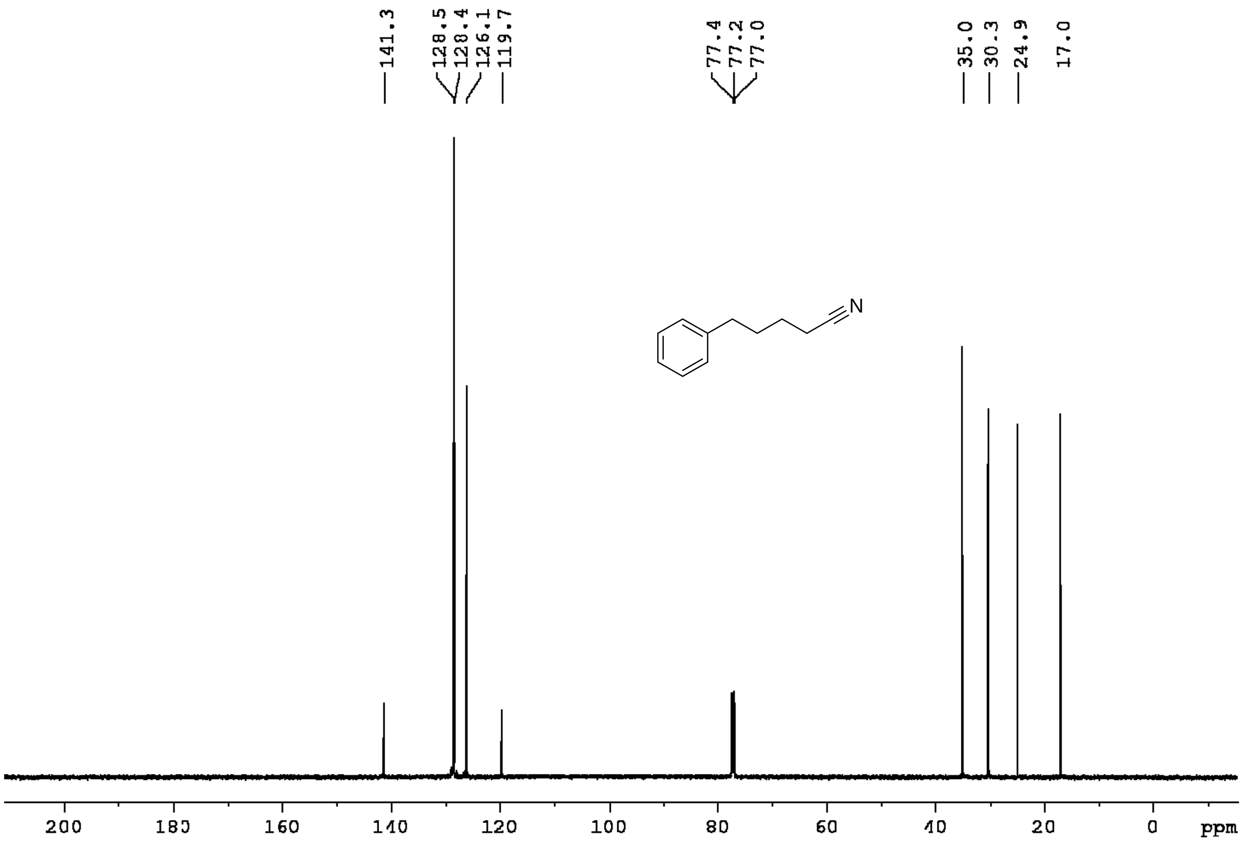

^{1}H-NMR (600 MHz, $CDCl_3$) 2,2,2-Trifluoro-*N*-(5-phenylpentyl)acetamide (9a)

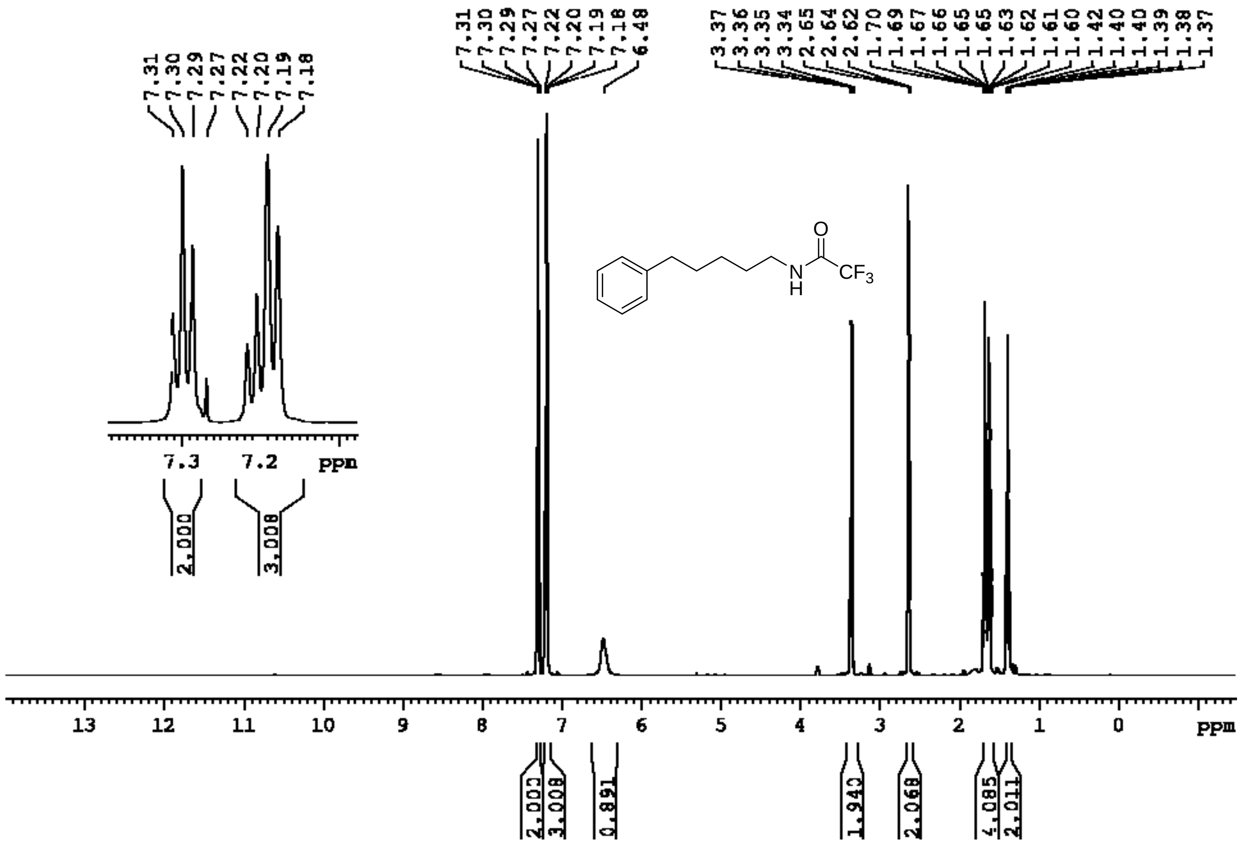

^{13}C-NMR (151 MHz, $CDCl_3$) 2,2,2-Trifluoro-*N*-(5-phenylpentyl)acetamide (9a)

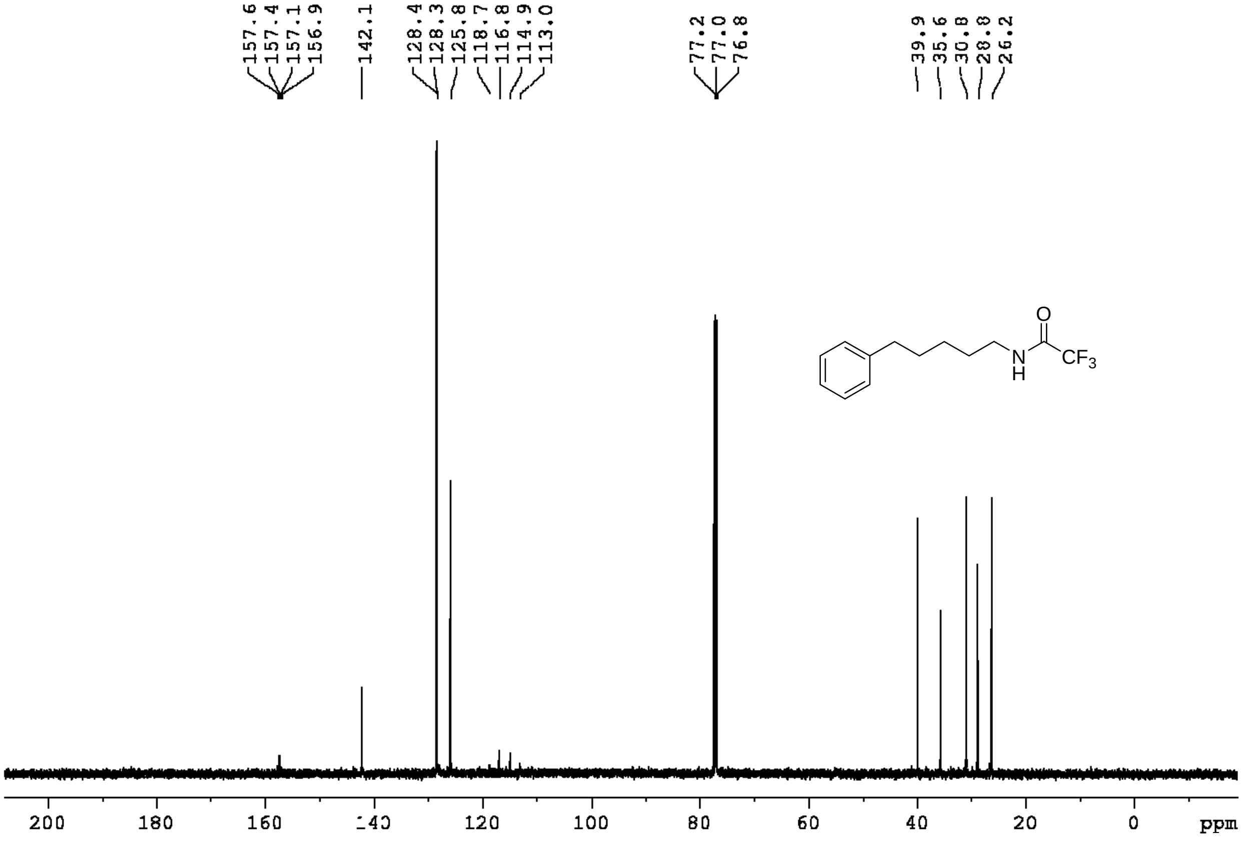

^{1}H-NMR (600 MHz, $CDCl_3$) 2-(6-phenylhexyl)isoindoline-1,3-dione

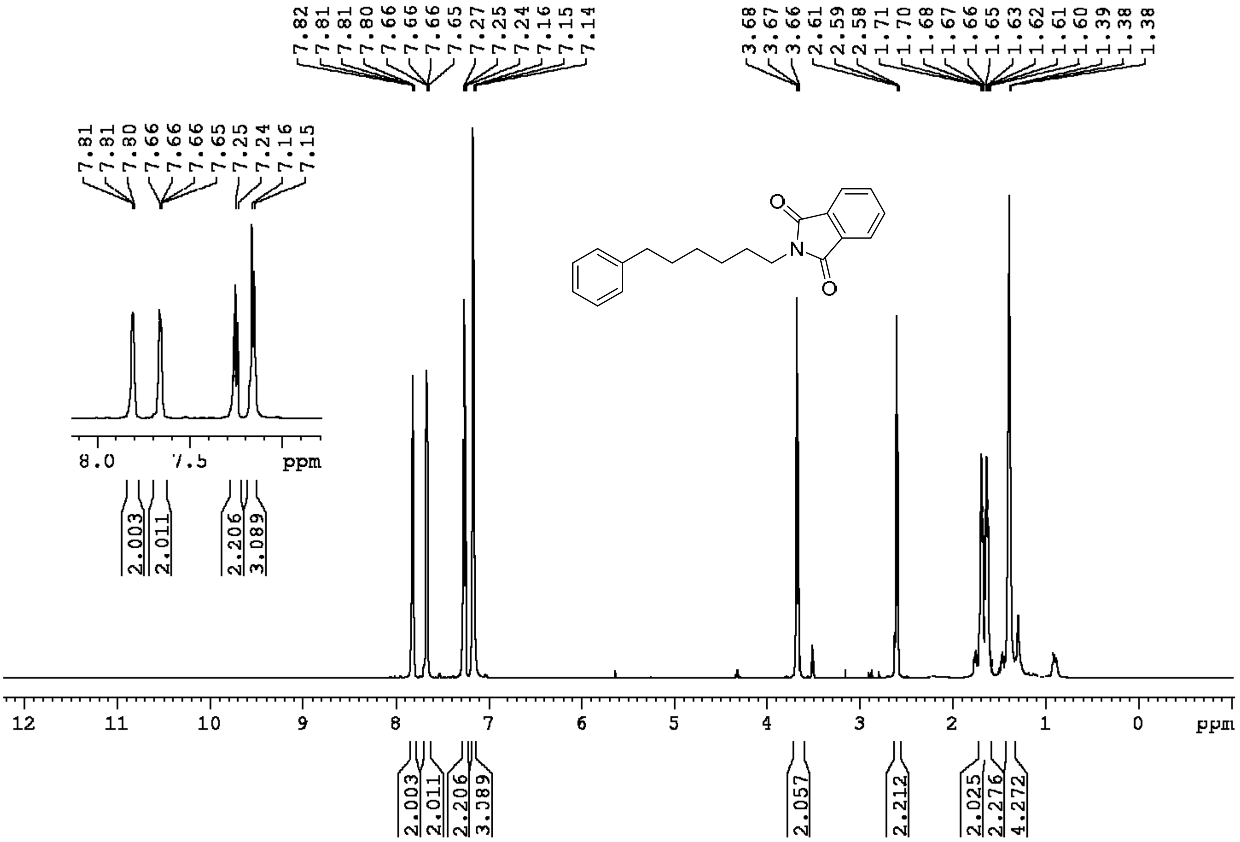

^{13}C-NMR (151 MHz, $CDCl_3$) 2-(6-phenylhexyl)isoindoline-1,3-dione

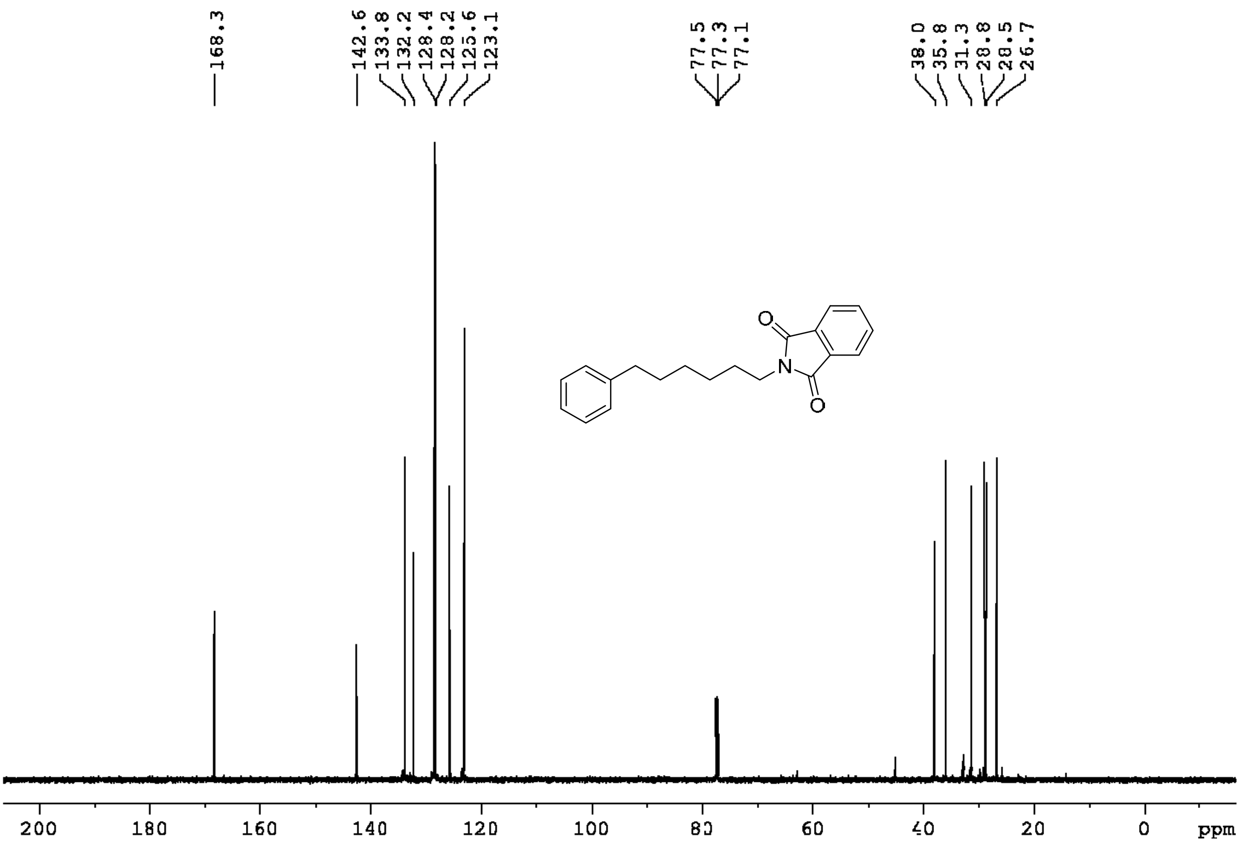

^{1}H-NMR (600 MHz, $CDCl_3$) 2,2,2-Trifluoro-*N*-(6-phenylhexyl)acetamide (10a)

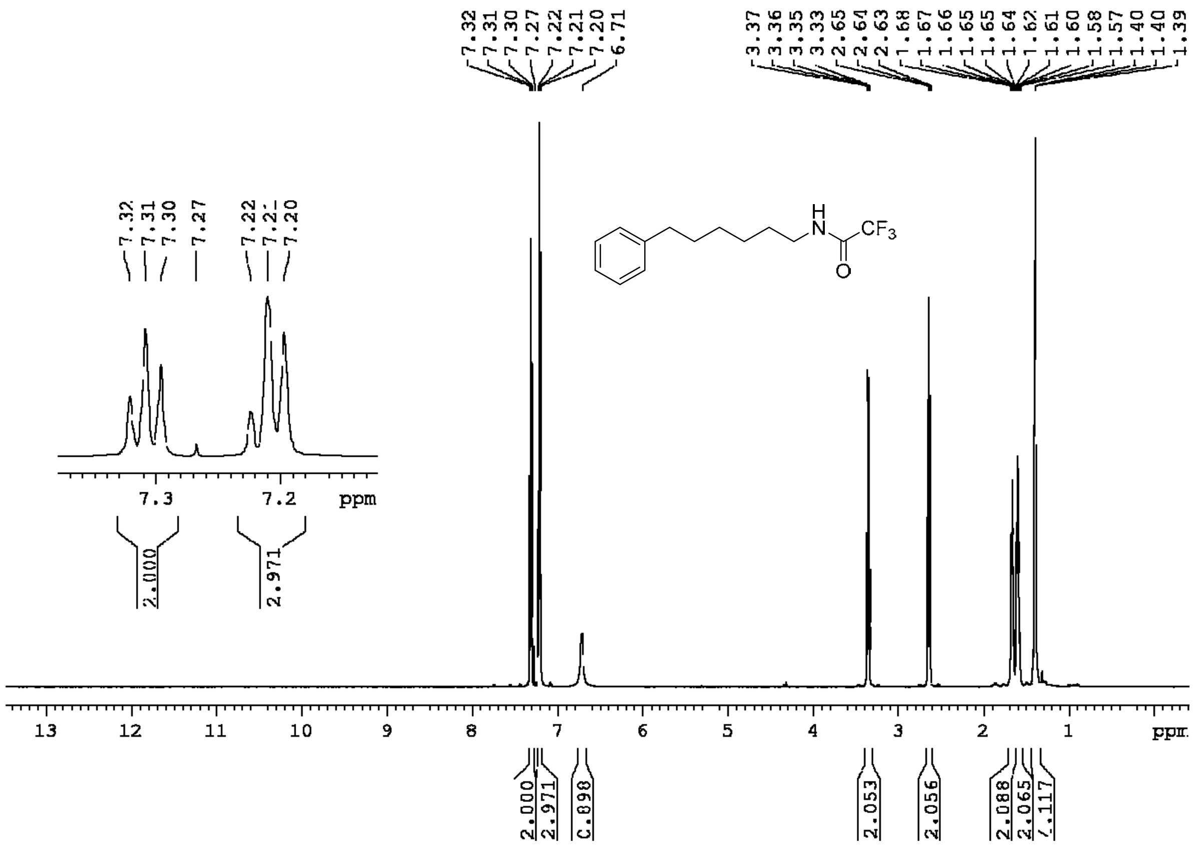

^{13}C-NMR (151 MHz, $CDCl_3$) 2,2,2-Trifluoro-*N*-(6-phenylhexyl)acetamide(10a)

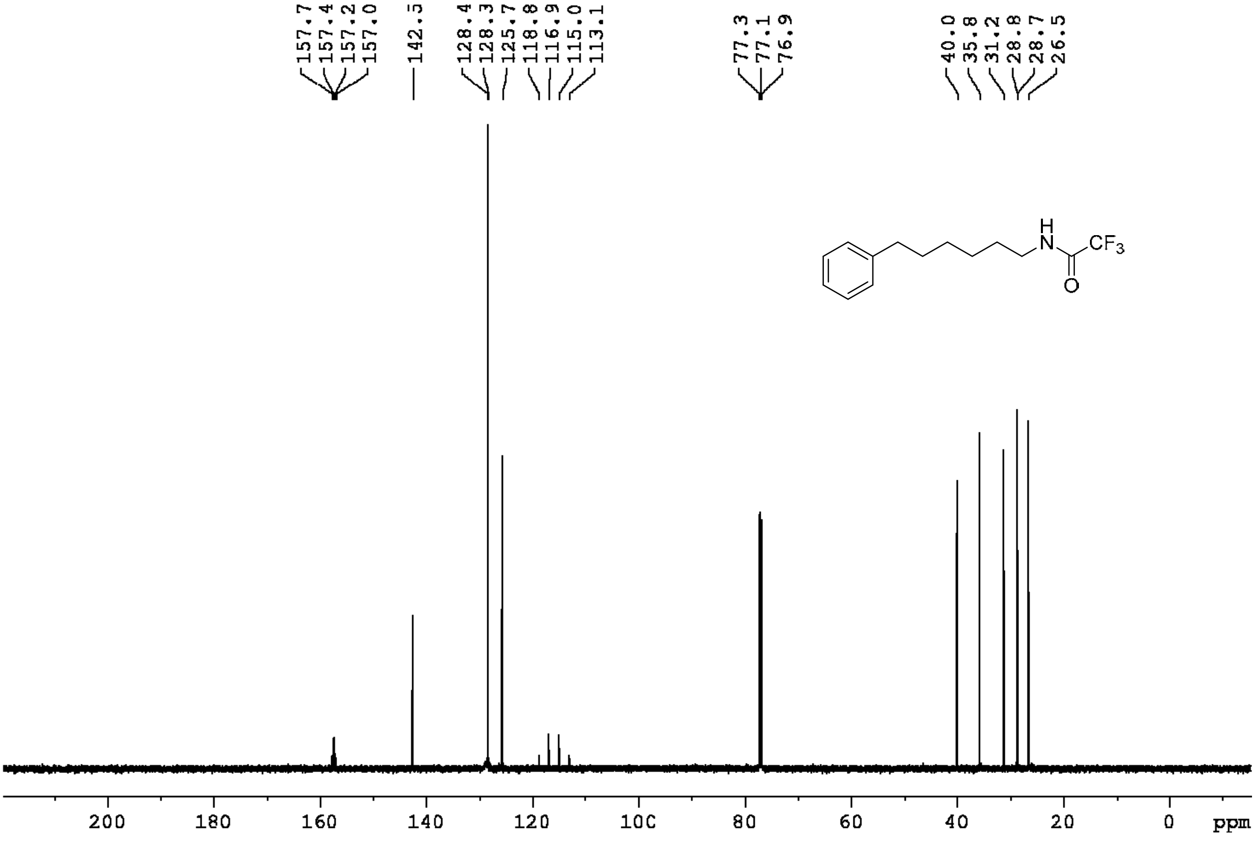

^{1}H-NMR (600 MHz, $CDCl_3$) *N*-(2,3-dihydro-1H-inden-1-yl)-2,2,2-trifluoroacetamide (11a)

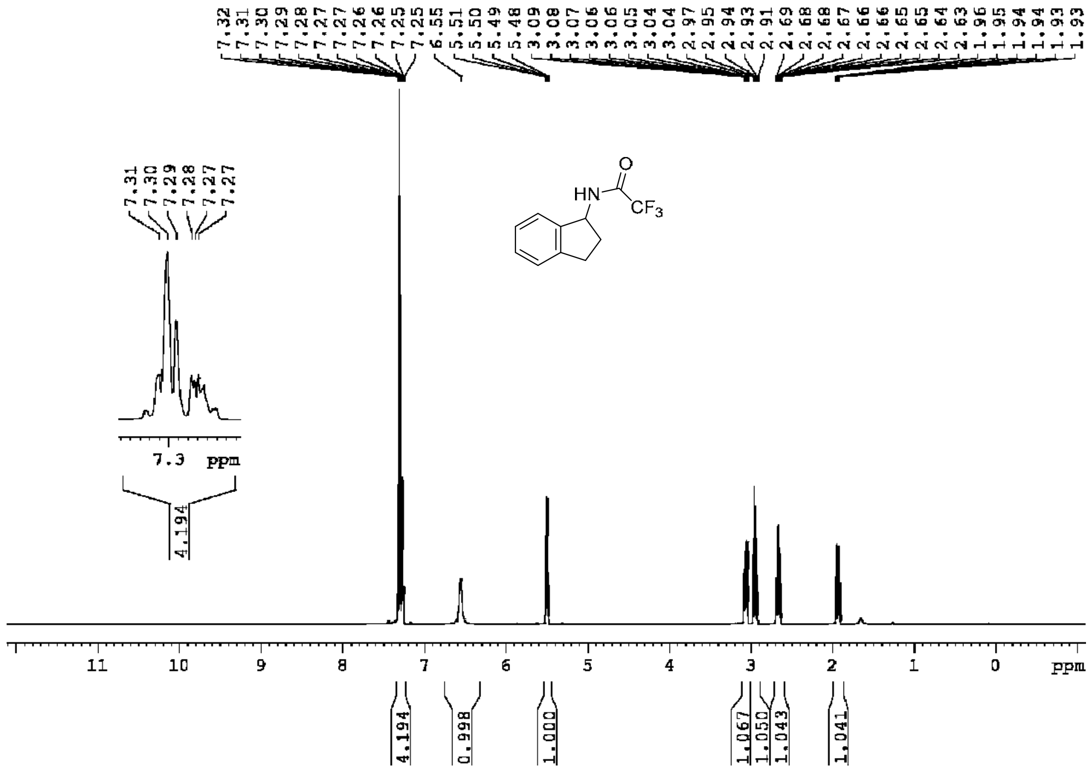

^{13}C-NMR (151 MHz, $CDCl_3$) *N*-(2,3-dihydro-1H-inden-1-yl)-2,2,2-trifluoroacetamide (11a)

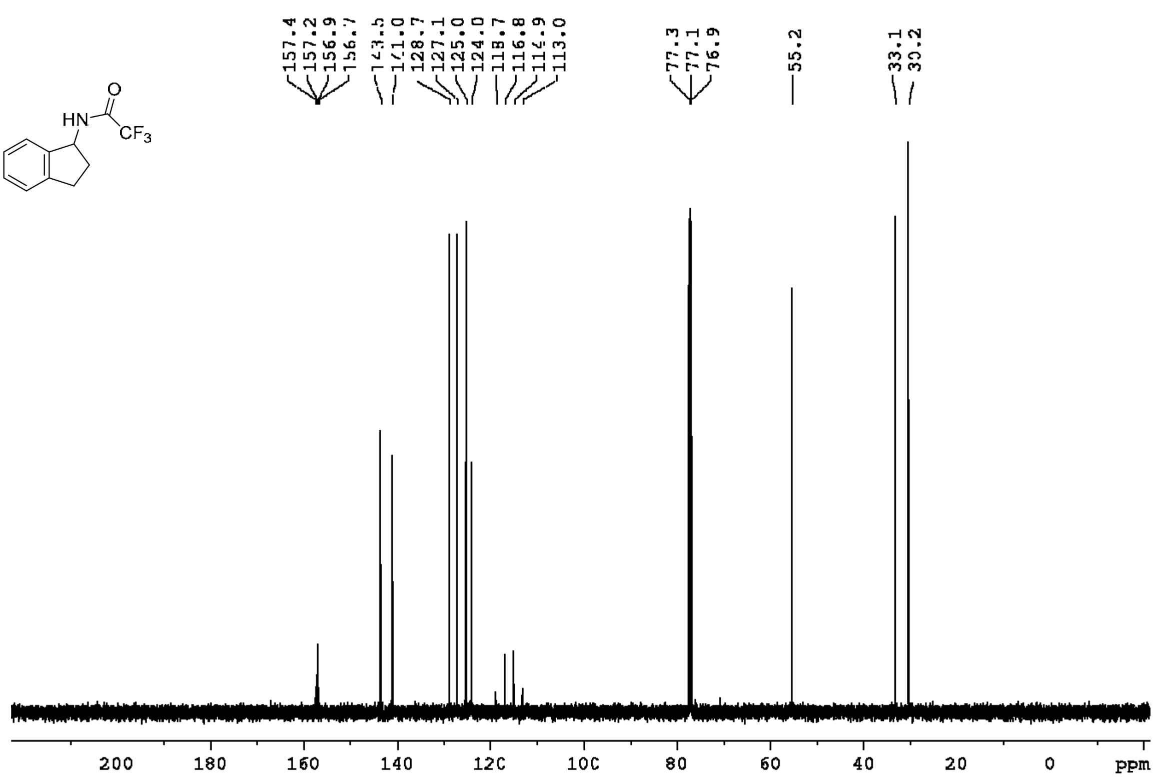

^{1}H NMR (600 MHz, $CDCl_3$) of (cis)-2,2,2-trifluoro-N-(2-phenylcyclohexyl)acetamide (*Cis*-12a)

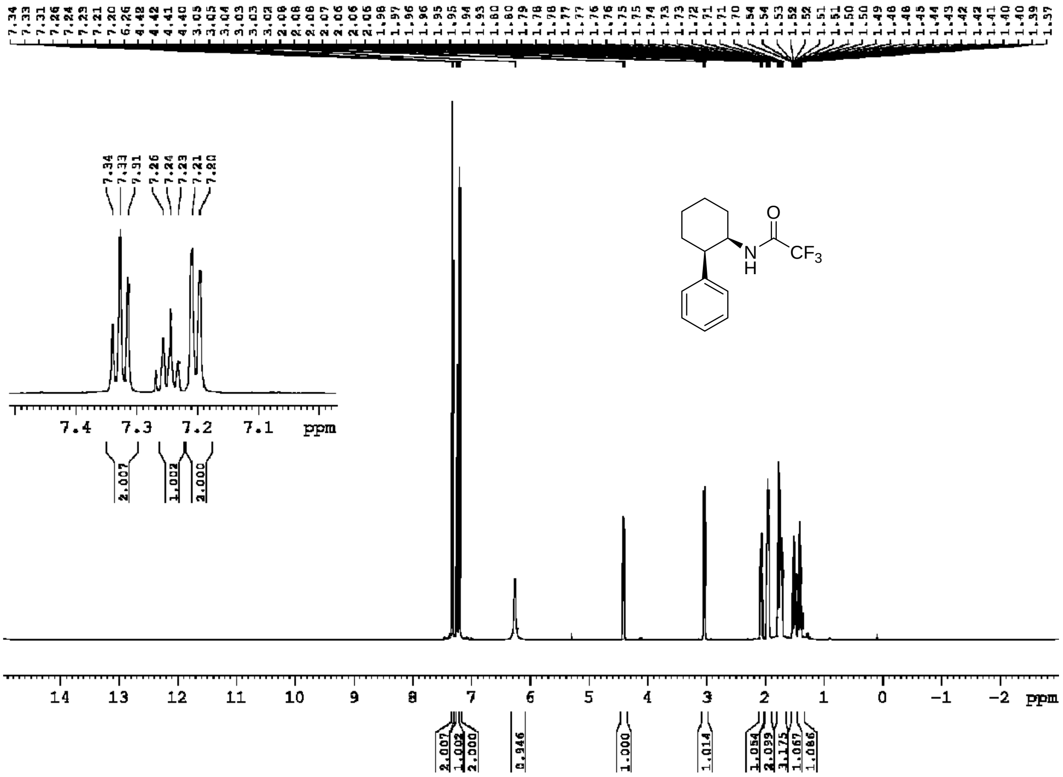

^{13}C NMR (151 MHz, $CDCl_3$) of (cis)-2,2,2-trifluoro-N-(2-phenylcyclohexyl)acetamide (*Cis*-12a)

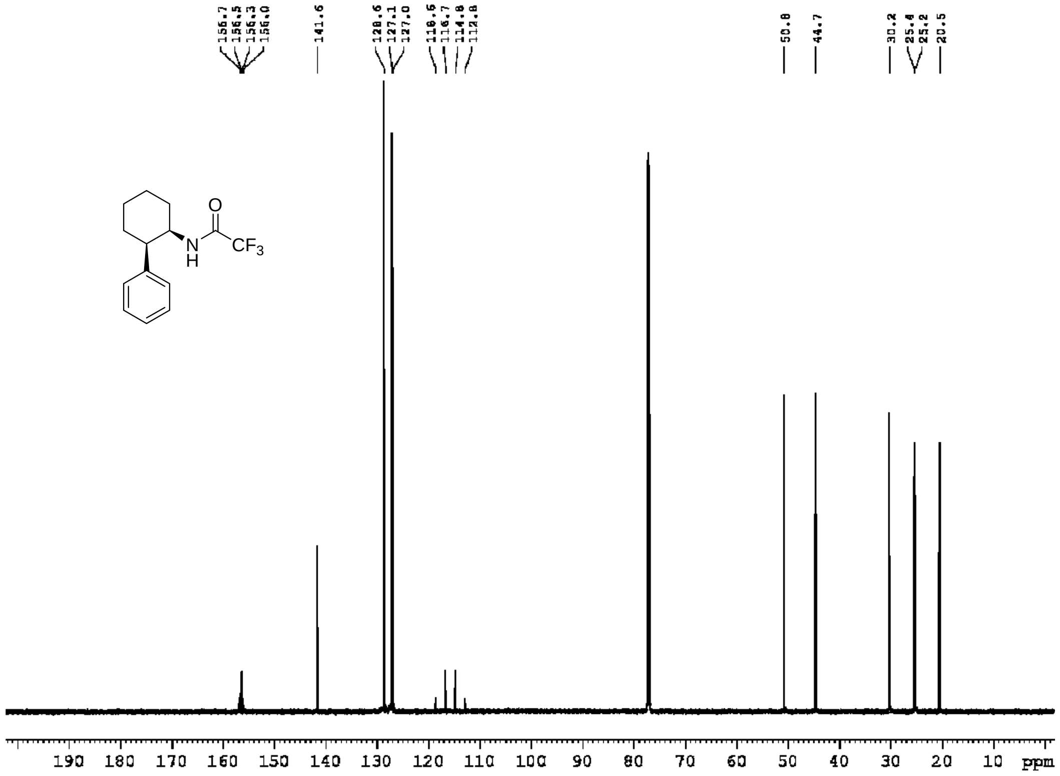

^{1}H NMR (600 MHz, $CDCl_3$) of (trans)-2,2,2-trifluoro-N-(2-phenylcyclohexyl)acetamide (*Trans*-12a)

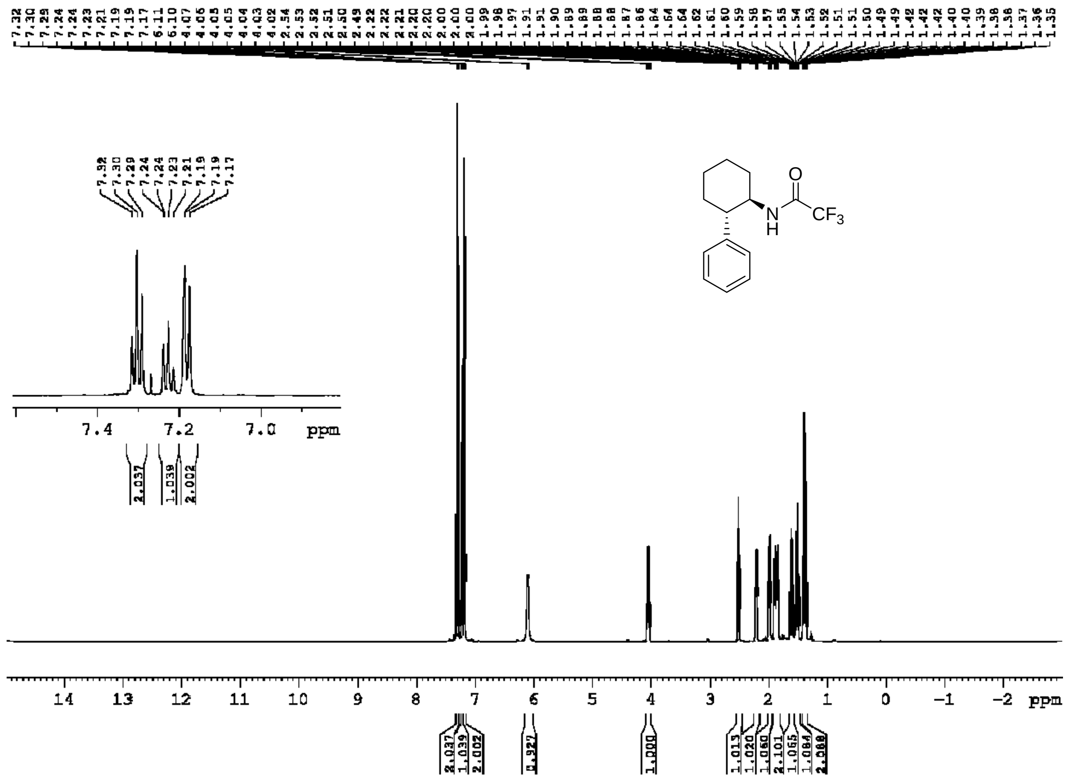

^{13}C NMR (151 MHz, $CDCl_3$) of (trans)-2,2,2-trifluoro-N-(2-phenylcyclohexyl)acetamide (*Trans*-12a)

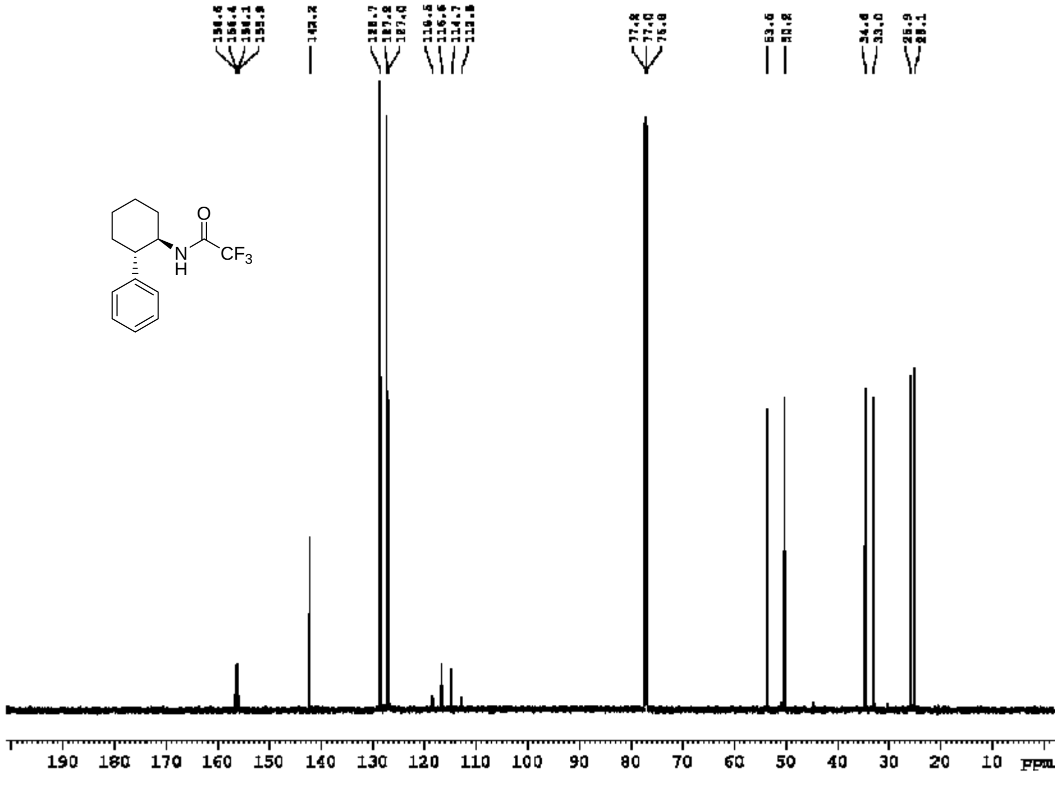

^{1}H NMR (500 MHz, DMSO-d_6 – 120 °C) *N*-ethyl-2,2,2-trifluoro-N-(2-(trifluoromethyl)benzyl)acetamide (13)

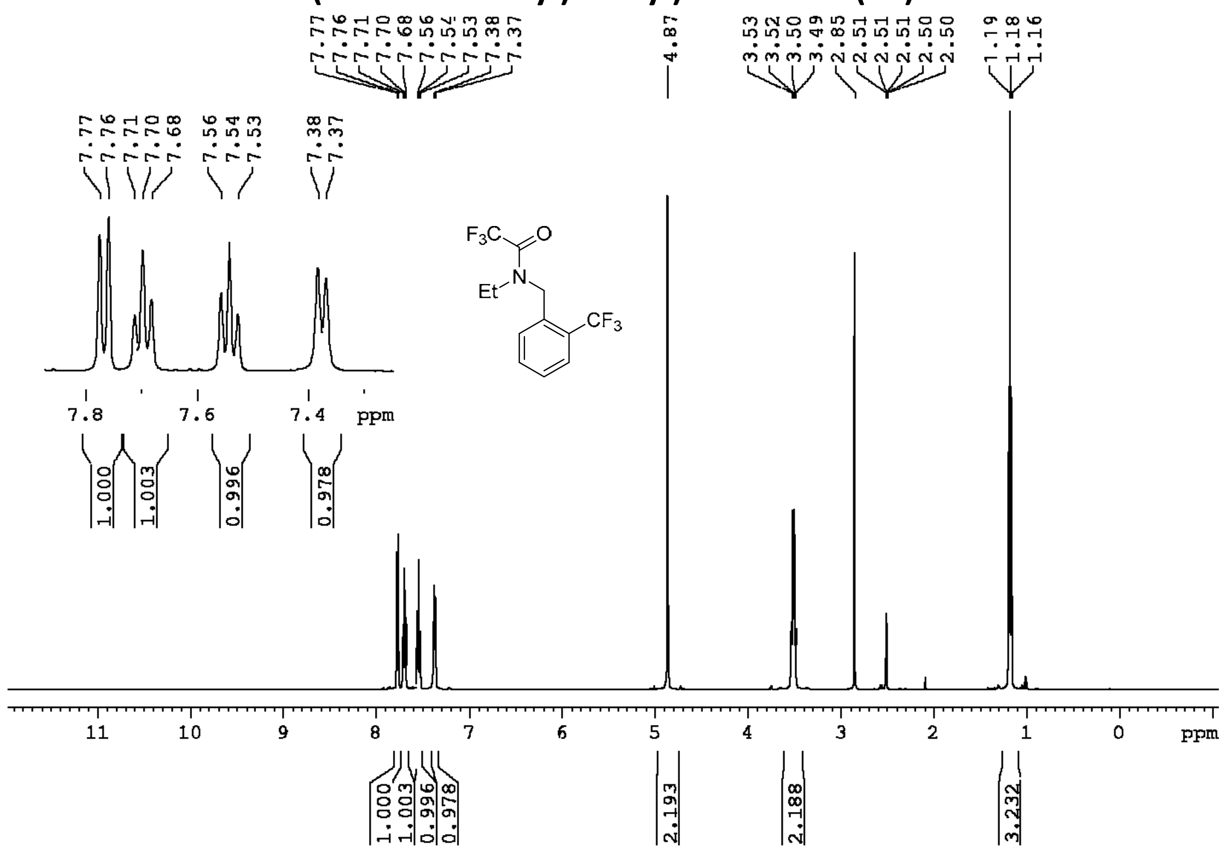

^{13}C NMR (126 MHz, DMSO-d_6 – 120 °C) *N*-ethyl-2,2,2-trifluoro-N-(2-(trifluoromethyl)benzyl)acetamide (13)

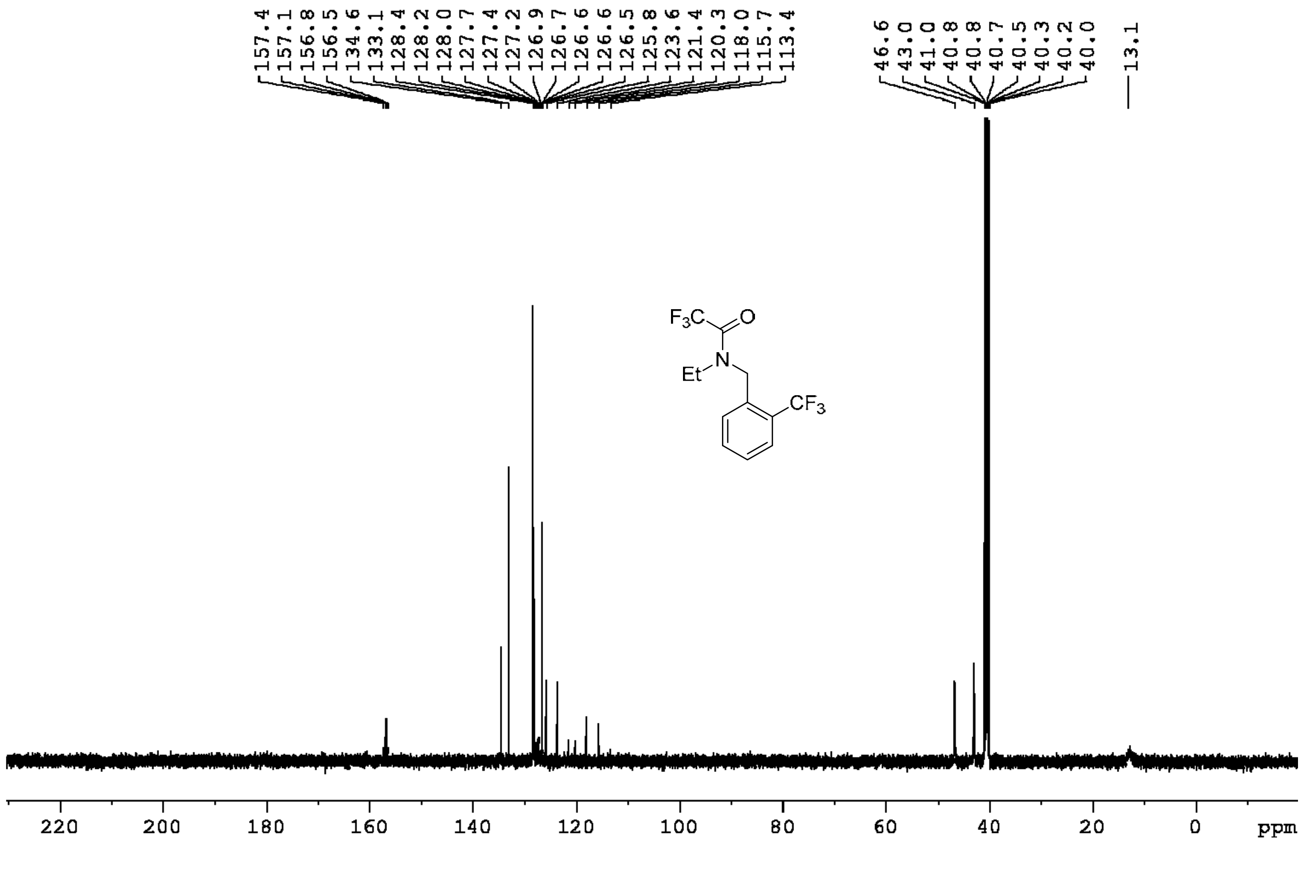

^{1}H NMR (500 MHz, DMSO-d6, 120 °C) of N-(2-bromophenethyl)-N-ethyl-2,2,2-trifluoroacetamide (14)

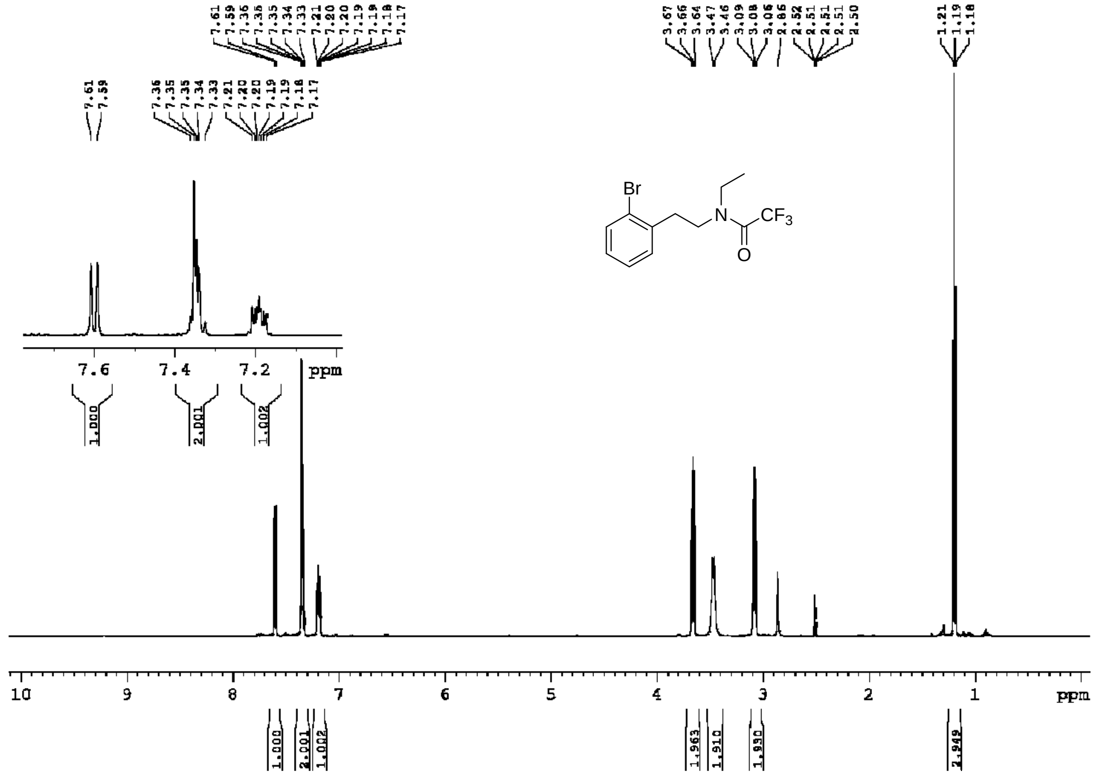

^{13}C NMR (126 MHz, DMSO-d6, 120 °C) of N-(2-bromophenethyl)-N-ethyl-2,2,2-trifluoroacetamide (14)

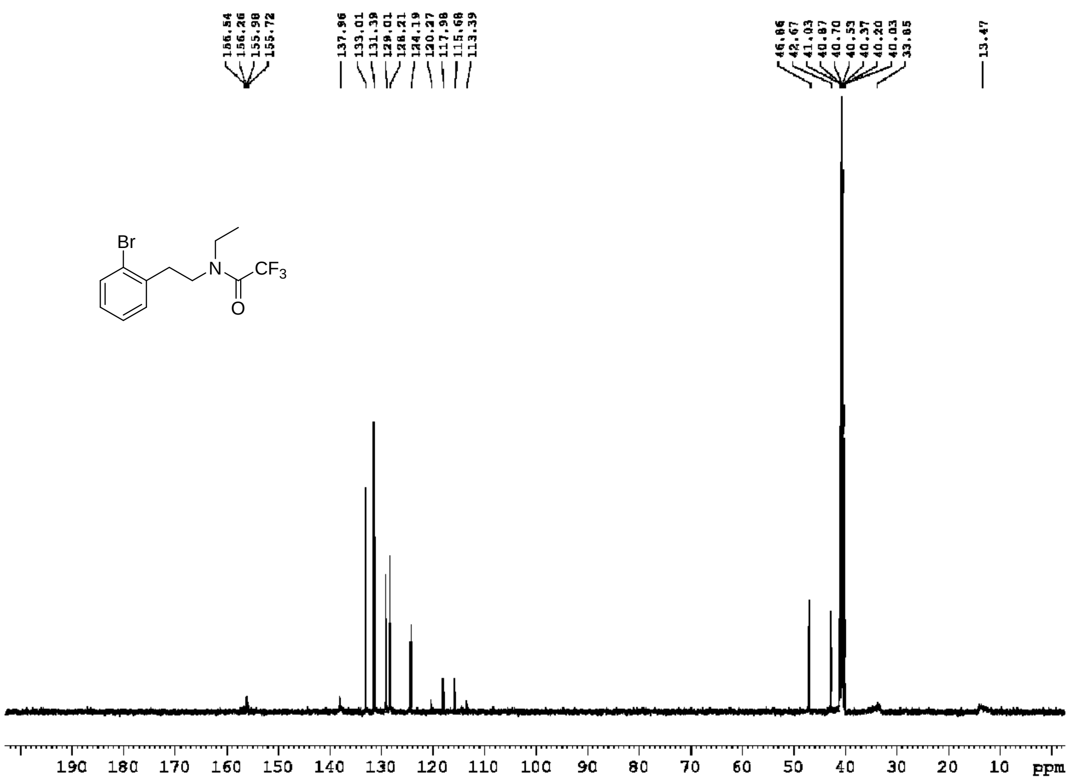

^{1}H-NMR (500 MHz, DMSO-d_6, 120 °C) *N*-(3-(2-Bromophenyl)propyl)-N-ethyl-2,2,2-trifluoroacetamide (15)

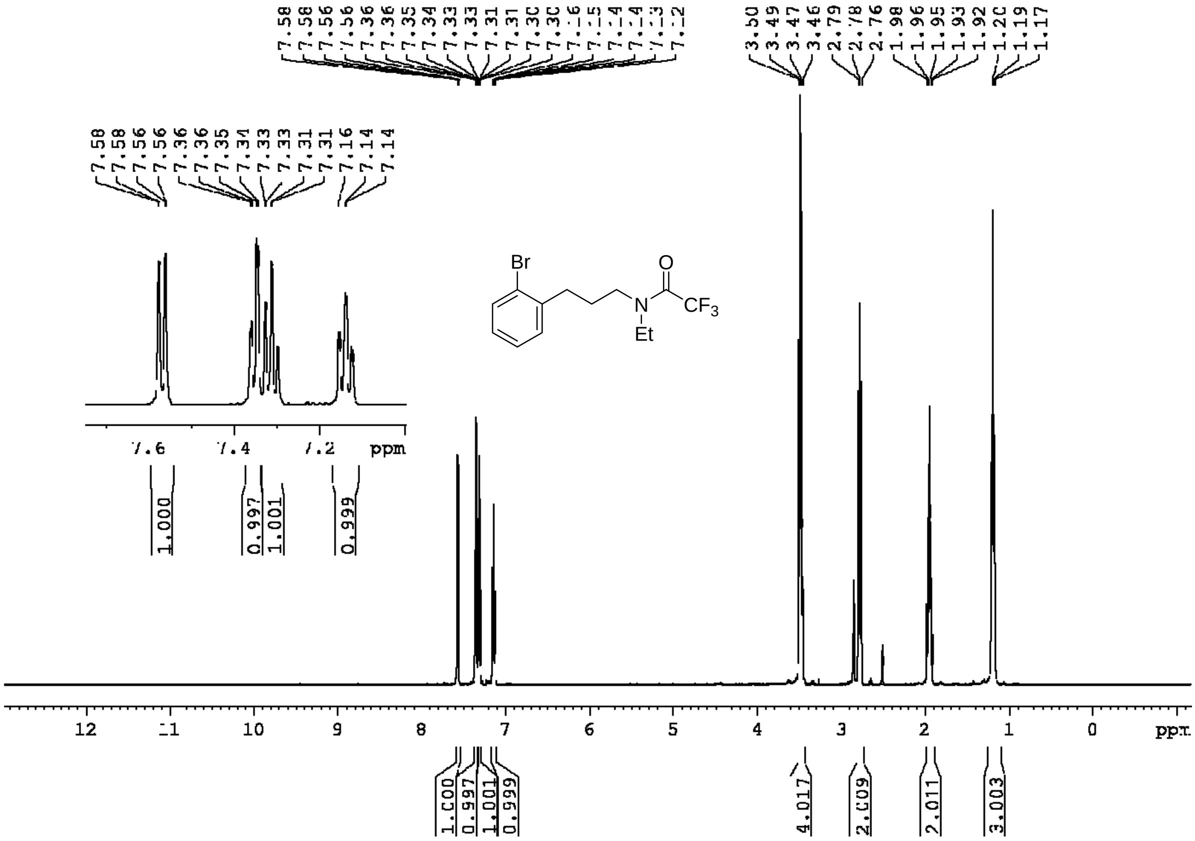

^{13}C-NMR (126 MHz , DMSO-d_6, 120 °C) *N*-(3-(2-Bromophenyl)propyl)-N-ethyl-2,2,2-trifluoroacetamide (15)

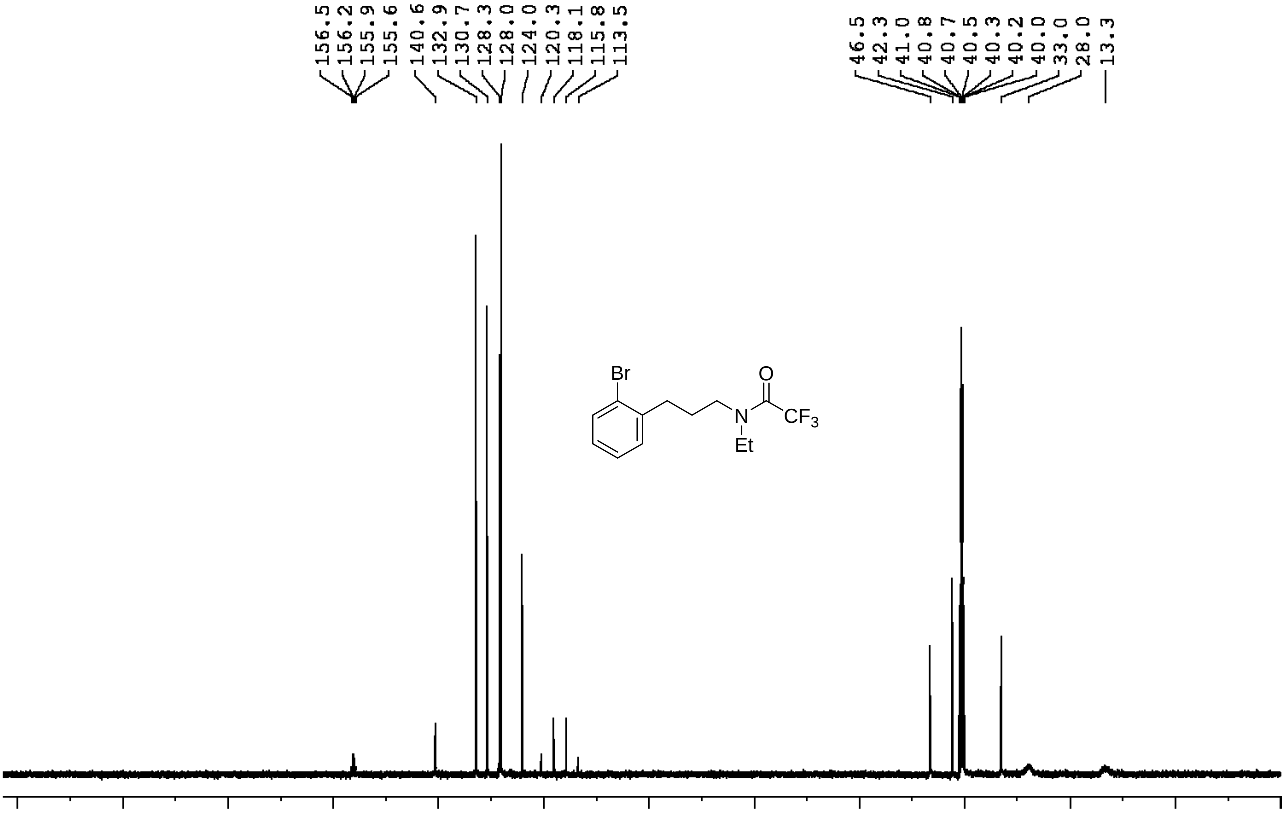

Supplement: Supplementary file 1 — Supplementary [file ANIE-56-13351-s001.pdf]
